# Comparative transcriptome analysis of transcripts of uncertain coding potential in septic myocardial depression

**DOI:** 10.1186/s12872-021-01973-z

**Published:** 2021-04-08

**Authors:** Tie-Ning Zhang, Ri Wen, Ni Yang, Chun-Feng Liu

**Affiliations:** Department of Pediatrics, PICU, Shengjing Hospital of China Medical University, No. 36, SanHao Street, Shenyang City, 110004 Liaoning Province People’s Republic of China

**Keywords:** TUCPs, Sepsis, Myocardial dysfunction, RNA-seq

## Abstract

**Background:**

Septic shock with myocardial depression is very common in intensive care units. However, the exact molecular mechanisms underlying sepsis-induced myocardial depression remain unclear. Whether the profiles of transcripts of uncertain coding potential (TUCPs) differ between patients with and without myocardial depression is also unknown. Our study aimed to find expression differences between groups of TUCPs and determine their potential functions in a preclinical model.

**Methods:**

We generated rat models of hypodynamic septic shock induced by lipopolysaccharide. A total of 12 rats were established and left ventricular tissue from each was collected. We performed RNA-seq to identify TUCPs in each sample. Transcripts with an corrected P value of < 0.05 were defined as differentially expressed (DE). We also performed GO terms and KEGG analysis to identify the potential functions of DE TUCPs.

**Results:**

A total of 4,851 TUCPs were identified in heart samples, 85 of which were expressed differently between the sepsis and control groups. Further bioinformatic analyses suggested that TUCPs play important roles in myocardial contraction, energy regulation, and metabolic processes, and are also involved in the regulation of several pathways.

**Conclusion:**

Our results demonstrate that TUCPs both participate in and mediate the pathological process of myocardial depression. Our study improves the understanding of the basic molecular mechanisms underlying myocardial depression from a novel perspective.

**Supplementary Information:**

The online version contains supplementary material available at 10.1186/s12872-021-01973-z.

## Introduction

Sepsis is defined as a potentially life-threatening condition, which is characterized as an uncontrolled host response to infection [1, 2]. Notably, the condition called “septic shock” refers to circulatory and cellular abnormalities during sepsis, which are profound enough to greatly increase mortality [1]. The previous study has recognized that sepsis-induced myocardial depression, also called sepsis-induced heart dysfunction, is a potential and serious complication of sepsis [3]. It is characterized by impaired myocardial contractility and reduced ejection fraction, which could lead to higher mortality, especially in pediatric patients [3–5]. Since a thorough pathophysiologic understanding of sepsis-induced myocardial depression has not been defined, an imperative for more studies on this issue are required.

Recent studies have indicated that non-coding RNAs including microRNAs and long non-coding RNAs (lncRNAs) are involved in the process of sepsis-induced myocardial depression [6, 7]. However, little is known regarding transcripts of uncertain coding potential (TUCPs), which are an important component of RNA in cells. TUCPs are recognized as part of the lncRNAs, but are excluded by Pfam scan criteria during the steps for filtering lncRNAs [8]. Functional studies have suggested that many TUCP transcripts could encode small peptides [9]. TUCPs were previously annotated as pseudogenes, but could be involved in different functions as non-coding regulatory agents [10]. However, to the best of our knowledge, no studies have explored the expression patterns and potential functions of TUCPs in sepsis-induced myocardial depression.

We hypothesized that TUCPs might be involved in the pathological process of sepsis-induced myocardial depression. We performed this preliminary work in a preclinical model to identify the potential role of TUCPs in sepsis-induced myocardial depression. Our study aimed to describe the features of TUCPs and determine their potential functions in this condition. This first study on TUCPs in sepsis provides a useful resource for studying TUCPs’ functional roles and obtaining a new perspective toward understanding the pathophysiological process of sepsis-induced myocardial depression.

## Methods

### Animal model of septic shock

All experimental protocols were approved by Shengjing Hospital of China Medical University (2019PS073K). All methods were carried out in accordance with relevant guidelines and regulations. The study was carried out in compliance with the ARRIVE guidelines (http://www.nc3rs.org.uk/page.asp?id=1357). An adolescent rat model of septic shock was established by the intraperitoneal injection of lipopolysaccharide (LPS), as in our previous study [6, 7]. Briefly, male pathogen-free Wistar rats from Changsheng Bio Company (Benxi, China) weighing from 170 to 190 g were anesthetized with 20% urethane (1 g/kg i.p.). We cannulated the left femoral artery to continue monitoring the mean arterial pressure (MAP) of the animals (Biopac MP150; Biopac Systems, Goleta, CA, USA). To develop a model of septic shock with myocardial dysfunction, we challenged the rats with a large dose of *Escherichia coli* 055:B5 (L-2880; Sigma–Aldrich, St. Louis, MI, USA; 20 mg/kg, 10 mg LPS dissolved in 1 mL of 0.9% saline); septic shock was considered to have been established when MAP decreased to 25%–30% of the baseline value. The left ventricle of the heart was excised after 12 h of LPS or saline administration, immediately snap-frozen in liquid nitrogen, and stored at − 80℃ for further experiments.

### RNA-seq

Total RNAs from rats subjected to septic shock (n = 6) and controls (n = 6) were isolated and quality-controlled. All information regarding each relative kit and detailed procedures about the RNA extraction, RNA quality control and library preparation were displayed in our previous studeis [6, 7]. The preparation of whole-transcriptome libraries and next-generation sequencing were conducted by Novogene Bioinformatics Technology Corporation (Beijing, China). RNA-seq was performed on an Illumina Hiseq 4000 platform and 150-bp paired-end reads were generated in accordance with Illumina’s protocol. All of the downstream bioinformatic analyses were based on the clean data of high quality.

### Differential expression analysis

The Ballgown suite includes functions for interactive exploration of the transcriptome assembly, visualization of transcript structures and feature-specific abundances for each locus, and post hoc annotation of assembled features to annotated features. Cuffdiff provides statistical routines for determining the differential expression in digital transcript or gene expression data using a model based on the negative binomial distribution [11]. Transcripts with an corrected P value (also called as q valude) of < 0.05 were defined as differentially expressed, which was consistent with our previous study [6, 7].

### Target gene prediction

The investigation of target gene prediction of TUCPs was acting on neighboring target genes (co-location analyses, cis role of TUCP). To investigate their possible functions, we searched the coding genes within 100 kb upstream and downstream of each TUCP [12, 13]. Trans role of target gene prediction is TUCP to identify each other by the expression level (co-expression analyses). We clustered the genes from different samples with WGCNA to search common expression modules and then analyzed their function through functional enrichment analysis [14]. We used co-localized and co-expressed mRNAs to predict the potential roles of TUCP transcripts during sepsis-induced myocardial depression by Gene Ontology (GO) enrichment and KEGG pathway analyses.

### GO and KEGG enrichment analyses

GO enrichment analysis of differentially expressed genes or lncRNA target genes was implemented using the GOseq R package, in which gene length bias was corrected [15]. GO terms with a corrected P value less than 0.05 were considered significantly enriched among the differentially expressed genes. Additionally, we used KOBAS software to test the statistical enrichment of differentially expressed genes or TUCP target genes in KEGG pathways (www.kegg.jp/kegg/kegg1.html) [16, 17].

## Results

### RNA sequencing identified the features of TUCPs

**Fig. 1:**
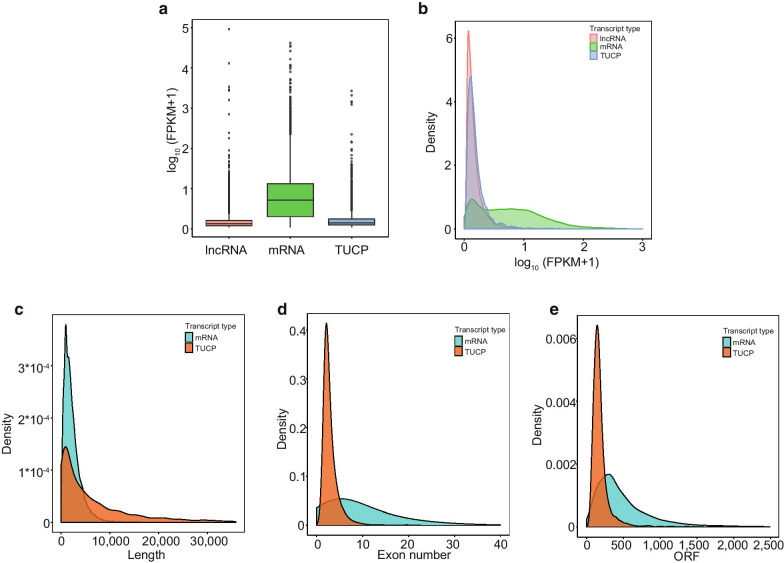
Characterization of TUCPs in rat heart tissue. **a**–**c** Box plot, density distribution diagram, and violin plot showing the expression features of lncRNAs, mRNAs, and TUCPs in rat heart tissue all samples; **d–e** Density distribution diagram showing the expression features of length, exon number, and opening reading frame (ORF) of TCUPs and mRNAs. FPKM, fragments per kilobase million

In our previous studies, we found that LPS-treated rats showed a gradual decline in cardiac function as evidenced by significant decreases in heart rate, LV peak rate of a pressure rise, and an LV peak rate of pressure decay, as well as a prolonged relaxation time constant, and there is a positive relationship between MAP and heart function [18]. Therefore, we used a rat sepsis model and measured the MAP to represent changes in heart function. We found that septic shock occurred approximately 2 h after LPS administration and lasted for the rest of the observation period [6, 7]. The left ventricle of the heart was then excised for RNA-seq and the determination of TUCPs. We identified 4,851 TUCPs from both the sepsis group and the control group. Detailed information regarding each TUCP including chromosome location, start and end locations, exon number, length, and open reading frame (ORF) is shown in Additional file 2: Table S1. The sequence of each TUCP is shown in Additional file 3: Table S2. To clarify the basic characteristics of TUCPs, we first compared their expression level with those of lncRNAs and mRNAs. We found that the expression level of TUCPs was similar to that of lncRNAs in these 12 samples, but was lower than that of mRNAs (Fig. 1a, b). In addition, we performed a comparison of the TUCPs with mRNAs in terms of length, exon number, and ORF. Our findings suggested that the TUCPs were longer than the mRNAs; however, the TUCPs had fewer number of exons and shorter ORFs than the mRNAs (Fig. 1c–e).

### Differential expression (DE) and cluster analysis of TUCPs

**Fig. 2:**
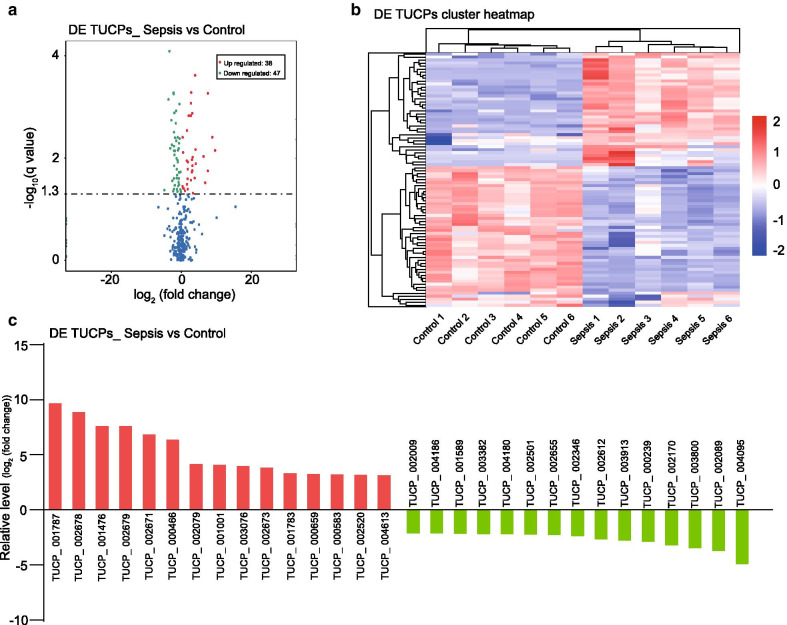
The expression profiling changes of TUCPs in rat heart tissue. **a** Volcano Plot indicating up- and down-regulated TUCPs of rat heart tissue in the sepsis group compared with control group, up- and down-regulated genes are colored in red and blue, respectively; **b** Heatmap of TUCPs showing hierarchical clustering of changed TUCPs of rats heart tissue in sepsis group compared with control group, up- and down-regulated genes are colored in red and blue, respectively; **c** Differences in the expression of TUCPs in rat heart tissue from sepsis and normal group. *DE* differential expression

To determine whether TUCPs are involved in the pathogenesis of sepsis-induced myocardial depression, the expression pattern of TUCPs was analyzed and compared between the sepsis group and the control group. We analyzed DE using significance analysis with the threshold of q value < 0.05 (corrected P value). The results revealed 85 TUCPs that were differentially expressed between the sepsis group and the control group, including 38 that were upregulated and 47 that were downregulated in the sepsis group (Fig. 2a). The chromosome distribution of the DE TUCPs is shown in Additional file 1: Figure S1. In addition, the expression pattern of the DE TUCPs is shown using a cluster heatmap in Fig. 2b. Furthermore, the relative expression levels of the top 15 upregulated and downregulated TUCPs are shown in Fig. 2c. Detailed information on the 85 DE TUCPs including the relative expression levels in the two groups, fold change, and q value is displayed in Additional file 4: Table S3. Notably, three TUCPs (TUCP_000356, TUCP_002674, and TUCP_004817) were not detected in the control group but were expressed in the sepsis group. Besides, all the co-located and co-expressed genes for each DE TUCP were shown in Additional file 5: Table S4 and Additional file 6: Table S5, respectively. These results reflect distinct TUCP expression profiles between sepsis-induced myocardial depression and the control group, implying the critical role of TUCPs in the pathophysiology of septic myocardial depression.

### GO analysis of DE TUCPs in sepsis

**Fig. 3:**
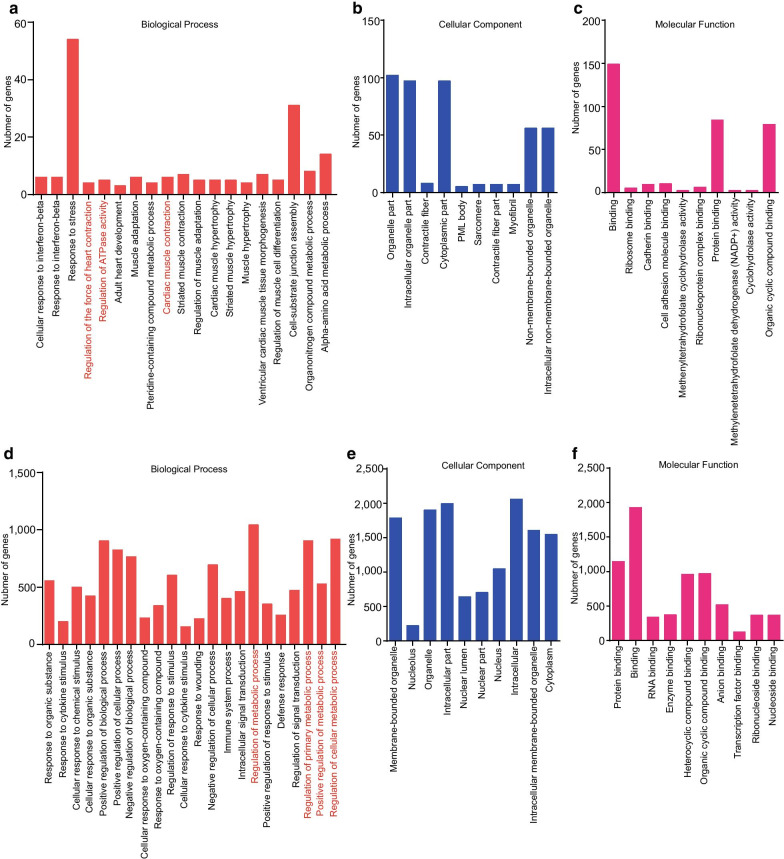
Gene Ontology (GO) analysis of differentially expressed TUCPs and TUCP target genes in rat heart tissue from sepsis group and control group. **a**–**c** GO categories (biological process, cellular components, and molecular function) of differential TUCPs and TCUP target genes in rat heart tissue from sepsis group and control group via co-location analyses; **d**–**f** GO categories (biological process, cellular components, and molecular function) of differential TUCPs and TCUP target genes in rat heart tissue from sepsis group and control group via co-expression analyses;

To elucidate the possible functional significance of the observed changes in TUCPs between the sepsis group and the control group, we performed a GO term enrichment analysis based on co-location analyses (cis role of TUCPs) and co-expression analyses (trans role of TUCPs, Additional file 7: Table S6 and Additional file 8: Table S7). There were 20,121 background genes of GO terms in total. We summarized the GO terms significantly enriched for the TUCPs regarding co-location analyses (Fig. 3a–c) and co-expression analyses (Fig. 3d–f), for the categories of biological process, cellular component, and molecular function, respectively. For co-location, the GO terms “Regulation of the force of heart contraction,” “Regulation of ATPase activity,” and “Cardiac muscle contraction” were enriched, suggesting that several TUCPs participate in energy production and myocardial contraction as co-location analyses, highlighting the critical role of TUCPs in the pathogenesis of sepsis-induced myocardial depression. For co-expression, we found that TUCPs take part in metabolic processes in myocardial tissue. For example, the GO terms “Regulation of metabolic process,” “Regulation of primary metabolic process,” “Positive regulation of metabolic process,” and “Regulation of cellular metabolic process” were enriched, which showed that TUCPs have potential roles in regulating metabolism. Based on GO analysis, we found that complex pathological processes are involved in sepsis-induced myocardial depression and that TUCPs play key roles in this disease.

### KEGG analysis of DE TUCPs in sepsis

**Fig. 4:**
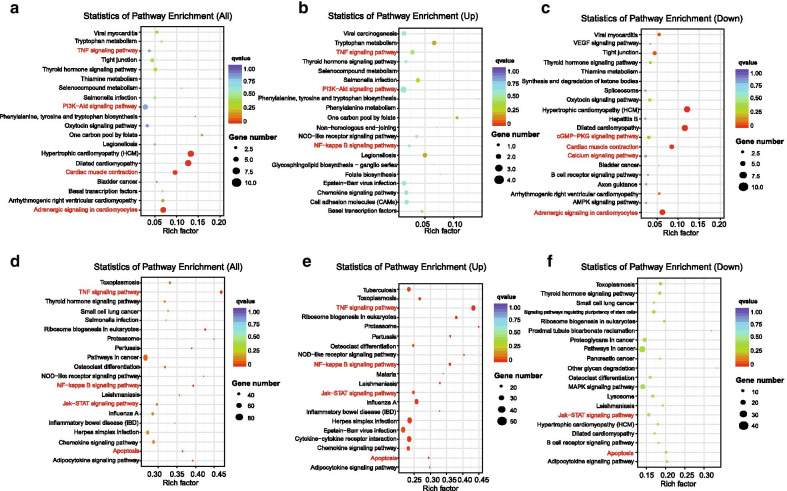
Kyoto Encyclopedia of Genes and Genomes (KEGG) analysis (www.kegg.jp/kegg/kegg1.html) of differentially expressed TUCPs and TUCP target genes in rat heart tissue from sepsis group and control group. **a**–**c** KEGG analysis of differential TUCPs and TUCP target genes in rat heart tissue from sepsis group and control group via co-location analyses (All enriched pathway, up-regulated pathway, down-regulated pathway, respectively); **d**–**f** KEGG analysis of differential TUCPs and TUCP target genes in rat heart tissue from sepsis group and control group via co-expression analyses (All enriched pathway, up-regulated pathway, down-regulated pathway, respectively). Rich factor refers to the ratio of the number of DE genes to the total number of genes in this pathway

To determine whether some specific pathways changed in sepsis-induced myocardial depression, we performed KEGG enrichment analysis in TUCPs based on co-location analyses and co-expression analyses (Additional file 9: Table S8 and Additional file 10: Table S9). For co-location (Fig. 4a–c), we found that TUCPs could affect heart contraction by influencing some specific pathways. For example, “cGMP − PKG signaling pathway,” “Cardiac muscle contraction,” “Calcium signaling pathway,” and “Adrenergic signaling in cardiomyocytes” were downregulated, which was consistent with the pathophysiological changes of sepsis-induced myocardial depression and also similar to the GO enrichment results, indicating the critical role of TUCPs in regulating heart contraction. For co-expression (Fig. 4d–f), “TNF signaling pathway,” “NF-kappa B signaling pathway,” “Jak–STAT signaling pathway,” and “Apoptosis” were enriched, suggesting that TUCPs could function in these pathways in sepsis-induced myocardial depression. Our KEGG enrichment analysis also suggested the significance of TUCPs in sepsis-induced myocardial depression.

## Discussion

According to clinical epidemiological studies, the estimated incidence of sepsis and the associated in-hospital mortality rate in the US are approximately 5.9% and 15.6%, respectively, making it a major public health problem [19]. Notably, septic shock, which is a subtype of sepsis with circulatory and cellular/metabolic dysfunction, is still associated with an even higher risk of mortality. Sepsis-induced myocardial depression, as a main complication of septic shock, is one of the most important factors contributing to the high mortality of septic shock. However, the basic pathophysiological mechanisms underlying sepsis-induced myocardial depression remain unclear and should be investigated in further studies.

Studies on gene regulatory networks have focused on protein-coding genes. However, recently, with the development of next-generation high-throughput sequencing, genomic analyses have determined that approximately 90% of non-coding sequences in the human genome are transcribed into non-coding RNAs (ncRNAs), which play key regulatory roles in multiple biological processes [20]. Previous studies showed that lncRNAs, microRNAs, and even circRNAs play important roles in the regulation of sepsis-induced myocardial depression [6, 7]. Notably, TUCPs, as part of the lncRNAs, despite previously being annotated as pseudogenes, could be involved in different functions as non-coding regulatory agents. However, it had remained unknown whether the expression pattern of TCUPs in sepsis-induced myocardial depression differs from that in healthy controls. Therefore, to clarify the potential function of TUCPs, we performed this study to identify the basic characteristics and DE TUCPs in sepsis-induced myocardial depression. The results revealed 4,851 TUCPs from both the sepsis group and the control group, based on a total of 12 samples of left ventricular tissue. Through further analyses, we reported 85 TUCPs that were differentially expressed between the sepsis group and the control group, including 38 that were upregulated and 47 that were downregulated in the sepsis group. The differential expression of these TUCPs suggested that they could play critical roles in the pathology of sepsis-induced myocardial depression.

Although we identified 85 TUCPs that were differentially expressed between the sepsis group and the control group, the potential functions of these TUCPs were unclear. Therefore, to clarify these functions, we performed GO term and KEGG pathway enrichment analyses of the TUCPs with their target mRNAs. The aim was to evaluate their potential regulatory roles and provide compelling evidence that TUCPs participate in the pathogenesis of sepsis-induced myocardial depression. Notably, we found that TUCPs could participate in energy production and myocardial contraction in this disease, which are associated with the basic pathophysiological changes of sepsis-induced myocardial depression, highlighting the key roles of TUCPs in this disease. For example, the GO terms “Regulation of the force of heart contraction,” “Regulation of ATPase activity,” and “Cardiac muscle contraction” as well as some specific pathways including “cGMP–PKG signaling pathway,” “Cardiac muscle contraction,” “Calcium signaling pathway,” and “Adrenergic signaling in cardiomyocytes” were enriched based on co-location analyses. Thus, TUCPs can serve as important therapeutic targets for sepsis-induced myocardial depression. Further research is needed to confirm the roles of TUCPs in energy production and myocardial contraction, and to evaluate their value as therapeutic targets.

As for the GO and KEGG analyses based on co-expression, TUCPs were also proved to participate in sepsis-induced myocardial depression. Considering that the metabolism in sepsis differs from that under physiological conditions, clarifying how metabolism is regulated in this disease could provide new insight into its pathogenesis. The GO terms “Regulation of metabolic process,” “Regulation of primary metabolic process,” “Positive regulation of metabolic process,” and “Regulation of cellular metabolic process” were enriched, suggesting that TUCPs could affect the metabolic process in sepsis and play a regulatory role in this disease. Besides, some specific pathways including “TNF signaling pathway,” “NF-kappa B signaling pathway,” “Jak–STAT signaling pathway,” and “Apoptosis” were enriched based on KEGG analyses. Although reported studies [21–24] have stated that these four common pathways play potential roles in sepsis, the regulatory relationship between TUCPs and these pathways is still unclear, and should be analyzed in more depth in future basic studies.

Although our study is the first to illustrate the potential role of TUCPs in sepsis-induced myocardial depression, several limitations should be noticed and need future studies to solve these questions. We only identify potential impact of TUCPs through analysis at RNA expression level and there is no proteomic analysis of protein expression levels to confirm these associated proteins are actually affected. Therefore, in the futhur study, we will choose several specific TUCPs and demonstrate the causal relationship between the TUCPs and their expression of these potential target genes, aiming to confirm the regulatory role of TUCPs in sepsis-induced myocardial depression. Additionally, preclinical findings from rodents have not always borne out in human studies, and we will perform relative studies to confirm the validity of our results in human patients. What’s more, we didn’t explore the possibility of TUCPs to encode peptides. This could be a potential mechanism of action of the TUCPs, and in this case both TUCP and peptide could have their own biological activity in sepsis-induced myocardial depression. The furture research will evaluate the possibility of several specific TUCPs to encode peptides and thus validate their functions in the disease.

## Conclusion

This is the first study to investigate the role of TUCPs in the pathological process of myocardial depression in a model of septic shock. We analyzed the TUCPs differentially expressed between sepsis-induced myocardial depression and a control group using RNA-seq. Our findings expand our knowledge about the roles of TUCPs in myocardial depression. Further bioinformatic analyses led to the proposal that TUCPs play a key role in the pathogenesis of sepsis-induced myocardial depression. Study of TUCPs may provide new insights into the pathophysiology of sepsis-induced myocardial depression from a novel perspective, as well as potentially providing therapeutic targets in sepsis-induced myocardial depression.

## Supplementary Information

**Additional file 1.** **Figure S1:** The chromosome distribution of the DE TUCPs. DE, differential expression.

**Additional file 2.** **Table S1:** Detailed information regarding each TUCP in sepsis group and control group.

**Additional file 3.** **Table S2:** Detailed information regarding the sequence of each TUCP.

**Additional file 4.** **Table S3:** Detailed information on the 85 DE TUCPs between sepsis group and control group. DE, differential expression.

**Additional file 5.** **Table S4:** Detailed information on all the co-located genes for each DE TUCP.

**Additional file 6.** **Table 5:** Detailed information on all the co-expressed genes for each DE TUCP.

**Additional file 7.** **Table S6:** Detailed information GO terms based on co-location analysis. Table Note: CAD_item, the number of co-located genes in this GO term; CAD_list, all co-located genes number of GO terms; Bg_item, total gene number of the specific GO term; Bg_list, total gene number of all GO terms

**Additional file 8.** **Table S7:** Detailed information GO terms based on co-expression analysis. Table Note: CAD_item, the DE gene number in this GO term; CAD_list, all DE gene number of GO terms; Bg_item, total gene number of the specific GO term; Bg_list, total gene number of all GO terms.

**Additional file 9.** **Table S8:** Detailed information KEGG pathways based on co-location analysis.

**Additional file 10.** **Table S9:** Detailed information KEGG pathways based on co-location analysis.

## Data Availability

Sequencing data were deposited with the NCBI Sequence Read Archive (SRA) under accession number PRJNA527717 (https://www.ncbi.nlm.nih.gov/sra/?term=PRJNA527717).
