# Supplementary figures and images for: Comparative transcriptome analysis of transcripts of uncertain coding potential in septic myocardial depression
Source: BMC Cardiovasc Disord. 2021 Apr 8;21:166. doi: 10.1186/s12872-021-01973-z (PMC8028820; doi:10.1186/s12872-021-01973-z)

DE TUCP\_Sepsis vs Control

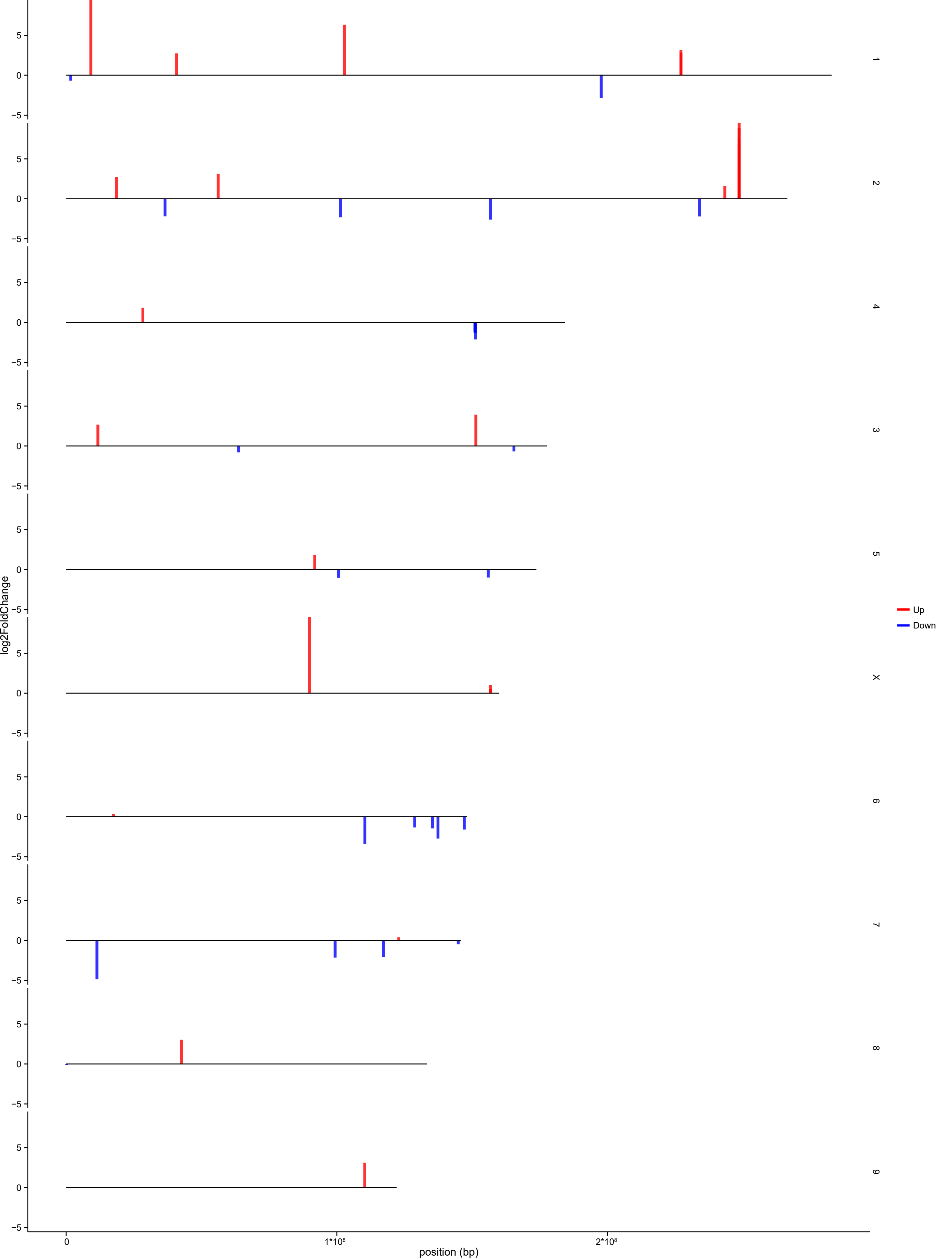

Supplement: Supplementary file 1 — Additional file 1. Figure S1: The chromosome distribution of the DE TUCPs. DE, differential expression. [file 12872_2021_1973_MOESM1_ESM.pdf]
