# Supplementary material for: Comparative transcriptome analysis of transcripts of uncertain coding potential in septic myocardial depression
Source: BMC Cardiovasc Disord. 2021 Apr 8;21:166. doi: 10.1186/s12872-021-01973-z (PMC8028820; doi:10.1186/s12872-021-01973-z)
Supplement: Supplementary file 6 — Additional file 6. Table 5: Detailed information on all the co-expressed genes for each DE TUCP. [file 12872_2021_1973_MOESM6_ESM.pdf]

TUCP_ID	TUCP_Gene_ID	mRNA_ID	Pearson_correlation	Pvalue
TUCP_001017	XLOC_038541	ENSRNOT00000035840	0.955299206	1.30E-06
TUCP_001017	XLOC_038541	ENSRNOT00000015961	0.960269422	7.29E-07
TUCP_003185	XLOC_137338	ENSRNOT00000085195	0.999404969	5.87E-16
TUCP_003185	XLOC_137338	ENSRNOT00000013705	1	0
TUCP_003185	XLOC_137338	ENSRNOT00000040851	1	0
TUCP_003185	XLOC_137338	ENSRNOT00000075676	0.958697783	8.83E-07
TUCP_003185	XLOC_137338	ENSRNOT00000058389	1	0
TUCP_003185	XLOC_137338	ENSRNOT00000086827	0.95592964	1.22E-06
TUCP_003185	XLOC_137338	ENSRNOT00000023542	1	0
TUCP_003185	XLOC_137338	ENSRNOT00000028653	1	0
TUCP_003185	XLOC_137338	ENSRNOT00000017230	0.955378945	1.29E-06
TUCP_003185	XLOC_137338	ENSRNOT00000092890	0.955137645	1.33E-06
TUCP_003185	XLOC_137338	ENSRNOT00000012174	1	0
TUCP_003185	XLOC_137338	ENSRNOT00000051680	0.994991197	2.46E-11
TUCP_003185	XLOC_137338	ENSRNOT00000089118	1	0
TUCP_003185	XLOC_137338	ENSRNOT00000025245	0.999967061	3.05E-22
TUCP_003185	XLOC_137338	ENSRNOT00000015138	0.999902179	7.05E-20
TUCP_003185	XLOC_137338	ENSRNOT00000080185	0.99902259	7.01E-15
TUCP_003185	XLOC_137338	ENSRNOT00000090729	0.965956282	3.40E-07
TUCP_003185	XLOC_137338	ENSRNOT00000049165	0.973440588	9.95E-08
TUCP_003185	XLOC_137338	ENSRNOT00000086502	0.96495109	3.93E-07
TUCP_003185	XLOC_137338	ENSRNOT00000079661	1	0
TUCP_003185	XLOC_137338	ENSRNOT00000076338	0.985532463	4.87E-09
TUCP_003185	XLOC_137338	ENSRNOT00000042220	1	1.33E-79
TUCP_003185	XLOC_137338	ENSRNOT00000005031	0.952974465	1.67E-06
TUCP_003185	XLOC_137338	ENSRNOT00000079167	0.979848988	2.53E-08
TUCP_003185	XLOC_137338	ENSRNOT00000061950	0.962035217	5.83E-07
TUCP_003185	XLOC_137338	ENSRNOT00000086710	0.999839781	8.31E-19
TUCP_003185	XLOC_137338	ENSRNOT00000075226	1	0
TUCP_003185	XLOC_137338	ENSRNOT00000000351	1	0
TUCP_003185	XLOC_137338	ENSRNOT00000092294	0.986520908	3.43E-09
TUCP_000923	XLOC_033936	ENSRNOT00000061253	0.950930897	2.06E-06
TUCP_000922	XLOC_033920	ENSRNOT00000010835	0.964313549	4.29E-07
TUCP_000922	XLOC_033920	ENSRNOT00000054980	0.951589561	1.93E-06
TUCP_000922	XLOC_033920	ENSRNOT00000057965	0.951549555	1.94E-06
TUCP_000922	XLOC_033920	ENSRNOT00000031193	0.959362675	8.15E-07
TUCP_000922	XLOC_033920	ENSRNOT00000091282	-0.965324966	3.72E-07
TUCP_000922	XLOC_033920	ENSRNOT00000038546	-0.953043641	1.66E-06
TUCP_000922	XLOC_033920	ENSRNOT00000030749	-0.956007458	1.21E-06
TUCP_000922	XLOC_033920	ENSRNOT00000021578	0.953513432	1.58E-06
TUCP_000922	XLOC_033920	ENSRNOT00000078856	-0.950253517	2.21E-06
TUCP_000922	XLOC_033920	ENSRNOT00000033359	0.954959516	1.35E-06
TUCP_000922	XLOC_033920	ENSRNOT00000001679	0.957420939	1.03E-06
TUCP_000922	XLOC_033920	ENSRNOT00000074212	0.974396065	8.30E-08
TUCP_000922	XLOC_033920	ENSRNOT00000021729	0.951649463	1.92E-06
TUCP_000922	XLOC_033920	ENSRNOT00000015643	0.960110985	7.44E-07
TUCP_000922	XLOC_033920	ENSRNOT00000082570	-0.957711876	9.92E-07
TUCP_000922	XLOC_033920	ENSRNOT00000005143	0.956887446	1.09E-06
TUCP_000922	XLOC_033920	ENSRNOT00000012314	-0.95451652	1.42E-06
TUCP_000922	XLOC_033920	ENSRNOT00000030109	0.961896382	5.93E-07

TUCP_000922	XLOC_033920	ENSRNOT00000009319	-0.950248239	2.21E-06
TUCP_000922	XLOC_033920	ENSRNOT00000013191	-0.951144467	2.02E-06
TUCP_000922	XLOC_033920	ENSRNOT00000028066	-0.953421758	1.60E-06
TUCP_000922	XLOC_033920	ENSRNOT00000044726	0.95933282	8.18E-07
TUCP_000922	XLOC_033920	ENSRNOT00000010344	-0.951051829	2.04E-06
TUCP_002714	XLOC_114926	ENSRNOT00000032386	0.963455163	4.83E-07
TUCP_000284	XLOC_008664	ENSRNOT00000042890	0.961965737	5.88E-07
TUCP_000926	XLOC_033948	ENSRNOT00000022384	-0.951077333	2.03E-06
TUCP_004698	XLOC_206559	ENSRNOT00000014041	0.98792666	1.98E-09
TUCP_000928	XLOC_034230	ENSRNOT00000090123	0.972592216	1.16E-07
TUCP_000928	XLOC_034230	ENSRNOT00000026633	0.954846941	1.37E-06
TUCP_000928	XLOC_034230	ENSRNOT00000071764	0.952947223	1.68E-06
TUCP_000928	XLOC_034230	ENSRNOT00000032780	0.956135416	1.19E-06
TUCP_000928	XLOC_034230	ENSRNOT00000016991	0.968910624	2.17E-07
TUCP_000928	XLOC_034230	ENSRNOT00000055669	0.951731561	1.90E-06
TUCP_000928	XLOC_034230	ENSRNOT00000081047	0.952123828	1.83E-06
TUCP_000928	XLOC_034230	ENSRNOT00000014537	0.954433263	1.43E-06
TUCP_000928	XLOC_034230	ENSRNOT00000072518	0.95097453	2.05E-06
TUCP_000928	XLOC_034230	ENSRNOT00000051132	0.953398188	1.60E-06
TUCP_000928	XLOC_034230	ENSRNOT00000003891	0.967767032	2.60E-07
TUCP_000928	XLOC_034230	ENSRNOT00000021408	0.956958851	1.08E-06
TUCP_000928	XLOC_034230	ENSRNOT00000077406	0.961953158	5.89E-07
TUCP_000928	XLOC_034230	ENSRNOT00000025656	0.967743052	2.61E-07
TUCP_000928	XLOC_034230	ENSRNOT00000035474	0.951247765	2.00E-06
TUCP_000928	XLOC_034230	ENSRNOT00000021037	0.95610159	1.19E-06
TUCP_000928	XLOC_034230	ENSRNOT00000076872	0.954472656	1.43E-06
TUCP_000928	XLOC_034230	ENSRNOT00000060819	0.972228709	1.24E-07
TUCP_000928	XLOC_034230	ENSRNOT00000093720	0.952214982	1.81E-06
TUCP_000928	XLOC_034230	ENSRNOT00000005490	0.973062603	1.07E-07
TUCP_000928	XLOC_034230	ENSRNOT00000000468	0.95360545	1.57E-06
TUCP_000928	XLOC_034230	ENSRNOT00000090918	0.955619125	1.26E-06
TUCP_000928	XLOC_034230	ENSRNOT00000046973	0.950044122	2.25E-06
TUCP_000928	XLOC_034230	ENSRNOT00000012187	0.950066027	2.25E-06
TUCP_000928	XLOC_034230	ENSRNOT00000064798	0.953980147	1.50E-06
TUCP_000928	XLOC_034230	ENSRNOT00000015609	0.960055882	7.49E-07
TUCP_000928	XLOC_034230	ENSRNOT00000024969	0.951137576	2.02E-06
TUCP_000928	XLOC_034230	ENSRNOT00000022485	0.961672164	6.11E-07
TUCP_000928	XLOC_034230	ENSRNOT00000033235	0.95317526	1.64E-06
TUCP_000928	XLOC_034230	ENSRNOT00000060362	0.960132598	7.42E-07
TUCP_000928	XLOC_034230	ENSRNOT00000010399	0.958070988	9.51E-07
TUCP_000928	XLOC_034230	ENSRNOT00000072132	0.963778878	4.62E-07
TUCP_000928	XLOC_034230	ENSRNOT00000083220	0.952280675	1.80E-06
TUCP_000928	XLOC_034230	ENSRNOT00000015265	0.968289249	2.39E-07
TUCP_000928	XLOC_034230	ENSRNOT00000042498	0.963416251	4.85E-07
TUCP_000928	XLOC_034230	ENSRNOT00000015793	0.954150125	1.48E-06
TUCP_000928	XLOC_034230	ENSRNOT00000015874	0.955184913	1.32E-06
TUCP_000928	XLOC_034230	ENSRNOT00000038066	0.958613239	8.92E-07
TUCP_000928	XLOC_034230	ENSRNOT00000015799	0.950689242	2.11E-06
TUCP_000928	XLOC_034230	ENSRNOT00000078102	0.955798162	1.23E-06
TUCP_000884	XLOC_032870	ENSRNOT00000051450	0.951207148	2.01E-06
TUCP_000884	XLOC_032870	ENSRNOT00000087753	0.954617148	1.40E-06

TUCP_004008	XLOC_175736	ENSRNOT00000015576	0.950730572	2.10E-06
TUCP_004008	XLOC_175736	ENSRNOT00000019262	-0.955247127	1.31E-06
TUCP_004008	XLOC_175736	ENSRNOT00000087190	0.954755764	1.38E-06
TUCP_004008	XLOC_175736	ENSRNOT00000013053	0.9688552	2.19E-07
TUCP_004008	XLOC_175736	ENSRNOT00000092211	0.960186075	7.37E-07
TUCP_004008	XLOC_175736	ENSRNOT00000073079	0.950784584	2.09E-06
TUCP_004008	XLOC_175736	ENSRNOT00000044452	0.961848455	5.97E-07
TUCP_004008	XLOC_175736	ENSRNOT00000021055	-0.950354454	2.18E-06
TUCP_004008	XLOC_175736	ENSRNOT00000090654	0.951371106	1.97E-06
TUCP_004008	XLOC_175736	ENSRNOT00000077935	0.960801094	6.82E-07
TUCP_000887	XLOC_033296	ENSRNOT00000075521	0.950836336	2.08E-06
TUCP_000887	XLOC_033296	ENSRNOT00000090456	0.957873573	9.73E-07
TUCP_000887	XLOC_033296	ENSRNOT00000006186	0.960587992	7.01E-07
TUCP_000887	XLOC_033296	ENSRNOT00000048910	-0.953967769	1.51E-06
TUCP_000887	XLOC_033296	ENSRNOT00000020357	0.951838539	1.88E-06
TUCP_000887	XLOC_033296	ENSRNOT00000019104	-0.956114974	1.19E-06
TUCP_000887	XLOC_033296	ENSRNOT00000010728	0.962848228	5.24E-07
TUCP_000887	XLOC_033296	ENSRNOT00000082863	0.972009346	1.29E-07
TUCP_000887	XLOC_033296	ENSRNOT00000005659	0.959131212	8.38E-07
TUCP_000887	XLOC_033296	ENSRNOT00000000718	0.964824473	4.00E-07
TUCP_000887	XLOC_033296	ENSRNOT00000007855	0.963786032	4.62E-07
TUCP_000887	XLOC_033296	ENSRNOT00000052063	0.968634124	2.27E-07
TUCP_000887	XLOC_033296	ENSRNOT00000056813	0.963050225	5.10E-07
TUCP_000887	XLOC_033296	ENSRNOT00000018074	-0.954905659	1.36E-06
TUCP_000887	XLOC_033296	ENSRNOT00000084069	0.951956899	1.86E-06
TUCP_000887	XLOC_033296	ENSRNOT00000012187	0.950640534	2.12E-06
TUCP_000887	XLOC_033296	ENSRNOT00000050868	0.959815113	7.71E-07
TUCP_000887	XLOC_033296	ENSRNOT00000006055	0.965829428	3.46E-07
TUCP_000887	XLOC_033296	ENSRNOT00000005126	-0.959904007	7.63E-07
TUCP_000887	XLOC_033296	ENSRNOT00000065300	0.961444739	6.29E-07
TUCP_000887	XLOC_033296	ENSRNOT00000025079	-0.962494096	5.49E-07
TUCP_000887	XLOC_033296	ENSRNOT00000038247	0.964067575	4.44E-07
TUCP_000887	XLOC_033296	ENSRNOT00000012977	0.954067168	1.49E-06
TUCP_000887	XLOC_033296	ENSRNOT00000091448	-0.957769618	9.85E-07
TUCP_000887	XLOC_033296	ENSRNOT00000009811	0.959721271	7.80E-07
TUCP_000887	XLOC_033296	ENSRNOT00000068134	0.955839899	1.23E-06
TUCP_000887	XLOC_033296	ENSRNOT00000026718	0.958041028	9.54E-07
TUCP_000887	XLOC_033296	ENSRNOT00000000577	0.970531051	1.67E-07
TUCP_000887	XLOC_033296	ENSRNOT00000032780	0.960418779	7.16E-07
TUCP_000887	XLOC_033296	ENSRNOT00000077231	0.971567821	1.40E-07
TUCP_000887	XLOC_033296	ENSRNOT00000072518	0.951431712	1.96E-06
TUCP_000887	XLOC_033296	ENSRNOT00000016333	-0.963779247	4.62E-07
TUCP_000887	XLOC_033296	ENSRNOT00000021523	0.953546244	1.58E-06
TUCP_000887	XLOC_033296	ENSRNOT00000051991	0.950270491	2.20E-06
TUCP_000887	XLOC_033296	ENSRNOT00000006835	0.9800512	2.41E-08
TUCP_000887	XLOC_033296	ENSRNOT00000010141	0.96300167	5.13E-07
TUCP_000887	XLOC_033296	ENSRNOT00000022502	-0.950085002	2.24E-06
TUCP_000887	XLOC_033296	ENSRNOT00000044346	-0.968471511	2.33E-07
TUCP_000887	XLOC_033296	ENSRNOT00000037133	0.950346999	2.19E-06
TUCP_000887	XLOC_033296	ENSRNOT00000068633	0.950852534	2.08E-06
TUCP_000887	XLOC_033296	ENSRNOT00000037446	0.958247887	9.31E-07

TUCP_000887	XLOC_033296	ENSRNOT00000087443	0.957923211	9.68E-07
TUCP_000887	XLOC_033296	ENSRNOT00000000036	0.970891992	1.57E-07
TUCP_000887	XLOC_033296	ENSRNOT00000026481	0.961959109	5.89E-07
TUCP_000887	XLOC_033296	ENSRNOT00000021408	0.955585029	1.26E-06
TUCP_000887	XLOC_033296	ENSRNOT00000015116	0.952880781	1.69E-06
TUCP_000887	XLOC_033296	ENSRNOT00000001081	0.95748511	1.02E-06
TUCP_000887	XLOC_033296	ENSRNOT00000086119	-0.962449854	5.52E-07
TUCP_000887	XLOC_033296	ENSRNOT00000067735	-0.966875687	2.97E-07
TUCP_000887	XLOC_033296	ENSRNOT00000022190	-0.950296049	2.20E-06
TUCP_000887	XLOC_033296	ENSRNOT00000022552	-0.9555363	1.27E-06
TUCP_000887	XLOC_033296	ENSRNOT00000019133	0.97298062	1.08E-07
TUCP_000887	XLOC_033296	ENSRNOT00000065983	0.958202753	9.36E-07
TUCP_000887	XLOC_033296	ENSRNOT00000030782	-0.952450995	1.77E-06
TUCP_000887	XLOC_033296	ENSRNOT00000079698	-0.957933595	9.67E-07
TUCP_000887	XLOC_033296	ENSRNOT00000006824	-0.950620422	2.13E-06
TUCP_000887	XLOC_033296	ENSRNOT00000061267	0.957331972	1.04E-06
TUCP_000887	XLOC_033296	ENSRNOT00000080207	0.971665951	1.37E-07
TUCP_000887	XLOC_033296	ENSRNOT00000090461	0.951231037	2.00E-06
TUCP_000887	XLOC_033296	ENSRNOT00000064588	0.950051254	2.25E-06
TUCP_000887	XLOC_033296	ENSRNOT00000086590	0.951379249	1.97E-06
TUCP_000887	XLOC_033296	ENSRNOT00000016445	0.967379261	2.75E-07
TUCP_000887	XLOC_033296	ENSRNOT00000021496	-0.95032123	2.19E-06
TUCP_000887	XLOC_033296	ENSRNOT00000077555	0.96426184	4.32E-07
TUCP_000887	XLOC_033296	ENSRNOT00000011880	0.961280176	6.42E-07
TUCP_000887	XLOC_033296	ENSRNOT00000034961	-0.952402299	1.78E-06
TUCP_000887	XLOC_033296	ENSRNOT00000006034	-0.950325564	2.19E-06
TUCP_000887	XLOC_033296	ENSRNOT00000087429	0.969438264	1.99E-07
TUCP_000887	XLOC_033296	ENSRNOT00000078070	0.958051833	9.53E-07
TUCP_000887	XLOC_033296	ENSRNOT00000025434	0.965434611	3.67E-07
TUCP_000887	XLOC_033296	ENSRNOT00000012424	0.98109721	1.84E-08
TUCP_000887	XLOC_033296	ENSRNOT00000020363	0.968081348	2.47E-07
TUCP_000887	XLOC_033296	ENSRNOT00000083247	-0.960467673	7.12E-07
TUCP_000887	XLOC_033296	ENSRNOT00000008427	-0.957447273	1.02E-06
TUCP_000887	XLOC_033296	ENSRNOT00000077999	0.954867261	1.37E-06
TUCP_000887	XLOC_033296	ENSRNOT00000067610	-0.958873077	8.65E-07
TUCP_000887	XLOC_033296	ENSRNOT00000032505	-0.978593677	3.42E-08
TUCP_000887	XLOC_033296	ENSRNOT00000027344	0.957952915	9.64E-07
TUCP_000887	XLOC_033296	ENSRNOT00000029833	0.953636427	1.56E-06
TUCP_000887	XLOC_033296	ENSRNOT00000012702	-0.951196197	2.01E-06
TUCP_000887	XLOC_033296	ENSRNOT00000034487	-0.953538923	1.58E-06
TUCP_000887	XLOC_033296	ENSRNOT00000028057	0.953435697	1.59E-06
TUCP_000887	XLOC_033296	ENSRNOT00000009691	0.968239464	2.41E-07
TUCP_000887	XLOC_033296	ENSRNOT00000020417	-0.961561656	6.20E-07
TUCP_000887	XLOC_033296	ENSRNOT00000019554	0.969087635	2.11E-07
TUCP_000887	XLOC_033296	ENSRNOT00000066133	-0.959333681	8.18E-07
TUCP_000887	XLOC_033296	ENSRNOT00000029202	-0.95063747	2.12E-06
TUCP_000887	XLOC_033296	ENSRNOT00000011984	0.961211712	6.48E-07
TUCP_000887	XLOC_033296	ENSRNOT00000005489	0.952251109	1.80E-06
TUCP_000887	XLOC_033296	ENSRNOT00000026293	0.955683829	1.25E-06
TUCP_000887	XLOC_033296	ENSRNOT00000019487	0.984790549	6.25E-09
TUCP_000887	XLOC_033296	ENSRNOT00000080602	0.963923677	4.53E-07

TUCP_000887	XLOC_033296	ENSRNOT00000054828	0.950775375	2.10E-06
TUCP_000887	XLOC_033296	ENSRNOT00000001923	0.963875479	4.56E-07
TUCP_000887	XLOC_033296	ENSRNOT00000079002	-0.952750737	1.71E-06
TUCP_000887	XLOC_033296	ENSRNOT00000055992	-0.966468216	3.16E-07
TUCP_000887	XLOC_033296	ENSRNOT00000010218	0.960841334	6.79E-07
TUCP_000887	XLOC_033296	ENSRNOT00000052422	0.959523143	7.99E-07
TUCP_000887	XLOC_033296	ENSRNOT00000055386	0.970695057	1.62E-07
TUCP_000887	XLOC_033296	ENSRNOT00000011466	0.952857636	1.69E-06
TUCP_000887	XLOC_033296	ENSRNOT00000084709	0.957198042	1.05E-06
TUCP_000887	XLOC_033296	ENSRNOT00000037679	0.96433141	4.28E-07
TUCP_000887	XLOC_033296	ENSRNOT00000014124	0.950357556	2.18E-06
TUCP_000887	XLOC_033296	ENSRNOT00000078906	-0.952469685	1.76E-06
TUCP_000887	XLOC_033296	ENSRNOT00000028888	0.952697286	1.72E-06
TUCP_000887	XLOC_033296	ENSRNOT00000013538	0.9551258	1.33E-06
TUCP_000887	XLOC_033296	ENSRNOT00000010986	0.975608835	6.53E-08
TUCP_000887	XLOC_033296	ENSRNOT00000060698	-0.95482377	1.37E-06
TUCP_000887	XLOC_033296	ENSRNOT00000058351	0.954671063	1.40E-06
TUCP_000887	XLOC_033296	ENSRNOT00000020990	-0.952988279	1.67E-06
TUCP_000887	XLOC_033296	ENSRNOT00000023330	0.964128277	4.40E-07
TUCP_000887	XLOC_033296	ENSRNOT00000061100	0.952550951	1.75E-06
TUCP_000887	XLOC_033296	ENSRNOT00000005387	0.95055173	2.14E-06
TUCP_000887	XLOC_033296	ENSRNOT00000083414	0.952745022	1.71E-06
TUCP_000887	XLOC_033296	ENSRNOT00000016401	0.972627833	1.16E-07
TUCP_000887	XLOC_033296	ENSRNOT00000025097	0.984070081	7.87E-09
TUCP_000887	XLOC_033296	ENSRNOT00000026457	0.974044448	8.88E-08
TUCP_000887	XLOC_033296	ENSRNOT00000067901	-0.958933129	8.59E-07
TUCP_000887	XLOC_033296	ENSRNOT00000044009	0.952331855	1.79E-06
TUCP_000887	XLOC_033296	ENSRNOT00000036613	-0.955890122	1.22E-06
TUCP_000887	XLOC_033296	ENSRNOT00000044628	0.953853782	1.52E-06
TUCP_000887	XLOC_033296	ENSRNOT00000066793	-0.952055074	1.84E-06
TUCP_000887	XLOC_033296	ENSRNOT00000078862	0.96268049	5.35E-07
TUCP_000887	XLOC_033296	ENSRNOT00000003989	0.95220467	1.81E-06
TUCP_000887	XLOC_033296	ENSRNOT00000000337	0.951882741	1.87E-06
TUCP_000887	XLOC_033296	ENSRNOT00000079996	0.950710553	2.11E-06
TUCP_000887	XLOC_033296	ENSRNOT00000024548	0.969761908	1.89E-07
TUCP_003758	XLOC_165125	ENSRNOT00000020297	0.957712625	9.92E-07
TUCP_003758	XLOC_165125	ENSRNOT00000014946	0.953522675	1.58E-06
TUCP_003758	XLOC_165125	ENSRNOT00000090621	0.977432526	4.44E-08
TUCP_003758	XLOC_165125	ENSRNOT00000075397	0.952087896	1.83E-06
TUCP_003758	XLOC_165125	ENSRNOT00000007350	0.973700748	9.48E-08
TUCP_003758	XLOC_165125	ENSRNOT00000068353	0.963247336	4.96E-07
TUCP_003758	XLOC_165125	ENSRNOT00000091399	0.963913376	4.54E-07
TUCP_003758	XLOC_165125	ENSRNOT00000058382	0.971952333	1.30E-07
TUCP_003758	XLOC_165125	ENSRNOT00000033437	0.984258624	7.41E-09
TUCP_003758	XLOC_165125	ENSRNOT00000020910	0.958459306	9.08E-07
TUCP_003758	XLOC_165125	ENSRNOT00000084567	0.957005175	1.08E-06
TUCP_003758	XLOC_165125	ENSRNOT00000045926	0.950481363	2.16E-06
TUCP_003758	XLOC_165125	ENSRNOT00000010419	0.950760683	2.10E-06
TUCP_003758	XLOC_165125	ENSRNOT00000002028	0.953729929	1.55E-06
TUCP_003758	XLOC_165125	ENSRNOT00000016306	-0.950140711	2.23E-06
TUCP_003758	XLOC_165125	ENSRNOT00000087604	0.952795064	1.70E-06

TUCP_003758	XLOC_165125	ENSRNOT00000037409	0.966754767	3.02E-07
TUCP_003758	XLOC_165125	ENSRNOT00000083568	0.957510449	1.02E-06
TUCP_003758	XLOC_165125	ENSRNOT00000026141	0.953378222	1.60E-06
TUCP_003758	XLOC_165125	ENSRNOT00000036137	0.954629661	1.40E-06
TUCP_003758	XLOC_165125	ENSRNOT00000065978	0.972281704	1.23E-07
TUCP_003758	XLOC_165125	ENSRNOT00000075480	-0.963700242	4.67E-07
TUCP_003758	XLOC_165125	ENSRNOT00000027760	0.972374907	1.21E-07
TUCP_003758	XLOC_165125	ENSRNOT00000047234	0.961268889	6.43E-07
TUCP_003758	XLOC_165125	ENSRNOT00000065871	0.950991853	2.05E-06
TUCP_003758	XLOC_165125	ENSRNOT00000056100	-0.951331293	1.98E-06
TUCP_003758	XLOC_165125	ENSRNOT00000065045	0.957241173	1.05E-06
TUCP_003758	XLOC_165125	ENSRNOT00000005297	0.974557565	8.05E-08
TUCP_003758	XLOC_165125	ENSRNOT00000066708	0.956017747	1.20E-06
TUCP_003758	XLOC_165125	ENSRNOT00000011832	-0.960233048	7.33E-07
TUCP_003758	XLOC_165125	ENSRNOT00000073643	-0.953707416	1.55E-06
TUCP_003758	XLOC_165125	ENSRNOT00000087278	-0.960718359	6.90E-07
TUCP_003758	XLOC_165125	ENSRNOT00000015678	-0.953575905	1.57E-06
TUCP_003758	XLOC_165125	ENSRNOT00000011930	0.961132849	6.54E-07
TUCP_003758	XLOC_165125	ENSRNOT00000028089	0.961913173	5.92E-07
TUCP_003758	XLOC_165125	ENSRNOT00000033806	0.950592902	2.13E-06
TUCP_003758	XLOC_165125	ENSRNOT00000009150	0.971391883	1.44E-07
TUCP_003758	XLOC_165125	ENSRNOT00000028828	0.952166203	1.82E-06
TUCP_003758	XLOC_165125	ENSRNOT00000048781	0.964426132	4.23E-07
TUCP_003758	XLOC_165125	ENSRNOT00000072299	0.954665216	1.40E-06
TUCP_003758	XLOC_165125	ENSRNOT00000085334	0.95757395	1.01E-06
TUCP_003758	XLOC_165125	ENSRNOT00000001403	0.957211122	1.05E-06
TUCP_003758	XLOC_165125	ENSRNOT00000014506	-0.962555679	5.44E-07
TUCP_003758	XLOC_165125	ENSRNOT00000076230	0.956258378	1.17E-06
TUCP_003758	XLOC_165125	ENSRNOT00000066952	0.957590619	1.01E-06
TUCP_003758	XLOC_165125	ENSRNOT00000035427	0.952710571	1.72E-06
TUCP_003758	XLOC_165125	ENSRNOT00000036658	-0.962603579	5.41E-07
TUCP_003758	XLOC_165125	ENSRNOT00000089611	0.95607831	1.20E-06
TUCP_003758	XLOC_165125	ENSRNOT00000023667	0.965192479	3.80E-07
TUCP_003758	XLOC_165125	ENSRNOT00000082138	-0.955185192	1.32E-06
TUCP_003758	XLOC_165125	ENSRNOT00000079138	0.964572865	4.14E-07
TUCP_003758	XLOC_165125	ENSRNOT00000027739	0.958501487	9.04E-07
TUCP_003758	XLOC_165125	ENSRNOT00000034883	-0.951333213	1.98E-06
TUCP_003758	XLOC_165125	ENSRNOT00000064983	0.953993256	1.50E-06
TUCP_003758	XLOC_165125	ENSRNOT00000035605	0.959162646	8.35E-07
TUCP_003758	XLOC_165125	ENSRNOT00000081351	0.964411756	4.23E-07
TUCP_003758	XLOC_165125	ENSRNOT00000046102	-0.950533464	2.15E-06
TUCP_003758	XLOC_165125	ENSRNOT00000092083	0.954869342	1.37E-06
TUCP_003758	XLOC_165125	ENSRNOT00000032324	0.964687686	4.08E-07
TUCP_003758	XLOC_165125	ENSRNOT00000017624	0.979525532	2.74E-08
TUCP_003758	XLOC_165125	ENSRNOT00000036092	0.9504441	2.17E-06
TUCP_003758	XLOC_165125	ENSRNOT00000085954	0.953801891	1.53E-06
TUCP_003758	XLOC_165125	ENSRNOT00000040169	0.976164994	5.82E-08
TUCP_003758	XLOC_165125	ENSRNOT00000015091	0.955977182	1.21E-06
TUCP_003758	XLOC_165125	ENSRNOT00000037015	0.954980274	1.35E-06
TUCP_003758	XLOC_165125	ENSRNOT00000001490	0.966410538	3.18E-07
TUCP_003758	XLOC_165125	ENSRNOT00000011675	0.95810737	9.47E-07

TUCP_003758	XLOC_165125	ENSRNOT00000080591	0.960080163	7.47E-07
TUCP_003758	XLOC_165125	ENSRNOT00000015239	-0.972121531	1.27E-07
TUCP_003758	XLOC_165125	ENSRNOT00000074649	0.951157867	2.02E-06
TUCP_003758	XLOC_165125	ENSRNOT00000023256	0.950076083	2.25E-06
TUCP_003758	XLOC_165125	ENSRNOT00000077146	-0.95589674	1.22E-06
TUCP_003758	XLOC_165125	ENSRNOT00000075209	-0.962955692	5.16E-07
TUCP_003758	XLOC_165125	ENSRNOT00000025966	0.974188169	8.64E-08
TUCP_003758	XLOC_165125	ENSRNOT00000003765	0.950242178	2.21E-06
TUCP_003758	XLOC_165125	ENSRNOT00000042799	0.952964153	1.68E-06
TUCP_003758	XLOC_165125	ENSRNOT00000010993	0.955217664	1.32E-06
TUCP_003758	XLOC_165125	ENSRNOT00000017765	0.962705971	5.34E-07
TUCP_003758	XLOC_165125	ENSRNOT00000057738	0.97791774	3.98E-08
TUCP_003758	XLOC_165125	ENSRNOT00000023014	0.959090205	8.42E-07
TUCP_003758	XLOC_165125	ENSRNOT00000015537	0.960772263	6.85E-07
TUCP_003758	XLOC_165125	ENSRNOT00000025009	0.954401881	1.44E-06
TUCP_003758	XLOC_165125	ENSRNOT00000039102	0.951784973	1.89E-06
TUCP_003758	XLOC_165125	ENSRNOT00000032106	0.974792409	7.68E-08
TUCP_003758	XLOC_165125	ENSRNOT00000024306	-0.953781103	1.54E-06
TUCP_003758	XLOC_165125	ENSRNOT00000000120	0.954571174	1.41E-06
TUCP_003758	XLOC_165125	ENSRNOT00000074150	0.967635648	2.65E-07
TUCP_003758	XLOC_165125	ENSRNOT00000012528	0.954234023	1.46E-06
TUCP_003758	XLOC_165125	ENSRNOT00000061345	-0.962742729	5.31E-07
TUCP_003758	XLOC_165125	ENSRNOT00000016568	0.975011538	7.36E-08
TUCP_003758	XLOC_165125	ENSRNOT00000077503	0.95660938	1.13E-06
TUCP_003758	XLOC_165125	ENSRNOT00000025384	0.954485471	1.42E-06
TUCP_003758	XLOC_165125	ENSRNOT00000051494	0.95763963	1.00E-06
TUCP_003758	XLOC_165125	ENSRNOT00000028907	0.953348342	1.61E-06
TUCP_003758	XLOC_165125	ENSRNOT00000027967	0.969399088	2.01E-07
TUCP_003758	XLOC_165125	ENSRNOT00000025147	0.957860335	9.75E-07
TUCP_003758	XLOC_165125	ENSRNOT00000002358	-0.951303504	1.99E-06
TUCP_003758	XLOC_165125	ENSRNOT00000036613	-0.958178912	9.39E-07
TUCP_003758	XLOC_165125	ENSRNOT00000020929	0.951646433	1.92E-06
TUCP_003758	XLOC_165125	ENSRNOT00000008113	-0.961328022	6.38E-07
TUCP_000883	XLOC_032621	ENSRNOT00000074990	0.990229428	6.90E-10
TUCP_000883	XLOC_032621	ENSRNOT00000068444	0.990229428	6.90E-10
TUCP_000883	XLOC_032621	ENSRNOT00000081370	0.991083531	4.37E-10
TUCP_000883	XLOC_032621	ENSRNOT00000078645	0.978366353	3.60E-08
TUCP_000883	XLOC_032621	ENSRNOT00000075987	0.972129558	1.26E-07
TUCP_000883	XLOC_032621	ENSRNOT00000082043	0.985280584	5.31E-09
TUCP_000883	XLOC_032621	ENSRNOT00000087323	0.976129685	5.86E-08
TUCP_000883	XLOC_032621	ENSRNOT00000074930	0.990229428	6.90E-10
TUCP_000883	XLOC_032621	ENSRNOT00000080575	0.991283946	3.90E-10
TUCP_000883	XLOC_032621	ENSRNOT00000057761	0.968048377	2.49E-07
TUCP_000883	XLOC_032621	ENSRNOT00000078369	0.971709911	1.36E-07
TUCP_000883	XLOC_032621	ENSRNOT00000092982	0.990229428	6.90E-10
TUCP_000883	XLOC_032621	ENSRNOT00000088367	0.990229428	6.90E-10
TUCP_000883	XLOC_032621	ENSRNOT00000061874	0.975215923	7.06E-08
TUCP_000883	XLOC_032621	ENSRNOT00000079606	0.975399839	6.81E-08
TUCP_000883	XLOC_032621	ENSRNOT00000081996	0.990229428	6.90E-10
TUCP_000883	XLOC_032621	ENSRNOT00000064751	0.964567329	4.14E-07
TUCP_000883	XLOC_032621	ENSRNOT00000022280	0.983985498	8.08E-09

TUCP_000883	XLOC_032621	ENSRNOT00000082058	0.990229428	6.90E-10
TUCP_000883	XLOC_032621	ENSRNOT00000088905	0.990229428	6.90E-10
TUCP_000883	XLOC_032621	ENSRNOT00000086831	0.990229428	6.90E-10
TUCP_000883	XLOC_032621	ENSRNOT00000045099	0.990229428	6.90E-10
TUCP_000883	XLOC_032621	ENSRNOT00000086745	0.954312933	1.45E-06
TUCP_000883	XLOC_032621	ENSRNOT00000085849	0.969187649	2.08E-07
TUCP_000883	XLOC_032621	ENSRNOT00000086397	0.964540084	4.16E-07
TUCP_000883	XLOC_032621	ENSRNOT00000077789	0.980120168	2.37E-08
TUCP_000883	XLOC_032621	ENSRNOT00000073828	0.955295474	1.30E-06
TUCP_000883	XLOC_032621	ENSRNOT00000087025	0.990229428	6.90E-10
TUCP_000883	XLOC_032621	ENSRNOT00000019853	0.990229428	6.90E-10
TUCP_000883	XLOC_032621	ENSRNOT00000073853	0.973898268	9.13E-08
TUCP_000883	XLOC_032621	ENSRNOT00000065912	0.975424932	6.77E-08
TUCP_000883	XLOC_032621	ENSRNOT00000078683	0.963823915	4.59E-07
TUCP_000883	XLOC_032621	ENSRNOT00000093083	0.991522302	3.40E-10
TUCP_003755	XLOC_164804	ENSRNOT00000084500	0.984887141	6.05E-09
TUCP_003755	XLOC_164804	ENSRNOT00000093467	0.996134431	6.75E-12
TUCP_003755	XLOC_164804	ENSRNOT00000029340	0.999116572	4.23E-15
TUCP_003755	XLOC_164804	ENSRNOT00000077972	0.962758032	5.30E-07
TUCP_003755	XLOC_164804	ENSRNOT00000038646	1	0
TUCP_003755	XLOC_164804	ENSRNOT00000012095	0.957750287	9.88E-07
TUCP_003755	XLOC_164804	ENSRNOT00000017909	0.975587192	6.56E-08
TUCP_003755	XLOC_164804	ENSRNOT00000092579	1	4.25E-78
TUCP_003755	XLOC_164804	ENSRNOT00000089235	0.998661162	3.38E-14
TUCP_003755	XLOC_164804	ENSRNOT00000036383	0.988460809	1.58E-09
TUCP_003755	XLOC_164804	ENSRNOT00000076528	0.997115373	1.57E-12
TUCP_003755	XLOC_164804	ENSRNOT00000075193	0.964326971	4.29E-07
TUCP_003755	XLOC_164804	ENSRNOT00000083034	0.990934646	4.75E-10
TUCP_003755	XLOC_164804	ENSRNOT00000089607	0.995091455	2.23E-11
TUCP_003755	XLOC_164804	ENSRNOT00000086014	0.960893638	6.74E-07
TUCP_003755	XLOC_164804	ENSRNOT00000054939	0.958021495	9.57E-07
TUCP_003755	XLOC_164804	ENSRNOT00000052315	0.983687082	8.85E-09
TUCP_003755	XLOC_164804	ENSRNOT00000088168	1	0
TUCP_003755	XLOC_164804	ENSRNOT00000046231	0.96225463	5.66E-07
TUCP_003755	XLOC_164804	ENSRNOT00000013459	0.979098017	3.03E-08
TUCP_003755	XLOC_164804	ENSRNOT00000078137	1	0
TUCP_003755	XLOC_164804	ENSRNOT00000077764	0.977143528	4.73E-08
TUCP_003755	XLOC_164804	ENSRNOT00000043661	0.966975951	2.93E-07
TUCP_003755	XLOC_164804	ENSRNOT00000092740	1	0
TUCP_003755	XLOC_164804	ENSRNOT00000000117	0.990989174	4.61E-10
TUCP_003755	XLOC_164804	ENSRNOT00000071877	0.967113583	2.87E-07
TUCP_003755	XLOC_164804	ENSRNOT00000070958	1	0
TUCP_003755	XLOC_164804	ENSRNOT00000076757	0.959205354	8.31E-07
TUCP_003755	XLOC_164804	ENSRNOT00000085310	0.965230243	3.78E-07
TUCP_003755	XLOC_164804	ENSRNOT00000047241	0.966576993	3.11E-07
TUCP_003755	XLOC_164804	ENSRNOT00000027268	1	1.33E-79
TUCP_003755	XLOC_164804	ENSRNOT00000004459	1	0
TUCP_003755	XLOC_164804	ENSRNOT00000048373	0.959096578	8.42E-07
TUCP_003755	XLOC_164804	ENSRNOT00000061516	0.987595648	2.27E-09
TUCP_003755	XLOC_164804	ENSRNOT00000088148	1	0
TUCP_003755	XLOC_164804	ENSRNOT00000088527	1	0

TUCP_003755	XLOC_164804	ENSRNOT00000078701	1	0
TUCP_003755	XLOC_164804	ENSRNOT00000052308	0.962646127	5.38E-07
TUCP_003755	XLOC_164804	ENSRNOT00000021748	1	0
TUCP_003755	XLOC_164804	ENSRNOT00000089085	1	0
TUCP_003755	XLOC_164804	ENSRNOT00000008153	0.999703506	1.80E-17
TUCP_003755	XLOC_164804	ENSRNOT00000085896	0.971605382	1.39E-07
TUCP_003755	XLOC_164804	ENSRNOT00000079189	0.984972387	5.89E-09
TUCP_003755	XLOC_164804	ENSRNOT00000090078	1	0
TUCP_003755	XLOC_164804	ENSRNOT00000078020	0.958421666	9.13E-07
TUCP_003755	XLOC_164804	ENSRNOT00000092425	0.956092273	1.19E-06
TUCP_003755	XLOC_164804	ENSRNOT00000009776	1	1.33E-79
TUCP_003120	XLOC_133211	ENSRNOT00000043172	0.952635447	1.73E-06
TUCP_003120	XLOC_133211	ENSRNOT00000055259	0.973567049	9.72E-08
TUCP_003120	XLOC_133211	ENSRNOT00000049822	0.952298189	1.80E-06
TUCP_003756	XLOC_164891	ENSRNOT00000082982	0.951624326	1.92E-06
TUCP_003756	XLOC_164891	ENSRNOT00000019057	0.960371374	7.20E-07
TUCP_003756	XLOC_164891	ENSRNOT00000024933	0.951744	1.90E-06
TUCP_003756	XLOC_164891	ENSRNOT00000067011	-0.962189803	5.71E-07
TUCP_003756	XLOC_164891	ENSRNOT00000000804	0.966942697	2.94E-07
TUCP_003756	XLOC_164891	ENSRNOT00000002247	0.964899619	3.96E-07
TUCP_003756	XLOC_164891	ENSRNOT00000010030	0.952566367	1.75E-06
TUCP_000888	XLOC_033315	ENSRNOT00000081297	0.958468355	9.07E-07
TUCP_000888	XLOC_033315	ENSRNOT00000023196	0.953189007	1.64E-06
TUCP_000888	XLOC_033315	ENSRNOT00000003177	0.957969002	9.63E-07
TUCP_000888	XLOC_033315	ENSRNOT00000011633	0.966655346	3.07E-07
TUCP_000888	XLOC_033315	ENSRNOT00000024021	0.969574202	1.95E-07
TUCP_000888	XLOC_033315	ENSRNOT00000007491	0.95012342	2.24E-06
TUCP_000888	XLOC_033315	ENSRNOT00000016859	0.954245257	1.46E-06
TUCP_000888	XLOC_033315	ENSRNOT00000080271	0.96156307	6.19E-07
TUCP_000888	XLOC_033315	ENSRNOT00000040731	0.950252166	2.21E-06
TUCP_000888	XLOC_033315	ENSRNOT00000059744	0.956750789	1.11E-06
TUCP_000889	XLOC_033326	ENSRNOT00000006796	0.955929731	1.22E-06
TUCP_000285	XLOC_008666	ENSRNOT00000005190	0.957370117	1.03E-06
TUCP_000285	XLOC_008666	ENSRNOT00000042890	0.962395203	5.56E-07
TUCP_003234	XLOC_138628	ENSRNOT00000023060	0.952201684	1.81E-06
TUCP_003234	XLOC_138628	ENSRNOT00000080124	0.957625773	1.00E-06
TUCP_003234	XLOC_138628	ENSRNOT00000085120	0.954513797	1.42E-06
TUCP_003234	XLOC_138628	ENSRNOT00000077409	0.9539852	1.50E-06
TUCP_001151	XLOC_043177	ENSRNOT00000030102	0.960938109	6.71E-07
TUCP_001151	XLOC_043177	ENSRNOT00000008148	0.963867234	4.56E-07
TUCP_001151	XLOC_043177	ENSRNOT00000090451	-0.953947358	1.51E-06
TUCP_001151	XLOC_043177	ENSRNOT00000011659	0.96031514	7.25E-07
TUCP_001151	XLOC_043177	ENSRNOT00000041908	0.960113445	7.44E-07
TUCP_001151	XLOC_043177	ENSRNOT00000064752	-0.963287355	4.94E-07
TUCP_003861	XLOC_169474	ENSRNOT00000076404	0.962479165	5.50E-07
TUCP_003861	XLOC_169474	ENSRNOT00000090944	0.957609875	1.00E-06
TUCP_003861	XLOC_169474	ENSRNOT00000028458	0.953422808	1.60E-06
TUCP_003861	XLOC_169474	ENSRNOT00000089226	0.95590626	1.22E-06
TUCP_003861	XLOC_169474	ENSRNOT00000013260	0.964903248	3.95E-07
TUCP_003861	XLOC_169474	ENSRNOT00000014627	0.952712649	1.72E-06
TUCP_003861	XLOC_169474	ENSRNOT00000087138	0.950481958	2.16E-06

TUCP_003861	XLOC_169474	ENSRNOT00000010609	0.972802109	1.12E-07
TUCP_003861	XLOC_169474	ENSRNOT00000006670	0.958283798	9.28E-07
TUCP_003861	XLOC_169474	ENSRNOT00000071998	0.959448755	8.07E-07
TUCP_003861	XLOC_169474	ENSRNOT00000085517	0.955060185	1.34E-06
TUCP_003861	XLOC_169474	ENSRNOT00000005144	0.952114658	1.83E-06
TUCP_003861	XLOC_169474	ENSRNOT00000003840	0.953291728	1.62E-06
TUCP_003861	XLOC_169474	ENSRNOT00000079464	0.950723985	2.11E-06
TUCP_003861	XLOC_169474	ENSRNOT00000008971	0.969148853	2.09E-07
TUCP_003861	XLOC_169474	ENSRNOT00000012528	0.950906111	2.07E-06
TUCP_003861	XLOC_169474	ENSRNOT00000078675	0.950479212	2.16E-06
TUCP_003861	XLOC_169474	ENSRNOT00000027170	0.96127822	6.42E-07
TUCP_003861	XLOC_169474	ENSRNOT00000011956	0.955697306	1.25E-06
TUCP_003861	XLOC_169474	ENSRNOT00000012755	0.960338674	7.23E-07
TUCP_003861	XLOC_169474	ENSRNOT00000037790	0.957360563	1.03E-06
TUCP_003861	XLOC_169474	ENSRNOT00000092952	0.952849833	1.70E-06
TUCP_003861	XLOC_169474	ENSRNOT00000074054	0.982318659	1.32E-08
TUCP_003861	XLOC_169474	ENSRNOT00000016967	0.951717384	1.91E-06
TUCP_001969	XLOC_079634	ENSRNOT00000039729	0.964624714	4.11E-07
TUCP_001969	XLOC_079634	ENSRNOT00000040165	0.969068361	2.12E-07
TUCP_001969	XLOC_079634	ENSRNOT00000027891	0.950152486	2.23E-06
TUCP_001969	XLOC_079634	ENSRNOT00000032782	-0.974357206	8.36E-08
TUCP_001969	XLOC_079634	ENSRNOT00000013053	0.951691141	1.91E-06
TUCP_001969	XLOC_079634	ENSRNOT00000030344	0.961209527	6.48E-07
TUCP_001969	XLOC_079634	ENSRNOT00000017232	0.954154404	1.48E-06
TUCP_001969	XLOC_079634	ENSRNOT00000028887	0.95230979	1.79E-06
TUCP_001969	XLOC_079634	ENSRNOT00000005647	-0.956731736	1.11E-06
TUCP_001969	XLOC_079634	ENSRNOT00000022253	0.966767774	3.02E-07
TUCP_001969	XLOC_079634	ENSRNOT00000011134	0.95771477	9.92E-07
TUCP_001969	XLOC_079634	ENSRNOT00000059487	0.955614257	1.26E-06
TUCP_001969	XLOC_079634	ENSRNOT00000025308	0.965763842	3.50E-07
TUCP_001969	XLOC_079634	ENSRNOT00000024313	-0.955805161	1.23E-06
TUCP_001969	XLOC_079634	ENSRNOT00000082645	0.968233065	2.42E-07
TUCP_001969	XLOC_079634	ENSRNOT00000065288	0.96492742	3.94E-07
TUCP_001969	XLOC_079634	ENSRNOT00000007688	0.971620557	1.38E-07
TUCP_001969	XLOC_079634	ENSRNOT00000011344	0.955101897	1.33E-06
TUCP_001969	XLOC_079634	ENSRNOT00000009840	0.950962211	2.06E-06
TUCP_001969	XLOC_079634	ENSRNOT00000035048	0.965302796	3.74E-07
TUCP_001969	XLOC_079634	ENSRNOT00000011481	0.961949777	5.89E-07
TUCP_001969	XLOC_079634	ENSRNOT00000086459	0.957547177	1.01E-06
TUCP_001969	XLOC_079634	ENSRNOT00000015247	0.95027419	2.20E-06
TUCP_001969	XLOC_079634	ENSRNOT00000073952	0.950714643	2.11E-06
TUCP_001969	XLOC_079634	ENSRNOT00000044866	0.959552745	7.97E-07
TUCP_001969	XLOC_079634	ENSRNOT00000048770	0.964898703	3.96E-07
TUCP_001969	XLOC_079634	ENSRNOT00000024568	0.952215417	1.81E-06
TUCP_001969	XLOC_079634	ENSRNOT00000089185	0.950274325	2.20E-06
TUCP_001969	XLOC_079634	ENSRNOT00000023728	0.952646814	1.73E-06
TUCP_001969	XLOC_079634	ENSRNOT00000002936	0.953292478	1.62E-06
TUCP_003245	XLOC_139083	ENSRNOT00000033695	-0.979370531	2.84E-08
TUCP_003245	XLOC_139083	ENSRNOT00000087619	0.981141155	1.82E-08
TUCP_003245	XLOC_139083	ENSRNOT00000093462	0.983634103	9.00E-09
TUCP_003245	XLOC_139083	ENSRNOT00000057872	0.971047592	1.53E-07

TUCP_000282	XLOC_008659	ENSRNOT00000042890	0.968569626	2.29E-07
TUCP_000282	XLOC_008659	ENSRNOT00000086144	0.959173336	8.34E-07
TUCP_000282	XLOC_008659	ENSRNOT00000065111	0.960740945	6.88E-07
TUCP_002561	XLOC_108796	ENSRNOT00000084075	0.984165458	7.63E-09
TUCP_002561	XLOC_108796	ENSRNOT00000015366	0.960330587	7.24E-07
TUCP_002561	XLOC_108796	ENSRNOT00000012880	0.950889973	2.07E-06
TUCP_002561	XLOC_108796	ENSRNOT00000074465	0.988682795	1.43E-09
TUCP_002561	XLOC_108796	ENSRNOT00000079805	0.985425193	5.05E-09
TUCP_002561	XLOC_108796	ENSRNOT00000040912	0.988682795	1.43E-09
TUCP_002561	XLOC_108796	ENSRNOT00000072648	0.988682795	1.43E-09
TUCP_002561	XLOC_108796	ENSRNOT00000007398	0.976576144	5.34E-08
TUCP_002561	XLOC_108796	ENSRNOT00000070803	0.988682795	1.43E-09
TUCP_002561	XLOC_108796	ENSRNOT00000012196	0.988682795	1.43E-09
TUCP_002561	XLOC_108796	ENSRNOT00000085772	0.988682795	1.43E-09
TUCP_002561	XLOC_108796	ENSRNOT00000093284	0.98860631	1.48E-09
TUCP_002561	XLOC_108796	ENSRNOT00000086374	0.985950835	4.21E-09
TUCP_002561	XLOC_108796	ENSRNOT00000015270	0.988682795	1.43E-09
TUCP_002561	XLOC_108796	ENSRNOT00000046192	0.988682795	1.43E-09
TUCP_002561	XLOC_108796	ENSRNOT00000044226	0.988682795	1.43E-09
TUCP_002561	XLOC_108796	ENSRNOT00000081898	0.983462197	9.48E-09
TUCP_002561	XLOC_108796	ENSRNOT00000080291	0.986732571	3.17E-09
TUCP_002561	XLOC_108796	ENSRNOT00000064601	0.965189694	3.80E-07
TUCP_002561	XLOC_108796	ENSRNOT00000004293	0.983072938	1.06E-08
TUCP_002561	XLOC_108796	ENSRNOT00000078231	0.955117062	1.33E-06
TUCP_002561	XLOC_108796	ENSRNOT00000015870	0.974340677	8.39E-08
TUCP_002561	XLOC_108796	ENSRNOT00000001464	0.988682795	1.43E-09
TUCP_002561	XLOC_108796	ENSRNOT00000090716	0.983713397	8.78E-09
TUCP_002561	XLOC_108796	ENSRNOT00000083542	0.958682288	8.85E-07
TUCP_002561	XLOC_108796	ENSRNOT00000076044	0.988682795	1.43E-09
TUCP_002561	XLOC_108796	ENSRNOT00000074353	0.988682795	1.43E-09
TUCP_002561	XLOC_108796	ENSRNOT00000087122	0.961896925	5.93E-07
TUCP_002561	XLOC_108796	ENSRNOT00000074295	0.991280276	3.91E-10
TUCP_002561	XLOC_108796	ENSRNOT00000057873	0.988682795	1.43E-09
TUCP_002561	XLOC_108796	ENSRNOT00000083002	0.973093414	1.06E-07
TUCP_002561	XLOC_108796	ENSRNOT00000088399	0.988708228	1.42E-09
TUCP_002561	XLOC_108796	ENSRNOT00000086248	0.988682795	1.43E-09
TUCP_002561	XLOC_108796	ENSRNOT00000089750	0.988669143	1.44E-09
TUCP_002561	XLOC_108796	ENSRNOT00000030517	0.962535772	5.46E-07
TUCP_002561	XLOC_108796	ENSRNOT00000034772	0.979870674	2.52E-08
TUCP_002561	XLOC_108796	ENSRNOT00000089305	0.958326039	9.23E-07
TUCP_002561	XLOC_108796	ENSRNOT00000078121	0.957735519	9.89E-07
TUCP_002561	XLOC_108796	ENSRNOT00000002834	0.988682795	1.43E-09
TUCP_002561	XLOC_108796	ENSRNOT00000080812	0.985978675	4.17E-09
TUCP_002561	XLOC_108796	ENSRNOT00000092755	0.988682795	1.43E-09
TUCP_002561	XLOC_108796	ENSRNOT00000031683	0.988682795	1.43E-09
TUCP_002561	XLOC_108796	ENSRNOT00000016843	0.953242851	1.63E-06
TUCP_002561	XLOC_108796	ENSRNOT00000087764	0.966389369	3.19E-07
TUCP_002560	XLOC_108796	ENSRNOT00000065143	0.965993981	3.38E-07
TUCP_002560	XLOC_108796	ENSRNOT00000044885	0.952934959	1.68E-06
TUCP_002560	XLOC_108796	ENSRNOT00000086590	0.959476826	8.04E-07
TUCP_002560	XLOC_108796	ENSRNOT00000006186	0.961405493	6.32E-07

TUCP_002560	XLOC_108796	ENSRNOT00000082998	0.97119187	1.49E-07
TUCP_002560	XLOC_108796	ENSRNOT00000081246	0.968618304	2.27E-07
TUCP_002560	XLOC_108796	ENSRNOT00000048910	-0.956275097	1.17E-06
TUCP_002560	XLOC_108796	ENSRNOT00000027083	0.955414189	1.29E-06
TUCP_002560	XLOC_108796	ENSRNOT00000009877	0.95604004	1.20E-06
TUCP_002560	XLOC_108796	ENSRNOT00000007351	-0.967924325	2.53E-07
TUCP_002560	XLOC_108796	ENSRNOT00000019104	-0.953666539	1.56E-06
TUCP_002560	XLOC_108796	ENSRNOT00000021348	0.952967559	1.67E-06
TUCP_002560	XLOC_108796	ENSRNOT00000063930	-0.952141901	1.82E-06
TUCP_002560	XLOC_108796	ENSRNOT00000005958	0.987988376	1.93E-09
TUCP_002560	XLOC_108796	ENSRNOT00000082508	-0.9675966	2.66E-07
TUCP_002560	XLOC_108796	ENSRNOT00000003605	-0.950810892	2.09E-06
TUCP_002560	XLOC_108796	ENSRNOT00000023638	0.98128994	1.75E-08
TUCP_002560	XLOC_108796	ENSRNOT00000009563	-0.958942034	8.58E-07
TUCP_002560	XLOC_108796	ENSRNOT00000059524	-0.9574466	1.02E-06
TUCP_002560	XLOC_108796	ENSRNOT00000082863	0.959347323	8.17E-07
TUCP_002560	XLOC_108796	ENSRNOT00000028628	-0.95783346	9.78E-07
TUCP_002560	XLOC_108796	ENSRNOT00000084709	0.958071683	9.51E-07
TUCP_002560	XLOC_108796	ENSRNOT00000017361	0.957199916	1.05E-06
TUCP_002560	XLOC_108796	ENSRNOT00000005220	0.959031678	8.48E-07
TUCP_002560	XLOC_108796	ENSRNOT00000023144	0.963410292	4.86E-07
TUCP_002560	XLOC_108796	ENSRNOT00000049459	-0.950373991	2.18E-06
TUCP_002560	XLOC_108796	ENSRNOT00000045326	0.967586006	2.67E-07
TUCP_002560	XLOC_108796	ENSRNOT00000071166	0.960110474	7.44E-07
TUCP_002560	XLOC_108796	ENSRNOT00000016649	0.966687281	3.06E-07
TUCP_002560	XLOC_108796	ENSRNOT00000086310	-0.95480991	1.38E-06
TUCP_002560	XLOC_108796	ENSRNOT00000090342	0.978315216	3.64E-08
TUCP_002560	XLOC_108796	ENSRNOT00000028856	0.968275451	2.40E-07
TUCP_002560	XLOC_108796	ENSRNOT00000000743	0.958986979	8.53E-07
TUCP_002560	XLOC_108796	ENSRNOT00000004140	0.955624751	1.26E-06
TUCP_002560	XLOC_108796	ENSRNOT00000065300	0.96314795	5.03E-07
TUCP_002560	XLOC_108796	ENSRNOT00000030850	0.963459887	4.82E-07
TUCP_002560	XLOC_108796	ENSRNOT00000067170	-0.974675492	7.86E-08
TUCP_002560	XLOC_108796	ENSRNOT00000043216	0.964369777	4.26E-07
TUCP_002560	XLOC_108796	ENSRNOT00000091128	-0.976683186	5.22E-08
TUCP_002560	XLOC_108796	ENSRNOT00000001586	-0.964080267	4.43E-07
TUCP_002560	XLOC_108796	ENSRNOT00000081981	0.994332183	4.56E-11
TUCP_002560	XLOC_108796	ENSRNOT00000086893	-0.952011813	1.85E-06
TUCP_002560	XLOC_108796	ENSRNOT00000026543	0.957914748	9.69E-07
TUCP_002560	XLOC_108796	ENSRNOT00000083369	0.957908425	9.69E-07
TUCP_002560	XLOC_108796	ENSRNOT00000006752	0.97007639	1.80E-07
TUCP_002560	XLOC_108796	ENSRNOT00000004172	0.973847254	9.22E-08
TUCP_002560	XLOC_108796	ENSRNOT00000014979	0.960160827	7.39E-07
TUCP_002560	XLOC_108796	ENSRNOT00000019213	0.954236832	1.46E-06
TUCP_002560	XLOC_108796	ENSRNOT00000077201	-0.955394814	1.29E-06
TUCP_002560	XLOC_108796	ENSRNOT00000018533	0.966929971	2.95E-07
TUCP_002560	XLOC_108796	ENSRNOT00000073564	-0.955926994	1.22E-06
TUCP_002560	XLOC_108796	ENSRNOT00000024969	0.978808603	3.25E-08
TUCP_002560	XLOC_108796	ENSRNOT00000085251	-0.958873521	8.65E-07
TUCP_002560	XLOC_108796	ENSRNOT00000001507	0.951716922	1.91E-06
TUCP_002560	XLOC_108796	ENSRNOT00000026783	0.961903254	5.93E-07

TUCP_002560	XLOC_108796	ENSRNOT00000042125	0.983380064	9.71E-09
TUCP_002560	XLOC_108796	ENSRNOT00000028646	0.956978306	1.08E-06
TUCP_002560	XLOC_108796	ENSRNOT00000025079	-0.954017776	1.50E-06
TUCP_002560	XLOC_108796	ENSRNOT00000048754	-0.970918583	1.56E-07
TUCP_002560	XLOC_108796	ENSRNOT00000045699	0.952761637	1.71E-06
TUCP_002560	XLOC_108796	ENSRNOT00000087443	0.965548094	3.61E-07
TUCP_002560	XLOC_108796	ENSRNOT00000029439	0.957469685	1.02E-06
TUCP_002560	XLOC_108796	ENSRNOT00000045285	0.95331625	1.61E-06
TUCP_002560	XLOC_108796	ENSRNOT00000002443	0.961331165	6.38E-07
TUCP_002560	XLOC_108796	ENSRNOT00000084031	0.952031294	1.85E-06
TUCP_002560	XLOC_108796	ENSRNOT00000035320	-0.955035572	1.34E-06
TUCP_002560	XLOC_108796	ENSRNOT00000076820	0.953347146	1.61E-06
TUCP_002560	XLOC_108796	ENSRNOT00000071764	0.955230663	1.31E-06
TUCP_002560	XLOC_108796	ENSRNOT00000079109	0.955946129	1.21E-06
TUCP_002560	XLOC_108796	ENSRNOT00000024402	0.956883403	1.09E-06
TUCP_002560	XLOC_108796	ENSRNOT00000004007	-0.963880248	4.56E-07
TUCP_002560	XLOC_108796	ENSRNOT00000020681	-0.95126333	1.99E-06
TUCP_002560	XLOC_108796	ENSRNOT00000002837	-0.956593103	1.13E-06
TUCP_002560	XLOC_108796	ENSRNOT00000026174	0.951692873	1.91E-06
TUCP_002560	XLOC_108796	ENSRNOT00000019519	0.965339865	3.72E-07
TUCP_002560	XLOC_108796	ENSRNOT00000089933	0.979770172	2.58E-08
TUCP_002560	XLOC_108796	ENSRNOT00000077908	0.954595701	1.41E-06
TUCP_002560	XLOC_108796	ENSRNOT00000091602	-0.964904347	3.95E-07
TUCP_002560	XLOC_108796	ENSRNOT00000037456	0.96245213	5.52E-07
TUCP_002560	XLOC_108796	ENSRNOT00000080608	0.981421115	1.69E-08
TUCP_002560	XLOC_108796	ENSRNOT00000090123	0.964996769	3.90E-07
TUCP_002560	XLOC_108796	ENSRNOT00000026481	0.964049396	4.45E-07
TUCP_002560	XLOC_108796	ENSRNOT00000065168	0.951589133	1.93E-06
TUCP_002560	XLOC_108796	ENSRNOT00000005490	0.95763279	1.00E-06
TUCP_002560	XLOC_108796	ENSRNOT00000067554	0.952818053	1.70E-06
TUCP_002560	XLOC_108796	ENSRNOT00000072208	0.961952871	5.89E-07
TUCP_002560	XLOC_108796	ENSRNOT00000032780	0.982557703	1.23E-08
TUCP_002560	XLOC_108796	ENSRNOT00000009708	-0.981678474	1.58E-08
TUCP_002560	XLOC_108796	ENSRNOT00000074323	0.951321633	1.98E-06
TUCP_002560	XLOC_108796	ENSRNOT00000064535	-0.955804206	1.23E-06
TUCP_002560	XLOC_108796	ENSRNOT00000015188	0.973177021	1.05E-07
TUCP_002560	XLOC_108796	ENSRNOT00000033677	-0.96599092	3.38E-07
TUCP_002560	XLOC_108796	ENSRNOT00000079866	0.961948469	5.89E-07
TUCP_002560	XLOC_108796	ENSRNOT00000072518	0.966990938	2.92E-07
TUCP_002560	XLOC_108796	ENSRNOT00000023133	0.958284457	9.27E-07
TUCP_002560	XLOC_108796	ENSRNOT00000085279	0.963584873	4.74E-07
TUCP_002560	XLOC_108796	ENSRNOT00000004046	-0.979087328	3.04E-08
TUCP_002560	XLOC_108796	ENSRNOT00000011499	-0.958253271	9.31E-07
TUCP_002560	XLOC_108796	ENSRNOT00000061392	0.979597217	2.69E-08
TUCP_002560	XLOC_108796	ENSRNOT00000038247	0.961294647	6.41E-07
TUCP_002560	XLOC_108796	ENSRNOT00000002410	0.960302986	7.26E-07
TUCP_002560	XLOC_108796	ENSRNOT00000010927	0.957750353	9.87E-07
TUCP_002560	XLOC_108796	ENSRNOT00000037039	-0.954744525	1.39E-06
TUCP_002560	XLOC_108796	ENSRNOT00000021030	-0.964514841	4.17E-07
TUCP_002560	XLOC_108796	ENSRNOT00000091909	0.967033494	2.90E-07
TUCP_002560	XLOC_108796	ENSRNOT00000015006	0.95544661	1.28E-06

TUCP_002560	XLOC_108796	ENSRNOT00000077727	-0.954383227	1.44E-06
TUCP_002560	XLOC_108796	ENSRNOT00000022502	-0.953754891	1.54E-06
TUCP_002560	XLOC_108796	ENSRNOT00000044346	-0.954652208	1.40E-06
TUCP_002560	XLOC_108796	ENSRNOT00000024119	0.955250821	1.31E-06
TUCP_002560	XLOC_108796	ENSRNOT00000010541	0.954592968	1.41E-06
TUCP_002560	XLOC_108796	ENSRNOT00000017556	0.969529571	1.97E-07
TUCP_002560	XLOC_108796	ENSRNOT00000004032	0.96299678	5.13E-07
TUCP_002560	XLOC_108796	ENSRNOT00000084385	0.965654803	3.55E-07
TUCP_002560	XLOC_108796	ENSRNOT00000047192	-0.973382977	1.01E-07
TUCP_002560	XLOC_108796	ENSRNOT00000015479	0.952124916	1.83E-06
TUCP_002560	XLOC_108796	ENSRNOT00000090912	0.950924276	2.06E-06
TUCP_002560	XLOC_108796	ENSRNOT00000044562	0.962086311	5.79E-07
TUCP_002560	XLOC_108796	ENSRNOT00000021803	-0.965300055	3.74E-07
TUCP_002560	XLOC_108796	ENSRNOT00000001081	0.977207452	4.66E-08
TUCP_002560	XLOC_108796	ENSRNOT00000005228	0.96805964	2.48E-07
TUCP_002560	XLOC_108796	ENSRNOT00000061304	-0.951099981	2.03E-06
TUCP_002560	XLOC_108796	ENSRNOT00000083220	0.974834153	7.62E-08
TUCP_002560	XLOC_108796	ENSRNOT00000025983	0.951889047	1.87E-06
TUCP_002560	XLOC_108796	ENSRNOT00000086119	-0.968034415	2.49E-07
TUCP_002560	XLOC_108796	ENSRNOT00000016722	0.956983062	1.08E-06
TUCP_002560	XLOC_108796	ENSRNOT00000022190	-0.956001301	1.21E-06
TUCP_002560	XLOC_108796	ENSRNOT00000016132	-0.963715396	4.66E-07
TUCP_002560	XLOC_108796	ENSRNOT00000000120	0.970774262	1.60E-07
TUCP_002560	XLOC_108796	ENSRNOT00000058312	0.965831744	3.46E-07
TUCP_002560	XLOC_108796	ENSRNOT00000019133	0.97685495	5.03E-08
TUCP_002560	XLOC_108796	ENSRNOT00000065983	0.960894506	6.74E-07
TUCP_002560	XLOC_108796	ENSRNOT00000030782	-0.955535908	1.27E-06
TUCP_002560	XLOC_108796	ENSRNOT00000020437	0.955842881	1.23E-06
TUCP_002560	XLOC_108796	ENSRNOT00000079698	-0.953500416	1.58E-06
TUCP_002560	XLOC_108796	ENSRNOT00000067780	-0.956962658	1.08E-06
TUCP_002560	XLOC_108796	ENSRNOT00000015265	0.964917551	3.95E-07
TUCP_002560	XLOC_108796	ENSRNOT00000034692	-0.955307634	1.30E-06
TUCP_002560	XLOC_108796	ENSRNOT00000048063	0.950740775	2.10E-06
TUCP_002560	XLOC_108796	ENSRNOT00000052422	0.978721221	3.32E-08
TUCP_002560	XLOC_108796	ENSRNOT00000003787	-0.951141109	2.02E-06
TUCP_002560	XLOC_108796	ENSRNOT00000068381	0.963488801	4.81E-07
TUCP_002560	XLOC_108796	ENSRNOT00000031977	0.950724118	2.11E-06
TUCP_002560	XLOC_108796	ENSRNOT00000020910	0.95594387	1.21E-06
TUCP_002560	XLOC_108796	ENSRNOT00000031979	-0.958566219	8.97E-07
TUCP_002560	XLOC_108796	ENSRNOT00000015609	0.964077164	4.44E-07
TUCP_002560	XLOC_108796	ENSRNOT00000033975	0.952253027	1.80E-06
TUCP_002560	XLOC_108796	ENSRNOT00000004319	-0.966630729	3.08E-07
TUCP_002560	XLOC_108796	ENSRNOT00000078102	0.985397555	5.10E-09
TUCP_002560	XLOC_108796	ENSRNOT00000019820	0.973601435	9.66E-08
TUCP_002560	XLOC_108796	ENSRNOT00000000181	0.950409505	2.17E-06
TUCP_002560	XLOC_108796	ENSRNOT00000009811	0.977428703	4.44E-08
TUCP_002560	XLOC_108796	ENSRNOT00000010873	-0.951968931	1.86E-06
TUCP_002560	XLOC_108796	ENSRNOT00000087095	0.971430928	1.43E-07
TUCP_002560	XLOC_108796	ENSRNOT00000027526	0.966290689	3.24E-07
TUCP_002560	XLOC_108796	ENSRNOT00000005459	0.956462372	1.14E-06
TUCP_002560	XLOC_108796	ENSRNOT00000088599	0.960000016	7.54E-07

TUCP_002560	XLOC_108796	ENSRNOT00000055321	0.960856117	6.78E-07
TUCP_002560	XLOC_108796	ENSRNOT00000026531	-0.951797363	1.89E-06
TUCP_002560	XLOC_108796	ENSRNOT00000048933	-0.953058548	1.66E-06
TUCP_002560	XLOC_108796	ENSRNOT00000025067	-0.955113709	1.33E-06
TUCP_002560	XLOC_108796	ENSRNOT00000016129	0.953484089	1.59E-06
TUCP_002560	XLOC_108796	ENSRNOT00000081047	0.975384187	6.83E-08
TUCP_002560	XLOC_108796	ENSRNOT00000010030	-0.954639452	1.40E-06
TUCP_002560	XLOC_108796	ENSRNOT00000073559	0.971340083	1.45E-07
TUCP_002560	XLOC_108796	ENSRNOT00000011880	0.955905442	1.22E-06
TUCP_002560	XLOC_108796	ENSRNOT00000005073	0.951251396	2.00E-06
TUCP_002560	XLOC_108796	ENSRNOT00000005486	-0.963471561	4.82E-07
TUCP_002560	XLOC_108796	ENSRNOT00000023989	0.971509501	1.41E-07
TUCP_002560	XLOC_108796	ENSRNOT00000032248	0.954888322	1.36E-06
TUCP_002560	XLOC_108796	ENSRNOT00000003991	0.961325541	6.39E-07
TUCP_002560	XLOC_108796	ENSRNOT00000021408	0.967224715	2.82E-07
TUCP_002560	XLOC_108796	ENSRNOT00000019502	0.968049879	2.49E-07
TUCP_002560	XLOC_108796	ENSRNOT00000078475	0.954999226	1.35E-06
TUCP_002560	XLOC_108796	ENSRNOT00000089211	0.95517322	1.32E-06
TUCP_002560	XLOC_108796	ENSRNOT00000050515	0.971050539	1.53E-07
TUCP_002560	XLOC_108796	ENSRNOT00000013067	-0.957366511	1.03E-06
TUCP_002560	XLOC_108796	ENSRNOT00000041277	-0.952184803	1.82E-06
TUCP_002560	XLOC_108796	ENSRNOT00000080207	0.969471402	1.98E-07
TUCP_002560	XLOC_108796	ENSRNOT00000035591	-0.965911516	3.42E-07
TUCP_002560	XLOC_108796	ENSRNOT00000027910	0.978226518	3.72E-08
TUCP_002560	XLOC_108796	ENSRNOT00000060362	0.977258302	4.61E-08
TUCP_002560	XLOC_108796	ENSRNOT00000052224	0.968703111	2.24E-07
TUCP_002560	XLOC_108796	ENSRNOT00000078070	0.98057211	2.11E-08
TUCP_002560	XLOC_108796	ENSRNOT00000046745	0.982910458	1.12E-08
TUCP_002560	XLOC_108796	ENSRNOT00000021015	0.952238828	1.81E-06
TUCP_002560	XLOC_108796	ENSRNOT00000087502	0.960583852	7.01E-07
TUCP_002560	XLOC_108796	ENSRNOT00000024322	0.959314839	8.20E-07
TUCP_002560	XLOC_108796	ENSRNOT00000088182	0.965979908	3.39E-07
TUCP_002560	XLOC_108796	ENSRNOT00000061201	-0.953281473	1.62E-06
TUCP_002560	XLOC_108796	ENSRNOT00000000577	0.950461991	2.16E-06
TUCP_002560	XLOC_108796	ENSRNOT00000026643	0.954287452	1.46E-06
TUCP_002560	XLOC_108796	ENSRNOT00000057146	0.955751803	1.24E-06
TUCP_002560	XLOC_108796	ENSRNOT00000073526	0.957307586	1.04E-06
TUCP_002560	XLOC_108796	ENSRNOT00000010784	0.966324114	3.22E-07
TUCP_002560	XLOC_108796	ENSRNOT00000013801	0.952379111	1.78E-06
TUCP_002560	XLOC_108796	ENSRNOT00000021944	0.95028376	2.20E-06
TUCP_002560	XLOC_108796	ENSRNOT00000078390	-0.956136229	1.19E-06
TUCP_002560	XLOC_108796	ENSRNOT00000000013	0.977109765	4.76E-08
TUCP_002560	XLOC_108796	ENSRNOT00000077999	0.964101451	4.42E-07
TUCP_002560	XLOC_108796	ENSRNOT00000092029	0.968253934	2.41E-07
TUCP_002560	XLOC_108796	ENSRNOT00000025832	0.950040094	2.25E-06
TUCP_002560	XLOC_108796	ENSRNOT00000019656	-0.960396098	7.18E-07
TUCP_002560	XLOC_108796	ENSRNOT00000017181	-0.952287298	1.80E-06
TUCP_002560	XLOC_108796	ENSRNOT00000017753	0.980918069	1.93E-08
TUCP_002560	XLOC_108796	ENSRNOT00000051022	0.965158637	3.81E-07
TUCP_002560	XLOC_108796	ENSRNOT00000006499	0.959962174	7.58E-07
TUCP_002560	XLOC_108796	ENSRNOT00000005909	0.971199902	1.49E-07

TUCP_002560	XLOC_108796	ENSRNOT00000029125	0.991067586	4.41E-10
TUCP_002560	XLOC_108796	ENSRNOT00000074649	0.96839616	2.35E-07
TUCP_002560	XLOC_108796	ENSRNOT00000029833	0.978387866	3.58E-08
TUCP_002560	XLOC_108796	ENSRNOT00000034487	-0.950886316	2.07E-06
TUCP_002560	XLOC_108796	ENSRNOT00000031904	0.951476057	1.95E-06
TUCP_002560	XLOC_108796	ENSRNOT00000009129	0.950624899	2.13E-06
TUCP_002560	XLOC_108796	ENSRNOT00000017319	0.962941932	5.17E-07
TUCP_002560	XLOC_108796	ENSRNOT00000086122	0.955139664	1.33E-06
TUCP_002560	XLOC_108796	ENSRNOT00000022074	0.967181375	2.84E-07
TUCP_002560	XLOC_108796	ENSRNOT00000040069	0.97021145	1.76E-07
TUCP_002560	XLOC_108796	ENSRNOT00000036951	0.961437783	6.29E-07
TUCP_002560	XLOC_108796	ENSRNOT00000016519	0.96254773	5.45E-07
TUCP_002560	XLOC_108796	ENSRNOT00000000302	0.95385584	1.52E-06
TUCP_002560	XLOC_108796	ENSRNOT00000060002	-0.956235288	1.17E-06
TUCP_002560	XLOC_108796	ENSRNOT00000036959	-0.95897422	8.54E-07
TUCP_002560	XLOC_108796	ENSRNOT00000015799	0.981105112	1.84E-08
TUCP_002560	XLOC_108796	ENSRNOT00000075424	0.955498739	1.28E-06
TUCP_002560	XLOC_108796	ENSRNOT00000031401	-0.955696774	1.25E-06
TUCP_002560	XLOC_108796	ENSRNOT00000085228	0.98078982	2.00E-08
TUCP_002560	XLOC_108796	ENSRNOT00000078250	0.958040377	9.55E-07
TUCP_002560	XLOC_108796	ENSRNOT00000058842	0.969907604	1.85E-07
TUCP_002560	XLOC_108796	ENSRNOT00000002052	-0.950398213	2.18E-06
TUCP_002560	XLOC_108796	ENSRNOT00000066133	-0.96595773	3.40E-07
TUCP_002560	XLOC_108796	ENSRNOT00000077231	0.963750682	4.64E-07
TUCP_002560	XLOC_108796	ENSRNOT00000035700	0.970533951	1.66E-07
TUCP_002560	XLOC_108796	ENSRNOT00000059181	0.959699207	7.82E-07
TUCP_002560	XLOC_108796	ENSRNOT00000029202	-0.964919793	3.94E-07
TUCP_002560	XLOC_108796	ENSRNOT00000054828	0.971911798	1.31E-07
TUCP_002560	XLOC_108796	ENSRNOT00000009803	0.973433091	9.97E-08
TUCP_002560	XLOC_108796	ENSRNOT00000003690	0.963093554	5.07E-07
TUCP_002560	XLOC_108796	ENSRNOT00000028876	-0.953709285	1.55E-06
TUCP_002560	XLOC_108796	ENSRNOT00000016991	0.95376109	1.54E-06
TUCP_002560	XLOC_108796	ENSRNOT00000011350	0.972039411	1.28E-07
TUCP_002560	XLOC_108796	ENSRNOT00000085756	0.957290326	1.04E-06
TUCP_002560	XLOC_108796	ENSRNOT00000008901	-0.975901208	6.15E-08
TUCP_002560	XLOC_108796	ENSRNOT00000009496	0.972359699	1.21E-07
TUCP_002560	XLOC_108796	ENSRNOT00000005659	0.963807063	4.60E-07
TUCP_002560	XLOC_108796	ENSRNOT00000017761	0.968606034	2.28E-07
TUCP_002560	XLOC_108796	ENSRNOT00000055442	0.957757298	9.87E-07
TUCP_002560	XLOC_108796	ENSRNOT00000020663	-0.955458748	1.28E-06
TUCP_002560	XLOC_108796	ENSRNOT00000025887	-0.959226861	8.29E-07
TUCP_002560	XLOC_108796	ENSRNOT00000078994	0.959295303	8.22E-07
TUCP_002560	XLOC_108796	ENSRNOT00000002114	-0.953718831	1.55E-06
TUCP_002560	XLOC_108796	ENSRNOT00000022142	0.968039746	2.49E-07
TUCP_002560	XLOC_108796	ENSRNOT00000055992	-0.958971242	8.55E-07
TUCP_002560	XLOC_108796	ENSRNOT00000017063	-0.953706342	1.55E-06
TUCP_002560	XLOC_108796	ENSRNOT00000027970	0.977025168	4.85E-08
TUCP_002560	XLOC_108796	ENSRNOT00000010748	0.987846892	2.05E-09
TUCP_002560	XLOC_108796	ENSRNOT00000012334	0.952105065	1.83E-06
TUCP_002560	XLOC_108796	ENSRNOT00000028885	-0.962222974	5.69E-07
TUCP_002560	XLOC_108796	ENSRNOT00000044742	0.950970056	2.05E-06

TUCP_002560	XLOC_108796	ENSRNOT00000005478	-0.957894796	9.71E-07
TUCP_002560	XLOC_108796	ENSRNOT00000050834	-0.968933709	2.16E-07
TUCP_002560	XLOC_108796	ENSRNOT00000052120	0.966601813	3.09E-07
TUCP_002560	XLOC_108796	ENSRNOT00000004762	0.951307887	1.99E-06
TUCP_002560	XLOC_108796	ENSRNOT00000035474	0.984818335	6.19E-09
TUCP_002560	XLOC_108796	ENSRNOT00000039341	0.954744151	1.39E-06
TUCP_002560	XLOC_108796	ENSRNOT00000084422	0.977740285	4.15E-08
TUCP_002560	XLOC_108796	ENSRNOT00000033235	0.979258014	2.92E-08
TUCP_002560	XLOC_108796	ENSRNOT00000018074	-0.963133828	5.04E-07
TUCP_002560	XLOC_108796	ENSRNOT00000056130	0.950307299	2.19E-06
TUCP_002560	XLOC_108796	ENSRNOT00000076872	0.981845509	1.51E-08
TUCP_002560	XLOC_108796	ENSRNOT00000091205	0.981312525	1.74E-08
TUCP_002560	XLOC_108796	ENSRNOT00000001234	-0.952357766	1.78E-06
TUCP_002560	XLOC_108796	ENSRNOT00000014124	0.974098933	8.79E-08
TUCP_002560	XLOC_108796	ENSRNOT00000085519	0.964277295	4.31E-07
TUCP_002560	XLOC_108796	ENSRNOT00000040476	0.983049977	1.07E-08
TUCP_002560	XLOC_108796	ENSRNOT00000013632	0.953476268	1.59E-06
TUCP_002560	XLOC_108796	ENSRNOT00000038182	-0.959385673	8.13E-07
TUCP_002560	XLOC_108796	ENSRNOT00000010983	0.958272041	9.29E-07
TUCP_002560	XLOC_108796	ENSRNOT00000060696	0.954340432	1.45E-06
TUCP_002560	XLOC_108796	ENSRNOT00000004787	-0.964059065	4.45E-07
TUCP_002560	XLOC_108796	ENSRNOT00000077406	0.958357898	9.19E-07
TUCP_002560	XLOC_108796	ENSRNOT00000060698	-0.953663182	1.56E-06
TUCP_002560	XLOC_108796	ENSRNOT00000009900	-0.964724172	4.05E-07
TUCP_002560	XLOC_108796	ENSRNOT00000058351	0.977923422	3.98E-08
TUCP_002560	XLOC_108796	ENSRNOT00000014814	0.98154435	1.63E-08
TUCP_002560	XLOC_108796	ENSRNOT00000026760	-0.955829674	1.23E-06
TUCP_002560	XLOC_108796	ENSRNOT00000019172	-0.954816237	1.37E-06
TUCP_002560	XLOC_108796	ENSRNOT00000067181	0.951477987	1.95E-06
TUCP_002560	XLOC_108796	ENSRNOT00000064798	0.978720903	3.32E-08
TUCP_002560	XLOC_108796	ENSRNOT00000019177	0.970254611	1.74E-07
TUCP_002560	XLOC_108796	ENSRNOT00000024491	-0.959074404	8.44E-07
TUCP_002560	XLOC_108796	ENSRNOT00000084597	-0.95165811	1.92E-06
TUCP_002560	XLOC_108796	ENSRNOT00000023537	-0.952279035	1.80E-06
TUCP_002560	XLOC_108796	ENSRNOT00000065529	0.950586508	2.13E-06
TUCP_002560	XLOC_108796	ENSRNOT00000027452	0.95051201	2.15E-06
TUCP_002560	XLOC_108796	ENSRNOT00000017776	0.976519828	5.40E-08
TUCP_002560	XLOC_108796	ENSRNOT00000040393	0.968321932	2.38E-07
TUCP_002560	XLOC_108796	ENSRNOT00000017165	-0.951168002	2.01E-06
TUCP_002560	XLOC_108796	ENSRNOT00000012187	0.969097989	2.11E-07
TUCP_002560	XLOC_108796	ENSRNOT00000067901	-0.969608484	1.94E-07
TUCP_002560	XLOC_108796	ENSRNOT00000004463	0.969085142	2.11E-07
TUCP_002560	XLOC_108796	ENSRNOT00000008757	0.964518458	4.17E-07
TUCP_002560	XLOC_108796	ENSRNOT00000008982	0.991197829	4.10E-10
TUCP_002560	XLOC_108796	ENSRNOT00000021674	0.951705894	1.91E-06
TUCP_002560	XLOC_108796	ENSRNOT00000064700	0.962556756	5.44E-07
TUCP_002560	XLOC_108796	ENSRNOT00000047210	0.965367714	3.70E-07
TUCP_002560	XLOC_108796	ENSRNOT00000036613	-0.95871453	8.81E-07
TUCP_002560	XLOC_108796	ENSRNOT00000008987	-0.950592956	2.13E-06
TUCP_002560	XLOC_108796	ENSRNOT00000015537	0.95792401	9.68E-07
TUCP_002560	XLOC_108796	ENSRNOT00000025705	0.954354637	1.45E-06

TUCP_002560	XLOC_108796	ENSRNOT00000002109	0.974385702	8.32E-08
TUCP_002560	XLOC_108796	ENSRNOT00000083957	0.956164968	1.18E-06
TUCP_002560	XLOC_108796	ENSRNOT00000005311	0.951121454	2.02E-06
TUCP_002560	XLOC_108796	ENSRNOT00000017687	-0.95994038	7.60E-07
TUCP_002560	XLOC_108796	ENSRNOT00000036931	-0.95657666	1.13E-06
TUCP_002560	XLOC_108796	ENSRNOT00000011682	-0.954961825	1.35E-06
TUCP_002560	XLOC_108796	ENSRNOT00000079996	0.973911891	9.11E-08
TUCP_002560	XLOC_108796	ENSRNOT00000072357	-0.954263766	1.46E-06
TUCP_002408	XLOC_101523	ENSRNOT00000004508	0.951400368	1.97E-06
TUCP_002408	XLOC_101523	ENSRNOT00000010992	0.964305087	4.30E-07
TUCP_002408	XLOC_101523	ENSRNOT00000044896	0.952098761	1.83E-06
TUCP_002408	XLOC_101523	ENSRNOT00000025092	0.97618654	5.79E-08
TUCP_002408	XLOC_101523	ENSRNOT00000070999	0.95767489	9.96E-07
TUCP_002408	XLOC_101523	ENSRNOT00000058852	-0.964639625	4.10E-07
TUCP_002408	XLOC_101523	ENSRNOT00000021116	0.956205334	1.18E-06
TUCP_002408	XLOC_101523	ENSRNOT00000076669	0.95844148	9.10E-07
TUCP_002408	XLOC_101523	ENSRNOT00000043156	0.95444674	1.43E-06
TUCP_002408	XLOC_101523	ENSRNOT00000021132	0.959674702	7.85E-07
TUCP_002408	XLOC_101523	ENSRNOT00000059458	0.959786448	7.74E-07
TUCP_002408	XLOC_101523	ENSRNOT00000015203	0.956844811	1.10E-06
TUCP_002562	XLOC_108825	ENSRNOT00000065143	0.965052006	3.87E-07
TUCP_002562	XLOC_108825	ENSRNOT00000031904	0.951812415	1.89E-06
TUCP_002562	XLOC_108825	ENSRNOT00000082998	0.962417327	5.54E-07
TUCP_002562	XLOC_108825	ENSRNOT00000081246	0.970384543	1.71E-07
TUCP_002562	XLOC_108825	ENSRNOT00000048910	-0.950676782	2.12E-06
TUCP_002562	XLOC_108825	ENSRNOT00000027083	0.952117765	1.83E-06
TUCP_002562	XLOC_108825	ENSRNOT00000009877	0.972194446	1.25E-07
TUCP_002562	XLOC_108825	ENSRNOT00000007351	-0.962935164	5.18E-07
TUCP_002562	XLOC_108825	ENSRNOT00000021348	0.958453944	9.09E-07
TUCP_002562	XLOC_108825	ENSRNOT00000023133	0.956046932	1.20E-06
TUCP_002562	XLOC_108825	ENSRNOT00000071560	0.954834122	1.37E-06
TUCP_002562	XLOC_108825	ENSRNOT00000025936	-0.955825829	1.23E-06
TUCP_002562	XLOC_108825	ENSRNOT00000082508	-0.95782345	9.79E-07
TUCP_002562	XLOC_108825	ENSRNOT00000023638	0.965868039	3.45E-07
TUCP_002562	XLOC_108825	ENSRNOT00000005228	0.974169641	8.67E-08
TUCP_002562	XLOC_108825	ENSRNOT00000005220	0.953812064	1.53E-06
TUCP_002562	XLOC_108825	ENSRNOT00000023144	0.967958177	2.52E-07
TUCP_002562	XLOC_108825	ENSRNOT00000019502	0.957274725	1.04E-06
TUCP_002562	XLOC_108825	ENSRNOT00000016649	0.954586124	1.41E-06
TUCP_002562	XLOC_108825	ENSRNOT00000090342	0.974249894	8.54E-08
TUCP_002562	XLOC_108825	ENSRNOT00000000743	0.962481818	5.50E-07
TUCP_002562	XLOC_108825	ENSRNOT00000065300	0.959561291	7.96E-07
TUCP_002562	XLOC_108825	ENSRNOT00000080608	0.953467151	1.59E-06
TUCP_002562	XLOC_108825	ENSRNOT00000043216	0.972536369	1.18E-07
TUCP_002562	XLOC_108825	ENSRNOT00000091128	-0.980715134	2.03E-08
TUCP_002562	XLOC_108825	ENSRNOT00000009803	0.976759422	5.14E-08
TUCP_002562	XLOC_108825	ENSRNOT00000081981	0.985646417	4.68E-09
TUCP_002562	XLOC_108825	ENSRNOT00000009496	0.961463536	6.27E-07
TUCP_002562	XLOC_108825	ENSRNOT00000028563	0.950010458	2.26E-06
TUCP_002562	XLOC_108825	ENSRNOT00000083369	0.953251211	1.63E-06
TUCP_002562	XLOC_108825	ENSRNOT00000003158	-0.95783525	9.78E-07

TUCP_002562	XLOC_108825	ENSRNOT00000004172	0.951998758	1.85E-06
TUCP_002562	XLOC_108825	ENSRNOT00000015073	0.952253402	1.80E-06
TUCP_002562	XLOC_108825	ENSRNOT00000019213	0.954556706	1.41E-06
TUCP_002562	XLOC_108825	ENSRNOT00000077201	-0.953561305	1.57E-06
TUCP_002562	XLOC_108825	ENSRNOT00000005942	0.95209704	1.83E-06
TUCP_002562	XLOC_108825	ENSRNOT00000066810	0.96562137	3.57E-07
TUCP_002562	XLOC_108825	ENSRNOT00000018533	0.976411639	5.53E-08
TUCP_002562	XLOC_108825	ENSRNOT00000073564	-0.963044949	5.10E-07
TUCP_002562	XLOC_108825	ENSRNOT00000024969	0.980004293	2.43E-08
TUCP_002562	XLOC_108825	ENSRNOT00000026783	0.958633481	8.90E-07
TUCP_002562	XLOC_108825	ENSRNOT00000028646	0.969225871	2.06E-07
TUCP_002562	XLOC_108825	ENSRNOT00000011384	0.960664951	6.94E-07
TUCP_002562	XLOC_108825	ENSRNOT00000048754	-0.9527525	1.71E-06
TUCP_002562	XLOC_108825	ENSRNOT00000038247	0.959142782	8.37E-07
TUCP_002562	XLOC_108825	ENSRNOT00000017063	-0.950027442	2.26E-06
TUCP_002562	XLOC_108825	ENSRNOT00000071764	0.966950305	2.94E-07
TUCP_002562	XLOC_108825	ENSRNOT00000079109	0.954793466	1.38E-06
TUCP_002562	XLOC_108825	ENSRNOT00000024402	0.953345531	1.61E-06
TUCP_002562	XLOC_108825	ENSRNOT00000025372	0.963363827	4.89E-07
TUCP_002562	XLOC_108825	ENSRNOT00000089933	0.955288702	1.31E-06
TUCP_002562	XLOC_108825	ENSRNOT00000009811	0.95316681	1.64E-06
TUCP_002562	XLOC_108825	ENSRNOT00000083215	0.963021173	5.12E-07
TUCP_002562	XLOC_108825	ENSRNOT00000072208	0.954643506	1.40E-06
TUCP_002562	XLOC_108825	ENSRNOT00000032780	0.968342433	2.37E-07
TUCP_002562	XLOC_108825	ENSRNOT00000009708	-0.971203406	1.49E-07
TUCP_002562	XLOC_108825	ENSRNOT00000017319	0.958910266	8.61E-07
TUCP_002562	XLOC_108825	ENSRNOT00000015188	0.953083654	1.65E-06
TUCP_002562	XLOC_108825	ENSRNOT00000033677	-0.952126814	1.83E-06
TUCP_002562	XLOC_108825	ENSRNOT00000079866	0.956272947	1.17E-06
TUCP_002562	XLOC_108825	ENSRNOT00000041513	0.95824078	9.32E-07
TUCP_002562	XLOC_108825	ENSRNOT00000019820	0.951771491	1.89E-06
TUCP_002562	XLOC_108825	ENSRNOT00000005958	0.979404981	2.82E-08
TUCP_002562	XLOC_108825	ENSRNOT00000042125	0.971100667	1.51E-07
TUCP_002562	XLOC_108825	ENSRNOT00000074847	-0.954743558	1.39E-06
TUCP_002562	XLOC_108825	ENSRNOT00000010927	0.953938061	1.51E-06
TUCP_002562	XLOC_108825	ENSRNOT00000004032	0.972065795	1.28E-07
TUCP_002562	XLOC_108825	ENSRNOT00000084385	0.960865447	6.77E-07
TUCP_002562	XLOC_108825	ENSRNOT00000012895	0.961568552	6.19E-07
TUCP_002562	XLOC_108825	ENSRNOT00000027537	0.951001322	2.05E-06
TUCP_002562	XLOC_108825	ENSRNOT00000091909	0.953319979	1.61E-06
TUCP_002562	XLOC_108825	ENSRNOT00000020147	0.950878408	2.07E-06
TUCP_002562	XLOC_108825	ENSRNOT00000090918	0.95063058	2.13E-06
TUCP_002562	XLOC_108825	ENSRNOT00000001081	0.95620803	1.18E-06
TUCP_002562	XLOC_108825	ENSRNOT00000083220	0.975645904	6.48E-08
TUCP_002562	XLOC_108825	ENSRNOT00000067846	-0.955794029	1.23E-06
TUCP_002562	XLOC_108825	ENSRNOT00000015609	0.95200089	1.85E-06
TUCP_002562	XLOC_108825	ENSRNOT00000058312	0.965210267	3.79E-07
TUCP_002562	XLOC_108825	ENSRNOT00000060362	0.989232232	1.12E-09
TUCP_002562	XLOC_108825	ENSRNOT00000022213	0.954891275	1.36E-06
TUCP_002562	XLOC_108825	ENSRNOT00000022610	0.954103511	1.48E-06
TUCP_002562	XLOC_108825	ENSRNOT00000020437	0.968336957	2.38E-07

TUCP_002562	XLOC_108825	ENSRNOT00000006828	0.964777201	4.02E-07
TUCP_002562	XLOC_108825	ENSRNOT00000010898	0.963274924	4.95E-07
TUCP_002562	XLOC_108825	ENSRNOT00000015265	0.971022133	1.53E-07
TUCP_002562	XLOC_108825	ENSRNOT00000087095	0.967863046	2.56E-07
TUCP_002562	XLOC_108825	ENSRNOT00000048063	0.958844576	8.68E-07
TUCP_002562	XLOC_108825	ENSRNOT00000052422	0.957824264	9.79E-07
TUCP_002562	XLOC_108825	ENSRNOT00000001242	0.956762764	1.11E-06
TUCP_002562	XLOC_108825	ENSRNOT00000031979	-0.951102042	2.03E-06
TUCP_002562	XLOC_108825	ENSRNOT00000086119	-0.951940794	1.86E-06
TUCP_002562	XLOC_108825	ENSRNOT00000078102	0.981213116	1.79E-08
TUCP_002562	XLOC_108825	ENSRNOT00000025983	0.967984705	2.51E-07
TUCP_002562	XLOC_108825	ENSRNOT00000027526	0.963490529	4.80E-07
TUCP_002562	XLOC_108825	ENSRNOT00000058072	0.950291713	2.20E-06
TUCP_002562	XLOC_108825	ENSRNOT00000081047	0.964800812	4.01E-07
TUCP_002562	XLOC_108825	ENSRNOT00000073559	0.957224177	1.05E-06
TUCP_002562	XLOC_108825	ENSRNOT00000023989	0.955411362	1.29E-06
TUCP_002562	XLOC_108825	ENSRNOT00000049819	0.956479108	1.14E-06
TUCP_002562	XLOC_108825	ENSRNOT00000003991	0.970716555	1.61E-07
TUCP_002562	XLOC_108825	ENSRNOT00000021408	0.952838285	1.70E-06
TUCP_002562	XLOC_108825	ENSRNOT00000027910	0.972501159	1.18E-07
TUCP_002562	XLOC_108825	ENSRNOT00000080207	0.964556416	4.15E-07
TUCP_002562	XLOC_108825	ENSRNOT00000035591	-0.957023994	1.07E-06
TUCP_002562	XLOC_108825	ENSRNOT00000000302	0.955566013	1.27E-06
TUCP_002562	XLOC_108825	ENSRNOT00000052224	0.957176843	1.06E-06
TUCP_002562	XLOC_108825	ENSRNOT00000078070	0.973557591	9.74E-08
TUCP_002562	XLOC_108825	ENSRNOT00000046745	0.963669902	4.69E-07
TUCP_002562	XLOC_108825	ENSRNOT00000092029	0.9628646	5.23E-07
TUCP_002562	XLOC_108825	ENSRNOT00000088182	0.982232377	1.35E-08
TUCP_002562	XLOC_108825	ENSRNOT00000085228	0.969396097	2.01E-07
TUCP_002562	XLOC_108825	ENSRNOT00000091265	0.95982054	7.71E-07
TUCP_002562	XLOC_108825	ENSRNOT00000073526	0.956657305	1.12E-06
TUCP_002562	XLOC_108825	ENSRNOT00000078390	-0.955779999	1.24E-06
TUCP_002562	XLOC_108825	ENSRNOT00000000013	0.953428027	1.60E-06
TUCP_002562	XLOC_108825	ENSRNOT00000025832	0.971147572	1.50E-07
TUCP_002562	XLOC_108825	ENSRNOT00000017753	0.975636191	6.49E-08
TUCP_002562	XLOC_108825	ENSRNOT00000051022	0.953004049	1.67E-06
TUCP_002562	XLOC_108825	ENSRNOT00000006499	0.962323051	5.61E-07
TUCP_002562	XLOC_108825	ENSRNOT00000005909	0.971515431	1.41E-07
TUCP_002562	XLOC_108825	ENSRNOT00000029833	0.960931351	6.71E-07
TUCP_002562	XLOC_108825	ENSRNOT00000009198	0.968806266	2.21E-07
TUCP_002562	XLOC_108825	ENSRNOT00000009129	0.972068731	1.28E-07
TUCP_002562	XLOC_108825	ENSRNOT00000036951	0.96360136	4.73E-07
TUCP_002562	XLOC_108825	ENSRNOT00000016519	0.95926005	8.25E-07
TUCP_002562	XLOC_108825	ENSRNOT00000008929	0.95394766	1.51E-06
TUCP_002562	XLOC_108825	ENSRNOT00000004046	-0.966282208	3.24E-07
TUCP_002562	XLOC_108825	ENSRNOT00000040393	0.955304331	1.30E-06
TUCP_002562	XLOC_108825	ENSRNOT00000058842	0.964675082	4.08E-07
TUCP_002562	XLOC_108825	ENSRNOT00000000157	0.959296945	8.22E-07
TUCP_002562	XLOC_108825	ENSRNOT00000035700	0.971354927	1.45E-07
TUCP_002562	XLOC_108825	ENSRNOT00000076669	0.959893045	7.64E-07
TUCP_002562	XLOC_108825	ENSRNOT00000016991	0.950316996	2.19E-06

TUCP_002562	XLOC_108825	ENSRNOT00000011350	0.96001415	7.53E-07
TUCP_002562	XLOC_108825	ENSRNOT00000085756	0.976065141	5.94E-08
TUCP_002562	XLOC_108825	ENSRNOT00000008901	-0.964212515	4.35E-07
TUCP_002562	XLOC_108825	ENSRNOT00000005659	0.956556921	1.13E-06
TUCP_002562	XLOC_108825	ENSRNOT00000050834	-0.955527217	1.27E-06
TUCP_002562	XLOC_108825	ENSRNOT00000054828	0.96161059	6.16E-07
TUCP_002562	XLOC_108825	ENSRNOT00000020663	-0.952842923	1.70E-06
TUCP_002562	XLOC_108825	ENSRNOT00000059458	0.956701856	1.11E-06
TUCP_002562	XLOC_108825	ENSRNOT00000055992	-0.959447403	8.07E-07
TUCP_002562	XLOC_108825	ENSRNOT00000077406	0.960288914	7.28E-07
TUCP_002562	XLOC_108825	ENSRNOT00000040069	0.980306935	2.26E-08
TUCP_002562	XLOC_108825	ENSRNOT00000010748	0.968226818	2.42E-07
TUCP_002562	XLOC_108825	ENSRNOT00000032992	0.968595066	2.28E-07
TUCP_002562	XLOC_108825	ENSRNOT00000008757	0.953395696	1.60E-06
TUCP_002562	XLOC_108825	ENSRNOT00000005478	-0.950893049	2.07E-06
TUCP_002562	XLOC_108825	ENSRNOT00000046058	0.956459067	1.15E-06
TUCP_002562	XLOC_108825	ENSRNOT00000052120	0.951380846	1.97E-06
TUCP_002562	XLOC_108825	ENSRNOT00000026543	0.972212832	1.25E-07
TUCP_002562	XLOC_108825	ENSRNOT00000035474	0.976557459	5.36E-08
TUCP_002562	XLOC_108825	ENSRNOT00000080695	0.950257631	2.21E-06
TUCP_002562	XLOC_108825	ENSRNOT00000086336	0.954785321	1.38E-06
TUCP_002562	XLOC_108825	ENSRNOT00000084422	0.959783899	7.74E-07
TUCP_002562	XLOC_108825	ENSRNOT00000061392	0.963438133	4.84E-07
TUCP_002562	XLOC_108825	ENSRNOT00000076872	0.98364773	8.96E-09
TUCP_002562	XLOC_108825	ENSRNOT00000091205	0.970195989	1.76E-07
TUCP_002562	XLOC_108825	ENSRNOT00000014124	0.95014827	2.23E-06
TUCP_002562	XLOC_108825	ENSRNOT00000085519	0.952380977	1.78E-06
TUCP_002562	XLOC_108825	ENSRNOT00000040476	0.964072241	4.44E-07
TUCP_002562	XLOC_108825	ENSRNOT00000029125	0.990583089	5.74E-10
TUCP_002562	XLOC_108825	ENSRNOT00000057146	0.961846585	5.97E-07
TUCP_002562	XLOC_108825	ENSRNOT00000009900	-0.951258189	2.00E-06
TUCP_002562	XLOC_108825	ENSRNOT00000027970	0.951560063	1.94E-06
TUCP_002562	XLOC_108825	ENSRNOT00000014814	0.988075842	1.86E-09
TUCP_002562	XLOC_108825	ENSRNOT00000004412	0.956116764	1.19E-06
TUCP_002562	XLOC_108825	ENSRNOT00000067181	0.952847493	1.70E-06
TUCP_002562	XLOC_108825	ENSRNOT00000064798	0.979182806	2.97E-08
TUCP_002562	XLOC_108825	ENSRNOT00000019177	0.974832875	7.62E-08
TUCP_002562	XLOC_108825	ENSRNOT00000033235	0.979024027	3.09E-08
TUCP_002562	XLOC_108825	ENSRNOT00000027452	0.955950949	1.21E-06
TUCP_002562	XLOC_108825	ENSRNOT00000017776	0.954574145	1.41E-06
TUCP_002562	XLOC_108825	ENSRNOT00000075708	0.950825152	2.08E-06
TUCP_002562	XLOC_108825	ENSRNOT00000012187	0.966530297	3.13E-07
TUCP_002562	XLOC_108825	ENSRNOT00000004463	0.957089514	1.07E-06
TUCP_002562	XLOC_108825	ENSRNOT00000027964	0.961414404	6.31E-07
TUCP_002562	XLOC_108825	ENSRNOT00000008982	0.981129242	1.83E-08
TUCP_002562	XLOC_108825	ENSRNOT00000021674	0.952931203	1.68E-06
TUCP_002562	XLOC_108825	ENSRNOT00000047192	-0.96475125	4.04E-07
TUCP_002562	XLOC_108825	ENSRNOT00000027615	0.957289128	1.04E-06
TUCP_002562	XLOC_108825	ENSRNOT00000083799	0.954540693	1.42E-06
TUCP_002562	XLOC_108825	ENSRNOT00000012463	-0.951753831	1.90E-06
TUCP_002562	XLOC_108825	ENSRNOT00000083957	0.958572085	8.96E-07

TUCP_002562	XLOC_108825	ENSRNOT00000015799	0.971011985	1.54E-07
TUCP_002562	XLOC_108825	ENSRNOT00000011682	-0.952103233	1.83E-06
TUCP_002562	XLOC_108825	ENSRNOT00000079996	0.95902155	8.49E-07
TUCP_002565	XLOC_108833	ENSRNOT00000065143	0.951731458	1.90E-06
TUCP_002565	XLOC_108833	ENSRNOT00000044885	0.950333811	2.19E-06
TUCP_002565	XLOC_108833	ENSRNOT00000007379	0.958359535	9.19E-07
TUCP_002565	XLOC_108833	ENSRNOT00000027526	0.951931306	1.86E-06
TUCP_002565	XLOC_108833	ENSRNOT00000032780	0.962244093	5.67E-07
TUCP_002565	XLOC_108833	ENSRNOT00000081981	0.963885703	4.55E-07
TUCP_002565	XLOC_108833	ENSRNOT00000011350	0.955822601	1.23E-06
TUCP_002565	XLOC_108833	ENSRNOT00000063873	0.950004997	2.26E-06
TUCP_002565	XLOC_108833	ENSRNOT00000005958	0.971707471	1.36E-07
TUCP_002565	XLOC_108833	ENSRNOT00000049819	0.95281591	1.70E-06
TUCP_002565	XLOC_108833	ENSRNOT00000061392	0.951723978	1.90E-06
TUCP_002565	XLOC_108833	ENSRNOT00000005228	0.95112469	2.02E-06
TUCP_002565	XLOC_108833	ENSRNOT00000008757	0.955185421	1.32E-06
TUCP_002565	XLOC_108833	ENSRNOT00000012966	0.964041456	4.46E-07
TUCP_002565	XLOC_108833	ENSRNOT00000079177	0.950678417	2.12E-06
TUCP_002565	XLOC_108833	ENSRNOT00000008929	0.954908474	1.36E-06
TUCP_002565	XLOC_108833	ENSRNOT00000052224	0.961814599	6.00E-07
TUCP_002565	XLOC_108833	ENSRNOT00000078102	0.969087647	2.11E-07
TUCP_002565	XLOC_108833	ENSRNOT00000056130	0.953334879	1.61E-06
TUCP_002565	XLOC_108833	ENSRNOT00000076872	0.958588729	8.95E-07
TUCP_002565	XLOC_108833	ENSRNOT00000088182	0.955013621	1.35E-06
TUCP_002565	XLOC_108833	ENSRNOT00000059744	0.950372324	2.18E-06
TUCP_002565	XLOC_108833	ENSRNOT00000040476	0.956753256	1.11E-06
TUCP_002565	XLOC_108833	ENSRNOT00000009803	0.979179717	2.98E-08
TUCP_002565	XLOC_108833	ENSRNOT00000029125	0.953502212	1.58E-06
TUCP_002565	XLOC_108833	ENSRNOT00000042125	0.951816237	1.89E-06
TUCP_002565	XLOC_108833	ENSRNOT00000014814	0.96365127	4.70E-07
TUCP_002565	XLOC_108833	ENSRNOT00000077201	-0.955173164	1.32E-06
TUCP_002565	XLOC_108833	ENSRNOT00000087095	0.957848676	9.76E-07
TUCP_002565	XLOC_108833	ENSRNOT00000024969	0.973768192	9.36E-08
TUCP_002565	XLOC_108833	ENSRNOT00000033235	0.955101536	1.33E-06
TUCP_002565	XLOC_108833	ENSRNOT00000060362	0.957466812	1.02E-06
TUCP_002565	XLOC_108833	ENSRNOT00000085756	0.962167889	5.73E-07
TUCP_002565	XLOC_108833	ENSRNOT00000017063	-0.957373986	1.03E-06
TUCP_002565	XLOC_108833	ENSRNOT00000016519	0.974513798	8.11E-08
TUCP_002565	XLOC_108833	ENSRNOT00000015265	0.956136098	1.19E-06
TUCP_002565	XLOC_108833	ENSRNOT00000017753	0.956161731	1.18E-06
TUCP_002565	XLOC_108833	ENSRNOT00000004046	-0.964859435	3.98E-07
TUCP_002565	XLOC_108833	ENSRNOT00000025372	0.961414582	6.31E-07
TUCP_002565	XLOC_108833	ENSRNOT00000057146	0.959347506	8.17E-07
TUCP_002565	XLOC_108833	ENSRNOT00000058842	0.957851252	9.76E-07
TUCP_002565	XLOC_108833	ENSRNOT00000083220	0.95839128	9.16E-07
TUCP_002565	XLOC_108833	ENSRNOT00000008982	0.954262889	1.46E-06
TUCP_002565	XLOC_108833	ENSRNOT00000031568	0.956649803	1.12E-06
TUCP_002564	XLOC_108829	ENSRNOT00000084133	1	0
TUCP_002564	XLOC_108829	ENSRNOT00000093745	0.981891039	1.49E-08
TUCP_002564	XLOC_108829	ENSRNOT00000065542	0.99828699	1.16E-13
TUCP_002564	XLOC_108829	ENSRNOT00000082464	1	0

TUCP_002564	XLOC_108829	ENSRNOT00000087659	1	0
TUCP_002564	XLOC_108829	ENSRNOT00000084582	0.976633579	5.28E-08
TUCP_002564	XLOC_108829	ENSRNOT00000085078	1	0
TUCP_002564	XLOC_108829	ENSRNOT00000014028	1	0
TUCP_002564	XLOC_108829	ENSRNOT00000049363	1	0
TUCP_002564	XLOC_108829	ENSRNOT00000065473	0.999949098	2.69E-21
TUCP_002564	XLOC_108829	ENSRNOT00000000142	0.998996155	8.01E-15
TUCP_002564	XLOC_108829	ENSRNOT00000082288	0.999998398	8.30E-29
TUCP_002564	XLOC_108829	ENSRNOT00000039664	1	0
TUCP_002564	XLOC_108829	ENSRNOT00000065402	1	0
TUCP_002564	XLOC_108829	ENSRNOT00000064551	0.965365872	3.70E-07
TUCP_002564	XLOC_108829	ENSRNOT00000084141	1	0
TUCP_002564	XLOC_108829	ENSRNOT00000082435	1	0
TUCP_002564	XLOC_108829	ENSRNOT00000082928	0.996801059	2.62E-12
TUCP_002564	XLOC_108829	ENSRNOT00000066546	0.956953619	1.08E-06
TUCP_002564	XLOC_108829	ENSRNOT00000046013	0.988138626	1.81E-09
TUCP_002564	XLOC_108829	ENSRNOT00000067931	0.978893628	3.18E-08
TUCP_002564	XLOC_108829	ENSRNOT00000086425	0.992023315	2.51E-10
TUCP_002564	XLOC_108829	ENSRNOT00000093696	0.956927219	1.09E-06
TUCP_002564	XLOC_108829	ENSRNOT00000036228	0.982337157	1.31E-08
TUCP_002564	XLOC_108829	ENSRNOT00000014030	1	0
TUCP_002564	XLOC_108829	ENSRNOT00000081966	1	4.25E-78
TUCP_002564	XLOC_108829	ENSRNOT00000075906	1	1.33E-79
TUCP_002564	XLOC_108829	ENSRNOT00000088396	0.981336994	1.73E-08
TUCP_002564	XLOC_108829	ENSRNOT00000049259	0.965500259	3.63E-07
TUCP_002564	XLOC_108829	ENSRNOT00000092838	0.982579749	1.23E-08
TUCP_002564	XLOC_108829	ENSRNOT00000093442	0.959217317	8.30E-07
TUCP_002567	XLOC_108858	ENSRNOT00000065143	0.956713886	1.11E-06
TUCP_002567	XLOC_108858	ENSRNOT00000005958	0.973678059	9.52E-08
TUCP_002567	XLOC_108858	ENSRNOT00000000743	0.954866254	1.37E-06
TUCP_002567	XLOC_108858	ENSRNOT00000027083	0.962025736	5.83E-07
TUCP_002567	XLOC_108858	ENSRNOT00000066041	-0.950069432	2.25E-06
TUCP_002567	XLOC_108858	ENSRNOT00000029833	0.961751802	6.05E-07
TUCP_002567	XLOC_108858	ENSRNOT00000001290	0.954548079	1.42E-06
TUCP_002567	XLOC_108858	ENSRNOT00000023638	0.950880816	2.07E-06
TUCP_002567	XLOC_108858	ENSRNOT00000005228	0.976161342	5.83E-08
TUCP_002567	XLOC_108858	ENSRNOT00000008677	0.952940368	1.68E-06
TUCP_002567	XLOC_108858	ENSRNOT00000028625	-0.962866274	5.22E-07
TUCP_002567	XLOC_108858	ENSRNOT00000081768	0.951305215	1.99E-06
TUCP_002567	XLOC_108858	ENSRNOT00000082869	0.967228534	2.82E-07
TUCP_002567	XLOC_108858	ENSRNOT00000019502	0.950954622	2.06E-06
TUCP_002567	XLOC_108858	ENSRNOT00000016649	0.955222795	1.31E-06
TUCP_002567	XLOC_108858	ENSRNOT00000028856	0.951184569	2.01E-06
TUCP_002567	XLOC_108858	ENSRNOT00000091128	-0.966780695	3.01E-07
TUCP_002567	XLOC_108858	ENSRNOT00000009803	0.960655419	6.95E-07
TUCP_002567	XLOC_108858	ENSRNOT00000081981	0.966700237	3.05E-07
TUCP_002567	XLOC_108858	ENSRNOT00000012187	0.978451349	3.53E-08
TUCP_002567	XLOC_108858	ENSRNOT00000024969	0.974441284	8.23E-08
TUCP_002567	XLOC_108858	ENSRNOT00000085251	-0.953606889	1.57E-06
TUCP_002567	XLOC_108858	ENSRNOT00000028646	0.968932053	2.16E-07
TUCP_002567	XLOC_108858	ENSRNOT00000038247	0.959594377	7.92E-07

TUCP_002567	XLOC_108858	ENSRNOT00000044628	0.964247661	4.33E-07
TUCP_002567	XLOC_108858	ENSRNOT00000076611	-0.959524257	7.99E-07
TUCP_002567	XLOC_108858	ENSRNOT00000004007	-0.952895453	1.69E-06
TUCP_002567	XLOC_108858	ENSRNOT00000089933	0.952833465	1.70E-06
TUCP_002567	XLOC_108858	ENSRNOT00000091602	-0.951706696	1.91E-06
TUCP_002567	XLOC_108858	ENSRNOT00000031568	0.954255178	1.46E-06
TUCP_002567	XLOC_108858	ENSRNOT00000009811	0.951355473	1.98E-06
TUCP_002567	XLOC_108858	ENSRNOT00000021471	-0.951255836	2.00E-06
TUCP_002567	XLOC_108858	ENSRNOT00000085228	0.951498143	1.95E-06
TUCP_002567	XLOC_108858	ENSRNOT00000032780	0.966564528	3.11E-07
TUCP_002567	XLOC_108858	ENSRNOT00000077231	0.952926827	1.68E-06
TUCP_002567	XLOC_108858	ENSRNOT00000087592	0.954558345	1.41E-06
TUCP_002567	XLOC_108858	ENSRNOT00000061392	0.96366342	4.69E-07
TUCP_002567	XLOC_108858	ENSRNOT00000002410	0.954258234	1.46E-06
TUCP_002567	XLOC_108858	ENSRNOT00000008655	0.971838187	1.33E-07
TUCP_002567	XLOC_108858	ENSRNOT00000003714	0.957949603	9.65E-07
TUCP_002567	XLOC_108858	ENSRNOT00000010541	0.954099938	1.49E-06
TUCP_002567	XLOC_108858	ENSRNOT00000087443	0.953642277	1.56E-06
TUCP_002567	XLOC_108858	ENSRNOT00000015116	0.966476018	3.15E-07
TUCP_002567	XLOC_108858	ENSRNOT00000001081	0.954657173	1.40E-06
TUCP_002567	XLOC_108858	ENSRNOT00000083220	0.963115545	5.05E-07
TUCP_002567	XLOC_108858	ENSRNOT00000015609	0.95103432	2.04E-06
TUCP_002567	XLOC_108858	ENSRNOT00000019133	0.964826176	4.00E-07
TUCP_002567	XLOC_108858	ENSRNOT00000052018	0.951411984	1.97E-06
TUCP_002567	XLOC_108858	ENSRNOT00000010898	0.956396657	1.15E-06
TUCP_002567	XLOC_108858	ENSRNOT00000071493	-0.962794209	5.27E-07
TUCP_002567	XLOC_108858	ENSRNOT00000015265	0.960316673	7.25E-07
TUCP_002567	XLOC_108858	ENSRNOT00000067181	0.954800558	1.38E-06
TUCP_002567	XLOC_108858	ENSRNOT00000052422	0.965074848	3.86E-07
TUCP_002567	XLOC_108858	ENSRNOT00000057146	0.952978465	1.67E-06
TUCP_002567	XLOC_108858	ENSRNOT00000080207	0.963479423	4.81E-07
TUCP_002567	XLOC_108858	ENSRNOT00000004319	-0.956681939	1.12E-06
TUCP_002567	XLOC_108858	ENSRNOT00000078102	0.973199015	1.04E-07
TUCP_002567	XLOC_108858	ENSRNOT00000070893	0.965262864	3.76E-07
TUCP_002567	XLOC_108858	ENSRNOT00000007314	-0.952994464	1.67E-06
TUCP_002567	XLOC_108858	ENSRNOT00000090123	0.96633344	3.22E-07
TUCP_002567	XLOC_108858	ENSRNOT00000023144	0.956557675	1.13E-06
TUCP_002567	XLOC_108858	ENSRNOT00000088599	0.952447672	1.77E-06
TUCP_002567	XLOC_108858	ENSRNOT00000063873	0.953534983	1.58E-06
TUCP_002567	XLOC_108858	ENSRNOT00000081047	0.972267526	1.23E-07
TUCP_002567	XLOC_108858	ENSRNOT00000067688	-0.960164501	7.39E-07
TUCP_002567	XLOC_108858	ENSRNOT00000088353	-0.974923226	7.49E-08
TUCP_002567	XLOC_108858	ENSRNOT00000021408	0.966356927	3.21E-07
TUCP_002567	XLOC_108858	ENSRNOT00000006034	-0.950134076	2.23E-06
TUCP_002567	XLOC_108858	ENSRNOT00000087429	0.957974142	9.62E-07
TUCP_002567	XLOC_108858	ENSRNOT00000060362	0.961485409	6.26E-07
TUCP_002567	XLOC_108858	ENSRNOT00000052224	0.982035467	1.43E-08
TUCP_002567	XLOC_108858	ENSRNOT00000078070	0.96821526	2.42E-07
TUCP_002567	XLOC_108858	ENSRNOT00000088182	0.954460556	1.43E-06
TUCP_002567	XLOC_108858	ENSRNOT00000078390	-0.959822558	7.71E-07
TUCP_002567	XLOC_108858	ENSRNOT00000017181	-0.96146272	6.27E-07

TUCP_002567	XLOC_108858	ENSRNOT00000017753	0.965596041	3.58E-07
TUCP_002567	XLOC_108858	ENSRNOT00000051022	0.951803944	1.89E-06
TUCP_002567	XLOC_108858	ENSRNOT00000025936	-0.962400107	5.56E-07
TUCP_002567	XLOC_108858	ENSRNOT00000022074	0.956720105	1.11E-06
TUCP_002567	XLOC_108858	ENSRNOT00000016519	0.95622354	1.18E-06
TUCP_002567	XLOC_108858	ENSRNOT00000004046	-0.961880105	5.95E-07
TUCP_002567	XLOC_108858	ENSRNOT00000019886	0.951366635	1.97E-06
TUCP_002567	XLOC_108858	ENSRNOT00000058842	0.959504747	8.01E-07
TUCP_002567	XLOC_108858	ENSRNOT00000001586	-0.954637245	1.40E-06
TUCP_002567	XLOC_108858	ENSRNOT00000077894	-0.956231994	1.18E-06
TUCP_002567	XLOC_108858	ENSRNOT00000000728	-0.952148398	1.82E-06
TUCP_002567	XLOC_108858	ENSRNOT00000008901	-0.959767073	7.76E-07
TUCP_002567	XLOC_108858	ENSRNOT00000050834	-0.954721395	1.39E-06
TUCP_002567	XLOC_108858	ENSRNOT00000054828	0.953204303	1.63E-06
TUCP_002567	XLOC_108858	ENSRNOT00000002443	0.953982773	1.50E-06
TUCP_002567	XLOC_108858	ENSRNOT00000080608	0.955890831	1.22E-06
TUCP_002567	XLOC_108858	ENSRNOT00000027970	0.955502584	1.27E-06
TUCP_002567	XLOC_108858	ENSRNOT00000010748	0.958718365	8.81E-07
TUCP_002567	XLOC_108858	ENSRNOT00000008757	0.954269765	1.46E-06
TUCP_002567	XLOC_108858	ENSRNOT00000005478	-0.951813116	1.89E-06
TUCP_002567	XLOC_108858	ENSRNOT00000035474	0.958926252	8.59E-07
TUCP_002567	XLOC_108858	ENSRNOT00000076872	0.953279167	1.62E-06
TUCP_002567	XLOC_108858	ENSRNOT00000080110	0.955626468	1.26E-06
TUCP_002567	XLOC_108858	ENSRNOT00000029125	0.956544829	1.13E-06
TUCP_002567	XLOC_108858	ENSRNOT00000042125	0.973357373	1.01E-07
TUCP_002567	XLOC_108858	ENSRNOT00000009900	-0.953277198	1.62E-06
TUCP_002567	XLOC_108858	ENSRNOT00000081400	0.958692481	8.84E-07
TUCP_002567	XLOC_108858	ENSRNOT00000014814	0.963945775	4.52E-07
TUCP_002567	XLOC_108858	ENSRNOT00000087095	0.957387992	1.03E-06
TUCP_002567	XLOC_108858	ENSRNOT00000064798	0.952988994	1.67E-06
TUCP_002567	XLOC_108858	ENSRNOT00000019177	0.950169088	2.22E-06
TUCP_002567	XLOC_108858	ENSRNOT00000014261	0.954784358	1.38E-06
TUCP_002567	XLOC_108858	ENSRNOT00000033235	0.959502295	8.01E-07
TUCP_002567	XLOC_108858	ENSRNOT00000043535	0.952273104	1.80E-06
TUCP_002567	XLOC_108858	ENSRNOT00000077201	-0.955842744	1.23E-06
TUCP_002567	XLOC_108858	ENSRNOT00000008982	0.957780691	9.84E-07
TUCP_002567	XLOC_108858	ENSRNOT00000047192	-0.973880868	9.16E-08
TUCP_002567	XLOC_108858	ENSRNOT00000038066	0.950068626	2.25E-06
TUCP_002567	XLOC_108858	ENSRNOT00000015799	0.960595932	7.00E-07
TUCP_002567	XLOC_108858	ENSRNOT00000011682	-0.974440149	8.23E-08
TUCP_002402	XLOC_101398	ENSRNOT00000022351	0.951492572	1.95E-06
TUCP_002403	XLOC_101418	ENSRNOT00000090828	-0.971277581	1.47E-07
TUCP_002403	XLOC_101418	ENSRNOT00000076050	-0.95835213	9.20E-07
TUCP_002400	XLOC_101329	ENSRNOT00000081235	0.955811684	1.23E-06
TUCP_002401	XLOC_101341	ENSRNOT00000006034	-0.960876203	6.76E-07
TUCP_002401	XLOC_101341	ENSRNOT00000026915	0.950276028	2.20E-06
TUCP_002401	XLOC_101341	ENSRNOT00000008221	0.952922394	1.68E-06
TUCP_002401	XLOC_101341	ENSRNOT00000047093	0.952351503	1.79E-06
TUCP_002401	XLOC_101341	ENSRNOT00000030445	0.972315617	1.22E-07
TUCP_002401	XLOC_101341	ENSRNOT00000046906	-0.970499444	1.67E-07
TUCP_002406	XLOC_101419	ENSRNOT00000079080	0.954757925	1.38E-06

TUCP_002404	XLOC_101418	ENSRNOT00000050854	0.960779489	6.84E-07
TUCP_002404	XLOC_101418	ENSRNOT00000085000	0.95488461	1.36E-06
TUCP_002404	XLOC_101418	ENSRNOT00000074650	0.967509605	2.70E-07
TUCP_003108	XLOC_132831	ENSRNOT00000044428	0.952658204	1.73E-06
TUCP_003108	XLOC_132831	ENSRNOT00000010216	0.955913913	1.22E-06
TUCP_002810	XLOC_118753	ENSRNOT00000031331	0.953652832	1.56E-06
TUCP_002761	XLOC_117228	ENSRNOT00000085476	0.961311748	6.40E-07
TUCP_002761	XLOC_117228	ENSRNOT00000083608	0.954388872	1.44E-06
TUCP_002761	XLOC_117228	ENSRNOT00000078176	0.964418824	4.23E-07
TUCP_002761	XLOC_117228	ENSRNOT00000021193	0.951325853	1.98E-06
TUCP_002761	XLOC_117228	ENSRNOT00000047560	0.956910394	1.09E-06
TUCP_002761	XLOC_117228	ENSRNOT00000024758	0.953951994	1.51E-06
TUCP_002761	XLOC_117228	ENSRNOT00000088719	0.977912681	3.99E-08
TUCP_002761	XLOC_117228	ENSRNOT00000033187	0.97087449	1.57E-07
TUCP_002815	XLOC_119222	ENSRNOT00000077169	-0.959786972	7.74E-07
TUCP_002815	XLOC_119222	ENSRNOT00000084633	0.955068576	1.34E-06
TUCP_002814	XLOC_119094	ENSRNOT00000084133	1	0
TUCP_002814	XLOC_119094	ENSRNOT00000093745	0.981891039	1.49E-08
TUCP_002814	XLOC_119094	ENSRNOT00000065542	0.99828699	1.16E-13
TUCP_002814	XLOC_119094	ENSRNOT00000082464	1	0
TUCP_002814	XLOC_119094	ENSRNOT00000087659	1	0
TUCP_002814	XLOC_119094	ENSRNOT00000084582	0.976633579	5.28E-08
TUCP_002814	XLOC_119094	ENSRNOT00000085078	1	0
TUCP_002814	XLOC_119094	ENSRNOT00000014028	1	0
TUCP_002814	XLOC_119094	ENSRNOT00000049363	1	0
TUCP_002814	XLOC_119094	ENSRNOT00000065473	0.999949098	2.69E-21
TUCP_002814	XLOC_119094	ENSRNOT00000000142	0.998996155	8.01E-15
TUCP_002814	XLOC_119094	ENSRNOT00000082288	0.999998398	8.30E-29
TUCP_002814	XLOC_119094	ENSRNOT00000039664	1	0
TUCP_002814	XLOC_119094	ENSRNOT00000065402	1	0
TUCP_002814	XLOC_119094	ENSRNOT00000064551	0.965365872	3.70E-07
TUCP_002814	XLOC_119094	ENSRNOT00000084141	1	1.33E-79
TUCP_002814	XLOC_119094	ENSRNOT00000082435	1	0
TUCP_002814	XLOC_119094	ENSRNOT00000082928	0.996801059	2.62E-12
TUCP_002814	XLOC_119094	ENSRNOT00000066546	0.956953619	1.08E-06
TUCP_002814	XLOC_119094	ENSRNOT00000046013	0.988138626	1.81E-09
TUCP_002814	XLOC_119094	ENSRNOT00000067931	0.978893628	3.18E-08
TUCP_002814	XLOC_119094	ENSRNOT00000086425	0.992023315	2.51E-10
TUCP_002814	XLOC_119094	ENSRNOT00000093696	0.956927219	1.09E-06
TUCP_002814	XLOC_119094	ENSRNOT00000036228	0.982337157	1.31E-08
TUCP_002814	XLOC_119094	ENSRNOT00000014030	1	0
TUCP_002814	XLOC_119094	ENSRNOT00000081966	1	1.33E-79
TUCP_002814	XLOC_119094	ENSRNOT00000075906	1	1.33E-79
TUCP_002814	XLOC_119094	ENSRNOT00000088396	0.981336994	1.73E-08
TUCP_002814	XLOC_119094	ENSRNOT00000049259	0.965500259	3.63E-07
TUCP_002814	XLOC_119094	ENSRNOT00000092838	0.982579749	1.23E-08
TUCP_002814	XLOC_119094	ENSRNOT00000093442	0.959217317	8.30E-07
TUCP_002765	XLOC_117240	ENSRNOT00000001019	0.9532082	1.63E-06
TUCP_003192	XLOC_137559	ENSRNOT00000024128	0.965571967	3.60E-07
TUCP_003192	XLOC_137559	ENSRNOT00000051265	0.969464673	1.99E-07
TUCP_003192	XLOC_137559	ENSRNOT00000008492	0.952172556	1.82E-06

TUCP_003192	XLOC_137559	ENSRNOT00000011833	0.958522148	9.02E-07
TUCP_003192	XLOC_137559	ENSRNOT00000020390	0.953040365	1.66E-06
TUCP_003100	XLOC_132620	ENSRNOT00000026106	0.959767013	7.76E-07
TUCP_001550	XLOC_060077	ENSRNOT00000080185	0.955381468	1.29E-06
TUCP_001550	XLOC_060077	ENSRNOT00000013705	0.950982709	2.05E-06
TUCP_001550	XLOC_060077	ENSRNOT00000000351	0.950982709	2.05E-06
TUCP_001550	XLOC_060077	ENSRNOT00000040851	0.950982709	2.05E-06
TUCP_001550	XLOC_060077	ENSRNOT00000092890	0.95256214	1.75E-06
TUCP_001550	XLOC_060077	ENSRNOT00000002881	0.953543782	1.58E-06
TUCP_001550	XLOC_060077	ENSRNOT00000061950	0.959209935	8.30E-07
TUCP_001550	XLOC_060077	ENSRNOT00000086710	0.952867204	1.69E-06
TUCP_001550	XLOC_060077	ENSRNOT00000086827	0.972734456	1.13E-07
TUCP_001550	XLOC_060077	ENSRNOT00000082031	0.96040664	7.17E-07
TUCP_001550	XLOC_060077	ENSRNOT00000028653	0.950982709	2.05E-06
TUCP_001550	XLOC_060077	ENSRNOT00000058389	0.950982709	2.05E-06
TUCP_001550	XLOC_060077	ENSRNOT00000075226	0.950982709	2.05E-06
TUCP_001550	XLOC_060077	ENSRNOT00000089118	0.950982709	2.05E-06
TUCP_001550	XLOC_060077	ENSRNOT00000023542	0.950982709	2.05E-06
TUCP_001550	XLOC_060077	ENSRNOT00000079661	0.950982709	2.05E-06
TUCP_001550	XLOC_060077	ENSRNOT00000025245	0.951256264	2.00E-06
TUCP_001550	XLOC_060077	ENSRNOT00000012174	0.950982709	2.05E-06
TUCP_001550	XLOC_060077	ENSRNOT00000042220	0.950982709	2.05E-06
TUCP_003102	XLOC_132760	ENSRNOT00000076400	0.965618207	3.57E-07
TUCP_003102	XLOC_132760	ENSRNOT00000055536	0.965915585	3.42E-07
TUCP_003102	XLOC_132760	ENSRNOT00000010680	0.954036578	1.50E-06
TUCP_003102	XLOC_132760	ENSRNOT00000064983	0.969189211	2.08E-07
TUCP_003102	XLOC_132760	ENSRNOT00000016088	0.962527311	5.46E-07
TUCP_003102	XLOC_132760	ENSRNOT00000009975	0.989606166	9.39E-10
TUCP_003102	XLOC_132760	ENSRNOT00000072682	0.965687293	3.54E-07
TUCP_003102	XLOC_132760	ENSRNOT00000015062	0.969624874	1.94E-07
TUCP_003102	XLOC_132760	ENSRNOT00000085954	0.951519338	1.94E-06
TUCP_003102	XLOC_132760	ENSRNOT00000001642	0.956417021	1.15E-06
TUCP_003102	XLOC_132760	ENSRNOT00000027544	0.958664745	8.87E-07
TUCP_003102	XLOC_132760	ENSRNOT00000081631	0.95782533	9.79E-07
TUCP_003102	XLOC_132760	ENSRNOT00000021715	0.953893266	1.52E-06
TUCP_003102	XLOC_132760	ENSRNOT00000017829	0.955855571	1.23E-06
TUCP_003102	XLOC_132760	ENSRNOT00000065897	0.965935206	3.41E-07
TUCP_003102	XLOC_132760	ENSRNOT00000023014	0.958305486	9.25E-07
TUCP_003102	XLOC_132760	ENSRNOT00000083594	0.962474012	5.50E-07
TUCP_003102	XLOC_132760	ENSRNOT00000073493	0.952694705	1.72E-06
TUCP_003102	XLOC_132760	ENSRNOT00000036137	0.954246312	1.46E-06
TUCP_003102	XLOC_132760	ENSRNOT00000056050	0.979697655	2.63E-08
TUCP_003102	XLOC_132760	ENSRNOT00000046552	0.954662934	1.40E-06
TUCP_003102	XLOC_132760	ENSRNOT00000060977	0.962390484	5.56E-07
TUCP_003102	XLOC_132760	ENSRNOT00000065801	0.960231873	7.33E-07
TUCP_003102	XLOC_132760	ENSRNOT00000088385	0.961702499	6.08E-07
TUCP_003102	XLOC_132760	ENSRNOT00000023613	0.953197033	1.63E-06
TUCP_003102	XLOC_132760	ENSRNOT00000092066	-0.961807992	6.00E-07
TUCP_003102	XLOC_132760	ENSRNOT00000016485	0.975721561	6.38E-08
TUCP_003102	XLOC_132760	ENSRNOT00000065615	0.950655158	2.12E-06
TUCP_003102	XLOC_132760	ENSRNOT00000011675	0.951996641	1.85E-06

TUCP_003102	XLOC_132760	ENSRNOT00000008361	0.955409588	1.29E-06
TUCP_003102	XLOC_132760	ENSRNOT00000042638	0.964110542	4.41E-07
TUCP_003102	XLOC_132760	ENSRNOT00000005770	0.974710744	7.81E-08
TUCP_003102	XLOC_132760	ENSRNOT00000006697	0.961936777	5.90E-07
TUCP_003102	XLOC_132760	ENSRNOT00000066952	0.95172239	1.90E-06
TUCP_003102	XLOC_132760	ENSRNOT00000018640	0.950913458	2.07E-06
TUCP_003102	XLOC_132760	ENSRNOT00000054685	0.950984891	2.05E-06
TUCP_003102	XLOC_132760	ENSRNOT00000085334	0.984214224	7.52E-09
TUCP_003102	XLOC_132760	ENSRNOT00000034482	0.973799505	9.31E-08
TUCP_003102	XLOC_132760	ENSRNOT00000043827	0.965736574	3.51E-07
TUCP_003102	XLOC_132760	ENSRNOT00000081226	0.965339293	3.72E-07
TUCP_003102	XLOC_132760	ENSRNOT00000061054	0.952952958	1.68E-06
TUCP_003102	XLOC_132760	ENSRNOT00000008764	0.980197819	2.32E-08
TUCP_003102	XLOC_132760	ENSRNOT00000012757	0.963151772	5.03E-07
TUCP_003102	XLOC_132760	ENSRNOT00000009009	0.962192868	5.71E-07
TUCP_003102	XLOC_132760	ENSRNOT00000037902	0.966470561	3.15E-07
TUCP_003102	XLOC_132760	ENSRNOT00000020929	0.962978368	5.15E-07
TUCP_003102	XLOC_132760	ENSRNOT00000079128	0.953026626	1.66E-06
TUCP_003102	XLOC_132760	ENSRNOT00000006109	0.964557308	4.15E-07
TUCP_003102	XLOC_132760	ENSRNOT00000057738	0.954466897	1.43E-06
TUCP_003102	XLOC_132760	ENSRNOT00000065871	0.954118215	1.48E-06
TUCP_003102	XLOC_132760	ENSRNOT00000092757	0.951573742	1.93E-06
TUCP_003102	XLOC_132760	ENSRNOT00000025355	0.953368369	1.61E-06
TUCP_003102	XLOC_132760	ENSRNOT00000078800	0.968178719	2.44E-07
TUCP_002768	XLOC_117242	ENSRNOT00000074650	0.997078706	1.67E-12
TUCP_003104	XLOC_132831	ENSRNOT00000092968	0.959180323	8.33E-07
TUCP_003104	XLOC_132831	ENSRNOT00000090449	0.953515758	1.58E-06
TUCP_000283	XLOC_008661	ENSRNOT00000042890	0.958549963	8.99E-07
TUCP_000283	XLOC_008661	ENSRNOT00000065111	0.959359844	8.15E-07
TUCP_003106	XLOC_132831	ENSRNOT00000029197	0.976843923	5.04E-08
TUCP_001960	XLOC_079154	ENSRNOT00000010992	0.958190041	9.38E-07
TUCP_001960	XLOC_079154	ENSRNOT00000004834	0.968173022	2.44E-07
TUCP_001960	XLOC_079154	ENSRNOT00000013932	0.959294539	8.22E-07
TUCP_001960	XLOC_079154	ENSRNOT00000040513	0.957198197	1.05E-06
TUCP_001960	XLOC_079154	ENSRNOT00000068405	0.961642677	6.13E-07
TUCP_001960	XLOC_079154	ENSRNOT00000004740	0.951093694	2.03E-06
TUCP_001960	XLOC_079154	ENSRNOT00000015076	0.952082076	1.84E-06
TUCP_001960	XLOC_079154	ENSRNOT00000018293	0.950179152	2.22E-06
TUCP_001960	XLOC_079154	ENSRNOT00000021865	0.963506121	4.79E-07
TUCP_001960	XLOC_079154	ENSRNOT00000039842	0.957128129	1.06E-06
TUCP_001960	XLOC_079154	ENSRNOT00000039809	0.957092749	1.07E-06
TUCP_001960	XLOC_079154	ENSRNOT00000082100	0.956005801	1.21E-06
TUCP_001960	XLOC_079154	ENSRNOT00000029664	0.954170939	1.47E-06
TUCP_001960	XLOC_079154	ENSRNOT00000082253	0.955879631	1.22E-06
TUCP_001960	XLOC_079154	ENSRNOT00000013260	0.955008243	1.35E-06
TUCP_001960	XLOC_079154	ENSRNOT00000017629	0.959162378	8.35E-07
TUCP_001960	XLOC_079154	ENSRNOT00000087226	0.954897031	1.36E-06
TUCP_001960	XLOC_079154	ENSRNOT00000078175	0.961349273	6.37E-07
TUCP_001960	XLOC_079154	ENSRNOT00000054674	0.963436905	4.84E-07
TUCP_003190	XLOC_137467	ENSRNOT00000040187	0.953160462	1.64E-06
TUCP_003190	XLOC_137467	ENSRNOT00000028609	0.957517124	1.01E-06

TUCP_003190	XLOC_137467	ENSRNOT00000072452	0.954901108	1.36E-06
TUCP_003339	XLOC_144383	ENSRNOT00000021590	0.950915633	2.07E-06
TUCP_003339	XLOC_144383	ENSRNOT00000065111	0.980464913	2.17E-08
TUCP_003339	XLOC_144383	ENSRNOT00000024583	-0.958941066	8.58E-07
TUCP_003339	XLOC_144383	ENSRNOT00000061569	0.969131821	2.10E-07
TUCP_003339	XLOC_144383	ENSRNOT00000015888	0.968511807	2.31E-07
TUCP_003339	XLOC_144383	ENSRNOT00000091315	0.978007549	3.91E-08
TUCP_003196	XLOC_137866	ENSRNOT00000080232	0.961542493	6.21E-07
TUCP_003338	XLOC_144326	ENSRNOT00000000008	1	0
TUCP_003338	XLOC_144326	ENSRNOT00000060048	0.998711457	2.79E-14
TUCP_003338	XLOC_144326	ENSRNOT00000082304	0.996875989	2.33E-12
TUCP_003338	XLOC_144326	ENSRNOT00000006219	1	0
TUCP_003338	XLOC_144326	ENSRNOT00000065376	1	0
TUCP_003338	XLOC_144326	ENSRNOT00000081433	0.984469223	6.93E-09
TUCP_003338	XLOC_144326	ENSRNOT00000083575	1	0
TUCP_003338	XLOC_144326	ENSRNOT00000087739	1	0
TUCP_003338	XLOC_144326	ENSRNOT00000079826	1	0
TUCP_003338	XLOC_144326	ENSRNOT00000025770	0.993828251	6.98E-11
TUCP_003338	XLOC_144326	ENSRNOT00000088244	0.973479481	9.88E-08
TUCP_003338	XLOC_144326	ENSRNOT00000048523	1	0
TUCP_003338	XLOC_144326	ENSRNOT00000079365	0.958936699	8.58E-07
TUCP_003338	XLOC_144326	ENSRNOT00000020229	0.960002785	7.54E-07
TUCP_003338	XLOC_144326	ENSRNOT00000026428	0.996986683	1.95E-12
TUCP_003338	XLOC_144326	ENSRNOT00000085673	1	0
TUCP_003338	XLOC_144326	ENSRNOT00000072972	0.981476166	1.67E-08
TUCP_003338	XLOC_144326	ENSRNOT00000050573	1	0
TUCP_003338	XLOC_144326	ENSRNOT00000089876	-0.953630353	1.56E-06
TUCP_003338	XLOC_144326	ENSRNOT00000074378	0.998901807	1.26E-14
TUCP_003338	XLOC_144326	ENSRNOT00000093571	1	0
TUCP_003338	XLOC_144326	ENSRNOT00000090581	1	0
TUCP_003338	XLOC_144326	ENSRNOT00000076239	0.98020752	2.31E-08
TUCP_003338	XLOC_144326	ENSRNOT00000065485	1	0
TUCP_003338	XLOC_144326	ENSRNOT00000006356	0.999544279	1.55E-16
TUCP_003338	XLOC_144326	ENSRNOT00000087199	0.974421555	8.26E-08
TUCP_003338	XLOC_144326	ENSRNOT00000022091	1	0
TUCP_003338	XLOC_144326	ENSRNOT00000064764	0.98460888	6.63E-09
TUCP_003338	XLOC_144326	ENSRNOT00000076927	0.971163156	1.50E-07
TUCP_003338	XLOC_144326	ENSRNOT00000092395	0.999522978	1.94E-16
TUCP_001403	XLOC_053911	ENSRNOT00000011951	0.951081425	2.03E-06
TUCP_001403	XLOC_053911	ENSRNOT00000015547	0.952141289	1.82E-06
TUCP_001403	XLOC_053911	ENSRNOT00000081375	0.964072465	4.44E-07
TUCP_002986	XLOC_128384	ENSRNOT00000014865	-0.955641203	1.26E-06
TUCP_003243	XLOC_139063	ENSRNOT00000088441	0.987262749	2.58E-09
TUCP_003243	XLOC_139063	ENSRNOT00000015138	0.955447585	1.28E-06
TUCP_003243	XLOC_139063	ENSRNOT00000080185	0.95317855	1.64E-06
TUCP_003243	XLOC_139063	ENSRNOT00000089118	0.956342845	1.16E-06
TUCP_003243	XLOC_139063	ENSRNOT00000013705	0.956342845	1.16E-06
TUCP_003243	XLOC_139063	ENSRNOT00000000351	0.956342845	1.16E-06
TUCP_003243	XLOC_139063	ENSRNOT00000040851	0.956342845	1.16E-06
TUCP_003243	XLOC_139063	ENSRNOT00000058389	0.956342845	1.16E-06
TUCP_003243	XLOC_139063	ENSRNOT00000086710	0.956957911	1.08E-06

TUCP_003243	XLOC_139063	ENSRNOT00000025245	0.956074141	1.20E-06
TUCP_003243	XLOC_139063	ENSRNOT00000028653	0.956342845	1.16E-06
TUCP_003243	XLOC_139063	ENSRNOT00000079167	0.956928054	1.09E-06
TUCP_003243	XLOC_139063	ENSRNOT00000085195	0.96585402	3.45E-07
TUCP_003243	XLOC_139063	ENSRNOT00000023542	0.956342845	1.16E-06
TUCP_003243	XLOC_139063	ENSRNOT00000042220	0.956342845	1.16E-06
TUCP_003243	XLOC_139063	ENSRNOT00000079661	0.956342845	1.16E-06
TUCP_003243	XLOC_139063	ENSRNOT00000012174	0.956342845	1.16E-06
TUCP_003243	XLOC_139063	ENSRNOT00000075226	0.956342845	1.16E-06
TUCP_000924	XLOC_033943	ENSRNOT00000022384	-0.979770548	2.58E-08
TUCP_001636	XLOC_064916	ENSRNOT00000081224	0.951727565	1.90E-06
TUCP_004408	XLOC_194445	ENSRNOT00000024161	0.956995405	1.08E-06
TUCP_004409	XLOC_194475	ENSRNOT00000087673	0.954870434	1.37E-06
TUCP_004409	XLOC_194475	ENSRNOT00000086027	0.953019092	1.67E-06
TUCP_004404	XLOC_194388	ENSRNOT00000074990	0.961170909	6.51E-07
TUCP_004404	XLOC_194388	ENSRNOT00000022280	0.963598871	4.73E-07
TUCP_004404	XLOC_194388	ENSRNOT00000081370	0.963180893	5.01E-07
TUCP_004404	XLOC_194388	ENSRNOT00000075987	0.963200542	5.00E-07
TUCP_004404	XLOC_194388	ENSRNOT00000082043	0.961149616	6.53E-07
TUCP_004404	XLOC_194388	ENSRNOT00000087323	0.962918039	5.19E-07
TUCP_004404	XLOC_194388	ENSRNOT00000074930	0.961170909	6.51E-07
TUCP_004404	XLOC_194388	ENSRNOT00000080575	0.960505837	7.08E-07
TUCP_004404	XLOC_194388	ENSRNOT00000078369	0.960717628	6.90E-07
TUCP_004404	XLOC_194388	ENSRNOT00000092982	0.961170909	6.51E-07
TUCP_004404	XLOC_194388	ENSRNOT00000088367	0.961170909	6.51E-07
TUCP_004404	XLOC_194388	ENSRNOT00000078153	0.963105915	5.06E-07
TUCP_004404	XLOC_194388	ENSRNOT00000081996	0.961170909	6.51E-07
TUCP_004404	XLOC_194388	ENSRNOT00000068444	0.961170909	6.51E-07
TUCP_004404	XLOC_194388	ENSRNOT00000082058	0.961170909	6.51E-07
TUCP_004404	XLOC_194388	ENSRNOT00000088905	0.961170909	6.51E-07
TUCP_004404	XLOC_194388	ENSRNOT00000086831	0.961170909	6.51E-07
TUCP_004404	XLOC_194388	ENSRNOT00000045099	0.961170909	6.51E-07
TUCP_004404	XLOC_194388	ENSRNOT00000086745	0.963915534	4.53E-07
TUCP_004404	XLOC_194388	ENSRNOT00000085849	0.953232914	1.63E-06
TUCP_004404	XLOC_194388	ENSRNOT00000077789	0.964344452	4.27E-07
TUCP_004404	XLOC_194388	ENSRNOT00000090207	0.978353819	3.61E-08
TUCP_004404	XLOC_194388	ENSRNOT00000087025	0.961170909	6.51E-07
TUCP_004404	XLOC_194388	ENSRNOT00000019853	0.961170909	6.51E-07
TUCP_004404	XLOC_194388	ENSRNOT00000065912	0.961579796	6.18E-07
TUCP_004404	XLOC_194388	ENSRNOT00000093083	0.96049318	7.09E-07
TUCP_001913	XLOC_078876	ENSRNOT00000008888	0.958106808	9.47E-07
TUCP_001915	XLOC_078877	ENSRNOT00000079730	0.983525749	9.30E-09
TUCP_004401	XLOC_194334	ENSRNOT00000085394	0.957341594	1.04E-06
TUCP_003523	XLOC_153375	ENSRNOT00000082084	0.956466665	1.14E-06
TUCP_003523	XLOC_153375	ENSRNOT00000081106	0.954973099	1.35E-06
TUCP_003523	XLOC_153375	ENSRNOT00000047784	0.95416026	1.48E-06
TUCP_003044	XLOC_129609	ENSRNOT00000037067	-0.959919692	7.62E-07
TUCP_003198	XLOC_137908	ENSRNOT00000015373	0.971602351	1.39E-07
TUCP_001402	XLOC_053817	ENSRNOT00000065312	0.991576416	3.29E-10
TUCP_001402	XLOC_053817	ENSRNOT00000083651	0.961809153	6.00E-07
TUCP_001402	XLOC_053817	ENSRNOT00000040740	0.95655159	1.13E-06

TUCP_003040	XLOC_129278	ENSRNOT00000055034	0.952744474	1.71E-06
TUCP_003040	XLOC_129278	ENSRNOT00000074346	0.95486886	1.37E-06
TUCP_003040	XLOC_129278	ENSRNOT00000046837	0.961020406	6.64E-07
TUCP_003043	XLOC_129574	ENSRNOT00000000008	1	0
TUCP_003043	XLOC_129574	ENSRNOT00000060048	0.998711457	2.79E-14
TUCP_003043	XLOC_129574	ENSRNOT00000082304	0.996875989	2.33E-12
TUCP_003043	XLOC_129574	ENSRNOT00000006219	1	0
TUCP_003043	XLOC_129574	ENSRNOT00000065376	1	0
TUCP_003043	XLOC_129574	ENSRNOT00000081433	0.984469223	6.93E-09
TUCP_003043	XLOC_129574	ENSRNOT00000083575	1	0
TUCP_003043	XLOC_129574	ENSRNOT00000087739	1	0
TUCP_003043	XLOC_129574	ENSRNOT00000079826	1	0
TUCP_003043	XLOC_129574	ENSRNOT00000025770	0.993828251	6.98E-11
TUCP_003043	XLOC_129574	ENSRNOT00000088244	0.973479481	9.88E-08
TUCP_003043	XLOC_129574	ENSRNOT00000048523	1	0
TUCP_003043	XLOC_129574	ENSRNOT00000079365	0.958936699	8.58E-07
TUCP_003043	XLOC_129574	ENSRNOT00000020229	0.960002785	7.54E-07
TUCP_003043	XLOC_129574	ENSRNOT00000026428	0.996986683	1.95E-12
TUCP_003043	XLOC_129574	ENSRNOT00000085673	1	0
TUCP_003043	XLOC_129574	ENSRNOT00000072972	0.981476166	1.67E-08
TUCP_003043	XLOC_129574	ENSRNOT00000050573	1	0
TUCP_003043	XLOC_129574	ENSRNOT00000089876	-0.953630353	1.56E-06
TUCP_003043	XLOC_129574	ENSRNOT00000074378	0.998901807	1.26E-14
TUCP_003043	XLOC_129574	ENSRNOT00000093571	1	0
TUCP_003043	XLOC_129574	ENSRNOT00000090581	1	0
TUCP_003043	XLOC_129574	ENSRNOT00000076239	0.98020752	2.31E-08
TUCP_003043	XLOC_129574	ENSRNOT00000065485	1	0
TUCP_003043	XLOC_129574	ENSRNOT00000006356	0.999544279	1.55E-16
TUCP_003043	XLOC_129574	ENSRNOT00000087199	0.974421555	8.26E-08
TUCP_003043	XLOC_129574	ENSRNOT00000022091	1	0
TUCP_003043	XLOC_129574	ENSRNOT00000064764	0.98460888	6.63E-09
TUCP_003043	XLOC_129574	ENSRNOT00000076927	0.971163156	1.50E-07
TUCP_003043	XLOC_129574	ENSRNOT00000092395	0.999522978	1.94E-16
TUCP_001608	XLOC_063433	ENSRNOT00000004905	0.957253131	1.05E-06
TUCP_001608	XLOC_063433	ENSRNOT00000025689	0.952572152	1.74E-06
TUCP_001608	XLOC_063433	ENSRNOT00000072427	0.953916383	1.51E-06
TUCP_001608	XLOC_063433	ENSRNOT00000004359	0.952462191	1.76E-06
TUCP_001608	XLOC_063433	ENSRNOT00000042352	0.970542619	1.66E-07
TUCP_001608	XLOC_063433	ENSRNOT00000009066	0.967691496	2.63E-07
TUCP_000281	XLOC_008618	ENSRNOT00000082403	-0.954789426	1.38E-06
TUCP_000566	XLOC_020107	ENSRNOT00000016464	0.965083259	3.85E-07
TUCP_000566	XLOC_020107	ENSRNOT00000084928	0.957281213	1.04E-06
TUCP_000566	XLOC_020107	ENSRNOT00000067875	0.952172409	1.82E-06
TUCP_000566	XLOC_020107	ENSRNOT00000000018	0.967239833	2.81E-07
TUCP_000566	XLOC_020107	ENSRNOT00000025843	0.963291749	4.94E-07
TUCP_003048	XLOC_129803	ENSRNOT00000026966	0.970322306	1.73E-07
TUCP_003048	XLOC_129803	ENSRNOT00000068416	0.999999782	3.88E-33
TUCP_003048	XLOC_129803	ENSRNOT00000086036	0.999958274	9.96E-22
TUCP_003048	XLOC_129803	ENSRNOT00000026803	0.985046075	5.74E-09
TUCP_003048	XLOC_129803	ENSRNOT00000047527	0.963071698	5.08E-07
TUCP_003048	XLOC_129803	ENSRNOT00000042251	0.999344295	9.53E-16

TUCP_003048	XLOC_129803	ENSRNOT00000002984	0.998397069	8.31E-14
TUCP_003048	XLOC_129803	ENSRNOT00000083764	0.999541808	1.59E-16
TUCP_003048	XLOC_129803	ENSRNOT00000076802	0.962695843	5.34E-07
TUCP_003048	XLOC_129803	ENSRNOT00000092365	0.999161651	3.26E-15
TUCP_003048	XLOC_129803	ENSRNOT00000079275	0.999901911	7.15E-20
TUCP_003048	XLOC_129803	ENSRNOT00000066062	-0.959706712	7.82E-07
TUCP_003048	XLOC_129803	ENSRNOT00000045301	1	0
TUCP_003048	XLOC_129803	ENSRNOT00000041186	0.999975782	6.56E-23
TUCP_003048	XLOC_129803	ENSRNOT00000010092	0.977711778	4.17E-08
TUCP_003048	XLOC_129803	ENSRNOT00000076182	1	0
TUCP_003048	XLOC_129803	ENSRNOT00000087404	0.951157664	2.02E-06
TUCP_003048	XLOC_129803	ENSRNOT00000052003	0.95837331	9.18E-07
TUCP_003048	XLOC_129803	ENSRNOT00000081709	0.998485154	6.27E-14
TUCP_003048	XLOC_129803	ENSRNOT00000005726	0.959610119	7.91E-07
TUCP_003048	XLOC_129803	ENSRNOT00000014103	1	0
TUCP_003048	XLOC_129803	ENSRNOT00000057433	0.964930601	3.94E-07
TUCP_003048	XLOC_129803	ENSRNOT00000082289	0.998931566	1.09E-14
TUCP_003048	XLOC_129803	ENSRNOT00000092516	0.957459323	1.02E-06
TUCP_003048	XLOC_129803	ENSRNOT00000008898	0.990039768	7.59E-10
TUCP_003048	XLOC_129803	ENSRNOT00000083493	0.995461232	1.51E-11
TUCP_003048	XLOC_129803	ENSRNOT00000092563	0.987201084	2.65E-09
TUCP_003048	XLOC_129803	ENSRNOT00000088893	1	0
TUCP_003048	XLOC_129803	ENSRNOT00000076693	0.955029582	1.34E-06
TUCP_003048	XLOC_129803	ENSRNOT00000081014	1	0
TUCP_003048	XLOC_129803	ENSRNOT00000067543	1	0
TUCP_003048	XLOC_129803	ENSRNOT00000050968	0.996816433	2.56E-12
TUCP_003048	XLOC_129803	ENSRNOT00000042201	0.960235595	7.32E-07
TUCP_003048	XLOC_129803	ENSRNOT00000004373	0.957315473	1.04E-06
TUCP_003048	XLOC_129803	ENSRNOT00000063929	1	0
TUCP_003048	XLOC_129803	ENSRNOT00000057472	1	0
TUCP_003048	XLOC_129803	ENSRNOT00000041617	0.952449765	1.77E-06
TUCP_003048	XLOC_129803	ENSRNOT00000091763	0.986310385	3.70E-09
TUCP_003048	XLOC_129803	ENSRNOT00000022200	0.998491303	6.14E-14
TUCP_003048	XLOC_129803	ENSRNOT00000067379	0.997260967	1.21E-12
TUCP_003048	XLOC_129803	ENSRNOT00000020786	0.976428448	5.51E-08
TUCP_003048	XLOC_129803	ENSRNOT00000050543	0.999722223	1.30E-17
TUCP_003048	XLOC_129803	ENSRNOT00000089306	0.999866649	3.32E-19
TUCP_003048	XLOC_129803	ENSRNOT00000052367	0.997268394	1.19E-12
TUCP_003048	XLOC_129803	ENSRNOT00000090658	1	0
TUCP_003048	XLOC_129803	ENSRNOT00000022571	0.995290013	1.81E-11
TUCP_003048	XLOC_129803	ENSRNOT00000044520	0.981817158	1.52E-08
TUCP_003048	XLOC_129803	ENSRNOT00000065448	0.963648868	4.70E-07
TUCP_002967	XLOC_128223	ENSRNOT00000088280	0.95321887	1.63E-06
TUCP_002966	XLOC_128222	ENSRNOT00000092026	0.96620614	3.28E-07
TUCP_002966	XLOC_128222	ENSRNOT00000093075	1	0
TUCP_002966	XLOC_128222	ENSRNOT00000058260	1	0
TUCP_002966	XLOC_128222	ENSRNOT00000088887	1	0
TUCP_002966	XLOC_128222	ENSRNOT00000084986	1	0
TUCP_002966	XLOC_128222	ENSRNOT00000074346	0.9702256	1.75E-07
TUCP_002966	XLOC_128222	ENSRNOT00000073209	0.983711602	8.79E-09
TUCP_002966	XLOC_128222	ENSRNOT00000080155	0.973069673	1.07E-07

TUCP_002966	XLOC_128222	ENSRNOT00000072573	0.996213272	6.09E-12
TUCP_002966	XLOC_128222	ENSRNOT00000017828	0.996001583	7.99E-12
TUCP_002966	XLOC_128222	ENSRNOT00000080444	0.969625907	1.93E-07
TUCP_002966	XLOC_128222	ENSRNOT00000071312	1	0
TUCP_002966	XLOC_128222	ENSRNOT00000088245	1	0
TUCP_002966	XLOC_128222	ENSRNOT00000036565	0.969065335	2.12E-07
TUCP_002966	XLOC_128222	ENSRNOT00000058833	0.998673261	3.23E-14
TUCP_002966	XLOC_128222	ENSRNOT00000078052	1	0
TUCP_002966	XLOC_128222	ENSRNOT00000078494	0.999927093	1.62E-20
TUCP_002966	XLOC_128222	ENSRNOT00000085653	1	0
TUCP_002966	XLOC_128222	ENSRNOT00000057950	0.969329371	2.03E-07
TUCP_002966	XLOC_128222	ENSRNOT00000081992	0.999320526	1.14E-15
TUCP_002966	XLOC_128222	ENSRNOT00000038553	0.955765808	1.24E-06
TUCP_002966	XLOC_128222	ENSRNOT00000084120	0.958115447	9.46E-07
TUCP_002966	XLOC_128222	ENSRNOT00000055406	1	0
TUCP_002966	XLOC_128222	ENSRNOT00000070817	0.998968079	9.20E-15
TUCP_002966	XLOC_128222	ENSRNOT00000026753	1	0
TUCP_002966	XLOC_128222	ENSRNOT00000080533	0.996682529	3.15E-12
TUCP_002966	XLOC_128222	ENSRNOT00000072774	0.979665261	2.65E-08
TUCP_002966	XLOC_128222	ENSRNOT00000086031	1	0
TUCP_002966	XLOC_128222	ENSRNOT00000014312	1	0
TUCP_002966	XLOC_128222	ENSRNOT00000042882	0.963218427	4.98E-07
TUCP_002966	XLOC_128222	ENSRNOT00000088012	0.956765806	1.11E-06
TUCP_002966	XLOC_128222	ENSRNOT00000051231	0.999242378	1.96E-15
TUCP_002966	XLOC_128222	ENSRNOT00000089407	0.992986194	1.32E-10
TUCP_002966	XLOC_128222	ENSRNOT00000055034	0.998331441	1.02E-13
TUCP_002966	XLOC_128222	ENSRNOT00000046837	0.97252559	1.18E-07
TUCP_002963	XLOC_128011	ENSRNOT00000024186	0.985200254	5.45E-09
TUCP_002963	XLOC_128011	ENSRNOT00000074753	0.986206896	3.84E-09
TUCP_002963	XLOC_128011	ENSRNOT00000004876	0.967696156	2.62E-07
TUCP_002963	XLOC_128011	ENSRNOT00000041904	0.988092533	1.85E-09
TUCP_002963	XLOC_128011	ENSRNOT00000083487	0.98408912	7.82E-09
TUCP_002963	XLOC_128011	ENSRNOT00000077990	0.988092533	1.85E-09
TUCP_002963	XLOC_128011	ENSRNOT00000038826	0.988496221	1.56E-09
TUCP_002963	XLOC_128011	ENSRNOT00000046842	0.972697302	1.14E-07
TUCP_002963	XLOC_128011	ENSRNOT00000037169	0.968244541	2.41E-07
TUCP_002963	XLOC_128011	ENSRNOT00000021243	0.968929586	2.16E-07
TUCP_002963	XLOC_128011	ENSRNOT00000060250	0.987337326	2.51E-09
TUCP_002963	XLOC_128011	ENSRNOT00000091934	0.988092533	1.85E-09
TUCP_002963	XLOC_128011	ENSRNOT00000046585	0.990854241	4.96E-10
TUCP_002963	XLOC_128011	ENSRNOT00000080794	0.975494654	6.68E-08
TUCP_002963	XLOC_128011	ENSRNOT00000014456	0.974708393	7.81E-08
TUCP_002963	XLOC_128011	ENSRNOT00000031205	0.991570487	3.30E-10
TUCP_002963	XLOC_128011	ENSRNOT00000044145	0.988092533	1.85E-09
TUCP_002963	XLOC_128011	ENSRNOT00000088381	0.993459627	9.32E-11
TUCP_002963	XLOC_128011	ENSRNOT00000011743	0.982598339	1.22E-08
TUCP_002963	XLOC_128011	ENSRNOT00000089929	0.988092533	1.85E-09
TUCP_002963	XLOC_128011	ENSRNOT00000088624	0.988092533	1.85E-09
TUCP_002963	XLOC_128011	ENSRNOT00000085659	0.98172192	1.56E-08
TUCP_002963	XLOC_128011	ENSRNOT00000015866	0.988358505	1.65E-09
TUCP_002963	XLOC_128011	ENSRNOT00000078633	0.988092533	1.85E-09

TUCP_002963	XLOC_128011	ENSRNOT00000034907	0.992360862	2.02E-10
TUCP_002963	XLOC_128011	ENSRNOT00000093230	0.958622806	8.91E-07
TUCP_002963	XLOC_128011	ENSRNOT00000051835	0.988092533	1.85E-09
TUCP_002963	XLOC_128011	ENSRNOT00000009825	0.951867264	1.88E-06
TUCP_002963	XLOC_128011	ENSRNOT00000079087	0.988092533	1.85E-09
TUCP_002963	XLOC_128011	ENSRNOT00000037206	0.988092533	1.85E-09
TUCP_002963	XLOC_128011	ENSRNOT00000083261	0.966624475	3.08E-07
TUCP_002963	XLOC_128011	ENSRNOT00000080590	0.987699996	2.17E-09
TUCP_002963	XLOC_128011	ENSRNOT00000027552	0.987329272	2.52E-09
TUCP_002963	XLOC_128011	ENSRNOT00000077014	0.984155653	7.66E-09
TUCP_002963	XLOC_128011	ENSRNOT00000080453	0.988237068	1.74E-09
TUCP_002963	XLOC_128011	ENSRNOT00000089057	0.968683873	2.25E-07
TUCP_002963	XLOC_128011	ENSRNOT00000066839	0.975293717	6.96E-08
TUCP_002963	XLOC_128011	ENSRNOT00000061284	0.951447118	1.96E-06
TUCP_002963	XLOC_128011	ENSRNOT00000013423	0.969488743	1.98E-07
TUCP_002963	XLOC_128011	ENSRNOT00000071884	0.963854623	4.57E-07
TUCP_002963	XLOC_128011	ENSRNOT00000004128	0.965534244	3.61E-07
TUCP_002963	XLOC_128011	ENSRNOT00000057729	0.986249572	3.78E-09
TUCP_002963	XLOC_128011	ENSRNOT00000078663	0.958516417	9.02E-07
TUCP_002963	XLOC_128011	ENSRNOT00000064250	0.96947992	1.98E-07
TUCP_002963	XLOC_128011	ENSRNOT00000021608	0.96114511	6.53E-07
TUCP_002963	XLOC_128011	ENSRNOT00000029634	0.98057974	2.11E-08
TUCP_002963	XLOC_128011	ENSRNOT00000093579	0.966580276	3.10E-07
TUCP_002963	XLOC_128011	ENSRNOT00000065460	0.988092533	1.85E-09
TUCP_002963	XLOC_128011	ENSRNOT00000003817	0.981726723	1.56E-08
TUCP_002963	XLOC_128011	ENSRNOT00000016713	0.988092533	1.85E-09
TUCP_002963	XLOC_128011	ENSRNOT00000079260	0.991507823	3.43E-10
TUCP_002963	XLOC_128011	ENSRNOT00000039294	0.988092533	1.85E-09
TUCP_002963	XLOC_128011	ENSRNOT00000024337	0.96124143	6.45E-07
TUCP_002962	XLOC_127866	ENSRNOT00000024186	0.983200502	1.02E-08
TUCP_002962	XLOC_127866	ENSRNOT00000074753	0.998182349	1.56E-13
TUCP_002962	XLOC_127866	ENSRNOT00000004876	0.959071443	8.44E-07
TUCP_002962	XLOC_127866	ENSRNOT00000041904	1	0
TUCP_002962	XLOC_127866	ENSRNOT00000083487	0.989515314	9.80E-10
TUCP_002962	XLOC_127866	ENSRNOT00000077990	1	0
TUCP_002962	XLOC_127866	ENSRNOT00000038826	0.99977672	4.37E-18
TUCP_002962	XLOC_127866	ENSRNOT00000046842	0.973644314	9.58E-08
TUCP_002962	XLOC_127866	ENSRNOT00000037169	0.980329406	2.24E-08
TUCP_002962	XLOC_127866	ENSRNOT00000021243	0.987425406	2.42E-09
TUCP_002962	XLOC_127866	ENSRNOT00000060250	0.999933853	9.97E-21
TUCP_002962	XLOC_127866	ENSRNOT00000091934	1	1.33E-79
TUCP_002962	XLOC_127866	ENSRNOT00000046585	0.982941597	1.11E-08
TUCP_002962	XLOC_127866	ENSRNOT00000080794	0.990839253	5.00E-10
TUCP_002962	XLOC_127866	ENSRNOT00000014456	0.991697153	3.06E-10
TUCP_002962	XLOC_127866	ENSRNOT00000031205	0.991394766	3.66E-10
TUCP_002962	XLOC_127866	ENSRNOT00000044145	1	1.33E-79
TUCP_002962	XLOC_127866	ENSRNOT00000088381	0.989013276	1.24E-09
TUCP_002962	XLOC_127866	ENSRNOT00000011743	0.98678427	3.11E-09
TUCP_002962	XLOC_127866	ENSRNOT00000089929	1	1.33E-79
TUCP_002962	XLOC_127866	ENSRNOT00000088624	1	0
TUCP_002962	XLOC_127866	ENSRNOT00000085659	0.989908621	8.10E-10

TUCP_002962	XLOC_127866	ENSRNOT00000015866	0.994066664	5.73E-11
TUCP_002962	XLOC_127866	ENSRNOT00000078633	1	0
TUCP_002962	XLOC_127866	ENSRNOT00000034907	0.995318542	1.76E-11
TUCP_002962	XLOC_127866	ENSRNOT00000093230	0.958295175	9.26E-07
TUCP_002962	XLOC_127866	ENSRNOT00000051835	1	0
TUCP_002962	XLOC_127866	ENSRNOT00000079087	1	1.33E-79
TUCP_002962	XLOC_127866	ENSRNOT00000037206	1	0
TUCP_002962	XLOC_127866	ENSRNOT00000083261	0.970991658	1.54E-07
TUCP_002962	XLOC_127866	ENSRNOT00000080590	0.999889216	1.31E-19
TUCP_002962	XLOC_127866	ENSRNOT00000027552	0.999801024	2.46E-18
TUCP_002962	XLOC_127866	ENSRNOT00000077014	0.995074376	2.26E-11
TUCP_002962	XLOC_127866	ENSRNOT00000080453	0.99994332	4.61E-21
TUCP_002962	XLOC_127866	ENSRNOT00000089057	0.968243551	2.41E-07
TUCP_002962	XLOC_127866	ENSRNOT00000066839	0.991868612	2.76E-10
TUCP_002962	XLOC_127866	ENSRNOT00000061284	0.955566942	1.27E-06
TUCP_002962	XLOC_127866	ENSRNOT00000013423	0.959677621	7.84E-07
TUCP_002962	XLOC_127866	ENSRNOT00000071884	0.974769955	7.72E-08
TUCP_002962	XLOC_127866	ENSRNOT00000004128	0.961829362	5.99E-07
TUCP_002962	XLOC_127866	ENSRNOT00000026316	0.964408046	4.24E-07
TUCP_002962	XLOC_127866	ENSRNOT00000057729	0.999384184	6.97E-16
TUCP_002962	XLOC_127866	ENSRNOT00000078663	0.975192577	7.10E-08
TUCP_002962	XLOC_127866	ENSRNOT00000064250	0.983131977	1.05E-08
TUCP_002962	XLOC_127866	ENSRNOT00000021608	0.965228881	3.78E-07
TUCP_002962	XLOC_127866	ENSRNOT00000029634	0.995816134	1.00E-11
TUCP_002962	XLOC_127866	ENSRNOT00000093579	0.958564264	8.97E-07
TUCP_002962	XLOC_127866	ENSRNOT00000086349	0.957242511	1.05E-06
TUCP_002962	XLOC_127866	ENSRNOT00000065460	1	4.25E-78
TUCP_002962	XLOC_127866	ENSRNOT00000003817	0.996778712	2.72E-12
TUCP_002962	XLOC_127866	ENSRNOT00000016713	1	1.33E-79
TUCP_002962	XLOC_127866	ENSRNOT00000079260	0.996448245	4.42E-12
TUCP_002962	XLOC_127866	ENSRNOT00000039294	1	1.33E-79
TUCP_002962	XLOC_127866	ENSRNOT00000092913	0.952980228	1.67E-06
TUCP_002969	XLOC_128258	ENSRNOT00000010873	-0.951198515	2.01E-06
TUCP_002969	XLOC_128258	ENSRNOT00000014821	-0.956939512	1.08E-06
TUCP_002969	XLOC_128258	ENSRNOT00000044885	0.967852657	2.56E-07
TUCP_002969	XLOC_128258	ENSRNOT00000084055	-0.951771406	1.89E-06
TUCP_002969	XLOC_128258	ENSRNOT00000003690	0.952894691	1.69E-06
TUCP_002969	XLOC_128258	ENSRNOT00000085736	-0.964270649	4.32E-07
TUCP_002969	XLOC_128258	ENSRNOT00000032780	0.963096087	5.07E-07
TUCP_002969	XLOC_128258	ENSRNOT00000081981	0.965198794	3.79E-07
TUCP_002969	XLOC_128258	ENSRNOT00000011350	0.951940738	1.86E-06
TUCP_002969	XLOC_128258	ENSRNOT00000063873	0.953233205	1.63E-06
TUCP_002969	XLOC_128258	ENSRNOT00000084422	0.957210522	1.05E-06
TUCP_002969	XLOC_128258	ENSRNOT00000014924	-0.951987506	1.85E-06
TUCP_002969	XLOC_128258	ENSRNOT00000002443	0.953937233	1.51E-06
TUCP_002969	XLOC_128258	ENSRNOT00000000181	0.961715035	6.07E-07
TUCP_002969	XLOC_128258	ENSRNOT00000002114	-0.954522708	1.42E-06
TUCP_002969	XLOC_128258	ENSRNOT00000022142	0.95414482	1.48E-06
TUCP_002969	XLOC_128258	ENSRNOT00000080608	0.961471891	6.27E-07
TUCP_002969	XLOC_128258	ENSRNOT00000009563	-0.976664346	5.24E-08
TUCP_002969	XLOC_128258	ENSRNOT00000010748	0.954523938	1.42E-06

TUCP_002969	XLOC_128258	ENSRNOT00000012334	0.957912866	9.69E-07
TUCP_002969	XLOC_128258	ENSRNOT00000015537	0.958679128	8.85E-07
TUCP_002969	XLOC_128258	ENSRNOT00000019133	0.952503064	1.76E-06
TUCP_002969	XLOC_128258	ENSRNOT00000010784	0.951083885	2.03E-06
TUCP_002969	XLOC_128258	ENSRNOT00000049459	-0.96153139	6.22E-07
TUCP_002969	XLOC_128258	ENSRNOT00000045326	0.958774334	8.75E-07
TUCP_002969	XLOC_128258	ENSRNOT00000003714	0.960043398	7.50E-07
TUCP_002969	XLOC_128258	ENSRNOT00000015947	0.969302506	2.04E-07
TUCP_002969	XLOC_128258	ENSRNOT00000046745	0.953205512	1.63E-06
TUCP_002969	XLOC_128258	ENSRNOT00000028856	0.953205792	1.63E-06
TUCP_002969	XLOC_128258	ENSRNOT00000078856	-0.951462317	1.96E-06
TUCP_002969	XLOC_128258	ENSRNOT00000014124	0.950819883	2.09E-06
TUCP_002969	XLOC_128258	ENSRNOT00000000120	0.971667222	1.37E-07
TUCP_002969	XLOC_128258	ENSRNOT00000005958	0.961369391	6.35E-07
TUCP_002969	XLOC_128258	ENSRNOT00000029125	0.950062471	2.25E-06
TUCP_002969	XLOC_128258	ENSRNOT00000055321	0.95060475	2.13E-06
TUCP_002969	XLOC_128258	ENSRNOT00000013801	0.951644367	1.92E-06
TUCP_002969	XLOC_128258	ENSRNOT00000042125	0.957885916	9.72E-07
TUCP_002969	XLOC_128258	ENSRNOT00000006752	0.952499291	1.76E-06
TUCP_002969	XLOC_128258	ENSRNOT00000060698	-0.957093894	1.07E-06
TUCP_002969	XLOC_128258	ENSRNOT00000004172	0.952208148	1.81E-06
TUCP_002969	XLOC_128258	ENSRNOT00000027970	0.967075694	2.88E-07
TUCP_002969	XLOC_128258	ENSRNOT00000058351	0.97548938	6.69E-08
TUCP_002969	XLOC_128258	ENSRNOT00000027616	-0.953480972	1.59E-06
TUCP_002969	XLOC_128258	ENSRNOT00000002109	0.955446248	1.28E-06
TUCP_002969	XLOC_128258	ENSRNOT00000004319	-0.952597352	1.74E-06
TUCP_002969	XLOC_128258	ENSRNOT00000024969	0.950054223	2.25E-06
TUCP_002969	XLOC_128258	ENSRNOT00000023537	-0.96005495	7.49E-07
TUCP_002969	XLOC_128258	ENSRNOT00000059181	0.952050463	1.84E-06
TUCP_002969	XLOC_128258	ENSRNOT00000017776	0.954520792	1.42E-06
TUCP_002969	XLOC_128258	ENSRNOT00000005901	0.954435917	1.43E-06
TUCP_002969	XLOC_128258	ENSRNOT00000086893	-0.956194317	1.18E-06
TUCP_002969	XLOC_128258	ENSRNOT00000000013	0.957430252	1.02E-06
TUCP_002969	XLOC_128258	ENSRNOT00000067780	-0.962659952	5.37E-07
TUCP_002969	XLOC_128258	ENSRNOT00000084031	0.955235507	1.31E-06
TUCP_002969	XLOC_128258	ENSRNOT00000008982	0.964315888	4.29E-07
TUCP_002969	XLOC_128258	ENSRNOT00000034692	-0.956138506	1.19E-06
TUCP_002969	XLOC_128258	ENSRNOT00000004046	-0.966574371	3.11E-07
TUCP_002969	XLOC_128258	ENSRNOT00000000170	-0.950399978	2.17E-06
TUCP_002969	XLOC_128258	ENSRNOT00000020681	-0.95932882	8.18E-07
TUCP_002969	XLOC_128258	ENSRNOT00000040476	0.963096242	5.07E-07
TUCP_002969	XLOC_128258	ENSRNOT00000031977	0.961202037	6.49E-07
TUCP_002969	XLOC_128258	ENSRNOT00000089933	0.955368729	1.29E-06
TUCP_002969	XLOC_128258	ENSRNOT00000055442	0.955553012	1.27E-06
TUCP_002969	XLOC_128258	ENSRNOT00000010983	0.952860838	1.69E-06
TUCP_002969	XLOC_128258	ENSRNOT00000078102	0.960801007	6.82E-07
TUCP_002969	XLOC_128258	ENSRNOT00000066133	-0.950516714	2.15E-06
TUCP_002968	XLOC_128225	ENSRNOT00000030300	0.960691615	6.92E-07
TUCP_002968	XLOC_128225	ENSRNOT00000086650	-0.962641202	5.38E-07
TUCP_002880	XLOC_122988	ENSRNOT00000085195	0.999404969	5.87E-16
TUCP_002880	XLOC_122988	ENSRNOT00000013705	1	0

TUCP_002880	XLOC_122988	ENSRNOT00000040851	1	0
TUCP_002880	XLOC_122988	ENSRNOT00000075676	0.958697783	8.83E-07
TUCP_002880	XLOC_122988	ENSRNOT00000058389	1	0
TUCP_002880	XLOC_122988	ENSRNOT00000086827	0.95592964	1.22E-06
TUCP_002880	XLOC_122988	ENSRNOT00000023542	1	0
TUCP_002880	XLOC_122988	ENSRNOT00000028653	1	0
TUCP_002880	XLOC_122988	ENSRNOT00000017230	0.955378945	1.29E-06
TUCP_002880	XLOC_122988	ENSRNOT00000092890	0.955137645	1.33E-06
TUCP_002880	XLOC_122988	ENSRNOT00000012174	1	0
TUCP_002880	XLOC_122988	ENSRNOT00000051680	0.994991197	2.46E-11
TUCP_002880	XLOC_122988	ENSRNOT00000089118	1	1.33E-79
TUCP_002880	XLOC_122988	ENSRNOT00000025245	0.999967061	3.05E-22
TUCP_002880	XLOC_122988	ENSRNOT00000015138	0.999902179	7.05E-20
TUCP_002880	XLOC_122988	ENSRNOT00000080185	0.99902259	7.01E-15
TUCP_002880	XLOC_122988	ENSRNOT00000090729	0.965956282	3.40E-07
TUCP_002880	XLOC_122988	ENSRNOT00000049165	0.973440588	9.95E-08
TUCP_002880	XLOC_122988	ENSRNOT00000086502	0.96495109	3.93E-07
TUCP_002880	XLOC_122988	ENSRNOT00000079661	1	0
TUCP_002880	XLOC_122988	ENSRNOT00000076338	0.985532463	4.87E-09
TUCP_002880	XLOC_122988	ENSRNOT00000042220	1	4.25E-78
TUCP_002880	XLOC_122988	ENSRNOT00000005031	0.952974465	1.67E-06
TUCP_002880	XLOC_122988	ENSRNOT00000079167	0.979848988	2.53E-08
TUCP_002880	XLOC_122988	ENSRNOT00000061950	0.962035217	5.83E-07
TUCP_002880	XLOC_122988	ENSRNOT00000086710	0.999839781	8.31E-19
TUCP_002880	XLOC_122988	ENSRNOT00000075226	1	0
TUCP_002880	XLOC_122988	ENSRNOT00000000351	1	0
TUCP_002880	XLOC_122988	ENSRNOT00000092294	0.986520908	3.43E-09
TUCP_003614	XLOC_158914	ENSRNOT00000059895	0.967046449	2.90E-07
TUCP_003614	XLOC_158914	ENSRNOT00000059172	0.970209107	1.76E-07
TUCP_003614	XLOC_158914	ENSRNOT00000013836	0.954532441	1.42E-06
TUCP_003614	XLOC_158914	ENSRNOT00000008154	0.95394938	1.51E-06
TUCP_003614	XLOC_158914	ENSRNOT00000072396	0.957669718	9.97E-07
TUCP_003614	XLOC_158914	ENSRNOT00000079549	0.955053424	1.34E-06
TUCP_003614	XLOC_158914	ENSRNOT00000093577	0.950796314	2.09E-06
TUCP_003619	XLOC_158964	ENSRNOT00000023332	0.973273522	1.03E-07
TUCP_003619	XLOC_158964	ENSRNOT00000077185	0.958912949	8.61E-07
TUCP_000885	XLOC_032964	ENSRNOT00000055536	0.961361716	6.36E-07
TUCP_000885	XLOC_032964	ENSRNOT00000010852	0.986337394	3.66E-09
TUCP_000885	XLOC_032964	ENSRNOT00000082711	0.956431491	1.15E-06
TUCP_000885	XLOC_032964	ENSRNOT00000017805	0.953320184	1.61E-06
TUCP_000885	XLOC_032964	ENSRNOT00000001893	0.962572706	5.43E-07
TUCP_000885	XLOC_032964	ENSRNOT00000061054	0.952839283	1.70E-06
TUCP_000885	XLOC_032964	ENSRNOT00000081226	0.957126118	1.06E-06
TUCP_000885	XLOC_032964	ENSRNOT00000014926	0.956640518	1.12E-06
TUCP_000885	XLOC_032964	ENSRNOT00000009080	-0.956072205	1.20E-06
TUCP_000885	XLOC_032964	ENSRNOT00000057738	0.956978456	1.08E-06
TUCP_000885	XLOC_032964	ENSRNOT00000081631	0.959080825	8.43E-07
TUCP_000885	XLOC_032964	ENSRNOT00000007461	0.951683294	1.91E-06
TUCP_000885	XLOC_032964	ENSRNOT00000049349	0.95086272	2.08E-06
TUCP_000885	XLOC_032964	ENSRNOT00000013025	-0.95006013	2.25E-06
TUCP_000885	XLOC_032964	ENSRNOT00000011832	-0.950447901	2.16E-06

TUCP_000885	XLOC_032964	ENSRNOT00000026426	0.957463634	1.02E-06
TUCP_000885	XLOC_032964	ENSRNOT00000009462	0.956136424	1.19E-06
TUCP_000885	XLOC_032964	ENSRNOT00000005343	-0.974121305	8.75E-08
TUCP_000885	XLOC_032964	ENSRNOT00000086706	0.953076352	1.66E-06
TUCP_000885	XLOC_032964	ENSRNOT00000073370	0.964927854	3.94E-07
TUCP_000885	XLOC_032964	ENSRNOT00000016485	0.958103073	9.48E-07
TUCP_000885	XLOC_032964	ENSRNOT00000065615	0.972811575	1.12E-07
TUCP_000885	XLOC_032964	ENSRNOT00000087838	-0.950707899	2.11E-06
TUCP_000885	XLOC_032964	ENSRNOT00000024452	-0.951624251	1.92E-06
TUCP_000885	XLOC_032964	ENSRNOT00000008244	0.958501414	9.04E-07
TUCP_000885	XLOC_032964	ENSRNOT00000042638	0.971660349	1.37E-07
TUCP_000885	XLOC_032964	ENSRNOT00000072580	-0.952407252	1.77E-06
TUCP_000885	XLOC_032964	ENSRNOT00000039540	0.95668635	1.12E-06
TUCP_000885	XLOC_032964	ENSRNOT00000000469	0.961433051	6.30E-07
TUCP_000885	XLOC_032964	ENSRNOT00000038652	-0.961855914	5.96E-07
TUCP_000885	XLOC_032964	ENSRNOT00000017384	0.96953681	1.96E-07
TUCP_000885	XLOC_032964	ENSRNOT00000028685	0.954467241	1.43E-06
TUCP_000885	XLOC_032964	ENSRNOT00000065076	0.953833411	1.53E-06
TUCP_000885	XLOC_032964	ENSRNOT00000080577	0.967463974	2.72E-07
TUCP_000885	XLOC_032964	ENSRNOT00000057562	0.956603789	1.13E-06
TUCP_000885	XLOC_032964	ENSRNOT00000072118	0.958247018	9.32E-07
TUCP_000885	XLOC_032964	ENSRNOT00000054685	0.962334353	5.60E-07
TUCP_000885	XLOC_032964	ENSRNOT00000085334	0.95780152	9.82E-07
TUCP_000885	XLOC_032964	ENSRNOT00000090700	0.958065508	9.52E-07
TUCP_000885	XLOC_032964	ENSRNOT00000057953	0.96343845	4.84E-07
TUCP_000885	XLOC_032964	ENSRNOT00000074515	-0.951444699	1.96E-06
TUCP_000885	XLOC_032964	ENSRNOT00000008764	0.968008088	2.50E-07
TUCP_000885	XLOC_032964	ENSRNOT00000009009	0.950339294	2.19E-06
TUCP_000885	XLOC_032964	ENSRNOT00000016060	0.98107776	1.85E-08
TUCP_000885	XLOC_032964	ENSRNOT00000035309	-0.962217333	5.69E-07
TUCP_000885	XLOC_032964	ENSRNOT00000052033	-0.954374906	1.44E-06
TUCP_000885	XLOC_032964	ENSRNOT00000086260	-0.966787287	3.01E-07
TUCP_000885	XLOC_032964	ENSRNOT00000065871	0.95003262	2.26E-06
TUCP_000885	XLOC_032964	ENSRNOT00000036357	0.950021886	2.26E-06
TUCP_000885	XLOC_032964	ENSRNOT00000025355	0.961574971	6.18E-07
TUCP_000885	XLOC_032964	ENSRNOT00000091046	0.950618312	2.13E-06
TUCP_001788	XLOC_073522	ENSRNOT00000008961	0.950201498	2.22E-06
TUCP_001788	XLOC_073522	ENSRNOT00000022724	0.955079979	1.34E-06
TUCP_001788	XLOC_073522	ENSRNOT00000026303	0.964779954	4.02E-07
TUCP_001788	XLOC_073522	ENSRNOT00000073547	0.954142674	1.48E-06
TUCP_001788	XLOC_073522	ENSRNOT00000042865	-0.954751204	1.38E-06
TUCP_001788	XLOC_073522	ENSRNOT00000092125	0.951565573	1.93E-06
TUCP_001788	XLOC_073522	ENSRNOT00000058626	0.971825912	1.33E-07
TUCP_001788	XLOC_073522	ENSRNOT00000005085	0.955957961	1.21E-06
TUCP_001788	XLOC_073522	ENSRNOT00000031667	0.954357374	1.44E-06
TUCP_001788	XLOC_073522	ENSRNOT00000009077	0.973232779	1.03E-07
TUCP_001788	XLOC_073522	ENSRNOT00000088151	0.983833298	8.46E-09
TUCP_001788	XLOC_073522	ENSRNOT00000010264	0.960484894	7.10E-07
TUCP_001658	XLOC_065680	ENSRNOT00000010092	0.952126226	1.83E-06
TUCP_001658	XLOC_065680	ENSRNOT00000078434	0.958724139	8.80E-07
TUCP_001658	XLOC_065680	ENSRNOT00000077134	0.950428847	2.17E-06

TUCP_001658	XLOC_065680	ENSRNOT00000052003	0.953991598	1.50E-06
TUCP_001658	XLOC_065680	ENSRNOT00000076693	0.959726204	7.80E-07
TUCP_001658	XLOC_065680	ENSRNOT00000047527	0.953696687	1.55E-06
TUCP_001658	XLOC_065680	ENSRNOT00000039263	0.953901725	1.52E-06
TUCP_001658	XLOC_065680	ENSRNOT00000020071	0.95022404	2.21E-06
TUCP_001658	XLOC_065680	ENSRNOT00000047700	0.962561763	5.44E-07
TUCP_001658	XLOC_065680	ENSRNOT00000076802	0.95035553	2.18E-06
TUCP_001658	XLOC_065680	ENSRNOT00000038494	0.96041675	7.16E-07
TUCP_001658	XLOC_065680	ENSRNOT00000057433	0.963484909	4.81E-07
TUCP_001658	XLOC_065680	ENSRNOT00000082143	0.952744426	1.71E-06
TUCP_001658	XLOC_065680	ENSRNOT00000087603	0.961009542	6.65E-07
TUCP_001658	XLOC_065680	ENSRNOT00000072810	0.95383773	1.53E-06
TUCP_001601	XLOC_063401	ENSRNOT00000013914	-0.964868668	3.97E-07
TUCP_001601	XLOC_063401	ENSRNOT00000012635	-0.966409735	3.18E-07
TUCP_001601	XLOC_063401	ENSRNOT00000009708	-0.951703609	1.91E-06
TUCP_001601	XLOC_063401	ENSRNOT00000051554	-0.966127912	3.32E-07
TUCP_001601	XLOC_063401	ENSRNOT00000049927	-0.961713404	6.08E-07
TUCP_001601	XLOC_063401	ENSRNOT00000077579	-0.957449684	1.02E-06
TUCP_001601	XLOC_063401	ENSRNOT00000020663	-0.964044038	4.46E-07
TUCP_001601	XLOC_063401	ENSRNOT00000073636	0.955649815	1.25E-06
TUCP_001601	XLOC_063401	ENSRNOT00000028589	-0.95616549	1.18E-06
TUCP_001601	XLOC_063401	ENSRNOT00000072110	-0.963109227	5.06E-07
TUCP_001601	XLOC_063401	ENSRNOT00000083173	-0.951596742	1.93E-06
TUCP_001601	XLOC_063401	ENSRNOT00000024120	-0.970504418	1.67E-07
TUCP_001601	XLOC_063401	ENSRNOT00000028625	-0.973486951	9.87E-08
TUCP_001601	XLOC_063401	ENSRNOT00000016386	-0.961038221	6.62E-07
TUCP_001601	XLOC_063401	ENSRNOT00000021075	-0.964429175	4.22E-07
TUCP_001601	XLOC_063401	ENSRNOT00000021073	-0.95647737	1.14E-06
TUCP_001601	XLOC_063401	ENSRNOT00000025550	-0.954317759	1.45E-06
TUCP_001601	XLOC_063401	ENSRNOT00000015808	-0.957349905	1.03E-06
TUCP_001601	XLOC_063401	ENSRNOT00000008364	-0.95436896	1.44E-06
TUCP_001601	XLOC_063401	ENSRNOT00000019463	-0.960177142	7.38E-07
TUCP_001601	XLOC_063401	ENSRNOT00000060432	-0.954347309	1.45E-06
TUCP_001601	XLOC_063401	ENSRNOT00000091864	-0.950591833	2.13E-06
TUCP_001601	XLOC_063401	ENSRNOT00000004942	-0.963636721	4.71E-07
TUCP_001601	XLOC_063401	ENSRNOT00000006699	-0.960690909	6.92E-07
TUCP_001601	XLOC_063401	ENSRNOT00000007002	-0.972337282	1.22E-07
TUCP_001601	XLOC_063401	ENSRNOT00000003540	-0.974486787	8.16E-08
TUCP_001601	XLOC_063401	ENSRNOT00000055988	-0.962086816	5.79E-07
TUCP_001601	XLOC_063401	ENSRNOT00000025936	-0.950423078	2.17E-06
TUCP_001601	XLOC_063401	ENSRNOT00000067846	-0.97504335	7.31E-08
TUCP_001601	XLOC_063401	ENSRNOT00000061916	-0.950985389	2.05E-06
TUCP_001601	XLOC_063401	ENSRNOT00000002052	-0.968307581	2.39E-07
TUCP_001601	XLOC_063401	ENSRNOT00000001968	-0.95764269	1.00E-06
TUCP_001601	XLOC_063401	ENSRNOT00000006900	-0.95734098	1.04E-06
TUCP_001601	XLOC_063401	ENSRNOT00000090420	-0.953837229	1.53E-06
TUCP_001601	XLOC_063401	ENSRNOT00000019886	0.951849244	1.88E-06
TUCP_003617	XLOC_158936	ENSRNOT00000006947	0.976559777	5.36E-08
TUCP_003617	XLOC_158936	ENSRNOT00000005505	0.959835083	7.69E-07
TUCP_003617	XLOC_158936	ENSRNOT00000028818	0.953200631	1.63E-06
TUCP_003617	XLOC_158936	ENSRNOT00000067889	0.953852795	1.53E-06

TUCP_003617	XLOC_158936	ENSRNOT00000083134	0.957580735	1.01E-06
TUCP_003617	XLOC_158936	ENSRNOT00000010142	0.977568185	4.31E-08
TUCP_003617	XLOC_158936	ENSRNOT00000047453	0.958419012	9.13E-07
TUCP_003617	XLOC_158936	ENSRNOT00000038449	0.96114734	6.53E-07
TUCP_003617	XLOC_158936	ENSRNOT00000081604	0.967358403	2.76E-07
TUCP_003617	XLOC_158936	ENSRNOT00000013343	0.953249664	1.63E-06
TUCP_003617	XLOC_158936	ENSRNOT00000006109	0.950235965	2.21E-06
TUCP_003617	XLOC_158936	ENSRNOT00000073493	0.954694207	1.39E-06
TUCP_000678	XLOC_025601	ENSRNOT00000008685	0.974973091	7.41E-08
TUCP_000678	XLOC_025601	ENSRNOT00000056243	0.951235393	2.00E-06
TUCP_000678	XLOC_025601	ENSRNOT00000009111	0.961685082	6.10E-07
TUCP_000678	XLOC_025601	ENSRNOT00000054915	0.953338165	1.61E-06
TUCP_000678	XLOC_025601	ENSRNOT00000089193	0.954333753	1.45E-06
TUCP_003529	XLOC_153608	ENSRNOT00000085943	0.982115243	1.40E-08
TUCP_003529	XLOC_153608	ENSRNOT00000011342	0.974496895	8.14E-08
TUCP_003529	XLOC_153608	ENSRNOT00000076466	0.96221506	5.69E-07
TUCP_003529	XLOC_153608	ENSRNOT00000072396	0.999859744	4.27E-19
TUCP_003529	XLOC_153608	ENSRNOT00000093577	0.973330006	1.02E-07
TUCP_001218	XLOC_045681	ENSRNOT00000085696	-0.959817663	7.71E-07
TUCP_004246	XLOC_186698	ENSRNOT00000005958	0.950007939	2.26E-06
TUCP_004246	XLOC_186698	ENSRNOT00000000383	-0.951929993	1.86E-06
TUCP_004246	XLOC_186698	ENSRNOT00000090057	0.959458073	8.06E-07
TUCP_004246	XLOC_186698	ENSRNOT00000061253	0.975204789	7.08E-08
TUCP_004246	XLOC_186698	ENSRNOT00000041044	0.95714708	1.06E-06
TUCP_004246	XLOC_186698	ENSRNOT00000067400	0.959044712	8.47E-07
TUCP_004531	XLOC_199661	ENSRNOT00000092569	-0.953796462	1.53E-06
TUCP_003964	XLOC_174241	ENSRNOT00000084133	1	0
TUCP_003964	XLOC_174241	ENSRNOT00000093745	0.981891039	1.49E-08
TUCP_003964	XLOC_174241	ENSRNOT00000065542	0.99828699	1.16E-13
TUCP_003964	XLOC_174241	ENSRNOT00000082464	1	0
TUCP_003964	XLOC_174241	ENSRNOT00000087659	1	0
TUCP_003964	XLOC_174241	ENSRNOT00000084582	0.976633579	5.28E-08
TUCP_003964	XLOC_174241	ENSRNOT00000085078	1	1.33E-79
TUCP_003964	XLOC_174241	ENSRNOT00000014028	1	1.33E-79
TUCP_003964	XLOC_174241	ENSRNOT00000049363	1	1.33E-79
TUCP_003964	XLOC_174241	ENSRNOT00000065473	0.999949098	2.69E-21
TUCP_003964	XLOC_174241	ENSRNOT00000000142	0.998996155	8.01E-15
TUCP_003964	XLOC_174241	ENSRNOT00000082288	0.999998398	8.30E-29
TUCP_003964	XLOC_174241	ENSRNOT00000039664	1	0
TUCP_003964	XLOC_174241	ENSRNOT00000065402	1	1.33E-79
TUCP_003964	XLOC_174241	ENSRNOT00000064551	0.965365872	3.70E-07
TUCP_003964	XLOC_174241	ENSRNOT00000084141	1	0
TUCP_003964	XLOC_174241	ENSRNOT00000082435	1	0
TUCP_003964	XLOC_174241	ENSRNOT00000082928	0.996801059	2.62E-12
TUCP_003964	XLOC_174241	ENSRNOT00000066546	0.956953619	1.08E-06
TUCP_003964	XLOC_174241	ENSRNOT00000046013	0.988138626	1.81E-09
TUCP_003964	XLOC_174241	ENSRNOT00000067931	0.978893628	3.18E-08
TUCP_003964	XLOC_174241	ENSRNOT00000086425	0.992023315	2.51E-10
TUCP_003964	XLOC_174241	ENSRNOT00000093696	0.956927219	1.09E-06
TUCP_003964	XLOC_174241	ENSRNOT00000036228	0.982337157	1.31E-08
TUCP_003964	XLOC_174241	ENSRNOT00000014030	1	0

TUCP_003964	XLOC_174241	ENSRNOT00000081966	1	1.33E-79
TUCP_003964	XLOC_174241	ENSRNOT00000075906	1	0
TUCP_003964	XLOC_174241	ENSRNOT00000088396	0.981336994	1.73E-08
TUCP_003964	XLOC_174241	ENSRNOT00000049259	0.965500259	3.63E-07
TUCP_003964	XLOC_174241	ENSRNOT00000092838	0.982579749	1.23E-08
TUCP_003964	XLOC_174241	ENSRNOT00000093442	0.959217317	8.30E-07
TUCP_004245	XLOC_186698	ENSRNOT00000009811	0.963524159	4.78E-07
TUCP_004245	XLOC_186698	ENSRNOT00000065168	0.955515126	1.27E-06
TUCP_004245	XLOC_186698	ENSRNOT00000005486	-0.953650861	1.56E-06
TUCP_004245	XLOC_186698	ENSRNOT00000067554	0.976525295	5.40E-08
TUCP_004245	XLOC_186698	ENSRNOT00000083214	0.959328962	8.18E-07
TUCP_004245	XLOC_186698	ENSRNOT00000082711	0.962889676	5.21E-07
TUCP_004245	XLOC_186698	ENSRNOT00000010784	0.976511462	5.41E-08
TUCP_004245	XLOC_186698	ENSRNOT00000026531	-0.954006052	1.50E-06
TUCP_004245	XLOC_186698	ENSRNOT00000029328	0.96740788	2.74E-07
TUCP_004245	XLOC_186698	ENSRNOT00000085619	0.951192369	2.01E-06
TUCP_004245	XLOC_186698	ENSRNOT00000000468	0.952995297	1.67E-06
TUCP_004245	XLOC_186698	ENSRNOT00000077231	0.95517817	1.32E-06
TUCP_004245	XLOC_186698	ENSRNOT00000005839	0.971350377	1.45E-07
TUCP_004245	XLOC_186698	ENSRNOT00000017687	-0.952078654	1.84E-06
TUCP_004245	XLOC_186698	ENSRNOT00000000181	0.959845607	7.68E-07
TUCP_004245	XLOC_186698	ENSRNOT00000080443	0.95112475	2.02E-06
TUCP_004245	XLOC_186698	ENSRNOT00000058563	0.956290407	1.17E-06
TUCP_004245	XLOC_186698	ENSRNOT00000023989	0.955023957	1.34E-06
TUCP_004245	XLOC_186698	ENSRNOT00000073559	0.959101546	8.41E-07
TUCP_004245	XLOC_186698	ENSRNOT00000005405	0.966409535	3.18E-07
TUCP_004245	XLOC_186698	ENSRNOT00000047264	0.952137532	1.82E-06
TUCP_004245	XLOC_186698	ENSRNOT00000080577	0.967497229	2.71E-07
TUCP_004245	XLOC_186698	ENSRNOT00000089319	0.95157209	1.93E-06
TUCP_004245	XLOC_186698	ENSRNOT00000009462	0.969294578	2.04E-07
TUCP_004245	XLOC_186698	ENSRNOT00000027012	0.951453921	1.96E-06
TUCP_004245	XLOC_186698	ENSRNOT00000082904	0.950012812	2.26E-06
TUCP_004245	XLOC_186698	ENSRNOT00000078994	0.962651675	5.38E-07
TUCP_004245	XLOC_186698	ENSRNOT00000081725	0.9509977	2.05E-06
TUCP_004245	XLOC_186698	ENSRNOT00000074972	0.956581322	1.13E-06
TUCP_004245	XLOC_186698	ENSRNOT00000057361	0.972799714	1.12E-07
TUCP_004245	XLOC_186698	ENSRNOT00000086706	0.966373249	3.20E-07
TUCP_004245	XLOC_186698	ENSRNOT00000073370	0.956859752	1.09E-06
TUCP_004245	XLOC_186698	ENSRNOT00000004017	0.972294541	1.23E-07
TUCP_004245	XLOC_186698	ENSRNOT00000021114	0.972181138	1.25E-07
TUCP_004245	XLOC_186698	ENSRNOT00000046745	0.955676109	1.25E-06
TUCP_004245	XLOC_186698	ENSRNOT00000055607	-0.95042129	2.17E-06
TUCP_004245	XLOC_186698	ENSRNOT00000006340	0.951812065	1.89E-06
TUCP_004245	XLOC_186698	ENSRNOT00000017556	0.964819346	4.00E-07
TUCP_004245	XLOC_186698	ENSRNOT00000080608	0.956299875	1.17E-06
TUCP_004245	XLOC_186698	ENSRNOT00000030850	0.951181723	2.01E-06
TUCP_004245	XLOC_186698	ENSRNOT00000040476	0.951059587	2.04E-06
TUCP_004245	XLOC_186698	ENSRNOT00000002410	0.950032794	2.26E-06
TUCP_004245	XLOC_186698	ENSRNOT00000001893	0.978724367	3.31E-08
TUCP_004245	XLOC_186698	ENSRNOT00000061201	-0.950091386	2.24E-06
TUCP_004245	XLOC_186698	ENSRNOT00000013801	0.955449956	1.28E-06

TUCP_004245	XLOC_186698	ENSRNOT00000007028	-0.976952464	4.93E-08
TUCP_004245	XLOC_186698	ENSRNOT00000000469	0.954745667	1.39E-06
TUCP_004245	XLOC_186698	ENSRNOT00000003265	0.957538744	1.01E-06
TUCP_004245	XLOC_186698	ENSRNOT00000020028	0.95161927	1.92E-06
TUCP_004245	XLOC_186698	ENSRNOT00000015006	0.951423348	1.96E-06
TUCP_004245	XLOC_186698	ENSRNOT00000057562	0.9803946	2.21E-08
TUCP_004245	XLOC_186698	ENSRNOT00000001289	0.963103205	5.06E-07
TUCP_004245	XLOC_186698	ENSRNOT00000072118	0.958276089	9.28E-07
TUCP_004245	XLOC_186698	ENSRNOT00000002109	0.958507569	9.03E-07
TUCP_004245	XLOC_186698	ENSRNOT00000015769	0.960570096	7.02E-07
TUCP_004245	XLOC_186698	ENSRNOT00000022190	-0.950507165	2.15E-06
TUCP_004245	XLOC_186698	ENSRNOT00000013049	0.950709135	2.11E-06
TUCP_004245	XLOC_186698	ENSRNOT00000024119	0.954280176	1.46E-06
TUCP_004245	XLOC_186698	ENSRNOT00000019133	0.950321885	2.19E-06
TUCP_004245	XLOC_186698	ENSRNOT00000035277	0.951801156	1.89E-06
TUCP_004245	XLOC_186698	ENSRNOT00000087583	0.954807661	1.38E-06
TUCP_004245	XLOC_186698	ENSRNOT00000074683	0.955261074	1.31E-06
TUCP_004245	XLOC_186698	ENSRNOT00000015223	0.951156027	2.02E-06
TUCP_004245	XLOC_186698	ENSRNOT00000019921	0.955763926	1.24E-06
TUCP_004245	XLOC_186698	ENSRNOT00000022074	0.950678389	2.12E-06
TUCP_004245	XLOC_186698	ENSRNOT00000018122	0.951779368	1.89E-06
TUCP_004245	XLOC_186698	ENSRNOT00000013321	0.95207092	1.84E-06
TUCP_004245	XLOC_186698	ENSRNOT00000015898	0.957707433	9.92E-07
TUCP_004245	XLOC_186698	ENSRNOT00000026312	0.962340852	5.60E-07
TUCP_004245	XLOC_186698	ENSRNOT00000090057	0.969255796	2.05E-07
TUCP_004245	XLOC_186698	ENSRNOT00000010748	0.950551518	2.14E-06
TUCP_004245	XLOC_186698	ENSRNOT00000041365	0.952700008	1.72E-06
TUCP_004245	XLOC_186698	ENSRNOT00000057953	0.969073239	2.12E-07
TUCP_004245	XLOC_186698	ENSRNOT00000086260	-0.952102969	1.83E-06
TUCP_004245	XLOC_186698	ENSRNOT00000068381	0.960025254	7.52E-07
TUCP_004245	XLOC_186698	ENSRNOT00000007821	0.968062707	2.48E-07
TUCP_004245	XLOC_186698	ENSRNOT00000063951	0.952561976	1.75E-06
TUCP_004245	XLOC_186698	ENSRNOT00000089933	0.951330624	1.98E-06
TUCP_004245	XLOC_186698	ENSRNOT00000015799	0.955417406	1.29E-06
TUCP_004534	XLOC_199820	ENSRNOT00000027644	0.961054087	6.61E-07
TUCP_004534	XLOC_199820	ENSRNOT00000090621	-0.957146322	1.06E-06
TUCP_004534	XLOC_199820	ENSRNOT00000001979	0.951233377	2.00E-06
TUCP_004534	XLOC_199820	ENSRNOT00000067375	0.970256801	1.74E-07
TUCP_004534	XLOC_199820	ENSRNOT00000049349	-0.975235917	7.04E-08
TUCP_004534	XLOC_199820	ENSRNOT00000078326	0.958989328	8.53E-07
TUCP_004534	XLOC_199820	ENSRNOT00000010031	0.951437218	1.96E-06
TUCP_004534	XLOC_199820	ENSRNOT00000014063	0.968913547	2.17E-07
TUCP_004534	XLOC_199820	ENSRNOT00000015962	0.961529803	6.22E-07
TUCP_004534	XLOC_199820	ENSRNOT00000052063	-0.960202237	7.35E-07
TUCP_004534	XLOC_199820	ENSRNOT00000042459	0.961944302	5.90E-07
TUCP_004534	XLOC_199820	ENSRNOT00000037203	0.954958058	1.35E-06
TUCP_004534	XLOC_199820	ENSRNOT00000087386	0.957750403	9.87E-07
TUCP_004534	XLOC_199820	ENSRNOT00000000618	0.968951204	2.16E-07
TUCP_004534	XLOC_199820	ENSRNOT00000006591	0.957505491	1.02E-06
TUCP_004534	XLOC_199820	ENSRNOT00000018622	0.964952966	3.93E-07
TUCP_004534	XLOC_199820	ENSRNOT00000087075	0.964309723	4.30E-07

TUCP_004534	XLOC_199820	ENSRNOT00000022126	0.954616358	1.40E-06
TUCP_004534	XLOC_199820	ENSRNOT00000086559	0.9805066	2.15E-08
TUCP_004534	XLOC_199820	ENSRNOT00000008274	0.983700677	8.82E-09
TUCP_004534	XLOC_199820	ENSRNOT00000045041	0.958266871	9.29E-07
TUCP_004534	XLOC_199820	ENSRNOT00000007588	0.953480337	1.59E-06
TUCP_004534	XLOC_199820	ENSRNOT00000077999	-0.957518933	1.01E-06
TUCP_004534	XLOC_199820	ENSRNOT00000001891	0.980353964	2.23E-08
TUCP_004534	XLOC_199820	ENSRNOT00000067626	0.9753059	6.94E-08
TUCP_004534	XLOC_199820	ENSRNOT00000026726	0.961937656	5.90E-07
TUCP_004534	XLOC_199820	ENSRNOT00000056644	0.973933649	9.07E-08
TUCP_004534	XLOC_199820	ENSRNOT00000005686	0.956702591	1.11E-06
TUCP_004534	XLOC_199820	ENSRNOT00000014694	0.956282384	1.17E-06
TUCP_004534	XLOC_199820	ENSRNOT00000017268	0.957836217	9.78E-07
TUCP_004534	XLOC_199820	ENSRNOT00000080418	0.955457842	1.28E-06
TUCP_004534	XLOC_199820	ENSRNOT00000067391	0.96418677	4.37E-07
TUCP_004534	XLOC_199820	ENSRNOT00000090004	0.950600981	2.13E-06
TUCP_004534	XLOC_199820	ENSRNOT00000091368	0.967419017	2.74E-07
TUCP_004534	XLOC_199820	ENSRNOT00000037333	0.971418792	1.43E-07
TUCP_004534	XLOC_199820	ENSRNOT00000029179	0.951864923	1.88E-06
TUCP_004534	XLOC_199820	ENSRNOT00000000469	-0.965084006	3.85E-07
TUCP_004534	XLOC_199820	ENSRNOT00000025506	0.960142329	7.41E-07
TUCP_004534	XLOC_199820	ENSRNOT00000006395	0.955541054	1.27E-06
TUCP_004534	XLOC_199820	ENSRNOT00000007326	0.960744468	6.87E-07
TUCP_004534	XLOC_199820	ENSRNOT00000017813	0.954464351	1.43E-06
TUCP_004534	XLOC_199820	ENSRNOT00000000812	0.968209399	2.42E-07
TUCP_004534	XLOC_199820	ENSRNOT00000072357	0.959599481	7.92E-07
TUCP_004534	XLOC_199820	ENSRNOT00000013067	0.955652662	1.25E-06
TUCP_004534	XLOC_199820	ENSRNOT00000074515	0.969851062	1.86E-07
TUCP_004534	XLOC_199820	ENSRNOT00000082627	0.972091288	1.27E-07
TUCP_004534	XLOC_199820	ENSRNOT00000027145	0.974279258	8.49E-08
TUCP_004534	XLOC_199820	ENSRNOT00000002313	0.956966136	1.08E-06
TUCP_004534	XLOC_199820	ENSRNOT00000071494	0.956500104	1.14E-06
TUCP_004534	XLOC_199820	ENSRNOT00000072458	0.950453846	2.16E-06
TUCP_004534	XLOC_199820	ENSRNOT00000044449	0.973451291	9.93E-08
TUCP_004534	XLOC_199820	ENSRNOT00000035513	0.99063047	5.60E-10
TUCP_004534	XLOC_199820	ENSRNOT00000019531	0.959809332	7.72E-07
TUCP_004534	XLOC_199820	ENSRNOT00000065023	0.968364111	2.37E-07
TUCP_004534	XLOC_199820	ENSRNOT00000071929	0.965115642	3.84E-07
TUCP_004534	XLOC_199820	ENSRNOT00000002462	0.95271743	1.72E-06
TUCP_004534	XLOC_199820	ENSRNOT00000027604	0.966488618	3.15E-07
TUCP_004534	XLOC_199820	ENSRNOT00000063875	0.95058307	2.14E-06
TUCP_004534	XLOC_199820	ENSRNOT00000027609	0.951866082	1.88E-06
TUCP_004534	XLOC_199820	ENSRNOT00000003992	0.955293488	1.30E-06
TUCP_004534	XLOC_199820	ENSRNOT00000012767	-0.96294501	5.17E-07
TUCP_004534	XLOC_199820	ENSRNOT00000038032	0.960505295	7.08E-07
TUCP_004534	XLOC_199820	ENSRNOT00000077706	-0.963997837	4.48E-07
TUCP_004534	XLOC_199820	ENSRNOT00000089980	0.953436032	1.59E-06
TUCP_004534	XLOC_199820	ENSRNOT00000055607	0.952415473	1.77E-06
TUCP_004534	XLOC_199820	ENSRNOT00000089294	0.966058477	3.35E-07
TUCP_004534	XLOC_199820	ENSRNOT00000001631	0.978714943	3.32E-08
TUCP_004534	XLOC_199820	ENSRNOT00000061201	0.952641082	1.73E-06

TUCP_004534	XLOC_199820	ENSRNOT00000078793	-0.968101292	2.47E-07
TUCP_004534	XLOC_199820	ENSRNOT00000033724	0.955709479	1.25E-06
TUCP_004534	XLOC_199820	ENSRNOT00000024924	0.980375321	2.22E-08
TUCP_004534	XLOC_199820	ENSRNOT00000082108	0.97505129	7.30E-08
TUCP_004534	XLOC_199820	ENSRNOT00000018877	0.984827302	6.17E-09
TUCP_004534	XLOC_199820	ENSRNOT00000025104	0.9698523	1.86E-07
TUCP_004534	XLOC_199820	ENSRNOT00000014384	0.964898024	3.96E-07
TUCP_004534	XLOC_199820	ENSRNOT00000031672	0.963622735	4.72E-07
TUCP_004534	XLOC_199820	ENSRNOT00000086456	0.960844504	6.79E-07
TUCP_004534	XLOC_199820	ENSRNOT00000056145	0.954712102	1.39E-06
TUCP_004534	XLOC_199820	ENSRNOT00000030823	0.974974416	7.41E-08
TUCP_004534	XLOC_199820	ENSRNOT00000001227	0.955106527	1.33E-06
TUCP_004534	XLOC_199820	ENSRNOT00000048933	0.950447332	2.16E-06
TUCP_004534	XLOC_199820	ENSRNOT00000012432	0.96221339	5.69E-07
TUCP_004534	XLOC_199820	ENSRNOT00000076388	0.979852015	2.53E-08
TUCP_004534	XLOC_199820	ENSRNOT00000086147	0.975115765	7.21E-08
TUCP_004534	XLOC_199820	ENSRNOT00000064535	0.953781014	1.54E-06
TUCP_004534	XLOC_199820	ENSRNOT00000018341	0.984295195	7.33E-09
TUCP_004534	XLOC_199820	ENSRNOT00000087033	0.973961378	9.02E-08
TUCP_004534	XLOC_199820	ENSRNOT00000082963	0.964173269	4.38E-07
TUCP_004534	XLOC_199820	ENSRNOT00000072632	0.960467187	7.12E-07
TUCP_004534	XLOC_199820	ENSRNOT00000011556	0.952162632	1.82E-06
TUCP_004534	XLOC_199820	ENSRNOT00000079085	0.960195749	7.36E-07
TUCP_004534	XLOC_199820	ENSRNOT00000038811	0.968302727	2.39E-07
TUCP_004534	XLOC_199820	ENSRNOT00000017995	-0.956710481	1.11E-06
TUCP_004534	XLOC_199820	ENSRNOT00000008919	0.961702347	6.08E-07
TUCP_004534	XLOC_199820	ENSRNOT00000021534	0.958850834	8.67E-07
TUCP_004534	XLOC_199820	ENSRNOT00000007917	0.980462657	2.17E-08
TUCP_004534	XLOC_199820	ENSRNOT00000016776	0.953993307	1.50E-06
TUCP_004534	XLOC_199820	ENSRNOT00000034386	0.958532199	9.01E-07
TUCP_004534	XLOC_199820	ENSRNOT00000017955	0.961181302	6.50E-07
TUCP_004534	XLOC_199820	ENSRNOT00000019778	0.970761591	1.60E-07
TUCP_004534	XLOC_199820	ENSRNOT00000048022	0.97009839	1.79E-07
TUCP_004534	XLOC_199820	ENSRNOT00000001597	0.955883927	1.22E-06
TUCP_004534	XLOC_199820	ENSRNOT00000039607	0.950081027	2.24E-06
TUCP_004534	XLOC_199820	ENSRNOT00000082183	-0.953730236	1.55E-06
TUCP_004534	XLOC_199820	ENSRNOT00000072069	0.96932405	2.03E-07
TUCP_004534	XLOC_199820	ENSRNOT00000075400	0.969649463	1.93E-07
TUCP_004534	XLOC_199820	ENSRNOT00000009080	0.963008546	5.13E-07
TUCP_004534	XLOC_199820	ENSRNOT00000023320	0.955235537	1.31E-06
TUCP_004534	XLOC_199820	ENSRNOT00000020453	0.970204187	1.76E-07
TUCP_004534	XLOC_199820	ENSRNOT00000075494	0.95718693	1.05E-06
TUCP_004534	XLOC_199820	ENSRNOT00000009564	0.963944702	4.52E-07
TUCP_004534	XLOC_199820	ENSRNOT00000041237	0.968899284	2.17E-07
TUCP_004534	XLOC_199820	ENSRNOT00000023430	0.964956912	3.92E-07
TUCP_004534	XLOC_199820	ENSRNOT00000001859	0.982242249	1.35E-08
TUCP_004534	XLOC_199820	ENSRNOT00000003733	0.959193325	8.32E-07
TUCP_004534	XLOC_199820	ENSRNOT00000021110	0.976444837	5.49E-08
TUCP_004534	XLOC_199820	ENSRNOT00000083864	0.963489658	4.81E-07
TUCP_004534	XLOC_199820	ENSRNOT00000020853	0.979661347	2.65E-08
TUCP_004534	XLOC_199820	ENSRNOT00000001774	0.981835264	1.51E-08

TUCP_004534	XLOC_199820	ENSRNOT00000080013	0.982871459	1.13E-08
TUCP_004534	XLOC_199820	ENSRNOT00000013759	0.975601066	6.54E-08
TUCP_004534	XLOC_199820	ENSRNOT00000013613	0.973960722	9.03E-08
TUCP_004534	XLOC_199820	ENSRNOT00000010419	-0.973580615	9.70E-08
TUCP_004534	XLOC_199820	ENSRNOT00000027553	0.983781732	8.60E-09
TUCP_004534	XLOC_199820	ENSRNOT00000015476	0.954060728	1.49E-06
TUCP_004534	XLOC_199820	ENSRNOT00000021621	0.959462192	8.05E-07
TUCP_004534	XLOC_199820	ENSRNOT00000022827	0.966226599	3.27E-07
TUCP_004534	XLOC_199820	ENSRNOT00000091808	0.977085632	4.79E-08
TUCP_004534	XLOC_199820	ENSRNOT00000025079	0.953321301	1.61E-06
TUCP_004534	XLOC_199820	ENSRNOT00000077063	0.971550786	1.40E-07
TUCP_004534	XLOC_199820	ENSRNOT00000011432	0.971504591	1.41E-07
TUCP_004534	XLOC_199820	ENSRNOT00000014177	0.970742965	1.61E-07
TUCP_004534	XLOC_199820	ENSRNOT00000007824	0.957388126	1.03E-06
TUCP_004534	XLOC_199820	ENSRNOT00000024677	-0.953367633	1.61E-06
TUCP_004534	XLOC_199820	ENSRNOT00000034983	0.974257132	8.53E-08
TUCP_004534	XLOC_199820	ENSRNOT00000020707	0.957163689	1.06E-06
TUCP_004534	XLOC_199820	ENSRNOT00000021937	0.972441456	1.20E-07
TUCP_004534	XLOC_199820	ENSRNOT00000052098	0.968100847	2.47E-07
TUCP_004534	XLOC_199820	ENSRNOT00000072762	0.950717435	2.11E-06
TUCP_004534	XLOC_199820	ENSRNOT00000014211	0.971084566	1.52E-07
TUCP_004534	XLOC_199820	ENSRNOT00000032893	0.954114055	1.48E-06
TUCP_004534	XLOC_199820	ENSRNOT00000008140	0.955022226	1.34E-06
TUCP_004534	XLOC_199820	ENSRNOT00000056079	0.960600318	7.00E-07
TUCP_004534	XLOC_199820	ENSRNOT00000026724	0.977785977	4.10E-08
TUCP_004534	XLOC_199820	ENSRNOT00000085545	0.955459003	1.28E-06
TUCP_004534	XLOC_199820	ENSRNOT00000018028	0.95461096	1.41E-06
TUCP_004534	XLOC_199820	ENSRNOT00000015351	0.963894748	4.55E-07
TUCP_004534	XLOC_199820	ENSRNOT00000004351	0.952902272	1.69E-06
TUCP_004534	XLOC_199820	ENSRNOT00000014502	0.961327076	6.38E-07
TUCP_004534	XLOC_199820	ENSRNOT00000003787	0.959904914	7.63E-07
TUCP_004534	XLOC_199820	ENSRNOT00000005608	0.951191169	2.01E-06
TUCP_004534	XLOC_199820	ENSRNOT00000022343	0.9515282	1.94E-06
TUCP_004534	XLOC_199820	ENSRNOT00000072165	0.971236795	1.48E-07
TUCP_004534	XLOC_199820	ENSRNOT00000008317	0.969034721	2.13E-07
TUCP_004534	XLOC_199820	ENSRNOT00000091541	0.970333313	1.72E-07
TUCP_004534	XLOC_199820	ENSRNOT00000068026	0.96709576	2.87E-07
TUCP_004534	XLOC_199820	ENSRNOT00000056923	0.975530415	6.63E-08
TUCP_004534	XLOC_199820	ENSRNOT00000005996	0.959607401	7.91E-07
TUCP_004534	XLOC_199820	ENSRNOT00000008889	0.967958341	2.52E-07
TUCP_004534	XLOC_199820	ENSRNOT00000017805	-0.966262576	3.25E-07
TUCP_004534	XLOC_199820	ENSRNOT00000075057	0.966282127	3.24E-07
TUCP_004534	XLOC_199820	ENSRNOT00000016699	0.955217294	1.32E-06
TUCP_004534	XLOC_199820	ENSRNOT00000017746	0.975438776	6.76E-08
TUCP_004534	XLOC_199820	ENSRNOT00000000552	0.954087147	1.49E-06
TUCP_004534	XLOC_199820	ENSRNOT00000016286	0.957631429	1.00E-06
TUCP_004534	XLOC_199820	ENSRNOT00000050696	-0.95897127	8.55E-07
TUCP_004534	XLOC_199820	ENSRNOT00000037946	0.966924259	2.95E-07
TUCP_004534	XLOC_199820	ENSRNOT00000070825	0.960416196	7.16E-07
TUCP_004534	XLOC_199820	ENSRNOT00000038677	0.955109588	1.33E-06
TUCP_004534	XLOC_199820	ENSRNOT00000024753	0.975789928	6.29E-08

TUCP_004534	XLOC_199820	ENSRNOT00000070916	0.965110606	3.84E-07
TUCP_004534	XLOC_199820	ENSRNOT00000024452	0.958984883	8.53E-07
TUCP_004534	XLOC_199820	ENSRNOT00000024326	0.955150425	1.33E-06
TUCP_004534	XLOC_199820	ENSRNOT00000024456	0.967871461	2.55E-07
TUCP_004534	XLOC_199820	ENSRNOT00000000253	0.961227606	6.47E-07
TUCP_004534	XLOC_199820	ENSRNOT00000000258	0.969365043	2.02E-07
TUCP_004534	XLOC_199820	ENSRNOT00000067524	-0.9547643	1.38E-06
TUCP_004534	XLOC_199820	ENSRNOT00000002511	0.981327085	1.73E-08
TUCP_004534	XLOC_199820	ENSRNOT00000012017	0.954671578	1.40E-06
TUCP_004534	XLOC_199820	ENSRNOT00000020417	0.95232717	1.79E-06
TUCP_004534	XLOC_199820	ENSRNOT00000021677	0.960490256	7.10E-07
TUCP_004534	XLOC_199820	ENSRNOT00000006804	-0.955970741	1.21E-06
TUCP_004534	XLOC_199820	ENSRNOT00000020809	0.972187322	1.25E-07
TUCP_004534	XLOC_199820	ENSRNOT00000001605	0.977944622	3.96E-08
TUCP_004534	XLOC_199820	ENSRNOT00000003359	0.951723978	1.90E-06
TUCP_004534	XLOC_199820	ENSRNOT00000038511	0.959200927	8.31E-07
TUCP_004534	XLOC_199820	ENSRNOT00000019044	0.951804225	1.89E-06
TUCP_004534	XLOC_199820	ENSRNOT00000020982	0.961621544	6.15E-07
TUCP_004534	XLOC_199820	ENSRNOT00000086847	0.954992875	1.35E-06
TUCP_004534	XLOC_199820	ENSRNOT00000018846	0.96477857	4.02E-07
TUCP_004534	XLOC_199820	ENSRNOT00000025086	0.963852073	4.57E-07
TUCP_004534	XLOC_199820	ENSRNOT00000028876	0.959858881	7.67E-07
TUCP_004534	XLOC_199820	ENSRNOT00000079784	0.977023345	4.85E-08
TUCP_004534	XLOC_199820	ENSRNOT00000013553	-0.951469997	1.95E-06
TUCP_004534	XLOC_199820	ENSRNOT00000011694	0.968772028	2.22E-07
TUCP_004534	XLOC_199820	ENSRNOT00000002851	0.959740213	7.78E-07
TUCP_004534	XLOC_199820	ENSRNOT00000068184	0.976885302	5.00E-08
TUCP_004534	XLOC_199820	ENSRNOT00000031692	0.975594731	6.55E-08
TUCP_004534	XLOC_199820	ENSRNOT00000089016	0.984566572	6.72E-09
TUCP_004534	XLOC_199820	ENSRNOT00000022864	0.962874467	5.22E-07
TUCP_004534	XLOC_199820	ENSRNOT00000006578	0.967047399	2.90E-07
TUCP_004534	XLOC_199820	ENSRNOT00000028900	0.967016449	2.91E-07
TUCP_004534	XLOC_199820	ENSRNOT00000072890	0.950309593	2.19E-06
TUCP_004534	XLOC_199820	ENSRNOT00000087556	0.980894825	1.94E-08
TUCP_004534	XLOC_199820	ENSRNOT00000029753	0.961481669	6.26E-07
TUCP_004534	XLOC_199820	ENSRNOT00000040981	0.967270502	2.80E-07
TUCP_004534	XLOC_199820	ENSRNOT00000056725	0.950206473	2.22E-06
TUCP_004534	XLOC_199820	ENSRNOT00000010630	-0.950237118	2.21E-06
TUCP_004534	XLOC_199820	ENSRNOT00000038464	0.96331294	4.92E-07
TUCP_004534	XLOC_199820	ENSRNOT00000008359	0.965532238	3.62E-07
TUCP_004534	XLOC_199820	ENSRNOT00000039291	0.964486843	4.19E-07
TUCP_004534	XLOC_199820	ENSRNOT00000035486	0.965806893	3.48E-07
TUCP_004534	XLOC_199820	ENSRNOT00000080174	0.965723661	3.52E-07
TUCP_004534	XLOC_199820	ENSRNOT00000011237	0.95985888	7.67E-07
TUCP_004534	XLOC_199820	ENSRNOT00000013341	0.956497251	1.14E-06
TUCP_004534	XLOC_199820	ENSRNOT00000013344	0.957977697	9.62E-07
TUCP_004534	XLOC_199820	ENSRNOT00000015759	0.962567825	5.43E-07
TUCP_004534	XLOC_199820	ENSRNOT00000030501	0.970900785	1.56E-07
TUCP_004534	XLOC_199820	ENSRNOT00000017204	0.973683369	9.51E-08
TUCP_004534	XLOC_199820	ENSRNOT00000008772	0.95360947	1.57E-06
TUCP_004534	XLOC_199820	ENSRNOT00000005658	0.957389449	1.03E-06

TUCP_004534	XLOC_199820	ENSRNOT00000009275	0.96713294	2.86E-07
TUCP_004534	XLOC_199820	ENSRNOT00000022086	0.964709628	4.06E-07
TUCP_004534	XLOC_199820	ENSRNOT00000024714	0.967131183	2.86E-07
TUCP_004534	XLOC_199820	ENSRNOT00000024712	0.956241171	1.17E-06
TUCP_004534	XLOC_199820	ENSRNOT00000017384	-0.956970295	1.08E-06
TUCP_004534	XLOC_199820	ENSRNOT00000081787	0.960460048	7.12E-07
TUCP_004534	XLOC_199820	ENSRNOT00000000203	0.965737047	3.51E-07
TUCP_004534	XLOC_199820	ENSRNOT00000002799	0.961942466	5.90E-07
TUCP_004534	XLOC_199820	ENSRNOT00000054685	-0.950214769	2.21E-06
TUCP_004534	XLOC_199820	ENSRNOT00000024882	0.964147585	4.39E-07
TUCP_004534	XLOC_199820	ENSRNOT00000023456	0.971820096	1.33E-07
TUCP_004534	XLOC_199820	ENSRNOT00000016012	0.975510155	6.66E-08
TUCP_004534	XLOC_199820	ENSRNOT00000002334	0.950481816	2.16E-06
TUCP_004534	XLOC_199820	ENSRNOT00000013587	0.964957169	3.92E-07
TUCP_004534	XLOC_199820	ENSRNOT00000089545	0.954526958	1.42E-06
TUCP_004534	XLOC_199820	ENSRNOT00000003728	0.963946916	4.52E-07
TUCP_004534	XLOC_199820	ENSRNOT00000019040	0.968943102	2.16E-07
TUCP_004534	XLOC_199820	ENSRNOT00000035400	0.977204951	4.67E-08
TUCP_004534	XLOC_199820	ENSRNOT00000015460	0.959491864	8.02E-07
TUCP_004534	XLOC_199820	ENSRNOT00000003667	0.970407765	1.70E-07
TUCP_004534	XLOC_199820	ENSRNOT00000077727	0.963398552	4.86E-07
TUCP_004534	XLOC_199820	ENSRNOT00000082124	0.969961057	1.83E-07
TUCP_004534	XLOC_199820	ENSRNOT00000025036	0.985534573	4.87E-09
TUCP_004534	XLOC_199820	ENSRNOT00000020197	0.960069091	7.48E-07
TUCP_004534	XLOC_199820	ENSRNOT00000070886	0.975221495	7.06E-08
TUCP_004534	XLOC_199820	ENSRNOT00000014044	0.962628332	5.39E-07
TUCP_004534	XLOC_199820	ENSRNOT00000090156	0.952015603	1.85E-06
TUCP_004534	XLOC_199820	ENSRNOT00000066521	0.963674651	4.69E-07
TUCP_004534	XLOC_199820	ENSRNOT00000003544	0.95533585	1.30E-06
TUCP_004534	XLOC_199820	ENSRNOT00000006306	0.958972484	8.54E-07
TUCP_004534	XLOC_199820	ENSRNOT00000004196	-0.974157946	8.69E-08
TUCP_004534	XLOC_199820	ENSRNOT00000007882	0.97239193	1.21E-07
TUCP_004534	XLOC_199820	ENSRNOT00000006946	0.973620539	9.62E-08
TUCP_004534	XLOC_199820	ENSRNOT00000086576	0.97193235	1.31E-07
TUCP_004534	XLOC_199820	ENSRNOT00000089611	-0.952763186	1.71E-06
TUCP_004534	XLOC_199820	ENSRNOT00000039012	0.968762144	2.22E-07
TUCP_004534	XLOC_199820	ENSRNOT00000023854	0.958906927	8.61E-07
TUCP_004534	XLOC_199820	ENSRNOT00000008253	0.952022009	1.85E-06
TUCP_004534	XLOC_199820	ENSRNOT00000082894	0.959717215	7.81E-07
TUCP_004534	XLOC_199820	ENSRNOT00000025824	0.95411626	1.48E-06
TUCP_004534	XLOC_199820	ENSRNOT00000039593	0.951680971	1.91E-06
TUCP_004534	XLOC_199820	ENSRNOT00000022676	0.958596605	8.94E-07
TUCP_004534	XLOC_199820	ENSRNOT00000083983	0.96982548	1.87E-07
TUCP_004534	XLOC_199820	ENSRNOT00000007549	0.970848192	1.58E-07
TUCP_004534	XLOC_199820	ENSRNOT00000017626	0.967112579	2.87E-07
TUCP_004534	XLOC_199820	ENSRNOT00000076485	0.958097977	9.48E-07
TUCP_004534	XLOC_199820	ENSRNOT00000022774	0.972316214	1.22E-07
TUCP_004534	XLOC_199820	ENSRNOT00000025134	0.977300347	4.57E-08
TUCP_004534	XLOC_199820	ENSRNOT00000087359	-0.963536945	4.77E-07
TUCP_004534	XLOC_199820	ENSRNOT00000014586	0.970866124	1.57E-07
TUCP_004534	XLOC_199820	ENSRNOT00000072580	0.965796782	3.48E-07

TUCP_004534	XLOC_199820	ENSRNOT00000014832	0.959697857	7.83E-07
TUCP_004534	XLOC_199820	ENSRNOT00000089879	0.963156001	5.03E-07
TUCP_004534	XLOC_199820	ENSRNOT00000018473	0.966336976	3.22E-07
TUCP_004534	XLOC_199820	ENSRNOT00000026118	0.972796466	1.12E-07
TUCP_004534	XLOC_199820	ENSRNOT00000037955	0.953562216	1.57E-06
TUCP_004534	XLOC_199820	ENSRNOT00000017765	-0.952216884	1.81E-06
TUCP_004534	XLOC_199820	ENSRNOT00000017339	0.97519412	7.10E-08
TUCP_004534	XLOC_199820	ENSRNOT00000088752	0.95411323	1.48E-06
TUCP_004534	XLOC_199820	ENSRNOT00000050617	0.950703702	2.11E-06
TUCP_004534	XLOC_199820	ENSRNOT00000015297	0.95243368	1.77E-06
TUCP_004534	XLOC_199820	ENSRNOT00000050227	0.968439225	2.34E-07
TUCP_004534	XLOC_199820	ENSRNOT00000093552	0.968965758	2.15E-07
TUCP_004534	XLOC_199820	ENSRNOT00000025791	0.960160993	7.39E-07
TUCP_004534	XLOC_199820	ENSRNOT00000088884	0.956523831	1.14E-06
TUCP_004534	XLOC_199820	ENSRNOT00000075544	0.964515477	4.17E-07
TUCP_004534	XLOC_199820	ENSRNOT00000009994	0.956366126	1.16E-06
TUCP_004534	XLOC_199820	ENSRNOT00000077367	0.956072419	1.20E-06
TUCP_004534	XLOC_199820	ENSRNOT00000038994	0.961131429	6.54E-07
TUCP_004534	XLOC_199820	ENSRNOT00000024841	0.965501392	3.63E-07
TUCP_004534	XLOC_199820	ENSRNOT00000020409	0.953339363	1.61E-06
TUCP_004534	XLOC_199820	ENSRNOT00000009345	0.97536019	6.86E-08
TUCP_004534	XLOC_199820	ENSRNOT00000009468	0.965799629	3.48E-07
TUCP_004534	XLOC_199820	ENSRNOT00000048529	0.975281816	6.97E-08
TUCP_004534	XLOC_199820	ENSRNOT00000000124	0.96412265	4.41E-07
TUCP_004534	XLOC_199820	ENSRNOT00000043581	0.976181555	5.80E-08
TUCP_004534	XLOC_199820	ENSRNOT00000064636	0.959808834	7.72E-07
TUCP_004534	XLOC_199820	ENSRNOT00000078906	0.950823356	2.09E-06
TUCP_004534	XLOC_199820	ENSRNOT00000013733	0.973581836	9.69E-08
TUCP_004534	XLOC_199820	ENSRNOT00000066561	0.9778417	4.05E-08
TUCP_004534	XLOC_199820	ENSRNOT00000004094	0.954702328	1.39E-06
TUCP_004534	XLOC_199820	ENSRNOT00000006266	0.97286918	1.11E-07
TUCP_004534	XLOC_199820	ENSRNOT00000020909	0.959696113	7.83E-07
TUCP_004534	XLOC_199820	ENSRNOT00000016376	0.969594763	1.94E-07
TUCP_004534	XLOC_199820	ENSRNOT00000011511	0.973393275	1.00E-07
TUCP_004534	XLOC_199820	ENSRNOT00000065722	0.966717	3.04E-07
TUCP_004534	XLOC_199820	ENSRNOT00000065642	0.973501643	9.84E-08
TUCP_004534	XLOC_199820	ENSRNOT00000036195	0.970574326	1.65E-07
TUCP_004534	XLOC_199820	ENSRNOT00000009763	0.955438263	1.28E-06
TUCP_004534	XLOC_199820	ENSRNOT00000031291	0.962456043	5.52E-07
TUCP_004534	XLOC_199820	ENSRNOT00000074586	0.964096025	4.42E-07
TUCP_004534	XLOC_199820	ENSRNOT00000057758	0.974845355	7.60E-08
TUCP_004534	XLOC_199820	ENSRNOT00000022637	0.961339989	6.37E-07
TUCP_004534	XLOC_199820	ENSRNOT00000004241	0.950176563	2.22E-06
TUCP_004534	XLOC_199820	ENSRNOT00000003087	-0.951384812	1.97E-06
TUCP_004534	XLOC_199820	ENSRNOT00000083666	0.958682871	8.85E-07
TUCP_004534	XLOC_199820	ENSRNOT00000019358	0.978423748	3.55E-08
TUCP_004534	XLOC_199820	ENSRNOT00000087011	0.978924199	3.16E-08
TUCP_004534	XLOC_199820	ENSRNOT00000084567	-0.95691167	1.09E-06
TUCP_004534	XLOC_199820	ENSRNOT00000005286	0.975597685	6.54E-08
TUCP_004534	XLOC_199820	ENSRNOT00000056675	0.962129937	5.76E-07
TUCP_004534	XLOC_199820	ENSRNOT00000013295	0.983928933	8.22E-09

TUCP_004534	XLOC_199820	ENSRNOT00000066947	0.951542369	1.94E-06
TUCP_004534	XLOC_199820	ENSRNOT00000021455	0.959709917	7.81E-07
TUCP_004534	XLOC_199820	ENSRNOT00000039177	0.962636681	5.39E-07
TUCP_004534	XLOC_199820	ENSRNOT00000076191	0.960945278	6.70E-07
TUCP_004534	XLOC_199820	ENSRNOT00000013228	-0.956903556	1.09E-06
TUCP_004534	XLOC_199820	ENSRNOT00000017784	0.978874792	3.20E-08
TUCP_004534	XLOC_199820	ENSRNOT00000018405	0.973712887	9.46E-08
TUCP_004534	XLOC_199820	ENSRNOT00000021458	0.958700568	8.83E-07
TUCP_004534	XLOC_199820	ENSRNOT00000026496	0.95410913	1.48E-06
TUCP_004534	XLOC_199820	ENSRNOT00000082739	0.95889884	8.62E-07
TUCP_004534	XLOC_199820	ENSRNOT00000026552	0.957573713	1.01E-06
TUCP_004534	XLOC_199820	ENSRNOT00000065045	-0.963394157	4.87E-07
TUCP_004534	XLOC_199820	ENSRNOT00000023463	0.985047451	5.74E-09
TUCP_004534	XLOC_199820	ENSRNOT00000034266	0.972041685	1.28E-07
TUCP_004534	XLOC_199820	ENSRNOT00000028781	-0.960909371	6.73E-07
TUCP_004534	XLOC_199820	ENSRNOT00000046490	0.984734684	6.36E-09
TUCP_004534	XLOC_199820	ENSRNOT00000001973	0.967164192	2.84E-07
TUCP_004534	XLOC_199820	ENSRNOT00000046552	-0.960011499	7.53E-07
TUCP_004534	XLOC_199820	ENSRNOT00000024775	0.950396349	2.18E-06
TUCP_004534	XLOC_199820	ENSRNOT00000024801	0.982539846	1.24E-08
TUCP_004534	XLOC_199820	ENSRNOT00000009544	0.951201249	2.01E-06
TUCP_004534	XLOC_199820	ENSRNOT00000054957	0.965488729	3.64E-07
TUCP_004534	XLOC_199820	ENSRNOT00000079011	0.962630408	5.39E-07
TUCP_004534	XLOC_199820	ENSRNOT00000041808	0.966942036	2.94E-07
TUCP_004534	XLOC_199820	ENSRNOT00000021134	0.973357272	1.01E-07
TUCP_004534	XLOC_199820	ENSRNOT00000013571	0.959342376	8.17E-07
TUCP_004534	XLOC_199820	ENSRNOT00000014002	0.959698803	7.82E-07
TUCP_004534	XLOC_199820	ENSRNOT00000034669	0.961758976	6.04E-07
TUCP_004534	XLOC_199820	ENSRNOT00000077784	0.954727725	1.39E-06
TUCP_004534	XLOC_199820	ENSRNOT00000011898	0.960588379	7.01E-07
TUCP_004534	XLOC_199820	ENSRNOT00000011952	0.958253183	9.31E-07
TUCP_004534	XLOC_199820	ENSRNOT00000016324	0.967589278	2.67E-07
TUCP_004534	XLOC_199820	ENSRNOT00000006824	0.96619891	3.28E-07
TUCP_004534	XLOC_199820	ENSRNOT00000012775	0.963654606	4.70E-07
TUCP_004534	XLOC_199820	ENSRNOT00000077040	0.97752254	4.35E-08
TUCP_004534	XLOC_199820	ENSRNOT00000086260	0.953534774	1.58E-06
TUCP_004534	XLOC_199820	ENSRNOT00000066793	0.956707419	1.11E-06
TUCP_004534	XLOC_199820	ENSRNOT00000001153	-0.953703388	1.55E-06
TUCP_004534	XLOC_199820	ENSRNOT00000025517	-0.95210267	1.83E-06
TUCP_004534	XLOC_199820	ENSRNOT00000073193	0.960165732	7.39E-07
TUCP_004534	XLOC_199820	ENSRNOT00000020316	0.965133286	3.83E-07
TUCP_004534	XLOC_199820	ENSRNOT00000006709	0.972120995	1.27E-07
TUCP_004534	XLOC_199820	ENSRNOT00000029704	0.967168741	2.84E-07
TUCP_004534	XLOC_199820	ENSRNOT00000012495	0.955970676	1.21E-06
TUCP_004534	XLOC_199820	ENSRNOT00000019816	0.957539429	1.01E-06
TUCP_004534	XLOC_199820	ENSRNOT00000025221	0.981480794	1.66E-08
TUCP_004534	XLOC_199820	ENSRNOT00000025634	0.967610509	2.66E-07
TUCP_004534	XLOC_199820	ENSRNOT00000026909	0.951276806	1.99E-06
TUCP_004534	XLOC_199820	ENSRNOT00000008244	-0.950877354	2.07E-06
TUCP_004534	XLOC_199820	ENSRNOT00000066120	0.964764129	4.03E-07
TUCP_004534	XLOC_199820	ENSRNOT00000077730	0.977384367	4.49E-08

TUCP_004534	XLOC_199820	ENSRNOT00000000717	0.970958427	1.55E-07
TUCP_004534	XLOC_199820	ENSRNOT00000049048	0.978677419	3.35E-08
TUCP_004534	XLOC_199820	ENSRNOT00000004370	0.956782996	1.10E-06
TUCP_004534	XLOC_199820	ENSRNOT00000014235	-0.952045571	1.84E-06
TUCP_004534	XLOC_199820	ENSRNOT00000030222	0.980939808	1.92E-08
TUCP_004534	XLOC_199820	ENSRNOT00000089066	-0.954702162	1.39E-06
TUCP_004534	XLOC_199820	ENSRNOT00000072722	0.959338726	8.18E-07
TUCP_004534	XLOC_199820	ENSRNOT00000080216	0.982162009	1.38E-08
TUCP_004534	XLOC_199820	ENSRNOT00000003551	0.963009442	5.13E-07
TUCP_004534	XLOC_199820	ENSRNOT00000027187	0.954977907	1.35E-06
TUCP_004534	XLOC_199820	ENSRNOT00000005155	-0.973050427	1.07E-07
TUCP_004534	XLOC_199820	ENSRNOT00000023543	0.966732365	3.03E-07
TUCP_004534	XLOC_199820	ENSRNOT00000023014	-0.954232564	1.46E-06
TUCP_004534	XLOC_199820	ENSRNOT00000028737	0.95720356	1.05E-06
TUCP_004534	XLOC_199820	ENSRNOT00000018634	0.950551147	2.14E-06
TUCP_004534	XLOC_199820	ENSRNOT00000017618	0.957744679	9.88E-07
TUCP_004534	XLOC_199820	ENSRNOT00000085458	0.978424701	3.55E-08
TUCP_004534	XLOC_199820	ENSRNOT00000039572	0.96655726	3.11E-07
TUCP_004534	XLOC_199820	ENSRNOT00000038652	0.957506581	1.02E-06
TUCP_004534	XLOC_199820	ENSRNOT00000013535	0.981366718	1.71E-08
TUCP_004534	XLOC_199820	ENSRNOT00000066199	0.961658178	6.12E-07
TUCP_004534	XLOC_199820	ENSRNOT00000026767	-0.960632293	6.97E-07
TUCP_004534	XLOC_199820	ENSRNOT00000047698	0.950733216	2.10E-06
TUCP_004534	XLOC_199820	ENSRNOT00000075630	0.973958643	9.03E-08
TUCP_004534	XLOC_199820	ENSRNOT00000024938	0.977568953	4.31E-08
TUCP_004534	XLOC_199820	ENSRNOT00000081678	-0.952740023	1.71E-06
TUCP_004534	XLOC_199820	ENSRNOT00000046639	-0.959904768	7.63E-07
TUCP_004534	XLOC_199820	ENSRNOT00000011320	0.978809513	3.25E-08
TUCP_003963	XLOC_174241	ENSRNOT00000000109	0.965194097	3.79E-07
TUCP_003963	XLOC_174241	ENSRNOT00000054858	0.950193055	2.22E-06
TUCP_004240	XLOC_186273	ENSRNOT00000039045	0.964368475	4.26E-07
TUCP_004240	XLOC_186273	ENSRNOT00000076052	0.950964045	2.06E-06
TUCP_004240	XLOC_186273	ENSRNOT00000024281	0.971411288	1.43E-07
TUCP_004240	XLOC_186273	ENSRNOT00000010588	0.955716597	1.24E-06
TUCP_004240	XLOC_186273	ENSRNOT00000030991	0.953572215	1.57E-06
TUCP_003961	XLOC_173614	ENSRNOT00000061493	-0.954450722	1.43E-06
TUCP_001865	XLOC_077400	ENSRNOT00000091652	0.987801248	2.08E-09
TUCP_001865	XLOC_077400	ENSRNOT00000036564	0.991033026	4.50E-10
TUCP_001865	XLOC_077400	ENSRNOT00000000109	0.950148452	2.23E-06
TUCP_001865	XLOC_077400	ENSRNOT00000080865	0.99123585	4.01E-10
TUCP_001865	XLOC_077400	ENSRNOT00000073534	0.983828155	8.48E-09
TUCP_001865	XLOC_077400	ENSRNOT00000012161	0.982865915	1.13E-08
TUCP_001865	XLOC_077400	ENSRNOT00000087758	0.972055179	1.28E-07
TUCP_001865	XLOC_077400	ENSRNOT00000049832	0.967430745	2.73E-07
TUCP_001865	XLOC_077400	ENSRNOT00000093019	0.99123585	4.01E-10
TUCP_001865	XLOC_077400	ENSRNOT00000024825	0.964917902	3.95E-07
TUCP_001865	XLOC_077400	ENSRNOT00000072734	0.951480411	1.95E-06
TUCP_001865	XLOC_077400	ENSRNOT00000085908	0.985038962	5.76E-09
TUCP_001865	XLOC_077400	ENSRNOT00000046744	0.99123585	4.01E-10
TUCP_001865	XLOC_077400	ENSRNOT00000076429	0.99123585	4.01E-10
TUCP_001865	XLOC_077400	ENSRNOT00000084667	0.99252912	1.81E-10

TUCP_001865	XLOC_077400	ENSRNOT00000055412	0.99123585	4.01E-10
TUCP_001865	XLOC_077400	ENSRNOT00000093201	0.990370949	6.41E-10
TUCP_001865	XLOC_077400	ENSRNOT00000072724	0.99123585	4.01E-10
TUCP_001865	XLOC_077400	ENSRNOT00000066017	0.97696124	4.92E-08
TUCP_001865	XLOC_077400	ENSRNOT00000082760	0.975469777	6.71E-08
TUCP_001865	XLOC_077400	ENSRNOT00000073148	0.972084157	1.27E-07
TUCP_001865	XLOC_077400	ENSRNOT00000054858	0.974331941	8.41E-08
TUCP_001865	XLOC_077400	ENSRNOT00000084375	0.99123585	4.01E-10
TUCP_001865	XLOC_077400	ENSRNOT00000006249	0.952858674	1.69E-06
TUCP_001865	XLOC_077400	ENSRNOT00000042369	0.984666827	6.51E-09
TUCP_001865	XLOC_077400	ENSRNOT00000059326	0.952656293	1.73E-06
TUCP_001865	XLOC_077400	ENSRNOT00000086554	0.99123585	4.01E-10
TUCP_001865	XLOC_077400	ENSRNOT00000047921	0.99123585	4.01E-10
TUCP_001865	XLOC_077400	ENSRNOT00000064261	0.991033514	4.50E-10
TUCP_001865	XLOC_077400	ENSRNOT00000086660	0.988879228	1.31E-09
TUCP_001865	XLOC_077400	ENSRNOT00000083977	0.99123585	4.01E-10
TUCP_001865	XLOC_077400	ENSRNOT00000092933	0.99123585	4.01E-10
TUCP_001865	XLOC_077400	ENSRNOT00000085447	0.992277476	2.14E-10
TUCP_001865	XLOC_077400	ENSRNOT00000041281	0.974925171	7.49E-08
TUCP_003219	XLOC_138410	ENSRNOT00000020297	0.963430388	4.84E-07
TUCP_003219	XLOC_138410	ENSRNOT00000006188	-0.955167486	1.32E-06
TUCP_003219	XLOC_138410	ENSRNOT00000090621	0.965309056	3.73E-07
TUCP_003219	XLOC_138410	ENSRNOT00000016088	0.951387974	1.97E-06
TUCP_003219	XLOC_138410	ENSRNOT00000090946	0.95892649	8.59E-07
TUCP_003219	XLOC_138410	ENSRNOT00000075397	0.958319055	9.24E-07
TUCP_003219	XLOC_138410	ENSRNOT00000008253	-0.961679211	6.10E-07
TUCP_003219	XLOC_138410	ENSRNOT00000085954	0.951815244	1.89E-06
TUCP_003219	XLOC_138410	ENSRNOT00000032926	-0.970757611	1.60E-07
TUCP_003219	XLOC_138410	ENSRNOT00000009080	-0.958294009	9.26E-07
TUCP_003219	XLOC_138410	ENSRNOT00000038780	0.96559346	3.58E-07
TUCP_003219	XLOC_138410	ENSRNOT00000063930	-0.96697488	2.93E-07
TUCP_003219	XLOC_138410	ENSRNOT00000000181	0.952281286	1.80E-06
TUCP_003219	XLOC_138410	ENSRNOT00000003087	0.951908213	1.87E-06
TUCP_003219	XLOC_138410	ENSRNOT00000068353	0.957077188	1.07E-06
TUCP_003219	XLOC_138410	ENSRNOT00000049349	0.95863266	8.90E-07
TUCP_003219	XLOC_138410	ENSRNOT00000013025	-0.952903759	1.69E-06
TUCP_003219	XLOC_138410	ENSRNOT00000089319	0.958675651	8.85E-07
TUCP_003219	XLOC_138410	ENSRNOT00000020910	0.958033795	9.55E-07
TUCP_003219	XLOC_138410	ENSRNOT00000087011	-0.950200828	2.22E-06
TUCP_003219	XLOC_138410	ENSRNOT00000022086	-0.955619432	1.26E-06
TUCP_003219	XLOC_138410	ENSRNOT00000057422	-0.961660566	6.12E-07
TUCP_003219	XLOC_138410	ENSRNOT00000036051	0.952672901	1.73E-06
TUCP_003219	XLOC_138410	ENSRNOT00000064776	-0.957060061	1.07E-06
TUCP_003219	XLOC_138410	ENSRNOT00000004391	0.969933128	1.84E-07
TUCP_003219	XLOC_138410	ENSRNOT00000074023	0.970049257	1.81E-07
TUCP_003219	XLOC_138410	ENSRNOT00000031692	-0.96038175	7.19E-07
TUCP_003219	XLOC_138410	ENSRNOT00000019180	-0.97643064	5.51E-08
TUCP_003219	XLOC_138410	ENSRNOT00000024710	-0.953535967	1.58E-06
TUCP_003219	XLOC_138410	ENSRNOT00000042459	-0.954318756	1.45E-06
TUCP_003219	XLOC_138410	ENSRNOT00000072069	-0.952310344	1.79E-06
TUCP_003219	XLOC_138410	ENSRNOT00000013759	-0.953021362	1.67E-06

TUCP_003219	XLOC_138410	ENSRNOT00000018341	-0.95613729	1.19E-06
TUCP_003219	XLOC_138410	ENSRNOT00000010419	0.960402451	7.17E-07
TUCP_003219	XLOC_138410	ENSRNOT00000021534	-0.950708149	2.11E-06
TUCP_003219	XLOC_138410	ENSRNOT00000054685	0.961387108	6.34E-07
TUCP_003219	XLOC_138410	ENSRNOT00000022746	-0.981598915	1.61E-08
TUCP_003219	XLOC_138410	ENSRNOT00000016306	-0.959211225	8.30E-07
TUCP_003219	XLOC_138410	ENSRNOT00000087604	0.96103844	6.62E-07
TUCP_003219	XLOC_138410	ENSRNOT00000079742	0.9541011	1.49E-06
TUCP_003219	XLOC_138410	ENSRNOT00000004722	-0.951154785	2.02E-06
TUCP_003219	XLOC_138410	ENSRNOT00000065978	0.957747906	9.88E-07
TUCP_003219	XLOC_138410	ENSRNOT00000075480	-0.95955175	7.97E-07
TUCP_003219	XLOC_138410	ENSRNOT00000005620	-0.968412944	2.35E-07
TUCP_003219	XLOC_138410	ENSRNOT00000020051	0.953772278	1.54E-06
TUCP_003219	XLOC_138410	ENSRNOT00000064643	0.959349242	8.16E-07
TUCP_003219	XLOC_138410	ENSRNOT00000027760	0.975720611	6.38E-08
TUCP_003219	XLOC_138410	ENSRNOT00000020681	-0.952925806	1.68E-06
TUCP_003219	XLOC_138410	ENSRNOT00000042903	0.963862934	4.57E-07
TUCP_003219	XLOC_138410	ENSRNOT00000089933	0.954540495	1.42E-06
TUCP_003219	XLOC_138410	ENSRNOT00000056100	-0.951343789	1.98E-06
TUCP_003219	XLOC_138410	ENSRNOT00000000477	-0.954055667	1.49E-06
TUCP_003219	XLOC_138410	ENSRNOT00000060002	-0.95944616	8.07E-07
TUCP_003219	XLOC_138410	ENSRNOT00000081226	0.95578734	1.24E-06
TUCP_003219	XLOC_138410	ENSRNOT00000017648	-0.951370709	1.97E-06
TUCP_003219	XLOC_138410	ENSRNOT00000065045	0.951994711	1.85E-06
TUCP_003219	XLOC_138410	ENSRNOT00000030798	-0.956045261	1.20E-06
TUCP_003219	XLOC_138410	ENSRNOT00000007369	0.952283517	1.80E-06
TUCP_003219	XLOC_138410	ENSRNOT00000018634	-0.975333302	6.90E-08
TUCP_003219	XLOC_138410	ENSRNOT00000000383	-0.959036277	8.48E-07
TUCP_003219	XLOC_138410	ENSRNOT00000031701	-0.962473943	5.50E-07
TUCP_003219	XLOC_138410	ENSRNOT00000072849	-0.954502148	1.42E-06
TUCP_003219	XLOC_138410	ENSRNOT00000073643	-0.953918037	1.51E-06
TUCP_003219	XLOC_138410	ENSRNOT00000078349	-0.953529629	1.58E-06
TUCP_003219	XLOC_138410	ENSRNOT00000003940	-0.955879993	1.22E-06
TUCP_003219	XLOC_138410	ENSRNOT00000024947	-0.97381933	9.27E-08
TUCP_003219	XLOC_138410	ENSRNOT00000046552	0.955476084	1.28E-06
TUCP_003219	XLOC_138410	ENSRNOT00000077727	-0.95294643	1.68E-06
TUCP_003219	XLOC_138410	ENSRNOT00000011930	0.960621059	6.98E-07
TUCP_003219	XLOC_138410	ENSRNOT00000067391	-0.955505357	1.27E-06
TUCP_003219	XLOC_138410	ENSRNOT00000037333	-0.953516318	1.58E-06
TUCP_003219	XLOC_138410	ENSRNOT00000029179	-0.961452012	6.28E-07
TUCP_003219	XLOC_138410	ENSRNOT00000090156	-0.958437988	9.11E-07
TUCP_003219	XLOC_138410	ENSRNOT00000085545	-0.962479911	5.50E-07
TUCP_003219	XLOC_138410	ENSRNOT00000019302	-0.967513255	2.70E-07
TUCP_003219	XLOC_138410	ENSRNOT00000025856	-0.953357092	1.61E-06
TUCP_003219	XLOC_138410	ENSRNOT00000083344	0.952115505	1.83E-06
TUCP_003219	XLOC_138410	ENSRNOT00000082627	-0.963742951	4.64E-07
TUCP_003219	XLOC_138410	ENSRNOT00000003544	-0.953218436	1.63E-06
TUCP_003219	XLOC_138410	ENSRNOT00000011082	-0.959083385	8.43E-07
TUCP_003219	XLOC_138410	ENSRNOT00000087748	-0.960249458	7.31E-07
TUCP_003219	XLOC_138410	ENSRNOT00000085334	0.967295792	2.79E-07
TUCP_003219	XLOC_138410	ENSRNOT00000014506	-0.96390394	4.54E-07

TUCP_003219	XLOC_138410	ENSRNOT00000023262	-0.971760078	1.35E-07
TUCP_003219	XLOC_138410	ENSRNOT00000013067	-0.958814814	8.71E-07
TUCP_003219	XLOC_138410	ENSRNOT00000004351	-0.980469247	2.17E-08
TUCP_003219	XLOC_138410	ENSRNOT00000078185	-0.962399689	5.56E-07
TUCP_003219	XLOC_138410	ENSRNOT00000036658	-0.967630609	2.65E-07
TUCP_003219	XLOC_138410	ENSRNOT00000089611	0.96853677	2.30E-07
TUCP_003219	XLOC_138410	ENSRNOT00000086260	-0.960554658	7.04E-07
TUCP_003219	XLOC_138410	ENSRNOT00000008772	-0.970015028	1.82E-07
TUCP_003219	XLOC_138410	ENSRNOT00000066098	-0.953348483	1.61E-06
TUCP_003219	XLOC_138410	ENSRNOT00000068026	-0.973319004	1.02E-07
TUCP_003219	XLOC_138410	ENSRNOT00000029701	0.984555643	6.74E-09
TUCP_003219	XLOC_138410	ENSRNOT00000074586	-0.9548037	1.38E-06
TUCP_003219	XLOC_138410	ENSRNOT00000058382	0.956384526	1.16E-06
TUCP_003219	XLOC_138410	ENSRNOT00000064983	0.95044489	2.17E-06
TUCP_003219	XLOC_138410	ENSRNOT00000026531	-0.965457095	3.65E-07
TUCP_003219	XLOC_138410	ENSRNOT00000035605	0.951681732	1.91E-06
TUCP_003219	XLOC_138410	ENSRNOT00000020316	-0.950042407	2.25E-06
TUCP_003219	XLOC_138410	ENSRNOT00000017746	-0.958891082	8.63E-07
TUCP_003219	XLOC_138410	ENSRNOT00000018153	-0.960674065	6.93E-07
TUCP_003219	XLOC_138410	ENSRNOT00000028290	-0.957700583	9.93E-07
TUCP_003219	XLOC_138410	ENSRNOT00000017626	-0.954215858	1.47E-06
TUCP_003219	XLOC_138410	ENSRNOT00000003106	-0.950980212	2.05E-06
TUCP_003219	XLOC_138410	ENSRNOT00000017291	-0.959994096	7.55E-07
TUCP_003219	XLOC_138410	ENSRNOT00000015476	-0.962071691	5.80E-07
TUCP_003219	XLOC_138410	ENSRNOT00000071897	-0.951582005	1.93E-06
TUCP_003219	XLOC_138410	ENSRNOT00000066923	-0.968720371	2.24E-07
TUCP_003219	XLOC_138410	ENSRNOT00000040169	0.966861189	2.98E-07
TUCP_003219	XLOC_138410	ENSRNOT00000005343	-0.951418986	1.96E-06
TUCP_003219	XLOC_138410	ENSRNOT00000009327	-0.960421636	7.16E-07
TUCP_003219	XLOC_138410	ENSRNOT00000015518	-0.962030916	5.83E-07
TUCP_003219	XLOC_138410	ENSRNOT00000012216	-0.964388185	4.25E-07
TUCP_003219	XLOC_138410	ENSRNOT00000055602	-0.96177982	6.02E-07
TUCP_003219	XLOC_138410	ENSRNOT00000037015	0.96858911	2.28E-07
TUCP_003219	XLOC_138410	ENSRNOT00000008074	-0.962972655	5.15E-07
TUCP_003219	XLOC_138410	ENSRNOT00000024452	-0.958192266	9.38E-07
TUCP_003219	XLOC_138410	ENSRNOT00000008244	0.95017021	2.22E-06
TUCP_003219	XLOC_138410	ENSRNOT00000015139	-0.950430075	2.17E-06
TUCP_003219	XLOC_138410	ENSRNOT00000061201	-0.958358076	9.19E-07
TUCP_003219	XLOC_138410	ENSRNOT00000002187	-0.952425854	1.77E-06
TUCP_003219	XLOC_138410	ENSRNOT00000066952	0.972431382	1.20E-07
TUCP_003219	XLOC_138410	ENSRNOT00000011130	-0.963144539	5.03E-07
TUCP_003219	XLOC_138410	ENSRNOT00000007882	-0.952767045	1.71E-06
TUCP_003219	XLOC_138410	ENSRNOT00000049285	-0.951196002	2.01E-06
TUCP_003219	XLOC_138410	ENSRNOT00000015239	-0.957316197	1.04E-06
TUCP_003219	XLOC_138410	ENSRNOT00000077146	-0.954839733	1.37E-06
TUCP_003219	XLOC_138410	ENSRNOT00000087329	-0.972408125	1.20E-07
TUCP_003219	XLOC_138410	ENSRNOT00000013463	0.95366542	1.56E-06
TUCP_003219	XLOC_138410	ENSRNOT00000039177	-0.95085835	2.08E-06
TUCP_003219	XLOC_138410	ENSRNOT00000089673	-0.954589501	1.41E-06
TUCP_003219	XLOC_138410	ENSRNOT00000057953	0.955122933	1.33E-06
TUCP_003219	XLOC_138410	ENSRNOT00000029704	-0.956869728	1.09E-06

TUCP_003219	XLOC_138410	ENSRNOT00000075209	-0.952929598	1.68E-06
TUCP_003219	XLOC_138410	ENSRNOT00000057738	0.982747385	1.17E-08
TUCP_003219	XLOC_138410	ENSRNOT00000025966	0.950005381	2.26E-06
TUCP_003219	XLOC_138410	ENSRNOT00000009202	0.969658211	1.92E-07
TUCP_003219	XLOC_138410	ENSRNOT00000066133	-0.950911685	2.07E-06
TUCP_003219	XLOC_138410	ENSRNOT00000010993	0.957027978	1.07E-06
TUCP_003219	XLOC_138410	ENSRNOT00000075630	-0.951165137	2.01E-06
TUCP_003219	XLOC_138410	ENSRNOT00000012432	-0.951640158	1.92E-06
TUCP_003219	XLOC_138410	ENSRNOT00000080216	-0.951910819	1.87E-06
TUCP_003219	XLOC_138410	ENSRNOT00000035385	0.954150536	1.48E-06
TUCP_003219	XLOC_138410	ENSRNOT00000005873	-0.979790782	2.57E-08
TUCP_003219	XLOC_138410	ENSRNOT00000014924	-0.955666585	1.25E-06
TUCP_003219	XLOC_138410	ENSRNOT00000023543	-0.955208625	1.32E-06
TUCP_003219	XLOC_138410	ENSRNOT00000065897	0.952694956	1.72E-06
TUCP_003219	XLOC_138410	ENSRNOT00000002114	-0.954693658	1.39E-06
TUCP_003219	XLOC_138410	ENSRNOT00000028737	-0.953258417	1.62E-06
TUCP_003219	XLOC_138410	ENSRNOT00000035145	-0.960330702	7.24E-07
TUCP_003219	XLOC_138410	ENSRNOT00000085295	-0.965082762	3.85E-07
TUCP_003219	XLOC_138410	ENSRNOT00000015537	0.953114532	1.65E-06
TUCP_003219	XLOC_138410	ENSRNOT00000023014	0.965386902	3.69E-07
TUCP_003219	XLOC_138410	ENSRNOT00000002445	-0.952017147	1.85E-06
TUCP_003219	XLOC_138410	ENSRNOT00000023965	-0.957753482	9.87E-07
TUCP_003219	XLOC_138410	ENSRNOT00000000120	0.96135124	6.36E-07
TUCP_003219	XLOC_138410	ENSRNOT00000074150	0.950704018	2.11E-06
TUCP_003219	XLOC_138410	ENSRNOT00000093672	-0.957953497	9.64E-07
TUCP_003219	XLOC_138410	ENSRNOT00000060698	-0.953773577	1.54E-06
TUCP_003219	XLOC_138410	ENSRNOT00000026760	-0.954452129	1.43E-06
TUCP_003219	XLOC_138410	ENSRNOT00000075800	-0.970859959	1.58E-07
TUCP_003219	XLOC_138410	ENSRNOT00000082709	-0.959441643	8.07E-07
TUCP_003219	XLOC_138410	ENSRNOT00000015495	-0.951300163	1.99E-06
TUCP_003219	XLOC_138410	ENSRNOT00000017998	0.951234009	2.00E-06
TUCP_003219	XLOC_138410	ENSRNOT00000016568	0.972593505	1.16E-07
TUCP_003219	XLOC_138410	ENSRNOT00000027360	-0.950143272	2.23E-06
TUCP_003219	XLOC_138410	ENSRNOT00000013431	-0.951559606	1.94E-06
TUCP_003219	XLOC_138410	ENSRNOT00000082904	0.956937645	1.08E-06
TUCP_003219	XLOC_138410	ENSRNOT00000015223	0.955401118	1.29E-06
TUCP_003219	XLOC_138410	ENSRNOT00000088679	-0.958174397	9.40E-07
TUCP_003219	XLOC_138410	ENSRNOT00000051494	0.976145777	5.84E-08
TUCP_003219	XLOC_138410	ENSRNOT00000080410	-0.950040744	2.25E-06
TUCP_003219	XLOC_138410	ENSRNOT00000027967	0.959434292	8.08E-07
TUCP_003219	XLOC_138410	ENSRNOT00000025705	0.955254965	1.31E-06
TUCP_003219	XLOC_138410	ENSRNOT00000021030	-0.951949605	1.86E-06
TUCP_003219	XLOC_138410	ENSRNOT00000088073	-0.957035183	1.07E-06
TUCP_003219	XLOC_138410	ENSRNOT00000008113	-0.950897845	2.07E-06
TUCP_001861	XLOC_077321	ENSRNOT00000085599	0.982429661	1.28E-08
TUCP_001861	XLOC_077321	ENSRNOT00000006689	0.974407935	8.28E-08
TUCP_001861	XLOC_077321	ENSRNOT00000081534	0.980161392	2.34E-08
TUCP_001861	XLOC_077321	ENSRNOT00000085301	0.98002468	2.42E-08
TUCP_001861	XLOC_077321	ENSRNOT00000038729	0.982795166	1.15E-08
TUCP_001861	XLOC_077321	ENSRNOT00000086385	0.982795166	1.15E-08
TUCP_001861	XLOC_077321	ENSRNOT00000078561	0.96616209	3.30E-07

TUCP_001861	XLOC_077321	ENSRNOT00000087613	0.982795166	1.15E-08
TUCP_001861	XLOC_077321	ENSRNOT00000073350	0.967528886	2.69E-07
TUCP_001861	XLOC_077321	ENSRNOT00000023062	0.982795166	1.15E-08
TUCP_001861	XLOC_077321	ENSRNOT00000058834	0.982795166	1.15E-08
TUCP_001861	XLOC_077321	ENSRNOT00000079420	0.982795166	1.15E-08
TUCP_001861	XLOC_077321	ENSRNOT00000090761	0.982795166	1.15E-08
TUCP_001861	XLOC_077321	ENSRNOT00000055923	0.982795166	1.15E-08
TUCP_001861	XLOC_077321	ENSRNOT00000015840	0.966635582	3.08E-07
TUCP_001861	XLOC_077321	ENSRNOT00000071930	0.982795166	1.15E-08
TUCP_001861	XLOC_077321	ENSRNOT00000001996	0.973131253	1.05E-07
TUCP_001861	XLOC_077321	ENSRNOT00000076756	0.982795166	1.15E-08
TUCP_001861	XLOC_077321	ENSRNOT00000081527	0.981289264	1.75E-08
TUCP_001861	XLOC_077321	ENSRNOT00000085067	0.964389244	4.25E-07
TUCP_001861	XLOC_077321	ENSRNOT00000091663	0.975843555	6.22E-08
TUCP_001861	XLOC_077321	ENSRNOT00000081780	0.982795166	1.15E-08
TUCP_001861	XLOC_077321	ENSRNOT00000089114	0.982795166	1.15E-08
TUCP_001861	XLOC_077321	ENSRNOT00000021609	0.954345024	1.45E-06
TUCP_001861	XLOC_077321	ENSRNOT00000072402	0.973994864	8.97E-08
TUCP_001861	XLOC_077321	ENSRNOT00000073316	0.982795166	1.15E-08
TUCP_001861	XLOC_077321	ENSRNOT00000014498	0.968496189	2.32E-07
TUCP_001861	XLOC_077321	ENSRNOT00000083296	0.958849301	8.67E-07
TUCP_001861	XLOC_077321	ENSRNOT00000071663	0.982795166	1.15E-08
TUCP_001861	XLOC_077321	ENSRNOT00000042860	0.982795166	1.15E-08
TUCP_001861	XLOC_077321	ENSRNOT00000088421	0.982795166	1.15E-08
TUCP_001861	XLOC_077321	ENSRNOT00000038898	0.982795166	1.15E-08
TUCP_001861	XLOC_077321	ENSRNOT00000064936	0.964400664	4.24E-07
TUCP_001861	XLOC_077321	ENSRNOT00000042777	0.969844454	1.87E-07
TUCP_001861	XLOC_077321	ENSRNOT00000010029	0.982795166	1.15E-08
TUCP_001860	XLOC_077315	ENSRNOT00000085599	0.999938502	6.93E-21
TUCP_001860	XLOC_077315	ENSRNOT00000006689	0.9944189	4.22E-11
TUCP_001860	XLOC_077315	ENSRNOT00000081534	0.987999291	1.92E-09
TUCP_001860	XLOC_077315	ENSRNOT00000085301	0.99793679	2.93E-13
TUCP_001860	XLOC_077315	ENSRNOT00000038729	1	1.33E-79
TUCP_001860	XLOC_077315	ENSRNOT00000086385	1	1.33E-79
TUCP_001860	XLOC_077315	ENSRNOT00000077695	0.96038616	7.19E-07
TUCP_001860	XLOC_077315	ENSRNOT00000078561	0.986503348	3.45E-09
TUCP_001860	XLOC_077315	ENSRNOT00000087613	1	0
TUCP_001860	XLOC_077315	ENSRNOT00000026032	0.953050675	1.66E-06
TUCP_001860	XLOC_077315	ENSRNOT00000073350	0.9658754	3.44E-07
TUCP_001860	XLOC_077315	ENSRNOT00000023062	1	1.33E-79
TUCP_001860	XLOC_077315	ENSRNOT00000058834	1	0
TUCP_001860	XLOC_077315	ENSRNOT00000079420	1	0
TUCP_001860	XLOC_077315	ENSRNOT00000090761	1	0
TUCP_001860	XLOC_077315	ENSRNOT00000055923	1	0
TUCP_001860	XLOC_077315	ENSRNOT00000092416	0.963085379	5.07E-07
TUCP_001860	XLOC_077315	ENSRNOT00000071930	1	0
TUCP_001860	XLOC_077315	ENSRNOT00000001996	0.974721715	7.79E-08
TUCP_001860	XLOC_077315	ENSRNOT00000076756	1	0
TUCP_001860	XLOC_077315	ENSRNOT00000081527	0.999420479	5.14E-16
TUCP_001860	XLOC_077315	ENSRNOT00000085067	0.960262084	7.30E-07
TUCP_001860	XLOC_077315	ENSRNOT00000091663	0.99642667	4.56E-12

TUCP_001860	XLOC_077315	ENSRNOT00000081780	1	0
TUCP_001860	XLOC_077315	ENSRNOT00000093689	0.953599805	1.57E-06
TUCP_001860	XLOC_077315	ENSRNOT00000089114	1	0
TUCP_001860	XLOC_077315	ENSRNOT00000021609	0.977017895	4.86E-08
TUCP_001860	XLOC_077315	ENSRNOT00000073316	1	0
TUCP_001860	XLOC_077315	ENSRNOT00000086557	0.977413455	4.46E-08
TUCP_001860	XLOC_077315	ENSRNOT00000071663	1	0
TUCP_001860	XLOC_077315	ENSRNOT00000042860	1	1.33E-79
TUCP_001860	XLOC_077315	ENSRNOT00000072962	0.971287317	1.46E-07
TUCP_001860	XLOC_077315	ENSRNOT00000088421	1	0
TUCP_001860	XLOC_077315	ENSRNOT00000038898	1	0
TUCP_001860	XLOC_077315	ENSRNOT00000015840	0.959452012	8.06E-07
TUCP_001860	XLOC_077315	ENSRNOT00000064936	0.985957557	4.20E-09
TUCP_001860	XLOC_077315	ENSRNOT00000042777	0.990642579	5.56E-10
TUCP_001860	XLOC_077315	ENSRNOT00000010029	1	0
TUCP_004249	XLOC_186698	ENSRNOT00000005958	0.96821316	2.42E-07
TUCP_004249	XLOC_186698	ENSRNOT00000000743	0.963279304	4.94E-07
TUCP_004249	XLOC_186698	ENSRNOT00000027083	0.953499378	1.58E-06
TUCP_004249	XLOC_186698	ENSRNOT00000021348	0.956261682	1.17E-06
TUCP_004249	XLOC_186698	ENSRNOT00000066041	-0.950482033	2.16E-06
TUCP_004249	XLOC_186698	ENSRNOT00000023133	0.960744594	6.87E-07
TUCP_004249	XLOC_186698	ENSRNOT00000073559	0.960901588	6.74E-07
TUCP_004249	XLOC_186698	ENSRNOT00000001855	0.96038561	7.19E-07
TUCP_004249	XLOC_186698	ENSRNOT00000023638	0.967216059	2.82E-07
TUCP_004249	XLOC_186698	ENSRNOT00000005228	0.951354271	1.98E-06
TUCP_004249	XLOC_186698	ENSRNOT00000006175	0.960220265	7.34E-07
TUCP_004249	XLOC_186698	ENSRNOT00000023144	0.984037159	7.95E-09
TUCP_004249	XLOC_186698	ENSRNOT00000082869	0.959953231	7.58E-07
TUCP_004249	XLOC_186698	ENSRNOT00000016649	0.960520411	7.07E-07
TUCP_004249	XLOC_186698	ENSRNOT00000026941	0.957102371	1.06E-06
TUCP_004249	XLOC_186698	ENSRNOT00000016975	0.955176279	1.32E-06
TUCP_004249	XLOC_186698	ENSRNOT00000091128	-0.957112147	1.06E-06
TUCP_004249	XLOC_186698	ENSRNOT00000081981	0.966577388	3.11E-07
TUCP_004249	XLOC_186698	ENSRNOT00000033017	0.982820996	1.14E-08
TUCP_004249	XLOC_186698	ENSRNOT00000026543	0.978080587	3.84E-08
TUCP_004249	XLOC_186698	ENSRNOT00000012187	0.96131534	6.39E-07
TUCP_004249	XLOC_186698	ENSRNOT00000066810	0.95390906	1.52E-06
TUCP_004249	XLOC_186698	ENSRNOT00000024969	0.973292033	1.02E-07
TUCP_004249	XLOC_186698	ENSRNOT00000067400	0.963825195	4.59E-07
TUCP_004249	XLOC_186698	ENSRNOT00000013049	0.956576793	1.13E-06
TUCP_004249	XLOC_186698	ENSRNOT00000060943	0.953619106	1.56E-06
TUCP_004249	XLOC_186698	ENSRNOT00000025075	0.963996417	4.48E-07
TUCP_004249	XLOC_186698	ENSRNOT00000035989	0.952967212	1.67E-06
TUCP_004249	XLOC_186698	ENSRNOT00000057160	0.971810486	1.34E-07
TUCP_004249	XLOC_186698	ENSRNOT00000024402	0.966263545	3.25E-07
TUCP_004249	XLOC_186698	ENSRNOT00000089933	0.971759361	1.35E-07
TUCP_004249	XLOC_186698	ENSRNOT00000009811	0.967642654	2.65E-07
TUCP_004249	XLOC_186698	ENSRNOT00000065168	0.967919196	2.54E-07
TUCP_004249	XLOC_186698	ENSRNOT00000085228	0.955587029	1.26E-06
TUCP_004249	XLOC_186698	ENSRNOT00000020134	0.961699016	6.09E-07
TUCP_004249	XLOC_186698	ENSRNOT00000077231	0.950990417	2.05E-06

TUCP_004249	XLOC_186698	ENSRNOT00000017021	0.950923796	2.06E-06
TUCP_004249	XLOC_186698	ENSRNOT00000061392	0.955167037	1.32E-06
TUCP_004249	XLOC_186698	ENSRNOT00000002410	0.958839645	8.68E-07
TUCP_004249	XLOC_186698	ENSRNOT00000008655	0.953422409	1.60E-06
TUCP_004249	XLOC_186698	ENSRNOT00000088054	0.962016006	5.84E-07
TUCP_004249	XLOC_186698	ENSRNOT00000041044	0.971241552	1.48E-07
TUCP_004249	XLOC_186698	ENSRNOT00000010541	0.969614177	1.94E-07
TUCP_004249	XLOC_186698	ENSRNOT00000047192	-0.975797722	6.28E-08
TUCP_004249	XLOC_186698	ENSRNOT00000091909	0.95681522	1.10E-06
TUCP_004249	XLOC_186698	ENSRNOT00000083220	0.973076776	1.06E-07
TUCP_004249	XLOC_186698	ENSRNOT00000003780	0.950258958	2.21E-06
TUCP_004249	XLOC_186698	ENSRNOT00000026643	0.953690976	1.55E-06
TUCP_004249	XLOC_186698	ENSRNOT00000019133	0.957262447	1.04E-06
TUCP_004249	XLOC_186698	ENSRNOT00000013274	0.951699646	1.91E-06
TUCP_004249	XLOC_186698	ENSRNOT00000052018	0.956328435	1.16E-06
TUCP_004249	XLOC_186698	ENSRNOT00000010898	0.951079948	2.03E-06
TUCP_004249	XLOC_186698	ENSRNOT00000047093	0.962016176	5.84E-07
TUCP_004249	XLOC_186698	ENSRNOT00000033975	0.965917598	3.42E-07
TUCP_004249	XLOC_186698	ENSRNOT00000090123	0.956312533	1.16E-06
TUCP_004249	XLOC_186698	ENSRNOT00000088599	0.962933523	5.18E-07
TUCP_004249	XLOC_186698	ENSRNOT00000080024	0.95789971	9.70E-07
TUCP_004249	XLOC_186698	ENSRNOT00000081047	0.973793101	9.32E-08
TUCP_004249	XLOC_186698	ENSRNOT00000081512	0.972470847	1.19E-07
TUCP_004249	XLOC_186698	ENSRNOT00000023989	0.950249692	2.21E-06
TUCP_004249	XLOC_186698	ENSRNOT00000087429	0.95921779	8.30E-07
TUCP_004249	XLOC_186698	ENSRNOT00000060362	0.966976016	2.93E-07
TUCP_004249	XLOC_186698	ENSRNOT00000084088	0.961193194	6.49E-07
TUCP_004249	XLOC_186698	ENSRNOT00000027910	0.959587074	7.93E-07
TUCP_004249	XLOC_186698	ENSRNOT00000052224	0.95946337	8.05E-07
TUCP_004249	XLOC_186698	ENSRNOT00000078070	0.973134698	1.05E-07
TUCP_004249	XLOC_186698	ENSRNOT00000076729	0.956313389	1.16E-06
TUCP_004249	XLOC_186698	ENSRNOT00000091265	0.954094176	1.49E-06
TUCP_004249	XLOC_186698	ENSRNOT00000016191	0.955089527	1.33E-06
TUCP_004249	XLOC_186698	ENSRNOT00000078390	-0.954823999	1.37E-06
TUCP_004249	XLOC_186698	ENSRNOT00000065501	0.955954636	1.21E-06
TUCP_004249	XLOC_186698	ENSRNOT00000017753	0.951312122	1.99E-06
TUCP_004249	XLOC_186698	ENSRNOT00000003568	0.960117642	7.43E-07
TUCP_004249	XLOC_186698	ENSRNOT00000031904	0.975798138	6.28E-08
TUCP_004249	XLOC_186698	ENSRNOT00000022074	0.957570851	1.01E-06
TUCP_004249	XLOC_186698	ENSRNOT00000027337	0.950823524	2.08E-06
TUCP_004249	XLOC_186698	ENSRNOT00000004046	-0.961454773	6.28E-07
TUCP_004249	XLOC_186698	ENSRNOT00000010367	0.961912329	5.92E-07
TUCP_004249	XLOC_186698	ENSRNOT00000076669	0.960384258	7.19E-07
TUCP_004249	XLOC_186698	ENSRNOT00000038807	0.962012736	5.84E-07
TUCP_004249	XLOC_186698	ENSRNOT00000050834	-0.951795095	1.89E-06
TUCP_004249	XLOC_186698	ENSRNOT00000066024	0.968181699	2.43E-07
TUCP_004249	XLOC_186698	ENSRNOT00000025808	0.957750455	9.87E-07
TUCP_004249	XLOC_186698	ENSRNOT00000012736	0.954695778	1.39E-06
TUCP_004249	XLOC_186698	ENSRNOT00000027970	0.951373674	1.97E-06
TUCP_004249	XLOC_186698	ENSRNOT00000010748	0.958271083	9.29E-07
TUCP_004249	XLOC_186698	ENSRNOT00000083127	0.953851649	1.53E-06

TUCP_004249	XLOC_186698	ENSRNOT00000078306	0.961626838	6.14E-07
TUCP_004249	XLOC_186698	ENSRNOT00000035474	0.952848433	1.70E-06
TUCP_004249	XLOC_186698	ENSRNOT00000080695	0.97481101	7.66E-08
TUCP_004249	XLOC_186698	ENSRNOT00000029125	0.960808675	6.82E-07
TUCP_004249	XLOC_186698	ENSRNOT00000060696	0.976553724	5.37E-08
TUCP_004249	XLOC_186698	ENSRNOT00000042125	0.973056732	1.07E-07
TUCP_004249	XLOC_186698	ENSRNOT00000014814	0.952101085	1.83E-06
TUCP_004249	XLOC_186698	ENSRNOT00000080207	0.969894804	1.85E-07
TUCP_004249	XLOC_186698	ENSRNOT00000008982	0.954129083	1.48E-06
TUCP_004249	XLOC_186698	ENSRNOT00000090057	0.959971834	7.57E-07
TUCP_004249	XLOC_186698	ENSRNOT00000067866	0.95233257	1.79E-06
TUCP_004249	XLOC_186698	ENSRNOT00000037800	0.953200513	1.63E-06
TUCP_004249	XLOC_186698	ENSRNOT00000022796	0.960506169	7.08E-07
TUCP_004249	XLOC_186698	ENSRNOT00000005311	0.952669343	1.73E-06
TUCP_004249	XLOC_186698	ENSRNOT00000015799	0.987432801	2.42E-09
TUCP_002588	XLOC_109609	ENSRNOT00000024186	0.983200502	1.02E-08
TUCP_002588	XLOC_109609	ENSRNOT00000074753	0.998182349	1.56E-13
TUCP_002588	XLOC_109609	ENSRNOT00000004876	0.959071443	8.44E-07
TUCP_002588	XLOC_109609	ENSRNOT00000041904	1	0
TUCP_002588	XLOC_109609	ENSRNOT00000083487	0.989515314	9.80E-10
TUCP_002588	XLOC_109609	ENSRNOT00000077990	1	0
TUCP_002588	XLOC_109609	ENSRNOT00000038826	0.99977672	4.37E-18
TUCP_002588	XLOC_109609	ENSRNOT00000046842	0.973644314	9.58E-08
TUCP_002588	XLOC_109609	ENSRNOT00000037169	0.980329406	2.24E-08
TUCP_002588	XLOC_109609	ENSRNOT00000021243	0.987425406	2.42E-09
TUCP_002588	XLOC_109609	ENSRNOT00000060250	0.999933853	9.97E-21
TUCP_002588	XLOC_109609	ENSRNOT00000091934	1	0
TUCP_002588	XLOC_109609	ENSRNOT00000046585	0.982941597	1.11E-08
TUCP_002588	XLOC_109609	ENSRNOT00000080794	0.990839253	5.00E-10
TUCP_002588	XLOC_109609	ENSRNOT00000014456	0.991697153	3.06E-10
TUCP_002588	XLOC_109609	ENSRNOT00000031205	0.991394766	3.66E-10
TUCP_002588	XLOC_109609	ENSRNOT00000044145	1	0
TUCP_002588	XLOC_109609	ENSRNOT00000088381	0.989013276	1.24E-09
TUCP_002588	XLOC_109609	ENSRNOT00000011743	0.98678427	3.11E-09
TUCP_002588	XLOC_109609	ENSRNOT00000089929	1	0
TUCP_002588	XLOC_109609	ENSRNOT00000088624	1	0
TUCP_002588	XLOC_109609	ENSRNOT00000085659	0.989908621	8.10E-10
TUCP_002588	XLOC_109609	ENSRNOT00000015866	0.994066664	5.73E-11
TUCP_002588	XLOC_109609	ENSRNOT00000078633	1	0
TUCP_002588	XLOC_109609	ENSRNOT00000034907	0.995318542	1.76E-11
TUCP_002588	XLOC_109609	ENSRNOT00000093230	0.958295175	9.26E-07
TUCP_002588	XLOC_109609	ENSRNOT00000051835	1	0
TUCP_002588	XLOC_109609	ENSRNOT00000079087	1	0
TUCP_002588	XLOC_109609	ENSRNOT00000037206	1	0
TUCP_002588	XLOC_109609	ENSRNOT00000083261	0.970991658	1.54E-07
TUCP_002588	XLOC_109609	ENSRNOT00000080590	0.999889216	1.31E-19
TUCP_002588	XLOC_109609	ENSRNOT00000027552	0.999801024	2.46E-18
TUCP_002588	XLOC_109609	ENSRNOT00000077014	0.995074376	2.26E-11
TUCP_002588	XLOC_109609	ENSRNOT00000080453	0.99994332	4.61E-21
TUCP_002588	XLOC_109609	ENSRNOT00000089057	0.968243551	2.41E-07
TUCP_002588	XLOC_109609	ENSRNOT00000066839	0.991868612	2.76E-10

TUCP_002588	XLOC_109609	ENSRNOT00000061284	0.955566942	1.27E-06
TUCP_002588	XLOC_109609	ENSRNOT00000013423	0.959677621	7.84E-07
TUCP_002588	XLOC_109609	ENSRNOT00000071884	0.974769955	7.72E-08
TUCP_002588	XLOC_109609	ENSRNOT00000004128	0.961829362	5.99E-07
TUCP_002588	XLOC_109609	ENSRNOT00000026316	0.964408046	4.24E-07
TUCP_002588	XLOC_109609	ENSRNOT00000057729	0.999384184	6.97E-16
TUCP_002588	XLOC_109609	ENSRNOT00000078663	0.975192577	7.10E-08
TUCP_002588	XLOC_109609	ENSRNOT00000064250	0.983131977	1.05E-08
TUCP_002588	XLOC_109609	ENSRNOT00000021608	0.965228881	3.78E-07
TUCP_002588	XLOC_109609	ENSRNOT00000029634	0.995816134	1.00E-11
TUCP_002588	XLOC_109609	ENSRNOT00000093579	0.958564264	8.97E-07
TUCP_002588	XLOC_109609	ENSRNOT00000086349	0.957242511	1.05E-06
TUCP_002588	XLOC_109609	ENSRNOT00000065460	1	0
TUCP_002588	XLOC_109609	ENSRNOT00000003817	0.996778712	2.72E-12
TUCP_002588	XLOC_109609	ENSRNOT00000016713	1	0
TUCP_002588	XLOC_109609	ENSRNOT00000079260	0.996448245	4.42E-12
TUCP_002588	XLOC_109609	ENSRNOT00000039294	1	0
TUCP_002588	XLOC_109609	ENSRNOT00000092913	0.952980228	1.67E-06
TUCP_002586	XLOC_109608	ENSRNOT00000093579	0.955919097	1.22E-06
TUCP_002586	XLOC_109608	ENSRNOT00000012170	0.970605802	1.64E-07
TUCP_002586	XLOC_109608	ENSRNOT00000020329	0.950338901	2.19E-06
TUCP_002586	XLOC_109608	ENSRNOT00000061139	0.966877392	2.97E-07
TUCP_002586	XLOC_109608	ENSRNOT00000029531	-0.950335181	2.19E-06
TUCP_002585	XLOC_109601	ENSRNOT00000071884	0.962746932	5.31E-07
TUCP_002585	XLOC_109601	ENSRNOT00000009825	0.955011701	1.35E-06
TUCP_002585	XLOC_109601	ENSRNOT00000040270	0.961736747	6.06E-07
TUCP_002585	XLOC_109601	ENSRNOT00000008041	0.960560435	7.03E-07
TUCP_002585	XLOC_109601	ENSRNOT00000093579	0.971846748	1.33E-07
TUCP_002585	XLOC_109601	ENSRNOT00000078663	0.951475969	1.95E-06
TUCP_002585	XLOC_109601	ENSRNOT00000085659	0.955772035	1.24E-06
TUCP_004835	XLOC_214873	ENSRNOT00000093467	0.968025767	2.49E-07
TUCP_004835	XLOC_214873	ENSRNOT00000029340	0.962076241	5.80E-07
TUCP_004835	XLOC_214873	ENSRNOT00000038646	0.964533064	4.16E-07
TUCP_004835	XLOC_214873	ENSRNOT00000092579	0.964533064	4.16E-07
TUCP_004835	XLOC_214873	ENSRNOT00000082082	0.95412077	1.48E-06
TUCP_004835	XLOC_214873	ENSRNOT00000036383	0.985300353	5.27E-09
TUCP_004835	XLOC_214873	ENSRNOT00000027882	-0.960528131	7.06E-07
TUCP_004835	XLOC_214873	ENSRNOT00000076528	0.963812757	4.60E-07
TUCP_004835	XLOC_214873	ENSRNOT00000083034	0.957568197	1.01E-06
TUCP_004835	XLOC_214873	ENSRNOT00000089607	0.954415807	1.44E-06
TUCP_004835	XLOC_214873	ENSRNOT00000021748	0.964533064	4.16E-07
TUCP_004835	XLOC_214873	ENSRNOT00000013459	0.998059381	2.16E-13
TUCP_004835	XLOC_214873	ENSRNOT00000078137	0.964533064	4.16E-07
TUCP_004835	XLOC_214873	ENSRNOT00000092740	0.964533064	4.16E-07
TUCP_004835	XLOC_214873	ENSRNOT00000067034	0.962109367	5.77E-07
TUCP_004835	XLOC_214873	ENSRNOT00000000117	0.952306264	1.79E-06
TUCP_004835	XLOC_214873	ENSRNOT00000071877	0.958667496	8.86E-07
TUCP_004835	XLOC_214873	ENSRNOT00000070958	0.964533064	4.16E-07
TUCP_004835	XLOC_214873	ENSRNOT00000027268	0.964533064	4.16E-07
TUCP_004835	XLOC_214873	ENSRNOT00000004459	0.964533064	4.16E-07
TUCP_004835	XLOC_214873	ENSRNOT00000089235	0.965272383	3.75E-07

TUCP_004835	XLOC_214873	ENSRNOT00000088168	0.964533064	4.16E-07
TUCP_004835	XLOC_214873	ENSRNOT00000088148	0.964533064	4.16E-07
TUCP_004835	XLOC_214873	ENSRNOT00000088527	0.964533064	4.16E-07
TUCP_004835	XLOC_214873	ENSRNOT00000078701	0.964533064	4.16E-07
TUCP_004835	XLOC_214873	ENSRNOT00000090078	0.964533064	4.16E-07
TUCP_004835	XLOC_214873	ENSRNOT00000089085	0.964533064	4.16E-07
TUCP_004835	XLOC_214873	ENSRNOT00000008153	0.965249175	3.76E-07
TUCP_004835	XLOC_214873	ENSRNOT00000007971	0.978314808	3.64E-08
TUCP_004835	XLOC_214873	ENSRNOT00000079189	0.953380386	1.60E-06
TUCP_004835	XLOC_214873	ENSRNOT00000078020	0.954527139	1.42E-06
TUCP_004835	XLOC_214873	ENSRNOT00000009776	0.964533064	4.16E-07
TUCP_003989	XLOC_175146	ENSRNOT00000084075	0.967925883	2.53E-07
TUCP_003989	XLOC_175146	ENSRNOT00000074465	0.950020565	2.26E-06
TUCP_003989	XLOC_175146	ENSRNOT00000079805	0.951965221	1.86E-06
TUCP_003989	XLOC_175146	ENSRNOT00000040912	0.950020565	2.26E-06
TUCP_003989	XLOC_175146	ENSRNOT00000072648	0.950020565	2.26E-06
TUCP_003989	XLOC_175146	ENSRNOT00000070803	0.950020565	2.26E-06
TUCP_003989	XLOC_175146	ENSRNOT00000012196	0.950020565	2.26E-06
TUCP_003989	XLOC_175146	ENSRNOT00000085772	0.950020565	2.26E-06
TUCP_003989	XLOC_175146	ENSRNOT00000004577	0.966304535	3.23E-07
TUCP_003989	XLOC_175146	ENSRNOT00000015270	0.950020565	2.26E-06
TUCP_003989	XLOC_175146	ENSRNOT00000046192	0.950020565	2.26E-06
TUCP_003989	XLOC_175146	ENSRNOT00000044226	0.950020565	2.26E-06
TUCP_003989	XLOC_175146	ENSRNOT00000081954	0.966718282	3.04E-07
TUCP_003989	XLOC_175146	ENSRNOT00000080291	0.961530478	6.22E-07
TUCP_003989	XLOC_175146	ENSRNOT00000001464	0.950020565	2.26E-06
TUCP_003989	XLOC_175146	ENSRNOT00000090716	0.952991583	1.67E-06
TUCP_003989	XLOC_175146	ENSRNOT00000057873	0.950020565	2.26E-06
TUCP_003989	XLOC_175146	ENSRNOT00000076044	0.950020565	2.26E-06
TUCP_003989	XLOC_175146	ENSRNOT00000087764	0.959480462	8.04E-07
TUCP_003989	XLOC_175146	ENSRNOT00000093284	0.951700504	1.91E-06
TUCP_003989	XLOC_175146	ENSRNOT00000083002	0.959174169	8.34E-07
TUCP_003989	XLOC_175146	ENSRNOT00000086248	0.950020565	2.26E-06
TUCP_003989	XLOC_175146	ENSRNOT00000089750	0.950025677	2.26E-06
TUCP_003989	XLOC_175146	ENSRNOT00000034772	0.968558446	2.30E-07
TUCP_003989	XLOC_175146	ENSRNOT00000026473	0.950939074	2.06E-06
TUCP_003989	XLOC_175146	ENSRNOT00000002834	0.950020565	2.26E-06
TUCP_003989	XLOC_175146	ENSRNOT00000092755	0.950020565	2.26E-06
TUCP_003989	XLOC_175146	ENSRNOT00000031683	0.950020565	2.26E-06
TUCP_003989	XLOC_175146	ENSRNOT00000074353	0.950020565	2.26E-06
TUCP_004836	XLOC_215132	ENSRNOT00000042495	0.954104418	1.48E-06
TUCP_004836	XLOC_215132	ENSRNOT00000029915	0.99515174	2.09E-11
TUCP_004836	XLOC_215132	ENSRNOT00000090352	0.969250952	2.06E-07
TUCP_004836	XLOC_215132	ENSRNOT00000004047	0.959739386	7.79E-07
TUCP_004836	XLOC_215132	ENSRNOT00000027343	0.964626542	4.11E-07
TUCP_004836	XLOC_215132	ENSRNOT00000079494	0.952438226	1.77E-06
TUCP_004836	XLOC_215132	ENSRNOT00000055506	0.991839662	2.81E-10
TUCP_004830	XLOC_214643	ENSRNOT00000065387	0.957322131	1.04E-06
TUCP_004830	XLOC_214643	ENSRNOT00000047349	0.971806197	1.34E-07
TUCP_004830	XLOC_214643	ENSRNOT00000071028	-0.958479772	9.06E-07
TUCP_003821	XLOC_167348	ENSRNOT00000036383	0.953068019	1.66E-06

TUCP_003821	XLOC_167348	ENSRNOT00000007971	0.998101458	1.94E-13
TUCP_003821	XLOC_167348	ENSRNOT00000067034	0.950291377	2.20E-06
TUCP_003821	XLOC_167348	ENSRNOT00000027882	-0.96775174	2.60E-07
TUCP_003821	XLOC_167348	ENSRNOT00000013459	0.971271488	1.47E-07
TUCP_004832	XLOC_214832	ENSRNOT00000009649	0.954692366	1.39E-06
TUCP_004832	XLOC_214832	ENSRNOT00000077451	0.950261375	2.20E-06
TUCP_003980	XLOC_174431	ENSRNOT00000084075	0.999809585	1.97E-18
TUCP_003980	XLOC_174431	ENSRNOT00000012880	0.964032406	4.46E-07
TUCP_003980	XLOC_174431	ENSRNOT00000074465	0.997636835	5.78E-13
TUCP_003980	XLOC_174431	ENSRNOT00000090898	0.958627187	8.91E-07
TUCP_003980	XLOC_174431	ENSRNOT00000079805	0.995381355	1.64E-11
TUCP_003980	XLOC_174431	ENSRNOT00000040912	0.997636835	5.78E-13
TUCP_003980	XLOC_174431	ENSRNOT00000072648	0.997636835	5.78E-13
TUCP_003980	XLOC_174431	ENSRNOT00000007398	0.985624996	4.72E-09
TUCP_003980	XLOC_174431	ENSRNOT00000070803	0.997636835	5.78E-13
TUCP_003980	XLOC_174431	ENSRNOT00000012196	0.997636835	5.78E-13
TUCP_003980	XLOC_174431	ENSRNOT00000085772	0.997636835	5.78E-13
TUCP_003980	XLOC_174431	ENSRNOT00000004577	0.95072582	2.11E-06
TUCP_003980	XLOC_174431	ENSRNOT00000093284	0.998090195	1.99E-13
TUCP_003980	XLOC_174431	ENSRNOT00000086374	0.989119382	1.18E-09
TUCP_003980	XLOC_174431	ENSRNOT00000015270	0.997636835	5.78E-13
TUCP_003980	XLOC_174431	ENSRNOT00000046192	0.997636835	5.78E-13
TUCP_003980	XLOC_174431	ENSRNOT00000044226	0.997636835	5.78E-13
TUCP_003980	XLOC_174431	ENSRNOT00000081898	0.990511931	5.96E-10
TUCP_003980	XLOC_174431	ENSRNOT00000081954	0.97659645	5.32E-08
TUCP_003980	XLOC_174431	ENSRNOT00000080291	0.999580216	1.03E-16
TUCP_003980	XLOC_174431	ENSRNOT00000004293	0.976315231	5.64E-08
TUCP_003980	XLOC_174431	ENSRNOT00000078231	0.953800499	1.53E-06
TUCP_003980	XLOC_174431	ENSRNOT00000002834	0.997636835	5.78E-13
TUCP_003980	XLOC_174431	ENSRNOT00000001464	0.997636835	5.78E-13
TUCP_003980	XLOC_174431	ENSRNOT00000008542	0.952676457	1.73E-06
TUCP_003980	XLOC_174431	ENSRNOT00000090716	0.991599267	3.25E-10
TUCP_003980	XLOC_174431	ENSRNOT00000083542	0.968569224	2.29E-07
TUCP_003980	XLOC_174431	ENSRNOT00000076044	0.997636835	5.78E-13
TUCP_003980	XLOC_174431	ENSRNOT00000074353	0.997636835	5.78E-13
TUCP_003980	XLOC_174431	ENSRNOT00000043510	0.953043894	1.66E-06
TUCP_003980	XLOC_174431	ENSRNOT00000087122	0.978695408	3.34E-08
TUCP_003980	XLOC_174431	ENSRNOT00000074295	0.980939932	1.92E-08
TUCP_003980	XLOC_174431	ENSRNOT00000057873	0.997636835	5.78E-13
TUCP_003980	XLOC_174431	ENSRNOT00000083002	0.993013635	1.30E-10
TUCP_003980	XLOC_174431	ENSRNOT00000088399	0.997628293	5.89E-13
TUCP_003980	XLOC_174431	ENSRNOT00000086248	0.997636835	5.78E-13
TUCP_003980	XLOC_174431	ENSRNOT00000089750	0.997670871	5.38E-13
TUCP_003980	XLOC_174431	ENSRNOT00000030517	0.95553958	1.27E-06
TUCP_003980	XLOC_174431	ENSRNOT00000034772	0.996867996	2.36E-12
TUCP_003980	XLOC_174431	ENSRNOT00000026473	0.954315347	1.45E-06
TUCP_003980	XLOC_174431	ENSRNOT00000089305	0.968755543	2.23E-07
TUCP_003980	XLOC_174431	ENSRNOT00000064601	0.964400661	4.24E-07
TUCP_003980	XLOC_174431	ENSRNOT00000015870	0.960282781	7.28E-07
TUCP_003980	XLOC_174431	ENSRNOT00000080812	0.987600689	2.26E-09
TUCP_003980	XLOC_174431	ENSRNOT00000092755	0.997636835	5.78E-13

TUCP_003980	XLOC_174431	ENSRNOT00000031683	0.997636835	5.78E-13
TUCP_003980	XLOC_174431	ENSRNOT00000087764	0.986058921	4.05E-09
TUCP_003982	XLOC_174489	ENSRNOT00000092026	0.96620614	3.28E-07
TUCP_003982	XLOC_174489	ENSRNOT00000093075	1	0
TUCP_003982	XLOC_174489	ENSRNOT00000058260	1	0
TUCP_003982	XLOC_174489	ENSRNOT00000088887	1	0
TUCP_003982	XLOC_174489	ENSRNOT00000084986	1	0
TUCP_003982	XLOC_174489	ENSRNOT00000074346	0.9702256	1.75E-07
TUCP_003982	XLOC_174489	ENSRNOT00000073209	0.983711602	8.79E-09
TUCP_003982	XLOC_174489	ENSRNOT00000080155	0.973069673	1.07E-07
TUCP_003982	XLOC_174489	ENSRNOT00000072573	0.996213272	6.09E-12
TUCP_003982	XLOC_174489	ENSRNOT00000017828	0.996001583	7.99E-12
TUCP_003982	XLOC_174489	ENSRNOT00000080444	0.969625907	1.93E-07
TUCP_003982	XLOC_174489	ENSRNOT00000071312	1	0
TUCP_003982	XLOC_174489	ENSRNOT00000088245	1	0
TUCP_003982	XLOC_174489	ENSRNOT00000036565	0.969065335	2.12E-07
TUCP_003982	XLOC_174489	ENSRNOT00000058833	0.998673261	3.23E-14
TUCP_003982	XLOC_174489	ENSRNOT00000078052	1	0
TUCP_003982	XLOC_174489	ENSRNOT00000078494	0.999927093	1.62E-20
TUCP_003982	XLOC_174489	ENSRNOT00000085653	1	0
TUCP_003982	XLOC_174489	ENSRNOT00000057950	0.969329371	2.03E-07
TUCP_003982	XLOC_174489	ENSRNOT00000081992	0.999320526	1.14E-15
TUCP_003982	XLOC_174489	ENSRNOT00000038553	0.955765808	1.24E-06
TUCP_003982	XLOC_174489	ENSRNOT00000084120	0.958115447	9.46E-07
TUCP_003982	XLOC_174489	ENSRNOT00000055406	1	1.33E-79
TUCP_003982	XLOC_174489	ENSRNOT00000070817	0.998968079	9.20E-15
TUCP_003982	XLOC_174489	ENSRNOT00000026753	1	1.33E-79
TUCP_003982	XLOC_174489	ENSRNOT00000080533	0.996682529	3.15E-12
TUCP_003982	XLOC_174489	ENSRNOT00000072774	0.979665261	2.65E-08
TUCP_003982	XLOC_174489	ENSRNOT00000086031	1	0
TUCP_003982	XLOC_174489	ENSRNOT00000014312	1	0
TUCP_003982	XLOC_174489	ENSRNOT00000042882	0.963218427	4.98E-07
TUCP_003982	XLOC_174489	ENSRNOT00000088012	0.956765806	1.11E-06
TUCP_003982	XLOC_174489	ENSRNOT00000051231	0.999242378	1.96E-15
TUCP_003982	XLOC_174489	ENSRNOT00000089407	0.992986194	1.32E-10
TUCP_003982	XLOC_174489	ENSRNOT00000055034	0.998331441	1.02E-13
TUCP_003982	XLOC_174489	ENSRNOT00000046837	0.97252559	1.18E-07
TUCP_003983	XLOC_174490	ENSRNOT00000085476	0.988087438	1.85E-09
TUCP_003983	XLOC_174490	ENSRNOT00000083608	0.96644664	3.17E-07
TUCP_003983	XLOC_174490	ENSRNOT00000076035	0.970963518	1.55E-07
TUCP_003983	XLOC_174490	ENSRNOT00000029298	0.959537701	7.98E-07
TUCP_003983	XLOC_174490	ENSRNOT00000078176	0.972062478	1.28E-07
TUCP_003983	XLOC_174490	ENSRNOT00000047560	0.95410051	1.49E-06
TUCP_003983	XLOC_174490	ENSRNOT00000040386	0.950470536	2.16E-06
TUCP_003983	XLOC_174490	ENSRNOT00000024758	0.970757479	1.60E-07
TUCP_003983	XLOC_174490	ENSRNOT00000088719	0.996914267	2.19E-12
TUCP_003983	XLOC_174490	ENSRNOT00000033187	0.966927181	2.95E-07
TUCP_003984	XLOC_174788	ENSRNOT00000036506	0.970626425	1.64E-07
TUCP_003984	XLOC_174788	ENSRNOT00000005958	0.95069767	2.11E-06
TUCP_003984	XLOC_174788	ENSRNOT00000006188	-0.968737669	2.23E-07
TUCP_003984	XLOC_174788	ENSRNOT00000075656	-0.971336883	1.45E-07

TUCP_003984	XLOC_174788	ENSRNOT00000013745	-0.977042186	4.83E-08
TUCP_003984	XLOC_174788	ENSRNOT00000018500	-0.951767397	1.90E-06
TUCP_003984	XLOC_174788	ENSRNOT00000075397	0.969278029	2.05E-07
TUCP_003984	XLOC_174788	ENSRNOT00000012617	-0.962631757	5.39E-07
TUCP_003984	XLOC_174788	ENSRNOT00000032926	-0.97121766	1.48E-07
TUCP_003984	XLOC_174788	ENSRNOT00000019104	-0.955405821	1.29E-06
TUCP_003984	XLOC_174788	ENSRNOT00000026279	-0.960912506	6.73E-07
TUCP_003984	XLOC_174788	ENSRNOT00000063930	-0.966284382	3.24E-07
TUCP_003984	XLOC_174788	ENSRNOT00000000181	0.953466488	1.59E-06
TUCP_003984	XLOC_174788	ENSRNOT00000090499	-0.956388635	1.15E-06
TUCP_003984	XLOC_174788	ENSRNOT00000046215	-0.96450029	4.18E-07
TUCP_003984	XLOC_174788	ENSRNOT00000087604	0.957965636	9.63E-07
TUCP_003984	XLOC_174788	ENSRNOT00000073492	-0.968123232	2.46E-07
TUCP_003984	XLOC_174788	ENSRNOT00000064013	-0.950809481	2.09E-06
TUCP_003984	XLOC_174788	ENSRNOT00000001855	0.96444912	4.21E-07
TUCP_003984	XLOC_174788	ENSRNOT00000023638	0.960657242	6.95E-07
TUCP_003984	XLOC_174788	ENSRNOT00000001691	0.980569993	2.11E-08
TUCP_003984	XLOC_174788	ENSRNOT00000089319	0.971607221	1.39E-07
TUCP_003984	XLOC_174788	ENSRNOT00000007169	-0.951117004	2.02E-06
TUCP_003984	XLOC_174788	ENSRNOT00000000083	-0.954206078	1.47E-06
TUCP_003984	XLOC_174788	ENSRNOT00000086706	0.957214721	1.05E-06
TUCP_003984	XLOC_174788	ENSRNOT00000088555	-0.954772048	1.38E-06
TUCP_003984	XLOC_174788	ENSRNOT00000045233	-0.952674503	1.73E-06
TUCP_003984	XLOC_174788	ENSRNOT00000074023	0.981251122	1.77E-08
TUCP_003984	XLOC_174788	ENSRNOT00000013852	0.950584135	2.14E-06
TUCP_003984	XLOC_174788	ENSRNOT00000013851	-0.970612353	1.64E-07
TUCP_003984	XLOC_174788	ENSRNOT00000089418	0.955342095	1.30E-06
TUCP_003984	XLOC_174788	ENSRNOT00000024710	-0.958191574	9.38E-07
TUCP_003984	XLOC_174788	ENSRNOT00000055321	0.963176776	5.01E-07
TUCP_003984	XLOC_174788	ENSRNOT00000055540	0.95028203	2.20E-06
TUCP_003984	XLOC_174788	ENSRNOT00000045926	0.954883626	1.36E-06
TUCP_003984	XLOC_174788	ENSRNOT00000076105	-0.966054632	3.35E-07
TUCP_003984	XLOC_174788	ENSRNOT00000028703	-0.952594787	1.74E-06
TUCP_003984	XLOC_174788	ENSRNOT00000021534	-0.95082483	2.08E-06
TUCP_003984	XLOC_174788	ENSRNOT00000013516	0.950879443	2.07E-06
TUCP_003984	XLOC_174788	ENSRNOT00000003326	-0.968422386	2.35E-07
TUCP_003984	XLOC_174788	ENSRNOT00000022746	-0.969104639	2.10E-07
TUCP_003984	XLOC_174788	ENSRNOT00000004810	0.952466405	1.76E-06
TUCP_003984	XLOC_174788	ENSRNOT00000016306	-0.956982857	1.08E-06
TUCP_003984	XLOC_174788	ENSRNOT00000083677	-0.955318969	1.30E-06
TUCP_003984	XLOC_174788	ENSRNOT00000080677	-0.955114399	1.33E-06
TUCP_003984	XLOC_174788	ENSRNOT00000065304	-0.954457689	1.43E-06
TUCP_003984	XLOC_174788	ENSRNOT00000027301	-0.96975974	1.89E-07
TUCP_003984	XLOC_174788	ENSRNOT00000073370	0.951851732	1.88E-06
TUCP_003984	XLOC_174788	ENSRNOT00000004722	-0.959729899	7.79E-07
TUCP_003984	XLOC_174788	ENSRNOT00000007350	0.952148011	1.82E-06
TUCP_003984	XLOC_174788	ENSRNOT00000030373	-0.971205724	1.49E-07
TUCP_003984	XLOC_174788	ENSRNOT00000065978	0.960556899	7.04E-07
TUCP_003984	XLOC_174788	ENSRNOT00000075480	-0.970959194	1.55E-07
TUCP_003984	XLOC_174788	ENSRNOT00000012699	-0.952526239	1.75E-06
TUCP_003984	XLOC_174788	ENSRNOT00000020051	0.956024683	1.20E-06

TUCP_003984	XLOC_174788	ENSRNOT00000027760	0.963383691	4.87E-07
TUCP_003984	XLOC_174788	ENSRNOT00000083891	-0.966656235	3.07E-07
TUCP_003984	XLOC_174788	ENSRNOT00000042903	0.98584755	4.37E-09
TUCP_003984	XLOC_174788	ENSRNOT00000057898	-0.972770346	1.13E-07
TUCP_003984	XLOC_174788	ENSRNOT00000089933	0.968983181	2.15E-07
TUCP_003984	XLOC_174788	ENSRNOT00000083876	-0.961773493	6.03E-07
TUCP_003984	XLOC_174788	ENSRNOT00000009169	0.95145747	1.96E-06
TUCP_003984	XLOC_174788	ENSRNOT00000056100	-0.977305138	4.56E-08
TUCP_003984	XLOC_174788	ENSRNOT00000000477	-0.965485493	3.64E-07
TUCP_003984	XLOC_174788	ENSRNOT00000083351	-0.956574465	1.13E-06
TUCP_003984	XLOC_174788	ENSRNOT00000009817	-0.952988801	1.67E-06
TUCP_003984	XLOC_174788	ENSRNOT00000067554	0.951284756	1.99E-06
TUCP_003984	XLOC_174788	ENSRNOT00000055683	0.95160009	1.93E-06
TUCP_003984	XLOC_174788	ENSRNOT00000022000	-0.968349266	2.37E-07
TUCP_003984	XLOC_174788	ENSRNOT00000030798	-0.970225744	1.75E-07
TUCP_003984	XLOC_174788	ENSRNOT00000005834	0.966206295	3.28E-07
TUCP_003984	XLOC_174788	ENSRNOT00000005297	0.961263265	6.44E-07
TUCP_003984	XLOC_174788	ENSRNOT00000000048	0.972001926	1.29E-07
TUCP_003984	XLOC_174788	ENSRNOT00000088133	-0.957448731	1.02E-06
TUCP_003984	XLOC_174788	ENSRNOT00000031701	-0.952444442	1.77E-06
TUCP_003984	XLOC_174788	ENSRNOT00000075934	-0.963973425	4.50E-07
TUCP_003984	XLOC_174788	ENSRNOT00000016629	0.950769259	2.10E-06
TUCP_003984	XLOC_174788	ENSRNOT00000076181	-0.964553591	4.15E-07
TUCP_003984	XLOC_174788	ENSRNOT00000072849	-0.960211433	7.35E-07
TUCP_003984	XLOC_174788	ENSRNOT00000073643	-0.965486556	3.64E-07
TUCP_003984	XLOC_174788	ENSRNOT00000016254	-0.958128853	9.45E-07
TUCP_003984	XLOC_174788	ENSRNOT00000078349	-0.962806529	5.27E-07
TUCP_003984	XLOC_174788	ENSRNOT00000003940	-0.976049305	5.96E-08
TUCP_003984	XLOC_174788	ENSRNOT00000005620	-0.952347234	1.79E-06
TUCP_003984	XLOC_174788	ENSRNOT00000022502	-0.955785695	1.24E-06
TUCP_003984	XLOC_174788	ENSRNOT00000082914	0.954087406	1.49E-06
TUCP_003984	XLOC_174788	ENSRNOT00000004787	-0.954048628	1.49E-06
TUCP_003984	XLOC_174788	ENSRNOT00000037133	0.962111564	5.77E-07
TUCP_003984	XLOC_174788	ENSRNOT00000078816	0.967777922	2.59E-07
TUCP_003984	XLOC_174788	ENSRNOT00000017949	-0.952618972	1.74E-06
TUCP_003984	XLOC_174788	ENSRNOT00000026098	-0.952161877	1.82E-06
TUCP_003984	XLOC_174788	ENSRNOT00000083964	-0.968650622	2.26E-07
TUCP_003984	XLOC_174788	ENSRNOT00000090912	0.971495047	1.41E-07
TUCP_003984	XLOC_174788	ENSRNOT00000048781	0.961510364	6.24E-07
TUCP_003984	XLOC_174788	ENSRNOT00000013867	0.953163249	1.64E-06
TUCP_003984	XLOC_174788	ENSRNOT00000021802	0.952777686	1.71E-06
TUCP_003984	XLOC_174788	ENSRNOT00000091909	0.958459648	9.08E-07
TUCP_003984	XLOC_174788	ENSRNOT00000026831	0.955735568	1.24E-06
TUCP_003984	XLOC_174788	ENSRNOT00000009563	-0.968105075	2.46E-07
TUCP_003984	XLOC_174788	ENSRNOT00000074985	-0.955636358	1.26E-06
TUCP_003984	XLOC_174788	ENSRNOT00000025856	-0.967392833	2.75E-07
TUCP_003984	XLOC_174788	ENSRNOT00000004351	-0.957809971	9.81E-07
TUCP_003984	XLOC_174788	ENSRNOT00000003544	-0.95239397	1.78E-06
TUCP_003984	XLOC_174788	ENSRNOT00000021925	0.950072451	2.25E-06
TUCP_003984	XLOC_174788	ENSRNOT00000003780	0.951997953	1.85E-06
TUCP_003984	XLOC_174788	ENSRNOT00000001403	0.958816015	8.71E-07

TUCP_003984	XLOC_174788	ENSRNOT00000064452	-0.971740633	1.35E-07
TUCP_003984	XLOC_174788	ENSRNOT00000014506	-0.954344727	1.45E-06
TUCP_003984	XLOC_174788	ENSRNOT00000023262	-0.962337575	5.60E-07
TUCP_003984	XLOC_174788	ENSRNOT00000025913	-0.959950311	7.59E-07
TUCP_003984	XLOC_174788	ENSRNOT00000013067	-0.959032848	8.48E-07
TUCP_003984	XLOC_174788	ENSRNOT00000066952	0.966622092	3.08E-07
TUCP_003984	XLOC_174788	ENSRNOT00000078185	-0.951062152	2.04E-06
TUCP_003984	XLOC_174788	ENSRNOT00000024119	0.961292318	6.41E-07
TUCP_003984	XLOC_174788	ENSRNOT00000021636	-0.951386985	1.97E-06
TUCP_003984	XLOC_174788	ENSRNOT00000016220	-0.95086651	2.08E-06
TUCP_003984	XLOC_174788	ENSRNOT00000045946	0.971589283	1.39E-07
TUCP_003984	XLOC_174788	ENSRNOT00000086577	0.963801272	4.61E-07
TUCP_003984	XLOC_174788	ENSRNOT00000036658	-0.96757108	2.67E-07
TUCP_003984	XLOC_174788	ENSRNOT00000032363	0.972237122	1.24E-07
TUCP_003984	XLOC_174788	ENSRNOT00000055957	-0.951829175	1.88E-06
TUCP_003984	XLOC_174788	ENSRNOT00000032667	-0.960558521	7.03E-07
TUCP_003984	XLOC_174788	ENSRNOT00000065600	0.963132697	5.04E-07
TUCP_003984	XLOC_174788	ENSRNOT00000086260	-0.953016418	1.67E-06
TUCP_003984	XLOC_174788	ENSRNOT00000058351	0.951094459	2.03E-06
TUCP_003984	XLOC_174788	ENSRNOT00000049310	-0.953669808	1.56E-06
TUCP_003984	XLOC_174788	ENSRNOT00000041815	-0.967615169	2.66E-07
TUCP_003984	XLOC_174788	ENSRNOT00000033975	0.956573209	1.13E-06
TUCP_003984	XLOC_174788	ENSRNOT00000068026	-0.955146871	1.33E-06
TUCP_003984	XLOC_174788	ENSRNOT00000044608	-0.956813195	1.10E-06
TUCP_003984	XLOC_174788	ENSRNOT00000001099	-0.966305758	3.23E-07
TUCP_003984	XLOC_174788	ENSRNOT00000014821	-0.981349249	1.72E-08
TUCP_003984	XLOC_174788	ENSRNOT00000034883	-0.952134496	1.83E-06
TUCP_003984	XLOC_174788	ENSRNOT00000045765	-0.950351186	2.19E-06
TUCP_003984	XLOC_174788	ENSRNOT00000005459	0.952777212	1.71E-06
TUCP_003984	XLOC_174788	ENSRNOT00000088599	0.957443863	1.02E-06
TUCP_003984	XLOC_174788	ENSRNOT00000022190	-0.954400772	1.44E-06
TUCP_003984	XLOC_174788	ENSRNOT00000026531	-0.96303458	5.11E-07
TUCP_003984	XLOC_174788	ENSRNOT00000035605	0.957304237	1.04E-06
TUCP_003984	XLOC_174788	ENSRNOT00000050119	-0.970363843	1.71E-07
TUCP_003984	XLOC_174788	ENSRNOT00000084031	0.964283764	4.31E-07
TUCP_003984	XLOC_174788	ENSRNOT00000059181	0.960770615	6.85E-07
TUCP_003984	XLOC_174788	ENSRNOT00000009749	-0.956444571	1.15E-06
TUCP_003984	XLOC_174788	ENSRNOT00000016568	0.958187357	9.38E-07
TUCP_003984	XLOC_174788	ENSRNOT00000005910	-0.954752199	1.38E-06
TUCP_003984	XLOC_174788	ENSRNOT00000028290	-0.960352485	7.22E-07
TUCP_003984	XLOC_174788	ENSRNOT00000003412	-0.961254818	6.44E-07
TUCP_003984	XLOC_174788	ENSRNOT00000050609	-0.962570185	5.43E-07
TUCP_003984	XLOC_174788	ENSRNOT00000089211	0.952453419	1.77E-06
TUCP_003984	XLOC_174788	ENSRNOT00000050515	0.955556618	1.27E-06
TUCP_003984	XLOC_174788	ENSRNOT00000060595	-0.950479759	2.16E-06
TUCP_003984	XLOC_174788	ENSRNOT00000029701	0.965334476	3.72E-07
TUCP_003984	XLOC_174788	ENSRNOT00000006765	-0.962033978	5.83E-07
TUCP_003984	XLOC_174788	ENSRNOT00000073847	-0.955868727	1.22E-06
TUCP_003984	XLOC_174788	ENSRNOT00000015518	-0.950944635	2.06E-06
TUCP_003984	XLOC_174788	ENSRNOT00000024947	-0.960061275	7.48E-07
TUCP_003984	XLOC_174788	ENSRNOT00000011401	-0.962720598	5.33E-07

TUCP_003984	XLOC_174788	ENSRNOT00000055602	-0.965935535	3.41E-07
TUCP_003984	XLOC_174788	ENSRNOT00000037015	0.959407353	8.11E-07
TUCP_003984	XLOC_174788	ENSRNOT00000067135	-0.974508178	8.12E-08
TUCP_003984	XLOC_174788	ENSRNOT00000011563	-0.964112519	4.41E-07
TUCP_003984	XLOC_174788	ENSRNOT00000054976	-0.972743602	1.13E-07
TUCP_003984	XLOC_174788	ENSRNOT00000010516	-0.957020004	1.07E-06
TUCP_003984	XLOC_174788	ENSRNOT00000023876	0.960075455	7.47E-07
TUCP_003984	XLOC_174788	ENSRNOT00000002187	-0.960599253	7.00E-07
TUCP_003984	XLOC_174788	ENSRNOT00000025794	-0.961050361	6.61E-07
TUCP_003984	XLOC_174788	ENSRNOT00000011130	-0.953887344	1.52E-06
TUCP_003984	XLOC_174788	ENSRNOT00000039730	-0.957450256	1.02E-06
TUCP_003984	XLOC_174788	ENSRNOT00000083632	-0.970295951	1.73E-07
TUCP_003984	XLOC_174788	ENSRNOT00000030329	-0.964861675	3.98E-07
TUCP_003984	XLOC_174788	ENSRNOT00000087748	-0.967407034	2.74E-07
TUCP_003984	XLOC_174788	ENSRNOT00000003568	0.950920991	2.06E-06
TUCP_003984	XLOC_174788	ENSRNOT00000006366	-0.950469007	2.16E-06
TUCP_003984	XLOC_174788	ENSRNOT00000074649	0.953664869	1.56E-06
TUCP_003984	XLOC_174788	ENSRNOT00000016132	-0.961707276	6.08E-07
TUCP_003984	XLOC_174788	ENSRNOT00000067780	-0.953534128	1.58E-06
TUCP_003984	XLOC_174788	ENSRNOT00000077146	-0.970100126	1.79E-07
TUCP_003984	XLOC_174788	ENSRNOT00000026235	-0.958668825	8.86E-07
TUCP_003984	XLOC_174788	ENSRNOT00000023872	-0.97182302	1.33E-07
TUCP_003984	XLOC_174788	ENSRNOT00000010753	-0.952562355	1.75E-06
TUCP_003984	XLOC_174788	ENSRNOT00000004999	0.96423735	4.34E-07
TUCP_003984	XLOC_174788	ENSRNOT00000026312	0.952223537	1.81E-06
TUCP_003984	XLOC_174788	ENSRNOT00000013463	0.983436784	9.55E-09
TUCP_003984	XLOC_174788	ENSRNOT00000060002	-0.962748645	5.31E-07
TUCP_003984	XLOC_174788	ENSRNOT00000089673	-0.974679101	7.86E-08
TUCP_003984	XLOC_174788	ENSRNOT00000023763	-0.963692317	4.67E-07
TUCP_003984	XLOC_174788	ENSRNOT00000019465	-0.956792005	1.10E-06
TUCP_003984	XLOC_174788	ENSRNOT00000057738	0.965371029	3.70E-07
TUCP_003984	XLOC_174788	ENSRNOT00000058382	0.951126982	2.02E-06
TUCP_003984	XLOC_174788	ENSRNOT00000000263	0.967890433	2.55E-07
TUCP_003984	XLOC_174788	ENSRNOT00000007889	-0.95514153	1.33E-06
TUCP_003984	XLOC_174788	ENSRNOT00000023977	-0.964140305	4.40E-07
TUCP_003984	XLOC_174788	ENSRNOT00000009202	0.962093477	5.78E-07
TUCP_003984	XLOC_174788	ENSRNOT00000047663	-0.975426621	6.77E-08
TUCP_003984	XLOC_174788	ENSRNOT00000082151	-0.960014794	7.53E-07
TUCP_003984	XLOC_174788	ENSRNOT00000020272	-0.959542635	7.97E-07
TUCP_003984	XLOC_174788	ENSRNOT00000066133	-0.959955614	7.58E-07
TUCP_003984	XLOC_174788	ENSRNOT00000017420	-0.952766197	1.71E-06
TUCP_003984	XLOC_174788	ENSRNOT00000034411	0.952529328	1.75E-06
TUCP_003984	XLOC_174788	ENSRNOT00000001607	0.952930451	1.68E-06
TUCP_003984	XLOC_174788	ENSRNOT00000029202	-0.955518095	1.27E-06
TUCP_003984	XLOC_174788	ENSRNOT00000017291	-0.959811508	7.72E-07
TUCP_003984	XLOC_174788	ENSRNOT00000017907	-0.966827993	2.99E-07
TUCP_003984	XLOC_174788	ENSRNOT00000003691	-0.967982863	2.51E-07
TUCP_003984	XLOC_174788	ENSRNOT00000045532	-0.960530391	7.06E-07
TUCP_003984	XLOC_174788	ENSRNOT00000035385	0.958133586	9.44E-07
TUCP_003984	XLOC_174788	ENSRNOT00000000532	-0.954805282	1.38E-06
TUCP_003984	XLOC_174788	ENSRNOT00000002443	0.960427424	7.15E-07

TUCP_003984	XLOC_174788	ENSRNOT00000014924	-0.976040222	5.97E-08
TUCP_003984	XLOC_174788	ENSRNOT00000007738	-0.952965788	1.67E-06
TUCP_003984	XLOC_174788	ENSRNOT00000022144	-0.957161326	1.06E-06
TUCP_003984	XLOC_174788	ENSRNOT00000030692	-0.9606292	6.97E-07
TUCP_003984	XLOC_174788	ENSRNOT00000057537	0.95395908	1.51E-06
TUCP_003984	XLOC_174788	ENSRNOT00000007771	-0.958714864	8.81E-07
TUCP_003984	XLOC_174788	ENSRNOT00000002114	-0.980068392	2.40E-08
TUCP_003984	XLOC_174788	ENSRNOT00000022142	0.950721579	2.11E-06
TUCP_003984	XLOC_174788	ENSRNOT00000080608	0.965367018	3.70E-07
TUCP_003984	XLOC_174788	ENSRNOT00000027970	0.969474421	1.98E-07
TUCP_003984	XLOC_174788	ENSRNOT00000018634	-0.95430771	1.45E-06
TUCP_003984	XLOC_174788	ENSRNOT00000085971	-0.977226971	4.64E-08
TUCP_003984	XLOC_174788	ENSRNOT00000085295	-0.9644804	4.19E-07
TUCP_003984	XLOC_174788	ENSRNOT00000015537	0.951640741	1.92E-06
TUCP_003984	XLOC_174788	ENSRNOT00000008222	-0.965402989	3.68E-07
TUCP_003984	XLOC_174788	ENSRNOT00000023664	-0.951661545	1.92E-06
TUCP_003984	XLOC_174788	ENSRNOT00000016445	0.953070001	1.66E-06
TUCP_003984	XLOC_174788	ENSRNOT00000039341	0.951204416	2.01E-06
TUCP_003984	XLOC_174788	ENSRNOT00000036838	-0.977930857	3.97E-08
TUCP_003984	XLOC_174788	ENSRNOT00000039102	0.968851262	2.19E-07
TUCP_003984	XLOC_174788	ENSRNOT00000082138	-0.961224577	6.47E-07
TUCP_003984	XLOC_174788	ENSRNOT00000023807	-0.968475708	2.33E-07
TUCP_003984	XLOC_174788	ENSRNOT00000078856	-0.961326058	6.38E-07
TUCP_003984	XLOC_174788	ENSRNOT00000023965	-0.972408327	1.20E-07
TUCP_003984	XLOC_174788	ENSRNOT00000013933	-0.950150475	2.23E-06
TUCP_003984	XLOC_174788	ENSRNOT00000033048	-0.969311941	2.04E-07
TUCP_003984	XLOC_174788	ENSRNOT00000000120	0.983183998	1.03E-08
TUCP_003984	XLOC_174788	ENSRNOT00000019983	-0.952768658	1.71E-06
TUCP_003984	XLOC_174788	ENSRNOT00000003630	-0.956524036	1.14E-06
TUCP_003984	XLOC_174788	ENSRNOT00000010983	0.955478229	1.28E-06
TUCP_003984	XLOC_174788	ENSRNOT00000007369	0.962629332	5.39E-07
TUCP_003984	XLOC_174788	ENSRNOT00000042125	0.963054693	5.09E-07
TUCP_003984	XLOC_174788	ENSRNOT00000085466	0.951357299	1.98E-06
TUCP_003984	XLOC_174788	ENSRNOT00000028545	-0.964287417	4.31E-07
TUCP_003984	XLOC_174788	ENSRNOT00000060698	-0.957428849	1.03E-06
TUCP_003984	XLOC_174788	ENSRNOT00000001759	-0.965267422	3.76E-07
TUCP_003984	XLOC_174788	ENSRNOT00000035808	0.956921584	1.09E-06
TUCP_003984	XLOC_174788	ENSRNOT00000028225	0.95929091	8.22E-07
TUCP_003984	XLOC_174788	ENSRNOT00000026760	-0.96696694	2.93E-07
TUCP_003984	XLOC_174788	ENSRNOT00000075800	-0.967336013	2.77E-07
TUCP_003984	XLOC_174788	ENSRNOT00000082709	-0.978148781	3.78E-08
TUCP_003984	XLOC_174788	ENSRNOT00000015624	0.955135452	1.33E-06
TUCP_003984	XLOC_174788	ENSRNOT00000004319	-0.959210287	8.30E-07
TUCP_003984	XLOC_174788	ENSRNOT00000063821	-0.951198175	2.01E-06
TUCP_003984	XLOC_174788	ENSRNOT00000032248	0.952296672	1.80E-06
TUCP_003984	XLOC_174788	ENSRNOT00000019911	-0.959102149	8.41E-07
TUCP_003984	XLOC_174788	ENSRNOT00000013431	-0.956606638	1.13E-06
TUCP_003984	XLOC_174788	ENSRNOT00000015318	-0.961707102	6.08E-07
TUCP_003984	XLOC_174788	ENSRNOT00000032236	-0.950235478	2.21E-06
TUCP_003984	XLOC_174788	ENSRNOT00000016372	-0.958087408	9.49E-07
TUCP_003984	XLOC_174788	ENSRNOT00000005885	-0.968855873	2.19E-07

TUCP_003984	XLOC_174788	ENSRNOT00000051494	0.956018524	1.20E-06
TUCP_003984	XLOC_174788	ENSRNOT00000080410	-0.968687413	2.25E-07
TUCP_003984	XLOC_174788	ENSRNOT00000027967	0.956411308	1.15E-06
TUCP_003984	XLOC_174788	ENSRNOT00000049285	-0.966633185	3.08E-07
TUCP_003984	XLOC_174788	ENSRNOT00000019109	-0.955486173	1.28E-06
TUCP_003984	XLOC_174788	ENSRNOT00000012286	-0.980121915	2.36E-08
TUCP_003984	XLOC_174788	ENSRNOT00000037039	-0.965072651	3.86E-07
TUCP_003984	XLOC_174788	ENSRNOT00000090057	0.951746099	1.90E-06
TUCP_003984	XLOC_174788	ENSRNOT00000036613	-0.957358886	1.03E-06
TUCP_003984	XLOC_174788	ENSRNOT00000008987	-0.969507855	1.97E-07
TUCP_003984	XLOC_174788	ENSRNOT00000025705	0.965717926	3.52E-07
TUCP_003984	XLOC_174788	ENSRNOT00000054685	0.952457628	1.77E-06
TUCP_003984	XLOC_174788	ENSRNOT00000090355	-0.977060839	4.81E-08
TUCP_003984	XLOC_174788	ENSRNOT00000002445	-0.968686964	2.25E-07
TUCP_003984	XLOC_174788	ENSRNOT00000038212	-0.968825453	2.20E-07
TUCP_003984	XLOC_174788	ENSRNOT00000005257	0.961976106	5.87E-07
TUCP_003984	XLOC_174788	ENSRNOT00000085621	0.960322473	7.24E-07
TUCP_003984	XLOC_174788	ENSRNOT00000008113	-0.959361499	8.15E-07
TUCP_003984	XLOC_174788	ENSRNOT00000000334	-0.951007448	2.05E-06
TUCP_000943	XLOC_034904	ENSRNOT00000082585	0.958922807	8.60E-07
TUCP_003987	XLOC_175146	ENSRNOT00000044855	0.98435436	7.19E-09
TUCP_003589	XLOC_157709	ENSRNOT00000084500	0.976499889	5.43E-08
TUCP_003589	XLOC_157709	ENSRNOT00000093467	0.985463696	4.99E-09
TUCP_003589	XLOC_157709	ENSRNOT00000029340	0.991413351	3.62E-10
TUCP_003589	XLOC_157709	ENSRNOT00000077972	0.964601361	4.12E-07
TUCP_003589	XLOC_157709	ENSRNOT00000038646	0.993487726	9.12E-11
TUCP_003589	XLOC_157709	ENSRNOT00000017909	0.966731654	3.04E-07
TUCP_003589	XLOC_157709	ENSRNOT00000085479	0.96556545	3.60E-07
TUCP_003589	XLOC_157709	ENSRNOT00000092579	0.993487726	9.12E-11
TUCP_003589	XLOC_157709	ENSRNOT00000048373	0.955511868	1.27E-06
TUCP_003589	XLOC_157709	ENSRNOT00000036383	0.978884088	3.19E-08
TUCP_003589	XLOC_157709	ENSRNOT00000076528	0.998956472	9.73E-15
TUCP_003589	XLOC_157709	ENSRNOT00000077458	0.959164195	8.35E-07
TUCP_003589	XLOC_157709	ENSRNOT00000083034	0.992447229	1.91E-10
TUCP_003589	XLOC_157709	ENSRNOT00000089607	0.999177615	2.96E-15
TUCP_003589	XLOC_157709	ENSRNOT00000035915	0.956960879	1.08E-06
TUCP_003589	XLOC_157709	ENSRNOT00000052315	0.975231412	7.04E-08
TUCP_003589	XLOC_157709	ENSRNOT00000075193	0.972029351	1.29E-07
TUCP_003589	XLOC_157709	ENSRNOT00000088168	0.993487726	9.12E-11
TUCP_003589	XLOC_157709	ENSRNOT00000046231	0.952887301	1.69E-06
TUCP_003589	XLOC_157709	ENSRNOT00000013459	0.970404465	1.70E-07
TUCP_003589	XLOC_157709	ENSRNOT00000078137	0.993487726	9.12E-11
TUCP_003589	XLOC_157709	ENSRNOT00000077764	0.977957872	3.95E-08
TUCP_003589	XLOC_157709	ENSRNOT00000043661	0.957846633	9.76E-07
TUCP_003589	XLOC_157709	ENSRNOT00000092740	0.993487726	9.12E-11
TUCP_003589	XLOC_157709	ENSRNOT00000000117	0.983009457	1.08E-08
TUCP_003589	XLOC_157709	ENSRNOT00000071877	0.953927246	1.51E-06
TUCP_003589	XLOC_157709	ENSRNOT00000070958	0.993487726	9.12E-11
TUCP_003589	XLOC_157709	ENSRNOT00000076757	0.965734772	3.51E-07
TUCP_003589	XLOC_157709	ENSRNOT00000085310	0.956205971	1.18E-06
TUCP_003589	XLOC_157709	ENSRNOT00000047241	0.957361246	1.03E-06

TUCP_003589	XLOC_157709	ENSRNOT00000027268	0.993487726	9.12E-11
TUCP_003589	XLOC_157709	ENSRNOT00000004459	0.993487726	9.12E-11
TUCP_003589	XLOC_157709	ENSRNOT00000089235	0.990510426	5.96E-10
TUCP_003589	XLOC_157709	ENSRNOT00000061516	0.984422162	7.04E-09
TUCP_003589	XLOC_157709	ENSRNOT00000088148	0.993487726	9.12E-11
TUCP_003589	XLOC_157709	ENSRNOT00000088527	0.993487726	9.12E-11
TUCP_003589	XLOC_157709	ENSRNOT00000078701	0.993487726	9.12E-11
TUCP_003589	XLOC_157709	ENSRNOT00000052308	0.953292063	1.62E-06
TUCP_003589	XLOC_157709	ENSRNOT00000021748	0.993487726	9.12E-11
TUCP_003589	XLOC_157709	ENSRNOT00000089085	0.993487726	9.12E-11
TUCP_003589	XLOC_157709	ENSRNOT00000008153	0.994776859	3.03E-11
TUCP_003589	XLOC_157709	ENSRNOT00000085896	0.966698338	3.05E-07
TUCP_003589	XLOC_157709	ENSRNOT00000079189	0.973656349	9.56E-08
TUCP_003589	XLOC_157709	ENSRNOT00000090078	0.993487726	9.12E-11
TUCP_003589	XLOC_157709	ENSRNOT00000078020	0.960395871	7.18E-07
TUCP_003589	XLOC_157709	ENSRNOT00000009776	0.993487726	9.12E-11
TUCP_003232	XLOC_138424	ENSRNOT00000007761	0.952715212	1.72E-06
TUCP_003232	XLOC_138424	ENSRNOT00000031083	0.969131158	2.10E-07
TUCP_003232	XLOC_138424	ENSRNOT00000038188	0.957175777	1.06E-06
TUCP_003232	XLOC_138424	ENSRNOT00000064942	-0.950492286	2.15E-06
TUCP_003232	XLOC_138424	ENSRNOT00000090780	0.975263099	7.00E-08
TUCP_003889	XLOC_170989	ENSRNOT00000073970	-0.962289326	5.64E-07
TUCP_001907	XLOC_078874	ENSRNOT00000085042	-0.964117646	4.41E-07
TUCP_001907	XLOC_078874	ENSRNOT00000047522	-0.972417455	1.20E-07
TUCP_003128	XLOC_133580	ENSRNOT00000026966	0.970322306	1.73E-07
TUCP_003128	XLOC_133580	ENSRNOT00000068416	0.999999782	3.88E-33
TUCP_003128	XLOC_133580	ENSRNOT00000086036	0.999958274	9.96E-22
TUCP_003128	XLOC_133580	ENSRNOT00000026803	0.985046075	5.74E-09
TUCP_003128	XLOC_133580	ENSRNOT00000047527	0.963071698	5.08E-07
TUCP_003128	XLOC_133580	ENSRNOT00000042251	0.999344295	9.53E-16
TUCP_003128	XLOC_133580	ENSRNOT00000002984	0.998397069	8.31E-14
TUCP_003128	XLOC_133580	ENSRNOT00000083764	0.999541808	1.59E-16
TUCP_003128	XLOC_133580	ENSRNOT00000076802	0.962695843	5.34E-07
TUCP_003128	XLOC_133580	ENSRNOT00000092365	0.999161651	3.26E-15
TUCP_003128	XLOC_133580	ENSRNOT00000079275	0.999901911	7.15E-20
TUCP_003128	XLOC_133580	ENSRNOT00000066062	-0.959706712	7.82E-07
TUCP_003128	XLOC_133580	ENSRNOT00000045301	1	0
TUCP_003128	XLOC_133580	ENSRNOT00000041186	0.999975782	6.56E-23
TUCP_003128	XLOC_133580	ENSRNOT00000010092	0.977711778	4.17E-08
TUCP_003128	XLOC_133580	ENSRNOT00000076182	1	1.33E-79
TUCP_003128	XLOC_133580	ENSRNOT00000087404	0.951157664	2.02E-06
TUCP_003128	XLOC_133580	ENSRNOT00000052003	0.95837331	9.18E-07
TUCP_003128	XLOC_133580	ENSRNOT00000081709	0.998485154	6.27E-14
TUCP_003128	XLOC_133580	ENSRNOT00000005726	0.959610119	7.91E-07
TUCP_003128	XLOC_133580	ENSRNOT00000014103	1	4.25E-78
TUCP_003128	XLOC_133580	ENSRNOT00000057433	0.964930601	3.94E-07
TUCP_003128	XLOC_133580	ENSRNOT00000082289	0.998931566	1.09E-14
TUCP_003128	XLOC_133580	ENSRNOT00000092516	0.957459323	1.02E-06
TUCP_003128	XLOC_133580	ENSRNOT00000008898	0.990039768	7.59E-10
TUCP_003128	XLOC_133580	ENSRNOT00000083493	0.995461232	1.51E-11
TUCP_003128	XLOC_133580	ENSRNOT00000092563	0.987201084	2.65E-09

TUCP_003128	XLOC_133580	ENSRNOT00000088893	1	0
TUCP_003128	XLOC_133580	ENSRNOT00000076693	0.955029582	1.34E-06
TUCP_003128	XLOC_133580	ENSRNOT00000081014	1	1.33E-79
TUCP_003128	XLOC_133580	ENSRNOT00000067543	1	1.33E-79
TUCP_003128	XLOC_133580	ENSRNOT00000050968	0.996816433	2.56E-12
TUCP_003128	XLOC_133580	ENSRNOT00000042201	0.960235595	7.32E-07
TUCP_003128	XLOC_133580	ENSRNOT00000004373	0.957315473	1.04E-06
TUCP_003128	XLOC_133580	ENSRNOT00000063929	1	0
TUCP_003128	XLOC_133580	ENSRNOT00000057472	1	0
TUCP_003128	XLOC_133580	ENSRNOT00000041617	0.952449765	1.77E-06
TUCP_003128	XLOC_133580	ENSRNOT00000091763	0.986310385	3.70E-09
TUCP_003128	XLOC_133580	ENSRNOT00000022200	0.998491303	6.14E-14
TUCP_003128	XLOC_133580	ENSRNOT00000067379	0.997260967	1.21E-12
TUCP_003128	XLOC_133580	ENSRNOT00000020786	0.976428448	5.51E-08
TUCP_003128	XLOC_133580	ENSRNOT00000050543	0.999722223	1.30E-17
TUCP_003128	XLOC_133580	ENSRNOT00000089306	0.999866649	3.32E-19
TUCP_003128	XLOC_133580	ENSRNOT00000052367	0.997268394	1.19E-12
TUCP_003128	XLOC_133580	ENSRNOT00000090658	1	0
TUCP_003128	XLOC_133580	ENSRNOT00000022571	0.995290013	1.81E-11
TUCP_003128	XLOC_133580	ENSRNOT00000044520	0.981817158	1.52E-08
TUCP_003128	XLOC_133580	ENSRNOT00000065448	0.963648868	4.70E-07
TUCP_003093	XLOC_132420	ENSRNOT00000036565	0.975672313	6.44E-08
TUCP_003093	XLOC_132420	ENSRNOT00000093075	0.969097172	2.11E-07
TUCP_003093	XLOC_132420	ENSRNOT00000058260	0.969097172	2.11E-07
TUCP_003093	XLOC_132420	ENSRNOT00000088887	0.969097172	2.11E-07
TUCP_003093	XLOC_132420	ENSRNOT00000084986	0.969097172	2.11E-07
TUCP_003093	XLOC_132420	ENSRNOT00000072573	0.972592452	1.16E-07
TUCP_003093	XLOC_132420	ENSRNOT00000017828	0.961785641	6.02E-07
TUCP_003093	XLOC_132420	ENSRNOT00000071312	0.969097172	2.11E-07
TUCP_003093	XLOC_132420	ENSRNOT00000088245	0.969097172	2.11E-07
TUCP_003093	XLOC_132420	ENSRNOT00000058833	0.965830464	3.46E-07
TUCP_003093	XLOC_132420	ENSRNOT00000078052	0.969097172	2.11E-07
TUCP_003093	XLOC_132420	ENSRNOT00000078494	0.968561995	2.29E-07
TUCP_003093	XLOC_132420	ENSRNOT00000085653	0.969097172	2.11E-07
TUCP_003093	XLOC_132420	ENSRNOT00000081992	0.969433458	2.00E-07
TUCP_003093	XLOC_132420	ENSRNOT00000055406	0.969097172	2.11E-07
TUCP_003093	XLOC_132420	ENSRNOT00000070817	0.96635004	3.21E-07
TUCP_003093	XLOC_132420	ENSRNOT00000051285	0.964902205	3.95E-07
TUCP_003093	XLOC_132420	ENSRNOT00000026753	0.969097172	2.11E-07
TUCP_003093	XLOC_132420	ENSRNOT00000080533	0.964320637	4.29E-07
TUCP_003093	XLOC_132420	ENSRNOT00000086031	0.969097172	2.11E-07
TUCP_003093	XLOC_132420	ENSRNOT00000014312	0.969097172	2.11E-07
TUCP_003093	XLOC_132420	ENSRNOT00000051231	0.966865785	2.98E-07
TUCP_003093	XLOC_132420	ENSRNOT00000089407	0.95774737	9.88E-07
TUCP_003093	XLOC_132420	ENSRNOT00000090755	0.98210377	1.40E-08
TUCP_003093	XLOC_132420	ENSRNOT00000055034	0.96461781	4.12E-07
TUCP_003207	XLOC_138391	ENSRNOT00000071885	0.956073956	1.20E-06
TUCP_003207	XLOC_138391	ENSRNOT00000021946	0.954751192	1.38E-06
TUCP_003207	XLOC_138391	ENSRNOT00000006519	0.960467331	7.12E-07
TUCP_003207	XLOC_138391	ENSRNOT00000078710	0.980887193	1.95E-08
TUCP_003207	XLOC_138391	ENSRNOT00000090318	0.987930713	1.98E-09

TUCP_003207	XLOC_138391	ENSRNOT00000078140	0.981709807	1.56E-08
TUCP_003207	XLOC_138391	ENSRNOT00000041727	0.992639726	1.68E-10
TUCP_003207	XLOC_138391	ENSRNOT00000021040	-0.966802786	3.00E-07
TUCP_003207	XLOC_138391	ENSRNOT00000076451	0.972142504	1.26E-07
TUCP_000321	XLOC_010468	ENSRNOT00000008988	0.957790342	9.83E-07
TUCP_000321	XLOC_010468	ENSRNOT00000037375	0.952786846	1.71E-06
TUCP_000321	XLOC_010468	ENSRNOT00000064983	0.950692248	2.11E-06
TUCP_000321	XLOC_010468	ENSRNOT00000010701	0.958025945	9.56E-07
TUCP_000321	XLOC_010468	ENSRNOT00000034482	0.977122011	4.75E-08
TUCP_000321	XLOC_010468	ENSRNOT00000089358	0.956674433	1.12E-06
TUCP_000365	XLOC_012117	ENSRNOT00000082043	0.998796144	1.99E-14
TUCP_000365	XLOC_012117	ENSRNOT00000090080	0.970020271	1.81E-07
TUCP_000365	XLOC_012117	ENSRNOT00000068444	0.999873596	2.54E-19
TUCP_000365	XLOC_012117	ENSRNOT00000081370	0.999582436	9.99E-17
TUCP_000365	XLOC_012117	ENSRNOT00000078645	0.98816239	1.79E-09
TUCP_000365	XLOC_012117	ENSRNOT00000075987	0.98728828	2.56E-09
TUCP_000365	XLOC_012117	ENSRNOT00000074990	0.999873596	2.54E-19
TUCP_000365	XLOC_012117	ENSRNOT00000087323	0.994893311	2.71E-11
TUCP_000365	XLOC_012117	ENSRNOT00000074930	0.999873596	2.54E-19
TUCP_000365	XLOC_012117	ENSRNOT00000080575	0.999800559	2.48E-18
TUCP_000365	XLOC_012117	ENSRNOT00000057761	0.982438118	1.28E-08
TUCP_000365	XLOC_012117	ENSRNOT00000078369	0.992500618	1.84E-10
TUCP_000365	XLOC_012117	ENSRNOT00000092982	0.999873596	2.54E-19
TUCP_000365	XLOC_012117	ENSRNOT00000088367	0.999873596	2.54E-19
TUCP_000365	XLOC_012117	ENSRNOT00000061874	0.969839579	1.87E-07
TUCP_000365	XLOC_012117	ENSRNOT00000077789	0.994432324	4.17E-11
TUCP_000365	XLOC_012117	ENSRNOT00000081996	0.999873596	2.54E-19
TUCP_000365	XLOC_012117	ENSRNOT00000022280	0.981468069	1.67E-08
TUCP_000365	XLOC_012117	ENSRNOT00000082058	0.999873596	2.54E-19
TUCP_000365	XLOC_012117	ENSRNOT00000088905	0.999873596	2.54E-19
TUCP_000365	XLOC_012117	ENSRNOT00000086831	0.999873596	2.54E-19
TUCP_000365	XLOC_012117	ENSRNOT00000045099	0.999873596	2.54E-19
TUCP_000365	XLOC_012117	ENSRNOT00000086745	0.978947417	3.14E-08
TUCP_000365	XLOC_012117	ENSRNOT00000085849	0.987164539	2.69E-09
TUCP_000365	XLOC_012117	ENSRNOT00000086397	0.95829006	9.27E-07
TUCP_000365	XLOC_012117	ENSRNOT00000079606	0.959715564	7.81E-07
TUCP_000365	XLOC_012117	ENSRNOT00000090207	0.955041381	1.34E-06
TUCP_000365	XLOC_012117	ENSRNOT00000087025	0.999873596	2.54E-19
TUCP_000365	XLOC_012117	ENSRNOT00000019853	0.999873596	2.54E-19
TUCP_000365	XLOC_012117	ENSRNOT00000073853	0.989264053	1.10E-09
TUCP_000365	XLOC_012117	ENSRNOT00000065912	0.975458985	6.73E-08
TUCP_000365	XLOC_012117	ENSRNOT00000073828	0.970985169	1.54E-07
TUCP_000365	XLOC_012117	ENSRNOT00000078683	0.970037607	1.81E-07
TUCP_000365	XLOC_012117	ENSRNOT00000093083	0.999121754	4.11E-15
TUCP_000323	XLOC_010515	ENSRNOT00000084500	0.984887141	6.05E-09
TUCP_000323	XLOC_010515	ENSRNOT00000093467	0.996134431	6.75E-12
TUCP_000323	XLOC_010515	ENSRNOT00000029340	0.999116572	4.23E-15
TUCP_000323	XLOC_010515	ENSRNOT00000077972	0.962758032	5.30E-07
TUCP_000323	XLOC_010515	ENSRNOT00000038646	1	0
TUCP_000323	XLOC_010515	ENSRNOT00000012095	0.957750287	9.88E-07
TUCP_000323	XLOC_010515	ENSRNOT00000017909	0.975587192	6.56E-08

TUCP_000323	XLOC_010515	ENSRNOT00000092579	1	4.25E-78
TUCP_000323	XLOC_010515	ENSRNOT00000089235	0.998661162	3.38E-14
TUCP_000323	XLOC_010515	ENSRNOT00000036383	0.988460809	1.58E-09
TUCP_000323	XLOC_010515	ENSRNOT00000076528	0.997115373	1.57E-12
TUCP_000323	XLOC_010515	ENSRNOT00000075193	0.964326971	4.29E-07
TUCP_000323	XLOC_010515	ENSRNOT00000083034	0.990934646	4.75E-10
TUCP_000323	XLOC_010515	ENSRNOT00000089607	0.995091455	2.23E-11
TUCP_000323	XLOC_010515	ENSRNOT00000086014	0.960893638	6.74E-07
TUCP_000323	XLOC_010515	ENSRNOT00000054939	0.958021495	9.57E-07
TUCP_000323	XLOC_010515	ENSRNOT00000052315	0.983687082	8.85E-09
TUCP_000323	XLOC_010515	ENSRNOT00000088168	1	1.33E-79
TUCP_000323	XLOC_010515	ENSRNOT00000046231	0.96225463	5.66E-07
TUCP_000323	XLOC_010515	ENSRNOT00000013459	0.979098017	3.03E-08
TUCP_000323	XLOC_010515	ENSRNOT00000078137	1	1.33E-79
TUCP_000323	XLOC_010515	ENSRNOT00000077764	0.977143528	4.73E-08
TUCP_000323	XLOC_010515	ENSRNOT00000043661	0.966975951	2.93E-07
TUCP_000323	XLOC_010515	ENSRNOT00000092740	1	0
TUCP_000323	XLOC_010515	ENSRNOT00000000117	0.990989174	4.61E-10
TUCP_000323	XLOC_010515	ENSRNOT00000071877	0.967113583	2.87E-07
TUCP_000323	XLOC_010515	ENSRNOT00000070958	1	4.25E-78
TUCP_000323	XLOC_010515	ENSRNOT00000076757	0.959205354	8.31E-07
TUCP_000323	XLOC_010515	ENSRNOT00000085310	0.965230243	3.78E-07
TUCP_000323	XLOC_010515	ENSRNOT00000047241	0.966576993	3.11E-07
TUCP_000323	XLOC_010515	ENSRNOT00000027268	1	1.33E-79
TUCP_000323	XLOC_010515	ENSRNOT00000004459	1	0
TUCP_000323	XLOC_010515	ENSRNOT00000048373	0.959096578	8.42E-07
TUCP_000323	XLOC_010515	ENSRNOT00000061516	0.987595648	2.27E-09
TUCP_000323	XLOC_010515	ENSRNOT00000088148	1	0
TUCP_000323	XLOC_010515	ENSRNOT00000088527	1	0
TUCP_000323	XLOC_010515	ENSRNOT00000078701	1	1.33E-79
TUCP_000323	XLOC_010515	ENSRNOT00000052308	0.962646127	5.38E-07
TUCP_000323	XLOC_010515	ENSRNOT00000021748	1	0
TUCP_000323	XLOC_010515	ENSRNOT00000089085	1	0
TUCP_000323	XLOC_010515	ENSRNOT00000008153	0.999703506	1.80E-17
TUCP_000323	XLOC_010515	ENSRNOT00000085896	0.971605382	1.39E-07
TUCP_000323	XLOC_010515	ENSRNOT00000079189	0.984972387	5.89E-09
TUCP_000323	XLOC_010515	ENSRNOT00000090078	1	0
TUCP_000323	XLOC_010515	ENSRNOT00000078020	0.958421666	9.13E-07
TUCP_000323	XLOC_010515	ENSRNOT00000092425	0.956092273	1.19E-06
TUCP_000323	XLOC_010515	ENSRNOT00000009776	1	1.33E-79
TUCP_000249	XLOC_007621	ENSRNOT00000012161	0.951781481	1.89E-06
TUCP_000249	XLOC_007621	ENSRNOT00000090096	-0.956402689	1.15E-06
TUCP_000249	XLOC_007621	ENSRNOT00000081570	0.982183362	1.37E-08
TUCP_000249	XLOC_007621	ENSRNOT00000082601	0.979812414	2.55E-08
TUCP_000249	XLOC_007621	ENSRNOT00000078707	0.957116418	1.06E-06
TUCP_000329	XLOC_010523	ENSRNOT00000013242	-0.95796802	9.63E-07
TUCP_000329	XLOC_010523	ENSRNOT00000038020	0.957480491	1.02E-06
TUCP_000329	XLOC_010523	ENSRNOT00000086405	0.959261864	8.25E-07
TUCP_000329	XLOC_010523	ENSRNOT00000072306	0.952112783	1.83E-06
TUCP_000329	XLOC_010523	ENSRNOT00000087907	0.95079375	2.09E-06
TUCP_000329	XLOC_010523	ENSRNOT00000009904	0.966363212	3.20E-07

TUCP_000329	XLOC_010523	ENSRNOT00000010171	0.983249373	1.01E-08
TUCP_003056	XLOC_130489	ENSRNOT00000078372	0.950750898	2.10E-06
TUCP_003056	XLOC_130489	ENSRNOT00000056557	0.958488444	9.05E-07
TUCP_003056	XLOC_130489	ENSRNOT00000088078	0.958126622	9.45E-07
TUCP_003056	XLOC_130489	ENSRNOT00000017412	0.965701703	3.53E-07
TUCP_003056	XLOC_130489	ENSRNOT00000076618	0.958956119	8.56E-07
TUCP_003056	XLOC_130489	ENSRNOT00000082002	0.964393273	4.25E-07
TUCP_003378	XLOC_146336	ENSRNOT00000079517	-0.952388097	1.78E-06
TUCP_003378	XLOC_146336	ENSRNOT00000013817	0.954779031	1.38E-06
TUCP_003378	XLOC_146336	ENSRNOT00000018864	-0.951913607	1.87E-06
TUCP_003378	XLOC_146336	ENSRNOT00000021826	0.950612391	2.13E-06
TUCP_003378	XLOC_146336	ENSRNOT00000001602	0.962998239	5.13E-07
TUCP_003378	XLOC_146336	ENSRNOT00000031384	0.951370925	1.97E-06
TUCP_003378	XLOC_146336	ENSRNOT00000073667	-0.952446114	1.77E-06
TUCP_003378	XLOC_146336	ENSRNOT00000048687	0.954652267	1.40E-06
TUCP_003378	XLOC_146336	ENSRNOT00000065600	0.956215971	1.18E-06
TUCP_003378	XLOC_146336	ENSRNOT00000057537	0.961448269	6.29E-07
TUCP_003378	XLOC_146336	ENSRNOT00000083891	-0.956696214	1.11E-06
TUCP_003378	XLOC_146336	ENSRNOT00000021537	0.955540947	1.27E-06
TUCP_003378	XLOC_146336	ENSRNOT00000014888	0.959652725	7.87E-07
TUCP_003378	XLOC_146336	ENSRNOT00000037015	0.957066207	1.07E-06
TUCP_003378	XLOC_146336	ENSRNOT00000055630	0.956945746	1.08E-06
TUCP_003378	XLOC_146336	ENSRNOT00000068663	0.963612229	4.73E-07
TUCP_003378	XLOC_146336	ENSRNOT00000056651	0.953915141	1.51E-06
TUCP_003378	XLOC_146336	ENSRNOT00000040525	0.951706606	1.91E-06
TUCP_003378	XLOC_146336	ENSRNOT00000025309	0.961625625	6.14E-07
TUCP_003378	XLOC_146336	ENSRNOT00000068212	0.965655279	3.55E-07
TUCP_003378	XLOC_146336	ENSRNOT00000026518	0.953404209	1.60E-06
TUCP_004331	XLOC_191140	ENSRNOT00000071840	0.988457864	1.58E-09
TUCP_004331	XLOC_191140	ENSRNOT00000083608	0.978521528	3.47E-08
TUCP_004331	XLOC_191140	ENSRNOT00000085476	0.961348774	6.37E-07
TUCP_004331	XLOC_191140	ENSRNOT00000045584	0.988457864	1.58E-09
TUCP_004331	XLOC_191140	ENSRNOT00000077995	0.977852332	4.04E-08
TUCP_004331	XLOC_191140	ENSRNOT00000066004	0.98794305	1.97E-09
TUCP_004331	XLOC_191140	ENSRNOT00000064284	0.953844413	1.53E-06
TUCP_004331	XLOC_191140	ENSRNOT00000066685	0.97324662	1.03E-07
TUCP_004331	XLOC_191140	ENSRNOT00000082086	0.981740824	1.55E-08
TUCP_004331	XLOC_191140	ENSRNOT00000082084	0.984426397	7.03E-09
TUCP_004331	XLOC_191140	ENSRNOT00000092182	0.988457864	1.58E-09
TUCP_004331	XLOC_191140	ENSRNOT00000084461	0.988457864	1.58E-09
TUCP_004331	XLOC_191140	ENSRNOT00000047784	0.976227868	5.75E-08
TUCP_004331	XLOC_191140	ENSRNOT00000032631	0.988457864	1.58E-09
TUCP_004331	XLOC_191140	ENSRNOT00000079503	0.964035511	4.46E-07
TUCP_004331	XLOC_191140	ENSRNOT00000060113	0.959247933	8.27E-07
TUCP_004331	XLOC_191140	ENSRNOT00000054910	0.978228282	3.71E-08
TUCP_004331	XLOC_191140	ENSRNOT00000057176	0.988434925	1.60E-09
TUCP_004331	XLOC_191140	ENSRNOT00000078176	0.981265814	1.76E-08
TUCP_004331	XLOC_191140	ENSRNOT00000056364	0.975278865	6.98E-08
TUCP_004331	XLOC_191140	ENSRNOT00000089494	0.971644908	1.38E-07
TUCP_004331	XLOC_191140	ENSRNOT00000093431	0.988457864	1.58E-09
TUCP_004331	XLOC_191140	ENSRNOT00000060448	0.988457864	1.58E-09

TUCP_004331	XLOC_191140	ENSRNOT00000092901	0.988457864	1.58E-09
TUCP_004331	XLOC_191140	ENSRNOT00000089290	0.988343388	1.66E-09
TUCP_004331	XLOC_191140	ENSRNOT00000090211	0.985701094	4.60E-09
TUCP_004331	XLOC_191140	ENSRNOT00000075244	0.967857262	2.56E-07
TUCP_004331	XLOC_191140	ENSRNOT00000091828	0.982643817	1.20E-08
TUCP_004331	XLOC_191140	ENSRNOT00000091944	0.987686214	2.18E-09
TUCP_004331	XLOC_191140	ENSRNOT00000070855	0.988457864	1.58E-09
TUCP_004331	XLOC_191140	ENSRNOT00000009295	0.983019801	1.08E-08
TUCP_004331	XLOC_191140	ENSRNOT00000084109	0.986526458	3.42E-09
TUCP_004331	XLOC_191140	ENSRNOT00000016442	0.970369508	1.71E-07
TUCP_004331	XLOC_191140	ENSRNOT00000060645	0.953856037	1.52E-06
TUCP_004331	XLOC_191140	ENSRNOT00000041569	0.988457864	1.58E-09
TUCP_004331	XLOC_191140	ENSRNOT00000087091	0.988457864	1.58E-09
TUCP_004331	XLOC_191140	ENSRNOT00000003308	0.973490601	9.86E-08
TUCP_004331	XLOC_191140	ENSRNOT00000026113	0.988049918	1.88E-09
TUCP_004331	XLOC_191140	ENSRNOT00000014939	0.988281471	1.71E-09
TUCP_004331	XLOC_191140	ENSRNOT00000074800	0.956561151	1.13E-06
TUCP_004331	XLOC_191140	ENSRNOT00000058314	0.977406623	4.46E-08
TUCP_004331	XLOC_191140	ENSRNOT00000047870	0.965937158	3.41E-07
TUCP_004331	XLOC_191140	ENSRNOT00000071603	0.988410276	1.62E-09
TUCP_004331	XLOC_191140	ENSRNOT00000088376	0.950981725	2.05E-06
TUCP_004331	XLOC_191140	ENSRNOT00000024758	0.988001015	1.92E-09
TUCP_004331	XLOC_191140	ENSRNOT00000044232	0.96888897	2.18E-07
TUCP_004331	XLOC_191140	ENSRNOT00000023287	0.975519659	6.65E-08
TUCP_004331	XLOC_191140	ENSRNOT00000071714	0.964414133	4.23E-07
TUCP_004331	XLOC_191140	ENSRNOT00000075836	0.988457864	1.58E-09
TUCP_004331	XLOC_191140	ENSRNOT00000073229	0.988457864	1.58E-09
TUCP_004331	XLOC_191140	ENSRNOT00000046980	0.968516686	2.31E-07
TUCP_004331	XLOC_191140	ENSRNOT00000039652	0.956778034	1.10E-06
TUCP_004331	XLOC_191140	ENSRNOT00000060454	0.988457864	1.58E-09
TUCP_004331	XLOC_191140	ENSRNOT00000082776	0.951330626	1.98E-06
TUCP_004331	XLOC_191140	ENSRNOT00000088719	0.95006797	2.25E-06
TUCP_004331	XLOC_191140	ENSRNOT00000007683	0.988241838	1.74E-09
TUCP_004331	XLOC_191140	ENSRNOT00000092916	0.952470271	1.76E-06
TUCP_004331	XLOC_191140	ENSRNOT00000081106	0.987174616	2.67E-09
TUCP_004337	XLOC_191304	ENSRNOT00000064255	0.963168307	5.02E-07
TUCP_000244	XLOC_007621	ENSRNOT00000093581	0.978403667	3.57E-08
TUCP_000244	XLOC_007621	ENSRNOT00000076554	-0.987115235	2.74E-09
TUCP_000244	XLOC_007621	ENSRNOT00000085797	0.965673888	3.54E-07
TUCP_000244	XLOC_007621	ENSRNOT00000077390	0.966146943	3.31E-07
TUCP_003913	XLOC_172285	ENSRNOT00000000713	0.953572612	1.57E-06
TUCP_003913	XLOC_172285	ENSRNOT00000006992	0.950906002	2.07E-06
TUCP_003913	XLOC_172285	ENSRNOT00000019213	0.953846063	1.53E-06
TUCP_003913	XLOC_172285	ENSRNOT00000041908	0.962018359	5.84E-07
TUCP_003913	XLOC_172285	ENSRNOT00000013050	-0.965838538	3.46E-07
TUCP_003913	XLOC_172285	ENSRNOT00000074939	0.958729806	8.80E-07
TUCP_003913	XLOC_172285	ENSRNOT00000006055	0.952747949	1.71E-06
TUCP_003913	XLOC_172285	ENSRNOT00000071560	0.953983809	1.50E-06
TUCP_003913	XLOC_172285	ENSRNOT00000026065	0.988222998	1.75E-09
TUCP_003913	XLOC_172285	ENSRNOT00000022726	0.971215514	1.48E-07
TUCP_003913	XLOC_172285	ENSRNOT00000005659	0.971639979	1.38E-07

TUCP_003913	XLOC_172285	ENSRNOT00000044635	0.963914476	4.54E-07
TUCP_003913	XLOC_172285	ENSRNOT00000021116	0.953220814	1.63E-06
TUCP_003913	XLOC_172285	ENSRNOT00000018057	0.985036943	5.76E-09
TUCP_003913	XLOC_172285	ENSRNOT00000011442	-0.952649199	1.73E-06
TUCP_003913	XLOC_172285	ENSRNOT00000080654	0.962790615	5.28E-07
TUCP_003913	XLOC_172285	ENSRNOT00000010149	0.968247513	2.41E-07
TUCP_003913	XLOC_172285	ENSRNOT00000028563	0.966113979	3.32E-07
TUCP_003913	XLOC_172285	ENSRNOT00000015076	0.982950037	1.10E-08
TUCP_003913	XLOC_172285	ENSRNOT00000004698	0.963647951	4.70E-07
TUCP_003913	XLOC_172285	ENSRNOT00000010418	0.953141609	1.64E-06
TUCP_003913	XLOC_172285	ENSRNOT00000076516	0.961354416	6.36E-07
TUCP_003913	XLOC_172285	ENSRNOT00000015073	0.971162294	1.50E-07
TUCP_003913	XLOC_172285	ENSRNOT00000018533	0.951585629	1.93E-06
TUCP_003913	XLOC_172285	ENSRNOT00000013046	-0.955422795	1.29E-06
TUCP_003913	XLOC_172285	ENSRNOT00000026783	0.964258734	4.33E-07
TUCP_003913	XLOC_172285	ENSRNOT00000005126	-0.957879329	9.73E-07
TUCP_003913	XLOC_172285	ENSRNOT00000065300	0.959609063	7.91E-07
TUCP_003913	XLOC_172285	ENSRNOT00000011384	0.966194205	3.29E-07
TUCP_003913	XLOC_172285	ENSRNOT00000087306	0.951023977	2.04E-06
TUCP_003913	XLOC_172285	ENSRNOT00000082691	0.964740926	4.04E-07
TUCP_003913	XLOC_172285	ENSRNOT00000076820	0.955055368	1.34E-06
TUCP_003913	XLOC_172285	ENSRNOT00000006963	0.958402107	9.15E-07
TUCP_003913	XLOC_172285	ENSRNOT00000027944	0.988349712	1.66E-09
TUCP_003913	XLOC_172285	ENSRNOT00000068322	0.970067841	1.80E-07
TUCP_003913	XLOC_172285	ENSRNOT00000052075	0.973180392	1.04E-07
TUCP_003913	XLOC_172285	ENSRNOT00000037456	0.950298473	2.20E-06
TUCP_003913	XLOC_172285	ENSRNOT00000017319	0.964866663	3.97E-07
TUCP_003913	XLOC_172285	ENSRNOT00000055187	0.950452167	2.16E-06
TUCP_003913	XLOC_172285	ENSRNOT00000001809	0.961233839	6.46E-07
TUCP_003913	XLOC_172285	ENSRNOT00000016333	-0.961578074	6.18E-07
TUCP_003913	XLOC_172285	ENSRNOT00000078500	0.955009598	1.35E-06
TUCP_003913	XLOC_172285	ENSRNOT00000015612	0.974986186	7.40E-08
TUCP_003913	XLOC_172285	ENSRNOT00000067822	0.961907968	5.92E-07
TUCP_003913	XLOC_172285	ENSRNOT00000022594	0.963471093	4.82E-07
TUCP_003913	XLOC_172285	ENSRNOT00000034722	0.976684453	5.22E-08
TUCP_003913	XLOC_172285	ENSRNOT00000010927	0.977430215	4.44E-08
TUCP_003913	XLOC_172285	ENSRNOT00000051181	0.956664022	1.12E-06
TUCP_003913	XLOC_172285	ENSRNOT00000028483	0.953376517	1.60E-06
TUCP_003913	XLOC_172285	ENSRNOT00000071386	-0.956103701	1.19E-06
TUCP_003913	XLOC_172285	ENSRNOT00000054955	-0.959649387	7.87E-07
TUCP_003913	XLOC_172285	ENSRNOT00000068633	0.982901581	1.12E-08
TUCP_003913	XLOC_172285	ENSRNOT00000037446	0.951259324	2.00E-06
TUCP_003913	XLOC_172285	ENSRNOT00000058645	0.970772212	1.60E-07
TUCP_003913	XLOC_172285	ENSRNOT00000017948	0.967687683	2.63E-07
TUCP_003913	XLOC_172285	ENSRNOT00000084005	-0.95611685	1.19E-06
TUCP_003913	XLOC_172285	ENSRNOT00000023180	0.957754893	9.87E-07
TUCP_003913	XLOC_172285	ENSRNOT00000020892	-0.957018299	1.07E-06
TUCP_003913	XLOC_172285	ENSRNOT00000019554	0.967137944	2.86E-07
TUCP_003913	XLOC_172285	ENSRNOT00000077908	0.951677056	1.91E-06
TUCP_003913	XLOC_172285	ENSRNOT00000022213	0.952750409	1.71E-06
TUCP_003913	XLOC_172285	ENSRNOT00000022610	0.961925952	5.91E-07

TUCP_003913	XLOC_172285	ENSRNOT00000020437	0.954368024	1.44E-06
TUCP_003913	XLOC_172285	ENSRNOT00000064264	0.959048897	8.47E-07
TUCP_003913	XLOC_172285	ENSRNOT00000073346	0.953621206	1.56E-06
TUCP_003913	XLOC_172285	ENSRNOT00000023330	0.978150761	3.78E-08
TUCP_003913	XLOC_172285	ENSRNOT00000025983	0.960247265	7.31E-07
TUCP_003913	XLOC_172285	ENSRNOT00000007164	0.955735079	1.24E-06
TUCP_003913	XLOC_172285	ENSRNOT00000090451	-0.958570354	8.97E-07
TUCP_003913	XLOC_172285	ENSRNOT00000015325	-0.9629483	5.17E-07
TUCP_003913	XLOC_172285	ENSRNOT00000013661	0.974729838	7.78E-08
TUCP_003913	XLOC_172285	ENSRNOT00000093174	0.954476819	1.43E-06
TUCP_003913	XLOC_172285	ENSRNOT00000017629	0.952236444	1.81E-06
TUCP_003913	XLOC_172285	ENSRNOT00000080800	0.96802176	2.50E-07
TUCP_003913	XLOC_172285	ENSRNOT00000061100	0.95447726	1.43E-06
TUCP_003913	XLOC_172285	ENSRNOT00000020648	0.95694255	1.08E-06
TUCP_003913	XLOC_172285	ENSRNOT00000082228	0.956511802	1.14E-06
TUCP_003913	XLOC_172285	ENSRNOT00000034744	0.972438091	1.20E-07
TUCP_003913	XLOC_172285	ENSRNOT00000082273	0.952197002	1.81E-06
TUCP_003913	XLOC_172285	ENSRNOT00000021153	0.977126344	4.75E-08
TUCP_003913	XLOC_172285	ENSRNOT00000064902	0.958137174	9.44E-07
TUCP_003913	XLOC_172285	ENSRNOT00000025027	0.954419631	1.44E-06
TUCP_003913	XLOC_172285	ENSRNOT00000088995	0.980618138	2.09E-08
TUCP_003913	XLOC_172285	ENSRNOT00000017884	0.950754232	2.10E-06
TUCP_003913	XLOC_172285	ENSRNOT00000016722	0.964004874	4.48E-07
TUCP_003913	XLOC_172285	ENSRNOT00000012424	0.960951529	6.70E-07
TUCP_003913	XLOC_172285	ENSRNOT00000039631	0.96163245	6.14E-07
TUCP_003913	XLOC_172285	ENSRNOT00000090170	0.970010531	1.82E-07
TUCP_003913	XLOC_172285	ENSRNOT00000019656	-0.951735238	1.90E-06
TUCP_003913	XLOC_172285	ENSRNOT00000049538	0.95879239	8.73E-07
TUCP_003913	XLOC_172285	ENSRNOT00000005909	0.962958698	5.16E-07
TUCP_003913	XLOC_172285	ENSRNOT00000027054	0.959346541	8.17E-07
TUCP_003913	XLOC_172285	ENSRNOT00000009129	0.957997727	9.59E-07
TUCP_003913	XLOC_172285	ENSRNOT00000015635	-0.958156139	9.42E-07
TUCP_003913	XLOC_172285	ENSRNOT00000036951	0.968142824	2.45E-07
TUCP_003913	XLOC_172285	ENSRNOT00000026186	0.9760249	5.99E-08
TUCP_003913	XLOC_172285	ENSRNOT00000007091	0.959148701	8.37E-07
TUCP_003913	XLOC_172285	ENSRNOT00000075424	0.955014628	1.35E-06
TUCP_003913	XLOC_172285	ENSRNOT00000011663	0.962722273	5.33E-07
TUCP_003913	XLOC_172285	ENSRNOT00000057313	0.968396037	2.35E-07
TUCP_003913	XLOC_172285	ENSRNOT00000088196	0.962523142	5.47E-07
TUCP_003913	XLOC_172285	ENSRNOT00000023645	0.97557031	6.58E-08
TUCP_003913	XLOC_172285	ENSRNOT00000019553	0.954634939	1.40E-06
TUCP_003913	XLOC_172285	ENSRNOT00000035700	0.95120403	2.01E-06
TUCP_003913	XLOC_172285	ENSRNOT00000064840	0.968687707	2.25E-07
TUCP_003913	XLOC_172285	ENSRNOT00000011984	0.953153381	1.64E-06
TUCP_003913	XLOC_172285	ENSRNOT00000005977	0.960994203	6.66E-07
TUCP_003913	XLOC_172285	ENSRNOT00000007175	0.971832283	1.33E-07
TUCP_003913	XLOC_172285	ENSRNOT00000020669	0.960736217	6.88E-07
TUCP_003913	XLOC_172285	ENSRNOT00000074653	0.981648823	1.59E-08
TUCP_003913	XLOC_172285	ENSRNOT00000031230	0.956863799	1.09E-06
TUCP_003913	XLOC_172285	ENSRNOT00000010218	0.96307527	5.08E-07
TUCP_003913	XLOC_172285	ENSRNOT00000010588	0.968864764	2.19E-07

TUCP_003913	XLOC_172285	ENSRNOT00000002292	0.974406168	8.29E-08
TUCP_003913	XLOC_172285	ENSRNOT00000045083	0.989552063	9.63E-10
TUCP_003913	XLOC_172285	ENSRNOT00000035491	0.950783285	2.09E-06
TUCP_003913	XLOC_172285	ENSRNOT00000083369	0.950910534	2.07E-06
TUCP_003913	XLOC_172285	ENSRNOT00000092906	0.964300871	4.30E-07
TUCP_003913	XLOC_172285	ENSRNOT00000085596	0.965474929	3.65E-07
TUCP_003913	XLOC_172285	ENSRNOT00000082174	0.965139999	3.82E-07
TUCP_003913	XLOC_172285	ENSRNOT00000024060	0.989473415	1.00E-09
TUCP_003913	XLOC_172285	ENSRNOT00000038349	0.960354885	7.22E-07
TUCP_003913	XLOC_172285	ENSRNOT00000004412	0.963655412	4.70E-07
TUCP_003913	XLOC_172285	ENSRNOT00000019177	0.957616669	1.00E-06
TUCP_003913	XLOC_172285	ENSRNOT00000011784	0.958629907	8.90E-07
TUCP_003913	XLOC_172285	ENSRNOT00000084595	0.956171268	1.18E-06
TUCP_003913	XLOC_172285	ENSRNOT00000024941	0.962597424	5.41E-07
TUCP_003913	XLOC_172285	ENSRNOT00000066593	0.965054278	3.87E-07
TUCP_003913	XLOC_172285	ENSRNOT00000013031	-0.971654129	1.37E-07
TUCP_003913	XLOC_172285	ENSRNOT00000036750	0.95984082	7.69E-07
TUCP_003913	XLOC_172285	ENSRNOT00000027964	0.960484194	7.10E-07
TUCP_003913	XLOC_172285	ENSRNOT00000024659	0.962135569	5.75E-07
TUCP_003913	XLOC_172285	ENSRNOT00000003834	-0.956540988	1.13E-06
TUCP_003913	XLOC_172285	ENSRNOT00000026216	0.954688046	1.39E-06
TUCP_003913	XLOC_172285	ENSRNOT00000052288	0.969273322	2.05E-07
TUCP_003913	XLOC_172285	ENSRNOT00000048029	0.994724419	3.19E-11
TUCP_003913	XLOC_172285	ENSRNOT00000004140	0.950123063	2.24E-06
TUCP_003913	XLOC_172285	ENSRNOT00000010433	0.975416254	6.79E-08
TUCP_003913	XLOC_172285	ENSRNOT00000064930	0.965627476	3.57E-07
TUCP_003913	XLOC_172285	ENSRNOT00000079996	0.963445625	4.83E-07
TUCP_003913	XLOC_172285	ENSRNOT00000011325	0.958248177	9.31E-07
TUCP_004625	XLOC_203969	ENSRNOT00000003286	0.959459324	8.06E-07
TUCP_004625	XLOC_203969	ENSRNOT00000037191	0.9619519	5.89E-07
TUCP_004231	XLOC_185847	ENSRNOT00000009132	0.964960901	3.92E-07
TUCP_004231	XLOC_185847	ENSRNOT00000031365	0.959293564	8.22E-07
TUCP_004231	XLOC_185847	ENSRNOT00000074096	0.955416724	1.29E-06
TUCP_004231	XLOC_185847	ENSRNOT00000061430	0.969452521	1.99E-07
TUCP_004231	XLOC_185847	ENSRNOT00000005067	0.961257199	6.44E-07
TUCP_004231	XLOC_185847	ENSRNOT00000041762	0.957630983	1.00E-06
TUCP_003917	XLOC_172529	ENSRNOT00000067032	-0.966181933	3.29E-07
TUCP_003916	XLOC_172480	ENSRNOT00000013817	0.956369235	1.16E-06
TUCP_003916	XLOC_172480	ENSRNOT00000076404	0.953205606	1.63E-06
TUCP_003916	XLOC_172480	ENSRNOT00000025517	0.970505844	1.67E-07
TUCP_003916	XLOC_172480	ENSRNOT00000082183	0.964033374	4.46E-07
TUCP_003916	XLOC_172480	ENSRNOT00000013524	0.980711883	2.04E-08
TUCP_003916	XLOC_172480	ENSRNOT00000026391	0.978305385	3.65E-08
TUCP_003916	XLOC_172480	ENSRNOT00000013284	0.965035956	3.88E-07
TUCP_003916	XLOC_172480	ENSRNOT00000068212	0.983044853	1.07E-08
TUCP_003916	XLOC_172480	ENSRNOT00000026259	0.963409476	4.86E-07
TUCP_003916	XLOC_172480	ENSRNOT00000089243	0.961764726	6.04E-07
TUCP_003916	XLOC_172480	ENSRNOT00000040525	0.960019771	7.52E-07
TUCP_003916	XLOC_172480	ENSRNOT00000087138	0.955030413	1.34E-06
TUCP_003916	XLOC_172480	ENSRNOT00000027170	0.961771583	6.03E-07
TUCP_003916	XLOC_172480	ENSRNOT00000073041	0.974982835	7.40E-08

TUCP_003916	XLOC_172480	ENSRNOT00000022716	0.954933223	1.36E-06
TUCP_003916	XLOC_172480	ENSRNOT00000024066	0.959208943	8.30E-07
TUCP_003916	XLOC_172480	ENSRNOT00000046639	0.960646976	6.96E-07
TUCP_003916	XLOC_172480	ENSRNOT00000071998	0.968613294	2.28E-07
TUCP_003916	XLOC_172480	ENSRNOT00000076973	0.95910629	8.41E-07
TUCP_003916	XLOC_172480	ENSRNOT00000004176	0.954660776	1.40E-06
TUCP_003916	XLOC_172480	ENSRNOT00000037133	0.956009605	1.20E-06
TUCP_003916	XLOC_172480	ENSRNOT00000084567	0.969603632	1.94E-07
TUCP_003916	XLOC_172480	ENSRNOT00000064772	0.957806116	9.81E-07
TUCP_003916	XLOC_172480	ENSRNOT00000014127	0.972217997	1.24E-07
TUCP_003916	XLOC_172480	ENSRNOT00000044395	0.955799347	1.23E-06
TUCP_003916	XLOC_172480	ENSRNOT00000034298	0.962580623	5.43E-07
TUCP_003916	XLOC_172480	ENSRNOT00000049245	0.950374933	2.18E-06
TUCP_003916	XLOC_172480	ENSRNOT00000037015	0.95831754	9.24E-07
TUCP_003916	XLOC_172480	ENSRNOT00000068663	0.960535717	7.05E-07
TUCP_003916	XLOC_172480	ENSRNOT00000017384	0.950741924	2.10E-06
TUCP_003916	XLOC_172480	ENSRNOT00000017805	0.972424224	1.20E-07
TUCP_003916	XLOC_172480	ENSRNOT00000065076	0.965087695	3.85E-07
TUCP_003916	XLOC_172480	ENSRNOT00000048687	0.959909683	7.62E-07
TUCP_003916	XLOC_172480	ENSRNOT00000002996	0.950030109	2.26E-06
TUCP_003916	XLOC_172480	ENSRNOT00000027476	0.951583583	1.93E-06
TUCP_003916	XLOC_172480	ENSRNOT00000086917	0.959396985	8.12E-07
TUCP_003916	XLOC_172480	ENSRNOT00000054685	0.953600829	1.57E-06
TUCP_003916	XLOC_172480	ENSRNOT00000021531	0.958497801	9.04E-07
TUCP_003916	XLOC_172480	ENSRNOT00000016445	0.95967532	7.85E-07
TUCP_003916	XLOC_172480	ENSRNOT00000027264	0.967651931	2.64E-07
TUCP_003916	XLOC_172480	ENSRNOT00000083585	0.960523639	7.07E-07
TUCP_003916	XLOC_172480	ENSRNOT00000036137	0.955021684	1.34E-06
TUCP_003916	XLOC_172480	ENSRNOT00000073327	0.961875368	5.95E-07
TUCP_003916	XLOC_172480	ENSRNOT00000020929	0.95073701	2.10E-06
TUCP_003916	XLOC_172480	ENSRNOT00000027967	0.96225037	5.67E-07
TUCP_003916	XLOC_172480	ENSRNOT00000076822	0.96376774	4.63E-07
TUCP_003916	XLOC_172480	ENSRNOT00000008244	0.957886637	9.72E-07
TUCP_003916	XLOC_172480	ENSRNOT00000011773	0.968971954	2.15E-07
TUCP_003916	XLOC_172480	ENSRNOT00000081678	0.961462984	6.27E-07
TUCP_003916	XLOC_172480	ENSRNOT00000088628	0.959648757	7.87E-07
TUCP_003916	XLOC_172480	ENSRNOT00000003780	0.955700016	1.25E-06
TUCP_003916	XLOC_172480	ENSRNOT00000014888	0.955672194	1.25E-06
TUCP_003916	XLOC_172480	ENSRNOT00000002621	0.956013423	1.20E-06
TUCP_003916	XLOC_172480	ENSRNOT00000010939	0.959038131	8.48E-07
TUCP_004622	XLOC_203765	ENSRNOT00000001215	-0.955569141	1.27E-06
TUCP_004622	XLOC_203765	ENSRNOT00000030074	-0.962152088	5.74E-07
TUCP_004622	XLOC_203765	ENSRNOT00000020297	-0.957452939	1.02E-06
TUCP_004622	XLOC_203765	ENSRNOT00000081796	-0.952710319	1.72E-06
TUCP_004622	XLOC_203765	ENSRNOT00000090621	-0.967493293	2.71E-07
TUCP_004622	XLOC_203765	ENSRNOT00000006460	-0.957891941	9.71E-07
TUCP_004622	XLOC_203765	ENSRNOT00000075397	-0.959683215	7.84E-07
TUCP_004622	XLOC_203765	ENSRNOT00000007350	-0.970345608	1.72E-07
TUCP_004622	XLOC_203765	ENSRNOT00000020051	-0.962396234	5.56E-07
TUCP_004622	XLOC_203765	ENSRNOT00000009080	0.95829676	9.26E-07
TUCP_004622	XLOC_203765	ENSRNOT00000006217	-0.959893737	7.64E-07

TUCP_004622	XLOC_203765	ENSRNOT00000015678	0.965786368	3.49E-07
TUCP_004622	XLOC_203765	ENSRNOT00000082914	-0.960815494	6.81E-07
TUCP_004622	XLOC_203765	ENSRNOT00000007698	0.963112032	5.06E-07
TUCP_004622	XLOC_203765	ENSRNOT00000013025	0.953888359	1.52E-06
TUCP_004622	XLOC_203765	ENSRNOT00000079138	-0.968314671	2.38E-07
TUCP_004622	XLOC_203765	ENSRNOT00000071998	-0.952754566	1.71E-06
TUCP_004622	XLOC_203765	ENSRNOT00000084567	-0.979212551	2.95E-08
TUCP_004622	XLOC_203765	ENSRNOT00000008361	-0.953588912	1.57E-06
TUCP_004622	XLOC_203765	ENSRNOT00000052063	-0.961943583	5.90E-07
TUCP_004622	XLOC_203765	ENSRNOT00000042638	-0.956834167	1.10E-06
TUCP_004622	XLOC_203765	ENSRNOT00000084069	-0.959531312	7.99E-07
TUCP_004622	XLOC_203765	ENSRNOT00000001679	-0.96447521	4.20E-07
TUCP_004622	XLOC_203765	ENSRNOT00000010419	-0.958029112	9.56E-07
TUCP_004622	XLOC_203765	ENSRNOT00000065076	-0.967251583	2.81E-07
TUCP_004622	XLOC_203765	ENSRNOT00000031672	0.951945676	1.86E-06
TUCP_004622	XLOC_203765	ENSRNOT00000013516	-0.967076293	2.88E-07
TUCP_004622	XLOC_203765	ENSRNOT00000054685	-0.953909458	1.52E-06
TUCP_004622	XLOC_203765	ENSRNOT00000022097	-0.951933496	1.86E-06
TUCP_004622	XLOC_203765	ENSRNOT00000012060	-0.964706594	4.06E-07
TUCP_004622	XLOC_203765	ENSRNOT00000073370	-0.963198508	5.00E-07
TUCP_004622	XLOC_203765	ENSRNOT00000083568	-0.974982035	7.40E-08
TUCP_004622	XLOC_203765	ENSRNOT00000025079	0.958851818	8.67E-07
TUCP_004622	XLOC_203765	ENSRNOT00000026141	-0.955258035	1.31E-06
TUCP_004622	XLOC_203765	ENSRNOT00000084709	-0.967474569	2.71E-07
TUCP_004622	XLOC_203765	ENSRNOT00000065978	-0.958965029	8.55E-07
TUCP_004622	XLOC_203765	ENSRNOT00000020929	-0.965192384	3.80E-07
TUCP_004622	XLOC_203765	ENSRNOT00000039177	0.955141032	1.33E-06
TUCP_004622	XLOC_203765	ENSRNOT00000017271	-0.954637893	1.40E-06
TUCP_004622	XLOC_203765	ENSRNOT00000056180	-0.964799291	4.01E-07
TUCP_004622	XLOC_203765	ENSRNOT00000034282	-0.953914454	1.52E-06
TUCP_004622	XLOC_203765	ENSRNOT00000027760	-0.963507236	4.79E-07
TUCP_004622	XLOC_203765	ENSRNOT00000012755	-0.950881904	2.07E-06
TUCP_004622	XLOC_203765	ENSRNOT00000065871	-0.976710383	5.19E-08
TUCP_004622	XLOC_203765	ENSRNOT00000024677	-0.975848211	6.21E-08
TUCP_004622	XLOC_203765	ENSRNOT00000056100	0.952644698	1.73E-06
TUCP_004622	XLOC_203765	ENSRNOT00000030082	-0.957089726	1.07E-06
TUCP_004622	XLOC_203765	ENSRNOT00000025517	-0.951025748	2.04E-06
TUCP_004622	XLOC_203765	ENSRNOT00000046807	0.956818757	1.10E-06
TUCP_004622	XLOC_203765	ENSRNOT00000073983	-0.951371167	1.97E-06
TUCP_004622	XLOC_203765	ENSRNOT00000047126	0.97447213	8.18E-08
TUCP_004622	XLOC_203765	ENSRNOT00000026391	-0.966008872	3.38E-07
TUCP_004622	XLOC_203765	ENSRNOT00000081226	-0.958709377	8.82E-07
TUCP_004622	XLOC_203765	ENSRNOT00000065045	-0.964041613	4.46E-07
TUCP_004622	XLOC_203765	ENSRNOT00000005686	0.963589595	4.74E-07
TUCP_004622	XLOC_203765	ENSRNOT00000072762	0.97143315	1.43E-07
TUCP_004622	XLOC_203765	ENSRNOT00000066708	-0.95494478	1.36E-06
TUCP_004622	XLOC_203765	ENSRNOT00000038652	0.954695097	1.39E-06
TUCP_004622	XLOC_203765	ENSRNOT00000011832	0.983857425	8.40E-09
TUCP_004622	XLOC_203765	ENSRNOT00000073643	0.967532709	2.69E-07
TUCP_004622	XLOC_203765	ENSRNOT00000087278	0.961282689	6.42E-07
TUCP_004622	XLOC_203765	ENSRNOT00000046552	-0.951433728	1.96E-06

TUCP_004622	XLOC_203765	ENSRNOT00000067011	-0.960786659	6.84E-07
TUCP_004622	XLOC_203765	ENSRNOT00000083968	0.956892109	1.09E-06
TUCP_004622	XLOC_203765	ENSRNOT00000009150	-0.956318523	1.16E-06
TUCP_004622	XLOC_203765	ENSRNOT00000021858	-0.965731545	3.51E-07
TUCP_004622	XLOC_203765	ENSRNOT00000083967	-0.958591018	8.94E-07
TUCP_004622	XLOC_203765	ENSRNOT00000048781	-0.955478482	1.28E-06
TUCP_004622	XLOC_203765	ENSRNOT00000021802	-0.952686442	1.72E-06
TUCP_004622	XLOC_203765	ENSRNOT00000068452	-0.967809471	2.58E-07
TUCP_004622	XLOC_203765	ENSRNOT00000024352	-0.964794008	4.01E-07
TUCP_004622	XLOC_203765	ENSRNOT00000072299	-0.969506176	1.97E-07
TUCP_004622	XLOC_203765	ENSRNOT00000072118	-0.963040592	5.10E-07
TUCP_004622	XLOC_203765	ENSRNOT00000003780	-0.962424978	5.54E-07
TUCP_004622	XLOC_203765	ENSRNOT00000085334	-0.951142919	2.02E-06
TUCP_004622	XLOC_203765	ENSRNOT00000027739	-0.958768954	8.76E-07
TUCP_004622	XLOC_203765	ENSRNOT00000072357	0.951402497	1.97E-06
TUCP_004622	XLOC_203765	ENSRNOT00000025913	0.950505456	2.15E-06
TUCP_004622	XLOC_203765	ENSRNOT00000013067	0.955960857	1.21E-06
TUCP_004622	XLOC_203765	ENSRNOT00000004196	-0.970593368	1.65E-07
TUCP_004622	XLOC_203765	ENSRNOT00000011956	-0.951017761	2.04E-06
TUCP_004622	XLOC_203765	ENSRNOT00000073919	-0.959682936	7.84E-07
TUCP_004622	XLOC_203765	ENSRNOT00000025966	-0.954103889	1.48E-06
TUCP_004622	XLOC_203765	ENSRNOT00000089611	-0.951503387	1.95E-06
TUCP_004622	XLOC_203765	ENSRNOT00000023667	-0.966540644	3.12E-07
TUCP_004622	XLOC_203765	ENSRNOT00000086260	0.965599982	3.58E-07
TUCP_004622	XLOC_203765	ENSRNOT00000045063	-0.962696059	5.34E-07
TUCP_004622	XLOC_203765	ENSRNOT00000008256	0.950469536	2.16E-06
TUCP_004622	XLOC_203765	ENSRNOT00000076404	-0.951682162	1.91E-06
TUCP_004622	XLOC_203765	ENSRNOT00000034883	0.957755359	9.87E-07
TUCP_004622	XLOC_203765	ENSRNOT00000001158	-0.951521014	1.94E-06
TUCP_004622	XLOC_203765	ENSRNOT00000046639	-0.956617767	1.12E-06
TUCP_004622	XLOC_203765	ENSRNOT00000035605	-0.961738578	6.06E-07
TUCP_004622	XLOC_203765	ENSRNOT00000081351	-0.951399702	1.97E-06
TUCP_004622	XLOC_203765	ENSRNOT00000092083	-0.954009396	1.50E-06
TUCP_004622	XLOC_203765	ENSRNOT00000018844	-0.954639011	1.40E-06
TUCP_004622	XLOC_203765	ENSRNOT00000017624	-0.978844923	3.22E-08
TUCP_004622	XLOC_203765	ENSRNOT00000003992	0.954175528	1.47E-06
TUCP_004622	XLOC_203765	ENSRNOT00000027170	-0.969182232	2.08E-07
TUCP_004622	XLOC_203765	ENSRNOT00000036092	-0.956643993	1.12E-06
TUCP_004622	XLOC_203765	ENSRNOT00000040169	-0.950361607	2.18E-06
TUCP_004622	XLOC_203765	ENSRNOT00000086798	0.957973268	9.62E-07
TUCP_004622	XLOC_203765	ENSRNOT00000081725	-0.974961526	7.43E-08
TUCP_004622	XLOC_203765	ENSRNOT00000020383	-0.971125975	1.51E-07
TUCP_004622	XLOC_203765	ENSRNOT00000005459	-0.965609035	3.58E-07
TUCP_004622	XLOC_203765	ENSRNOT00000012760	0.959033606	8.48E-07
TUCP_004622	XLOC_203765	ENSRNOT00000074515	0.958052366	9.53E-07
TUCP_004622	XLOC_203765	ENSRNOT00000037015	-0.952675256	1.73E-06
TUCP_004622	XLOC_203765	ENSRNOT00000027917	-0.967355828	2.76E-07
TUCP_004622	XLOC_203765	ENSRNOT00000004290	-0.965143898	3.82E-07
TUCP_004622	XLOC_203765	ENSRNOT00000076230	-0.964434636	4.22E-07
TUCP_004622	XLOC_203765	ENSRNOT00000001490	-0.959212677	8.30E-07
TUCP_004622	XLOC_203765	ENSRNOT00000011675	-0.974338473	8.39E-08

TUCP_004622	XLOC_203765	ENSRNOT00000055137	-0.96141454	6.31E-07
TUCP_004622	XLOC_203765	ENSRNOT00000075476	-0.960448385	7.13E-07
TUCP_004622	XLOC_203765	ENSRNOT00000028685	-0.952849991	1.70E-06
TUCP_004622	XLOC_203765	ENSRNOT00000010783	0.951516837	1.94E-06
TUCP_004622	XLOC_203765	ENSRNOT00000080591	-0.9571776	1.06E-06
TUCP_004622	XLOC_203765	ENSRNOT00000066032	-0.952561405	1.75E-06
TUCP_004622	XLOC_203765	ENSRNOT00000005161	-0.971081223	1.52E-07
TUCP_004622	XLOC_203765	ENSRNOT00000049285	0.953203799	1.63E-06
TUCP_004622	XLOC_203765	ENSRNOT00000093385	-0.954070599	1.49E-06
TUCP_004622	XLOC_203765	ENSRNOT00000015239	0.963403563	4.86E-07
TUCP_004622	XLOC_203765	ENSRNOT00000074649	-0.952545461	1.75E-06
TUCP_004622	XLOC_203765	ENSRNOT00000037955	0.969125608	2.10E-07
TUCP_004622	XLOC_203765	ENSRNOT00000077146	0.964042478	4.46E-07
TUCP_004622	XLOC_203765	ENSRNOT00000085911	0.952061125	1.84E-06
TUCP_004622	XLOC_203765	ENSRNOT00000001783	-0.956494085	1.14E-06
TUCP_004622	XLOC_203765	ENSRNOT00000017504	-0.957720188	9.91E-07
TUCP_004622	XLOC_203765	ENSRNOT00000086456	0.953884479	1.52E-06
TUCP_004622	XLOC_203765	ENSRNOT00000075209	0.954434686	1.43E-06
TUCP_004622	XLOC_203765	ENSRNOT00000044275	-0.950598467	2.13E-06
TUCP_004622	XLOC_203765	ENSRNOT00000058382	-0.976934706	4.95E-08
TUCP_004622	XLOC_203765	ENSRNOT00000006824	0.950172622	2.22E-06
TUCP_004622	XLOC_203765	ENSRNOT00000010993	-0.964211845	4.35E-07
TUCP_004622	XLOC_203765	ENSRNOT00000010852	-0.957621932	1.00E-06
TUCP_004622	XLOC_203765	ENSRNOT00000005489	-0.955502712	1.27E-06
TUCP_004622	XLOC_203765	ENSRNOT00000058111	0.962187548	5.71E-07
TUCP_004622	XLOC_203765	ENSRNOT00000017765	-0.97700654	4.87E-08
TUCP_004622	XLOC_203765	ENSRNOT00000057738	-0.968168844	2.44E-07
TUCP_004622	XLOC_203765	ENSRNOT00000018849	0.957936097	9.66E-07
TUCP_004622	XLOC_203765	ENSRNOT00000009994	0.95630041	1.17E-06
TUCP_004622	XLOC_203765	ENSRNOT00000077367	0.954590305	1.41E-06
TUCP_004622	XLOC_203765	ENSRNOT00000024841	0.958340465	9.21E-07
TUCP_004622	XLOC_203765	ENSRNOT00000036137	-0.959369105	8.15E-07
TUCP_004622	XLOC_203765	ENSRNOT00000007702	0.958651341	8.88E-07
TUCP_004622	XLOC_203765	ENSRNOT00000023014	-0.956552517	1.13E-06
TUCP_004622	XLOC_203765	ENSRNOT00000039102	-0.958008985	9.58E-07
TUCP_004622	XLOC_203765	ENSRNOT00000085517	-0.968696384	2.25E-07
TUCP_004622	XLOC_203765	ENSRNOT00000019358	0.967211669	2.82E-07
TUCP_004622	XLOC_203765	ENSRNOT00000009742	0.95070873	2.11E-06
TUCP_004622	XLOC_203765	ENSRNOT00000006835	-0.955216909	1.32E-06
TUCP_004622	XLOC_203765	ENSRNOT00000078906	0.967918275	2.54E-07
TUCP_004622	XLOC_203765	ENSRNOT00000007369	-0.950677595	2.12E-06
TUCP_004622	XLOC_203765	ENSRNOT00000085465	0.961854127	5.97E-07
TUCP_004622	XLOC_203765	ENSRNOT00000025460	-0.959527776	7.99E-07
TUCP_004622	XLOC_203765	ENSRNOT00000012528	-0.96204053	5.82E-07
TUCP_004622	XLOC_203765	ENSRNOT00000028225	-0.953860984	1.52E-06
TUCP_004622	XLOC_203765	ENSRNOT00000061345	0.974792784	7.68E-08
TUCP_004622	XLOC_203765	ENSRNOT00000017998	-0.955495256	1.28E-06
TUCP_004622	XLOC_203765	ENSRNOT00000081077	0.950104446	2.24E-06
TUCP_004622	XLOC_203765	ENSRNOT00000016568	-0.964472871	4.20E-07
TUCP_004622	XLOC_203765	ENSRNOT00000065529	-0.95114081	2.02E-06
TUCP_004622	XLOC_203765	ENSRNOT00000028907	-0.972226665	1.24E-07

TUCP_004622	XLOC_203765	ENSRNOT00000027967	-0.95917761	8.34E-07
TUCP_004622	XLOC_203765	ENSRNOT00000078793	-0.969006317	2.14E-07
TUCP_004622	XLOC_203765	ENSRNOT00000089086	0.965687155	3.54E-07
TUCP_004622	XLOC_203765	ENSRNOT00000081678	-0.950033785	2.25E-06
TUCP_004622	XLOC_203765	ENSRNOT00000036613	0.961957251	5.89E-07
TUCP_004622	XLOC_203765	ENSRNOT00000090268	0.954480436	1.43E-06
TUCP_004622	XLOC_203765	ENSRNOT00000024011	-0.951562036	1.94E-06
TUCP_004622	XLOC_203765	ENSRNOT00000025705	-0.950394148	2.18E-06
TUCP_004622	XLOC_203765	ENSRNOT00000092876	0.974127502	8.74E-08
TUCP_004622	XLOC_203765	ENSRNOT00000024253	-0.955158154	1.32E-06
TUCP_004622	XLOC_203765	ENSRNOT00000085669	-0.950655314	2.12E-06
TUCP_003129	XLOC_133698	ENSRNOT00000027644	-0.960504652	7.08E-07
TUCP_003129	XLOC_133698	ENSRNOT00000016083	-0.967046312	2.90E-07
TUCP_003129	XLOC_133698	ENSRNOT00000072069	-0.960669854	6.94E-07
TUCP_003129	XLOC_133698	ENSRNOT00000087386	-0.961519164	6.23E-07
TUCP_003129	XLOC_133698	ENSRNOT00000017268	-0.967705853	2.62E-07
TUCP_003129	XLOC_133698	ENSRNOT00000009080	-0.971339694	1.45E-07
TUCP_003129	XLOC_133698	ENSRNOT00000059124	-0.958107903	9.47E-07
TUCP_003129	XLOC_133698	ENSRNOT00000035400	-0.958208085	9.36E-07
TUCP_003129	XLOC_133698	ENSRNOT00000017204	-0.960670713	6.94E-07
TUCP_003129	XLOC_133698	ENSRNOT00000032439	-0.95350168	1.58E-06
TUCP_003129	XLOC_133698	ENSRNOT00000010720	-0.96230407	5.63E-07
TUCP_003129	XLOC_133698	ENSRNOT00000087011	-0.953463744	1.59E-06
TUCP_003129	XLOC_133698	ENSRNOT00000022086	-0.954550314	1.41E-06
TUCP_003129	XLOC_133698	ENSRNOT00000072970	-0.960014377	7.53E-07
TUCP_003129	XLOC_133698	ENSRNOT00000021110	-0.974044107	8.88E-08
TUCP_003129	XLOC_133698	ENSRNOT00000031692	-0.971007055	1.54E-07
TUCP_003129	XLOC_133698	ENSRNOT00000020853	-0.956199292	1.18E-06
TUCP_003129	XLOC_133698	ENSRNOT00000050649	-0.953761001	1.54E-06
TUCP_003129	XLOC_133698	ENSRNOT00000001774	-0.961982966	5.87E-07
TUCP_003129	XLOC_133698	ENSRNOT00000080013	-0.966824545	2.99E-07
TUCP_003129	XLOC_133698	ENSRNOT00000013759	-0.961689441	6.09E-07
TUCP_003129	XLOC_133698	ENSRNOT00000018341	-0.950562975	2.14E-06
TUCP_003129	XLOC_133698	ENSRNOT00000044847	-0.964467767	4.20E-07
TUCP_003129	XLOC_133698	ENSRNOT00000075400	-0.959102102	8.41E-07
TUCP_003129	XLOC_133698	ENSRNOT00000070886	-0.963956448	4.51E-07
TUCP_003129	XLOC_133698	ENSRNOT00000002799	-0.95867901	8.85E-07
TUCP_003129	XLOC_133698	ENSRNOT00000033312	-0.961801328	6.01E-07
TUCP_003129	XLOC_133698	ENSRNOT00000016012	-0.963629821	4.71E-07
TUCP_003129	XLOC_133698	ENSRNOT00000022126	-0.96761006	2.66E-07
TUCP_003129	XLOC_133698	ENSRNOT00000061735	-0.953478889	1.59E-06
TUCP_003129	XLOC_133698	ENSRNOT00000077063	-0.957722613	9.91E-07
TUCP_003129	XLOC_133698	ENSRNOT00000075057	-0.969057611	2.12E-07
TUCP_003129	XLOC_133698	ENSRNOT00000011432	-0.953272459	1.62E-06
TUCP_003129	XLOC_133698	ENSRNOT00000021458	-0.973186697	1.04E-07
TUCP_003129	XLOC_133698	ENSRNOT00000086559	-0.970677056	1.63E-07
TUCP_003129	XLOC_133698	ENSRNOT00000014384	-0.953922038	1.51E-06
TUCP_003129	XLOC_133698	ENSRNOT00000016455	-0.971853148	1.33E-07
TUCP_003129	XLOC_133698	ENSRNOT00000022432	-0.953805665	1.53E-06
TUCP_003129	XLOC_133698	ENSRNOT00000001891	-0.955079581	1.34E-06
TUCP_003129	XLOC_133698	ENSRNOT00000014177	-0.960465526	7.12E-07

TUCP_003129	XLOC_133698	ENSRNOT00000026496	-0.956306093	1.17E-06
TUCP_003129	XLOC_133698	ENSRNOT00000034983	-0.963914551	4.54E-07
TUCP_003129	XLOC_133698	ENSRNOT00000056644	-0.981370673	1.71E-08
TUCP_003129	XLOC_133698	ENSRNOT00000005686	-0.951617294	1.92E-06
TUCP_003129	XLOC_133698	ENSRNOT00000022637	-0.974285689	8.48E-08
TUCP_003129	XLOC_133698	ENSRNOT00000052098	-0.9553955	1.29E-06
TUCP_003129	XLOC_133698	ENSRNOT00000076349	0.967260765	2.80E-07
TUCP_003129	XLOC_133698	ENSRNOT00000006607	-0.958432893	9.11E-07
TUCP_003129	XLOC_133698	ENSRNOT00000046490	-0.951289085	1.99E-06
TUCP_003129	XLOC_133698	ENSRNOT00000001973	-0.979707534	2.62E-08
TUCP_003129	XLOC_133698	ENSRNOT00000072841	-0.955982934	1.21E-06
TUCP_003129	XLOC_133698	ENSRNOT00000024801	-0.964884243	3.96E-07
TUCP_003129	XLOC_133698	ENSRNOT00000009544	-0.962899594	5.20E-07
TUCP_003129	XLOC_133698	ENSRNOT00000082124	-0.955100986	1.33E-06
TUCP_003129	XLOC_133698	ENSRNOT00000000203	-0.964910229	3.95E-07
TUCP_003129	XLOC_133698	ENSRNOT00000030706	-0.953188352	1.64E-06
TUCP_003129	XLOC_133698	ENSRNOT00000085651	-0.977574753	4.30E-08
TUCP_003129	XLOC_133698	ENSRNOT00000020316	-0.970036185	1.81E-07
TUCP_003129	XLOC_133698	ENSRNOT00000090156	-0.95692831	1.09E-06
TUCP_003129	XLOC_133698	ENSRNOT00000000469	0.959808492	7.72E-07
TUCP_003129	XLOC_133698	ENSRNOT00000014694	-0.971679702	1.37E-07
TUCP_003129	XLOC_133698	ENSRNOT00000007326	-0.956527106	1.14E-06
TUCP_003129	XLOC_133698	ENSRNOT00000028468	-0.95654793	1.13E-06
TUCP_003129	XLOC_133698	ENSRNOT00000030823	-0.962555749	5.44E-07
TUCP_003129	XLOC_133698	ENSRNOT00000082627	-0.955621914	1.26E-06
TUCP_003129	XLOC_133698	ENSRNOT00000006306	-0.966542381	3.12E-07
TUCP_003129	XLOC_133698	ENSRNOT00000014502	-0.971517287	1.41E-07
TUCP_003129	XLOC_133698	ENSRNOT00000035513	-0.951596549	1.93E-06
TUCP_003129	XLOC_133698	ENSRNOT00000011898	-0.951604955	1.93E-06
TUCP_003129	XLOC_133698	ENSRNOT00000006308	-0.970852014	1.58E-07
TUCP_003129	XLOC_133698	ENSRNOT00000074515	-0.950898722	2.07E-06
TUCP_003129	XLOC_133698	ENSRNOT00000013778	-0.972500721	1.18E-07
TUCP_003129	XLOC_133698	ENSRNOT00000077784	-0.981015425	1.88E-08
TUCP_003129	XLOC_133698	ENSRNOT00000007882	-0.951135687	2.02E-06
TUCP_003129	XLOC_133698	ENSRNOT00000006946	-0.962856527	5.23E-07
TUCP_003129	XLOC_133698	ENSRNOT00000025104	-0.950225195	2.21E-06
TUCP_003129	XLOC_133698	ENSRNOT00000086576	-0.959209848	8.30E-07
TUCP_003129	XLOC_133698	ENSRNOT00000015759	-0.958985106	8.53E-07
TUCP_003129	XLOC_133698	ENSRNOT00000072458	-0.965672377	3.54E-07
TUCP_003129	XLOC_133698	ENSRNOT00000009437	-0.956967453	1.08E-06
TUCP_003129	XLOC_133698	ENSRNOT00000081037	-0.950216524	2.21E-06
TUCP_003129	XLOC_133698	ENSRNOT00000068026	-0.952393048	1.78E-06
TUCP_003129	XLOC_133698	ENSRNOT00000065023	-0.950254149	2.21E-06
TUCP_003129	XLOC_133698	ENSRNOT00000068440	-0.951898858	1.87E-06
TUCP_003129	XLOC_133698	ENSRNOT00000020558	-0.957233484	1.05E-06
TUCP_003129	XLOC_133698	ENSRNOT00000016699	-0.95522642	1.31E-06
TUCP_003129	XLOC_133698	ENSRNOT00000017746	-0.951999651	1.85E-06
TUCP_003129	XLOC_133698	ENSRNOT00000083983	-0.978725622	3.31E-08
TUCP_003129	XLOC_133698	ENSRNOT00000007549	-0.97252956	1.18E-07
TUCP_003129	XLOC_133698	ENSRNOT00000005343	-0.95548706	1.28E-06
TUCP_003129	XLOC_133698	ENSRNOT00000014586	-0.953359459	1.61E-06

TUCP_003129	XLOC_133698	ENSRNOT00000025634	-0.951691872	1.91E-06
TUCP_003129	XLOC_133698	ENSRNOT00000047303	0.959659998	7.86E-07
TUCP_003129	XLOC_133698	ENSRNOT00000072580	-0.968744625	2.23E-07
TUCP_003129	XLOC_133698	ENSRNOT00000024452	-0.955053711	1.34E-06
TUCP_003129	XLOC_133698	ENSRNOT00000001631	-0.976109476	5.89E-08
TUCP_003129	XLOC_133698	ENSRNOT00000015139	-0.955894407	1.22E-06
TUCP_003129	XLOC_133698	ENSRNOT00000026748	-0.95306113	1.66E-06
TUCP_003129	XLOC_133698	ENSRNOT00000001639	-0.95526098	1.31E-06
TUCP_003129	XLOC_133698	ENSRNOT00000077730	-0.962467785	5.51E-07
TUCP_003129	XLOC_133698	ENSRNOT00000002511	-0.952374663	1.78E-06
TUCP_003129	XLOC_133698	ENSRNOT00000018877	-0.970547989	1.66E-07
TUCP_003129	XLOC_133698	ENSRNOT00000020982	-0.964653602	4.09E-07
TUCP_003129	XLOC_133698	ENSRNOT00000012017	-0.957474703	1.02E-06
TUCP_003129	XLOC_133698	ENSRNOT00000024714	-0.956841751	1.10E-06
TUCP_003129	XLOC_133698	ENSRNOT00000030222	-0.963105781	5.06E-07
TUCP_003129	XLOC_133698	ENSRNOT00000017339	-0.974163725	8.68E-08
TUCP_003129	XLOC_133698	ENSRNOT00000088752	-0.97576614	6.32E-08
TUCP_003129	XLOC_133698	ENSRNOT00000006029	-0.964672192	4.08E-07
TUCP_003129	XLOC_133698	ENSRNOT00000087342	-0.953117536	1.65E-06
TUCP_003129	XLOC_133698	ENSRNOT00000052033	-0.968403796	2.35E-07
TUCP_003129	XLOC_133698	ENSRNOT00000025036	-0.956186877	1.18E-06
TUCP_003129	XLOC_133698	ENSRNOT00000056862	-0.966920826	2.95E-07
TUCP_003129	XLOC_133698	ENSRNOT00000050227	-0.972834479	1.11E-07
TUCP_003129	XLOC_133698	ENSRNOT00000040202	-0.952362123	1.78E-06
TUCP_003129	XLOC_133698	ENSRNOT00000042459	-0.954525175	1.42E-06
TUCP_003129	XLOC_133698	ENSRNOT00000012432	-0.977325576	4.54E-08
TUCP_003129	XLOC_133698	ENSRNOT00000080216	-0.964677427	4.08E-07
TUCP_003129	XLOC_133698	ENSRNOT00000023463	-0.950642112	2.12E-06
TUCP_003129	XLOC_133698	ENSRNOT00000088884	-0.966349502	3.21E-07
TUCP_003129	XLOC_133698	ENSRNOT00000076388	-0.972697029	1.14E-07
TUCP_003129	XLOC_133698	ENSRNOT00000064542	-0.969470647	1.98E-07
TUCP_003129	XLOC_133698	ENSRNOT00000005155	0.967672491	2.63E-07
TUCP_003129	XLOC_133698	ENSRNOT00000002111	-0.95887743	8.64E-07
TUCP_003129	XLOC_133698	ENSRNOT00000091541	-0.973887864	9.15E-08
TUCP_003129	XLOC_133698	ENSRNOT00000014095	-0.957897597	9.71E-07
TUCP_003129	XLOC_133698	ENSRNOT00000038994	-0.975255734	7.01E-08
TUCP_003129	XLOC_133698	ENSRNOT00000072733	-0.956593879	1.13E-06
TUCP_003129	XLOC_133698	ENSRNOT00000091965	-0.960326194	7.24E-07
TUCP_003129	XLOC_133698	ENSRNOT00000089885	-0.966303334	3.23E-07
TUCP_003129	XLOC_133698	ENSRNOT00000017618	-0.965175831	3.80E-07
TUCP_003129	XLOC_133698	ENSRNOT00000024924	-0.95920648	8.31E-07
TUCP_003129	XLOC_133698	ENSRNOT00000048529	-0.954545126	1.42E-06
TUCP_003129	XLOC_133698	ENSRNOT00000079085	-0.973395516	1.00E-07
TUCP_003129	XLOC_133698	ENSRNOT00000015130	-0.955131428	1.33E-06
TUCP_003129	XLOC_133698	ENSRNOT00000038652	-0.972498526	1.18E-07
TUCP_003129	XLOC_133698	ENSRNOT00000013535	-0.953976757	1.50E-06
TUCP_003129	XLOC_133698	ENSRNOT00000023543	-0.950091059	2.24E-06
TUCP_003129	XLOC_133698	ENSRNOT00000089016	-0.954646919	1.40E-06
TUCP_003129	XLOC_133698	ENSRNOT00000000737	-0.972971616	1.09E-07
TUCP_003129	XLOC_133698	ENSRNOT00000010906	-0.950322919	2.19E-06
TUCP_003129	XLOC_133698	ENSRNOT00000028900	-0.953107048	1.65E-06

TUCP_003129	XLOC_133698	ENSRNOT00000002524	-0.954338762	1.45E-06
TUCP_003129	XLOC_133698	ENSRNOT00000016376	-0.966704067	3.05E-07
TUCP_003129	XLOC_133698	ENSRNOT00000072722	-0.955696517	1.25E-06
TUCP_003129	XLOC_133698	ENSRNOT00000020900	-0.952969727	1.67E-06
TUCP_003129	XLOC_133698	ENSRNOT00000025928	-0.979182397	2.97E-08
TUCP_003129	XLOC_133698	ENSRNOT00000087556	-0.962509678	5.48E-07
TUCP_003129	XLOC_133698	ENSRNOT00000038464	-0.952186804	1.82E-06
TUCP_003129	XLOC_133698	ENSRNOT00000018684	-0.960565368	7.03E-07
TUCP_003129	XLOC_133698	ENSRNOT00000088073	-0.953263826	1.62E-06
TUCP_003129	XLOC_133698	ENSRNOT00000065642	-0.951192419	2.01E-06
TUCP_003129	XLOC_133698	ENSRNOT00000092641	-0.950798213	2.09E-06
TUCP_004584	XLOC_201051	ENSRNOT00000025052	0.981289184	1.75E-08
TUCP_004584	XLOC_201051	ENSRNOT00000067655	0.952643953	1.73E-06
TUCP_004584	XLOC_201051	ENSRNOT00000093612	0.96145405	6.28E-07
TUCP_004584	XLOC_201051	ENSRNOT00000092261	0.974503312	8.13E-08
TUCP_004239	XLOC_186262	ENSRNOT00000027611	0.961440816	6.29E-07
TUCP_004239	XLOC_186262	ENSRNOT00000067761	0.950071683	2.25E-06
TUCP_004239	XLOC_186262	ENSRNOT00000041908	0.962326861	5.61E-07
TUCP_004239	XLOC_186262	ENSRNOT00000021348	0.958830365	8.69E-07
TUCP_004239	XLOC_186262	ENSRNOT00000086336	0.952316772	1.79E-06
TUCP_004239	XLOC_186262	ENSRNOT00000021614	0.962872973	5.22E-07
TUCP_004239	XLOC_186262	ENSRNOT00000022726	0.966712897	3.04E-07
TUCP_004239	XLOC_186262	ENSRNOT00000044635	0.959588716	7.93E-07
TUCP_004239	XLOC_186262	ENSRNOT00000021116	0.979272187	2.91E-08
TUCP_004239	XLOC_186262	ENSRNOT00000082100	0.973572049	9.71E-08
TUCP_004239	XLOC_186262	ENSRNOT00000084601	0.955608867	1.26E-06
TUCP_004239	XLOC_186262	ENSRNOT00000081246	0.956150528	1.19E-06
TUCP_004239	XLOC_186262	ENSRNOT00000043216	0.966432136	3.17E-07
TUCP_004239	XLOC_186262	ENSRNOT00000027414	0.956433874	1.15E-06
TUCP_004239	XLOC_186262	ENSRNOT00000028563	0.971102826	1.51E-07
TUCP_004239	XLOC_186262	ENSRNOT00000015076	0.961504583	6.24E-07
TUCP_004239	XLOC_186262	ENSRNOT00000022800	0.960413049	7.16E-07
TUCP_004239	XLOC_186262	ENSRNOT00000015073	0.970432383	1.69E-07
TUCP_004239	XLOC_186262	ENSRNOT00000025832	0.954491986	1.42E-06
TUCP_004239	XLOC_186262	ENSRNOT00000012379	0.959632081	7.89E-07
TUCP_004239	XLOC_186262	ENSRNOT00000080465	0.971460601	1.42E-07
TUCP_004239	XLOC_186262	ENSRNOT00000036951	0.962510084	5.48E-07
TUCP_004239	XLOC_186262	ENSRNOT00000071764	0.951361763	1.98E-06
TUCP_004239	XLOC_186262	ENSRNOT00000074939	0.969413267	2.00E-07
TUCP_004239	XLOC_186262	ENSRNOT00000076453	0.956637142	1.12E-06
TUCP_004239	XLOC_186262	ENSRNOT00000032944	0.97483338	7.62E-08
TUCP_004239	XLOC_186262	ENSRNOT00000083215	0.961604839	6.16E-07
TUCP_004239	XLOC_186262	ENSRNOT00000055187	0.966103232	3.33E-07
TUCP_004239	XLOC_186262	ENSRNOT00000001809	0.987404526	2.44E-09
TUCP_004239	XLOC_186262	ENSRNOT00000081632	0.953791716	1.53E-06
TUCP_004239	XLOC_186262	ENSRNOT00000067822	0.961930849	5.91E-07
TUCP_004239	XLOC_186262	ENSRNOT00000010149	0.967237041	2.81E-07
TUCP_004239	XLOC_186262	ENSRNOT00000008018	0.950906806	2.07E-06
TUCP_004239	XLOC_186262	ENSRNOT00000004032	0.953485762	1.59E-06
TUCP_004239	XLOC_186262	ENSRNOT00000017948	0.96484919	3.98E-07
TUCP_004239	XLOC_186262	ENSRNOT00000012895	0.953056958	1.66E-06

TUCP_004239	XLOC_186262	ENSRNOT00000055401	0.980636196	2.08E-08
TUCP_004239	XLOC_186262	ENSRNOT00000066526	0.964548164	4.16E-07
TUCP_004239	XLOC_186262	ENSRNOT00000023180	0.960396467	7.18E-07
TUCP_004239	XLOC_186262	ENSRNOT00000039852	0.975117965	7.20E-08
TUCP_004239	XLOC_186262	ENSRNOT00000070815	0.982576068	1.23E-08
TUCP_004239	XLOC_186262	ENSRNOT00000004581	0.95602667	1.20E-06
TUCP_004239	XLOC_186262	ENSRNOT00000004359	0.97092366	1.56E-07
TUCP_004239	XLOC_186262	ENSRNOT00000013274	0.957348152	1.03E-06
TUCP_004239	XLOC_186262	ENSRNOT00000022610	0.96255904	5.44E-07
TUCP_004239	XLOC_186262	ENSRNOT00000020437	0.963528912	4.78E-07
TUCP_004239	XLOC_186262	ENSRNOT00000051720	0.959441272	8.07E-07
TUCP_004239	XLOC_186262	ENSRNOT00000009430	0.954351602	1.45E-06
TUCP_004239	XLOC_186262	ENSRNOT00000029419	0.951091011	2.03E-06
TUCP_004239	XLOC_186262	ENSRNOT00000027520	0.96367558	4.69E-07
TUCP_004239	XLOC_186262	ENSRNOT00000026174	0.951083392	2.03E-06
TUCP_004239	XLOC_186262	ENSRNOT00000047387	0.951108538	2.03E-06
TUCP_004239	XLOC_186262	ENSRNOT00000013661	0.982436426	1.28E-08
TUCP_004239	XLOC_186262	ENSRNOT00000058072	0.970213544	1.76E-07
TUCP_004239	XLOC_186262	ENSRNOT00000045083	0.970609092	1.64E-07
TUCP_004239	XLOC_186262	ENSRNOT00000017629	0.96327647	4.95E-07
TUCP_004239	XLOC_186262	ENSRNOT00000032199	0.959033046	8.48E-07
TUCP_004239	XLOC_186262	ENSRNOT00000063956	0.962453909	5.52E-07
TUCP_004239	XLOC_186262	ENSRNOT00000003991	0.960338211	7.23E-07
TUCP_004239	XLOC_186262	ENSRNOT00000004508	0.964370405	4.26E-07
TUCP_004239	XLOC_186262	ENSRNOT00000034744	0.975707001	6.40E-08
TUCP_004239	XLOC_186262	ENSRNOT00000021153	0.970511522	1.67E-07
TUCP_004239	XLOC_186262	ENSRNOT00000064902	0.958033505	9.55E-07
TUCP_004239	XLOC_186262	ENSRNOT00000025027	0.956931783	1.09E-06
TUCP_004239	XLOC_186262	ENSRNOT00000076729	0.955135725	1.33E-06
TUCP_004239	XLOC_186262	ENSRNOT00000036442	0.95905428	8.46E-07
TUCP_004239	XLOC_186262	ENSRNOT00000081897	0.95185666	1.88E-06
TUCP_004239	XLOC_186262	ENSRNOT00000074075	0.959075115	8.44E-07
TUCP_004239	XLOC_186262	ENSRNOT00000039631	0.984899377	6.03E-09
TUCP_004239	XLOC_186262	ENSRNOT00000027452	0.9557464	1.24E-06
TUCP_004239	XLOC_186262	ENSRNOT00000049538	0.977856056	4.04E-08
TUCP_004239	XLOC_186262	ENSRNOT00000000713	0.98300356	1.09E-08
TUCP_004239	XLOC_186262	ENSRNOT00000066736	0.96974051	1.90E-07
TUCP_004239	XLOC_186262	ENSRNOT00000009198	0.96203579	5.83E-07
TUCP_004239	XLOC_186262	ENSRNOT00000027054	0.972118455	1.27E-07
TUCP_004239	XLOC_186262	ENSRNOT00000009129	0.964457967	4.21E-07
TUCP_004239	XLOC_186262	ENSRNOT00000006963	0.960025447	7.52E-07
TUCP_004239	XLOC_186262	ENSRNOT00000011663	0.966111962	3.33E-07
TUCP_004239	XLOC_186262	ENSRNOT00000024420	0.962321349	5.61E-07
TUCP_004239	XLOC_186262	ENSRNOT00000000157	0.956638747	1.12E-06
TUCP_004239	XLOC_186262	ENSRNOT00000023645	0.977078983	4.79E-08
TUCP_004239	XLOC_186262	ENSRNOT00000084734	0.960008029	7.53E-07
TUCP_004239	XLOC_186262	ENSRNOT00000085596	0.959260869	8.25E-07
TUCP_004239	XLOC_186262	ENSRNOT00000020070	0.957777256	9.84E-07
TUCP_004239	XLOC_186262	ENSRNOT00000074653	0.957568106	1.01E-06
TUCP_004239	XLOC_186262	ENSRNOT00000031230	0.97687086	5.01E-08
TUCP_004239	XLOC_186262	ENSRNOT00000010588	0.950393702	2.18E-06

TUCP_004239	XLOC_186262	ENSRNOT00000002292	0.969271792	2.05E-07
TUCP_004239	XLOC_186262	ENSRNOT00000035491	0.956657577	1.12E-06
TUCP_004239	XLOC_186262	ENSRNOT00000009342	0.963998912	4.48E-07
TUCP_004239	XLOC_186262	ENSRNOT00000082174	0.953250069	1.63E-06
TUCP_004239	XLOC_186262	ENSRNOT00000004412	0.968421143	2.35E-07
TUCP_004239	XLOC_186262	ENSRNOT00000035255	0.963148484	5.03E-07
TUCP_004239	XLOC_186262	ENSRNOT00000024941	0.953612293	1.56E-06
TUCP_004239	XLOC_186262	ENSRNOT00000034704	0.975204631	7.08E-08
TUCP_004239	XLOC_186262	ENSRNOT00000013985	0.966894004	2.96E-07
TUCP_004239	XLOC_186262	ENSRNOT00000036750	0.971457027	1.42E-07
TUCP_004239	XLOC_186262	ENSRNOT00000027964	0.955807562	1.23E-06
TUCP_004239	XLOC_186262	ENSRNOT00000060669	0.967717591	2.62E-07
TUCP_004239	XLOC_186262	ENSRNOT00000024659	0.962089441	5.79E-07
TUCP_004239	XLOC_186262	ENSRNOT00000072081	0.964274517	4.32E-07
TUCP_004239	XLOC_186262	ENSRNOT00000029001	0.960690089	6.92E-07
TUCP_004239	XLOC_186262	ENSRNOT00000010433	0.959590507	7.93E-07
TUCP_004239	XLOC_186262	ENSRNOT00000083799	0.953346229	1.61E-06
TUCP_004239	XLOC_186262	ENSRNOT00000009066	0.976316793	5.64E-08
TUCP_003918	XLOC_172539	ENSRNOT00000028833	0.954267715	1.46E-06
TUCP_003918	XLOC_172539	ENSRNOT00000045223	0.957211513	1.05E-06
TUCP_003918	XLOC_172539	ENSRNOT00000041044	0.958440737	9.10E-07
TUCP_003918	XLOC_172539	ENSRNOT00000065600	0.961528124	6.22E-07
TUCP_003918	XLOC_172539	ENSRNOT00000057160	0.956462977	1.14E-06
TUCP_003918	XLOC_172539	ENSRNOT00000066024	0.956581302	1.13E-06
TUCP_003918	XLOC_172539	ENSRNOT00000022419	0.958838159	8.68E-07
TUCP_003918	XLOC_172539	ENSRNOT00000060499	0.959174337	8.34E-07
TUCP_004583	XLOC_201051	ENSRNOT00000065387	0.972014574	1.29E-07
TUCP_004582	XLOC_200952	ENSRNOT00000048263	0.950074071	2.25E-06
TUCP_004582	XLOC_200952	ENSRNOT00000064936	0.974604288	7.97E-08
TUCP_004582	XLOC_200952	ENSRNOT00000081455	0.990562861	5.80E-10
TUCP_004582	XLOC_200952	ENSRNOT00000034090	0.996989789	1.94E-12
TUCP_004582	XLOC_200952	ENSRNOT00000015840	0.955557163	1.27E-06
TUCP_000362	XLOC_012011	ENSRNOT00000025097	0.953595402	1.57E-06
TUCP_000362	XLOC_012011	ENSRNOT00000071780	0.957748414	9.88E-07
TUCP_000362	XLOC_012011	ENSRNOT00000075521	0.988812619	1.35E-09
TUCP_000362	XLOC_012011	ENSRNOT00000084046	0.957555228	1.01E-06
TUCP_000362	XLOC_012011	ENSRNOT00000063865	0.967551613	2.68E-07
TUCP_000362	XLOC_012011	ENSRNOT00000000577	0.959703878	7.82E-07
TUCP_000362	XLOC_012011	ENSRNOT00000067610	-0.96694787	2.94E-07
TUCP_000362	XLOC_012011	ENSRNOT00000020174	-0.95035127	2.19E-06
TUCP_000362	XLOC_012011	ENSRNOT00000025434	0.969313413	2.04E-07
TUCP_000362	XLOC_012011	ENSRNOT00000018122	0.951123235	2.02E-06
TUCP_000362	XLOC_012011	ENSRNOT00000028888	0.968837581	2.20E-07
TUCP_000362	XLOC_012011	ENSRNOT00000017571	-0.959816889	7.71E-07
TUCP_000362	XLOC_012011	ENSRNOT00000083968	-0.954552613	1.41E-06
TUCP_000362	XLOC_012011	ENSRNOT00000019487	0.961818161	5.99E-07
TUCP_000362	XLOC_012011	ENSRNOT00000006835	0.956836049	1.10E-06
TUCP_000362	XLOC_012011	ENSRNOT00000009691	0.956727347	1.11E-06
TUCP_000362	XLOC_012011	ENSRNOT00000064588	0.960008503	7.53E-07
TUCP_000362	XLOC_012011	ENSRNOT00000063917	-0.9522156	1.81E-06
TUCP_000286	XLOC_008670	ENSRNOT00000072396	0.952787008	1.71E-06

TUCP_000286	XLOC_008670	ENSRNOT00000061569	0.950794296	2.09E-06
TUCP_000286	XLOC_008670	ENSRNOT00000021590	0.961880168	5.95E-07
TUCP_000286	XLOC_008670	ENSRNOT00000091315	0.952027461	1.85E-06
TUCP_000286	XLOC_008670	ENSRNOT00000065111	0.976190542	5.79E-08
TUCP_002979	XLOC_128384	ENSRNOT00000091542	-0.955643056	1.26E-06
TUCP_001404	XLOC_054315	ENSRNOT00000071840	0.963430539	4.84E-07
TUCP_001404	XLOC_054315	ENSRNOT00000066382	0.954464879	1.43E-06
TUCP_001404	XLOC_054315	ENSRNOT00000045584	0.963430539	4.84E-07
TUCP_001404	XLOC_054315	ENSRNOT00000077995	0.956356609	1.16E-06
TUCP_001404	XLOC_054315	ENSRNOT00000066004	0.962632034	5.39E-07
TUCP_001404	XLOC_054315	ENSRNOT00000075836	0.963430539	4.84E-07
TUCP_001404	XLOC_054315	ENSRNOT00000082086	0.950730988	2.10E-06
TUCP_001404	XLOC_054315	ENSRNOT00000092186	0.981734922	1.55E-08
TUCP_001404	XLOC_054315	ENSRNOT00000092182	0.963430539	4.84E-07
TUCP_001404	XLOC_054315	ENSRNOT00000084461	0.963430539	4.84E-07
TUCP_001404	XLOC_054315	ENSRNOT00000060448	0.963430539	4.84E-07
TUCP_001404	XLOC_054315	ENSRNOT00000032631	0.963430539	4.84E-07
TUCP_001404	XLOC_054315	ENSRNOT00000057176	0.963390786	4.87E-07
TUCP_001404	XLOC_054315	ENSRNOT00000078176	0.955143699	1.33E-06
TUCP_001404	XLOC_054315	ENSRNOT00000056364	0.963626039	4.72E-07
TUCP_001404	XLOC_054315	ENSRNOT00000093431	0.963430539	4.84E-07
TUCP_001404	XLOC_054315	ENSRNOT00000092901	0.963430539	4.84E-07
TUCP_001404	XLOC_054315	ENSRNOT00000089290	0.963883337	4.55E-07
TUCP_001404	XLOC_054315	ENSRNOT00000090211	0.963064519	5.09E-07
TUCP_001404	XLOC_054315	ENSRNOT00000091828	0.970989118	1.54E-07
TUCP_001404	XLOC_054315	ENSRNOT00000091944	0.96223357	5.68E-07
TUCP_001404	XLOC_054315	ENSRNOT00000070855	0.963430539	4.84E-07
TUCP_001404	XLOC_054315	ENSRNOT00000084109	0.961979476	5.87E-07
TUCP_001404	XLOC_054315	ENSRNOT00000007683	0.96437743	4.26E-07
TUCP_001404	XLOC_054315	ENSRNOT00000060645	0.965543622	3.61E-07
TUCP_001404	XLOC_054315	ENSRNOT00000041569	0.963430539	4.84E-07
TUCP_001404	XLOC_054315	ENSRNOT00000087091	0.963430539	4.84E-07
TUCP_001404	XLOC_054315	ENSRNOT00000003308	0.960441534	7.14E-07
TUCP_001404	XLOC_054315	ENSRNOT00000026113	0.962825579	5.25E-07
TUCP_001404	XLOC_054315	ENSRNOT00000014939	0.961034152	6.63E-07
TUCP_001404	XLOC_054315	ENSRNOT00000058314	0.951038519	2.04E-06
TUCP_001404	XLOC_054315	ENSRNOT00000047870	0.978001456	3.91E-08
TUCP_001404	XLOC_054315	ENSRNOT00000071603	0.963349604	4.90E-07
TUCP_001404	XLOC_054315	ENSRNOT00000088376	0.98541349	5.07E-09
TUCP_001404	XLOC_054315	ENSRNOT00000039313	0.970374431	1.71E-07
TUCP_001404	XLOC_054315	ENSRNOT00000044232	0.993825175	7.00E-11
TUCP_001404	XLOC_054315	ENSRNOT00000023287	0.952694994	1.72E-06
TUCP_001404	XLOC_054315	ENSRNOT00000073229	0.963430539	4.84E-07
TUCP_001404	XLOC_054315	ENSRNOT00000060454	0.963430539	4.84E-07
TUCP_001404	XLOC_054315	ENSRNOT00000009295	0.957040227	1.07E-06
TUCP_003814	XLOC_166902	ENSRNOT00000087408	0.950243435	2.21E-06
TUCP_003814	XLOC_166902	ENSRNOT00000011659	0.961493265	6.25E-07
TUCP_003814	XLOC_166902	ENSRNOT00000009473	0.955710345	1.25E-06
TUCP_003814	XLOC_166902	ENSRNOT00000022286	0.963601679	4.73E-07
TUCP_003814	XLOC_166902	ENSRNOT00000009718	0.98208756	1.41E-08
TUCP_003814	XLOC_166902	ENSRNOT00000024110	0.966448751	3.16E-07

TUCP_003814	XLOC_166902	ENSRNOT00000004400	0.956128925	1.19E-06
TUCP_003814	XLOC_166902	ENSRNOT00000073703	-0.953376562	1.60E-06
TUCP_003814	XLOC_166902	ENSRNOT00000020726	0.952685344	1.72E-06
TUCP_003814	XLOC_166902	ENSRNOT00000011290	0.965316444	3.73E-07
TUCP_003814	XLOC_166902	ENSRNOT00000090784	-0.968317907	2.38E-07
TUCP_003814	XLOC_166902	ENSRNOT00000031047	0.960905211	6.73E-07
TUCP_003814	XLOC_166902	ENSRNOT00000018952	0.959564952	7.95E-07
TUCP_003814	XLOC_166902	ENSRNOT00000044087	0.993495154	9.07E-11
TUCP_004188	XLOC_183726	ENSRNOT00000045677	0.950884811	2.07E-06
TUCP_004188	XLOC_183726	ENSRNOT00000011342	0.95017073	2.22E-06
TUCP_004188	XLOC_183726	ENSRNOT00000081235	0.954443716	1.43E-06
TUCP_004188	XLOC_183726	ENSRNOT00000076466	0.954737822	1.39E-06
TUCP_004188	XLOC_183726	ENSRNOT00000006604	0.963866167	4.57E-07
TUCP_004188	XLOC_183726	ENSRNOT00000091315	0.953401525	1.60E-06
TUCP_000184	XLOC_006006	ENSRNOT00000016581	0.952570784	1.75E-06
TUCP_000187	XLOC_006154	ENSRNOT00000026860	0.952423911	1.77E-06
TUCP_000187	XLOC_006154	ENSRNOT00000006186	0.980514157	2.14E-08
TUCP_000187	XLOC_006154	ENSRNOT00000026941	0.960220198	7.34E-07
TUCP_000187	XLOC_006154	ENSRNOT00000009877	0.95504092	1.34E-06
TUCP_000187	XLOC_006154	ENSRNOT00000021348	0.97087107	1.57E-07
TUCP_000187	XLOC_006154	ENSRNOT00000068353	0.959285696	8.23E-07
TUCP_000187	XLOC_006154	ENSRNOT00000021614	0.956621947	1.12E-06
TUCP_000187	XLOC_006154	ENSRNOT00000044635	0.987996156	1.92E-09
TUCP_000187	XLOC_006154	ENSRNOT00000082100	0.975646969	6.48E-08
TUCP_000187	XLOC_006154	ENSRNOT00000086310	-0.950028063	2.26E-06
TUCP_000187	XLOC_006154	ENSRNOT00000028856	0.950955773	2.06E-06
TUCP_000187	XLOC_006154	ENSRNOT00000052063	0.961637821	6.13E-07
TUCP_000187	XLOC_006154	ENSRNOT00000055540	0.956658399	1.12E-06
TUCP_000187	XLOC_006154	ENSRNOT00000006752	0.967192145	2.83E-07
TUCP_000187	XLOC_006154	ENSRNOT00000065300	0.980800469	1.99E-08
TUCP_000187	XLOC_006154	ENSRNOT00000019923	0.957737384	9.89E-07
TUCP_000187	XLOC_006154	ENSRNOT00000004400	0.969445854	1.99E-07
TUCP_000187	XLOC_006154	ENSRNOT00000035320	-0.950438154	2.17E-06
TUCP_000187	XLOC_006154	ENSRNOT00000076820	0.979711949	2.62E-08
TUCP_000187	XLOC_006154	ENSRNOT00000036951	0.96881503	2.20E-07
TUCP_000187	XLOC_006154	ENSRNOT00000022534	0.95702831	1.07E-06
TUCP_000187	XLOC_006154	ENSRNOT00000077999	0.961968111	5.88E-07
TUCP_000187	XLOC_006154	ENSRNOT00000068134	0.96022974	7.33E-07
TUCP_000187	XLOC_006154	ENSRNOT00000003146	-0.953706697	1.55E-06
TUCP_000187	XLOC_006154	ENSRNOT00000083215	0.972587302	1.16E-07
TUCP_000187	XLOC_006154	ENSRNOT00000085228	0.964478921	4.20E-07
TUCP_000187	XLOC_006154	ENSRNOT00000006969	0.966529435	3.13E-07
TUCP_000187	XLOC_006154	ENSRNOT00000017319	0.965680828	3.54E-07
TUCP_000187	XLOC_006154	ENSRNOT00000055187	0.981543719	1.64E-08
TUCP_000187	XLOC_006154	ENSRNOT00000015188	0.972145057	1.26E-07
TUCP_000187	XLOC_006154	ENSRNOT00000079866	0.965857328	3.45E-07
TUCP_000187	XLOC_006154	ENSRNOT00000005686	-0.9504567	2.16E-06
TUCP_000187	XLOC_006154	ENSRNOT00000010149	0.958453724	9.09E-07
TUCP_000187	XLOC_006154	ENSRNOT00000087371	0.956633144	1.12E-06
TUCP_000187	XLOC_006154	ENSRNOT00000037446	0.960825971	6.80E-07
TUCP_000187	XLOC_006154	ENSRNOT00000023619	0.950703186	2.11E-06

TUCP_000187	XLOC_006154	ENSRNOT00000084709	0.955850618	1.23E-06
TUCP_000187	XLOC_006154	ENSRNOT00000068033	0.957952451	9.64E-07
TUCP_000187	XLOC_006154	ENSRNOT00000009156	0.960188019	7.37E-07
TUCP_000187	XLOC_006154	ENSRNOT00000026481	0.974477136	8.17E-08
TUCP_000187	XLOC_006154	ENSRNOT00000001403	0.955278169	1.31E-06
TUCP_000187	XLOC_006154	ENSRNOT00000088196	0.968223946	2.42E-07
TUCP_000187	XLOC_006154	ENSRNOT00000016132	-0.960192798	7.36E-07
TUCP_000187	XLOC_006154	ENSRNOT00000013067	-0.951363612	1.97E-06
TUCP_000187	XLOC_006154	ENSRNOT00000034692	-0.958044274	9.54E-07
TUCP_000187	XLOC_006154	ENSRNOT00000088191	0.978220939	3.72E-08
TUCP_000187	XLOC_006154	ENSRNOT00000031979	-0.966334868	3.22E-07
TUCP_000187	XLOC_006154	ENSRNOT00000079895	0.985429798	5.05E-09
TUCP_000187	XLOC_006154	ENSRNOT00000078102	0.956509041	1.14E-06
TUCP_000187	XLOC_006154	ENSRNOT00000013661	0.968642038	2.27E-07
TUCP_000187	XLOC_006154	ENSRNOT00000081047	0.954885261	1.36E-06
TUCP_000187	XLOC_006154	ENSRNOT00000011880	0.95499026	1.35E-06
TUCP_000187	XLOC_006154	ENSRNOT00000020885	0.97250311	1.18E-07
TUCP_000187	XLOC_006154	ENSRNOT00000003991	0.958369934	9.18E-07
TUCP_000187	XLOC_006154	ENSRNOT00000016649	0.951348265	1.98E-06
TUCP_000187	XLOC_006154	ENSRNOT00000021153	0.974371543	8.34E-08
TUCP_000187	XLOC_006154	ENSRNOT00000077701	0.957620401	1.00E-06
TUCP_000187	XLOC_006154	ENSRNOT00000064902	0.960895409	6.74E-07
TUCP_000187	XLOC_006154	ENSRNOT00000025027	0.954371228	1.44E-06
TUCP_000187	XLOC_006154	ENSRNOT00000078070	0.961407072	6.32E-07
TUCP_000187	XLOC_006154	ENSRNOT00000046745	0.968434248	2.34E-07
TUCP_000187	XLOC_006154	ENSRNOT00000021015	0.971572178	1.39E-07
TUCP_000187	XLOC_006154	ENSRNOT00000076669	0.951867462	1.88E-06
TUCP_000187	XLOC_006154	ENSRNOT00000003840	0.968114186	2.46E-07
TUCP_000187	XLOC_006154	ENSRNOT00000090170	0.95036162	2.18E-06
TUCP_000187	XLOC_006154	ENSRNOT00000091488	0.959510683	8.01E-07
TUCP_000187	XLOC_006154	ENSRNOT00000027452	0.955329986	1.30E-06
TUCP_000187	XLOC_006154	ENSRNOT00000019656	-0.971527755	1.40E-07
TUCP_000187	XLOC_006154	ENSRNOT00000032505	-0.953011888	1.67E-06
TUCP_000187	XLOC_006154	ENSRNOT00000074649	0.974363622	8.35E-08
TUCP_000187	XLOC_006154	ENSRNOT00000006963	0.954769823	1.38E-06
TUCP_000187	XLOC_006154	ENSRNOT00000060002	-0.952456327	1.77E-06
TUCP_000187	XLOC_006154	ENSRNOT00000066133	-0.961051741	6.61E-07
TUCP_000187	XLOC_006154	ENSRNOT00000085596	0.978574822	3.43E-08
TUCP_000187	XLOC_006154	ENSRNOT00000059458	0.956286425	1.17E-06
TUCP_000187	XLOC_006154	ENSRNOT00000055992	-0.95562096	1.26E-06
TUCP_000187	XLOC_006154	ENSRNOT00000064535	-0.960894729	6.74E-07
TUCP_000187	XLOC_006154	ENSRNOT00000010748	0.969701317	1.91E-07
TUCP_000187	XLOC_006154	ENSRNOT00000035474	0.970018998	1.81E-07
TUCP_000187	XLOC_006154	ENSRNOT00000091205	0.977344606	4.53E-08
TUCP_000187	XLOC_006154	ENSRNOT00000004787	-0.957146435	1.06E-06
TUCP_000187	XLOC_006154	ENSRNOT00000040069	0.968515813	2.31E-07
TUCP_000187	XLOC_006154	ENSRNOT00000014814	0.956263389	1.17E-06
TUCP_000187	XLOC_006154	ENSRNOT00000004412	0.97367678	9.52E-08
TUCP_000187	XLOC_006154	ENSRNOT00000066968	0.954990797	1.35E-06
TUCP_000187	XLOC_006154	ENSRNOT00000080207	0.957372298	1.03E-06
TUCP_000187	XLOC_006154	ENSRNOT00000026174	0.98201408	1.44E-08

TUCP_000187	XLOC_006154	ENSRNOT00000017995	0.953526963	1.58E-06
TUCP_000187	XLOC_006154	ENSRNOT00000034704	0.954249782	1.46E-06
TUCP_000187	XLOC_006154	ENSRNOT00000005144	0.971413776	1.43E-07
TUCP_000187	XLOC_006154	ENSRNOT00000026457	0.954925599	1.36E-06
TUCP_000187	XLOC_006154	ENSRNOT00000018998	0.964577585	4.14E-07
TUCP_000187	XLOC_006154	ENSRNOT00000004463	0.973130675	1.05E-07
TUCP_000187	XLOC_006154	ENSRNOT00000072081	0.954945734	1.36E-06
TUCP_000187	XLOC_006154	ENSRNOT00000008982	0.974509928	8.12E-08
TUCP_000187	XLOC_006154	ENSRNOT00000048029	0.974641029	7.92E-08
TUCP_000187	XLOC_006154	ENSRNOT00000024253	0.953435761	1.59E-06
TUCP_000187	XLOC_006154	ENSRNOT00000081796	0.968294248	2.39E-07
TUCP_000187	XLOC_006154	ENSRNOT00000074939	0.959447151	8.07E-07
TUCP_000187	XLOC_006154	ENSRNOT00000063930	-0.951225046	2.00E-06
TUCP_000187	XLOC_006154	ENSRNOT00000006055	0.952226788	1.81E-06
TUCP_000187	XLOC_006154	ENSRNOT00000020453	-0.952920585	1.68E-06
TUCP_000187	XLOC_006154	ENSRNOT00000019502	0.960435448	7.14E-07
TUCP_000187	XLOC_006154	ENSRNOT00000018057	0.973505415	9.83E-08
TUCP_000187	XLOC_006154	ENSRNOT00000038703	0.961110641	6.56E-07
TUCP_000187	XLOC_006154	ENSRNOT00000043216	0.975748227	6.34E-08
TUCP_000187	XLOC_006154	ENSRNOT00000081981	0.959104496	8.41E-07
TUCP_000187	XLOC_006154	ENSRNOT00000013758	0.9624932	5.49E-07
TUCP_000187	XLOC_006154	ENSRNOT00000028563	0.966912723	2.95E-07
TUCP_000187	XLOC_006154	ENSRNOT00000010418	0.973619301	9.63E-08
TUCP_000187	XLOC_006154	ENSRNOT00000015479	0.969848727	1.87E-07
TUCP_000187	XLOC_006154	ENSRNOT00000021629	-0.95884597	8.68E-07
TUCP_000187	XLOC_006154	ENSRNOT00000047836	-0.952607841	1.74E-06
TUCP_000187	XLOC_006154	ENSRNOT00000079746	0.966106511	3.33E-07
TUCP_000187	XLOC_006154	ENSRNOT00000025079	-0.965487232	3.64E-07
TUCP_000187	XLOC_006154	ENSRNOT00000048754	-0.969057362	2.12E-07
TUCP_000187	XLOC_006154	ENSRNOT00000079109	0.961501411	6.24E-07
TUCP_000187	XLOC_006154	ENSRNOT00000011926	0.966427046	3.17E-07
TUCP_000187	XLOC_006154	ENSRNOT00000054761	-0.958969374	8.55E-07
TUCP_000187	XLOC_006154	ENSRNOT00000007824	-0.952123539	1.83E-06
TUCP_000187	XLOC_006154	ENSRNOT00000052075	0.956295613	1.17E-06
TUCP_000187	XLOC_006154	ENSRNOT00000091602	-0.952547075	1.75E-06
TUCP_000187	XLOC_006154	ENSRNOT00000012671	0.953927618	1.51E-06
TUCP_000187	XLOC_006154	ENSRNOT00000035989	0.961239646	6.46E-07
TUCP_000187	XLOC_006154	ENSRNOT00000071540	0.968533869	2.30E-07
TUCP_000187	XLOC_006154	ENSRNOT00000022594	0.953042448	1.66E-06
TUCP_000187	XLOC_006154	ENSRNOT00000011654	-0.957751132	9.87E-07
TUCP_000187	XLOC_006154	ENSRNOT00000060811	0.951164845	2.01E-06
TUCP_000187	XLOC_006154	ENSRNOT00000087443	0.958688717	8.84E-07
TUCP_000187	XLOC_006154	ENSRNOT00000021803	-0.965062944	3.87E-07
TUCP_000187	XLOC_006154	ENSRNOT00000020147	0.960597884	7.00E-07
TUCP_000187	XLOC_006154	ENSRNOT00000024352	0.979203351	2.96E-08
TUCP_000187	XLOC_006154	ENSRNOT00000058312	0.957157352	1.06E-06
TUCP_000187	XLOC_006154	ENSRNOT00000013274	0.961017079	6.64E-07
TUCP_000187	XLOC_006154	ENSRNOT00000022610	0.951103705	2.03E-06
TUCP_000187	XLOC_006154	ENSRNOT00000022342	0.957238616	1.05E-06
TUCP_000187	XLOC_006154	ENSRNOT00000016739	0.966790846	3.01E-07
TUCP_000187	XLOC_006154	ENSRNOT00000009221	0.970496897	1.68E-07

TUCP_000187	XLOC_006154	ENSRNOT00000026089	0.964537381	4.16E-07
TUCP_000187	XLOC_006154	ENSRNOT00000005587	-0.961476998	6.26E-07
TUCP_000187	XLOC_006154	ENSRNOT00000061100	0.9787651	3.28E-08
TUCP_000187	XLOC_006154	ENSRNOT00000005073	0.960693863	6.92E-07
TUCP_000187	XLOC_006154	ENSRNOT00000065529	0.957486166	1.02E-06
TUCP_000187	XLOC_006154	ENSRNOT00000034744	0.97476963	7.72E-08
TUCP_000187	XLOC_006154	ENSRNOT00000017776	0.972346683	1.22E-07
TUCP_000187	XLOC_006154	ENSRNOT00000027910	0.987025742	2.83E-09
TUCP_000187	XLOC_006154	ENSRNOT00000090262	0.963387804	4.87E-07
TUCP_000187	XLOC_006154	ENSRNOT00000002771	0.957900773	9.70E-07
TUCP_000187	XLOC_006154	ENSRNOT00000039631	0.952853029	1.69E-06
TUCP_000187	XLOC_006154	ENSRNOT00000066032	0.956629153	1.12E-06
TUCP_000187	XLOC_006154	ENSRNOT00000047975	0.965610153	3.58E-07
TUCP_000187	XLOC_006154	ENSRNOT00000027615	0.971552908	1.40E-07
TUCP_000187	XLOC_006154	ENSRNOT00000034487	-0.950140786	2.23E-06
TUCP_000187	XLOC_006154	ENSRNOT00000001783	0.951355117	1.98E-06
TUCP_000187	XLOC_006154	ENSRNOT00000036061	0.95982769	7.70E-07
TUCP_000187	XLOC_006154	ENSRNOT00000057313	0.978953997	3.14E-08
TUCP_000187	XLOC_006154	ENSRNOT00000050138	0.980135657	2.36E-08
TUCP_000187	XLOC_006154	ENSRNOT00000003359	-0.956655194	1.12E-06
TUCP_000187	XLOC_006154	ENSRNOT00000011984	0.969829992	1.87E-07
TUCP_000187	XLOC_006154	ENSRNOT00000020669	0.97415836	8.69E-08
TUCP_000187	XLOC_006154	ENSRNOT00000074653	0.969619034	1.94E-07
TUCP_000187	XLOC_006154	ENSRNOT00000004570	0.952903424	1.69E-06
TUCP_000187	XLOC_006154	ENSRNOT00000031230	0.972546497	1.17E-07
TUCP_000187	XLOC_006154	ENSRNOT00000022142	0.955338561	1.30E-06
TUCP_000187	XLOC_006154	ENSRNOT00000076300	-0.957216238	1.05E-06
TUCP_000187	XLOC_006154	ENSRNOT00000025086	-0.960370321	7.20E-07
TUCP_000187	XLOC_006154	ENSRNOT00000002292	0.972360835	1.21E-07
TUCP_000187	XLOC_006154	ENSRNOT00000052120	0.956873076	1.09E-06
TUCP_000187	XLOC_006154	ENSRNOT00000019820	0.967340267	2.77E-07
TUCP_000187	XLOC_006154	ENSRNOT00000004172	0.965161548	3.81E-07
TUCP_000187	XLOC_006154	ENSRNOT00000029125	0.974653534	7.90E-08
TUCP_000187	XLOC_006154	ENSRNOT00000085279	0.957751743	9.87E-07
TUCP_000187	XLOC_006154	ENSRNOT00000074847	-0.952134901	1.83E-06
TUCP_000187	XLOC_006154	ENSRNOT00000046058	0.960048471	7.50E-07
TUCP_000187	XLOC_006154	ENSRNOT00000005389	0.963385863	4.87E-07
TUCP_000187	XLOC_006154	ENSRNOT00000010927	0.984304368	7.31E-09
TUCP_000187	XLOC_006154	ENSRNOT00000077888	-0.972430576	1.20E-07
TUCP_000187	XLOC_006154	ENSRNOT00000015092	0.958181136	9.39E-07
TUCP_000187	XLOC_006154	ENSRNOT00000003182	0.952754955	1.71E-06
TUCP_000187	XLOC_006154	ENSRNOT00000010224	0.962890147	5.21E-07
TUCP_000187	XLOC_006154	ENSRNOT00000025147	0.967709507	2.62E-07
TUCP_000187	XLOC_006154	ENSRNOT00000087278	-0.962486973	5.49E-07
TUCP_000187	XLOC_006154	ENSRNOT00000007175	0.974793554	7.68E-08
TUCP_000187	XLOC_006154	ENSRNOT00000078862	0.955399984	1.29E-06
TUCP_000187	XLOC_006154	ENSRNOT00000039551	-0.959029731	8.49E-07
TUCP_000187	XLOC_006154	ENSRNOT00000082998	0.976549165	5.37E-08
TUCP_000187	XLOC_006154	ENSRNOT00000007350	0.958976293	8.54E-07
TUCP_000187	XLOC_006154	ENSRNOT00000007351	-0.969680577	1.92E-07
TUCP_000187	XLOC_006154	ENSRNOT00000083578	0.972262609	1.23E-07

TUCP_000187	XLOC_006154	ENSRNOT00000026065	0.975463884	6.72E-08
TUCP_000187	XLOC_006154	ENSRNOT00000023638	0.975917479	6.13E-08
TUCP_000187	XLOC_006154	ENSRNOT00000005228	0.950793009	2.09E-06
TUCP_000187	XLOC_006154	ENSRNOT00000022726	0.977392577	4.48E-08
TUCP_000187	XLOC_006154	ENSRNOT00000005220	0.974315324	8.43E-08
TUCP_000187	XLOC_006154	ENSRNOT00000045326	0.962112693	5.77E-07
TUCP_000187	XLOC_006154	ENSRNOT00000092418	0.950079584	2.24E-06
TUCP_000187	XLOC_006154	ENSRNOT00000091128	-0.957710373	9.92E-07
TUCP_000187	XLOC_006154	ENSRNOT00000043554	0.950296889	2.20E-06
TUCP_000187	XLOC_006154	ENSRNOT00000028405	-0.952722505	1.72E-06
TUCP_000187	XLOC_006154	ENSRNOT00000013695	-0.954474178	1.43E-06
TUCP_000187	XLOC_006154	ENSRNOT00000088125	0.975311257	6.93E-08
TUCP_000187	XLOC_006154	ENSRNOT00000038247	0.956618388	1.12E-06
TUCP_000187	XLOC_006154	ENSRNOT00000027944	0.961334778	6.38E-07
TUCP_000187	XLOC_006154	ENSRNOT00000024402	0.960176273	7.38E-07
TUCP_000187	XLOC_006154	ENSRNOT00000024406	-0.95454645	1.42E-06
TUCP_000187	XLOC_006154	ENSRNOT00000019519	0.955667639	1.25E-06
TUCP_000187	XLOC_006154	ENSRNOT00000089933	0.957081809	1.07E-06
TUCP_000187	XLOC_006154	ENSRNOT00000077908	0.977164452	4.71E-08
TUCP_000187	XLOC_006154	ENSRNOT00000010463	0.962292482	5.64E-07
TUCP_000187	XLOC_006154	ENSRNOT00000084012	0.972566773	1.17E-07
TUCP_000187	XLOC_006154	ENSRNOT00000074323	0.964570561	4.14E-07
TUCP_000187	XLOC_006154	ENSRNOT00000033677	-0.962461627	5.51E-07
TUCP_000187	XLOC_006154	ENSRNOT00000036931	-0.954978052	1.35E-06
TUCP_000187	XLOC_006154	ENSRNOT00000067822	0.982682538	1.19E-08
TUCP_000187	XLOC_006154	ENSRNOT00000024060	0.978491689	3.50E-08
TUCP_000187	XLOC_006154	ENSRNOT00000011784	0.962931843	5.18E-07
TUCP_000187	XLOC_006154	ENSRNOT00000051181	0.96154364	6.21E-07
TUCP_000187	XLOC_006154	ENSRNOT00000077727	-0.957191261	1.05E-06
TUCP_000187	XLOC_006154	ENSRNOT00000068745	0.966840517	2.99E-07
TUCP_000187	XLOC_006154	ENSRNOT00000004032	0.979203099	2.96E-08
TUCP_000187	XLOC_006154	ENSRNOT00000048613	0.98398412	8.08E-09
TUCP_000187	XLOC_006154	ENSRNOT00000017948	0.956907138	1.09E-06
TUCP_000187	XLOC_006154	ENSRNOT00000091909	0.977304883	4.56E-08
TUCP_000187	XLOC_006154	ENSRNOT00000001081	0.966863145	2.98E-07
TUCP_000187	XLOC_006154	ENSRNOT00000086119	-0.962322941	5.61E-07
TUCP_000187	XLOC_006154	ENSRNOT00000077220	-0.953563073	1.57E-06
TUCP_000187	XLOC_006154	ENSRNOT00000065983	0.966011042	3.37E-07
TUCP_000187	XLOC_006154	ENSRNOT00000022213	0.969734449	1.90E-07
TUCP_000187	XLOC_006154	ENSRNOT00000073919	0.953181493	1.64E-06
TUCP_000187	XLOC_006154	ENSRNOT00000067780	-0.95423106	1.46E-06
TUCP_000187	XLOC_006154	ENSRNOT00000015609	0.954937057	1.36E-06
TUCP_000187	XLOC_006154	ENSRNOT00000086590	0.972856002	1.11E-07
TUCP_000187	XLOC_006154	ENSRNOT00000025983	0.953739711	1.54E-06
TUCP_000187	XLOC_006154	ENSRNOT00000020155	0.950683309	2.11E-06
TUCP_000187	XLOC_006154	ENSRNOT00000015325	-0.95394165	1.51E-06
TUCP_000187	XLOC_006154	ENSRNOT00000004698	0.954090444	1.49E-06
TUCP_000187	XLOC_006154	ENSRNOT00000039842	0.959380669	8.13E-07
TUCP_000187	XLOC_006154	ENSRNOT00000045083	0.968566662	2.29E-07
TUCP_000187	XLOC_006154	ENSRNOT00000017629	0.973292122	1.02E-07
TUCP_000187	XLOC_006154	ENSRNOT00000065947	0.965358478	3.71E-07

TUCP_000187	XLOC_006154	ENSRNOT00000008803	0.95217491	1.82E-06
TUCP_000187	XLOC_006154	ENSRNOT00000021116	0.974007077	8.95E-08
TUCP_000187	XLOC_006154	ENSRNOT00000078475	0.986753253	3.14E-09
TUCP_000187	XLOC_006154	ENSRNOT00000004740	0.958706294	8.82E-07
TUCP_000187	XLOC_006154	ENSRNOT00000016722	0.989591572	9.45E-10
TUCP_000187	XLOC_006154	ENSRNOT00000073526	0.965413146	3.68E-07
TUCP_000187	XLOC_006154	ENSRNOT00000000013	0.971483271	1.42E-07
TUCP_000187	XLOC_006154	ENSRNOT00000025523	0.95254549	1.75E-06
TUCP_000187	XLOC_006154	ENSRNOT00000005161	0.955438998	1.28E-06
TUCP_000187	XLOC_006154	ENSRNOT00000009198	0.963039232	5.11E-07
TUCP_000187	XLOC_006154	ENSRNOT00000009129	0.959020379	8.50E-07
TUCP_000187	XLOC_006154	ENSRNOT00000004999	0.953702487	1.55E-06
TUCP_000187	XLOC_006154	ENSRNOT00000009122	0.957319425	1.04E-06
TUCP_000187	XLOC_006154	ENSRNOT00000088054	0.961219246	6.47E-07
TUCP_000187	XLOC_006154	ENSRNOT00000019554	0.964443643	4.22E-07
TUCP_000187	XLOC_006154	ENSRNOT00000019553	0.954497722	1.42E-06
TUCP_000187	XLOC_006154	ENSRNOT00000035700	0.974022604	8.92E-08
TUCP_000187	XLOC_006154	ENSRNOT00000029202	-0.961157439	6.52E-07
TUCP_000187	XLOC_006154	ENSRNOT00000092029	0.954605515	1.41E-06
TUCP_000187	XLOC_006154	ENSRNOT00000005489	0.968583725	2.29E-07
TUCP_000187	XLOC_006154	ENSRNOT00000084422	0.950032151	2.26E-06
TUCP_000187	XLOC_006154	ENSRNOT00000050834	-0.95308623	1.65E-06
TUCP_000187	XLOC_006154	ENSRNOT00000054828	0.956160036	1.18E-06
TUCP_000187	XLOC_006154	ENSRNOT00000027275	0.952862725	1.69E-06
TUCP_000187	XLOC_006154	ENSRNOT00000080928	0.9789876	3.11E-08
TUCP_000187	XLOC_006154	ENSRNOT00000009342	0.971797001	1.34E-07
TUCP_000187	XLOC_006154	ENSRNOT00000018074	-0.962986914	5.14E-07
TUCP_000187	XLOC_006154	ENSRNOT00000037679	0.953541832	1.58E-06
TUCP_000187	XLOC_006154	ENSRNOT00000014124	0.955596524	1.26E-06
TUCP_000187	XLOC_006154	ENSRNOT00000078906	-0.951921564	1.87E-06
TUCP_000187	XLOC_006154	ENSRNOT00000084049	0.9596186	7.90E-07
TUCP_000187	XLOC_006154	ENSRNOT00000009900	-0.956046414	1.20E-06
TUCP_000187	XLOC_006154	ENSRNOT00000085060	0.954057098	1.49E-06
TUCP_000187	XLOC_006154	ENSRNOT00000014979	0.95807179	9.51E-07
TUCP_000187	XLOC_006154	ENSRNOT00000019172	-0.960961892	6.69E-07
TUCP_000187	XLOC_006154	ENSRNOT00000064798	0.967185969	2.84E-07
TUCP_000187	XLOC_006154	ENSRNOT00000019177	0.964409646	4.24E-07
TUCP_000187	XLOC_006154	ENSRNOT00000019910	-0.971223551	1.48E-07
TUCP_000187	XLOC_006154	ENSRNOT00000040393	0.982480132	1.26E-08
TUCP_000187	XLOC_006154	ENSRNOT00000066593	0.963089571	5.07E-07
TUCP_000187	XLOC_006154	ENSRNOT00000067901	-0.962618033	5.40E-07
TUCP_000187	XLOC_006154	ENSRNOT00000036750	0.98343519	9.55E-09
TUCP_000187	XLOC_006154	ENSRNOT00000024659	0.978038822	3.88E-08
TUCP_000187	XLOC_006154	ENSRNOT00000011041	0.951676772	1.91E-06
TUCP_000187	XLOC_006154	ENSRNOT00000021674	0.989459236	1.01E-09
TUCP_000187	XLOC_006154	ENSRNOT00000064700	0.951313763	1.98E-06
TUCP_000187	XLOC_006154	ENSRNOT00000036613	-0.961600373	6.16E-07
TUCP_000187	XLOC_006154	ENSRNOT00000052288	0.972464495	1.19E-07
TUCP_000187	XLOC_006154	ENSRNOT00000025705	0.952965925	1.67E-06
TUCP_000187	XLOC_006154	ENSRNOT00000004140	0.9854766	4.97E-09
TUCP_000187	XLOC_006154	ENSRNOT00000010433	0.952406222	1.78E-06

TUCP_000187	XLOC_006154	ENSRNOT00000048910	-0.963308084	4.92E-07
TUCP_000187	XLOC_006154	ENSRNOT00000019213	0.986478743	3.48E-09
TUCP_000187	XLOC_006154	ENSRNOT00000071560	0.964701264	4.07E-07
TUCP_000187	XLOC_006154	ENSRNOT00000082508	-0.957017783	1.07E-06
TUCP_000187	XLOC_006154	ENSRNOT00000007164	0.961386451	6.34E-07
TUCP_000187	XLOC_006154	ENSRNOT00000049459	-0.955431654	1.28E-06
TUCP_000187	XLOC_006154	ENSRNOT00000086548	0.958366793	9.18E-07
TUCP_000187	XLOC_006154	ENSRNOT00000047387	0.959461319	8.05E-07
TUCP_000187	XLOC_006154	ENSRNOT00000001118	0.976965071	4.91E-08
TUCP_000187	XLOC_006154	ENSRNOT00000091596	-0.956413203	1.15E-06
TUCP_000187	XLOC_006154	ENSRNOT00000011384	0.959765189	7.76E-07
TUCP_000187	XLOC_006154	ENSRNOT00000015076	0.978298432	3.66E-08
TUCP_000187	XLOC_006154	ENSRNOT00000043345	0.954244169	1.46E-06
TUCP_000187	XLOC_006154	ENSRNOT00000076516	0.955536121	1.27E-06
TUCP_000187	XLOC_006154	ENSRNOT00000015073	0.988432606	1.60E-09
TUCP_000187	XLOC_006154	ENSRNOT00000018533	0.962626232	5.39E-07
TUCP_000187	XLOC_006154	ENSRNOT00000073564	-0.956355364	1.16E-06
TUCP_000187	XLOC_006154	ENSRNOT00000001507	0.982451994	1.27E-08
TUCP_000187	XLOC_006154	ENSRNOT00000083568	0.951126007	2.02E-06
TUCP_000187	XLOC_006154	ENSRNOT00000018584	0.955960769	1.21E-06
TUCP_000187	XLOC_006154	ENSRNOT00000082691	0.954753904	1.38E-06
TUCP_000187	XLOC_006154	ENSRNOT00000037456	0.981649759	1.59E-08
TUCP_000187	XLOC_006154	ENSRNOT00000025510	-0.973791669	9.32E-08
TUCP_000187	XLOC_006154	ENSRNOT00000021496	-0.95382065	1.53E-06
TUCP_000187	XLOC_006154	ENSRNOT00000075424	0.984561029	6.73E-09
TUCP_000187	XLOC_006154	ENSRNOT00000016027	-0.950551799	2.14E-06
TUCP_000187	XLOC_006154	ENSRNOT00000012177	-0.970720716	1.61E-07
TUCP_000187	XLOC_006154	ENSRNOT00000093357	0.961155188	6.53E-07
TUCP_000187	XLOC_006154	ENSRNOT00000068633	0.972749436	1.13E-07
TUCP_000187	XLOC_006154	ENSRNOT00000075452	0.951713046	1.91E-06
TUCP_000187	XLOC_006154	ENSRNOT00000081682	-0.951246051	2.00E-06
TUCP_000187	XLOC_006154	ENSRNOT00000020892	-0.967127574	2.86E-07
TUCP_000187	XLOC_006154	ENSRNOT00000020437	0.959964341	7.57E-07
TUCP_000187	XLOC_006154	ENSRNOT00000006828	0.950164814	2.23E-06
TUCP_000187	XLOC_006154	ENSRNOT00000006824	-0.955691335	1.25E-06
TUCP_000187	XLOC_006154	ENSRNOT00000050515	0.950847278	2.08E-06
TUCP_000187	XLOC_006154	ENSRNOT00000088509	0.977537034	4.34E-08
TUCP_000187	XLOC_006154	ENSRNOT00000086868	0.978011173	3.90E-08
TUCP_000187	XLOC_006154	ENSRNOT00000045488	0.97012214	1.78E-07
TUCP_000187	XLOC_006154	ENSRNOT00000093174	0.987773203	2.11E-09
TUCP_000187	XLOC_006154	ENSRNOT00000026783	0.970830512	1.58E-07
TUCP_000187	XLOC_006154	ENSRNOT00000020648	0.973152778	1.05E-07
TUCP_000187	XLOC_006154	ENSRNOT00000037588	-0.95198438	1.85E-06
TUCP_000187	XLOC_006154	ENSRNOT00000004508	0.957708278	9.92E-07
TUCP_000187	XLOC_006154	ENSRNOT00000036666	0.961187126	6.50E-07
TUCP_000187	XLOC_006154	ENSRNOT00000089211	0.975298758	6.95E-08
TUCP_000187	XLOC_006154	ENSRNOT00000020383	0.971994622	1.29E-07
TUCP_000187	XLOC_006154	ENSRNOT00000000302	0.977135118	4.74E-08
TUCP_000187	XLOC_006154	ENSRNOT00000087502	0.971624312	1.38E-07
TUCP_000187	XLOC_006154	ENSRNOT00000087997	0.954330635	1.45E-06
TUCP_000187	XLOC_006154	ENSRNOT00000012424	0.963597998	4.74E-07

TUCP_000187	XLOC_006154	ENSRNOT00000025832	0.957787123	9.83E-07
TUCP_000187	XLOC_006154	ENSRNOT00000076729	0.95500941	1.35E-06
TUCP_000187	XLOC_006154	ENSRNOT00000005909	0.981993414	1.45E-08
TUCP_000187	XLOC_006154	ENSRNOT00000086122	0.961610084	6.16E-07
TUCP_000187	XLOC_006154	ENSRNOT00000007091	0.982614724	1.21E-08
TUCP_000187	XLOC_006154	ENSRNOT00000023645	0.970351136	1.72E-07
TUCP_000187	XLOC_006154	ENSRNOT00000042799	0.967778273	2.59E-07
TUCP_000187	XLOC_006154	ENSRNOT00000058362	0.968600978	2.28E-07
TUCP_000187	XLOC_006154	ENSRNOT00000008901	-0.956004645	1.21E-06
TUCP_000187	XLOC_006154	ENSRNOT00000005659	0.985690744	4.61E-09
TUCP_000187	XLOC_006154	ENSRNOT00000017761	0.970430608	1.69E-07
TUCP_000187	XLOC_006154	ENSRNOT00000023543	-0.951061254	2.04E-06
TUCP_000187	XLOC_006154	ENSRNOT00000015612	0.950828936	2.08E-06
TUCP_000187	XLOC_006154	ENSRNOT00000039341	0.953440328	1.59E-06
TUCP_000187	XLOC_006154	ENSRNOT00000037405	0.961698405	6.09E-07
TUCP_000187	XLOC_006154	ENSRNOT00000085519	0.966169178	3.30E-07
TUCP_000187	XLOC_006154	ENSRNOT00000082174	0.971547267	1.40E-07
TUCP_000187	XLOC_006154	ENSRNOT00000026760	-0.958275726	9.28E-07
TUCP_000187	XLOC_006154	ENSRNOT00000023330	0.959197408	8.32E-07
TUCP_000187	XLOC_006154	ENSRNOT00000024491	-0.951582734	1.93E-06
TUCP_000187	XLOC_006154	ENSRNOT00000084595	0.96808324	2.47E-07
TUCP_000187	XLOC_006154	ENSRNOT00000013031	-0.950574699	2.14E-06
TUCP_000187	XLOC_006154	ENSRNOT00000027964	0.987179999	2.67E-09
TUCP_000187	XLOC_006154	ENSRNOT00000047210	0.981660074	1.58E-08
TUCP_000187	XLOC_006154	ENSRNOT00000064930	0.977252587	4.62E-08
TUCP_000187	XLOC_006154	ENSRNOT00000079996	0.980148893	2.35E-08
TUCP_003815	XLOC_166994	ENSRNOT00000079742	0.959995856	7.54E-07
TUCP_003815	XLOC_166994	ENSRNOT00000011032	0.951579856	1.93E-06
TUCP_003815	XLOC_166994	ENSRNOT00000056234	0.951105413	2.03E-06
TUCP_000181	XLOC_005879	ENSRNOT00000021917	0.971931197	1.31E-07
TUCP_000181	XLOC_005879	ENSRNOT00000085444	0.954840939	1.37E-06
TUCP_000181	XLOC_005879	ENSRNOT00000033420	0.957660782	9.98E-07
TUCP_000180	XLOC_005776	ENSRNOT00000085380	0.963872193	4.56E-07
TUCP_000180	XLOC_005776	ENSRNOT00000090914	0.952252951	1.80E-06
TUCP_000180	XLOC_005776	ENSRNOT00000046842	0.95281159	1.70E-06
TUCP_000180	XLOC_005776	ENSRNOT00000022585	0.965473756	3.65E-07
TUCP_000180	XLOC_005776	ENSRNOT00000022559	0.964061534	4.44E-07
TUCP_000180	XLOC_005776	ENSRNOT00000030327	0.962984203	5.14E-07
TUCP_000183	XLOC_005932	ENSRNOT00000066391	0.964039072	4.46E-07
TUCP_000183	XLOC_005932	ENSRNOT00000072802	0.952918478	1.68E-06
TUCP_000183	XLOC_005932	ENSRNOT00000054939	0.951754576	1.90E-06
TUCP_000183	XLOC_005932	ENSRNOT00000066821	0.966094662	3.33E-07
TUCP_003126	XLOC_133460	ENSRNOT00000066477	0.985107358	5.63E-09
TUCP_003126	XLOC_133460	ENSRNOT00000059854	0.965254263	3.76E-07
TUCP_003126	XLOC_133460	ENSRNOT00000006188	0.961744036	6.05E-07
TUCP_003126	XLOC_133460	ENSRNOT00000024369	0.952559147	1.75E-06
TUCP_003126	XLOC_133460	ENSRNOT00000012617	0.953710654	1.55E-06
TUCP_003126	XLOC_133460	ENSRNOT00000018936	0.950568797	2.14E-06
TUCP_003126	XLOC_133460	ENSRNOT00000007259	0.954565895	1.41E-06
TUCP_003126	XLOC_133460	ENSRNOT00000014559	0.952934909	1.68E-06
TUCP_003126	XLOC_133460	ENSRNOT00000022144	0.97451112	8.12E-08

TUCP_003126	XLOC_133460	ENSRNOT00000004408	0.970790966	1.59E-07
TUCP_003126	XLOC_133460	ENSRNOT00000028290	0.957081072	1.07E-06
TUCP_003126	XLOC_133460	ENSRNOT00000010591	0.968413379	2.35E-07
TUCP_003126	XLOC_133460	ENSRNOT00000044299	0.95375591	1.54E-06
TUCP_003126	XLOC_133460	ENSRNOT00000031701	0.973076533	1.06E-07
TUCP_003126	XLOC_133460	ENSRNOT00000073105	0.965450275	3.66E-07
TUCP_003126	XLOC_133460	ENSRNOT00000091139	0.954480608	1.43E-06
TUCP_003126	XLOC_133460	ENSRNOT00000090064	0.971796646	1.34E-07
TUCP_003126	XLOC_133460	ENSRNOT00000016254	0.965642244	3.56E-07
TUCP_003126	XLOC_133460	ENSRNOT00000009345	0.95098992	2.05E-06
TUCP_003126	XLOC_133460	ENSRNOT00000011694	0.953575046	1.57E-06
TUCP_003126	XLOC_133460	ENSRNOT00000008222	0.957282986	1.04E-06
TUCP_003126	XLOC_133460	ENSRNOT00000088555	0.981927911	1.47E-08
TUCP_003126	XLOC_133460	ENSRNOT00000076485	0.952609872	1.74E-06
TUCP_003126	XLOC_133460	ENSRNOT00000088282	0.955924431	1.22E-06
TUCP_003126	XLOC_133460	ENSRNOT00000093353	0.957968195	9.63E-07
TUCP_003126	XLOC_133460	ENSRNOT00000045250	0.968022624	2.50E-07
TUCP_003126	XLOC_133460	ENSRNOT00000038212	0.952643672	1.73E-06
TUCP_003126	XLOC_133460	ENSRNOT00000010700	0.958454082	9.09E-07
TUCP_003126	XLOC_133460	ENSRNOT00000023965	0.950523882	2.15E-06
TUCP_003126	XLOC_133460	ENSRNOT00000011563	0.962190683	5.71E-07
TUCP_003126	XLOC_133460	ENSRNOT00000054976	0.953613711	1.56E-06
TUCP_003126	XLOC_133460	ENSRNOT00000026846	0.969563191	1.95E-07
TUCP_003126	XLOC_133460	ENSRNOT00000060690	0.968531237	2.31E-07
TUCP_003126	XLOC_133460	ENSRNOT00000002187	0.974414595	8.27E-08
TUCP_003126	XLOC_133460	ENSRNOT00000080675	0.971484541	1.42E-07
TUCP_003126	XLOC_133460	ENSRNOT00000080410	0.959022708	8.49E-07
TUCP_003126	XLOC_133460	ENSRNOT00000074985	0.957967709	9.63E-07
TUCP_003126	XLOC_133460	ENSRNOT00000014301	0.991291905	3.89E-10
TUCP_003126	XLOC_133460	ENSRNOT00000004351	0.954191552	1.47E-06
TUCP_003126	XLOC_133460	ENSRNOT00000075800	0.953031657	1.66E-06
TUCP_003126	XLOC_133460	ENSRNOT00000066545	0.960642519	6.96E-07
TUCP_003126	XLOC_133460	ENSRNOT00000084597	0.954040847	1.49E-06
TUCP_003126	XLOC_133460	ENSRNOT00000077243	0.959906515	7.63E-07
TUCP_003126	XLOC_133460	ENSRNOT00000067780	0.957162596	1.06E-06
TUCP_003126	XLOC_133460	ENSRNOT00000079227	0.956024247	1.20E-06
TUCP_003126	XLOC_133460	ENSRNOT00000019109	0.965709218	3.52E-07
TUCP_003126	XLOC_133460	ENSRNOT00000045315	0.950385696	2.18E-06
TUCP_003126	XLOC_133460	ENSRNOT00000060002	0.960039043	7.50E-07
TUCP_003126	XLOC_133460	ENSRNOT00000073910	0.951047259	2.04E-06
TUCP_003126	XLOC_133460	ENSRNOT00000000170	0.955987643	1.21E-06
TUCP_003126	XLOC_133460	ENSRNOT00000066120	0.951342544	1.98E-06
TUCP_003126	XLOC_133460	ENSRNOT00000078806	0.9672196	2.82E-07
TUCP_003126	XLOC_133460	ENSRNOT00000015055	0.975059532	7.29E-08
TUCP_001124	XLOC_042595	ENSRNOT00000024366	0.955108358	1.33E-06
TUCP_001124	XLOC_042595	ENSRNOT00000028540	0.955608963	1.26E-06
TUCP_001124	XLOC_042595	ENSRNOT00000090688	0.96847239	2.33E-07
TUCP_001124	XLOC_042595	ENSRNOT00000001899	0.95255425	1.75E-06
TUCP_001124	XLOC_042595	ENSRNOT00000035992	0.961131726	6.54E-07
TUCP_001124	XLOC_042595	ENSRNOT00000021752	0.960913269	6.73E-07
TUCP_001124	XLOC_042595	ENSRNOT00000002291	0.964326193	4.29E-07

TUCP_001124	XLOC_042595	ENSRNOT00000025325	0.969640871	1.93E-07
TUCP_001124	XLOC_042595	ENSRNOT00000023744	0.957406188	1.03E-06
TUCP_001124	XLOC_042595	ENSRNOT00000010332	0.958263993	9.30E-07
TUCP_001124	XLOC_042595	ENSRNOT00000026641	0.951973248	1.86E-06
TUCP_001124	XLOC_042595	ENSRNOT00000066024	0.952316032	1.79E-06
TUCP_001124	XLOC_042595	ENSRNOT00000001826	0.955648408	1.25E-06
TUCP_001124	XLOC_042595	ENSRNOT00000056430	0.974687152	7.84E-08
TUCP_001124	XLOC_042595	ENSRNOT00000085765	0.968381606	2.36E-07
TUCP_001125	XLOC_042598	ENSRNOT00000008573	0.954995059	1.35E-06
TUCP_001125	XLOC_042598	ENSRNOT00000009979	0.950626486	2.13E-06
TUCP_001125	XLOC_042598	ENSRNOT00000018543	0.956048333	1.20E-06
TUCP_001125	XLOC_042598	ENSRNOT00000080271	0.952129732	1.83E-06
TUCP_001125	XLOC_042598	ENSRNOT00000041709	0.955365988	1.29E-06
TUCP_001125	XLOC_042598	ENSRNOT00000081768	0.971839211	1.33E-07
TUCP_001125	XLOC_042598	ENSRNOT00000045326	0.953257468	1.62E-06
TUCP_001125	XLOC_042598	ENSRNOT00000019502	0.952476769	1.76E-06
TUCP_001125	XLOC_042598	ENSRNOT00000028856	0.975923626	6.12E-08
TUCP_001125	XLOC_042598	ENSRNOT00000060282	0.972355105	1.21E-07
TUCP_001125	XLOC_042598	ENSRNOT00000017968	0.962905871	5.20E-07
TUCP_001125	XLOC_042598	ENSRNOT00000038577	0.959359306	8.15E-07
TUCP_001125	XLOC_042598	ENSRNOT00000080654	0.973651847	9.57E-08
TUCP_001125	XLOC_042598	ENSRNOT00000086893	-0.956957551	1.08E-06
TUCP_001125	XLOC_042598	ENSRNOT00000043554	0.952475963	1.76E-06
TUCP_001125	XLOC_042598	ENSRNOT00000026783	0.950205156	2.22E-06
TUCP_001125	XLOC_042598	ENSRNOT00000032158	0.951620954	1.92E-06
TUCP_001125	XLOC_042598	ENSRNOT00000073971	0.9545663	1.41E-06
TUCP_001125	XLOC_042598	ENSRNOT00000008703	0.957819936	9.80E-07
TUCP_001125	XLOC_042598	ENSRNOT00000016801	0.956729412	1.11E-06
TUCP_001125	XLOC_042598	ENSRNOT00000016490	0.975776676	6.31E-08
TUCP_001125	XLOC_042598	ENSRNOT00000016519	0.95261321	1.74E-06
TUCP_001125	XLOC_042598	ENSRNOT00000052075	0.974226859	8.58E-08
TUCP_001125	XLOC_042598	ENSRNOT00000026186	0.961126495	6.55E-07
TUCP_001125	XLOC_042598	ENSRNOT00000079109	0.951117606	2.02E-06
TUCP_001125	XLOC_042598	ENSRNOT00000017319	0.953987056	1.50E-06
TUCP_001125	XLOC_042598	ENSRNOT00000057807	-0.955174846	1.32E-06
TUCP_001125	XLOC_042598	ENSRNOT00000026121	0.957300655	1.04E-06
TUCP_001125	XLOC_042598	ENSRNOT00000072518	0.952002144	1.85E-06
TUCP_001125	XLOC_042598	ENSRNOT00000034722	0.972103025	1.27E-07
TUCP_001125	XLOC_042598	ENSRNOT00000021037	0.952826498	1.70E-06
TUCP_001125	XLOC_042598	ENSRNOT00000084385	0.955238416	1.31E-06
TUCP_001125	XLOC_042598	ENSRNOT00000044562	0.960105955	7.44E-07
TUCP_001125	XLOC_042598	ENSRNOT00000027200	-0.95181687	1.89E-06
TUCP_001125	XLOC_042598	ENSRNOT00000014508	0.975391299	6.82E-08
TUCP_001125	XLOC_042598	ENSRNOT00000016321	-0.961060306	6.60E-07
TUCP_001125	XLOC_042598	ENSRNOT00000008817	0.954009071	1.50E-06
TUCP_001125	XLOC_042598	ENSRNOT00000027335	0.957742509	9.88E-07
TUCP_001125	XLOC_042598	ENSRNOT00000071493	-0.952438138	1.77E-06
TUCP_001125	XLOC_042598	ENSRNOT00000052422	0.957685773	9.95E-07
TUCP_001125	XLOC_042598	ENSRNOT00000084496	0.955420215	1.29E-06
TUCP_001125	XLOC_042598	ENSRNOT00000001242	0.952352178	1.79E-06
TUCP_001125	XLOC_042598	ENSRNOT00000023330	0.965358046	3.71E-07

TUCP_001125	XLOC_042598	ENSRNOT00000010873	-0.963018042	5.12E-07
TUCP_001125	XLOC_042598	ENSRNOT00000007164	0.953585023	1.57E-06
TUCP_001125	XLOC_042598	ENSRNOT00000021790	0.95265934	1.73E-06
TUCP_001125	XLOC_042598	ENSRNOT00000090451	-0.954174013	1.47E-06
TUCP_001125	XLOC_042598	ENSRNOT00000078639	0.95135221	1.98E-06
TUCP_001125	XLOC_042598	ENSRNOT00000022286	0.956077734	1.20E-06
TUCP_001125	XLOC_042598	ENSRNOT00000016129	0.955317754	1.30E-06
TUCP_001125	XLOC_042598	ENSRNOT00000016289	0.95369326	1.55E-06
TUCP_001125	XLOC_042598	ENSRNOT00000009328	0.974700066	7.82E-08
TUCP_001125	XLOC_042598	ENSRNOT00000048490	-0.958239821	9.32E-07
TUCP_001125	XLOC_042598	ENSRNOT00000029660	0.951954552	1.86E-06
TUCP_001125	XLOC_042598	ENSRNOT00000026755	0.966263419	3.25E-07
TUCP_001125	XLOC_042598	ENSRNOT00000087997	0.955110483	1.33E-06
TUCP_001125	XLOC_042598	ENSRNOT00000065618	0.956180015	1.18E-06
TUCP_001125	XLOC_042598	ENSRNOT00000003770	-0.952630068	1.73E-06
TUCP_001125	XLOC_042598	ENSRNOT00000015609	0.962948182	5.17E-07
TUCP_001125	XLOC_042598	ENSRNOT00000055318	0.954293423	1.45E-06
TUCP_001125	XLOC_042598	ENSRNOT00000051022	0.973223111	1.04E-07
TUCP_001125	XLOC_042598	ENSRNOT00000087725	0.963829381	4.59E-07
TUCP_001125	XLOC_042598	ENSRNOT00000046594	0.961441218	6.29E-07
TUCP_001125	XLOC_042598	ENSRNOT00000029833	0.960600559	7.00E-07
TUCP_001125	XLOC_042598	ENSRNOT00000036951	0.952015382	1.85E-06
TUCP_001125	XLOC_042598	ENSRNOT00000008022	0.963682616	4.68E-07
TUCP_001125	XLOC_042598	ENSRNOT00000009691	0.951765915	1.90E-06
TUCP_001125	XLOC_042598	ENSRNOT00000013762	0.978826216	3.23E-08
TUCP_001125	XLOC_042598	ENSRNOT00000022963	0.971887383	1.32E-07
TUCP_001125	XLOC_042598	ENSRNOT00000011663	0.958167549	9.40E-07
TUCP_001125	XLOC_042598	ENSRNOT00000043953	0.970454875	1.69E-07
TUCP_001125	XLOC_042598	ENSRNOT00000058842	0.951563095	1.94E-06
TUCP_001125	XLOC_042598	ENSRNOT00000083255	0.952913564	1.68E-06
TUCP_001125	XLOC_042598	ENSRNOT00000077894	-0.957551292	1.01E-06
TUCP_001125	XLOC_042598	ENSRNOT00000003690	0.971269009	1.47E-07
TUCP_001125	XLOC_042598	ENSRNOT00000054980	0.956133766	1.19E-06
TUCP_001125	XLOC_042598	ENSRNOT00000005876	0.963788959	4.61E-07
TUCP_001125	XLOC_042598	ENSRNOT00000001682	0.951663667	1.92E-06
TUCP_001125	XLOC_042598	ENSRNOT00000077406	0.950481782	2.16E-06
TUCP_001125	XLOC_042598	ENSRNOT00000004762	0.965963897	3.40E-07
TUCP_001125	XLOC_042598	ENSRNOT00000016859	0.959852952	7.68E-07
TUCP_001125	XLOC_042598	ENSRNOT00000074659	0.981324462	1.73E-08
TUCP_001125	XLOC_042598	ENSRNOT00000056130	0.956381292	1.16E-06
TUCP_001125	XLOC_042598	ENSRNOT00000004172	0.955123853	1.33E-06
TUCP_001125	XLOC_042598	ENSRNOT00000059744	0.966428305	3.17E-07
TUCP_001125	XLOC_042598	ENSRNOT00000057879	0.950275416	2.20E-06
TUCP_001125	XLOC_042598	ENSRNOT00000010218	0.960433938	7.15E-07
TUCP_001125	XLOC_042598	ENSRNOT00000007768	0.960879685	6.76E-07
TUCP_001125	XLOC_042598	ENSRNOT00000019177	0.956152562	1.19E-06
TUCP_001125	XLOC_042598	ENSRNOT00000083414	0.962813075	5.26E-07
TUCP_001125	XLOC_042598	ENSRNOT00000009473	0.951132471	2.02E-06
TUCP_001125	XLOC_042598	ENSRNOT00000008210	0.968885652	2.18E-07
TUCP_001125	XLOC_042598	ENSRNOT00000022947	-0.957289849	1.04E-06
TUCP_001125	XLOC_042598	ENSRNOT00000010433	0.951248166	2.00E-06

TUCP_001125	XLOC_042598	ENSRNOT00000011682	-0.951242113	2.00E-06
TUCP_000024	XLOC_000371	ENSRNOT00000025981	0.964732429	4.05E-07
TUCP_000024	XLOC_000371	ENSRNOT00000012671	0.961594053	6.17E-07
TUCP_000024	XLOC_000371	ENSRNOT00000021833	0.950647193	2.12E-06
TUCP_000024	XLOC_000371	ENSRNOT00000068405	0.970876706	1.57E-07
TUCP_000024	XLOC_000371	ENSRNOT00000038647	0.958964807	8.55E-07
TUCP_000024	XLOC_000371	ENSRNOT00000047387	0.956140066	1.19E-06
TUCP_000024	XLOC_000371	ENSRNOT00000076669	0.967590025	2.67E-07
TUCP_000024	XLOC_000371	ENSRNOT00000085596	0.952460317	1.77E-06
TUCP_000024	XLOC_000371	ENSRNOT00000091910	0.953201022	1.63E-06
TUCP_000024	XLOC_000371	ENSRNOT00000039842	0.963945531	4.52E-07
TUCP_000024	XLOC_000371	ENSRNOT00000055187	0.952000348	1.85E-06
TUCP_000024	XLOC_000371	ENSRNOT00000021348	0.962472219	5.50E-07
TUCP_000024	XLOC_000371	ENSRNOT00000017629	0.967876063	2.55E-07
TUCP_000024	XLOC_000371	ENSRNOT00000067866	0.95295382	1.68E-06
TUCP_000024	XLOC_000371	ENSRNOT00000025859	0.976930766	4.95E-08
TUCP_000024	XLOC_000371	ENSRNOT00000008803	0.953647559	1.56E-06
TUCP_000024	XLOC_000371	ENSRNOT00000031230	0.961596671	6.17E-07
TUCP_000024	XLOC_000371	ENSRNOT00000005664	0.961079538	6.59E-07
TUCP_000024	XLOC_000371	ENSRNOT00000032274	0.958586367	8.95E-07
TUCP_000024	XLOC_000371	ENSRNOT00000004508	0.953977123	1.50E-06
TUCP_000024	XLOC_000371	ENSRNOT00000006175	0.970543548	1.66E-07
TUCP_000024	XLOC_000371	ENSRNOT00000052288	0.953869799	1.52E-06
TUCP_000024	XLOC_000371	ENSRNOT00000023144	0.956201434	1.18E-06
TUCP_000024	XLOC_000371	ENSRNOT00000034704	0.955808316	1.23E-06
TUCP_000024	XLOC_000371	ENSRNOT00000021116	0.955007328	1.35E-06
TUCP_000024	XLOC_000371	ENSRNOT00000039809	0.970475432	1.68E-07
TUCP_000024	XLOC_000371	ENSRNOT00000082100	0.960342781	7.23E-07
TUCP_000024	XLOC_000371	ENSRNOT00000076729	0.962682793	5.35E-07
TUCP_000024	XLOC_000371	ENSRNOT00000090665	0.977871643	4.03E-08
TUCP_000024	XLOC_000371	ENSRNOT00000003296	0.958350594	9.20E-07
TUCP_000024	XLOC_000371	ENSRNOT00000010992	0.982832463	1.14E-08
TUCP_000024	XLOC_000371	ENSRNOT00000026543	0.977184243	4.69E-08
TUCP_000024	XLOC_000371	ENSRNOT00000015073	0.951341042	1.98E-06
TUCP_000024	XLOC_000371	ENSRNOT00000025832	0.966523271	3.13E-07
TUCP_000024	XLOC_000371	ENSRNOT00000004412	0.953612131	1.56E-06
TUCP_000024	XLOC_000371	ENSRNOT00000060499	0.951882933	1.87E-06
TUCP_000024	XLOC_000371	ENSRNOT00000004359	0.959531421	7.99E-07
TUCP_000024	XLOC_000371	ENSRNOT00000071573	0.960057295	7.49E-07
TUCP_000024	XLOC_000371	ENSRNOT00000023696	0.959553217	7.96E-07
TUCP_000024	XLOC_000371	ENSRNOT00000013274	0.957786559	9.83E-07
TUCP_000024	XLOC_000371	ENSRNOT00000025092	0.984517933	6.83E-09
TUCP_000024	XLOC_000371	ENSRNOT00000016209	0.96013133	7.42E-07
TUCP_000024	XLOC_000371	ENSRNOT00000036750	0.950292774	2.20E-06
TUCP_000024	XLOC_000371	ENSRNOT00000027964	0.951110108	2.03E-06
TUCP_000024	XLOC_000371	ENSRNOT00000024420	0.962414663	5.55E-07
TUCP_000024	XLOC_000371	ENSRNOT00000044956	-0.951141156	2.02E-06
TUCP_000024	XLOC_000371	ENSRNOT00000035989	0.967178826	2.84E-07
TUCP_000024	XLOC_000371	ENSRNOT00000057160	0.954131807	1.48E-06
TUCP_000024	XLOC_000371	ENSRNOT00000005355	0.965188495	3.80E-07
TUCP_000024	XLOC_000371	ENSRNOT00000088054	0.950431239	2.17E-06

TUCP_000024	XLOC_000371	ENSRNOT00000057313	0.958168279	9.40E-07
TUCP_000024	XLOC_000371	ENSRNOT00000083799	0.955724318	1.24E-06
TUCP_000024	XLOC_000371	ENSRNOT00000009066	0.954525008	1.42E-06
TUCP_000025	XLOC_000371	ENSRNOT00000010992	0.958286404	9.27E-07
TUCP_000025	XLOC_000371	ENSRNOT00000025859	0.967736167	2.61E-07
TUCP_000025	XLOC_000371	ENSRNOT00000009712	0.958491784	9.05E-07
TUCP_000025	XLOC_000371	ENSRNOT00000006175	0.957790434	9.83E-07
TUCP_000025	XLOC_000371	ENSRNOT00000089971	0.968488495	2.32E-07
TUCP_000025	XLOC_000371	ENSRNOT00000022522	0.968722759	2.24E-07
TUCP_000025	XLOC_000371	ENSRNOT00000000676	0.954030477	1.50E-06
TUCP_000025	XLOC_000371	ENSRNOT00000038647	0.975140886	7.17E-08
TUCP_000025	XLOC_000371	ENSRNOT00000014664	0.959152266	8.36E-07
TUCP_000025	XLOC_000371	ENSRNOT00000091910	0.97882878	3.23E-08
TUCP_000025	XLOC_000371	ENSRNOT00000075653	0.964776346	4.02E-07
TUCP_000025	XLOC_000371	ENSRNOT00000010035	0.992409494	1.96E-10
TUCP_000025	XLOC_000371	ENSRNOT00000078675	0.964369269	4.26E-07
TUCP_000025	XLOC_000371	ENSRNOT00000066739	0.960902069	6.74E-07
TUCP_000025	XLOC_000371	ENSRNOT00000090665	0.975032209	7.33E-08
TUCP_000025	XLOC_000371	ENSRNOT00000074361	0.952757309	1.71E-06
TUCP_000025	XLOC_000371	ENSRNOT00000085027	0.968440801	2.34E-07
TUCP_001120	XLOC_042333	ENSRNOT00000031901	-0.960863703	6.77E-07
TUCP_001120	XLOC_042333	ENSRNOT00000076187	0.952508486	1.76E-06
TUCP_001120	XLOC_042333	ENSRNOT00000091052	0.952230606	1.81E-06
TUCP_001120	XLOC_042333	ENSRNOT00000038553	0.964326315	4.29E-07
TUCP_000188	XLOC_006166	ENSRNOT00000060500	0.950296368	2.20E-06
TUCP_000188	XLOC_006166	ENSRNOT00000032992	0.975999478	6.02E-08
TUCP_000188	XLOC_006166	ENSRNOT00000089082	0.953652528	1.56E-06
TUCP_000188	XLOC_006166	ENSRNOT00000027561	0.957947019	9.65E-07
TUCP_000188	XLOC_006166	ENSRNOT00000021592	0.951275852	1.99E-06
TUCP_000188	XLOC_006166	ENSRNOT00000036054	0.954836462	1.37E-06
TUCP_000188	XLOC_006166	ENSRNOT00000005521	0.955742626	1.24E-06
TUCP_004841	XLOC_215395	ENSRNOT00000074753	0.963484041	4.81E-07
TUCP_004841	XLOC_215395	ENSRNOT00000041904	0.963910348	4.54E-07
TUCP_004841	XLOC_215395	ENSRNOT00000083487	0.959363682	8.15E-07
TUCP_004841	XLOC_215395	ENSRNOT00000077990	0.963910348	4.54E-07
TUCP_004841	XLOC_215395	ENSRNOT00000038826	0.966612636	3.09E-07
TUCP_004841	XLOC_215395	ENSRNOT00000046842	0.996792954	2.66E-12
TUCP_004841	XLOC_215395	ENSRNOT00000060250	0.9634578	4.83E-07
TUCP_004841	XLOC_215395	ENSRNOT00000091934	0.963910348	4.54E-07
TUCP_004841	XLOC_215395	ENSRNOT00000089929	0.963910348	4.54E-07
TUCP_004841	XLOC_215395	ENSRNOT00000014456	0.951560295	1.94E-06
TUCP_004841	XLOC_215395	ENSRNOT00000044145	0.963910348	4.54E-07
TUCP_004841	XLOC_215395	ENSRNOT00000011743	0.992583164	1.75E-10
TUCP_004841	XLOC_215395	ENSRNOT00000034907	0.968072636	2.48E-07
TUCP_004841	XLOC_215395	ENSRNOT00000088624	0.963910348	4.54E-07
TUCP_004841	XLOC_215395	ENSRNOT00000064250	0.953551615	1.57E-06
TUCP_004841	XLOC_215395	ENSRNOT00000051835	0.963910348	4.54E-07
TUCP_004841	XLOC_215395	ENSRNOT00000079087	0.963910348	4.54E-07
TUCP_004841	XLOC_215395	ENSRNOT00000037206	0.963910348	4.54E-07
TUCP_004841	XLOC_215395	ENSRNOT00000083261	0.968516215	2.31E-07
TUCP_004841	XLOC_215395	ENSRNOT00000080794	0.971493911	1.41E-07

TUCP_004841	XLOC_215395	ENSRNOT00000080590	0.96330042	4.93E-07
TUCP_004841	XLOC_215395	ENSRNOT00000027552	0.963819019	4.59E-07
TUCP_004841	XLOC_215395	ENSRNOT00000077014	0.95430504	1.45E-06
TUCP_004841	XLOC_215395	ENSRNOT00000080453	0.962617044	5.40E-07
TUCP_004841	XLOC_215395	ENSRNOT00000030327	0.963691222	4.68E-07
TUCP_004841	XLOC_215395	ENSRNOT00000057729	0.962130651	5.76E-07
TUCP_004841	XLOC_215395	ENSRNOT00000078633	0.963910348	4.54E-07
TUCP_004841	XLOC_215395	ENSRNOT00000066839	0.973083877	1.06E-07
TUCP_004841	XLOC_215395	ENSRNOT00000085380	0.960982561	6.67E-07
TUCP_004841	XLOC_215395	ENSRNOT00000029634	0.959262768	8.25E-07
TUCP_004841	XLOC_215395	ENSRNOT00000086349	0.951576822	1.93E-06
TUCP_004841	XLOC_215395	ENSRNOT00000065460	0.963910348	4.54E-07
TUCP_004841	XLOC_215395	ENSRNOT00000003817	0.958737925	8.79E-07
TUCP_004841	XLOC_215395	ENSRNOT00000016713	0.963910348	4.54E-07
TUCP_004841	XLOC_215395	ENSRNOT00000022559	0.958616422	8.92E-07
TUCP_004841	XLOC_215395	ENSRNOT00000079260	0.977995625	3.92E-08
TUCP_004841	XLOC_215395	ENSRNOT00000039294	0.963910348	4.54E-07
TUCP_004841	XLOC_215395	ENSRNOT00000092913	0.956604937	1.13E-06
TUCP_004758	XLOC_209622	ENSRNOT00000086502	0.974269348	8.51E-08
TUCP_004758	XLOC_209622	ENSRNOT00000042165	0.979567753	2.71E-08
TUCP_004758	XLOC_209622	ENSRNOT00000031586	0.952667185	1.73E-06
TUCP_004758	XLOC_209622	ENSRNOT00000092890	0.950578905	2.14E-06
TUCP_004758	XLOC_209622	ENSRNOT00000008278	0.989944146	7.96E-10
TUCP_004052	XLOC_178233	ENSRNOT00000073235	0.951501213	1.95E-06
TUCP_004052	XLOC_178233	ENSRNOT00000027339	0.951881153	1.87E-06
TUCP_004052	XLOC_178233	ENSRNOT00000000036	0.972825481	1.12E-07
TUCP_004052	XLOC_178233	ENSRNOT00000023858	0.95345498	1.59E-06
TUCP_004052	XLOC_178233	ENSRNOT00000014867	0.978646803	3.37E-08
TUCP_004052	XLOC_178233	ENSRNOT00000000337	0.979079526	3.05E-08
TUCP_004052	XLOC_178233	ENSRNOT00000063831	-0.95290237	1.69E-06
TUCP_004051	XLOC_178179	ENSRNOT00000008879	0.953375596	1.60E-06
TUCP_000432	XLOC_014439	ENSRNOT00000092026	0.980482968	2.16E-08
TUCP_000432	XLOC_014439	ENSRNOT00000093075	0.997361897	1.00E-12
TUCP_000432	XLOC_014439	ENSRNOT00000058260	0.997361897	1.00E-12
TUCP_000432	XLOC_014439	ENSRNOT00000088887	0.997361897	1.00E-12
TUCP_000432	XLOC_014439	ENSRNOT00000084986	0.997361897	1.00E-12
TUCP_000432	XLOC_014439	ENSRNOT00000074346	0.96539464	3.69E-07
TUCP_000432	XLOC_014439	ENSRNOT00000073209	0.983528001	9.29E-09
TUCP_000432	XLOC_014439	ENSRNOT00000080155	0.971162955	1.50E-07
TUCP_000432	XLOC_014439	ENSRNOT00000072573	0.994487606	3.97E-11
TUCP_000432	XLOC_014439	ENSRNOT00000017828	0.99260546	1.72E-10
TUCP_000432	XLOC_014439	ENSRNOT00000080444	0.9680038	2.50E-07
TUCP_000432	XLOC_014439	ENSRNOT00000071312	0.997361897	1.00E-12
TUCP_000432	XLOC_014439	ENSRNOT00000088245	0.997361897	1.00E-12
TUCP_000432	XLOC_014439	ENSRNOT00000036565	0.963452937	4.83E-07
TUCP_000432	XLOC_014439	ENSRNOT00000058833	0.99559564	1.30E-11
TUCP_000432	XLOC_014439	ENSRNOT00000078052	0.997361897	1.00E-12
TUCP_000432	XLOC_014439	ENSRNOT00000078494	0.997185298	1.38E-12
TUCP_000432	XLOC_014439	ENSRNOT00000085653	0.997361897	1.00E-12
TUCP_000432	XLOC_014439	ENSRNOT00000057950	0.965805912	3.48E-07
TUCP_000432	XLOC_014439	ENSRNOT00000081992	0.996596668	3.58E-12

TUCP_000432	XLOC_014439	ENSRNOT00000084120	0.952236507	1.81E-06
TUCP_000432	XLOC_014439	ENSRNOT00000055406	0.997361897	1.00E-12
TUCP_000432	XLOC_014439	ENSRNOT00000070817	0.995941962	8.61E-12
TUCP_000432	XLOC_014439	ENSRNOT00000026753	0.997361897	1.00E-12
TUCP_000432	XLOC_014439	ENSRNOT00000080533	0.993352988	1.01E-10
TUCP_000432	XLOC_014439	ENSRNOT00000072774	0.975354675	6.87E-08
TUCP_000432	XLOC_014439	ENSRNOT00000086031	0.997361897	1.00E-12
TUCP_000432	XLOC_014439	ENSRNOT00000014312	0.997361897	1.00E-12
TUCP_000432	XLOC_014439	ENSRNOT00000042882	0.974463352	8.19E-08
TUCP_000432	XLOC_014439	ENSRNOT00000088012	0.951739449	1.90E-06
TUCP_000432	XLOC_014439	ENSRNOT00000051231	0.999383805	6.99E-16
TUCP_000432	XLOC_014439	ENSRNOT00000089407	0.989343425	1.06E-09
TUCP_000432	XLOC_014439	ENSRNOT00000055034	0.99418431	5.19E-11
TUCP_000432	XLOC_014439	ENSRNOT00000046837	0.968234539	2.41E-07
TUCP_004751	XLOC_209444	ENSRNOT00000080613	0.984844887	6.14E-09
TUCP_004751	XLOC_209444	ENSRNOT00000020941	-0.951285927	1.99E-06
TUCP_003811	XLOC_166670	ENSRNOT00000001215	0.956769555	1.11E-06
TUCP_003811	XLOC_166670	ENSRNOT00000006188	-0.96373216	4.65E-07
TUCP_003811	XLOC_166670	ENSRNOT00000006186	0.963900289	4.54E-07
TUCP_003811	XLOC_166670	ENSRNOT00000026941	0.961819313	5.99E-07
TUCP_003811	XLOC_166670	ENSRNOT00000013745	-0.956902409	1.09E-06
TUCP_003811	XLOC_166670	ENSRNOT00000075397	0.973460811	9.92E-08
TUCP_003811	XLOC_166670	ENSRNOT00000032926	-0.961282018	6.42E-07
TUCP_003811	XLOC_166670	ENSRNOT00000019104	-0.971116223	1.51E-07
TUCP_003811	XLOC_166670	ENSRNOT00000073559	0.95155762	1.94E-06
TUCP_003811	XLOC_166670	ENSRNOT00000068353	0.959565429	7.95E-07
TUCP_003811	XLOC_166670	ENSRNOT00000001691	0.958717297	8.81E-07
TUCP_003811	XLOC_166670	ENSRNOT00000020910	0.975291987	6.96E-08
TUCP_003811	XLOC_166670	ENSRNOT00000025067	-0.951610147	1.93E-06
TUCP_003811	XLOC_166670	ENSRNOT00000086706	0.958741896	8.78E-07
TUCP_003811	XLOC_166670	ENSRNOT00000086310	-0.966021946	3.37E-07
TUCP_003811	XLOC_166670	ENSRNOT00000010030	-0.951592178	1.93E-06
TUCP_003811	XLOC_166670	ENSRNOT00000028856	0.952561724	1.75E-06
TUCP_003811	XLOC_166670	ENSRNOT00000000743	0.955942133	1.21E-06
TUCP_003811	XLOC_166670	ENSRNOT00000014384	-0.952899533	1.69E-06
TUCP_003811	XLOC_166670	ENSRNOT00000030850	0.966894347	2.96E-07
TUCP_003811	XLOC_166670	ENSRNOT00000033017	0.959734296	7.79E-07
TUCP_003811	XLOC_166670	ENSRNOT00000055540	0.963175547	5.01E-07
TUCP_003811	XLOC_166670	ENSRNOT00000006752	0.968952136	2.16E-07
TUCP_003811	XLOC_166670	ENSRNOT00000077201	-0.953599837	1.57E-06
TUCP_003811	XLOC_166670	ENSRNOT00000085251	-0.964339405	4.28E-07
TUCP_003811	XLOC_166670	ENSRNOT00000072771	0.957720807	9.91E-07
TUCP_003811	XLOC_166670	ENSRNOT00000065300	0.974106614	8.78E-08
TUCP_003811	XLOC_166670	ENSRNOT00000045699	0.954608327	1.41E-06
TUCP_003811	XLOC_166670	ENSRNOT00000065978	0.951560635	1.94E-06
TUCP_003811	XLOC_166670	ENSRNOT00000007351	-0.950537386	2.15E-06
TUCP_003811	XLOC_166670	ENSRNOT00000035320	-0.964457056	4.21E-07
TUCP_003811	XLOC_166670	ENSRNOT00000057160	0.966048616	3.36E-07
TUCP_003811	XLOC_166670	ENSRNOT00000091448	-0.954146605	1.48E-06
TUCP_003811	XLOC_166670	ENSRNOT00000077999	0.971155594	1.50E-07
TUCP_003811	XLOC_166670	ENSRNOT00000003412	-0.958006776	9.58E-07

TUCP_003811	XLOC_166670	ENSRNOT00000085228	0.969710667	1.91E-07
TUCP_003811	XLOC_166670	ENSRNOT00000015188	0.96642565	3.18E-07
TUCP_003811	XLOC_166670	ENSRNOT00000079866	0.954622268	1.40E-06
TUCP_003811	XLOC_166670	ENSRNOT00000005686	-0.963221404	4.98E-07
TUCP_003811	XLOC_166670	ENSRNOT00000036959	-0.964744214	4.04E-07
TUCP_003811	XLOC_166670	ENSRNOT00000000048	0.951083635	2.03E-06
TUCP_003811	XLOC_166670	ENSRNOT00000008655	0.955344075	1.30E-06
TUCP_003811	XLOC_166670	ENSRNOT00000087278	-0.957498343	1.02E-06
TUCP_003811	XLOC_166670	ENSRNOT00000041044	0.960314729	7.25E-07
TUCP_003811	XLOC_166670	ENSRNOT00000067391	-0.954102141	1.48E-06
TUCP_003811	XLOC_166670	ENSRNOT00000092945	-0.952347982	1.79E-06
TUCP_003811	XLOC_166670	ENSRNOT00000009150	0.955148895	1.33E-06
TUCP_003811	XLOC_166670	ENSRNOT00000084709	0.971659306	1.37E-07
TUCP_003811	XLOC_166670	ENSRNOT00000068033	0.95784631	9.76E-07
TUCP_003811	XLOC_166670	ENSRNOT00000090912	0.964003711	4.48E-07
TUCP_003811	XLOC_166670	ENSRNOT00000026525	-0.955420692	1.29E-06
TUCP_003811	XLOC_166670	ENSRNOT00000082709	-0.950027714	2.26E-06
TUCP_003811	XLOC_166670	ENSRNOT00000025856	-0.968829679	2.20E-07
TUCP_003811	XLOC_166670	ENSRNOT00000026643	0.983507517	9.35E-09
TUCP_003811	XLOC_166670	ENSRNOT00000001403	0.960676252	6.93E-07
TUCP_003811	XLOC_166670	ENSRNOT00000072357	-0.962304598	5.63E-07
TUCP_003811	XLOC_166670	ENSRNOT00000016132	-0.97634695	5.60E-08
TUCP_003811	XLOC_166670	ENSRNOT00000058495	-0.957047107	1.07E-06
TUCP_003811	XLOC_166670	ENSRNOT00000013067	-0.973434542	9.97E-08
TUCP_003811	XLOC_166670	ENSRNOT00000016220	-0.958098961	9.48E-07
TUCP_003811	XLOC_166670	ENSRNOT00000002313	-0.95357765	1.57E-06
TUCP_003811	XLOC_166670	ENSRNOT00000048063	0.960955663	6.69E-07
TUCP_003811	XLOC_166670	ENSRNOT00000033975	0.971804077	1.34E-07
TUCP_003811	XLOC_166670	ENSRNOT00000079895	0.960283385	7.28E-07
TUCP_003811	XLOC_166670	ENSRNOT00000078102	0.972641519	1.15E-07
TUCP_003811	XLOC_166670	ENSRNOT00000074586	-0.955530635	1.27E-06
TUCP_003811	XLOC_166670	ENSRNOT00000063873	0.955374794	1.29E-06
TUCP_003811	XLOC_166670	ENSRNOT00000084031	0.968502847	2.32E-07
TUCP_003811	XLOC_166670	ENSRNOT00000011880	0.961841599	5.98E-07
TUCP_003811	XLOC_166670	ENSRNOT00000029202	-0.977521957	4.35E-08
TUCP_003811	XLOC_166670	ENSRNOT00000028290	-0.955282178	1.31E-06
TUCP_003811	XLOC_166670	ENSRNOT00000003992	-0.956778935	1.10E-06
TUCP_003811	XLOC_166670	ENSRNOT00000050515	0.971746228	1.35E-07
TUCP_003811	XLOC_166670	ENSRNOT00000012942	0.957388658	1.03E-06
TUCP_003811	XLOC_166670	ENSRNOT00000084088	0.953439896	1.59E-06
TUCP_003811	XLOC_166670	ENSRNOT00000052224	0.977065642	4.81E-08
TUCP_003811	XLOC_166670	ENSRNOT00000078070	0.988573972	1.50E-09
TUCP_003811	XLOC_166670	ENSRNOT00000020951	0.957376298	1.03E-06
TUCP_003811	XLOC_166670	ENSRNOT00000046745	0.970221472	1.75E-07
TUCP_003811	XLOC_166670	ENSRNOT00000082146	-0.962271076	5.65E-07
TUCP_003811	XLOC_166670	ENSRNOT00000023876	0.956962552	1.08E-06
TUCP_003811	XLOC_166670	ENSRNOT00000061201	-0.951133465	2.02E-06
TUCP_003811	XLOC_166670	ENSRNOT00000045233	-0.957696693	9.94E-07
TUCP_003811	XLOC_166670	ENSRNOT00000019656	-0.957633583	1.00E-06
TUCP_003811	XLOC_166670	ENSRNOT00000049285	-0.95725923	1.05E-06
TUCP_003811	XLOC_166670	ENSRNOT00000003568	0.961262163	6.44E-07

TUCP_003811	XLOC_166670	ENSRNOT00000006366	-0.950924963	2.06E-06
TUCP_003811	XLOC_166670	ENSRNOT00000074649	0.973129092	1.05E-07
TUCP_003811	XLOC_166670	ENSRNOT00000033196	-0.957508561	1.02E-06
TUCP_003811	XLOC_166670	ENSRNOT00000077146	-0.972613088	1.16E-07
TUCP_003811	XLOC_166670	ENSRNOT00000052063	0.96260619	5.41E-07
TUCP_003811	XLOC_166670	ENSRNOT00000060002	-0.96875011	2.23E-07
TUCP_003811	XLOC_166670	ENSRNOT00000089673	-0.958979686	8.54E-07
TUCP_003811	XLOC_166670	ENSRNOT00000004046	-0.980497263	2.15E-08
TUCP_003811	XLOC_166670	ENSRNOT00000010367	0.972106009	1.27E-07
TUCP_003811	XLOC_166670	ENSRNOT00000057738	0.950535444	2.15E-06
TUCP_003811	XLOC_166670	ENSRNOT00000023977	-0.96934193	2.03E-07
TUCP_003811	XLOC_166670	ENSRNOT00000066133	-0.975076705	7.26E-08
TUCP_003811	XLOC_166670	ENSRNOT00000048933	-0.959176606	8.34E-07
TUCP_003811	XLOC_166670	ENSRNOT00000029136	-0.954958287	1.35E-06
TUCP_003811	XLOC_166670	ENSRNOT00000088509	0.957656735	9.98E-07
TUCP_003811	XLOC_166670	ENSRNOT00000086893	-0.95210155	1.83E-06
TUCP_003811	XLOC_166670	ENSRNOT00000019487	0.953397347	1.60E-06
TUCP_003811	XLOC_166670	ENSRNOT00000030692	-0.963740047	4.64E-07
TUCP_003811	XLOC_166670	ENSRNOT00000055992	-0.962831787	5.25E-07
TUCP_003811	XLOC_166670	ENSRNOT00000040069	0.967402027	2.74E-07
TUCP_003811	XLOC_166670	ENSRNOT00000015624	0.959702565	7.82E-07
TUCP_003811	XLOC_166670	ENSRNOT00000073235	0.950932923	2.06E-06
TUCP_003811	XLOC_166670	ENSRNOT00000085295	-0.953319532	1.61E-06
TUCP_003811	XLOC_166670	ENSRNOT00000008222	-0.950138084	2.23E-06
TUCP_003811	XLOC_166670	ENSRNOT00000035474	0.973401739	1.00E-07
TUCP_003811	XLOC_166670	ENSRNOT00000039102	0.96049325	7.09E-07
TUCP_003811	XLOC_166670	ENSRNOT00000023807	-0.95585512	1.23E-06
TUCP_003811	XLOC_166670	ENSRNOT00000078856	-0.966462306	3.16E-07
TUCP_003811	XLOC_166670	ENSRNOT00000091205	0.97897125	3.13E-08
TUCP_003811	XLOC_166670	ENSRNOT00000009742	-0.956869462	1.09E-06
TUCP_003811	XLOC_166670	ENSRNOT00000010983	0.95729992	1.04E-06
TUCP_003811	XLOC_166670	ENSRNOT00000007369	0.960176519	7.38E-07
TUCP_003811	XLOC_166670	ENSRNOT00000004787	-0.975015856	7.35E-08
TUCP_003811	XLOC_166670	ENSRNOT00000060698	-0.968284577	2.40E-07
TUCP_003811	XLOC_166670	ENSRNOT00000064535	-0.961404242	6.32E-07
TUCP_003811	XLOC_166670	ENSRNOT00000014814	0.969884987	1.85E-07
TUCP_003811	XLOC_166670	ENSRNOT00000022000	-0.954545626	1.42E-06
TUCP_003811	XLOC_166670	ENSRNOT00000010748	0.980545897	2.12E-08
TUCP_003811	XLOC_166670	ENSRNOT00000080207	0.977036911	4.84E-08
TUCP_003811	XLOC_166670	ENSRNOT00000061100	0.956091181	1.19E-06
TUCP_003811	XLOC_166670	ENSRNOT00000065529	0.964033892	4.46E-07
TUCP_003811	XLOC_166670	ENSRNOT00000017776	0.970789973	1.59E-07
TUCP_003811	XLOC_166670	ENSRNOT00000017995	0.957627579	1.00E-06
TUCP_003811	XLOC_166670	ENSRNOT00000021534	-0.958364575	9.19E-07
TUCP_003811	XLOC_166670	ENSRNOT00000088679	-0.95931011	8.20E-07
TUCP_003811	XLOC_166670	ENSRNOT00000004463	0.966513683	3.13E-07
TUCP_003811	XLOC_166670	ENSRNOT00000059807	0.956553513	1.13E-06
TUCP_003811	XLOC_166670	ENSRNOT00000016776	-0.959822135	7.71E-07
TUCP_003811	XLOC_166670	ENSRNOT00000090057	0.955129989	1.33E-06
TUCP_003811	XLOC_166670	ENSRNOT00000022097	0.958189509	9.38E-07
TUCP_003811	XLOC_166670	ENSRNOT00000008987	-0.976350718	5.60E-08

TUCP_003811	XLOC_166670	ENSRNOT00000092876	-0.963048226	5.10E-07
TUCP_003811	XLOC_166670	ENSRNOT00000024253	0.950084476	2.24E-06
TUCP_003811	XLOC_166670	ENSRNOT00000088073	-0.950796564	2.09E-06
TUCP_003811	XLOC_166670	ENSRNOT00000065143	0.967298098	2.79E-07
TUCP_003811	XLOC_166670	ENSRNOT00000040060	-0.960918369	6.72E-07
TUCP_003811	XLOC_166670	ENSRNOT00000027083	0.972366592	1.21E-07
TUCP_003811	XLOC_166670	ENSRNOT00000066041	-0.959140129	8.37E-07
TUCP_003811	XLOC_166670	ENSRNOT00000063930	-0.970741027	1.61E-07
TUCP_003811	XLOC_166670	ENSRNOT00000013116	-0.959770435	7.76E-07
TUCP_003811	XLOC_166670	ENSRNOT00000001290	0.950539541	2.14E-06
TUCP_003811	XLOC_166670	ENSRNOT00000001855	0.966065817	3.35E-07
TUCP_003811	XLOC_166670	ENSRNOT00000009563	-0.971655815	1.37E-07
TUCP_003811	XLOC_166670	ENSRNOT00000019502	0.959237022	8.28E-07
TUCP_003811	XLOC_166670	ENSRNOT00000087838	-0.95742437	1.03E-06
TUCP_003811	XLOC_166670	ENSRNOT00000081981	0.987726162	2.15E-09
TUCP_003811	XLOC_166670	ENSRNOT00000026531	-0.96559815	3.58E-07
TUCP_003811	XLOC_166670	ENSRNOT00000014479	-0.959642527	7.88E-07
TUCP_003811	XLOC_166670	ENSRNOT00000003326	-0.969876125	1.86E-07
TUCP_003811	XLOC_166670	ENSRNOT00000021629	-0.958199155	9.37E-07
TUCP_003811	XLOC_166670	ENSRNOT00000016306	-0.967282344	2.79E-07
TUCP_003811	XLOC_166670	ENSRNOT00000015476	-0.961418609	6.31E-07
TUCP_003811	XLOC_166670	ENSRNOT00000025079	-0.967050884	2.89E-07
TUCP_003811	XLOC_166670	ENSRNOT00000048754	-0.968540576	2.30E-07
TUCP_003811	XLOC_166670	ENSRNOT00000044628	0.957109213	1.06E-06
TUCP_003811	XLOC_166670	ENSRNOT00000025075	0.952447602	1.77E-06
TUCP_003811	XLOC_166670	ENSRNOT00000004007	-0.968235266	2.41E-07
TUCP_003811	XLOC_166670	ENSRNOT00000020681	-0.959832116	7.70E-07
TUCP_003811	XLOC_166670	ENSRNOT00000057898	-0.968714085	2.24E-07
TUCP_003811	XLOC_166670	ENSRNOT00000091602	-0.968902994	2.17E-07
TUCP_003811	XLOC_166670	ENSRNOT00000090338	-0.958537116	9.00E-07
TUCP_003811	XLOC_166670	ENSRNOT00000056100	-0.956834354	1.10E-06
TUCP_003811	XLOC_166670	ENSRNOT00000009811	0.978553472	3.45E-08
TUCP_003811	XLOC_166670	ENSRNOT00000006680	0.9530031	1.67E-06
TUCP_003811	XLOC_166670	ENSRNOT00000036838	-0.955314131	1.30E-06
TUCP_003811	XLOC_166670	ENSRNOT00000009817	-0.954109946	1.48E-06
TUCP_003811	XLOC_166670	ENSRNOT00000090129	-0.950064751	2.25E-06
TUCP_003811	XLOC_166670	ENSRNOT00000009708	-0.965594376	3.58E-07
TUCP_003811	XLOC_166670	ENSRNOT00000077231	0.968457957	2.33E-07
TUCP_003811	XLOC_166670	ENSRNOT00000060696	0.962658553	5.37E-07
TUCP_003811	XLOC_166670	ENSRNOT00000018634	-0.960026188	7.52E-07
TUCP_003811	XLOC_166670	ENSRNOT00000012583	-0.951858855	1.88E-06
TUCP_003811	XLOC_166670	ENSRNOT00000073643	-0.96229144	5.64E-07
TUCP_003811	XLOC_166670	ENSRNOT00000078349	-0.963469426	4.82E-07
TUCP_003811	XLOC_166670	ENSRNOT00000022502	-0.970780849	1.60E-07
TUCP_003811	XLOC_166670	ENSRNOT00000026928	-0.950908724	2.07E-06
TUCP_003811	XLOC_166670	ENSRNOT00000005329	-0.960555202	7.04E-07
TUCP_003811	XLOC_166670	ENSRNOT00000017556	0.963031643	5.11E-07
TUCP_003811	XLOC_166670	ENSRNOT00000087443	0.970159301	1.77E-07
TUCP_003811	XLOC_166670	ENSRNOT00000001759	-0.955850865	1.23E-06
TUCP_003811	XLOC_166670	ENSRNOT00000021803	-0.967951537	2.52E-07
TUCP_003811	XLOC_166670	ENSRNOT00000020147	0.953268316	1.62E-06

TUCP_003811	XLOC_166670	ENSRNOT00000024352	0.969467245	1.99E-07
TUCP_003811	XLOC_166670	ENSRNOT00000058351	0.978220636	3.72E-08
TUCP_003811	XLOC_166670	ENSRNOT00000083220	0.980187452	2.33E-08
TUCP_003811	XLOC_166670	ENSRNOT00000072118	0.954027325	1.50E-06
TUCP_003811	XLOC_166670	ENSRNOT00000003780	0.962688064	5.35E-07
TUCP_003811	XLOC_166670	ENSRNOT00000003787	-0.957155038	1.06E-06
TUCP_003811	XLOC_166670	ENSRNOT00000023262	-0.956640995	1.12E-06
TUCP_003811	XLOC_166670	ENSRNOT00000058312	0.951374268	1.97E-06
TUCP_003811	XLOC_166670	ENSRNOT00000060362	0.958095731	9.48E-07
TUCP_003811	XLOC_166670	ENSRNOT00000067009	-0.96022565	7.33E-07
TUCP_003811	XLOC_166670	ENSRNOT00000019816	-0.955001262	1.35E-06
TUCP_003811	XLOC_166670	ENSRNOT00000045946	0.957601922	1.00E-06
TUCP_003811	XLOC_166670	ENSRNOT00000010674	-0.951584798	1.93E-06
TUCP_003811	XLOC_166670	ENSRNOT00000016739	0.950059805	2.25E-06
TUCP_003811	XLOC_166670	ENSRNOT00000023664	-0.951601634	1.93E-06
TUCP_003811	XLOC_166670	ENSRNOT00000068381	0.95937116	8.14E-07
TUCP_003811	XLOC_166670	ENSRNOT00000068026	-0.956593961	1.13E-06
TUCP_003811	XLOC_166670	ENSRNOT00000020994	-0.959296523	8.22E-07
TUCP_003811	XLOC_166670	ENSRNOT00000007314	-0.970901879	1.56E-07
TUCP_003811	XLOC_166670	ENSRNOT00000014821	-0.964969555	3.92E-07
TUCP_003811	XLOC_166670	ENSRNOT00000058382	0.962014906	5.84E-07
TUCP_003811	XLOC_166670	ENSRNOT00000088599	0.973968668	9.01E-08
TUCP_003811	XLOC_166670	ENSRNOT00000005587	-0.953864122	1.52E-06
TUCP_003811	XLOC_166670	ENSRNOT00000064068	-0.957751523	9.87E-07
TUCP_003811	XLOC_166670	ENSRNOT00000023567	-0.963067228	5.09E-07
TUCP_003811	XLOC_166670	ENSRNOT00000023537	-0.963782136	4.62E-07
TUCP_003811	XLOC_166670	ENSRNOT00000070912	-0.958734624	8.79E-07
TUCP_003811	XLOC_166670	ENSRNOT00000026398	-0.957911735	9.69E-07
TUCP_003811	XLOC_166670	ENSRNOT00000050609	-0.961014551	6.64E-07
TUCP_003811	XLOC_166670	ENSRNOT00000005459	0.966117976	3.32E-07
TUCP_003811	XLOC_166670	ENSRNOT00000027910	0.982311534	1.32E-08
TUCP_003811	XLOC_166670	ENSRNOT00000037015	0.950589467	2.13E-06
TUCP_003811	XLOC_166670	ENSRNOT00000067135	-0.961670719	6.11E-07
TUCP_003811	XLOC_166670	ENSRNOT00000088182	0.953949436	1.51E-06
TUCP_003811	XLOC_166670	ENSRNOT00000054976	-0.962014541	5.84E-07
TUCP_003811	XLOC_166670	ENSRNOT00000032780	0.970028436	1.81E-07
TUCP_003811	XLOC_166670	ENSRNOT00000016191	0.957609479	1.00E-06
TUCP_003811	XLOC_166670	ENSRNOT00000028885	-0.958719336	8.81E-07
TUCP_003811	XLOC_166670	ENSRNOT00000016372	-0.964287355	4.31E-07
TUCP_003811	XLOC_166670	ENSRNOT00000088202	-0.952072862	1.84E-06
TUCP_003811	XLOC_166670	ENSRNOT00000029833	0.961218475	6.47E-07
TUCP_003811	XLOC_166670	ENSRNOT00000021145	-0.950288122	2.20E-06
TUCP_003811	XLOC_166670	ENSRNOT00000082151	-0.971596079	1.39E-07
TUCP_003811	XLOC_166670	ENSRNOT00000008982	0.984282298	7.36E-09
TUCP_003811	XLOC_166670	ENSRNOT00000001607	0.955659994	1.25E-06
TUCP_003811	XLOC_166670	ENSRNOT00000047192	-0.975120374	7.20E-08
TUCP_003811	XLOC_166670	ENSRNOT00000085971	-0.971459354	1.42E-07
TUCP_003811	XLOC_166670	ENSRNOT00000057537	0.950909902	2.07E-06
TUCP_003811	XLOC_166670	ENSRNOT00000022142	0.973579507	9.70E-08
TUCP_003811	XLOC_166670	ENSRNOT00000076300	-0.954591934	1.41E-06
TUCP_003811	XLOC_166670	ENSRNOT00000078390	-0.963219464	4.98E-07

TUCP_003811	XLOC_166670	ENSRNOT00000015537	0.968271443	2.40E-07
TUCP_003811	XLOC_166670	ENSRNOT00000052120	0.959546995	7.97E-07
TUCP_003811	XLOC_166670	ENSRNOT00000078306	0.966405263	3.19E-07
TUCP_003811	XLOC_166670	ENSRNOT00000011499	-0.95937114	8.14E-07
TUCP_003811	XLOC_166670	ENSRNOT00000015946	-0.962092415	5.78E-07
TUCP_003811	XLOC_166670	ENSRNOT00000004172	0.959231583	8.28E-07
TUCP_003811	XLOC_166670	ENSRNOT00000023965	-0.954907704	1.36E-06
TUCP_003811	XLOC_166670	ENSRNOT00000040476	0.957543267	1.01E-06
TUCP_003811	XLOC_166670	ENSRNOT00000090355	-0.951754623	1.90E-06
TUCP_003811	XLOC_166670	ENSRNOT00000002410	0.951698113	1.91E-06
TUCP_003811	XLOC_166670	ENSRNOT00000029125	0.982341929	1.31E-08
TUCP_003811	XLOC_166670	ENSRNOT00000085279	0.95161395	1.93E-06
TUCP_003811	XLOC_166670	ENSRNOT00000004319	-0.969117283	2.10E-07
TUCP_003811	XLOC_166670	ENSRNOT00000025819	-0.950251213	2.21E-06
TUCP_003811	XLOC_166670	ENSRNOT00000015318	-0.952871045	1.69E-06
TUCP_003811	XLOC_166670	ENSRNOT00000083632	-0.967353994	2.76E-07
TUCP_003811	XLOC_166670	ENSRNOT00000025147	0.950390625	2.18E-06
TUCP_003811	XLOC_166670	ENSRNOT00000045532	-0.957081604	1.07E-06
TUCP_003811	XLOC_166670	ENSRNOT00000025887	-0.961289749	6.41E-07
TUCP_003811	XLOC_166670	ENSRNOT00000078862	0.955501634	1.27E-06
TUCP_003811	XLOC_166670	ENSRNOT00000005311	0.972221754	1.24E-07
TUCP_003811	XLOC_166670	ENSRNOT00000005257	0.965597852	3.58E-07
TUCP_003811	XLOC_166670	ENSRNOT00000015799	0.981093998	1.84E-08
TUCP_003811	XLOC_166670	ENSRNOT00000036506	0.957052287	1.07E-06
TUCP_003811	XLOC_166670	ENSRNOT00000005958	0.984587128	6.68E-09
TUCP_003811	XLOC_166670	ENSRNOT00000039551	-0.966039704	3.36E-07
TUCP_003811	XLOC_166670	ENSRNOT00000082999	-0.950870936	2.08E-06
TUCP_003811	XLOC_166670	ENSRNOT00000082998	0.97430582	8.45E-08
TUCP_003811	XLOC_166670	ENSRNOT00000075656	-0.954945439	1.36E-06
TUCP_003811	XLOC_166670	ENSRNOT00000015006	0.953310234	1.62E-06
TUCP_003811	XLOC_166670	ENSRNOT00000007350	0.956920456	1.09E-06
TUCP_003811	XLOC_166670	ENSRNOT00000004956	0.953090641	1.65E-06
TUCP_003811	XLOC_166670	ENSRNOT00000083578	0.962531069	5.46E-07
TUCP_003811	XLOC_166670	ENSRNOT00000023638	0.986934693	2.93E-09
TUCP_003811	XLOC_166670	ENSRNOT00000005228	0.959468259	8.05E-07
TUCP_003811	XLOC_166670	ENSRNOT00000059524	-0.969113212	2.10E-07
TUCP_003811	XLOC_166670	ENSRNOT00000017361	0.958186569	9.38E-07
TUCP_003811	XLOC_166670	ENSRNOT00000005220	0.969122079	2.10E-07
TUCP_003811	XLOC_166670	ENSRNOT00000023144	0.971815346	1.34E-07
TUCP_003811	XLOC_166670	ENSRNOT00000045326	0.965154678	3.82E-07
TUCP_003811	XLOC_166670	ENSRNOT00000016649	0.974801218	7.67E-08
TUCP_003811	XLOC_166670	ENSRNOT00000090342	0.954435387	1.43E-06
TUCP_003811	XLOC_166670	ENSRNOT00000074023	0.973536551	9.78E-08
TUCP_003811	XLOC_166670	ENSRNOT00000019465	-0.957744094	9.88E-07
TUCP_003811	XLOC_166670	ENSRNOT00000009073	-0.952227231	1.81E-06
TUCP_003811	XLOC_166670	ENSRNOT00000067170	-0.970468086	1.68E-07
TUCP_003811	XLOC_166670	ENSRNOT00000091128	-0.966941954	2.94E-07
TUCP_003811	XLOC_166670	ENSRNOT00000001586	-0.9730392	1.07E-07
TUCP_003811	XLOC_166670	ENSRNOT00000026543	0.954408465	1.44E-06
TUCP_003811	XLOC_166670	ENSRNOT00000019213	0.959006639	8.51E-07
TUCP_003811	XLOC_166670	ENSRNOT00000012187	0.966290157	3.24E-07

TUCP_003811	XLOC_166670	ENSRNOT00000024969	0.978379042	3.59E-08
TUCP_003811	XLOC_166670	ENSRNOT00000013695	-0.955760859	1.24E-06
TUCP_003811	XLOC_166670	ENSRNOT00000081621	0.957561467	1.01E-06
TUCP_003811	XLOC_166670	ENSRNOT00000027301	-0.95180943	1.89E-06
TUCP_003811	XLOC_166670	ENSRNOT00000038247	0.958814275	8.71E-07
TUCP_003811	XLOC_166670	ENSRNOT00000009000	-0.950103431	2.24E-06
TUCP_003811	XLOC_166670	ENSRNOT00000024402	0.971647086	1.38E-07
TUCP_003811	XLOC_166670	ENSRNOT00000089545	-0.957677799	9.96E-07
TUCP_003811	XLOC_166670	ENSRNOT00000019519	0.952259604	1.80E-06
TUCP_003811	XLOC_166670	ENSRNOT00000089933	0.99188731	2.73E-10
TUCP_003811	XLOC_166670	ENSRNOT00000077908	0.956304286	1.17E-06
TUCP_003811	XLOC_166670	ENSRNOT00000083876	-0.960095277	7.45E-07
TUCP_003811	XLOC_166670	ENSRNOT00000026481	0.971741097	1.35E-07
TUCP_003811	XLOC_166670	ENSRNOT00000067554	0.959484018	8.03E-07
TUCP_003811	XLOC_166670	ENSRNOT00000026718	0.951953483	1.86E-06
TUCP_003811	XLOC_166670	ENSRNOT00000072208	0.962251125	5.67E-07
TUCP_003811	XLOC_166670	ENSRNOT00000010784	0.952907086	1.69E-06
TUCP_003811	XLOC_166670	ENSRNOT00000084012	0.969935242	1.84E-07
TUCP_003811	XLOC_166670	ENSRNOT00000033677	-0.968606868	2.28E-07
TUCP_003811	XLOC_166670	ENSRNOT00000019820	0.975475452	6.71E-08
TUCP_003811	XLOC_166670	ENSRNOT00000083351	-0.95196877	1.86E-06
TUCP_003811	XLOC_166670	ENSRNOT00000011832	-0.956826314	1.10E-06
TUCP_003811	XLOC_166670	ENSRNOT00000016428	0.954897885	1.36E-06
TUCP_003811	XLOC_166670	ENSRNOT00000021030	-0.964993588	3.90E-07
TUCP_003811	XLOC_166670	ENSRNOT00000091909	0.979695897	2.63E-08
TUCP_003811	XLOC_166670	ENSRNOT00000077727	-0.965417494	3.68E-07
TUCP_003811	XLOC_166670	ENSRNOT00000011930	0.95818486	9.38E-07
TUCP_003811	XLOC_166670	ENSRNOT00000044346	-0.961062089	6.60E-07
TUCP_003811	XLOC_166670	ENSRNOT00000073492	-0.952649236	1.73E-06
TUCP_003811	XLOC_166670	ENSRNOT00000010541	0.971730934	1.36E-07
TUCP_003811	XLOC_166670	ENSRNOT00000017949	-0.958717644	8.81E-07
TUCP_003811	XLOC_166670	ENSRNOT00000013864	-0.955859585	1.23E-06
TUCP_003811	XLOC_166670	ENSRNOT00000001081	0.969077936	2.11E-07
TUCP_003811	XLOC_166670	ENSRNOT00000013025	-0.960693233	6.92E-07
TUCP_003811	XLOC_166670	ENSRNOT00000003544	-0.96400201	4.48E-07
TUCP_003811	XLOC_166670	ENSRNOT00000086119	-0.974152838	8.70E-08
TUCP_003811	XLOC_166670	ENSRNOT00000022190	-0.974264888	8.51E-08
TUCP_003811	XLOC_166670	ENSRNOT00000025913	-0.973610941	9.64E-08
TUCP_003811	XLOC_166670	ENSRNOT00000019386	-0.965864766	3.45E-07
TUCP_003811	XLOC_166670	ENSRNOT00000077220	-0.956554425	1.13E-06
TUCP_003811	XLOC_166670	ENSRNOT00000065983	0.960747274	6.87E-07
TUCP_003811	XLOC_166670	ENSRNOT00000030782	-0.953906711	1.52E-06
TUCP_003811	XLOC_166670	ENSRNOT00000067780	-0.963959152	4.51E-07
TUCP_003811	XLOC_166670	ENSRNOT00000086577	0.958241132	9.32E-07
TUCP_003811	XLOC_166670	ENSRNOT00000025966	0.968884901	2.18E-07
TUCP_003811	XLOC_166670	ENSRNOT00000007738	-0.959679469	7.84E-07
TUCP_003811	XLOC_166670	ENSRNOT00000052422	0.963443303	4.84E-07
TUCP_003811	XLOC_166670	ENSRNOT00000025980	-0.951409777	1.97E-06
TUCP_003811	XLOC_166670	ENSRNOT00000026174	0.951930775	1.86E-06
TUCP_003811	XLOC_166670	ENSRNOT00000034883	-0.969771299	1.89E-07
TUCP_003811	XLOC_166670	ENSRNOT00000020155	0.962754376	5.30E-07

TUCP_003811	XLOC_166670	ENSRNOT00000082869	0.952315939	1.79E-06
TUCP_003811	XLOC_166670	ENSRNOT00000055321	0.966154185	3.30E-07
TUCP_003811	XLOC_166670	ENSRNOT00000005910	-0.96712255	2.86E-07
TUCP_003811	XLOC_166670	ENSRNOT00000078475	0.965830536	3.46E-07
TUCP_003811	XLOC_166670	ENSRNOT00000087429	0.963408423	4.86E-07
TUCP_003811	XLOC_166670	ENSRNOT00000029701	0.95193053	1.86E-06
TUCP_003811	XLOC_166670	ENSRNOT00000035591	-0.953297002	1.62E-06
TUCP_003811	XLOC_166670	ENSRNOT00000016722	0.95686223	1.09E-06
TUCP_003811	XLOC_166670	ENSRNOT00000000013	0.968104024	2.46E-07
TUCP_003811	XLOC_166670	ENSRNOT00000080591	0.952530209	1.75E-06
TUCP_003811	XLOC_166670	ENSRNOT00000030329	-0.95026791	2.20E-06
TUCP_003811	XLOC_166670	ENSRNOT00000031904	0.964513678	4.18E-07
TUCP_003811	XLOC_166670	ENSRNOT00000022074	0.96198455	5.87E-07
TUCP_003811	XLOC_166670	ENSRNOT00000004999	0.963405087	4.86E-07
TUCP_003811	XLOC_166670	ENSRNOT00000009122	0.957247213	1.05E-06
TUCP_003811	XLOC_166670	ENSRNOT00000013463	0.967629985	2.65E-07
TUCP_003811	XLOC_166670	ENSRNOT00000088054	0.958298819	9.26E-07
TUCP_003811	XLOC_166670	ENSRNOT00000036658	-0.953355788	1.61E-06
TUCP_003811	XLOC_166670	ENSRNOT00000047663	-0.961165118	6.52E-07
TUCP_003811	XLOC_166670	ENSRNOT00000005486	-0.966634966	3.08E-07
TUCP_003811	XLOC_166670	ENSRNOT00000017907	-0.969460207	1.99E-07
TUCP_003811	XLOC_166670	ENSRNOT00000092029	0.954453292	1.43E-06
TUCP_003811	XLOC_166670	ENSRNOT00000005489	0.958737798	8.79E-07
TUCP_003811	XLOC_166670	ENSRNOT00000025794	-0.971444473	1.43E-07
TUCP_003811	XLOC_166670	ENSRNOT00000058111	-0.954806326	1.38E-06
TUCP_003811	XLOC_166670	ENSRNOT00000084422	0.971405962	1.43E-07
TUCP_003811	XLOC_166670	ENSRNOT00000050834	-0.977637994	4.24E-08
TUCP_003811	XLOC_166670	ENSRNOT00000054828	0.959930468	7.60E-07
TUCP_003811	XLOC_166670	ENSRNOT00000002443	0.97745088	4.42E-08
TUCP_003811	XLOC_166670	ENSRNOT00000002114	-0.976890438	4.99E-08
TUCP_003811	XLOC_166670	ENSRNOT00000002445	-0.962028804	5.83E-07
TUCP_003811	XLOC_166670	ENSRNOT00000027970	0.984545226	6.77E-09
TUCP_003811	XLOC_166670	ENSRNOT00000046215	-0.968816389	2.20E-07
TUCP_003811	XLOC_166670	ENSRNOT00000025009	0.959773908	7.75E-07
TUCP_003811	XLOC_166670	ENSRNOT00000000083	-0.957187221	1.05E-06
TUCP_003811	XLOC_166670	ENSRNOT00000018074	-0.97007252	1.80E-07
TUCP_003811	XLOC_166670	ENSRNOT00000037679	0.950266985	2.20E-06
TUCP_003811	XLOC_166670	ENSRNOT00000014124	0.969614526	1.94E-07
TUCP_003811	XLOC_166670	ENSRNOT00000000120	0.989823743	8.45E-10
TUCP_003811	XLOC_166670	ENSRNOT00000013632	0.96444659	4.21E-07
TUCP_003811	XLOC_166670	ENSRNOT00000028545	-0.957061082	1.07E-06
TUCP_003811	XLOC_166670	ENSRNOT00000009900	-0.972562061	1.17E-07
TUCP_003811	XLOC_166670	ENSRNOT00000017753	0.95237952	1.78E-06
TUCP_003811	XLOC_166670	ENSRNOT00000019172	-0.965363608	3.70E-07
TUCP_003811	XLOC_166670	ENSRNOT00000064798	0.959368791	8.15E-07
TUCP_003811	XLOC_166670	ENSRNOT00000015495	-0.952039838	1.84E-06
TUCP_003811	XLOC_166670	ENSRNOT00000032248	0.969251187	2.06E-07
TUCP_003811	XLOC_166670	ENSRNOT00000019911	-0.955577893	1.26E-06
TUCP_003811	XLOC_166670	ENSRNOT00000040393	0.97682869	5.06E-08
TUCP_003811	XLOC_166670	ENSRNOT00000032236	-0.951526245	1.94E-06
TUCP_003811	XLOC_166670	ENSRNOT00000067901	-0.971247014	1.47E-07

TUCP_003811	XLOC_166670	ENSRNOT00000012286	-0.951936001	1.86E-06
TUCP_003811	XLOC_166670	ENSRNOT00000021674	0.96199546	5.86E-07
TUCP_003811	XLOC_166670	ENSRNOT00000064700	0.96536516	3.70E-07
TUCP_003811	XLOC_166670	ENSRNOT00000036613	-0.980330806	2.24E-08
TUCP_003811	XLOC_166670	ENSRNOT00000028876	-0.954102166	1.48E-06
TUCP_003811	XLOC_166670	ENSRNOT00000025705	0.966775355	3.02E-07
TUCP_003811	XLOC_166670	ENSRNOT00000019983	-0.957341668	1.04E-06
TUCP_003811	XLOC_166670	ENSRNOT00000004140	0.959012503	8.50E-07
TUCP_003811	XLOC_166670	ENSRNOT00000008113	-0.959815303	7.71E-07
TUCP_003811	XLOC_166670	ENSRNOT00000022552	-0.955558285	1.27E-06
TUCP_003811	XLOC_166670	ENSRNOT00000036194	-0.952649144	1.73E-06
TUCP_003811	XLOC_166670	ENSRNOT00000086590	0.951728436	1.90E-06
TUCP_003811	XLOC_166670	ENSRNOT00000048910	-0.959397067	8.12E-07
TUCP_003811	XLOC_166670	ENSRNOT00000009645	-0.950307478	2.19E-06
TUCP_003811	XLOC_166670	ENSRNOT00000026279	-0.965611123	3.58E-07
TUCP_003811	XLOC_166670	ENSRNOT00000082508	-0.965930298	3.41E-07
TUCP_003811	XLOC_166670	ENSRNOT00000082863	0.970270195	1.74E-07
TUCP_003811	XLOC_166670	ENSRNOT00000049459	-0.958728062	8.80E-07
TUCP_003811	XLOC_166670	ENSRNOT00000014309	-0.953003013	1.67E-06
TUCP_003811	XLOC_166670	ENSRNOT00000071166	0.96003063	7.51E-07
TUCP_003811	XLOC_166670	ENSRNOT00000074389	-0.952849892	1.70E-06
TUCP_003811	XLOC_166670	ENSRNOT00000028646	0.952396951	1.78E-06
TUCP_003811	XLOC_166670	ENSRNOT00000066204	-0.961590471	6.17E-07
TUCP_003811	XLOC_166670	ENSRNOT00000039836	-0.952099803	1.83E-06
TUCP_003811	XLOC_166670	ENSRNOT00000009496	0.950191661	2.22E-06
TUCP_003811	XLOC_166670	ENSRNOT00000076872	0.955136137	1.33E-06
TUCP_003811	XLOC_166670	ENSRNOT00000073564	-0.961944927	5.90E-07
TUCP_003811	XLOC_166670	ENSRNOT00000001507	0.960039653	7.50E-07
TUCP_003811	XLOC_166670	ENSRNOT00000083568	0.969572772	1.95E-07
TUCP_003811	XLOC_166670	ENSRNOT00000039177	-0.957835409	9.78E-07
TUCP_003811	XLOC_166670	ENSRNOT00000081047	0.982138007	1.39E-08
TUCP_003811	XLOC_166670	ENSRNOT00000002837	-0.967901952	2.54E-07
TUCP_003811	XLOC_166670	ENSRNOT00000037456	0.963575881	4.75E-07
TUCP_003811	XLOC_166670	ENSRNOT00000012881	-0.964391924	4.25E-07
TUCP_003811	XLOC_166670	ENSRNOT00000000577	0.961673222	6.11E-07
TUCP_003811	XLOC_166670	ENSRNOT00000075424	0.954362827	1.44E-06
TUCP_003811	XLOC_166670	ENSRNOT00000041513	0.955878995	1.22E-06
TUCP_003811	XLOC_166670	ENSRNOT00000023989	0.963529444	4.78E-07
TUCP_003811	XLOC_166670	ENSRNOT00000088133	-0.968401669	2.35E-07
TUCP_003811	XLOC_166670	ENSRNOT00000080677	-0.968867276	2.19E-07
TUCP_003811	XLOC_166670	ENSRNOT00000037039	-0.965010603	3.89E-07
TUCP_003811	XLOC_166670	ENSRNOT00000007824	-0.956839367	1.10E-06
TUCP_003811	XLOC_166670	ENSRNOT00000003714	0.96222688	5.68E-07
TUCP_003811	XLOC_166670	ENSRNOT00000083968	-0.9521068	1.83E-06
TUCP_003811	XLOC_166670	ENSRNOT00000037133	0.968370066	2.36E-07
TUCP_003811	XLOC_166670	ENSRNOT00000024119	0.961757315	6.04E-07
TUCP_003811	XLOC_166670	ENSRNOT00000083964	-0.965524975	3.62E-07
TUCP_003811	XLOC_166670	ENSRNOT00000075452	0.952652426	1.73E-06
TUCP_003811	XLOC_166670	ENSRNOT00000061304	-0.957757257	9.87E-07
TUCP_003811	XLOC_166670	ENSRNOT00000032363	0.963551796	4.77E-07
TUCP_003811	XLOC_166670	ENSRNOT00000016445	0.960079416	7.47E-07

TUCP_003811	XLOC_166670	ENSRNOT00000019133	0.974682457	7.85E-08
TUCP_003811	XLOC_166670	ENSRNOT00000078185	-0.950702933	2.11E-06
TUCP_003811	XLOC_166670	ENSRNOT00000002151	-0.952494746	1.76E-06
TUCP_003811	XLOC_166670	ENSRNOT00000002187	-0.951541653	1.94E-06
TUCP_003811	XLOC_166670	ENSRNOT00000002152	-0.950253162	2.21E-06
TUCP_003811	XLOC_166670	ENSRNOT00000006824	-0.962341645	5.60E-07
TUCP_003811	XLOC_166670	ENSRNOT00000012775	-0.950853853	2.08E-06
TUCP_003811	XLOC_166670	ENSRNOT00000086260	-0.956479899	1.14E-06
TUCP_003811	XLOC_166670	ENSRNOT00000018328	-0.952015419	1.85E-06
TUCP_003811	XLOC_166670	ENSRNOT00000061392	0.97461189	7.96E-08
TUCP_003811	XLOC_166670	ENSRNOT00000044608	-0.96437765	4.26E-07
TUCP_003811	XLOC_166670	ENSRNOT00000001099	-0.953183321	1.64E-06
TUCP_003811	XLOC_166670	ENSRNOT00000090123	0.959961929	7.58E-07
TUCP_003811	XLOC_166670	ENSRNOT00000010683	-0.956814	1.10E-06
TUCP_003811	XLOC_166670	ENSRNOT00000045488	0.952290154	1.80E-06
TUCP_003811	XLOC_166670	ENSRNOT00000050119	-0.950323665	2.19E-06
TUCP_003811	XLOC_166670	ENSRNOT00000090853	-0.959728982	7.80E-07
TUCP_003811	XLOC_166670	ENSRNOT00000093174	0.952856981	1.69E-06
TUCP_003811	XLOC_166670	ENSRNOT00000089211	0.968475007	2.33E-07
TUCP_003811	XLOC_166670	ENSRNOT00000020383	0.957912613	9.69E-07
TUCP_003811	XLOC_166670	ENSRNOT00000015518	-0.956657583	1.12E-06
TUCP_003811	XLOC_166670	ENSRNOT00000000302	0.974061743	8.85E-08
TUCP_003811	XLOC_166670	ENSRNOT00000055602	-0.961327249	6.38E-07
TUCP_003811	XLOC_166670	ENSRNOT00000015092	0.964835009	3.99E-07
TUCP_003811	XLOC_166670	ENSRNOT00000004290	0.953768679	1.54E-06
TUCP_003811	XLOC_166670	ENSRNOT00000017181	-0.954450119	1.43E-06
TUCP_003811	XLOC_166670	ENSRNOT00000005909	0.959360819	8.15E-07
TUCP_003811	XLOC_166670	ENSRNOT00000049725	-0.95469348	1.39E-06
TUCP_003811	XLOC_166670	ENSRNOT00000086122	0.963481197	4.81E-07
TUCP_003811	XLOC_166670	ENSRNOT00000026235	-0.957199063	1.05E-06
TUCP_003811	XLOC_166670	ENSRNOT00000010753	-0.951532295	1.94E-06
TUCP_003811	XLOC_166670	ENSRNOT00000023763	-0.959835892	7.69E-07
TUCP_003811	XLOC_166670	ENSRNOT00000091596	-0.954290451	1.46E-06
TUCP_003811	XLOC_166670	ENSRNOT00000003940	-0.969729129	1.90E-07
TUCP_003811	XLOC_166670	ENSRNOT00000058842	0.956519693	1.14E-06
TUCP_003811	XLOC_166670	ENSRNOT00000042799	0.959277271	8.24E-07
TUCP_003811	XLOC_166670	ENSRNOT00000059181	0.966669425	3.06E-07
TUCP_003811	XLOC_166670	ENSRNOT00000017291	-0.959390999	8.12E-07
TUCP_003811	XLOC_166670	ENSRNOT00000003691	-0.972511342	1.18E-07
TUCP_003811	XLOC_166670	ENSRNOT00000003690	0.95541688	1.29E-06
TUCP_003811	XLOC_166670	ENSRNOT00000038807	0.96220546	5.70E-07
TUCP_003811	XLOC_166670	ENSRNOT00000036931	-0.9500356	2.25E-06
TUCP_003811	XLOC_166670	ENSRNOT00000008901	-0.972171245	1.25E-07
TUCP_003811	XLOC_166670	ENSRNOT00000005659	0.958392531	9.16E-07
TUCP_003811	XLOC_166670	ENSRNOT00000081077	-0.954837447	1.37E-06
TUCP_003811	XLOC_166670	ENSRNOT00000017761	0.97564551	6.48E-08
TUCP_003811	XLOC_166670	ENSRNOT00000023543	-0.95806395	9.52E-07
TUCP_003811	XLOC_166670	ENSRNOT00000026691	0.953130447	1.65E-06
TUCP_003811	XLOC_166670	ENSRNOT00000080608	0.979200732	2.96E-08
TUCP_003811	XLOC_166670	ENSRNOT00000008757	0.951192578	2.01E-06
TUCP_003811	XLOC_166670	ENSRNOT00000005478	-0.952741312	1.71E-06

TUCP_003811	XLOC_166670	ENSRNOT00000016740	0.950083489	2.24E-06
TUCP_003811	XLOC_166670	ENSRNOT00000080695	0.973381101	1.01E-07
TUCP_003811	XLOC_166670	ENSRNOT00000039341	0.963452458	4.83E-07
TUCP_003811	XLOC_166670	ENSRNOT00000055386	0.963019311	5.12E-07
TUCP_003811	XLOC_166670	ENSRNOT00000038212	-0.963671149	4.69E-07
TUCP_003811	XLOC_166670	ENSRNOT00000085519	0.954770876	1.38E-06
TUCP_003811	XLOC_166670	ENSRNOT00000042125	0.995890479	9.17E-12
TUCP_003811	XLOC_166670	ENSRNOT00000025460	0.952925813	1.68E-06
TUCP_003811	XLOC_166670	ENSRNOT00000026760	-0.972017747	1.29E-07
TUCP_003811	XLOC_166670	ENSRNOT00000017687	-0.955077716	1.34E-06
TUCP_003811	XLOC_166670	ENSRNOT00000024491	-0.967503851	2.70E-07
TUCP_003811	XLOC_166670	ENSRNOT00000084597	-0.95180094	1.89E-06
TUCP_003811	XLOC_166670	ENSRNOT00000016568	0.956652144	1.12E-06
TUCP_003811	XLOC_166670	ENSRNOT00000024947	-0.962860134	5.23E-07
TUCP_003811	XLOC_166670	ENSRNOT00000027967	0.968636704	2.27E-07
TUCP_003811	XLOC_166670	ENSRNOT00000047210	0.974477617	8.17E-08
TUCP_003811	XLOC_166670	ENSRNOT00000080110	0.955221378	1.31E-06
TUCP_003811	XLOC_166670	ENSRNOT00000083957	0.966323261	3.22E-07
TUCP_003811	XLOC_166670	ENSRNOT00000046639	0.953211929	1.63E-06
TUCP_003811	XLOC_166670	ENSRNOT00000005885	-0.955911247	1.22E-06
TUCP_003811	XLOC_166670	ENSRNOT00000079996	0.963482645	4.81E-07
TUCP_004754	XLOC_209508	ENSRNOT00000092105	0.988121184	1.83E-09
TUCP_004754	XLOC_209508	ENSRNOT00000031076	0.983495451	9.38E-09
TUCP_004754	XLOC_209508	ENSRNOT00000072306	0.991887782	2.73E-10
TUCP_004754	XLOC_209508	ENSRNOT00000087907	0.992484443	1.86E-10
TUCP_004754	XLOC_209508	ENSRNOT00000000215	0.982052617	1.42E-08
TUCP_004754	XLOC_209508	ENSRNOT00000078672	0.988728213	1.41E-09
TUCP_004756	XLOC_209565	ENSRNOT00000040509	0.976031179	5.98E-08
TUCP_004756	XLOC_209565	ENSRNOT00000012170	0.951059759	2.04E-06
TUCP_004756	XLOC_209565	ENSRNOT00000089396	0.960864385	6.77E-07
TUCP_004757	XLOC_209565	ENSRNOT00000072396	0.950286685	2.20E-06
TUCP_002086	XLOC_085776	ENSRNOT00000039729	0.969774204	1.89E-07
TUCP_002086	XLOC_085776	ENSRNOT00000027891	0.977877471	4.02E-08
TUCP_002086	XLOC_085776	ENSRNOT00000055127	0.955539129	1.27E-06
TUCP_002086	XLOC_085776	ENSRNOT00000066764	0.967946968	2.52E-07
TUCP_002086	XLOC_085776	ENSRNOT00000026677	0.951743914	1.90E-06
TUCP_002086	XLOC_085776	ENSRNOT00000019311	0.961561262	6.20E-07
TUCP_002086	XLOC_085776	ENSRNOT00000021816	0.951505077	1.95E-06
TUCP_002086	XLOC_085776	ENSRNOT00000003320	0.974583089	8.01E-08
TUCP_002086	XLOC_085776	ENSRNOT00000083702	0.987190815	2.66E-09
TUCP_002086	XLOC_085776	ENSRNOT00000028067	0.960657895	6.95E-07
TUCP_002086	XLOC_085776	ENSRNOT00000000640	0.96103747	6.62E-07
TUCP_002086	XLOC_085776	ENSRNOT00000028007	0.983473551	9.44E-09
TUCP_002086	XLOC_085776	ENSRNOT00000092857	0.950797479	2.09E-06
TUCP_002086	XLOC_085776	ENSRNOT00000007358	0.965280729	3.75E-07
TUCP_002086	XLOC_085776	ENSRNOT00000084429	0.954620381	1.40E-06
TUCP_002086	XLOC_085776	ENSRNOT00000055888	0.951528876	1.94E-06
TUCP_002086	XLOC_085776	ENSRNOT00000004637	0.970815752	1.59E-07
TUCP_002086	XLOC_085776	ENSRNOT00000020475	0.950265717	2.20E-06
TUCP_002086	XLOC_085776	ENSRNOT00000010079	0.953672653	1.55E-06
TUCP_002086	XLOC_085776	ENSRNOT00000056551	0.950791964	2.09E-06

TUCP_002086	XLOC_085776	ENSRNOT00000066618	0.950431827	2.17E-06
TUCP_002086	XLOC_085776	ENSRNOT00000045139	0.950060453	2.25E-06
TUCP_002086	XLOC_085776	ENSRNOT00000015576	0.961151646	6.53E-07
TUCP_002086	XLOC_085776	ENSRNOT00000090186	0.96866894	2.26E-07
TUCP_002086	XLOC_085776	ENSRNOT00000057016	0.952822372	1.70E-06
TUCP_002086	XLOC_085776	ENSRNOT00000022253	0.969158686	2.09E-07
TUCP_002086	XLOC_085776	ENSRNOT00000022373	0.952956149	1.68E-06
TUCP_002086	XLOC_085776	ENSRNOT00000056995	0.976835667	5.05E-08
TUCP_002086	XLOC_085776	ENSRNOT00000080900	0.95049251	2.15E-06
TUCP_002086	XLOC_085776	ENSRNOT00000033719	0.956907542	1.09E-06
TUCP_002086	XLOC_085776	ENSRNOT00000011134	0.968911509	2.17E-07
TUCP_002086	XLOC_085776	ENSRNOT00000088553	0.951187718	2.01E-06
TUCP_002086	XLOC_085776	ENSRNOT00000091499	0.97442693	8.25E-08
TUCP_002086	XLOC_085776	ENSRNOT00000032782	-0.953991193	1.50E-06
TUCP_002086	XLOC_085776	ENSRNOT00000081893	0.963197382	5.00E-07
TUCP_002086	XLOC_085776	ENSRNOT00000039005	0.960651833	6.95E-07
TUCP_002086	XLOC_085776	ENSRNOT00000001570	0.951531588	1.94E-06
TUCP_002086	XLOC_085776	ENSRNOT00000004320	0.953286574	1.62E-06
TUCP_002086	XLOC_085776	ENSRNOT00000019361	0.962719507	5.33E-07
TUCP_002086	XLOC_085776	ENSRNOT00000082645	0.96392033	4.53E-07
TUCP_002086	XLOC_085776	ENSRNOT00000065288	0.954337238	1.45E-06
TUCP_002086	XLOC_085776	ENSRNOT00000014912	0.954394585	1.44E-06
TUCP_002086	XLOC_085776	ENSRNOT00000090867	0.963829483	4.59E-07
TUCP_002086	XLOC_085776	ENSRNOT00000029990	0.953229008	1.63E-06
TUCP_002086	XLOC_085776	ENSRNOT00000035523	0.951517248	1.94E-06
TUCP_002086	XLOC_085776	ENSRNOT00000017878	0.990177982	7.08E-10
TUCP_002086	XLOC_085776	ENSRNOT00000092211	0.957436134	1.02E-06
TUCP_002086	XLOC_085776	ENSRNOT00000023504	0.958838188	8.68E-07
TUCP_002086	XLOC_085776	ENSRNOT00000028423	0.964100708	4.42E-07
TUCP_002086	XLOC_085776	ENSRNOT00000033984	0.953370317	1.61E-06
TUCP_002086	XLOC_085776	ENSRNOT00000006246	0.968233996	2.42E-07
TUCP_002086	XLOC_085776	ENSRNOT00000030344	0.957834283	9.78E-07
TUCP_002086	XLOC_085776	ENSRNOT00000011481	0.983524483	9.30E-09
TUCP_002086	XLOC_085776	ENSRNOT00000086390	0.95650414	1.14E-06
TUCP_002086	XLOC_085776	ENSRNOT00000049536	0.956404567	1.15E-06
TUCP_002086	XLOC_085776	ENSRNOT00000044319	0.955320951	1.30E-06
TUCP_002086	XLOC_085776	ENSRNOT00000086459	0.964810239	4.01E-07
TUCP_002086	XLOC_085776	ENSRNOT00000027949	0.956630195	1.12E-06
TUCP_002086	XLOC_085776	ENSRNOT00000073952	0.954753518	1.38E-06
TUCP_002086	XLOC_085776	ENSRNOT00000036753	0.964758186	4.04E-07
TUCP_002086	XLOC_085776	ENSRNOT00000010673	0.969657343	1.92E-07
TUCP_002086	XLOC_085776	ENSRNOT00000080956	0.95379459	1.53E-06
TUCP_002086	XLOC_085776	ENSRNOT00000026331	0.9614872	6.25E-07
TUCP_002086	XLOC_085776	ENSRNOT00000039377	0.951823387	1.88E-06
TUCP_002086	XLOC_085776	ENSRNOT00000081036	0.952327605	1.79E-06
TUCP_002086	XLOC_085776	ENSRNOT00000050656	0.979045352	3.07E-08
TUCP_002086	XLOC_085776	ENSRNOT00000009840	0.969571983	1.95E-07
TUCP_002086	XLOC_085776	ENSRNOT00000032015	0.956842687	1.10E-06
TUCP_002086	XLOC_085776	ENSRNOT00000013904	0.961691537	6.09E-07
TUCP_002086	XLOC_085776	ENSRNOT00000057992	0.965396268	3.69E-07
TUCP_002086	XLOC_085776	ENSRNOT00000044254	0.95317367	1.64E-06

TUCP_002086	XLOC_085776	ENSRNOT00000002936	0.989203282	1.13E-09
TUCP_002086	XLOC_085776	ENSRNOT00000054757	0.954682607	1.39E-06
TUCP_000755	XLOC_028292	ENSRNOT00000083701	0.953207531	1.63E-06
TUCP_002084	XLOC_085596	ENSRNOT00000018693	0.953010849	1.67E-06
TUCP_002085	XLOC_085612	ENSRNOT00000090831	0.954379722	1.44E-06
TUCP_000619	XLOC_022408	ENSRNOT00000073559	0.955798144	1.23E-06
TUCP_000619	XLOC_022408	ENSRNOT00000081246	0.974459917	8.20E-08
TUCP_000619	XLOC_022408	ENSRNOT00000080768	0.95697946	1.08E-06
TUCP_000619	XLOC_022408	ENSRNOT00000072241	0.952258902	1.80E-06
TUCP_000619	XLOC_022408	ENSRNOT00000007351	-0.958869257	8.65E-07
TUCP_000619	XLOC_022408	ENSRNOT00000021851	0.95959849	7.92E-07
TUCP_000619	XLOC_022408	ENSRNOT00000023989	0.953835567	1.53E-06
TUCP_000619	XLOC_022408	ENSRNOT00000035346	-0.960360227	7.21E-07
TUCP_000619	XLOC_022408	ENSRNOT00000021614	0.95624672	1.17E-06
TUCP_000619	XLOC_022408	ENSRNOT00000003991	0.95662781	1.12E-06
TUCP_000619	XLOC_022408	ENSRNOT00000004032	0.962305486	5.63E-07
TUCP_000619	XLOC_022408	ENSRNOT00000063956	0.964295771	4.30E-07
TUCP_000619	XLOC_022408	ENSRNOT00000076763	0.976124685	5.87E-08
TUCP_000619	XLOC_022408	ENSRNOT00000043216	0.95371445	1.55E-06
TUCP_000619	XLOC_022408	ENSRNOT00000027411	0.957658389	9.98E-07
TUCP_000619	XLOC_022408	ENSRNOT00000073526	0.965430543	3.67E-07
TUCP_000619	XLOC_022408	ENSRNOT00000066526	0.953339431	1.61E-06
TUCP_000619	XLOC_022408	ENSRNOT00000012048	0.979005789	3.10E-08
TUCP_000619	XLOC_022408	ENSRNOT00000027194	0.951298725	1.99E-06
TUCP_000619	XLOC_022408	ENSRNOT00000045882	0.953301083	1.62E-06
TUCP_000619	XLOC_022408	ENSRNOT00000058072	0.970711356	1.62E-07
TUCP_000619	XLOC_022408	ENSRNOT00000075558	0.960146814	7.40E-07
TUCP_000619	XLOC_022408	ENSRNOT00000029866	0.981946373	1.47E-08
TUCP_000619	XLOC_022408	ENSRNOT00000019923	0.969753518	1.89E-07
TUCP_000619	XLOC_022408	ENSRNOT00000029439	0.972535878	1.18E-07
TUCP_000619	XLOC_022408	ENSRNOT00000045285	0.973436203	9.96E-08
TUCP_000619	XLOC_022408	ENSRNOT00000028759	0.954310856	1.45E-06
TUCP_000619	XLOC_022408	ENSRNOT00000021650	0.952516529	1.76E-06
TUCP_000619	XLOC_022408	ENSRNOT00000072592	0.950251306	2.21E-06
TUCP_000619	XLOC_022408	ENSRNOT00000076453	0.955908842	1.22E-06
TUCP_000619	XLOC_022408	ENSRNOT00000000157	0.962564927	5.44E-07
TUCP_000618	XLOC_022357	ENSRNOT00000043917	0.958830566	8.69E-07
TUCP_000618	XLOC_022357	ENSRNOT00000092974	0.961878929	5.95E-07
TUCP_000618	XLOC_022357	ENSRNOT00000021320	0.972922392	1.10E-07
TUCP_002080	XLOC_085316	ENSRNOT00000013913	0.951133143	2.02E-06
TUCP_002080	XLOC_085316	ENSRNOT00000018543	0.951449387	1.96E-06
TUCP_002080	XLOC_085316	ENSRNOT00000083255	0.960207397	7.35E-07
TUCP_002080	XLOC_085316	ENSRNOT00000089783	0.964653474	4.09E-07
TUCP_002080	XLOC_085316	ENSRNOT00000090925	0.967343738	2.77E-07
TUCP_002080	XLOC_085316	ENSRNOT00000054980	0.96452988	4.17E-07
TUCP_002080	XLOC_085316	ENSRNOT00000005934	0.95557881	1.26E-06
TUCP_002080	XLOC_085316	ENSRNOT00000066028	0.959539789	7.98E-07
TUCP_002080	XLOC_085316	ENSRNOT00000074659	0.953422048	1.60E-06
TUCP_002080	XLOC_085316	ENSRNOT00000024601	0.960797977	6.83E-07
TUCP_002080	XLOC_085316	ENSRNOT00000009328	0.964928205	3.94E-07
TUCP_002080	XLOC_085316	ENSRNOT00000087625	0.95505484	1.34E-06

TUCP_002080	XLOC_085316	ENSRNOT00000024771	0.958711998	8.82E-07
TUCP_002080	XLOC_085316	ENSRNOT00000049474	0.972785255	1.12E-07
TUCP_002080	XLOC_085316	ENSRNOT00000023710	0.954677254	1.40E-06
TUCP_002080	XLOC_085316	ENSRNOT00000028185	0.954467664	1.43E-06
TUCP_002080	XLOC_085316	ENSRNOT00000068037	0.956472856	1.14E-06
TUCP_002080	XLOC_085316	ENSRNOT00000016490	0.950262907	2.20E-06
TUCP_002080	XLOC_085316	ENSRNOT00000080654	0.969398441	2.01E-07
TUCP_002080	XLOC_085316	ENSRNOT00000009497	0.960765158	6.85E-07
TUCP_002080	XLOC_085316	ENSRNOT00000004003	0.952182774	1.82E-06
TUCP_002080	XLOC_085316	ENSRNOT00000058160	0.960370631	7.20E-07
TUCP_002080	XLOC_085316	ENSRNOT00000043554	0.950361916	2.18E-06
TUCP_002080	XLOC_085316	ENSRNOT00000079483	0.96313325	5.04E-07
TUCP_002080	XLOC_085316	ENSRNOT00000000713	0.951971912	1.86E-06
TUCP_002080	XLOC_085316	ENSRNOT00000070812	0.95265377	1.73E-06
TUCP_002080	XLOC_085316	ENSRNOT00000022862	0.955723999	1.24E-06
TUCP_002080	XLOC_085316	ENSRNOT00000002289	0.958465166	9.08E-07
TUCP_002080	XLOC_085316	ENSRNOT00000032158	0.951339972	1.98E-06
TUCP_002080	XLOC_085316	ENSRNOT00000028927	0.959243317	8.27E-07
TUCP_002080	XLOC_085316	ENSRNOT00000050364	0.959277139	8.24E-07
TUCP_002080	XLOC_085316	ENSRNOT00000027809	0.953700401	1.55E-06
TUCP_002080	XLOC_085316	ENSRNOT00000027684	0.961776359	6.03E-07
TUCP_002080	XLOC_085316	ENSRNOT00000046251	0.957216831	1.05E-06
TUCP_002080	XLOC_085316	ENSRNOT00000087697	0.951795003	1.89E-06
TUCP_002080	XLOC_085316	ENSRNOT00000008350	0.956818831	1.10E-06
TUCP_002080	XLOC_085316	ENSRNOT00000001242	0.950951995	2.06E-06
TUCP_002080	XLOC_085316	ENSRNOT00000008573	0.956933803	1.09E-06
TUCP_002081	XLOC_085316	ENSRNOT00000091609	0.96302074	5.12E-07
TUCP_002081	XLOC_085316	ENSRNOT00000071251	0.951834846	1.88E-06
TUCP_002081	XLOC_085316	ENSRNOT00000002907	0.966467346	3.16E-07
TUCP_002365	XLOC_099421	ENSRNOT00000021833	-0.970895759	1.57E-07
TUCP_002365	XLOC_099421	ENSRNOT00000009817	0.951190382	2.01E-06
TUCP_002365	XLOC_099421	ENSRNOT00000026396	0.959875212	7.66E-07
TUCP_002365	XLOC_099421	ENSRNOT00000027234	-0.950686373	2.11E-06
TUCP_002365	XLOC_099421	ENSRNOT00000002222	-0.951166034	2.01E-06
TUCP_002365	XLOC_099421	ENSRNOT00000071540	-0.953740619	1.54E-06
TUCP_002365	XLOC_099421	ENSRNOT00000031458	-0.958228123	9.34E-07
TUCP_002365	XLOC_099421	ENSRNOT00000071737	0.95688688	1.09E-06
TUCP_002365	XLOC_099421	ENSRNOT00000078349	0.950273242	2.20E-06
TUCP_002365	XLOC_099421	ENSRNOT00000045545	0.953561871	1.57E-06
TUCP_002365	XLOC_099421	ENSRNOT00000064902	-0.950813805	2.09E-06
TUCP_002365	XLOC_099421	ENSRNOT00000023807	0.951481858	1.95E-06
TUCP_002365	XLOC_099421	ENSRNOT00000067135	0.953932164	1.51E-06
TUCP_002365	XLOC_099421	ENSRNOT00000071853	0.957704363	9.93E-07
TUCP_002365	XLOC_099421	ENSRNOT00000074985	0.954134038	1.48E-06
TUCP_002365	XLOC_099421	ENSRNOT00000065983	-0.952113187	1.83E-06
TUCP_002365	XLOC_099421	ENSRNOT00000067901	0.95078989	2.09E-06
TUCP_002365	XLOC_099421	ENSRNOT00000067840	-0.953923585	1.51E-06
TUCP_002365	XLOC_099421	ENSRNOT00000009225	0.955129879	1.33E-06
TUCP_002365	XLOC_099421	ENSRNOT00000022924	0.950633949	2.12E-06
TUCP_002365	XLOC_099421	ENSRNOT00000079895	-0.952077648	1.84E-06
TUCP_002365	XLOC_099421	ENSRNOT00000009634	0.969988515	1.82E-07

TUCP_002362	XLOC_099421	ENSRNOT00000066250	-0.955808813	1.23E-06
TUCP_000613	XLOC_022053	ENSRNOT00000027644	0.958631026	8.90E-07
TUCP_000613	XLOC_022053	ENSRNOT00000016083	0.950746458	2.10E-06
TUCP_000613	XLOC_022053	ENSRNOT00000067375	0.971466718	1.42E-07
TUCP_000613	XLOC_022053	ENSRNOT00000049349	-0.974731347	7.78E-08
TUCP_000613	XLOC_022053	ENSRNOT00000089313	0.968548997	2.30E-07
TUCP_000613	XLOC_022053	ENSRNOT00000078737	0.953489604	1.59E-06
TUCP_000613	XLOC_022053	ENSRNOT00000078326	0.956302331	1.17E-06
TUCP_000613	XLOC_022053	ENSRNOT00000007851	0.969778104	1.89E-07
TUCP_000613	XLOC_022053	ENSRNOT00000014063	0.963659267	4.70E-07
TUCP_000613	XLOC_022053	ENSRNOT00000004159	0.953277203	1.62E-06
TUCP_000613	XLOC_022053	ENSRNOT00000015962	0.967938921	2.53E-07
TUCP_000613	XLOC_022053	ENSRNOT00000014384	0.965666109	3.55E-07
TUCP_000613	XLOC_022053	ENSRNOT00000042459	0.971265583	1.47E-07
TUCP_000613	XLOC_022053	ENSRNOT00000055279	0.950901269	2.07E-06
TUCP_000613	XLOC_022053	ENSRNOT00000037203	0.950445536	2.16E-06
TUCP_000613	XLOC_022053	ENSRNOT00000087386	0.963291609	4.94E-07
TUCP_000613	XLOC_022053	ENSRNOT00000057562	-0.951600202	1.93E-06
TUCP_000613	XLOC_022053	ENSRNOT00000018622	0.968277095	2.40E-07
TUCP_000613	XLOC_022053	ENSRNOT00000087075	0.958308586	9.25E-07
TUCP_000613	XLOC_022053	ENSRNOT00000000617	0.957105519	1.06E-06
TUCP_000613	XLOC_022053	ENSRNOT00000022126	0.954817691	1.37E-06
TUCP_000613	XLOC_022053	ENSRNOT00000086559	0.9782329	3.71E-08
TUCP_000613	XLOC_022053	ENSRNOT00000008274	0.969751976	1.90E-07
TUCP_000613	XLOC_022053	ENSRNOT00000045041	0.969596534	1.94E-07
TUCP_000613	XLOC_022053	ENSRNOT00000007588	0.970477395	1.68E-07
TUCP_000613	XLOC_022053	ENSRNOT00000013571	0.955126926	1.33E-06
TUCP_000613	XLOC_022053	ENSRNOT00000067626	0.95989053	7.64E-07
TUCP_000613	XLOC_022053	ENSRNOT00000003797	0.951275072	1.99E-06
TUCP_000613	XLOC_022053	ENSRNOT00000056644	0.99057539	5.76E-10
TUCP_000613	XLOC_022053	ENSRNOT00000065275	0.95703722	1.07E-06
TUCP_000613	XLOC_022053	ENSRNOT00000017268	0.988563725	1.51E-09
TUCP_000613	XLOC_022053	ENSRNOT00000067391	0.958960225	8.56E-07
TUCP_000613	XLOC_022053	ENSRNOT00000091368	0.952651411	1.73E-06
TUCP_000613	XLOC_022053	ENSRNOT00000037333	0.960346602	7.22E-07
TUCP_000613	XLOC_022053	ENSRNOT00000029179	0.956642208	1.12E-06
TUCP_000613	XLOC_022053	ENSRNOT00000000469	-0.971355313	1.45E-07
TUCP_000613	XLOC_022053	ENSRNOT00000025506	0.955593446	1.26E-06
TUCP_000613	XLOC_022053	ENSRNOT00000007326	0.961406659	6.32E-07
TUCP_000613	XLOC_022053	ENSRNOT00000017813	0.950429876	2.17E-06
TUCP_000613	XLOC_022053	ENSRNOT00000000812	0.957429234	1.03E-06
TUCP_000613	XLOC_022053	ENSRNOT00000000814	0.951574552	1.93E-06
TUCP_000613	XLOC_022053	ENSRNOT00000072357	0.953662323	1.56E-06
TUCP_000613	XLOC_022053	ENSRNOT00000074515	0.970220712	1.75E-07
TUCP_000613	XLOC_022053	ENSRNOT00000082627	0.974281099	8.49E-08
TUCP_000613	XLOC_022053	ENSRNOT00000027145	0.965665088	3.55E-07
TUCP_000613	XLOC_022053	ENSRNOT00000072458	0.967921988	2.53E-07
TUCP_000613	XLOC_022053	ENSRNOT00000044449	0.971388294	1.44E-07
TUCP_000613	XLOC_022053	ENSRNOT00000022827	0.964877708	3.97E-07
TUCP_000613	XLOC_022053	ENSRNOT00000019531	0.961666795	6.11E-07
TUCP_000613	XLOC_022053	ENSRNOT00000065023	0.95620082	1.18E-06

TUCP_000613	XLOC_022053	ENSRNOT00000075444	0.952008058	1.85E-06
TUCP_000613	XLOC_022053	ENSRNOT00000020558	0.955444099	1.28E-06
TUCP_000613	XLOC_022053	ENSRNOT00000027604	0.958768744	8.76E-07
TUCP_000613	XLOC_022053	ENSRNOT00000075598	0.960730005	6.89E-07
TUCP_000613	XLOC_022053	ENSRNOT00000009327	0.952447255	1.77E-06
TUCP_000613	XLOC_022053	ENSRNOT00000017883	-0.954148692	1.48E-06
TUCP_000613	XLOC_022053	ENSRNOT00000055607	0.961883888	5.94E-07
TUCP_000613	XLOC_022053	ENSRNOT00000082149	0.951145352	2.02E-06
TUCP_000613	XLOC_022053	ENSRNOT00000017889	0.964257561	4.33E-07
TUCP_000613	XLOC_022053	ENSRNOT00000016324	0.961347413	6.37E-07
TUCP_000613	XLOC_022053	ENSRNOT00000001631	0.987442694	2.41E-09
TUCP_000613	XLOC_022053	ENSRNOT00000061201	0.96751572	2.70E-07
TUCP_000613	XLOC_022053	ENSRNOT00000027512	0.962240543	5.67E-07
TUCP_000613	XLOC_022053	ENSRNOT00000001639	0.964975961	3.91E-07
TUCP_000613	XLOC_022053	ENSRNOT00000022170	0.955103721	1.33E-06
TUCP_000613	XLOC_022053	ENSRNOT00000035277	-0.950242779	2.21E-06
TUCP_000613	XLOC_022053	ENSRNOT00000018877	0.983750898	8.68E-09
TUCP_000613	XLOC_022053	ENSRNOT00000022343	0.954884311	1.36E-06
TUCP_000613	XLOC_022053	ENSRNOT00000044847	0.965751944	3.50E-07
TUCP_000613	XLOC_022053	ENSRNOT00000085915	0.955304942	1.30E-06
TUCP_000613	XLOC_022053	ENSRNOT00000015831	0.968437999	2.34E-07
TUCP_000613	XLOC_022053	ENSRNOT00000006395	0.951572754	1.93E-06
TUCP_000613	XLOC_022053	ENSRNOT00000030823	0.983385543	9.70E-09
TUCP_000613	XLOC_022053	ENSRNOT00000001227	0.955600759	1.26E-06
TUCP_000613	XLOC_022053	ENSRNOT00000068435	0.955476806	1.28E-06
TUCP_000613	XLOC_022053	ENSRNOT00000012432	0.968687858	2.25E-07
TUCP_000613	XLOC_022053	ENSRNOT00000076388	0.979343741	2.86E-08
TUCP_000613	XLOC_022053	ENSRNOT00000008317	0.959865502	7.67E-07
TUCP_000613	XLOC_022053	ENSRNOT00000087033	0.953733364	1.54E-06
TUCP_000613	XLOC_022053	ENSRNOT00000082963	0.976142146	5.85E-08
TUCP_000613	XLOC_022053	ENSRNOT00000072632	0.96365478	4.70E-07
TUCP_000613	XLOC_022053	ENSRNOT00000016856	0.960082595	7.46E-07
TUCP_000613	XLOC_022053	ENSRNOT00000013936	0.952068139	1.84E-06
TUCP_000613	XLOC_022053	ENSRNOT00000079085	0.975191195	7.10E-08
TUCP_000613	XLOC_022053	ENSRNOT00000068666	0.950895453	2.07E-06
TUCP_000613	XLOC_022053	ENSRNOT00000060690	0.952748687	1.71E-06
TUCP_000613	XLOC_022053	ENSRNOT00000038811	0.963040196	5.10E-07
TUCP_000613	XLOC_022053	ENSRNOT00000008919	0.956142537	1.19E-06
TUCP_000613	XLOC_022053	ENSRNOT00000000618	0.950253365	2.21E-06
TUCP_000613	XLOC_022053	ENSRNOT00000007917	0.98676923	3.12E-09
TUCP_000613	XLOC_022053	ENSRNOT00000034386	0.960531099	7.06E-07
TUCP_000613	XLOC_022053	ENSRNOT00000019778	0.95213645	1.83E-06
TUCP_000613	XLOC_022053	ENSRNOT00000018684	0.966340179	3.22E-07
TUCP_000613	XLOC_022053	ENSRNOT00000048022	0.967543968	2.69E-07
TUCP_000613	XLOC_022053	ENSRNOT00000024707	0.953384005	1.60E-06
TUCP_000613	XLOC_022053	ENSRNOT00000001597	0.96550081	3.63E-07
TUCP_000613	XLOC_022053	ENSRNOT00000009468	0.970177061	1.77E-07
TUCP_000613	XLOC_022053	ENSRNOT00000072069	0.963558015	4.76E-07
TUCP_000613	XLOC_022053	ENSRNOT00000075400	0.966714531	3.04E-07
TUCP_000613	XLOC_022053	ENSRNOT00000009080	0.9622929	5.63E-07
TUCP_000613	XLOC_022053	ENSRNOT00000023320	0.980635512	2.08E-08

TUCP_000613	XLOC_022053	ENSRNOT00000020453	0.966442276	3.17E-07
TUCP_000613	XLOC_022053	ENSRNOT00000075494	0.96923354	2.06E-07
TUCP_000613	XLOC_022053	ENSRNOT00000009564	0.969889002	1.85E-07
TUCP_000613	XLOC_022053	ENSRNOT00000041237	0.957664828	9.97E-07
TUCP_000613	XLOC_022053	ENSRNOT00000023430	0.975058718	7.29E-08
TUCP_000613	XLOC_022053	ENSRNOT00000001859	0.978868961	3.20E-08
TUCP_000613	XLOC_022053	ENSRNOT00000003733	0.952605404	1.74E-06
TUCP_000613	XLOC_022053	ENSRNOT00000021114	-0.955248771	1.31E-06
TUCP_000613	XLOC_022053	ENSRNOT00000021110	0.965672806	3.54E-07
TUCP_000613	XLOC_022053	ENSRNOT00000083864	0.969139478	2.09E-07
TUCP_000613	XLOC_022053	ENSRNOT00000020853	0.980769204	2.01E-08
TUCP_000613	XLOC_022053	ENSRNOT00000001774	0.97168261	1.37E-07
TUCP_000613	XLOC_022053	ENSRNOT00000080013	0.978890069	3.19E-08
TUCP_000613	XLOC_022053	ENSRNOT00000013759	0.976282438	5.68E-08
TUCP_000613	XLOC_022053	ENSRNOT00000025683	0.962041718	5.82E-07
TUCP_000613	XLOC_022053	ENSRNOT00000013613	0.964500394	4.18E-07
TUCP_000613	XLOC_022053	ENSRNOT00000019056	0.96028463	7.28E-07
TUCP_000613	XLOC_022053	ENSRNOT00000078884	0.956115263	1.19E-06
TUCP_000613	XLOC_022053	ENSRNOT00000027553	0.98197214	1.46E-08
TUCP_000613	XLOC_022053	ENSRNOT00000033312	0.952555003	1.75E-06
TUCP_000613	XLOC_022053	ENSRNOT00000015473	0.958470965	9.07E-07
TUCP_000613	XLOC_022053	ENSRNOT00000015476	0.954118209	1.48E-06
TUCP_000613	XLOC_022053	ENSRNOT00000021621	0.951369248	1.97E-06
TUCP_000613	XLOC_022053	ENSRNOT00000077063	0.969880256	1.86E-07
TUCP_000613	XLOC_022053	ENSRNOT00000011432	0.964892873	3.96E-07
TUCP_000613	XLOC_022053	ENSRNOT00000014177	0.981250159	1.77E-08
TUCP_000613	XLOC_022053	ENSRNOT00000022432	0.952403968	1.78E-06
TUCP_000613	XLOC_022053	ENSRNOT00000034983	0.975073422	7.27E-08
TUCP_000613	XLOC_022053	ENSRNOT00000020707	0.971624954	1.38E-07
TUCP_000613	XLOC_022053	ENSRNOT00000021937	0.956104452	1.19E-06
TUCP_000613	XLOC_022053	ENSRNOT00000052098	0.967915159	2.54E-07
TUCP_000613	XLOC_022053	ENSRNOT00000018634	0.955178473	1.32E-06
TUCP_000613	XLOC_022053	ENSRNOT00000014211	0.955500607	1.28E-06
TUCP_000613	XLOC_022053	ENSRNOT00000008140	0.963003238	5.13E-07
TUCP_000613	XLOC_022053	ENSRNOT00000072841	0.952739284	1.71E-06
TUCP_000613	XLOC_022053	ENSRNOT00000085545	0.966737117	3.03E-07
TUCP_000613	XLOC_022053	ENSRNOT00000015351	0.978053356	3.86E-08
TUCP_000613	XLOC_022053	ENSRNOT00000073466	-0.95608906	1.19E-06
TUCP_000613	XLOC_022053	ENSRNOT00000004351	0.959965201	7.57E-07
TUCP_000613	XLOC_022053	ENSRNOT00000014502	0.979260705	2.92E-08
TUCP_000613	XLOC_022053	ENSRNOT00000003787	0.960318438	7.25E-07
TUCP_000613	XLOC_022053	ENSRNOT00000005608	0.951794202	1.89E-06
TUCP_000613	XLOC_022053	ENSRNOT00000025104	0.970958049	1.55E-07
TUCP_000613	XLOC_022053	ENSRNOT00000072165	0.9656596	3.55E-07
TUCP_000613	XLOC_022053	ENSRNOT00000015759	0.980987031	1.90E-08
TUCP_000613	XLOC_022053	ENSRNOT00000091541	0.986660658	3.25E-09
TUCP_000613	XLOC_022053	ENSRNOT00000050774	0.957349915	1.03E-06
TUCP_000613	XLOC_022053	ENSRNOT00000008725	0.959608987	7.91E-07
TUCP_000613	XLOC_022053	ENSRNOT00000068026	0.964853336	3.98E-07
TUCP_000613	XLOC_022053	ENSRNOT00000056923	0.968139964	2.45E-07
TUCP_000613	XLOC_022053	ENSRNOT00000005996	0.959648556	7.87E-07

TUCP_000613	XLOC_022053	ENSRNOT00000008889	0.966525724	3.13E-07
TUCP_000613	XLOC_022053	ENSRNOT00000075057	0.975207015	7.08E-08
TUCP_000613	XLOC_022053	ENSRNOT00000017746	0.975919941	6.12E-08
TUCP_000613	XLOC_022053	ENSRNOT00000016286	0.959614375	7.91E-07
TUCP_000613	XLOC_022053	ENSRNOT00000037946	0.973919704	9.10E-08
TUCP_000613	XLOC_022053	ENSRNOT00000070825	0.95060466	2.13E-06
TUCP_000613	XLOC_022053	ENSRNOT00000066923	0.95274327	1.71E-06
TUCP_000613	XLOC_022053	ENSRNOT00000038677	0.97382753	9.26E-08
TUCP_000613	XLOC_022053	ENSRNOT00000024753	0.963558128	4.76E-07
TUCP_000613	XLOC_022053	ENSRNOT00000070916	0.972268672	1.23E-07
TUCP_000613	XLOC_022053	ENSRNOT00000054970	0.957226085	1.05E-06
TUCP_000613	XLOC_022053	ENSRNOT00000024452	0.968442741	2.34E-07
TUCP_000613	XLOC_022053	ENSRNOT00000000253	0.956054738	1.20E-06
TUCP_000613	XLOC_022053	ENSRNOT00000000258	0.956139572	1.19E-06
TUCP_000613	XLOC_022053	ENSRNOT00000067524	-0.950038845	2.25E-06
TUCP_000613	XLOC_022053	ENSRNOT00000002511	0.97486483	7.57E-08
TUCP_000613	XLOC_022053	ENSRNOT00000023211	0.956741725	1.11E-06
TUCP_000613	XLOC_022053	ENSRNOT00000016376	0.959159119	8.35E-07
TUCP_000613	XLOC_022053	ENSRNOT00000081593	0.956048113	1.20E-06
TUCP_000613	XLOC_022053	ENSRNOT00000047571	0.956837269	1.10E-06
TUCP_000613	XLOC_022053	ENSRNOT00000025036	0.959807365	7.72E-07
TUCP_000613	XLOC_022053	ENSRNOT00000020809	0.965897809	3.43E-07
TUCP_000613	XLOC_022053	ENSRNOT00000001605	0.976980181	4.90E-08
TUCP_000613	XLOC_022053	ENSRNOT00000075630	0.971789996	1.34E-07
TUCP_000613	XLOC_022053	ENSRNOT00000021677	0.975295896	6.95E-08
TUCP_000613	XLOC_022053	ENSRNOT00000019044	0.966625969	3.08E-07
TUCP_000613	XLOC_022053	ENSRNOT00000020982	0.959193922	8.32E-07
TUCP_000613	XLOC_022053	ENSRNOT00000086847	0.956234701	1.17E-06
TUCP_000613	XLOC_022053	ENSRNOT00000018846	0.976259094	5.71E-08
TUCP_000613	XLOC_022053	ENSRNOT00000012847	0.964402657	4.24E-07
TUCP_000613	XLOC_022053	ENSRNOT00000028876	0.954457332	1.43E-06
TUCP_000613	XLOC_022053	ENSRNOT00000014002	0.969336329	2.03E-07
TUCP_000613	XLOC_022053	ENSRNOT00000011694	0.963595185	4.74E-07
TUCP_000613	XLOC_022053	ENSRNOT00000002851	0.950520957	2.15E-06
TUCP_000613	XLOC_022053	ENSRNOT00000055250	0.962782103	5.28E-07
TUCP_000613	XLOC_022053	ENSRNOT00000068184	0.969973458	1.83E-07
TUCP_000613	XLOC_022053	ENSRNOT00000031692	0.981073792	1.85E-08
TUCP_000613	XLOC_022053	ENSRNOT00000089016	0.981486908	1.66E-08
TUCP_000613	XLOC_022053	ENSRNOT00000000737	0.965166062	3.81E-07
TUCP_000613	XLOC_022053	ENSRNOT00000022864	0.950077175	2.25E-06
TUCP_000613	XLOC_022053	ENSRNOT00000006578	0.965230456	3.77E-07
TUCP_000613	XLOC_022053	ENSRNOT00000028900	0.965308455	3.73E-07
TUCP_000613	XLOC_022053	ENSRNOT00000072722	0.955854717	1.23E-06
TUCP_000613	XLOC_022053	ENSRNOT00000087556	0.974857837	7.59E-08
TUCP_000613	XLOC_022053	ENSRNOT00000029753	0.95314804	1.64E-06
TUCP_000613	XLOC_022053	ENSRNOT00000040981	0.967840165	2.57E-07
TUCP_000613	XLOC_022053	ENSRNOT00000056725	0.950120398	2.24E-06
TUCP_000613	XLOC_022053	ENSRNOT00000038464	0.95905608	8.46E-07
TUCP_000613	XLOC_022053	ENSRNOT00000008359	0.970294813	1.73E-07
TUCP_000613	XLOC_022053	ENSRNOT00000092641	0.974403914	8.29E-08
TUCP_000613	XLOC_022053	ENSRNOT00000035486	0.975479355	6.70E-08

TUCP_000613	XLOC_022053	ENSRNOT00000080174	0.96765421	2.64E-07
TUCP_000613	XLOC_022053	ENSRNOT00000011237	0.955690962	1.25E-06
TUCP_000613	XLOC_022053	ENSRNOT00000013344	0.957861276	9.75E-07
TUCP_000613	XLOC_022053	ENSRNOT00000030501	0.96136634	6.35E-07
TUCP_000613	XLOC_022053	ENSRNOT00000017204	0.982632131	1.21E-08
TUCP_000613	XLOC_022053	ENSRNOT00000008772	0.963068332	5.09E-07
TUCP_000613	XLOC_022053	ENSRNOT00000005658	0.951745074	1.90E-06
TUCP_000613	XLOC_022053	ENSRNOT00000009275	0.958497652	9.04E-07
TUCP_000613	XLOC_022053	ENSRNOT00000022086	0.972147032	1.26E-07
TUCP_000613	XLOC_022053	ENSRNOT00000008074	0.950722981	2.11E-06
TUCP_000613	XLOC_022053	ENSRNOT00000024714	0.95645098	1.15E-06
TUCP_000613	XLOC_022053	ENSRNOT00000018341	0.975087016	7.25E-08
TUCP_000613	XLOC_022053	ENSRNOT00000081787	0.96212075	5.76E-07
TUCP_000613	XLOC_022053	ENSRNOT00000070886	0.979576195	2.70E-08
TUCP_000613	XLOC_022053	ENSRNOT00000002799	0.966308337	3.23E-07
TUCP_000613	XLOC_022053	ENSRNOT00000024882	0.97254337	1.17E-07
TUCP_000613	XLOC_022053	ENSRNOT00000023456	0.960309743	7.26E-07
TUCP_000613	XLOC_022053	ENSRNOT00000016012	0.967400845	2.74E-07
TUCP_000613	XLOC_022053	ENSRNOT00000002334	0.954975536	1.35E-06
TUCP_000613	XLOC_022053	ENSRNOT00000023037	-0.955177546	1.32E-06
TUCP_000613	XLOC_022053	ENSRNOT00000090232	0.955911094	1.22E-06
TUCP_000613	XLOC_022053	ENSRNOT00000013587	0.958305494	9.25E-07
TUCP_000613	XLOC_022053	ENSRNOT00000003728	0.962036116	5.83E-07
TUCP_000613	XLOC_022053	ENSRNOT00000001891	0.977201607	4.67E-08
TUCP_000613	XLOC_022053	ENSRNOT00000039709	0.953130227	1.65E-06
TUCP_000613	XLOC_022053	ENSRNOT00000019040	0.963807052	4.60E-07
TUCP_000613	XLOC_022053	ENSRNOT00000035400	0.979733641	2.60E-08
TUCP_000613	XLOC_022053	ENSRNOT00000006607	0.96757163	2.67E-07
TUCP_000613	XLOC_022053	ENSRNOT00000073005	0.951245231	2.00E-06
TUCP_000613	XLOC_022053	ENSRNOT00000077727	0.956719453	1.11E-06
TUCP_000613	XLOC_022053	ENSRNOT00000082124	0.975524212	6.64E-08
TUCP_000613	XLOC_022053	ENSRNOT00000000203	0.967168437	2.84E-07
TUCP_000613	XLOC_022053	ENSRNOT00000014044	0.962417585	5.54E-07
TUCP_000613	XLOC_022053	ENSRNOT00000090156	0.955451666	1.28E-06
TUCP_000613	XLOC_022053	ENSRNOT00000086147	0.983940378	8.19E-09
TUCP_000613	XLOC_022053	ENSRNOT00000031653	0.951138328	2.02E-06
TUCP_000613	XLOC_022053	ENSRNOT00000004196	-0.958005401	9.58E-07
TUCP_000613	XLOC_022053	ENSRNOT00000007882	0.97680274	5.09E-08
TUCP_000613	XLOC_022053	ENSRNOT00000006946	0.986828165	3.05E-09
TUCP_000613	XLOC_022053	ENSRNOT00000086576	0.976906445	4.98E-08
TUCP_000613	XLOC_022053	ENSRNOT00000022451	0.952472074	1.76E-06
TUCP_000613	XLOC_022053	ENSRNOT00000039012	0.959273686	8.24E-07
TUCP_000613	XLOC_022053	ENSRNOT00000023854	0.958518191	9.02E-07
TUCP_000613	XLOC_022053	ENSRNOT00000001160	0.957571334	1.01E-06
TUCP_000613	XLOC_022053	ENSRNOT00000082894	0.960095671	7.45E-07
TUCP_000613	XLOC_022053	ENSRNOT00000056918	0.951091899	2.03E-06
TUCP_000613	XLOC_022053	ENSRNOT00000079801	0.951372309	1.97E-06
TUCP_000613	XLOC_022053	ENSRNOT00000018153	0.953500292	1.58E-06
TUCP_000613	XLOC_022053	ENSRNOT00000083983	0.991214378	4.06E-10
TUCP_000613	XLOC_022053	ENSRNOT00000007549	0.989630197	9.28E-10
TUCP_000613	XLOC_022053	ENSRNOT00000017626	0.958898764	8.62E-07

TUCP_000613	XLOC_022053	ENSRNOT00000076485	0.955126609	1.33E-06
TUCP_000613	XLOC_022053	ENSRNOT00000022774	0.967955876	2.52E-07
TUCP_000613	XLOC_022053	ENSRNOT00000025134	0.97337379	1.01E-07
TUCP_000613	XLOC_022053	ENSRNOT00000087359	-0.963247475	4.96E-07
TUCP_000613	XLOC_022053	ENSRNOT00000008630	-0.955019414	1.34E-06
TUCP_000613	XLOC_022053	ENSRNOT00000029704	0.966606789	3.09E-07
TUCP_000613	XLOC_022053	ENSRNOT00000029115	0.952063889	1.84E-06
TUCP_000613	XLOC_022053	ENSRNOT00000015139	0.961139581	6.54E-07
TUCP_000613	XLOC_022053	ENSRNOT00000014832	0.971618514	1.38E-07
TUCP_000613	XLOC_022053	ENSRNOT00000089879	0.965512243	3.63E-07
TUCP_000613	XLOC_022053	ENSRNOT00000015130	0.95858664	8.95E-07
TUCP_000613	XLOC_022053	ENSRNOT00000018473	0.954585306	1.41E-06
TUCP_000613	XLOC_022053	ENSRNOT00000026118	0.954143078	1.48E-06
TUCP_000613	XLOC_022053	ENSRNOT00000007276	0.955509894	1.27E-06
TUCP_000613	XLOC_022053	ENSRNOT00000017339	0.984241013	7.46E-09
TUCP_000613	XLOC_022053	ENSRNOT00000088752	0.976969792	4.91E-08
TUCP_000613	XLOC_022053	ENSRNOT00000056862	0.980121075	2.36E-08
TUCP_000613	XLOC_022053	ENSRNOT00000050227	0.980003371	2.44E-08
TUCP_000613	XLOC_022053	ENSRNOT00000093552	0.953578455	1.57E-06
TUCP_000613	XLOC_022053	ENSRNOT00000088884	0.952182358	1.82E-06
TUCP_000613	XLOC_022053	ENSRNOT00000075544	0.957194585	1.05E-06
TUCP_000613	XLOC_022053	ENSRNOT00000077363	0.963504923	4.80E-07
TUCP_000613	XLOC_022053	ENSRNOT00000038994	0.961175943	6.51E-07
TUCP_000613	XLOC_022053	ENSRNOT00000024841	0.95412085	1.48E-06
TUCP_000613	XLOC_022053	ENSRNOT00000009345	0.968885405	2.18E-07
TUCP_000613	XLOC_022053	ENSRNOT00000024924	0.982071286	1.42E-08
TUCP_000613	XLOC_022053	ENSRNOT00000048529	0.965151917	3.82E-07
TUCP_000613	XLOC_022053	ENSRNOT00000000124	0.964706348	4.06E-07
TUCP_000613	XLOC_022053	ENSRNOT00000043581	0.96078178	6.84E-07
TUCP_000613	XLOC_022053	ENSRNOT00000010419	-0.957864226	9.74E-07
TUCP_000613	XLOC_022053	ENSRNOT00000064636	0.959449182	8.07E-07
TUCP_000613	XLOC_022053	ENSRNOT00000066561	0.961201425	6.49E-07
TUCP_000613	XLOC_022053	ENSRNOT00000004094	0.962373722	5.58E-07
TUCP_000613	XLOC_022053	ENSRNOT00000020909	0.964157994	4.39E-07
TUCP_000613	XLOC_022053	ENSRNOT00000067905	0.957591361	1.01E-06
TUCP_000613	XLOC_022053	ENSRNOT00000011511	0.969881588	1.86E-07
TUCP_000613	XLOC_022053	ENSRNOT00000020900	0.955498416	1.28E-06
TUCP_000613	XLOC_022053	ENSRNOT00000012376	0.950478083	2.16E-06
TUCP_000613	XLOC_022053	ENSRNOT00000065642	0.982455659	1.27E-08
TUCP_000613	XLOC_022053	ENSRNOT00000036195	0.964274208	4.32E-07
TUCP_000613	XLOC_022053	ENSRNOT00000031291	0.958536626	9.00E-07
TUCP_000613	XLOC_022053	ENSRNOT00000074586	0.963841908	4.58E-07
TUCP_000613	XLOC_022053	ENSRNOT00000057758	0.960356815	7.21E-07
TUCP_000613	XLOC_022053	ENSRNOT00000059124	0.954155708	1.48E-06
TUCP_000613	XLOC_022053	ENSRNOT00000022637	0.964306328	4.30E-07
TUCP_000613	XLOC_022053	ENSRNOT00000083666	0.963899792	4.54E-07
TUCP_000613	XLOC_022053	ENSRNOT00000019358	0.959294822	8.22E-07
TUCP_000613	XLOC_022053	ENSRNOT00000010720	0.976866092	5.02E-08
TUCP_000613	XLOC_022053	ENSRNOT00000087011	0.973327264	1.02E-07
TUCP_000613	XLOC_022053	ENSRNOT00000019180	0.95118228	2.01E-06
TUCP_000613	XLOC_022053	ENSRNOT00000007028	0.953774835	1.54E-06

TUCP_000613	XLOC_022053	ENSRNOT00000005286	0.975885206	6.17E-08
TUCP_000613	XLOC_022053	ENSRNOT00000056675	0.95897707	8.54E-07
TUCP_000613	XLOC_022053	ENSRNOT00000013295	0.977449024	4.42E-08
TUCP_000613	XLOC_022053	ENSRNOT00000061735	0.96169941	6.09E-07
TUCP_000613	XLOC_022053	ENSRNOT00000021455	0.953598727	1.57E-06
TUCP_000613	XLOC_022053	ENSRNOT00000076191	0.971738451	1.35E-07
TUCP_000613	XLOC_022053	ENSRNOT00000017784	0.959561509	7.96E-07
TUCP_000613	XLOC_022053	ENSRNOT00000022676	0.950162022	2.23E-06
TUCP_000613	XLOC_022053	ENSRNOT00000021458	0.963606887	4.73E-07
TUCP_000613	XLOC_022053	ENSRNOT00000026496	0.979632627	2.67E-08
TUCP_000613	XLOC_022053	ENSRNOT00000082739	0.967008129	2.91E-07
TUCP_000613	XLOC_022053	ENSRNOT00000026552	0.950254042	2.21E-06
TUCP_000613	XLOC_022053	ENSRNOT00000012179	0.961744458	6.05E-07
TUCP_000613	XLOC_022053	ENSRNOT00000023463	0.978171755	3.76E-08
TUCP_000613	XLOC_022053	ENSRNOT00000034266	0.971009617	1.54E-07
TUCP_000613	XLOC_022053	ENSRNOT00000028781	-0.965812445	3.47E-07
TUCP_000613	XLOC_022053	ENSRNOT00000046490	0.979620371	2.68E-08
TUCP_000613	XLOC_022053	ENSRNOT00000001973	0.9614409	6.29E-07
TUCP_000613	XLOC_022053	ENSRNOT00000046552	-0.951609778	1.93E-06
TUCP_000613	XLOC_022053	ENSRNOT00000024775	0.957358801	1.03E-06
TUCP_000613	XLOC_022053	ENSRNOT00000024801	0.974259017	8.52E-08
TUCP_000613	XLOC_022053	ENSRNOT00000009544	0.965901327	3.43E-07
TUCP_000613	XLOC_022053	ENSRNOT00000054957	0.957623701	1.00E-06
TUCP_000613	XLOC_022053	ENSRNOT00000041808	0.96283241	5.25E-07
TUCP_000613	XLOC_022053	ENSRNOT00000021134	0.962780171	5.28E-07
TUCP_000613	XLOC_022053	ENSRNOT00000026724	0.979731616	2.60E-08
TUCP_000613	XLOC_022053	ENSRNOT00000001893	-0.953882911	1.52E-06
TUCP_000613	XLOC_022053	ENSRNOT00000024981	0.956440839	1.15E-06
TUCP_000613	XLOC_022053	ENSRNOT00000077784	0.965061681	3.87E-07
TUCP_000613	XLOC_022053	ENSRNOT00000081037	0.957719164	9.91E-07
TUCP_000613	XLOC_022053	ENSRNOT00000011898	0.95426783	1.46E-06
TUCP_000613	XLOC_022053	ENSRNOT00000020543	0.9639919	4.49E-07
TUCP_000613	XLOC_022053	ENSRNOT00000011952	0.95123291	2.00E-06
TUCP_000613	XLOC_022053	ENSRNOT00000013778	0.972353815	1.21E-07
TUCP_000613	XLOC_022053	ENSRNOT00000078185	0.950868203	2.08E-06
TUCP_000613	XLOC_022053	ENSRNOT00000077730	0.980527555	2.13E-08
TUCP_000613	XLOC_022053	ENSRNOT00000077040	0.96669306	3.05E-07
TUCP_000613	XLOC_022053	ENSRNOT00000018325	0.954201137	1.47E-06
TUCP_000613	XLOC_022053	ENSRNOT00000017955	0.964456966	4.21E-07
TUCP_000613	XLOC_022053	ENSRNOT00000089980	0.951655106	1.92E-06
TUCP_000613	XLOC_022053	ENSRNOT00000020316	0.964616307	4.12E-07
TUCP_000613	XLOC_022053	ENSRNOT00000077256	0.95766481	9.97E-07
TUCP_000613	XLOC_022053	ENSRNOT00000006709	0.978796316	3.26E-08
TUCP_000613	XLOC_022053	ENSRNOT00000014586	0.970082437	1.80E-07
TUCP_000613	XLOC_022053	ENSRNOT00000012495	0.971067235	1.52E-07
TUCP_000613	XLOC_022053	ENSRNOT00000060012	0.960792882	6.83E-07
TUCP_000613	XLOC_022053	ENSRNOT00000025221	0.979744249	2.60E-08
TUCP_000613	XLOC_022053	ENSRNOT00000025634	0.982146715	1.39E-08
TUCP_000613	XLOC_022053	ENSRNOT00000005693	0.955837014	1.23E-06
TUCP_000613	XLOC_022053	ENSRNOT00000066120	0.974132841	8.73E-08
TUCP_000613	XLOC_022053	ENSRNOT00000012428	0.959263245	8.25E-07

TUCP_000613	XLOC_022053	ENSRNOT00000000717	0.957736232	9.89E-07
TUCP_000613	XLOC_022053	ENSRNOT00000049048	0.97073754	1.61E-07
TUCP_000613	XLOC_022053	ENSRNOT00000030222	0.975118008	7.20E-08
TUCP_000613	XLOC_022053	ENSRNOT00000089066	-0.964753265	4.04E-07
TUCP_000613	XLOC_022053	ENSRNOT00000087342	0.960369631	7.20E-07
TUCP_000613	XLOC_022053	ENSRNOT00000035513	0.979470953	2.77E-08
TUCP_000613	XLOC_022053	ENSRNOT00000080216	0.963328832	4.91E-07
TUCP_000613	XLOC_022053	ENSRNOT00000005873	0.95048177	2.16E-06
TUCP_000613	XLOC_022053	ENSRNOT00000005155	-0.951444745	1.96E-06
TUCP_000613	XLOC_022053	ENSRNOT00000023543	0.962753443	5.30E-07
TUCP_000613	XLOC_022053	ENSRNOT00000017618	0.973379764	1.01E-07
TUCP_000613	XLOC_022053	ENSRNOT00000085458	0.965862173	3.45E-07
TUCP_000613	XLOC_022053	ENSRNOT00000038652	0.959982759	7.56E-07
TUCP_000613	XLOC_022053	ENSRNOT00000013535	0.984482205	6.90E-09
TUCP_000613	XLOC_022053	ENSRNOT00000066199	0.978471776	3.51E-08
TUCP_000613	XLOC_022053	ENSRNOT00000047698	0.955202067	1.32E-06
TUCP_000613	XLOC_022053	ENSRNOT00000024938	0.971289132	1.46E-07
TUCP_000613	XLOC_022053	ENSRNOT00000017683	0.956419491	1.15E-06
TUCP_000613	XLOC_022053	ENSRNOT00000018043	0.95339689	1.60E-06
TUCP_000613	XLOC_022053	ENSRNOT00000011320	0.960603057	7.00E-07
TUCP_002089	XLOC_085984	ENSRNOT00000055735	0.955004414	1.35E-06
TUCP_002089	XLOC_085984	ENSRNOT00000026860	0.952910021	1.68E-06
TUCP_002089	XLOC_085984	ENSRNOT00000066739	0.950318723	2.19E-06
TUCP_002089	XLOC_085984	ENSRNOT00000006186	0.956684321	1.12E-06
TUCP_002089	XLOC_085984	ENSRNOT00000026941	0.967529981	2.69E-07
TUCP_002089	XLOC_085984	ENSRNOT00000019213	0.973227227	1.04E-07
TUCP_002089	XLOC_085984	ENSRNOT00000007351	-0.953172848	1.64E-06
TUCP_002089	XLOC_085984	ENSRNOT00000074939	0.98711765	2.73E-09
TUCP_002089	XLOC_085984	ENSRNOT00000066041	-0.950627358	2.13E-06
TUCP_002089	XLOC_085984	ENSRNOT00000006055	0.984056525	7.90E-09
TUCP_002089	XLOC_085984	ENSRNOT00000071560	0.962870514	5.22E-07
TUCP_002089	XLOC_085984	ENSRNOT00000078445	-0.950752396	2.10E-06
TUCP_002089	XLOC_085984	ENSRNOT00000026065	0.95533992	1.30E-06
TUCP_002089	XLOC_085984	ENSRNOT00000023638	0.952344714	1.79E-06
TUCP_002089	XLOC_085984	ENSRNOT00000005228	0.9523723	1.78E-06
TUCP_002089	XLOC_085984	ENSRNOT00000088125	0.990172501	7.10E-10
TUCP_002089	XLOC_085984	ENSRNOT00000006175	0.957980651	9.61E-07
TUCP_002089	XLOC_085984	ENSRNOT00000005220	0.962285744	5.64E-07
TUCP_002089	XLOC_085984	ENSRNOT00000007164	0.954372602	1.44E-06
TUCP_002089	XLOC_085984	ENSRNOT00000036750	0.985048704	5.74E-09
TUCP_002089	XLOC_085984	ENSRNOT00000065947	0.956293712	1.17E-06
TUCP_002089	XLOC_085984	ENSRNOT00000021116	0.957295082	1.04E-06
TUCP_002089	XLOC_085984	ENSRNOT00000016649	0.95334154	1.61E-06
TUCP_002089	XLOC_085984	ENSRNOT00000082100	0.971585143	1.39E-07
TUCP_002089	XLOC_085984	ENSRNOT00000037699	0.953999524	1.50E-06
TUCP_002089	XLOC_085984	ENSRNOT00000040393	0.962831324	5.25E-07
TUCP_002089	XLOC_085984	ENSRNOT00000052063	0.95315974	1.64E-06
TUCP_002089	XLOC_085984	ENSRNOT00000018057	0.971104468	1.51E-07
TUCP_002089	XLOC_085984	ENSRNOT00000056813	0.981203163	1.79E-08
TUCP_002089	XLOC_085984	ENSRNOT00000043216	0.96067619	6.93E-07
TUCP_002089	XLOC_085984	ENSRNOT00000091128	-0.952104614	1.83E-06

TUCP_002089	XLOC_085984	ENSRNOT00000011384	0.956013612	1.20E-06
TUCP_002089	XLOC_085984	ENSRNOT00000026543	0.95381354	1.53E-06
TUCP_002089	XLOC_085984	ENSRNOT00000015076	0.978655452	3.37E-08
TUCP_002089	XLOC_085984	ENSRNOT00000044742	0.971602722	1.39E-07
TUCP_002089	XLOC_085984	ENSRNOT00000043345	0.972056739	1.28E-07
TUCP_002089	XLOC_085984	ENSRNOT00000010418	0.981840967	1.51E-08
TUCP_002089	XLOC_085984	ENSRNOT00000015073	0.966221126	3.27E-07
TUCP_002089	XLOC_085984	ENSRNOT00000043554	0.951533421	1.94E-06
TUCP_002089	XLOC_085984	ENSRNOT00000025689	0.955170734	1.32E-06
TUCP_002089	XLOC_085984	ENSRNOT00000012187	0.955273758	1.31E-06
TUCP_002089	XLOC_085984	ENSRNOT00000004698	0.977483394	4.39E-08
TUCP_002089	XLOC_085984	ENSRNOT00000064802	0.955610247	1.26E-06
TUCP_002089	XLOC_085984	ENSRNOT00000001507	0.969970019	1.83E-07
TUCP_002089	XLOC_085984	ENSRNOT00000026783	0.951996813	1.85E-06
TUCP_002089	XLOC_085984	ENSRNOT00000071573	0.955628654	1.26E-06
TUCP_002089	XLOC_085984	ENSRNOT00000005126	-0.968725864	2.24E-07
TUCP_002089	XLOC_085984	ENSRNOT00000012736	0.969676872	1.92E-07
TUCP_002089	XLOC_085984	ENSRNOT00000065300	0.96582728	3.47E-07
TUCP_002089	XLOC_085984	ENSRNOT00000025079	-0.957679426	9.96E-07
TUCP_002089	XLOC_085984	ENSRNOT00000048754	-0.953601181	1.57E-06
TUCP_002089	XLOC_085984	ENSRNOT00000038247	0.967249029	2.81E-07
TUCP_002089	XLOC_085984	ENSRNOT00000012977	0.963153699	5.03E-07
TUCP_002089	XLOC_085984	ENSRNOT00000021496	-0.955746115	1.24E-06
TUCP_002089	XLOC_085984	ENSRNOT00000036951	0.960701206	6.91E-07
TUCP_002089	XLOC_085984	ENSRNOT00000071764	0.95504176	1.34E-06
TUCP_002089	XLOC_085984	ENSRNOT00000035989	0.968290692	2.39E-07
TUCP_002089	XLOC_085984	ENSRNOT00000027944	0.950325962	2.19E-06
TUCP_002089	XLOC_085984	ENSRNOT00000024402	0.951531459	1.94E-06
TUCP_002089	XLOC_085984	ENSRNOT00000075326	0.957173132	1.06E-06
TUCP_002089	XLOC_085984	ENSRNOT00000021348	0.986428078	3.54E-09
TUCP_002089	XLOC_085984	ENSRNOT00000025097	0.969942695	1.84E-07
TUCP_002089	XLOC_085984	ENSRNOT00000068322	0.970298503	1.73E-07
TUCP_002089	XLOC_085984	ENSRNOT00000017629	0.990207246	6.98E-10
TUCP_002089	XLOC_085984	ENSRNOT00000077908	0.964735497	4.05E-07
TUCP_002089	XLOC_085984	ENSRNOT00000012671	0.966205995	3.28E-07
TUCP_002089	XLOC_085984	ENSRNOT00000085158	0.953656365	1.56E-06
TUCP_002089	XLOC_085984	ENSRNOT00000010463	0.963647533	4.70E-07
TUCP_002089	XLOC_085984	ENSRNOT00000064840	0.95817139	9.40E-07
TUCP_002089	XLOC_085984	ENSRNOT00000003146	-0.963358916	4.89E-07
TUCP_002089	XLOC_085984	ENSRNOT00000026718	0.970414035	1.70E-07
TUCP_002089	XLOC_085984	ENSRNOT00000085228	0.961727746	6.06E-07
TUCP_002089	XLOC_085984	ENSRNOT00000084012	0.957903962	9.70E-07
TUCP_002089	XLOC_085984	ENSRNOT00000022534	0.951827692	1.88E-06
TUCP_002089	XLOC_085984	ENSRNOT00000017319	0.961866593	5.96E-07
TUCP_002089	XLOC_085984	ENSRNOT00000055187	0.964747603	4.04E-07
TUCP_002089	XLOC_085984	ENSRNOT00000001809	0.95608028	1.20E-06
TUCP_002089	XLOC_085984	ENSRNOT00000004834	0.967967237	2.52E-07
TUCP_002089	XLOC_085984	ENSRNOT00000079866	0.958827176	8.69E-07
TUCP_002089	XLOC_085984	ENSRNOT00000024548	0.976480682	5.45E-08
TUCP_002089	XLOC_085984	ENSRNOT00000067822	0.979879821	2.51E-08
TUCP_002089	XLOC_085984	ENSRNOT00000024060	0.96236282	5.58E-07

TUCP_002089	XLOC_085984	ENSRNOT00000010927	0.959489302	8.03E-07
TUCP_002089	XLOC_085984	ENSRNOT00000087371	0.981239375	1.77E-08
TUCP_002089	XLOC_085984	ENSRNOT00000091909	0.955709773	1.25E-06
TUCP_002089	XLOC_085984	ENSRNOT00000088054	0.963565244	4.76E-07
TUCP_002089	XLOC_085984	ENSRNOT00000068633	0.978462759	3.52E-08
TUCP_002089	XLOC_085984	ENSRNOT00000010541	0.960690317	6.92E-07
TUCP_002089	XLOC_085984	ENSRNOT00000037446	0.996515937	4.02E-12
TUCP_002089	XLOC_085984	ENSRNOT00000023619	0.950162249	2.23E-06
TUCP_002089	XLOC_085984	ENSRNOT00000058645	0.956518936	1.14E-06
TUCP_002089	XLOC_085984	ENSRNOT00000017948	0.982477434	1.26E-08
TUCP_002089	XLOC_085984	ENSRNOT00000009156	0.95625932	1.17E-06
TUCP_002089	XLOC_085984	ENSRNOT00000026481	0.966753362	3.03E-07
TUCP_002089	XLOC_085984	ENSRNOT00000025859	0.950631139	2.13E-06
TUCP_002089	XLOC_085984	ENSRNOT00000023180	0.957676568	9.96E-07
TUCP_002089	XLOC_085984	ENSRNOT00000057313	0.978433296	3.54E-08
TUCP_002089	XLOC_085984	ENSRNOT00000021803	-0.951451002	1.96E-06
TUCP_002089	XLOC_085984	ENSRNOT00000029994	0.956424344	1.15E-06
TUCP_002089	XLOC_085984	ENSRNOT00000001081	0.956176081	1.18E-06
TUCP_002089	XLOC_085984	ENSRNOT00000000676	0.963536385	4.77E-07
TUCP_002089	XLOC_085984	ENSRNOT00000015609	0.954122491	1.48E-06
TUCP_002089	XLOC_085984	ENSRNOT00000019222	0.9806106	2.09E-08
TUCP_002089	XLOC_085984	ENSRNOT00000028070	0.967525461	2.69E-07
TUCP_002089	XLOC_085984	ENSRNOT00000004359	0.971309532	1.46E-07
TUCP_002089	XLOC_085984	ENSRNOT00000013274	0.987527433	2.33E-09
TUCP_002089	XLOC_085984	ENSRNOT00000023144	0.957075368	1.07E-06
TUCP_002089	XLOC_085984	ENSRNOT00000022610	0.950830199	2.08E-06
TUCP_002089	XLOC_085984	ENSRNOT00000020437	0.953010985	1.67E-06
TUCP_002089	XLOC_085984	ENSRNOT00000045574	0.953170037	1.64E-06
TUCP_002089	XLOC_085984	ENSRNOT00000010898	0.955556443	1.27E-06
TUCP_002089	XLOC_085984	ENSRNOT00000047093	0.958563404	8.97E-07
TUCP_002089	XLOC_085984	ENSRNOT00000005355	0.965069575	3.86E-07
TUCP_002089	XLOC_085984	ENSRNOT00000078675	0.968343292	2.37E-07
TUCP_002089	XLOC_085984	ENSRNOT00000079895	0.977399448	4.47E-08
TUCP_002089	XLOC_085984	ENSRNOT00000026089	0.955703294	1.25E-06
TUCP_002089	XLOC_085984	ENSRNOT00000005659	0.961286834	6.42E-07
TUCP_002089	XLOC_085984	ENSRNOT00000027520	0.95402201	1.50E-06
TUCP_002089	XLOC_085984	ENSRNOT00000026174	0.960691202	6.92E-07
TUCP_002089	XLOC_085984	ENSRNOT00000044635	0.96306657	5.09E-07
TUCP_002089	XLOC_085984	ENSRNOT00000047387	0.977153471	4.72E-08
TUCP_002089	XLOC_085984	ENSRNOT00000013661	0.954290704	1.46E-06
TUCP_002089	XLOC_085984	ENSRNOT00000091910	0.970922825	1.56E-07
TUCP_002089	XLOC_085984	ENSRNOT00000045488	0.973869469	9.18E-08
TUCP_002089	XLOC_085984	ENSRNOT00000039842	0.963735394	4.65E-07
TUCP_002089	XLOC_085984	ENSRNOT00000093174	0.958882526	8.64E-07
TUCP_002089	XLOC_085984	ENSRNOT00000083127	0.975603614	6.53E-08
TUCP_002089	XLOC_085984	ENSRNOT00000077555	0.973794137	9.31E-08
TUCP_002089	XLOC_085984	ENSRNOT00000011880	0.951660586	1.92E-06
TUCP_002089	XLOC_085984	ENSRNOT00000013260	0.954714554	1.39E-06
TUCP_002089	XLOC_085984	ENSRNOT00000017021	0.969724596	1.90E-07
TUCP_002089	XLOC_085984	ENSRNOT00000082228	0.967871424	2.55E-07
TUCP_002089	XLOC_085984	ENSRNOT00000003991	0.950611457	2.13E-06

TUCP_002089	XLOC_085984	ENSRNOT00000004508	0.954571647	1.41E-06
TUCP_002089	XLOC_085984	ENSRNOT00000034744	0.958506697	9.03E-07
TUCP_002089	XLOC_085984	ENSRNOT00000078475	0.966942924	2.94E-07
TUCP_002089	XLOC_085984	ENSRNOT00000087429	0.968668259	2.26E-07
TUCP_002089	XLOC_085984	ENSRNOT00000009683	0.991215095	4.06E-10
TUCP_002089	XLOC_085984	ENSRNOT00000020383	0.958680773	8.85E-07
TUCP_002089	XLOC_085984	ENSRNOT00000084088	0.974421669	8.26E-08
TUCP_002089	XLOC_085984	ENSRNOT00000017776	0.951020585	2.04E-06
TUCP_002089	XLOC_085984	ENSRNOT00000064902	0.979698091	2.63E-08
TUCP_002089	XLOC_085984	ENSRNOT00000088995	0.960934416	6.71E-07
TUCP_002089	XLOC_085984	ENSRNOT00000078070	0.950936907	2.06E-06
TUCP_002089	XLOC_085984	ENSRNOT00000002771	0.971171467	1.49E-07
TUCP_002089	XLOC_085984	ENSRNOT00000065983	0.960531234	7.06E-07
TUCP_002089	XLOC_085984	ENSRNOT00000079442	0.959228148	8.29E-07
TUCP_002089	XLOC_085984	ENSRNOT00000024322	0.970619543	1.64E-07
TUCP_002089	XLOC_085984	ENSRNOT00000090665	0.962532691	5.46E-07
TUCP_002089	XLOC_085984	ENSRNOT00000005144	0.96924961	2.06E-07
TUCP_002089	XLOC_085984	ENSRNOT00000016722	0.969706815	1.91E-07
TUCP_002089	XLOC_085984	ENSRNOT00000091265	0.960476355	7.11E-07
TUCP_002089	XLOC_085984	ENSRNOT00000012424	0.95997155	7.57E-07
TUCP_002089	XLOC_085984	ENSRNOT00000090170	0.959558491	7.96E-07
TUCP_002089	XLOC_085984	ENSRNOT00000026457	0.982879027	1.13E-08
TUCP_002089	XLOC_085984	ENSRNOT00000000658	0.974563782	8.04E-08
TUCP_002089	XLOC_085984	ENSRNOT00000083247	-0.95677872	1.10E-06
TUCP_002089	XLOC_085984	ENSRNOT00000078390	-0.954376402	1.44E-06
TUCP_002089	XLOC_085984	ENSRNOT00000089971	0.957958048	9.64E-07
TUCP_002089	XLOC_085984	ENSRNOT00000065501	0.97850324	3.49E-08
TUCP_002089	XLOC_085984	ENSRNOT00000006554	0.967813928	2.58E-07
TUCP_002089	XLOC_085984	ENSRNOT00000091488	0.970831466	1.58E-07
TUCP_002089	XLOC_085984	ENSRNOT00000025523	0.968653684	2.26E-07
TUCP_002089	XLOC_085984	ENSRNOT00000027452	0.969557692	1.96E-07
TUCP_002089	XLOC_085984	ENSRNOT00000076729	0.975334695	6.90E-08
TUCP_002089	XLOC_085984	ENSRNOT00000032505	-0.95638549	1.15E-06
TUCP_002089	XLOC_085984	ENSRNOT00000070933	0.961375667	6.34E-07
TUCP_002089	XLOC_085984	ENSRNOT00000005909	0.957801603	9.82E-07
TUCP_002089	XLOC_085984	ENSRNOT00000045882	0.959247765	8.27E-07
TUCP_002089	XLOC_085984	ENSRNOT00000034704	0.977464353	4.41E-08
TUCP_002089	XLOC_085984	ENSRNOT00000012702	-0.953697682	1.55E-06
TUCP_002089	XLOC_085984	ENSRNOT00000023330	0.958336482	9.22E-07
TUCP_002089	XLOC_085984	ENSRNOT00000025092	0.953235601	1.63E-06
TUCP_002089	XLOC_085984	ENSRNOT00000055582	0.953322229	1.61E-06
TUCP_002089	XLOC_085984	ENSRNOT00000037790	0.955803988	1.23E-06
TUCP_002089	XLOC_085984	ENSRNOT00000009691	0.950663839	2.12E-06
TUCP_002089	XLOC_085984	ENSRNOT00000027337	0.952725467	1.72E-06
TUCP_002089	XLOC_085984	ENSRNOT00000006969	0.969574612	1.95E-07
TUCP_002089	XLOC_085984	ENSRNOT00000007091	0.954065391	1.49E-06
TUCP_002089	XLOC_085984	ENSRNOT00000010224	0.953193591	1.64E-06
TUCP_002089	XLOC_085984	ENSRNOT00000090420	-0.95468002	1.40E-06
TUCP_002089	XLOC_085984	ENSRNOT00000075424	0.972163449	1.26E-07
TUCP_002089	XLOC_085984	ENSRNOT00000078929	0.95686194	1.09E-06
TUCP_002089	XLOC_085984	ENSRNOT00000081897	0.956557666	1.13E-06

TUCP_002089	XLOC_085984	ENSRNOT00000090424	0.952863422	1.69E-06
TUCP_002089	XLOC_085984	ENSRNOT00000073930	0.951505975	1.95E-06
TUCP_002089	XLOC_085984	ENSRNOT00000025075	0.956106835	1.19E-06
TUCP_002089	XLOC_085984	ENSRNOT00000019554	0.962417012	5.54E-07
TUCP_002089	XLOC_085984	ENSRNOT00000001222	0.969629911	1.93E-07
TUCP_002089	XLOC_085984	ENSRNOT00000023645	0.969936158	1.84E-07
TUCP_002089	XLOC_085984	ENSRNOT00000027234	0.957485115	1.02E-06
TUCP_002089	XLOC_085984	ENSRNOT00000010992	0.969404819	2.01E-07
TUCP_002089	XLOC_085984	ENSRNOT00000025832	0.96788211	2.55E-07
TUCP_002089	XLOC_085984	ENSRNOT00000038647	0.963628328	4.72E-07
TUCP_002089	XLOC_085984	ENSRNOT00000076669	0.965573501	3.59E-07
TUCP_002089	XLOC_085984	ENSRNOT00000011984	0.991743167	2.98E-10
TUCP_002089	XLOC_085984	ENSRNOT00000007175	0.951555952	1.94E-06
TUCP_002089	XLOC_085984	ENSRNOT00000086119	-0.960853542	6.78E-07
TUCP_002089	XLOC_085984	ENSRNOT00000026293	0.984702065	6.43E-09
TUCP_002089	XLOC_085984	ENSRNOT00000074653	0.98931811	1.08E-09
TUCP_002089	XLOC_085984	ENSRNOT00000089007	-0.958071177	9.51E-07
TUCP_002089	XLOC_085984	ENSRNOT00000039809	0.962827293	5.25E-07
TUCP_002089	XLOC_085984	ENSRNOT00000031230	0.988113858	1.83E-09
TUCP_002089	XLOC_085984	ENSRNOT00000041096	0.951548906	1.94E-06
TUCP_002089	XLOC_085984	ENSRNOT00000001923	0.955502317	1.27E-06
TUCP_002089	XLOC_085984	ENSRNOT00000010748	0.955544246	1.27E-06
TUCP_002089	XLOC_085984	ENSRNOT00000080928	0.962834189	5.25E-07
TUCP_002089	XLOC_085984	ENSRNOT00000052288	0.967579263	2.67E-07
TUCP_002089	XLOC_085984	ENSRNOT00000039631	0.967903552	2.54E-07
TUCP_002089	XLOC_085984	ENSRNOT00000045083	0.968313712	2.39E-07
TUCP_002089	XLOC_085984	ENSRNOT00000008427	-0.953060316	1.66E-06
TUCP_002089	XLOC_085984	ENSRNOT00000021061	-0.957957027	9.64E-07
TUCP_002089	XLOC_085984	ENSRNOT00000018074	-0.950385328	2.18E-06
TUCP_002089	XLOC_085984	ENSRNOT00000037679	0.970142784	1.78E-07
TUCP_002089	XLOC_085984	ENSRNOT00000027910	0.956062617	1.20E-06
TUCP_002089	XLOC_085984	ENSRNOT00000085596	0.994487787	3.97E-11
TUCP_002089	XLOC_085984	ENSRNOT00000071694	0.983431477	9.56E-09
TUCP_002089	XLOC_085984	ENSRNOT00000035491	0.972963716	1.09E-07
TUCP_002089	XLOC_085984	ENSRNOT00000060696	0.962134995	5.75E-07
TUCP_002089	XLOC_085984	ENSRNOT00000048029	0.967422294	2.74E-07
TUCP_002089	XLOC_085984	ENSRNOT00000010986	0.961781228	6.02E-07
TUCP_002089	XLOC_085984	ENSRNOT00000009900	-0.952703961	1.72E-06
TUCP_002089	XLOC_085984	ENSRNOT00000004412	0.985152156	5.54E-09
TUCP_002089	XLOC_085984	ENSRNOT00000066968	0.963506024	4.79E-07
TUCP_002089	XLOC_085984	ENSRNOT00000064798	0.958973528	8.54E-07
TUCP_002089	XLOC_085984	ENSRNOT00000080207	0.96436248	4.26E-07
TUCP_002089	XLOC_085984	ENSRNOT00000026280	0.961316872	6.39E-07
TUCP_002089	XLOC_085984	ENSRNOT00000061100	0.984285704	7.35E-09
TUCP_002089	XLOC_085984	ENSRNOT00000005387	0.974077841	8.83E-08
TUCP_002089	XLOC_085984	ENSRNOT00000024941	0.994075084	5.69E-11
TUCP_002089	XLOC_085984	ENSRNOT00000005381	0.957032049	1.07E-06
TUCP_002089	XLOC_085984	ENSRNOT00000043535	0.959924753	7.61E-07
TUCP_002089	XLOC_085984	ENSRNOT00000082174	0.985650909	4.68E-09
TUCP_002089	XLOC_085984	ENSRNOT00000018584	0.960113215	7.44E-07
TUCP_002089	XLOC_085984	ENSRNOT00000066593	0.950386406	2.18E-06

TUCP_002089	XLOC_085984	ENSRNOT00000013985	0.959255721	8.26E-07
TUCP_002089	XLOC_085984	ENSRNOT00000004463	0.965175764	3.80E-07
TUCP_002089	XLOC_085984	ENSRNOT00000027964	0.958386031	9.16E-07
TUCP_002089	XLOC_085984	ENSRNOT00000023428	0.959376394	8.14E-07
TUCP_002089	XLOC_085984	ENSRNOT00000024420	0.958510757	9.03E-07
TUCP_002089	XLOC_085984	ENSRNOT00000011041	0.965004432	3.90E-07
TUCP_002089	XLOC_085984	ENSRNOT00000021674	0.973459509	9.92E-08
TUCP_002089	XLOC_085984	ENSRNOT00000047192	-0.950006185	2.26E-06
TUCP_002089	XLOC_085984	ENSRNOT00000047210	0.963406695	4.86E-07
TUCP_002089	XLOC_085984	ENSRNOT00000002292	0.985982478	4.16E-09
TUCP_002089	XLOC_085984	ENSRNOT00000029001	0.965375116	3.70E-07
TUCP_002089	XLOC_085984	ENSRNOT00000078862	0.95264955	1.73E-06
TUCP_002089	XLOC_085984	ENSRNOT00000004140	0.963282036	4.94E-07
TUCP_002089	XLOC_085984	ENSRNOT00000085501	-0.95317173	1.64E-06
TUCP_002089	XLOC_085984	ENSRNOT00000064930	0.952537623	1.75E-06
TUCP_002089	XLOC_085984	ENSRNOT00000083799	0.978427967	3.55E-08
TUCP_002089	XLOC_085984	ENSRNOT00000009066	0.958005881	9.58E-07
TUCP_002089	XLOC_085984	ENSRNOT00000082253	0.95338436	1.60E-06
TUCP_002242	XLOC_093574	ENSRNOT00000021348	0.968505149	2.31E-07
TUCP_002242	XLOC_093574	ENSRNOT00000065168	0.964896834	3.96E-07
TUCP_002242	XLOC_093574	ENSRNOT00000085158	0.952950086	1.68E-06
TUCP_002242	XLOC_093574	ENSRNOT00000038647	0.969643413	1.93E-07
TUCP_002242	XLOC_093574	ENSRNOT00000060186	0.955937512	1.21E-06
TUCP_002242	XLOC_093574	ENSRNOT00000071764	0.970546423	1.66E-07
TUCP_002242	XLOC_093574	ENSRNOT00000091910	0.969178281	2.08E-07
TUCP_002242	XLOC_093574	ENSRNOT00000020134	0.974469293	8.18E-08
TUCP_002242	XLOC_093574	ENSRNOT00000016555	0.959962248	7.58E-07
TUCP_002242	XLOC_093574	ENSRNOT00000074939	0.969519147	1.97E-07
TUCP_002242	XLOC_093574	ENSRNOT00000077555	0.953094952	1.65E-06
TUCP_002242	XLOC_093574	ENSRNOT00000073559	0.958816558	8.71E-07
TUCP_002242	XLOC_093574	ENSRNOT00000017021	0.974736069	7.77E-08
TUCP_002242	XLOC_093574	ENSRNOT00000041096	0.952783994	1.71E-06
TUCP_002242	XLOC_093574	ENSRNOT00000087592	0.950009398	2.26E-06
TUCP_002242	XLOC_093574	ENSRNOT00000043856	0.95074664	2.10E-06
TUCP_002242	XLOC_093574	ENSRNOT00000032274	0.976565374	5.35E-08
TUCP_002242	XLOC_093574	ENSRNOT00000071733	0.950973638	2.05E-06
TUCP_002242	XLOC_093574	ENSRNOT00000024941	0.952159078	1.82E-06
TUCP_002242	XLOC_093574	ENSRNOT00000006175	0.981377695	1.71E-08
TUCP_002242	XLOC_093574	ENSRNOT00000009683	0.974125277	8.75E-08
TUCP_002242	XLOC_093574	ENSRNOT00000083127	0.951600295	1.93E-06
TUCP_002242	XLOC_093574	ENSRNOT00000026543	0.953940525	1.51E-06
TUCP_002242	XLOC_093574	ENSRNOT00000004359	0.953775174	1.54E-06
TUCP_002242	XLOC_093574	ENSRNOT00000024322	0.97035394	1.72E-07
TUCP_002242	XLOC_093574	ENSRNOT00000023490	0.950287809	2.20E-06
TUCP_002242	XLOC_093574	ENSRNOT00000027454	0.966212753	3.28E-07
TUCP_002242	XLOC_093574	ENSRNOT00000076729	0.955119627	1.33E-06
TUCP_002242	XLOC_093574	ENSRNOT00000090665	0.954340206	1.45E-06
TUCP_002242	XLOC_093574	ENSRNOT00000034704	0.960817002	6.81E-07
TUCP_002242	XLOC_093574	ENSRNOT00000016975	0.970995132	1.54E-07
TUCP_002242	XLOC_093574	ENSRNOT00000066670	0.953333535	1.61E-06
TUCP_002242	XLOC_093574	ENSRNOT00000091265	0.964571775	4.14E-07

TUCP_002242	XLOC_093574	ENSRNOT00000009712	0.955106841	1.33E-06
TUCP_002242	XLOC_093574	ENSRNOT00000055401	0.951249043	2.00E-06
TUCP_002242	XLOC_093574	ENSRNOT00000071697	0.953511062	1.58E-06
TUCP_002242	XLOC_093574	ENSRNOT00000060696	0.955318282	1.30E-06
TUCP_002242	XLOC_093574	ENSRNOT00000089971	0.984375193	7.14E-09
TUCP_002242	XLOC_093574	ENSRNOT00000065501	0.953823504	1.53E-06
TUCP_002242	XLOC_093574	ENSRNOT00000020028	0.952727716	1.72E-06
TUCP_002242	XLOC_093574	ENSRNOT00000043345	0.960092087	7.45E-07
TUCP_002242	XLOC_093574	ENSRNOT00000000676	0.970526221	1.67E-07
TUCP_002242	XLOC_093574	ENSRNOT00000027452	0.962475815	5.50E-07
TUCP_002242	XLOC_093574	ENSRNOT00000004412	0.951990974	1.85E-06
TUCP_002242	XLOC_093574	ENSRNOT00000024128	0.950359007	2.18E-06
TUCP_002242	XLOC_093574	ENSRNOT00000012048	0.959221841	8.29E-07
TUCP_002242	XLOC_093574	ENSRNOT00000072196	0.966075473	3.34E-07
TUCP_002242	XLOC_093574	ENSRNOT00000019222	0.96577657	3.49E-07
TUCP_002242	XLOC_093574	ENSRNOT00000066739	0.971061583	1.52E-07
TUCP_002242	XLOC_093574	ENSRNOT00000045882	0.978287318	3.66E-08
TUCP_002242	XLOC_093574	ENSRNOT00000025832	0.956814387	1.10E-06
TUCP_002242	XLOC_093574	ENSRNOT00000043535	0.964381227	4.25E-07
TUCP_002242	XLOC_093574	ENSRNOT00000013274	0.974793507	7.68E-08
TUCP_002242	XLOC_093574	ENSRNOT00000085027	0.952641347	1.73E-06
TUCP_002242	XLOC_093574	ENSRNOT00000044742	0.97366523	9.54E-08
TUCP_002242	XLOC_093574	ENSRNOT00000072534	0.953935998	1.51E-06
TUCP_002242	XLOC_093574	ENSRNOT00000000468	0.950169828	2.22E-06
TUCP_002242	XLOC_093574	ENSRNOT00000080088	0.950206796	2.22E-06
TUCP_002242	XLOC_093574	ENSRNOT00000014537	0.955961618	1.21E-06
TUCP_002242	XLOC_093574	ENSRNOT00000014664	0.973813073	9.28E-08
TUCP_002242	XLOC_093574	ENSRNOT00000031230	0.964404652	4.24E-07
TUCP_002242	XLOC_093574	ENSRNOT00000023428	0.980627847	2.08E-08
TUCP_002242	XLOC_093574	ENSRNOT00000075326	0.987557667	2.30E-09
TUCP_002242	XLOC_093574	ENSRNOT00000022796	0.951127784	2.02E-06
TUCP_002242	XLOC_093574	ENSRNOT00000073991	0.964384944	4.25E-07
TUCP_002242	XLOC_093574	ENSRNOT00000029001	0.964274507	4.32E-07
TUCP_002242	XLOC_093574	ENSRNOT00000029167	0.973713309	9.46E-08
TUCP_002242	XLOC_093574	ENSRNOT00000083799	0.959058656	8.46E-07
TUCP_002243	XLOC_093638	ENSRNOT00000084500	0.977097736	4.78E-08
TUCP_002243	XLOC_093638	ENSRNOT00000093467	0.988979816	1.26E-09
TUCP_002243	XLOC_093638	ENSRNOT00000029340	0.993506308	9.00E-11
TUCP_002243	XLOC_093638	ENSRNOT00000038646	0.994661577	3.38E-11
TUCP_002243	XLOC_093638	ENSRNOT00000012095	0.966714188	3.04E-07
TUCP_002243	XLOC_093638	ENSRNOT00000017909	0.967168975	2.84E-07
TUCP_002243	XLOC_093638	ENSRNOT00000092579	0.994661577	3.38E-11
TUCP_002243	XLOC_093638	ENSRNOT00000061516	0.981321509	1.74E-08
TUCP_002243	XLOC_093638	ENSRNOT00000036383	0.980301589	2.26E-08
TUCP_002243	XLOC_093638	ENSRNOT00000076528	0.99053819	5.88E-10
TUCP_002243	XLOC_093638	ENSRNOT00000083034	0.985296936	5.28E-09
TUCP_002243	XLOC_093638	ENSRNOT00000089607	0.987830255	2.06E-09
TUCP_002243	XLOC_093638	ENSRNOT00000086014	0.956747667	1.11E-06
TUCP_002243	XLOC_093638	ENSRNOT00000054939	0.978428388	3.55E-08
TUCP_002243	XLOC_093638	ENSRNOT00000052315	0.975806284	6.27E-08
TUCP_002243	XLOC_093638	ENSRNOT00000046231	0.953139626	1.64E-06

TUCP_002243	XLOC_093638	ENSRNOT00000013459	0.9708982	1.57E-07
TUCP_002243	XLOC_093638	ENSRNOT00000078137	0.994661577	3.38E-11
TUCP_002243	XLOC_093638	ENSRNOT00000077764	0.962673796	5.36E-07
TUCP_002243	XLOC_093638	ENSRNOT00000043661	0.958065065	9.52E-07
TUCP_002243	XLOC_093638	ENSRNOT00000092740	0.994661577	3.38E-11
TUCP_002243	XLOC_093638	ENSRNOT00000067034	0.954760093	1.38E-06
TUCP_002243	XLOC_093638	ENSRNOT00000000117	0.997702666	5.02E-13
TUCP_002243	XLOC_093638	ENSRNOT00000071877	0.955607418	1.26E-06
TUCP_002243	XLOC_093638	ENSRNOT00000070958	0.994661577	3.38E-11
TUCP_002243	XLOC_093638	ENSRNOT00000035538	0.953234927	1.63E-06
TUCP_002243	XLOC_093638	ENSRNOT00000085310	0.956427819	1.15E-06
TUCP_002243	XLOC_093638	ENSRNOT00000047241	0.957669489	9.97E-07
TUCP_002243	XLOC_093638	ENSRNOT00000027268	0.994661577	3.38E-11
TUCP_002243	XLOC_093638	ENSRNOT00000004459	0.994661577	3.38E-11
TUCP_002243	XLOC_093638	ENSRNOT00000089235	0.992314021	2.09E-10
TUCP_002243	XLOC_093638	ENSRNOT00000088168	0.994661577	3.38E-11
TUCP_002243	XLOC_093638	ENSRNOT00000088148	0.994661577	3.38E-11
TUCP_002243	XLOC_093638	ENSRNOT00000088527	0.994661577	3.38E-11
TUCP_002243	XLOC_093638	ENSRNOT00000078701	0.994661577	3.38E-11
TUCP_002243	XLOC_093638	ENSRNOT00000052308	0.953549322	1.58E-06
TUCP_002243	XLOC_093638	ENSRNOT00000021748	0.994661577	3.38E-11
TUCP_002243	XLOC_093638	ENSRNOT00000089085	0.994661577	3.38E-11
TUCP_002243	XLOC_093638	ENSRNOT00000008153	0.994441026	4.14E-11
TUCP_002243	XLOC_093638	ENSRNOT00000085896	0.972049493	1.28E-07
TUCP_002243	XLOC_093638	ENSRNOT00000079189	0.981646568	1.59E-08
TUCP_002243	XLOC_093638	ENSRNOT00000090078	0.994661577	3.38E-11
TUCP_002243	XLOC_093638	ENSRNOT00000078020	0.952293645	1.80E-06
TUCP_002243	XLOC_093638	ENSRNOT00000092425	0.967119244	2.86E-07
TUCP_002243	XLOC_093638	ENSRNOT00000009776	0.994661577	3.38E-11
TUCP_002248	XLOC_093808	ENSRNOT00000005653	0.970488264	1.68E-07
TUCP_002248	XLOC_093808	ENSRNOT00000059169	0.978031239	3.88E-08
TUCP_002248	XLOC_093808	ENSRNOT00000010561	0.9553557	1.30E-06
TUCP_002248	XLOC_093808	ENSRNOT00000010680	0.970191789	1.76E-07
TUCP_002248	XLOC_093808	ENSRNOT00000035973	-0.964188223	4.37E-07
TUCP_002248	XLOC_093808	ENSRNOT00000020832	0.957095486	1.07E-06
TUCP_002248	XLOC_093808	ENSRNOT00000013582	-0.96221135	5.70E-07
TUCP_002248	XLOC_093808	ENSRNOT00000076695	0.954117593	1.48E-06
TUCP_002248	XLOC_093808	ENSRNOT00000079432	0.98109107	1.84E-08
TUCP_002248	XLOC_093808	ENSRNOT00000026381	-0.983466422	9.46E-09
TUCP_002248	XLOC_093808	ENSRNOT00000018864	-0.952589416	1.74E-06
TUCP_002248	XLOC_093808	ENSRNOT00000018711	-0.962910539	5.19E-07
TUCP_002248	XLOC_093808	ENSRNOT00000064625	0.957059784	1.07E-06
TUCP_002248	XLOC_093808	ENSRNOT00000061132	0.95237557	1.78E-06
TUCP_002249	XLOC_093848	ENSRNOT00000019104	0.971949471	1.30E-07
TUCP_002249	XLOC_093848	ENSRNOT00000019848	0.955113878	1.33E-06
TUCP_002249	XLOC_093848	ENSRNOT00000077451	0.95401109	1.50E-06
TUCP_002249	XLOC_093848	ENSRNOT00000015566	-0.950079893	2.24E-06
TUCP_002249	XLOC_093848	ENSRNOT00000025067	0.970195756	1.76E-07
TUCP_002249	XLOC_093848	ENSRNOT00000006913	0.965749274	3.50E-07
TUCP_002249	XLOC_093848	ENSRNOT00000030914	0.972050442	1.28E-07
TUCP_002249	XLOC_093848	ENSRNOT00000086310	0.96239696	5.56E-07

TUCP_002249	XLOC_093848	ENSRNOT00000011130	0.950786236	2.09E-06
TUCP_002249	XLOC_093848	ENSRNOT00000014384	0.953729605	1.55E-06
TUCP_002249	XLOC_093848	ENSRNOT00000030850	-0.95034087	2.19E-06
TUCP_002249	XLOC_093848	ENSRNOT00000074606	0.952562718	1.75E-06
TUCP_002249	XLOC_093848	ENSRNOT00000086893	0.951885579	1.87E-06
TUCP_002249	XLOC_093848	ENSRNOT00000055540	-0.958204088	9.36E-07
TUCP_002249	XLOC_093848	ENSRNOT00000003247	0.959880611	7.65E-07
TUCP_002249	XLOC_093848	ENSRNOT00000025284	0.978348388	3.61E-08
TUCP_002249	XLOC_093848	ENSRNOT00000022126	0.957442285	1.02E-06
TUCP_002249	XLOC_093848	ENSRNOT00000064153	-0.953598327	1.57E-06
TUCP_002249	XLOC_093848	ENSRNOT00000035320	0.971314675	1.46E-07
TUCP_002249	XLOC_093848	ENSRNOT00000017469	0.967033001	2.90E-07
TUCP_002249	XLOC_093848	ENSRNOT00000077999	-0.952380107	1.78E-06
TUCP_002249	XLOC_093848	ENSRNOT00000046807	0.950871848	2.07E-06
TUCP_002249	XLOC_093848	ENSRNOT00000026398	0.966155389	3.30E-07
TUCP_002249	XLOC_093848	ENSRNOT00000024264	0.96276696	5.29E-07
TUCP_002249	XLOC_093848	ENSRNOT00000015188	-0.960833348	6.80E-07
TUCP_002249	XLOC_093848	ENSRNOT00000018488	0.960052435	7.49E-07
TUCP_002249	XLOC_093848	ENSRNOT00000080522	0.972298414	1.23E-07
TUCP_002249	XLOC_093848	ENSRNOT00000030007	0.951825086	1.88E-06
TUCP_002249	XLOC_093848	ENSRNOT00000082686	0.965425578	3.67E-07
TUCP_002249	XLOC_093848	ENSRNOT00000010141	-0.95240487	1.78E-06
TUCP_002249	XLOC_093848	ENSRNOT00000026462	0.967574587	2.67E-07
TUCP_002249	XLOC_093848	ENSRNOT00000084709	-0.955420193	1.29E-06
TUCP_002249	XLOC_093848	ENSRNOT00000029179	0.964251964	4.33E-07
TUCP_002249	XLOC_093848	ENSRNOT00000026481	-0.953098421	1.65E-06
TUCP_002249	XLOC_093848	ENSRNOT00000044562	-0.958461918	9.08E-07
TUCP_002249	XLOC_093848	ENSRNOT00000043752	0.958412265	9.14E-07
TUCP_002249	XLOC_093848	ENSRNOT00000017813	0.958719266	8.81E-07
TUCP_002249	XLOC_093848	ENSRNOT00000026525	0.969790541	1.88E-07
TUCP_002249	XLOC_093848	ENSRNOT00000001404	0.974667338	7.87E-08
TUCP_002249	XLOC_093848	ENSRNOT00000001401	-0.957340046	1.04E-06
TUCP_002249	XLOC_093848	ENSRNOT00000016132	0.956539849	1.13E-06
TUCP_002249	XLOC_093848	ENSRNOT00000001366	0.961437246	6.29E-07
TUCP_002249	XLOC_093848	ENSRNOT00000013067	0.953780211	1.54E-06
TUCP_002249	XLOC_093848	ENSRNOT00000001360	0.958567234	8.97E-07
TUCP_002249	XLOC_093848	ENSRNOT00000046486	0.956786077	1.10E-06
TUCP_002249	XLOC_093848	ENSRNOT00000016220	0.959650966	7.87E-07
TUCP_002249	XLOC_093848	ENSRNOT00000017081	0.964639332	4.10E-07
TUCP_002249	XLOC_093848	ENSRNOT00000002313	0.966347196	3.21E-07
TUCP_002249	XLOC_093848	ENSRNOT00000037389	0.952992993	1.67E-06
TUCP_002249	XLOC_093848	ENSRNOT00000016637	0.972089736	1.27E-07
TUCP_002249	XLOC_093848	ENSRNOT00000031979	0.964138011	4.40E-07
TUCP_002249	XLOC_093848	ENSRNOT00000076387	-0.950260432	2.21E-06
TUCP_002249	XLOC_093848	ENSRNOT00000045765	0.952915637	1.68E-06
TUCP_002249	XLOC_093848	ENSRNOT00000071929	0.958829812	8.69E-07
TUCP_002249	XLOC_093848	ENSRNOT00000064305	0.96614214	3.31E-07
TUCP_002249	XLOC_093848	ENSRNOT00000000532	0.963366085	4.89E-07
TUCP_002249	XLOC_093848	ENSRNOT00000016458	-0.960046347	7.50E-07
TUCP_002249	XLOC_093848	ENSRNOT00000003992	0.966658639	3.07E-07
TUCP_002249	XLOC_093848	ENSRNOT00000046983	0.95779528	9.82E-07

TUCP_002249	XLOC_093848	ENSRNOT00000050515	-0.952960474	1.68E-06
TUCP_002249	XLOC_093848	ENSRNOT00000078073	0.966870718	2.97E-07
TUCP_002249	XLOC_093848	ENSRNOT00000064901	-0.954265342	1.46E-06
TUCP_002249	XLOC_093848	ENSRNOT00000087997	-0.955429602	1.29E-06
TUCP_002249	XLOC_093848	ENSRNOT00000055607	0.964264293	4.32E-07
TUCP_002249	XLOC_093848	ENSRNOT00000032289	0.963045267	5.10E-07
TUCP_002249	XLOC_093848	ENSRNOT00000042633	0.952080266	1.84E-06
TUCP_002249	XLOC_093848	ENSRNOT00000019157	0.958215055	9.35E-07
TUCP_002249	XLOC_093848	ENSRNOT00000003207	0.951656604	1.92E-06
TUCP_002249	XLOC_093848	ENSRNOT00000006366	0.966339921	3.22E-07
TUCP_002249	XLOC_093848	ENSRNOT00000079698	0.977722019	4.16E-08
TUCP_002249	XLOC_093848	ENSRNOT00000044847	0.959492788	8.02E-07
TUCP_002249	XLOC_093848	ENSRNOT00000064723	0.962477883	5.50E-07
TUCP_002249	XLOC_093848	ENSRNOT00000073834	0.958418239	9.13E-07
TUCP_002249	XLOC_093848	ENSRNOT00000086456	0.961731535	6.06E-07
TUCP_002249	XLOC_093848	ENSRNOT00000036959	0.978826547	3.23E-08
TUCP_002249	XLOC_093848	ENSRNOT00000075209	0.956919268	1.09E-06
TUCP_002249	XLOC_093848	ENSRNOT00000056145	0.952048049	1.84E-06
TUCP_002249	XLOC_093848	ENSRNOT00000001227	0.951357757	1.98E-06
TUCP_002249	XLOC_093848	ENSRNOT00000066133	0.953948231	1.51E-06
TUCP_002249	XLOC_093848	ENSRNOT00000067741	0.956664269	1.12E-06
TUCP_002249	XLOC_093848	ENSRNOT00000048933	0.965710077	3.52E-07
TUCP_002249	XLOC_093848	ENSRNOT00000051953	0.962621991	5.40E-07
TUCP_002249	XLOC_093848	ENSRNOT00000035385	-0.953682632	1.55E-06
TUCP_002249	XLOC_093848	ENSRNOT00000000728	0.958570041	8.97E-07
TUCP_002249	XLOC_093848	ENSRNOT00000019487	-0.963492018	4.80E-07
TUCP_002249	XLOC_093848	ENSRNOT00000071192	0.956103951	1.19E-06
TUCP_002249	XLOC_093848	ENSRNOT00000018173	0.979016872	3.09E-08
TUCP_002249	XLOC_093848	ENSRNOT00000064535	0.9546923	1.39E-06
TUCP_002249	XLOC_093848	ENSRNOT00000010748	-0.952576612	1.74E-06
TUCP_002249	XLOC_093848	ENSRNOT00000004762	-0.952133875	1.83E-06
TUCP_002249	XLOC_093848	ENSRNOT00000040060	0.955965086	1.21E-06
TUCP_002249	XLOC_093848	ENSRNOT00000007044	0.965932467	3.41E-07
TUCP_002249	XLOC_093848	ENSRNOT00000010983	-0.950134856	2.23E-06
TUCP_002249	XLOC_093848	ENSRNOT00000004787	0.954422068	1.43E-06
TUCP_002249	XLOC_093848	ENSRNOT00000058351	-0.955958491	1.21E-06
TUCP_002249	XLOC_093848	ENSRNOT00000004941	0.961111434	6.56E-07
TUCP_002249	XLOC_093848	ENSRNOT00000027753	0.951221072	2.00E-06
TUCP_002249	XLOC_093848	ENSRNOT00000023628	0.951462011	1.96E-06
TUCP_002249	XLOC_093848	ENSRNOT00000088679	0.956393173	1.15E-06
TUCP_002249	XLOC_093848	ENSRNOT00000016776	0.963625275	4.72E-07
TUCP_002249	XLOC_093848	ENSRNOT00000025913	0.954820328	1.37E-06
TUCP_002249	XLOC_093848	ENSRNOT00000031935	0.953022694	1.66E-06
TUCP_002249	XLOC_093848	ENSRNOT00000024253	-0.962602637	5.41E-07
TUCP_002249	XLOC_093848	ENSRNOT00000045833	-0.956344015	1.16E-06
TUCP_002249	XLOC_093848	ENSRNOT00000063930	0.950454384	2.16E-06
TUCP_002249	XLOC_093848	ENSRNOT00000046215	0.962304978	5.63E-07
TUCP_002249	XLOC_093848	ENSRNOT00000013116	0.959520099	8.00E-07
TUCP_002249	XLOC_093848	ENSRNOT00000013025	0.95273378	1.72E-06
TUCP_002249	XLOC_093848	ENSRNOT00000014479	0.978447122	3.53E-08
TUCP_002249	XLOC_093848	ENSRNOT00000027220	0.960748659	6.87E-07

TUCP_002249	XLOC_093848	ENSRNOT00000003326	0.966849222	2.98E-07
TUCP_002249	XLOC_093848	ENSRNOT00000016306	0.961472436	6.27E-07
TUCP_002249	XLOC_093848	ENSRNOT00000015476	0.957173479	1.06E-06
TUCP_002249	XLOC_093848	ENSRNOT00000073019	-0.956762236	1.11E-06
TUCP_002249	XLOC_093848	ENSRNOT00000004007	0.98824911	1.73E-09
TUCP_002249	XLOC_093848	ENSRNOT00000022432	0.963730991	4.65E-07
TUCP_002249	XLOC_093848	ENSRNOT00000009817	0.962192899	5.71E-07
TUCP_002249	XLOC_093848	ENSRNOT00000009708	0.965256167	3.76E-07
TUCP_002249	XLOC_093848	ENSRNOT00000006443	0.95556697	1.27E-06
TUCP_002249	XLOC_093848	ENSRNOT00000025294	0.967219432	2.82E-07
TUCP_002249	XLOC_093848	ENSRNOT00000021523	-0.964534482	4.16E-07
TUCP_002249	XLOC_093848	ENSRNOT00000072762	0.951666238	1.92E-06
TUCP_002249	XLOC_093848	ENSRNOT00000012583	0.960500124	7.09E-07
TUCP_002249	XLOC_093848	ENSRNOT00000010318	0.953021625	1.67E-06
TUCP_002249	XLOC_093848	ENSRNOT00000052002	-0.953712266	1.55E-06
TUCP_002249	XLOC_093848	ENSRNOT00000022502	0.972555486	1.17E-07
TUCP_002249	XLOC_093848	ENSRNOT00000026928	0.963739413	4.65E-07
TUCP_002249	XLOC_093848	ENSRNOT00000087443	-0.950577432	2.14E-06
TUCP_002249	XLOC_093848	ENSRNOT00000018028	0.969479326	1.98E-07
TUCP_002249	XLOC_093848	ENSRNOT00000021803	0.961621987	6.15E-07
TUCP_002249	XLOC_093848	ENSRNOT00000003070	0.951247505	2.00E-06
TUCP_002249	XLOC_093848	ENSRNOT00000039850	0.953088473	1.65E-06
TUCP_002249	XLOC_093848	ENSRNOT00000003787	0.950802558	2.09E-06
TUCP_002249	XLOC_093848	ENSRNOT00000023262	0.954804462	1.38E-06
TUCP_002249	XLOC_093848	ENSRNOT00000014859	0.955031248	1.34E-06
TUCP_002249	XLOC_093848	ENSRNOT00000010674	0.970099973	1.79E-07
TUCP_002249	XLOC_093848	ENSRNOT00000068381	-0.962498406	5.48E-07
TUCP_002249	XLOC_093848	ENSRNOT00000007314	0.955233749	1.31E-06
TUCP_002249	XLOC_093848	ENSRNOT00000090456	-0.963763782	4.63E-07
TUCP_002249	XLOC_093848	ENSRNOT00000005170	0.950404085	2.17E-06
TUCP_002249	XLOC_093848	ENSRNOT00000023567	0.967275282	2.80E-07
TUCP_002249	XLOC_093848	ENSRNOT00000071897	0.961406446	6.32E-07
TUCP_002249	XLOC_093848	ENSRNOT00000024285	0.96030211	7.26E-07
TUCP_002249	XLOC_093848	ENSRNOT00000041277	0.955171966	1.32E-06
TUCP_002249	XLOC_093848	ENSRNOT00000071482	0.954672427	1.40E-06
TUCP_002249	XLOC_093848	ENSRNOT00000028744	0.964304076	4.30E-07
TUCP_002249	XLOC_093848	ENSRNOT00000028885	0.958675903	8.85E-07
TUCP_002249	XLOC_093848	ENSRNOT00000067901	0.964909399	3.95E-07
TUCP_002249	XLOC_093848	ENSRNOT00000088202	0.959169133	8.34E-07
TUCP_002249	XLOC_093848	ENSRNOT00000029833	-0.967635192	2.65E-07
TUCP_002249	XLOC_093848	ENSRNOT00000061916	0.96412061	4.41E-07
TUCP_002249	XLOC_093848	ENSRNOT00000078161	0.962580403	5.43E-07
TUCP_002249	XLOC_093848	ENSRNOT00000011572	0.966972425	2.93E-07
TUCP_002249	XLOC_093848	ENSRNOT00000001607	-0.970738344	1.61E-07
TUCP_002249	XLOC_093848	ENSRNOT00000036282	-0.955371902	1.29E-06
TUCP_002249	XLOC_093848	ENSRNOT00000020669	-0.952240607	1.81E-06
TUCP_002249	XLOC_093848	ENSRNOT00000085971	0.951012664	2.05E-06
TUCP_002249	XLOC_093848	ENSRNOT00000020663	0.954104854	1.48E-06
TUCP_002249	XLOC_093848	ENSRNOT00000022142	-0.96345257	4.83E-07
TUCP_002249	XLOC_093848	ENSRNOT00000076300	0.962754998	5.30E-07
TUCP_002249	XLOC_093848	ENSRNOT00000015537	-0.961995718	5.86E-07

TUCP_002249	XLOC_093848	ENSRNOT00000011499	0.950927372	2.06E-06
TUCP_002249	XLOC_093848	ENSRNOT00000015946	0.977484192	4.39E-08
TUCP_002249	XLOC_093848	ENSRNOT00000004172	-0.952292335	1.80E-06
TUCP_002249	XLOC_093848	ENSRNOT00000001234	0.955150476	1.33E-06
TUCP_002249	XLOC_093848	ENSRNOT00000023962	0.967331952	2.77E-07
TUCP_002249	XLOC_093848	ENSRNOT00000085279	-0.960194278	7.36E-07
TUCP_002249	XLOC_093848	ENSRNOT00000006570	0.965970606	3.39E-07
TUCP_002249	XLOC_093848	ENSRNOT00000025819	0.967212095	2.82E-07
TUCP_002249	XLOC_093848	ENSRNOT00000015318	0.953210378	1.63E-06
TUCP_002249	XLOC_093848	ENSRNOT00000010224	-0.950238328	2.21E-06
TUCP_002249	XLOC_093848	ENSRNOT00000005486	0.971563704	1.40E-07
TUCP_002249	XLOC_093848	ENSRNOT00000045532	0.952155955	1.82E-06
TUCP_002249	XLOC_093848	ENSRNOT00000004770	0.954758305	1.38E-06
TUCP_002249	XLOC_093848	ENSRNOT00000017687	0.969164172	2.08E-07
TUCP_002249	XLOC_093848	ENSRNOT00000039296	-0.956319318	1.16E-06
TUCP_002249	XLOC_093848	ENSRNOT00000039551	0.962859098	5.23E-07
TUCP_002249	XLOC_093848	ENSRNOT00000017269	0.955752162	1.24E-06
TUCP_002249	XLOC_093848	ENSRNOT00000087386	0.954099345	1.49E-06
TUCP_002249	XLOC_093848	ENSRNOT00000050795	-0.951575197	1.93E-06
TUCP_002249	XLOC_093848	ENSRNOT00000083573	0.983547242	9.24E-09
TUCP_002249	XLOC_093848	ENSRNOT00000083578	-0.951884193	1.87E-06
TUCP_002249	XLOC_093848	ENSRNOT00000059524	0.957579885	1.01E-06
TUCP_002249	XLOC_093848	ENSRNOT00000017362	0.966664344	3.07E-07
TUCP_002249	XLOC_093848	ENSRNOT00000005658	0.953472567	1.59E-06
TUCP_002249	XLOC_093848	ENSRNOT00000045326	-0.95065575	2.12E-06
TUCP_002249	XLOC_093848	ENSRNOT00000090342	-0.957041724	1.07E-06
TUCP_002249	XLOC_093848	ENSRNOT00000019465	0.969998528	1.82E-07
TUCP_002249	XLOC_093848	ENSRNOT00000067170	0.967877307	2.55E-07
TUCP_002249	XLOC_093848	ENSRNOT00000060432	0.976320902	5.63E-08
TUCP_002249	XLOC_093848	ENSRNOT00000001586	0.962366856	5.58E-07
TUCP_002249	XLOC_093848	ENSRNOT00000029332	0.966565074	3.11E-07
TUCP_002249	XLOC_093848	ENSRNOT00000081786	0.965870747	3.44E-07
TUCP_002249	XLOC_093848	ENSRNOT00000019820	-0.955800099	1.23E-06
TUCP_002249	XLOC_093848	ENSRNOT00000013695	0.956370645	1.16E-06
TUCP_002249	XLOC_093848	ENSRNOT00000006050	0.955939534	1.21E-06
TUCP_002249	XLOC_093848	ENSRNOT00000009000	0.954082077	1.49E-06
TUCP_002249	XLOC_093848	ENSRNOT00000089545	0.964672975	4.08E-07
TUCP_002249	XLOC_093848	ENSRNOT00000019519	-0.956748027	1.11E-06
TUCP_002249	XLOC_093848	ENSRNOT00000077908	-0.953252882	1.63E-06
TUCP_002249	XLOC_093848	ENSRNOT00000090461	-0.959194086	8.32E-07
TUCP_002249	XLOC_093848	ENSRNOT00000010783	0.959216072	8.30E-07
TUCP_002249	XLOC_093848	ENSRNOT00000016330	0.985457735	5.00E-09
TUCP_002249	XLOC_093848	ENSRNOT00000019121	0.953914152	1.52E-06
TUCP_002249	XLOC_093848	ENSRNOT00000036931	0.957290809	1.04E-06
TUCP_002249	XLOC_093848	ENSRNOT00000012364	0.953640153	1.56E-06
TUCP_002249	XLOC_093848	ENSRNOT00000016389	0.970068092	1.80E-07
TUCP_002249	XLOC_093848	ENSRNOT00000064679	0.95456717	1.41E-06
TUCP_002249	XLOC_093848	ENSRNOT00000032301	0.951432539	1.96E-06
TUCP_002249	XLOC_093848	ENSRNOT00000021030	0.971007809	1.54E-07
TUCP_002249	XLOC_093848	ENSRNOT00000077727	0.950604352	2.13E-06
TUCP_002249	XLOC_093848	ENSRNOT00000044346	0.963476088	4.81E-07

TUCP_002249	XLOC_093848	ENSRNOT00000000206	0.960759864	6.86E-07
TUCP_002249	XLOC_093848	ENSRNOT00000091864	0.964698528	4.07E-07
TUCP_002249	XLOC_093848	ENSRNOT00000001081	-0.954296985	1.45E-06
TUCP_002249	XLOC_093848	ENSRNOT00000051843	0.974499093	8.14E-08
TUCP_002249	XLOC_093848	ENSRNOT00000003540	0.951433166	1.96E-06
TUCP_002249	XLOC_093848	ENSRNOT00000067739	0.970187788	1.76E-07
TUCP_002249	XLOC_093848	ENSRNOT00000019386	0.963783873	4.62E-07
TUCP_002249	XLOC_093848	ENSRNOT00000006308	0.950193669	2.22E-06
TUCP_002249	XLOC_093848	ENSRNOT00000030782	0.955314857	1.30E-06
TUCP_002249	XLOC_093848	ENSRNOT00000083110	0.964825728	4.00E-07
TUCP_002249	XLOC_093848	ENSRNOT00000036658	0.955787249	1.24E-06
TUCP_002249	XLOC_093848	ENSRNOT00000030803	0.963531152	4.78E-07
TUCP_002249	XLOC_093848	ENSRNOT00000052422	-0.964131718	4.40E-07
TUCP_002249	XLOC_093848	ENSRNOT00000064588	-0.955824412	1.23E-06
TUCP_002249	XLOC_093848	ENSRNOT00000086590	-0.953043193	1.66E-06
TUCP_002249	XLOC_093848	ENSRNOT00000071239	0.955596876	1.26E-06
TUCP_002249	XLOC_093848	ENSRNOT00000086608	0.960139832	7.41E-07
TUCP_002249	XLOC_093848	ENSRNOT00000014585	0.952554017	1.75E-06
TUCP_002249	XLOC_093848	ENSRNOT00000000013	-0.954548475	1.42E-06
TUCP_002249	XLOC_093848	ENSRNOT00000080222	0.969446969	1.99E-07
TUCP_002249	XLOC_093848	ENSRNOT00000046594	-0.961786322	6.02E-07
TUCP_002249	XLOC_093848	ENSRNOT00000061191	0.968499868	2.32E-07
TUCP_002249	XLOC_093848	ENSRNOT00000029202	0.966461779	3.16E-07
TUCP_002249	XLOC_093848	ENSRNOT00000017907	0.955852345	1.23E-06
TUCP_002249	XLOC_093848	ENSRNOT00000028434	0.955904556	1.22E-06
TUCP_002249	XLOC_093848	ENSRNOT00000025794	0.954330409	1.45E-06
TUCP_002249	XLOC_093848	ENSRNOT00000058111	0.961244908	6.45E-07
TUCP_002249	XLOC_093848	ENSRNOT00000054828	-0.97212358	1.27E-07
TUCP_002249	XLOC_093848	ENSRNOT00000009994	0.956806716	1.10E-06
TUCP_002249	XLOC_093848	ENSRNOT00000005762	0.952307024	1.79E-06
TUCP_002249	XLOC_093848	ENSRNOT00000077646	0.955222504	1.31E-06
TUCP_002249	XLOC_093848	ENSRNOT00000021075	0.963392711	4.87E-07
TUCP_002249	XLOC_093848	ENSRNOT00000000083	0.95902905	8.49E-07
TUCP_002249	XLOC_093848	ENSRNOT00000087838	0.965173156	3.81E-07
TUCP_002249	XLOC_093848	ENSRNOT00000016265	0.955145525	1.33E-06
TUCP_002249	XLOC_093848	ENSRNOT00000027575	0.966033773	3.36E-07
TUCP_002249	XLOC_093848	ENSRNOT00000028225	-0.952124827	1.83E-06
TUCP_002249	XLOC_093848	ENSRNOT00000019172	0.975583287	6.56E-08
TUCP_002249	XLOC_093848	ENSRNOT00000020994	0.962411305	5.55E-07
TUCP_002249	XLOC_093848	ENSRNOT00000016372	0.967840567	2.57E-07
TUCP_002249	XLOC_093848	ENSRNOT00000008757	-0.95962278	7.90E-07
TUCP_002249	XLOC_093848	ENSRNOT00000045226	0.960075358	7.47E-07
TUCP_002249	XLOC_093848	ENSRNOT00000036194	0.954414934	1.44E-06
TUCP_002249	XLOC_093848	ENSRNOT00000023977	0.954432707	1.43E-06
TUCP_002249	XLOC_093848	ENSRNOT00000077186	0.953113026	1.65E-06
TUCP_002249	XLOC_093848	ENSRNOT00000014780	0.958444107	9.10E-07
TUCP_002249	XLOC_093848	ENSRNOT00000020357	-0.953119509	1.65E-06
TUCP_002249	XLOC_093848	ENSRNOT00000009645	0.95048215	2.16E-06
TUCP_002249	XLOC_093848	ENSRNOT00000026279	0.972119534	1.27E-07
TUCP_002249	XLOC_093848	ENSRNOT00000087062	-0.957170778	1.06E-06
TUCP_002249	XLOC_093848	ENSRNOT00000082508	0.958178321	9.39E-07

TUCP_002249	XLOC_093848	ENSRNOT00000022259	0.958942355	8.58E-07
TUCP_002249	XLOC_093848	ENSRNOT00000082863	-0.956626608	1.12E-06
TUCP_002249	XLOC_093848	ENSRNOT00000010333	0.953134521	1.65E-06
TUCP_002249	XLOC_093848	ENSRNOT00000065942	0.964577359	4.14E-07
TUCP_002249	XLOC_093848	ENSRNOT00000014309	0.952042255	1.84E-06
TUCP_002249	XLOC_093848	ENSRNOT00000060282	-0.963275724	4.95E-07
TUCP_002249	XLOC_093848	ENSRNOT00000074389	0.962605951	5.41E-07
TUCP_002249	XLOC_093848	ENSRNOT00000066204	0.954489001	1.42E-06
TUCP_002249	XLOC_093848	ENSRNOT00000018101	0.954187632	1.47E-06
TUCP_002249	XLOC_093848	ENSRNOT00000003012	0.972189838	1.25E-07
TUCP_002249	XLOC_093848	ENSRNOT00000090970	-0.951296535	1.99E-06
TUCP_002249	XLOC_093848	ENSRNOT00000007341	0.960052036	7.49E-07
TUCP_002249	XLOC_093848	ENSRNOT00000087604	-0.952914661	1.68E-06
TUCP_002249	XLOC_093848	ENSRNOT00000005620	0.951791768	1.89E-06
TUCP_002249	XLOC_093848	ENSRNOT00000039177	0.955754433	1.24E-06
TUCP_002249	XLOC_093848	ENSRNOT00000016801	-0.952459826	1.77E-06
TUCP_002249	XLOC_093848	ENSRNOT00000035628	0.957209461	1.05E-06
TUCP_002249	XLOC_093848	ENSRNOT00000000577	-0.968225646	2.42E-07
TUCP_002249	XLOC_093848	ENSRNOT00000063917	0.966370251	3.20E-07
TUCP_002249	XLOC_093848	ENSRNOT00000001979	0.968989898	2.14E-07
TUCP_002249	XLOC_093848	ENSRNOT00000083968	0.967001293	2.92E-07
TUCP_002249	XLOC_093848	ENSRNOT00000083964	0.951309124	1.99E-06
TUCP_002249	XLOC_093848	ENSRNOT00000026726	0.951127412	2.02E-06
TUCP_002249	XLOC_093848	ENSRNOT00000061304	0.976560821	5.36E-08
TUCP_002249	XLOC_093848	ENSRNOT00000020892	0.95659455	1.13E-06
TUCP_002249	XLOC_093848	ENSRNOT00000019133	-0.950752111	2.10E-06
TUCP_002249	XLOC_093848	ENSRNOT00000002151	0.963470416	4.82E-07
TUCP_002249	XLOC_093848	ENSRNOT00000020537	0.960414675	7.16E-07
TUCP_002249	XLOC_093848	ENSRNOT00000006824	0.953102646	1.65E-06
TUCP_002249	XLOC_093848	ENSRNOT00000012775	0.960612957	6.99E-07
TUCP_002249	XLOC_093848	ENSRNOT00000000177	0.951657686	1.92E-06
TUCP_002249	XLOC_093848	ENSRNOT00000018328	0.966537804	3.12E-07
TUCP_002249	XLOC_093848	ENSRNOT00000010683	0.9543978	1.44E-06
TUCP_002249	XLOC_093848	ENSRNOT00000031261	-0.977631658	4.25E-08
TUCP_002249	XLOC_093848	ENSRNOT00000067836	0.984444354	6.99E-09
TUCP_002249	XLOC_093848	ENSRNOT00000019816	0.962066259	5.80E-07
TUCP_002249	XLOC_093848	ENSRNOT00000073847	0.954030404	1.50E-06
TUCP_002249	XLOC_093848	ENSRNOT00000064068	0.972306924	1.22E-07
TUCP_002249	XLOC_093848	ENSRNOT00000011174	0.963580348	4.75E-07
TUCP_002249	XLOC_093848	ENSRNOT00000056015	0.969275133	2.05E-07
TUCP_002249	XLOC_093848	ENSRNOT00000090784	0.953646711	1.56E-06
TUCP_002249	XLOC_093848	ENSRNOT00000068528	0.957616703	1.00E-06
TUCP_002249	XLOC_093848	ENSRNOT00000087502	-0.951298135	1.99E-06
TUCP_002249	XLOC_093848	ENSRNOT00000039000	0.974839404	7.61E-08
TUCP_002249	XLOC_093848	ENSRNOT00000010906	0.966025176	3.37E-07
TUCP_002249	XLOC_093848	ENSRNOT00000021944	-0.973720983	9.44E-08
TUCP_002249	XLOC_093848	ENSRNOT00000017224	0.958450527	9.09E-07
TUCP_002249	XLOC_093848	ENSRNOT00000049725	0.952586281	1.74E-06
TUCP_002249	XLOC_093848	ENSRNOT00000087346	-0.968046701	2.49E-07
TUCP_002249	XLOC_093848	ENSRNOT00000077231	-0.957255881	1.05E-06
TUCP_002249	XLOC_093848	ENSRNOT00000059181	-0.966621806	3.08E-07

TUCP_002249	XLOC_093848	ENSRNOT00000003691	0.961457181	6.28E-07
TUCP_002249	XLOC_093848	ENSRNOT00000003690	-0.959498769	8.02E-07
TUCP_002249	XLOC_093848	ENSRNOT00000022389	0.956121116	1.19E-06
TUCP_002249	XLOC_093848	ENSRNOT00000064849	0.951633449	1.92E-06
TUCP_002249	XLOC_093848	ENSRNOT00000008901	0.977930984	3.97E-08
TUCP_002249	XLOC_093848	ENSRNOT00000025887	0.983838385	8.45E-09
TUCP_002249	XLOC_093848	ENSRNOT00000023017	0.961943006	5.90E-07
TUCP_002249	XLOC_093848	ENSRNOT00000080608	-0.960735287	6.88E-07
TUCP_002249	XLOC_093848	ENSRNOT00000017486	-0.967695052	2.62E-07
TUCP_002249	XLOC_093848	ENSRNOT00000017340	0.954307359	1.45E-06
TUCP_002249	XLOC_093848	ENSRNOT00000024011	-0.953009679	1.67E-06
TUCP_002249	XLOC_093848	ENSRNOT00000079304	0.95981492	7.71E-07
TUCP_002249	XLOC_093848	ENSRNOT00000085519	-0.961375942	6.34E-07
TUCP_002249	XLOC_093848	ENSRNOT00000012625	0.96659951	3.10E-07
TUCP_002249	XLOC_093848	ENSRNOT00000080677	0.95965735	7.86E-07
TUCP_002249	XLOC_093848	ENSRNOT00000025460	-0.95512362	1.33E-06
TUCP_002249	XLOC_093848	ENSRNOT00000025784	0.969155424	2.09E-07
TUCP_002249	XLOC_093848	ENSRNOT00000026760	0.95042081	2.17E-06
TUCP_002249	XLOC_093848	ENSRNOT00000005705	0.956288513	1.17E-06
TUCP_002249	XLOC_093848	ENSRNOT00000024491	0.960755369	6.86E-07
TUCP_002249	XLOC_093848	ENSRNOT00000037588	0.968494771	2.32E-07
TUCP_002249	XLOC_093848	ENSRNOT00000027360	0.961245913	6.45E-07
TUCP_002249	XLOC_093848	ENSRNOT00000023383	0.954385263	1.44E-06
TUCP_002249	XLOC_093848	ENSRNOT00000044486	0.975216456	7.06E-08
TUCP_002249	XLOC_093848	ENSRNOT00000079414	0.970618162	1.64E-07
TUCP_002249	XLOC_093848	ENSRNOT00000079002	0.971806854	1.34E-07
TUCP_002249	XLOC_093848	ENSRNOT00000078349	0.967022998	2.91E-07
TUCP_002249	XLOC_093848	ENSRNOT00000000134	0.974602188	7.98E-08
TUCP_002249	XLOC_093848	ENSRNOT00000079996	-0.955058445	1.34E-06
TUCP_001001	XLOC_037807	ENSRNOT00000077186	0.963133645	5.04E-07
TUCP_001001	XLOC_037807	ENSRNOT00000073521	0.96655583	3.12E-07
TUCP_001001	XLOC_037807	ENSRNOT00000017340	0.956205544	1.18E-06
TUCP_001001	XLOC_037807	ENSRNOT00000060757	0.95191381	1.87E-06
TUCP_001001	XLOC_037807	ENSRNOT00000011344	0.953030645	1.66E-06
TUCP_001001	XLOC_037807	ENSRNOT00000027220	0.961712211	6.08E-07
TUCP_001001	XLOC_037807	ENSRNOT00000001050	0.951572078	1.93E-06
TUCP_001001	XLOC_037807	ENSRNOT00000047572	0.951023808	2.04E-06
TUCP_001001	XLOC_037807	ENSRNOT00000009094	0.963900621	4.54E-07
TUCP_001001	XLOC_037807	ENSRNOT00000021506	0.963114882	5.05E-07
TUCP_001001	XLOC_037807	ENSRNOT00000026387	0.955731792	1.24E-06
TUCP_001001	XLOC_037807	ENSRNOT00000001360	0.95100469	2.05E-06
TUCP_001001	XLOC_037807	ENSRNOT00000072077	0.970890543	1.57E-07
TUCP_001001	XLOC_037807	ENSRNOT00000017224	0.958298511	9.26E-07
TUCP_001001	XLOC_037807	ENSRNOT00000092196	0.975924338	6.12E-08
TUCP_001001	XLOC_037807	ENSRNOT00000000527	0.950550427	2.14E-06
TUCP_001091	XLOC_041597	ENSRNOT00000072939	0.969505018	1.97E-07
TUCP_001696	XLOC_069431	ENSRNOT00000077569	0.963411628	4.86E-07
TUCP_000659	XLOC_025126	ENSRNOT00000025731	0.973868791	9.18E-08
TUCP_000659	XLOC_025126	ENSRNOT00000003414	0.958145522	9.43E-07
TUCP_000659	XLOC_025126	ENSRNOT00000025252	0.955488953	1.28E-06
TUCP_000659	XLOC_025126	ENSRNOT00000000990	0.951863832	1.88E-06

TUCP_000659	XLOC_025126	ENSRNOT00000040856	0.960837306	6.79E-07
TUCP_000659	XLOC_025126	ENSRNOT00000085151	0.958025589	9.56E-07
TUCP_000659	XLOC_025126	ENSRNOT00000014481	0.956702056	1.11E-06
TUCP_000659	XLOC_025126	ENSRNOT00000000995	0.962977392	5.15E-07
TUCP_000659	XLOC_025126	ENSRNOT00000014533	0.974635624	7.92E-08
TUCP_000659	XLOC_025126	ENSRNOT00000068354	0.972930347	1.09E-07
TUCP_000659	XLOC_025126	ENSRNOT00000051067	0.961921818	5.91E-07
TUCP_000659	XLOC_025126	ENSRNOT00000003082	0.987359484	2.49E-09
TUCP_000659	XLOC_025126	ENSRNOT00000045993	0.956239658	1.17E-06
TUCP_000659	XLOC_025126	ENSRNOT00000044751	0.983502034	9.36E-09
TUCP_000659	XLOC_025126	ENSRNOT00000031109	0.967703278	2.62E-07
TUCP_000659	XLOC_025126	ENSRNOT00000012963	0.969186139	2.08E-07
TUCP_000659	XLOC_025126	ENSRNOT00000050350	0.952012721	1.85E-06
TUCP_000659	XLOC_025126	ENSRNOT00000010968	0.980230706	2.30E-08
TUCP_000659	XLOC_025126	ENSRNOT00000091476	0.950362074	2.18E-06
TUCP_000659	XLOC_025126	ENSRNOT00000009448	0.979642718	2.66E-08
TUCP_000659	XLOC_025126	ENSRNOT00000000110	0.968014879	2.50E-07
TUCP_000659	XLOC_025126	ENSRNOT00000009136	0.9591784	8.34E-07
TUCP_000659	XLOC_025126	ENSRNOT00000089457	0.966307061	3.23E-07
TUCP_000659	XLOC_025126	ENSRNOT00000023067	0.969292974	2.04E-07
TUCP_000659	XLOC_025126	ENSRNOT00000042973	0.963580215	4.75E-07
TUCP_000659	XLOC_025126	ENSRNOT00000003458	0.980820658	1.98E-08
TUCP_000659	XLOC_025126	ENSRNOT00000042117	0.979344625	2.86E-08
TUCP_000659	XLOC_025126	ENSRNOT00000001050	0.951607694	1.93E-06
TUCP_000659	XLOC_025126	ENSRNOT00000063849	0.959968115	7.57E-07
TUCP_000659	XLOC_025126	ENSRNOT00000050063	0.96553276	3.62E-07
TUCP_000659	XLOC_025126	ENSRNOT00000000256	0.974560652	8.04E-08
TUCP_000659	XLOC_025126	ENSRNOT00000082497	0.966462162	3.16E-07
TUCP_000659	XLOC_025126	ENSRNOT00000003075	0.984979349	5.87E-09
TUCP_000659	XLOC_025126	ENSRNOT00000008813	0.973429863	9.97E-08
TUCP_000659	XLOC_025126	ENSRNOT00000074629	0.963911781	4.54E-07
TUCP_000659	XLOC_025126	ENSRNOT00000093046	0.964487706	4.19E-07
TUCP_000659	XLOC_025126	ENSRNOT00000003343	0.951374075	1.97E-06
TUCP_000659	XLOC_025126	ENSRNOT00000000637	0.96220059	5.70E-07
TUCP_000659	XLOC_025126	ENSRNOT00000019389	0.962818094	5.26E-07
TUCP_000659	XLOC_025126	ENSRNOT00000028527	0.958331272	9.22E-07
TUCP_000659	XLOC_025126	ENSRNOT00000083004	0.952243699	1.81E-06
TUCP_000659	XLOC_025126	ENSRNOT00000057504	0.958377531	9.17E-07
TUCP_000659	XLOC_025126	ENSRNOT00000004965	0.98477538	6.28E-09
TUCP_000659	XLOC_025126	ENSRNOT00000011369	0.963256416	4.96E-07
TUCP_000659	XLOC_025126	ENSRNOT00000082393	0.953748237	1.54E-06
TUCP_000659	XLOC_025126	ENSRNOT00000071612	0.966169093	3.30E-07
TUCP_000659	XLOC_025126	ENSRNOT00000039215	0.961339176	6.37E-07
TUCP_000659	XLOC_025126	ENSRNOT00000006196	0.979942074	2.47E-08
TUCP_000659	XLOC_025126	ENSRNOT00000073388	0.957173634	1.06E-06
TUCP_000659	XLOC_025126	ENSRNOT00000073138	0.971163003	1.50E-07
TUCP_000659	XLOC_025126	ENSRNOT00000045357	0.961532831	6.22E-07
TUCP_000659	XLOC_025126	ENSRNOT00000009260	0.954097901	1.49E-06
TUCP_000659	XLOC_025126	ENSRNOT00000014560	0.954186124	1.47E-06
TUCP_000659	XLOC_025126	ENSRNOT00000040116	0.981271366	1.76E-08
TUCP_000659	XLOC_025126	ENSRNOT00000022128	0.957949441	9.65E-07

TUCP_000659	XLOC_025126	ENSRNOT00000013191	0.956482446	1.14E-06
TUCP_000659	XLOC_025126	ENSRNOT00000023483	0.964624196	4.11E-07
TUCP_000659	XLOC_025126	ENSRNOT00000014317	0.965321186	3.73E-07
TUCP_000659	XLOC_025126	ENSRNOT00000050415	0.952390031	1.78E-06
TUCP_000659	XLOC_025126	ENSRNOT00000066776	0.967254275	2.81E-07
TUCP_001695	XLOC_069431	ENSRNOT00000006452	0.952109514	1.83E-06
TUCP_001695	XLOC_069431	ENSRNOT00000008525	0.96133888	6.37E-07
TUCP_001692	XLOC_069370	ENSRNOT00000027520	0.968400865	2.35E-07
TUCP_001692	XLOC_069370	ENSRNOT00000064840	0.955861478	1.22E-06
TUCP_001692	XLOC_069370	ENSRNOT00000004698	0.957034905	1.07E-06
TUCP_001692	XLOC_069370	ENSRNOT00000074939	0.961235787	6.46E-07
TUCP_001692	XLOC_069370	ENSRNOT00000014887	0.960365183	7.21E-07
TUCP_001692	XLOC_069370	ENSRNOT00000010218	0.954737071	1.39E-06
TUCP_001692	XLOC_069370	ENSRNOT00000087371	0.959786381	7.74E-07
TUCP_001692	XLOC_069370	ENSRNOT00000039344	0.985304863	5.27E-09
TUCP_001692	XLOC_069370	ENSRNOT00000078175	0.958374157	9.18E-07
TUCP_001692	XLOC_069370	ENSRNOT00000071694	0.959818307	7.71E-07
TUCP_001692	XLOC_069370	ENSRNOT00000068322	0.959047611	8.47E-07
TUCP_001692	XLOC_069370	ENSRNOT00000027985	-0.95380831	1.53E-06
TUCP_001692	XLOC_069370	ENSRNOT00000023180	0.969309444	2.04E-07
TUCP_001692	XLOC_069370	ENSRNOT00000028070	0.951925872	1.87E-06
TUCP_001692	XLOC_069370	ENSRNOT00000020168	0.958923783	8.59E-07
TUCP_001692	XLOC_069370	ENSRNOT00000024941	0.961516011	6.23E-07
TUCP_001692	XLOC_069370	ENSRNOT00000044742	0.951583349	1.93E-06
TUCP_001692	XLOC_069370	ENSRNOT00000026457	0.956835503	1.10E-06
TUCP_001692	XLOC_069370	ENSRNOT00000023428	0.953232311	1.63E-06
TUCP_001692	XLOC_069370	ENSRNOT00000071764	0.951519422	1.94E-06
TUCP_001692	XLOC_069370	ENSRNOT00000001809	0.950941877	2.06E-06
TUCP_001692	XLOC_069370	ENSRNOT00000024548	0.954679807	1.40E-06
TUCP_000662	XLOC_025214	ENSRNOT00000028484	-0.952984406	1.67E-06
TUCP_000662	XLOC_025214	ENSRNOT00000065673	-0.963521548	4.78E-07
TUCP_000662	XLOC_025214	ENSRNOT00000035628	-0.961045528	6.62E-07
TUCP_000662	XLOC_025214	ENSRNOT00000000576	-0.950199308	2.22E-06
TUCP_000662	XLOC_025214	ENSRNOT00000045950	-0.957346422	1.03E-06
TUCP_000662	XLOC_025214	ENSRNOT00000087113	-0.950352774	2.18E-06
TUCP_000662	XLOC_025214	ENSRNOT00000079962	0.958418439	9.13E-07
TUCP_000662	XLOC_025214	ENSRNOT00000018711	-0.954475509	1.43E-06
TUCP_000662	XLOC_025214	ENSRNOT00000018864	-0.963543065	4.77E-07
TUCP_000662	XLOC_025214	ENSRNOT00000036338	-0.970892335	1.57E-07
TUCP_000662	XLOC_025214	ENSRNOT00000075493	-0.953885802	1.52E-06
TUCP_000662	XLOC_025214	ENSRNOT00000020932	-0.952781044	1.71E-06
TUCP_000662	XLOC_025214	ENSRNOT00000022377	-0.954394303	1.44E-06
TUCP_000662	XLOC_025214	ENSRNOT00000021708	-0.95628716	1.17E-06
TUCP_000662	XLOC_025214	ENSRNOT00000010602	-0.960520527	7.07E-07
TUCP_000662	XLOC_025214	ENSRNOT00000002040	-0.950172547	2.22E-06
TUCP_000662	XLOC_025214	ENSRNOT00000011339	-0.957242568	1.05E-06
TUCP_000662	XLOC_025214	ENSRNOT00000084563	-0.976231041	5.74E-08
TUCP_000662	XLOC_025214	ENSRNOT00000032289	-0.95582366	1.23E-06
TUCP_000662	XLOC_025214	ENSRNOT00000028780	-0.966248658	3.26E-07
TUCP_000662	XLOC_025214	ENSRNOT00000039850	-0.964976119	3.91E-07
TUCP_000662	XLOC_025214	ENSRNOT00000085311	-0.956833594	1.10E-06

TUCP_000662	XLOC_025214	ENSRNOT00000015199	-0.963058725	5.09E-07
TUCP_000662	XLOC_025214	ENSRNOT00000003540	-0.952401343	1.78E-06
TUCP_000662	XLOC_025214	ENSRNOT00000026641	0.951933016	1.86E-06
TUCP_000662	XLOC_025214	ENSRNOT00000014585	-0.950614211	2.13E-06
TUCP_000662	XLOC_025214	ENSRNOT00000016541	-0.968483189	2.32E-07
TUCP_000662	XLOC_025214	ENSRNOT00000022620	0.950752549	2.10E-06
TUCP_000662	XLOC_025214	ENSRNOT00000028703	-0.957775218	9.85E-07
TUCP_000662	XLOC_025214	ENSRNOT00000021893	-0.96594692	3.41E-07
TUCP_000662	XLOC_025214	ENSRNOT00000002151	-0.952108084	1.83E-06
TUCP_000662	XLOC_025214	ENSRNOT00000021636	-0.959700261	7.82E-07
TUCP_000662	XLOC_025214	ENSRNOT00000022241	-0.957318628	1.04E-06
TUCP_000662	XLOC_025214	ENSRNOT00000022107	-0.961110934	6.56E-07
TUCP_000662	XLOC_025214	ENSRNOT00000023914	0.954724693	1.39E-06
TUCP_000662	XLOC_025214	ENSRNOT00000054966	-0.956555881	1.13E-06
TUCP_000662	XLOC_025214	ENSRNOT00000039296	0.95386234	1.52E-06
TUCP_000662	XLOC_025214	ENSRNOT00000059599	-0.96549451	3.64E-07
TUCP_001758	XLOC_072863	ENSRNOT00000014041	0.978732934	3.31E-08
TUCP_001757	XLOC_072771	ENSRNOT00000031205	0.950035684	2.25E-06
TUCP_001757	XLOC_072771	ENSRNOT00000040509	0.981145527	1.82E-08
TUCP_001757	XLOC_072771	ENSRNOT00000011342	0.965083522	3.85E-07
TUCP_001757	XLOC_072771	ENSRNOT00000088381	0.95895898	8.56E-07
TUCP_001757	XLOC_072771	ENSRNOT00000093579	0.974938715	7.47E-08
TUCP_001757	XLOC_072771	ENSRNOT00000015889	0.964131899	4.40E-07
TUCP_001757	XLOC_072771	ENSRNOT00000076466	0.953558673	1.57E-06
TUCP_001757	XLOC_072771	ENSRNOT00000085943	0.9604699	7.11E-07
TUCP_001757	XLOC_072771	ENSRNOT00000012170	0.956623286	1.12E-06
TUCP_001757	XLOC_072771	ENSRNOT00000046585	0.964533405	4.16E-07
TUCP_003888	XLOC_170952	ENSRNOT00000005584	0.967767685	2.60E-07
TUCP_001508	XLOC_058020	ENSRNOT00000091652	0.958567915	8.97E-07
TUCP_001508	XLOC_058020	ENSRNOT00000075569	0.968689391	2.25E-07
TUCP_001508	XLOC_058020	ENSRNOT00000080865	0.964997401	3.90E-07
TUCP_001508	XLOC_058020	ENSRNOT00000073534	0.957437059	1.02E-06
TUCP_001508	XLOC_058020	ENSRNOT00000093019	0.964997401	3.90E-07
TUCP_001508	XLOC_058020	ENSRNOT00000024825	0.956669129	1.12E-06
TUCP_001508	XLOC_058020	ENSRNOT00000085908	0.962566994	5.44E-07
TUCP_001508	XLOC_058020	ENSRNOT00000046744	0.964997401	3.90E-07
TUCP_001508	XLOC_058020	ENSRNOT00000076429	0.964997401	3.90E-07
TUCP_001508	XLOC_058020	ENSRNOT00000084667	0.963755375	4.64E-07
TUCP_001508	XLOC_058020	ENSRNOT00000055412	0.964997401	3.90E-07
TUCP_001508	XLOC_058020	ENSRNOT00000093201	0.965732139	3.51E-07
TUCP_001508	XLOC_058020	ENSRNOT00000072724	0.964997401	3.90E-07
TUCP_001508	XLOC_058020	ENSRNOT00000066017	0.968723133	2.24E-07
TUCP_001508	XLOC_058020	ENSRNOT00000073148	0.983495241	9.38E-09
TUCP_001508	XLOC_058020	ENSRNOT00000077462	0.958789407	8.73E-07
TUCP_001508	XLOC_058020	ENSRNOT00000084375	0.964997401	3.90E-07
TUCP_001508	XLOC_058020	ENSRNOT00000009061	0.964970822	3.92E-07
TUCP_001508	XLOC_058020	ENSRNOT00000042369	0.968610247	2.28E-07
TUCP_001508	XLOC_058020	ENSRNOT00000086554	0.964997401	3.90E-07
TUCP_001508	XLOC_058020	ENSRNOT00000047921	0.964997401	3.90E-07
TUCP_001508	XLOC_058020	ENSRNOT00000064261	0.9650602	3.87E-07
TUCP_001508	XLOC_058020	ENSRNOT00000086660	0.965624291	3.57E-07

TUCP_001508	XLOC_058020	ENSRNOT00000080933	0.964964564	3.92E-07
TUCP_001508	XLOC_058020	ENSRNOT00000083977	0.964997401	3.90E-07
TUCP_001508	XLOC_058020	ENSRNOT00000092933	0.964997401	3.90E-07
TUCP_001508	XLOC_058020	ENSRNOT00000085447	0.960329887	7.24E-07
TUCP_001754	XLOC_072675	ENSRNOT00000024186	0.980359789	2.23E-08
TUCP_001754	XLOC_072675	ENSRNOT00000074753	0.99694144	2.10E-12
TUCP_001754	XLOC_072675	ENSRNOT00000004876	0.954570781	1.41E-06
TUCP_001754	XLOC_072675	ENSRNOT00000041904	0.997505277	7.58E-13
TUCP_001754	XLOC_072675	ENSRNOT00000083487	0.982686696	1.19E-08
TUCP_001754	XLOC_072675	ENSRNOT00000077990	0.997505277	7.58E-13
TUCP_001754	XLOC_072675	ENSRNOT00000038826	0.997050257	1.75E-12
TUCP_001754	XLOC_072675	ENSRNOT00000037094	0.96125155	6.45E-07
TUCP_001754	XLOC_072675	ENSRNOT00000046842	0.969605339	1.94E-07
TUCP_001754	XLOC_072675	ENSRNOT00000037169	0.976994931	4.88E-08
TUCP_001754	XLOC_072675	ENSRNOT00000021243	0.983846097	8.43E-09
TUCP_001754	XLOC_072675	ENSRNOT00000060250	0.998251224	1.28E-13
TUCP_001754	XLOC_072675	ENSRNOT00000091934	0.997505277	7.58E-13
TUCP_001754	XLOC_072675	ENSRNOT00000086027	0.958590417	8.94E-07
TUCP_001754	XLOC_072675	ENSRNOT00000046585	0.97905047	3.07E-08
TUCP_001754	XLOC_072675	ENSRNOT00000080794	0.986754577	3.14E-09
TUCP_001754	XLOC_072675	ENSRNOT00000014456	0.988315364	1.68E-09
TUCP_001754	XLOC_072675	ENSRNOT00000031205	0.987614056	2.25E-09
TUCP_001754	XLOC_072675	ENSRNOT00000044145	0.997505277	7.58E-13
TUCP_001754	XLOC_072675	ENSRNOT00000088381	0.985502425	4.92E-09
TUCP_001754	XLOC_072675	ENSRNOT00000011743	0.983178649	1.03E-08
TUCP_001754	XLOC_072675	ENSRNOT00000089929	0.997505277	7.58E-13
TUCP_001754	XLOC_072675	ENSRNOT00000088624	0.997505277	7.58E-13
TUCP_001754	XLOC_072675	ENSRNOT00000085659	0.986438735	3.53E-09
TUCP_001754	XLOC_072675	ENSRNOT00000015866	0.989664257	9.13E-10
TUCP_001754	XLOC_072675	ENSRNOT00000078633	0.997505277	7.58E-13
TUCP_001754	XLOC_072675	ENSRNOT00000034907	0.98946022	1.01E-09
TUCP_001754	XLOC_072675	ENSRNOT00000093230	0.951738643	1.90E-06
TUCP_001754	XLOC_072675	ENSRNOT00000051835	0.997505277	7.58E-13
TUCP_001754	XLOC_072675	ENSRNOT00000079087	0.997505277	7.58E-13
TUCP_001754	XLOC_072675	ENSRNOT00000037206	0.997505277	7.58E-13
TUCP_001754	XLOC_072675	ENSRNOT00000083261	0.973232557	1.03E-07
TUCP_001754	XLOC_072675	ENSRNOT00000080590	0.997289696	1.15E-12
TUCP_001754	XLOC_072675	ENSRNOT00000027552	0.997982197	2.63E-13
TUCP_001754	XLOC_072675	ENSRNOT00000077014	0.998367197	9.11E-14
TUCP_001754	XLOC_072675	ENSRNOT00000080453	0.997632769	5.83E-13
TUCP_001754	XLOC_072675	ENSRNOT00000089057	0.957841579	9.77E-07
TUCP_001754	XLOC_072675	ENSRNOT00000066839	0.988299753	1.69E-09
TUCP_001754	XLOC_072675	ENSRNOT00000061284	0.959795753	7.73E-07
TUCP_001754	XLOC_072675	ENSRNOT00000020206	0.95204606	1.84E-06
TUCP_001754	XLOC_072675	ENSRNOT00000013423	0.958037586	9.55E-07
TUCP_001754	XLOC_072675	ENSRNOT00000071884	0.970015549	1.82E-07
TUCP_001754	XLOC_072675	ENSRNOT00000004128	0.952537834	1.75E-06
TUCP_001754	XLOC_072675	ENSRNOT00000026316	0.960383694	7.19E-07
TUCP_001754	XLOC_072675	ENSRNOT00000057729	0.996643296	3.34E-12
TUCP_001754	XLOC_072675	ENSRNOT00000078663	0.980380211	2.22E-08
TUCP_001754	XLOC_072675	ENSRNOT00000064250	0.987607449	2.25E-09

TUCP_001754	XLOC_072675	ENSRNOT00000021608	0.969846859	1.87E-07
TUCP_001754	XLOC_072675	ENSRNOT00000029634	0.992514336	1.83E-10
TUCP_001754	XLOC_072675	ENSRNOT00000093579	0.952428226	1.77E-06
TUCP_001754	XLOC_072675	ENSRNOT00000086349	0.950204432	2.22E-06
TUCP_001754	XLOC_072675	ENSRNOT00000065460	0.997505277	7.58E-13
TUCP_001754	XLOC_072675	ENSRNOT00000003817	0.998449398	7.04E-14
TUCP_001754	XLOC_072675	ENSRNOT00000016713	0.997505277	7.58E-13
TUCP_001754	XLOC_072675	ENSRNOT00000079260	0.992826177	1.48E-10
TUCP_001754	XLOC_072675	ENSRNOT00000039294	0.997505277	7.58E-13
TUCP_001754	XLOC_072675	ENSRNOT00000092913	0.954776031	1.38E-06
TUCP_001752	XLOC_072642	ENSRNOT00000018970	0.959730361	7.79E-07
TUCP_001752	XLOC_072642	ENSRNOT00000045493	0.961102905	6.57E-07
TUCP_001752	XLOC_072642	ENSRNOT00000000697	0.952309783	1.79E-06
TUCP_001752	XLOC_072642	ENSRNOT00000023623	0.950249423	2.21E-06
TUCP_001752	XLOC_072642	ENSRNOT00000028588	0.972812595	1.12E-07
TUCP_001752	XLOC_072642	ENSRNOT00000024583	-0.971002694	1.54E-07
TUCP_001752	XLOC_072642	ENSRNOT00000023661	0.956625706	1.12E-06
TUCP_001752	XLOC_072642	ENSRNOT00000074810	0.962040115	5.82E-07
TUCP_001752	XLOC_072642	ENSRNOT00000086027	0.957585062	1.01E-06
TUCP_001752	XLOC_072642	ENSRNOT00000024575	0.957151613	1.06E-06
TUCP_001752	XLOC_072642	ENSRNOT00000091315	0.957017171	1.07E-06
TUCP_001698	XLOC_069431	ENSRNOT00000090572	0.954857959	1.37E-06
TUCP_000508	XLOC_017500	ENSRNOT00000000117	0.953278232	1.62E-06
TUCP_000508	XLOC_017500	ENSRNOT00000065194	0.979658752	2.65E-08
TUCP_000508	XLOC_017500	ENSRNOT00000054939	0.988530498	1.53E-09
TUCP_000508	XLOC_017500	ENSRNOT00000066391	0.990550364	5.84E-10
TUCP_000508	XLOC_017500	ENSRNOT00000066821	0.97042276	1.70E-07
TUCP_000508	XLOC_017500	ENSRNOT00000072802	0.967250486	2.81E-07
TUCP_001052	XLOC_040163	ENSRNOT00000009975	0.954960224	1.35E-06
TUCP_001052	XLOC_040163	ENSRNOT00000072682	0.954330739	1.45E-06
TUCP_001052	XLOC_040163	ENSRNOT00000024583	-0.951350589	1.98E-06
TUCP_001052	XLOC_040163	ENSRNOT00000001642	0.953009576	1.67E-06
TUCP_001052	XLOC_040163	ENSRNOT00000027752	0.959364863	8.15E-07
TUCP_001052	XLOC_040163	ENSRNOT00000007601	0.953962852	1.51E-06
TUCP_001052	XLOC_040163	ENSRNOT00000004764	0.952750497	1.71E-06
TUCP_001052	XLOC_040163	ENSRNOT00000065801	0.974787511	7.69E-08
TUCP_001052	XLOC_040163	ENSRNOT00000010142	0.953611329	1.56E-06
TUCP_001052	XLOC_040163	ENSRNOT00000015864	0.975176128	7.12E-08
TUCP_001052	XLOC_040163	ENSRNOT00000038449	0.952968549	1.67E-06
TUCP_001052	XLOC_040163	ENSRNOT00000079152	0.975943093	6.09E-08
TUCP_001052	XLOC_040163	ENSRNOT00000017282	0.96084334	6.79E-07
TUCP_001052	XLOC_040163	ENSRNOT00000013088	0.955394192	1.29E-06
TUCP_001052	XLOC_040163	ENSRNOT00000040904	0.952393317	1.78E-06
TUCP_001052	XLOC_040163	ENSRNOT00000023890	-0.95519644	1.32E-06
TUCP_001052	XLOC_040163	ENSRNOT00000085334	0.95290976	1.68E-06
TUCP_001052	XLOC_040163	ENSRNOT00000043827	0.957703323	9.93E-07
TUCP_001052	XLOC_040163	ENSRNOT00000093008	0.954755663	1.38E-06
TUCP_001052	XLOC_040163	ENSRNOT00000012757	0.972087092	1.27E-07
TUCP_001052	XLOC_040163	ENSRNOT00000035427	0.9570476	1.07E-06
TUCP_001052	XLOC_040163	ENSRNOT00000012247	0.953165506	1.64E-06
TUCP_001052	XLOC_040163	ENSRNOT00000023623	0.951941022	1.86E-06

TUCP_001052	XLOC_040163	ENSRNOT00000023607	0.960558892	7.03E-07
TUCP_001052	XLOC_040163	ENSRNOT00000006109	0.969561847	1.96E-07
TUCP_001052	XLOC_040163	ENSRNOT00000002814	0.978278016	3.67E-08
TUCP_001052	XLOC_040163	ENSRNOT00000065811	0.967832926	2.57E-07
TUCP_001052	XLOC_040163	ENSRNOT00000074131	0.966374318	3.20E-07
TUCP_001052	XLOC_040163	ENSRNOT00000023661	0.954196773	1.47E-06
TUCP_000058	XLOC_001127	ENSRNOT00000064764	0.965972273	3.39E-07
TUCP_000116	XLOC_002765	ENSRNOT00000071840	0.979971774	2.45E-08
TUCP_000116	XLOC_002765	ENSRNOT00000083608	0.981365397	1.72E-08
TUCP_000116	XLOC_002765	ENSRNOT00000076035	0.958670301	8.86E-07
TUCP_000116	XLOC_002765	ENSRNOT00000045584	0.979971774	2.45E-08
TUCP_000116	XLOC_002765	ENSRNOT00000077995	0.969724167	1.90E-07
TUCP_000116	XLOC_002765	ENSRNOT00000066004	0.979018084	3.09E-08
TUCP_000116	XLOC_002765	ENSRNOT00000029197	0.970036053	1.81E-07
TUCP_000116	XLOC_002765	ENSRNOT00000066685	0.973839701	9.23E-08
TUCP_000116	XLOC_002765	ENSRNOT00000089494	0.965908346	3.42E-07
TUCP_000116	XLOC_002765	ENSRNOT00000092182	0.979971774	2.45E-08
TUCP_000116	XLOC_002765	ENSRNOT00000084461	0.979971774	2.45E-08
TUCP_000116	XLOC_002765	ENSRNOT00000060448	0.979971774	2.45E-08
TUCP_000116	XLOC_002765	ENSRNOT00000032631	0.979971774	2.45E-08
TUCP_000116	XLOC_002765	ENSRNOT00000054910	0.958571821	8.96E-07
TUCP_000116	XLOC_002765	ENSRNOT00000057176	0.980225444	2.30E-08
TUCP_000116	XLOC_002765	ENSRNOT00000078176	0.970933853	1.56E-07
TUCP_000116	XLOC_002765	ENSRNOT00000056364	0.965492268	3.64E-07
TUCP_000116	XLOC_002765	ENSRNOT00000093431	0.979971774	2.45E-08
TUCP_000116	XLOC_002765	ENSRNOT00000024758	0.955686893	1.25E-06
TUCP_000116	XLOC_002765	ENSRNOT00000092901	0.979971774	2.45E-08
TUCP_000116	XLOC_002765	ENSRNOT00000089290	0.980695834	2.04E-08
TUCP_000116	XLOC_002765	ENSRNOT00000090211	0.977927355	3.98E-08
TUCP_000116	XLOC_002765	ENSRNOT00000091828	0.976012671	6.01E-08
TUCP_000116	XLOC_002765	ENSRNOT00000091944	0.979901325	2.50E-08
TUCP_000116	XLOC_002765	ENSRNOT00000070855	0.979971774	2.45E-08
TUCP_000116	XLOC_002765	ENSRNOT00000046980	0.956870174	1.09E-06
TUCP_000116	XLOC_002765	ENSRNOT00000084109	0.981094273	1.84E-08
TUCP_000116	XLOC_002765	ENSRNOT00000060645	0.954081657	1.49E-06
TUCP_000116	XLOC_002765	ENSRNOT00000041569	0.979971774	2.45E-08
TUCP_000116	XLOC_002765	ENSRNOT00000087091	0.979971774	2.45E-08
TUCP_000116	XLOC_002765	ENSRNOT00000003308	0.980938433	1.92E-08
TUCP_000116	XLOC_002765	ENSRNOT00000026113	0.982862153	1.13E-08
TUCP_000116	XLOC_002765	ENSRNOT00000014939	0.979394817	2.83E-08
TUCP_000116	XLOC_002765	ENSRNOT00000058314	0.980433947	2.19E-08
TUCP_000116	XLOC_002765	ENSRNOT00000047870	0.956347386	1.16E-06
TUCP_000116	XLOC_002765	ENSRNOT00000071603	0.979872976	2.51E-08
TUCP_000116	XLOC_002765	ENSRNOT00000044232	0.961830467	5.98E-07
TUCP_000116	XLOC_002765	ENSRNOT00000007683	0.979307636	2.89E-08
TUCP_000116	XLOC_002765	ENSRNOT00000071714	0.957699564	9.93E-07
TUCP_000116	XLOC_002765	ENSRNOT00000075836	0.979971774	2.45E-08
TUCP_000116	XLOC_002765	ENSRNOT00000073229	0.979971774	2.45E-08
TUCP_000116	XLOC_002765	ENSRNOT00000060454	0.979971774	2.45E-08
TUCP_000116	XLOC_002765	ENSRNOT00000009295	0.975272955	6.98E-08
TUCP_000117	XLOC_002878	ENSRNOT00000026803	0.957078703	1.07E-06

TUCP_000117	XLOC_002878	ENSRNOT00000020786	0.955370508	1.29E-06
TUCP_000117	XLOC_002878	ENSRNOT00000050968	0.951267971	1.99E-06
TUCP_000117	XLOC_002878	ENSRNOT00000072398	0.952597414	1.74E-06
TUCP_000117	XLOC_002878	ENSRNOT00000042201	0.974910552	7.51E-08
TUCP_000117	XLOC_002878	ENSRNOT00000085875	0.955090001	1.33E-06
TUCP_001058	XLOC_040406	ENSRNOT00000003378	0.977656007	4.22E-08
TUCP_001058	XLOC_040406	ENSRNOT00000086014	0.960435123	7.14E-07
TUCP_001059	XLOC_040425	ENSRNOT00000063994	0.952221726	1.81E-06
TUCP_001059	XLOC_040425	ENSRNOT00000033017	0.961310215	6.40E-07
TUCP_001059	XLOC_040425	ENSRNOT00000012811	0.967424307	2.74E-07
TUCP_001059	XLOC_040425	ENSRNOT00000030445	0.9506689	2.12E-06
TUCP_000051	XLOC_001127	ENSRNOT00000021972	0.977779959	4.11E-08
TUCP_000057	XLOC_001127	ENSRNOT00000000008	1	0
TUCP_000057	XLOC_001127	ENSRNOT00000060048	0.998711457	2.79E-14
TUCP_000057	XLOC_001127	ENSRNOT00000082304	0.996875989	2.33E-12
TUCP_000057	XLOC_001127	ENSRNOT00000006219	1	4.25E-78
TUCP_000057	XLOC_001127	ENSRNOT00000065376	1	0
TUCP_000057	XLOC_001127	ENSRNOT00000081433	0.984469223	6.93E-09
TUCP_000057	XLOC_001127	ENSRNOT00000083575	1	0
TUCP_000057	XLOC_001127	ENSRNOT00000087739	1	1.33E-79
TUCP_000057	XLOC_001127	ENSRNOT00000079826	1	1.33E-79
TUCP_000057	XLOC_001127	ENSRNOT00000025770	0.993828251	6.98E-11
TUCP_000057	XLOC_001127	ENSRNOT00000088244	0.973479481	9.88E-08
TUCP_000057	XLOC_001127	ENSRNOT00000048523	1	0
TUCP_000057	XLOC_001127	ENSRNOT00000079365	0.958936699	8.58E-07
TUCP_000057	XLOC_001127	ENSRNOT00000020229	0.960002785	7.54E-07
TUCP_000057	XLOC_001127	ENSRNOT00000026428	0.996986683	1.95E-12
TUCP_000057	XLOC_001127	ENSRNOT00000085673	1	0
TUCP_000057	XLOC_001127	ENSRNOT00000072972	0.981476166	1.67E-08
TUCP_000057	XLOC_001127	ENSRNOT00000050573	1	1.33E-79
TUCP_000057	XLOC_001127	ENSRNOT00000089876	-0.953630353	1.56E-06
TUCP_000057	XLOC_001127	ENSRNOT00000074378	0.998901807	1.26E-14
TUCP_000057	XLOC_001127	ENSRNOT00000093571	1	0
TUCP_000057	XLOC_001127	ENSRNOT00000090581	1	0
TUCP_000057	XLOC_001127	ENSRNOT00000076239	0.98020752	2.31E-08
TUCP_000057	XLOC_001127	ENSRNOT00000065485	1	1.33E-79
TUCP_000057	XLOC_001127	ENSRNOT00000006356	0.999544279	1.55E-16
TUCP_000057	XLOC_001127	ENSRNOT00000087199	0.974421555	8.26E-08
TUCP_000057	XLOC_001127	ENSRNOT00000022091	1	0
TUCP_000057	XLOC_001127	ENSRNOT00000064764	0.98460888	6.63E-09
TUCP_000057	XLOC_001127	ENSRNOT00000076927	0.971163156	1.50E-07
TUCP_000057	XLOC_001127	ENSRNOT00000092395	0.999522978	1.94E-16
TUCP_001194	XLOC_044866	ENSRNOT00000073559	0.959666807	7.85E-07
TUCP_001194	XLOC_044866	ENSRNOT00000039809	0.951879189	1.87E-06
TUCP_001194	XLOC_044866	ENSRNOT00000081246	0.970312637	1.73E-07
TUCP_001194	XLOC_044866	ENSRNOT00000039852	0.961241029	6.45E-07
TUCP_001194	XLOC_044866	ENSRNOT00000009877	0.956753373	1.11E-06
TUCP_001194	XLOC_044866	ENSRNOT00000016555	0.986389365	3.60E-09
TUCP_001194	XLOC_044866	ENSRNOT00000021348	0.97731504	4.55E-08
TUCP_001194	XLOC_044866	ENSRNOT00000078445	-0.96202433	5.84E-07
TUCP_001194	XLOC_044866	ENSRNOT00000005228	0.951378107	1.97E-06

TUCP_001194	XLOC_044866	ENSRNOT00000006175	0.968097408	2.47E-07
TUCP_001194	XLOC_044866	ENSRNOT00000076763	0.952941525	1.68E-06
TUCP_001194	XLOC_044866	ENSRNOT00000043216	0.966462433	3.16E-07
TUCP_001194	XLOC_044866	ENSRNOT00000009129	0.962158909	5.73E-07
TUCP_001194	XLOC_044866	ENSRNOT00000026543	0.966447605	3.17E-07
TUCP_001194	XLOC_044866	ENSRNOT00000044742	0.972681347	1.14E-07
TUCP_001194	XLOC_044866	ENSRNOT00000066670	0.985722011	4.56E-09
TUCP_001194	XLOC_044866	ENSRNOT00000005942	0.950197931	2.22E-06
TUCP_001194	XLOC_044866	ENSRNOT00000012463	-0.951689651	1.91E-06
TUCP_001194	XLOC_044866	ENSRNOT00000025832	0.984340292	7.22E-09
TUCP_001194	XLOC_044866	ENSRNOT00000026783	0.951276684	1.99E-06
TUCP_001194	XLOC_044866	ENSRNOT00000012379	0.952469767	1.76E-06
TUCP_001194	XLOC_044866	ENSRNOT00000029866	0.954006472	1.50E-06
TUCP_001194	XLOC_044866	ENSRNOT00000071764	0.991083513	4.37E-10
TUCP_001194	XLOC_044866	ENSRNOT00000075326	0.969695486	1.91E-07
TUCP_001194	XLOC_044866	ENSRNOT00000014537	0.952857325	1.69E-06
TUCP_001194	XLOC_044866	ENSRNOT00000085228	0.952463646	1.76E-06
TUCP_001194	XLOC_044866	ENSRNOT00000020134	0.952118412	1.83E-06
TUCP_001194	XLOC_044866	ENSRNOT00000001809	0.965172064	3.81E-07
TUCP_001194	XLOC_044866	ENSRNOT00000087592	0.97244154	1.20E-07
TUCP_001194	XLOC_044866	ENSRNOT00000032274	0.994514892	3.87E-11
TUCP_001194	XLOC_044866	ENSRNOT00000058956	0.957390031	1.03E-06
TUCP_001194	XLOC_044866	ENSRNOT00000017948	0.963859778	4.57E-07
TUCP_001194	XLOC_044866	ENSRNOT00000012895	0.969954799	1.83E-07
TUCP_001194	XLOC_044866	ENSRNOT00000004905	0.954635994	1.40E-06
TUCP_001194	XLOC_044866	ENSRNOT00000055401	0.95875064	8.77E-07
TUCP_001194	XLOC_044866	ENSRNOT00000000468	0.957418114	1.03E-06
TUCP_001194	XLOC_044866	ENSRNOT00000072196	0.969345162	2.03E-07
TUCP_001194	XLOC_044866	ENSRNOT00000020028	0.950846496	2.08E-06
TUCP_001194	XLOC_044866	ENSRNOT00000090918	0.955693656	1.25E-06
TUCP_001194	XLOC_044866	ENSRNOT00000023180	0.972303265	1.23E-07
TUCP_001194	XLOC_044866	ENSRNOT00000000676	0.968053673	2.48E-07
TUCP_001194	XLOC_044866	ENSRNOT00000070815	0.958665077	8.87E-07
TUCP_001194	XLOC_044866	ENSRNOT00000019222	0.952319145	1.79E-06
TUCP_001194	XLOC_044866	ENSRNOT00000028070	0.954488812	1.42E-06
TUCP_001194	XLOC_044866	ENSRNOT00000004359	0.951263326	1.99E-06
TUCP_001194	XLOC_044866	ENSRNOT00000060362	0.968783226	2.22E-07
TUCP_001194	XLOC_044866	ENSRNOT00000013274	0.972714497	1.14E-07
TUCP_001194	XLOC_044866	ENSRNOT00000022610	0.968497584	2.32E-07
TUCP_001194	XLOC_044866	ENSRNOT00000020437	0.96473009	4.05E-07
TUCP_001194	XLOC_044866	ENSRNOT00000006828	0.953845674	1.53E-06
TUCP_001194	XLOC_044866	ENSRNOT00000010898	0.967473855	2.71E-07
TUCP_001194	XLOC_044866	ENSRNOT00000005355	0.956425858	1.15E-06
TUCP_001194	XLOC_044866	ENSRNOT00000029167	0.97777899	4.11E-08
TUCP_001194	XLOC_044866	ENSRNOT00000027520	0.975670446	6.45E-08
TUCP_001194	XLOC_044866	ENSRNOT00000004698	0.960893649	6.74E-07
TUCP_001194	XLOC_044866	ENSRNOT00000091910	0.95176107	1.90E-06
TUCP_001194	XLOC_044866	ENSRNOT00000058072	0.956540164	1.13E-06
TUCP_001194	XLOC_044866	ENSRNOT00000084240	0.950207529	2.22E-06
TUCP_001194	XLOC_044866	ENSRNOT00000017629	0.953601287	1.57E-06
TUCP_001194	XLOC_044866	ENSRNOT00000017021	0.961076128	6.59E-07

TUCP_001194	XLOC_044866	ENSRNOT00000003991	0.956815329	1.10E-06
TUCP_001194	XLOC_044866	ENSRNOT00000009683	0.969297138	2.04E-07
TUCP_001194	XLOC_044866	ENSRNOT00000043535	0.952492421	1.76E-06
TUCP_001194	XLOC_044866	ENSRNOT00000064902	0.951259569	2.00E-06
TUCP_001194	XLOC_044866	ENSRNOT00000024322	0.9678289	2.57E-07
TUCP_001194	XLOC_044866	ENSRNOT00000023490	0.970495548	1.68E-07
TUCP_001194	XLOC_044866	ENSRNOT00000091265	0.97556645	6.58E-08
TUCP_001194	XLOC_044866	ENSRNOT00000089971	0.958564382	8.97E-07
TUCP_001194	XLOC_044866	ENSRNOT00000027454	0.969299908	2.04E-07
TUCP_001194	XLOC_044866	ENSRNOT00000027452	0.96800914	2.50E-07
TUCP_001194	XLOC_044866	ENSRNOT00000076729	0.954458992	1.43E-06
TUCP_001194	XLOC_044866	ENSRNOT00000017753	0.961520666	6.23E-07
TUCP_001194	XLOC_044866	ENSRNOT00000045882	0.967389437	2.75E-07
TUCP_001194	XLOC_044866	ENSRNOT00000066736	0.961232438	6.46E-07
TUCP_001194	XLOC_044866	ENSRNOT00000009280	0.96346978	4.82E-07
TUCP_001194	XLOC_044866	ENSRNOT00000072534	0.964320152	4.29E-07
TUCP_001194	XLOC_044866	ENSRNOT00000028759	0.95717546	1.06E-06
TUCP_001194	XLOC_044866	ENSRNOT00000036951	0.965130569	3.83E-07
TUCP_001194	XLOC_044866	ENSRNOT00000026457	0.959441533	8.07E-07
TUCP_001194	XLOC_044866	ENSRNOT00000000157	0.962654633	5.37E-07
TUCP_001194	XLOC_044866	ENSRNOT00000064840	0.957540546	1.01E-06
TUCP_001194	XLOC_044866	ENSRNOT00000076669	0.95907818	8.44E-07
TUCP_001194	XLOC_044866	ENSRNOT00000085596	0.950048691	2.25E-06
TUCP_001194	XLOC_044866	ENSRNOT00000031230	0.972849043	1.11E-07
TUCP_001194	XLOC_044866	ENSRNOT00000071733	0.96019697	7.36E-07
TUCP_001194	XLOC_044866	ENSRNOT00000002292	0.953665542	1.56E-06
TUCP_001194	XLOC_044866	ENSRNOT00000086336	0.954058518	1.49E-06
TUCP_001194	XLOC_044866	ENSRNOT00000074939	0.981808754	1.52E-08
TUCP_001194	XLOC_044866	ENSRNOT00000076872	0.952560718	1.75E-06
TUCP_001194	XLOC_044866	ENSRNOT00000071694	0.953672734	1.55E-06
TUCP_001194	XLOC_044866	ENSRNOT00000041384	-0.9777716	4.12E-08
TUCP_001194	XLOC_044866	ENSRNOT00000004412	0.968994116	2.14E-07
TUCP_001194	XLOC_044866	ENSRNOT00000012048	0.950071375	2.25E-06
TUCP_001194	XLOC_044866	ENSRNOT00000064798	0.965333416	3.72E-07
TUCP_001194	XLOC_044866	ENSRNOT00000022485	0.974025025	8.92E-08
TUCP_001194	XLOC_044866	ENSRNOT00000033235	0.953422981	1.60E-06
TUCP_001194	XLOC_044866	ENSRNOT00000024941	0.952128032	1.83E-06
TUCP_001194	XLOC_044866	ENSRNOT00000034704	0.966704984	3.05E-07
TUCP_001194	XLOC_044866	ENSRNOT00000013985	0.951013318	2.05E-06
TUCP_001194	XLOC_044866	ENSRNOT00000002522	-0.953330603	1.61E-06
TUCP_001194	XLOC_044866	ENSRNOT00000036750	0.95556176	1.27E-06
TUCP_001194	XLOC_044866	ENSRNOT00000023428	0.985078487	5.68E-09
TUCP_001194	XLOC_044866	ENSRNOT00000024420	0.953741142	1.54E-06
TUCP_001194	XLOC_044866	ENSRNOT00000049538	0.955762389	1.24E-06
TUCP_001194	XLOC_044866	ENSRNOT00000083799	0.969762772	1.89E-07
TUCP_001194	XLOC_044866	ENSRNOT00000009066	0.96755948	2.68E-07
TUCP_000055	XLOC_001127	ENSRNOT00000076978	0.959129491	8.38E-07
TUCP_000055	XLOC_001127	ENSRNOT00000050345	0.964594009	4.13E-07
TUCP_003441	XLOC_148563	ENSRNOT00000002308	0.954868777	1.37E-06
TUCP_003441	XLOC_148563	ENSRNOT00000085465	0.966080536	3.34E-07
TUCP_003440	XLOC_148551	ENSRNOT00000061303	0.970823816	1.59E-07

TUCP_003568	XLOC_156604	ENSRNOT00000061569	0.95009599	2.24E-06
TUCP_003569	XLOC_156680	ENSRNOT00000086061	0.951104736	2.03E-06
TUCP_003569	XLOC_156680	ENSRNOT00000076294	0.964968431	3.92E-07
TUCP_003569	XLOC_156680	ENSRNOT00000078157	0.958064648	9.52E-07
TUCP_003569	XLOC_156680	ENSRNOT00000040840	0.965447064	3.66E-07
TUCP_003569	XLOC_156680	ENSRNOT00000083263	0.960044891	7.50E-07
TUCP_001573	XLOC_060823	ENSRNOT00000045926	0.950173389	2.22E-06
TUCP_001573	XLOC_060823	ENSRNOT00000079548	-0.954126795	1.48E-06
TUCP_001573	XLOC_060823	ENSRNOT00000011032	0.957804358	9.81E-07
TUCP_001573	XLOC_060823	ENSRNOT00000019278	0.95131482	1.98E-06
TUCP_001573	XLOC_060823	ENSRNOT00000065600	0.951290437	1.99E-06
TUCP_001573	XLOC_060823	ENSRNOT00000079742	0.951412421	1.97E-06
TUCP_001573	XLOC_060823	ENSRNOT00000034300	0.956802575	1.10E-06
TUCP_003444	XLOC_148684	ENSRNOT00000006597	0.956250467	1.17E-06
TUCP_003444	XLOC_148684	ENSRNOT00000001912	0.952608643	1.74E-06
TUCP_001571	XLOC_060667	ENSRNOT00000067846	-0.952493491	1.76E-06
TUCP_001571	XLOC_060667	ENSRNOT00000078274	0.950357803	2.18E-06
TUCP_001571	XLOC_060667	ENSRNOT00000004046	-0.950219088	2.21E-06
TUCP_001571	XLOC_060667	ENSRNOT00000049819	0.953216435	1.63E-06
TUCP_001571	XLOC_060667	ENSRNOT00000014814	0.954903478	1.36E-06
TUCP_003446	XLOC_148940	ENSRNOT00000071840	1	0
TUCP_003446	XLOC_148940	ENSRNOT00000083608	0.992618428	1.70E-10
TUCP_003446	XLOC_148940	ENSRNOT00000076035	0.956476518	1.14E-06
TUCP_003446	XLOC_148940	ENSRNOT00000085476	0.96207118	5.80E-07
TUCP_003446	XLOC_148940	ENSRNOT00000066382	0.96329105	4.94E-07
TUCP_003446	XLOC_148940	ENSRNOT00000045584	1	0
TUCP_003446	XLOC_148940	ENSRNOT00000077995	0.994428404	4.19E-11
TUCP_003446	XLOC_148940	ENSRNOT00000066004	0.999857217	4.67E-19
TUCP_003446	XLOC_148940	ENSRNOT00000064284	0.968782027	2.22E-07
TUCP_003446	XLOC_148940	ENSRNOT00000068197	0.955470548	1.28E-06
TUCP_003446	XLOC_148940	ENSRNOT00000082086	0.97275676	1.13E-07
TUCP_003446	XLOC_148940	ENSRNOT00000089494	0.983505294	9.35E-09
TUCP_003446	XLOC_148940	ENSRNOT00000083747	0.957764469	9.86E-07
TUCP_003446	XLOC_148940	ENSRNOT00000092182	1	0
TUCP_003446	XLOC_148940	ENSRNOT00000084461	1	0
TUCP_003446	XLOC_148940	ENSRNOT00000060448	1	0
TUCP_003446	XLOC_148940	ENSRNOT00000032631	1	0
TUCP_003446	XLOC_148940	ENSRNOT00000079503	0.957317866	1.04E-06
TUCP_003446	XLOC_148940	ENSRNOT00000027950	0.960989928	6.66E-07
TUCP_003446	XLOC_148940	ENSRNOT00000060113	0.975275164	6.98E-08
TUCP_003446	XLOC_148940	ENSRNOT00000054910	0.990580154	5.75E-10
TUCP_003446	XLOC_148940	ENSRNOT00000057176	0.999998955	9.83E-30
TUCP_003446	XLOC_148940	ENSRNOT00000078176	0.994350784	4.49E-11
TUCP_003446	XLOC_148940	ENSRNOT00000056364	0.990647125	5.55E-10
TUCP_003446	XLOC_148940	ENSRNOT00000093431	1	0
TUCP_003446	XLOC_148940	ENSRNOT00000024758	0.981367686	1.71E-08
TUCP_003446	XLOC_148940	ENSRNOT00000092901	1	0
TUCP_003446	XLOC_148940	ENSRNOT00000089290	0.999988082	1.89E-24
TUCP_003446	XLOC_148940	ENSRNOT00000090211	0.998865151	1.48E-14
TUCP_003446	XLOC_148940	ENSRNOT00000075244	0.962646865	5.38E-07
TUCP_003446	XLOC_148940	ENSRNOT00000091828	0.996371069	4.93E-12

TUCP_003446	XLOC_148940	ENSRNOT00000091944	0.999786107	3.52E-18
TUCP_003446	XLOC_148940	ENSRNOT00000070855	1	0
TUCP_003446	XLOC_148940	ENSRNOT00000009295	0.995887068	9.21E-12
TUCP_003446	XLOC_148940	ENSRNOT00000084109	0.999673655	2.91E-17
TUCP_003446	XLOC_148940	ENSRNOT00000060645	0.973934385	9.07E-08
TUCP_003446	XLOC_148940	ENSRNOT00000041569	1	0
TUCP_003446	XLOC_148940	ENSRNOT00000087091	1	0
TUCP_003446	XLOC_148940	ENSRNOT00000003308	0.990930758	4.76E-10
TUCP_003446	XLOC_148940	ENSRNOT00000026113	0.999851454	5.69E-19
TUCP_003446	XLOC_148940	ENSRNOT00000014939	0.999846854	6.63E-19
TUCP_003446	XLOC_148940	ENSRNOT00000058314	0.99088442	4.88E-10
TUCP_003446	XLOC_148940	ENSRNOT00000047870	0.981575908	1.62E-08
TUCP_003446	XLOC_148940	ENSRNOT00000071603	0.999995875	9.40E-27
TUCP_003446	XLOC_148940	ENSRNOT00000088376	0.970305372	1.73E-07
TUCP_003446	XLOC_148940	ENSRNOT00000060454	1	0
TUCP_003446	XLOC_148940	ENSRNOT00000029298	0.957304374	1.04E-06
TUCP_003446	XLOC_148940	ENSRNOT00000044232	0.98372207	8.76E-09
TUCP_003446	XLOC_148940	ENSRNOT00000023287	0.974929583	7.48E-08
TUCP_003446	XLOC_148940	ENSRNOT00000071714	0.978813661	3.24E-08
TUCP_003446	XLOC_148940	ENSRNOT00000075836	1	0
TUCP_003446	XLOC_148940	ENSRNOT00000073229	1	0
TUCP_003446	XLOC_148940	ENSRNOT00000077731	0.965942857	3.41E-07
TUCP_003446	XLOC_148940	ENSRNOT00000046980	0.982667176	1.20E-08
TUCP_003446	XLOC_148940	ENSRNOT00000039652	0.972574547	1.17E-07
TUCP_003446	XLOC_148940	ENSRNOT00000066685	0.981130123	1.83E-08
TUCP_003446	XLOC_148940	ENSRNOT00000082776	0.966379954	3.20E-07
TUCP_003446	XLOC_148940	ENSRNOT00000088719	0.965171519	3.81E-07
TUCP_003446	XLOC_148940	ENSRNOT00000007683	0.999755897	6.82E-18
TUCP_003446	XLOC_148940	ENSRNOT00000092916	0.96606874	3.35E-07
TUCP_003446	XLOC_148940	ENSRNOT00000081106	0.95354262	1.58E-06
TUCP_000541	XLOC_019107	ENSRNOT00000000036	0.957305971	1.04E-06
TUCP_000541	XLOC_019107	ENSRNOT00000091417	0.975193871	7.10E-08
TUCP_000541	XLOC_019107	ENSRNOT00000000337	0.962445469	5.52E-07
TUCP_001673	XLOC_066715	ENSRNOT00000061027	0.95523819	1.31E-06
TUCP_001673	XLOC_066715	ENSRNOT00000093441	0.95792975	9.67E-07
TUCP_001674	XLOC_066715	ENSRNOT00000063862	0.977217292	4.65E-08
TUCP_003567	XLOC_156585	ENSRNOT00000084481	0.958537698	9.00E-07
TUCP_003567	XLOC_156585	ENSRNOT00000015427	0.955620715	1.26E-06
TUCP_000669	XLOC_025214	ENSRNOT00000026893	0.973561347	9.73E-08
TUCP_001708	XLOC_069783	ENSRNOT00000000383	-0.950420446	2.17E-06
TUCP_001709	XLOC_069785	ENSRNOT00000005343	-0.962025647	5.83E-07
TUCP_001588	XLOC_061713	ENSRNOT00000013423	0.958179498	9.39E-07
TUCP_001588	XLOC_061713	ENSRNOT00000037094	0.952252062	1.80E-06
TUCP_001588	XLOC_061713	ENSRNOT00000054947	0.964832163	3.99E-07
TUCP_001588	XLOC_061713	ENSRNOT00000024337	0.972612035	1.16E-07
TUCP_001588	XLOC_061713	ENSRNOT00000046585	0.953175869	1.64E-06
TUCP_001588	XLOC_061713	ENSRNOT00000089934	0.952061001	1.84E-06
TUCP_001589	XLOC_061713	ENSRNOT00000008988	0.959397529	8.12E-07
TUCP_001589	XLOC_061713	ENSRNOT00000003556	0.950707103	2.11E-06
TUCP_001589	XLOC_061713	ENSRNOT00000003511	0.952229442	1.81E-06
TUCP_001589	XLOC_061713	ENSRNOT00000074810	0.954491501	1.42E-06

TUCP_001589	XLOC_061713	ENSRNOT00000006697	0.956472788	1.14E-06
TUCP_001589	XLOC_061713	ENSRNOT00000005505	0.952428701	1.77E-06
TUCP_001589	XLOC_061713	ENSRNOT00000087799	0.954192007	1.47E-06
TUCP_001589	XLOC_061713	ENSRNOT00000009132	0.952021056	1.85E-06
TUCP_001589	XLOC_061713	ENSRNOT00000023623	0.956588448	1.13E-06
TUCP_001589	XLOC_061713	ENSRNOT00000009975	0.968876594	2.18E-07
TUCP_001589	XLOC_061713	ENSRNOT00000072682	0.966651195	3.07E-07
TUCP_001589	XLOC_061713	ENSRNOT00000055877	0.950870341	2.08E-06
TUCP_001589	XLOC_061713	ENSRNOT00000088385	0.971671298	1.37E-07
TUCP_001589	XLOC_061713	ENSRNOT00000050941	0.961524574	6.22E-07
TUCP_001589	XLOC_061713	ENSRNOT00000015864	0.963123164	5.05E-07
TUCP_001589	XLOC_061713	ENSRNOT00000024583	-0.953295576	1.62E-06
TUCP_001589	XLOC_061713	ENSRNOT00000010701	0.970220879	1.75E-07
TUCP_001589	XLOC_061713	ENSRNOT00000023661	0.957768077	9.85E-07
TUCP_001589	XLOC_061713	ENSRNOT00000034482	0.965075642	3.86E-07
TUCP_001589	XLOC_061713	ENSRNOT00000091367	0.950225551	2.21E-06
TUCP_001589	XLOC_061713	ENSRNOT00000019660	0.950821317	2.09E-06
TUCP_001583	XLOC_061573	ENSRNOT00000040016	0.957874207	9.73E-07
TUCP_001580	XLOC_061424	ENSRNOT00000019680	-0.953954931	1.51E-06
TUCP_001580	XLOC_061424	ENSRNOT00000028625	-0.955770956	1.24E-06
TUCP_001580	XLOC_061424	ENSRNOT00000031568	0.972382642	1.21E-07
TUCP_001580	XLOC_061424	ENSRNOT00000009096	-0.952282078	1.80E-06
TUCP_001581	XLOC_061567	ENSRNOT00000037655	0.952032336	1.84E-06
TUCP_001581	XLOC_061567	ENSRNOT00000076060	0.95077764	2.09E-06
TUCP_001581	XLOC_061567	ENSRNOT00000084784	0.960708708	6.90E-07
TUCP_001581	XLOC_061567	ENSRNOT00000056557	0.955536182	1.27E-06
TUCP_002563	XLOC_108829	ENSRNOT00000025981	0.966842513	2.99E-07
TUCP_002563	XLOC_108829	ENSRNOT00000055735	0.956675561	1.12E-06
TUCP_002563	XLOC_108829	ENSRNOT00000083799	0.952499458	1.76E-06
TUCP_002563	XLOC_108829	ENSRNOT00000068405	0.974508063	8.12E-08
TUCP_002563	XLOC_108829	ENSRNOT00000047387	0.953454125	1.59E-06
TUCP_002563	XLOC_108829	ENSRNOT00000076669	0.974672365	7.87E-08
TUCP_002563	XLOC_108829	ENSRNOT00000085596	0.97332292	1.02E-07
TUCP_002563	XLOC_108829	ENSRNOT00000084012	0.95174554	1.90E-06
TUCP_002563	XLOC_108829	ENSRNOT00000091910	0.950916291	2.07E-06
TUCP_002563	XLOC_108829	ENSRNOT00000012093	0.952342668	1.79E-06
TUCP_002563	XLOC_108829	ENSRNOT00000039842	0.974361721	8.36E-08
TUCP_002563	XLOC_108829	ENSRNOT00000055187	0.956308425	1.17E-06
TUCP_002563	XLOC_108829	ENSRNOT00000026293	0.953168328	1.64E-06
TUCP_002563	XLOC_108829	ENSRNOT00000004834	0.976104547	5.89E-08
TUCP_002563	XLOC_108829	ENSRNOT00000006401	-0.953305933	1.62E-06
TUCP_002563	XLOC_108829	ENSRNOT00000022022	0.957780068	9.84E-07
TUCP_002563	XLOC_108829	ENSRNOT00000057826	0.953061285	1.66E-06
TUCP_002563	XLOC_108829	ENSRNOT00000031230	0.960158045	7.39E-07
TUCP_002563	XLOC_108829	ENSRNOT00000082699	0.956226836	1.18E-06
TUCP_002563	XLOC_108829	ENSRNOT00000004508	0.955601704	1.26E-06
TUCP_002563	XLOC_108829	ENSRNOT00000040513	0.959733787	7.79E-07
TUCP_002563	XLOC_108829	ENSRNOT00000004740	0.952496218	1.76E-06
TUCP_002563	XLOC_108829	ENSRNOT00000009683	0.952223891	1.81E-06
TUCP_002563	XLOC_108829	ENSRNOT00000052288	0.967426136	2.73E-07
TUCP_002563	XLOC_108829	ENSRNOT00000004359	0.952344685	1.79E-06

TUCP_002563	XLOC_108829	ENSRNOT00000084088	0.966281413	3.24E-07
TUCP_002563	XLOC_108829	ENSRNOT00000039809	0.978856953	3.21E-08
TUCP_002563	XLOC_108829	ENSRNOT00000082100	0.950166895	2.23E-06
TUCP_002563	XLOC_108829	ENSRNOT00000029664	0.95601774	1.20E-06
TUCP_002563	XLOC_108829	ENSRNOT00000068633	0.961836632	5.98E-07
TUCP_002563	XLOC_108829	ENSRNOT00000076729	0.975390672	6.82E-08
TUCP_002563	XLOC_108829	ENSRNOT00000090665	0.965603218	3.58E-07
TUCP_002563	XLOC_108829	ENSRNOT00000003296	0.965616066	3.57E-07
TUCP_002563	XLOC_108829	ENSRNOT00000017948	0.961122135	6.55E-07
TUCP_002563	XLOC_108829	ENSRNOT00000058852	-0.954583468	1.41E-06
TUCP_002563	XLOC_108829	ENSRNOT00000054674	0.956050077	1.20E-06
TUCP_002563	XLOC_108829	ENSRNOT00000071694	0.955898238	1.22E-06
TUCP_002563	XLOC_108829	ENSRNOT00000025859	0.97490611	7.51E-08
TUCP_002563	XLOC_108829	ENSRNOT00000013758	0.951476766	1.95E-06
TUCP_002563	XLOC_108829	ENSRNOT00000034503	0.962395106	5.56E-07
TUCP_002563	XLOC_108829	ENSRNOT00000021865	0.952462783	1.76E-06
TUCP_002563	XLOC_108829	ENSRNOT00000005355	0.967355533	2.76E-07
TUCP_002563	XLOC_108829	ENSRNOT00000035989	0.990293473	6.68E-10
TUCP_002563	XLOC_108829	ENSRNOT00000004412	0.96539426	3.69E-07
TUCP_002563	XLOC_108829	ENSRNOT00000067181	0.959298636	8.21E-07
TUCP_002563	XLOC_108829	ENSRNOT00000010992	0.979096962	3.03E-08
TUCP_002563	XLOC_108829	ENSRNOT00000015076	0.955777726	1.24E-06
TUCP_002563	XLOC_108829	ENSRNOT00000024639	0.954069964	1.49E-06
TUCP_002563	XLOC_108829	ENSRNOT00000024942	0.956276141	1.17E-06
TUCP_002563	XLOC_108829	ENSRNOT00000006055	0.959265178	8.25E-07
TUCP_002563	XLOC_108829	ENSRNOT00000071573	0.966365439	3.20E-07
TUCP_002563	XLOC_108829	ENSRNOT00000028431	0.955359267	1.30E-06
TUCP_002563	XLOC_108829	ENSRNOT00000034704	0.962776752	5.29E-07
TUCP_002563	XLOC_108829	ENSRNOT00000013274	0.956227469	1.18E-06
TUCP_002563	XLOC_108829	ENSRNOT00000085027	0.968806692	2.21E-07
TUCP_002563	XLOC_108829	ENSRNOT00000066593	0.95597595	1.21E-06
TUCP_002563	XLOC_108829	ENSRNOT00000025092	0.959852772	7.68E-07
TUCP_002563	XLOC_108829	ENSRNOT00000051496	0.953102114	1.65E-06
TUCP_002563	XLOC_108829	ENSRNOT00000010898	0.960426759	7.15E-07
TUCP_002563	XLOC_108829	ENSRNOT00000090376	0.955582311	1.26E-06
TUCP_002563	XLOC_108829	ENSRNOT00000024420	0.965407934	3.68E-07
TUCP_002563	XLOC_108829	ENSRNOT00000027762	0.95552352	1.27E-06
TUCP_002563	XLOC_108829	ENSRNOT00000067867	0.952860588	1.69E-06
TUCP_002563	XLOC_108829	ENSRNOT00000016739	0.958696624	8.83E-07
TUCP_002563	XLOC_108829	ENSRNOT00000010242	0.980098346	2.38E-08
TUCP_002563	XLOC_108829	ENSRNOT00000028842	0.962580797	5.43E-07
TUCP_002563	XLOC_108829	ENSRNOT00000090424	0.958447174	9.10E-07
TUCP_002563	XLOC_108829	ENSRNOT00000027828	0.952064844	1.84E-06
TUCP_002563	XLOC_108829	ENSRNOT00000017629	0.976119951	5.88E-08
TUCP_002563	XLOC_108829	ENSRNOT00000086352	0.950893562	2.07E-06
TUCP_002563	XLOC_108829	ENSRNOT00000001222	0.966405008	3.19E-07
TUCP_002563	XLOC_108829	ENSRNOT00000017422	-0.957151421	1.06E-06
TUCP_002563	XLOC_108829	ENSRNOT00000057313	0.97003329	1.81E-07
TUCP_001584	XLOC_061675	ENSRNOT00000072735	-0.964446651	4.21E-07
TUCP_001274	XLOC_048267	ENSRNOT00000002309	0.951263355	1.99E-06
TUCP_001274	XLOC_048267	ENSRNOT00000084177	-0.961644423	6.13E-07

TUCP_001274	XLOC_048267	ENSRNOT00000072716	0.955892912	1.22E-06
TUCP_001274	XLOC_048267	ENSRNOT00000024237	0.965527362	3.62E-07
TUCP_001274	XLOC_048267	ENSRNOT00000002638	-0.951354975	1.98E-06
TUCP_001274	XLOC_048267	ENSRNOT00000032106	0.967731969	2.61E-07
TUCP_001274	XLOC_048267	ENSRNOT00000005811	-0.95156901	1.93E-06
TUCP_001274	XLOC_048267	ENSRNOT00000008440	0.965206181	3.79E-07
TUCP_001274	XLOC_048267	ENSRNOT00000028677	0.961881175	5.95E-07
TUCP_001274	XLOC_048267	ENSRNOT00000006160	0.966857427	2.98E-07
TUCP_001274	XLOC_048267	ENSRNOT00000011773	0.973722576	9.44E-08
TUCP_001274	XLOC_048267	ENSRNOT00000083344	0.961282173	6.42E-07
TUCP_001274	XLOC_048267	ENSRNOT00000091211	0.970561479	1.66E-07
TUCP_001275	XLOC_048268	ENSRNOT00000025081	0.961575589	6.18E-07
TUCP_001275	XLOC_048268	ENSRNOT00000002560	0.951230338	2.00E-06
TUCP_001275	XLOC_048268	ENSRNOT00000077526	0.961881882	5.94E-07
TUCP_001275	XLOC_048268	ENSRNOT00000024393	0.971343651	1.45E-07
TUCP_001275	XLOC_048268	ENSRNOT00000089889	0.95356456	1.57E-06
TUCP_001275	XLOC_048268	ENSRNOT00000041494	0.957300674	1.04E-06
TUCP_001275	XLOC_048268	ENSRNOT00000013580	0.955601342	1.26E-06
TUCP_001275	XLOC_048268	ENSRNOT00000081512	0.950531798	2.15E-06
TUCP_001275	XLOC_048268	ENSRNOT00000037133	0.958022776	9.57E-07
TUCP_001275	XLOC_048268	ENSRNOT00000045223	0.951978974	1.85E-06
TUCP_001275	XLOC_048268	ENSRNOT00000044395	0.954100271	1.49E-06
TUCP_001275	XLOC_048268	ENSRNOT00000047093	0.956036389	1.20E-06
TUCP_001275	XLOC_048268	ENSRNOT00000066132	0.956682723	1.12E-06
TUCP_001276	XLOC_048269	ENSRNOT00000015181	0.962214706	5.69E-07
TUCP_001276	XLOC_048269	ENSRNOT00000025624	0.959189209	8.32E-07
TUCP_001277	XLOC_048271	ENSRNOT00000031904	0.957367142	1.03E-06
TUCP_001277	XLOC_048271	ENSRNOT00000082999	-0.955769095	1.24E-06
TUCP_001277	XLOC_048271	ENSRNOT00000072007	-0.961389397	6.33E-07
TUCP_001277	XLOC_048271	ENSRNOT00000027083	0.951996443	1.85E-06
TUCP_001277	XLOC_048271	ENSRNOT00000084088	0.961844674	5.97E-07
TUCP_001277	XLOC_048271	ENSRNOT00000023302	0.960525	7.06E-07
TUCP_001277	XLOC_048271	ENSRNOT00000002560	0.975901301	6.15E-08
TUCP_001277	XLOC_048271	ENSRNOT00000089007	-0.951750087	1.90E-06
TUCP_001277	XLOC_048271	ENSRNOT00000066024	0.963119652	5.05E-07
TUCP_001277	XLOC_048271	ENSRNOT00000083127	0.952961118	1.68E-06
TUCP_001277	XLOC_048271	ENSRNOT00000023144	0.954073949	1.49E-06
TUCP_001277	XLOC_048271	ENSRNOT00000080695	0.962361803	5.58E-07
TUCP_001277	XLOC_048271	ENSRNOT00000078070	0.963828623	4.59E-07
TUCP_001277	XLOC_048271	ENSRNOT00000026909	-0.955685122	1.25E-06
TUCP_001277	XLOC_048271	ENSRNOT00000010180	-0.961446924	6.29E-07
TUCP_001277	XLOC_048271	ENSRNOT00000060696	0.953786758	1.54E-06
TUCP_001277	XLOC_048271	ENSRNOT00000026543	0.956265505	1.17E-06
TUCP_001277	XLOC_048271	ENSRNOT00000065501	0.952698096	1.72E-06
TUCP_001277	XLOC_048271	ENSRNOT00000018742	-0.952205493	1.81E-06
TUCP_001277	XLOC_048271	ENSRNOT00000092203	0.95698221	1.08E-06
TUCP_001277	XLOC_048271	ENSRNOT00000067846	-0.956137821	1.19E-06
TUCP_001277	XLOC_048271	ENSRNOT00000047093	0.972114084	1.27E-07
TUCP_001277	XLOC_048271	ENSRNOT00000050180	0.955043137	1.34E-06
TUCP_001277	XLOC_048271	ENSRNOT00000057160	0.968334596	2.38E-07
TUCP_001277	XLOC_048271	ENSRNOT00000024393	0.982522283	1.25E-08

TUCP_001277	XLOC_048271	ENSRNOT00000090420	-0.950932591	2.06E-06
TUCP_001277	XLOC_048271	ENSRNOT00000044395	0.964732464	4.05E-07
TUCP_001277	XLOC_048271	ENSRNOT00000001222	0.958281341	9.28E-07
TUCP_001277	XLOC_048271	ENSRNOT00000022552	-0.951205785	2.01E-06
TUCP_001379	XLOC_053306	ENSRNOT00000028485	0.954421067	1.43E-06
TUCP_001272	XLOC_048239	ENSRNOT00000026949	-0.961948899	5.89E-07
TUCP_001272	XLOC_048239	ENSRNOT00000004698	0.95278835	1.71E-06
TUCP_001272	XLOC_048239	ENSRNOT00000093640	0.959014484	8.50E-07
TUCP_001272	XLOC_048239	ENSRNOT00000025394	0.968948036	2.16E-07
TUCP_001272	XLOC_048239	ENSRNOT00000073636	0.950256399	2.21E-06
TUCP_001272	XLOC_048239	ENSRNOT00000026065	0.950980148	2.05E-06
TUCP_001272	XLOC_048239	ENSRNOT00000005228	0.950131512	2.23E-06
TUCP_001272	XLOC_048239	ENSRNOT00000052288	0.952965033	1.67E-06
TUCP_001272	XLOC_048239	ENSRNOT00000088995	0.952669203	1.73E-06
TUCP_001272	XLOC_048239	ENSRNOT00000003013	0.950527644	2.15E-06
TUCP_001272	XLOC_048239	ENSRNOT00000064900	0.976298406	5.66E-08
TUCP_001272	XLOC_048239	ENSRNOT00000068633	0.959479728	8.04E-07
TUCP_001272	XLOC_048239	ENSRNOT00000019769	-0.950424539	2.17E-06
TUCP_001272	XLOC_048239	ENSRNOT00000017948	0.960120936	7.43E-07
TUCP_001272	XLOC_048239	ENSRNOT00000011384	0.954756689	1.38E-06
TUCP_001272	XLOC_048239	ENSRNOT00000013031	-0.960098543	7.45E-07
TUCP_001272	XLOC_048239	ENSRNOT00000006195	0.950244303	2.21E-06
TUCP_001272	XLOC_048239	ENSRNOT00000004412	0.953515941	1.58E-06
TUCP_001272	XLOC_048239	ENSRNOT00000057814	0.953559996	1.57E-06
TUCP_001272	XLOC_048239	ENSRNOT00000078639	0.95710558	1.06E-06
TUCP_001272	XLOC_048239	ENSRNOT00000013046	-0.98030095	2.26E-08
TUCP_001272	XLOC_048239	ENSRNOT00000087725	0.963377834	4.88E-07
TUCP_001272	XLOC_048239	ENSRNOT00000084595	0.953931967	1.51E-06
TUCP_001272	XLOC_048239	ENSRNOT00000009129	0.953841125	1.53E-06
TUCP_001272	XLOC_048239	ENSRNOT00000020437	0.961131218	6.55E-07
TUCP_001272	XLOC_048239	ENSRNOT00000073346	0.963675715	4.69E-07
TUCP_001272	XLOC_048239	ENSRNOT00000006963	0.958732682	8.79E-07
TUCP_001272	XLOC_048239	ENSRNOT00000011622	0.953687959	1.55E-06
TUCP_001272	XLOC_048239	ENSRNOT00000049538	0.966634935	3.08E-07
TUCP_001272	XLOC_048239	ENSRNOT00000044177	0.95756851	1.01E-06
TUCP_001272	XLOC_048239	ENSRNOT00000027944	0.964272436	4.32E-07
TUCP_001272	XLOC_048239	ENSRNOT00000026087	-0.970703237	1.62E-07
TUCP_001272	XLOC_048239	ENSRNOT00000010242	0.950437193	2.17E-06
TUCP_001272	XLOC_048239	ENSRNOT00000028842	0.978138017	3.79E-08
TUCP_001272	XLOC_048239	ENSRNOT00000057313	0.95055269	2.14E-06
TUCP_001272	XLOC_048239	ENSRNOT00000006800	0.950751451	2.10E-06
TUCP_001272	XLOC_048239	ENSRNOT00000004026	0.965258313	3.76E-07
TUCP_001272	XLOC_048239	ENSRNOT00000085648	0.958023224	9.56E-07
TUCP_000225	XLOC_006985	ENSRNOT00000071545	0.951844221	1.88E-06
TUCP_001377	XLOC_053305	ENSRNOT00000009280	-0.951136574	2.02E-06
TUCP_001377	XLOC_053305	ENSRNOT00000015529	-0.952544151	1.75E-06
TUCP_001377	XLOC_053305	ENSRNOT00000082687	0.952920363	1.68E-06
TUCP_001377	XLOC_053305	ENSRNOT00000019453	-0.950976433	2.05E-06
TUCP_001377	XLOC_053305	ENSRNOT00000004742	0.953705634	1.55E-06
TUCP_001377	XLOC_053305	ENSRNOT00000042714	-0.952657087	1.73E-06
TUCP_001377	XLOC_053305	ENSRNOT00000016384	0.956540181	1.13E-06

TUCP_001377	XLOC_053305	ENSRNOT00000093640	-0.95094338	2.06E-06
TUCP_001377	XLOC_053305	ENSRNOT00000088995	-0.95029522	2.20E-06
TUCP_001377	XLOC_053305	ENSRNOT00000020098	0.951646501	1.92E-06
TUCP_001377	XLOC_053305	ENSRNOT00000085501	0.963143612	5.03E-07
TUCP_001377	XLOC_053305	ENSRNOT00000005126	0.950388802	2.18E-06
TUCP_001377	XLOC_053305	ENSRNOT00000010385	0.965827892	3.47E-07
TUCP_001278	XLOC_048272	ENSRNOT00000026543	0.963103024	5.06E-07
TUCP_001278	XLOC_048272	ENSRNOT00000090376	0.955199732	1.32E-06
TUCP_001278	XLOC_048272	ENSRNOT00000023144	0.961569848	6.19E-07
TUCP_001278	XLOC_048272	ENSRNOT00000080695	0.96811702	2.46E-07
TUCP_001278	XLOC_048272	ENSRNOT00000057160	0.962792998	5.28E-07
TUCP_001278	XLOC_048272	ENSRNOT00000078070	0.95250604	1.76E-06
TUCP_001278	XLOC_048272	ENSRNOT00000067400	0.959912086	7.62E-07
TUCP_001279	XLOC_048274	ENSRNOT00000012671	0.954400615	1.44E-06
TUCP_001279	XLOC_048274	ENSRNOT00000023144	0.979198149	2.96E-08
TUCP_001279	XLOC_048274	ENSRNOT00000085158	0.956321187	1.16E-06
TUCP_001279	XLOC_048274	ENSRNOT00000050180	0.965580165	3.59E-07
TUCP_001279	XLOC_048274	ENSRNOT00000039809	0.953256746	1.62E-06
TUCP_001279	XLOC_048274	ENSRNOT00000000743	0.961544321	6.21E-07
TUCP_001279	XLOC_048274	ENSRNOT00000027083	0.955891699	1.22E-06
TUCP_001279	XLOC_048274	ENSRNOT00000084088	0.972368967	1.21E-07
TUCP_001279	XLOC_048274	ENSRNOT00000081512	0.962975378	5.15E-07
TUCP_001279	XLOC_048274	ENSRNOT00000023302	0.965180077	3.80E-07
TUCP_001279	XLOC_048274	ENSRNOT00000002560	0.975826333	6.24E-08
TUCP_001279	XLOC_048274	ENSRNOT00000089007	-0.954797552	1.38E-06
TUCP_001279	XLOC_048274	ENSRNOT00000066024	0.975352737	6.87E-08
TUCP_001279	XLOC_048274	ENSRNOT00000025808	0.953594619	1.57E-06
TUCP_001279	XLOC_048274	ENSRNOT00000031904	0.974043194	8.88E-08
TUCP_001279	XLOC_048274	ENSRNOT00000012736	0.95992424	7.61E-07
TUCP_001279	XLOC_048274	ENSRNOT00000055992	-0.950192242	2.22E-06
TUCP_001279	XLOC_048274	ENSRNOT00000087429	0.956165157	1.18E-06
TUCP_001279	XLOC_048274	ENSRNOT00000040513	0.950594578	2.13E-06
TUCP_001279	XLOC_048274	ENSRNOT00000079072	0.958808184	8.71E-07
TUCP_001279	XLOC_048274	ENSRNOT00000024402	0.960272855	7.29E-07
TUCP_001279	XLOC_048274	ENSRNOT00000083127	0.964654307	4.09E-07
TUCP_001279	XLOC_048274	ENSRNOT00000078306	0.958546286	8.99E-07
TUCP_001279	XLOC_048274	ENSRNOT00000080695	0.981656062	1.59E-08
TUCP_001279	XLOC_048274	ENSRNOT00000041044	0.959069761	8.45E-07
TUCP_001279	XLOC_048274	ENSRNOT00000000302	0.95315203	1.64E-06
TUCP_001279	XLOC_048274	ENSRNOT00000016649	0.957329281	1.04E-06
TUCP_001279	XLOC_048274	ENSRNOT00000078070	0.973246658	1.03E-07
TUCP_001279	XLOC_048274	ENSRNOT00000088054	0.957269508	1.04E-06
TUCP_001279	XLOC_048274	ENSRNOT00000026941	0.966079829	3.34E-07
TUCP_001279	XLOC_048274	ENSRNOT00000010541	0.952805259	1.70E-06
TUCP_001279	XLOC_048274	ENSRNOT00000010180	-0.952650548	1.73E-06
TUCP_001279	XLOC_048274	ENSRNOT00000047192	-0.958700297	8.83E-07
TUCP_001279	XLOC_048274	ENSRNOT00000033017	0.960947085	6.70E-07
TUCP_001279	XLOC_048274	ENSRNOT00000060696	0.969818722	1.87E-07
TUCP_001279	XLOC_048274	ENSRNOT00000042125	0.957878872	9.73E-07
TUCP_001279	XLOC_048274	ENSRNOT00000026543	0.976089866	5.91E-08
TUCP_001279	XLOC_048274	ENSRNOT00000065501	0.967944162	2.53E-07

TUCP_001279	XLOC_048274	ENSRNOT00000026293	0.964874138	3.97E-07
TUCP_001279	XLOC_048274	ENSRNOT00000076729	0.95538548	1.29E-06
TUCP_001279	XLOC_048274	ENSRNOT00000080207	0.967708626	2.62E-07
TUCP_001279	XLOC_048274	ENSRNOT00000067400	0.957809563	9.81E-07
TUCP_001279	XLOC_048274	ENSRNOT00000090665	0.961023958	6.63E-07
TUCP_001279	XLOC_048274	ENSRNOT00000076669	0.957771968	9.85E-07
TUCP_001279	XLOC_048274	ENSRNOT00000005387	0.953572801	1.57E-06
TUCP_001279	XLOC_048274	ENSRNOT00000006175	0.964630383	4.11E-07
TUCP_001279	XLOC_048274	ENSRNOT00000027337	0.952039896	1.84E-06
TUCP_001279	XLOC_048274	ENSRNOT00000024393	0.968938806	2.16E-07
TUCP_001279	XLOC_048274	ENSRNOT00000025075	0.963625321	4.72E-07
TUCP_001279	XLOC_048274	ENSRNOT00000035989	0.967608422	2.66E-07
TUCP_001279	XLOC_048274	ENSRNOT00000057160	0.981590208	1.61E-08
TUCP_001279	XLOC_048274	ENSRNOT00000083799	0.951537932	1.94E-06
TUCP_001279	XLOC_048274	ENSRNOT00000047093	0.981403262	1.70E-08
TUCP_001279	XLOC_048274	ENSRNOT00000090420	-0.952111528	1.83E-06
TUCP_001279	XLOC_048274	ENSRNOT00000022796	0.952756091	1.71E-06
TUCP_001279	XLOC_048274	ENSRNOT00000045223	0.953270223	1.62E-06
TUCP_001279	XLOC_048274	ENSRNOT00000044395	0.956816321	1.10E-06
TUCP_001279	XLOC_048274	ENSRNOT00000015799	0.9601237	7.43E-07
TUCP_001279	XLOC_048274	ENSRNOT00000001222	0.969489898	1.98E-07
TUCP_001279	XLOC_048274	ENSRNOT00000022552	-0.950167309	2.23E-06
TUCP_001279	XLOC_048274	ENSRNOT00000050834	-0.951481981	1.95E-06
TUCP_001373	XLOC_053127	ENSRNOT00000015944	0.953116868	1.65E-06
TUCP_001373	XLOC_053127	ENSRNOT00000030327	0.962600796	5.41E-07
TUCP_001373	XLOC_053127	ENSRNOT00000085380	0.965826067	3.47E-07
TUCP_001528	XLOC_059064	ENSRNOT00000051155	0.952301351	1.79E-06
TUCP_001409	XLOC_055092	ENSRNOT00000024186	0.981360569	1.72E-08
TUCP_001409	XLOC_055092	ENSRNOT00000074753	0.997613734	6.07E-13
TUCP_001409	XLOC_055092	ENSRNOT00000004876	0.955908848	1.22E-06
TUCP_001409	XLOC_055092	ENSRNOT00000041904	0.998440622	7.24E-14
TUCP_001409	XLOC_055092	ENSRNOT00000083487	0.984524361	6.81E-09
TUCP_001409	XLOC_055092	ENSRNOT00000077990	0.998440622	7.24E-14
TUCP_001409	XLOC_055092	ENSRNOT00000038826	0.99803399	2.31E-13
TUCP_001409	XLOC_055092	ENSRNOT00000037094	0.958570108	8.97E-07
TUCP_001409	XLOC_055092	ENSRNOT00000046842	0.970852846	1.58E-07
TUCP_001409	XLOC_055092	ENSRNOT00000037169	0.97809782	3.83E-08
TUCP_001409	XLOC_055092	ENSRNOT00000021243	0.985003139	5.83E-09
TUCP_001409	XLOC_055092	ENSRNOT00000060250	0.999016651	7.23E-15
TUCP_001409	XLOC_055092	ENSRNOT00000091934	0.998440622	7.24E-14
TUCP_001409	XLOC_055092	ENSRNOT00000086027	0.957045379	1.07E-06
TUCP_001409	XLOC_055092	ENSRNOT00000046585	0.980270891	2.28E-08
TUCP_001409	XLOC_055092	ENSRNOT00000080794	0.988018754	1.91E-09
TUCP_001409	XLOC_055092	ENSRNOT00000014456	0.989432851	1.02E-09
TUCP_001409	XLOC_055092	ENSRNOT00000031205	0.988814872	1.35E-09
TUCP_001409	XLOC_055092	ENSRNOT00000044145	0.998440622	7.24E-14
TUCP_001409	XLOC_055092	ENSRNOT00000088381	0.986645802	3.27E-09
TUCP_001409	XLOC_055092	ENSRNOT00000011743	0.984340931	7.22E-09
TUCP_001409	XLOC_055092	ENSRNOT00000089929	0.998440622	7.24E-14
TUCP_001409	XLOC_055092	ENSRNOT00000088624	0.998440622	7.24E-14
TUCP_001409	XLOC_055092	ENSRNOT00000085659	0.987573913	2.29E-09

TUCP_001409	XLOC_055092	ENSRNOT00000015866	0.990996238	4.59E-10
TUCP_001409	XLOC_055092	ENSRNOT00000078633	0.998440622	7.24E-14
TUCP_001409	XLOC_055092	ENSRNOT00000034907	0.991097297	4.34E-10
TUCP_001409	XLOC_055092	ENSRNOT00000093230	0.953506479	1.58E-06
TUCP_001409	XLOC_055092	ENSRNOT00000051835	0.998440622	7.24E-14
TUCP_001409	XLOC_055092	ENSRNOT00000079087	0.998440622	7.24E-14
TUCP_001409	XLOC_055092	ENSRNOT00000037206	0.998440622	7.24E-14
TUCP_001409	XLOC_055092	ENSRNOT00000083261	0.973165212	1.05E-07
TUCP_001409	XLOC_055092	ENSRNOT00000080590	0.998246919	1.30E-13
TUCP_001409	XLOC_055092	ENSRNOT00000027552	0.998776062	2.16E-14
TUCP_001409	XLOC_055092	ENSRNOT00000077014	0.998089746	2.00E-13
TUCP_001409	XLOC_055092	ENSRNOT00000080453	0.998529562	5.40E-14
TUCP_001409	XLOC_055092	ENSRNOT00000089057	0.960417998	7.16E-07
TUCP_001409	XLOC_055092	ENSRNOT00000066839	0.989456446	1.01E-09
TUCP_001409	XLOC_055092	ENSRNOT00000061284	0.959306158	8.21E-07
TUCP_001409	XLOC_055092	ENSRNOT00000013423	0.958777459	8.75E-07
TUCP_001409	XLOC_055092	ENSRNOT00000071884	0.97141319	1.43E-07
TUCP_001409	XLOC_055092	ENSRNOT00000004128	0.954879295	1.37E-06
TUCP_001409	XLOC_055092	ENSRNOT00000026316	0.961624323	6.15E-07
TUCP_001409	XLOC_055092	ENSRNOT00000057729	0.997629885	5.87E-13
TUCP_001409	XLOC_055092	ENSRNOT00000078663	0.979698144	2.63E-08
TUCP_001409	XLOC_055092	ENSRNOT00000064250	0.987077649	2.78E-09
TUCP_001409	XLOC_055092	ENSRNOT00000021608	0.969279845	2.05E-07
TUCP_001409	XLOC_055092	ENSRNOT00000029634	0.993616792	8.26E-11
TUCP_001409	XLOC_055092	ENSRNOT00000093579	0.954108205	1.48E-06
TUCP_001409	XLOC_055092	ENSRNOT00000086349	0.952072573	1.84E-06
TUCP_001409	XLOC_055092	ENSRNOT00000065460	0.998440622	7.24E-14
TUCP_001409	XLOC_055092	ENSRNOT00000003817	0.998512004	5.73E-14
TUCP_001409	XLOC_055092	ENSRNOT00000016713	0.998440622	7.24E-14
TUCP_001409	XLOC_055092	ENSRNOT00000079260	0.993995895	6.08E-11
TUCP_001409	XLOC_055092	ENSRNOT00000039294	0.998440622	7.24E-14
TUCP_001409	XLOC_055092	ENSRNOT00000092913	0.954794354	1.38E-06
TUCP_004605	XLOC_202883	ENSRNOT00000052270	-0.978351071	3.61E-08
TUCP_004605	XLOC_202883	ENSRNOT00000056727	-0.969270149	2.05E-07
TUCP_004605	XLOC_202883	ENSRNOT00000012883	-0.96607472	3.34E-07
TUCP_000804	XLOC_030654	ENSRNOT00000085599	0.990508967	5.97E-10
TUCP_000804	XLOC_030654	ENSRNOT00000006689	0.983681642	8.87E-09
TUCP_000804	XLOC_030654	ENSRNOT00000081534	0.981134306	1.82E-08
TUCP_000804	XLOC_030654	ENSRNOT00000085301	0.986667804	3.24E-09
TUCP_000804	XLOC_030654	ENSRNOT00000038729	0.990729517	5.31E-10
TUCP_000804	XLOC_030654	ENSRNOT00000086385	0.990729517	5.31E-10
TUCP_000804	XLOC_030654	ENSRNOT00000077695	0.969156445	2.09E-07
TUCP_000804	XLOC_030654	ENSRNOT00000078561	0.991033614	4.50E-10
TUCP_000804	XLOC_030654	ENSRNOT00000087613	0.990729517	5.31E-10
TUCP_000804	XLOC_030654	ENSRNOT00000023062	0.990729517	5.31E-10
TUCP_000804	XLOC_030654	ENSRNOT00000058834	0.990729517	5.31E-10
TUCP_000804	XLOC_030654	ENSRNOT00000079420	0.990729517	5.31E-10
TUCP_000804	XLOC_030654	ENSRNOT00000090761	0.990729517	5.31E-10
TUCP_000804	XLOC_030654	ENSRNOT00000055923	0.990729517	5.31E-10
TUCP_000804	XLOC_030654	ENSRNOT00000092416	0.97065563	1.63E-07
TUCP_000804	XLOC_030654	ENSRNOT00000071930	0.990729517	5.31E-10

TUCP_000804	XLOC_030654	ENSRNOT00000001996	0.967401359	2.74E-07
TUCP_000804	XLOC_030654	ENSRNOT00000076756	0.990729517	5.31E-10
TUCP_000804	XLOC_030654	ENSRNOT00000081527	0.989665435	9.12E-10
TUCP_000804	XLOC_030654	ENSRNOT00000091663	0.985389504	5.12E-09
TUCP_000804	XLOC_030654	ENSRNOT00000081780	0.990729517	5.31E-10
TUCP_000804	XLOC_030654	ENSRNOT00000089114	0.990729517	5.31E-10
TUCP_000804	XLOC_030654	ENSRNOT00000021609	0.964892497	3.96E-07
TUCP_000804	XLOC_030654	ENSRNOT00000093444	0.965543909	3.61E-07
TUCP_000804	XLOC_030654	ENSRNOT00000073316	0.990729517	5.31E-10
TUCP_000804	XLOC_030654	ENSRNOT00000086557	0.981871759	1.50E-08
TUCP_000804	XLOC_030654	ENSRNOT00000071663	0.990729517	5.31E-10
TUCP_000804	XLOC_030654	ENSRNOT00000042860	0.990729517	5.31E-10
TUCP_000804	XLOC_030654	ENSRNOT00000072962	0.958717727	8.81E-07
TUCP_000804	XLOC_030654	ENSRNOT00000088421	0.990729517	5.31E-10
TUCP_000804	XLOC_030654	ENSRNOT00000038898	0.990729517	5.31E-10
TUCP_000804	XLOC_030654	ENSRNOT00000064936	0.974413682	8.27E-08
TUCP_000804	XLOC_030654	ENSRNOT00000042777	0.979494463	2.76E-08
TUCP_000804	XLOC_030654	ENSRNOT00000010029	0.990729517	5.31E-10
TUCP_003695	XLOC_161318	ENSRNOT00000018515	0.957662384	9.98E-07
TUCP_003695	XLOC_161318	ENSRNOT00000068747	0.955574144	1.26E-06
TUCP_001401	XLOC_053795	ENSRNOT00000021243	0.955467158	1.28E-06
TUCP_001401	XLOC_053795	ENSRNOT00000061047	0.955460952	1.28E-06
TUCP_001401	XLOC_053795	ENSRNOT00000064613	0.953806292	1.53E-06
TUCP_001401	XLOC_053795	ENSRNOT00000042721	0.95432864	1.45E-06
TUCP_000801	XLOC_030520	ENSRNOT00000024793	0.952103906	1.83E-06
TUCP_000801	XLOC_030520	ENSRNOT00000018776	0.96089697	6.74E-07
TUCP_003692	XLOC_161316	ENSRNOT00000025981	0.953999554	1.50E-06
TUCP_003692	XLOC_161316	ENSRNOT00000028542	0.950744621	2.10E-06
TUCP_003692	XLOC_161316	ENSRNOT00000023744	0.956606218	1.13E-06
TUCP_003692	XLOC_161316	ENSRNOT00000014888	0.958895936	8.62E-07
TUCP_003692	XLOC_161316	ENSRNOT00000057537	0.955358146	1.30E-06
TUCP_003692	XLOC_161316	ENSRNOT00000032992	0.952904748	1.69E-06
TUCP_003692	XLOC_161316	ENSRNOT00000004740	0.951944287	1.86E-06
TUCP_003692	XLOC_161316	ENSRNOT00000036072	0.96409713	4.42E-07
TUCP_003692	XLOC_161316	ENSRNOT00000080695	0.981786692	1.53E-08
TUCP_003692	XLOC_161316	ENSRNOT00000000302	0.957889502	9.72E-07
TUCP_003692	XLOC_161316	ENSRNOT00000092418	0.951913008	1.87E-06
TUCP_003692	XLOC_161316	ENSRNOT00000076285	0.96458617	4.13E-07
TUCP_003692	XLOC_161316	ENSRNOT00000018787	0.966844581	2.98E-07
TUCP_003692	XLOC_161316	ENSRNOT00000013758	0.954498012	1.42E-06
TUCP_003692	XLOC_161316	ENSRNOT00000010824	0.953283641	1.62E-06
TUCP_003692	XLOC_161316	ENSRNOT00000067400	0.951883149	1.87E-06
TUCP_003692	XLOC_161316	ENSRNOT00000040513	0.967407156	2.74E-07
TUCP_003692	XLOC_161316	ENSRNOT00000033661	0.951713594	1.91E-06
TUCP_003692	XLOC_161316	ENSRNOT00000023696	0.953942444	1.51E-06
TUCP_003692	XLOC_161316	ENSRNOT00000030400	0.952829982	1.70E-06
TUCP_003692	XLOC_161316	ENSRNOT00000090376	0.963675094	4.69E-07
TUCP_003692	XLOC_161316	ENSRNOT00000035989	0.966839465	2.99E-07
TUCP_003692	XLOC_161316	ENSRNOT00000057160	0.963476139	4.81E-07
TUCP_003692	XLOC_161316	ENSRNOT00000016739	0.979091871	3.04E-08
TUCP_003692	XLOC_161316	ENSRNOT00000009221	0.960567609	7.03E-07

TUCP_003692	XLOC_161316	ENSRNOT00000042799	0.963985158	4.49E-07
TUCP_002160	XLOC_090304	ENSRNOT00000041533	0.954356988	1.44E-06
TUCP_002160	XLOC_090304	ENSRNOT00000091367	0.96064894	6.96E-07
TUCP_002160	XLOC_090304	ENSRNOT00000074810	0.958570958	8.96E-07
TUCP_002160	XLOC_090304	ENSRNOT00000054963	0.951810498	1.89E-06
TUCP_002160	XLOC_090304	ENSRNOT00000091315	0.961839415	5.98E-07
TUCP_002160	XLOC_090304	ENSRNOT00000079882	0.95261581	1.74E-06
TUCP_002569	XLOC_108859	ENSRNOT00000043784	0.951516393	1.94E-06
TUCP_002569	XLOC_108859	ENSRNOT00000036766	0.954437668	1.43E-06
TUCP_002569	XLOC_108859	ENSRNOT00000080557	0.986057238	4.05E-09
TUCP_002569	XLOC_108859	ENSRNOT00000012811	0.957415078	1.03E-06
TUCP_002569	XLOC_108859	ENSRNOT00000037800	0.961085772	6.58E-07
TUCP_002569	XLOC_108859	ENSRNOT00000010263	0.951623145	1.92E-06
TUCP_002569	XLOC_108859	ENSRNOT00000016975	0.982882421	1.12E-08
TUCP_002668	XLOC_113192	ENSRNOT00000042681	0.959853406	7.68E-07
TUCP_002568	XLOC_108859	ENSRNOT00000092029	0.958446249	9.10E-07
TUCP_002568	XLOC_108859	ENSRNOT00000057045	-0.951453099	1.96E-06
TUCP_002568	XLOC_108859	ENSRNOT00000065143	0.981536865	1.64E-08
TUCP_002568	XLOC_108859	ENSRNOT00000005958	0.98235204	1.31E-08
TUCP_002568	XLOC_108859	ENSRNOT00000017063	-0.969109899	2.10E-07
TUCP_002568	XLOC_108859	ENSRNOT00000029136	-0.952855641	1.69E-06
TUCP_002568	XLOC_108859	ENSRNOT00000027526	0.964077543	4.44E-07
TUCP_002568	XLOC_108859	ENSRNOT00000028793	0.959700616	7.82E-07
TUCP_002568	XLOC_108859	ENSRNOT00000082998	0.951411779	1.97E-06
TUCP_002568	XLOC_108859	ENSRNOT00000072208	0.963430433	4.84E-07
TUCP_002568	XLOC_108859	ENSRNOT00000055321	0.951442345	1.96E-06
TUCP_002568	XLOC_108859	ENSRNOT00000027083	0.951482642	1.95E-06
TUCP_002568	XLOC_108859	ENSRNOT00000087095	0.971160354	1.50E-07
TUCP_002568	XLOC_108859	ENSRNOT00000063873	0.955203288	1.32E-06
TUCP_002568	XLOC_108859	ENSRNOT00000081047	0.953156566	1.64E-06
TUCP_002568	XLOC_108859	ENSRNOT00000008372	0.966020229	3.37E-07
TUCP_002568	XLOC_108859	ENSRNOT00000067688	-0.962970521	5.15E-07
TUCP_002568	XLOC_108859	ENSRNOT00000050834	-0.952592855	1.74E-06
TUCP_002568	XLOC_108859	ENSRNOT00000002443	0.955118532	1.33E-06
TUCP_002568	XLOC_108859	ENSRNOT00000004046	-0.950594225	2.13E-06
TUCP_002568	XLOC_108859	ENSRNOT00000025936	-0.950954648	2.06E-06
TUCP_002568	XLOC_108859	ENSRNOT00000088353	-0.970624183	1.64E-07
TUCP_002568	XLOC_108859	ENSRNOT00000083173	-0.95075177	2.10E-06
TUCP_002568	XLOC_108859	ENSRNOT00000021471	-0.951102463	2.03E-06
TUCP_002568	XLOC_108859	ENSRNOT00000019502	0.955707125	1.25E-06
TUCP_002568	XLOC_108859	ENSRNOT00000027970	0.953568762	1.57E-06
TUCP_002568	XLOC_108859	ENSRNOT00000059524	-0.957751952	9.87E-07
TUCP_002568	XLOC_108859	ENSRNOT00000008757	0.963802754	4.61E-07
TUCP_002568	XLOC_108859	ENSRNOT00000022963	0.950162031	2.23E-06
TUCP_002568	XLOC_108859	ENSRNOT00000012942	0.955296313	1.30E-06
TUCP_002568	XLOC_108859	ENSRNOT00000028625	-0.966528971	3.13E-07
TUCP_002568	XLOC_108859	ENSRNOT00000007379	0.955010476	1.35E-06
TUCP_002568	XLOC_108859	ENSRNOT00000035474	0.954602743	1.41E-06
TUCP_002568	XLOC_108859	ENSRNOT00000048063	0.951443557	1.96E-06
TUCP_002568	XLOC_108859	ENSRNOT00000003714	0.952747229	1.71E-06
TUCP_002568	XLOC_108859	ENSRNOT00000052224	0.962353844	5.59E-07

TUCP_002568	XLOC_108859	ENSRNOT00000022142	0.958820985	8.70E-07
TUCP_002568	XLOC_108859	ENSRNOT00000056130	0.967763476	2.60E-07
TUCP_002568	XLOC_108859	ENSRNOT00000008929	0.963128764	5.04E-07
TUCP_002568	XLOC_108859	ENSRNOT00000070893	0.969138613	2.09E-07
TUCP_002568	XLOC_108859	ENSRNOT00000088182	0.951532487	1.94E-06
TUCP_002568	XLOC_108859	ENSRNOT00000084385	0.954501203	1.42E-06
TUCP_002568	XLOC_108859	ENSRNOT00000067170	-0.95357487	1.57E-06
TUCP_002568	XLOC_108859	ENSRNOT00000090342	0.963325235	4.91E-07
TUCP_002568	XLOC_108859	ENSRNOT00000001586	-0.955268463	1.31E-06
TUCP_002568	XLOC_108859	ENSRNOT00000029125	0.95999653	7.54E-07
TUCP_002568	XLOC_108859	ENSRNOT00000009496	0.959267743	8.25E-07
TUCP_002568	XLOC_108859	ENSRNOT00000044562	0.95267552	1.73E-06
TUCP_002568	XLOC_108859	ENSRNOT00000005329	-0.959812073	7.72E-07
TUCP_002568	XLOC_108859	ENSRNOT00000003158	-0.961030168	6.63E-07
TUCP_002568	XLOC_108859	ENSRNOT00000005228	0.954214433	1.47E-06
TUCP_002568	XLOC_108859	ENSRNOT00000014814	0.950120693	2.24E-06
TUCP_002568	XLOC_108859	ENSRNOT00000077201	-0.980652435	2.07E-08
TUCP_002568	XLOC_108859	ENSRNOT00000017181	-0.956575586	1.13E-06
TUCP_002568	XLOC_108859	ENSRNOT00000017753	0.957500462	1.02E-06
TUCP_002568	XLOC_108859	ENSRNOT00000051022	0.957778587	9.84E-07
TUCP_002568	XLOC_108859	ENSRNOT00000081981	0.972638434	1.15E-07
TUCP_002568	XLOC_108859	ENSRNOT00000024969	0.963391301	4.87E-07
TUCP_002568	XLOC_108859	ENSRNOT00000019886	0.950368493	2.18E-06
TUCP_002568	XLOC_108859	ENSRNOT00000033235	0.961945346	5.90E-07
TUCP_002568	XLOC_108859	ENSRNOT00000042125	0.965512302	3.63E-07
TUCP_002568	XLOC_108859	ENSRNOT00000004956	0.950014614	2.26E-06
TUCP_002568	XLOC_108859	ENSRNOT00000082869	0.961425031	6.30E-07
TUCP_002568	XLOC_108859	ENSRNOT00000012187	0.95186675	1.88E-06
TUCP_002568	XLOC_108859	ENSRNOT00000080608	0.959667959	7.85E-07
TUCP_002568	XLOC_108859	ENSRNOT00000008901	-0.956209005	1.18E-06
TUCP_002568	XLOC_108859	ENSRNOT00000071493	-0.957849494	9.76E-07
TUCP_002568	XLOC_108859	ENSRNOT00000073971	0.955671005	1.25E-06
TUCP_002568	XLOC_108859	ENSRNOT00000009803	0.959151466	8.36E-07
TUCP_002568	XLOC_108859	ENSRNOT00000047192	-0.952268414	1.80E-06
TUCP_002568	XLOC_108859	ENSRNOT00000003971	0.951555872	1.94E-06
TUCP_002568	XLOC_108859	ENSRNOT00000078102	0.968873466	2.18E-07
TUCP_002568	XLOC_108859	ENSRNOT00000076611	-0.954644192	1.40E-06
TUCP_002568	XLOC_108859	ENSRNOT00000085403	0.952779194	1.71E-06
TUCP_002568	XLOC_108859	ENSRNOT00000008982	0.956564824	1.13E-06
TUCP_002568	XLOC_108859	ENSRNOT00000025372	0.954601843	1.41E-06
TUCP_002568	XLOC_108859	ENSRNOT00000040476	0.952873002	1.69E-06
TUCP_002568	XLOC_108859	ENSRNOT00000016519	0.951579462	1.93E-06
TUCP_002568	XLOC_108859	ENSRNOT00000079917	0.959289411	8.22E-07
TUCP_002568	XLOC_108859	ENSRNOT00000004319	-0.954938658	1.36E-06
TUCP_002568	XLOC_108859	ENSRNOT00000058842	0.956192416	1.18E-06
TUCP_002568	XLOC_108859	ENSRNOT00000011682	-0.971422082	1.43E-07
TUCP_002568	XLOC_108859	ENSRNOT00000084422	0.9548846	1.36E-06
TUCP_002568	XLOC_108859	ENSRNOT00000079177	0.950293727	2.20E-06
TUCP_002568	XLOC_108859	ENSRNOT00000031568	0.975416149	6.79E-08
TUCP_003370	XLOC_146008	ENSRNOT00000085465	0.95298148	1.67E-06
TUCP_003370	XLOC_146008	ENSRNOT00000052002	-0.950189132	2.22E-06

TUCP_003370	XLOC_146008	ENSRNOT00000047126	0.957099758	1.06E-06
TUCP_003370	XLOC_146008	ENSRNOT00000068452	-0.962762882	5.30E-07
TUCP_003370	XLOC_146008	ENSRNOT00000048910	0.955594383	1.26E-06
TUCP_003370	XLOC_146008	ENSRNOT00000007702	0.975162939	7.14E-08
TUCP_003370	XLOC_146008	ENSRNOT00000012760	0.976308579	5.65E-08
TUCP_003370	XLOC_146008	ENSRNOT00000080613	0.951261716	2.00E-06
TUCP_003370	XLOC_146008	ENSRNOT00000030109	-0.961358413	6.36E-07
TUCP_003370	XLOC_146008	ENSRNOT00000086995	-0.955508097	1.27E-06
TUCP_003370	XLOC_146008	ENSRNOT00000034609	-0.950865965	2.08E-06
TUCP_003370	XLOC_146008	ENSRNOT00000003891	-0.959248247	8.27E-07
TUCP_003370	XLOC_146008	ENSRNOT00000081725	-0.960247749	7.31E-07
TUCP_004353	XLOC_192311	ENSRNOT00000072818	0.953003032	1.67E-06
TUCP_002663	XLOC_112907	ENSRNOT00000026633	-0.964639591	4.10E-07
TUCP_002663	XLOC_112907	ENSRNOT00000001215	-0.95529382	1.30E-06
TUCP_002663	XLOC_112907	ENSRNOT00000074583	0.953473645	1.59E-06
TUCP_002663	XLOC_112907	ENSRNOT00000010835	-0.971792435	1.34E-07
TUCP_002663	XLOC_112907	ENSRNOT00000081123	-0.960950078	6.70E-07
TUCP_002663	XLOC_112907	ENSRNOT00000081353	-0.955315215	1.30E-06
TUCP_002663	XLOC_112907	ENSRNOT00000065103	0.95228215	1.80E-06
TUCP_002663	XLOC_112907	ENSRNOT00000087080	-0.950700889	2.11E-06
TUCP_002663	XLOC_112907	ENSRNOT00000013768	0.955783552	1.24E-06
TUCP_002663	XLOC_112907	ENSRNOT00000057965	-0.966847081	2.98E-07
TUCP_002663	XLOC_112907	ENSRNOT00000091282	0.983345752	9.81E-09
TUCP_002663	XLOC_112907	ENSRNOT00000002443	-0.959731777	7.79E-07
TUCP_002663	XLOC_112907	ENSRNOT00000013058	-0.960811582	6.81E-07
TUCP_002663	XLOC_112907	ENSRNOT00000077479	0.955627898	1.26E-06
TUCP_002663	XLOC_112907	ENSRNOT00000086798	0.973455006	9.93E-08
TUCP_002663	XLOC_112907	ENSRNOT00000030749	0.965919383	3.42E-07
TUCP_002663	XLOC_112907	ENSRNOT00000015434	-0.956562201	1.13E-06
TUCP_002663	XLOC_112907	ENSRNOT00000006111	0.956570342	1.13E-06
TUCP_002663	XLOC_112907	ENSRNOT00000038178	0.956205584	1.18E-06
TUCP_002663	XLOC_112907	ENSRNOT00000014560	0.968730324	2.23E-07
TUCP_002663	XLOC_112907	ENSRNOT00000052002	-0.957760419	9.86E-07
TUCP_002663	XLOC_112907	ENSRNOT00000005459	-0.950168956	2.23E-06
TUCP_002663	XLOC_112907	ENSRNOT00000045326	-0.952430573	1.77E-06
TUCP_002663	XLOC_112907	ENSRNOT00000025222	0.957818729	9.80E-07
TUCP_002663	XLOC_112907	ENSRNOT00000029660	-0.956883816	1.09E-06
TUCP_002663	XLOC_112907	ENSRNOT00000029510	-0.956723065	1.11E-06
TUCP_002663	XLOC_112907	ENSRNOT00000020951	-0.964367429	4.26E-07
TUCP_002663	XLOC_112907	ENSRNOT00000076467	0.968565054	2.29E-07
TUCP_002663	XLOC_112907	ENSRNOT00000078856	0.95934474	8.17E-07
TUCP_002663	XLOC_112907	ENSRNOT00000004290	-0.974443548	8.23E-08
TUCP_002663	XLOC_112907	ENSRNOT00000023619	-0.952606739	1.74E-06
TUCP_002663	XLOC_112907	ENSRNOT00000009742	0.95034937	2.19E-06
TUCP_002663	XLOC_112907	ENSRNOT00000008104	-0.955168431	1.32E-06
TUCP_002663	XLOC_112907	ENSRNOT00000030850	-0.956075166	1.20E-06
TUCP_002663	XLOC_112907	ENSRNOT00000038546	0.952773687	1.71E-06
TUCP_002663	XLOC_112907	ENSRNOT00000001679	-0.962280237	5.64E-07
TUCP_002663	XLOC_112907	ENSRNOT00000074212	-0.980945632	1.92E-08
TUCP_002663	XLOC_112907	ENSRNOT00000068452	-0.954973118	1.35E-06
TUCP_002663	XLOC_112907	ENSRNOT00000091325	0.953543353	1.58E-06

TUCP_002663	XLOC_112907	ENSRNOT00000083268	0.950871418	2.08E-06
TUCP_002663	XLOC_112907	ENSRNOT00000012996	-0.965445258	3.66E-07
TUCP_002663	XLOC_112907	ENSRNOT00000021729	-0.971210698	1.48E-07
TUCP_002663	XLOC_112907	ENSRNOT00000066776	0.959688585	7.83E-07
TUCP_002663	XLOC_112907	ENSRNOT00000050868	-0.950489525	2.16E-06
TUCP_002663	XLOC_112907	ENSRNOT00000050350	0.968036678	2.49E-07
TUCP_002663	XLOC_112907	ENSRNOT00000012314	0.963045431	5.10E-07
TUCP_002663	XLOC_112907	ENSRNOT00000010399	-0.958953191	8.56E-07
TUCP_002663	XLOC_112907	ENSRNOT00000015092	-0.950843166	2.08E-06
TUCP_002663	XLOC_112907	ENSRNOT00000073019	-0.960923715	6.72E-07
TUCP_002663	XLOC_112907	ENSRNOT00000020005	-0.950307781	2.19E-06
TUCP_002663	XLOC_112907	ENSRNOT00000084031	-0.955491273	1.28E-06
TUCP_002663	XLOC_112907	ENSRNOT00000004956	-0.956557756	1.13E-06
TUCP_002663	XLOC_112907	ENSRNOT00000030109	-0.976942529	4.94E-08
TUCP_002663	XLOC_112907	ENSRNOT00000009319	0.953158121	1.64E-06
TUCP_002663	XLOC_112907	ENSRNOT00000024033	-0.959956077	7.58E-07
TUCP_002663	XLOC_112907	ENSRNOT00000021578	-0.950949372	2.06E-06
TUCP_002663	XLOC_112907	ENSRNOT00000013191	0.95423189	1.46E-06
TUCP_002663	XLOC_112907	ENSRNOT00000028066	0.954156432	1.48E-06
TUCP_002663	XLOC_112907	ENSRNOT00000064667	0.976010506	6.01E-08
TUCP_002663	XLOC_112907	ENSRNOT00000090774	0.962586474	5.42E-07
TUCP_002663	XLOC_112907	ENSRNOT00000038066	-0.966813959	3.00E-07
TUCP_002663	XLOC_112907	ENSRNOT00000025252	0.9685543	2.30E-07
TUCP_002663	XLOC_112907	ENSRNOT00000077803	-0.965336315	3.72E-07
TUCP_002663	XLOC_112907	ENSRNOT00000050415	0.956270323	1.17E-06
TUCP_002663	XLOC_112907	ENSRNOT00000067009	0.956971899	1.08E-06
TUCP_002663	XLOC_112907	ENSRNOT00000040847	0.963038051	5.11E-07
TUCP_002663	XLOC_112907	ENSRNOT00000077274	0.953035544	1.66E-06
TUCP_002068	XLOC_084778	ENSRNOT00000039045	0.952282197	1.80E-06
TUCP_002068	XLOC_084778	ENSRNOT00000004249	0.96131419	6.39E-07
TUCP_002068	XLOC_084778	ENSRNOT00000001682	0.95069833	2.11E-06
TUCP_002068	XLOC_084778	ENSRNOT00000014988	0.971202424	1.49E-07
TUCP_002068	XLOC_084778	ENSRNOT00000022573	-0.951075166	2.03E-06
TUCP_002106	XLOC_087136	ENSRNOT00000006429	0.950051324	2.25E-06
TUCP_002106	XLOC_087136	ENSRNOT00000009111	0.951858415	1.88E-06
TUCP_002106	XLOC_087136	ENSRNOT00000054915	0.952138235	1.82E-06
TUCP_002106	XLOC_087136	ENSRNOT00000004387	0.957881614	9.72E-07
TUCP_002106	XLOC_087136	ENSRNOT00000020174	-0.958513077	9.03E-07
TUCP_002106	XLOC_087136	ENSRNOT00000046594	0.960366701	7.21E-07
TUCP_002106	XLOC_087136	ENSRNOT00000083414	0.954348803	1.45E-06
TUCP_002106	XLOC_087136	ENSRNOT00000088581	0.955362158	1.29E-06
TUCP_002106	XLOC_087136	ENSRNOT00000064588	0.956197618	1.18E-06
TUCP_002105	XLOC_087128	ENSRNOT00000042432	0.982955485	1.10E-08
TUCP_002105	XLOC_087128	ENSRNOT00000093696	0.953425354	1.60E-06
TUCP_002105	XLOC_087128	ENSRNOT00000029865	0.953671802	1.55E-06
TUCP_002105	XLOC_087128	ENSRNOT00000007657	0.952423074	1.77E-06
TUCP_003593	XLOC_158142	ENSRNOT00000012413	0.955769461	1.24E-06
TUCP_003593	XLOC_158142	ENSRNOT00000026153	-0.959862791	7.67E-07
TUCP_003593	XLOC_158142	ENSRNOT00000088503	-0.974321941	8.42E-08
TUCP_003592	XLOC_158102	ENSRNOT00000036565	0.95245552	1.77E-06
TUCP_003592	XLOC_158102	ENSRNOT00000093075	0.988421647	1.61E-09

TUCP_003592	XLOC_158102	ENSRNOT00000058260	0.988421647	1.61E-09
TUCP_003592	XLOC_158102	ENSRNOT00000088887	0.988421647	1.61E-09
TUCP_003592	XLOC_158102	ENSRNOT00000084986	0.988421647	1.61E-09
TUCP_003592	XLOC_158102	ENSRNOT00000074346	0.985664014	4.66E-09
TUCP_003592	XLOC_158102	ENSRNOT00000073209	0.99829721	1.12E-13
TUCP_003592	XLOC_158102	ENSRNOT00000080155	0.967587561	2.67E-07
TUCP_003592	XLOC_158102	ENSRNOT00000072573	0.981000976	1.89E-08
TUCP_003592	XLOC_158102	ENSRNOT00000017828	0.983114017	1.05E-08
TUCP_003592	XLOC_158102	ENSRNOT00000080444	0.971369219	1.44E-07
TUCP_003592	XLOC_158102	ENSRNOT00000071312	0.988421647	1.61E-09
TUCP_003592	XLOC_158102	ENSRNOT00000088245	0.988421647	1.61E-09
TUCP_003592	XLOC_158102	ENSRNOT00000058833	0.986328927	3.68E-09
TUCP_003592	XLOC_158102	ENSRNOT00000078052	0.988421647	1.61E-09
TUCP_003592	XLOC_158102	ENSRNOT00000078494	0.988166365	1.79E-09
TUCP_003592	XLOC_158102	ENSRNOT00000085653	0.988421647	1.61E-09
TUCP_003592	XLOC_158102	ENSRNOT00000057950	0.957350268	1.03E-06
TUCP_003592	XLOC_158102	ENSRNOT00000081992	0.984791723	6.25E-09
TUCP_003592	XLOC_158102	ENSRNOT00000038553	0.963451635	4.83E-07
TUCP_003592	XLOC_158102	ENSRNOT00000055406	0.988421647	1.61E-09
TUCP_003592	XLOC_158102	ENSRNOT00000070817	0.98671254	3.19E-09
TUCP_003592	XLOC_158102	ENSRNOT00000026753	0.988421647	1.61E-09
TUCP_003592	XLOC_158102	ENSRNOT00000080533	0.983907676	8.27E-09
TUCP_003592	XLOC_158102	ENSRNOT00000072774	0.965278013	3.75E-07
TUCP_003592	XLOC_158102	ENSRNOT00000086031	0.988421647	1.61E-09
TUCP_003592	XLOC_158102	ENSRNOT00000014312	0.988421647	1.61E-09
TUCP_003592	XLOC_158102	ENSRNOT00000067655	0.970462546	1.69E-07
TUCP_003592	XLOC_158102	ENSRNOT00000051231	0.987082274	2.77E-09
TUCP_003592	XLOC_158102	ENSRNOT00000089407	0.979729379	2.61E-08
TUCP_003592	XLOC_158102	ENSRNOT00000055034	0.987841696	2.05E-09
TUCP_003592	XLOC_158102	ENSRNOT00000046837	0.959812805	7.72E-07
TUCP_003592	XLOC_158102	ENSRNOT00000093612	0.96108055	6.59E-07
TUCP_002101	XLOC_086877	ENSRNOT00000026966	0.970322306	1.73E-07
TUCP_002101	XLOC_086877	ENSRNOT00000068416	0.999999782	3.88E-33
TUCP_002101	XLOC_086877	ENSRNOT00000086036	0.999958274	9.96E-22
TUCP_002101	XLOC_086877	ENSRNOT00000026803	0.985046075	5.74E-09
TUCP_002101	XLOC_086877	ENSRNOT00000047527	0.963071698	5.08E-07
TUCP_002101	XLOC_086877	ENSRNOT00000042251	0.999344295	9.53E-16
TUCP_002101	XLOC_086877	ENSRNOT00000002984	0.998397069	8.31E-14
TUCP_002101	XLOC_086877	ENSRNOT00000083764	0.999541808	1.59E-16
TUCP_002101	XLOC_086877	ENSRNOT00000076802	0.962695843	5.34E-07
TUCP_002101	XLOC_086877	ENSRNOT00000092365	0.999161651	3.26E-15
TUCP_002101	XLOC_086877	ENSRNOT00000079275	0.999901911	7.15E-20
TUCP_002101	XLOC_086877	ENSRNOT00000066062	-0.959706712	7.82E-07
TUCP_002101	XLOC_086877	ENSRNOT00000045301	1	0
TUCP_002101	XLOC_086877	ENSRNOT00000041186	0.999975782	6.56E-23
TUCP_002101	XLOC_086877	ENSRNOT00000010092	0.977711778	4.17E-08
TUCP_002101	XLOC_086877	ENSRNOT00000076182	1	0
TUCP_002101	XLOC_086877	ENSRNOT00000087404	0.951157664	2.02E-06
TUCP_002101	XLOC_086877	ENSRNOT00000052003	0.95837331	9.18E-07
TUCP_002101	XLOC_086877	ENSRNOT00000081709	0.998485154	6.27E-14
TUCP_002101	XLOC_086877	ENSRNOT00000005726	0.959610119	7.91E-07

TUCP_002101	XLOC_086877	ENSRNOT00000014103	1	0
TUCP_002101	XLOC_086877	ENSRNOT00000057433	0.964930601	3.94E-07
TUCP_002101	XLOC_086877	ENSRNOT00000082289	0.998931566	1.09E-14
TUCP_002101	XLOC_086877	ENSRNOT00000092516	0.957459323	1.02E-06
TUCP_002101	XLOC_086877	ENSRNOT00000008898	0.990039768	7.59E-10
TUCP_002101	XLOC_086877	ENSRNOT00000083493	0.995461232	1.51E-11
TUCP_002101	XLOC_086877	ENSRNOT00000092563	0.987201084	2.65E-09
TUCP_002101	XLOC_086877	ENSRNOT00000088893	1	0
TUCP_002101	XLOC_086877	ENSRNOT00000076693	0.955029582	1.34E-06
TUCP_002101	XLOC_086877	ENSRNOT00000081014	1	0
TUCP_002101	XLOC_086877	ENSRNOT00000067543	1	0
TUCP_002101	XLOC_086877	ENSRNOT00000050968	0.996816433	2.56E-12
TUCP_002101	XLOC_086877	ENSRNOT00000042201	0.960235595	7.32E-07
TUCP_002101	XLOC_086877	ENSRNOT00000004373	0.957315473	1.04E-06
TUCP_002101	XLOC_086877	ENSRNOT00000063929	1	0
TUCP_002101	XLOC_086877	ENSRNOT00000057472	1	0
TUCP_002101	XLOC_086877	ENSRNOT00000041617	0.952449765	1.77E-06
TUCP_002101	XLOC_086877	ENSRNOT00000091763	0.986310385	3.70E-09
TUCP_002101	XLOC_086877	ENSRNOT00000022200	0.998491303	6.14E-14
TUCP_002101	XLOC_086877	ENSRNOT00000067379	0.997260967	1.21E-12
TUCP_002101	XLOC_086877	ENSRNOT00000020786	0.976428448	5.51E-08
TUCP_002101	XLOC_086877	ENSRNOT00000050543	0.999722223	1.30E-17
TUCP_002101	XLOC_086877	ENSRNOT00000089306	0.999866649	3.32E-19
TUCP_002101	XLOC_086877	ENSRNOT00000052367	0.997268394	1.19E-12
TUCP_002101	XLOC_086877	ENSRNOT00000090658	1	0
TUCP_002101	XLOC_086877	ENSRNOT00000022571	0.995290013	1.81E-11
TUCP_002101	XLOC_086877	ENSRNOT00000044520	0.981817158	1.52E-08
TUCP_002101	XLOC_086877	ENSRNOT00000065448	0.963648868	4.70E-07
TUCP_002100	XLOC_086877	ENSRNOT00000026966	0.970322306	1.73E-07
TUCP_002100	XLOC_086877	ENSRNOT00000068416	0.999999782	3.88E-33
TUCP_002100	XLOC_086877	ENSRNOT00000086036	0.999958274	9.96E-22
TUCP_002100	XLOC_086877	ENSRNOT00000026803	0.985046075	5.74E-09
TUCP_002100	XLOC_086877	ENSRNOT00000047527	0.963071698	5.08E-07
TUCP_002100	XLOC_086877	ENSRNOT00000042251	0.999344295	9.53E-16
TUCP_002100	XLOC_086877	ENSRNOT00000002984	0.998397069	8.31E-14
TUCP_002100	XLOC_086877	ENSRNOT00000083764	0.999541808	1.59E-16
TUCP_002100	XLOC_086877	ENSRNOT00000076802	0.962695843	5.34E-07
TUCP_002100	XLOC_086877	ENSRNOT00000092365	0.999161651	3.26E-15
TUCP_002100	XLOC_086877	ENSRNOT00000079275	0.999901911	7.15E-20
TUCP_002100	XLOC_086877	ENSRNOT00000066062	-0.959706712	7.82E-07
TUCP_002100	XLOC_086877	ENSRNOT00000045301	1	0
TUCP_002100	XLOC_086877	ENSRNOT00000041186	0.999975782	6.56E-23
TUCP_002100	XLOC_086877	ENSRNOT00000010092	0.977711778	4.17E-08
TUCP_002100	XLOC_086877	ENSRNOT00000076182	1	0
TUCP_002100	XLOC_086877	ENSRNOT00000087404	0.951157664	2.02E-06
TUCP_002100	XLOC_086877	ENSRNOT00000052003	0.95837331	9.18E-07
TUCP_002100	XLOC_086877	ENSRNOT00000081709	0.998485154	6.27E-14
TUCP_002100	XLOC_086877	ENSRNOT00000005726	0.959610119	7.91E-07
TUCP_002100	XLOC_086877	ENSRNOT00000014103	1	4.25E-78
TUCP_002100	XLOC_086877	ENSRNOT00000057433	0.964930601	3.94E-07
TUCP_002100	XLOC_086877	ENSRNOT00000082289	0.998931566	1.09E-14

TUCP_002100	XLOC_086877	ENSRNOT00000092516	0.957459323	1.02E-06
TUCP_002100	XLOC_086877	ENSRNOT00000008898	0.990039768	7.59E-10
TUCP_002100	XLOC_086877	ENSRNOT00000083493	0.995461232	1.51E-11
TUCP_002100	XLOC_086877	ENSRNOT00000092563	0.987201084	2.65E-09
TUCP_002100	XLOC_086877	ENSRNOT00000088893	1	0
TUCP_002100	XLOC_086877	ENSRNOT00000076693	0.955029582	1.34E-06
TUCP_002100	XLOC_086877	ENSRNOT00000081014	1	0
TUCP_002100	XLOC_086877	ENSRNOT00000067543	1	0
TUCP_002100	XLOC_086877	ENSRNOT00000050968	0.996816433	2.56E-12
TUCP_002100	XLOC_086877	ENSRNOT00000042201	0.960235595	7.32E-07
TUCP_002100	XLOC_086877	ENSRNOT00000004373	0.957315473	1.04E-06
TUCP_002100	XLOC_086877	ENSRNOT00000063929	1	0
TUCP_002100	XLOC_086877	ENSRNOT00000057472	1	0
TUCP_002100	XLOC_086877	ENSRNOT00000041617	0.952449765	1.77E-06
TUCP_002100	XLOC_086877	ENSRNOT00000091763	0.986310385	3.70E-09
TUCP_002100	XLOC_086877	ENSRNOT00000022200	0.998491303	6.14E-14
TUCP_002100	XLOC_086877	ENSRNOT00000067379	0.997260967	1.21E-12
TUCP_002100	XLOC_086877	ENSRNOT00000020786	0.976428448	5.51E-08
TUCP_002100	XLOC_086877	ENSRNOT00000050543	0.999722223	1.30E-17
TUCP_002100	XLOC_086877	ENSRNOT00000089306	0.999866649	3.32E-19
TUCP_002100	XLOC_086877	ENSRNOT00000052367	0.997268394	1.19E-12
TUCP_002100	XLOC_086877	ENSRNOT00000090658	1	0
TUCP_002100	XLOC_086877	ENSRNOT00000022571	0.995290013	1.81E-11
TUCP_002100	XLOC_086877	ENSRNOT00000044520	0.981817158	1.52E-08
TUCP_002100	XLOC_086877	ENSRNOT00000065448	0.963648868	4.70E-07
TUCP_002060	XLOC_084113	ENSRNOT00000081292	0.957040748	1.07E-06
TUCP_002066	XLOC_084467	ENSRNOT00000036143	0.965111737	3.84E-07
TUCP_001479	XLOC_057126	ENSRNOT00000087287	0.960840975	6.79E-07
TUCP_001479	XLOC_057126	ENSRNOT00000055536	0.97062327	1.64E-07
TUCP_001479	XLOC_057126	ENSRNOT00000083256	0.96542042	3.67E-07
TUCP_001479	XLOC_057126	ENSRNOT00000016088	0.977865924	4.03E-08
TUCP_001479	XLOC_057126	ENSRNOT00000009975	0.962245	5.67E-07
TUCP_001479	XLOC_057126	ENSRNOT00000090700	0.96413268	4.40E-07
TUCP_001479	XLOC_057126	ENSRNOT00000081226	0.974128275	8.74E-08
TUCP_001479	XLOC_057126	ENSRNOT00000001642	0.965092306	3.85E-07
TUCP_001479	XLOC_057126	ENSRNOT00000057738	0.953533378	1.58E-06
TUCP_001479	XLOC_057126	ENSRNOT00000027544	0.97972511	2.61E-08
TUCP_001479	XLOC_057126	ENSRNOT00000081631	0.95208073	1.84E-06
TUCP_001479	XLOC_057126	ENSRNOT00000013343	0.95249956	1.76E-06
TUCP_001479	XLOC_057126	ENSRNOT00000012386	0.963286762	4.94E-07
TUCP_001479	XLOC_057126	ENSRNOT00000065897	0.967949099	2.52E-07
TUCP_001479	XLOC_057126	ENSRNOT00000083594	0.954610288	1.41E-06
TUCP_001479	XLOC_057126	ENSRNOT00000073493	0.973382757	1.01E-07
TUCP_001479	XLOC_057126	ENSRNOT00000005770	0.973790875	9.32E-08
TUCP_001479	XLOC_057126	ENSRNOT00000011589	0.961780445	6.02E-07
TUCP_001479	XLOC_057126	ENSRNOT00000027752	0.960783984	6.84E-07
TUCP_001479	XLOC_057126	ENSRNOT00000057953	0.965119908	3.83E-07
TUCP_001479	XLOC_057126	ENSRNOT00000022255	0.965597189	3.58E-07
TUCP_001479	XLOC_057126	ENSRNOT00000092066	-0.960741518	6.88E-07
TUCP_001479	XLOC_057126	ENSRNOT00000016485	0.974564443	8.03E-08
TUCP_001479	XLOC_057126	ENSRNOT00000047139	0.957482826	1.02E-06

TUCP_001479	XLOC_057126	ENSRNOT00000085334	0.977015183	4.86E-08
TUCP_001479	XLOC_057126	ENSRNOT00000035427	0.961444185	6.29E-07
TUCP_001479	XLOC_057126	ENSRNOT00000081604	0.961480621	6.26E-07
TUCP_001479	XLOC_057126	ENSRNOT00000043827	0.966400732	3.19E-07
TUCP_001479	XLOC_057126	ENSRNOT00000061054	0.972634486	1.15E-07
TUCP_001479	XLOC_057126	ENSRNOT00000077578	-0.958673476	8.86E-07
TUCP_001479	XLOC_057126	ENSRNOT00000082904	0.956232898	1.18E-06
TUCP_001479	XLOC_057126	ENSRNOT00000008764	0.97302099	1.08E-07
TUCP_001479	XLOC_057126	ENSRNOT00000012757	0.97767328	4.21E-08
TUCP_001479	XLOC_057126	ENSRNOT00000009009	0.953178195	1.64E-06
TUCP_001479	XLOC_057126	ENSRNOT00000037902	0.970293342	1.73E-07
TUCP_001479	XLOC_057126	ENSRNOT00000077060	0.960253453	7.31E-07
TUCP_001479	XLOC_057126	ENSRNOT00000035309	-0.970631193	1.64E-07
TUCP_001479	XLOC_057126	ENSRNOT00000026212	0.952955697	1.68E-06
TUCP_001479	XLOC_057126	ENSRNOT00000012247	0.97386218	9.20E-08
TUCP_001479	XLOC_057126	ENSRNOT00000083134	0.959748095	7.78E-07
TUCP_001479	XLOC_057126	ENSRNOT00000006109	0.974318602	8.43E-08
TUCP_001479	XLOC_057126	ENSRNOT00000052033	-0.960699222	6.91E-07
TUCP_001479	XLOC_057126	ENSRNOT00000038449	0.967480604	2.71E-07
TUCP_001479	XLOC_057126	ENSRNOT00000065629	0.952168763	1.82E-06
TUCP_001479	XLOC_057126	ENSRNOT00000025355	0.96966787	1.92E-07
TUCP_001476	XLOC_056833	ENSRNOT00000074583	0.992436233	1.93E-10
TUCP_001476	XLOC_056833	ENSRNOT00000032802	0.960362851	7.21E-07
TUCP_001476	XLOC_056833	ENSRNOT00000003759	0.962214384	5.69E-07
TUCP_001476	XLOC_056833	ENSRNOT00000089265	0.951759169	1.90E-06
TUCP_001476	XLOC_056833	ENSRNOT00000028066	0.983307197	9.93E-09
TUCP_001476	XLOC_056833	ENSRNOT00000013182	0.963010148	5.13E-07
TUCP_001476	XLOC_056833	ENSRNOT00000091282	0.966840221	2.99E-07
TUCP_001476	XLOC_056833	ENSRNOT00000004713	0.982313008	1.32E-08
TUCP_001476	XLOC_056833	ENSRNOT00000068354	0.951887965	1.87E-06
TUCP_001476	XLOC_056833	ENSRNOT00000022167	0.969894199	1.85E-07
TUCP_001476	XLOC_056833	ENSRNOT00000050099	0.950401345	2.17E-06
TUCP_001476	XLOC_056833	ENSRNOT00000011509	0.965617445	3.57E-07
TUCP_001476	XLOC_056833	ENSRNOT00000050350	0.962250618	5.67E-07
TUCP_001476	XLOC_056833	ENSRNOT00000014560	0.980478946	2.16E-08
TUCP_001476	XLOC_056833	ENSRNOT00000024069	0.951089105	2.03E-06
TUCP_001476	XLOC_056833	ENSRNOT00000009448	0.953293564	1.62E-06
TUCP_001476	XLOC_056833	ENSRNOT00000010580	0.956687792	1.12E-06
TUCP_001476	XLOC_056833	ENSRNOT00000025222	0.953530801	1.58E-06
TUCP_001476	XLOC_056833	ENSRNOT00000021359	0.954929485	1.36E-06
TUCP_001476	XLOC_056833	ENSRNOT00000010050	0.965683818	3.54E-07
TUCP_001476	XLOC_056833	ENSRNOT00000001110	0.951075748	2.03E-06
TUCP_001476	XLOC_056833	ENSRNOT00000003778	0.985487745	4.95E-09
TUCP_001476	XLOC_056833	ENSRNOT00000013809	0.95658715	1.13E-06
TUCP_001476	XLOC_056833	ENSRNOT00000051067	0.966819255	3.00E-07
TUCP_001476	XLOC_056833	ENSRNOT00000076879	0.950055857	2.25E-06
TUCP_001476	XLOC_056833	ENSRNOT00000038546	0.973582027	9.69E-08
TUCP_001476	XLOC_056833	ENSRNOT00000080163	0.955334914	1.30E-06
TUCP_001476	XLOC_056833	ENSRNOT00000078299	0.955980462	1.21E-06
TUCP_001476	XLOC_056833	ENSRNOT00000006196	0.955335645	1.30E-06
TUCP_001476	XLOC_056833	ENSRNOT00000077479	0.954692653	1.39E-06

TUCP_001476	XLOC_056833	ENSRNOT00000012963	0.952545942	1.75E-06
TUCP_001476	XLOC_056833	ENSRNOT00000077119	0.960503854	7.08E-07
TUCP_001476	XLOC_056833	ENSRNOT00000083452	0.951006948	2.05E-06
TUCP_001476	XLOC_056833	ENSRNOT00000075936	0.962923842	5.18E-07
TUCP_001476	XLOC_056833	ENSRNOT00000060300	0.956861697	1.09E-06
TUCP_001476	XLOC_056833	ENSRNOT00000066776	0.956192881	1.18E-06
TUCP_001476	XLOC_056833	ENSRNOT00000012314	0.982572815	1.23E-08
TUCP_001476	XLOC_056833	ENSRNOT00000023416	0.964380692	4.25E-07
TUCP_001476	XLOC_056833	ENSRNOT00000082393	0.980820851	1.98E-08
TUCP_001476	XLOC_056833	ENSRNOT00000040736	0.987952323	1.96E-09
TUCP_001476	XLOC_056833	ENSRNOT00000014533	0.964818049	4.00E-07
TUCP_001476	XLOC_056833	ENSRNOT00000032236	0.953354411	1.61E-06
TUCP_001476	XLOC_056833	ENSRNOT00000000637	0.968632218	2.27E-07
TUCP_001476	XLOC_056833	ENSRNOT00000029184	0.955088078	1.33E-06
TUCP_001476	XLOC_056833	ENSRNOT00000073138	0.963951319	4.51E-07
TUCP_001476	XLOC_056833	ENSRNOT00000013989	0.952534742	1.75E-06
TUCP_001476	XLOC_056833	ENSRNOT00000022128	0.970616952	1.64E-07
TUCP_001476	XLOC_056833	ENSRNOT00000070792	0.973075081	1.07E-07
TUCP_001476	XLOC_056833	ENSRNOT00000013191	0.983129534	1.05E-08
TUCP_001476	XLOC_056833	ENSRNOT00000045107	0.955338566	1.30E-06
TUCP_001476	XLOC_056833	ENSRNOT00000050415	0.966280202	3.24E-07
TUCP_001476	XLOC_056833	ENSRNOT00000038610	0.952752468	1.71E-06
TUCP_001476	XLOC_056833	ENSRNOT00000090774	0.990453833	6.14E-10
TUCP_001476	XLOC_056833	ENSRNOT00000019389	0.95165045	1.92E-06
TUCP_001477	XLOC_057009	ENSRNOT00000084799	0.958905498	8.61E-07
TUCP_001477	XLOC_057009	ENSRNOT00000082043	0.970956564	1.55E-07
TUCP_001477	XLOC_057009	ENSRNOT00000090080	0.950507386	2.15E-06
TUCP_001477	XLOC_057009	ENSRNOT00000068444	0.975326034	6.91E-08
TUCP_001477	XLOC_057009	ENSRNOT00000081370	0.976343599	5.61E-08
TUCP_001477	XLOC_057009	ENSRNOT00000078645	0.954956273	1.35E-06
TUCP_001477	XLOC_057009	ENSRNOT00000075987	0.952626085	1.74E-06
TUCP_001477	XLOC_057009	ENSRNOT00000074990	0.975326034	6.91E-08
TUCP_001477	XLOC_057009	ENSRNOT00000087323	0.974471788	8.18E-08
TUCP_001477	XLOC_057009	ENSRNOT00000074930	0.975326034	6.91E-08
TUCP_001477	XLOC_057009	ENSRNOT00000080575	0.975314852	6.93E-08
TUCP_001477	XLOC_057009	ENSRNOT00000078369	0.955154323	1.32E-06
TUCP_001477	XLOC_057009	ENSRNOT00000090207	0.950785217	2.09E-06
TUCP_001477	XLOC_057009	ENSRNOT00000088367	0.975326034	6.91E-08
TUCP_001477	XLOC_057009	ENSRNOT00000081996	0.975326034	6.91E-08
TUCP_001477	XLOC_057009	ENSRNOT00000064751	0.955650915	1.25E-06
TUCP_001477	XLOC_057009	ENSRNOT00000076699	0.951638918	1.92E-06
TUCP_001477	XLOC_057009	ENSRNOT00000082058	0.975326034	6.91E-08
TUCP_001477	XLOC_057009	ENSRNOT00000088905	0.975326034	6.91E-08
TUCP_001477	XLOC_057009	ENSRNOT00000086831	0.975326034	6.91E-08
TUCP_001477	XLOC_057009	ENSRNOT00000045099	0.975326034	6.91E-08
TUCP_001477	XLOC_057009	ENSRNOT00000085849	0.963955483	4.51E-07
TUCP_001477	XLOC_057009	ENSRNOT00000086397	0.954447303	1.43E-06
TUCP_001477	XLOC_057009	ENSRNOT00000077789	0.961610935	6.16E-07
TUCP_001477	XLOC_057009	ENSRNOT00000092982	0.975326034	6.91E-08
TUCP_001477	XLOC_057009	ENSRNOT00000087025	0.975326034	6.91E-08
TUCP_001477	XLOC_057009	ENSRNOT00000019853	0.975326034	6.91E-08

TUCP_001477	XLOC_057009	ENSRNOT00000073853	0.956396839	1.15E-06
TUCP_001477	XLOC_057009	ENSRNOT00000065912	0.953092364	1.65E-06
TUCP_001477	XLOC_057009	ENSRNOT00000093083	0.972895728	1.10E-07
TUCP_001475	XLOC_056807	ENSRNOT00000081455	0.951375956	1.97E-06
TUCP_001475	XLOC_056807	ENSRNOT00000034090	0.960394627	7.18E-07
TUCP_001475	XLOC_056807	ENSRNOT00000026679	0.954659257	1.40E-06
TUCP_001472	XLOC_056807	ENSRNOT00000085110	0.976087919	5.91E-08
TUCP_001472	XLOC_056807	ENSRNOT00000008037	0.992783501	1.52E-10
TUCP_001470	XLOC_056727	ENSRNOT00000084500	0.984887141	6.05E-09
TUCP_001470	XLOC_056727	ENSRNOT00000093467	0.996134431	6.75E-12
TUCP_001470	XLOC_056727	ENSRNOT00000029340	0.999116572	4.23E-15
TUCP_001470	XLOC_056727	ENSRNOT00000077972	0.962758032	5.30E-07
TUCP_001470	XLOC_056727	ENSRNOT00000038646	1	4.25E-78
TUCP_001470	XLOC_056727	ENSRNOT00000012095	0.957750287	9.88E-07
TUCP_001470	XLOC_056727	ENSRNOT00000017909	0.975587192	6.56E-08
TUCP_001470	XLOC_056727	ENSRNOT00000092579	1	1.33E-79
TUCP_001470	XLOC_056727	ENSRNOT00000089235	0.998661162	3.38E-14
TUCP_001470	XLOC_056727	ENSRNOT00000036383	0.988460809	1.58E-09
TUCP_001470	XLOC_056727	ENSRNOT00000076528	0.997115373	1.57E-12
TUCP_001470	XLOC_056727	ENSRNOT00000075193	0.964326971	4.29E-07
TUCP_001470	XLOC_056727	ENSRNOT00000083034	0.990934646	4.75E-10
TUCP_001470	XLOC_056727	ENSRNOT00000089607	0.995091455	2.23E-11
TUCP_001470	XLOC_056727	ENSRNOT00000086014	0.960893638	6.74E-07
TUCP_001470	XLOC_056727	ENSRNOT00000054939	0.958021495	9.57E-07
TUCP_001470	XLOC_056727	ENSRNOT00000052315	0.983687082	8.85E-09
TUCP_001470	XLOC_056727	ENSRNOT00000088168	1	0
TUCP_001470	XLOC_056727	ENSRNOT00000046231	0.96225463	5.66E-07
TUCP_001470	XLOC_056727	ENSRNOT00000013459	0.979098017	3.03E-08
TUCP_001470	XLOC_056727	ENSRNOT00000078137	1	0
TUCP_001470	XLOC_056727	ENSRNOT00000077764	0.977143528	4.73E-08
TUCP_001470	XLOC_056727	ENSRNOT00000043661	0.966975951	2.93E-07
TUCP_001470	XLOC_056727	ENSRNOT00000092740	1	1.33E-79
TUCP_001470	XLOC_056727	ENSRNOT00000000117	0.990989174	4.61E-10
TUCP_001470	XLOC_056727	ENSRNOT00000071877	0.967113583	2.87E-07
TUCP_001470	XLOC_056727	ENSRNOT00000070958	1	1.33E-79
TUCP_001470	XLOC_056727	ENSRNOT00000076757	0.959205354	8.31E-07
TUCP_001470	XLOC_056727	ENSRNOT00000085310	0.965230243	3.78E-07
TUCP_001470	XLOC_056727	ENSRNOT00000047241	0.966576993	3.11E-07
TUCP_001470	XLOC_056727	ENSRNOT00000027268	1	1.33E-79
TUCP_001470	XLOC_056727	ENSRNOT00000004459	1	0
TUCP_001470	XLOC_056727	ENSRNOT00000048373	0.959096578	8.42E-07
TUCP_001470	XLOC_056727	ENSRNOT00000061516	0.987595648	2.27E-09
TUCP_001470	XLOC_056727	ENSRNOT00000088148	1	0
TUCP_001470	XLOC_056727	ENSRNOT00000088527	1	0
TUCP_001470	XLOC_056727	ENSRNOT00000078701	1	1.33E-79
TUCP_001470	XLOC_056727	ENSRNOT00000052308	0.962646127	5.38E-07
TUCP_001470	XLOC_056727	ENSRNOT00000021748	1	1.33E-79
TUCP_001470	XLOC_056727	ENSRNOT00000089085	1	1.33E-79
TUCP_001470	XLOC_056727	ENSRNOT00000008153	0.999703506	1.80E-17
TUCP_001470	XLOC_056727	ENSRNOT00000085896	0.971605382	1.39E-07
TUCP_001470	XLOC_056727	ENSRNOT00000079189	0.984972387	5.89E-09

TUCP_001470	XLOC_056727	ENSRNOT00000090078	1	0
TUCP_001470	XLOC_056727	ENSRNOT00000078020	0.958421666	9.13E-07
TUCP_001470	XLOC_056727	ENSRNOT00000092425	0.956092273	1.19E-06
TUCP_001470	XLOC_056727	ENSRNOT00000009776	1	1.33E-79
TUCP_000660	XLOC_025126	ENSRNOT00000072028	-0.954016964	1.50E-06
TUCP_003933	XLOC_172933	ENSRNOT00000085599	0.999938502	6.93E-21
TUCP_003933	XLOC_172933	ENSRNOT00000006689	0.9944189	4.22E-11
TUCP_003933	XLOC_172933	ENSRNOT00000081534	0.987999291	1.92E-09
TUCP_003933	XLOC_172933	ENSRNOT00000085301	0.99793679	2.93E-13
TUCP_003933	XLOC_172933	ENSRNOT00000038729	1	4.25E-78
TUCP_003933	XLOC_172933	ENSRNOT00000086385	1	0
TUCP_003933	XLOC_172933	ENSRNOT00000077695	0.96038616	7.19E-07
TUCP_003933	XLOC_172933	ENSRNOT00000078561	0.986503348	3.45E-09
TUCP_003933	XLOC_172933	ENSRNOT00000087613	1	1.33E-79
TUCP_003933	XLOC_172933	ENSRNOT00000026032	0.953050675	1.66E-06
TUCP_003933	XLOC_172933	ENSRNOT00000073350	0.9658754	3.44E-07
TUCP_003933	XLOC_172933	ENSRNOT00000023062	1	0
TUCP_003933	XLOC_172933	ENSRNOT00000058834	1	4.25E-78
TUCP_003933	XLOC_172933	ENSRNOT00000079420	1	0
TUCP_003933	XLOC_172933	ENSRNOT00000090761	1	0
TUCP_003933	XLOC_172933	ENSRNOT00000055923	1	0
TUCP_003933	XLOC_172933	ENSRNOT00000092416	0.963085379	5.07E-07
TUCP_003933	XLOC_172933	ENSRNOT00000071930	1	4.25E-78
TUCP_003933	XLOC_172933	ENSRNOT00000001996	0.974721715	7.79E-08
TUCP_003933	XLOC_172933	ENSRNOT00000076756	1	0
TUCP_003933	XLOC_172933	ENSRNOT00000081527	0.999420479	5.14E-16
TUCP_003933	XLOC_172933	ENSRNOT00000085067	0.960262084	7.30E-07
TUCP_003933	XLOC_172933	ENSRNOT00000091663	0.99642667	4.56E-12
TUCP_003933	XLOC_172933	ENSRNOT00000081780	1	0
TUCP_003933	XLOC_172933	ENSRNOT00000093689	0.953599805	1.57E-06
TUCP_003933	XLOC_172933	ENSRNOT00000089114	1	1.33E-79
TUCP_003933	XLOC_172933	ENSRNOT00000021609	0.977017895	4.86E-08
TUCP_003933	XLOC_172933	ENSRNOT00000073316	1	0
TUCP_003933	XLOC_172933	ENSRNOT00000086557	0.977413455	4.46E-08
TUCP_003933	XLOC_172933	ENSRNOT00000071663	1	0
TUCP_003933	XLOC_172933	ENSRNOT00000042860	1	1.33E-79
TUCP_003933	XLOC_172933	ENSRNOT00000072962	0.971287317	1.46E-07
TUCP_003933	XLOC_172933	ENSRNOT00000088421	1	0
TUCP_003933	XLOC_172933	ENSRNOT00000038898	1	4.25E-78
TUCP_003933	XLOC_172933	ENSRNOT00000015840	0.959452012	8.06E-07
TUCP_003933	XLOC_172933	ENSRNOT00000064936	0.985957557	4.20E-09
TUCP_003933	XLOC_172933	ENSRNOT00000042777	0.990642579	5.56E-10
TUCP_003933	XLOC_172933	ENSRNOT00000010029	1	0
TUCP_003109	XLOC_132831	ENSRNOT00000083199	0.965615256	3.57E-07
TUCP_001798	XLOC_074271	ENSRNOT00000055353	0.956431287	1.15E-06
TUCP_001419	XLOC_055356	ENSRNOT00000081570	0.952718917	1.72E-06
TUCP_001419	XLOC_055356	ENSRNOT00000091652	0.99190187	2.71E-10
TUCP_001419	XLOC_055356	ENSRNOT00000036564	0.988107914	1.84E-09
TUCP_001419	XLOC_055356	ENSRNOT00000000109	0.954585136	1.41E-06
TUCP_001419	XLOC_055356	ENSRNOT00000080865	0.994948838	2.57E-11
TUCP_001419	XLOC_055356	ENSRNOT00000073534	0.988306556	1.69E-09

TUCP_001419	XLOC_055356	ENSRNOT00000012161	0.985577802	4.80E-09
TUCP_001419	XLOC_055356	ENSRNOT00000087758	0.972826714	1.11E-07
TUCP_001419	XLOC_055356	ENSRNOT00000049832	0.969375698	2.02E-07
TUCP_001419	XLOC_055356	ENSRNOT00000093019	0.994948838	2.57E-11
TUCP_001419	XLOC_055356	ENSRNOT00000024825	0.970225213	1.75E-07
TUCP_001419	XLOC_055356	ENSRNOT00000059266	0.951110297	2.03E-06
TUCP_001419	XLOC_055356	ENSRNOT00000072734	0.956283353	1.17E-06
TUCP_001419	XLOC_055356	ENSRNOT00000085908	0.989430935	1.02E-09
TUCP_001419	XLOC_055356	ENSRNOT00000046744	0.994948838	2.57E-11
TUCP_001419	XLOC_055356	ENSRNOT00000076429	0.994948838	2.57E-11
TUCP_001419	XLOC_055356	ENSRNOT00000084667	0.995849616	9.63E-12
TUCP_001419	XLOC_055356	ENSRNOT00000055412	0.994948838	2.57E-11
TUCP_001419	XLOC_055356	ENSRNOT00000093201	0.99421963	5.03E-11
TUCP_001419	XLOC_055356	ENSRNOT00000072724	0.994948838	2.57E-11
TUCP_001419	XLOC_055356	ENSRNOT00000066017	0.981632746	1.60E-08
TUCP_001419	XLOC_055356	ENSRNOT00000082760	0.979655266	2.65E-08
TUCP_001419	XLOC_055356	ENSRNOT00000073148	0.976944301	4.94E-08
TUCP_001419	XLOC_055356	ENSRNOT00000077462	0.95244122	1.77E-06
TUCP_001419	XLOC_055356	ENSRNOT00000084375	0.994948838	2.57E-11
TUCP_001419	XLOC_055356	ENSRNOT00000006249	0.957686369	9.95E-07
TUCP_001419	XLOC_055356	ENSRNOT00000054858	0.97853328	3.46E-08
TUCP_001419	XLOC_055356	ENSRNOT00000042369	0.988907913	1.30E-09
TUCP_001419	XLOC_055356	ENSRNOT00000059326	0.950208414	2.22E-06
TUCP_001419	XLOC_055356	ENSRNOT00000086554	0.994948838	2.57E-11
TUCP_001419	XLOC_055356	ENSRNOT00000047921	0.994948838	2.57E-11
TUCP_001419	XLOC_055356	ENSRNOT00000064261	0.994789575	3.00E-11
TUCP_001419	XLOC_055356	ENSRNOT00000086660	0.992966857	1.34E-10
TUCP_001419	XLOC_055356	ENSRNOT00000080933	0.953109328	1.65E-06
TUCP_001419	XLOC_055356	ENSRNOT00000083977	0.994948838	2.57E-11
TUCP_001419	XLOC_055356	ENSRNOT00000092933	0.994948838	2.57E-11
TUCP_001419	XLOC_055356	ENSRNOT00000085447	0.995353749	1.69E-11
TUCP_001419	XLOC_055356	ENSRNOT00000041281	0.979612444	2.68E-08
TUCP_003180	XLOC_136527	ENSRNOT00000090914	0.952621178	1.74E-06
TUCP_003180	XLOC_136527	ENSRNOT00000067616	0.975856825	6.20E-08
TUCP_003180	XLOC_136527	ENSRNOT00000022559	0.954505118	1.42E-06
TUCP_003628	XLOC_159330	ENSRNOT00000026710	0.951725741	1.90E-06
TUCP_003628	XLOC_159330	ENSRNOT00000004919	0.956079871	1.20E-06
TUCP_003628	XLOC_159330	ENSRNOT00000026558	0.974738532	7.77E-08
TUCP_003628	XLOC_159330	ENSRNOT00000014824	0.950818268	2.09E-06
TUCP_003628	XLOC_159330	ENSRNOT00000066764	0.966934427	2.94E-07
TUCP_003628	XLOC_159330	ENSRNOT00000086541	0.958703655	8.82E-07
TUCP_003628	XLOC_159330	ENSRNOT00000083702	0.951893609	1.87E-06
TUCP_003628	XLOC_159330	ENSRNOT00000028067	0.955899554	1.22E-06
TUCP_003628	XLOC_159330	ENSRNOT00000000640	0.95260463	1.74E-06
TUCP_003628	XLOC_159330	ENSRNOT00000004363	0.955608237	1.26E-06
TUCP_003628	XLOC_159330	ENSRNOT00000022633	0.953765608	1.54E-06
TUCP_003628	XLOC_159330	ENSRNOT00000020475	0.969149354	2.09E-07
TUCP_003628	XLOC_159330	ENSRNOT00000032249	0.975798601	6.28E-08
TUCP_003628	XLOC_159330	ENSRNOT00000029443	0.955297372	1.30E-06
TUCP_003628	XLOC_159330	ENSRNOT00000090303	0.97055127	1.66E-07
TUCP_003628	XLOC_159330	ENSRNOT00000003823	0.96941359	2.00E-07

TUCP_003628	XLOC_159330	ENSRNOT00000019680	0.96051824	7.07E-07
TUCP_003628	XLOC_159330	ENSRNOT00000019501	0.965404961	3.68E-07
TUCP_003628	XLOC_159330	ENSRNOT00000007634	-0.955024774	1.34E-06
TUCP_003628	XLOC_159330	ENSRNOT00000008365	0.967711161	2.62E-07
TUCP_003628	XLOC_159330	ENSRNOT00000076421	0.961710342	6.08E-07
TUCP_003628	XLOC_159330	ENSRNOT00000047011	0.983230066	1.02E-08
TUCP_003628	XLOC_159330	ENSRNOT00000086459	0.9551674	1.32E-06
TUCP_003628	XLOC_159330	ENSRNOT00000044788	0.951959342	1.86E-06
TUCP_003628	XLOC_159330	ENSRNOT00000018511	0.95124418	2.00E-06
TUCP_003628	XLOC_159330	ENSRNOT00000035123	0.962169087	5.73E-07
TUCP_003628	XLOC_159330	ENSRNOT00000033984	0.95021725	2.21E-06
TUCP_003628	XLOC_159330	ENSRNOT00000086390	0.961354186	6.36E-07
TUCP_003628	XLOC_159330	ENSRNOT00000067912	0.977862664	4.03E-08
TUCP_003628	XLOC_159330	ENSRNOT00000059662	0.959705251	7.82E-07
TUCP_003628	XLOC_159330	ENSRNOT00000009247	0.956137427	1.19E-06
TUCP_003628	XLOC_159330	ENSRNOT00000025197	0.963810677	4.60E-07
TUCP_003628	XLOC_159330	ENSRNOT00000039377	0.955725502	1.24E-06
TUCP_003628	XLOC_159330	ENSRNOT00000055179	0.958740349	8.79E-07
TUCP_003628	XLOC_159330	ENSRNOT00000078746	0.958988507	8.53E-07
TUCP_003628	XLOC_159330	ENSRNOT00000079310	0.968883777	2.18E-07
TUCP_003628	XLOC_159330	ENSRNOT00000088712	0.967347492	2.77E-07
TUCP_000934	XLOC_034573	ENSRNOT00000046775	0.951770096	1.89E-06
TUCP_000934	XLOC_034573	ENSRNOT00000020329	0.961571412	6.19E-07
TUCP_001449	XLOC_056232	ENSRNOT00000079019	0.956833363	1.10E-06
TUCP_001449	XLOC_056232	ENSRNOT00000023623	0.952954469	1.68E-06
TUCP_001449	XLOC_056232	ENSRNOT00000066598	0.950771994	2.10E-06
TUCP_001449	XLOC_056232	ENSRNOT00000072682	0.960405399	7.17E-07
TUCP_001449	XLOC_056232	ENSRNOT00000044419	0.963402343	4.86E-07
TUCP_001449	XLOC_056232	ENSRNOT00000092757	0.950088106	2.24E-06
TUCP_001449	XLOC_056232	ENSRNOT00000003932	0.969496814	1.98E-07
TUCP_003186	XLOC_137407	ENSRNOT00000023416	0.952359729	1.78E-06
TUCP_003186	XLOC_137407	ENSRNOT00000074583	0.953081865	1.65E-06
TUCP_003186	XLOC_137407	ENSRNOT00000018755	0.964137026	4.40E-07
TUCP_003186	XLOC_137407	ENSRNOT00000078299	0.995151694	2.09E-11
TUCP_003186	XLOC_137407	ENSRNOT00000040736	0.968352362	2.37E-07
TUCP_003186	XLOC_137407	ENSRNOT00000068354	0.966599949	3.09E-07
TUCP_003186	XLOC_137407	ENSRNOT00000029184	0.968889929	2.18E-07
TUCP_003186	XLOC_137407	ENSRNOT00000025257	0.976482237	5.45E-08
TUCP_003186	XLOC_137407	ENSRNOT00000003458	0.960678553	6.93E-07
TUCP_003186	XLOC_137407	ENSRNOT00000004347	0.953092218	1.65E-06
TUCP_003186	XLOC_137407	ENSRNOT00000003778	0.953438248	1.59E-06
TUCP_003186	XLOC_137407	ENSRNOT00000002916	-0.950431461	2.17E-06
TUCP_003186	XLOC_137407	ENSRNOT00000051028	-0.96487126	3.97E-07
TUCP_003186	XLOC_137407	ENSRNOT00000031693	0.955932358	1.22E-06
TUCP_003186	XLOC_137407	ENSRNOT00000082393	0.959040786	8.47E-07
TUCP_003186	XLOC_137407	ENSRNOT00000022167	0.977562636	4.31E-08
TUCP_003186	XLOC_137407	ENSRNOT00000052130	0.950083629	2.24E-06
TUCP_003597	XLOC_158359	ENSRNOT00000006992	0.961146403	6.53E-07
TUCP_003597	XLOC_158359	ENSRNOT00000067984	0.964613017	4.12E-07
TUCP_003597	XLOC_158359	ENSRNOT00000076343	0.962026105	5.83E-07
TUCP_003597	XLOC_158359	ENSRNOT00000004836	0.962735631	5.32E-07

TUCP_003597	XLOC_158359	ENSRNOT00000059458	0.960156298	7.40E-07
TUCP_003597	XLOC_158359	ENSRNOT00000040069	0.9582239	9.34E-07
TUCP_003597	XLOC_158359	ENSRNOT00000016504	0.954909807	1.36E-06
TUCP_003597	XLOC_158359	ENSRNOT00000016739	0.960003945	7.54E-07
TUCP_003597	XLOC_158359	ENSRNOT00000052288	0.970820687	1.59E-07
TUCP_003597	XLOC_158359	ENSRNOT00000021116	0.954736112	1.39E-06
TUCP_003597	XLOC_158359	ENSRNOT00000092418	0.973591895	9.68E-08
TUCP_003597	XLOC_158359	ENSRNOT00000076285	0.957426354	1.03E-06
TUCP_003597	XLOC_158359	ENSRNOT00000008786	-0.962452939	5.52E-07
TUCP_003597	XLOC_158359	ENSRNOT00000001449	0.950846029	2.08E-06
TUCP_003597	XLOC_158359	ENSRNOT00000044896	0.963398254	4.86E-07
TUCP_003597	XLOC_158359	ENSRNOT00000022800	0.950589093	2.13E-06
TUCP_003597	XLOC_158359	ENSRNOT00000076516	0.967080207	2.88E-07
TUCP_003597	XLOC_158359	ENSRNOT00000015073	0.961341167	6.37E-07
TUCP_003597	XLOC_158359	ENSRNOT00000076793	0.97108305	1.52E-07
TUCP_003597	XLOC_158359	ENSRNOT00000085332	-0.973602991	9.66E-08
TUCP_003597	XLOC_158359	ENSRNOT00000013046	-0.951041757	2.04E-06
TUCP_003597	XLOC_158359	ENSRNOT00000071573	0.958589045	8.95E-07
TUCP_003597	XLOC_158359	ENSRNOT00000027964	0.971336731	1.45E-07
TUCP_003597	XLOC_158359	ENSRNOT00000006828	0.955813603	1.23E-06
TUCP_003597	XLOC_158359	ENSRNOT00000087306	0.960875658	6.76E-07
TUCP_003597	XLOC_158359	ENSRNOT00000003182	0.960060121	7.48E-07
TUCP_003597	XLOC_158359	ENSRNOT00000090376	0.969001812	2.14E-07
TUCP_003597	XLOC_158359	ENSRNOT00000072081	0.953392251	1.60E-06
TUCP_003597	XLOC_158359	ENSRNOT00000046058	0.955027235	1.34E-06
TUCP_003597	XLOC_158359	ENSRNOT00000088191	0.971462091	1.42E-07
TUCP_003597	XLOC_158359	ENSRNOT00000057313	0.960714595	6.90E-07
TUCP_003597	XLOC_158359	ENSRNOT00000009221	0.952764027	1.71E-06
TUCP_003597	XLOC_158359	ENSRNOT00000042799	0.957379644	1.03E-06
TUCP_003621	XLOC_159011	ENSRNOT00000092026	0.96620614	3.28E-07
TUCP_003621	XLOC_159011	ENSRNOT00000093075	1	0
TUCP_003621	XLOC_159011	ENSRNOT00000058260	1	0
TUCP_003621	XLOC_159011	ENSRNOT00000088887	1	0
TUCP_003621	XLOC_159011	ENSRNOT00000084986	1	0
TUCP_003621	XLOC_159011	ENSRNOT00000074346	0.9702256	1.75E-07
TUCP_003621	XLOC_159011	ENSRNOT00000073209	0.983711602	8.79E-09
TUCP_003621	XLOC_159011	ENSRNOT00000080155	0.973069673	1.07E-07
TUCP_003621	XLOC_159011	ENSRNOT00000072573	0.996213272	6.09E-12
TUCP_003621	XLOC_159011	ENSRNOT00000017828	0.996001583	7.99E-12
TUCP_003621	XLOC_159011	ENSRNOT00000080444	0.969625907	1.93E-07
TUCP_003621	XLOC_159011	ENSRNOT00000071312	1	0
TUCP_003621	XLOC_159011	ENSRNOT00000088245	1	0
TUCP_003621	XLOC_159011	ENSRNOT00000036565	0.969065335	2.12E-07
TUCP_003621	XLOC_159011	ENSRNOT00000058833	0.998673261	3.23E-14
TUCP_003621	XLOC_159011	ENSRNOT00000078052	1	0
TUCP_003621	XLOC_159011	ENSRNOT00000078494	0.999927093	1.62E-20
TUCP_003621	XLOC_159011	ENSRNOT00000085653	1	0
TUCP_003621	XLOC_159011	ENSRNOT00000057950	0.969329371	2.03E-07
TUCP_003621	XLOC_159011	ENSRNOT00000081992	0.999320526	1.14E-15
TUCP_003621	XLOC_159011	ENSRNOT00000038553	0.955765808	1.24E-06
TUCP_003621	XLOC_159011	ENSRNOT00000084120	0.958115447	9.46E-07

TUCP_003621	XLOC_159011	ENSRNOT00000055406	1	0
TUCP_003621	XLOC_159011	ENSRNOT00000070817	0.998968079	9.20E-15
TUCP_003621	XLOC_159011	ENSRNOT00000026753	1	0
TUCP_003621	XLOC_159011	ENSRNOT00000080533	0.996682529	3.15E-12
TUCP_003621	XLOC_159011	ENSRNOT00000072774	0.979665261	2.65E-08
TUCP_003621	XLOC_159011	ENSRNOT00000086031	1	0
TUCP_003621	XLOC_159011	ENSRNOT00000014312	1	0
TUCP_003621	XLOC_159011	ENSRNOT00000042882	0.963218427	4.98E-07
TUCP_003621	XLOC_159011	ENSRNOT00000088012	0.956765806	1.11E-06
TUCP_003621	XLOC_159011	ENSRNOT00000051231	0.999242378	1.96E-15
TUCP_003621	XLOC_159011	ENSRNOT00000089407	0.992986194	1.32E-10
TUCP_003621	XLOC_159011	ENSRNOT00000055034	0.998331441	1.02E-13
TUCP_003621	XLOC_159011	ENSRNOT00000046837	0.97252559	1.18E-07
TUCP_003620	XLOC_159010	ENSRNOT00000084616	-0.984671292	6.50E-09
TUCP_003620	XLOC_159010	ENSRNOT00000034642	0.983228972	1.02E-08
TUCP_003620	XLOC_159010	ENSRNOT00000074087	0.97652988	5.39E-08
TUCP_003620	XLOC_159010	ENSRNOT00000045905	0.953659213	1.56E-06
TUCP_003620	XLOC_159010	ENSRNOT00000067500	0.970204559	1.76E-07
TUCP_003622	XLOC_159100	ENSRNOT00000065811	0.965781613	3.49E-07
TUCP_003622	XLOC_159100	ENSRNOT00000038780	0.963746082	4.64E-07
TUCP_003624	XLOC_159120	ENSRNOT00000032439	0.953069363	1.66E-06
TUCP_003624	XLOC_159120	ENSRNOT00000020854	0.969546939	1.96E-07
TUCP_001756	XLOC_072762	ENSRNOT00000074071	0.956221789	1.18E-06
TUCP_003740	XLOC_164007	ENSRNOT00000089602	0.977738125	4.15E-08
TUCP_002618	XLOC_110175	ENSRNOT00000060943	0.96009737	7.45E-07
TUCP_002618	XLOC_110175	ENSRNOT00000023302	0.95499016	1.35E-06
TUCP_003747	XLOC_164519	ENSRNOT00000024186	0.983200502	1.02E-08
TUCP_003747	XLOC_164519	ENSRNOT00000074753	0.998182349	1.56E-13
TUCP_003747	XLOC_164519	ENSRNOT00000004876	0.959071443	8.44E-07
TUCP_003747	XLOC_164519	ENSRNOT00000041904	1	0
TUCP_003747	XLOC_164519	ENSRNOT00000083487	0.989515314	9.80E-10
TUCP_003747	XLOC_164519	ENSRNOT00000077990	1	0
TUCP_003747	XLOC_164519	ENSRNOT00000038826	0.99977672	4.37E-18
TUCP_003747	XLOC_164519	ENSRNOT00000046842	0.973644314	9.58E-08
TUCP_003747	XLOC_164519	ENSRNOT00000037169	0.980329406	2.24E-08
TUCP_003747	XLOC_164519	ENSRNOT00000021243	0.987425406	2.42E-09
TUCP_003747	XLOC_164519	ENSRNOT00000060250	0.999933853	9.97E-21
TUCP_003747	XLOC_164519	ENSRNOT00000091934	1	1.33E-79
TUCP_003747	XLOC_164519	ENSRNOT00000046585	0.982941597	1.11E-08
TUCP_003747	XLOC_164519	ENSRNOT00000080794	0.990839253	5.00E-10
TUCP_003747	XLOC_164519	ENSRNOT00000014456	0.991697153	3.06E-10
TUCP_003747	XLOC_164519	ENSRNOT00000031205	0.991394766	3.66E-10
TUCP_003747	XLOC_164519	ENSRNOT00000044145	1	0
TUCP_003747	XLOC_164519	ENSRNOT00000088381	0.989013276	1.24E-09
TUCP_003747	XLOC_164519	ENSRNOT00000011743	0.98678427	3.11E-09
TUCP_003747	XLOC_164519	ENSRNOT00000089929	1	0
TUCP_003747	XLOC_164519	ENSRNOT00000088624	1	0
TUCP_003747	XLOC_164519	ENSRNOT00000085659	0.989908621	8.10E-10
TUCP_003747	XLOC_164519	ENSRNOT00000015866	0.994066664	5.73E-11
TUCP_003747	XLOC_164519	ENSRNOT00000078633	1	0
TUCP_003747	XLOC_164519	ENSRNOT00000034907	0.995318542	1.76E-11

TUCP_003747	XLOC_164519	ENSRNOT00000093230	0.958295175	9.26E-07
TUCP_003747	XLOC_164519	ENSRNOT00000051835	1	0
TUCP_003747	XLOC_164519	ENSRNOT00000079087	1	0
TUCP_003747	XLOC_164519	ENSRNOT00000037206	1	1.33E-79
TUCP_003747	XLOC_164519	ENSRNOT00000083261	0.970991658	1.54E-07
TUCP_003747	XLOC_164519	ENSRNOT00000080590	0.999889216	1.31E-19
TUCP_003747	XLOC_164519	ENSRNOT00000027552	0.999801024	2.46E-18
TUCP_003747	XLOC_164519	ENSRNOT00000077014	0.995074376	2.26E-11
TUCP_003747	XLOC_164519	ENSRNOT00000080453	0.99994332	4.61E-21
TUCP_003747	XLOC_164519	ENSRNOT00000089057	0.968243551	2.41E-07
TUCP_003747	XLOC_164519	ENSRNOT00000066839	0.991868612	2.76E-10
TUCP_003747	XLOC_164519	ENSRNOT00000061284	0.955566942	1.27E-06
TUCP_003747	XLOC_164519	ENSRNOT00000013423	0.959677621	7.84E-07
TUCP_003747	XLOC_164519	ENSRNOT00000071884	0.974769955	7.72E-08
TUCP_003747	XLOC_164519	ENSRNOT00000004128	0.961829362	5.99E-07
TUCP_003747	XLOC_164519	ENSRNOT00000026316	0.964408046	4.24E-07
TUCP_003747	XLOC_164519	ENSRNOT00000057729	0.999384184	6.97E-16
TUCP_003747	XLOC_164519	ENSRNOT00000078663	0.975192577	7.10E-08
TUCP_003747	XLOC_164519	ENSRNOT00000064250	0.983131977	1.05E-08
TUCP_003747	XLOC_164519	ENSRNOT00000021608	0.965228881	3.78E-07
TUCP_003747	XLOC_164519	ENSRNOT00000029634	0.995816134	1.00E-11
TUCP_003747	XLOC_164519	ENSRNOT00000093579	0.958564264	8.97E-07
TUCP_003747	XLOC_164519	ENSRNOT00000086349	0.957242511	1.05E-06
TUCP_003747	XLOC_164519	ENSRNOT00000065460	1	0
TUCP_003747	XLOC_164519	ENSRNOT00000003817	0.996778712	2.72E-12
TUCP_003747	XLOC_164519	ENSRNOT00000016713	1	0
TUCP_003747	XLOC_164519	ENSRNOT00000079260	0.996448245	4.42E-12
TUCP_003747	XLOC_164519	ENSRNOT00000039294	1	1.33E-79
TUCP_003747	XLOC_164519	ENSRNOT00000092913	0.952980228	1.67E-06
TUCP_003744	XLOC_164137	ENSRNOT00000041645	0.953409318	1.60E-06
TUCP_003745	XLOC_164442	ENSRNOT00000090755	0.982604671	1.22E-08
TUCP_003745	XLOC_164442	ENSRNOT00000051285	0.950563838	2.14E-06
TUCP_003745	XLOC_164442	ENSRNOT00000011604	0.95235793	1.78E-06
TUCP_003745	XLOC_164442	ENSRNOT00000036565	0.981843117	1.51E-08
TUCP_003745	XLOC_164442	ENSRNOT00000072573	0.953780949	1.54E-06
TUCP_002612	XLOC_109992	ENSRNOT00000023302	0.96394516	4.52E-07
TUCP_002612	XLOC_109992	ENSRNOT00000086917	0.950231027	2.21E-06
TUCP_002612	XLOC_109992	ENSRNOT00000002403	0.952652235	1.73E-06
TUCP_002612	XLOC_109992	ENSRNOT00000047093	0.965611041	3.58E-07
TUCP_002612	XLOC_109992	ENSRNOT00000025075	0.952109352	1.83E-06
TUCP_002612	XLOC_109992	ENSRNOT00000050180	0.975487835	6.69E-08
TUCP_002612	XLOC_109992	ENSRNOT00000041494	0.95816762	9.40E-07
TUCP_002612	XLOC_109992	ENSRNOT00000018179	0.965882022	3.44E-07
TUCP_002612	XLOC_109992	ENSRNOT00000081512	0.95582706	1.23E-06
TUCP_002612	XLOC_109992	ENSRNOT00000086905	0.956630671	1.12E-06
TUCP_002612	XLOC_109992	ENSRNOT00000045223	0.953121814	1.65E-06
TUCP_002612	XLOC_109992	ENSRNOT00000066024	0.958162848	9.41E-07
TUCP_002612	XLOC_109992	ENSRNOT00000025808	0.955745077	1.24E-06
TUCP_002612	XLOC_109992	ENSRNOT00000031904	0.950924853	2.06E-06
TUCP_002612	XLOC_109992	ENSRNOT00000016975	0.958751049	8.77E-07
TUCP_002610	XLOC_109989	ENSRNOT00000036050	0.951706657	1.91E-06

TUCP_003730	XLOC_163033	ENSRNOT00000020425	0.957861352	9.75E-07
TUCP_003730	XLOC_163033	ENSRNOT00000092968	0.955709538	1.25E-06
TUCP_003730	XLOC_163033	ENSRNOT00000078419	0.950894094	2.07E-06
TUCP_002615	XLOC_109997	ENSRNOT00000036766	0.954939161	1.36E-06
TUCP_004193	XLOC_184374	ENSRNOT00000066125	0.956166987	1.18E-06
TUCP_003596	XLOC_158306	ENSRNOT00000024186	0.965082092	3.86E-07
TUCP_003596	XLOC_158306	ENSRNOT00000074753	0.969275309	2.05E-07
TUCP_003596	XLOC_158306	ENSRNOT00000004876	0.966843802	2.98E-07
TUCP_003596	XLOC_158306	ENSRNOT00000041904	0.973586165	9.69E-08
TUCP_003596	XLOC_158306	ENSRNOT00000061194	0.955488317	1.28E-06
TUCP_003596	XLOC_158306	ENSRNOT00000083487	0.977060289	4.81E-08
TUCP_003596	XLOC_158306	ENSRNOT00000077990	0.973586165	9.69E-08
TUCP_003596	XLOC_158306	ENSRNOT00000038826	0.974340471	8.39E-08
TUCP_003596	XLOC_158306	ENSRNOT00000046842	0.96622216	3.27E-07
TUCP_003596	XLOC_158306	ENSRNOT00000060250	0.972340817	1.22E-07
TUCP_003596	XLOC_158306	ENSRNOT00000091934	0.973586165	9.69E-08
TUCP_003596	XLOC_158306	ENSRNOT00000061139	0.953088572	1.65E-06
TUCP_003596	XLOC_158306	ENSRNOT00000046585	0.975406567	6.80E-08
TUCP_003596	XLOC_158306	ENSRNOT00000089929	0.973586165	9.69E-08
TUCP_003596	XLOC_158306	ENSRNOT00000014456	0.956643465	1.12E-06
TUCP_003596	XLOC_158306	ENSRNOT00000031205	0.974472587	8.18E-08
TUCP_003596	XLOC_158306	ENSRNOT00000044145	0.973586165	9.69E-08
TUCP_003596	XLOC_158306	ENSRNOT00000088381	0.977011171	4.87E-08
TUCP_003596	XLOC_158306	ENSRNOT00000072939	0.955826929	1.23E-06
TUCP_003596	XLOC_158306	ENSRNOT00000011743	0.973718043	9.45E-08
TUCP_003596	XLOC_158306	ENSRNOT00000034907	0.981047569	1.87E-08
TUCP_003596	XLOC_158306	ENSRNOT00000088624	0.973586165	9.69E-08
TUCP_003596	XLOC_158306	ENSRNOT00000085659	0.972879897	1.10E-07
TUCP_003596	XLOC_158306	ENSRNOT00000015866	0.971933483	1.31E-07
TUCP_003596	XLOC_158306	ENSRNOT00000064250	0.956464379	1.14E-06
TUCP_003596	XLOC_158306	ENSRNOT00000093230	0.954684689	1.39E-06
TUCP_003596	XLOC_158306	ENSRNOT00000051835	0.973586165	9.69E-08
TUCP_003596	XLOC_158306	ENSRNOT00000079087	0.973586165	9.69E-08
TUCP_003596	XLOC_158306	ENSRNOT00000037206	0.973586165	9.69E-08
TUCP_003596	XLOC_158306	ENSRNOT00000083261	0.96339198	4.87E-07
TUCP_003596	XLOC_158306	ENSRNOT00000080794	0.95947493	8.04E-07
TUCP_003596	XLOC_158306	ENSRNOT00000080590	0.97296448	1.09E-07
TUCP_003596	XLOC_158306	ENSRNOT00000027552	0.972428268	1.20E-07
TUCP_003596	XLOC_158306	ENSRNOT00000077014	0.96518749	3.80E-07
TUCP_003596	XLOC_158306	ENSRNOT00000080453	0.973575988	9.71E-08
TUCP_003596	XLOC_158306	ENSRNOT00000089057	0.95925116	8.26E-07
TUCP_003596	XLOC_158306	ENSRNOT00000057729	0.974409793	8.28E-08
TUCP_003596	XLOC_158306	ENSRNOT00000078633	0.973586165	9.69E-08
TUCP_003596	XLOC_158306	ENSRNOT00000071884	0.967815948	2.58E-07
TUCP_003596	XLOC_158306	ENSRNOT00000066839	0.957246138	1.05E-06
TUCP_003596	XLOC_158306	ENSRNOT00000029634	0.964321141	4.29E-07
TUCP_003596	XLOC_158306	ENSRNOT00000093579	0.96560623	3.58E-07
TUCP_003596	XLOC_158306	ENSRNOT00000086349	0.967505902	2.70E-07
TUCP_003596	XLOC_158306	ENSRNOT00000065460	0.973586165	9.69E-08
TUCP_003596	XLOC_158306	ENSRNOT00000003817	0.96378451	4.62E-07
TUCP_003596	XLOC_158306	ENSRNOT00000016713	0.973586165	9.69E-08

TUCP_003596	XLOC_158306	ENSRNOT00000079260	0.978201592	3.74E-08
TUCP_003596	XLOC_158306	ENSRNOT00000039294	0.973586165	9.69E-08
TUCP_001006	XLOC_037837	ENSRNOT00000025052	0.984949445	5.93E-09
TUCP_001006	XLOC_037837	ENSRNOT00000067655	0.999608344	7.25E-17
TUCP_001006	XLOC_037837	ENSRNOT00000074346	0.968508239	2.31E-07
TUCP_001006	XLOC_037837	ENSRNOT00000073209	0.979439701	2.80E-08
TUCP_001006	XLOC_037837	ENSRNOT00000092261	0.991318278	3.83E-10
TUCP_001006	XLOC_037837	ENSRNOT00000093612	0.997937181	2.93E-13
TUCP_001493	XLOC_057848	ENSRNOT00000001077	0.952192137	1.81E-06
TUCP_001493	XLOC_057848	ENSRNOT00000001158	0.958323749	9.23E-07
TUCP_001493	XLOC_057848	ENSRNOT00000085362	0.962376245	5.57E-07
TUCP_001493	XLOC_057848	ENSRNOT00000084177	-0.950277368	2.20E-06
TUCP_001493	XLOC_057848	ENSRNOT00000026391	0.950600318	2.13E-06
TUCP_001493	XLOC_057848	ENSRNOT00000012340	0.970114072	1.79E-07
TUCP_001493	XLOC_057848	ENSRNOT00000064039	0.962269427	5.65E-07
TUCP_001493	XLOC_057848	ENSRNOT00000006217	0.962037435	5.83E-07
TUCP_001493	XLOC_057848	ENSRNOT00000003087	0.971301048	1.46E-07
TUCP_001493	XLOC_057848	ENSRNOT00000026518	0.958462039	9.08E-07
TUCP_001493	XLOC_057848	ENSRNOT00000078625	0.957323587	1.04E-06
TUCP_001493	XLOC_057848	ENSRNOT00000001697	0.971158979	1.50E-07
TUCP_001493	XLOC_057848	ENSRNOT00000024066	0.954727235	1.39E-06
TUCP_001493	XLOC_057848	ENSRNOT00000077706	0.951051864	2.04E-06
TUCP_001493	XLOC_057848	ENSRNOT00000076973	0.960717426	6.90E-07
TUCP_001493	XLOC_057848	ENSRNOT00000036051	0.955084641	1.33E-06
TUCP_001493	XLOC_057848	ENSRNOT00000085517	0.964822687	4.00E-07
TUCP_001493	XLOC_057848	ENSRNOT00000036357	0.964189352	4.37E-07
TUCP_001493	XLOC_057848	ENSRNOT00000028685	0.963720782	4.66E-07
TUCP_001493	XLOC_057848	ENSRNOT00000083344	0.955145014	1.33E-06
TUCP_001493	XLOC_057848	ENSRNOT00000027476	0.970429948	1.69E-07
TUCP_001493	XLOC_057848	ENSRNOT00000036137	0.951769687	1.90E-06
TUCP_001493	XLOC_057848	ENSRNOT00000080090	0.952796157	1.70E-06
TUCP_001493	XLOC_057848	ENSRNOT00000012060	0.953904181	1.52E-06
TUCP_001493	XLOC_057848	ENSRNOT00000011956	0.954114278	1.48E-06
TUCP_001493	XLOC_057848	ENSRNOT00000004551	0.951543258	1.94E-06
TUCP_001493	XLOC_057848	ENSRNOT00000012755	0.971593203	1.39E-07
TUCP_001493	XLOC_057848	ENSRNOT00000089068	0.950616759	2.13E-06
TUCP_001493	XLOC_057848	ENSRNOT00000079128	0.952296321	1.80E-06
TUCP_001493	XLOC_057848	ENSRNOT00000081678	0.950897234	2.07E-06
TUCP_001493	XLOC_057848	ENSRNOT00000023178	0.953442728	1.59E-06
TUCP_001493	XLOC_057848	ENSRNOT00000017504	0.960296413	7.27E-07
TUCP_001493	XLOC_057848	ENSRNOT00000025037	0.964825808	4.00E-07
TUCP_001493	XLOC_057848	ENSRNOT00000028677	0.959801209	7.73E-07
TUCP_001493	XLOC_057848	ENSRNOT00000010369	0.950590259	2.13E-06
TUCP_001991	XLOC_080885	ENSRNOT00000028992	0.953669849	1.56E-06
TUCP_001990	XLOC_080848	ENSRNOT00000006995	0.950879573	2.07E-06
TUCP_001990	XLOC_080848	ENSRNOT00000021850	0.954987474	1.35E-06
TUCP_001990	XLOC_080848	ENSRNOT00000040859	0.956428101	1.15E-06
TUCP_001990	XLOC_080848	ENSRNOT00000020339	0.960861766	6.77E-07
TUCP_001990	XLOC_080848	ENSRNOT00000024266	0.957585059	1.01E-06
TUCP_001990	XLOC_080848	ENSRNOT00000020155	0.959390419	8.12E-07
TUCP_001990	XLOC_080848	ENSRNOT00000026941	0.950577457	2.14E-06

TUCP_001990	XLOC_080848	ENSRNOT00000055683	0.957926327	9.67E-07
TUCP_001990	XLOC_080848	ENSRNOT00000024224	0.979761583	2.58E-08
TUCP_001990	XLOC_080848	ENSRNOT00000038807	0.961771592	6.03E-07
TUCP_001990	XLOC_080848	ENSRNOT00000023744	0.964435186	4.22E-07
TUCP_001990	XLOC_080848	ENSRNOT00000065333	0.950898501	2.07E-06
TUCP_001990	XLOC_080848	ENSRNOT00000051294	0.953841845	1.53E-06
TUCP_001990	XLOC_080848	ENSRNOT00000026273	0.962916409	5.19E-07
TUCP_001990	XLOC_080848	ENSRNOT00000040629	0.960441332	7.14E-07
TUCP_001990	XLOC_080848	ENSRNOT00000005292	0.972068861	1.28E-07
TUCP_001990	XLOC_080848	ENSRNOT00000001826	0.952285972	1.80E-06
TUCP_001990	XLOC_080848	ENSRNOT00000041096	0.956449394	1.15E-06
TUCP_001990	XLOC_080848	ENSRNOT00000085765	0.950956042	2.06E-06
TUCP_001990	XLOC_080848	ENSRNOT00000031026	0.966707944	3.05E-07
TUCP_001990	XLOC_080848	ENSRNOT00000034261	0.953292659	1.62E-06
TUCP_001990	XLOC_080848	ENSRNOT00000001855	0.957999977	9.59E-07
TUCP_001990	XLOC_080848	ENSRNOT00000001691	0.960523048	7.07E-07
TUCP_001990	XLOC_080848	ENSRNOT00000077526	0.956722807	1.11E-06
TUCP_001990	XLOC_080848	ENSRNOT00000039523	0.976196154	5.78E-08
TUCP_001990	XLOC_080848	ENSRNOT00000035992	0.970879307	1.57E-07
TUCP_001990	XLOC_080848	ENSRNOT00000025323	0.958158908	9.41E-07
TUCP_001990	XLOC_080848	ENSRNOT00000021769	0.959537906	7.98E-07
TUCP_001990	XLOC_080848	ENSRNOT00000028561	0.953960262	1.51E-06
TUCP_001990	XLOC_080848	ENSRNOT00000023140	0.968102556	2.46E-07
TUCP_001990	XLOC_080848	ENSRNOT00000005387	0.962253558	5.66E-07
TUCP_001990	XLOC_080848	ENSRNOT00000007255	0.957756145	9.87E-07
TUCP_001990	XLOC_080848	ENSRNOT00000010853	0.967192947	2.83E-07
TUCP_001990	XLOC_080848	ENSRNOT00000037699	0.971336219	1.45E-07
TUCP_001990	XLOC_080848	ENSRNOT00000020794	0.97478629	7.69E-08
TUCP_001990	XLOC_080848	ENSRNOT00000010541	0.955152261	1.32E-06
TUCP_001990	XLOC_080848	ENSRNOT00000065230	0.953384863	1.60E-06
TUCP_001990	XLOC_080848	ENSRNOT00000092741	0.956855278	1.09E-06
TUCP_001990	XLOC_080848	ENSRNOT00000075656	-0.961429292	6.30E-07
TUCP_001990	XLOC_080848	ENSRNOT00000022788	0.969624163	1.94E-07
TUCP_001990	XLOC_080848	ENSRNOT00000016947	0.958300751	9.26E-07
TUCP_001990	XLOC_080848	ENSRNOT00000055582	0.962942961	5.17E-07
TUCP_001990	XLOC_080848	ENSRNOT00000009712	0.953509615	1.58E-06
TUCP_001990	XLOC_080848	ENSRNOT00000016191	0.95520724	1.32E-06
TUCP_001990	XLOC_080848	ENSRNOT00000065501	0.953560917	1.57E-06
TUCP_001990	XLOC_080848	ENSRNOT00000025362	-0.950169932	2.22E-06
TUCP_001990	XLOC_080848	ENSRNOT00000060696	0.954824352	1.37E-06
TUCP_001990	XLOC_080848	ENSRNOT00000021158	0.975082402	7.26E-08
TUCP_001990	XLOC_080848	ENSRNOT00000021729	0.956112514	1.19E-06
TUCP_001990	XLOC_080848	ENSRNOT00000084317	0.96151975	6.23E-07
TUCP_001990	XLOC_080848	ENSRNOT00000026789	0.950784282	2.09E-06
TUCP_001990	XLOC_080848	ENSRNOT00000001409	0.951419975	1.96E-06
TUCP_001990	XLOC_080848	ENSRNOT00000066739	0.965452377	3.66E-07
TUCP_001990	XLOC_080848	ENSRNOT00000026280	0.953469851	1.59E-06
TUCP_001990	XLOC_080848	ENSRNOT00000031487	0.954813235	1.38E-06
TUCP_001990	XLOC_080848	ENSRNOT00000060595	-0.961393485	6.33E-07
TUCP_001990	XLOC_080848	ENSRNOT00000028057	0.957800359	9.82E-07
TUCP_001990	XLOC_080848	ENSRNOT00000039232	0.96225458	5.66E-07

TUCP_001990	XLOC_080848	ENSRNOT00000063772	0.961376345	6.34E-07
TUCP_001990	XLOC_080848	ENSRNOT00000002403	0.961813392	6.00E-07
TUCP_001990	XLOC_080848	ENSRNOT00000079072	0.971403418	1.44E-07
TUCP_001990	XLOC_080848	ENSRNOT00000026057	-0.957585677	1.01E-06
TUCP_001990	XLOC_080848	ENSRNOT00000067904	0.956369795	1.16E-06
TUCP_001990	XLOC_080848	ENSRNOT00000026210	0.965726501	3.52E-07
TUCP_001990	XLOC_080848	ENSRNOT00000017252	0.957293368	1.04E-06
TUCP_001990	XLOC_080848	ENSRNOT00000009122	0.951629429	1.92E-06
TUCP_001990	XLOC_080848	ENSRNOT00000000658	0.961285714	6.42E-07
TUCP_001990	XLOC_080848	ENSRNOT00000021925	0.975314865	6.93E-08
TUCP_001990	XLOC_080848	ENSRNOT00000040637	0.956033219	1.20E-06
TUCP_001990	XLOC_080848	ENSRNOT00000052072	0.962568846	5.43E-07
TUCP_001990	XLOC_080848	ENSRNOT00000028847	0.961046693	6.62E-07
TUCP_001990	XLOC_080848	ENSRNOT00000086577	0.960055538	7.49E-07
TUCP_001990	XLOC_080848	ENSRNOT00000013580	0.950506489	2.15E-06
TUCP_001990	XLOC_080848	ENSRNOT00000021285	0.954093573	1.49E-06
TUCP_001990	XLOC_080848	ENSRNOT00000019028	0.950401461	2.17E-06
TUCP_001990	XLOC_080848	ENSRNOT00000021716	0.964881735	3.97E-07
TUCP_001990	XLOC_080848	ENSRNOT00000033976	0.956306852	1.17E-06
TUCP_001990	XLOC_080848	ENSRNOT00000033975	0.958460305	9.08E-07
TUCP_001990	XLOC_080848	ENSRNOT00000081881	0.959800616	7.73E-07
TUCP_001990	XLOC_080848	ENSRNOT00000019911	-0.950719294	2.11E-06
TUCP_001990	XLOC_080848	ENSRNOT00000028801	0.959412335	8.10E-07
TUCP_001990	XLOC_080848	ENSRNOT00000044608	-0.955598486	1.26E-06
TUCP_001004	XLOC_037807	ENSRNOT00000071840	0.993316765	1.04E-10
TUCP_001004	XLOC_037807	ENSRNOT00000083608	0.983938319	8.19E-09
TUCP_001004	XLOC_037807	ENSRNOT00000086969	0.968516334	2.31E-07
TUCP_001004	XLOC_037807	ENSRNOT00000085476	0.952244626	1.81E-06
TUCP_001004	XLOC_037807	ENSRNOT00000066382	0.97727505	4.59E-08
TUCP_001004	XLOC_037807	ENSRNOT00000045584	0.993316765	1.04E-10
TUCP_001004	XLOC_037807	ENSRNOT00000077995	0.986641169	3.28E-09
TUCP_001004	XLOC_037807	ENSRNOT00000066004	0.99268073	1.63E-10
TUCP_001004	XLOC_037807	ENSRNOT00000064284	0.957340228	1.04E-06
TUCP_001004	XLOC_037807	ENSRNOT00000068197	0.957930083	9.67E-07
TUCP_001004	XLOC_037807	ENSRNOT00000082086	0.960937095	6.71E-07
TUCP_001004	XLOC_037807	ENSRNOT00000089494	0.968368084	2.37E-07
TUCP_001004	XLOC_037807	ENSRNOT00000083747	0.955069192	1.34E-06
TUCP_001004	XLOC_037807	ENSRNOT00000092182	0.993316765	1.04E-10
TUCP_001004	XLOC_037807	ENSRNOT00000084461	0.993316765	1.04E-10
TUCP_001004	XLOC_037807	ENSRNOT00000060448	0.993316765	1.04E-10
TUCP_001004	XLOC_037807	ENSRNOT00000032631	0.993316765	1.04E-10
TUCP_001004	XLOC_037807	ENSRNOT00000079503	0.976338567	5.61E-08
TUCP_001004	XLOC_037807	ENSRNOT00000060113	0.993539035	8.77E-11
TUCP_001004	XLOC_037807	ENSRNOT00000054910	0.977321084	4.55E-08
TUCP_001004	XLOC_037807	ENSRNOT00000057176	0.993286755	1.06E-10
TUCP_001004	XLOC_037807	ENSRNOT00000078176	0.985578607	4.80E-09
TUCP_001004	XLOC_037807	ENSRNOT00000056364	0.99553412	1.39E-11
TUCP_001004	XLOC_037807	ENSRNOT00000093431	0.993316765	1.04E-10
TUCP_001004	XLOC_037807	ENSRNOT00000024758	0.969084387	2.11E-07
TUCP_001004	XLOC_037807	ENSRNOT00000092901	0.993316765	1.04E-10
TUCP_001004	XLOC_037807	ENSRNOT00000089290	0.993227874	1.11E-10

TUCP_001004	XLOC_037807	ENSRNOT00000090211	0.996582517	3.65E-12
TUCP_001004	XLOC_037807	ENSRNOT00000075244	0.966301388	3.23E-07
TUCP_001004	XLOC_037807	ENSRNOT00000091828	0.993018618	1.29E-10
TUCP_001004	XLOC_037807	ENSRNOT00000091944	0.993994793	6.09E-11
TUCP_001004	XLOC_037807	ENSRNOT00000070855	0.993316765	1.04E-10
TUCP_001004	XLOC_037807	ENSRNOT00000046980	0.972385539	1.21E-07
TUCP_001004	XLOC_037807	ENSRNOT00000084109	0.991452844	3.54E-10
TUCP_001004	XLOC_037807	ENSRNOT00000060645	0.957099372	1.06E-06
TUCP_001004	XLOC_037807	ENSRNOT00000041569	0.993316765	1.04E-10
TUCP_001004	XLOC_037807	ENSRNOT00000087091	0.993316765	1.04E-10
TUCP_001004	XLOC_037807	ENSRNOT00000003308	0.989303265	1.08E-09
TUCP_001004	XLOC_037807	ENSRNOT00000026113	0.992823876	1.48E-10
TUCP_001004	XLOC_037807	ENSRNOT00000014939	0.99187368	2.75E-10
TUCP_001004	XLOC_037807	ENSRNOT00000058314	0.981562965	1.63E-08
TUCP_001004	XLOC_037807	ENSRNOT00000047870	0.972950726	1.09E-07
TUCP_001004	XLOC_037807	ENSRNOT00000071603	0.993293839	1.06E-10
TUCP_001004	XLOC_037807	ENSRNOT00000088376	0.976417717	5.52E-08
TUCP_001004	XLOC_037807	ENSRNOT00000060454	0.993316765	1.04E-10
TUCP_001004	XLOC_037807	ENSRNOT00000044232	0.977298726	4.57E-08
TUCP_001004	XLOC_037807	ENSRNOT00000023287	0.974191779	8.64E-08
TUCP_001004	XLOC_037807	ENSRNOT00000071714	0.968169591	2.44E-07
TUCP_001004	XLOC_037807	ENSRNOT00000075836	0.993316765	1.04E-10
TUCP_001004	XLOC_037807	ENSRNOT00000073229	0.993316765	1.04E-10
TUCP_001004	XLOC_037807	ENSRNOT00000077731	0.970925282	1.56E-07
TUCP_001004	XLOC_037807	ENSRNOT00000039652	0.967433024	2.73E-07
TUCP_001004	XLOC_037807	ENSRNOT00000066685	0.965749141	3.50E-07
TUCP_001004	XLOC_037807	ENSRNOT00000082776	0.9698919	1.85E-07
TUCP_001004	XLOC_037807	ENSRNOT00000088719	0.953479279	1.59E-06
TUCP_001004	XLOC_037807	ENSRNOT00000007683	0.993290961	1.06E-10
TUCP_001004	XLOC_037807	ENSRNOT00000092916	0.984145728	7.68E-09
TUCP_001004	XLOC_037807	ENSRNOT00000009295	0.987415996	2.43E-09
TUCP_001994	XLOC_081004	ENSRNOT00000084133	1	0
TUCP_001994	XLOC_081004	ENSRNOT00000093745	0.981891039	1.49E-08
TUCP_001994	XLOC_081004	ENSRNOT00000065542	0.99828699	1.16E-13
TUCP_001994	XLOC_081004	ENSRNOT00000082464	1	0
TUCP_001994	XLOC_081004	ENSRNOT00000087659	1	0
TUCP_001994	XLOC_081004	ENSRNOT00000084582	0.976633579	5.28E-08
TUCP_001994	XLOC_081004	ENSRNOT00000085078	1	0
TUCP_001994	XLOC_081004	ENSRNOT00000014028	1	0
TUCP_001994	XLOC_081004	ENSRNOT00000049363	1	0
TUCP_001994	XLOC_081004	ENSRNOT00000065473	0.999949098	2.69E-21
TUCP_001994	XLOC_081004	ENSRNOT00000000142	0.998996155	8.01E-15
TUCP_001994	XLOC_081004	ENSRNOT00000082288	0.999998398	8.30E-29
TUCP_001994	XLOC_081004	ENSRNOT00000039664	1	0
TUCP_001994	XLOC_081004	ENSRNOT00000065402	1	0
TUCP_001994	XLOC_081004	ENSRNOT00000064551	0.965365872	3.70E-07
TUCP_001994	XLOC_081004	ENSRNOT00000084141	1	0
TUCP_001994	XLOC_081004	ENSRNOT00000082435	1	0
TUCP_001994	XLOC_081004	ENSRNOT00000082928	0.996801059	2.62E-12
TUCP_001994	XLOC_081004	ENSRNOT00000066546	0.956953619	1.08E-06
TUCP_001994	XLOC_081004	ENSRNOT00000046013	0.988138626	1.81E-09

TUCP_001994	XLOC_081004	ENSRNOT00000067931	0.978893628	3.18E-08
TUCP_001994	XLOC_081004	ENSRNOT00000086425	0.992023315	2.51E-10
TUCP_001994	XLOC_081004	ENSRNOT00000093696	0.956927219	1.09E-06
TUCP_001994	XLOC_081004	ENSRNOT00000036228	0.982337157	1.31E-08
TUCP_001994	XLOC_081004	ENSRNOT00000014030	1	0
TUCP_001994	XLOC_081004	ENSRNOT00000081966	1	4.25E-78
TUCP_001994	XLOC_081004	ENSRNOT00000075906	1	1.33E-79
TUCP_001994	XLOC_081004	ENSRNOT00000088396	0.981336994	1.73E-08
TUCP_001994	XLOC_081004	ENSRNOT00000049259	0.965500259	3.63E-07
TUCP_001994	XLOC_081004	ENSRNOT00000092838	0.982579749	1.23E-08
TUCP_001994	XLOC_081004	ENSRNOT00000093442	0.959217317	8.30E-07
TUCP_001996	XLOC_081086	ENSRNOT00000071885	0.958090055	9.49E-07
TUCP_001996	XLOC_081086	ENSRNOT00000021946	0.957122151	1.06E-06
TUCP_001996	XLOC_081086	ENSRNOT00000006519	0.955882415	1.22E-06
TUCP_001996	XLOC_081086	ENSRNOT00000078710	0.977425162	4.45E-08
TUCP_001996	XLOC_081086	ENSRNOT00000090318	0.989663612	9.13E-10
TUCP_001996	XLOC_081086	ENSRNOT00000078140	0.979115365	3.02E-08
TUCP_001996	XLOC_081086	ENSRNOT00000041727	0.990462598	6.12E-10
TUCP_001996	XLOC_081086	ENSRNOT00000021040	-0.963678883	4.68E-07
TUCP_001996	XLOC_081086	ENSRNOT00000076451	0.972542394	1.17E-07
TUCP_001996	XLOC_081086	ENSRNOT00000048293	0.952919014	1.68E-06
TUCP_001999	XLOC_081227	ENSRNOT00000005490	0.954281044	1.46E-06
TUCP_001999	XLOC_081227	ENSRNOT00000050279	0.959691892	7.83E-07
TUCP_001999	XLOC_081227	ENSRNOT00000000468	0.962861086	5.23E-07
TUCP_001999	XLOC_081227	ENSRNOT00000057361	0.956488176	1.14E-06
TUCP_001999	XLOC_081227	ENSRNOT00000025223	0.950108735	2.24E-06
TUCP_001999	XLOC_081227	ENSRNOT00000003286	0.95149148	1.95E-06
TUCP_001998	XLOC_081176	ENSRNOT00000081981	0.959575371	7.94E-07
TUCP_001998	XLOC_081176	ENSRNOT00000005958	0.954911162	1.36E-06
TUCP_001998	XLOC_081176	ENSRNOT00000083629	0.95089927	2.07E-06
TUCP_001998	XLOC_081176	ENSRNOT00000020147	0.955297488	1.30E-06
TUCP_001998	XLOC_081176	ENSRNOT00000080768	0.952230422	1.81E-06
TUCP_001998	XLOC_081176	ENSRNOT00000005798	0.95923913	8.27E-07
TUCP_001998	XLOC_081176	ENSRNOT00000004046	-0.961062616	6.60E-07
TUCP_001002	XLOC_037807	ENSRNOT00000068416	0.957787791	9.83E-07
TUCP_001002	XLOC_037807	ENSRNOT00000042251	0.957989849	9.60E-07
TUCP_001002	XLOC_037807	ENSRNOT00000002984	0.957519136	1.01E-06
TUCP_001002	XLOC_037807	ENSRNOT00000020071	0.96697024	2.93E-07
TUCP_001002	XLOC_037807	ENSRNOT00000083764	0.955359609	1.30E-06
TUCP_001002	XLOC_037807	ENSRNOT00000084847	0.95733094	1.04E-06
TUCP_001002	XLOC_037807	ENSRNOT00000092365	0.954279205	1.46E-06
TUCP_001002	XLOC_037807	ENSRNOT00000074738	0.95079958	2.09E-06
TUCP_001002	XLOC_037807	ENSRNOT00000079275	0.960220462	7.34E-07
TUCP_001002	XLOC_037807	ENSRNOT00000066062	-0.959587006	7.93E-07
TUCP_001002	XLOC_037807	ENSRNOT00000031025	0.950158439	2.23E-06
TUCP_001002	XLOC_037807	ENSRNOT00000045301	0.957830293	9.78E-07
TUCP_001002	XLOC_037807	ENSRNOT00000041186	0.956988894	1.08E-06
TUCP_001002	XLOC_037807	ENSRNOT00000010092	0.975492297	6.68E-08
TUCP_001002	XLOC_037807	ENSRNOT00000051892	0.956045762	1.20E-06
TUCP_001002	XLOC_037807	ENSRNOT00000077134	0.952821149	1.70E-06
TUCP_001002	XLOC_037807	ENSRNOT00000038494	0.960680302	6.93E-07

TUCP_001002	XLOC_037807	ENSRNOT00000081830	0.976404222	5.54E-08
TUCP_001002	XLOC_037807	ENSRNOT00000047527	0.982168899	1.38E-08
TUCP_001002	XLOC_037807	ENSRNOT00000027933	0.951624772	1.92E-06
TUCP_001002	XLOC_037807	ENSRNOT00000039263	0.97051608	1.67E-07
TUCP_001002	XLOC_037807	ENSRNOT00000087603	0.973090443	1.06E-07
TUCP_001002	XLOC_037807	ENSRNOT00000088707	0.958261739	9.30E-07
TUCP_001002	XLOC_037807	ENSRNOT00000054757	0.954331103	1.45E-06
TUCP_001002	XLOC_037807	ENSRNOT00000041741	0.953883665	1.52E-06
TUCP_001002	XLOC_037807	ENSRNOT00000017071	0.951448131	1.96E-06
TUCP_001002	XLOC_037807	ENSRNOT00000014103	0.957830293	9.78E-07
TUCP_001002	XLOC_037807	ENSRNOT00000057433	0.987288496	2.56E-09
TUCP_001002	XLOC_037807	ENSRNOT00000082289	0.965800467	3.48E-07
TUCP_001002	XLOC_037807	ENSRNOT00000092516	0.972032854	1.29E-07
TUCP_001002	XLOC_037807	ENSRNOT00000082143	0.987825302	2.06E-09
TUCP_001002	XLOC_037807	ENSRNOT00000085570	0.978391994	3.58E-08
TUCP_001002	XLOC_037807	ENSRNOT00000083493	0.9516184	1.92E-06
TUCP_001002	XLOC_037807	ENSRNOT00000056551	0.954729477	1.39E-06
TUCP_001002	XLOC_037807	ENSRNOT00000088893	0.957830293	9.78E-07
TUCP_001002	XLOC_037807	ENSRNOT00000076693	0.977432909	4.44E-08
TUCP_001002	XLOC_037807	ENSRNOT00000081014	0.957830293	9.78E-07
TUCP_001002	XLOC_037807	ENSRNOT00000067543	0.957830293	9.78E-07
TUCP_001002	XLOC_037807	ENSRNOT00000076802	0.981078502	1.85E-08
TUCP_001002	XLOC_037807	ENSRNOT00000050968	0.968324441	2.38E-07
TUCP_001002	XLOC_037807	ENSRNOT00000083067	0.966166731	3.30E-07
TUCP_001002	XLOC_037807	ENSRNOT00000039876	0.961230554	6.46E-07
TUCP_001002	XLOC_037807	ENSRNOT00000086036	0.956998733	1.08E-06
TUCP_001002	XLOC_037807	ENSRNOT00000050543	0.959845843	7.68E-07
TUCP_001002	XLOC_037807	ENSRNOT00000002695	0.950913932	2.07E-06
TUCP_001002	XLOC_037807	ENSRNOT00000063929	0.957830293	9.78E-07
TUCP_001002	XLOC_037807	ENSRNOT00000005726	0.978994208	3.11E-08
TUCP_001002	XLOC_037807	ENSRNOT00000078434	0.960617992	6.98E-07
TUCP_001002	XLOC_037807	ENSRNOT00000022200	0.96309556	5.07E-07
TUCP_001002	XLOC_037807	ENSRNOT00000020786	0.961800408	6.01E-07
TUCP_001002	XLOC_037807	ENSRNOT00000076182	0.957830293	9.78E-07
TUCP_001002	XLOC_037807	ENSRNOT00000030228	0.957380268	1.03E-06
TUCP_001002	XLOC_037807	ENSRNOT00000089306	0.960363855	7.21E-07
TUCP_001002	XLOC_037807	ENSRNOT00000052367	0.964583891	4.13E-07
TUCP_001002	XLOC_037807	ENSRNOT00000090658	0.957830293	9.78E-07
TUCP_001002	XLOC_037807	ENSRNOT00000047700	0.967304483	2.79E-07
TUCP_001002	XLOC_037807	ENSRNOT00000093404	0.954289637	1.46E-06
TUCP_001002	XLOC_037807	ENSRNOT00000052003	0.972489011	1.19E-07
TUCP_001002	XLOC_037807	ENSRNOT00000057472	0.957830293	9.78E-07
TUCP_001002	XLOC_037807	ENSRNOT00000065448	0.955643083	1.26E-06
TUCP_001002	XLOC_037807	ENSRNOT00000072810	0.983119569	1.05E-08
TUCP_002577	XLOC_109413	ENSRNOT00000011263	0.952620348	1.74E-06
TUCP_002577	XLOC_109413	ENSRNOT00000040773	0.966174957	3.29E-07
TUCP_002577	XLOC_109413	ENSRNOT00000049732	0.953938814	1.51E-06
TUCP_002577	XLOC_109413	ENSRNOT00000025950	0.966613847	3.09E-07
TUCP_002577	XLOC_109413	ENSRNOT00000080851	0.973010641	1.08E-07
TUCP_002577	XLOC_109413	ENSRNOT00000013732	0.958010512	9.58E-07
TUCP_002577	XLOC_109413	ENSRNOT00000040170	-0.954041883	1.49E-06

TUCP_002577	XLOC_109413	ENSRNOT00000025797	-0.973116554	1.06E-07
TUCP_002577	XLOC_109413	ENSRNOT00000084722	0.961759823	6.04E-07
TUCP_002577	XLOC_109413	ENSRNOT00000092125	0.957492024	1.02E-06
TUCP_002577	XLOC_109413	ENSRNOT00000084282	-0.953038255	1.66E-06
TUCP_002572	XLOC_108866	ENSRNOT00000065143	0.954280012	1.46E-06
TUCP_002572	XLOC_108866	ENSRNOT00000005958	0.96284581	5.24E-07
TUCP_002572	XLOC_108866	ENSRNOT00000067688	-0.952384836	1.78E-06
TUCP_002572	XLOC_108866	ENSRNOT00000088353	-0.967150348	2.85E-07
TUCP_002572	XLOC_108866	ENSRNOT00000005228	0.950814293	2.09E-06
TUCP_002572	XLOC_108866	ENSRNOT00000008677	0.952185027	1.82E-06
TUCP_002572	XLOC_108866	ENSRNOT00000011052	0.951947846	1.86E-06
TUCP_002572	XLOC_108866	ENSRNOT00000028625	-0.950594706	2.13E-06
TUCP_002572	XLOC_108866	ENSRNOT00000024149	0.950988448	2.05E-06
TUCP_002572	XLOC_108866	ENSRNOT00000082869	0.972008955	1.29E-07
TUCP_002572	XLOC_108866	ENSRNOT00000078102	0.950286517	2.20E-06
TUCP_002572	XLOC_108866	ENSRNOT00000012895	0.957843139	9.77E-07
TUCP_002572	XLOC_108866	ENSRNOT00000020322	0.955008704	1.35E-06
TUCP_002572	XLOC_108866	ENSRNOT00000050279	0.95354843	1.58E-06
TUCP_002572	XLOC_108866	ENSRNOT00000012187	0.955326757	1.30E-06
TUCP_002572	XLOC_108866	ENSRNOT00000005942	0.957203475	1.05E-06
TUCP_002572	XLOC_108866	ENSRNOT00000017753	0.96517358	3.81E-07
TUCP_002572	XLOC_108866	ENSRNOT00000024969	0.952664308	1.73E-06
TUCP_002572	XLOC_108866	ENSRNOT00000033235	0.967830506	2.57E-07
TUCP_002572	XLOC_108866	ENSRNOT00000056430	0.95511541	1.33E-06
TUCP_002572	XLOC_108866	ENSRNOT00000060362	0.954460753	1.43E-06
TUCP_002572	XLOC_108866	ENSRNOT00000090688	0.956889685	1.09E-06
TUCP_002572	XLOC_108866	ENSRNOT00000017181	-0.950279081	2.20E-06
TUCP_002572	XLOC_108866	ENSRNOT00000015520	0.952271164	1.80E-06
TUCP_002572	XLOC_108866	ENSRNOT00000010898	0.954389464	1.44E-06
TUCP_002572	XLOC_108866	ENSRNOT00000066810	0.965543385	3.61E-07
TUCP_002572	XLOC_108866	ENSRNOT00000008372	0.95753075	1.01E-06
TUCP_002572	XLOC_108866	ENSRNOT00000025372	0.955270129	1.31E-06
TUCP_002572	XLOC_108866	ENSRNOT00000070893	0.967212896	2.82E-07
TUCP_002572	XLOC_108866	ENSRNOT00000011682	-0.966429049	3.17E-07
TUCP_002572	XLOC_108866	ENSRNOT00000079177	0.953624508	1.56E-06
TUCP_002572	XLOC_108866	ENSRNOT00000031568	0.963054558	5.09E-07
TUCP_002573	XLOC_109039	ENSRNOT00000070972	0.952214468	1.81E-06
TUCP_002573	XLOC_109039	ENSRNOT00000051494	0.960908881	6.73E-07
TUCP_002573	XLOC_109039	ENSRNOT00000045533	0.956036267	1.20E-06
TUCP_002479	XLOC_105023	ENSRNOT00000074951	0.957166866	1.06E-06
TUCP_002571	XLOC_108864	ENSRNOT00000090123	0.960155539	7.40E-07
TUCP_002571	XLOC_108864	ENSRNOT00000026710	-0.95255697	1.75E-06
TUCP_002571	XLOC_108864	ENSRNOT00000065143	0.955783175	1.24E-06
TUCP_002571	XLOC_108864	ENSRNOT00000024149	0.954310469	1.45E-06
TUCP_002571	XLOC_108864	ENSRNOT00000005958	0.973300684	1.02E-07
TUCP_002571	XLOC_108864	ENSRNOT00000017063	-0.951566968	1.93E-06
TUCP_002571	XLOC_108864	ENSRNOT00000008929	0.955199938	1.32E-06
TUCP_002571	XLOC_108864	ENSRNOT00000077894	-0.961168795	6.51E-07
TUCP_002571	XLOC_108864	ENSRNOT00000082869	0.966765943	3.02E-07
TUCP_002571	XLOC_108864	ENSRNOT00000032780	0.950539948	2.14E-06
TUCP_002571	XLOC_108864	ENSRNOT00000081981	0.960870473	6.76E-07

TUCP_002571	XLOC_108864	ENSRNOT00000000728	-0.953764607	1.54E-06
TUCP_002571	XLOC_108864	ENSRNOT00000008901	-0.950100897	2.24E-06
TUCP_002571	XLOC_108864	ENSRNOT00000028625	-0.957492015	1.02E-06
TUCP_002571	XLOC_108864	ENSRNOT00000067688	-0.966299456	3.24E-07
TUCP_002571	XLOC_108864	ENSRNOT00000033235	0.968181436	2.43E-07
TUCP_002571	XLOC_108864	ENSRNOT00000021471	-0.950401079	2.17E-06
TUCP_002571	XLOC_108864	ENSRNOT00000023133	0.953319431	1.61E-06
TUCP_002571	XLOC_108864	ENSRNOT00000025936	-0.963880287	4.56E-07
TUCP_002571	XLOC_108864	ENSRNOT00000073559	0.95099226	2.05E-06
TUCP_002571	XLOC_108864	ENSRNOT00000001290	0.96187426	5.95E-07
TUCP_002571	XLOC_108864	ENSRNOT00000002410	0.956562445	1.13E-06
TUCP_002571	XLOC_108864	ENSRNOT00000077406	0.950543866	2.14E-06
TUCP_002571	XLOC_108864	ENSRNOT00000005228	0.966561512	3.11E-07
TUCP_002571	XLOC_108864	ENSRNOT00000010748	0.950797376	2.09E-06
TUCP_002571	XLOC_108864	ENSRNOT00000008677	0.963607674	4.73E-07
TUCP_002571	XLOC_108864	ENSRNOT00000008757	0.95320258	1.63E-06
TUCP_002571	XLOC_108864	ENSRNOT00000070893	0.970650869	1.63E-07
TUCP_002571	XLOC_108864	ENSRNOT00000079177	0.958428372	9.12E-07
TUCP_002571	XLOC_108864	ENSRNOT00000081768	0.95488334	1.36E-06
TUCP_002571	XLOC_108864	ENSRNOT00000035474	0.956363227	1.16E-06
TUCP_002571	XLOC_108864	ENSRNOT00000017753	0.970962334	1.55E-07
TUCP_002571	XLOC_108864	ENSRNOT00000019502	0.953203825	1.63E-06
TUCP_002571	XLOC_108864	ENSRNOT00000052224	0.969328301	2.03E-07
TUCP_002571	XLOC_108864	ENSRNOT00000088353	-0.97356119	9.73E-08
TUCP_002571	XLOC_108864	ENSRNOT00000005942	0.958970074	8.55E-07
TUCP_002571	XLOC_108864	ENSRNOT00000092029	0.95245476	1.77E-06
TUCP_002571	XLOC_108864	ENSRNOT00000084385	0.950954213	2.06E-06
TUCP_002571	XLOC_108864	ENSRNOT00000090342	0.950996101	2.05E-06
TUCP_002571	XLOC_108864	ENSRNOT00000012895	0.953532925	1.58E-06
TUCP_002571	XLOC_108864	ENSRNOT00000001586	-0.952529434	1.75E-06
TUCP_002571	XLOC_108864	ENSRNOT00000080608	0.950903086	2.07E-06
TUCP_002571	XLOC_108864	ENSRNOT00000009496	0.951756834	1.90E-06
TUCP_002571	XLOC_108864	ENSRNOT00000042125	0.959206618	8.31E-07
TUCP_002571	XLOC_108864	ENSRNOT00000078390	-0.954236127	1.46E-06
TUCP_002571	XLOC_108864	ENSRNOT00000008655	0.964665097	4.09E-07
TUCP_002571	XLOC_108864	ENSRNOT00000020322	0.956571923	1.13E-06
TUCP_002571	XLOC_108864	ENSRNOT00000021408	0.958935944	8.58E-07
TUCP_002571	XLOC_108864	ENSRNOT00000077201	-0.952476	1.76E-06
TUCP_002571	XLOC_108864	ENSRNOT00000017181	-0.959536861	7.98E-07
TUCP_002571	XLOC_108864	ENSRNOT00000087095	0.963016454	5.12E-07
TUCP_002571	XLOC_108864	ENSRNOT00000064798	0.950941869	2.06E-06
TUCP_002571	XLOC_108864	ENSRNOT00000014261	0.951970512	1.86E-06
TUCP_002571	XLOC_108864	ENSRNOT00000015799	0.955448511	1.28E-06
TUCP_002571	XLOC_108864	ENSRNOT00000015116	0.955366688	1.29E-06
TUCP_002571	XLOC_108864	ENSRNOT00000029833	0.953062734	1.66E-06
TUCP_002571	XLOC_108864	ENSRNOT00000060362	0.955109847	1.33E-06
TUCP_002571	XLOC_108864	ENSRNOT00000043535	0.952824401	1.70E-06
TUCP_002571	XLOC_108864	ENSRNOT00000028646	0.956887453	1.09E-06
TUCP_002571	XLOC_108864	ENSRNOT00000012187	0.972391266	1.21E-07
TUCP_002571	XLOC_108864	ENSRNOT00000022074	0.951587947	1.93E-06
TUCP_002571	XLOC_108864	ENSRNOT00000044628	0.954843933	1.37E-06

TUCP_002571	XLOC_108864	ENSRNOT00000081047	0.966368899	3.20E-07
TUCP_002571	XLOC_108864	ENSRNOT00000091128	-0.956766428	1.11E-06
TUCP_002571	XLOC_108864	ENSRNOT00000071493	-0.957380955	1.03E-06
TUCP_002571	XLOC_108864	ENSRNOT00000010898	0.952822242	1.70E-06
TUCP_002571	XLOC_108864	ENSRNOT00000047192	-0.970212402	1.76E-07
TUCP_002571	XLOC_108864	ENSRNOT00000066810	0.951179137	2.01E-06
TUCP_002571	XLOC_108864	ENSRNOT00000076611	-0.956634435	1.12E-06
TUCP_002571	XLOC_108864	ENSRNOT00000015520	0.958154839	9.42E-07
TUCP_002571	XLOC_108864	ENSRNOT00000052422	0.956968536	1.08E-06
TUCP_002571	XLOC_108864	ENSRNOT00000051022	0.953990395	1.50E-06
TUCP_002571	XLOC_108864	ENSRNOT00000031568	0.963134268	5.04E-07
TUCP_002571	XLOC_108864	ENSRNOT00000057146	0.952155355	1.82E-06
TUCP_002571	XLOC_108864	ENSRNOT00000019886	0.957177044	1.06E-06
TUCP_002571	XLOC_108864	ENSRNOT00000058842	0.9520241	1.85E-06
TUCP_002571	XLOC_108864	ENSRNOT00000011682	-0.977765906	4.12E-08
TUCP_002571	XLOC_108864	ENSRNOT00000083220	0.951790435	1.89E-06
TUCP_002571	XLOC_108864	ENSRNOT00000078102	0.964429105	4.22E-07
TUCP_002571	XLOC_108864	ENSRNOT00000061392	0.955219534	1.32E-06
TUCP_002571	XLOC_108864	ENSRNOT00000024969	0.964792187	4.02E-07
TUCP_002474	XLOC_104687	ENSRNOT00000006188	0.961530865	6.22E-07
TUCP_002474	XLOC_104687	ENSRNOT00000013745	0.990086806	7.42E-10
TUCP_002474	XLOC_104687	ENSRNOT00000013743	-0.955129395	1.33E-06
TUCP_002474	XLOC_104687	ENSRNOT00000075397	-0.955040291	1.34E-06
TUCP_002474	XLOC_104687	ENSRNOT00000032926	0.957663725	9.98E-07
TUCP_002474	XLOC_104687	ENSRNOT00000019104	0.975767234	6.32E-08
TUCP_002474	XLOC_104687	ENSRNOT00000019848	0.97510107	7.23E-08
TUCP_002474	XLOC_104687	ENSRNOT00000015568	0.969120147	2.10E-07
TUCP_002474	XLOC_104687	ENSRNOT00000021944	-0.952312356	1.79E-06
TUCP_002474	XLOC_104687	ENSRNOT00000002222	-0.953683332	1.55E-06
TUCP_002474	XLOC_104687	ENSRNOT00000083173	0.953493876	1.58E-06
TUCP_002474	XLOC_104687	ENSRNOT00000001691	-0.963609007	4.73E-07
TUCP_002474	XLOC_104687	ENSRNOT00000089319	-0.972927154	1.09E-07
TUCP_002474	XLOC_104687	ENSRNOT00000020267	-0.967080324	2.88E-07
TUCP_002474	XLOC_104687	ENSRNOT00000079138	-0.961644945	6.13E-07
TUCP_002474	XLOC_104687	ENSRNOT00000020265	0.961778616	6.02E-07
TUCP_002474	XLOC_104687	ENSRNOT00000078737	0.965225168	3.78E-07
TUCP_002474	XLOC_104687	ENSRNOT00000064305	0.979037445	3.08E-08
TUCP_002474	XLOC_104687	ENSRNOT00000086706	-0.959425044	8.09E-07
TUCP_002474	XLOC_104687	ENSRNOT00000004017	-0.955303007	1.30E-06
TUCP_002474	XLOC_104687	ENSRNOT00000086310	0.969236068	2.06E-07
TUCP_002474	XLOC_104687	ENSRNOT00000045233	0.954243346	1.46E-06
TUCP_002474	XLOC_104687	ENSRNOT00000011130	0.96781784	2.58E-07
TUCP_002474	XLOC_104687	ENSRNOT00000037955	0.952591808	1.74E-06
TUCP_002474	XLOC_104687	ENSRNOT00000025309	-0.957911767	9.69E-07
TUCP_002474	XLOC_104687	ENSRNOT00000025308	0.965722099	3.52E-07
TUCP_002474	XLOC_104687	ENSRNOT00000012663	0.956256176	1.17E-06
TUCP_002474	XLOC_104687	ENSRNOT00000086893	0.952817548	1.70E-06
TUCP_002474	XLOC_104687	ENSRNOT00000055540	-0.955892166	1.22E-06
TUCP_002474	XLOC_104687	ENSRNOT00000006752	-0.952894375	1.69E-06
TUCP_002474	XLOC_104687	ENSRNOT00000003247	0.967132299	2.86E-07
TUCP_002474	XLOC_104687	ENSRNOT00000085311	0.964434923	4.22E-07

TUCP_002474	XLOC_104687	ENSRNOT00000031672	0.962995892	5.13E-07
TUCP_002474	XLOC_104687	ENSRNOT00000015986	0.963076657	5.08E-07
TUCP_002474	XLOC_104687	ENSRNOT00000025284	0.987113325	2.74E-09
TUCP_002474	XLOC_104687	ENSRNOT00000018622	0.955415499	1.29E-06
TUCP_002474	XLOC_104687	ENSRNOT00000065304	0.975631974	6.50E-08
TUCP_002474	XLOC_104687	ENSRNOT00000022620	-0.979939279	2.47E-08
TUCP_002474	XLOC_104687	ENSRNOT00000004722	0.964151578	4.39E-07
TUCP_002474	XLOC_104687	ENSRNOT00000015247	0.977297695	4.57E-08
TUCP_002474	XLOC_104687	ENSRNOT00000057045	0.956042281	1.20E-06
TUCP_002474	XLOC_104687	ENSRNOT00000035320	0.983397552	9.66E-09
TUCP_002474	XLOC_104687	ENSRNOT00000022241	0.960960261	6.69E-07
TUCP_002474	XLOC_104687	ENSRNOT00000084674	0.962172181	5.72E-07
TUCP_002474	XLOC_104687	ENSRNOT00000083964	0.986834753	3.05E-09
TUCP_002474	XLOC_104687	ENSRNOT00000077999	-0.959999149	7.54E-07
TUCP_002474	XLOC_104687	ENSRNOT00000026398	0.987121178	2.73E-09
TUCP_002474	XLOC_104687	ENSRNOT00000077856	0.953396245	1.60E-06
TUCP_002474	XLOC_104687	ENSRNOT00000003794	0.955372673	1.29E-06
TUCP_002474	XLOC_104687	ENSRNOT00000015188	-0.954294264	1.45E-06
TUCP_002474	XLOC_104687	ENSRNOT00000017648	0.972238256	1.24E-07
TUCP_002474	XLOC_104687	ENSRNOT00000018488	0.956647087	1.12E-06
TUCP_002474	XLOC_104687	ENSRNOT00000039335	0.9683388	2.38E-07
TUCP_002474	XLOC_104687	ENSRNOT00000075630	0.955694637	1.25E-06
TUCP_002474	XLOC_104687	ENSRNOT00000040629	-0.978205453	3.73E-08
TUCP_002474	XLOC_104687	ENSRNOT00000083287	0.954022778	1.50E-06
TUCP_002474	XLOC_104687	ENSRNOT00000082686	0.966325795	3.22E-07
TUCP_002474	XLOC_104687	ENSRNOT00000076181	0.992748614	1.56E-10
TUCP_002474	XLOC_104687	ENSRNOT00000083283	0.954239994	1.46E-06
TUCP_002474	XLOC_104687	ENSRNOT00000080410	0.952528226	1.75E-06
TUCP_002474	XLOC_104687	ENSRNOT00000022000	0.958700866	8.83E-07
TUCP_002474	XLOC_104687	ENSRNOT00000026462	0.979254608	2.92E-08
TUCP_002474	XLOC_104687	ENSRNOT00000080418	0.967638074	2.65E-07
TUCP_002474	XLOC_104687	ENSRNOT00000067391	0.975327119	6.91E-08
TUCP_002474	XLOC_104687	ENSRNOT00000029076	0.956901659	1.09E-06
TUCP_002474	XLOC_104687	ENSRNOT00000029179	0.966037959	3.36E-07
TUCP_002474	XLOC_104687	ENSRNOT00000026481	-0.951453533	1.96E-06
TUCP_002474	XLOC_104687	ENSRNOT00000001322	0.964969864	3.92E-07
TUCP_002474	XLOC_104687	ENSRNOT00000056145	0.961899888	5.93E-07
TUCP_002474	XLOC_104687	ENSRNOT00000023638	-0.950752644	2.10E-06
TUCP_002474	XLOC_104687	ENSRNOT00000026525	0.964106518	4.42E-07
TUCP_002474	XLOC_104687	ENSRNOT00000011082	0.968139251	2.45E-07
TUCP_002474	XLOC_104687	ENSRNOT00000025856	0.964496501	4.19E-07
TUCP_002474	XLOC_104687	ENSRNOT00000079644	0.956924142	1.09E-06
TUCP_002474	XLOC_104687	ENSRNOT00000000817	0.97271663	1.14E-07
TUCP_002474	XLOC_104687	ENSRNOT00000084313	0.990527012	5.91E-10
TUCP_002474	XLOC_104687	ENSRNOT00000001401	-0.976274051	5.69E-08
TUCP_002474	XLOC_104687	ENSRNOT00000016132	0.968622594	2.27E-07
TUCP_002474	XLOC_104687	ENSRNOT00000001366	0.95680279	1.10E-06
TUCP_002474	XLOC_104687	ENSRNOT00000058495	0.960512201	7.08E-07
TUCP_002474	XLOC_104687	ENSRNOT00000013067	0.961491854	6.25E-07
TUCP_002474	XLOC_104687	ENSRNOT00000016520	-0.952459556	1.77E-06
TUCP_002474	XLOC_104687	ENSRNOT00000001360	0.991440805	3.57E-10

TUCP_002474	XLOC_104687	ENSRNOT00000023506	0.959106305	8.41E-07
TUCP_002474	XLOC_104687	ENSRNOT00000016220	0.966975258	2.93E-07
TUCP_002474	XLOC_104687	ENSRNOT00000017081	0.969008342	2.14E-07
TUCP_002474	XLOC_104687	ENSRNOT00000002313	0.953440354	1.59E-06
TUCP_002474	XLOC_104687	ENSRNOT00000037389	0.969010606	2.14E-07
TUCP_002474	XLOC_104687	ENSRNOT00000016637	0.954074018	1.49E-06
TUCP_002474	XLOC_104687	ENSRNOT00000083995	0.960675061	6.93E-07
TUCP_002474	XLOC_104687	ENSRNOT00000065288	0.971568757	1.39E-07
TUCP_002474	XLOC_104687	ENSRNOT00000020910	-0.951035078	2.04E-06
TUCP_002474	XLOC_104687	ENSRNOT00000041815	0.966786686	3.01E-07
TUCP_002474	XLOC_104687	ENSRNOT00000060607	0.972415535	1.20E-07
TUCP_002474	XLOC_104687	ENSRNOT00000054948	-0.956508014	1.14E-06
TUCP_002474	XLOC_104687	ENSRNOT00000074586	0.9550135	1.35E-06
TUCP_002474	XLOC_104687	ENSRNOT00000045765	0.9717209	1.36E-07
TUCP_002474	XLOC_104687	ENSRNOT00000071929	0.967798724	2.58E-07
TUCP_002474	XLOC_104687	ENSRNOT00000024992	0.952587729	1.74E-06
TUCP_002474	XLOC_104687	ENSRNOT00000000532	0.991735452	2.99E-10
TUCP_002474	XLOC_104687	ENSRNOT00000002465	-0.95795316	9.64E-07
TUCP_002474	XLOC_104687	ENSRNOT00000029202	0.965971032	3.39E-07
TUCP_002474	XLOC_104687	ENSRNOT00000023989	-0.953602768	1.57E-06
TUCP_002474	XLOC_104687	ENSRNOT00000003992	0.973022752	1.08E-07
TUCP_002474	XLOC_104687	ENSRNOT00000016351	0.964907815	3.95E-07
TUCP_002474	XLOC_104687	ENSRNOT00000050515	-0.972813436	1.12E-07
TUCP_002474	XLOC_104687	ENSRNOT00000021152	-0.976048363	5.96E-08
TUCP_002474	XLOC_104687	ENSRNOT00000078073	0.95496287	1.35E-06
TUCP_002474	XLOC_104687	ENSRNOT00000011401	0.974561727	8.04E-08
TUCP_002474	XLOC_104687	ENSRNOT00000055602	0.979246934	2.93E-08
TUCP_002474	XLOC_104687	ENSRNOT00000055607	0.965347091	3.71E-07
TUCP_002474	XLOC_104687	ENSRNOT00000031555	0.950124225	2.23E-06
TUCP_002474	XLOC_104687	ENSRNOT00000011563	0.950890237	2.07E-06
TUCP_002474	XLOC_104687	ENSRNOT00000000263	-0.971839119	1.33E-07
TUCP_002474	XLOC_104687	ENSRNOT00000086350	0.955541393	1.27E-06
TUCP_002474	XLOC_104687	ENSRNOT00000023876	-0.964305857	4.30E-07
TUCP_002474	XLOC_104687	ENSRNOT00000003106	0.954269114	1.46E-06
TUCP_002474	XLOC_104687	ENSRNOT00000002187	0.958731693	8.79E-07
TUCP_002474	XLOC_104687	ENSRNOT00000012625	0.978102445	3.82E-08
TUCP_002474	XLOC_104687	ENSRNOT00000009840	0.952150576	1.82E-06
TUCP_002474	XLOC_104687	ENSRNOT00000049285	0.971729979	1.36E-07
TUCP_002474	XLOC_104687	ENSRNOT00000006366	0.985878886	4.32E-09
TUCP_002474	XLOC_104687	ENSRNOT00000074649	-0.954697317	1.39E-06
TUCP_002474	XLOC_104687	ENSRNOT00000049536	0.961298158	6.41E-07
TUCP_002474	XLOC_104687	ENSRNOT00000035277	-0.95035198	2.19E-06
TUCP_002474	XLOC_104687	ENSRNOT00000033196	0.958375704	9.18E-07
TUCP_002474	XLOC_104687	ENSRNOT00000077146	0.956646309	1.12E-06
TUCP_002474	XLOC_104687	ENSRNOT00000022478	0.970918267	1.56E-07
TUCP_002474	XLOC_104687	ENSRNOT00000042459	0.956835239	1.10E-06
TUCP_002474	XLOC_104687	ENSRNOT00000064723	0.972667871	1.15E-07
TUCP_002474	XLOC_104687	ENSRNOT00000073834	0.978995254	3.11E-08
TUCP_002474	XLOC_104687	ENSRNOT00000028807	0.961065391	6.60E-07
TUCP_002474	XLOC_104687	ENSRNOT00000060002	0.960697602	6.91E-07
TUCP_002474	XLOC_104687	ENSRNOT00000086456	0.968672104	2.25E-07

TUCP_002474	XLOC_104687	ENSRNOT00000036959	0.979585832	2.70E-08
TUCP_002474	XLOC_104687	ENSRNOT00000027301	0.980101358	2.38E-08
TUCP_002474	XLOC_104687	ENSRNOT00000075209	0.954958799	1.35E-06
TUCP_002474	XLOC_104687	ENSRNOT00000089185	0.972764977	1.13E-07
TUCP_002474	XLOC_104687	ENSRNOT00000023977	0.976268599	5.70E-08
TUCP_002474	XLOC_104687	ENSRNOT00000066133	0.953824572	1.53E-06
TUCP_002474	XLOC_104687	ENSRNOT00000017420	0.952544479	1.75E-06
TUCP_002474	XLOC_104687	ENSRNOT00000009666	0.954372664	1.44E-06
TUCP_002474	XLOC_104687	ENSRNOT00000051953	0.951547644	1.94E-06
TUCP_002474	XLOC_104687	ENSRNOT00000006765	0.978681682	3.35E-08
TUCP_002474	XLOC_104687	ENSRNOT00000005604	-0.951069477	2.03E-06
TUCP_002474	XLOC_104687	ENSRNOT00000035385	-0.974859089	7.58E-08
TUCP_002474	XLOC_104687	ENSRNOT00000025067	0.961937141	5.90E-07
TUCP_002474	XLOC_104687	ENSRNOT00000086628	-0.963283455	4.94E-07
TUCP_002474	XLOC_104687	ENSRNOT00000008317	0.952560383	1.75E-06
TUCP_002474	XLOC_104687	ENSRNOT00000014559	0.954486713	1.42E-06
TUCP_002474	XLOC_104687	ENSRNOT00000005644	0.950391808	2.18E-06
TUCP_002474	XLOC_104687	ENSRNOT00000014885	0.984369662	7.16E-09
TUCP_002474	XLOC_104687	ENSRNOT00000023786	0.968028328	2.49E-07
TUCP_002474	XLOC_104687	ENSRNOT00000040548	0.959724526	7.80E-07
TUCP_002474	XLOC_104687	ENSRNOT00000028290	0.973266056	1.03E-07
TUCP_002474	XLOC_104687	ENSRNOT00000064535	0.955610489	1.26E-06
TUCP_002474	XLOC_104687	ENSRNOT00000015624	-0.960495292	7.09E-07
TUCP_002474	XLOC_104687	ENSRNOT00000013867	-0.959392743	8.12E-07
TUCP_002474	XLOC_104687	ENSRNOT00000085295	0.970578172	1.65E-07
TUCP_002474	XLOC_104687	ENSRNOT00000041091	0.962130196	5.76E-07
TUCP_002474	XLOC_104687	ENSRNOT00000008222	0.975964827	6.07E-08
TUCP_002474	XLOC_104687	ENSRNOT00000010962	0.975200197	7.09E-08
TUCP_002474	XLOC_104687	ENSRNOT00000020702	0.98916998	1.15E-09
TUCP_002474	XLOC_104687	ENSRNOT00000009749	0.982344722	1.31E-08
TUCP_002474	XLOC_104687	ENSRNOT00000039102	-0.969119778	2.10E-07
TUCP_002474	XLOC_104687	ENSRNOT00000086260	0.952069993	1.84E-06
TUCP_002474	XLOC_104687	ENSRNOT00000001135	0.973237763	1.03E-07
TUCP_002474	XLOC_104687	ENSRNOT00000013933	0.956121366	1.19E-06
TUCP_002474	XLOC_104687	ENSRNOT00000001036	0.966651822	3.07E-07
TUCP_002474	XLOC_104687	ENSRNOT00000060692	0.958986972	8.53E-07
TUCP_002474	XLOC_104687	ENSRNOT00000010983	-0.970422723	1.70E-07
TUCP_002474	XLOC_104687	ENSRNOT00000007369	-0.954851057	1.37E-06
TUCP_002474	XLOC_104687	ENSRNOT00000004787	0.966551134	3.12E-07
TUCP_002474	XLOC_104687	ENSRNOT00000085466	-0.963210906	4.99E-07
TUCP_002474	XLOC_104687	ENSRNOT00000038811	0.95024628	2.21E-06
TUCP_002474	XLOC_104687	ENSRNOT00000007367	0.967023621	2.91E-07
TUCP_002474	XLOC_104687	ENSRNOT00000060757	0.965474977	3.65E-07
TUCP_002474	XLOC_104687	ENSRNOT00000027970	-0.951985009	1.85E-06
TUCP_002474	XLOC_104687	ENSRNOT00000045946	-0.951595995	1.93E-06
TUCP_002474	XLOC_104687	ENSRNOT00000005148	0.966652606	3.07E-07
TUCP_002474	XLOC_104687	ENSRNOT00000017998	-0.950699211	2.11E-06
TUCP_002474	XLOC_104687	ENSRNOT00000017995	-0.964156519	4.39E-07
TUCP_002474	XLOC_104687	ENSRNOT00000060595	0.954820066	1.37E-06
TUCP_002474	XLOC_104687	ENSRNOT00000013431	0.992509767	1.83E-10
TUCP_002474	XLOC_104687	ENSRNOT00000028703	0.978849003	3.22E-08

TUCP_002474	XLOC_104687	ENSRNOT00000021534	0.965169844	3.81E-07
TUCP_002474	XLOC_104687	ENSRNOT00000023628	0.973252674	1.03E-07
TUCP_002474	XLOC_104687	ENSRNOT00000088679	0.954179856	1.47E-06
TUCP_002474	XLOC_104687	ENSRNOT00000023256	-0.962045576	5.82E-07
TUCP_002474	XLOC_104687	ENSRNOT00000016776	0.977967585	3.94E-08
TUCP_002474	XLOC_104687	ENSRNOT00000061619	0.953002103	1.67E-06
TUCP_002474	XLOC_104687	ENSRNOT00000088677	0.982720787	1.18E-08
TUCP_002474	XLOC_104687	ENSRNOT00000008987	0.97082372	1.59E-07
TUCP_002474	XLOC_104687	ENSRNOT00000061580	0.964963214	3.92E-07
TUCP_002474	XLOC_104687	ENSRNOT00000024253	-0.951132099	2.02E-06
TUCP_002474	XLOC_104687	ENSRNOT00000019473	0.952556115	1.75E-06
TUCP_002474	XLOC_104687	ENSRNOT00000091116	0.955123913	1.33E-06
TUCP_002474	XLOC_104687	ENSRNOT00000059854	0.95342711	1.60E-06
TUCP_002474	XLOC_104687	ENSRNOT00000039607	0.973072584	1.07E-07
TUCP_002474	XLOC_104687	ENSRNOT00000027083	-0.950878401	2.07E-06
TUCP_002474	XLOC_104687	ENSRNOT00000002053	0.970391703	1.71E-07
TUCP_002474	XLOC_104687	ENSRNOT00000063930	0.971936956	1.31E-07
TUCP_002474	XLOC_104687	ENSRNOT00000074420	0.950928485	2.06E-06
TUCP_002474	XLOC_104687	ENSRNOT00000046215	0.986727299	3.17E-09
TUCP_002474	XLOC_104687	ENSRNOT00000002037	0.966028253	3.37E-07
TUCP_002474	XLOC_104687	ENSRNOT00000020155	-0.959129622	8.38E-07
TUCP_002474	XLOC_104687	ENSRNOT00000001855	-0.952456064	1.77E-06
TUCP_002474	XLOC_104687	ENSRNOT00000072771	-0.968762865	2.22E-07
TUCP_002474	XLOC_104687	ENSRNOT00000041237	0.975911355	6.13E-08
TUCP_002474	XLOC_104687	ENSRNOT00000006050	0.963963839	4.50E-07
TUCP_002474	XLOC_104687	ENSRNOT00000002040	0.966326097	3.22E-07
TUCP_002474	XLOC_104687	ENSRNOT00000003733	0.95988827	7.64E-07
TUCP_002474	XLOC_104687	ENSRNOT00000089829	0.966180071	3.29E-07
TUCP_002474	XLOC_104687	ENSRNOT00000013613	0.961069426	6.60E-07
TUCP_002474	XLOC_104687	ENSRNOT00000027653	0.965883122	3.44E-07
TUCP_002474	XLOC_104687	ENSRNOT00000067566	0.964069386	4.44E-07
TUCP_002474	XLOC_104687	ENSRNOT00000014479	0.975505618	6.66E-08
TUCP_002474	XLOC_104687	ENSRNOT00000003326	0.976780851	5.11E-08
TUCP_002474	XLOC_104687	ENSRNOT00000016306	0.970143969	1.78E-07
TUCP_002474	XLOC_104687	ENSRNOT00000006205	0.955492925	1.28E-06
TUCP_002474	XLOC_104687	ENSRNOT00000086813	0.953969475	1.51E-06
TUCP_002474	XLOC_104687	ENSRNOT00000015476	0.954464894	1.43E-06
TUCP_002474	XLOC_104687	ENSRNOT00000017692	0.98789173	2.01E-09
TUCP_002474	XLOC_104687	ENSRNOT00000047530	-0.963924312	4.53E-07
TUCP_002474	XLOC_104687	ENSRNOT00000021455	0.966566691	3.11E-07
TUCP_002474	XLOC_104687	ENSRNOT00000064671	0.9565498	1.13E-06
TUCP_002474	XLOC_104687	ENSRNOT00000007824	0.974490145	8.15E-08
TUCP_002474	XLOC_104687	ENSRNOT00000000120	-0.95663176	1.12E-06
TUCP_002474	XLOC_104687	ENSRNOT00000057898	0.984071776	7.86E-09
TUCP_002474	XLOC_104687	ENSRNOT00000024677	-0.953242716	1.63E-06
TUCP_002474	XLOC_104687	ENSRNOT00000091602	0.95554346	1.27E-06
TUCP_002474	XLOC_104687	ENSRNOT00000090338	0.961529008	6.22E-07
TUCP_002474	XLOC_104687	ENSRNOT00000056100	0.974566097	8.03E-08
TUCP_002474	XLOC_104687	ENSRNOT00000001077	-0.970481393	1.68E-07
TUCP_002474	XLOC_104687	ENSRNOT00000007044	0.966702642	3.05E-07
TUCP_002474	XLOC_104687	ENSRNOT00000023567	0.97601757	6.00E-08

TUCP_002474	XLOC_104687	ENSRNOT00000009817	0.98010169	2.38E-08
TUCP_002474	XLOC_104687	ENSRNOT00000026258	-0.950448424	2.16E-06
TUCP_002474	XLOC_104687	ENSRNOT00000025294	0.980382095	2.21E-08
TUCP_002474	XLOC_104687	ENSRNOT00000015618	0.957480258	1.02E-06
TUCP_002474	XLOC_104687	ENSRNOT00000018634	0.961785174	6.02E-07
TUCP_002474	XLOC_104687	ENSRNOT00000071540	-0.963732184	4.65E-07
TUCP_002474	XLOC_104687	ENSRNOT00000012583	0.970226732	1.75E-07
TUCP_002474	XLOC_104687	ENSRNOT00000024568	0.957859428	9.75E-07
TUCP_002474	XLOC_104687	ENSRNOT00000091739	0.962449056	5.52E-07
TUCP_002474	XLOC_104687	ENSRNOT00000072849	0.952492654	1.76E-06
TUCP_002474	XLOC_104687	ENSRNOT00000073643	0.973992036	8.97E-08
TUCP_002474	XLOC_104687	ENSRNOT00000039523	-0.954607315	1.41E-06
TUCP_002474	XLOC_104687	ENSRNOT00000078349	0.983264591	1.01E-08
TUCP_002474	XLOC_104687	ENSRNOT00000011654	0.965226125	3.78E-07
TUCP_002474	XLOC_104687	ENSRNOT00000022502	0.983142245	1.04E-08
TUCP_002474	XLOC_104687	ENSRNOT00000005961	0.967465399	2.72E-07
TUCP_002474	XLOC_104687	ENSRNOT00000026928	0.982431309	1.28E-08
TUCP_002474	XLOC_104687	ENSRNOT00000026920	0.970401198	1.70E-07
TUCP_002474	XLOC_104687	ENSRNOT00000078816	-0.950308816	2.19E-06
TUCP_002474	XLOC_104687	ENSRNOT00000085545	0.959483702	8.03E-07
TUCP_002474	XLOC_104687	ENSRNOT00000009900	0.961489071	6.25E-07
TUCP_002474	XLOC_104687	ENSRNOT00000021802	-0.974597163	7.98E-08
TUCP_002474	XLOC_104687	ENSRNOT00000021803	0.966936332	2.94E-07
TUCP_002474	XLOC_104687	ENSRNOT00000018646	0.955361038	1.29E-06
TUCP_002474	XLOC_104687	ENSRNOT00000024352	-0.951017398	2.04E-06
TUCP_002474	XLOC_104687	ENSRNOT00000039850	0.959604491	7.91E-07
TUCP_002474	XLOC_104687	ENSRNOT00000082627	0.950208059	2.22E-06
TUCP_002474	XLOC_104687	ENSRNOT00000087748	0.981029014	1.87E-08
TUCP_002474	XLOC_104687	ENSRNOT00000003787	0.97237769	1.21E-07
TUCP_002474	XLOC_104687	ENSRNOT00000014506	0.964637715	4.10E-07
TUCP_002474	XLOC_104687	ENSRNOT00000023262	0.966671643	3.06E-07
TUCP_002474	XLOC_104687	ENSRNOT00000059246	-0.95502328	1.34E-06
TUCP_002474	XLOC_104687	ENSRNOT00000017469	0.975940688	6.10E-08
TUCP_002474	XLOC_104687	ENSRNOT00000014859	0.966023702	3.37E-07
TUCP_002474	XLOC_104687	ENSRNOT00000001916	0.973003052	1.08E-07
TUCP_002474	XLOC_104687	ENSRNOT00000022343	0.959704364	7.82E-07
TUCP_002474	XLOC_104687	ENSRNOT00000004941	0.98724148	2.61E-09
TUCP_002474	XLOC_104687	ENSRNOT00000008865	0.952007825	1.85E-06
TUCP_002474	XLOC_104687	ENSRNOT00000010893	0.964330569	4.28E-07
TUCP_002474	XLOC_104687	ENSRNOT00000055957	0.989613629	9.35E-10
TUCP_002474	XLOC_104687	ENSRNOT00000010674	0.988862078	1.32E-09
TUCP_002474	XLOC_104687	ENSRNOT00000023664	0.98782556	2.06E-09
TUCP_002474	XLOC_104687	ENSRNOT00000009225	0.954559309	1.41E-06
TUCP_002474	XLOC_104687	ENSRNOT00000016730	0.952790468	1.71E-06
TUCP_002474	XLOC_104687	ENSRNOT00000068026	0.957429062	1.03E-06
TUCP_002474	XLOC_104687	ENSRNOT00000090456	-0.951486007	1.95E-06
TUCP_002474	XLOC_104687	ENSRNOT00000014821	0.957725307	9.90E-07
TUCP_002474	XLOC_104687	ENSRNOT00000007259	0.973974298	9.00E-08
TUCP_002474	XLOC_104687	ENSRNOT00000018549	0.96918227	2.08E-07
TUCP_002474	XLOC_104687	ENSRNOT00000026531	0.959007498	8.51E-07
TUCP_002474	XLOC_104687	ENSRNOT00000064068	0.971486353	1.42E-07

TUCP_002474	XLOC_104687	ENSRNOT00000005172	-0.96664603	3.07E-07
TUCP_002474	XLOC_104687	ENSRNOT00000005170	0.974658059	7.89E-08
TUCP_002474	XLOC_104687	ENSRNOT00000017746	0.957263343	1.04E-06
TUCP_002474	XLOC_104687	ENSRNOT00000016286	0.95840465	9.14E-07
TUCP_002474	XLOC_104687	ENSRNOT00000070825	0.955280944	1.31E-06
TUCP_002474	XLOC_104687	ENSRNOT00000003412	0.97414837	8.71E-08
TUCP_002474	XLOC_104687	ENSRNOT00000056015	0.983560558	9.20E-09
TUCP_002474	XLOC_104687	ENSRNOT00000021216	-0.958860346	8.66E-07
TUCP_002474	XLOC_104687	ENSRNOT00000066923	0.959211768	8.30E-07
TUCP_002474	XLOC_104687	ENSRNOT00000024285	0.978720769	3.32E-08
TUCP_002474	XLOC_104687	ENSRNOT00000041277	0.971166892	1.50E-07
TUCP_002474	XLOC_104687	ENSRNOT00000071482	0.964958673	3.92E-07
TUCP_002474	XLOC_104687	ENSRNOT00000028744	0.975568325	6.58E-08
TUCP_002474	XLOC_104687	ENSRNOT00000009135	-0.951170441	2.01E-06
TUCP_002474	XLOC_104687	ENSRNOT00000044073	0.989568418	9.56E-10
TUCP_002474	XLOC_104687	ENSRNOT00000002771	-0.95146916	1.95E-06
TUCP_002474	XLOC_104687	ENSRNOT00000067135	0.962927325	5.18E-07
TUCP_002474	XLOC_104687	ENSRNOT00000054976	0.971110196	1.51E-07
TUCP_002474	XLOC_104687	ENSRNOT00000010516	0.965481059	3.64E-07
TUCP_002474	XLOC_104687	ENSRNOT00000028887	0.965910389	3.42E-07
TUCP_002474	XLOC_104687	ENSRNOT00000000527	0.954098176	1.49E-06
TUCP_002474	XLOC_104687	ENSRNOT00000010510	0.956753407	1.11E-06
TUCP_002474	XLOC_104687	ENSRNOT00000000258	0.962760413	5.30E-07
TUCP_002474	XLOC_104687	ENSRNOT00000000529	0.96590636	3.43E-07
TUCP_002474	XLOC_104687	ENSRNOT00000081110	0.95029817	2.20E-06
TUCP_002474	XLOC_104687	ENSRNOT00000067901	0.950266618	2.20E-06
TUCP_002474	XLOC_104687	ENSRNOT00000071745	0.959404599	8.11E-07
TUCP_002474	XLOC_104687	ENSRNOT00000001734	0.961961728	5.88E-07
TUCP_002474	XLOC_104687	ENSRNOT00000088202	0.990453229	6.15E-10
TUCP_002474	XLOC_104687	ENSRNOT00000088201	0.953686408	1.55E-06
TUCP_002474	XLOC_104687	ENSRNOT00000011874	-0.952074687	1.84E-06
TUCP_002474	XLOC_104687	ENSRNOT00000061916	0.963963769	4.50E-07
TUCP_002474	XLOC_104687	ENSRNOT00000078161	0.976494371	5.43E-08
TUCP_002474	XLOC_104687	ENSRNOT00000021145	0.972618837	1.16E-07
TUCP_002474	XLOC_104687	ENSRNOT00000003600	0.958882917	8.64E-07
TUCP_002474	XLOC_104687	ENSRNOT00000031401	0.954997172	1.35E-06
TUCP_002474	XLOC_104687	ENSRNOT00000006802	-0.962644145	5.38E-07
TUCP_002474	XLOC_104687	ENSRNOT00000003605	0.971430335	1.43E-07
TUCP_002474	XLOC_104687	ENSRNOT00000082151	0.981353373	1.72E-08
TUCP_002474	XLOC_104687	ENSRNOT00000011572	0.984387458	7.12E-09
TUCP_002474	XLOC_104687	ENSRNOT00000000155	-0.954216981	1.47E-06
TUCP_002474	XLOC_104687	ENSRNOT00000034411	-0.972619073	1.16E-07
TUCP_002474	XLOC_104687	ENSRNOT00000001607	-0.984818301	6.19E-09
TUCP_002474	XLOC_104687	ENSRNOT00000033324	-0.961903791	5.93E-07
TUCP_002474	XLOC_104687	ENSRNOT00000036282	-0.96793841	2.53E-07
TUCP_002474	XLOC_104687	ENSRNOT00000046126	0.96591564	3.42E-07
TUCP_002474	XLOC_104687	ENSRNOT00000085971	0.969551263	1.96E-07
TUCP_002474	XLOC_104687	ENSRNOT00000086847	0.952178219	1.82E-06
TUCP_002474	XLOC_104687	ENSRNOT00000020663	0.957991028	9.60E-07
TUCP_002474	XLOC_104687	ENSRNOT00000044975	0.951191001	2.01E-06
TUCP_002474	XLOC_104687	ENSRNOT00000007771	0.981791088	1.53E-08

TUCP_002474	XLOC_104687	ENSRNOT00000022142	-0.961592952	6.17E-07
TUCP_002474	XLOC_104687	ENSRNOT00000076300	0.983931304	8.21E-09
TUCP_002474	XLOC_104687	ENSRNOT00000014433	0.958579316	8.96E-07
TUCP_002474	XLOC_104687	ENSRNOT00000052120	-0.96634429	3.21E-07
TUCP_002474	XLOC_104687	ENSRNOT00000065764	0.961459064	6.28E-07
TUCP_002474	XLOC_104687	ENSRNOT00000019820	-0.962398927	5.56E-07
TUCP_002474	XLOC_104687	ENSRNOT00000015946	0.96023582	7.32E-07
TUCP_002474	XLOC_104687	ENSRNOT00000005257	-0.958893024	8.63E-07
TUCP_002474	XLOC_104687	ENSRNOT00000023965	0.982606605	1.22E-08
TUCP_002474	XLOC_104687	ENSRNOT00000001234	0.962920066	5.19E-07
TUCP_002474	XLOC_104687	ENSRNOT00000058763	0.981938354	1.47E-08
TUCP_002474	XLOC_104687	ENSRNOT00000090355	0.971387671	1.44E-07
TUCP_002474	XLOC_104687	ENSRNOT00000075934	0.977456807	4.42E-08
TUCP_002474	XLOC_104687	ENSRNOT00000004319	0.957173456	1.06E-06
TUCP_002474	XLOC_104687	ENSRNOT00000015769	-0.95384626	1.53E-06
TUCP_002474	XLOC_104687	ENSRNOT00000026364	0.969264615	2.05E-07
TUCP_002474	XLOC_104687	ENSRNOT00000025819	0.974162669	8.68E-08
TUCP_002474	XLOC_104687	ENSRNOT00000015318	0.976652051	5.25E-08
TUCP_002474	XLOC_104687	ENSRNOT00000028907	-0.95679559	1.10E-06
TUCP_002474	XLOC_104687	ENSRNOT00000010224	-0.952009652	1.85E-06
TUCP_002474	XLOC_104687	ENSRNOT00000045532	0.99391067	6.53E-11
TUCP_002474	XLOC_104687	ENSRNOT00000025887	0.980639644	2.07E-08
TUCP_002474	XLOC_104687	ENSRNOT00000066211	0.959929715	7.61E-07
TUCP_002474	XLOC_104687	ENSRNOT00000003940	0.962403325	5.55E-07
TUCP_002474	XLOC_104687	ENSRNOT00000017687	0.970722989	1.61E-07
TUCP_002474	XLOC_104687	ENSRNOT00000039296	-0.964142164	4.40E-07
TUCP_002474	XLOC_104687	ENSRNOT00000036195	0.956550149	1.13E-06
TUCP_002474	XLOC_104687	ENSRNOT00000059599	0.974080983	8.82E-08
TUCP_002474	XLOC_104687	ENSRNOT00000039291	0.950898629	2.07E-06
TUCP_002474	XLOC_104687	ENSRNOT00000082999	0.973338932	1.01E-07
TUCP_002474	XLOC_104687	ENSRNOT00000082998	-0.958698141	8.83E-07
TUCP_002474	XLOC_104687	ENSRNOT00000075656	0.962110355	5.77E-07
TUCP_002474	XLOC_104687	ENSRNOT00000004810	-0.951015742	2.05E-06
TUCP_002474	XLOC_104687	ENSRNOT00000011237	0.960901452	6.74E-07
TUCP_002474	XLOC_104687	ENSRNOT00000083573	0.95608504	1.19E-06
TUCP_002474	XLOC_104687	ENSRNOT00000083578	-0.966366313	3.20E-07
TUCP_002474	XLOC_104687	ENSRNOT00000045443	0.954581749	1.41E-06
TUCP_002474	XLOC_104687	ENSRNOT00000059524	0.979689062	2.63E-08
TUCP_002474	XLOC_104687	ENSRNOT00000050721	0.967335986	2.77E-07
TUCP_002474	XLOC_104687	ENSRNOT00000017361	-0.961368025	6.35E-07
TUCP_002474	XLOC_104687	ENSRNOT00000017362	0.983764369	8.65E-09
TUCP_002474	XLOC_104687	ENSRNOT00000005658	0.972546614	1.17E-07
TUCP_002474	XLOC_104687	ENSRNOT00000027125	0.953306297	1.62E-06
TUCP_002474	XLOC_104687	ENSRNOT00000090342	-0.950995491	2.05E-06
TUCP_002474	XLOC_104687	ENSRNOT00000074023	-0.965175918	3.80E-07
TUCP_002474	XLOC_104687	ENSRNOT00000019465	0.982219813	1.36E-08
TUCP_002474	XLOC_104687	ENSRNOT00000009073	0.978051848	3.87E-08
TUCP_002474	XLOC_104687	ENSRNOT00000024712	0.966545626	3.12E-07
TUCP_002474	XLOC_104687	ENSRNOT00000067170	0.972749424	1.13E-07
TUCP_002474	XLOC_104687	ENSRNOT00000060432	0.973003594	1.08E-07
TUCP_002474	XLOC_104687	ENSRNOT00000001586	0.968655285	2.26E-07

TUCP_002474	XLOC_104687	ENSRNOT00000029332	0.950399187	2.17E-06
TUCP_002474	XLOC_104687	ENSRNOT00000017385	0.955372179	1.29E-06
TUCP_002474	XLOC_104687	ENSRNOT00000013516	-0.952849726	1.70E-06
TUCP_002474	XLOC_104687	ENSRNOT00000013043	0.950588879	2.13E-06
TUCP_002474	XLOC_104687	ENSRNOT00000013695	0.977269185	4.60E-08
TUCP_002474	XLOC_104687	ENSRNOT00000016541	0.972238214	1.24E-07
TUCP_002474	XLOC_104687	ENSRNOT00000081621	-0.980979956	1.90E-08
TUCP_002474	XLOC_104687	ENSRNOT00000012060	-0.961641725	6.13E-07
TUCP_002474	XLOC_104687	ENSRNOT00000040060	0.952688562	1.72E-06
TUCP_002474	XLOC_104687	ENSRNOT00000030373	0.953546433	1.58E-06
TUCP_002474	XLOC_104687	ENSRNOT00000005033	0.967154391	2.85E-07
TUCP_002474	XLOC_104687	ENSRNOT00000075480	0.959039683	8.48E-07
TUCP_002474	XLOC_104687	ENSRNOT00000020051	-0.955589226	1.26E-06
TUCP_002474	XLOC_104687	ENSRNOT00000014044	0.950206614	2.22E-06
TUCP_002474	XLOC_104687	ENSRNOT00000009000	0.977022038	4.85E-08
TUCP_002474	XLOC_104687	ENSRNOT00000012114	0.972070371	1.28E-07
TUCP_002474	XLOC_104687	ENSRNOT00000068033	-0.950101704	2.24E-06
TUCP_002474	XLOC_104687	ENSRNOT00000068601	0.957146207	1.06E-06
TUCP_002474	XLOC_104687	ENSRNOT00000085403	-0.967696416	2.62E-07
TUCP_002474	XLOC_104687	ENSRNOT00000089545	0.976197756	5.78E-08
TUCP_002474	XLOC_104687	ENSRNOT00000079185	0.985588628	4.78E-09
TUCP_002474	XLOC_104687	ENSRNOT00000077908	-0.959550819	7.97E-07
TUCP_002474	XLOC_104687	ENSRNOT00000083876	0.965724832	3.52E-07
TUCP_002474	XLOC_104687	ENSRNOT00000065673	0.9698355	1.87E-07
TUCP_002474	XLOC_104687	ENSRNOT00000073983	-0.952050627	1.84E-06
TUCP_002474	XLOC_104687	ENSRNOT00000010783	0.955973569	1.21E-06
TUCP_002474	XLOC_104687	ENSRNOT00000011948	0.965986142	3.39E-07
TUCP_002474	XLOC_104687	ENSRNOT00000033677	0.964092288	4.43E-07
TUCP_002474	XLOC_104687	ENSRNOT00000016330	0.970863484	1.57E-07
TUCP_002474	XLOC_104687	ENSRNOT00000019121	0.96705299	2.89E-07
TUCP_002474	XLOC_104687	ENSRNOT00000011499	0.970236522	1.75E-07
TUCP_002474	XLOC_104687	ENSRNOT00000016389	0.971549005	1.40E-07
TUCP_002474	XLOC_104687	ENSRNOT00000036338	0.955696521	1.25E-06
TUCP_002474	XLOC_104687	ENSRNOT00000021030	0.970744619	1.61E-07
TUCP_002474	XLOC_104687	ENSRNOT00000047572	0.964607174	4.12E-07
TUCP_002474	XLOC_104687	ENSRNOT00000070794	0.963183413	5.01E-07
TUCP_002474	XLOC_104687	ENSRNOT00000020932	0.971079705	1.52E-07
TUCP_002474	XLOC_104687	ENSRNOT00000073492	0.984515724	6.83E-09
TUCP_002474	XLOC_104687	ENSRNOT00000042633	0.979539037	2.73E-08
TUCP_002474	XLOC_104687	ENSRNOT00000030975	0.957010855	1.08E-06
TUCP_002474	XLOC_104687	ENSRNOT00000092068	-0.955383029	1.29E-06
TUCP_002474	XLOC_104687	ENSRNOT00000016947	-0.957217664	1.05E-06
TUCP_002474	XLOC_104687	ENSRNOT00000091863	0.966010067	3.37E-07
TUCP_002474	XLOC_104687	ENSRNOT00000028405	0.972516494	1.18E-07
TUCP_002474	XLOC_104687	ENSRNOT00000082709	0.985635689	4.70E-09
TUCP_002474	XLOC_104687	ENSRNOT00000003544	0.964275381	4.32E-07
TUCP_002474	XLOC_104687	ENSRNOT00000022194	0.951889783	1.87E-06
TUCP_002474	XLOC_104687	ENSRNOT00000064450	0.956676925	1.12E-06
TUCP_002474	XLOC_104687	ENSRNOT00000003540	0.950845264	2.08E-06
TUCP_002474	XLOC_104687	ENSRNOT00000064452	0.968145983	2.45E-07
TUCP_002474	XLOC_104687	ENSRNOT00000025913	0.955907425	1.22E-06

TUCP_002474	XLOC_104687	ENSRNOT00000019386	0.982802958	1.15E-08
TUCP_002474	XLOC_104687	ENSRNOT00000077220	0.959985045	7.55E-07
TUCP_002474	XLOC_104687	ENSRNOT00000025916	0.957572434	1.01E-06
TUCP_002474	XLOC_104687	ENSRNOT00000030782	0.95383897	1.53E-06
TUCP_002474	XLOC_104687	ENSRNOT00000007736	0.958060664	9.52E-07
TUCP_002474	XLOC_104687	ENSRNOT00000021636	0.971903679	1.32E-07
TUCP_002474	XLOC_104687	ENSRNOT00000036658	0.975585205	6.56E-08
TUCP_002474	XLOC_104687	ENSRNOT00000089611	-0.954923444	1.36E-06
TUCP_002474	XLOC_104687	ENSRNOT00000073910	0.97031906	1.73E-07
TUCP_002474	XLOC_104687	ENSRNOT00000007738	0.975036737	7.32E-08
TUCP_002474	XLOC_104687	ENSRNOT00000022107	0.959112647	8.40E-07
TUCP_002474	XLOC_104687	ENSRNOT00000030803	0.958737006	8.79E-07
TUCP_002474	XLOC_104687	ENSRNOT00000002645	0.952487136	1.76E-06
TUCP_002474	XLOC_104687	ENSRNOT00000086119	0.960123076	7.43E-07
TUCP_002474	XLOC_104687	ENSRNOT00000008253	0.951503395	1.95E-06
TUCP_002474	XLOC_104687	ENSRNOT00000041116	0.964609573	4.12E-07
TUCP_002474	XLOC_104687	ENSRNOT00000034883	0.95045028	2.16E-06
TUCP_002474	XLOC_104687	ENSRNOT00000056918	0.952652883	1.73E-06
TUCP_002474	XLOC_104687	ENSRNOT00000025827	-0.95097336	2.05E-06
TUCP_002474	XLOC_104687	ENSRNOT00000025824	0.970399582	1.70E-07
TUCP_002474	XLOC_104687	ENSRNOT00000072357	0.966149158	3.31E-07
TUCP_002474	XLOC_104687	ENSRNOT00000000707	0.963834377	4.59E-07
TUCP_002474	XLOC_104687	ENSRNOT00000022676	0.976050167	5.96E-08
TUCP_002474	XLOC_104687	ENSRNOT00000067739	0.976675793	5.23E-08
TUCP_002474	XLOC_104687	ENSRNOT00000017232	0.983153535	1.04E-08
TUCP_002474	XLOC_104687	ENSRNOT00000086608	0.955205631	1.32E-06
TUCP_002474	XLOC_104687	ENSRNOT00000090987	0.962625393	5.39E-07
TUCP_002474	XLOC_104687	ENSRNOT00000029701	-0.96831184	2.39E-07
TUCP_002474	XLOC_104687	ENSRNOT00000014585	0.966213694	3.28E-07
TUCP_002474	XLOC_104687	ENSRNOT00000029704	0.963656154	4.70E-07
TUCP_002474	XLOC_104687	ENSRNOT00000003691	0.979549967	2.72E-08
TUCP_002474	XLOC_104687	ENSRNOT00000074261	0.95145681	1.96E-06
TUCP_002474	XLOC_104687	ENSRNOT00000051522	-0.953124085	1.65E-06
TUCP_002474	XLOC_104687	ENSRNOT00000015199	0.959801265	7.73E-07
TUCP_002474	XLOC_104687	ENSRNOT00000001227	0.958496134	9.05E-07
TUCP_002474	XLOC_104687	ENSRNOT00000004842	0.978977627	3.12E-08
TUCP_002474	XLOC_104687	ENSRNOT00000089879	0.972018906	1.29E-07
TUCP_002474	XLOC_104687	ENSRNOT00000046486	0.957282564	1.04E-06
TUCP_002474	XLOC_104687	ENSRNOT00000083351	0.95210196	1.83E-06
TUCP_002474	XLOC_104687	ENSRNOT00000018477	0.952122687	1.83E-06
TUCP_002474	XLOC_104687	ENSRNOT00000080222	0.964367014	4.26E-07
TUCP_002474	XLOC_104687	ENSRNOT00000011344	0.952246114	1.80E-06
TUCP_002474	XLOC_104687	ENSRNOT00000018473	0.951626862	1.92E-06
TUCP_002474	XLOC_104687	ENSRNOT00000030329	0.991708703	3.04E-10
TUCP_002474	XLOC_104687	ENSRNOT00000033450	0.951305045	1.99E-06
TUCP_002474	XLOC_104687	ENSRNOT00000001935	0.972084736	1.27E-07
TUCP_002474	XLOC_104687	ENSRNOT00000070912	0.961963317	5.88E-07
TUCP_002474	XLOC_104687	ENSRNOT00000016758	0.952809868	1.70E-06
TUCP_002474	XLOC_104687	ENSRNOT00000015213	0.968349198	2.37E-07
TUCP_002474	XLOC_104687	ENSRNOT00000023078	-0.959535615	7.98E-07
TUCP_002474	XLOC_104687	ENSRNOT00000004999	-0.979078946	3.05E-08

TUCP_002474	XLOC_104687	ENSRNOT00000009122	-0.965088225	3.85E-07
TUCP_002474	XLOC_104687	ENSRNOT00000013463	-0.979975481	2.45E-08
TUCP_002474	XLOC_104687	ENSRNOT00000039177	0.975747315	6.34E-08
TUCP_002474	XLOC_104687	ENSRNOT00000050617	0.955673689	1.25E-06
TUCP_002474	XLOC_104687	ENSRNOT00000013093	0.962577223	5.43E-07
TUCP_002474	XLOC_104687	ENSRNOT00000007889	0.959248034	8.27E-07
TUCP_002474	XLOC_104687	ENSRNOT00000047663	0.97510785	7.22E-08
TUCP_002474	XLOC_104687	ENSRNOT00000061191	0.963423319	4.85E-07
TUCP_002474	XLOC_104687	ENSRNOT00000005486	0.976385545	5.56E-08
TUCP_002474	XLOC_104687	ENSRNOT00000017907	0.986862654	3.01E-09
TUCP_002474	XLOC_104687	ENSRNOT00000025791	0.968090394	2.47E-07
TUCP_002474	XLOC_104687	ENSRNOT00000028434	0.971921714	1.31E-07
TUCP_002474	XLOC_104687	ENSRNOT00000025794	0.965391219	3.69E-07
TUCP_002474	XLOC_104687	ENSRNOT00000058111	0.978643881	3.38E-08
TUCP_002474	XLOC_104687	ENSRNOT00000050834	0.957458676	1.02E-06
TUCP_002474	XLOC_104687	ENSRNOT00000021705	0.96443379	4.22E-07
TUCP_002474	XLOC_104687	ENSRNOT00000009994	0.969332428	2.03E-07
TUCP_002474	XLOC_104687	ENSRNOT00000021826	-0.956393618	1.15E-06
TUCP_002474	XLOC_104687	ENSRNOT00000002114	0.969094021	2.11E-07
TUCP_002474	XLOC_104687	ENSRNOT00000002445	0.954709449	1.39E-06
TUCP_002474	XLOC_104687	ENSRNOT00000009993	0.983833162	8.47E-09
TUCP_002474	XLOC_104687	ENSRNOT00000027973	0.973973641	9.00E-08
TUCP_002474	XLOC_104687	ENSRNOT00000024841	0.951572475	1.93E-06
TUCP_002474	XLOC_104687	ENSRNOT00000006376	0.958272908	9.29E-07
TUCP_002474	XLOC_104687	ENSRNOT00000005762	0.976418566	5.52E-08
TUCP_002474	XLOC_104687	ENSRNOT00000044147	0.971245325	1.48E-07
TUCP_002474	XLOC_104687	ENSRNOT00000027875	-0.957563003	1.01E-06
TUCP_002474	XLOC_104687	ENSRNOT00000027759	0.987886388	2.01E-09
TUCP_002474	XLOC_104687	ENSRNOT00000018101	0.959553615	7.96E-07
TUCP_002474	XLOC_104687	ENSRNOT00000000083	0.98513523	5.58E-09
TUCP_002474	XLOC_104687	ENSRNOT00000028737	0.975130922	7.19E-08
TUCP_002474	XLOC_104687	ENSRNOT00000018378	0.96079687	6.83E-07
TUCP_002474	XLOC_104687	ENSRNOT00000033048	0.975271963	6.99E-08
TUCP_002474	XLOC_104687	ENSRNOT00000064636	0.961835817	5.98E-07
TUCP_002474	XLOC_104687	ENSRNOT00000028545	0.992688852	1.63E-10
TUCP_002474	XLOC_104687	ENSRNOT00000001759	0.954785394	1.38E-06
TUCP_002474	XLOC_104687	ENSRNOT00000028225	-0.963550294	4.77E-07
TUCP_002474	XLOC_104687	ENSRNOT00000019172	0.971764989	1.35E-07
TUCP_002474	XLOC_104687	ENSRNOT00000049927	0.961251056	6.45E-07
TUCP_002474	XLOC_104687	ENSRNOT00000068267	0.984908444	6.01E-09
TUCP_002474	XLOC_104687	ENSRNOT00000020994	0.962957428	5.16E-07
TUCP_002474	XLOC_104687	ENSRNOT00000068269	0.955973963	1.21E-06
TUCP_002474	XLOC_104687	ENSRNOT00000019916	0.980928768	1.92E-08
TUCP_002474	XLOC_104687	ENSRNOT00000019911	0.96129172	6.41E-07
TUCP_002474	XLOC_104687	ENSRNOT00000016372	0.971577	1.39E-07
TUCP_002474	XLOC_104687	ENSRNOT00000077727	0.961085296	6.58E-07
TUCP_002474	XLOC_104687	ENSRNOT00000033015	0.957110885	1.06E-06
TUCP_002474	XLOC_104687	ENSRNOT00000024656	0.954653603	1.40E-06
TUCP_002474	XLOC_104687	ENSRNOT00000020272	0.95627755	1.17E-06
TUCP_002474	XLOC_104687	ENSRNOT00000077163	0.957428335	1.03E-06
TUCP_002474	XLOC_104687	ENSRNOT00000064700	-0.964746519	4.04E-07

TUCP_002474	XLOC_104687	ENSRNOT00000025705	-0.961367756	6.35E-07
TUCP_002474	XLOC_104687	ENSRNOT00000010020	0.98706016	2.80E-09
TUCP_002474	XLOC_104687	ENSRNOT00000074494	0.964340594	4.28E-07
TUCP_002474	XLOC_104687	ENSRNOT00000036931	0.969287663	2.04E-07
TUCP_002474	XLOC_104687	ENSRNOT00000049193	0.965204946	3.79E-07
TUCP_002474	XLOC_104687	ENSRNOT00000036194	0.988956404	1.27E-09
TUCP_002474	XLOC_104687	ENSRNOT00000000334	0.971810417	1.34E-07
TUCP_002474	XLOC_104687	ENSRNOT00000077186	0.977663252	4.22E-08
TUCP_002474	XLOC_104687	ENSRNOT00000081314	0.974429918	8.25E-08
TUCP_002474	XLOC_104687	ENSRNOT00000039729	0.952395842	1.78E-06
TUCP_002474	XLOC_104687	ENSRNOT00000014780	0.962344003	5.60E-07
TUCP_002474	XLOC_104687	ENSRNOT00000006460	-0.950664206	2.12E-06
TUCP_002474	XLOC_104687	ENSRNOT00000014785	0.984053655	7.91E-09
TUCP_002474	XLOC_104687	ENSRNOT00000014373	0.973192134	1.04E-07
TUCP_002474	XLOC_104687	ENSRNOT00000026279	0.987549753	2.31E-09
TUCP_002474	XLOC_104687	ENSRNOT00000087062	-0.951125588	2.02E-06
TUCP_002474	XLOC_104687	ENSRNOT00000003085	0.965739993	3.51E-07
TUCP_002474	XLOC_104687	ENSRNOT00000082508	0.973510656	9.83E-08
TUCP_002474	XLOC_104687	ENSRNOT00000007169	0.958239885	9.32E-07
TUCP_002474	XLOC_104687	ENSRNOT00000056199	0.979887951	2.51E-08
TUCP_002474	XLOC_104687	ENSRNOT00000065942	0.971075318	1.52E-07
TUCP_002474	XLOC_104687	ENSRNOT00000087011	0.953996755	1.50E-06
TUCP_002474	XLOC_104687	ENSRNOT00000071166	-0.967433512	2.73E-07
TUCP_002474	XLOC_104687	ENSRNOT00000084563	0.950722061	2.11E-06
TUCP_002474	XLOC_104687	ENSRNOT00000074389	0.982546212	1.24E-08
TUCP_002474	XLOC_104687	ENSRNOT00000019180	0.957734127	9.89E-07
TUCP_002474	XLOC_104687	ENSRNOT00000066204	0.981451347	1.68E-08
TUCP_002474	XLOC_104687	ENSRNOT00000018674	0.954233091	1.46E-06
TUCP_002474	XLOC_104687	ENSRNOT00000076105	0.980096681	2.38E-08
TUCP_002474	XLOC_104687	ENSRNOT00000003012	0.981899702	1.48E-08
TUCP_002474	XLOC_104687	ENSRNOT00000022746	0.960783007	6.84E-07
TUCP_002474	XLOC_104687	ENSRNOT00000001507	-0.950557494	2.14E-06
TUCP_002474	XLOC_104687	ENSRNOT00000073569	0.972332593	1.22E-07
TUCP_002474	XLOC_104687	ENSRNOT00000087604	-0.966164064	3.30E-07
TUCP_002474	XLOC_104687	ENSRNOT00000091527	0.951736122	1.90E-06
TUCP_002474	XLOC_104687	ENSRNOT00000067741	0.957570346	1.01E-06
TUCP_002474	XLOC_104687	ENSRNOT00000005620	0.981215017	1.79E-08
TUCP_002474	XLOC_104687	ENSRNOT00000010157	-0.955287041	1.31E-06
TUCP_002474	XLOC_104687	ENSRNOT00000017375	0.960516504	7.07E-07
TUCP_002474	XLOC_104687	ENSRNOT00000039377	0.950086198	2.24E-06
TUCP_002474	XLOC_104687	ENSRNOT00000019759	-0.951121969	2.02E-06
TUCP_002474	XLOC_104687	ENSRNOT00000042903	-0.955140952	1.33E-06
TUCP_002474	XLOC_104687	ENSRNOT00000000477	0.987886122	2.01E-09
TUCP_002474	XLOC_104687	ENSRNOT00000012699	0.96520259	3.79E-07
TUCP_002474	XLOC_104687	ENSRNOT00000091139	0.952802132	1.70E-06
TUCP_002474	XLOC_104687	ENSRNOT00000012881	0.968098283	2.47E-07
TUCP_002474	XLOC_104687	ENSRNOT00000035628	0.976914619	4.97E-08
TUCP_002474	XLOC_104687	ENSRNOT00000000577	-0.950394925	2.18E-06
TUCP_002474	XLOC_104687	ENSRNOT00000000576	0.960536706	7.05E-07
TUCP_002474	XLOC_104687	ENSRNOT00000026654	0.952124764	1.83E-06
TUCP_002474	XLOC_104687	ENSRNOT00000065045	-0.959794492	7.73E-07

TUCP_002474	XLOC_104687	ENSRNOT00000001806	0.954201486	1.47E-06
TUCP_002474	XLOC_104687	ENSRNOT00000031487	-0.96367246	4.69E-07
TUCP_002474	XLOC_104687	ENSRNOT00000088133	0.974734271	7.77E-08
TUCP_002474	XLOC_104687	ENSRNOT00000016254	0.973703348	9.48E-08
TUCP_002474	XLOC_104687	ENSRNOT00000027760	-0.950262958	2.20E-06
TUCP_002474	XLOC_104687	ENSRNOT00000004007	0.970582607	1.65E-07
TUCP_002474	XLOC_104687	ENSRNOT00000001979	0.967932676	2.53E-07
TUCP_002474	XLOC_104687	ENSRNOT00000003715	0.962788682	5.28E-07
TUCP_002474	XLOC_104687	ENSRNOT00000083968	0.969698965	1.91E-07
TUCP_002474	XLOC_104687	ENSRNOT00000024119	-0.960064992	7.48E-07
TUCP_002474	XLOC_104687	ENSRNOT00000007223	0.95142687	1.96E-06
TUCP_002474	XLOC_104687	ENSRNOT00000002837	0.968333556	2.38E-07
TUCP_002474	XLOC_104687	ENSRNOT00000071192	0.958801135	8.72E-07
TUCP_002474	XLOC_104687	ENSRNOT00000026726	0.969643953	1.93E-07
TUCP_002474	XLOC_104687	ENSRNOT00000033795	0.970246071	1.75E-07
TUCP_002474	XLOC_104687	ENSRNOT00000029996	0.955238874	1.31E-06
TUCP_002474	XLOC_104687	ENSRNOT00000026831	-0.964084746	4.43E-07
TUCP_002474	XLOC_104687	ENSRNOT00000007821	-0.953041632	1.66E-06
TUCP_002474	XLOC_104687	ENSRNOT00000081688	0.951633325	1.92E-06
TUCP_002474	XLOC_104687	ENSRNOT00000037039	0.981531777	1.64E-08
TUCP_002474	XLOC_104687	ENSRNOT00000074985	0.954397283	1.44E-06
TUCP_002474	XLOC_104687	ENSRNOT00000061304	0.981068848	1.86E-08
TUCP_002474	XLOC_104687	ENSRNOT00000032363	-0.959398419	8.12E-07
TUCP_002474	XLOC_104687	ENSRNOT00000027200	0.952645262	1.73E-06
TUCP_002474	XLOC_104687	ENSRNOT00000016447	0.977335529	4.53E-08
TUCP_002474	XLOC_104687	ENSRNOT00000066888	0.961671767	6.11E-07
TUCP_002474	XLOC_104687	ENSRNOT00000078185	0.962114955	5.77E-07
TUCP_002474	XLOC_104687	ENSRNOT00000002151	0.981406379	1.70E-08
TUCP_002474	XLOC_104687	ENSRNOT00000002152	0.954145336	1.48E-06
TUCP_002474	XLOC_104687	ENSRNOT00000006824	0.964991755	3.90E-07
TUCP_002474	XLOC_104687	ENSRNOT00000012775	0.954323751	1.45E-06
TUCP_002474	XLOC_104687	ENSRNOT00000077040	0.950342469	2.19E-06
TUCP_002474	XLOC_104687	ENSRNOT00000082138	0.959870098	7.66E-07
TUCP_002474	XLOC_104687	ENSRNOT00000000177	0.951551486	1.94E-06
TUCP_002474	XLOC_104687	ENSRNOT00000018328	0.968777212	2.22E-07
TUCP_002474	XLOC_104687	ENSRNOT00000064290	0.956068881	1.20E-06
TUCP_002474	XLOC_104687	ENSRNOT00000001099	0.957944908	9.65E-07
TUCP_002474	XLOC_104687	ENSRNOT00000012657	0.951738188	1.90E-06
TUCP_002474	XLOC_104687	ENSRNOT00000050119	0.976784316	5.11E-08
TUCP_002474	XLOC_104687	ENSRNOT00000007790	0.960274437	7.29E-07
TUCP_002474	XLOC_104687	ENSRNOT00000083843	0.968702304	2.24E-07
TUCP_002474	XLOC_104687	ENSRNOT00000021548	0.950016201	2.26E-06
TUCP_002474	XLOC_104687	ENSRNOT00000083421	0.958949041	8.57E-07
TUCP_002474	XLOC_104687	ENSRNOT00000021716	-0.967980142	2.51E-07
TUCP_002474	XLOC_104687	ENSRNOT00000055888	0.95669634	1.11E-06
TUCP_002474	XLOC_104687	ENSRNOT00000082429	0.958024672	9.56E-07
TUCP_002474	XLOC_104687	ENSRNOT00000018864	0.956948526	1.08E-06
TUCP_002474	XLOC_104687	ENSRNOT00000036838	0.981024464	1.88E-08
TUCP_002474	XLOC_104687	ENSRNOT00000010079	0.955877052	1.22E-06
TUCP_002474	XLOC_104687	ENSRNOT00000031261	-0.951165548	2.01E-06
TUCP_002474	XLOC_104687	ENSRNOT00000067836	0.964929	3.94E-07

TUCP_002474	XLOC_104687	ENSRNOT00000019816	0.964048475	4.45E-07
TUCP_002474	XLOC_104687	ENSRNOT00000022117	0.964722129	4.06E-07
TUCP_002474	XLOC_104687	ENSRNOT00000073847	0.964934687	3.94E-07
TUCP_002474	XLOC_104687	ENSRNOT00000015518	0.968014036	2.50E-07
TUCP_002474	XLOC_104687	ENSRNOT00000008126	0.952117921	1.83E-06
TUCP_002474	XLOC_104687	ENSRNOT00000064901	-0.9637995	4.61E-07
TUCP_002474	XLOC_104687	ENSRNOT00000068528	0.952343419	1.79E-06
TUCP_002474	XLOC_104687	ENSRNOT00000059487	0.98120898	1.79E-08
TUCP_002474	XLOC_104687	ENSRNOT00000041106	-0.961017771	6.64E-07
TUCP_002474	XLOC_104687	ENSRNOT00000019713	0.987019822	2.84E-09
TUCP_002474	XLOC_104687	ENSRNOT00000037363	0.950683489	2.11E-06
TUCP_002474	XLOC_104687	ENSRNOT00000085214	0.964842505	3.99E-07
TUCP_002474	XLOC_104687	ENSRNOT00000083633	0.966639156	3.08E-07
TUCP_002474	XLOC_104687	ENSRNOT00000083632	0.958854101	8.67E-07
TUCP_002474	XLOC_104687	ENSRNOT00000000717	0.962674477	5.36E-07
TUCP_002474	XLOC_104687	ENSRNOT00000049048	0.958323549	9.23E-07
TUCP_002474	XLOC_104687	ENSRNOT00000017224	0.977572736	4.30E-08
TUCP_002474	XLOC_104687	ENSRNOT00000049725	0.960677886	6.93E-07
TUCP_002474	XLOC_104687	ENSRNOT00000089062	-0.967808569	2.58E-07
TUCP_002474	XLOC_104687	ENSRNOT00000023872	0.966613741	3.09E-07
TUCP_002474	XLOC_104687	ENSRNOT00000010753	0.987098845	2.75E-09
TUCP_002474	XLOC_104687	ENSRNOT00000055281	0.958405342	9.14E-07
TUCP_002474	XLOC_104687	ENSRNOT00000023763	0.987109238	2.74E-09
TUCP_002474	XLOC_104687	ENSRNOT00000023641	0.961179684	6.50E-07
TUCP_002474	XLOC_104687	ENSRNOT00000009202	-0.969594319	1.94E-07
TUCP_002474	XLOC_104687	ENSRNOT00000028893	0.980239777	2.30E-08
TUCP_002474	XLOC_104687	ENSRNOT00000059181	-0.973142349	1.05E-07
TUCP_002474	XLOC_104687	ENSRNOT00000030798	0.979451997	2.79E-08
TUCP_002474	XLOC_104687	ENSRNOT00000007077	0.950996874	2.05E-06
TUCP_002474	XLOC_104687	ENSRNOT00000007079	0.96561481	3.57E-07
TUCP_002474	XLOC_104687	ENSRNOT00000014809	0.974445999	8.22E-08
TUCP_002474	XLOC_104687	ENSRNOT00000064849	0.954944389	1.36E-06
TUCP_002474	XLOC_104687	ENSRNOT00000004972	-0.9588942	8.63E-07
TUCP_002474	XLOC_104687	ENSRNOT00000073564	0.952408295	1.77E-06
TUCP_002474	XLOC_104687	ENSRNOT00000007276	0.957101147	1.06E-06
TUCP_002474	XLOC_104687	ENSRNOT00000008905	0.951025989	2.04E-06
TUCP_002474	XLOC_104687	ENSRNOT00000074081	0.958610244	8.92E-07
TUCP_002474	XLOC_104687	ENSRNOT00000023017	0.975581641	6.56E-08
TUCP_002474	XLOC_104687	ENSRNOT00000080608	-0.970402409	1.70E-07
TUCP_002474	XLOC_104687	ENSRNOT00000022065	0.969310901	2.04E-07
TUCP_002474	XLOC_104687	ENSRNOT00000017340	0.972288048	1.23E-07
TUCP_002474	XLOC_104687	ENSRNOT00000016740	-0.96274158	5.31E-07
TUCP_002474	XLOC_104687	ENSRNOT00000038212	0.978196494	3.74E-08
TUCP_002474	XLOC_104687	ENSRNOT00000085458	0.951716808	1.91E-06
TUCP_002474	XLOC_104687	ENSRNOT00000032893	0.951821811	1.88E-06
TUCP_002474	XLOC_104687	ENSRNOT00000080677	0.969129021	2.10E-07
TUCP_002474	XLOC_104687	ENSRNOT00000025460	-0.959694677	7.83E-07
TUCP_002474	XLOC_104687	ENSRNOT00000087033	0.962363143	5.58E-07
TUCP_002474	XLOC_104687	ENSRNOT00000025784	0.95411307	1.48E-06
TUCP_002474	XLOC_104687	ENSRNOT00000025782	0.951749063	1.90E-06
TUCP_002474	XLOC_104687	ENSRNOT00000026760	0.97101965	1.53E-07

TUCP_002474	XLOC_104687	ENSRNOT00000075800	0.973679001	9.52E-08
TUCP_002474	XLOC_104687	ENSRNOT00000024491	0.954054536	1.49E-06
TUCP_002474	XLOC_104687	ENSRNOT00000084597	0.956612427	1.13E-06
TUCP_002474	XLOC_104687	ENSRNOT00000063821	0.991443567	3.56E-10
TUCP_002474	XLOC_104687	ENSRNOT00000037588	0.950244878	2.21E-06
TUCP_002474	XLOC_104687	ENSRNOT00000027360	0.973675744	9.53E-08
TUCP_002474	XLOC_104687	ENSRNOT00000024947	0.962520054	5.47E-07
TUCP_002474	XLOC_104687	ENSRNOT00000041227	0.954425038	1.43E-06
TUCP_002474	XLOC_104687	ENSRNOT00000002355	0.972176065	1.25E-07
TUCP_002474	XLOC_104687	ENSRNOT00000024938	0.957301217	1.04E-06
TUCP_002474	XLOC_104687	ENSRNOT00000044254	0.961243413	6.45E-07
TUCP_002474	XLOC_104687	ENSRNOT00000044486	0.969648692	1.93E-07
TUCP_002474	XLOC_104687	ENSRNOT00000079414	0.954502885	1.42E-06
TUCP_002474	XLOC_104687	ENSRNOT00000079002	0.959334947	8.18E-07
TUCP_002474	XLOC_104687	ENSRNOT00000024011	-0.956840339	1.10E-06
TUCP_002474	XLOC_104687	ENSRNOT00000089673	0.967834811	2.57E-07
TUCP_002474	XLOC_104687	ENSRNOT00000000134	0.967923495	2.53E-07
TUCP_002474	XLOC_104687	ENSRNOT00000005885	0.961079631	6.59E-07
TUCP_002474	XLOC_104687	ENSRNOT00000011326	0.975867499	6.19E-08
TUCP_002474	XLOC_104687	ENSRNOT00000080285	0.953687765	1.55E-06
TUCP_002578	XLOC_109451	ENSRNOT00000082290	-0.955140895	1.33E-06
TUCP_002470	XLOC_104512	ENSRNOT00000074753	0.957963156	9.63E-07
TUCP_002470	XLOC_104512	ENSRNOT00000041904	0.966110957	3.33E-07
TUCP_002470	XLOC_104512	ENSRNOT00000080193	-0.951825755	1.88E-06
TUCP_002470	XLOC_104512	ENSRNOT00000081798	0.983790828	8.58E-09
TUCP_002470	XLOC_104512	ENSRNOT00000083487	0.950502442	2.15E-06
TUCP_002470	XLOC_104512	ENSRNOT00000077990	0.966110957	3.33E-07
TUCP_002470	XLOC_104512	ENSRNOT00000038826	0.96886269	2.19E-07
TUCP_002470	XLOC_104512	ENSRNOT00000081020	0.965984758	3.39E-07
TUCP_002470	XLOC_104512	ENSRNOT00000077014	0.955486876	1.28E-06
TUCP_002470	XLOC_104512	ENSRNOT00000060250	0.965516898	3.62E-07
TUCP_002470	XLOC_104512	ENSRNOT00000091934	0.966110957	3.33E-07
TUCP_002470	XLOC_104512	ENSRNOT00000080794	0.960922234	6.72E-07
TUCP_002470	XLOC_104512	ENSRNOT00000014456	0.990144553	7.20E-10
TUCP_002470	XLOC_104512	ENSRNOT00000031205	0.950882926	2.07E-06
TUCP_002470	XLOC_104512	ENSRNOT00000079260	0.96659934	3.10E-07
TUCP_002470	XLOC_104512	ENSRNOT00000044145	0.966110957	3.33E-07
TUCP_002470	XLOC_104512	ENSRNOT00000088381	0.950675025	2.12E-06
TUCP_002470	XLOC_104512	ENSRNOT00000011743	0.954193032	1.47E-06
TUCP_002470	XLOC_104512	ENSRNOT00000089929	0.966110957	3.33E-07
TUCP_002470	XLOC_104512	ENSRNOT00000088624	0.966110957	3.33E-07
TUCP_002470	XLOC_104512	ENSRNOT00000085659	0.954105058	1.48E-06
TUCP_002470	XLOC_104512	ENSRNOT00000015866	0.961412993	6.31E-07
TUCP_002470	XLOC_104512	ENSRNOT00000034907	0.973259885	1.03E-07
TUCP_002470	XLOC_104512	ENSRNOT00000051835	0.966110957	3.33E-07
TUCP_002470	XLOC_104512	ENSRNOT00000079087	0.966110957	3.33E-07
TUCP_002470	XLOC_104512	ENSRNOT00000037206	0.966110957	3.33E-07
TUCP_002470	XLOC_104512	ENSRNOT00000015353	0.972239617	1.24E-07
TUCP_002470	XLOC_104512	ENSRNOT00000080590	0.96531784	3.73E-07
TUCP_002470	XLOC_104512	ENSRNOT00000027552	0.966137687	3.31E-07
TUCP_002470	XLOC_104512	ENSRNOT00000047694	0.9701847	1.76E-07

TUCP_002470	XLOC_104512	ENSRNOT00000018923	0.961722326	6.07E-07
TUCP_002470	XLOC_104512	ENSRNOT00000080453	0.964874389	3.97E-07
TUCP_002470	XLOC_104512	ENSRNOT00000066839	0.976622648	5.29E-08
TUCP_002470	XLOC_104512	ENSRNOT00000078633	0.966110957	3.33E-07
TUCP_002470	XLOC_104512	ENSRNOT00000004128	0.953202588	1.63E-06
TUCP_002470	XLOC_104512	ENSRNOT00000026316	0.971583954	1.39E-07
TUCP_002470	XLOC_104512	ENSRNOT00000057729	0.96389863	4.55E-07
TUCP_002470	XLOC_104512	ENSRNOT00000021243	0.950524947	2.15E-06
TUCP_002470	XLOC_104512	ENSRNOT00000029634	0.963279588	4.94E-07
TUCP_002470	XLOC_104512	ENSRNOT00000065460	0.966110957	3.33E-07
TUCP_002470	XLOC_104512	ENSRNOT00000003817	0.969066146	2.12E-07
TUCP_002470	XLOC_104512	ENSRNOT00000016713	0.966110957	3.33E-07
TUCP_002470	XLOC_104512	ENSRNOT00000041074	0.993457827	9.34E-11
TUCP_002470	XLOC_104512	ENSRNOT00000039294	0.966110957	3.33E-07
TUCP_002470	XLOC_104512	ENSRNOT00000092913	0.968429205	2.34E-07
TUCP_002804	XLOC_118473	ENSRNOT00000080988	0.956428625	1.15E-06
TUCP_002804	XLOC_118473	ENSRNOT00000027407	0.957497774	1.02E-06
TUCP_002804	XLOC_118473	ENSRNOT00000011230	0.971033389	1.53E-07
TUCP_002804	XLOC_118473	ENSRNOT00000027083	0.951005012	2.05E-06
TUCP_002804	XLOC_118473	ENSRNOT00000048914	0.95055779	2.14E-06
TUCP_002804	XLOC_118473	ENSRNOT00000079633	-0.964425436	4.23E-07
TUCP_002804	XLOC_118473	ENSRNOT00000074939	0.969102777	2.11E-07
TUCP_002804	XLOC_118473	ENSRNOT00000066041	-0.953229084	1.63E-06
TUCP_002804	XLOC_118473	ENSRNOT00000023326	0.974124937	8.75E-08
TUCP_002804	XLOC_118473	ENSRNOT00000026065	0.950857449	2.08E-06
TUCP_002804	XLOC_118473	ENSRNOT00000012056	0.968698629	2.25E-07
TUCP_002804	XLOC_118473	ENSRNOT00000040650	0.953998406	1.50E-06
TUCP_002804	XLOC_118473	ENSRNOT00000005228	0.95787177	9.74E-07
TUCP_002804	XLOC_118473	ENSRNOT00000007164	0.969824195	1.87E-07
TUCP_002804	XLOC_118473	ENSRNOT00000019502	0.954834681	1.37E-06
TUCP_002804	XLOC_118473	ENSRNOT00000028856	0.955388674	1.29E-06
TUCP_002804	XLOC_118473	ENSRNOT00000043535	0.985614985	4.74E-09
TUCP_002804	XLOC_118473	ENSRNOT00000056813	0.95454293	1.42E-06
TUCP_002804	XLOC_118473	ENSRNOT00000080654	0.963296714	4.93E-07
TUCP_002804	XLOC_118473	ENSRNOT00000044742	0.965701561	3.53E-07
TUCP_002804	XLOC_118473	ENSRNOT00000009497	0.954755025	1.38E-06
TUCP_002804	XLOC_118473	ENSRNOT00000007185	-0.950106042	2.24E-06
TUCP_002804	XLOC_118473	ENSRNOT00000068633	0.959475127	8.04E-07
TUCP_002804	XLOC_118473	ENSRNOT00000043554	0.976787653	5.10E-08
TUCP_002804	XLOC_118473	ENSRNOT00000012187	0.964075743	4.44E-07
TUCP_002804	XLOC_118473	ENSRNOT00000005942	0.951073665	2.03E-06
TUCP_002804	XLOC_118473	ENSRNOT00000057814	0.959910783	7.62E-07
TUCP_002804	XLOC_118473	ENSRNOT00000026789	0.977598328	4.28E-08
TUCP_002804	XLOC_118473	ENSRNOT00000085251	-0.951741847	1.90E-06
TUCP_002804	XLOC_118473	ENSRNOT00000006055	0.962852346	5.23E-07
TUCP_002804	XLOC_118473	ENSRNOT00000025550	-0.960271675	7.29E-07
TUCP_002804	XLOC_118473	ENSRNOT00000012977	0.95911192	8.40E-07
TUCP_002804	XLOC_118473	ENSRNOT00000011384	0.95737976	1.03E-06
TUCP_002804	XLOC_118473	ENSRNOT00000010418	0.950203474	2.22E-06
TUCP_002804	XLOC_118473	ENSRNOT00000038247	0.962270906	5.65E-07
TUCP_002804	XLOC_118473	ENSRNOT00000044628	0.97802124	3.89E-08

TUCP_002804	XLOC_118473	ENSRNOT00000011622	0.956664017	1.12E-06
TUCP_002804	XLOC_118473	ENSRNOT00000021582	0.974582431	8.01E-08
TUCP_002804	XLOC_118473	ENSRNOT00000022534	0.952169205	1.82E-06
TUCP_002804	XLOC_118473	ENSRNOT00000027944	0.953703455	1.55E-06
TUCP_002804	XLOC_118473	ENSRNOT00000016490	0.95646324	1.14E-06
TUCP_002804	XLOC_118473	ENSRNOT00000052075	0.959959649	7.58E-07
TUCP_002804	XLOC_118473	ENSRNOT00000010463	0.954320826	1.45E-06
TUCP_002804	XLOC_118473	ENSRNOT00000026718	0.978910549	3.17E-08
TUCP_002804	XLOC_118473	ENSRNOT00000000713	0.957161635	1.06E-06
TUCP_002804	XLOC_118473	ENSRNOT00000000689	0.95382643	1.53E-06
TUCP_002804	XLOC_118473	ENSRNOT00000017319	0.962824682	5.25E-07
TUCP_002804	XLOC_118473	ENSRNOT00000057807	-0.9651859	3.80E-07
TUCP_002804	XLOC_118473	ENSRNOT00000026231	0.966133026	3.31E-07
TUCP_002804	XLOC_118473	ENSRNOT00000005909	0.951065681	2.04E-06
TUCP_002804	XLOC_118473	ENSRNOT00000051294	0.957616748	1.00E-06
TUCP_002804	XLOC_118473	ENSRNOT00000009894	-0.965448886	3.66E-07
TUCP_002804	XLOC_118473	ENSRNOT00000067822	0.953906689	1.52E-06
TUCP_002804	XLOC_118473	ENSRNOT00000024060	0.959484165	8.03E-07
TUCP_002804	XLOC_118473	ENSRNOT00000008018	0.951147159	2.02E-06
TUCP_002804	XLOC_118473	ENSRNOT00000016384	-0.9626409	5.38E-07
TUCP_002804	XLOC_118473	ENSRNOT00000054955	-0.953098065	1.65E-06
TUCP_002804	XLOC_118473	ENSRNOT00000025362	-0.952911434	1.68E-06
TUCP_002804	XLOC_118473	ENSRNOT00000010541	0.950451307	2.16E-06
TUCP_002804	XLOC_118473	ENSRNOT00000017948	0.965269535	3.75E-07
TUCP_002804	XLOC_118473	ENSRNOT00000015119	-0.955683012	1.25E-06
TUCP_002804	XLOC_118473	ENSRNOT00000090918	0.950763516	2.10E-06
TUCP_002804	XLOC_118473	ENSRNOT00000023180	0.96044114	7.14E-07
TUCP_002804	XLOC_118473	ENSRNOT00000039852	0.95979462	7.73E-07
TUCP_002804	XLOC_118473	ENSRNOT00000015609	0.964617357	4.12E-07
TUCP_002804	XLOC_118473	ENSRNOT00000028070	0.985278432	5.31E-09
TUCP_002804	XLOC_118473	ENSRNOT00000034722	0.959572876	7.95E-07
TUCP_002804	XLOC_118473	ENSRNOT00000020437	0.958008862	9.58E-07
TUCP_002804	XLOC_118473	ENSRNOT00000027335	0.951220823	2.00E-06
TUCP_002804	XLOC_118473	ENSRNOT00000010898	0.960894739	6.74E-07
TUCP_002804	XLOC_118473	ENSRNOT00000073675	-0.959856922	7.67E-07
TUCP_002804	XLOC_118473	ENSRNOT00000023645	0.959104524	8.41E-07
TUCP_002804	XLOC_118473	ENSRNOT00000001242	0.955811448	1.23E-06
TUCP_002804	XLOC_118473	ENSRNOT00000023330	0.960616385	6.98E-07
TUCP_002804	XLOC_118473	ENSRNOT00000078589	-0.962104068	5.78E-07
TUCP_002804	XLOC_118473	ENSRNOT00000010873	-0.95637473	1.16E-06
TUCP_002804	XLOC_118473	ENSRNOT00000006549	-0.952177194	1.82E-06
TUCP_002804	XLOC_118473	ENSRNOT00000004698	0.968861035	2.19E-07
TUCP_002804	XLOC_118473	ENSRNOT00000082388	0.971369183	1.44E-07
TUCP_002804	XLOC_118473	ENSRNOT00000045083	0.958622505	8.91E-07
TUCP_002804	XLOC_118473	ENSRNOT00000014537	0.952891941	1.69E-06
TUCP_002804	XLOC_118473	ENSRNOT00000077555	0.977172752	4.70E-08
TUCP_002804	XLOC_118473	ENSRNOT00000046323	0.950363396	2.18E-06
TUCP_002804	XLOC_118473	ENSRNOT00000017021	0.961147784	6.53E-07
TUCP_002804	XLOC_118473	ENSRNOT00000021408	0.954525273	1.42E-06
TUCP_002804	XLOC_118473	ENSRNOT00000009440	-0.960965514	6.68E-07
TUCP_002804	XLOC_118473	ENSRNOT00000087429	0.953558503	1.57E-06

TUCP_002804	XLOC_118473	ENSRNOT00000009683	0.958697764	8.83E-07
TUCP_002804	XLOC_118473	ENSRNOT00000048490	-0.960332761	7.24E-07
TUCP_002804	XLOC_118473	ENSRNOT00000084088	0.955086856	1.33E-06
TUCP_002804	XLOC_118473	ENSRNOT00000089193	0.952400224	1.78E-06
TUCP_002804	XLOC_118473	ENSRNOT00000058116	-0.962929348	5.18E-07
TUCP_002804	XLOC_118473	ENSRNOT00000064900	0.965992572	3.38E-07
TUCP_002804	XLOC_118473	ENSRNOT00000002771	0.954500505	1.42E-06
TUCP_002804	XLOC_118473	ENSRNOT00000024322	0.961751492	6.05E-07
TUCP_002804	XLOC_118473	ENSRNOT00000074075	0.956169377	1.18E-06
TUCP_002804	XLOC_118473	ENSRNOT00000013301	-0.974219607	8.59E-08
TUCP_002804	XLOC_118473	ENSRNOT00000078390	-0.954377628	1.44E-06
TUCP_002804	XLOC_118473	ENSRNOT00000090170	0.979908267	2.49E-08
TUCP_002804	XLOC_118473	ENSRNOT00000027452	0.951225465	2.00E-06
TUCP_002804	XLOC_118473	ENSRNOT00000017181	-0.951563522	1.94E-06
TUCP_002804	XLOC_118473	ENSRNOT00000049538	0.95894898	8.57E-07
TUCP_002804	XLOC_118473	ENSRNOT00000070933	0.960426598	7.15E-07
TUCP_002804	XLOC_118473	ENSRNOT00000004274	-0.950926482	2.06E-06
TUCP_002804	XLOC_118473	ENSRNOT00000087725	0.985600879	4.76E-09
TUCP_002804	XLOC_118473	ENSRNOT00000046594	0.961223073	6.47E-07
TUCP_002804	XLOC_118473	ENSRNOT00000025936	-0.963539357	4.77E-07
TUCP_002804	XLOC_118473	ENSRNOT00000027125	-0.963285565	4.94E-07
TUCP_002804	XLOC_118473	ENSRNOT00000086329	0.95603305	1.20E-06
TUCP_002804	XLOC_118473	ENSRNOT00000036951	0.958104146	9.47E-07
TUCP_002804	XLOC_118473	ENSRNOT00000072416	-0.952282859	1.80E-06
TUCP_002804	XLOC_118473	ENSRNOT00000009691	0.96016359	7.39E-07
TUCP_002804	XLOC_118473	ENSRNOT00000002173	-0.959881101	7.65E-07
TUCP_002804	XLOC_118473	ENSRNOT00000090420	-0.960793728	6.83E-07
TUCP_002804	XLOC_118473	ENSRNOT00000026653	0.950138038	2.23E-06
TUCP_002804	XLOC_118473	ENSRNOT00000078929	0.978167868	3.77E-08
TUCP_002804	XLOC_118473	ENSRNOT00000043953	0.950069175	2.25E-06
TUCP_002804	XLOC_118473	ENSRNOT00000021061	-0.956696147	1.12E-06
TUCP_002804	XLOC_118473	ENSRNOT00000019554	0.952696748	1.72E-06
TUCP_002804	XLOC_118473	ENSRNOT00000092158	0.966595358	3.10E-07
TUCP_002804	XLOC_118473	ENSRNOT00000017422	-0.958223165	9.34E-07
TUCP_002804	XLOC_118473	ENSRNOT00000027666	0.963855874	4.57E-07
TUCP_002804	XLOC_118473	ENSRNOT00000051953	-0.950659926	2.12E-06
TUCP_002804	XLOC_118473	ENSRNOT00000064840	0.960185144	7.37E-07
TUCP_002804	XLOC_118473	ENSRNOT00000077894	-0.956000028	1.21E-06
TUCP_002804	XLOC_118473	ENSRNOT00000085596	0.961890633	5.94E-07
TUCP_002804	XLOC_118473	ENSRNOT00000090925	0.966880573	2.97E-07
TUCP_002804	XLOC_118473	ENSRNOT00000000728	-0.964638725	4.10E-07
TUCP_002804	XLOC_118473	ENSRNOT00000054980	0.965129863	3.83E-07
TUCP_002804	XLOC_118473	ENSRNOT00000074653	0.9703024	1.73E-07
TUCP_002804	XLOC_118473	ENSRNOT00000031230	0.956452846	1.15E-06
TUCP_002804	XLOC_118473	ENSRNOT00000041096	0.961821319	5.99E-07
TUCP_002804	XLOC_118473	ENSRNOT00000001923	0.963298977	4.93E-07
TUCP_002804	XLOC_118473	ENSRNOT00000080110	0.95014586	2.23E-06
TUCP_002804	XLOC_118473	ENSRNOT00000010218	0.968363142	2.37E-07
TUCP_002804	XLOC_118473	ENSRNOT00000066028	0.978893891	3.18E-08
TUCP_002804	XLOC_118473	ENSRNOT00000005478	-0.952623973	1.74E-06
TUCP_002804	XLOC_118473	ENSRNOT00000002292	0.970648235	1.63E-07

TUCP_002804	XLOC_118473	ENSRNOT00000086065	0.95237607	1.78E-06
TUCP_002804	XLOC_118473	ENSRNOT00000035491	0.967761398	2.60E-07
TUCP_002804	XLOC_118473	ENSRNOT00000086336	0.954815721	1.37E-06
TUCP_002804	XLOC_118473	ENSRNOT00000008427	-0.970160311	1.77E-07
TUCP_002804	XLOC_118473	ENSRNOT00000067181	0.962674076	5.36E-07
TUCP_002804	XLOC_118473	ENSRNOT00000034704	0.958437326	9.11E-07
TUCP_002804	XLOC_118473	ENSRNOT00000082174	0.967209401	2.83E-07
TUCP_002804	XLOC_118473	ENSRNOT00000010986	0.96834141	2.37E-07
TUCP_002804	XLOC_118473	ENSRNOT00000009900	-0.956412855	1.15E-06
TUCP_002804	XLOC_118473	ENSRNOT00000004412	0.96220041	5.70E-07
TUCP_002804	XLOC_118473	ENSRNOT00000066968	0.959734563	7.79E-07
TUCP_002804	XLOC_118473	ENSRNOT00000077465	0.974748644	7.75E-08
TUCP_002804	XLOC_118473	ENSRNOT00000026280	0.960569991	7.02E-07
TUCP_002804	XLOC_118473	ENSRNOT00000061100	0.959101317	8.41E-07
TUCP_002804	XLOC_118473	ENSRNOT00000084595	0.953510468	1.58E-06
TUCP_002804	XLOC_118473	ENSRNOT00000024941	0.954995543	1.35E-06
TUCP_002804	XLOC_118473	ENSRNOT00000022862	0.957505393	1.02E-06
TUCP_002804	XLOC_118473	ENSRNOT00000026457	0.954236072	1.46E-06
TUCP_002804	XLOC_118473	ENSRNOT00000023428	0.961041925	6.62E-07
TUCP_002804	XLOC_118473	ENSRNOT00000011041	0.953100147	1.65E-06
TUCP_002804	XLOC_118473	ENSRNOT00000021285	0.952033599	1.84E-06
TUCP_002804	XLOC_118473	ENSRNOT00000048029	0.951269003	1.99E-06
TUCP_002804	XLOC_118473	ENSRNOT00000010433	0.950998617	2.05E-06
TUCP_002804	XLOC_118473	ENSRNOT00000011682	-0.963721342	4.66E-07
TUCP_002804	XLOC_118473	ENSRNOT00000083799	0.959744513	7.78E-07
TUCP_002804	XLOC_118473	ENSRNOT00000024548	0.969772329	1.89E-07
TUCP_002804	XLOC_118473	ENSRNOT00000006499	0.953877385	1.52E-06
TUCP_002805	XLOC_118501	ENSRNOT00000079449	-0.959848107	7.68E-07
TUCP_002774	XLOC_117242	ENSRNOT00000000008	0.989114125	1.18E-09
TUCP_002774	XLOC_117242	ENSRNOT00000060048	0.986656697	3.26E-09
TUCP_002774	XLOC_117242	ENSRNOT00000082304	0.996003582	7.97E-12
TUCP_002774	XLOC_117242	ENSRNOT00000006219	0.989114125	1.18E-09
TUCP_002774	XLOC_117242	ENSRNOT00000065376	0.989114125	1.18E-09
TUCP_002774	XLOC_117242	ENSRNOT00000081433	0.995934073	8.69E-12
TUCP_002774	XLOC_117242	ENSRNOT00000083575	0.989114125	1.18E-09
TUCP_002774	XLOC_117242	ENSRNOT00000087739	0.989114125	1.18E-09
TUCP_002774	XLOC_117242	ENSRNOT00000079826	0.989114125	1.18E-09
TUCP_002774	XLOC_117242	ENSRNOT00000025770	0.979660248	2.65E-08
TUCP_002774	XLOC_117242	ENSRNOT00000088244	0.975909669	6.14E-08
TUCP_002774	XLOC_117242	ENSRNOT00000048523	0.989114125	1.18E-09
TUCP_002774	XLOC_117242	ENSRNOT00000020229	0.968803766	2.21E-07
TUCP_002774	XLOC_117242	ENSRNOT00000026428	0.986605112	3.32E-09
TUCP_002774	XLOC_117242	ENSRNOT00000085673	0.989114125	1.18E-09
TUCP_002774	XLOC_117242	ENSRNOT00000072972	0.959844999	7.69E-07
TUCP_002774	XLOC_117242	ENSRNOT00000050573	0.989114125	1.18E-09
TUCP_002774	XLOC_117242	ENSRNOT00000089876	-0.968347211	2.37E-07
TUCP_002774	XLOC_117242	ENSRNOT00000074378	0.986794797	3.09E-09
TUCP_002774	XLOC_117242	ENSRNOT00000093571	0.989114125	1.18E-09
TUCP_002774	XLOC_117242	ENSRNOT00000090581	0.989114125	1.18E-09
TUCP_002774	XLOC_117242	ENSRNOT00000076239	0.985479999	4.96E-09
TUCP_002774	XLOC_117242	ENSRNOT00000065485	0.989114125	1.18E-09

TUCP_002774	XLOC_117242	ENSRNOT00000006356	0.987585778	2.27E-09
TUCP_002774	XLOC_117242	ENSRNOT00000087199	0.963516193	4.79E-07
TUCP_002774	XLOC_117242	ENSRNOT00000022091	0.989114125	1.18E-09
TUCP_002774	XLOC_117242	ENSRNOT00000064764	0.97892993	3.16E-08
TUCP_002774	XLOC_117242	ENSRNOT00000076927	0.956827811	1.10E-06
TUCP_002774	XLOC_117242	ENSRNOT00000092395	0.98776511	2.12E-09
TUCP_002775	XLOC_117255	ENSRNOT00000036527	0.970101149	1.79E-07
TUCP_002775	XLOC_117255	ENSRNOT00000081047	0.951382669	1.97E-06
TUCP_002775	XLOC_117255	ENSRNOT00000017021	0.956730601	1.11E-06
TUCP_002775	XLOC_117255	ENSRNOT00000087592	0.960656866	6.95E-07
TUCP_002775	XLOC_117255	ENSRNOT00000080110	0.963257957	4.96E-07
TUCP_002775	XLOC_117255	ENSRNOT00000051991	0.951883237	1.87E-06
TUCP_002775	XLOC_117255	ENSRNOT00000008655	0.960811858	6.81E-07
TUCP_002775	XLOC_117255	ENSRNOT00000005228	0.964648115	4.10E-07
TUCP_002775	XLOC_117255	ENSRNOT00000087429	0.95993969	7.60E-07
TUCP_002775	XLOC_117255	ENSRNOT00000073235	0.95588474	1.22E-06
TUCP_002775	XLOC_117255	ENSRNOT00000024149	0.953999944	1.50E-06
TUCP_002775	XLOC_117255	ENSRNOT00000052224	0.957617083	1.00E-06
TUCP_002775	XLOC_117255	ENSRNOT00000033420	0.959320389	8.19E-07
TUCP_002775	XLOC_117255	ENSRNOT00000012895	0.956026095	1.20E-06
TUCP_002775	XLOC_117255	ENSRNOT00000026915	0.979856546	2.53E-08
TUCP_002775	XLOC_117255	ENSRNOT00000015116	0.959427761	8.09E-07
TUCP_002775	XLOC_117255	ENSRNOT00000027454	0.969703669	1.91E-07
TUCP_002775	XLOC_117255	ENSRNOT00000066670	0.954620992	1.40E-06
TUCP_002775	XLOC_117255	ENSRNOT00000012187	0.960924745	6.72E-07
TUCP_002775	XLOC_117255	ENSRNOT00000005942	0.956031273	1.20E-06
TUCP_002775	XLOC_117255	ENSRNOT00000017753	0.951626032	1.92E-06
TUCP_002775	XLOC_117255	ENSRNOT00000014261	0.95978116	7.75E-07
TUCP_002775	XLOC_117255	ENSRNOT00000085251	-0.951801955	1.89E-06
TUCP_002775	XLOC_117255	ENSRNOT00000022485	0.958657703	8.87E-07
TUCP_002775	XLOC_117255	ENSRNOT00000043535	0.957913215	9.69E-07
TUCP_002775	XLOC_117255	ENSRNOT00000028646	0.950688029	2.11E-06
TUCP_002775	XLOC_117255	ENSRNOT00000044628	0.950362719	2.18E-06
TUCP_002775	XLOC_117255	ENSRNOT00000010898	0.950249897	2.21E-06
TUCP_002775	XLOC_117255	ENSRNOT00000023428	0.955781104	1.24E-06
TUCP_002775	XLOC_117255	ENSRNOT00000021285	0.950747735	2.10E-06
TUCP_002800	XLOC_118219	ENSRNOT00000006246	0.979307956	2.89E-08
TUCP_002800	XLOC_118219	ENSRNOT00000027891	0.965819486	3.47E-07
TUCP_002800	XLOC_118219	ENSRNOT00000045937	0.956477319	1.14E-06
TUCP_002800	XLOC_118219	ENSRNOT00000080666	-0.951552932	1.94E-06
TUCP_002800	XLOC_118219	ENSRNOT00000066764	0.97826078	3.69E-08
TUCP_002800	XLOC_118219	ENSRNOT00000026677	0.966846952	2.98E-07
TUCP_002800	XLOC_118219	ENSRNOT00000075656	0.966133294	3.31E-07
TUCP_002800	XLOC_118219	ENSRNOT00000013745	0.951353224	1.98E-06
TUCP_002800	XLOC_118219	ENSRNOT00000035523	0.971670147	1.37E-07
TUCP_002800	XLOC_118219	ENSRNOT00000011907	0.990173764	7.10E-10
TUCP_002800	XLOC_118219	ENSRNOT00000082611	0.954173318	1.47E-06
TUCP_002800	XLOC_118219	ENSRNOT00000024959	0.969285819	2.04E-07
TUCP_002800	XLOC_118219	ENSRNOT00000083702	0.962877607	5.22E-07
TUCP_002800	XLOC_118219	ENSRNOT00000033719	0.954571219	1.41E-06
TUCP_002800	XLOC_118219	ENSRNOT00000015568	0.975399304	6.81E-08

TUCP_002800	XLOC_118219	ENSRNOT00000089228	-0.951270598	1.99E-06
TUCP_002800	XLOC_118219	ENSRNOT00000013342	0.955165884	1.32E-06
TUCP_002800	XLOC_118219	ENSRNOT00000003085	0.956760954	1.11E-06
TUCP_002800	XLOC_118219	ENSRNOT00000013058	-0.952183013	1.82E-06
TUCP_002800	XLOC_118219	ENSRNOT00000022633	0.983531564	9.28E-09
TUCP_002800	XLOC_118219	ENSRNOT00000060607	0.950896537	2.07E-06
TUCP_002800	XLOC_118219	ENSRNOT00000014163	0.955701463	1.25E-06
TUCP_002800	XLOC_118219	ENSRNOT00000079644	0.95387282	1.52E-06
TUCP_002800	XLOC_118219	ENSRNOT00000029620	0.97645012	5.48E-08
TUCP_002800	XLOC_118219	ENSRNOT00000038178	0.96398568	4.49E-07
TUCP_002800	XLOC_118219	ENSRNOT00000002530	-0.95420339	1.47E-06
TUCP_002800	XLOC_118219	ENSRNOT00000020267	-0.958690164	8.84E-07
TUCP_002800	XLOC_118219	ENSRNOT00000020265	0.976872605	5.01E-08
TUCP_002800	XLOC_118219	ENSRNOT00000019501	0.971638935	1.38E-07
TUCP_002800	XLOC_118219	ENSRNOT00000009173	-0.953244123	1.63E-06
TUCP_002800	XLOC_118219	ENSRNOT00000045233	0.962140927	5.75E-07
TUCP_002800	XLOC_118219	ENSRNOT00000018696	0.954159815	1.48E-06
TUCP_002800	XLOC_118219	ENSRNOT00000091050	0.963394081	4.87E-07
TUCP_002800	XLOC_118219	ENSRNOT00000001196	0.977329404	4.54E-08
TUCP_002800	XLOC_118219	ENSRNOT00000046126	0.959234932	8.28E-07
TUCP_002800	XLOC_118219	ENSRNOT00000023033	0.963740682	4.64E-07
TUCP_002800	XLOC_118219	ENSRNOT00000000334	0.960167944	7.39E-07
TUCP_002800	XLOC_118219	ENSRNOT00000076105	0.96739602	2.75E-07
TUCP_002800	XLOC_118219	ENSRNOT00000068683	0.980084223	2.39E-08
TUCP_002800	XLOC_118219	ENSRNOT00000010252	0.976755548	5.14E-08
TUCP_002800	XLOC_118219	ENSRNOT00000027949	0.961651143	6.12E-07
TUCP_002800	XLOC_118219	ENSRNOT00000067566	0.950271622	2.20E-06
TUCP_002800	XLOC_118219	ENSRNOT00000013043	0.979529555	2.74E-08
TUCP_002800	XLOC_118219	ENSRNOT00000034603	0.977585633	4.29E-08
TUCP_002800	XLOC_118219	ENSRNOT00000015986	0.964748742	4.04E-07
TUCP_002800	XLOC_118219	ENSRNOT00000030975	0.970980056	1.54E-07
TUCP_002800	XLOC_118219	ENSRNOT00000073569	0.954789606	1.38E-06
TUCP_002800	XLOC_118219	ENSRNOT00000050119	0.956402903	1.15E-06
TUCP_002800	XLOC_118219	ENSRNOT00000027301	0.973763784	9.37E-08
TUCP_002800	XLOC_118219	ENSRNOT00000051349	0.958234368	9.33E-07
TUCP_002800	XLOC_118219	ENSRNOT00000022648	0.980210635	2.31E-08
TUCP_002800	XLOC_118219	ENSRNOT00000006765	0.956147757	1.19E-06
TUCP_002800	XLOC_118219	ENSRNOT00000089309	0.984165833	7.63E-09
TUCP_002800	XLOC_118219	ENSRNOT00000009313	0.976092737	5.91E-08
TUCP_002800	XLOC_118219	ENSRNOT00000015247	0.955605494	1.26E-06
TUCP_002800	XLOC_118219	ENSRNOT00000030373	0.973754828	9.38E-08
TUCP_002800	XLOC_118219	ENSRNOT00000057045	0.952962838	1.68E-06
TUCP_002800	XLOC_118219	ENSRNOT00000012699	0.960898763	6.74E-07
TUCP_002800	XLOC_118219	ENSRNOT00000017375	0.965375998	3.70E-07
TUCP_002800	XLOC_118219	ENSRNOT00000009247	0.966344138	3.21E-07
TUCP_002800	XLOC_118219	ENSRNOT00000082427	0.985293405	5.29E-09
TUCP_002800	XLOC_118219	ENSRNOT00000029635	0.985165085	5.52E-09
TUCP_002800	XLOC_118219	ENSRNOT00000001036	0.973209373	1.04E-07
TUCP_002800	XLOC_118219	ENSRNOT00000024568	0.969555064	1.96E-07
TUCP_002800	XLOC_118219	ENSRNOT00000010620	0.983746917	8.69E-09
TUCP_002800	XLOC_118219	ENSRNOT00000011731	0.963748665	4.64E-07

TUCP_002800	XLOC_118219	ENSRNOT00000082116	0.957641531	1.00E-06
TUCP_002800	XLOC_118219	ENSRNOT00000084674	0.970664297	1.63E-07
TUCP_002800	XLOC_118219	ENSRNOT00000083876	0.970144571	1.78E-07
TUCP_002800	XLOC_118219	ENSRNOT00000002936	0.965942772	3.41E-07
TUCP_002800	XLOC_118219	ENSRNOT00000032249	0.967924448	2.53E-07
TUCP_002800	XLOC_118219	ENSRNOT00000009319	0.967285365	2.79E-07
TUCP_002800	XLOC_118219	ENSRNOT00000003412	0.964950297	3.93E-07
TUCP_002800	XLOC_118219	ENSRNOT00000014684	0.972033276	1.29E-07
TUCP_002800	XLOC_118219	ENSRNOT00000007771	0.958695589	8.83E-07
TUCP_002800	XLOC_118219	ENSRNOT00000091863	0.962312603	5.62E-07
TUCP_002800	XLOC_118219	ENSRNOT00000081530	-0.968349139	2.37E-07
TUCP_002800	XLOC_118219	ENSRNOT00000090303	0.977388274	4.48E-08
TUCP_002800	XLOC_118219	ENSRNOT00000007358	0.966446257	3.17E-07
TUCP_002800	XLOC_118219	ENSRNOT00000022000	0.952289299	1.80E-06
TUCP_002800	XLOC_118219	ENSRNOT00000015618	0.968110309	2.46E-07
TUCP_002800	XLOC_118219	ENSRNOT00000040629	-0.952271136	1.80E-06
TUCP_002800	XLOC_118219	ENSRNOT00000000049	0.959678564	7.84E-07
TUCP_002800	XLOC_118219	ENSRNOT00000074286	-0.954158049	1.48E-06
TUCP_002800	XLOC_118219	ENSRNOT00000075934	0.950421119	2.17E-06
TUCP_002800	XLOC_118219	ENSRNOT00000046888	0.966037412	3.36E-07
TUCP_002800	XLOC_118219	ENSRNOT00000006111	0.975922551	6.12E-08
TUCP_002800	XLOC_118219	ENSRNOT00000015576	0.983877974	8.35E-09
TUCP_002800	XLOC_118219	ENSRNOT00000015579	0.975922102	6.12E-08
TUCP_002800	XLOC_118219	ENSRNOT00000037339	0.962018877	5.84E-07
TUCP_002800	XLOC_118219	ENSRNOT00000019680	0.961465068	6.27E-07
TUCP_002800	XLOC_118219	ENSRNOT00000055062	0.971934005	1.31E-07
TUCP_002800	XLOC_118219	ENSRNOT00000005644	0.970032731	1.81E-07
TUCP_002800	XLOC_118219	ENSRNOT00000047634	0.98273892	1.17E-08
TUCP_002800	XLOC_118219	ENSRNOT00000054897	-0.972495984	1.18E-07
TUCP_002800	XLOC_118219	ENSRNOT00000078816	-0.954652947	1.40E-06
TUCP_002800	XLOC_118219	ENSRNOT00000025197	0.991994168	2.56E-10
TUCP_002800	XLOC_118219	ENSRNOT00000083964	0.950135774	2.23E-06
TUCP_002800	XLOC_118219	ENSRNOT00000012646	0.965104248	3.84E-07
TUCP_002800	XLOC_118219	ENSRNOT00000001759	0.976317582	5.64E-08
TUCP_002800	XLOC_118219	ENSRNOT00000001228	0.956935372	1.08E-06
TUCP_002800	XLOC_118219	ENSRNOT00000091866	0.964524735	4.17E-07
TUCP_002800	XLOC_118219	ENSRNOT00000081327	0.981483138	1.66E-08
TUCP_002800	XLOC_118219	ENSRNOT00000032363	-0.954299447	1.45E-06
TUCP_002800	XLOC_118219	ENSRNOT00000083995	0.984896515	6.03E-09
TUCP_002800	XLOC_118219	ENSRNOT00000016447	0.965756544	3.50E-07
TUCP_002800	XLOC_118219	ENSRNOT00000033984	0.973826892	9.26E-08
TUCP_002800	XLOC_118219	ENSRNOT00000033048	0.952231578	1.81E-06
TUCP_002800	XLOC_118219	ENSRNOT00000018511	0.975747139	6.34E-08
TUCP_002800	XLOC_118219	ENSRNOT00000066888	0.965608696	3.58E-07
TUCP_002800	XLOC_118219	ENSRNOT00000007169	0.975787493	6.29E-08
TUCP_002800	XLOC_118219	ENSRNOT00000037844	0.951059525	2.04E-06
TUCP_002800	XLOC_118219	ENSRNOT00000007889	0.980627495	2.08E-08
TUCP_002800	XLOC_118219	ENSRNOT00000044983	0.954864997	1.37E-06
TUCP_002800	XLOC_118219	ENSRNOT00000084927	0.959505193	8.01E-07
TUCP_002800	XLOC_118219	ENSRNOT00000056166	-0.969623495	1.94E-07
TUCP_002800	XLOC_118219	ENSRNOT00000036585	-0.961498812	6.25E-07

TUCP_002800	XLOC_118219	ENSRNOT00000078746	0.984951863	5.93E-09
TUCP_002800	XLOC_118219	ENSRNOT00000091272	0.951977328	1.86E-06
TUCP_002800	XLOC_118219	ENSRNOT00000016730	0.977986142	3.92E-08
TUCP_002800	XLOC_118219	ENSRNOT00000065764	0.961766106	6.03E-07
TUCP_002800	XLOC_118219	ENSRNOT00000023856	0.973646813	9.58E-08
TUCP_002800	XLOC_118219	ENSRNOT00000010264	0.95003236	2.26E-06
TUCP_002800	XLOC_118219	ENSRNOT00000029819	0.967209244	2.83E-07
TUCP_002800	XLOC_118219	ENSRNOT00000092075	0.952162127	1.82E-06
TUCP_002800	XLOC_118219	ENSRNOT00000011371	0.959889326	7.64E-07
TUCP_002800	XLOC_118219	ENSRNOT00000071927	0.968474363	2.33E-07
TUCP_002800	XLOC_118219	ENSRNOT00000014824	0.97541703	6.78E-08
TUCP_002800	XLOC_118219	ENSRNOT00000018549	0.974433211	8.24E-08
TUCP_002800	XLOC_118219	ENSRNOT00000086541	0.976647071	5.26E-08
TUCP_002800	XLOC_118219	ENSRNOT00000019311	0.961995861	5.86E-07
TUCP_002800	XLOC_118219	ENSRNOT00000012657	0.95498103	1.35E-06
TUCP_002800	XLOC_118219	ENSRNOT00000021816	0.960035664	7.51E-07
TUCP_002800	XLOC_118219	ENSRNOT00000065103	0.952966222	1.67E-06
TUCP_002800	XLOC_118219	ENSRNOT00000024992	0.988659788	1.45E-09
TUCP_002800	XLOC_118219	ENSRNOT00000078644	-0.955934276	1.22E-06
TUCP_002800	XLOC_118219	ENSRNOT00000028067	0.968546082	2.30E-07
TUCP_002800	XLOC_118219	ENSRNOT00000026558	0.953735393	1.54E-06
TUCP_002800	XLOC_118219	ENSRNOT00000005694	0.950883881	2.07E-06
TUCP_002800	XLOC_118219	ENSRNOT00000004363	0.973951254	9.04E-08
TUCP_002800	XLOC_118219	ENSRNOT00000027212	0.970370447	1.71E-07
TUCP_002800	XLOC_118219	ENSRNOT00000013989	0.953101209	1.65E-06
TUCP_002800	XLOC_118219	ENSRNOT00000056995	0.957072887	1.07E-06
TUCP_002800	XLOC_118219	ENSRNOT00000083351	0.963435558	4.84E-07
TUCP_002800	XLOC_118219	ENSRNOT00000010079	0.950749163	2.10E-06
TUCP_002800	XLOC_118219	ENSRNOT00000030251	-0.958820963	8.70E-07
TUCP_002800	XLOC_118219	ENSRNOT00000023100	0.978463785	3.52E-08
TUCP_002800	XLOC_118219	ENSRNOT00000007949	0.986713793	3.19E-09
TUCP_002800	XLOC_118219	ENSRNOT00000000681	0.955888517	1.22E-06
TUCP_002800	XLOC_118219	ENSRNOT00000083814	0.969672449	1.92E-07
TUCP_002800	XLOC_118219	ENSRNOT00000021400	0.966435578	3.17E-07
TUCP_002800	XLOC_118219	ENSRNOT00000060595	0.967599987	2.66E-07
TUCP_002800	XLOC_118219	ENSRNOT00000024286	0.979879308	2.51E-08
TUCP_002800	XLOC_118219	ENSRNOT00000037397	0.951826033	1.88E-06
TUCP_002800	XLOC_118219	ENSRNOT00000055888	0.980880265	1.95E-08
TUCP_002800	XLOC_118219	ENSRNOT00000028181	0.958552529	8.98E-07
TUCP_002800	XLOC_118219	ENSRNOT00000091499	0.952533525	1.75E-06
TUCP_002800	XLOC_118219	ENSRNOT00000059487	0.956793182	1.10E-06
TUCP_002800	XLOC_118219	ENSRNOT00000039005	0.951116851	2.02E-06
TUCP_002800	XLOC_118219	ENSRNOT00000000263	-0.969216374	2.07E-07
TUCP_002800	XLOC_118219	ENSRNOT00000086350	0.965865788	3.45E-07
TUCP_002800	XLOC_118219	ENSRNOT00000010516	0.973336045	1.02E-07
TUCP_002800	XLOC_118219	ENSRNOT00000044975	0.989612187	9.36E-10
TUCP_002800	XLOC_118219	ENSRNOT00000045443	0.97541467	6.79E-08
TUCP_002800	XLOC_118219	ENSRNOT00000023506	0.973600219	9.66E-08
TUCP_002800	XLOC_118219	ENSRNOT00000015131	0.983434603	9.56E-09
TUCP_002800	XLOC_118219	ENSRNOT00000000529	0.969144802	2.09E-07
TUCP_002800	XLOC_118219	ENSRNOT00000044788	0.973560235	9.73E-08

TUCP_002800	XLOC_118219	ENSRNOT00000039730	0.96569545	3.53E-07
TUCP_002800	XLOC_118219	ENSRNOT00000030329	0.961854088	5.97E-07
TUCP_002800	XLOC_118219	ENSRNOT00000026057	0.956878269	1.09E-06
TUCP_002800	XLOC_118219	ENSRNOT00000084021	0.963675084	4.69E-07
TUCP_002800	XLOC_118219	ENSRNOT00000090026	-0.958809217	8.71E-07
TUCP_002800	XLOC_118219	ENSRNOT00000003567	0.982984792	1.09E-08
TUCP_002800	XLOC_118219	ENSRNOT00000088201	0.989728948	8.85E-10
TUCP_002800	XLOC_118219	ENSRNOT00000049536	0.978154373	3.78E-08
TUCP_002800	XLOC_118219	ENSRNOT00000086459	0.958441419	9.10E-07
TUCP_002800	XLOC_118219	ENSRNOT00000045311	0.959343037	8.17E-07
TUCP_002800	XLOC_118219	ENSRNOT00000022478	0.956314839	1.16E-06
TUCP_002800	XLOC_118219	ENSRNOT00000014980	-0.952934458	1.68E-06
TUCP_002800	XLOC_118219	ENSRNOT00000023872	0.953716397	1.55E-06
TUCP_002800	XLOC_118219	ENSRNOT00000023078	-0.966789996	3.01E-07
TUCP_002800	XLOC_118219	ENSRNOT00000071133	0.974445859	8.22E-08
TUCP_002800	XLOC_118219	ENSRNOT00000015525	0.980781308	2.00E-08
TUCP_002800	XLOC_118219	ENSRNOT00000092857	0.988840567	1.34E-09
TUCP_002800	XLOC_118219	ENSRNOT00000008365	0.964775935	4.03E-07
TUCP_002800	XLOC_118219	ENSRNOT00000018061	0.976040964	5.97E-08
TUCP_002800	XLOC_118219	ENSRNOT00000089185	0.967492629	2.71E-07
TUCP_002800	XLOC_118219	ENSRNOT00000031951	0.976116758	5.88E-08
TUCP_002800	XLOC_118219	ENSRNOT00000020272	0.980764714	2.01E-08
TUCP_002800	XLOC_118219	ENSRNOT00000017420	0.988132365	1.82E-09
TUCP_002800	XLOC_118219	ENSRNOT00000017907	0.955166647	1.32E-06
TUCP_002800	XLOC_118219	ENSRNOT00000077894	0.95338643	1.60E-06
TUCP_002800	XLOC_118219	ENSRNOT00000015763	0.976911323	4.97E-08
TUCP_002800	XLOC_118219	ENSRNOT00000014809	0.978129978	3.80E-08
TUCP_002800	XLOC_118219	ENSRNOT00000031138	-0.958943529	8.57E-07
TUCP_002800	XLOC_118219	ENSRNOT00000019644	0.977969209	3.94E-08
TUCP_002800	XLOC_118219	ENSRNOT00000015320	0.965789426	3.48E-07
TUCP_002800	XLOC_118219	ENSRNOT00000000640	0.960961518	6.69E-07
TUCP_002800	XLOC_118219	ENSRNOT00000020475	0.985406004	5.09E-09
TUCP_002800	XLOC_118219	ENSRNOT00000016263	0.963622183	4.72E-07
TUCP_002800	XLOC_118219	ENSRNOT00000047011	0.962862707	5.23E-07
TUCP_002800	XLOC_118219	ENSRNOT00000039377	0.981939346	1.47E-08
TUCP_002800	XLOC_118219	ENSRNOT00000013933	0.971284943	1.47E-07
TUCP_002800	XLOC_118219	ENSRNOT00000006376	0.979372988	2.84E-08
TUCP_002800	XLOC_118219	ENSRNOT00000041091	0.971484775	1.42E-07
TUCP_002800	XLOC_118219	ENSRNOT00000018782	0.984721651	6.39E-09
TUCP_002800	XLOC_118219	ENSRNOT00000077479	0.950468974	2.16E-06
TUCP_002800	XLOC_118219	ENSRNOT00000077401	0.977844894	4.05E-08
TUCP_002800	XLOC_118219	ENSRNOT00000024464	0.96681812	3.00E-07
TUCP_002800	XLOC_118219	ENSRNOT00000003823	0.958571125	8.96E-07
TUCP_002800	XLOC_118219	ENSRNOT00000022373	0.972718051	1.14E-07
TUCP_002800	XLOC_118219	ENSRNOT00000057016	0.960297383	7.27E-07
TUCP_002800	XLOC_118219	ENSRNOT00000036838	0.966040169	3.36E-07
TUCP_002800	XLOC_118219	ENSRNOT00000061973	0.985822518	4.41E-09
TUCP_002800	XLOC_118219	ENSRNOT00000050223	0.97006155	1.80E-07
TUCP_002800	XLOC_118219	ENSRNOT00000068078	0.982082196	1.41E-08
TUCP_002800	XLOC_118219	ENSRNOT00000009993	0.965846399	3.46E-07
TUCP_002800	XLOC_118219	ENSRNOT00000026844	-0.957019305	1.07E-06

TUCP_002800	XLOC_118219	ENSRNOT00000064452	0.953985849	1.50E-06
TUCP_002800	XLOC_118219	ENSRNOT00000012625	0.956856984	1.09E-06
TUCP_002800	XLOC_118219	ENSRNOT00000000039	0.961059596	6.60E-07
TUCP_002800	XLOC_118219	ENSRNOT00000037181	0.97424969	8.54E-08
TUCP_002800	XLOC_118219	ENSRNOT00000081272	0.953704771	1.55E-06
TUCP_002800	XLOC_118219	ENSRNOT00000045735	-0.958911659	8.61E-07
TUCP_002800	XLOC_118219	ENSRNOT00000019768	-0.974799276	7.67E-08
TUCP_002800	XLOC_118219	ENSRNOT00000066618	0.972004565	1.29E-07
TUCP_002800	XLOC_118219	ENSRNOT00000041815	0.964960437	3.92E-07
TUCP_002800	XLOC_118219	ENSRNOT00000028130	0.962278469	5.65E-07
TUCP_002800	XLOC_118219	ENSRNOT00000068267	0.960766094	6.85E-07
TUCP_002800	XLOC_118219	ENSRNOT00000009840	0.960995508	6.66E-07
TUCP_002800	XLOC_118219	ENSRNOT00000084552	0.966021258	3.37E-07
TUCP_002800	XLOC_118219	ENSRNOT00000019916	0.959327693	8.19E-07
TUCP_002800	XLOC_118219	ENSRNOT00000019911	0.952352456	1.79E-06
TUCP_002800	XLOC_118219	ENSRNOT00000034305	-0.951840302	1.88E-06
TUCP_002800	XLOC_118219	ENSRNOT00000086390	0.98314524	1.04E-08
TUCP_002800	XLOC_118219	ENSRNOT00000039215	0.950983425	2.05E-06
TUCP_002800	XLOC_118219	ENSRNOT00000049193	0.961366721	6.35E-07
TUCP_002800	XLOC_118219	ENSRNOT00000002355	0.96398957	4.49E-07
TUCP_002800	XLOC_118219	ENSRNOT00000036753	0.96913312	2.10E-07
TUCP_002800	XLOC_118219	ENSRNOT00000044467	0.980750592	2.02E-08
TUCP_002800	XLOC_118219	ENSRNOT00000044254	0.963254832	4.96E-07
TUCP_002800	XLOC_118219	ENSRNOT00000036972	0.968880305	2.18E-07
TUCP_002800	XLOC_118219	ENSRNOT00000029443	0.994432772	4.17E-11
TUCP_002800	XLOC_118219	ENSRNOT00000045532	0.950173356	2.22E-06
TUCP_002800	XLOC_118219	ENSRNOT00000092211	0.950174987	2.22E-06
TUCP_002800	XLOC_118219	ENSRNOT00000015387	0.978175091	3.76E-08
TUCP_002800	XLOC_118219	ENSRNOT00000058611	0.95780866	9.81E-07
TUCP_002800	XLOC_118219	ENSRNOT00000032719	0.97637315	5.57E-08
TUCP_002800	XLOC_118219	ENSRNOT00000023252	0.963131012	5.04E-07
TUCP_002800	XLOC_118219	ENSRNOT00000090355	0.967140705	2.86E-07
TUCP_002800	XLOC_118219	ENSRNOT00000073239	0.957215089	1.05E-06
TUCP_002800	XLOC_118219	ENSRNOT00000005885	0.971744506	1.35E-07
TUCP_002800	XLOC_118219	ENSRNOT00000011326	0.957862218	9.75E-07
TUCP_002800	XLOC_118219	ENSRNOT00000028801	-0.957801815	9.82E-07
TUCP_002800	XLOC_118219	ENSRNOT00000087481	0.973611435	9.64E-08
TUCP_002777	XLOC_117262	ENSRNOT00000035672	0.968920405	2.17E-07
TUCP_000985	XLOC_036063	ENSRNOT00000004698	0.952456026	1.77E-06
TUCP_000985	XLOC_036063	ENSRNOT00000012093	0.958876118	8.64E-07
TUCP_000985	XLOC_036063	ENSRNOT00000012156	0.966635017	3.08E-07
TUCP_000985	XLOC_036063	ENSRNOT00000078445	-0.967066751	2.89E-07
TUCP_000985	XLOC_036063	ENSRNOT00000087592	0.977732251	4.15E-08
TUCP_000985	XLOC_036063	ENSRNOT00000055992	-0.956754686	1.11E-06
TUCP_000985	XLOC_036063	ENSRNOT00000051991	0.950250444	2.21E-06
TUCP_000985	XLOC_036063	ENSRNOT00000004524	0.955839533	1.23E-06
TUCP_000985	XLOC_036063	ENSRNOT00000087429	0.950245855	2.21E-06
TUCP_000985	XLOC_036063	ENSRNOT00000000184	-0.957104339	1.06E-06
TUCP_000985	XLOC_036063	ENSRNOT00000023144	0.951311519	1.99E-06
TUCP_000985	XLOC_036063	ENSRNOT00000080800	0.954438315	1.43E-06
TUCP_000985	XLOC_036063	ENSRNOT00000003296	0.957406425	1.03E-06

TUCP_000985	XLOC_036063	ENSRNOT00000015116	0.953928928	1.51E-06
TUCP_000985	XLOC_036063	ENSRNOT00000027454	0.962719273	5.33E-07
TUCP_000985	XLOC_036063	ENSRNOT00000000036	0.95291153	1.68E-06
TUCP_000985	XLOC_036063	ENSRNOT00000005228	0.951262494	2.00E-06
TUCP_000985	XLOC_036063	ENSRNOT00000014814	0.951057875	2.04E-06
TUCP_000985	XLOC_036063	ENSRNOT00000080207	0.960796601	6.83E-07
TUCP_000985	XLOC_036063	ENSRNOT00000061267	0.973953031	9.04E-08
TUCP_000985	XLOC_036063	ENSRNOT00000006055	0.965363242	3.70E-07
TUCP_000985	XLOC_036063	ENSRNOT00000005126	-0.969556746	1.96E-07
TUCP_000985	XLOC_036063	ENSRNOT00000016325	-0.952674435	1.73E-06
TUCP_000985	XLOC_036063	ENSRNOT00000009280	0.953654538	1.56E-06
TUCP_000985	XLOC_036063	ENSRNOT00000026457	0.962106322	5.77E-07
TUCP_000985	XLOC_036063	ENSRNOT00000051550	0.961476786	6.26E-07
TUCP_000985	XLOC_036063	ENSRNOT00000088465	0.952880101	1.69E-06
TUCP_000985	XLOC_036063	ENSRNOT00000023858	0.972685131	1.14E-07
TUCP_000985	XLOC_036063	ENSRNOT00000000337	0.976609903	5.30E-08
TUCP_003134	XLOC_134195	ENSRNOT00000045301	0.9511389	2.02E-06
TUCP_003134	XLOC_134195	ENSRNOT00000041186	0.951720075	1.90E-06
TUCP_003134	XLOC_134195	ENSRNOT00000067379	0.957422685	1.03E-06
TUCP_003134	XLOC_134195	ENSRNOT00000065448	0.954429131	1.43E-06
TUCP_003134	XLOC_134195	ENSRNOT00000026803	0.964550553	4.15E-07
TUCP_003134	XLOC_134195	ENSRNOT00000076182	0.9511389	2.02E-06
TUCP_003134	XLOC_134195	ENSRNOT00000088893	0.9511389	2.02E-06
TUCP_003134	XLOC_134195	ENSRNOT00000020786	0.954928017	1.36E-06
TUCP_003134	XLOC_134195	ENSRNOT00000057472	0.9511389	2.02E-06
TUCP_003134	XLOC_134195	ENSRNOT00000081014	0.9511389	2.02E-06
TUCP_003134	XLOC_134195	ENSRNOT00000050543	0.954883456	1.36E-06
TUCP_003134	XLOC_134195	ENSRNOT00000079275	0.950728796	2.10E-06
TUCP_003134	XLOC_134195	ENSRNOT00000088078	0.951296879	1.99E-06
TUCP_003134	XLOC_134195	ENSRNOT00000090658	0.9511389	2.02E-06
TUCP_003134	XLOC_134195	ENSRNOT00000050968	0.952499494	1.76E-06
TUCP_003134	XLOC_134195	ENSRNOT00000084034	-0.963051113	5.10E-07
TUCP_003134	XLOC_134195	ENSRNOT00000014103	0.9511389	2.02E-06
TUCP_003134	XLOC_134195	ENSRNOT00000042201	0.962648304	5.38E-07
TUCP_003134	XLOC_134195	ENSRNOT00000067543	0.9511389	2.02E-06
TUCP_003134	XLOC_134195	ENSRNOT00000063929	0.9511389	2.02E-06
TUCP_003134	XLOC_134195	ENSRNOT00000068416	0.951048211	2.04E-06
TUCP_000987	XLOC_036069	ENSRNOT00000085158	0.966917077	2.95E-07
TUCP_000987	XLOC_036069	ENSRNOT00000006175	0.955675531	1.25E-06
TUCP_000987	XLOC_036069	ENSRNOT00000008221	0.987065296	2.79E-09
TUCP_000987	XLOC_036069	ENSRNOT00000027454	0.964102828	4.42E-07
TUCP_000987	XLOC_036069	ENSRNOT00000023144	0.954476486	1.43E-06
TUCP_000987	XLOC_036069	ENSRNOT00000047093	0.969740232	1.90E-07
TUCP_000987	XLOC_036069	ENSRNOT00000081512	0.95108482	2.03E-06
TUCP_000987	XLOC_036069	ENSRNOT00000026293	0.95558175	1.26E-06
TUCP_000987	XLOC_036069	ENSRNOT00000006454	0.953855598	1.52E-06
TUCP_000987	XLOC_036069	ENSRNOT00000064946	0.95747011	1.02E-06
TUCP_000987	XLOC_036069	ENSRNOT00000012156	0.958992005	8.52E-07
TUCP_000987	XLOC_036069	ENSRNOT00000016975	0.95481786	1.37E-06
TUCP_000987	XLOC_036069	ENSRNOT00000087592	0.955535206	1.27E-06
TUCP_000986	XLOC_036067	ENSRNOT00000051991	0.954902479	1.36E-06

TUCP_000986	XLOC_036067	ENSRNOT00000027454	0.958093803	9.49E-07
TUCP_000986	XLOC_036067	ENSRNOT00000023858	0.950876739	2.07E-06
TUCP_000986	XLOC_036067	ENSRNOT00000061267	0.950429908	2.17E-06
TUCP_000986	XLOC_036067	ENSRNOT00000046229	0.954432401	1.43E-06
TUCP_000986	XLOC_036067	ENSRNOT00000012156	0.959818693	7.71E-07
TUCP_000986	XLOC_036067	ENSRNOT00000087592	0.967080736	2.88E-07
TUCP_000986	XLOC_036067	ENSRNOT00000000337	0.961825134	5.99E-07
TUCP_000980	XLOC_035788	ENSRNOT00000056200	0.964951402	3.93E-07
TUCP_000983	XLOC_035791	ENSRNOT00000038580	0.966795573	3.01E-07
TUCP_000983	XLOC_035791	ENSRNOT00000072795	0.956514699	1.14E-06
TUCP_000983	XLOC_035791	ENSRNOT00000010601	0.9553012	1.30E-06
TUCP_002809	XLOC_118745	ENSRNOT00000001912	0.963359933	4.89E-07
TUCP_001163	XLOC_044017	ENSRNOT00000021694	0.955742397	1.24E-06
TUCP_001163	XLOC_044017	ENSRNOT00000081314	-0.95922118	8.29E-07
TUCP_001163	XLOC_044017	ENSRNOT00000026550	0.979346534	2.86E-08
TUCP_001163	XLOC_044017	ENSRNOT00000006460	0.962082009	5.79E-07
TUCP_001163	XLOC_044017	ENSRNOT00000087329	-0.951886752	1.87E-06
TUCP_001163	XLOC_044017	ENSRNOT00000077578	-0.961621272	6.15E-07
TUCP_001163	XLOC_044017	ENSRNOT00000020472	-0.960758954	6.86E-07
TUCP_001163	XLOC_044017	ENSRNOT00000065897	0.959638041	7.88E-07
TUCP_001163	XLOC_044017	ENSRNOT00000081226	0.951928336	1.86E-06
TUCP_001163	XLOC_044017	ENSRNOT00000073493	0.951859245	1.88E-06
TUCP_001163	XLOC_044017	ENSRNOT00000071897	-0.953570453	1.57E-06
TUCP_001163	XLOC_044017	ENSRNOT00000020446	0.9615857	6.18E-07
TUCP_001163	XLOC_044017	ENSRNOT00000056199	-0.954835684	1.37E-06
TUCP_001163	XLOC_044017	ENSRNOT00000029701	0.963327361	4.91E-07
TUCP_001163	XLOC_044017	ENSRNOT00000072716	0.959663166	7.86E-07
TUCP_001163	XLOC_044017	ENSRNOT00000038449	0.962449805	5.52E-07
TUCP_001163	XLOC_044017	ENSRNOT00000078752	-0.957982961	9.61E-07
TUCP_001163	XLOC_044017	ENSRNOT00000033806	0.962158042	5.73E-07
TUCP_001163	XLOC_044017	ENSRNOT00000007963	-0.961663591	6.11E-07
TUCP_001163	XLOC_044017	ENSRNOT00000016939	-0.960493823	7.09E-07
TUCP_001163	XLOC_044017	ENSRNOT00000048781	0.952573837	1.74E-06
TUCP_001163	XLOC_044017	ENSRNOT00000075934	-0.954139876	1.48E-06
TUCP_001163	XLOC_044017	ENSRNOT00000087905	-0.958638144	8.89E-07
TUCP_001163	XLOC_044017	ENSRNOT00000005064	-0.96165914	6.12E-07
TUCP_001163	XLOC_044017	ENSRNOT00000085334	0.953978785	1.50E-06
TUCP_001163	XLOC_044017	ENSRNOT00000081604	0.951171221	2.01E-06
TUCP_001163	XLOC_044017	ENSRNOT00000035427	0.959846545	7.68E-07
TUCP_001163	XLOC_044017	ENSRNOT00000087604	0.964147116	4.39E-07
TUCP_001163	XLOC_044017	ENSRNOT00000059246	0.964885509	3.96E-07
TUCP_001163	XLOC_044017	ENSRNOT00000050902	-0.951109639	2.03E-06
TUCP_001163	XLOC_044017	ENSRNOT00000005770	0.959161175	8.35E-07
TUCP_001163	XLOC_044017	ENSRNOT00000012757	0.95387364	1.52E-06
TUCP_001163	XLOC_044017	ENSRNOT00000005873	-0.9656369	3.56E-07
TUCP_001163	XLOC_044017	ENSRNOT00000016265	-0.950949893	2.06E-06
TUCP_001163	XLOC_044017	ENSRNOT00000005620	-0.953549959	1.57E-06
TUCP_001163	XLOC_044017	ENSRNOT00000012247	0.95968735	7.84E-07
TUCP_001163	XLOC_044017	ENSRNOT00000036658	-0.963856565	4.57E-07
TUCP_001163	XLOC_044017	ENSRNOT00000089611	0.955240569	1.31E-06
TUCP_001163	XLOC_044017	ENSRNOT00000034282	0.954751871	1.38E-06

TUCP_001163	XLOC_044017	ENSRNOT00000075209	-0.952609913	1.74E-06
TUCP_001163	XLOC_044017	ENSRNOT00000012474	0.958292231	9.27E-07
TUCP_001163	XLOC_044017	ENSRNOT00000092757	0.957151501	1.06E-06
TUCP_001163	XLOC_044017	ENSRNOT00000009202	0.958077029	9.50E-07
TUCP_001812	XLOC_075160	ENSRNOT00000005048	0.954987016	1.35E-06
TUCP_001164	XLOC_044017	ENSRNOT00000092026	0.96620614	3.28E-07
TUCP_001164	XLOC_044017	ENSRNOT00000093075	1	1.33E-79
TUCP_001164	XLOC_044017	ENSRNOT00000058260	1	0
TUCP_001164	XLOC_044017	ENSRNOT00000088887	1	0
TUCP_001164	XLOC_044017	ENSRNOT00000084986	1	1.33E-79
TUCP_001164	XLOC_044017	ENSRNOT00000074346	0.9702256	1.75E-07
TUCP_001164	XLOC_044017	ENSRNOT00000073209	0.983711602	8.79E-09
TUCP_001164	XLOC_044017	ENSRNOT00000080155	0.973069673	1.07E-07
TUCP_001164	XLOC_044017	ENSRNOT00000072573	0.996213272	6.09E-12
TUCP_001164	XLOC_044017	ENSRNOT00000017828	0.996001583	7.99E-12
TUCP_001164	XLOC_044017	ENSRNOT00000080444	0.969625907	1.93E-07
TUCP_001164	XLOC_044017	ENSRNOT00000071312	1	4.25E-78
TUCP_001164	XLOC_044017	ENSRNOT00000088245	1	4.25E-78
TUCP_001164	XLOC_044017	ENSRNOT00000036565	0.969065335	2.12E-07
TUCP_001164	XLOC_044017	ENSRNOT00000058833	0.998673261	3.23E-14
TUCP_001164	XLOC_044017	ENSRNOT00000078052	1	1.33E-79
TUCP_001164	XLOC_044017	ENSRNOT00000078494	0.999927093	1.62E-20
TUCP_001164	XLOC_044017	ENSRNOT00000085653	1	0
TUCP_001164	XLOC_044017	ENSRNOT00000057950	0.969329371	2.03E-07
TUCP_001164	XLOC_044017	ENSRNOT00000081992	0.999320526	1.14E-15
TUCP_001164	XLOC_044017	ENSRNOT00000038553	0.955765808	1.24E-06
TUCP_001164	XLOC_044017	ENSRNOT00000084120	0.958115447	9.46E-07
TUCP_001164	XLOC_044017	ENSRNOT00000055406	1	0
TUCP_001164	XLOC_044017	ENSRNOT00000070817	0.998968079	9.20E-15
TUCP_001164	XLOC_044017	ENSRNOT00000026753	1	0
TUCP_001164	XLOC_044017	ENSRNOT00000080533	0.996682529	3.15E-12
TUCP_001164	XLOC_044017	ENSRNOT00000072774	0.979665261	2.65E-08
TUCP_001164	XLOC_044017	ENSRNOT00000086031	1	0
TUCP_001164	XLOC_044017	ENSRNOT00000014312	1	0
TUCP_001164	XLOC_044017	ENSRNOT00000042882	0.963218427	4.98E-07
TUCP_001164	XLOC_044017	ENSRNOT00000088012	0.956765806	1.11E-06
TUCP_001164	XLOC_044017	ENSRNOT00000051231	0.999242378	1.96E-15
TUCP_001164	XLOC_044017	ENSRNOT00000089407	0.992986194	1.32E-10
TUCP_001164	XLOC_044017	ENSRNOT00000055034	0.998331441	1.02E-13
TUCP_001164	XLOC_044017	ENSRNOT00000046837	0.97252559	1.18E-07
TUCP_003428	XLOC_148227	ENSRNOT00000009811	0.951860086	1.88E-06
TUCP_003428	XLOC_148227	ENSRNOT00000065168	0.957092903	1.07E-06
TUCP_003428	XLOC_148227	ENSRNOT00000005958	0.959484614	8.03E-07
TUCP_003428	XLOC_148227	ENSRNOT00000067554	0.976405	5.54E-08
TUCP_003428	XLOC_148227	ENSRNOT00000083214	0.962572108	5.43E-07
TUCP_003428	XLOC_148227	ENSRNOT00000010784	0.956229564	1.18E-06
TUCP_003428	XLOC_148227	ENSRNOT00000061253	0.956906479	1.09E-06
TUCP_003428	XLOC_148227	ENSRNOT00000080024	0.956118218	1.19E-06
TUCP_003428	XLOC_148227	ENSRNOT00000023133	0.978208149	3.73E-08
TUCP_003428	XLOC_148227	ENSRNOT00000023989	0.956429488	1.15E-06
TUCP_003428	XLOC_148227	ENSRNOT00000061392	0.951570022	1.93E-06

TUCP_003428	XLOC_148227	ENSRNOT00000080608	0.958857343	8.66E-07
TUCP_003428	XLOC_148227	ENSRNOT00000047264	0.95136565	1.97E-06
TUCP_003428	XLOC_148227	ENSRNOT00000027970	0.950836992	2.08E-06
TUCP_003428	XLOC_148227	ENSRNOT00000057361	0.965214262	3.78E-07
TUCP_003428	XLOC_148227	ENSRNOT00000017556	0.958853064	8.67E-07
TUCP_003428	XLOC_148227	ENSRNOT00000025194	0.955610423	1.26E-06
TUCP_003428	XLOC_148227	ENSRNOT00000011662	0.950292759	2.20E-06
TUCP_003428	XLOC_148227	ENSRNOT00000001893	0.954624109	1.40E-06
TUCP_003428	XLOC_148227	ENSRNOT00000033017	0.960003754	7.54E-07
TUCP_003428	XLOC_148227	ENSRNOT00000083220	0.952697642	1.72E-06
TUCP_003428	XLOC_148227	ENSRNOT00000013049	0.973191678	1.04E-07
TUCP_003428	XLOC_148227	ENSRNOT00000087583	0.950417543	2.17E-06
TUCP_003428	XLOC_148227	ENSRNOT00000074972	0.968337239	2.38E-07
TUCP_003428	XLOC_148227	ENSRNOT00000045285	0.969488223	1.98E-07
TUCP_003428	XLOC_148227	ENSRNOT00000090057	0.976863358	5.02E-08
TUCP_003428	XLOC_148227	ENSRNOT00000040476	0.950954187	2.06E-06
TUCP_003428	XLOC_148227	ENSRNOT00000015799	0.968731984	2.23E-07
TUCP_000295	XLOC_009318	ENSRNOT00000026657	0.970710813	1.62E-07
TUCP_000052	XLOC_001127	ENSRNOT00000027411	0.964193181	4.36E-07
TUCP_000052	XLOC_001127	ENSRNOT00000086575	0.959362758	8.15E-07
TUCP_000052	XLOC_001127	ENSRNOT00000091013	0.959054277	8.46E-07
TUCP_000052	XLOC_001127	ENSRNOT00000075558	0.953504746	1.58E-06
TUCP_002985	XLOC_128384	ENSRNOT00000014865	-0.954986591	1.35E-06
TUCP_002982	XLOC_128384	ENSRNOT00000024186	0.955825978	1.23E-06
TUCP_002982	XLOC_128384	ENSRNOT00000074753	0.972956104	1.09E-07
TUCP_002982	XLOC_128384	ENSRNOT00000041904	0.973495287	9.85E-08
TUCP_002982	XLOC_128384	ENSRNOT00000083487	0.962452498	5.52E-07
TUCP_002982	XLOC_128384	ENSRNOT00000077990	0.973495287	9.85E-08
TUCP_002982	XLOC_128384	ENSRNOT00000038826	0.97215897	1.26E-07
TUCP_002982	XLOC_128384	ENSRNOT00000046842	0.960372815	7.20E-07
TUCP_002982	XLOC_128384	ENSRNOT00000021243	0.981715716	1.56E-08
TUCP_002982	XLOC_128384	ENSRNOT00000060250	0.97423234	8.57E-08
TUCP_002982	XLOC_128384	ENSRNOT00000091934	0.973495287	9.85E-08
TUCP_002982	XLOC_128384	ENSRNOT00000046585	0.958020327	9.57E-07
TUCP_002982	XLOC_128384	ENSRNOT00000080794	0.981259719	1.76E-08
TUCP_002982	XLOC_128384	ENSRNOT00000014456	0.95257527	1.74E-06
TUCP_002982	XLOC_128384	ENSRNOT00000031205	0.963287721	4.94E-07
TUCP_002982	XLOC_128384	ENSRNOT00000044145	0.973495287	9.85E-08
TUCP_002982	XLOC_128384	ENSRNOT00000088381	0.965190702	3.80E-07
TUCP_002982	XLOC_128384	ENSRNOT00000011743	0.969535413	1.96E-07
TUCP_002982	XLOC_128384	ENSRNOT00000089929	0.973495287	9.85E-08
TUCP_002982	XLOC_128384	ENSRNOT00000088624	0.973495287	9.85E-08
TUCP_002982	XLOC_128384	ENSRNOT00000085659	0.972491034	1.18E-07
TUCP_002982	XLOC_128384	ENSRNOT00000015866	0.962993443	5.14E-07
TUCP_002982	XLOC_128384	ENSRNOT00000064613	0.956393946	1.15E-06
TUCP_002982	XLOC_128384	ENSRNOT00000078633	0.973495287	9.85E-08
TUCP_002982	XLOC_128384	ENSRNOT00000024575	0.960944221	6.70E-07
TUCP_002982	XLOC_128384	ENSRNOT00000034907	0.966075866	3.34E-07
TUCP_002982	XLOC_128384	ENSRNOT00000051835	0.973495287	9.85E-08
TUCP_002982	XLOC_128384	ENSRNOT00000009825	0.951366506	1.97E-06
TUCP_002982	XLOC_128384	ENSRNOT00000079087	0.973495287	9.85E-08

TUCP_002982	XLOC_128384	ENSRNOT00000037206	0.973495287	9.85E-08
TUCP_002982	XLOC_128384	ENSRNOT00000083261	0.979599376	2.69E-08
TUCP_002982	XLOC_128384	ENSRNOT00000080590	0.972519828	1.18E-07
TUCP_002982	XLOC_128384	ENSRNOT00000021608	0.968742424	2.23E-07
TUCP_002982	XLOC_128384	ENSRNOT00000072138	0.958256722	9.31E-07
TUCP_002982	XLOC_128384	ENSRNOT00000077014	0.975702935	6.40E-08
TUCP_002982	XLOC_128384	ENSRNOT00000080453	0.972702682	1.14E-07
TUCP_002982	XLOC_128384	ENSRNOT00000057729	0.969463507	1.99E-07
TUCP_002982	XLOC_128384	ENSRNOT00000061284	0.95815764	9.41E-07
TUCP_002982	XLOC_128384	ENSRNOT00000071884	0.955175138	1.32E-06
TUCP_002982	XLOC_128384	ENSRNOT00000066839	0.973326686	1.02E-07
TUCP_002982	XLOC_128384	ENSRNOT00000078663	0.979306467	2.89E-08
TUCP_002982	XLOC_128384	ENSRNOT00000064250	0.97047393	1.68E-07
TUCP_002982	XLOC_128384	ENSRNOT00000027552	0.973560423	9.73E-08
TUCP_002982	XLOC_128384	ENSRNOT00000029634	0.969047777	2.12E-07
TUCP_002982	XLOC_128384	ENSRNOT00000093579	0.950162462	2.23E-06
TUCP_002982	XLOC_128384	ENSRNOT00000065460	0.973495287	9.85E-08
TUCP_002982	XLOC_128384	ENSRNOT00000003817	0.973926585	9.08E-08
TUCP_002982	XLOC_128384	ENSRNOT00000016713	0.973495287	9.85E-08
TUCP_002982	XLOC_128384	ENSRNOT00000022559	0.965407019	3.68E-07
TUCP_002982	XLOC_128384	ENSRNOT00000079260	0.971734054	1.36E-07
TUCP_002982	XLOC_128384	ENSRNOT00000039294	0.973495287	9.85E-08
TUCP_002982	XLOC_128384	ENSRNOT00000026932	0.954562058	1.41E-06
TUCP_002982	XLOC_128384	ENSRNOT00000089934	0.953897801	1.52E-06
TUCP_002980	XLOC_128384	ENSRNOT00000085599	0.958991667	8.52E-07
TUCP_002980	XLOC_128384	ENSRNOT00000083624	0.981359808	1.72E-08
TUCP_002980	XLOC_128384	ENSRNOT00000085301	0.950798016	2.09E-06
TUCP_002980	XLOC_128384	ENSRNOT00000038729	0.959589578	7.93E-07
TUCP_002980	XLOC_128384	ENSRNOT00000086385	0.959589578	7.93E-07
TUCP_002980	XLOC_128384	ENSRNOT00000087613	0.959589578	7.93E-07
TUCP_002980	XLOC_128384	ENSRNOT00000093689	0.986774535	3.12E-09
TUCP_002980	XLOC_128384	ENSRNOT00000022441	0.965389033	3.69E-07
TUCP_002980	XLOC_128384	ENSRNOT00000023062	0.959589578	7.93E-07
TUCP_002980	XLOC_128384	ENSRNOT00000058834	0.959589578	7.93E-07
TUCP_002980	XLOC_128384	ENSRNOT00000079420	0.959589578	7.93E-07
TUCP_002980	XLOC_128384	ENSRNOT00000090761	0.959589578	7.93E-07
TUCP_002980	XLOC_128384	ENSRNOT00000055923	0.959589578	7.93E-07
TUCP_002980	XLOC_128384	ENSRNOT00000092416	0.961901829	5.93E-07
TUCP_002980	XLOC_128384	ENSRNOT00000071930	0.959589578	7.93E-07
TUCP_002980	XLOC_128384	ENSRNOT00000001996	0.963740009	4.64E-07
TUCP_002980	XLOC_128384	ENSRNOT00000076756	0.959589578	7.93E-07
TUCP_002980	XLOC_128384	ENSRNOT00000081527	0.957379409	1.03E-06
TUCP_002980	XLOC_128384	ENSRNOT00000091663	0.950084307	2.24E-06
TUCP_002980	XLOC_128384	ENSRNOT00000081780	0.959589578	7.93E-07
TUCP_002980	XLOC_128384	ENSRNOT00000089114	0.959589578	7.93E-07
TUCP_002980	XLOC_128384	ENSRNOT00000021609	0.955453125	1.28E-06
TUCP_002980	XLOC_128384	ENSRNOT00000073316	0.959589578	7.93E-07
TUCP_002980	XLOC_128384	ENSRNOT00000071663	0.959589578	7.93E-07
TUCP_002980	XLOC_128384	ENSRNOT00000042860	0.959589578	7.93E-07
TUCP_002980	XLOC_128384	ENSRNOT00000088421	0.959589578	7.93E-07
TUCP_002980	XLOC_128384	ENSRNOT00000038898	0.959589578	7.93E-07

TUCP_002980	XLOC_128384	ENSRNOT00000042777	0.959686865	7.84E-07
TUCP_002980	XLOC_128384	ENSRNOT00000010029	0.959589578	7.93E-07
TUCP_001979	XLOC_079977	ENSRNOT00000009919	0.954417919	1.44E-06
TUCP_002988	XLOC_128384	ENSRNOT00000002220	0.955870056	1.22E-06
TUCP_003350	XLOC_145072	ENSRNOT00000051363	0.953561452	1.57E-06
TUCP_003350	XLOC_145072	ENSRNOT00000023152	0.953737421	1.54E-06
TUCP_003353	XLOC_145172	ENSRNOT00000093579	0.954854208	1.37E-06
TUCP_003353	XLOC_145172	ENSRNOT00000040509	0.966799231	3.00E-07
TUCP_003353	XLOC_145172	ENSRNOT00000012170	0.990453681	6.14E-10
TUCP_003353	XLOC_145172	ENSRNOT00000061139	0.951285434	1.99E-06
TUCP_003352	XLOC_145171	ENSRNOT00000024186	0.983200502	1.02E-08
TUCP_003352	XLOC_145171	ENSRNOT00000074753	0.998182349	1.56E-13
TUCP_003352	XLOC_145171	ENSRNOT00000004876	0.959071443	8.44E-07
TUCP_003352	XLOC_145171	ENSRNOT00000041904	1	0
TUCP_003352	XLOC_145171	ENSRNOT00000083487	0.989515314	9.80E-10
TUCP_003352	XLOC_145171	ENSRNOT00000077990	1	0
TUCP_003352	XLOC_145171	ENSRNOT00000038826	0.99977672	4.37E-18
TUCP_003352	XLOC_145171	ENSRNOT00000046842	0.973644314	9.58E-08
TUCP_003352	XLOC_145171	ENSRNOT00000037169	0.980329406	2.24E-08
TUCP_003352	XLOC_145171	ENSRNOT00000021243	0.987425406	2.42E-09
TUCP_003352	XLOC_145171	ENSRNOT00000060250	0.999933853	9.97E-21
TUCP_003352	XLOC_145171	ENSRNOT00000091934	1	4.25E-78
TUCP_003352	XLOC_145171	ENSRNOT00000046585	0.982941597	1.11E-08
TUCP_003352	XLOC_145171	ENSRNOT00000080794	0.990839253	5.00E-10
TUCP_003352	XLOC_145171	ENSRNOT00000014456	0.991697153	3.06E-10
TUCP_003352	XLOC_145171	ENSRNOT00000031205	0.991394766	3.66E-10
TUCP_003352	XLOC_145171	ENSRNOT00000044145	1	1.33E-79
TUCP_003352	XLOC_145171	ENSRNOT00000088381	0.989013276	1.24E-09
TUCP_003352	XLOC_145171	ENSRNOT00000011743	0.98678427	3.11E-09
TUCP_003352	XLOC_145171	ENSRNOT00000089929	1	0
TUCP_003352	XLOC_145171	ENSRNOT00000088624	1	0
TUCP_003352	XLOC_145171	ENSRNOT00000085659	0.989908621	8.10E-10
TUCP_003352	XLOC_145171	ENSRNOT00000015866	0.994066664	5.73E-11
TUCP_003352	XLOC_145171	ENSRNOT00000078633	1	1.33E-79
TUCP_003352	XLOC_145171	ENSRNOT00000034907	0.995318542	1.76E-11
TUCP_003352	XLOC_145171	ENSRNOT00000093230	0.958295175	9.26E-07
TUCP_003352	XLOC_145171	ENSRNOT00000051835	1	0
TUCP_003352	XLOC_145171	ENSRNOT00000079087	1	0
TUCP_003352	XLOC_145171	ENSRNOT00000037206	1	0
TUCP_003352	XLOC_145171	ENSRNOT00000083261	0.970991658	1.54E-07
TUCP_003352	XLOC_145171	ENSRNOT00000080590	0.999889216	1.31E-19
TUCP_003352	XLOC_145171	ENSRNOT00000027552	0.999801024	2.46E-18
TUCP_003352	XLOC_145171	ENSRNOT00000077014	0.995074376	2.26E-11
TUCP_003352	XLOC_145171	ENSRNOT00000080453	0.99994332	4.61E-21
TUCP_003352	XLOC_145171	ENSRNOT00000089057	0.968243551	2.41E-07
TUCP_003352	XLOC_145171	ENSRNOT00000066839	0.991868612	2.76E-10
TUCP_003352	XLOC_145171	ENSRNOT00000061284	0.955566942	1.27E-06
TUCP_003352	XLOC_145171	ENSRNOT00000013423	0.959677621	7.84E-07
TUCP_003352	XLOC_145171	ENSRNOT00000071884	0.974769955	7.72E-08
TUCP_003352	XLOC_145171	ENSRNOT00000004128	0.961829362	5.99E-07
TUCP_003352	XLOC_145171	ENSRNOT00000026316	0.964408046	4.24E-07

TUCP_003352	XLOC_145171	ENSRNOT00000057729	0.999384184	6.97E-16
TUCP_003352	XLOC_145171	ENSRNOT00000078663	0.975192577	7.10E-08
TUCP_003352	XLOC_145171	ENSRNOT00000064250	0.983131977	1.05E-08
TUCP_003352	XLOC_145171	ENSRNOT00000021608	0.965228881	3.78E-07
TUCP_003352	XLOC_145171	ENSRNOT00000029634	0.995816134	1.00E-11
TUCP_003352	XLOC_145171	ENSRNOT00000093579	0.958564264	8.97E-07
TUCP_003352	XLOC_145171	ENSRNOT00000086349	0.957242511	1.05E-06
TUCP_003352	XLOC_145171	ENSRNOT00000065460	1	0
TUCP_003352	XLOC_145171	ENSRNOT00000003817	0.996778712	2.72E-12
TUCP_003352	XLOC_145171	ENSRNOT00000016713	1	1.33E-79
TUCP_003352	XLOC_145171	ENSRNOT00000079260	0.996448245	4.42E-12
TUCP_003352	XLOC_145171	ENSRNOT00000039294	1	0
TUCP_003352	XLOC_145171	ENSRNOT00000092913	0.952980228	1.67E-06
TUCP_003355	XLOC_145345	ENSRNOT00000055200	0.950106585	2.24E-06
TUCP_003354	XLOC_145345	ENSRNOT00000026641	0.955047718	1.34E-06
TUCP_004437	XLOC_195441	ENSRNOT00000009979	0.964951738	3.93E-07
TUCP_004437	XLOC_195441	ENSRNOT00000011230	0.950033818	2.25E-06
TUCP_004437	XLOC_195441	ENSRNOT00000009877	0.950533943	2.15E-06
TUCP_004437	XLOC_195441	ENSRNOT00000017668	0.979157326	2.99E-08
TUCP_004437	XLOC_195441	ENSRNOT00000078445	-0.950787316	2.09E-06
TUCP_004437	XLOC_195441	ENSRNOT00000023326	0.961329674	6.38E-07
TUCP_004437	XLOC_195441	ENSRNOT00000005228	0.964624521	4.11E-07
TUCP_004437	XLOC_195441	ENSRNOT00000027526	0.954808043	1.38E-06
TUCP_004437	XLOC_195441	ENSRNOT00000090342	0.95606975	1.20E-06
TUCP_004437	XLOC_195441	ENSRNOT00000060282	0.953963967	1.51E-06
TUCP_004437	XLOC_195441	ENSRNOT00000017968	0.954199756	1.47E-06
TUCP_004437	XLOC_195441	ENSRNOT00000091128	-0.958603596	8.93E-07
TUCP_004437	XLOC_195441	ENSRNOT00000027414	0.964792565	4.02E-07
TUCP_004437	XLOC_195441	ENSRNOT00000028563	0.952805712	1.70E-06
TUCP_004437	XLOC_195441	ENSRNOT00000006195	0.970041681	1.81E-07
TUCP_004437	XLOC_195441	ENSRNOT00000066670	0.968813249	2.20E-07
TUCP_004437	XLOC_195441	ENSRNOT00000012187	0.953281931	1.62E-06
TUCP_004437	XLOC_195441	ENSRNOT00000005942	0.962417101	5.54E-07
TUCP_004437	XLOC_195441	ENSRNOT00000012463	-0.976200397	5.78E-08
TUCP_004437	XLOC_195441	ENSRNOT00000026783	0.962181351	5.72E-07
TUCP_004437	XLOC_195441	ENSRNOT00000040731	0.957307701	1.04E-06
TUCP_004437	XLOC_195441	ENSRNOT00000012379	0.971309327	1.46E-07
TUCP_004437	XLOC_195441	ENSRNOT00000028646	0.952755346	1.71E-06
TUCP_004437	XLOC_195441	ENSRNOT00000011384	0.961437262	6.29E-07
TUCP_004437	XLOC_195441	ENSRNOT00000014701	0.955486731	1.28E-06
TUCP_004437	XLOC_195441	ENSRNOT00000027944	0.964077876	4.43E-07
TUCP_004437	XLOC_195441	ENSRNOT00000052075	0.966394007	3.19E-07
TUCP_004437	XLOC_195441	ENSRNOT00000067556	-0.954068865	1.49E-06
TUCP_004437	XLOC_195441	ENSRNOT00000055669	0.973182306	1.04E-07
TUCP_004437	XLOC_195441	ENSRNOT00000017319	0.969230058	2.06E-07
TUCP_004437	XLOC_195441	ENSRNOT00000072518	0.957972292	9.62E-07
TUCP_004437	XLOC_195441	ENSRNOT00000005911	0.973945353	9.05E-08
TUCP_004437	XLOC_195441	ENSRNOT00000021037	0.956717035	1.11E-06
TUCP_004437	XLOC_195441	ENSRNOT00000012895	0.950525968	2.15E-06
TUCP_004437	XLOC_195441	ENSRNOT00000003177	0.955068201	1.34E-06
TUCP_004437	XLOC_195441	ENSRNOT00000044562	0.953679778	1.55E-06

TUCP_004437	XLOC_195441	ENSRNOT00000015116	0.967003454	2.91E-07
TUCP_004437	XLOC_195441	ENSRNOT00000023180	0.966756615	3.02E-07
TUCP_004437	XLOC_195441	ENSRNOT00000016948	-0.976302114	5.66E-08
TUCP_004437	XLOC_195441	ENSRNOT00000015609	0.950135552	2.23E-06
TUCP_004437	XLOC_195441	ENSRNOT00000028070	0.951158526	2.02E-06
TUCP_004437	XLOC_195441	ENSRNOT00000016321	-0.959605551	7.91E-07
TUCP_004437	XLOC_195441	ENSRNOT00000008817	0.962267722	5.65E-07
TUCP_004437	XLOC_195441	ENSRNOT00000022610	0.962033471	5.83E-07
TUCP_004437	XLOC_195441	ENSRNOT00000020437	0.961685826	6.10E-07
TUCP_004437	XLOC_195441	ENSRNOT00000029833	0.960991431	6.66E-07
TUCP_004437	XLOC_195441	ENSRNOT00000025580	-0.954468016	1.43E-06
TUCP_004437	XLOC_195441	ENSRNOT00000052422	0.955405545	1.29E-06
TUCP_004437	XLOC_195441	ENSRNOT00000001242	0.962770562	5.29E-07
TUCP_004437	XLOC_195441	ENSRNOT00000078102	0.951022346	2.04E-06
TUCP_004437	XLOC_195441	ENSRNOT00000025983	0.970619954	1.64E-07
TUCP_004437	XLOC_195441	ENSRNOT00000021790	0.971849652	1.33E-07
TUCP_004437	XLOC_195441	ENSRNOT00000090451	-0.955222661	1.31E-06
TUCP_004437	XLOC_195441	ENSRNOT00000008929	0.970122557	1.78E-07
TUCP_004437	XLOC_195441	ENSRNOT00000016129	0.96737504	2.76E-07
TUCP_004437	XLOC_195441	ENSRNOT00000016289	0.965070836	3.86E-07
TUCP_004437	XLOC_195441	ENSRNOT00000021408	0.955869188	1.22E-06
TUCP_004437	XLOC_195441	ENSRNOT00000009440	-0.967809573	2.58E-07
TUCP_004437	XLOC_195441	ENSRNOT00000007379	0.964413432	4.23E-07
TUCP_004437	XLOC_195441	ENSRNOT00000065618	0.955373355	1.29E-06
TUCP_004437	XLOC_195441	ENSRNOT00000017753	0.962380837	5.57E-07
TUCP_004437	XLOC_195441	ENSRNOT00000051022	0.95183096	1.88E-06
TUCP_004437	XLOC_195441	ENSRNOT00000006499	0.96992828	1.84E-07
TUCP_004437	XLOC_195441	ENSRNOT00000005909	0.952987	1.67E-06
TUCP_004437	XLOC_195441	ENSRNOT00000019011	0.968267796	2.40E-07
TUCP_004437	XLOC_195441	ENSRNOT00000025936	-0.952980127	1.67E-06
TUCP_004437	XLOC_195441	ENSRNOT00000009129	0.964772852	4.03E-07
TUCP_004437	XLOC_195441	ENSRNOT00000036951	0.965306979	3.73E-07
TUCP_004437	XLOC_195441	ENSRNOT00000016519	0.955829276	1.23E-06
TUCP_004437	XLOC_195441	ENSRNOT00000026186	0.964234168	4.34E-07
TUCP_004437	XLOC_195441	ENSRNOT00000014988	0.973655818	9.56E-08
TUCP_004437	XLOC_195441	ENSRNOT00000022963	0.956057666	1.20E-06
TUCP_004437	XLOC_195441	ENSRNOT00000043953	0.963123857	5.05E-07
TUCP_004437	XLOC_195441	ENSRNOT00000058842	0.960758683	6.86E-07
TUCP_004437	XLOC_195441	ENSRNOT00000084734	0.953342879	1.61E-06
TUCP_004437	XLOC_195441	ENSRNOT00000035700	0.956314701	1.16E-06
TUCP_004437	XLOC_195441	ENSRNOT00000082672	0.969851307	1.86E-07
TUCP_004437	XLOC_195441	ENSRNOT00000064840	0.965590876	3.59E-07
TUCP_004437	XLOC_195441	ENSRNOT00000026755	0.980871952	1.95E-08
TUCP_004437	XLOC_195441	ENSRNOT00000054828	0.962341853	5.60E-07
TUCP_004437	XLOC_195441	ENSRNOT00000001682	0.953914005	1.52E-06
TUCP_004437	XLOC_195441	ENSRNOT00000077406	0.960243026	7.32E-07
TUCP_004437	XLOC_195441	ENSRNOT00000026227	0.971159242	1.50E-07
TUCP_004437	XLOC_195441	ENSRNOT00000008757	0.952487152	1.76E-06
TUCP_004437	XLOC_195441	ENSRNOT00000024149	0.971857946	1.33E-07
TUCP_004437	XLOC_195441	ENSRNOT00000035474	0.951210545	2.01E-06
TUCP_004437	XLOC_195441	ENSRNOT00000077311	0.95172471	1.90E-06

TUCP_004437	XLOC_195441	ENSRNOT00000086336	0.974623763	7.94E-08
TUCP_004437	XLOC_195441	ENSRNOT00000083369	0.964830896	3.99E-07
TUCP_004437	XLOC_195441	ENSRNOT00000076872	0.960773175	6.85E-07
TUCP_004437	XLOC_195441	ENSRNOT00000059744	0.95238537	1.78E-06
TUCP_004437	XLOC_195441	ENSRNOT00000010218	0.955604983	1.26E-06
TUCP_004437	XLOC_195441	ENSRNOT00000007768	0.982487656	1.26E-08
TUCP_004437	XLOC_195441	ENSRNOT00000087095	0.970674429	1.63E-07
TUCP_004437	XLOC_195441	ENSRNOT00000064798	0.963812532	4.60E-07
TUCP_004437	XLOC_195441	ENSRNOT00000019177	0.969668735	1.92E-07
TUCP_004437	XLOC_195441	ENSRNOT00000014261	0.954968385	1.35E-06
TUCP_004437	XLOC_195441	ENSRNOT00000022485	0.95904192	8.47E-07
TUCP_004437	XLOC_195441	ENSRNOT00000033235	0.963502401	4.80E-07
TUCP_004437	XLOC_195441	ENSRNOT00000091807	0.960956815	6.69E-07
TUCP_004437	XLOC_195441	ENSRNOT00000018533	0.957564174	1.01E-06
TUCP_004437	XLOC_195441	ENSRNOT00000011682	-0.951487048	1.95E-06
TUCP_001169	XLOC_044150	ENSRNOT00000074753	0.95422112	1.47E-06
TUCP_001169	XLOC_044150	ENSRNOT00000041904	0.960802577	6.82E-07
TUCP_001169	XLOC_044150	ENSRNOT00000077990	0.960802577	6.82E-07
TUCP_001169	XLOC_044150	ENSRNOT00000038826	0.958231671	9.33E-07
TUCP_001169	XLOC_044150	ENSRNOT00000021243	0.950413979	2.17E-06
TUCP_001169	XLOC_044150	ENSRNOT00000060250	0.961434838	6.30E-07
TUCP_001169	XLOC_044150	ENSRNOT00000091934	0.960802577	6.82E-07
TUCP_001169	XLOC_044150	ENSRNOT00000086027	0.96141872	6.31E-07
TUCP_001169	XLOC_044150	ENSRNOT00000046585	0.973485832	9.87E-08
TUCP_001169	XLOC_044150	ENSRNOT00000034907	0.955637858	1.26E-06
TUCP_001169	XLOC_044150	ENSRNOT00000031205	0.972925345	1.09E-07
TUCP_001169	XLOC_044150	ENSRNOT00000044145	0.960802577	6.82E-07
TUCP_001169	XLOC_044150	ENSRNOT00000088381	0.973303415	1.02E-07
TUCP_001169	XLOC_044150	ENSRNOT00000089929	0.960802577	6.82E-07
TUCP_001169	XLOC_044150	ENSRNOT00000088624	0.960802577	6.82E-07
TUCP_001169	XLOC_044150	ENSRNOT00000085659	0.967689322	2.63E-07
TUCP_001169	XLOC_044150	ENSRNOT00000015866	0.964219238	4.35E-07
TUCP_001169	XLOC_044150	ENSRNOT00000061284	0.968276993	2.40E-07
TUCP_001169	XLOC_044150	ENSRNOT00000024575	0.952640758	1.73E-06
TUCP_001169	XLOC_044150	ENSRNOT00000051835	0.960802577	6.82E-07
TUCP_001169	XLOC_044150	ENSRNOT00000079087	0.960802577	6.82E-07
TUCP_001169	XLOC_044150	ENSRNOT00000037206	0.960802577	6.82E-07
TUCP_001169	XLOC_044150	ENSRNOT00000080590	0.961066198	6.60E-07
TUCP_001169	XLOC_044150	ENSRNOT00000027552	0.961449096	6.29E-07
TUCP_001169	XLOC_044150	ENSRNOT00000077014	0.970135134	1.78E-07
TUCP_001169	XLOC_044150	ENSRNOT00000080453	0.960951971	6.70E-07
TUCP_001169	XLOC_044150	ENSRNOT00000057729	0.960658709	6.95E-07
TUCP_001169	XLOC_044150	ENSRNOT00000078633	0.960802577	6.82E-07
TUCP_001169	XLOC_044150	ENSRNOT00000071884	0.966257894	3.25E-07
TUCP_001169	XLOC_044150	ENSRNOT00000078663	0.969784672	1.89E-07
TUCP_001169	XLOC_044150	ENSRNOT00000064250	0.953236385	1.63E-06
TUCP_001169	XLOC_044150	ENSRNOT00000040509	0.958353911	9.20E-07
TUCP_001169	XLOC_044150	ENSRNOT00000029634	0.953748285	1.54E-06
TUCP_001169	XLOC_044150	ENSRNOT00000093579	0.976734665	5.16E-08
TUCP_001169	XLOC_044150	ENSRNOT00000065460	0.960802577	6.82E-07
TUCP_001169	XLOC_044150	ENSRNOT00000003817	0.959396723	8.12E-07

TUCP_001169	XLOC_044150	ENSRNOT00000016713	0.960802577	6.82E-07
TUCP_001169	XLOC_044150	ENSRNOT00000079260	0.955750434	1.24E-06
TUCP_001169	XLOC_044150	ENSRNOT00000039294	0.960802577	6.82E-07
TUCP_001169	XLOC_044150	ENSRNOT00000024337	0.960343705	7.23E-07
TUCP_004439	XLOC_195492	ENSRNOT00000082191	0.967744797	2.60E-07
TUCP_004439	XLOC_195492	ENSRNOT00000071706	0.959373956	8.14E-07
TUCP_004438	XLOC_195492	ENSRNOT00000006357	0.955309327	1.30E-06
TUCP_004438	XLOC_195492	ENSRNOT00000006860	0.970200681	1.76E-07
TUCP_004438	XLOC_195492	ENSRNOT00000082585	0.957548575	1.01E-06
TUCP_004438	XLOC_195492	ENSRNOT00000038439	0.964087713	4.43E-07
TUCP_004438	XLOC_195492	ENSRNOT00000056355	0.957478571	1.02E-06
TUCP_004438	XLOC_195492	ENSRNOT00000029435	0.956929503	1.09E-06
TUCP_004438	XLOC_195492	ENSRNOT00000047717	0.953926409	1.51E-06
TUCP_004438	XLOC_195492	ENSRNOT00000003515	0.952071891	1.84E-06
TUCP_004438	XLOC_195492	ENSRNOT00000047408	0.951628912	1.92E-06
TUCP_004438	XLOC_195492	ENSRNOT00000010168	0.96777687	2.59E-07
TUCP_004438	XLOC_195492	ENSRNOT00000012376	0.951019519	2.04E-06
TUCP_004438	XLOC_195492	ENSRNOT00000016419	0.955487095	1.28E-06
TUCP_004438	XLOC_195492	ENSRNOT00000068118	0.954259267	1.46E-06
TUCP_004438	XLOC_195492	ENSRNOT00000091456	0.964146266	4.39E-07
TUCP_004438	XLOC_195492	ENSRNOT00000019350	0.958957587	8.56E-07
TUCP_004438	XLOC_195492	ENSRNOT00000014182	0.962617528	5.40E-07
TUCP_004438	XLOC_195492	ENSRNOT00000029072	0.952303191	1.79E-06
TUCP_004438	XLOC_195492	ENSRNOT00000016464	0.95809738	9.48E-07
TUCP_004438	XLOC_195492	ENSRNOT00000026854	0.950894139	2.07E-06
TUCP_004438	XLOC_195492	ENSRNOT00000064116	0.955314082	1.30E-06
TUCP_001904	XLOC_078874	ENSRNOT00000026966	0.970322306	1.73E-07
TUCP_001904	XLOC_078874	ENSRNOT00000068416	0.999999782	3.88E-33
TUCP_001904	XLOC_078874	ENSRNOT00000086036	0.999958274	9.96E-22
TUCP_001904	XLOC_078874	ENSRNOT00000026803	0.985046075	5.74E-09
TUCP_001904	XLOC_078874	ENSRNOT00000047527	0.963071698	5.08E-07
TUCP_001904	XLOC_078874	ENSRNOT00000042251	0.999344295	9.53E-16
TUCP_001904	XLOC_078874	ENSRNOT00000002984	0.998397069	8.31E-14
TUCP_001904	XLOC_078874	ENSRNOT00000083764	0.999541808	1.59E-16
TUCP_001904	XLOC_078874	ENSRNOT00000076802	0.962695843	5.34E-07
TUCP_001904	XLOC_078874	ENSRNOT00000092365	0.999161651	3.26E-15
TUCP_001904	XLOC_078874	ENSRNOT00000079275	0.999901911	7.15E-20
TUCP_001904	XLOC_078874	ENSRNOT00000066062	-0.959706712	7.82E-07
TUCP_001904	XLOC_078874	ENSRNOT00000045301	1	0
TUCP_001904	XLOC_078874	ENSRNOT00000041186	0.999975782	6.56E-23
TUCP_001904	XLOC_078874	ENSRNOT00000010092	0.977711778	4.17E-08
TUCP_001904	XLOC_078874	ENSRNOT00000076182	1	1.33E-79
TUCP_001904	XLOC_078874	ENSRNOT00000087404	0.951157664	2.02E-06
TUCP_001904	XLOC_078874	ENSRNOT00000052003	0.95837331	9.18E-07
TUCP_001904	XLOC_078874	ENSRNOT00000081709	0.998485154	6.27E-14
TUCP_001904	XLOC_078874	ENSRNOT00000005726	0.959610119	7.91E-07
TUCP_001904	XLOC_078874	ENSRNOT00000014103	1	0
TUCP_001904	XLOC_078874	ENSRNOT00000057433	0.964930601	3.94E-07
TUCP_001904	XLOC_078874	ENSRNOT00000082289	0.998931566	1.09E-14
TUCP_001904	XLOC_078874	ENSRNOT00000092516	0.957459323	1.02E-06
TUCP_001904	XLOC_078874	ENSRNOT00000008898	0.990039768	7.59E-10

TUCP_001904	XLOC_078874	ENSRNOT00000083493	0.995461232	1.51E-11
TUCP_001904	XLOC_078874	ENSRNOT00000092563	0.987201084	2.65E-09
TUCP_001904	XLOC_078874	ENSRNOT00000088893	1	1.33E-79
TUCP_001904	XLOC_078874	ENSRNOT00000076693	0.955029582	1.34E-06
TUCP_001904	XLOC_078874	ENSRNOT00000081014	1	0
TUCP_001904	XLOC_078874	ENSRNOT00000067543	1	1.33E-79
TUCP_001904	XLOC_078874	ENSRNOT00000050968	0.996816433	2.56E-12
TUCP_001904	XLOC_078874	ENSRNOT00000042201	0.960235595	7.32E-07
TUCP_001904	XLOC_078874	ENSRNOT00000004373	0.957315473	1.04E-06
TUCP_001904	XLOC_078874	ENSRNOT00000063929	1	0
TUCP_001904	XLOC_078874	ENSRNOT00000057472	1	1.33E-79
TUCP_001904	XLOC_078874	ENSRNOT00000041617	0.952449765	1.77E-06
TUCP_001904	XLOC_078874	ENSRNOT00000091763	0.986310385	3.70E-09
TUCP_001904	XLOC_078874	ENSRNOT00000022200	0.998491303	6.14E-14
TUCP_001904	XLOC_078874	ENSRNOT00000067379	0.997260967	1.21E-12
TUCP_001904	XLOC_078874	ENSRNOT00000020786	0.976428448	5.51E-08
TUCP_001904	XLOC_078874	ENSRNOT00000050543	0.999722223	1.30E-17
TUCP_001904	XLOC_078874	ENSRNOT00000089306	0.999866649	3.32E-19
TUCP_001904	XLOC_078874	ENSRNOT00000052367	0.997268394	1.19E-12
TUCP_001904	XLOC_078874	ENSRNOT00000090658	1	0
TUCP_001904	XLOC_078874	ENSRNOT00000022571	0.995290013	1.81E-11
TUCP_001904	XLOC_078874	ENSRNOT00000044520	0.981817158	1.52E-08
TUCP_001904	XLOC_078874	ENSRNOT00000065448	0.963648868	4.70E-07
TUCP_001902	XLOC_078874	ENSRNOT00000026966	0.971037903	1.53E-07
TUCP_001902	XLOC_078874	ENSRNOT00000068416	0.994279089	4.78E-11
TUCP_001902	XLOC_078874	ENSRNOT00000086036	0.993920314	6.48E-11
TUCP_001902	XLOC_078874	ENSRNOT00000026803	0.976713022	5.19E-08
TUCP_001902	XLOC_078874	ENSRNOT00000081709	0.991758685	2.95E-10
TUCP_001902	XLOC_078874	ENSRNOT00000042251	0.993626405	8.20E-11
TUCP_001902	XLOC_078874	ENSRNOT00000002984	0.992849161	1.45E-10
TUCP_001902	XLOC_078874	ENSRNOT00000020071	0.965195355	3.79E-07
TUCP_001902	XLOC_078874	ENSRNOT00000083764	0.992762064	1.55E-10
TUCP_001902	XLOC_078874	ENSRNOT00000050543	0.994792055	2.99E-11
TUCP_001902	XLOC_078874	ENSRNOT00000092365	0.993952058	6.31E-11
TUCP_001902	XLOC_078874	ENSRNOT00000089306	0.994190692	5.16E-11
TUCP_001902	XLOC_078874	ENSRNOT00000066062	-0.966322167	3.22E-07
TUCP_001902	XLOC_078874	ENSRNOT00000045301	0.994316007	4.63E-11
TUCP_001902	XLOC_078874	ENSRNOT00000041186	0.99403051	5.91E-11
TUCP_001902	XLOC_078874	ENSRNOT00000010092	0.986562811	3.37E-09
TUCP_001902	XLOC_078874	ENSRNOT00000076182	0.994316007	4.63E-11
TUCP_001902	XLOC_078874	ENSRNOT00000038494	0.960699094	6.91E-07
TUCP_001902	XLOC_078874	ENSRNOT00000081830	0.952647856	1.73E-06
TUCP_001902	XLOC_078874	ENSRNOT00000047527	0.977930551	3.97E-08
TUCP_001902	XLOC_078874	ENSRNOT00000005726	0.976314831	5.64E-08
TUCP_001902	XLOC_078874	ENSRNOT00000014103	0.994316007	4.63E-11
TUCP_001902	XLOC_078874	ENSRNOT00000057433	0.97909683	3.03E-08
TUCP_001902	XLOC_078874	ENSRNOT00000082289	0.994549904	3.75E-11
TUCP_001902	XLOC_078874	ENSRNOT00000092516	0.958213916	9.35E-07
TUCP_001902	XLOC_078874	ENSRNOT00000082143	0.965794155	3.48E-07
TUCP_001902	XLOC_078874	ENSRNOT00000085570	0.954957516	1.35E-06
TUCP_001902	XLOC_078874	ENSRNOT00000083493	0.987866401	2.03E-09

TUCP_001902	XLOC_078874	ENSRNOT00000092563	0.983419335	9.60E-09
TUCP_001902	XLOC_078874	ENSRNOT00000088893	0.994316007	4.63E-11
TUCP_001902	XLOC_078874	ENSRNOT00000076693	0.971568045	1.40E-07
TUCP_001902	XLOC_078874	ENSRNOT00000081014	0.994316007	4.63E-11
TUCP_001902	XLOC_078874	ENSRNOT00000067543	0.994316007	4.63E-11
TUCP_001902	XLOC_078874	ENSRNOT00000076802	0.982810385	1.15E-08
TUCP_001902	XLOC_078874	ENSRNOT00000050968	0.992941349	1.36E-10
TUCP_001902	XLOC_078874	ENSRNOT00000042201	0.953191152	1.64E-06
TUCP_001902	XLOC_078874	ENSRNOT00000004373	0.957854753	9.76E-07
TUCP_001902	XLOC_078874	ENSRNOT00000063929	0.994316007	4.63E-11
TUCP_001902	XLOC_078874	ENSRNOT00000057472	0.994316007	4.63E-11
TUCP_001902	XLOC_078874	ENSRNOT00000041617	0.962310898	5.62E-07
TUCP_001902	XLOC_078874	ENSRNOT00000091763	0.984147025	7.68E-09
TUCP_001902	XLOC_078874	ENSRNOT00000022200	0.99333755	1.02E-10
TUCP_001902	XLOC_078874	ENSRNOT00000067379	0.99149545	3.45E-10
TUCP_001902	XLOC_078874	ENSRNOT00000020786	0.981788288	1.53E-08
TUCP_001902	XLOC_078874	ENSRNOT00000084847	0.96216939	5.73E-07
TUCP_001902	XLOC_078874	ENSRNOT00000079275	0.995365474	1.67E-11
TUCP_001902	XLOC_078874	ENSRNOT00000052367	0.9942345	4.97E-11
TUCP_001902	XLOC_078874	ENSRNOT00000090658	0.994316007	4.63E-11
TUCP_001902	XLOC_078874	ENSRNOT00000022571	0.990028385	7.64E-10
TUCP_001902	XLOC_078874	ENSRNOT00000088078	0.961020206	6.64E-07
TUCP_001902	XLOC_078874	ENSRNOT00000052003	0.972517491	1.18E-07
TUCP_001902	XLOC_078874	ENSRNOT00000008898	0.979165091	2.99E-08
TUCP_001902	XLOC_078874	ENSRNOT00000044520	0.968753338	2.23E-07
TUCP_001902	XLOC_078874	ENSRNOT00000065448	0.982960877	1.10E-08
TUCP_001902	XLOC_078874	ENSRNOT00000072810	0.956974256	1.08E-06
TUCP_002388	XLOC_100877	ENSRNOT00000081839	0.952584042	1.74E-06
TUCP_002502	XLOC_105647	ENSRNOT00000073718	0.951549793	1.94E-06
TUCP_002501	XLOC_105430	ENSRNOT00000045833	0.964652494	4.10E-07
TUCP_002501	XLOC_105430	ENSRNOT00000013743	0.982913965	1.11E-08
TUCP_002501	XLOC_105430	ENSRNOT00000007350	0.951750159	1.90E-06
TUCP_002501	XLOC_105430	ENSRNOT00000004400	0.965214763	3.78E-07
TUCP_002501	XLOC_105430	ENSRNOT00000087062	0.971340054	1.45E-07
TUCP_002501	XLOC_105430	ENSRNOT00000082914	0.950766898	2.10E-06
TUCP_002501	XLOC_105430	ENSRNOT00000048613	0.96302682	5.11E-07
TUCP_002501	XLOC_105430	ENSRNOT00000023437	0.954290905	1.46E-06
TUCP_002501	XLOC_105430	ENSRNOT00000013025	-0.950805388	2.09E-06
TUCP_002501	XLOC_105430	ENSRNOT00000004017	0.960860596	6.77E-07
TUCP_002501	XLOC_105430	ENSRNOT00000036051	0.95321083	1.63E-06
TUCP_002501	XLOC_105430	ENSRNOT00000001679	0.962637614	5.39E-07
TUCP_002501	XLOC_105430	ENSRNOT00000010418	0.95031789	2.19E-06
TUCP_002501	XLOC_105430	ENSRNOT00000015006	0.952159094	1.82E-06
TUCP_002501	XLOC_105430	ENSRNOT00000013516	0.969856132	1.86E-07
TUCP_002501	XLOC_105430	ENSRNOT00000078884	-0.953143613	1.64E-06
TUCP_002501	XLOC_105430	ENSRNOT00000001507	0.953250516	1.63E-06
TUCP_002501	XLOC_105430	ENSRNOT00000073370	0.968923627	2.17E-07
TUCP_002501	XLOC_105430	ENSRNOT00000018584	0.955047275	1.34E-06
TUCP_002501	XLOC_105430	ENSRNOT00000091637	0.959424223	8.09E-07
TUCP_002501	XLOC_105430	ENSRNOT00000023037	0.975946612	6.09E-08
TUCP_002501	XLOC_105430	ENSRNOT00000077060	0.9567106	1.11E-06

TUCP_002501	XLOC_105430	ENSRNOT00000017271	0.965652687	3.55E-07
TUCP_002501	XLOC_105430	ENSRNOT00000017373	0.955291781	1.30E-06
TUCP_002501	XLOC_105430	ENSRNOT00000034282	0.965515586	3.62E-07
TUCP_002501	XLOC_105430	ENSRNOT00000024677	0.969450927	1.99E-07
TUCP_002501	XLOC_105430	ENSRNOT00000037685	0.960909096	6.73E-07
TUCP_002501	XLOC_105430	ENSRNOT00000090461	0.961766472	6.03E-07
TUCP_002501	XLOC_105430	ENSRNOT00000030082	0.955407735	1.29E-06
TUCP_002501	XLOC_105430	ENSRNOT00000087287	0.96407104	4.44E-07
TUCP_002501	XLOC_105430	ENSRNOT00000026392	0.967318494	2.78E-07
TUCP_002501	XLOC_105430	ENSRNOT00000013286	0.957205725	1.05E-06
TUCP_002501	XLOC_105430	ENSRNOT00000011926	0.953052673	1.66E-06
TUCP_002501	XLOC_105430	ENSRNOT00000005686	-0.956524284	1.14E-06
TUCP_002501	XLOC_105430	ENSRNOT00000071540	0.95245794	1.77E-06
TUCP_002501	XLOC_105430	ENSRNOT00000019820	0.950692811	2.11E-06
TUCP_002501	XLOC_105430	ENSRNOT00000073860	-0.950037871	2.25E-06
TUCP_002501	XLOC_105430	ENSRNOT00000011832	-0.960293054	7.27E-07
TUCP_002501	XLOC_105430	ENSRNOT00000030074	0.972307577	1.22E-07
TUCP_002501	XLOC_105430	ENSRNOT00000083968	-0.951985909	1.85E-06
TUCP_002501	XLOC_105430	ENSRNOT00000083578	0.95586997	1.22E-06
TUCP_002501	XLOC_105430	ENSRNOT00000019688	0.954730656	1.39E-06
TUCP_002501	XLOC_105430	ENSRNOT00000084709	0.953173632	1.64E-06
TUCP_002501	XLOC_105430	ENSRNOT00000092068	0.950004252	2.26E-06
TUCP_002501	XLOC_105430	ENSRNOT00000009156	0.966956612	2.93E-07
TUCP_002501	XLOC_105430	ENSRNOT00000021802	0.952435028	1.77E-06
TUCP_002501	XLOC_105430	ENSRNOT00000003265	0.953385924	1.60E-06
TUCP_002501	XLOC_105430	ENSRNOT00000068452	0.969550633	1.96E-07
TUCP_002501	XLOC_105430	ENSRNOT00000007821	0.959572901	7.95E-07
TUCP_002501	XLOC_105430	ENSRNOT00000018122	0.968201495	2.43E-07
TUCP_002501	XLOC_105430	ENSRNOT00000072357	-0.954920119	1.36E-06
TUCP_002501	XLOC_105430	ENSRNOT00000059246	0.955597702	1.26E-06
TUCP_002501	XLOC_105430	ENSRNOT00000011956	0.951606621	1.93E-06
TUCP_002501	XLOC_105430	ENSRNOT00000024119	0.965541699	3.61E-07
TUCP_002501	XLOC_105430	ENSRNOT00000091427	-0.973424295	9.98E-08
TUCP_002501	XLOC_105430	ENSRNOT00000005604	0.950097962	2.24E-06
TUCP_002501	XLOC_105430	ENSRNOT00000024110	0.958604699	8.93E-07
TUCP_002501	XLOC_105430	ENSRNOT00000016637	-0.958075688	9.51E-07
TUCP_002501	XLOC_105430	ENSRNOT00000086260	-0.952184716	1.82E-06
TUCP_002501	XLOC_105430	ENSRNOT00000068381	0.962694004	5.34E-07
TUCP_002501	XLOC_105430	ENSRNOT00000054948	0.961069677	6.60E-07
TUCP_002501	XLOC_105430	ENSRNOT00000020383	0.954180415	1.47E-06
TUCP_002501	XLOC_105430	ENSRNOT00000058382	0.951244557	2.00E-06
TUCP_002501	XLOC_105430	ENSRNOT00000026531	-0.953904962	1.52E-06
TUCP_002501	XLOC_105430	ENSRNOT00000005172	0.959276248	8.24E-07
TUCP_002501	XLOC_105430	ENSRNOT00000011880	0.957035836	1.07E-06
TUCP_002501	XLOC_105430	ENSRNOT00000000898	0.953136264	1.65E-06
TUCP_002501	XLOC_105430	ENSRNOT00000031261	0.952354122	1.78E-06
TUCP_002501	XLOC_105430	ENSRNOT00000081725	0.960012489	7.53E-07
TUCP_002501	XLOC_105430	ENSRNOT00000089980	-0.960771958	6.85E-07
TUCP_002501	XLOC_105430	ENSRNOT00000005459	0.95436088	1.44E-06
TUCP_002501	XLOC_105430	ENSRNOT00000011675	0.950156377	2.23E-06
TUCP_002501	XLOC_105430	ENSRNOT00000020795	0.961912431	5.92E-07

TUCP_002501	XLOC_105430	ENSRNOT00000041106	0.96736701	2.76E-07
TUCP_002501	XLOC_105430	ENSRNOT00000023876	0.95615617	1.19E-06
TUCP_002501	XLOC_105430	ENSRNOT00000033359	0.979263009	2.92E-08
TUCP_002501	XLOC_105430	ENSRNOT00000002974	0.951142686	2.02E-06
TUCP_002501	XLOC_105430	ENSRNOT00000028888	0.958150883	9.42E-07
TUCP_002501	XLOC_105430	ENSRNOT00000066032	0.977717102	4.17E-08
TUCP_002501	XLOC_105430	ENSRNOT00000005161	0.955918063	1.22E-06
TUCP_002501	XLOC_105430	ENSRNOT00000019876	0.961823727	5.99E-07
TUCP_002501	XLOC_105430	ENSRNOT00000073919	0.961951379	5.89E-07
TUCP_002501	XLOC_105430	ENSRNOT00000030449	0.955932385	1.22E-06
TUCP_002501	XLOC_105430	ENSRNOT00000037955	-0.95870822	8.82E-07
TUCP_002501	XLOC_105430	ENSRNOT00000035277	0.973744062	9.40E-08
TUCP_002501	XLOC_105430	ENSRNOT00000086122	0.952249106	1.80E-06
TUCP_002501	XLOC_105430	ENSRNOT00000004999	0.953263504	1.62E-06
TUCP_002501	XLOC_105430	ENSRNOT00000025314	0.962232423	5.68E-07
TUCP_002501	XLOC_105430	ENSRNOT00000012386	0.950292799	2.20E-06
TUCP_002501	XLOC_105430	ENSRNOT00000086995	0.955867611	1.22E-06
TUCP_002501	XLOC_105430	ENSRNOT00000037246	0.950184852	2.22E-06
TUCP_002501	XLOC_105430	ENSRNOT00000010993	0.967264843	2.80E-07
TUCP_002501	XLOC_105430	ENSRNOT00000059181	0.950308015	2.19E-06
TUCP_002501	XLOC_105430	ENSRNOT00000017765	0.955427971	1.29E-06
TUCP_002501	XLOC_105430	ENSRNOT00000017761	0.958822006	8.70E-07
TUCP_002501	XLOC_105430	ENSRNOT00000009994	-0.960413534	7.16E-07
TUCP_002501	XLOC_105430	ENSRNOT00000005405	0.956284665	1.17E-06
TUCP_002501	XLOC_105430	ENSRNOT00000087408	0.971200985	1.49E-07
TUCP_002501	XLOC_105430	ENSRNOT00000009462	0.95991038	7.62E-07
TUCP_002501	XLOC_105430	ENSRNOT00000039102	0.96002477	7.52E-07
TUCP_002501	XLOC_105430	ENSRNOT00000085517	0.95765551	9.98E-07
TUCP_002501	XLOC_105430	ENSRNOT00000037405	0.959403716	8.11E-07
TUCP_002501	XLOC_105430	ENSRNOT00000009742	-0.951573192	1.93E-06
TUCP_002501	XLOC_105430	ENSRNOT00000013632	0.954849046	1.37E-06
TUCP_002501	XLOC_105430	ENSRNOT00000085279	0.95990646	7.63E-07
TUCP_002501	XLOC_105430	ENSRNOT00000025460	0.967846515	2.56E-07
TUCP_002501	XLOC_105430	ENSRNOT00000080774	0.951812832	1.89E-06
TUCP_002501	XLOC_105430	ENSRNOT00000026075	0.970770172	1.60E-07
TUCP_002501	XLOC_105430	ENSRNOT00000017998	0.963787762	4.61E-07
TUCP_002501	XLOC_105430	ENSRNOT00000021216	0.962136009	5.75E-07
TUCP_002501	XLOC_105430	ENSRNOT00000082904	0.950978411	2.05E-06
TUCP_002501	XLOC_105430	ENSRNOT00000015223	0.953352412	1.61E-06
TUCP_002501	XLOC_105430	ENSRNOT00000028907	0.97131907	1.46E-07
TUCP_002501	XLOC_105430	ENSRNOT00000010224	0.970638223	1.64E-07
TUCP_002501	XLOC_105430	ENSRNOT00000088214	0.954562608	1.41E-06
TUCP_002501	XLOC_105430	ENSRNOT00000035309	-0.950284063	2.20E-06
TUCP_002501	XLOC_105430	ENSRNOT00000024011	0.966551722	3.12E-07
TUCP_002501	XLOC_105430	ENSRNOT00000065722	-0.962620225	5.40E-07
TUCP_002501	XLOC_105430	ENSRNOT00000020051	0.967130318	2.86E-07
TUCP_002501	XLOC_105430	ENSRNOT00000092876	-0.950126687	2.23E-06
TUCP_002501	XLOC_105430	ENSRNOT00000074395	0.952125899	1.83E-06
TUCP_002501	XLOC_105430	ENSRNOT00000024253	0.964875962	3.97E-07
TUCP_002500	XLOC_105385	ENSRNOT00000088957	-0.9618868	5.94E-07
TUCP_002507	XLOC_105649	ENSRNOT00000085476	0.975262207	7.00E-08

TUCP_002507	XLOC_105649	ENSRNOT00000083608	0.958182783	9.39E-07
TUCP_002507	XLOC_105649	ENSRNOT00000076035	0.955881245	1.22E-06
TUCP_002507	XLOC_105649	ENSRNOT00000091828	0.951936887	1.86E-06
TUCP_002507	XLOC_105649	ENSRNOT00000078176	0.965633759	3.56E-07
TUCP_002507	XLOC_105649	ENSRNOT00000047560	0.955362527	1.29E-06
TUCP_002507	XLOC_105649	ENSRNOT00000040386	0.959609936	7.91E-07
TUCP_002507	XLOC_105649	ENSRNOT00000024758	0.966531254	3.13E-07
TUCP_002507	XLOC_105649	ENSRNOT00000088719	0.984410548	7.06E-09
TUCP_002507	XLOC_105649	ENSRNOT00000033187	0.970132449	1.78E-07
TUCP_001874	XLOC_077907	ENSRNOT00000007314	-0.976994573	4.88E-08
TUCP_001874	XLOC_077907	ENSRNOT00000003412	-0.952561807	1.75E-06
TUCP_001874	XLOC_077907	ENSRNOT00000007079	-0.95731201	1.04E-06
TUCP_001874	XLOC_077907	ENSRNOT00000035628	-0.957312167	1.04E-06
TUCP_001874	XLOC_077907	ENSRNOT00000009584	-0.953120627	1.65E-06
TUCP_001874	XLOC_077907	ENSRNOT00000027083	0.951198601	2.01E-06
TUCP_001874	XLOC_077907	ENSRNOT00000014924	-0.954192282	1.47E-06
TUCP_001874	XLOC_077907	ENSRNOT00000050834	-0.960680096	6.93E-07
TUCP_001874	XLOC_077907	ENSRNOT00000023567	-0.954900778	1.36E-06
TUCP_001874	XLOC_077907	ENSRNOT00000072007	-0.95210243	1.83E-06
TUCP_001874	XLOC_077907	ENSRNOT00000030692	-0.951472647	1.95E-06
TUCP_001874	XLOC_077907	ENSRNOT00000028290	-0.950369529	2.18E-06
TUCP_001874	XLOC_077907	ENSRNOT00000007771	-0.950233135	2.21E-06
TUCP_001874	XLOC_077907	ENSRNOT00000070912	-0.95737327	1.03E-06
TUCP_001874	XLOC_077907	ENSRNOT00000002445	-0.959578772	7.94E-07
TUCP_001874	XLOC_077907	ENSRNOT00000083173	-0.956296685	1.17E-06
TUCP_001874	XLOC_077907	ENSRNOT00000083351	-0.951556829	1.94E-06
TUCP_001874	XLOC_077907	ENSRNOT00000036338	-0.952853444	1.69E-06
TUCP_001874	XLOC_077907	ENSRNOT00000050609	-0.975334429	6.90E-08
TUCP_001874	XLOC_077907	ENSRNOT00000037039	-0.960445988	7.13E-07
TUCP_001874	XLOC_077907	ENSRNOT00000016428	0.953572344	1.57E-06
TUCP_001874	XLOC_077907	ENSRNOT00000003940	-0.958469685	9.07E-07
TUCP_001874	XLOC_077907	ENSRNOT00000028625	-0.950607957	2.13E-06
TUCP_001874	XLOC_077907	ENSRNOT00000028484	-0.952134818	1.83E-06
TUCP_001874	XLOC_077907	ENSRNOT00000055386	0.968061301	2.48E-07
TUCP_001874	XLOC_077907	ENSRNOT00000011930	0.950659734	2.12E-06
TUCP_001874	XLOC_077907	ENSRNOT00000003714	0.967719322	2.61E-07
TUCP_001874	XLOC_077907	ENSRNOT00000079985	-0.956354699	1.16E-06
TUCP_001874	XLOC_077907	ENSRNOT00000052224	0.957871927	9.74E-07
TUCP_001874	XLOC_077907	ENSRNOT00000078070	0.950512509	2.15E-06
TUCP_001874	XLOC_077907	ENSRNOT00000032289	-0.95643054	1.15E-06
TUCP_001874	XLOC_077907	ENSRNOT00000088353	-0.967411689	2.74E-07
TUCP_001874	XLOC_077907	ENSRNOT00000045233	-0.956209364	1.18E-06
TUCP_001874	XLOC_077907	ENSRNOT00000003715	-0.952342406	1.79E-06
TUCP_001874	XLOC_077907	ENSRNOT00000033048	-0.955041306	1.34E-06
TUCP_001874	XLOC_077907	ENSRNOT00000000206	-0.953462085	1.59E-06
TUCP_001874	XLOC_077907	ENSRNOT00000067170	-0.950842997	2.08E-06
TUCP_001874	XLOC_077907	ENSRNOT00000086350	-0.953206229	1.63E-06
TUCP_001874	XLOC_077907	ENSRNOT00000038182	-0.952840324	1.70E-06
TUCP_001874	XLOC_077907	ENSRNOT00000086893	-0.959682669	7.84E-07
TUCP_001874	XLOC_077907	ENSRNOT00000004787	-0.961892261	5.94E-07
TUCP_001874	XLOC_077907	ENSRNOT00000079002	-0.960568882	7.03E-07

TUCP_001874	XLOC_077907	ENSRNOT00000001759	-0.950567429	2.14E-06
TUCP_001874	XLOC_077907	ENSRNOT00000025856	-0.9505219	2.15E-06
TUCP_001874	XLOC_077907	ENSRNOT00000028589	-0.953308606	1.62E-06
TUCP_001874	XLOC_077907	ENSRNOT00000003326	-0.950443907	2.17E-06
TUCP_001874	XLOC_077907	ENSRNOT00000004319	-0.965295521	3.74E-07
TUCP_001874	XLOC_077907	ENSRNOT00000024491	-0.958122025	9.45E-07
TUCP_001874	XLOC_077907	ENSRNOT00000008911	-0.956973888	1.08E-06
TUCP_001874	XLOC_077907	ENSRNOT00000015170	-0.950694438	2.11E-06
TUCP_001874	XLOC_077907	ENSRNOT00000042125	0.952976958	1.67E-06
TUCP_001874	XLOC_077907	ENSRNOT00000024947	-0.964907693	3.95E-07
TUCP_001874	XLOC_077907	ENSRNOT00000028703	-0.956086909	1.19E-06
TUCP_001874	XLOC_077907	ENSRNOT00000015757	-0.973069855	1.07E-07
TUCP_001874	XLOC_077907	ENSRNOT00000006943	0.952473469	1.76E-06
TUCP_001874	XLOC_077907	ENSRNOT00000027141	-0.963223941	4.98E-07
TUCP_001874	XLOC_077907	ENSRNOT00000002151	-0.9555153	1.27E-06
TUCP_001874	XLOC_077907	ENSRNOT00000016220	-0.964510281	4.18E-07
TUCP_001874	XLOC_077907	ENSRNOT00000022000	-0.966503928	3.14E-07
TUCP_001874	XLOC_077907	ENSRNOT00000054966	-0.951397007	1.97E-06
TUCP_001874	XLOC_077907	ENSRNOT00000008865	-0.953214668	1.63E-06
TUCP_001874	XLOC_077907	ENSRNOT00000033196	-0.957514217	1.01E-06
TUCP_001874	XLOC_077907	ENSRNOT00000051554	-0.953190427	1.64E-06
TUCP_001874	XLOC_077907	ENSRNOT00000017949	-0.955712494	1.25E-06
TUCP_001874	XLOC_077907	ENSRNOT00000004007	-0.954132405	1.48E-06
TUCP_001874	XLOC_077907	ENSRNOT00000000177	-0.961712423	6.08E-07
TUCP_001874	XLOC_077907	ENSRNOT00000020910	0.95207519	1.84E-06
TUCP_001874	XLOC_077907	ENSRNOT00000074494	-0.956993052	1.08E-06
TUCP_001874	XLOC_077907	ENSRNOT00000003794	-0.956195644	1.18E-06
TUCP_001874	XLOC_077907	ENSRNOT00000091602	-0.957290753	1.04E-06
TUCP_002970	XLOC_128262	ENSRNOT00000061333	0.951613575	1.93E-06
TUCP_002970	XLOC_128262	ENSRNOT00000011827	0.96578743	3.49E-07
TUCP_002970	XLOC_128262	ENSRNOT00000017911	-0.954011179	1.50E-06
TUCP_002970	XLOC_128262	ENSRNOT00000009319	-0.950726983	2.11E-06
TUCP_002970	XLOC_128262	ENSRNOT00000079180	0.95075234	2.10E-06
TUCP_002970	XLOC_128262	ENSRNOT00000031026	0.961410522	6.32E-07
TUCP_002508	XLOC_105660	ENSRNOT00000033112	0.961361196	6.36E-07
TUCP_002508	XLOC_105660	ENSRNOT00000030737	0.957779218	9.84E-07
TUCP_004726	XLOC_209103	ENSRNOT00000065646	0.953576594	1.57E-06
TUCP_004726	XLOC_209103	ENSRNOT00000031626	0.954730023	1.39E-06
TUCP_002974	XLOC_128384	ENSRNOT00000085110	0.953165973	1.64E-06
TUCP_002974	XLOC_128384	ENSRNOT00000008037	0.95291215	1.68E-06
TUCP_003138	XLOC_134764	ENSRNOT00000024186	0.983200502	1.02E-08
TUCP_003138	XLOC_134764	ENSRNOT00000074753	0.998182349	1.56E-13
TUCP_003138	XLOC_134764	ENSRNOT00000004876	0.959071443	8.44E-07
TUCP_003138	XLOC_134764	ENSRNOT00000041904	1	0
TUCP_003138	XLOC_134764	ENSRNOT00000083487	0.989515314	9.80E-10
TUCP_003138	XLOC_134764	ENSRNOT00000077990	1	0
TUCP_003138	XLOC_134764	ENSRNOT00000038826	0.99977672	4.37E-18
TUCP_003138	XLOC_134764	ENSRNOT00000046842	0.973644314	9.58E-08
TUCP_003138	XLOC_134764	ENSRNOT00000037169	0.980329406	2.24E-08
TUCP_003138	XLOC_134764	ENSRNOT00000021243	0.987425406	2.42E-09
TUCP_003138	XLOC_134764	ENSRNOT00000060250	0.999933853	9.97E-21

TUCP_003138	XLOC_134764	ENSRNOT00000091934	1	0
TUCP_003138	XLOC_134764	ENSRNOT00000046585	0.982941597	1.11E-08
TUCP_003138	XLOC_134764	ENSRNOT00000080794	0.990839253	5.00E-10
TUCP_003138	XLOC_134764	ENSRNOT00000014456	0.991697153	3.06E-10
TUCP_003138	XLOC_134764	ENSRNOT00000031205	0.991394766	3.66E-10
TUCP_003138	XLOC_134764	ENSRNOT00000044145	1	0
TUCP_003138	XLOC_134764	ENSRNOT00000088381	0.989013276	1.24E-09
TUCP_003138	XLOC_134764	ENSRNOT00000011743	0.98678427	3.11E-09
TUCP_003138	XLOC_134764	ENSRNOT00000089929	1	0
TUCP_003138	XLOC_134764	ENSRNOT00000088624	1	0
TUCP_003138	XLOC_134764	ENSRNOT00000085659	0.989908621	8.10E-10
TUCP_003138	XLOC_134764	ENSRNOT00000015866	0.994066664	5.73E-11
TUCP_003138	XLOC_134764	ENSRNOT00000078633	1	0
TUCP_003138	XLOC_134764	ENSRNOT00000034907	0.995318542	1.76E-11
TUCP_003138	XLOC_134764	ENSRNOT00000093230	0.958295175	9.26E-07
TUCP_003138	XLOC_134764	ENSRNOT00000051835	1	0
TUCP_003138	XLOC_134764	ENSRNOT00000079087	1	0
TUCP_003138	XLOC_134764	ENSRNOT00000037206	1	0
TUCP_003138	XLOC_134764	ENSRNOT00000083261	0.970991658	1.54E-07
TUCP_003138	XLOC_134764	ENSRNOT00000080590	0.999889216	1.31E-19
TUCP_003138	XLOC_134764	ENSRNOT00000027552	0.999801024	2.46E-18
TUCP_003138	XLOC_134764	ENSRNOT00000077014	0.995074376	2.26E-11
TUCP_003138	XLOC_134764	ENSRNOT00000080453	0.99994332	4.61E-21
TUCP_003138	XLOC_134764	ENSRNOT00000089057	0.968243551	2.41E-07
TUCP_003138	XLOC_134764	ENSRNOT00000066839	0.991868612	2.76E-10
TUCP_003138	XLOC_134764	ENSRNOT00000061284	0.955566942	1.27E-06
TUCP_003138	XLOC_134764	ENSRNOT00000013423	0.959677621	7.84E-07
TUCP_003138	XLOC_134764	ENSRNOT00000071884	0.974769955	7.72E-08
TUCP_003138	XLOC_134764	ENSRNOT00000004128	0.961829362	5.99E-07
TUCP_003138	XLOC_134764	ENSRNOT00000026316	0.964408046	4.24E-07
TUCP_003138	XLOC_134764	ENSRNOT00000057729	0.999384184	6.97E-16
TUCP_003138	XLOC_134764	ENSRNOT00000078663	0.975192577	7.10E-08
TUCP_003138	XLOC_134764	ENSRNOT00000064250	0.983131977	1.05E-08
TUCP_003138	XLOC_134764	ENSRNOT00000021608	0.965228881	3.78E-07
TUCP_003138	XLOC_134764	ENSRNOT00000029634	0.995816134	1.00E-11
TUCP_003138	XLOC_134764	ENSRNOT00000093579	0.958564264	8.97E-07
TUCP_003138	XLOC_134764	ENSRNOT00000086349	0.957242511	1.05E-06
TUCP_003138	XLOC_134764	ENSRNOT00000065460	1	0
TUCP_003138	XLOC_134764	ENSRNOT00000003817	0.996778712	2.72E-12
TUCP_003138	XLOC_134764	ENSRNOT00000016713	1	1.33E-79
TUCP_003138	XLOC_134764	ENSRNOT00000079260	0.996448245	4.42E-12
TUCP_003138	XLOC_134764	ENSRNOT00000039294	1	0
TUCP_003138	XLOC_134764	ENSRNOT00000092913	0.952980228	1.67E-06
TUCP_002876	XLOC_122775	ENSRNOT00000078502	0.951253075	2.00E-06
TUCP_002876	XLOC_122775	ENSRNOT00000076227	0.951306127	1.99E-06
TUCP_002875	XLOC_122673	ENSRNOT00000091493	-0.96039598	7.18E-07
TUCP_002875	XLOC_122673	ENSRNOT00000024813	-0.953395824	1.60E-06
TUCP_002875	XLOC_122673	ENSRNOT00000020826	-0.96897346	2.15E-07
TUCP_000297	XLOC_009319	ENSRNOT00000012413	0.951925247	1.87E-06
TUCP_002878	XLOC_122775	ENSRNOT00000068171	0.954780462	1.38E-06
TUCP_001627	XLOC_064649	ENSRNOT00000093579	0.954657074	1.40E-06

TUCP_001627	XLOC_064649	ENSRNOT00000012170	0.965886893	3.44E-07
TUCP_001879	XLOC_077950	ENSRNOT00000049582	0.959396187	8.12E-07
TUCP_001626	XLOC_064609	ENSRNOT00000037509	0.954791868	1.38E-06
TUCP_004509	XLOC_198926	ENSRNOT00000061675	0.950982517	2.05E-06
TUCP_003533	XLOC_153788	ENSRNOT00000084004	0.950999814	2.05E-06
TUCP_003533	XLOC_153788	ENSRNOT00000011083	0.954077428	1.49E-06
TUCP_003533	XLOC_153788	ENSRNOT00000008760	0.961797541	6.01E-07
TUCP_002380	XLOC_100543	ENSRNOT00000087134	0.973584769	9.69E-08
TUCP_002380	XLOC_100543	ENSRNOT00000076187	0.960462999	7.12E-07
TUCP_002380	XLOC_100543	ENSRNOT00000055200	0.974728816	7.78E-08
TUCP_002380	XLOC_100543	ENSRNOT00000092026	0.973836787	9.24E-08
TUCP_001123	XLOC_042340	ENSRNOT00000002982	0.972648661	1.15E-07
TUCP_001123	XLOC_042340	ENSRNOT00000050180	0.956256994	1.17E-06
TUCP_001123	XLOC_042340	ENSRNOT00000024222	0.9533278	1.61E-06
TUCP_001123	XLOC_042340	ENSRNOT00000018179	0.98510581	5.63E-09
TUCP_001123	XLOC_042340	ENSRNOT00000086917	0.966174735	3.29E-07
TUCP_001123	XLOC_042340	ENSRNOT00000075621	0.953806899	1.53E-06
TUCP_001123	XLOC_042340	ENSRNOT00000024393	0.955522421	1.27E-06
TUCP_001123	XLOC_042340	ENSRNOT00000002560	0.966751571	3.03E-07
TUCP_001123	XLOC_042340	ENSRNOT00000047093	0.957163506	1.06E-06
TUCP_003430	XLOC_148230	ENSRNOT00000070972	0.960442324	7.14E-07
TUCP_003430	XLOC_148230	ENSRNOT00000000383	-0.950888976	2.07E-06
TUCP_003430	XLOC_148230	ENSRNOT00000061253	0.952381929	1.78E-06
TUCP_003430	XLOC_148230	ENSRNOT00000080024	0.95158997	1.93E-06
TUCP_002932	XLOC_126101	ENSRNOT00000084075	0.972997575	1.08E-07
TUCP_002932	XLOC_126101	ENSRNOT00000012880	0.992552752	1.78E-10
TUCP_002932	XLOC_126101	ENSRNOT00000074465	0.979732464	2.60E-08
TUCP_002932	XLOC_126101	ENSRNOT00000079805	0.975562352	6.59E-08
TUCP_002932	XLOC_126101	ENSRNOT00000040912	0.979732464	2.60E-08
TUCP_002932	XLOC_126101	ENSRNOT00000072648	0.979732464	2.60E-08
TUCP_002932	XLOC_126101	ENSRNOT00000007398	0.97745806	4.41E-08
TUCP_002932	XLOC_126101	ENSRNOT00000070803	0.979732464	2.60E-08
TUCP_002932	XLOC_126101	ENSRNOT00000012196	0.979732464	2.60E-08
TUCP_002932	XLOC_126101	ENSRNOT00000085772	0.979732464	2.60E-08
TUCP_002932	XLOC_126101	ENSRNOT00000093284	0.97947866	2.77E-08
TUCP_002932	XLOC_126101	ENSRNOT00000086374	0.986739212	3.16E-09
TUCP_002932	XLOC_126101	ENSRNOT00000015270	0.979732464	2.60E-08
TUCP_002932	XLOC_126101	ENSRNOT00000046192	0.979732464	2.60E-08
TUCP_002932	XLOC_126101	ENSRNOT00000044226	0.979732464	2.60E-08
TUCP_002932	XLOC_126101	ENSRNOT00000081898	0.959125446	8.39E-07
TUCP_002932	XLOC_126101	ENSRNOT00000080291	0.976541156	5.38E-08
TUCP_002932	XLOC_126101	ENSRNOT00000004293	0.965515997	3.62E-07
TUCP_002932	XLOC_126101	ENSRNOT00000078231	0.968816915	2.20E-07
TUCP_002932	XLOC_126101	ENSRNOT00000001464	0.979732464	2.60E-08
TUCP_002932	XLOC_126101	ENSRNOT00000090716	0.97446784	8.19E-08
TUCP_002932	XLOC_126101	ENSRNOT00000083542	0.966246061	3.26E-07
TUCP_002932	XLOC_126101	ENSRNOT00000054686	0.962419309	5.54E-07
TUCP_002932	XLOC_126101	ENSRNOT00000076044	0.979732464	2.60E-08
TUCP_002932	XLOC_126101	ENSRNOT00000074353	0.979732464	2.60E-08
TUCP_002932	XLOC_126101	ENSRNOT00000043510	0.981553213	1.63E-08
TUCP_002932	XLOC_126101	ENSRNOT00000087122	0.982188482	1.37E-08

TUCP_002932	XLOC_126101	ENSRNOT00000074295	0.970281685	1.74E-07
TUCP_002932	XLOC_126101	ENSRNOT00000057873	0.979732464	2.60E-08
TUCP_002932	XLOC_126101	ENSRNOT00000083002	0.984605197	6.64E-09
TUCP_002932	XLOC_126101	ENSRNOT00000088399	0.979723129	2.61E-08
TUCP_002932	XLOC_126101	ENSRNOT00000086248	0.979732464	2.60E-08
TUCP_002932	XLOC_126101	ENSRNOT00000089750	0.979885218	2.51E-08
TUCP_002932	XLOC_126101	ENSRNOT00000030517	0.965104177	3.84E-07
TUCP_002932	XLOC_126101	ENSRNOT00000034772	0.964237968	4.34E-07
TUCP_002932	XLOC_126101	ENSRNOT00000089305	0.993229098	1.11E-10
TUCP_002932	XLOC_126101	ENSRNOT00000064601	0.976288886	5.67E-08
TUCP_002932	XLOC_126101	ENSRNOT00000002834	0.979732464	2.60E-08
TUCP_002932	XLOC_126101	ENSRNOT00000080812	0.96730208	2.79E-07
TUCP_002932	XLOC_126101	ENSRNOT00000092755	0.979732464	2.60E-08
TUCP_002932	XLOC_126101	ENSRNOT00000031683	0.979732464	2.60E-08
TUCP_002932	XLOC_126101	ENSRNOT00000087764	0.975579917	6.56E-08
TUCP_004505	XLOC_198446	ENSRNOT00000090629	0.953476161	1.59E-06
TUCP_004505	XLOC_198446	ENSRNOT00000008961	0.977494012	4.38E-08
TUCP_004505	XLOC_198446	ENSRNOT00000039876	0.970623291	1.64E-07
TUCP_004505	XLOC_198446	ENSRNOT00000000681	0.950211774	2.22E-06
TUCP_004505	XLOC_198446	ENSRNOT00000055127	0.955473529	1.28E-06
TUCP_004505	XLOC_198446	ENSRNOT00000015387	0.953051921	1.66E-06
TUCP_004505	XLOC_198446	ENSRNOT00000037397	0.97332609	1.02E-07
TUCP_004505	XLOC_198446	ENSRNOT00000001570	0.972990573	1.08E-07
TUCP_004505	XLOC_198446	ENSRNOT00000067455	0.951919729	1.87E-06
TUCP_004505	XLOC_198446	ENSRNOT00000035523	0.975206023	7.08E-08
TUCP_004505	XLOC_198446	ENSRNOT00000024959	0.950543322	2.14E-06
TUCP_004505	XLOC_198446	ENSRNOT00000014158	0.977797822	4.09E-08
TUCP_004505	XLOC_198446	ENSRNOT00000033719	0.977629319	4.25E-08
TUCP_004505	XLOC_198446	ENSRNOT00000000640	0.961756583	6.04E-07
TUCP_004505	XLOC_198446	ENSRNOT00000056174	0.981534374	1.64E-08
TUCP_004505	XLOC_198446	ENSRNOT00000004363	0.95002905	2.26E-06
TUCP_004505	XLOC_198446	ENSRNOT00000074738	0.955566593	1.27E-06
TUCP_004505	XLOC_198446	ENSRNOT00000077022	0.968667058	2.26E-07
TUCP_004505	XLOC_198446	ENSRNOT00000071258	-0.954240475	1.46E-06
TUCP_004505	XLOC_198446	ENSRNOT00000032333	0.977523236	4.35E-08
TUCP_004505	XLOC_198446	ENSRNOT00000031025	0.972154725	1.26E-07
TUCP_004505	XLOC_198446	ENSRNOT00000056551	0.961535236	6.22E-07
TUCP_004505	XLOC_198446	ENSRNOT00000002695	0.97468163	7.85E-08
TUCP_004505	XLOC_198446	ENSRNOT00000046313	0.966918114	2.95E-07
TUCP_004505	XLOC_198446	ENSRNOT00000026303	0.961377148	6.34E-07
TUCP_004505	XLOC_198446	ENSRNOT00000001853	0.969989744	1.82E-07
TUCP_004505	XLOC_198446	ENSRNOT00000086541	0.953925402	1.51E-06
TUCP_004505	XLOC_198446	ENSRNOT00000057016	0.962928463	5.18E-07
TUCP_004505	XLOC_198446	ENSRNOT00000003823	0.968329561	2.38E-07
TUCP_004505	XLOC_198446	ENSRNOT00000060765	0.972804188	1.12E-07
TUCP_004505	XLOC_198446	ENSRNOT00000040162	0.980303085	2.26E-08
TUCP_004505	XLOC_198446	ENSRNOT00000058626	0.9710551	1.52E-07
TUCP_004505	XLOC_198446	ENSRNOT00000039263	0.952639248	1.73E-06
TUCP_004505	XLOC_198446	ENSRNOT00000084873	0.979612179	2.68E-08
TUCP_004505	XLOC_198446	ENSRNOT00000076407	0.959398392	8.12E-07
TUCP_004505	XLOC_198446	ENSRNOT00000088707	0.959941175	7.59E-07

TUCP_004505	XLOC_198446	ENSRNOT00000005361	0.979009005	3.10E-08
TUCP_004505	XLOC_198446	ENSRNOT00000019501	0.973940225	9.06E-08
TUCP_004505	XLOC_198446	ENSRNOT00000041741	0.955713831	1.25E-06
TUCP_004505	XLOC_198446	ENSRNOT00000034152	0.983007982	1.08E-08
TUCP_004505	XLOC_198446	ENSRNOT00000024718	0.970133921	1.78E-07
TUCP_004505	XLOC_198446	ENSRNOT00000009620	0.977453005	4.42E-08
TUCP_004505	XLOC_198446	ENSRNOT00000019311	0.956269157	1.17E-06
TUCP_004505	XLOC_198446	ENSRNOT00000004637	0.959940741	7.60E-07
TUCP_004505	XLOC_198446	ENSRNOT00000073547	0.959955994	7.58E-07
TUCP_004505	XLOC_198446	ENSRNOT00000067677	0.967930239	2.53E-07
TUCP_004505	XLOC_198446	ENSRNOT00000024464	0.973515985	9.82E-08
TUCP_004505	XLOC_198446	ENSRNOT00000005846	0.964723556	4.05E-07
TUCP_004505	XLOC_198446	ENSRNOT00000043434	0.968599587	2.28E-07
TUCP_004505	XLOC_198446	ENSRNOT00000044788	0.957055689	1.07E-06
TUCP_004505	XLOC_198446	ENSRNOT00000018511	0.970539154	1.66E-07
TUCP_004505	XLOC_198446	ENSRNOT00000051363	0.98252571	1.25E-08
TUCP_004505	XLOC_198446	ENSRNOT00000022648	0.979964135	2.46E-08
TUCP_004505	XLOC_198446	ENSRNOT00000025197	0.954633606	1.40E-06
TUCP_004505	XLOC_198446	ENSRNOT00000029990	0.954684843	1.39E-06
TUCP_004505	XLOC_198446	ENSRNOT00000084021	0.968327802	2.38E-07
TUCP_004505	XLOC_198446	ENSRNOT00000003567	0.970525203	1.67E-07
TUCP_004505	XLOC_198446	ENSRNOT00000016449	0.964184652	4.37E-07
TUCP_004505	XLOC_198446	ENSRNOT00000051349	0.975859272	6.20E-08
TUCP_004505	XLOC_198446	ENSRNOT00000087603	0.956196735	1.18E-06
TUCP_004505	XLOC_198446	ENSRNOT00000086390	0.953249694	1.63E-06
TUCP_004505	XLOC_198446	ENSRNOT00000009313	0.974768069	7.72E-08
TUCP_004505	XLOC_198446	ENSRNOT00000027949	0.955409305	1.29E-06
TUCP_004505	XLOC_198446	ENSRNOT00000023504	0.96495094	3.93E-07
TUCP_004505	XLOC_198446	ENSRNOT00000091506	0.955284943	1.31E-06
TUCP_004505	XLOC_198446	ENSRNOT00000036753	0.967312901	2.78E-07
TUCP_004505	XLOC_198446	ENSRNOT00000025880	0.977393985	4.48E-08
TUCP_004505	XLOC_198446	ENSRNOT00000023152	0.955157578	1.32E-06
TUCP_004505	XLOC_198446	ENSRNOT00000044983	0.968183029	2.43E-07
TUCP_004505	XLOC_198446	ENSRNOT00000014035	0.952215008	1.81E-06
TUCP_004505	XLOC_198446	ENSRNOT00000083943	0.978271917	3.68E-08
TUCP_004505	XLOC_198446	ENSRNOT00000090026	-0.957242061	1.05E-06
TUCP_004505	XLOC_198446	ENSRNOT00000008365	0.976390254	5.55E-08
TUCP_004505	XLOC_198446	ENSRNOT00000031951	0.960091114	7.46E-07
TUCP_004505	XLOC_198446	ENSRNOT00000071133	0.961067334	6.60E-07
TUCP_004505	XLOC_198446	ENSRNOT00000010264	0.977531493	4.34E-08
TUCP_004505	XLOC_198446	ENSRNOT00000010594	0.960110133	7.44E-07
TUCP_004505	XLOC_198446	ENSRNOT00000059660	0.952881901	1.69E-06
TUCP_004505	XLOC_198446	ENSRNOT00000054757	0.971445581	1.43E-07
TUCP_004504	XLOC_198438	ENSRNOT00000074753	0.966465955	3.16E-07
TUCP_004504	XLOC_198438	ENSRNOT00000041904	0.96401122	4.48E-07
TUCP_004504	XLOC_198438	ENSRNOT00000077990	0.96401122	4.48E-07
TUCP_004504	XLOC_198438	ENSRNOT00000038826	0.963652501	4.70E-07
TUCP_004504	XLOC_198438	ENSRNOT00000046842	0.962361688	5.58E-07
TUCP_004504	XLOC_198438	ENSRNOT00000021243	0.967346281	2.77E-07
TUCP_004504	XLOC_198438	ENSRNOT00000060250	0.964100943	4.42E-07
TUCP_004504	XLOC_198438	ENSRNOT00000091934	0.96401122	4.48E-07

TUCP_004504	XLOC_198438	ENSRNOT00000087656	0.963776626	4.62E-07
TUCP_004504	XLOC_198438	ENSRNOT00000046585	0.96932003	2.03E-07
TUCP_004504	XLOC_198438	ENSRNOT00000080794	0.962303228	5.63E-07
TUCP_004504	XLOC_198438	ENSRNOT00000014456	0.952172687	1.82E-06
TUCP_004504	XLOC_198438	ENSRNOT00000031205	0.963052377	5.10E-07
TUCP_004504	XLOC_198438	ENSRNOT00000079260	0.971011152	1.54E-07
TUCP_004504	XLOC_198438	ENSRNOT00000088381	0.969567048	1.95E-07
TUCP_004504	XLOC_198438	ENSRNOT00000011743	0.968150342	2.45E-07
TUCP_004504	XLOC_198438	ENSRNOT00000089929	0.96401122	4.48E-07
TUCP_004504	XLOC_198438	ENSRNOT00000088624	0.96401122	4.48E-07
TUCP_004504	XLOC_198438	ENSRNOT00000015866	0.972206412	1.25E-07
TUCP_004504	XLOC_198438	ENSRNOT00000034907	0.967381162	2.75E-07
TUCP_004504	XLOC_198438	ENSRNOT00000051835	0.96401122	4.48E-07
TUCP_004504	XLOC_198438	ENSRNOT00000079087	0.96401122	4.48E-07
TUCP_004504	XLOC_198438	ENSRNOT00000037206	0.96401122	4.48E-07
TUCP_004504	XLOC_198438	ENSRNOT00000080590	0.962568983	5.43E-07
TUCP_004504	XLOC_198438	ENSRNOT00000027552	0.961466628	6.27E-07
TUCP_004504	XLOC_198438	ENSRNOT00000077014	0.966575279	3.11E-07
TUCP_004504	XLOC_198438	ENSRNOT00000080453	0.962368921	5.58E-07
TUCP_004504	XLOC_198438	ENSRNOT00000057729	0.966303942	3.23E-07
TUCP_004504	XLOC_198438	ENSRNOT00000078633	0.96401122	4.48E-07
TUCP_004504	XLOC_198438	ENSRNOT00000020206	0.960695871	6.91E-07
TUCP_004504	XLOC_198438	ENSRNOT00000066839	0.967361988	2.76E-07
TUCP_004504	XLOC_198438	ENSRNOT00000026932	0.961519885	6.23E-07
TUCP_004504	XLOC_198438	ENSRNOT00000078663	0.972066834	1.28E-07
TUCP_004504	XLOC_198438	ENSRNOT00000037169	0.953739534	1.54E-06
TUCP_004504	XLOC_198438	ENSRNOT00000029634	0.958135894	9.44E-07
TUCP_004504	XLOC_198438	ENSRNOT00000093579	0.963190396	5.00E-07
TUCP_004504	XLOC_198438	ENSRNOT00000086349	0.950740387	2.10E-06
TUCP_004504	XLOC_198438	ENSRNOT00000065460	0.96401122	4.48E-07
TUCP_004504	XLOC_198438	ENSRNOT00000003817	0.95821769	9.35E-07
TUCP_004504	XLOC_198438	ENSRNOT00000016713	0.96401122	4.48E-07
TUCP_004504	XLOC_198438	ENSRNOT00000022559	0.965395375	3.69E-07
TUCP_004504	XLOC_198438	ENSRNOT00000044145	0.96401122	4.48E-07
TUCP_004504	XLOC_198438	ENSRNOT00000039294	0.96401122	4.48E-07
TUCP_004504	XLOC_198438	ENSRNOT00000085654	0.961240962	6.45E-07
TUCP_001870	XLOC_077795	ENSRNOT00000002330	0.953764578	1.54E-06
TUCP_001870	XLOC_077795	ENSRNOT00000072077	0.952932898	1.68E-06
TUCP_001870	XLOC_077795	ENSRNOT00000087346	-0.957409579	1.03E-06
TUCP_001870	XLOC_077795	ENSRNOT00000001577	-0.970723087	1.61E-07
TUCP_001870	XLOC_077795	ENSRNOT00000045545	0.959999314	7.54E-07
TUCP_001870	XLOC_077795	ENSRNOT00000030914	0.952582029	1.74E-06
TUCP_001870	XLOC_077795	ENSRNOT00000027220	0.961726191	6.07E-07
TUCP_001870	XLOC_077795	ENSRNOT00000065464	-0.953357254	1.61E-06
TUCP_001870	XLOC_077795	ENSRNOT00000071192	0.955346739	1.30E-06
TUCP_001870	XLOC_077795	ENSRNOT00000021944	-0.971788677	1.34E-07
TUCP_004506	XLOC_198676	ENSRNOT00000000211	0.969161252	2.09E-07
TUCP_004506	XLOC_198676	ENSRNOT00000061297	0.964085771	4.43E-07
TUCP_004506	XLOC_198676	ENSRNOT00000083410	0.969541731	1.96E-07
TUCP_004501	XLOC_198227	ENSRNOT00000064007	0.962044257	5.82E-07
TUCP_004501	XLOC_198227	ENSRNOT00000006886	0.956617564	1.13E-06

TUCP_004501	XLOC_198227	ENSRNOT00000016880	0.977060086	4.81E-08
TUCP_004501	XLOC_198227	ENSRNOT00000013255	0.957313793	1.04E-06
TUCP_004501	XLOC_198227	ENSRNOT00000009482	0.956392431	1.15E-06
TUCP_004501	XLOC_198227	ENSRNOT00000085690	0.95746582	1.02E-06
TUCP_004500	XLOC_198108	ENSRNOT00000091000	0.951620444	1.92E-06
TUCP_004503	XLOC_198409	ENSRNOT00000024186	0.964520932	4.17E-07
TUCP_004503	XLOC_198409	ENSRNOT00000074753	0.984644596	6.55E-09
TUCP_004503	XLOC_198409	ENSRNOT00000041904	0.984446026	6.98E-09
TUCP_004503	XLOC_198409	ENSRNOT00000083487	0.973735457	9.42E-08
TUCP_004503	XLOC_198409	ENSRNOT00000077990	0.984446026	6.98E-09
TUCP_004503	XLOC_198409	ENSRNOT00000038826	0.982349284	1.31E-08
TUCP_004503	XLOC_198409	ENSRNOT00000046842	0.954493345	1.42E-06
TUCP_004503	XLOC_198409	ENSRNOT00000037169	0.964279713	4.31E-07
TUCP_004503	XLOC_198409	ENSRNOT00000021243	0.999840653	8.09E-19
TUCP_004503	XLOC_198409	ENSRNOT00000060250	0.984178837	7.60E-09
TUCP_004503	XLOC_198409	ENSRNOT00000091934	0.984446026	6.98E-09
TUCP_004503	XLOC_198409	ENSRNOT00000046585	0.964071704	4.44E-07
TUCP_004503	XLOC_198409	ENSRNOT00000080794	0.985576469	4.80E-09
TUCP_004503	XLOC_198409	ENSRNOT00000014456	0.974013065	8.94E-08
TUCP_004503	XLOC_198409	ENSRNOT00000031205	0.972720678	1.14E-07
TUCP_004503	XLOC_198409	ENSRNOT00000044145	0.984446026	6.98E-09
TUCP_004503	XLOC_198409	ENSRNOT00000088381	0.971033062	1.53E-07
TUCP_004503	XLOC_198409	ENSRNOT00000011743	0.968589025	2.28E-07
TUCP_004503	XLOC_198409	ENSRNOT00000089929	0.984446026	6.98E-09
TUCP_004503	XLOC_198409	ENSRNOT00000088624	0.984446026	6.98E-09
TUCP_004503	XLOC_198409	ENSRNOT00000085659	0.972021993	1.29E-07
TUCP_004503	XLOC_198409	ENSRNOT00000015866	0.982374336	1.30E-08
TUCP_004503	XLOC_198409	ENSRNOT00000064613	0.960213031	7.34E-07
TUCP_004503	XLOC_198409	ENSRNOT00000034907	0.97564574	6.48E-08
TUCP_004503	XLOC_198409	ENSRNOT00000051835	0.984446026	6.98E-09
TUCP_004503	XLOC_198409	ENSRNOT00000009825	0.9584932	9.05E-07
TUCP_004503	XLOC_198409	ENSRNOT00000079087	0.984446026	6.98E-09
TUCP_004503	XLOC_198409	ENSRNOT00000037206	0.984446026	6.98E-09
TUCP_004503	XLOC_198409	ENSRNOT00000061185	0.966990753	2.92E-07
TUCP_004503	XLOC_198409	ENSRNOT00000083261	0.951038217	2.04E-06
TUCP_004503	XLOC_198409	ENSRNOT00000080590	0.98407546	7.85E-09
TUCP_004503	XLOC_198409	ENSRNOT00000027552	0.983018866	1.08E-08
TUCP_004503	XLOC_198409	ENSRNOT00000072138	0.952691887	1.72E-06
TUCP_004503	XLOC_198409	ENSRNOT00000077014	0.977072379	4.80E-08
TUCP_004503	XLOC_198409	ENSRNOT00000080453	0.983860432	8.39E-09
TUCP_004503	XLOC_198409	ENSRNOT00000057729	0.983223316	1.02E-08
TUCP_004503	XLOC_198409	ENSRNOT00000078633	0.984446026	6.98E-09
TUCP_004503	XLOC_198409	ENSRNOT00000013423	0.958186668	9.38E-07
TUCP_004503	XLOC_198409	ENSRNOT00000071884	0.957312305	1.04E-06
TUCP_004503	XLOC_198409	ENSRNOT00000066839	0.984798621	6.23E-09
TUCP_004503	XLOC_198409	ENSRNOT00000026316	0.959298706	8.21E-07
TUCP_004503	XLOC_198409	ENSRNOT00000078663	0.979939693	2.47E-08
TUCP_004503	XLOC_198409	ENSRNOT00000064250	0.963147355	5.03E-07
TUCP_004503	XLOC_198409	ENSRNOT00000021608	0.957368466	1.03E-06
TUCP_004503	XLOC_198409	ENSRNOT00000029634	0.979586859	2.70E-08
TUCP_004503	XLOC_198409	ENSRNOT00000065460	0.984446026	6.98E-09

TUCP_004503	XLOC_198409	ENSRNOT00000003817	0.979209924	2.95E-08
TUCP_004503	XLOC_198409	ENSRNOT00000016713	0.984446026	6.98E-09
TUCP_004503	XLOC_198409	ENSRNOT00000079260	0.978121755	3.81E-08
TUCP_004503	XLOC_198409	ENSRNOT00000039294	0.984446026	6.98E-09
TUCP_004503	XLOC_198409	ENSRNOT00000026932	0.979904415	2.50E-08
TUCP_001875	XLOC_077907	ENSRNOT00000049956	0.966486822	3.15E-07
TUCP_001875	XLOC_077907	ENSRNOT00000061253	0.95441066	1.44E-06
TUCP_001875	XLOC_077907	ENSRNOT00000088353	-0.955260158	1.31E-06
TUCP_001875	XLOC_077907	ENSRNOT00000012966	0.955984809	1.21E-06
TUCP_004134	XLOC_180414	ENSRNOT00000085599	0.999938502	6.93E-21
TUCP_004134	XLOC_180414	ENSRNOT00000006689	0.9944189	4.22E-11
TUCP_004134	XLOC_180414	ENSRNOT00000081534	0.987999291	1.92E-09
TUCP_004134	XLOC_180414	ENSRNOT00000085301	0.99793679	2.93E-13
TUCP_004134	XLOC_180414	ENSRNOT00000038729	1	1.33E-79
TUCP_004134	XLOC_180414	ENSRNOT00000086385	1	1.33E-79
TUCP_004134	XLOC_180414	ENSRNOT00000077695	0.96038616	7.19E-07
TUCP_004134	XLOC_180414	ENSRNOT00000078561	0.986503348	3.45E-09
TUCP_004134	XLOC_180414	ENSRNOT00000087613	1	1.33E-79
TUCP_004134	XLOC_180414	ENSRNOT00000026032	0.953050675	1.66E-06
TUCP_004134	XLOC_180414	ENSRNOT00000073350	0.9658754	3.44E-07
TUCP_004134	XLOC_180414	ENSRNOT00000023062	1	1.33E-79
TUCP_004134	XLOC_180414	ENSRNOT00000058834	1	4.25E-78
TUCP_004134	XLOC_180414	ENSRNOT00000079420	1	0
TUCP_004134	XLOC_180414	ENSRNOT00000090761	1	0
TUCP_004134	XLOC_180414	ENSRNOT00000055923	1	0
TUCP_004134	XLOC_180414	ENSRNOT00000092416	0.963085379	5.07E-07
TUCP_004134	XLOC_180414	ENSRNOT00000071930	1	0
TUCP_004134	XLOC_180414	ENSRNOT00000001996	0.974721715	7.79E-08
TUCP_004134	XLOC_180414	ENSRNOT00000076756	1	0
TUCP_004134	XLOC_180414	ENSRNOT00000081527	0.999420479	5.14E-16
TUCP_004134	XLOC_180414	ENSRNOT00000085067	0.960262084	7.30E-07
TUCP_004134	XLOC_180414	ENSRNOT00000091663	0.99642667	4.56E-12
TUCP_004134	XLOC_180414	ENSRNOT00000081780	1	0
TUCP_004134	XLOC_180414	ENSRNOT00000093689	0.953599805	1.57E-06
TUCP_004134	XLOC_180414	ENSRNOT00000089114	1	0
TUCP_004134	XLOC_180414	ENSRNOT00000021609	0.977017895	4.86E-08
TUCP_004134	XLOC_180414	ENSRNOT00000073316	1	0
TUCP_004134	XLOC_180414	ENSRNOT00000086557	0.977413455	4.46E-08
TUCP_004134	XLOC_180414	ENSRNOT00000071663	1	0
TUCP_004134	XLOC_180414	ENSRNOT00000042860	1	1.33E-79
TUCP_004134	XLOC_180414	ENSRNOT00000072962	0.971287317	1.46E-07
TUCP_004134	XLOC_180414	ENSRNOT00000088421	1	0
TUCP_004134	XLOC_180414	ENSRNOT00000038898	1	0
TUCP_004134	XLOC_180414	ENSRNOT00000015840	0.959452012	8.06E-07
TUCP_004134	XLOC_180414	ENSRNOT00000064936	0.985957557	4.20E-09
TUCP_004134	XLOC_180414	ENSRNOT00000042777	0.990642579	5.56E-10
TUCP_004134	XLOC_180414	ENSRNOT00000010029	1	0
TUCP_001971	XLOC_079682	ENSRNOT00000000008	0.997147692	1.48E-12
TUCP_001971	XLOC_079682	ENSRNOT00000060048	0.996022704	7.79E-12
TUCP_001971	XLOC_079682	ENSRNOT00000082304	0.993436469	9.49E-11
TUCP_001971	XLOC_079682	ENSRNOT00000006219	0.997147692	1.48E-12

TUCP_001971	XLOC_079682	ENSRNOT00000065376	0.997147692	1.48E-12
TUCP_001971	XLOC_079682	ENSRNOT00000081433	0.980336195	2.24E-08
TUCP_001971	XLOC_079682	ENSRNOT00000083575	0.997147692	1.48E-12
TUCP_001971	XLOC_079682	ENSRNOT00000087739	0.997147692	1.48E-12
TUCP_001971	XLOC_079682	ENSRNOT00000079826	0.997147692	1.48E-12
TUCP_001971	XLOC_079682	ENSRNOT00000025770	0.990537542	5.88E-10
TUCP_001971	XLOC_079682	ENSRNOT00000088244	0.973215814	1.04E-07
TUCP_001971	XLOC_079682	ENSRNOT00000048523	0.997147692	1.48E-12
TUCP_001971	XLOC_079682	ENSRNOT00000079365	0.976834515	5.05E-08
TUCP_001971	XLOC_079682	ENSRNOT00000020229	0.950713381	2.11E-06
TUCP_001971	XLOC_079682	ENSRNOT00000026428	0.99307183	1.24E-10
TUCP_001971	XLOC_079682	ENSRNOT00000085673	0.997147692	1.48E-12
TUCP_001971	XLOC_079682	ENSRNOT00000072972	0.975473144	6.71E-08
TUCP_001971	XLOC_079682	ENSRNOT00000050573	0.997147692	1.48E-12
TUCP_001971	XLOC_079682	ENSRNOT00000074378	0.995788134	1.04E-11
TUCP_001971	XLOC_079682	ENSRNOT00000093571	0.997147692	1.48E-12
TUCP_001971	XLOC_079682	ENSRNOT00000090581	0.997147692	1.48E-12
TUCP_001971	XLOC_079682	ENSRNOT00000076239	0.972541127	1.17E-07
TUCP_001971	XLOC_079682	ENSRNOT00000065485	0.997147692	1.48E-12
TUCP_001971	XLOC_079682	ENSRNOT00000006356	0.995963698	8.38E-12
TUCP_001971	XLOC_079682	ENSRNOT00000087199	0.969946069	1.84E-07
TUCP_001971	XLOC_079682	ENSRNOT00000022091	0.997147692	1.48E-12
TUCP_001971	XLOC_079682	ENSRNOT00000064764	0.979772159	2.58E-08
TUCP_001971	XLOC_079682	ENSRNOT00000076927	0.966832923	2.99E-07
TUCP_001971	XLOC_079682	ENSRNOT00000092395	0.996438934	4.48E-12
TUCP_004137	XLOC_180712	ENSRNOT00000027611	0.956833054	1.10E-06
TUCP_004137	XLOC_180712	ENSRNOT00000081246	0.968168454	2.44E-07
TUCP_004137	XLOC_180712	ENSRNOT00000009877	0.95127198	1.99E-06
TUCP_004137	XLOC_180712	ENSRNOT00000007351	-0.952627095	1.73E-06
TUCP_004137	XLOC_180712	ENSRNOT00000074939	0.973615773	9.63E-08
TUCP_004137	XLOC_180712	ENSRNOT00000021614	0.953503235	1.58E-06
TUCP_004137	XLOC_180712	ENSRNOT00000034146	0.956248378	1.17E-06
TUCP_004137	XLOC_180712	ENSRNOT00000021116	0.968581777	2.29E-07
TUCP_004137	XLOC_180712	ENSRNOT00000023823	0.956593334	1.13E-06
TUCP_004137	XLOC_180712	ENSRNOT00000084601	0.979773176	2.58E-08
TUCP_004137	XLOC_180712	ENSRNOT00000043216	0.966663786	3.07E-07
TUCP_004137	XLOC_180712	ENSRNOT00000044742	0.954442694	1.43E-06
TUCP_004137	XLOC_180712	ENSRNOT00000027414	0.960651219	6.95E-07
TUCP_004137	XLOC_180712	ENSRNOT00000028563	0.965828206	3.46E-07
TUCP_004137	XLOC_180712	ENSRNOT00000022800	0.953385518	1.60E-06
TUCP_004137	XLOC_180712	ENSRNOT00000066670	0.973183797	1.04E-07
TUCP_004137	XLOC_180712	ENSRNOT00000071971	0.951533907	1.94E-06
TUCP_004137	XLOC_180712	ENSRNOT00000015073	0.956006321	1.21E-06
TUCP_004137	XLOC_180712	ENSRNOT00000005942	0.950775701	2.09E-06
TUCP_004137	XLOC_180712	ENSRNOT00000012379	0.964515098	4.17E-07
TUCP_004137	XLOC_180712	ENSRNOT00000029866	0.953676576	1.55E-06
TUCP_004137	XLOC_180712	ENSRNOT00000080465	0.97060042	1.65E-07
TUCP_004137	XLOC_180712	ENSRNOT00000006963	0.95285215	1.69E-06
TUCP_004137	XLOC_180712	ENSRNOT00000071764	0.966661921	3.07E-07
TUCP_004137	XLOC_180712	ENSRNOT00000082699	0.952415715	1.77E-06
TUCP_004137	XLOC_180712	ENSRNOT00000021348	0.964608291	4.12E-07

TUCP_004137	XLOC_180712	ENSRNOT00000032944	0.97426506	8.51E-08
TUCP_004137	XLOC_180712	ENSRNOT00000083215	0.964900654	3.96E-07
TUCP_004137	XLOC_180712	ENSRNOT00000055187	0.960233705	7.33E-07
TUCP_004137	XLOC_180712	ENSRNOT00000001809	0.975489106	6.69E-08
TUCP_004137	XLOC_180712	ENSRNOT00000079866	0.954138131	1.48E-06
TUCP_004137	XLOC_180712	ENSRNOT00000034267	0.951122779	2.02E-06
TUCP_004137	XLOC_180712	ENSRNOT00000032274	0.969825902	1.87E-07
TUCP_004137	XLOC_180712	ENSRNOT00000010149	0.956609923	1.13E-06
TUCP_004137	XLOC_180712	ENSRNOT00000008018	0.964545887	4.16E-07
TUCP_004137	XLOC_180712	ENSRNOT00000004032	0.952922689	1.68E-06
TUCP_004137	XLOC_180712	ENSRNOT00000003991	0.963250855	4.96E-07
TUCP_004137	XLOC_180712	ENSRNOT00000017948	0.972663648	1.15E-07
TUCP_004137	XLOC_180712	ENSRNOT00000012895	0.969881436	1.86E-07
TUCP_004137	XLOC_180712	ENSRNOT00000055401	0.979009882	3.10E-08
TUCP_004137	XLOC_180712	ENSRNOT00000072196	0.952821008	1.70E-06
TUCP_004137	XLOC_180712	ENSRNOT00000039852	0.986694044	3.21E-09
TUCP_004137	XLOC_180712	ENSRNOT00000023180	0.955650042	1.25E-06
TUCP_004137	XLOC_180712	ENSRNOT00000000676	0.963759904	4.63E-07
TUCP_004137	XLOC_180712	ENSRNOT00000070815	0.972940913	1.09E-07
TUCP_004137	XLOC_180712	ENSRNOT00000028070	0.951368117	1.97E-06
TUCP_004137	XLOC_180712	ENSRNOT00000004359	0.95042984	2.17E-06
TUCP_004137	XLOC_180712	ENSRNOT00000013274	0.965392292	3.69E-07
TUCP_004137	XLOC_180712	ENSRNOT00000022610	0.966336988	3.22E-07
TUCP_004137	XLOC_180712	ENSRNOT00000020437	0.97453166	8.09E-08
TUCP_004137	XLOC_180712	ENSRNOT00000051720	0.95604802	1.20E-06
TUCP_004137	XLOC_180712	ENSRNOT00000010898	0.960375527	7.20E-07
TUCP_004137	XLOC_180712	ENSRNOT00000000157	0.971507261	1.41E-07
TUCP_004137	XLOC_180712	ENSRNOT00000009430	0.966096974	3.33E-07
TUCP_004137	XLOC_180712	ENSRNOT00000029167	0.97482847	7.63E-08
TUCP_004137	XLOC_180712	ENSRNOT00000029419	0.955282426	1.31E-06
TUCP_004137	XLOC_180712	ENSRNOT00000027520	0.9549672	1.35E-06
TUCP_004137	XLOC_180712	ENSRNOT00000047387	0.951701496	1.91E-06
TUCP_004137	XLOC_180712	ENSRNOT00000013661	0.960624295	6.98E-07
TUCP_004137	XLOC_180712	ENSRNOT00000058072	0.977200073	4.67E-08
TUCP_004137	XLOC_180712	ENSRNOT00000017629	0.960100403	7.45E-07
TUCP_004137	XLOC_180712	ENSRNOT00000022839	0.956785571	1.10E-06
TUCP_004137	XLOC_180712	ENSRNOT00000004508	0.971520318	1.41E-07
TUCP_004137	XLOC_180712	ENSRNOT00000034744	0.951567228	1.93E-06
TUCP_004137	XLOC_180712	ENSRNOT00000009440	-0.954833354	1.37E-06
TUCP_004137	XLOC_180712	ENSRNOT00000009683	0.955712634	1.25E-06
TUCP_004137	XLOC_180712	ENSRNOT00000021153	0.952347247	1.79E-06
TUCP_004137	XLOC_180712	ENSRNOT00000005262	0.95539809	1.29E-06
TUCP_004137	XLOC_180712	ENSRNOT00000024322	0.954549586	1.41E-06
TUCP_004137	XLOC_180712	ENSRNOT00000063956	0.950570602	2.14E-06
TUCP_004137	XLOC_180712	ENSRNOT00000081897	0.953045463	1.66E-06
TUCP_004137	XLOC_180712	ENSRNOT00000074075	0.953185575	1.64E-06
TUCP_004137	XLOC_180712	ENSRNOT00000039631	0.968774359	2.22E-07
TUCP_004137	XLOC_180712	ENSRNOT00000025832	0.965119683	3.83E-07
TUCP_004137	XLOC_180712	ENSRNOT00000076729	0.967578232	2.67E-07
TUCP_004137	XLOC_180712	ENSRNOT00000017753	0.950496671	2.15E-06
TUCP_004137	XLOC_180712	ENSRNOT00000000713	0.96793041	2.53E-07

TUCP_004137	XLOC_180712	ENSRNOT00000045882	0.955851191	1.23E-06
TUCP_004137	XLOC_180712	ENSRNOT00000066736	0.98308423	1.06E-08
TUCP_004137	XLOC_180712	ENSRNOT00000009198	0.961960958	5.88E-07
TUCP_004137	XLOC_180712	ENSRNOT00000027054	0.955609212	1.26E-06
TUCP_004137	XLOC_180712	ENSRNOT00000009129	0.97025303	1.75E-07
TUCP_004137	XLOC_180712	ENSRNOT00000072081	0.961098758	6.57E-07
TUCP_004137	XLOC_180712	ENSRNOT00000072534	0.964504186	4.18E-07
TUCP_004137	XLOC_180712	ENSRNOT00000036951	0.962128364	5.76E-07
TUCP_004137	XLOC_180712	ENSRNOT00000073991	0.957369213	1.03E-06
TUCP_004137	XLOC_180712	ENSRNOT00000091265	0.957264189	1.04E-06
TUCP_004137	XLOC_180712	ENSRNOT00000006800	0.966181932	3.29E-07
TUCP_004137	XLOC_180712	ENSRNOT00000044376	0.952474507	1.76E-06
TUCP_004137	XLOC_180712	ENSRNOT00000092158	0.957360547	1.03E-06
TUCP_004137	XLOC_180712	ENSRNOT00000023645	0.964271322	4.32E-07
TUCP_004137	XLOC_180712	ENSRNOT00000084734	0.960805451	6.82E-07
TUCP_004137	XLOC_180712	ENSRNOT00000010992	0.952401551	1.78E-06
TUCP_004137	XLOC_180712	ENSRNOT00000076669	0.964193894	4.36E-07
TUCP_004137	XLOC_180712	ENSRNOT00000085596	0.952679027	1.73E-06
TUCP_004137	XLOC_180712	ENSRNOT00000066526	0.968790938	2.21E-07
TUCP_004137	XLOC_180712	ENSRNOT00000031230	0.971980931	1.30E-07
TUCP_004137	XLOC_180712	ENSRNOT00000071733	0.954426862	1.43E-06
TUCP_004137	XLOC_180712	ENSRNOT00000002292	0.956478497	1.14E-06
TUCP_004137	XLOC_180712	ENSRNOT00000086336	0.964265878	4.32E-07
TUCP_004137	XLOC_180712	ENSRNOT00000023490	0.971140129	1.50E-07
TUCP_004137	XLOC_180712	ENSRNOT00000041384	-0.95113168	2.02E-06
TUCP_004137	XLOC_180712	ENSRNOT00000071697	0.951542018	1.94E-06
TUCP_004137	XLOC_180712	ENSRNOT00000004412	0.971875039	1.32E-07
TUCP_004137	XLOC_180712	ENSRNOT00000064798	0.954424105	1.43E-06
TUCP_004137	XLOC_180712	ENSRNOT00000027452	0.966649586	3.07E-07
TUCP_004137	XLOC_180712	ENSRNOT00000034704	0.982336049	1.31E-08
TUCP_004137	XLOC_180712	ENSRNOT00000082174	0.95241491	1.77E-06
TUCP_004137	XLOC_180712	ENSRNOT00000013985	0.968612699	2.28E-07
TUCP_004137	XLOC_180712	ENSRNOT00000036750	0.968070992	2.48E-07
TUCP_004137	XLOC_180712	ENSRNOT00000060669	0.959405119	8.11E-07
TUCP_004137	XLOC_180712	ENSRNOT00000023428	0.957869387	9.74E-07
TUCP_004137	XLOC_180712	ENSRNOT00000024420	0.958344551	9.21E-07
TUCP_004137	XLOC_180712	ENSRNOT00000049538	0.972189997	1.25E-07
TUCP_004137	XLOC_180712	ENSRNOT00000029001	0.961594728	6.17E-07
TUCP_004137	XLOC_180712	ENSRNOT00000083799	0.957527326	1.01E-06
TUCP_004137	XLOC_180712	ENSRNOT00000009066	0.959968697	7.57E-07
TUCP_004130	XLOC_180298	ENSRNOT00000033018	0.952084318	1.84E-06
TUCP_004508	XLOC_198778	ENSRNOT00000084500	0.967784772	2.59E-07
TUCP_004508	XLOC_198778	ENSRNOT00000071877	0.959275266	8.24E-07
TUCP_004508	XLOC_198778	ENSRNOT00000077972	0.953393672	1.60E-06
TUCP_004508	XLOC_198778	ENSRNOT00000052315	0.973708288	9.47E-08
TUCP_004508	XLOC_198778	ENSRNOT00000052308	0.965931678	3.41E-07
TUCP_004508	XLOC_198778	ENSRNOT00000048858	0.976247106	5.72E-08
TUCP_004508	XLOC_198778	ENSRNOT00000047241	0.96682242	2.99E-07
TUCP_004508	XLOC_198778	ENSRNOT00000043661	0.966651612	3.07E-07
TUCP_002598	XLOC_109621	ENSRNOT00000074990	0.988969567	1.26E-09
TUCP_002598	XLOC_109621	ENSRNOT00000068444	0.988969567	1.26E-09

TUCP_002598	XLOC_109621	ENSRNOT00000081370	0.989350739	1.06E-09
TUCP_002598	XLOC_109621	ENSRNOT00000078645	0.989802454	8.54E-10
TUCP_002598	XLOC_109621	ENSRNOT00000075987	0.987003394	2.86E-09
TUCP_002598	XLOC_109621	ENSRNOT00000082043	0.986928169	2.94E-09
TUCP_002598	XLOC_109621	ENSRNOT00000087323	0.981708157	1.56E-08
TUCP_002598	XLOC_109621	ENSRNOT00000074930	0.988969567	1.26E-09
TUCP_002598	XLOC_109621	ENSRNOT00000080575	0.989028565	1.23E-09
TUCP_002598	XLOC_109621	ENSRNOT00000057761	0.975412589	6.79E-08
TUCP_002598	XLOC_109621	ENSRNOT00000078369	0.983676245	8.88E-09
TUCP_002598	XLOC_109621	ENSRNOT00000092982	0.988969567	1.26E-09
TUCP_002598	XLOC_109621	ENSRNOT00000088367	0.988969567	1.26E-09
TUCP_002598	XLOC_109621	ENSRNOT00000079606	0.966198332	3.28E-07
TUCP_002598	XLOC_109621	ENSRNOT00000081996	0.988969567	1.26E-09
TUCP_002598	XLOC_109621	ENSRNOT00000064751	0.956445005	1.15E-06
TUCP_002598	XLOC_109621	ENSRNOT00000022280	0.98954896	9.65E-10
TUCP_002598	XLOC_109621	ENSRNOT00000082058	0.988969567	1.26E-09
TUCP_002598	XLOC_109621	ENSRNOT00000088905	0.988969567	1.26E-09
TUCP_002598	XLOC_109621	ENSRNOT00000086831	0.988969567	1.26E-09
TUCP_002598	XLOC_109621	ENSRNOT00000045099	0.988969567	1.26E-09
TUCP_002598	XLOC_109621	ENSRNOT00000086745	0.968313659	2.39E-07
TUCP_002598	XLOC_109621	ENSRNOT00000085849	0.9852386	5.38E-09
TUCP_002598	XLOC_109621	ENSRNOT00000077789	0.974510293	8.12E-08
TUCP_002598	XLOC_109621	ENSRNOT00000073828	0.965143232	3.82E-07
TUCP_002598	XLOC_109621	ENSRNOT00000087025	0.988969567	1.26E-09
TUCP_002598	XLOC_109621	ENSRNOT00000019853	0.988969567	1.26E-09
TUCP_002598	XLOC_109621	ENSRNOT00000073853	0.981147582	1.82E-08
TUCP_002598	XLOC_109621	ENSRNOT00000065912	0.959008008	8.51E-07
TUCP_002598	XLOC_109621	ENSRNOT00000093083	0.987712097	2.16E-09
TUCP_003897	XLOC_171339	ENSRNOT00000003511	0.973889137	9.15E-08
TUCP_003897	XLOC_171339	ENSRNOT00000005505	0.964042344	4.46E-07
TUCP_003897	XLOC_171339	ENSRNOT00000064070	0.952237951	1.81E-06
TUCP_003897	XLOC_171339	ENSRNOT00000000697	0.957021402	1.07E-06
TUCP_003897	XLOC_171339	ENSRNOT00000023623	0.978007116	3.91E-08
TUCP_003897	XLOC_171339	ENSRNOT00000016390	0.951497398	1.95E-06
TUCP_003897	XLOC_171339	ENSRNOT00000023607	0.9520885	1.83E-06
TUCP_003897	XLOC_171339	ENSRNOT00000087937	0.954741316	1.39E-06
TUCP_003897	XLOC_171339	ENSRNOT00000047453	0.97134576	1.45E-07
TUCP_003897	XLOC_171339	ENSRNOT00000072682	0.97898569	3.12E-08
TUCP_003897	XLOC_171339	ENSRNOT00000009975	0.956562771	1.13E-06
TUCP_003897	XLOC_171339	ENSRNOT00000010701	0.972355979	1.21E-07
TUCP_003897	XLOC_171339	ENSRNOT00000023661	0.970720366	1.61E-07
TUCP_003897	XLOC_171339	ENSRNOT00000002905	0.956729693	1.11E-06
TUCP_003897	XLOC_171339	ENSRNOT00000041762	0.986120228	3.96E-09
TUCP_003897	XLOC_171339	ENSRNOT00000064122	0.958304035	9.25E-07
TUCP_003897	XLOC_171339	ENSRNOT00000006697	0.962233384	5.68E-07
TUCP_001773	XLOC_073332	ENSRNOT00000090449	0.957789323	9.83E-07
TUCP_001782	XLOC_073522	ENSRNOT00000090350	0.956948908	1.08E-06
TUCP_001782	XLOC_073522	ENSRNOT00000088306	0.962751647	5.30E-07
TUCP_002596	XLOC_109621	ENSRNOT00000004876	0.961120126	6.55E-07
TUCP_002596	XLOC_109621	ENSRNOT00000061139	0.955434059	1.28E-06
TUCP_002597	XLOC_109621	ENSRNOT00000048498	0.979343473	2.86E-08

TUCP_002597	XLOC_109621	ENSRNOT00000072238	0.992713305	1.60E-10
TUCP_002597	XLOC_109621	ENSRNOT00000033379	0.967678135	2.63E-07
TUCP_002597	XLOC_109621	ENSRNOT00000057051	0.988809994	1.36E-09
TUCP_002597	XLOC_109621	ENSRNOT00000004279	0.955084175	1.33E-06
TUCP_002597	XLOC_109621	ENSRNOT00000046837	0.953061804	1.66E-06
TUCP_003834	XLOC_167932	ENSRNOT00000009666	0.955823606	1.23E-06
TUCP_003834	XLOC_167932	ENSRNOT00000013863	0.954291451	1.45E-06
TUCP_003834	XLOC_167932	ENSRNOT00000025846	0.970608931	1.64E-07
TUCP_003834	XLOC_167932	ENSRNOT00000006549	0.955764761	1.24E-06
TUCP_003834	XLOC_167932	ENSRNOT00000058116	0.958695846	8.83E-07
TUCP_003834	XLOC_167932	ENSRNOT00000006402	0.952634663	1.73E-06
TUCP_003834	XLOC_167932	ENSRNOT00000027083	-0.962631899	5.39E-07
TUCP_003834	XLOC_167932	ENSRNOT00000048914	-0.959841474	7.69E-07
TUCP_003834	XLOC_167932	ENSRNOT00000079633	0.952014272	1.85E-06
TUCP_003834	XLOC_167932	ENSRNOT00000025391	-0.970934095	1.56E-07
TUCP_003834	XLOC_167932	ENSRNOT00000005262	-0.954783868	1.38E-06
TUCP_003834	XLOC_167932	ENSRNOT00000021471	0.953233615	1.63E-06
TUCP_003834	XLOC_167932	ENSRNOT00000015566	-0.953200608	1.63E-06
TUCP_003834	XLOC_167932	ENSRNOT00000022055	0.953688807	1.55E-06
TUCP_003834	XLOC_167932	ENSRNOT00000023326	-0.955666662	1.25E-06
TUCP_003834	XLOC_167932	ENSRNOT00000016351	0.952212422	1.81E-06
TUCP_003834	XLOC_167932	ENSRNOT00000013026	-0.956977215	1.08E-06
TUCP_003834	XLOC_167932	ENSRNOT00000008372	-0.968625526	2.27E-07
TUCP_003834	XLOC_167932	ENSRNOT00000059524	0.950097624	2.24E-06
TUCP_003834	XLOC_167932	ENSRNOT00000064160	-0.951030611	2.04E-06
TUCP_003834	XLOC_167932	ENSRNOT00000017476	0.967362175	2.76E-07
TUCP_003834	XLOC_167932	ENSRNOT00000002291	-0.952377274	1.78E-06
TUCP_003834	XLOC_167932	ENSRNOT00000012635	0.95553153	1.27E-06
TUCP_003834	XLOC_167932	ENSRNOT00000029664	-0.952077133	1.84E-06
TUCP_003834	XLOC_167932	ENSRNOT00000002715	0.956686251	1.12E-06
TUCP_003834	XLOC_167932	ENSRNOT00000078077	-0.963404426	4.86E-07
TUCP_003834	XLOC_167932	ENSRNOT00000025550	0.987116428	2.74E-09
TUCP_003834	XLOC_167932	ENSRNOT00000025196	0.95164723	1.92E-06
TUCP_003834	XLOC_167932	ENSRNOT00000058852	0.967084112	2.88E-07
TUCP_003834	XLOC_167932	ENSRNOT00000092037	0.952562384	1.75E-06
TUCP_003834	XLOC_167932	ENSRNOT00000018003	-0.957380535	1.03E-06
TUCP_003834	XLOC_167932	ENSRNOT00000001468	0.971463157	1.42E-07
TUCP_003834	XLOC_167932	ENSRNOT00000021073	0.967716375	2.62E-07
TUCP_003834	XLOC_167932	ENSRNOT00000078390	0.95753814	1.01E-06
TUCP_003834	XLOC_167932	ENSRNOT00000008464	0.971757092	1.35E-07
TUCP_003834	XLOC_167932	ENSRNOT00000034503	-0.957581671	1.01E-06
TUCP_003834	XLOC_167932	ENSRNOT00000019630	-0.959126989	8.39E-07
TUCP_003834	XLOC_167932	ENSRNOT00000003158	0.97037025	1.71E-07
TUCP_003834	XLOC_167932	ENSRNOT00000055251	-0.950747069	2.10E-06
TUCP_003834	XLOC_167932	ENSRNOT00000020322	-0.950551382	2.14E-06
TUCP_003834	XLOC_167932	ENSRNOT00000081400	-0.967847678	2.56E-07
TUCP_003834	XLOC_167932	ENSRNOT00000084595	-0.951096416	2.03E-06
TUCP_003834	XLOC_167932	ENSRNOT00000007002	0.952990149	1.67E-06
TUCP_003834	XLOC_167932	ENSRNOT00000005894	-0.950461256	2.16E-06
TUCP_003834	XLOC_167932	ENSRNOT00000025936	0.976635283	5.27E-08
TUCP_003834	XLOC_167932	ENSRNOT00000086812	-0.956395948	1.15E-06

TUCP_003834	XLOC_167932	ENSRNOT00000011682	0.969596017	1.94E-07
TUCP_003834	XLOC_167932	ENSRNOT00000027121	-0.954012191	1.50E-06
TUCP_003834	XLOC_167932	ENSRNOT00000051496	-0.958356656	9.20E-07
TUCP_003834	XLOC_167932	ENSRNOT00000014749	-0.968699186	2.25E-07
TUCP_003834	XLOC_167932	ENSRNOT00000010898	-0.953912856	1.52E-06
TUCP_003834	XLOC_167932	ENSRNOT00000056608	0.969340439	2.03E-07
TUCP_003834	XLOC_167932	ENSRNOT00000009247	0.951063627	2.04E-06
TUCP_003834	XLOC_167932	ENSRNOT00000006900	0.968239577	2.41E-07
TUCP_003834	XLOC_167932	ENSRNOT00000082699	-0.964521637	4.17E-07
TUCP_003834	XLOC_167932	ENSRNOT00000031549	-0.958319396	9.24E-07
TUCP_003834	XLOC_167932	ENSRNOT00000090420	0.954853927	1.37E-06
TUCP_003834	XLOC_167932	ENSRNOT00000021592	-0.972431014	1.20E-07
TUCP_003834	XLOC_167932	ENSRNOT00000017422	0.956681831	1.12E-06
TUCP_003834	XLOC_167932	ENSRNOT00000019886	-0.955962588	1.21E-06
TUCP_003834	XLOC_167932	ENSRNOT00000078589	0.958520272	9.02E-07
TUCP_003834	XLOC_167932	ENSRNOT00000081881	-0.967784445	2.59E-07
TUCP_003834	XLOC_167932	ENSRNOT00000091065	-0.955983696	1.21E-06
TUCP_003834	XLOC_167932	ENSRNOT00000028625	0.978086522	3.84E-08
TUCP_003834	XLOC_167932	ENSRNOT00000078685	-0.964445115	4.22E-07
TUCP_003835	XLOC_167971	ENSRNOT00000039045	0.955515839	1.27E-06
TUCP_003835	XLOC_167971	ENSRNOT00000013620	0.95539731	1.29E-06
TUCP_003835	XLOC_167971	ENSRNOT00000000713	0.950379058	2.18E-06
TUCP_003835	XLOC_167971	ENSRNOT00000009979	0.963578573	4.75E-07
TUCP_003835	XLOC_167971	ENSRNOT00000081246	0.969712643	1.91E-07
TUCP_003835	XLOC_167971	ENSRNOT00000009877	0.988619816	1.47E-09
TUCP_003835	XLOC_167971	ENSRNOT00000007351	-0.953917191	1.51E-06
TUCP_003835	XLOC_167971	ENSRNOT00000017668	0.956752272	1.11E-06
TUCP_003835	XLOC_167971	ENSRNOT00000021614	0.977195244	4.68E-08
TUCP_003835	XLOC_167971	ENSRNOT00000026065	0.9636118	4.73E-07
TUCP_003835	XLOC_167971	ENSRNOT00000005228	0.954113532	1.48E-06
TUCP_003835	XLOC_167971	ENSRNOT00000022726	0.975789127	6.29E-08
TUCP_003835	XLOC_167971	ENSRNOT00000005659	0.953780349	1.54E-06
TUCP_003835	XLOC_167971	ENSRNOT00000027526	0.952932291	1.68E-06
TUCP_003835	XLOC_167971	ENSRNOT00000086548	0.968460961	2.33E-07
TUCP_003835	XLOC_167971	ENSRNOT00000019502	0.957178115	1.06E-06
TUCP_003835	XLOC_167971	ENSRNOT00000090342	0.959496749	8.02E-07
TUCP_003835	XLOC_167971	ENSRNOT00000017968	0.950632929	2.13E-06
TUCP_003835	XLOC_167971	ENSRNOT00000038703	0.965600601	3.58E-07
TUCP_003835	XLOC_167971	ENSRNOT00000043216	0.977621488	4.26E-08
TUCP_003835	XLOC_167971	ENSRNOT00000081981	0.9520775	1.84E-06
TUCP_003835	XLOC_167971	ENSRNOT00000009496	0.966468203	3.16E-07
TUCP_003835	XLOC_167971	ENSRNOT00000065383	0.962872629	5.22E-07
TUCP_003835	XLOC_167971	ENSRNOT00000027414	0.981639469	1.59E-08
TUCP_003835	XLOC_167971	ENSRNOT00000028563	0.990665557	5.49E-10
TUCP_003835	XLOC_167971	ENSRNOT00000083369	0.968747918	2.23E-07
TUCP_003835	XLOC_167971	ENSRNOT00000003158	-0.954535202	1.42E-06
TUCP_003835	XLOC_167971	ENSRNOT00000076516	0.968084534	2.47E-07
TUCP_003835	XLOC_167971	ENSRNOT00000015073	0.973907754	9.12E-08
TUCP_003835	XLOC_167971	ENSRNOT00000005942	0.953537623	1.58E-06
TUCP_003835	XLOC_167971	ENSRNOT00000018533	0.975035282	7.32E-08
TUCP_003835	XLOC_167971	ENSRNOT00000026783	0.974515598	8.11E-08

TUCP_003835	XLOC_167971	ENSRNOT00000012379	0.984726158	6.38E-09
TUCP_003835	XLOC_167971	ENSRNOT00000011384	0.965320002	3.73E-07
TUCP_003835	XLOC_167971	ENSRNOT00000014701	0.963406004	4.86E-07
TUCP_003835	XLOC_167971	ENSRNOT00000006963	0.981472246	1.67E-08
TUCP_003835	XLOC_167971	ENSRNOT00000087306	0.954824585	1.37E-06
TUCP_003835	XLOC_167971	ENSRNOT00000076820	0.962677196	5.36E-07
TUCP_003835	XLOC_167971	ENSRNOT00000073879	0.954019528	1.50E-06
TUCP_003835	XLOC_167971	ENSRNOT00000079109	0.987409547	2.44E-09
TUCP_003835	XLOC_167971	ENSRNOT00000027944	0.968773034	2.22E-07
TUCP_003835	XLOC_167971	ENSRNOT00000016801	0.955605884	1.26E-06
TUCP_003835	XLOC_167971	ENSRNOT00000025372	0.964771862	4.03E-07
TUCP_003835	XLOC_167971	ENSRNOT00000076453	0.956994859	1.08E-06
TUCP_003835	XLOC_167971	ENSRNOT00000052075	0.969268614	2.05E-07
TUCP_003835	XLOC_167971	ENSRNOT00000037456	0.952104093	1.83E-06
TUCP_003835	XLOC_167971	ENSRNOT00000032944	0.978177497	3.76E-08
TUCP_003835	XLOC_167971	ENSRNOT00000083215	0.975450214	6.74E-08
TUCP_003835	XLOC_167971	ENSRNOT00000017319	0.961651788	6.12E-07
TUCP_003835	XLOC_167971	ENSRNOT00000055187	0.956786334	1.10E-06
TUCP_003835	XLOC_167971	ENSRNOT00000005934	0.958667573	8.86E-07
TUCP_003835	XLOC_167971	ENSRNOT00000001809	0.955056361	1.34E-06
TUCP_003835	XLOC_167971	ENSRNOT00000035346	-0.956267865	1.17E-06
TUCP_003835	XLOC_167971	ENSRNOT00000024060	0.955803788	1.23E-06
TUCP_003835	XLOC_167971	ENSRNOT00000010149	0.973683432	9.51E-08
TUCP_003835	XLOC_167971	ENSRNOT00000051181	0.955854825	1.23E-06
TUCP_003835	XLOC_167971	ENSRNOT00000064774	0.95263001	1.73E-06
TUCP_003835	XLOC_167971	ENSRNOT00000004032	0.978064312	3.86E-08
TUCP_003835	XLOC_167971	ENSRNOT00000084385	0.969679558	1.92E-07
TUCP_003835	XLOC_167971	ENSRNOT00000012895	0.95801729	9.57E-07
TUCP_003835	XLOC_167971	ENSRNOT00000044562	0.951001309	2.05E-06
TUCP_003835	XLOC_167971	ENSRNOT00000027537	0.951517994	1.94E-06
TUCP_003835	XLOC_167971	ENSRNOT00000034744	0.964337354	4.28E-07
TUCP_003835	XLOC_167971	ENSRNOT00000012560	0.958812311	8.71E-07
TUCP_003835	XLOC_167971	ENSRNOT00000070815	0.963769967	4.63E-07
TUCP_003835	XLOC_167971	ENSRNOT00000085332	-0.952660157	1.73E-06
TUCP_003835	XLOC_167971	ENSRNOT00000023477	0.953408168	1.60E-06
TUCP_003835	XLOC_167971	ENSRNOT00000022213	0.960644523	6.96E-07
TUCP_003835	XLOC_167971	ENSRNOT00000022610	0.972256733	1.24E-07
TUCP_003835	XLOC_167971	ENSRNOT00000020437	0.980754397	2.01E-08
TUCP_003835	XLOC_167971	ENSRNOT00000006828	0.983109545	1.05E-08
TUCP_003835	XLOC_167971	ENSRNOT00000003971	0.952840616	1.70E-06
TUCP_003835	XLOC_167971	ENSRNOT00000003991	0.962577943	5.43E-07
TUCP_003835	XLOC_167971	ENSRNOT00000008982	0.956226411	1.18E-06
TUCP_003835	XLOC_167971	ENSRNOT00000001242	0.962877713	5.22E-07
TUCP_003835	XLOC_167971	ENSRNOT00000079917	0.954164146	1.48E-06
TUCP_003835	XLOC_167971	ENSRNOT00000078102	0.960132077	7.42E-07
TUCP_003835	XLOC_167971	ENSRNOT00000086868	0.956761877	1.11E-06
TUCP_003835	XLOC_167971	ENSRNOT00000025983	0.977955899	3.95E-08
TUCP_003835	XLOC_167971	ENSRNOT00000008929	0.962791472	5.28E-07
TUCP_003835	XLOC_167971	ENSRNOT00000013661	0.963048646	5.10E-07
TUCP_003835	XLOC_167971	ENSRNOT00000058072	0.954229471	1.46E-06
TUCP_003835	XLOC_167971	ENSRNOT00000016129	0.967127617	2.86E-07

TUCP_003835	XLOC_167971	ENSRNOT00000045083	0.950931839	2.06E-06
TUCP_003835	XLOC_167971	ENSRNOT00000033235	0.962541669	5.45E-07
TUCP_003835	XLOC_167971	ENSRNOT00000020648	0.953514215	1.58E-06
TUCP_003835	XLOC_167971	ENSRNOT00000022839	0.96706485	2.89E-07
TUCP_003835	XLOC_167971	ENSRNOT00000021116	0.964509627	4.18E-07
TUCP_003835	XLOC_167971	ENSRNOT00000032158	0.95257838	1.74E-06
TUCP_003835	XLOC_167971	ENSRNOT00000021153	0.981383559	1.71E-08
TUCP_003835	XLOC_167971	ENSRNOT00000027910	0.95027892	2.20E-06
TUCP_003835	XLOC_167971	ENSRNOT00000025027	0.956777371	1.10E-06
TUCP_003835	XLOC_167971	ENSRNOT00000087502	0.955415076	1.29E-06
TUCP_003835	XLOC_167971	ENSRNOT00000025832	0.953060648	1.66E-06
TUCP_003835	XLOC_167971	ENSRNOT00000047975	0.959101098	8.41E-07
TUCP_003835	XLOC_167971	ENSRNOT00000049538	0.968301723	2.39E-07
TUCP_003835	XLOC_167971	ENSRNOT00000051022	0.9543758	1.44E-06
TUCP_003835	XLOC_167971	ENSRNOT00000005909	0.975995492	6.03E-08
TUCP_003835	XLOC_167971	ENSRNOT00000009198	0.992566137	1.77E-10
TUCP_003835	XLOC_167971	ENSRNOT00000009129	0.98257251	1.23E-08
TUCP_003835	XLOC_167971	ENSRNOT00000025394	0.955702246	1.25E-06
TUCP_003835	XLOC_167971	ENSRNOT00000014979	0.970265953	1.74E-07
TUCP_003835	XLOC_167971	ENSRNOT00000036951	0.97935	2.86E-08
TUCP_003835	XLOC_167971	ENSRNOT00000026186	0.951979843	1.85E-06
TUCP_003835	XLOC_167971	ENSRNOT00000026121	0.954576366	1.41E-06
TUCP_003835	XLOC_167971	ENSRNOT00000011663	0.965931731	3.41E-07
TUCP_003835	XLOC_167971	ENSRNOT00000078250	0.950466317	2.16E-06
TUCP_003835	XLOC_167971	ENSRNOT00000023645	0.958944782	8.57E-07
TUCP_003835	XLOC_167971	ENSRNOT00000084734	0.964719287	4.06E-07
TUCP_003835	XLOC_167971	ENSRNOT00000006426	0.980995217	1.89E-08
TUCP_003835	XLOC_167971	ENSRNOT00000035700	0.988216984	1.75E-09
TUCP_003835	XLOC_167971	ENSRNOT00000026755	0.9646619	4.09E-07
TUCP_003835	XLOC_167971	ENSRNOT00000092029	0.967783455	2.59E-07
TUCP_003835	XLOC_167971	ENSRNOT00000058362	0.95732201	1.04E-06
TUCP_003835	XLOC_167971	ENSRNOT00000007175	0.963996173	4.48E-07
TUCP_003835	XLOC_167971	ENSRNOT00000085756	0.966041929	3.36E-07
TUCP_003835	XLOC_167971	ENSRNOT00000020669	0.962137681	5.75E-07
TUCP_003835	XLOC_167971	ENSRNOT00000059458	0.965615895	3.57E-07
TUCP_003835	XLOC_167971	ENSRNOT00000077406	0.954709057	1.39E-06
TUCP_003835	XLOC_167971	ENSRNOT00000040069	0.956179242	1.18E-06
TUCP_003835	XLOC_167971	ENSRNOT00000064320	0.952531699	1.75E-06
TUCP_003835	XLOC_167971	ENSRNOT00000046058	0.973486621	9.87E-08
TUCP_003835	XLOC_167971	ENSRNOT00000035474	0.962984851	5.14E-07
TUCP_003835	XLOC_167971	ENSRNOT00000077311	0.972491229	1.18E-07
TUCP_003835	XLOC_167971	ENSRNOT00000086336	0.967608503	2.66E-07
TUCP_003835	XLOC_167971	ENSRNOT00000006195	0.978416417	3.56E-08
TUCP_003835	XLOC_167971	ENSRNOT00000004172	0.950107661	2.24E-06
TUCP_003835	XLOC_167971	ENSRNOT00000065921	0.96461935	4.11E-07
TUCP_003835	XLOC_167971	ENSRNOT00000029125	0.962801326	5.27E-07
TUCP_003835	XLOC_167971	ENSRNOT00000014814	0.950144658	2.23E-06
TUCP_003835	XLOC_167971	ENSRNOT00000004412	0.955664256	1.25E-06
TUCP_003835	XLOC_167971	ENSRNOT00000007768	0.953644281	1.56E-06
TUCP_003835	XLOC_167971	ENSRNOT00000087095	0.971545331	1.40E-07
TUCP_003835	XLOC_167971	ENSRNOT00000064798	0.967743481	2.61E-07

TUCP_003835	XLOC_167971	ENSRNOT00000019177	0.975655024	6.47E-08
TUCP_003835	XLOC_167971	ENSRNOT00000010927	0.970015035	1.82E-07
TUCP_003835	XLOC_167971	ENSRNOT00000084595	0.954017866	1.50E-06
TUCP_003835	XLOC_167971	ENSRNOT00000027964	0.972760016	1.13E-07
TUCP_003835	XLOC_167971	ENSRNOT00000024659	0.975612515	6.52E-08
TUCP_003835	XLOC_167971	ENSRNOT00000072081	0.982514015	1.25E-08
TUCP_003835	XLOC_167971	ENSRNOT00000018990	0.96785239	2.56E-07
TUCP_003835	XLOC_167971	ENSRNOT00000017753	0.965087525	3.85E-07
TUCP_003835	XLOC_167971	ENSRNOT00000012463	-0.955381504	1.29E-06
TUCP_003835	XLOC_167971	ENSRNOT00000004140	0.952529546	1.75E-06
TUCP_003835	XLOC_167971	ENSRNOT00000010433	0.962184142	5.72E-07
TUCP_003835	XLOC_167971	ENSRNOT00000027615	0.951498989	1.95E-06
TUCP_003835	XLOC_167971	ENSRNOT00000079996	0.959563139	7.96E-07
TUCP_003836	XLOC_167975	ENSRNOT00000025981	0.958583934	8.95E-07
TUCP_003836	XLOC_167975	ENSRNOT00000026710	-0.950467355	2.16E-06
TUCP_003836	XLOC_167975	ENSRNOT00000022839	0.962155697	5.74E-07
TUCP_003836	XLOC_167975	ENSRNOT00000083215	0.962731933	5.32E-07
TUCP_003836	XLOC_167975	ENSRNOT00000025394	0.958215018	9.35E-07
TUCP_003836	XLOC_167975	ENSRNOT00000021471	-0.955750304	1.24E-06
TUCP_003836	XLOC_167975	ENSRNOT00000051294	0.954088762	1.49E-06
TUCP_003836	XLOC_167975	ENSRNOT00000078503	0.965536613	3.61E-07
TUCP_003836	XLOC_167975	ENSRNOT00000021321	0.960256271	7.30E-07
TUCP_003836	XLOC_167975	ENSRNOT00000084595	0.951061626	2.04E-06
TUCP_003836	XLOC_167975	ENSRNOT00000003991	0.957537842	1.01E-06
TUCP_003836	XLOC_167975	ENSRNOT00000016036	0.958420105	9.13E-07
TUCP_003836	XLOC_167975	ENSRNOT00000022271	0.958685237	8.84E-07
TUCP_003836	XLOC_167975	ENSRNOT00000021592	0.968447563	2.34E-07
TUCP_003836	XLOC_167975	ENSRNOT00000064900	0.954501923	1.42E-06
TUCP_003836	XLOC_167975	ENSRNOT00000021073	-0.953327251	1.61E-06
TUCP_003836	XLOC_167975	ENSRNOT00000075279	0.954629457	1.40E-06
TUCP_003836	XLOC_167975	ENSRNOT00000005894	0.970028522	1.81E-07
TUCP_003836	XLOC_167975	ENSRNOT00000025550	-0.951825372	1.88E-06
TUCP_003836	XLOC_167975	ENSRNOT00000025196	-0.958989687	8.53E-07
TUCP_003836	XLOC_167975	ENSRNOT00000023678	0.966025631	3.37E-07
TUCP_003836	XLOC_167975	ENSRNOT00000012895	0.953677129	1.55E-06
TUCP_003836	XLOC_167975	ENSRNOT00000003965	0.969183741	2.08E-07
TUCP_003836	XLOC_167975	ENSRNOT00000020322	0.953043984	1.66E-06
TUCP_003836	XLOC_167975	ENSRNOT00000067181	0.957555925	1.01E-06
TUCP_003836	XLOC_167975	ENSRNOT00000066736	0.95108831	2.03E-06
TUCP_003836	XLOC_167975	ENSRNOT00000086812	0.952523898	1.75E-06
TUCP_003836	XLOC_167975	ENSRNOT00000002576	0.957006147	1.08E-06
TUCP_003836	XLOC_167975	ENSRNOT00000056998	0.952319489	1.79E-06
TUCP_003836	XLOC_167975	ENSRNOT00000086329	0.951103696	2.03E-06
TUCP_003836	XLOC_167975	ENSRNOT00000027121	0.950930575	2.06E-06
TUCP_003836	XLOC_167975	ENSRNOT00000014749	0.968814718	2.20E-07
TUCP_003836	XLOC_167975	ENSRNOT00000039235	0.964961918	3.92E-07
TUCP_003836	XLOC_167975	ENSRNOT00000049538	0.954453113	1.43E-06
TUCP_003836	XLOC_167975	ENSRNOT00000082699	0.967965278	2.52E-07
TUCP_003836	XLOC_167975	ENSRNOT00000028847	0.960957405	6.69E-07
TUCP_003836	XLOC_167975	ENSRNOT00000076453	0.952691852	1.72E-06
TUCP_003836	XLOC_167975	ENSRNOT00000085648	0.951032098	2.04E-06

TUCP_003836	XLOC_167975	ENSRNOT00000008372	0.970731692	1.61E-07
TUCP_001783	XLOC_073522	ENSRNOT00000077022	0.952351779	1.79E-06
TUCP_001783	XLOC_073522	ENSRNOT00000071258	-0.950267682	2.20E-06
TUCP_001783	XLOC_073522	ENSRNOT00000031025	0.960471703	7.11E-07
TUCP_001783	XLOC_073522	ENSRNOT00000017042	0.968652582	2.26E-07
TUCP_001783	XLOC_073522	ENSRNOT00000017071	0.973673732	9.53E-08
TUCP_001783	XLOC_073522	ENSRNOT00000002695	0.960481322	7.10E-07
TUCP_001783	XLOC_073522	ENSRNOT00000010092	0.971540482	1.40E-07
TUCP_001783	XLOC_073522	ENSRNOT00000052003	0.983237737	1.01E-08
TUCP_001783	XLOC_073522	ENSRNOT00000039263	0.960599676	7.00E-07
TUCP_001783	XLOC_073522	ENSRNOT00000087603	0.956956985	1.08E-06
TUCP_001783	XLOC_073522	ENSRNOT00000088707	0.980041149	2.41E-08
TUCP_001783	XLOC_073522	ENSRNOT00000041741	0.971290674	1.46E-07
TUCP_001783	XLOC_073522	ENSRNOT00000009620	0.956315746	1.16E-06
TUCP_001783	XLOC_073522	ENSRNOT00000025564	0.96429699	4.30E-07
TUCP_001783	XLOC_073522	ENSRNOT00000086899	0.974990901	7.39E-08
TUCP_001783	XLOC_073522	ENSRNOT00000028058	0.982562878	1.23E-08
TUCP_001783	XLOC_073522	ENSRNOT00000083067	0.953663549	1.56E-06
TUCP_001783	XLOC_073522	ENSRNOT00000039876	0.980047209	2.41E-08
TUCP_001783	XLOC_073522	ENSRNOT00000016449	0.957172819	1.06E-06
TUCP_001783	XLOC_073522	ENSRNOT00000005726	0.971464827	1.42E-07
TUCP_001783	XLOC_073522	ENSRNOT00000012957	0.98067329	2.06E-08
TUCP_001783	XLOC_073522	ENSRNOT00000020786	0.964973473	3.91E-07
TUCP_001783	XLOC_073522	ENSRNOT00000057433	0.971204797	1.49E-07
TUCP_001783	XLOC_073522	ENSRNOT00000038494	0.958117015	9.46E-07
TUCP_001783	XLOC_073522	ENSRNOT00000023152	0.960402623	7.17E-07
TUCP_003833	XLOC_167877	ENSRNOT00000070848	0.965593833	3.58E-07
TUCP_003833	XLOC_167877	ENSRNOT00000092026	0.968600311	2.28E-07
TUCP_003833	XLOC_167877	ENSRNOT00000093075	0.993919386	6.48E-11
TUCP_003833	XLOC_167877	ENSRNOT00000058260	0.993919386	6.48E-11
TUCP_003833	XLOC_167877	ENSRNOT00000088887	0.993919386	6.48E-11
TUCP_003833	XLOC_167877	ENSRNOT00000084986	0.993919386	6.48E-11
TUCP_003833	XLOC_167877	ENSRNOT00000074346	0.959377556	8.14E-07
TUCP_003833	XLOC_167877	ENSRNOT00000073209	0.975177758	7.12E-08
TUCP_003833	XLOC_167877	ENSRNOT00000080155	0.975067681	7.28E-08
TUCP_003833	XLOC_167877	ENSRNOT00000072573	0.989256901	1.11E-09
TUCP_003833	XLOC_167877	ENSRNOT00000017828	0.988961606	1.27E-09
TUCP_003833	XLOC_167877	ENSRNOT00000080444	0.965795161	3.48E-07
TUCP_003833	XLOC_167877	ENSRNOT00000071312	0.993919386	6.48E-11
TUCP_003833	XLOC_167877	ENSRNOT00000076187	0.96814435	2.45E-07
TUCP_003833	XLOC_167877	ENSRNOT00000036565	0.959261631	8.25E-07
TUCP_003833	XLOC_167877	ENSRNOT00000058833	0.992033704	2.49E-10
TUCP_003833	XLOC_167877	ENSRNOT00000078052	0.993919386	6.48E-11
TUCP_003833	XLOC_167877	ENSRNOT00000078494	0.993713963	7.65E-11
TUCP_003833	XLOC_167877	ENSRNOT00000085653	0.993919386	6.48E-11
TUCP_003833	XLOC_167877	ENSRNOT00000057950	0.958481107	9.06E-07
TUCP_003833	XLOC_167877	ENSRNOT00000081992	0.995555253	1.36E-11
TUCP_003833	XLOC_167877	ENSRNOT00000038553	0.977834971	4.06E-08
TUCP_003833	XLOC_167877	ENSRNOT00000084120	0.977871667	4.03E-08
TUCP_003833	XLOC_167877	ENSRNOT00000055406	0.993919386	6.48E-11
TUCP_003833	XLOC_167877	ENSRNOT00000088245	0.993919386	6.48E-11

TUCP_003833	XLOC_167877	ENSRNOT00000070817	0.992393644	1.98E-10
TUCP_003833	XLOC_167877	ENSRNOT00000026753	0.993919386	6.48E-11
TUCP_003833	XLOC_167877	ENSRNOT00000080533	0.999583688	9.84E-17
TUCP_003833	XLOC_167877	ENSRNOT00000072774	0.971499057	1.41E-07
TUCP_003833	XLOC_167877	ENSRNOT00000086031	0.993919386	6.48E-11
TUCP_003833	XLOC_167877	ENSRNOT00000014312	0.993919386	6.48E-11
TUCP_003833	XLOC_167877	ENSRNOT00000042882	0.951870882	1.88E-06
TUCP_003833	XLOC_167877	ENSRNOT00000051231	0.992737836	1.57E-10
TUCP_003833	XLOC_167877	ENSRNOT00000089407	0.985633663	4.70E-09
TUCP_003833	XLOC_167877	ENSRNOT00000055034	0.989325507	1.07E-09
TUCP_003833	XLOC_167877	ENSRNOT00000046837	0.961205118	6.48E-07
TUCP_004295	XLOC_188553	ENSRNOT00000084500	0.984887141	6.05E-09
TUCP_004295	XLOC_188553	ENSRNOT00000093467	0.996134431	6.75E-12
TUCP_004295	XLOC_188553	ENSRNOT00000029340	0.999116572	4.23E-15
TUCP_004295	XLOC_188553	ENSRNOT00000077972	0.962758032	5.30E-07
TUCP_004295	XLOC_188553	ENSRNOT00000038646	1	0
TUCP_004295	XLOC_188553	ENSRNOT00000012095	0.957750287	9.88E-07
TUCP_004295	XLOC_188553	ENSRNOT00000017909	0.975587192	6.56E-08
TUCP_004295	XLOC_188553	ENSRNOT00000092579	1	0
TUCP_004295	XLOC_188553	ENSRNOT00000089235	0.998661162	3.38E-14
TUCP_004295	XLOC_188553	ENSRNOT00000036383	0.988460809	1.58E-09
TUCP_004295	XLOC_188553	ENSRNOT00000076528	0.997115373	1.57E-12
TUCP_004295	XLOC_188553	ENSRNOT00000075193	0.964326971	4.29E-07
TUCP_004295	XLOC_188553	ENSRNOT00000083034	0.990934646	4.75E-10
TUCP_004295	XLOC_188553	ENSRNOT00000089607	0.995091455	2.23E-11
TUCP_004295	XLOC_188553	ENSRNOT00000086014	0.960893638	6.74E-07
TUCP_004295	XLOC_188553	ENSRNOT00000054939	0.958021495	9.57E-07
TUCP_004295	XLOC_188553	ENSRNOT00000052315	0.983687082	8.85E-09
TUCP_004295	XLOC_188553	ENSRNOT00000088168	1	0
TUCP_004295	XLOC_188553	ENSRNOT00000046231	0.96225463	5.66E-07
TUCP_004295	XLOC_188553	ENSRNOT00000013459	0.979098017	3.03E-08
TUCP_004295	XLOC_188553	ENSRNOT00000078137	1	0
TUCP_004295	XLOC_188553	ENSRNOT00000077764	0.977143528	4.73E-08
TUCP_004295	XLOC_188553	ENSRNOT00000043661	0.966975951	2.93E-07
TUCP_004295	XLOC_188553	ENSRNOT00000092740	1	0
TUCP_004295	XLOC_188553	ENSRNOT00000000117	0.990989174	4.61E-10
TUCP_004295	XLOC_188553	ENSRNOT00000071877	0.967113583	2.87E-07
TUCP_004295	XLOC_188553	ENSRNOT00000070958	1	0
TUCP_004295	XLOC_188553	ENSRNOT00000076757	0.959205354	8.31E-07
TUCP_004295	XLOC_188553	ENSRNOT00000085310	0.965230243	3.78E-07
TUCP_004295	XLOC_188553	ENSRNOT00000047241	0.966576993	3.11E-07
TUCP_004295	XLOC_188553	ENSRNOT00000027268	1	0
TUCP_004295	XLOC_188553	ENSRNOT00000004459	1	0
TUCP_004295	XLOC_188553	ENSRNOT00000048373	0.959096578	8.42E-07
TUCP_004295	XLOC_188553	ENSRNOT00000061516	0.987595648	2.27E-09
TUCP_004295	XLOC_188553	ENSRNOT00000088148	1	0
TUCP_004295	XLOC_188553	ENSRNOT00000088527	1	0
TUCP_004295	XLOC_188553	ENSRNOT00000078701	1	0
TUCP_004295	XLOC_188553	ENSRNOT00000052308	0.962646127	5.38E-07
TUCP_004295	XLOC_188553	ENSRNOT00000021748	1	0
TUCP_004295	XLOC_188553	ENSRNOT00000089085	1	0

TUCP_004295	XLOC_188553	ENSRNOT00000008153	0.999703506	1.80E-17
TUCP_004295	XLOC_188553	ENSRNOT00000085896	0.971605382	1.39E-07
TUCP_004295	XLOC_188553	ENSRNOT00000079189	0.984972387	5.89E-09
TUCP_004295	XLOC_188553	ENSRNOT00000090078	1	0
TUCP_004295	XLOC_188553	ENSRNOT00000078020	0.958421666	9.13E-07
TUCP_004295	XLOC_188553	ENSRNOT00000092425	0.956092273	1.19E-06
TUCP_004295	XLOC_188553	ENSRNOT00000009776	1	0
TUCP_004297	XLOC_188686	ENSRNOT00000011342	0.964383475	4.25E-07
TUCP_004297	XLOC_188686	ENSRNOT00000015889	0.967909279	2.54E-07
TUCP_004297	XLOC_188686	ENSRNOT00000055289	0.974229135	8.57E-08
TUCP_004297	XLOC_188686	ENSRNOT00000085943	0.967758239	2.60E-07
TUCP_004297	XLOC_188686	ENSRNOT00000076466	0.981118434	1.83E-08
TUCP_004297	XLOC_188686	ENSRNOT00000072396	0.966008277	3.38E-07
TUCP_004296	XLOC_188678	ENSRNOT00000082043	0.97289479	1.10E-07
TUCP_004296	XLOC_188678	ENSRNOT00000076074	0.965140336	3.82E-07
TUCP_004296	XLOC_188678	ENSRNOT00000068444	0.975714788	6.39E-08
TUCP_004296	XLOC_188678	ENSRNOT00000081370	0.973613771	9.64E-08
TUCP_004296	XLOC_188678	ENSRNOT00000078645	0.952991174	1.67E-06
TUCP_004296	XLOC_188678	ENSRNOT00000075987	0.954171736	1.47E-06
TUCP_004296	XLOC_188678	ENSRNOT00000015712	0.95097361	2.05E-06
TUCP_004296	XLOC_188678	ENSRNOT00000087323	0.964143501	4.39E-07
TUCP_004296	XLOC_188678	ENSRNOT00000074930	0.975714788	6.39E-08
TUCP_004296	XLOC_188678	ENSRNOT00000080575	0.975863841	6.19E-08
TUCP_004296	XLOC_188678	ENSRNOT00000057761	0.955085571	1.33E-06
TUCP_004296	XLOC_188678	ENSRNOT00000078369	0.959665583	7.86E-07
TUCP_004296	XLOC_188678	ENSRNOT00000092982	0.975714788	6.39E-08
TUCP_004296	XLOC_188678	ENSRNOT00000088367	0.975714788	6.39E-08
TUCP_004296	XLOC_188678	ENSRNOT00000086397	0.961624873	6.14E-07
TUCP_004296	XLOC_188678	ENSRNOT00000061874	0.977511415	4.36E-08
TUCP_004296	XLOC_188678	ENSRNOT00000081996	0.975714788	6.39E-08
TUCP_004296	XLOC_188678	ENSRNOT00000022280	0.952710285	1.72E-06
TUCP_004296	XLOC_188678	ENSRNOT00000082058	0.975714788	6.39E-08
TUCP_004296	XLOC_188678	ENSRNOT00000088905	0.975714788	6.39E-08
TUCP_004296	XLOC_188678	ENSRNOT00000086831	0.975714788	6.39E-08
TUCP_004296	XLOC_188678	ENSRNOT00000045099	0.975714788	6.39E-08
TUCP_004296	XLOC_188678	ENSRNOT00000086745	0.953597598	1.57E-06
TUCP_004296	XLOC_188678	ENSRNOT00000067349	0.974646455	7.91E-08
TUCP_004296	XLOC_188678	ENSRNOT00000077789	0.984058114	7.90E-09
TUCP_004296	XLOC_188678	ENSRNOT00000087025	0.975714788	6.39E-08
TUCP_004296	XLOC_188678	ENSRNOT00000019853	0.975714788	6.39E-08
TUCP_004296	XLOC_188678	ENSRNOT00000073853	0.962546603	5.45E-07
TUCP_004296	XLOC_188678	ENSRNOT00000074990	0.975714788	6.39E-08
TUCP_004296	XLOC_188678	ENSRNOT00000065912	0.963706402	4.67E-07
TUCP_004296	XLOC_188678	ENSRNOT00000078683	0.999088699	4.94E-15
TUCP_004296	XLOC_188678	ENSRNOT00000093083	0.977952991	3.95E-08
TUCP_004291	XLOC_188379	ENSRNOT00000093439	0.974965047	7.43E-08
TUCP_003839	XLOC_168268	ENSRNOT00000065023	0.968004035	2.50E-07
TUCP_003839	XLOC_168268	ENSRNOT00000080846	0.953785717	1.54E-06
TUCP_003839	XLOC_168268	ENSRNOT00000028217	0.980095057	2.38E-08
TUCP_003839	XLOC_168268	ENSRNOT00000070961	0.961269301	6.43E-07
TUCP_003839	XLOC_168268	ENSRNOT00000009468	0.972703901	1.14E-07

TUCP_003839	XLOC_168268	ENSRNOT00000024924	0.953235383	1.63E-06
TUCP_003839	XLOC_168268	ENSRNOT00000019350	0.951993436	1.85E-06
TUCP_003839	XLOC_168268	ENSRNOT00000075400	0.954964583	1.35E-06
TUCP_003839	XLOC_168268	ENSRNOT00000057902	0.955125271	1.33E-06
TUCP_003839	XLOC_168268	ENSRNOT00000076388	0.95326159	1.62E-06
TUCP_003839	XLOC_168268	ENSRNOT00000020982	0.956646705	1.12E-06
TUCP_003839	XLOC_168268	ENSRNOT00000001379	0.95304748	1.66E-06
TUCP_003839	XLOC_168268	ENSRNOT00000067725	0.957138947	1.06E-06
TUCP_003839	XLOC_168268	ENSRNOT00000032922	0.96247056	5.50E-07
TUCP_003839	XLOC_168268	ENSRNOT00000083983	0.958818673	8.70E-07
TUCP_003839	XLOC_168268	ENSRNOT00000019044	0.960584827	7.01E-07
TUCP_003839	XLOC_168268	ENSRNOT00000023132	0.951628741	1.92E-06
TUCP_003839	XLOC_168268	ENSRNOT00000035400	0.958186074	9.38E-07
TUCP_003839	XLOC_168268	ENSRNOT00000011940	0.967492644	2.71E-07
TUCP_003839	XLOC_168268	ENSRNOT00000001373	0.95388611	1.52E-06
TUCP_003839	XLOC_168268	ENSRNOT00000092322	0.971143282	1.50E-07
TUCP_003839	XLOC_168268	ENSRNOT00000041371	0.956109418	1.19E-06
TUCP_003839	XLOC_168268	ENSRNOT00000001970	0.952805038	1.70E-06
TUCP_003839	XLOC_168268	ENSRNOT00000017618	0.971540179	1.40E-07
TUCP_003839	XLOC_168268	ENSRNOT00000067905	0.963719399	4.66E-07
TUCP_003839	XLOC_168268	ENSRNOT00000020543	0.964219977	4.35E-07
TUCP_003839	XLOC_168268	ENSRNOT00000018982	0.956768557	1.11E-06
TUCP_003839	XLOC_168268	ENSRNOT00000024775	0.967001159	2.92E-07
TUCP_003839	XLOC_168268	ENSRNOT00000007940	0.965512965	3.63E-07
TUCP_003839	XLOC_168268	ENSRNOT00000014002	0.964826997	4.00E-07
TUCP_003839	XLOC_168268	ENSRNOT00000021036	0.968868266	2.19E-07
TUCP_003839	XLOC_168268	ENSRNOT00000036374	0.968711784	2.24E-07
TUCP_003839	XLOC_168268	ENSRNOT00000090003	0.968314624	2.38E-07
TUCP_003839	XLOC_168268	ENSRNOT00000004159	0.97054979	1.66E-07
TUCP_003839	XLOC_168268	ENSRNOT00000002198	0.961998451	5.86E-07
TUCP_003839	XLOC_168268	ENSRNOT00000035875	0.957550346	1.01E-06
TUCP_003839	XLOC_168268	ENSRNOT00000020853	0.961256965	6.44E-07
TUCP_003839	XLOC_168268	ENSRNOT00000065642	0.966386379	3.19E-07
TUCP_003839	XLOC_168268	ENSRNOT00000024981	0.958980515	8.54E-07
TUCP_003839	XLOC_168268	ENSRNOT00000068666	0.969618693	1.94E-07
TUCP_003839	XLOC_168268	ENSRNOT00000025506	0.959002891	8.51E-07
TUCP_003839	XLOC_168268	ENSRNOT00000024126	0.958112572	9.46E-07
TUCP_003839	XLOC_168268	ENSRNOT00000027512	0.962164117	5.73E-07
TUCP_003839	XLOC_168268	ENSRNOT00000013535	0.964086165	4.43E-07
TUCP_003839	XLOC_168268	ENSRNOT00000024690	0.954692856	1.39E-06
TUCP_003839	XLOC_168268	ENSRNOT00000019056	0.962013755	5.84E-07
TUCP_003839	XLOC_168268	ENSRNOT00000015130	0.954657689	1.40E-06
TUCP_003839	XLOC_168268	ENSRNOT00000075386	0.955886986	1.22E-06
TUCP_003839	XLOC_168268	ENSRNOT00000027450	0.974272694	8.50E-08
TUCP_003839	XLOC_168268	ENSRNOT00000007681	0.971734251	1.36E-07
TUCP_003839	XLOC_168268	ENSRNOT00000000812	0.95891391	8.60E-07
TUCP_003839	XLOC_168268	ENSRNOT00000005286	0.973138092	1.05E-07
TUCP_003839	XLOC_168268	ENSRNOT00000073926	0.994711576	3.23E-11
TUCP_003839	XLOC_168268	ENSRNOT00000001402	0.951836485	1.88E-06
TUCP_003839	XLOC_168268	ENSRNOT00000066199	0.959116368	8.40E-07
TUCP_003839	XLOC_168268	ENSRNOT00000031653	0.970854116	1.58E-07

TUCP_003839	XLOC_168268	ENSRNOT00000022170	0.974298355	8.46E-08
TUCP_003839	XLOC_168268	ENSRNOT00000015192	0.975843518	6.22E-08
TUCP_003839	XLOC_168268	ENSRNOT00000075057	0.964716202	4.06E-07
TUCP_003839	XLOC_168268	ENSRNOT00000056656	0.992914432	1.39E-10
TUCP_003839	XLOC_168268	ENSRNOT00000006860	0.965105094	3.84E-07
TUCP_003839	XLOC_168268	ENSRNOT00000047698	0.971893162	1.32E-07
TUCP_003839	XLOC_168268	ENSRNOT00000030501	0.952640764	1.73E-06
TUCP_003839	XLOC_168268	ENSRNOT00000026552	0.975835805	6.23E-08
TUCP_003839	XLOC_168268	ENSRNOT00000020558	0.956540886	1.13E-06
TUCP_003839	XLOC_168268	ENSRNOT00000011432	0.954985745	1.35E-06
TUCP_003839	XLOC_168268	ENSRNOT00000056644	0.955546022	1.27E-06
TUCP_003839	XLOC_168268	ENSRNOT00000081593	0.962249462	5.67E-07
TUCP_003839	XLOC_168268	ENSRNOT00000039709	0.953391372	1.60E-06
TUCP_003839	XLOC_168268	ENSRNOT00000008359	0.970878171	1.57E-07
TUCP_003839	XLOC_168268	ENSRNOT00000048022	0.95137855	1.97E-06
TUCP_003839	XLOC_168268	ENSRNOT00000016262	0.964554267	4.15E-07
TUCP_003839	XLOC_168268	ENSRNOT00000050774	0.952071293	1.84E-06
TUCP_003839	XLOC_168268	ENSRNOT00000076430	0.964672162	4.08E-07
TUCP_003839	XLOC_168268	ENSRNOT00000013587	0.957506915	1.02E-06
TUCP_003839	XLOC_168268	ENSRNOT00000040981	0.966000272	3.38E-07
TUCP_003839	XLOC_168268	ENSRNOT00000067004	0.961070584	6.60E-07
TUCP_003839	XLOC_168268	ENSRNOT00000018043	0.98237111	1.30E-08
TUCP_003839	XLOC_168268	ENSRNOT00000038464	0.958537236	9.00E-07
TUCP_003839	XLOC_168268	ENSRNOT00000077730	0.957352421	1.03E-06
TUCP_003839	XLOC_168268	ENSRNOT00000007300	0.955395107	1.29E-06
TUCP_003839	XLOC_168268	ENSRNOT00000074051	0.953138088	1.64E-06
TUCP_003839	XLOC_168268	ENSRNOT00000029166	0.973611414	9.64E-08
TUCP_003839	XLOC_168268	ENSRNOT00000017955	0.973429726	9.97E-08
TUCP_003839	XLOC_168268	ENSRNOT00000057758	0.957233616	1.05E-06
TUCP_003450	XLOC_148942	ENSRNOT00000088728	-0.962918815	5.19E-07
TUCP_003450	XLOC_148942	ENSRNOT00000022358	0.962210712	5.70E-07
TUCP_001787	XLOC_073522	ENSRNOT00000012957	0.980948774	1.91E-08
TUCP_001787	XLOC_073522	ENSRNOT00000039876	0.963742077	4.64E-07
TUCP_001787	XLOC_073522	ENSRNOT00000080851	0.951208478	2.01E-06
TUCP_001787	XLOC_073522	ENSRNOT00000086899	0.987036741	2.82E-09
TUCP_001787	XLOC_073522	ENSRNOT00000067677	0.95117151	2.01E-06
TUCP_001787	XLOC_073522	ENSRNOT00000025564	0.964049246	4.45E-07
TUCP_001787	XLOC_073522	ENSRNOT00000028058	0.965289553	3.74E-07
TUCP_001787	XLOC_073522	ENSRNOT00000088707	0.959580998	7.94E-07
TUCP_001787	XLOC_073522	ENSRNOT00000031172	-0.955334959	1.30E-06
TUCP_001787	XLOC_073522	ENSRNOT00000049732	0.952089625	1.83E-06
TUCP_001787	XLOC_073522	ENSRNOT00000052003	0.956303547	1.17E-06
TUCP_001787	XLOC_073522	ENSRNOT00000092125	0.962185943	5.71E-07
TUCP_001787	XLOC_073522	ENSRNOT00000016449	0.953742288	1.54E-06
TUCP_001787	XLOC_073522	ENSRNOT00000071258	-0.966526259	3.13E-07
TUCP_001787	XLOC_073522	ENSRNOT00000023152	0.980997885	1.89E-08
TUCP_004619	XLOC_203727	ENSRNOT00000006835	0.98553975	4.86E-09
TUCP_004619	XLOC_203727	ENSRNOT00000020297	0.954970968	1.35E-06
TUCP_004619	XLOC_203727	ENSRNOT00000039551	-0.956887818	1.09E-06
TUCP_004619	XLOC_203727	ENSRNOT00000090456	0.964088105	4.43E-07
TUCP_004619	XLOC_203727	ENSRNOT00000045833	0.95970339	7.82E-07

TUCP_004619	XLOC_203727	ENSRNOT00000048910	-0.958492132	9.05E-07
TUCP_004619	XLOC_203727	ENSRNOT00000020357	0.980422477	2.19E-08
TUCP_004619	XLOC_203727	ENSRNOT00000075397	0.962018352	5.84E-07
TUCP_004619	XLOC_203727	ENSRNOT00000077727	-0.966775054	3.02E-07
TUCP_004619	XLOC_203727	ENSRNOT00000007350	0.961632909	6.14E-07
TUCP_004619	XLOC_203727	ENSRNOT00000032926	-0.954334077	1.45E-06
TUCP_004619	XLOC_203727	ENSRNOT00000019104	-0.975506288	6.66E-08
TUCP_004619	XLOC_203727	ENSRNOT00000080602	0.980195855	2.32E-08
TUCP_004619	XLOC_203727	ENSRNOT00000063930	-0.959606559	7.91E-07
TUCP_004619	XLOC_203727	ENSRNOT00000074544	-0.967951061	2.52E-07
TUCP_004619	XLOC_203727	ENSRNOT00000087062	0.954774537	1.38E-06
TUCP_004619	XLOC_203727	ENSRNOT00000006217	0.967987522	2.51E-07
TUCP_004619	XLOC_203727	ENSRNOT00000001979	-0.959445646	8.07E-07
TUCP_004619	XLOC_203727	ENSRNOT00000068353	0.956246557	1.17E-06
TUCP_004619	XLOC_203727	ENSRNOT00000003667	-0.950528195	2.15E-06
TUCP_004619	XLOC_203727	ENSRNOT00000007698	-0.955350604	1.30E-06
TUCP_004619	XLOC_203727	ENSRNOT00000013025	-0.965667384	3.55E-07
TUCP_004619	XLOC_203727	ENSRNOT00000009565	-0.959424494	8.09E-07
TUCP_004619	XLOC_203727	ENSRNOT00000010728	0.981546249	1.63E-08
TUCP_004619	XLOC_203727	ENSRNOT00000082863	0.973636461	9.60E-08
TUCP_004619	XLOC_203727	ENSRNOT00000015476	-0.965458335	3.65E-07
TUCP_004619	XLOC_203727	ENSRNOT00000005659	0.966412052	3.18E-07
TUCP_004619	XLOC_203727	ENSRNOT00000009275	-0.95280471	1.70E-06
TUCP_004619	XLOC_203727	ENSRNOT00000045326	0.960466025	7.12E-07
TUCP_004619	XLOC_203727	ENSRNOT00000087011	-0.954324056	1.45E-06
TUCP_004619	XLOC_203727	ENSRNOT00000000718	0.957687724	9.95E-07
TUCP_004619	XLOC_203727	ENSRNOT00000002638	-0.956052652	1.20E-06
TUCP_004619	XLOC_203727	ENSRNOT00000006186	0.975667566	6.45E-08
TUCP_004619	XLOC_203727	ENSRNOT00000086456	-0.951645714	1.92E-06
TUCP_004619	XLOC_203727	ENSRNOT00000007855	0.952298748	1.79E-06
TUCP_004619	XLOC_203727	ENSRNOT00000074023	0.958274372	9.29E-07
TUCP_004619	XLOC_203727	ENSRNOT00000028589	-0.952894532	1.69E-06
TUCP_004619	XLOC_203727	ENSRNOT00000078872	-0.976033288	5.98E-08
TUCP_004619	XLOC_203727	ENSRNOT00000031692	-0.955283774	1.31E-06
TUCP_004619	XLOC_203727	ENSRNOT00000052063	0.981368973	1.71E-08
TUCP_004619	XLOC_203727	ENSRNOT00000006188	-0.954944293	1.36E-06
TUCP_004619	XLOC_203727	ENSRNOT00000091596	-0.950775952	2.09E-06
TUCP_004619	XLOC_203727	ENSRNOT00000039836	-0.960480994	7.10E-07
TUCP_004619	XLOC_203727	ENSRNOT00000010524	0.973393063	1.00E-07
TUCP_004619	XLOC_203727	ENSRNOT00000055540	0.9696009	1.94E-07
TUCP_004619	XLOC_203727	ENSRNOT00000006752	0.956418591	1.15E-06
TUCP_004619	XLOC_203727	ENSRNOT00000019057	-0.957860445	9.75E-07
TUCP_004619	XLOC_203727	ENSRNOT00000072890	-0.952918256	1.68E-06
TUCP_004619	XLOC_203727	ENSRNOT00000031672	-0.962750768	5.30E-07
TUCP_004619	XLOC_203727	ENSRNOT00000002028	0.9510362	2.04E-06
TUCP_004619	XLOC_203727	ENSRNOT00000036959	-0.956572298	1.13E-06
TUCP_004619	XLOC_203727	ENSRNOT00000021629	-0.969819895	1.87E-07
TUCP_004619	XLOC_203727	ENSRNOT00000016306	-0.952944262	1.68E-06
TUCP_004619	XLOC_203727	ENSRNOT00000001507	0.951135556	2.02E-06
TUCP_004619	XLOC_203727	ENSRNOT00000066947	-0.953206735	1.63E-06
TUCP_004619	XLOC_203727	ENSRNOT00000012060	0.956722245	1.11E-06

TUCP_004619	XLOC_203727	ENSRNOT00000021621	-0.974261722	8.52E-08
TUCP_004619	XLOC_203727	ENSRNOT00000020207	0.959843238	7.69E-07
TUCP_004619	XLOC_203727	ENSRNOT00000065300	0.962488039	5.49E-07
TUCP_004619	XLOC_203727	ENSRNOT00000025079	-0.970754861	1.60E-07
TUCP_004619	XLOC_203727	ENSRNOT00000048754	-0.955840694	1.23E-06
TUCP_004619	XLOC_203727	ENSRNOT00000084709	0.977172678	4.70E-08
TUCP_004619	XLOC_203727	ENSRNOT00000039177	-0.966802296	3.00E-07
TUCP_004619	XLOC_203727	ENSRNOT00000004400	0.951592505	1.93E-06
TUCP_004619	XLOC_203727	ENSRNOT00000035320	-0.950920517	2.06E-06
TUCP_004619	XLOC_203727	ENSRNOT00000017271	0.957644719	1.00E-06
TUCP_004619	XLOC_203727	ENSRNOT00000009080	-0.955785267	1.24E-06
TUCP_004619	XLOC_203727	ENSRNOT00000076610	-0.956551282	1.13E-06
TUCP_004619	XLOC_203727	ENSRNOT00000004007	-0.952366296	1.78E-06
TUCP_004619	XLOC_203727	ENSRNOT00000020681	-0.981645955	1.59E-08
TUCP_004619	XLOC_203727	ENSRNOT00000018028	-0.969787381	1.88E-07
TUCP_004619	XLOC_203727	ENSRNOT00000024677	0.965029981	3.88E-07
TUCP_004619	XLOC_203727	ENSRNOT00000019519	0.950700384	2.11E-06
TUCP_004619	XLOC_203727	ENSRNOT00000089933	0.953301945	1.62E-06
TUCP_004619	XLOC_203727	ENSRNOT00000077908	0.953859413	1.52E-06
TUCP_004619	XLOC_203727	ENSRNOT00000027145	-0.953660454	1.56E-06
TUCP_004619	XLOC_203727	ENSRNOT00000006843	-0.954803788	1.38E-06
TUCP_004619	XLOC_203727	ENSRNOT00000090461	0.962524466	5.47E-07
TUCP_004619	XLOC_203727	ENSRNOT00000009811	0.950963589	2.06E-06
TUCP_004619	XLOC_203727	ENSRNOT00000075452	0.95636074	1.16E-06
TUCP_004619	XLOC_203727	ENSRNOT00000068134	0.96559555	3.58E-07
TUCP_004619	XLOC_203727	ENSRNOT00000025510	-0.965655354	3.55E-07
TUCP_004619	XLOC_203727	ENSRNOT00000078906	-0.964249572	4.33E-07
TUCP_004619	XLOC_203727	ENSRNOT00000000577	0.974976078	7.41E-08
TUCP_004619	XLOC_203727	ENSRNOT00000032780	0.956300147	1.17E-06
TUCP_004619	XLOC_203727	ENSRNOT00000026391	0.95299929	1.67E-06
TUCP_004619	XLOC_203727	ENSRNOT00000074323	0.955569805	1.27E-06
TUCP_004619	XLOC_203727	ENSRNOT00000083578	0.954955601	1.35E-06
TUCP_004619	XLOC_203727	ENSRNOT00000033677	-0.954186605	1.47E-06
TUCP_004619	XLOC_203727	ENSRNOT00000018488	-0.95260156	1.74E-06
TUCP_004619	XLOC_203727	ENSRNOT00000077231	0.9565827	1.13E-06
TUCP_004619	XLOC_203727	ENSRNOT00000005686	-0.96655899	3.11E-07
TUCP_004619	XLOC_203727	ENSRNOT00000086608	-0.96087835	6.76E-07
TUCP_004619	XLOC_203727	ENSRNOT00000077367	-0.969662146	1.92E-07
TUCP_004619	XLOC_203727	ENSRNOT00000085279	0.950617218	2.13E-06
TUCP_004619	XLOC_203727	ENSRNOT00000072762	-0.950778109	2.09E-06
TUCP_004619	XLOC_203727	ENSRNOT00000012177	-0.957868843	9.74E-07
TUCP_004619	XLOC_203727	ENSRNOT00000014605	-0.961801294	6.01E-07
TUCP_004619	XLOC_203727	ENSRNOT00000046490	-0.951146207	2.02E-06
TUCP_004619	XLOC_203727	ENSRNOT00000011832	-0.961561227	6.20E-07
TUCP_004619	XLOC_203727	ENSRNOT00000008274	-0.956106738	1.19E-06
TUCP_004619	XLOC_203727	ENSRNOT00000087278	-0.970738363	1.61E-07
TUCP_004619	XLOC_203727	ENSRNOT00000078349	-0.952738438	1.72E-06
TUCP_004619	XLOC_203727	ENSRNOT00000015678	-0.980842659	1.97E-08
TUCP_004619	XLOC_203727	ENSRNOT00000030074	0.953945778	1.51E-06
TUCP_004619	XLOC_203727	ENSRNOT00000010141	0.982726215	1.18E-08
TUCP_004619	XLOC_203727	ENSRNOT00000007824	-0.95853363	9.00E-07

TUCP_004619	XLOC_203727	ENSRNOT00000022502	-0.971146715	1.50E-07
TUCP_004619	XLOC_203727	ENSRNOT00000011930	0.967208304	2.83E-07
TUCP_004619	XLOC_203727	ENSRNOT00000044346	-0.988368517	1.64E-09
TUCP_004619	XLOC_203727	ENSRNOT00000083968	-0.965821495	3.47E-07
TUCP_004619	XLOC_203727	ENSRNOT00000037133	0.952482157	1.76E-06
TUCP_004619	XLOC_203727	ENSRNOT00000009150	0.96103263	6.63E-07
TUCP_004619	XLOC_203727	ENSRNOT00000068745	0.961239331	6.46E-07
TUCP_004619	XLOC_203727	ENSRNOT00000087443	0.962174587	5.72E-07
TUCP_004619	XLOC_203727	ENSRNOT00000005489	0.973817603	9.27E-08
TUCP_004619	XLOC_203727	ENSRNOT00000029179	-0.952806289	1.70E-06
TUCP_004619	XLOC_203727	ENSRNOT00000016722	0.95217836	1.82E-06
TUCP_004619	XLOC_203727	ENSRNOT00000038543	0.957131808	1.06E-06
TUCP_004619	XLOC_203727	ENSRNOT00000018001	0.95340004	1.60E-06
TUCP_004619	XLOC_203727	ENSRNOT00000006395	-0.957946702	9.65E-07
TUCP_004619	XLOC_203727	ENSRNOT00000021803	-0.954578721	1.41E-06
TUCP_004619	XLOC_203727	ENSRNOT00000039857	-0.954034386	1.50E-06
TUCP_004619	XLOC_203727	ENSRNOT00000001081	0.955470912	1.28E-06
TUCP_004619	XLOC_203727	ENSRNOT00000026481	0.964281122	4.31E-07
TUCP_004619	XLOC_203727	ENSRNOT00000024352	0.952603808	1.74E-06
TUCP_004619	XLOC_203727	ENSRNOT00000082627	-0.952792656	1.71E-06
TUCP_004619	XLOC_203727	ENSRNOT00000003544	-0.965307604	3.73E-07
TUCP_004619	XLOC_203727	ENSRNOT00000028074	-0.954445941	1.43E-06
TUCP_004619	XLOC_203727	ENSRNOT00000086119	-0.957506027	1.02E-06
TUCP_004619	XLOC_203727	ENSRNOT00000067735	-0.958273511	9.29E-07
TUCP_004619	XLOC_203727	ENSRNOT00000020892	-0.962306295	5.62E-07
TUCP_004619	XLOC_203727	ENSRNOT00000014506	-0.965710705	3.52E-07
TUCP_004619	XLOC_203727	ENSRNOT00000023262	-0.957126723	1.06E-06
TUCP_004619	XLOC_203727	ENSRNOT00000025913	-0.958247359	9.32E-07
TUCP_004619	XLOC_203727	ENSRNOT00000058495	-0.957100024	1.06E-06
TUCP_004619	XLOC_203727	ENSRNOT00000019133	0.961230912	6.46E-07
TUCP_004619	XLOC_203727	ENSRNOT00000022432	-0.964073351	4.44E-07
TUCP_004619	XLOC_203727	ENSRNOT00000065983	0.951873703	1.88E-06
TUCP_004619	XLOC_203727	ENSRNOT00000030782	-0.968573886	2.29E-07
TUCP_004619	XLOC_203727	ENSRNOT00000072299	0.969557461	1.96E-07
TUCP_004619	XLOC_203727	ENSRNOT00000027141	-0.950992656	2.05E-06
TUCP_004619	XLOC_203727	ENSRNOT00000079698	-0.956000001	1.21E-06
TUCP_004619	XLOC_203727	ENSRNOT00000025104	-0.957103937	1.06E-06
TUCP_004619	XLOC_203727	ENSRNOT00000016220	-0.959868464	7.66E-07
TUCP_004619	XLOC_203727	ENSRNOT00000020537	-0.966806849	3.00E-07
TUCP_004619	XLOC_203727	ENSRNOT00000025966	0.972420773	1.20E-07
TUCP_004619	XLOC_203727	ENSRNOT00000002313	-0.950469291	2.16E-06
TUCP_004619	XLOC_203727	ENSRNOT00000012775	-0.964312061	4.29E-07
TUCP_004619	XLOC_203727	ENSRNOT00000016637	-0.953429073	1.60E-06
TUCP_004619	XLOC_203727	ENSRNOT00000089614	0.95145061	1.96E-06
TUCP_004619	XLOC_203727	ENSRNOT00000038994	-0.958831926	8.69E-07
TUCP_004619	XLOC_203727	ENSRNOT00000023667	0.970894292	1.57E-07
TUCP_004619	XLOC_203727	ENSRNOT00000086260	-0.956734072	1.11E-06
TUCP_004619	XLOC_203727	ENSRNOT00000003787	-0.950061104	2.25E-06
TUCP_004619	XLOC_203727	ENSRNOT00000000155	0.95299281	1.67E-06
TUCP_004619	XLOC_203727	ENSRNOT00000020910	0.961108649	6.56E-07
TUCP_004619	XLOC_203727	ENSRNOT00000026643	0.950100607	2.24E-06

TUCP_004619	XLOC_203727	ENSRNOT00000068026	-0.962449739	5.52E-07
TUCP_004619	XLOC_203727	ENSRNOT00000011898	-0.953462112	1.59E-06
TUCP_004619	XLOC_203727	ENSRNOT00000066793	-0.974352691	8.37E-08
TUCP_004619	XLOC_203727	ENSRNOT00000015495	-0.961513174	6.23E-07
TUCP_004619	XLOC_203727	ENSRNOT00000074586	-0.959493342	8.02E-07
TUCP_004619	XLOC_203727	ENSRNOT00000034883	-0.962806214	5.27E-07
TUCP_004619	XLOC_203727	ENSRNOT00000049459	-0.972264227	1.23E-07
TUCP_004619	XLOC_203727	ENSRNOT00000085736	-0.951833293	1.88E-06
TUCP_004619	XLOC_203727	ENSRNOT00000022190	-0.963576647	4.75E-07
TUCP_004619	XLOC_203727	ENSRNOT00000026531	-0.960312544	7.25E-07
TUCP_004619	XLOC_203727	ENSRNOT00000035605	0.957209239	1.05E-06
TUCP_004619	XLOC_203727	ENSRNOT00000081351	0.961586444	6.18E-07
TUCP_004619	XLOC_203727	ENSRNOT00000039593	-0.96778767	2.59E-07
TUCP_004619	XLOC_203727	ENSRNOT00000016132	-0.958481164	9.06E-07
TUCP_004619	XLOC_203727	ENSRNOT00000079801	-0.956966129	1.08E-06
TUCP_004619	XLOC_203727	ENSRNOT00000067739	-0.956775745	1.10E-06
TUCP_004619	XLOC_203727	ENSRNOT00000005910	-0.954046709	1.49E-06
TUCP_004619	XLOC_203727	ENSRNOT00000020648	0.950937501	2.06E-06
TUCP_004619	XLOC_203727	ENSRNOT00000017626	-0.953313182	1.61E-06
TUCP_004619	XLOC_203727	ENSRNOT00000003992	-0.964742281	4.04E-07
TUCP_004619	XLOC_203727	ENSRNOT00000076485	-0.95763992	1.00E-06
TUCP_004619	XLOC_203727	ENSRNOT00000046983	-0.952409268	1.77E-06
TUCP_004619	XLOC_203727	ENSRNOT00000000898	0.951560269	1.94E-06
TUCP_004619	XLOC_203727	ENSRNOT00000008030	0.970666751	1.63E-07
TUCP_004619	XLOC_203727	ENSRNOT00000019816	-0.965326141	3.72E-07
TUCP_004619	XLOC_203727	ENSRNOT00000050515	0.953717138	1.55E-06
TUCP_004619	XLOC_203727	ENSRNOT00000013067	-0.972795427	1.12E-07
TUCP_004619	XLOC_203727	ENSRNOT00000077706	0.954279966	1.46E-06
TUCP_004619	XLOC_203727	ENSRNOT00000020383	0.962134888	5.75E-07
TUCP_004619	XLOC_203727	ENSRNOT00000028744	-0.952340864	1.79E-06
TUCP_004619	XLOC_203727	ENSRNOT00000015518	-0.963597422	4.74E-07
TUCP_004619	XLOC_203727	ENSRNOT00000021553	0.954922348	1.36E-06
TUCP_004619	XLOC_203727	ENSRNOT00000073294	-0.956369683	1.16E-06
TUCP_004619	XLOC_203727	ENSRNOT00000078070	0.954041933	1.49E-06
TUCP_004619	XLOC_203727	ENSRNOT00000087997	0.954371269	1.44E-06
TUCP_004619	XLOC_203727	ENSRNOT00000082138	-0.952205025	1.81E-06
TUCP_004619	XLOC_203727	ENSRNOT00000068524	-0.952830416	1.70E-06
TUCP_004619	XLOC_203727	ENSRNOT00000024452	-0.95082138	2.09E-06
TUCP_004619	XLOC_203727	ENSRNOT00000089294	-0.961742872	6.05E-07
TUCP_004619	XLOC_203727	ENSRNOT00000084729	-0.955275097	1.31E-06
TUCP_004619	XLOC_203727	ENSRNOT00000012424	0.980952164	1.91E-08
TUCP_004619	XLOC_203727	ENSRNOT00000003106	-0.953364243	1.61E-06
TUCP_004619	XLOC_203727	ENSRNOT00000028885	-0.959487043	8.03E-07
TUCP_004619	XLOC_203727	ENSRNOT00000018475	-0.958389056	9.16E-07
TUCP_004619	XLOC_203727	ENSRNOT00000084012	0.953516248	1.58E-06
TUCP_004619	XLOC_203727	ENSRNOT00000077999	0.968112932	2.46E-07
TUCP_004619	XLOC_203727	ENSRNOT00000080222	-0.968503052	2.32E-07
TUCP_004619	XLOC_203727	ENSRNOT00000058362	0.950213187	2.22E-06
TUCP_004619	XLOC_203727	ENSRNOT00000019656	-0.951318516	1.98E-06
TUCP_004619	XLOC_203727	ENSRNOT00000049285	-0.95476511	1.38E-06
TUCP_004619	XLOC_203727	ENSRNOT00000026118	-0.964093876	4.43E-07

TUCP_004619	XLOC_203727	ENSRNOT00000032505	-0.970200034	1.76E-07
TUCP_004619	XLOC_203727	ENSRNOT00000015239	-0.96339524	4.87E-07
TUCP_004619	XLOC_203727	ENSRNOT00000028907	0.964940272	3.93E-07
TUCP_004619	XLOC_203727	ENSRNOT00000071929	-0.959805984	7.72E-07
TUCP_004619	XLOC_203727	ENSRNOT00000074649	0.958009701	9.58E-07
TUCP_004619	XLOC_203727	ENSRNOT00000058111	-0.950948213	2.06E-06
TUCP_004619	XLOC_203727	ENSRNOT00000037955	-0.957335162	1.04E-06
TUCP_004619	XLOC_203727	ENSRNOT00000034487	-0.95403913	1.49E-06
TUCP_004619	XLOC_203727	ENSRNOT00000018877	-0.952486598	1.76E-06
TUCP_004619	XLOC_203727	ENSRNOT00000067780	-0.95331331	1.61E-06
TUCP_004619	XLOC_203727	ENSRNOT00000077146	-0.968510886	2.31E-07
TUCP_004619	XLOC_203727	ENSRNOT00000014384	-0.959018464	8.50E-07
TUCP_004619	XLOC_203727	ENSRNOT00000088502	-0.952358303	1.78E-06
TUCP_004619	XLOC_203727	ENSRNOT00000017765	0.95777355	9.85E-07
TUCP_004619	XLOC_203727	ENSRNOT00000087346	0.950791429	2.09E-06
TUCP_004619	XLOC_203727	ENSRNOT00000004999	0.950862816	2.08E-06
TUCP_004619	XLOC_203727	ENSRNOT00000024938	-0.956185028	1.18E-06
TUCP_004619	XLOC_203727	ENSRNOT00000021145	-0.950108298	2.24E-06
TUCP_004619	XLOC_203727	ENSRNOT00000060002	-0.959038488	8.48E-07
TUCP_004619	XLOC_203727	ENSRNOT00000046102	-0.967266794	2.80E-07
TUCP_004619	XLOC_203727	ENSRNOT00000020417	-0.972277049	1.23E-07
TUCP_004619	XLOC_203727	ENSRNOT00000075209	-0.956289589	1.17E-06
TUCP_004619	XLOC_203727	ENSRNOT00000085971	-0.962315219	5.62E-07
TUCP_004619	XLOC_203727	ENSRNOT00000058382	0.954003324	1.50E-06
TUCP_004619	XLOC_203727	ENSRNOT00000043752	-0.950387799	2.18E-06
TUCP_004619	XLOC_203727	ENSRNOT00000006824	-0.969388179	2.01E-07
TUCP_004619	XLOC_203727	ENSRNOT00000025460	0.959605989	7.91E-07
TUCP_004619	XLOC_203727	ENSRNOT00000019554	0.964943577	3.93E-07
TUCP_004619	XLOC_203727	ENSRNOT00000047663	-0.951554543	1.94E-06
TUCP_004619	XLOC_203727	ENSRNOT00000066133	-0.979593097	2.69E-08
TUCP_004619	XLOC_203727	ENSRNOT00000054960	-0.950035511	2.25E-06
TUCP_004619	XLOC_203727	ENSRNOT00000011880	0.96388159	4.56E-07
TUCP_004619	XLOC_203727	ENSRNOT00000048933	-0.956086461	1.19E-06
TUCP_004619	XLOC_203727	ENSRNOT00000024253	0.972150844	1.26E-07
TUCP_004619	XLOC_203727	ENSRNOT00000029202	-0.96679966	3.00E-07
TUCP_004619	XLOC_203727	ENSRNOT00000075630	-0.950649936	2.12E-06
TUCP_004619	XLOC_203727	ENSRNOT00000003690	0.952839675	1.70E-06
TUCP_004619	XLOC_203727	ENSRNOT00000080216	-0.956112729	1.19E-06
TUCP_004619	XLOC_203727	ENSRNOT00000035385	0.957274984	1.04E-06
TUCP_004619	XLOC_203727	ENSRNOT00000084069	0.961784383	6.02E-07
TUCP_004619	XLOC_203727	ENSRNOT00000011350	0.95340733	1.60E-06
TUCP_004619	XLOC_203727	ENSRNOT00000008901	-0.953348837	1.61E-06
TUCP_004619	XLOC_203727	ENSRNOT00000010783	-0.952781414	1.71E-06
TUCP_004619	XLOC_203727	ENSRNOT00000061201	-0.955722643	1.24E-06
TUCP_004619	XLOC_203727	ENSRNOT00000019487	0.970997909	1.54E-07
TUCP_004619	XLOC_203727	ENSRNOT00000073919	0.974290811	8.47E-08
TUCP_004619	XLOC_203727	ENSRNOT00000009994	-0.950763983	2.10E-06
TUCP_004619	XLOC_203727	ENSRNOT00000001923	0.951337305	1.98E-06
TUCP_004619	XLOC_203727	ENSRNOT00000002114	-0.953605753	1.57E-06
TUCP_004619	XLOC_203727	ENSRNOT00000010915	-0.960418626	7.16E-07
TUCP_004619	XLOC_203727	ENSRNOT00000037679	0.955770249	1.24E-06

TUCP_004619	XLOC_203727	ENSRNOT00000064535	-0.978871342	3.20E-08
TUCP_004619	XLOC_203727	ENSRNOT00000017486	0.957477565	1.02E-06
TUCP_004619	XLOC_203727	ENSRNOT00000018341	-0.956640038	1.12E-06
TUCP_004619	XLOC_203727	ENSRNOT00000025086	-0.956275088	1.17E-06
TUCP_004619	XLOC_203727	ENSRNOT00000015537	0.984036773	7.95E-09
TUCP_004619	XLOC_203727	ENSRNOT00000018074	-0.966509677	3.14E-07
TUCP_004619	XLOC_203727	ENSRNOT00000024924	-0.951517762	1.94E-06
TUCP_004619	XLOC_203727	ENSRNOT00000072632	-0.961002219	6.65E-07
TUCP_004619	XLOC_203727	ENSRNOT00000016445	0.96543075	3.67E-07
TUCP_004619	XLOC_203727	ENSRNOT00000079011	-0.970494625	1.68E-07
TUCP_004619	XLOC_203727	ENSRNOT00000015946	-0.968260307	2.41E-07
TUCP_004619	XLOC_203727	ENSRNOT00000010621	-0.954989679	1.35E-06
TUCP_004619	XLOC_203727	ENSRNOT00000055386	0.979262024	2.92E-08
TUCP_004619	XLOC_203727	ENSRNOT00000011466	0.95507485	1.34E-06
TUCP_004619	XLOC_203727	ENSRNOT00000016401	0.950164401	2.23E-06
TUCP_004619	XLOC_203727	ENSRNOT00000087838	-0.950263238	2.20E-06
TUCP_004619	XLOC_203727	ENSRNOT00000037405	0.954585971	1.41E-06
TUCP_004619	XLOC_203727	ENSRNOT00000000013	0.956430539	1.15E-06
TUCP_004619	XLOC_203727	ENSRNOT00000090760	0.967116074	2.87E-07
TUCP_004619	XLOC_203727	ENSRNOT00000024306	-0.97286897	1.11E-07
TUCP_004619	XLOC_203727	ENSRNOT00000055992	-0.960503127	7.08E-07
TUCP_004619	XLOC_203727	ENSRNOT00000085519	0.969241829	2.06E-07
TUCP_004619	XLOC_203727	ENSRNOT00000000120	0.956236756	1.17E-06
TUCP_004619	XLOC_203727	ENSRNOT00000092876	-0.960230445	7.33E-07
TUCP_004619	XLOC_203727	ENSRNOT00000016389	-0.959545531	7.97E-07
TUCP_004619	XLOC_203727	ENSRNOT00000093672	-0.950688738	2.11E-06
TUCP_004619	XLOC_203727	ENSRNOT00000004787	-0.950512857	2.15E-06
TUCP_004619	XLOC_203727	ENSRNOT00000002510	-0.950196121	2.22E-06
TUCP_004619	XLOC_203727	ENSRNOT00000060698	-0.980380755	2.22E-08
TUCP_004619	XLOC_203727	ENSRNOT00000086590	0.973854613	9.21E-08
TUCP_004619	XLOC_203727	ENSRNOT00000058351	0.978881248	3.19E-08
TUCP_004619	XLOC_203727	ENSRNOT00000066561	-0.955164416	1.32E-06
TUCP_004619	XLOC_203727	ENSRNOT00000026760	-0.971314645	1.46E-07
TUCP_004619	XLOC_203727	ENSRNOT00000020990	-0.960327929	7.24E-07
TUCP_004619	XLOC_203727	ENSRNOT00000023543	-0.974306784	8.45E-08
TUCP_004619	XLOC_203727	ENSRNOT00000080207	0.952677985	1.73E-06
TUCP_004619	XLOC_203727	ENSRNOT00000024491	-0.951970965	1.86E-06
TUCP_004619	XLOC_203727	ENSRNOT00000026767	0.956691419	1.12E-06
TUCP_004619	XLOC_203727	ENSRNOT00000081077	-0.952734048	1.72E-06
TUCP_004619	XLOC_203727	ENSRNOT00000016568	0.962572979	5.43E-07
TUCP_004619	XLOC_203727	ENSRNOT00000037588	-0.971827678	1.33E-07
TUCP_004619	XLOC_203727	ENSRNOT00000002238	-0.959242408	8.27E-07
TUCP_004619	XLOC_203727	ENSRNOT00000019910	-0.967480325	2.71E-07
TUCP_004619	XLOC_203727	ENSRNOT00000010906	-0.958668453	8.86E-07
TUCP_004619	XLOC_203727	ENSRNOT00000025097	0.970630776	1.64E-07
TUCP_004619	XLOC_203727	ENSRNOT00000067901	-0.966172185	3.30E-07
TUCP_004619	XLOC_203727	ENSRNOT00000064930	0.955374285	1.29E-06
TUCP_004619	XLOC_203727	ENSRNOT00000091448	-0.959626575	7.89E-07
TUCP_004619	XLOC_203727	ENSRNOT00000087033	-0.960149791	7.40E-07
TUCP_004619	XLOC_203727	ENSRNOT00000027967	0.963021695	5.12E-07
TUCP_004619	XLOC_203727	ENSRNOT00000013516	0.953273287	1.62E-06

TUCP_004619	XLOC_203727	ENSRNOT00000024841	-0.970297855	1.73E-07
TUCP_004619	XLOC_203727	ENSRNOT00000023383	-0.967094012	2.88E-07
TUCP_004619	XLOC_203727	ENSRNOT00000017325	0.951911741	1.87E-06
TUCP_004619	XLOC_203727	ENSRNOT00000087556	-0.960910388	6.73E-07
TUCP_004619	XLOC_203727	ENSRNOT00000022097	0.965904469	3.43E-07
TUCP_004619	XLOC_203727	ENSRNOT00000036613	-0.979446365	2.79E-08
TUCP_004619	XLOC_203727	ENSRNOT00000079002	-0.957245491	1.05E-06
TUCP_004619	XLOC_203727	ENSRNOT00000023330	0.9520756	1.84E-06
TUCP_004619	XLOC_203727	ENSRNOT00000089211	0.952949701	1.68E-06
TUCP_004619	XLOC_203727	ENSRNOT00000008256	-0.951964142	1.86E-06
TUCP_004619	XLOC_203727	ENSRNOT00000078862	0.964629811	4.11E-07
TUCP_004619	XLOC_203727	ENSRNOT00000021030	-0.96486143	3.98E-07
TUCP_004619	XLOC_203727	ENSRNOT00000046639	0.954560323	1.41E-06
TUCP_004619	XLOC_203727	ENSRNOT00000036658	-0.952792724	1.71E-06
TUCP_004619	XLOC_203727	ENSRNOT00000008113	-0.968201039	2.43E-07
TUCP_004619	XLOC_203727	ENSRNOT00000079996	0.963503821	4.80E-07
TUCP_004619	XLOC_203727	ENSRNOT00000072357	-0.956032904	1.20E-06
TUCP_004619	XLOC_203727	ENSRNOT00000023977	-0.956562651	1.13E-06
TUCP_000334	XLOC_010523	ENSRNOT00000085599	0.999938502	6.93E-21
TUCP_000334	XLOC_010523	ENSRNOT00000006689	0.9944189	4.22E-11
TUCP_000334	XLOC_010523	ENSRNOT00000081534	0.987999291	1.92E-09
TUCP_000334	XLOC_010523	ENSRNOT00000085301	0.99793679	2.93E-13
TUCP_000334	XLOC_010523	ENSRNOT00000038729	1	4.25E-78
TUCP_000334	XLOC_010523	ENSRNOT00000086385	1	0
TUCP_000334	XLOC_010523	ENSRNOT00000077695	0.96038616	7.19E-07
TUCP_000334	XLOC_010523	ENSRNOT00000078561	0.986503348	3.45E-09
TUCP_000334	XLOC_010523	ENSRNOT00000087613	1	0
TUCP_000334	XLOC_010523	ENSRNOT00000026032	0.953050675	1.66E-06
TUCP_000334	XLOC_010523	ENSRNOT00000073350	0.9658754	3.44E-07
TUCP_000334	XLOC_010523	ENSRNOT00000023062	1	0
TUCP_000334	XLOC_010523	ENSRNOT00000058834	1	0
TUCP_000334	XLOC_010523	ENSRNOT00000079420	1	0
TUCP_000334	XLOC_010523	ENSRNOT00000090761	1	0
TUCP_000334	XLOC_010523	ENSRNOT00000055923	1	0
TUCP_000334	XLOC_010523	ENSRNOT00000092416	0.963085379	5.07E-07
TUCP_000334	XLOC_010523	ENSRNOT00000071930	1	1.33E-79
TUCP_000334	XLOC_010523	ENSRNOT00000001996	0.974721715	7.79E-08
TUCP_000334	XLOC_010523	ENSRNOT00000076756	1	0
TUCP_000334	XLOC_010523	ENSRNOT00000081527	0.999420479	5.14E-16
TUCP_000334	XLOC_010523	ENSRNOT00000085067	0.960262084	7.30E-07
TUCP_000334	XLOC_010523	ENSRNOT00000091663	0.99642667	4.56E-12
TUCP_000334	XLOC_010523	ENSRNOT00000081780	1	0
TUCP_000334	XLOC_010523	ENSRNOT00000093689	0.953599805	1.57E-06
TUCP_000334	XLOC_010523	ENSRNOT00000089114	1	0
TUCP_000334	XLOC_010523	ENSRNOT00000021609	0.977017895	4.86E-08
TUCP_000334	XLOC_010523	ENSRNOT00000073316	1	0
TUCP_000334	XLOC_010523	ENSRNOT00000086557	0.977413455	4.46E-08
TUCP_000334	XLOC_010523	ENSRNOT00000071663	1	0
TUCP_000334	XLOC_010523	ENSRNOT00000042860	1	0
TUCP_000334	XLOC_010523	ENSRNOT00000072962	0.971287317	1.46E-07
TUCP_000334	XLOC_010523	ENSRNOT00000088421	1	0

TUCP_000334	XLOC_010523	ENSRNOT00000038898	1	0
TUCP_000334	XLOC_010523	ENSRNOT00000015840	0.959452012	8.06E-07
TUCP_000334	XLOC_010523	ENSRNOT00000064936	0.985957557	4.20E-09
TUCP_000334	XLOC_010523	ENSRNOT00000042777	0.990642579	5.56E-10
TUCP_000334	XLOC_010523	ENSRNOT00000010029	1	0
TUCP_001206	XLOC_045178	ENSRNOT00000081570	0.962961027	5.16E-07
TUCP_001206	XLOC_045178	ENSRNOT00000091652	0.99819185	1.52E-13
TUCP_001206	XLOC_045178	ENSRNOT00000036564	0.972337506	1.22E-07
TUCP_001206	XLOC_045178	ENSRNOT00000000109	0.962179854	5.72E-07
TUCP_001206	XLOC_045178	ENSRNOT00000080865	1	1.33E-79
TUCP_001206	XLOC_045178	ENSRNOT00000073534	0.995808019	1.01E-11
TUCP_001206	XLOC_045178	ENSRNOT00000012161	0.987544327	2.31E-09
TUCP_001206	XLOC_045178	ENSRNOT00000078707	0.960060506	7.48E-07
TUCP_001206	XLOC_045178	ENSRNOT00000087758	0.968778527	2.22E-07
TUCP_001206	XLOC_045178	ENSRNOT00000049832	0.969039151	2.13E-07
TUCP_001206	XLOC_045178	ENSRNOT00000093019	1	0
TUCP_001206	XLOC_045178	ENSRNOT00000024825	0.980452701	2.18E-08
TUCP_001206	XLOC_045178	ENSRNOT00000059266	0.96033506	7.23E-07
TUCP_001206	XLOC_045178	ENSRNOT00000072734	0.965018115	3.89E-07
TUCP_001206	XLOC_045178	ENSRNOT00000085908	0.996653227	3.29E-12
TUCP_001206	XLOC_045178	ENSRNOT00000046744	1	0
TUCP_001206	XLOC_045178	ENSRNOT00000076429	1	1.33E-79
TUCP_001206	XLOC_045178	ENSRNOT00000084667	0.999660971	3.53E-17
TUCP_001206	XLOC_045178	ENSRNOT00000055412	1	0
TUCP_001206	XLOC_045178	ENSRNOT00000093201	0.9997022	1.84E-17
TUCP_001206	XLOC_045178	ENSRNOT00000072724	1	1.33E-79
TUCP_001206	XLOC_045178	ENSRNOT00000090323	0.95603782	1.20E-06
TUCP_001206	XLOC_045178	ENSRNOT00000082760	0.986293522	3.72E-09
TUCP_001206	XLOC_045178	ENSRNOT00000066017	0.989785653	8.61E-10
TUCP_001206	XLOC_045178	ENSRNOT00000073148	0.985721397	4.56E-09
TUCP_001206	XLOC_045178	ENSRNOT00000077462	0.960098183	7.45E-07
TUCP_001206	XLOC_045178	ENSRNOT00000084375	1	0
TUCP_001206	XLOC_045178	ENSRNOT00000085447	0.998400682	8.22E-14
TUCP_001206	XLOC_045178	ENSRNOT00000006249	0.966489607	3.15E-07
TUCP_001206	XLOC_045178	ENSRNOT00000087073	0.952450698	1.77E-06
TUCP_001206	XLOC_045178	ENSRNOT00000042369	0.995659369	1.20E-11
TUCP_001206	XLOC_045178	ENSRNOT00000086554	1	4.25E-78
TUCP_001206	XLOC_045178	ENSRNOT00000054858	0.985228824	5.40E-09
TUCP_001206	XLOC_045178	ENSRNOT00000047921	1	0
TUCP_001206	XLOC_045178	ENSRNOT00000064261	0.999977196	4.86E-23
TUCP_001206	XLOC_045178	ENSRNOT00000086660	0.999208903	2.44E-15
TUCP_001206	XLOC_045178	ENSRNOT00000080933	0.967360681	2.76E-07
TUCP_001206	XLOC_045178	ENSRNOT00000083977	1	0
TUCP_001206	XLOC_045178	ENSRNOT00000092933	1	0
TUCP_001206	XLOC_045178	ENSRNOT00000009061	0.953300672	1.62E-06
TUCP_001206	XLOC_045178	ENSRNOT00000041281	0.987828355	2.06E-09
TUCP_000332	XLOC_010523	ENSRNOT00000084075	0.985974704	4.17E-09
TUCP_000332	XLOC_010523	ENSRNOT00000012880	0.955992159	1.21E-06
TUCP_000332	XLOC_010523	ENSRNOT00000074465	0.991013038	4.55E-10
TUCP_000332	XLOC_010523	ENSRNOT00000079805	0.995676039	1.18E-11
TUCP_000332	XLOC_010523	ENSRNOT00000040912	0.991013038	4.55E-10

TUCP_000332	XLOC_010523	ENSRNOT00000072648	0.991013038	4.55E-10
TUCP_000332	XLOC_010523	ENSRNOT00000007398	0.978039655	3.88E-08
TUCP_000332	XLOC_010523	ENSRNOT00000070803	0.991013038	4.55E-10
TUCP_000332	XLOC_010523	ENSRNOT00000012196	0.991013038	4.55E-10
TUCP_000332	XLOC_010523	ENSRNOT00000085772	0.991013038	4.55E-10
TUCP_000332	XLOC_010523	ENSRNOT00000093284	0.990896147	4.85E-10
TUCP_000332	XLOC_010523	ENSRNOT00000086374	0.98488923	6.05E-09
TUCP_000332	XLOC_010523	ENSRNOT00000015270	0.991013038	4.55E-10
TUCP_000332	XLOC_010523	ENSRNOT00000046192	0.991013038	4.55E-10
TUCP_000332	XLOC_010523	ENSRNOT00000044226	0.991013038	4.55E-10
TUCP_000332	XLOC_010523	ENSRNOT00000081898	0.988616084	1.48E-09
TUCP_000332	XLOC_010523	ENSRNOT00000081954	0.950162038	2.23E-06
TUCP_000332	XLOC_010523	ENSRNOT00000080291	0.989501159	9.87E-10
TUCP_000332	XLOC_010523	ENSRNOT00000004293	0.981485872	1.66E-08
TUCP_000332	XLOC_010523	ENSRNOT00000078231	0.968938773	2.16E-07
TUCP_000332	XLOC_010523	ENSRNOT00000087723	0.958469185	9.07E-07
TUCP_000332	XLOC_010523	ENSRNOT00000002834	0.991013038	4.55E-10
TUCP_000332	XLOC_010523	ENSRNOT00000001464	0.991013038	4.55E-10
TUCP_000332	XLOC_010523	ENSRNOT00000090716	0.984782606	6.26E-09
TUCP_000332	XLOC_010523	ENSRNOT00000083542	0.960516165	7.07E-07
TUCP_000332	XLOC_010523	ENSRNOT00000021278	0.962112657	5.77E-07
TUCP_000332	XLOC_010523	ENSRNOT00000076044	0.991013038	4.55E-10
TUCP_000332	XLOC_010523	ENSRNOT00000074353	0.991013038	4.55E-10
TUCP_000332	XLOC_010523	ENSRNOT00000087122	0.973304784	1.02E-07
TUCP_000332	XLOC_010523	ENSRNOT00000074295	0.97003188	1.81E-07
TUCP_000332	XLOC_010523	ENSRNOT00000057873	0.991013038	4.55E-10
TUCP_000332	XLOC_010523	ENSRNOT00000083002	0.97640146	5.54E-08
TUCP_000332	XLOC_010523	ENSRNOT00000088399	0.990997922	4.59E-10
TUCP_000332	XLOC_010523	ENSRNOT00000086248	0.991013038	4.55E-10
TUCP_000332	XLOC_010523	ENSRNOT00000089750	0.990990332	4.61E-10
TUCP_000332	XLOC_010523	ENSRNOT00000030517	0.960702224	6.91E-07
TUCP_000332	XLOC_010523	ENSRNOT00000034772	0.981244025	1.77E-08
TUCP_000332	XLOC_010523	ENSRNOT00000089305	0.95159285	1.93E-06
TUCP_000332	XLOC_010523	ENSRNOT00000024580	0.95025522	2.21E-06
TUCP_000332	XLOC_010523	ENSRNOT00000064601	0.956809168	1.10E-06
TUCP_000332	XLOC_010523	ENSRNOT00000015870	0.950725392	2.11E-06
TUCP_000332	XLOC_010523	ENSRNOT00000080812	0.978133624	3.80E-08
TUCP_000332	XLOC_010523	ENSRNOT00000092755	0.991013038	4.55E-10
TUCP_000332	XLOC_010523	ENSRNOT00000031683	0.991013038	4.55E-10
TUCP_000332	XLOC_010523	ENSRNOT00000087764	0.980419127	2.19E-08
TUCP_000333	XLOC_010523	ENSRNOT00000082043	0.998965531	9.31E-15
TUCP_000333	XLOC_010523	ENSRNOT00000090080	0.970514074	1.67E-07
TUCP_000333	XLOC_010523	ENSRNOT00000068444	1	0
TUCP_000333	XLOC_010523	ENSRNOT00000081370	0.999740797	9.21E-18
TUCP_000333	XLOC_010523	ENSRNOT00000078645	0.989717078	8.90E-10
TUCP_000333	XLOC_010523	ENSRNOT00000075987	0.987945283	1.96E-09
TUCP_000333	XLOC_010523	ENSRNOT00000074990	1	0
TUCP_000333	XLOC_010523	ENSRNOT00000087323	0.995029331	2.37E-11
TUCP_000333	XLOC_010523	ENSRNOT00000074930	1	0
TUCP_000333	XLOC_010523	ENSRNOT00000080575	0.999929267	1.39E-20
TUCP_000333	XLOC_010523	ENSRNOT00000057761	0.982981445	1.09E-08

TUCP_000333	XLOC_010523	ENSRNOT00000078369	0.993028032	1.28E-10
TUCP_000333	XLOC_010523	ENSRNOT00000092982	1	0
TUCP_000333	XLOC_010523	ENSRNOT00000088367	1	0
TUCP_000333	XLOC_010523	ENSRNOT00000061874	0.968405099	2.35E-07
TUCP_000333	XLOC_010523	ENSRNOT00000077789	0.99346224	9.30E-11
TUCP_000333	XLOC_010523	ENSRNOT00000081996	1	0
TUCP_000333	XLOC_010523	ENSRNOT00000022280	0.982176915	1.37E-08
TUCP_000333	XLOC_010523	ENSRNOT00000082058	1	0
TUCP_000333	XLOC_010523	ENSRNOT00000088905	1	0
TUCP_000333	XLOC_010523	ENSRNOT00000086831	1	0
TUCP_000333	XLOC_010523	ENSRNOT00000045099	1	0
TUCP_000333	XLOC_010523	ENSRNOT00000086745	0.978585655	3.42E-08
TUCP_000333	XLOC_010523	ENSRNOT00000085849	0.988270765	1.71E-09
TUCP_000333	XLOC_010523	ENSRNOT00000086397	0.957188178	1.05E-06
TUCP_000333	XLOC_010523	ENSRNOT00000079606	0.960472013	7.11E-07
TUCP_000333	XLOC_010523	ENSRNOT00000090207	0.953487741	1.59E-06
TUCP_000333	XLOC_010523	ENSRNOT00000087025	1	0
TUCP_000333	XLOC_010523	ENSRNOT00000019853	1	0
TUCP_000333	XLOC_010523	ENSRNOT00000073853	0.989715487	8.91E-10
TUCP_000333	XLOC_010523	ENSRNOT00000065912	0.974234118	8.57E-08
TUCP_000333	XLOC_010523	ENSRNOT00000073828	0.971647353	1.38E-07
TUCP_000333	XLOC_010523	ENSRNOT00000078683	0.966682958	3.06E-07
TUCP_000333	XLOC_010523	ENSRNOT00000093083	0.999140292	3.69E-15
TUCP_000239	XLOC_007350	ENSRNOT00000055735	0.952416453	1.77E-06
TUCP_000239	XLOC_007350	ENSRNOT00000031904	0.96980691	1.88E-07
TUCP_000239	XLOC_007350	ENSRNOT00000066739	0.964240755	4.34E-07
TUCP_000239	XLOC_007350	ENSRNOT00000068405	0.957946343	9.65E-07
TUCP_000239	XLOC_007350	ENSRNOT00000039809	0.98122695	1.78E-08
TUCP_000239	XLOC_007350	ENSRNOT00000000743	0.955365054	1.29E-06
TUCP_000239	XLOC_007350	ENSRNOT00000081246	0.956948011	1.08E-06
TUCP_000239	XLOC_007350	ENSRNOT00000019213	0.971500539	1.41E-07
TUCP_000239	XLOC_007350	ENSRNOT00000007351	-0.964823993	4.00E-07
TUCP_000239	XLOC_007350	ENSRNOT00000074939	0.988516018	1.54E-09
TUCP_000239	XLOC_007350	ENSRNOT00000066041	-0.955194289	1.32E-06
TUCP_000239	XLOC_007350	ENSRNOT00000073559	0.958937413	8.58E-07
TUCP_000239	XLOC_007350	ENSRNOT00000071560	0.962602573	5.41E-07
TUCP_000239	XLOC_007350	ENSRNOT00000078445	-0.958481829	9.06E-07
TUCP_000239	XLOC_007350	ENSRNOT00000023638	0.958260452	9.30E-07
TUCP_000239	XLOC_007350	ENSRNOT00000005228	0.958974372	8.54E-07
TUCP_000239	XLOC_007350	ENSRNOT00000088125	0.978666949	3.36E-08
TUCP_000239	XLOC_007350	ENSRNOT00000034146	0.960611488	6.99E-07
TUCP_000239	XLOC_007350	ENSRNOT00000006175	0.980715328	2.03E-08
TUCP_000239	XLOC_007350	ENSRNOT00000005220	0.964803861	4.01E-07
TUCP_000239	XLOC_007350	ENSRNOT00000023144	0.973540015	9.77E-08
TUCP_000239	XLOC_007350	ENSRNOT00000021116	0.965665208	3.55E-07
TUCP_000239	XLOC_007350	ENSRNOT00000016649	0.961139165	6.54E-07
TUCP_000239	XLOC_007350	ENSRNOT00000082100	0.97198657	1.30E-07
TUCP_000239	XLOC_007350	ENSRNOT00000040393	0.95961955	7.90E-07
TUCP_000239	XLOC_007350	ENSRNOT00000026941	0.969104563	2.10E-07
TUCP_000239	XLOC_007350	ENSRNOT00000065300	0.959261769	8.25E-07
TUCP_000239	XLOC_007350	ENSRNOT00000056813	0.96560101	3.58E-07

TUCP_000239	XLOC_007350	ENSRNOT00000043216	0.969977323	1.83E-07
TUCP_000239	XLOC_007350	ENSRNOT00000091128	-0.95998993	7.55E-07
TUCP_000239	XLOC_007350	ENSRNOT00000044742	0.973416292	1.00E-07
TUCP_000239	XLOC_007350	ENSRNOT00000009712	0.951369569	1.97E-06
TUCP_000239	XLOC_007350	ENSRNOT00000013758	0.961219894	6.47E-07
TUCP_000239	XLOC_007350	ENSRNOT00000026543	0.979758159	2.59E-08
TUCP_000239	XLOC_007350	ENSRNOT00000015076	0.964795055	4.01E-07
TUCP_000239	XLOC_007350	ENSRNOT00000066670	0.952057919	1.84E-06
TUCP_000239	XLOC_007350	ENSRNOT00000010418	0.965718081	3.52E-07
TUCP_000239	XLOC_007350	ENSRNOT00000071971	0.954582927	1.41E-06
TUCP_000239	XLOC_007350	ENSRNOT00000015073	0.966551343	3.12E-07
TUCP_000239	XLOC_007350	ENSRNOT00000025689	0.960502378	7.08E-07
TUCP_000239	XLOC_007350	ENSRNOT00000012187	0.962108541	5.77E-07
TUCP_000239	XLOC_007350	ENSRNOT00000064802	0.957912542	9.69E-07
TUCP_000239	XLOC_007350	ENSRNOT00000001507	0.958438468	9.11E-07
TUCP_000239	XLOC_007350	ENSRNOT00000006055	0.980322856	2.25E-08
TUCP_000239	XLOC_007350	ENSRNOT00000071573	0.960723148	6.89E-07
TUCP_000239	XLOC_007350	ENSRNOT00000005126	-0.961340225	6.37E-07
TUCP_000239	XLOC_007350	ENSRNOT00000028646	0.950261521	2.20E-06
TUCP_000239	XLOC_007350	ENSRNOT00000011384	0.957964621	9.63E-07
TUCP_000239	XLOC_007350	ENSRNOT00000038247	0.965307884	3.73E-07
TUCP_000239	XLOC_007350	ENSRNOT00000029439	0.951320765	1.98E-06
TUCP_000239	XLOC_007350	ENSRNOT00000093174	0.950063898	2.25E-06
TUCP_000239	XLOC_007350	ENSRNOT00000036951	0.960381404	7.19E-07
TUCP_000239	XLOC_007350	ENSRNOT00000014664	0.951825221	1.88E-06
TUCP_000239	XLOC_007350	ENSRNOT00000035989	0.979463334	2.78E-08
TUCP_000239	XLOC_007350	ENSRNOT00000057160	0.957255839	1.05E-06
TUCP_000239	XLOC_007350	ENSRNOT00000082699	0.964239427	4.34E-07
TUCP_000239	XLOC_007350	ENSRNOT00000024402	0.963146247	5.03E-07
TUCP_000239	XLOC_007350	ENSRNOT00000075326	0.962892899	5.21E-07
TUCP_000239	XLOC_007350	ENSRNOT00000021348	0.993893351	6.62E-11
TUCP_000239	XLOC_007350	ENSRNOT00000017629	0.993992864	6.10E-11
TUCP_000239	XLOC_007350	ENSRNOT00000024941	0.978015933	3.90E-08
TUCP_000239	XLOC_007350	ENSRNOT00000012671	0.974975844	7.41E-08
TUCP_000239	XLOC_007350	ENSRNOT00000026481	0.956121165	1.19E-06
TUCP_000239	XLOC_007350	ENSRNOT00000065168	0.95658116	1.13E-06
TUCP_000239	XLOC_007350	ENSRNOT00000085158	0.960353255	7.22E-07
TUCP_000239	XLOC_007350	ENSRNOT00000010463	0.952128672	1.83E-06
TUCP_000239	XLOC_007350	ENSRNOT00000083215	0.962123542	5.76E-07
TUCP_000239	XLOC_007350	ENSRNOT00000026718	0.962556454	5.44E-07
TUCP_000239	XLOC_007350	ENSRNOT00000085228	0.964662486	4.09E-07
TUCP_000239	XLOC_007350	ENSRNOT00000084012	0.950547907	2.14E-06
TUCP_000239	XLOC_007350	ENSRNOT00000020134	0.959993375	7.55E-07
TUCP_000239	XLOC_007350	ENSRNOT00000017319	0.955968528	1.21E-06
TUCP_000239	XLOC_007350	ENSRNOT00000055187	0.971957804	1.30E-07
TUCP_000239	XLOC_007350	ENSRNOT00000001809	0.950544271	2.14E-06
TUCP_000239	XLOC_007350	ENSRNOT00000004834	0.961064791	6.60E-07
TUCP_000239	XLOC_007350	ENSRNOT00000079866	0.966018045	3.37E-07
TUCP_000239	XLOC_007350	ENSRNOT00000066526	0.952882234	1.69E-06
TUCP_000239	XLOC_007350	ENSRNOT00000009811	0.950257941	2.21E-06
TUCP_000239	XLOC_007350	ENSRNOT00000081632	0.952381696	1.78E-06

TUCP_000239	XLOC_007350	ENSRNOT00000087592	0.956906085	1.09E-06
TUCP_000239	XLOC_007350	ENSRNOT00000017021	0.973040752	1.07E-07
TUCP_000239	XLOC_007350	ENSRNOT00000067822	0.972043448	1.28E-07
TUCP_000239	XLOC_007350	ENSRNOT00000032274	0.970656983	1.63E-07
TUCP_000239	XLOC_007350	ENSRNOT00000087371	0.974545091	8.06E-08
TUCP_000239	XLOC_007350	ENSRNOT00000008018	0.951158512	2.02E-06
TUCP_000239	XLOC_007350	ENSRNOT00000088054	0.972389062	1.21E-07
TUCP_000239	XLOC_007350	ENSRNOT00000039842	0.966216202	3.27E-07
TUCP_000239	XLOC_007350	ENSRNOT00000068633	0.962063666	5.81E-07
TUCP_000239	XLOC_007350	ENSRNOT00000010541	0.969284073	2.05E-07
TUCP_000239	XLOC_007350	ENSRNOT00000037446	0.982190149	1.37E-08
TUCP_000239	XLOC_007350	ENSRNOT00000004032	0.954510059	1.42E-06
TUCP_000239	XLOC_007350	ENSRNOT00000017948	0.983516449	9.32E-09
TUCP_000239	XLOC_007350	ENSRNOT00000029994	0.957261682	1.05E-06
TUCP_000239	XLOC_007350	ENSRNOT00000012895	0.959599495	7.92E-07
TUCP_000239	XLOC_007350	ENSRNOT00000025859	0.974965799	7.43E-08
TUCP_000239	XLOC_007350	ENSRNOT00000073991	0.952519558	1.75E-06
TUCP_000239	XLOC_007350	ENSRNOT00000091909	0.959246783	8.27E-07
TUCP_000239	XLOC_007350	ENSRNOT00000090918	0.959675592	7.85E-07
TUCP_000239	XLOC_007350	ENSRNOT00000023180	0.952847074	1.70E-06
TUCP_000239	XLOC_007350	ENSRNOT00000000676	0.980922298	1.93E-08
TUCP_000239	XLOC_007350	ENSRNOT00000078675	0.971733755	1.36E-07
TUCP_000239	XLOC_007350	ENSRNOT00000019222	0.969210929	2.07E-07
TUCP_000239	XLOC_007350	ENSRNOT00000028070	0.964556091	4.15E-07
TUCP_000239	XLOC_007350	ENSRNOT00000004359	0.971222487	1.48E-07
TUCP_000239	XLOC_007350	ENSRNOT00000060362	0.959237507	8.28E-07
TUCP_000239	XLOC_007350	ENSRNOT00000013274	0.995289804	1.81E-11
TUCP_000239	XLOC_007350	ENSRNOT00000022213	0.950956051	2.06E-06
TUCP_000239	XLOC_007350	ENSRNOT00000022610	0.955638741	1.26E-06
TUCP_000239	XLOC_007350	ENSRNOT00000020437	0.964364659	4.26E-07
TUCP_000239	XLOC_007350	ENSRNOT00000045574	0.950696523	2.11E-06
TUCP_000239	XLOC_007350	ENSRNOT00000010898	0.975595955	6.54E-08
TUCP_000239	XLOC_007350	ENSRNOT00000047093	0.969843939	1.87E-07
TUCP_000239	XLOC_007350	ENSRNOT00000001222	0.971646577	1.38E-07
TUCP_000239	XLOC_007350	ENSRNOT00000066968	0.956007505	1.21E-06
TUCP_000239	XLOC_007350	ENSRNOT00000005355	0.973720697	9.45E-08
TUCP_000239	XLOC_007350	ENSRNOT00000002771	0.961537175	6.21E-07
TUCP_000239	XLOC_007350	ENSRNOT00000015609	0.950572185	2.14E-06
TUCP_000239	XLOC_007350	ENSRNOT00000079895	0.972105775	1.27E-07
TUCP_000239	XLOC_007350	ENSRNOT00000025981	0.961057696	6.61E-07
TUCP_000239	XLOC_007350	ENSRNOT00000027520	0.95747759	1.02E-06
TUCP_000239	XLOC_007350	ENSRNOT00000044635	0.955157955	1.32E-06
TUCP_000239	XLOC_007350	ENSRNOT00000047387	0.984211776	7.52E-09
TUCP_000239	XLOC_007350	ENSRNOT00000004698	0.973906203	9.12E-08
TUCP_000239	XLOC_007350	ENSRNOT00000091910	0.97703032	4.85E-08
TUCP_000239	XLOC_007350	ENSRNOT00000045488	0.970438954	1.69E-07
TUCP_000239	XLOC_007350	ENSRNOT00000058072	0.963357166	4.89E-07
TUCP_000239	XLOC_007350	ENSRNOT00000081047	0.954568866	1.41E-06
TUCP_000239	XLOC_007350	ENSRNOT00000084240	0.960707418	6.90E-07
TUCP_000239	XLOC_007350	ENSRNOT00000077555	0.964205753	4.36E-07
TUCP_000239	XLOC_007350	ENSRNOT00000026174	0.961911807	5.92E-07

TUCP_000239	XLOC_007350	ENSRNOT00000008803	0.958607491	8.93E-07
TUCP_000239	XLOC_007350	ENSRNOT00000003991	0.969236953	2.06E-07
TUCP_000239	XLOC_007350	ENSRNOT00000004508	0.970771142	1.60E-07
TUCP_000239	XLOC_007350	ENSRNOT00000071764	0.974600042	7.98E-08
TUCP_000239	XLOC_007350	ENSRNOT00000078475	0.9661019	3.33E-07
TUCP_000239	XLOC_007350	ENSRNOT00000087429	0.966269188	3.25E-07
TUCP_000239	XLOC_007350	ENSRNOT00000040513	0.954198972	1.47E-06
TUCP_000239	XLOC_007350	ENSRNOT00000009683	0.990963589	4.67E-10
TUCP_000239	XLOC_007350	ENSRNOT00000083127	0.980644099	2.07E-08
TUCP_000239	XLOC_007350	ENSRNOT00000024548	0.969386688	2.01E-07
TUCP_000239	XLOC_007350	ENSRNOT00000084088	0.980360114	2.23E-08
TUCP_000239	XLOC_007350	ENSRNOT00000034704	0.989834578	8.40E-10
TUCP_000239	XLOC_007350	ENSRNOT00000064902	0.972779352	1.12E-07
TUCP_000239	XLOC_007350	ENSRNOT00000009280	0.951118338	2.02E-06
TUCP_000239	XLOC_007350	ENSRNOT00000078070	0.957452912	1.02E-06
TUCP_000239	XLOC_007350	ENSRNOT00000029664	0.952404643	1.78E-06
TUCP_000239	XLOC_007350	ENSRNOT00000065983	0.950922442	2.06E-06
TUCP_000239	XLOC_007350	ENSRNOT00000079442	0.966666569	3.06E-07
TUCP_000239	XLOC_007350	ENSRNOT00000024322	0.972689519	1.14E-07
TUCP_000239	XLOC_007350	ENSRNOT00000005262	0.961667232	6.11E-07
TUCP_000239	XLOC_007350	ENSRNOT00000085027	0.968287596	2.39E-07
TUCP_000239	XLOC_007350	ENSRNOT00000016722	0.956525602	1.14E-06
TUCP_000239	XLOC_007350	ENSRNOT00000091265	0.978662681	3.36E-08
TUCP_000239	XLOC_007350	ENSRNOT00000026457	0.976433452	5.50E-08
TUCP_000239	XLOC_007350	ENSRNOT00000000658	0.96868926	2.25E-07
TUCP_000239	XLOC_007350	ENSRNOT00000016191	0.958433497	9.11E-07
TUCP_000239	XLOC_007350	ENSRNOT00000078390	-0.960588958	7.01E-07
TUCP_000239	XLOC_007350	ENSRNOT00000089971	0.966763071	3.02E-07
TUCP_000239	XLOC_007350	ENSRNOT00000065501	0.981741113	1.55E-08
TUCP_000239	XLOC_007350	ENSRNOT00000027454	0.962859349	5.23E-07
TUCP_000239	XLOC_007350	ENSRNOT00000091488	0.961518998	6.23E-07
TUCP_000239	XLOC_007350	ENSRNOT00000025523	0.950842423	2.08E-06
TUCP_000239	XLOC_007350	ENSRNOT00000025832	0.983450734	9.51E-09
TUCP_000239	XLOC_007350	ENSRNOT00000076729	0.991814558	2.85E-10
TUCP_000239	XLOC_007350	ENSRNOT00000027615	0.953773487	1.54E-06
TUCP_000239	XLOC_007350	ENSRNOT00000005909	0.957964301	9.63E-07
TUCP_000239	XLOC_007350	ENSRNOT00000090665	0.982555392	1.24E-08
TUCP_000239	XLOC_007350	ENSRNOT00000045882	0.977844337	4.05E-08
TUCP_000239	XLOC_007350	ENSRNOT00000036750	0.985194565	5.47E-09
TUCP_000239	XLOC_007350	ENSRNOT00000025936	-0.954344916	1.45E-06
TUCP_000239	XLOC_007350	ENSRNOT00000012702	-0.952099307	1.83E-06
TUCP_000239	XLOC_007350	ENSRNOT00000025092	0.975398181	6.81E-08
TUCP_000239	XLOC_007350	ENSRNOT00000009129	0.961210352	6.48E-07
TUCP_000239	XLOC_007350	ENSRNOT00000022074	0.951117505	2.02E-06
TUCP_000239	XLOC_007350	ENSRNOT00000037790	0.964100123	4.42E-07
TUCP_000239	XLOC_007350	ENSRNOT00000027337	0.965414615	3.68E-07
TUCP_000239	XLOC_007350	ENSRNOT00000006969	0.956128638	1.19E-06
TUCP_000239	XLOC_007350	ENSRNOT00000090420	-0.962922662	5.19E-07
TUCP_000239	XLOC_007350	ENSRNOT00000075424	0.963767915	4.63E-07
TUCP_000239	XLOC_007350	ENSRNOT00000076763	0.961631034	6.14E-07
TUCP_000239	XLOC_007350	ENSRNOT00000057313	0.978107255	3.82E-08

TUCP_000239	XLOC_007350	ENSRNOT00000024420	0.966305664	3.23E-07
TUCP_000239	XLOC_007350	ENSRNOT00000025075	0.971380448	1.44E-07
TUCP_000239	XLOC_007350	ENSRNOT00000000157	0.966371271	3.20E-07
TUCP_000239	XLOC_007350	ENSRNOT00000023645	0.961550831	6.20E-07
TUCP_000239	XLOC_007350	ENSRNOT00000027910	0.961404514	6.32E-07
TUCP_000239	XLOC_007350	ENSRNOT00000010992	0.987807097	2.08E-09
TUCP_000239	XLOC_007350	ENSRNOT00000081897	0.964540322	4.16E-07
TUCP_000239	XLOC_007350	ENSRNOT00000038647	0.977022386	4.85E-08
TUCP_000239	XLOC_007350	ENSRNOT00000020379	0.951826257	1.88E-06
TUCP_000239	XLOC_007350	ENSRNOT00000076669	0.988361605	1.65E-09
TUCP_000239	XLOC_007350	ENSRNOT00000011984	0.980293006	2.26E-08
TUCP_000239	XLOC_007350	ENSRNOT00000026293	0.981466193	1.67E-08
TUCP_000239	XLOC_007350	ENSRNOT00000086119	-0.955504469	1.27E-06
TUCP_000239	XLOC_007350	ENSRNOT00000043872	0.951557139	1.94E-06
TUCP_000239	XLOC_007350	ENSRNOT00000074653	0.972259179	1.23E-07
TUCP_000239	XLOC_007350	ENSRNOT00000089007	-0.959376283	8.14E-07
TUCP_000239	XLOC_007350	ENSRNOT00000088279	0.950612795	2.13E-06
TUCP_000239	XLOC_007350	ENSRNOT00000012736	0.973694631	9.49E-08
TUCP_000239	XLOC_007350	ENSRNOT00000041096	0.964698534	4.07E-07
TUCP_000239	XLOC_007350	ENSRNOT00000031230	0.991525835	3.39E-10
TUCP_000239	XLOC_007350	ENSRNOT00000037679	0.952174078	1.82E-06
TUCP_000239	XLOC_007350	ENSRNOT00000000302	0.954146437	1.48E-06
TUCP_000239	XLOC_007350	ENSRNOT00000080928	0.958938251	8.58E-07
TUCP_000239	XLOC_007350	ENSRNOT00000052288	0.963763988	4.63E-07
TUCP_000239	XLOC_007350	ENSRNOT00000039631	0.957206501	1.05E-06
TUCP_000239	XLOC_007350	ENSRNOT00000035474	0.95323487	1.63E-06
TUCP_000239	XLOC_007350	ENSRNOT00000086336	0.950231319	2.21E-06
TUCP_000239	XLOC_007350	ENSRNOT00000068322	0.960202326	7.35E-07
TUCP_000239	XLOC_007350	ENSRNOT00000067181	0.963737306	4.65E-07
TUCP_000239	XLOC_007350	ENSRNOT00000027762	0.954038051	1.50E-06
TUCP_000239	XLOC_007350	ENSRNOT00000003296	0.957622479	1.00E-06
TUCP_000239	XLOC_007350	ENSRNOT00000043345	0.980037535	2.41E-08
TUCP_000239	XLOC_007350	ENSRNOT00000085596	0.991410485	3.63E-10
TUCP_000239	XLOC_007350	ENSRNOT00000071694	0.974849435	7.60E-08
TUCP_000239	XLOC_007350	ENSRNOT00000083093	0.954073929	1.49E-06
TUCP_000239	XLOC_007350	ENSRNOT00000035491	0.95080115	2.09E-06
TUCP_000239	XLOC_007350	ENSRNOT00000060696	0.976019322	6.00E-08
TUCP_000239	XLOC_007350	ENSRNOT00000009900	-0.954012988	1.50E-06
TUCP_000239	XLOC_007350	ENSRNOT00000004412	0.990258347	6.80E-10
TUCP_000239	XLOC_007350	ENSRNOT00000012048	0.954894511	1.36E-06
TUCP_000239	XLOC_007350	ENSRNOT00000064798	0.965800994	3.48E-07
TUCP_000239	XLOC_007350	ENSRNOT00000080207	0.967512736	2.70E-07
TUCP_000239	XLOC_007350	ENSRNOT00000026280	0.96361334	4.73E-07
TUCP_000239	XLOC_007350	ENSRNOT00000024942	0.954996674	1.35E-06
TUCP_000239	XLOC_007350	ENSRNOT00000061100	0.97134771	1.45E-07
TUCP_000239	XLOC_007350	ENSRNOT00000005387	0.968953033	2.16E-07
TUCP_000239	XLOC_007350	ENSRNOT00000027452	0.980715238	2.03E-08
TUCP_000239	XLOC_007350	ENSRNOT00000005381	0.953585497	1.57E-06
TUCP_000239	XLOC_007350	ENSRNOT00000043535	0.963154071	5.03E-07
TUCP_000239	XLOC_007350	ENSRNOT00000082174	0.975432711	6.76E-08
TUCP_000239	XLOC_007350	ENSRNOT00000005144	0.956135622	1.19E-06

TUCP_000239	XLOC_007350	ENSRNOT00000013985	0.95989952	7.63E-07
TUCP_000239	XLOC_007350	ENSRNOT00000004463	0.961956822	5.89E-07
TUCP_000239	XLOC_007350	ENSRNOT00000027964	0.964483179	4.19E-07
TUCP_000239	XLOC_007350	ENSRNOT00000023428	0.971609243	1.39E-07
TUCP_000239	XLOC_007350	ENSRNOT00000051550	0.95262226	1.74E-06
TUCP_000239	XLOC_007350	ENSRNOT00000011041	0.973314523	1.02E-07
TUCP_000239	XLOC_007350	ENSRNOT00000021674	0.973495208	9.85E-08
TUCP_000239	XLOC_007350	ENSRNOT00000047192	-0.960478401	7.11E-07
TUCP_000239	XLOC_007350	ENSRNOT00000047210	0.957244235	1.05E-06
TUCP_000239	XLOC_007350	ENSRNOT00000002292	0.973709564	9.46E-08
TUCP_000239	XLOC_007350	ENSRNOT00000022796	0.95722384	1.05E-06
TUCP_000239	XLOC_007350	ENSRNOT00000010748	0.955946204	1.21E-06
TUCP_000239	XLOC_007350	ENSRNOT00000029001	0.969312385	2.04E-07
TUCP_000239	XLOC_007350	ENSRNOT00000004140	0.960624752	6.98E-07
TUCP_000239	XLOC_007350	ENSRNOT00000015799	0.959845577	7.68E-07
TUCP_000239	XLOC_007350	ENSRNOT00000083799	0.985531006	4.87E-09
TUCP_000239	XLOC_007350	ENSRNOT00000009066	0.961213177	6.48E-07
TUCP_004348	XLOC_192124	ENSRNOT00000075980	0.955409923	1.29E-06
TUCP_004610	XLOC_203061	ENSRNOT00000075521	0.968560417	2.29E-07
TUCP_004610	XLOC_203061	ENSRNOT00000006188	-0.952873486	1.69E-06
TUCP_004610	XLOC_203061	ENSRNOT00000006186	0.960477725	7.11E-07
TUCP_004610	XLOC_203061	ENSRNOT00000013745	-0.95115181	2.02E-06
TUCP_004610	XLOC_203061	ENSRNOT00000013743	0.985224106	5.41E-09
TUCP_004610	XLOC_203061	ENSRNOT00000075397	0.957489499	1.02E-06
TUCP_004610	XLOC_203061	ENSRNOT00000032926	-0.964353081	4.27E-07
TUCP_004610	XLOC_203061	ENSRNOT00000019104	-0.963431865	4.84E-07
TUCP_004610	XLOC_203061	ENSRNOT00000006217	0.962587975	5.42E-07
TUCP_004610	XLOC_203061	ENSRNOT00000025067	-0.951440408	1.96E-06
TUCP_004610	XLOC_203061	ENSRNOT00000086706	0.963075967	5.08E-07
TUCP_004610	XLOC_203061	ENSRNOT00000030914	-0.964929217	3.94E-07
TUCP_004610	XLOC_203061	ENSRNOT00000004017	0.978513818	3.48E-08
TUCP_004610	XLOC_203061	ENSRNOT00000085885	-0.956935035	1.09E-06
TUCP_004610	XLOC_203061	ENSRNOT00000011130	-0.96230057	5.63E-07
TUCP_004610	XLOC_203061	ENSRNOT00000031692	-0.952365668	1.78E-06
TUCP_004610	XLOC_203061	ENSRNOT00000052063	0.95545125	1.28E-06
TUCP_004610	XLOC_203061	ENSRNOT00000030850	0.955141752	1.33E-06
TUCP_004610	XLOC_203061	ENSRNOT00000055540	0.962037248	5.83E-07
TUCP_004610	XLOC_203061	ENSRNOT00000006752	0.959717533	7.81E-07
TUCP_004610	XLOC_203061	ENSRNOT00000031672	-0.957804345	9.81E-07
TUCP_004610	XLOC_203061	ENSRNOT00000072771	0.957279573	1.04E-06
TUCP_004610	XLOC_203061	ENSRNOT00000073370	0.974588887	8.00E-08
TUCP_004610	XLOC_203061	ENSRNOT00000035320	-0.95320305	1.63E-06
TUCP_004610	XLOC_203061	ENSRNOT00000077999	0.959653744	7.87E-07
TUCP_004610	XLOC_203061	ENSRNOT00000060002	-0.959999721	7.54E-07
TUCP_004610	XLOC_203061	ENSRNOT00000013286	0.974710855	7.81E-08
TUCP_004610	XLOC_203061	ENSRNOT00000015188	0.965279819	3.75E-07
TUCP_004610	XLOC_203061	ENSRNOT00000017648	-0.956614061	1.13E-06
TUCP_004610	XLOC_203061	ENSRNOT00000018488	-0.963168216	5.02E-07
TUCP_004610	XLOC_203061	ENSRNOT00000005686	-0.966878262	2.97E-07
TUCP_004610	XLOC_203061	ENSRNOT00000040629	0.95149726	1.95E-06
TUCP_004610	XLOC_203061	ENSRNOT00000075934	-0.954291636	1.45E-06

TUCP_004610	XLOC_203061	ENSRNOT00000030074	0.973674004	9.53E-08
TUCP_004610	XLOC_203061	ENSRNOT00000010141	0.953269889	1.62E-06
TUCP_004610	XLOC_203061	ENSRNOT00000067901	-0.967252231	2.81E-07
TUCP_004610	XLOC_203061	ENSRNOT00000067391	-0.952438729	1.77E-06
TUCP_004610	XLOC_203061	ENSRNOT00000009150	0.950721293	2.11E-06
TUCP_004610	XLOC_203061	ENSRNOT00000084709	0.97258767	1.16E-07
TUCP_004610	XLOC_203061	ENSRNOT00000029179	-0.958866479	8.65E-07
TUCP_004610	XLOC_203061	ENSRNOT00000009156	0.977814924	4.08E-08
TUCP_004610	XLOC_203061	ENSRNOT00000026481	0.960521207	7.07E-07
TUCP_004610	XLOC_203061	ENSRNOT00000082709	-0.957302082	1.04E-06
TUCP_004610	XLOC_203061	ENSRNOT00000001401	0.976928328	4.95E-08
TUCP_004610	XLOC_203061	ENSRNOT00000016132	-0.971683863	1.37E-07
TUCP_004610	XLOC_203061	ENSRNOT00000013067	-0.969515138	1.97E-07
TUCP_004610	XLOC_203061	ENSRNOT00000016220	-0.956419794	1.15E-06
TUCP_004610	XLOC_203061	ENSRNOT00000002645	-0.950457614	2.16E-06
TUCP_004610	XLOC_203061	ENSRNOT00000016637	-0.97828091	3.67E-08
TUCP_004610	XLOC_203061	ENSRNOT00000079455	0.950128095	2.23E-06
TUCP_004610	XLOC_203061	ENSRNOT00000054948	0.970961907	1.55E-07
TUCP_004610	XLOC_203061	ENSRNOT00000074586	-0.967569596	2.68E-07
TUCP_004610	XLOC_203061	ENSRNOT00000045765	-0.952704445	1.72E-06
TUCP_004610	XLOC_203061	ENSRNOT00000088214	0.967657146	2.64E-07
TUCP_004610	XLOC_203061	ENSRNOT00000011880	0.97309439	1.06E-07
TUCP_004610	XLOC_203061	ENSRNOT00000003992	-0.958266618	9.29E-07
TUCP_004610	XLOC_203061	ENSRNOT00000050515	0.957786688	9.83E-07
TUCP_004610	XLOC_203061	ENSRNOT00000077706	0.960573142	7.02E-07
TUCP_004610	XLOC_203061	ENSRNOT00000089980	-0.963956181	4.51E-07
TUCP_004610	XLOC_203061	ENSRNOT00000078073	-0.962616894	5.40E-07
TUCP_004610	XLOC_203061	ENSRNOT00000055602	-0.95688607	1.09E-06
TUCP_004610	XLOC_203061	ENSRNOT00000087997	0.955167326	1.32E-06
TUCP_004610	XLOC_203061	ENSRNOT00000055607	-0.964999737	3.90E-07
TUCP_004610	XLOC_203061	ENSRNOT00000023876	0.97579394	6.28E-08
TUCP_004610	XLOC_203061	ENSRNOT00000033359	0.960755849	6.86E-07
TUCP_004610	XLOC_203061	ENSRNOT00000091488	0.959942832	7.59E-07
TUCP_004610	XLOC_203061	ENSRNOT00000049285	-0.962745677	5.31E-07
TUCP_004610	XLOC_203061	ENSRNOT00000015239	-0.957155375	1.06E-06
TUCP_004610	XLOC_203061	ENSRNOT00000025934	-0.954974903	1.35E-06
TUCP_004610	XLOC_203061	ENSRNOT00000074649	0.960893999	6.74E-07
TUCP_004610	XLOC_203061	ENSRNOT00000035277	0.984888104	6.05E-09
TUCP_004610	XLOC_203061	ENSRNOT00000077146	-0.964054684	4.45E-07
TUCP_004610	XLOC_203061	ENSRNOT00000044847	-0.959983785	7.56E-07
TUCP_004610	XLOC_203061	ENSRNOT00000006969	0.959301708	8.21E-07
TUCP_004610	XLOC_203061	ENSRNOT00000086456	-0.959263543	8.25E-07
TUCP_004610	XLOC_203061	ENSRNOT00000075209	-0.960175873	7.38E-07
TUCP_004610	XLOC_203061	ENSRNOT00000043752	-0.95606877	1.20E-06
TUCP_004610	XLOC_203061	ENSRNOT00000019487	0.953188381	1.64E-06
TUCP_004610	XLOC_203061	ENSRNOT00000066133	-0.972926033	1.09E-07
TUCP_004610	XLOC_203061	ENSRNOT00000048933	-0.956149184	1.19E-06
TUCP_004610	XLOC_203061	ENSRNOT00000035385	0.983285716	9.99E-09
TUCP_004610	XLOC_203061	ENSRNOT00000040540	0.951639914	1.92E-06
TUCP_004610	XLOC_203061	ENSRNOT00000071192	-0.956694856	1.12E-06
TUCP_004610	XLOC_203061	ENSRNOT00000080443	0.961970165	5.88E-07

TUCP_004610	XLOC_203061	ENSRNOT00000064535	-0.969282948	2.05E-07
TUCP_004610	XLOC_203061	ENSRNOT00000010748	0.954007298	1.50E-06
TUCP_004610	XLOC_203061	ENSRNOT00000087408	0.981856662	1.50E-08
TUCP_004610	XLOC_203061	ENSRNOT00000085295	-0.964296801	4.30E-07
TUCP_004610	XLOC_203061	ENSRNOT00000072632	-0.955636541	1.26E-06
TUCP_004610	XLOC_203061	ENSRNOT00000009749	-0.953598512	1.57E-06
TUCP_004610	XLOC_203061	ENSRNOT00000039102	0.97004017	1.81E-07
TUCP_004610	XLOC_203061	ENSRNOT00000023807	-0.950505303	2.15E-06
TUCP_004610	XLOC_203061	ENSRNOT00000009742	-0.96096797	6.68E-07
TUCP_004610	XLOC_203061	ENSRNOT00000010983	0.954575904	1.41E-06
TUCP_004610	XLOC_203061	ENSRNOT00000060698	-0.957897703	9.71E-07
TUCP_004610	XLOC_203061	ENSRNOT00000045946	0.958638685	8.89E-07
TUCP_004610	XLOC_203061	ENSRNOT00000017998	0.979249336	2.93E-08
TUCP_004610	XLOC_203061	ENSRNOT00000061100	0.960509756	7.08E-07
TUCP_004610	XLOC_203061	ENSRNOT00000017995	0.955700882	1.25E-06
TUCP_004610	XLOC_203061	ENSRNOT00000017776	0.959673316	7.85E-07
TUCP_004610	XLOC_203061	ENSRNOT00000005144	0.953442024	1.59E-06
TUCP_004610	XLOC_203061	ENSRNOT00000023628	-0.955121481	1.33E-06
TUCP_004610	XLOC_203061	ENSRNOT00000088679	-0.952218867	1.81E-06
TUCP_004610	XLOC_203061	ENSRNOT00000016776	-0.958890629	8.63E-07
TUCP_004610	XLOC_203061	ENSRNOT00000008987	-0.95005701	2.25E-06
TUCP_004610	XLOC_203061	ENSRNOT00000024253	0.982876339	1.13E-08
TUCP_004610	XLOC_203061	ENSRNOT00000039607	-0.969838096	1.87E-07
TUCP_004610	XLOC_203061	ENSRNOT00000063930	-0.96838211	2.36E-07
TUCP_004610	XLOC_203061	ENSRNOT00000048613	0.961820803	5.99E-07
TUCP_004610	XLOC_203061	ENSRNOT00000023437	0.953407967	1.60E-06
TUCP_004610	XLOC_203061	ENSRNOT00000013025	-0.969546308	1.96E-07
TUCP_004610	XLOC_203061	ENSRNOT00000041237	-0.953096088	1.65E-06
TUCP_004610	XLOC_203061	ENSRNOT00000036051	0.966336749	3.22E-07
TUCP_004610	XLOC_203061	ENSRNOT00000018074	-0.96279378	5.27E-07
TUCP_004610	XLOC_203061	ENSRNOT00000001679	0.95831824	9.24E-07
TUCP_004610	XLOC_203061	ENSRNOT00000010418	0.962146346	5.74E-07
TUCP_004610	XLOC_203061	ENSRNOT00000078884	-0.957134205	1.06E-06
TUCP_004610	XLOC_203061	ENSRNOT00000003326	-0.958064894	9.52E-07
TUCP_004610	XLOC_203061	ENSRNOT00000016306	-0.954350895	1.45E-06
TUCP_004610	XLOC_203061	ENSRNOT00000015476	-0.964211245	4.35E-07
TUCP_004610	XLOC_203061	ENSRNOT00000021621	-0.950095797	2.24E-06
TUCP_004610	XLOC_203061	ENSRNOT00000020207	0.950559593	2.14E-06
TUCP_004610	XLOC_203061	ENSRNOT00000074683	0.973415235	1.00E-07
TUCP_004610	XLOC_203061	ENSRNOT00000025079	-0.96129829	6.41E-07
TUCP_004610	XLOC_203061	ENSRNOT00000003950	-0.960152681	7.40E-07
TUCP_004610	XLOC_203061	ENSRNOT00000007824	-0.954231613	1.46E-06
TUCP_004610	XLOC_203061	ENSRNOT00000057898	-0.955667509	1.25E-06
TUCP_004610	XLOC_203061	ENSRNOT00000024677	0.979406192	2.82E-08
TUCP_004610	XLOC_203061	ENSRNOT00000078680	-0.954730199	1.39E-06
TUCP_004610	XLOC_203061	ENSRNOT00000056100	-0.959047005	8.47E-07
TUCP_004610	XLOC_203061	ENSRNOT00000009817	-0.951067346	2.03E-06
TUCP_004610	XLOC_203061	ENSRNOT00000052002	0.958898416	8.62E-07
TUCP_004610	XLOC_203061	ENSRNOT00000077231	0.951016408	2.05E-06
TUCP_004610	XLOC_203061	ENSRNOT00000030798	-0.957863267	9.75E-07
TUCP_004610	XLOC_203061	ENSRNOT00000017486	0.969076278	2.11E-07

TUCP_004610	XLOC_203061	ENSRNOT00000071540	0.978786705	3.26E-08
TUCP_004610	XLOC_203061	ENSRNOT00000091739	-0.952657948	1.73E-06
TUCP_004610	XLOC_203061	ENSRNOT00000072849	-0.968655021	2.26E-07
TUCP_004610	XLOC_203061	ENSRNOT00000073643	-0.959981044	7.56E-07
TUCP_004610	XLOC_203061	ENSRNOT00000078349	-0.970159484	1.77E-07
TUCP_004610	XLOC_203061	ENSRNOT00000022502	-0.96493896	3.93E-07
TUCP_004610	XLOC_203061	ENSRNOT00000026928	-0.953220186	1.63E-06
TUCP_004610	XLOC_203061	ENSRNOT00000049082	-0.963602788	4.73E-07
TUCP_004610	XLOC_203061	ENSRNOT00000021802	0.954224476	1.47E-06
TUCP_004610	XLOC_203061	ENSRNOT00000021803	-0.952863454	1.69E-06
TUCP_004610	XLOC_203061	ENSRNOT00000082627	-0.96213129	5.75E-07
TUCP_004610	XLOC_203061	ENSRNOT00000018122	0.977200979	4.67E-08
TUCP_004610	XLOC_203061	ENSRNOT00000003787	-0.955311738	1.30E-06
TUCP_004610	XLOC_203061	ENSRNOT00000023262	-0.968975758	2.15E-07
TUCP_004610	XLOC_203061	ENSRNOT00000059246	0.95486061	1.37E-06
TUCP_004610	XLOC_203061	ENSRNOT00000077908	0.973821247	9.27E-08
TUCP_004610	XLOC_203061	ENSRNOT00000025104	-0.952165947	1.82E-06
TUCP_004610	XLOC_203061	ENSRNOT00000091427	-0.950132766	2.23E-06
TUCP_004610	XLOC_203061	ENSRNOT00000026312	0.967259596	2.80E-07
TUCP_004610	XLOC_203061	ENSRNOT00000005604	0.966508297	3.14E-07
TUCP_004610	XLOC_203061	ENSRNOT00000068381	0.975187117	7.11E-08
TUCP_004610	XLOC_203061	ENSRNOT00000068026	-0.961148487	6.53E-07
TUCP_004610	XLOC_203061	ENSRNOT00000026089	0.958020385	9.57E-07
TUCP_004610	XLOC_203061	ENSRNOT00000009634	-0.958390282	9.16E-07
TUCP_004610	XLOC_203061	ENSRNOT00000085621	0.965994176	3.38E-07
TUCP_004610	XLOC_203061	ENSRNOT00000090456	0.961090225	6.58E-07
TUCP_004610	XLOC_203061	ENSRNOT00000008883	0.951803729	1.89E-06
TUCP_004610	XLOC_203061	ENSRNOT00000058382	0.951874856	1.87E-06
TUCP_004610	XLOC_203061	ENSRNOT00000028695	-0.961918309	5.92E-07
TUCP_004610	XLOC_203061	ENSRNOT00000026531	-0.971766683	1.35E-07
TUCP_004610	XLOC_203061	ENSRNOT00000035605	0.955159894	1.32E-06
TUCP_004610	XLOC_203061	ENSRNOT00000005172	0.95398536	1.50E-06
TUCP_004610	XLOC_203061	ENSRNOT00000005170	-0.951097067	2.03E-06
TUCP_004610	XLOC_203061	ENSRNOT00000010463	0.951783788	1.89E-06
TUCP_004610	XLOC_203061	ENSRNOT00000071897	-0.955825105	1.23E-06
TUCP_004610	XLOC_203061	ENSRNOT00000000898	0.969947295	1.84E-07
TUCP_004610	XLOC_203061	ENSRNOT00000081725	0.95520505	1.32E-06
TUCP_004610	XLOC_203061	ENSRNOT00000005459	0.952050134	1.84E-06
TUCP_004610	XLOC_203061	ENSRNOT00000009135	0.956412613	1.15E-06
TUCP_004610	XLOC_203061	ENSRNOT00000002771	0.952716637	1.72E-06
TUCP_004610	XLOC_203061	ENSRNOT00000024452	-0.962149944	5.74E-07
TUCP_004610	XLOC_203061	ENSRNOT00000028888	0.976665828	5.24E-08
TUCP_004610	XLOC_203061	ENSRNOT00000018998	0.955049161	1.34E-06
TUCP_004610	XLOC_203061	ENSRNOT00000066032	0.971957954	1.30E-07
TUCP_004610	XLOC_203061	ENSRNOT00000025037	0.955431203	1.28E-06
TUCP_004610	XLOC_203061	ENSRNOT00000011572	-0.966440278	3.17E-07
TUCP_004610	XLOC_203061	ENSRNOT00000034411	0.95631817	1.16E-06
TUCP_004610	XLOC_203061	ENSRNOT00000001607	0.968245657	2.41E-07
TUCP_004610	XLOC_203061	ENSRNOT00000075630	-0.95330762	1.62E-06
TUCP_004610	XLOC_203061	ENSRNOT00000085971	-0.957059644	1.07E-06
TUCP_004610	XLOC_203061	ENSRNOT00000015898	0.958128857	9.45E-07

TUCP_004610	XLOC_203061	ENSRNOT00000076300	-0.961117561	6.56E-07
TUCP_004610	XLOC_203061	ENSRNOT00000077152	0.952821529	1.70E-06
TUCP_004610	XLOC_203061	ENSRNOT00000015537	0.953166416	1.64E-06
TUCP_004610	XLOC_203061	ENSRNOT00000019820	0.964468249	4.20E-07
TUCP_004610	XLOC_203061	ENSRNOT00000015946	-0.953646296	1.56E-06
TUCP_004610	XLOC_203061	ENSRNOT00000004172	0.955045227	1.34E-06
TUCP_004610	XLOC_203061	ENSRNOT00000023965	-0.963268834	4.95E-07
TUCP_004610	XLOC_203061	ENSRNOT00000085279	0.97689417	4.99E-08
TUCP_004610	XLOC_203061	ENSRNOT00000015769	0.963875969	4.56E-07
TUCP_004610	XLOC_203061	ENSRNOT00000005381	0.951312851	1.98E-06
TUCP_004610	XLOC_203061	ENSRNOT00000082904	0.954670777	1.40E-06
TUCP_004610	XLOC_203061	ENSRNOT00000015318	-0.954822264	1.37E-06
TUCP_004610	XLOC_203061	ENSRNOT00000015223	0.965529297	3.62E-07
TUCP_004610	XLOC_203061	ENSRNOT00000028907	0.979612858	2.68E-08
TUCP_004610	XLOC_203061	ENSRNOT00000010224	0.983583087	9.14E-09
TUCP_004610	XLOC_203061	ENSRNOT00000035309	-0.963698584	4.67E-07
TUCP_004610	XLOC_203061	ENSRNOT00000066211	-0.952669047	1.73E-06
TUCP_004610	XLOC_203061	ENSRNOT00000017687	-0.965879758	3.44E-07
TUCP_004610	XLOC_203061	ENSRNOT00000015006	0.955447156	1.28E-06
TUCP_004610	XLOC_203061	ENSRNOT00000007350	0.958823849	8.70E-07
TUCP_004610	XLOC_203061	ENSRNOT00000004400	0.964526969	4.17E-07
TUCP_004610	XLOC_203061	ENSRNOT00000083578	0.97599016	6.04E-08
TUCP_004610	XLOC_203061	ENSRNOT00000000181	0.958857158	8.66E-07
TUCP_004610	XLOC_203061	ENSRNOT00000090499	-0.952919414	1.68E-06
TUCP_004610	XLOC_203061	ENSRNOT00000005658	-0.95553309	1.27E-06
TUCP_004610	XLOC_203061	ENSRNOT00000045326	0.954656379	1.40E-06
TUCP_004610	XLOC_203061	ENSRNOT00000045833	0.980256381	2.29E-08
TUCP_004610	XLOC_203061	ENSRNOT00000074023	0.966215269	3.28E-07
TUCP_004610	XLOC_203061	ENSRNOT00000019465	-0.957263838	1.04E-06
TUCP_004610	XLOC_203061	ENSRNOT00000009073	-0.950036182	2.25E-06
TUCP_004610	XLOC_203061	ENSRNOT00000024710	-0.955999417	1.21E-06
TUCP_004610	XLOC_203061	ENSRNOT00000013516	0.980026445	2.42E-08
TUCP_004610	XLOC_203061	ENSRNOT00000016012	-0.958002128	9.59E-07
TUCP_004610	XLOC_203061	ENSRNOT00000012060	0.963880572	4.56E-07
TUCP_004610	XLOC_203061	ENSRNOT00000023037	0.958797073	8.73E-07
TUCP_004610	XLOC_203061	ENSRNOT00000034282	0.958764257	8.76E-07
TUCP_004610	XLOC_203061	ENSRNOT00000018028	-0.963135153	5.04E-07
TUCP_004610	XLOC_203061	ENSRNOT00000022924	-0.975081476	7.26E-08
TUCP_004610	XLOC_203061	ENSRNOT00000090461	0.978062979	3.86E-08
TUCP_004610	XLOC_203061	ENSRNOT00000073983	0.953024378	1.66E-06
TUCP_004610	XLOC_203061	ENSRNOT00000010783	-0.960213264	7.34E-07
TUCP_004610	XLOC_203061	ENSRNOT00000026075	0.955300425	1.30E-06
TUCP_004610	XLOC_203061	ENSRNOT00000016330	-0.960846341	6.79E-07
TUCP_004610	XLOC_203061	ENSRNOT00000036931	-0.957241178	1.05E-06
TUCP_004610	XLOC_203061	ENSRNOT00000016389	-0.950043882	2.25E-06
TUCP_004610	XLOC_203061	ENSRNOT00000011832	-0.965711924	3.52E-07
TUCP_004610	XLOC_203061	ENSRNOT00000021030	-0.950194789	2.22E-06
TUCP_004610	XLOC_203061	ENSRNOT00000006835	0.951027305	2.04E-06
TUCP_004610	XLOC_203061	ENSRNOT00000077727	-0.968451012	2.33E-07
TUCP_004610	XLOC_203061	ENSRNOT00000044346	-0.972369909	1.21E-07
TUCP_004610	XLOC_203061	ENSRNOT00000068745	0.95435935	1.44E-06

TUCP_004610	XLOC_203061	ENSRNOT00000091637	0.955328079	1.30E-06
TUCP_004610	XLOC_203061	ENSRNOT00000092068	0.955511875	1.27E-06
TUCP_004610	XLOC_203061	ENSRNOT00000013867	0.959651156	7.87E-07
TUCP_004610	XLOC_203061	ENSRNOT00000003265	0.951178815	2.01E-06
TUCP_004610	XLOC_203061	ENSRNOT00000022190	-0.958014911	9.57E-07
TUCP_004610	XLOC_203061	ENSRNOT00000067739	-0.959187953	8.33E-07
TUCP_004610	XLOC_203061	ENSRNOT00000030782	-0.965037767	3.88E-07
TUCP_004610	XLOC_203061	ENSRNOT00000007882	-0.952809444	1.70E-06
TUCP_004610	XLOC_203061	ENSRNOT00000073919	0.979363687	2.85E-08
TUCP_004610	XLOC_203061	ENSRNOT00000036658	-0.958589367	8.95E-07
TUCP_004610	XLOC_203061	ENSRNOT00000089611	0.95667565	1.12E-06
TUCP_004610	XLOC_203061	ENSRNOT00000056166	0.951646644	1.92E-06
TUCP_004610	XLOC_203061	ENSRNOT00000086590	0.966676731	3.06E-07
TUCP_004610	XLOC_203061	ENSRNOT00000020155	0.953813694	1.53E-06
TUCP_004610	XLOC_203061	ENSRNOT00000072357	-0.961523443	6.23E-07
TUCP_004610	XLOC_203061	ENSRNOT00000022676	-0.952303496	1.79E-06
TUCP_004610	XLOC_203061	ENSRNOT00000025913	-0.962322809	5.61E-07
TUCP_004610	XLOC_203061	ENSRNOT00000005910	-0.962315686	5.62E-07
TUCP_004610	XLOC_203061	ENSRNOT00000055886	0.963727322	4.65E-07
TUCP_004610	XLOC_203061	ENSRNOT00000016722	0.963437829	4.84E-07
TUCP_004610	XLOC_203061	ENSRNOT00000014832	-0.952481703	1.76E-06
TUCP_004610	XLOC_203061	ENSRNOT00000028685	0.952133126	1.83E-06
TUCP_004610	XLOC_203061	ENSRNOT00000000013	0.960929599	6.71E-07
TUCP_004610	XLOC_203061	ENSRNOT00000080222	-0.96908947	2.11E-07
TUCP_004610	XLOC_203061	ENSRNOT00000025523	0.950243891	2.21E-06
TUCP_004610	XLOC_203061	ENSRNOT00000037955	-0.95362374	1.56E-06
TUCP_004610	XLOC_203061	ENSRNOT00000055582	0.9604676	7.12E-07
TUCP_004610	XLOC_203061	ENSRNOT00000007276	-0.957178592	1.06E-06
TUCP_004610	XLOC_203061	ENSRNOT00000004999	0.979522776	2.74E-08
TUCP_004610	XLOC_203061	ENSRNOT00000013463	0.960923413	6.72E-07
TUCP_004610	XLOC_203061	ENSRNOT00000019554	0.958370476	9.18E-07
TUCP_004610	XLOC_203061	ENSRNOT00000047663	-0.968759495	2.22E-07
TUCP_004610	XLOC_203061	ENSRNOT00000000532	-0.952760549	1.71E-06
TUCP_004610	XLOC_203061	ENSRNOT00000029202	-0.968321665	2.38E-07
TUCP_004610	XLOC_203061	ENSRNOT00000002313	-0.954166477	1.47E-06
TUCP_004610	XLOC_203061	ENSRNOT00000025794	-0.951763157	1.90E-06
TUCP_004610	XLOC_203061	ENSRNOT00000063951	0.964107283	4.42E-07
TUCP_004610	XLOC_203061	ENSRNOT00000058111	-0.961271018	6.43E-07
TUCP_004610	XLOC_203061	ENSRNOT00000009994	-0.965531506	3.62E-07
TUCP_004610	XLOC_203061	ENSRNOT00000078994	0.954319572	1.45E-06
TUCP_004610	XLOC_203061	ENSRNOT00000002114	-0.964099337	4.42E-07
TUCP_004610	XLOC_203061	ENSRNOT00000024841	-0.954865679	1.37E-06
TUCP_004610	XLOC_203061	ENSRNOT00000009462	0.958267496	9.29E-07
TUCP_004610	XLOC_203061	ENSRNOT00000046215	-0.956039841	1.20E-06
TUCP_004610	XLOC_203061	ENSRNOT00000000083	-0.952260419	1.80E-06
TUCP_004610	XLOC_203061	ENSRNOT00000087838	-0.967340648	2.77E-07
TUCP_004610	XLOC_203061	ENSRNOT00000014124	0.9533367	1.61E-06
TUCP_004610	XLOC_203061	ENSRNOT00000013632	0.959931859	7.60E-07
TUCP_004610	XLOC_203061	ENSRNOT00000028225	0.972600895	1.16E-07
TUCP_004610	XLOC_203061	ENSRNOT00000019172	-0.960868565	6.77E-07
TUCP_004610	XLOC_203061	ENSRNOT00000015495	-0.953535794	1.58E-06

TUCP_004610	XLOC_203061	ENSRNOT00000021216	0.964203363	4.36E-07
TUCP_004610	XLOC_203061	ENSRNOT00000019911	-0.952797835	1.70E-06
TUCP_004610	XLOC_203061	ENSRNOT00000040393	0.950799623	2.09E-06
TUCP_004610	XLOC_203061	ENSRNOT00000025097	0.96159909	6.17E-07
TUCP_004610	XLOC_203061	ENSRNOT00000016372	-0.964105679	4.42E-07
TUCP_004610	XLOC_203061	ENSRNOT00000025314	0.965885045	3.44E-07
TUCP_004610	XLOC_203061	ENSRNOT00000064700	0.959263505	8.25E-07
TUCP_004610	XLOC_203061	ENSRNOT00000036613	-0.951121601	2.02E-06
TUCP_004610	XLOC_203061	ENSRNOT00000025705	0.958514041	9.03E-07
TUCP_004610	XLOC_203061	ENSRNOT00000065722	-0.965240623	3.77E-07
TUCP_004610	XLOC_203061	ENSRNOT00000023977	-0.960477027	7.11E-07
TUCP_004610	XLOC_203061	ENSRNOT00000027407	0.950455528	2.16E-06
TUCP_004610	XLOC_203061	ENSRNOT00000020357	0.960803091	6.82E-07
TUCP_004610	XLOC_203061	ENSRNOT00000020051	0.975670103	6.45E-08
TUCP_004610	XLOC_203061	ENSRNOT00000026279	-0.958741657	8.78E-07
TUCP_004610	XLOC_203061	ENSRNOT00000087062	0.986692172	3.21E-09
TUCP_004610	XLOC_203061	ENSRNOT00000003087	0.952732735	1.72E-06
TUCP_004610	XLOC_203061	ENSRNOT00000091751	0.955683101	1.25E-06
TUCP_004610	XLOC_203061	ENSRNOT00000049459	-0.951895719	1.87E-06
TUCP_004610	XLOC_203061	ENSRNOT00000087011	-0.956958225	1.08E-06
TUCP_004610	XLOC_203061	ENSRNOT00000017571	-0.962218103	5.69E-07
TUCP_004610	XLOC_203061	ENSRNOT00000013851	-0.966438691	3.17E-07
TUCP_004610	XLOC_203061	ENSRNOT00000003012	-0.956347601	1.16E-06
TUCP_004610	XLOC_203061	ENSRNOT00000001507	0.969319006	2.03E-07
TUCP_004610	XLOC_203061	ENSRNOT00000087604	0.9559474	1.21E-06
TUCP_004610	XLOC_203061	ENSRNOT00000018584	0.950491299	2.16E-06
TUCP_004610	XLOC_203061	ENSRNOT00000091527	-0.959530662	7.99E-07
TUCP_004610	XLOC_203061	ENSRNOT00000005620	-0.95114858	2.02E-06
TUCP_004610	XLOC_203061	ENSRNOT00000039177	-0.967158565	2.85E-07
TUCP_004610	XLOC_203061	ENSRNOT00000017271	0.975731509	6.37E-08
TUCP_004610	XLOC_203061	ENSRNOT00000017373	0.950734257	2.10E-06
TUCP_004610	XLOC_203061	ENSRNOT00000030082	0.978231387	3.71E-08
TUCP_004610	XLOC_203061	ENSRNOT00000000477	-0.954288173	1.46E-06
TUCP_004610	XLOC_203061	ENSRNOT00000000577	0.96731287	2.78E-07
TUCP_004610	XLOC_203061	ENSRNOT00000075424	0.958415297	9.13E-07
TUCP_004610	XLOC_203061	ENSRNOT00000004007	-0.950185465	2.22E-06
TUCP_004610	XLOC_203061	ENSRNOT00000083968	-0.978078436	3.84E-08
TUCP_004610	XLOC_203061	ENSRNOT00000033806	0.952448956	1.77E-06
TUCP_004610	XLOC_203061	ENSRNOT00000026831	0.969687599	1.92E-07
TUCP_004610	XLOC_203061	ENSRNOT00000068452	0.960529083	7.06E-07
TUCP_004610	XLOC_203061	ENSRNOT00000007821	0.959293779	8.22E-07
TUCP_004610	XLOC_203061	ENSRNOT00000020892	-0.950600205	2.13E-06
TUCP_004610	XLOC_203061	ENSRNOT00000011956	0.954114125	1.48E-06
TUCP_004610	XLOC_203061	ENSRNOT00000024119	0.969915377	1.85E-07
TUCP_004610	XLOC_203061	ENSRNOT00000006824	-0.954569349	1.41E-06
TUCP_004610	XLOC_203061	ENSRNOT00000024110	0.953460228	1.59E-06
TUCP_004610	XLOC_203061	ENSRNOT00000086260	-0.965907173	3.43E-07
TUCP_004610	XLOC_203061	ENSRNOT00000078743	-0.952526998	1.75E-06
TUCP_004610	XLOC_203061	ENSRNOT00000010683	-0.955658847	1.25E-06
TUCP_004610	XLOC_203061	ENSRNOT00000001158	0.953468135	1.59E-06
TUCP_004610	XLOC_203061	ENSRNOT00000050119	-0.950121418	2.24E-06

TUCP_004610	XLOC_203061	ENSRNOT00000021548	-0.952126382	1.83E-06
TUCP_004610	XLOC_203061	ENSRNOT00000036666	0.958062997	9.52E-07
TUCP_004610	XLOC_203061	ENSRNOT00000031261	0.963262083	4.96E-07
TUCP_004610	XLOC_203061	ENSRNOT00000019816	-0.959862257	7.67E-07
TUCP_004610	XLOC_203061	ENSRNOT00000020383	0.960831203	6.80E-07
TUCP_004610	XLOC_203061	ENSRNOT00000015518	-0.950375617	2.18E-06
TUCP_004610	XLOC_203061	ENSRNOT00000056015	-0.960906352	6.73E-07
TUCP_004610	XLOC_203061	ENSRNOT00000090784	-0.962450881	5.52E-07
TUCP_004610	XLOC_203061	ENSRNOT00000020795	0.959314334	8.20E-07
TUCP_004610	XLOC_203061	ENSRNOT00000087502	0.958253583	9.31E-07
TUCP_004610	XLOC_203061	ENSRNOT00000041106	0.971716156	1.36E-07
TUCP_004610	XLOC_203061	ENSRNOT00000012424	0.955213363	1.32E-06
TUCP_004610	XLOC_203061	ENSRNOT00000002974	0.975412816	6.79E-08
TUCP_004610	XLOC_203061	ENSRNOT00000000658	0.950147476	2.23E-06
TUCP_004610	XLOC_203061	ENSRNOT00000067610	-0.956016779	1.20E-06
TUCP_004610	XLOC_203061	ENSRNOT00000020005	0.962343309	5.60E-07
TUCP_004610	XLOC_203061	ENSRNOT00000083632	-0.95581271	1.23E-06
TUCP_004610	XLOC_203061	ENSRNOT00000086122	0.958445604	9.10E-07
TUCP_004610	XLOC_203061	ENSRNOT00000017504	0.958993964	8.52E-07
TUCP_004610	XLOC_203061	ENSRNOT00000087346	0.950566841	2.14E-06
TUCP_004610	XLOC_203061	ENSRNOT00000023763	-0.961819035	5.99E-07
TUCP_004610	XLOC_203061	ENSRNOT00000044009	0.950328205	2.19E-06
TUCP_004610	XLOC_203061	ENSRNOT00000010993	0.964851461	3.98E-07
TUCP_004610	XLOC_203061	ENSRNOT00000059181	0.970684493	1.62E-07
TUCP_004610	XLOC_203061	ENSRNOT00000003691	-0.963704387	4.67E-07
TUCP_004610	XLOC_203061	ENSRNOT00000003690	0.954096251	1.49E-06
TUCP_004610	XLOC_203061	ENSRNOT00000017765	0.950865422	2.08E-06
TUCP_004610	XLOC_203061	ENSRNOT00000017761	0.962347415	5.59E-07
TUCP_004610	XLOC_203061	ENSRNOT00000023543	-0.965279923	3.75E-07
TUCP_004610	XLOC_203061	ENSRNOT00000001923	0.958908195	8.61E-07
TUCP_004610	XLOC_203061	ENSRNOT00000025887	-0.96759336	2.67E-07
TUCP_004610	XLOC_203061	ENSRNOT00000005405	0.969106261	2.10E-07
TUCP_004610	XLOC_203061	ENSRNOT00000006366	-0.972446932	1.19E-07
TUCP_004610	XLOC_203061	ENSRNOT00000018634	-0.950193578	2.22E-06
TUCP_004610	XLOC_203061	ENSRNOT00000079264	-0.952862657	1.69E-06
TUCP_004610	XLOC_203061	ENSRNOT00000085517	0.953304063	1.62E-06
TUCP_004610	XLOC_203061	ENSRNOT00000037405	0.978681448	3.35E-08
TUCP_004610	XLOC_203061	ENSRNOT00000012625	-0.954964848	1.35E-06
TUCP_004610	XLOC_203061	ENSRNOT00000080677	-0.952783316	1.71E-06
TUCP_004610	XLOC_203061	ENSRNOT00000025460	0.976457502	5.48E-08
TUCP_004610	XLOC_203061	ENSRNOT00000026760	-0.97517642	7.12E-08
TUCP_004610	XLOC_203061	ENSRNOT00000037588	-0.957490839	1.02E-06
TUCP_004610	XLOC_203061	ENSRNOT00000027360	-0.960831816	6.80E-07
TUCP_004610	XLOC_203061	ENSRNOT00000067503	0.952236137	1.81E-06
TUCP_004610	XLOC_203061	ENSRNOT00000044486	-0.955149253	1.33E-06
TUCP_004610	XLOC_203061	ENSRNOT00000047210	0.953423507	1.60E-06
TUCP_004610	XLOC_203061	ENSRNOT00000024011	0.972703912	1.14E-07
TUCP_000235	XLOC_007153	ENSRNOT00000024186	0.983200502	1.02E-08
TUCP_000235	XLOC_007153	ENSRNOT00000074753	0.998182349	1.56E-13
TUCP_000235	XLOC_007153	ENSRNOT00000004876	0.959071443	8.44E-07
TUCP_000235	XLOC_007153	ENSRNOT00000041904	1	0

TUCP_000235	XLOC_007153	ENSRNOT00000083487	0.989515314	9.80E-10
TUCP_000235	XLOC_007153	ENSRNOT00000077990	1	0
TUCP_000235	XLOC_007153	ENSRNOT00000038826	0.99977672	4.37E-18
TUCP_000235	XLOC_007153	ENSRNOT00000046842	0.973644314	9.58E-08
TUCP_000235	XLOC_007153	ENSRNOT00000037169	0.980329406	2.24E-08
TUCP_000235	XLOC_007153	ENSRNOT00000021243	0.987425406	2.42E-09
TUCP_000235	XLOC_007153	ENSRNOT00000060250	0.999933853	9.97E-21
TUCP_000235	XLOC_007153	ENSRNOT00000091934	1	0
TUCP_000235	XLOC_007153	ENSRNOT00000046585	0.982941597	1.11E-08
TUCP_000235	XLOC_007153	ENSRNOT00000080794	0.990839253	5.00E-10
TUCP_000235	XLOC_007153	ENSRNOT00000014456	0.991697153	3.06E-10
TUCP_000235	XLOC_007153	ENSRNOT00000031205	0.991394766	3.66E-10
TUCP_000235	XLOC_007153	ENSRNOT00000044145	1	0
TUCP_000235	XLOC_007153	ENSRNOT00000088381	0.989013276	1.24E-09
TUCP_000235	XLOC_007153	ENSRNOT00000011743	0.98678427	3.11E-09
TUCP_000235	XLOC_007153	ENSRNOT00000089929	1	0
TUCP_000235	XLOC_007153	ENSRNOT00000088624	1	0
TUCP_000235	XLOC_007153	ENSRNOT00000085659	0.989908621	8.10E-10
TUCP_000235	XLOC_007153	ENSRNOT00000015866	0.994066664	5.73E-11
TUCP_000235	XLOC_007153	ENSRNOT00000078633	1	0
TUCP_000235	XLOC_007153	ENSRNOT00000034907	0.995318542	1.76E-11
TUCP_000235	XLOC_007153	ENSRNOT00000093230	0.958295175	9.26E-07
TUCP_000235	XLOC_007153	ENSRNOT00000051835	1	0
TUCP_000235	XLOC_007153	ENSRNOT00000079087	1	0
TUCP_000235	XLOC_007153	ENSRNOT00000037206	1	0
TUCP_000235	XLOC_007153	ENSRNOT00000083261	0.970991658	1.54E-07
TUCP_000235	XLOC_007153	ENSRNOT00000080590	0.999889216	1.31E-19
TUCP_000235	XLOC_007153	ENSRNOT00000027552	0.999801024	2.46E-18
TUCP_000235	XLOC_007153	ENSRNOT00000077014	0.995074376	2.26E-11
TUCP_000235	XLOC_007153	ENSRNOT00000080453	0.99994332	4.61E-21
TUCP_000235	XLOC_007153	ENSRNOT00000089057	0.968243551	2.41E-07
TUCP_000235	XLOC_007153	ENSRNOT00000066839	0.991868612	2.76E-10
TUCP_000235	XLOC_007153	ENSRNOT00000061284	0.955566942	1.27E-06
TUCP_000235	XLOC_007153	ENSRNOT00000013423	0.959677621	7.84E-07
TUCP_000235	XLOC_007153	ENSRNOT00000071884	0.974769955	7.72E-08
TUCP_000235	XLOC_007153	ENSRNOT00000004128	0.961829362	5.99E-07
TUCP_000235	XLOC_007153	ENSRNOT00000026316	0.964408046	4.24E-07
TUCP_000235	XLOC_007153	ENSRNOT00000057729	0.999384184	6.97E-16
TUCP_000235	XLOC_007153	ENSRNOT00000078663	0.975192577	7.10E-08
TUCP_000235	XLOC_007153	ENSRNOT00000064250	0.983131977	1.05E-08
TUCP_000235	XLOC_007153	ENSRNOT00000021608	0.965228881	3.78E-07
TUCP_000235	XLOC_007153	ENSRNOT00000029634	0.995816134	1.00E-11
TUCP_000235	XLOC_007153	ENSRNOT00000093579	0.958564264	8.97E-07
TUCP_000235	XLOC_007153	ENSRNOT00000086349	0.957242511	1.05E-06
TUCP_000235	XLOC_007153	ENSRNOT00000065460	1	0
TUCP_000235	XLOC_007153	ENSRNOT00000003817	0.996778712	2.72E-12
TUCP_000235	XLOC_007153	ENSRNOT00000016713	1	0
TUCP_000235	XLOC_007153	ENSRNOT00000079260	0.996448245	4.42E-12
TUCP_000235	XLOC_007153	ENSRNOT00000039294	1	0
TUCP_000235	XLOC_007153	ENSRNOT00000092913	0.952980228	1.67E-06
TUCP_000234	XLOC_007153	ENSRNOT00000088424	-0.95288828	1.69E-06

TUCP_004615	XLOC_203544	ENSRNOT00000054794	0.95583962	1.23E-06
TUCP_004615	XLOC_203544	ENSRNOT00000025434	0.960438909	7.14E-07
TUCP_004615	XLOC_203544	ENSRNOT00000092665	0.950642061	2.12E-06
TUCP_004614	XLOC_203501	ENSRNOT00000085110	0.989190073	1.14E-09
TUCP_004614	XLOC_203501	ENSRNOT00000008037	0.965549911	3.61E-07
TUCP_004614	XLOC_203501	ENSRNOT00000087720	0.999223088	2.23E-15
TUCP_004614	XLOC_203501	ENSRNOT00000083850	0.960939273	6.71E-07
TUCP_004340	XLOC_191424	ENSRNOT00000019886	0.9532334	1.63E-06
TUCP_004340	XLOC_191424	ENSRNOT00000089062	0.953510634	1.58E-06
TUCP_004340	XLOC_191424	ENSRNOT00000005486	-0.958211196	9.36E-07
TUCP_004340	XLOC_191424	ENSRNOT00000037363	-0.951003991	2.05E-06
TUCP_004340	XLOC_191424	ENSRNOT00000037039	-0.952086343	1.83E-06
TUCP_004340	XLOC_191424	ENSRNOT00000082999	-0.955314081	1.30E-06
TUCP_004340	XLOC_191424	ENSRNOT00000033324	0.953290078	1.62E-06
TUCP_004340	XLOC_191424	ENSRNOT00000081688	-0.960879693	6.76E-07
TUCP_004340	XLOC_191424	ENSRNOT00000061253	0.956491133	1.14E-06
TUCP_004340	XLOC_191424	ENSRNOT00000004972	0.965866385	3.45E-07
TUCP_004340	XLOC_191424	ENSRNOT00000071166	0.951035434	2.04E-06
TUCP_004340	XLOC_191424	ENSRNOT00000072007	-0.965629087	3.57E-07
TUCP_004340	XLOC_191424	ENSRNOT00000017556	0.951509404	1.95E-06
TUCP_004340	XLOC_191424	ENSRNOT00000010381	0.957129137	1.06E-06
TUCP_004340	XLOC_191424	ENSRNOT00000080608	0.952520156	1.75E-06
TUCP_004592	XLOC_201761	ENSRNOT00000016975	0.967366441	2.76E-07
TUCP_004592	XLOC_201761	ENSRNOT00000065168	0.950594276	2.13E-06
TUCP_004592	XLOC_201761	ENSRNOT00000006175	0.964699636	4.07E-07
TUCP_004592	XLOC_201761	ENSRNOT00000080557	0.954193248	1.47E-06
TUCP_004592	XLOC_201761	ENSRNOT00000020134	0.954648365	1.40E-06
TUCP_004592	XLOC_201761	ENSRNOT00000051265	0.955576261	1.26E-06
TUCP_004592	XLOC_201761	ENSRNOT00000022635	0.969562328	1.96E-07
TUCP_004221	XLOC_185327	ENSRNOT00000003921	0.953296354	1.62E-06
TUCP_004590	XLOC_201699	ENSRNOT00000076331	0.957881756	9.72E-07
TUCP_004591	XLOC_201760	ENSRNOT00000033468	0.951295536	1.99E-06
TUCP_004591	XLOC_201760	ENSRNOT00000085840	0.951615715	1.92E-06
TUCP_004597	XLOC_201951	ENSRNOT00000021437	0.950364402	2.18E-06
TUCP_004597	XLOC_201951	ENSRNOT00000012253	0.954238506	1.46E-06
TUCP_004597	XLOC_201951	ENSRNOT00000076680	0.952970968	1.67E-06
TUCP_004597	XLOC_201951	ENSRNOT00000016384	0.956487254	1.14E-06
TUCP_004597	XLOC_201951	ENSRNOT00000011174	0.958568335	8.97E-07
TUCP_004597	XLOC_201951	ENSRNOT00000088996	-0.953167361	1.64E-06
TUCP_004597	XLOC_201951	ENSRNOT00000022747	-0.971513564	1.41E-07
TUCP_004597	XLOC_201951	ENSRNOT00000085501	0.963788317	4.61E-07
TUCP_004597	XLOC_201951	ENSRNOT00000011442	0.951927721	1.86E-06
TUCP_003906	XLOC_172034	ENSRNOT00000000713	0.962927621	5.18E-07
TUCP_003906	XLOC_172034	ENSRNOT00000009877	0.950432109	2.17E-06
TUCP_003906	XLOC_172034	ENSRNOT00000086336	0.9564275	1.15E-06
TUCP_003906	XLOC_172034	ENSRNOT00000026065	0.955837362	1.23E-06
TUCP_003906	XLOC_172034	ENSRNOT00000012056	0.951252611	2.00E-06
TUCP_003906	XLOC_172034	ENSRNOT00000022726	0.967281545	2.80E-07
TUCP_003906	XLOC_172034	ENSRNOT00000044635	0.956267829	1.17E-06
TUCP_003906	XLOC_172034	ENSRNOT00000021116	0.955070369	1.34E-06
TUCP_003906	XLOC_172034	ENSRNOT00000043216	0.954771986	1.38E-06

TUCP_003906	XLOC_172034	ENSRNOT00000080654	0.959775441	7.75E-07
TUCP_003906	XLOC_172034	ENSRNOT00000027414	0.966069699	3.35E-07
TUCP_003906	XLOC_172034	ENSRNOT00000028563	0.971674872	1.37E-07
TUCP_003906	XLOC_172034	ENSRNOT00000006195	0.965712177	3.52E-07
TUCP_003906	XLOC_172034	ENSRNOT00000022800	0.974544194	8.07E-08
TUCP_003906	XLOC_172034	ENSRNOT00000076516	0.959216457	8.30E-07
TUCP_003906	XLOC_172034	ENSRNOT00000015073	0.960601372	7.00E-07
TUCP_003906	XLOC_172034	ENSRNOT00000091807	0.970492008	1.68E-07
TUCP_003906	XLOC_172034	ENSRNOT00000013046	-0.957275231	1.04E-06
TUCP_003906	XLOC_172034	ENSRNOT00000012379	0.951008227	2.05E-06
TUCP_003906	XLOC_172034	ENSRNOT00000011384	0.972016178	1.29E-07
TUCP_003906	XLOC_172034	ENSRNOT00000082691	0.963082612	5.08E-07
TUCP_003906	XLOC_172034	ENSRNOT00000017948	0.953545087	1.58E-06
TUCP_003906	XLOC_172034	ENSRNOT00000006963	0.972523845	1.18E-07
TUCP_003906	XLOC_172034	ENSRNOT00000079109	0.953384112	1.60E-06
TUCP_003906	XLOC_172034	ENSRNOT00000027944	0.96562507	3.57E-07
TUCP_003906	XLOC_172034	ENSRNOT00000028842	0.961136269	6.54E-07
TUCP_003906	XLOC_172034	ENSRNOT00000004003	0.963976163	4.50E-07
TUCP_003906	XLOC_172034	ENSRNOT00000027828	0.95671747	1.11E-06
TUCP_003906	XLOC_172034	ENSRNOT00000052075	0.95625007	1.17E-06
TUCP_003906	XLOC_172034	ENSRNOT00000085648	0.965978225	3.39E-07
TUCP_003906	XLOC_172034	ENSRNOT00000032944	0.959714436	7.81E-07
TUCP_003906	XLOC_172034	ENSRNOT00000083215	0.96213127	5.75E-07
TUCP_003906	XLOC_172034	ENSRNOT00000093640	0.953821027	1.53E-06
TUCP_003906	XLOC_172034	ENSRNOT00000055187	0.955124567	1.33E-06
TUCP_003906	XLOC_172034	ENSRNOT00000005684	0.956843308	1.10E-06
TUCP_003906	XLOC_172034	ENSRNOT00000078500	0.967725469	2.61E-07
TUCP_003906	XLOC_172034	ENSRNOT00000024060	0.965687241	3.54E-07
TUCP_003906	XLOC_172034	ENSRNOT00000010149	0.950117587	2.24E-06
TUCP_003906	XLOC_172034	ENSRNOT00000028760	0.971056526	1.52E-07
TUCP_003906	XLOC_172034	ENSRNOT00000068633	0.956736805	1.11E-06
TUCP_003906	XLOC_172034	ENSRNOT00000084661	0.955336142	1.30E-06
TUCP_003906	XLOC_172034	ENSRNOT00000012895	0.957192585	1.05E-06
TUCP_003906	XLOC_172034	ENSRNOT00000054874	0.951124709	2.02E-06
TUCP_003906	XLOC_172034	ENSRNOT00000090773	-0.959747482	7.78E-07
TUCP_003906	XLOC_172034	ENSRNOT00000020437	0.963715607	4.66E-07
TUCP_003906	XLOC_172034	ENSRNOT00000006828	0.954889173	1.36E-06
TUCP_003906	XLOC_172034	ENSRNOT00000073346	0.964799	4.01E-07
TUCP_003906	XLOC_172034	ENSRNOT00000005355	0.953778283	1.54E-06
TUCP_003906	XLOC_172034	ENSRNOT00000001242	0.955053073	1.34E-06
TUCP_003906	XLOC_172034	ENSRNOT00000087660	0.981701817	1.57E-08
TUCP_003906	XLOC_172034	ENSRNOT00000013661	0.954609684	1.41E-06
TUCP_003906	XLOC_172034	ENSRNOT00000045083	0.959605187	7.91E-07
TUCP_003906	XLOC_172034	ENSRNOT00000022839	0.971050847	1.53E-07
TUCP_003906	XLOC_172034	ENSRNOT00000034744	0.963845554	4.58E-07
TUCP_003906	XLOC_172034	ENSRNOT00000021153	0.975184701	7.11E-08
TUCP_003906	XLOC_172034	ENSRNOT00000064900	0.959976162	7.56E-07
TUCP_003906	XLOC_172034	ENSRNOT00000074075	0.950192298	2.22E-06
TUCP_003906	XLOC_172034	ENSRNOT00000090170	0.960585649	7.01E-07
TUCP_003906	XLOC_172034	ENSRNOT00000049538	0.984348435	7.21E-09
TUCP_003906	XLOC_172034	ENSRNOT00000005909	0.952959564	1.68E-06

TUCP_003906	XLOC_172034	ENSRNOT00000073337	0.960422674	7.16E-07
TUCP_003906	XLOC_172034	ENSRNOT00000009198	0.960881944	6.75E-07
TUCP_003906	XLOC_172034	ENSRNOT00000027054	0.958442168	9.10E-07
TUCP_003906	XLOC_172034	ENSRNOT00000009129	0.965409716	3.68E-07
TUCP_003906	XLOC_172034	ENSRNOT00000036951	0.95886146	8.66E-07
TUCP_003906	XLOC_172034	ENSRNOT00000044177	0.960784987	6.84E-07
TUCP_003906	XLOC_172034	ENSRNOT00000011663	0.970449418	1.69E-07
TUCP_003906	XLOC_172034	ENSRNOT00000006800	0.951974013	1.86E-06
TUCP_003906	XLOC_172034	ENSRNOT00000088196	0.960059492	7.48E-07
TUCP_003906	XLOC_172034	ENSRNOT00000023645	0.97268395	1.14E-07
TUCP_003906	XLOC_172034	ENSRNOT00000084734	0.959898507	7.63E-07
TUCP_003906	XLOC_172034	ENSRNOT00000005977	0.956775746	1.10E-06
TUCP_003906	XLOC_172034	ENSRNOT00000025394	0.977884734	4.01E-08
TUCP_003906	XLOC_172034	ENSRNOT00000074653	0.955726958	1.24E-06
TUCP_003906	XLOC_172034	ENSRNOT00000031230	0.952139024	1.82E-06
TUCP_003906	XLOC_172034	ENSRNOT00000064320	0.955250809	1.31E-06
TUCP_003906	XLOC_172034	ENSRNOT00000002292	0.953632199	1.56E-06
TUCP_003906	XLOC_172034	ENSRNOT00000009342	0.952173961	1.82E-06
TUCP_003906	XLOC_172034	ENSRNOT00000015076	0.96425832	4.33E-07
TUCP_003906	XLOC_172034	ENSRNOT00000004412	0.953053942	1.66E-06
TUCP_003906	XLOC_172034	ENSRNOT00000019177	0.950026728	2.26E-06
TUCP_003906	XLOC_172034	ENSRNOT00000010927	0.957534901	1.01E-06
TUCP_003906	XLOC_172034	ENSRNOT00000084595	0.953414565	1.60E-06
TUCP_003906	XLOC_172034	ENSRNOT00000022646	0.95964273	7.88E-07
TUCP_003906	XLOC_172034	ENSRNOT00000066593	0.964397526	4.24E-07
TUCP_003906	XLOC_172034	ENSRNOT00000027964	0.955901588	1.22E-06
TUCP_003906	XLOC_172034	ENSRNOT00000024659	0.950566692	2.14E-06
TUCP_003906	XLOC_172034	ENSRNOT00000024420	0.957347717	1.03E-06
TUCP_003906	XLOC_172034	ENSRNOT00000052288	0.951120153	2.02E-06
TUCP_003906	XLOC_172034	ENSRNOT00000048029	0.955915245	1.22E-06
TUCP_003906	XLOC_172034	ENSRNOT00000010433	0.958501828	9.04E-07
TUCP_004227	XLOC_185579	ENSRNOT00000024186	0.956241766	1.17E-06
TUCP_004227	XLOC_185579	ENSRNOT00000004876	0.953750079	1.54E-06
TUCP_004227	XLOC_185579	ENSRNOT00000031205	0.963337393	4.91E-07
TUCP_004227	XLOC_185579	ENSRNOT00000079260	0.960729582	6.89E-07
TUCP_004227	XLOC_185579	ENSRNOT00000088381	0.961122895	6.55E-07
TUCP_004227	XLOC_185579	ENSRNOT00000037094	0.956642876	1.12E-06
TUCP_004227	XLOC_185579	ENSRNOT00000046842	0.953793875	1.53E-06
TUCP_004227	XLOC_185579	ENSRNOT00000011743	0.957322003	1.04E-06
TUCP_004227	XLOC_185579	ENSRNOT00000081235	0.951489453	1.95E-06
TUCP_004227	XLOC_185579	ENSRNOT00000078663	0.950404265	2.17E-06
TUCP_004227	XLOC_185579	ENSRNOT00000027552	0.950000092	2.26E-06
TUCP_004227	XLOC_185579	ENSRNOT00000003817	0.951057691	2.04E-06
TUCP_004227	XLOC_185579	ENSRNOT00000091315	0.954000079	1.50E-06
TUCP_004227	XLOC_185579	ENSRNOT00000024337	0.957281691	1.04E-06
TUCP_004227	XLOC_185579	ENSRNOT00000077014	0.961364216	6.35E-07
TUCP_004227	XLOC_185579	ENSRNOT00000046585	0.959969918	7.57E-07
TUCP_004227	XLOC_185579	ENSRNOT00000089934	0.969090665	2.11E-07
TUCP_004227	XLOC_185579	ENSRNOT00000064250	0.964800027	4.01E-07
TUCP_004180	XLOC_182944	ENSRNOT00000002403	0.952058782	1.84E-06
TUCP_004180	XLOC_182944	ENSRNOT00000018179	0.971318025	1.46E-07

TUCP_004180	XLOC_182944	ENSRNOT00000024393	0.960923309	6.72E-07
TUCP_004180	XLOC_182944	ENSRNOT00000086917	0.960576609	7.02E-07
TUCP_004180	XLOC_182944	ENSRNOT00000050180	0.963724462	4.65E-07
TUCP_004180	XLOC_182944	ENSRNOT00000041494	0.963734732	4.65E-07
TUCP_004180	XLOC_182944	ENSRNOT00000002982	0.950252682	2.21E-06
TUCP_004180	XLOC_182944	ENSRNOT00000081512	0.950889416	2.07E-06
TUCP_004180	XLOC_182944	ENSRNOT00000023302	0.964530808	4.17E-07
TUCP_004180	XLOC_182944	ENSRNOT00000002560	0.950696076	2.11E-06
TUCP_004180	XLOC_182944	ENSRNOT00000045223	0.952093089	1.83E-06
TUCP_004180	XLOC_182944	ENSRNOT00000066024	0.950760797	2.10E-06
TUCP_004180	XLOC_182944	ENSRNOT00000086905	0.953705169	1.55E-06
TUCP_004180	XLOC_182944	ENSRNOT00000075621	0.954934158	1.36E-06
TUCP_004180	XLOC_182944	ENSRNOT00000047093	0.96422472	4.35E-07
TUCP_004183	XLOC_183135	ENSRNOT00000087686	-0.958661761	8.87E-07
TUCP_004187	XLOC_183713	ENSRNOT00000057752	0.964077841	4.43E-07
TUCP_004187	XLOC_183713	ENSRNOT00000007144	0.965361005	3.71E-07
TUCP_004187	XLOC_183713	ENSRNOT00000059157	0.95173604	1.90E-06
TUCP_004187	XLOC_183713	ENSRNOT00000024149	0.952793911	1.71E-06
TUCP_004187	XLOC_183713	ENSRNOT00000087697	0.952791559	1.71E-06
TUCP_004187	XLOC_183713	ENSRNOT00000081899	-0.951998958	1.85E-06
TUCP_004187	XLOC_183713	ENSRNOT00000077767	0.96428725	4.31E-07
TUCP_004187	XLOC_183713	ENSRNOT00000050179	0.957789074	9.83E-07
TUCP_004187	XLOC_183713	ENSRNOT00000019687	0.950325688	2.19E-06
TUCP_004187	XLOC_183713	ENSRNOT00000027511	0.958264774	9.30E-07
TUCP_004187	XLOC_183713	ENSRNOT00000020322	0.951793601	1.89E-06
TUCP_004187	XLOC_183713	ENSRNOT00000019630	0.961935339	5.90E-07
TUCP_004187	XLOC_183713	ENSRNOT00000008854	0.959574836	7.94E-07
TUCP_004187	XLOC_183713	ENSRNOT00000027630	0.97278528	1.12E-07
TUCP_004187	XLOC_183713	ENSRNOT00000086812	0.961689332	6.09E-07
TUCP_004187	XLOC_183713	ENSRNOT00000001912	0.961568345	6.19E-07
TUCP_004187	XLOC_183713	ENSRNOT00000033420	0.962753501	5.30E-07
TUCP_004187	XLOC_183713	ENSRNOT00000017353	0.95317925	1.64E-06
TUCP_004187	XLOC_183713	ENSRNOT00000003837	0.977769583	4.12E-08
TUCP_004187	XLOC_183713	ENSRNOT00000010712	-0.953468394	1.59E-06
TUCP_004187	XLOC_183713	ENSRNOT00000085444	0.953528979	1.58E-06
TUCP_004187	XLOC_183713	ENSRNOT00000091065	0.957850893	9.76E-07
TUCP_004186	XLOC_183530	ENSRNOT00000025410	0.950748485	2.10E-06
TUCP_004186	XLOC_183530	ENSRNOT00000075444	-0.961575977	6.18E-07
TUCP_004186	XLOC_183530	ENSRNOT00000063956	0.951099149	2.03E-06
TUCP_004186	XLOC_183530	ENSRNOT00000013743	0.95185234	1.88E-06
TUCP_004186	XLOC_183530	ENSRNOT00000082562	0.955498418	1.28E-06
TUCP_004186	XLOC_183530	ENSRNOT00000006969	0.971144671	1.50E-07
TUCP_004186	XLOC_183530	ENSRNOT00000058206	0.960229228	7.33E-07
TUCP_004186	XLOC_183530	ENSRNOT00000040540	0.955660069	1.25E-06
TUCP_004186	XLOC_183530	ENSRNOT00000071540	0.951271094	1.99E-06
TUCP_004186	XLOC_183530	ENSRNOT00000048613	0.969093195	2.11E-07
TUCP_004186	XLOC_183530	ENSRNOT00000023437	0.954684691	1.39E-06
TUCP_004186	XLOC_183530	ENSRNOT00000036666	0.968000481	2.50E-07
TUCP_004186	XLOC_183530	ENSRNOT00000087408	0.95412281	1.48E-06
TUCP_004186	XLOC_183530	ENSRNOT00000009135	0.962848445	5.24E-07
TUCP_004186	XLOC_183530	ENSRNOT00000020795	0.976077272	5.93E-08

TUCP_004186	XLOC_183530	ENSRNOT00000038703	0.950137465	2.23E-06
TUCP_004186	XLOC_183530	ENSRNOT00000037405	0.960381533	7.19E-07
TUCP_004186	XLOC_183530	ENSRNOT00000091488	0.952261507	1.80E-06
TUCP_004186	XLOC_183530	ENSRNOT00000085060	0.97971202	2.62E-08
TUCP_004186	XLOC_183530	ENSRNOT00000066032	0.958607675	8.93E-07
TUCP_004186	XLOC_183530	ENSRNOT00000015479	0.963263346	4.95E-07
TUCP_004186	XLOC_183530	ENSRNOT00000019876	0.962316293	5.62E-07
TUCP_004186	XLOC_183530	ENSRNOT00000001507	0.956099777	1.19E-06
TUCP_004186	XLOC_183530	ENSRNOT00000076085	-0.957048575	1.07E-06
TUCP_004186	XLOC_183530	ENSRNOT00000035277	0.958699188	8.83E-07
TUCP_004186	XLOC_183530	ENSRNOT00000019921	0.952204574	1.81E-06
TUCP_004186	XLOC_183530	ENSRNOT00000010224	0.956325758	1.16E-06
TUCP_004186	XLOC_183530	ENSRNOT00000016205	0.960445799	7.13E-07
TUCP_004186	XLOC_183530	ENSRNOT00000009634	-0.967314197	2.78E-07
TUCP_004186	XLOC_183530	ENSRNOT00000004486	0.969028428	2.13E-07
TUCP_004186	XLOC_183530	ENSRNOT00000009156	0.952666708	1.73E-06
TUCP_004186	XLOC_183530	ENSRNOT00000074395	0.96880478	2.21E-07
TUCP_004186	XLOC_183530	ENSRNOT00000037685	0.950313611	2.19E-06
TUCP_004186	XLOC_183530	ENSRNOT00000054948	0.966269612	3.25E-07
TUCP_000591	XLOC_021652	ENSRNOT00000084075	0.996106112	7.00E-12
TUCP_000591	XLOC_021652	ENSRNOT00000012880	0.968043129	2.49E-07
TUCP_000591	XLOC_021652	ENSRNOT00000074465	1	0
TUCP_000591	XLOC_021652	ENSRNOT00000090898	0.961670541	6.11E-07
TUCP_000591	XLOC_021652	ENSRNOT00000079805	0.997980451	2.64E-13
TUCP_000591	XLOC_021652	ENSRNOT00000040912	1	0
TUCP_000591	XLOC_021652	ENSRNOT00000072648	1	0
TUCP_000591	XLOC_021652	ENSRNOT00000007398	0.989054103	1.21E-09
TUCP_000591	XLOC_021652	ENSRNOT00000070803	1	0
TUCP_000591	XLOC_021652	ENSRNOT00000012196	1	0
TUCP_000591	XLOC_021652	ENSRNOT00000085772	1	0
TUCP_000591	XLOC_021652	ENSRNOT00000093284	0.999975856	6.46E-23
TUCP_000591	XLOC_021652	ENSRNOT00000086374	0.993568904	8.57E-11
TUCP_000591	XLOC_021652	ENSRNOT00000015270	1	0
TUCP_000591	XLOC_021652	ENSRNOT00000046192	1	0
TUCP_000591	XLOC_021652	ENSRNOT00000044226	1	0
TUCP_000591	XLOC_021652	ENSRNOT00000081898	0.989837012	8.39E-10
TUCP_000591	XLOC_021652	ENSRNOT00000081954	0.964005219	4.48E-07
TUCP_000591	XLOC_021652	ENSRNOT00000080291	0.998427671	7.55E-14
TUCP_000591	XLOC_021652	ENSRNOT00000004293	0.984239049	7.46E-09
TUCP_000591	XLOC_021652	ENSRNOT00000078231	0.960455489	7.13E-07
TUCP_000591	XLOC_021652	ENSRNOT00000002834	1	0
TUCP_000591	XLOC_021652	ENSRNOT00000001464	1	0
TUCP_000591	XLOC_021652	ENSRNOT00000090716	0.994769629	3.06E-11
TUCP_000591	XLOC_021652	ENSRNOT00000083542	0.972568514	1.17E-07
TUCP_000591	XLOC_021652	ENSRNOT00000076044	1	0
TUCP_000591	XLOC_021652	ENSRNOT00000074353	1	0
TUCP_000591	XLOC_021652	ENSRNOT00000043510	0.958144129	9.43E-07
TUCP_000591	XLOC_021652	ENSRNOT00000087122	0.980089513	2.38E-08
TUCP_000591	XLOC_021652	ENSRNOT00000074295	0.987777006	2.11E-09
TUCP_000591	XLOC_021652	ENSRNOT00000057873	1	0
TUCP_000591	XLOC_021652	ENSRNOT00000083002	0.988874897	1.32E-09

TUCP_000591	XLOC_021652	ENSRNOT00000088399	0.999999855	4.99E-34
TUCP_000591	XLOC_021652	ENSRNOT00000086248	1	0
TUCP_000591	XLOC_021652	ENSRNOT00000089750	0.999992758	1.57E-25
TUCP_000591	XLOC_021652	ENSRNOT00000030517	0.971225224	1.48E-07
TUCP_000591	XLOC_021652	ENSRNOT00000034772	0.994265187	4.84E-11
TUCP_000591	XLOC_021652	ENSRNOT00000089305	0.969641693	1.93E-07
TUCP_000591	XLOC_021652	ENSRNOT00000024580	0.954183021	1.47E-06
TUCP_000591	XLOC_021652	ENSRNOT00000064601	0.966578038	3.11E-07
TUCP_000591	XLOC_021652	ENSRNOT00000015870	0.965901306	3.43E-07
TUCP_000591	XLOC_021652	ENSRNOT00000080812	0.992998865	1.31E-10
TUCP_000591	XLOC_021652	ENSRNOT00000092755	1	0
TUCP_000591	XLOC_021652	ENSRNOT00000031683	1	0
TUCP_000591	XLOC_021652	ENSRNOT00000087764	0.988156196	1.80E-09
TUCP_000198	XLOC_006309	ENSRNOT00000085599	0.999938502	6.93E-21
TUCP_000198	XLOC_006309	ENSRNOT00000006689	0.9944189	4.22E-11
TUCP_000198	XLOC_006309	ENSRNOT00000081534	0.987999291	1.92E-09
TUCP_000198	XLOC_006309	ENSRNOT00000085301	0.99793679	2.93E-13
TUCP_000198	XLOC_006309	ENSRNOT00000038729	1	1.33E-79
TUCP_000198	XLOC_006309	ENSRNOT00000086385	1	1.33E-79
TUCP_000198	XLOC_006309	ENSRNOT00000077695	0.96038616	7.19E-07
TUCP_000198	XLOC_006309	ENSRNOT00000078561	0.986503348	3.45E-09
TUCP_000198	XLOC_006309	ENSRNOT00000087613	1	1.33E-79
TUCP_000198	XLOC_006309	ENSRNOT00000026032	0.953050675	1.66E-06
TUCP_000198	XLOC_006309	ENSRNOT00000073350	0.9658754	3.44E-07
TUCP_000198	XLOC_006309	ENSRNOT00000023062	1	0
TUCP_000198	XLOC_006309	ENSRNOT00000058834	1	0
TUCP_000198	XLOC_006309	ENSRNOT00000079420	1	1.33E-79
TUCP_000198	XLOC_006309	ENSRNOT00000090761	1	1.33E-79
TUCP_000198	XLOC_006309	ENSRNOT00000055923	1	0
TUCP_000198	XLOC_006309	ENSRNOT00000092416	0.963085379	5.07E-07
TUCP_000198	XLOC_006309	ENSRNOT00000071930	1	4.25E-78
TUCP_000198	XLOC_006309	ENSRNOT00000001996	0.974721715	7.79E-08
TUCP_000198	XLOC_006309	ENSRNOT00000076756	1	0
TUCP_000198	XLOC_006309	ENSRNOT00000081527	0.999420479	5.14E-16
TUCP_000198	XLOC_006309	ENSRNOT00000085067	0.960262084	7.30E-07
TUCP_000198	XLOC_006309	ENSRNOT00000091663	0.99642667	4.56E-12
TUCP_000198	XLOC_006309	ENSRNOT00000081780	1	0
TUCP_000198	XLOC_006309	ENSRNOT00000093689	0.953599805	1.57E-06
TUCP_000198	XLOC_006309	ENSRNOT00000089114	1	4.25E-78
TUCP_000198	XLOC_006309	ENSRNOT00000021609	0.977017895	4.86E-08
TUCP_000198	XLOC_006309	ENSRNOT00000073316	1	0
TUCP_000198	XLOC_006309	ENSRNOT00000086557	0.977413455	4.46E-08
TUCP_000198	XLOC_006309	ENSRNOT00000071663	1	0
TUCP_000198	XLOC_006309	ENSRNOT00000042860	1	1.33E-79
TUCP_000198	XLOC_006309	ENSRNOT00000072962	0.971287317	1.46E-07
TUCP_000198	XLOC_006309	ENSRNOT00000088421	1	0
TUCP_000198	XLOC_006309	ENSRNOT00000038898	1	0
TUCP_000198	XLOC_006309	ENSRNOT00000015840	0.959452012	8.06E-07
TUCP_000198	XLOC_006309	ENSRNOT00000064936	0.985957557	4.20E-09
TUCP_000198	XLOC_006309	ENSRNOT00000042777	0.990642579	5.56E-10
TUCP_000198	XLOC_006309	ENSRNOT00000010029	1	0

TUCP_003766	XLOC_165235	ENSRNOT00000024137	0.962283604	5.64E-07
TUCP_003766	XLOC_165235	ENSRNOT00000051265	0.958579153	8.96E-07
TUCP_003766	XLOC_165235	ENSRNOT00000036721	0.959555894	7.96E-07
TUCP_003766	XLOC_165235	ENSRNOT00000050485	0.985384177	5.13E-09
TUCP_003766	XLOC_165235	ENSRNOT00000076227	0.951501347	1.95E-06
TUCP_000190	XLOC_006177	ENSRNOT00000025981	0.959364003	8.15E-07
TUCP_000190	XLOC_006177	ENSRNOT00000010992	0.950590908	2.13E-06
TUCP_000190	XLOC_006177	ENSRNOT00000067528	0.95492013	1.36E-06
TUCP_000190	XLOC_006177	ENSRNOT00000060186	0.985028224	5.78E-09
TUCP_000190	XLOC_006177	ENSRNOT00000076669	0.961799069	6.01E-07
TUCP_000190	XLOC_006177	ENSRNOT00000081246	0.956787011	1.10E-06
TUCP_000190	XLOC_006177	ENSRNOT00000055401	0.960054681	7.49E-07
TUCP_000190	XLOC_006177	ENSRNOT00000020134	0.950158381	2.23E-06
TUCP_000190	XLOC_006177	ENSRNOT00000074939	0.95390483	1.52E-06
TUCP_000190	XLOC_006177	ENSRNOT00000034267	0.95254786	1.75E-06
TUCP_000190	XLOC_006177	ENSRNOT00000017021	0.95517205	1.32E-06
TUCP_000190	XLOC_006177	ENSRNOT00000031230	0.961900304	5.93E-07
TUCP_000190	XLOC_006177	ENSRNOT00000032274	0.971017941	1.53E-07
TUCP_000190	XLOC_006177	ENSRNOT00000004508	0.950519797	2.15E-06
TUCP_000190	XLOC_006177	ENSRNOT00000006175	0.959508569	8.01E-07
TUCP_000190	XLOC_006177	ENSRNOT00000043535	0.952538316	1.75E-06
TUCP_000190	XLOC_006177	ENSRNOT00000039809	0.952661243	1.73E-06
TUCP_000190	XLOC_006177	ENSRNOT00000005262	0.950964904	2.06E-06
TUCP_000190	XLOC_006177	ENSRNOT00000023490	0.958721815	8.81E-07
TUCP_000190	XLOC_006177	ENSRNOT00000076729	0.957245882	1.05E-06
TUCP_000190	XLOC_006177	ENSRNOT00000060819	0.95245791	1.77E-06
TUCP_000190	XLOC_006177	ENSRNOT00000003991	0.961912332	5.92E-07
TUCP_000190	XLOC_006177	ENSRNOT00000066670	0.959241347	8.27E-07
TUCP_000190	XLOC_006177	ENSRNOT00000076763	0.963200527	5.00E-07
TUCP_000190	XLOC_006177	ENSRNOT00000012895	0.970842411	1.58E-07
TUCP_000190	XLOC_006177	ENSRNOT00000025859	0.957082728	1.07E-06
TUCP_000190	XLOC_006177	ENSRNOT00000071697	0.964830464	3.99E-07
TUCP_000190	XLOC_006177	ENSRNOT00000084240	0.95652834	1.14E-06
TUCP_000190	XLOC_006177	ENSRNOT00000090918	0.952746098	1.71E-06
TUCP_000190	XLOC_006177	ENSRNOT00000000676	0.958031172	9.56E-07
TUCP_000190	XLOC_006177	ENSRNOT00000027452	0.959103671	8.41E-07
TUCP_000190	XLOC_006177	ENSRNOT00000004412	0.954120358	1.48E-06
TUCP_000190	XLOC_006177	ENSRNOT00000067181	0.954203362	1.47E-06
TUCP_000190	XLOC_006177	ENSRNOT00000077465	0.950792201	2.09E-06
TUCP_000190	XLOC_006177	ENSRNOT00000045882	0.978675122	3.35E-08
TUCP_000190	XLOC_006177	ENSRNOT00000066736	0.965809292	3.47E-07
TUCP_000190	XLOC_006177	ENSRNOT00000071241	0.950633844	2.12E-06
TUCP_000190	XLOC_006177	ENSRNOT00000025832	0.956366518	1.16E-06
TUCP_000190	XLOC_006177	ENSRNOT00000034704	0.960853167	6.78E-07
TUCP_000190	XLOC_006177	ENSRNOT00000013274	0.967410488	2.74E-07
TUCP_000190	XLOC_006177	ENSRNOT00000085027	0.960367239	7.20E-07
TUCP_000190	XLOC_006177	ENSRNOT00000029866	0.96457642	4.14E-07
TUCP_000190	XLOC_006177	ENSRNOT00000020437	0.951391343	1.97E-06
TUCP_000190	XLOC_006177	ENSRNOT00000010898	0.971594318	1.39E-07
TUCP_000190	XLOC_006177	ENSRNOT00000023428	0.966735741	3.03E-07
TUCP_000190	XLOC_006177	ENSRNOT00000029167	0.969068229	2.12E-07

TUCP_000190	XLOC_006177	ENSRNOT00000071764	0.965450237	3.66E-07
TUCP_000190	XLOC_006177	ENSRNOT00000027762	0.961845359	5.97E-07
TUCP_000190	XLOC_006177	ENSRNOT00000075326	0.954520028	1.42E-06
TUCP_000190	XLOC_006177	ENSRNOT00000021348	0.956673488	1.12E-06
TUCP_000190	XLOC_006177	ENSRNOT00000091265	0.963425373	4.85E-07
TUCP_000190	XLOC_006177	ENSRNOT00000092158	0.960996955	6.66E-07
TUCP_000190	XLOC_006177	ENSRNOT00000093714	0.952208718	1.81E-06
TUCP_000191	XLOC_006216	ENSRNOT00000008988	0.957084904	1.07E-06
TUCP_000191	XLOC_006216	ENSRNOT00000090914	0.957480707	1.02E-06
TUCP_000191	XLOC_006216	ENSRNOT00000029572	0.95249542	1.76E-06
TUCP_000191	XLOC_006216	ENSRNOT00000087937	0.966018419	3.37E-07
TUCP_000191	XLOC_006216	ENSRNOT00000039687	0.951500884	1.95E-06
TUCP_000191	XLOC_006216	ENSRNOT00000026106	0.957538844	1.01E-06
TUCP_000191	XLOC_006216	ENSRNOT00000088589	0.958682311	8.85E-07
TUCP_000196	XLOC_006308	ENSRNOT00000081570	0.962961027	5.16E-07
TUCP_000196	XLOC_006308	ENSRNOT00000091652	0.99819185	1.52E-13
TUCP_000196	XLOC_006308	ENSRNOT00000036564	0.972337506	1.22E-07
TUCP_000196	XLOC_006308	ENSRNOT00000000109	0.962179854	5.72E-07
TUCP_000196	XLOC_006308	ENSRNOT00000080865	1	0
TUCP_000196	XLOC_006308	ENSRNOT00000073534	0.995808019	1.01E-11
TUCP_000196	XLOC_006308	ENSRNOT00000012161	0.987544327	2.31E-09
TUCP_000196	XLOC_006308	ENSRNOT00000078707	0.960060506	7.48E-07
TUCP_000196	XLOC_006308	ENSRNOT00000087758	0.968778527	2.22E-07
TUCP_000196	XLOC_006308	ENSRNOT00000049832	0.969039151	2.13E-07
TUCP_000196	XLOC_006308	ENSRNOT00000093019	1	0
TUCP_000196	XLOC_006308	ENSRNOT00000024825	0.980452701	2.18E-08
TUCP_000196	XLOC_006308	ENSRNOT00000059266	0.96033506	7.23E-07
TUCP_000196	XLOC_006308	ENSRNOT00000072734	0.965018115	3.89E-07
TUCP_000196	XLOC_006308	ENSRNOT00000085908	0.996653227	3.29E-12
TUCP_000196	XLOC_006308	ENSRNOT00000046744	1	0
TUCP_000196	XLOC_006308	ENSRNOT00000076429	1	0
TUCP_000196	XLOC_006308	ENSRNOT00000084667	0.999660971	3.53E-17
TUCP_000196	XLOC_006308	ENSRNOT00000055412	1	0
TUCP_000196	XLOC_006308	ENSRNOT00000093201	0.9997022	1.84E-17
TUCP_000196	XLOC_006308	ENSRNOT00000072724	1	1.33E-79
TUCP_000196	XLOC_006308	ENSRNOT00000090323	0.95603782	1.20E-06
TUCP_000196	XLOC_006308	ENSRNOT00000082760	0.986293522	3.72E-09
TUCP_000196	XLOC_006308	ENSRNOT00000066017	0.989785653	8.61E-10
TUCP_000196	XLOC_006308	ENSRNOT00000073148	0.985721397	4.56E-09
TUCP_000196	XLOC_006308	ENSRNOT00000077462	0.960098183	7.45E-07
TUCP_000196	XLOC_006308	ENSRNOT00000084375	1	0
TUCP_000196	XLOC_006308	ENSRNOT00000085447	0.998400682	8.22E-14
TUCP_000196	XLOC_006308	ENSRNOT00000006249	0.966489607	3.15E-07
TUCP_000196	XLOC_006308	ENSRNOT00000087073	0.952450698	1.77E-06
TUCP_000196	XLOC_006308	ENSRNOT00000042369	0.995659369	1.20E-11
TUCP_000196	XLOC_006308	ENSRNOT00000086554	1	0
TUCP_000196	XLOC_006308	ENSRNOT00000054858	0.985228824	5.40E-09
TUCP_000196	XLOC_006308	ENSRNOT00000047921	1	0
TUCP_000196	XLOC_006308	ENSRNOT00000064261	0.999977196	4.86E-23
TUCP_000196	XLOC_006308	ENSRNOT00000086660	0.999208903	2.44E-15
TUCP_000196	XLOC_006308	ENSRNOT00000080933	0.967360681	2.76E-07

TUCP_000196	XLOC_006308	ENSRNOT00000083977	1	0
TUCP_000196	XLOC_006308	ENSRNOT00000092933	1	0
TUCP_000196	XLOC_006308	ENSRNOT00000009061	0.953300672	1.62E-06
TUCP_000196	XLOC_006308	ENSRNOT00000041281	0.987828355	2.06E-09
TUCP_000194	XLOC_006306	ENSRNOT00000046563	0.979015955	3.09E-08
TUCP_001111	XLOC_042311	ENSRNOT00000091072	0.967150136	2.85E-07
TUCP_001111	XLOC_042311	ENSRNOT00000036228	0.952458803	1.77E-06
TUCP_003809	XLOC_166462	ENSRNOT00000024725	0.969897899	1.85E-07
TUCP_000428	XLOC_014415	ENSRNOT00000088728	-0.956540047	1.13E-06
TUCP_000582	XLOC_020637	ENSRNOT00000000008	1	0
TUCP_000582	XLOC_020637	ENSRNOT00000060048	0.998711457	2.79E-14
TUCP_000582	XLOC_020637	ENSRNOT00000082304	0.996875989	2.33E-12
TUCP_000582	XLOC_020637	ENSRNOT00000006219	1	4.25E-78
TUCP_000582	XLOC_020637	ENSRNOT00000065376	1	0
TUCP_000582	XLOC_020637	ENSRNOT00000081433	0.984469223	6.93E-09
TUCP_000582	XLOC_020637	ENSRNOT00000083575	1	0
TUCP_000582	XLOC_020637	ENSRNOT00000087739	1	4.25E-78
TUCP_000582	XLOC_020637	ENSRNOT00000079826	1	0
TUCP_000582	XLOC_020637	ENSRNOT00000025770	0.993828251	6.98E-11
TUCP_000582	XLOC_020637	ENSRNOT00000088244	0.973479481	9.88E-08
TUCP_000582	XLOC_020637	ENSRNOT00000048523	1	0
TUCP_000582	XLOC_020637	ENSRNOT00000079365	0.958936699	8.58E-07
TUCP_000582	XLOC_020637	ENSRNOT00000020229	0.960002785	7.54E-07
TUCP_000582	XLOC_020637	ENSRNOT00000026428	0.996986683	1.95E-12
TUCP_000582	XLOC_020637	ENSRNOT00000085673	1	1.33E-79
TUCP_000582	XLOC_020637	ENSRNOT00000072972	0.981476166	1.67E-08
TUCP_000582	XLOC_020637	ENSRNOT00000050573	1	4.25E-78
TUCP_000582	XLOC_020637	ENSRNOT00000089876	-0.953630353	1.56E-06
TUCP_000582	XLOC_020637	ENSRNOT00000074378	0.998901807	1.26E-14
TUCP_000582	XLOC_020637	ENSRNOT00000093571	1	0
TUCP_000582	XLOC_020637	ENSRNOT00000090581	1	0
TUCP_000582	XLOC_020637	ENSRNOT00000076239	0.98020752	2.31E-08
TUCP_000582	XLOC_020637	ENSRNOT00000065485	1	1.33E-79
TUCP_000582	XLOC_020637	ENSRNOT00000006356	0.999544279	1.55E-16
TUCP_000582	XLOC_020637	ENSRNOT00000087199	0.974421555	8.26E-08
TUCP_000582	XLOC_020637	ENSRNOT00000022091	1	0
TUCP_000582	XLOC_020637	ENSRNOT00000064764	0.98460888	6.63E-09
TUCP_000582	XLOC_020637	ENSRNOT00000076927	0.971163156	1.50E-07
TUCP_000582	XLOC_020637	ENSRNOT00000092395	0.999522978	1.94E-16
TUCP_001115	XLOC_042333	ENSRNOT00000010263	0.966567067	3.11E-07
TUCP_000584	XLOC_020716	ENSRNOT00000013745	0.959193579	8.32E-07
TUCP_000584	XLOC_020716	ENSRNOT00000075397	-0.963770169	4.63E-07
TUCP_000584	XLOC_020716	ENSRNOT00000019104	0.958468914	9.07E-07
TUCP_000584	XLOC_020716	ENSRNOT00000019848	0.978724803	3.31E-08
TUCP_000584	XLOC_020716	ENSRNOT00000077451	0.951544625	1.94E-06
TUCP_000584	XLOC_020716	ENSRNOT00000006172	0.969866796	1.86E-07
TUCP_000584	XLOC_020716	ENSRNOT00000001691	-0.955520405	1.27E-06
TUCP_000584	XLOC_020716	ENSRNOT00000079644	0.958672539	8.86E-07
TUCP_000584	XLOC_020716	ENSRNOT00000020267	-0.961867044	5.96E-07
TUCP_000584	XLOC_020716	ENSRNOT00000079138	-0.952023724	1.85E-06
TUCP_000584	XLOC_020716	ENSRNOT00000064305	0.985438526	5.03E-09

TUCP_000584	XLOC_020716	ENSRNOT00000030914	0.957992814	9.60E-07
TUCP_000584	XLOC_020716	ENSRNOT00000029508	-0.96433886	4.28E-07
TUCP_000584	XLOC_020716	ENSRNOT00000085885	0.979051075	3.07E-08
TUCP_000584	XLOC_020716	ENSRNOT00000045233	0.957502818	1.02E-06
TUCP_000584	XLOC_020716	ENSRNOT00000042633	0.958163845	9.41E-07
TUCP_000584	XLOC_020716	ENSRNOT00000037955	0.950315598	2.19E-06
TUCP_000584	XLOC_020716	ENSRNOT00000025309	-0.958915042	8.60E-07
TUCP_000584	XLOC_020716	ENSRNOT00000025308	0.955408667	1.29E-06
TUCP_000584	XLOC_020716	ENSRNOT00000030850	-0.953376516	1.60E-06
TUCP_000584	XLOC_020716	ENSRNOT00000050063	0.955400864	1.29E-06
TUCP_000584	XLOC_020716	ENSRNOT00000055540	-0.960909343	6.73E-07
TUCP_000584	XLOC_020716	ENSRNOT00000003247	0.96770159	2.62E-07
TUCP_000584	XLOC_020716	ENSRNOT00000085311	0.952896042	1.69E-06
TUCP_000584	XLOC_020716	ENSRNOT00000015986	0.978717092	3.32E-08
TUCP_000584	XLOC_020716	ENSRNOT00000025284	0.979930089	2.48E-08
TUCP_000584	XLOC_020716	ENSRNOT00000072771	-0.961250848	6.45E-07
TUCP_000584	XLOC_020716	ENSRNOT00000022620	-0.959896049	7.64E-07
TUCP_000584	XLOC_020716	ENSRNOT00000057045	0.957097599	1.06E-06
TUCP_000584	XLOC_020716	ENSRNOT00000035320	0.96413341	4.40E-07
TUCP_000584	XLOC_020716	ENSRNOT00000084674	0.957376519	1.03E-06
TUCP_000584	XLOC_020716	ENSRNOT00000003414	0.960443243	7.14E-07
TUCP_000584	XLOC_020716	ENSRNOT00000046807	0.962640494	5.38E-07
TUCP_000584	XLOC_020716	ENSRNOT00000003412	0.971789003	1.34E-07
TUCP_000584	XLOC_020716	ENSRNOT00000077856	0.972877496	1.10E-07
TUCP_000584	XLOC_020716	ENSRNOT00000003794	0.972621931	1.16E-07
TUCP_000584	XLOC_020716	ENSRNOT00000018488	0.959974681	7.56E-07
TUCP_000584	XLOC_020716	ENSRNOT00000089673	0.970784513	1.60E-07
TUCP_000584	XLOC_020716	ENSRNOT00000080522	0.99025227	6.82E-10
TUCP_000584	XLOC_020716	ENSRNOT00000000049	0.961501457	6.24E-07
TUCP_000584	XLOC_020716	ENSRNOT00000000048	-0.962400186	5.56E-07
TUCP_000584	XLOC_020716	ENSRNOT00000075934	0.960071774	7.47E-07
TUCP_000584	XLOC_020716	ENSRNOT00000082686	0.986945972	2.92E-09
TUCP_000584	XLOC_020716	ENSRNOT00000076181	0.950503146	2.15E-06
TUCP_000584	XLOC_020716	ENSRNOT00000017269	0.954555846	1.41E-06
TUCP_000584	XLOC_020716	ENSRNOT00000026462	0.982300456	1.33E-08
TUCP_000584	XLOC_020716	ENSRNOT00000092945	0.962655848	5.37E-07
TUCP_000584	XLOC_020716	ENSRNOT00000029179	0.950378408	2.18E-06
TUCP_000584	XLOC_020716	ENSRNOT00000071388	-0.95588862	1.22E-06
TUCP_000584	XLOC_020716	ENSRNOT00000056145	0.955526899	1.27E-06
TUCP_000584	XLOC_020716	ENSRNOT00000026525	0.960881003	6.76E-07
TUCP_000584	XLOC_020716	ENSRNOT00000084313	0.955214047	1.32E-06
TUCP_000584	XLOC_020716	ENSRNOT00000001401	-0.973198689	1.04E-07
TUCP_000584	XLOC_020716	ENSRNOT00000016132	0.956313333	1.16E-06
TUCP_000584	XLOC_020716	ENSRNOT00000001366	0.992611235	1.71E-10
TUCP_000584	XLOC_020716	ENSRNOT00000001360	0.967909294	2.54E-07
TUCP_000584	XLOC_020716	ENSRNOT00000023506	0.963639187	4.71E-07
TUCP_000584	XLOC_020716	ENSRNOT00000017081	0.960460862	7.12E-07
TUCP_000584	XLOC_020716	ENSRNOT00000002313	0.966232751	3.27E-07
TUCP_000584	XLOC_020716	ENSRNOT00000032363	-0.965885864	3.44E-07
TUCP_000584	XLOC_020716	ENSRNOT00000016637	0.968077005	2.47E-07
TUCP_000584	XLOC_020716	ENSRNOT00000011714	0.975451483	6.74E-08

TUCP_000584	XLOC_020716	ENSRNOT00000045765	0.960064852	7.48E-07
TUCP_000584	XLOC_020716	ENSRNOT00000092876	0.953663231	1.56E-06
TUCP_000584	XLOC_020716	ENSRNOT00000000532	0.98434274	7.22E-09
TUCP_000584	XLOC_020716	ENSRNOT00000002465	-0.96011311	7.44E-07
TUCP_000584	XLOC_020716	ENSRNOT00000079677	0.959809436	7.72E-07
TUCP_000584	XLOC_020716	ENSRNOT00000003992	0.960308593	7.26E-07
TUCP_000584	XLOC_020716	ENSRNOT00000019028	-0.953060002	1.66E-06
TUCP_000584	XLOC_020716	ENSRNOT00000013146	-0.956084863	1.19E-06
TUCP_000584	XLOC_020716	ENSRNOT00000021400	0.979682353	2.64E-08
TUCP_000584	XLOC_020716	ENSRNOT00000012942	-0.952181704	1.82E-06
TUCP_000584	XLOC_020716	ENSRNOT00000086310	0.961702938	6.08E-07
TUCP_000584	XLOC_020716	ENSRNOT00000020951	-0.965424741	3.67E-07
TUCP_000584	XLOC_020716	ENSRNOT00000082146	0.950479793	2.16E-06
TUCP_000584	XLOC_020716	ENSRNOT00000023876	-0.964260914	4.32E-07
TUCP_000584	XLOC_020716	ENSRNOT00000034401	0.956217723	1.18E-06
TUCP_000584	XLOC_020716	ENSRNOT00000012625	0.980354401	2.23E-08
TUCP_000584	XLOC_020716	ENSRNOT00000003207	0.974711494	7.81E-08
TUCP_000584	XLOC_020716	ENSRNOT00000006366	0.972192482	1.25E-07
TUCP_000584	XLOC_020716	ENSRNOT00000033196	0.951450022	1.96E-06
TUCP_000584	XLOC_020716	ENSRNOT00000085911	0.972152574	1.26E-07
TUCP_000584	XLOC_020716	ENSRNOT00000044662	0.972545184	1.17E-07
TUCP_000584	XLOC_020716	ENSRNOT00000064723	0.958686287	8.84E-07
TUCP_000584	XLOC_020716	ENSRNOT00000073834	0.95452762	1.42E-06
TUCP_000584	XLOC_020716	ENSRNOT00000086456	0.968237968	2.41E-07
TUCP_000584	XLOC_020716	ENSRNOT00000036959	0.958050587	9.53E-07
TUCP_000584	XLOC_020716	ENSRNOT00000010367	-0.962340981	5.60E-07
TUCP_000584	XLOC_020716	ENSRNOT00000089185	0.954402679	1.44E-06
TUCP_000584	XLOC_020716	ENSRNOT00000023977	0.963110979	5.06E-07
TUCP_000584	XLOC_020716	ENSRNOT00000067741	0.981401938	1.70E-08
TUCP_000584	XLOC_020716	ENSRNOT00000009260	0.951994798	1.85E-06
TUCP_000584	XLOC_020716	ENSRNOT00000048933	0.950103399	2.24E-06
TUCP_000584	XLOC_020716	ENSRNOT00000006765	0.963965864	4.50E-07
TUCP_000584	XLOC_020716	ENSRNOT00000035385	-0.95137684	1.97E-06
TUCP_000584	XLOC_020716	ENSRNOT00000009994	0.965320098	3.73E-07
TUCP_000584	XLOC_020716	ENSRNOT00000014885	0.961814691	6.00E-07
TUCP_000584	XLOC_020716	ENSRNOT00000005644	0.9522497	1.80E-06
TUCP_000584	XLOC_020716	ENSRNOT00000071192	0.964482377	4.19E-07
TUCP_000584	XLOC_020716	ENSRNOT00000018173	0.964957327	3.92E-07
TUCP_000584	XLOC_020716	ENSRNOT00000040548	0.96705956	2.89E-07
TUCP_000584	XLOC_020716	ENSRNOT00000073239	0.98425965	7.41E-09
TUCP_000584	XLOC_020716	ENSRNOT00000040060	0.979240018	2.93E-08
TUCP_000584	XLOC_020716	ENSRNOT00000010962	0.955910019	1.22E-06
TUCP_000584	XLOC_020716	ENSRNOT00000045545	0.955794684	1.23E-06
TUCP_000584	XLOC_020716	ENSRNOT00000010620	0.956286832	1.17E-06
TUCP_000584	XLOC_020716	ENSRNOT00000039102	-0.964397251	4.24E-07
TUCP_000584	XLOC_020716	ENSRNOT00000078856	0.95044907	2.16E-06
TUCP_000584	XLOC_020716	ENSRNOT00000009742	0.968955827	2.16E-07
TUCP_000584	XLOC_020716	ENSRNOT00000001036	0.961301891	6.40E-07
TUCP_000584	XLOC_020716	ENSRNOT00000007044	0.992894574	1.41E-10
TUCP_000584	XLOC_020716	ENSRNOT00000007369	-0.956606705	1.13E-06
TUCP_000584	XLOC_020716	ENSRNOT00000085466	-0.957601044	1.00E-06

TUCP_000584	XLOC_020716	ENSRNOT00000060757	0.962512606	5.47E-07
TUCP_000584	XLOC_020716	ENSRNOT00000012996	-0.95694869	1.08E-06
TUCP_000584	XLOC_020716	ENSRNOT00000004941	0.958935899	8.58E-07
TUCP_000584	XLOC_020716	ENSRNOT00000070912	0.968605521	2.28E-07
TUCP_000584	XLOC_020716	ENSRNOT00000060595	0.973024737	1.08E-07
TUCP_000584	XLOC_020716	ENSRNOT00000023252	0.95343086	1.59E-06
TUCP_000584	XLOC_020716	ENSRNOT00000088679	0.973676152	9.52E-08
TUCP_000584	XLOC_020716	ENSRNOT00000016776	0.975826127	6.24E-08
TUCP_000584	XLOC_020716	ENSRNOT00000041106	-0.958870567	8.65E-07
TUCP_000584	XLOC_020716	ENSRNOT00000008987	0.961966517	5.88E-07
TUCP_000584	XLOC_020716	ENSRNOT00000090355	0.954436443	1.43E-06
TUCP_000584	XLOC_020716	ENSRNOT00000031935	0.976025633	5.99E-08
TUCP_000584	XLOC_020716	ENSRNOT00000019473	0.967380677	2.75E-07
TUCP_000584	XLOC_020716	ENSRNOT00000066041	0.95746514	1.02E-06
TUCP_000584	XLOC_020716	ENSRNOT00000081835	0.978221048	3.72E-08
TUCP_000584	XLOC_020716	ENSRNOT00000063930	0.955191215	1.32E-06
TUCP_000584	XLOC_020716	ENSRNOT00000013058	-0.953721397	1.55E-06
TUCP_000584	XLOC_020716	ENSRNOT00000013116	0.978558864	3.44E-08
TUCP_000584	XLOC_020716	ENSRNOT00000001855	-0.957415935	1.03E-06
TUCP_000584	XLOC_020716	ENSRNOT00000013025	0.953383934	1.60E-06
TUCP_000584	XLOC_020716	ENSRNOT00000038178	0.979318362	2.88E-08
TUCP_000584	XLOC_020716	ENSRNOT00000016077	-0.953738202	1.54E-06
TUCP_000584	XLOC_020716	ENSRNOT00000027301	0.974388648	8.31E-08
TUCP_000584	XLOC_020716	ENSRNOT00000091050	0.955517238	1.27E-06
TUCP_000584	XLOC_020716	ENSRNOT00000089418	-0.9510664	2.03E-06
TUCP_000584	XLOC_020716	ENSRNOT00000087838	0.962911186	5.19E-07
TUCP_000584	XLOC_020716	ENSRNOT00000028561	-0.95759663	1.01E-06
TUCP_000584	XLOC_020716	ENSRNOT00000009963	-0.957184581	1.05E-06
TUCP_000584	XLOC_020716	ENSRNOT00000067566	0.966786963	3.01E-07
TUCP_000584	XLOC_020716	ENSRNOT00000014479	0.974359764	8.36E-08
TUCP_000584	XLOC_020716	ENSRNOT00000027220	0.963962605	4.51E-07
TUCP_000584	XLOC_020716	ENSRNOT00000003326	0.983121601	1.05E-08
TUCP_000584	XLOC_020716	ENSRNOT00000016306	0.972380421	1.21E-07
TUCP_000584	XLOC_020716	ENSRNOT00000073019	-0.962053878	5.81E-07
TUCP_000584	XLOC_020716	ENSRNOT00000012699	0.95409582	1.49E-06
TUCP_000584	XLOC_020716	ENSRNOT00000004007	0.96874295	2.23E-07
TUCP_000584	XLOC_020716	ENSRNOT00000007821	-0.962441675	5.53E-07
TUCP_000584	XLOC_020716	ENSRNOT00000090338	0.966495497	3.14E-07
TUCP_000584	XLOC_020716	ENSRNOT00000056100	0.955337988	1.30E-06
TUCP_000584	XLOC_020716	ENSRNOT00000001077	-0.953786673	1.54E-06
TUCP_000584	XLOC_020716	ENSRNOT00000036838	0.962676923	5.36E-07
TUCP_000584	XLOC_020716	ENSRNOT00000006443	0.963695032	4.67E-07
TUCP_000584	XLOC_020716	ENSRNOT00000072762	0.952876128	1.69E-06
TUCP_000584	XLOC_020716	ENSRNOT00000012583	0.95349565	1.58E-06
TUCP_000584	XLOC_020716	ENSRNOT00000073643	0.974930923	7.48E-08
TUCP_000584	XLOC_020716	ENSRNOT00000078349	0.958615214	8.92E-07
TUCP_000584	XLOC_020716	ENSRNOT00000022502	0.964391582	4.25E-07
TUCP_000584	XLOC_020716	ENSRNOT00000026928	0.982246552	1.35E-08
TUCP_000584	XLOC_020716	ENSRNOT00000010252	0.965594853	3.58E-07
TUCP_000584	XLOC_020716	ENSRNOT00000021802	-0.958274833	9.29E-07
TUCP_000584	XLOC_020716	ENSRNOT00000025856	0.95847496	9.07E-07

TUCP_000584	XLOC_020716	ENSRNOT00000039850	0.962365001	5.58E-07
TUCP_000584	XLOC_020716	ENSRNOT00000078161	0.966368442	3.20E-07
TUCP_000584	XLOC_020716	ENSRNOT00000005694	0.953138883	1.64E-06
TUCP_000584	XLOC_020716	ENSRNOT00000023262	0.95678428	1.10E-06
TUCP_000584	XLOC_020716	ENSRNOT00000008813	0.952183412	1.82E-06
TUCP_000584	XLOC_020716	ENSRNOT00000050415	0.965387422	3.69E-07
TUCP_000584	XLOC_020716	ENSRNOT00000045946	-0.963182112	5.01E-07
TUCP_000584	XLOC_020716	ENSRNOT00000017254	0.953206688	1.63E-06
TUCP_000584	XLOC_020716	ENSRNOT00000055957	0.955222092	1.31E-06
TUCP_000584	XLOC_020716	ENSRNOT00000010674	0.970418546	1.70E-07
TUCP_000584	XLOC_020716	ENSRNOT00000023664	0.96389769	4.55E-07
TUCP_000584	XLOC_020716	ENSRNOT00000016730	0.966483908	3.15E-07
TUCP_000584	XLOC_020716	ENSRNOT00000007314	0.958580406	8.95E-07
TUCP_000584	XLOC_020716	ENSRNOT00000065103	0.976545803	5.37E-08
TUCP_000584	XLOC_020716	ENSRNOT00000064068	0.981539682	1.64E-08
TUCP_000584	XLOC_020716	ENSRNOT00000005170	0.952979345	1.67E-06
TUCP_000584	XLOC_020716	ENSRNOT00000023567	0.964633788	4.11E-07
TUCP_000584	XLOC_020716	ENSRNOT00000068269	0.968500505	2.32E-07
TUCP_000584	XLOC_020716	ENSRNOT00000026398	0.957496855	1.02E-06
TUCP_000584	XLOC_020716	ENSRNOT00000071897	0.977991413	3.92E-08
TUCP_000584	XLOC_020716	ENSRNOT00000050350	0.969634325	1.93E-07
TUCP_000584	XLOC_020716	ENSRNOT00000018494	0.966890812	2.96E-07
TUCP_000584	XLOC_020716	ENSRNOT00000024285	0.954941669	1.36E-06
TUCP_000584	XLOC_020716	ENSRNOT00000071482	0.963908148	4.54E-07
TUCP_000584	XLOC_020716	ENSRNOT00000009136	0.963582273	4.75E-07
TUCP_000584	XLOC_020716	ENSRNOT00000023067	0.979718146	2.61E-08
TUCP_000584	XLOC_020716	ENSRNOT00000027917	-0.95398636	1.50E-06
TUCP_000584	XLOC_020716	ENSRNOT00000010516	0.972028455	1.29E-07
TUCP_000584	XLOC_020716	ENSRNOT00000000527	0.986498309	3.45E-09
TUCP_000584	XLOC_020716	ENSRNOT00000039215	0.958780488	8.74E-07
TUCP_000584	XLOC_020716	ENSRNOT00000067135	0.950198218	2.22E-06
TUCP_000584	XLOC_020716	ENSRNOT00000081110	0.964106597	4.42E-07
TUCP_000584	XLOC_020716	ENSRNOT00000039730	0.954315466	1.45E-06
TUCP_000584	XLOC_020716	ENSRNOT00000028527	0.96689704	2.96E-07
TUCP_000584	XLOC_020716	ENSRNOT00000088202	0.973261384	1.03E-07
TUCP_000584	XLOC_020716	ENSRNOT00000091863	0.960576573	7.02E-07
TUCP_000584	XLOC_020716	ENSRNOT00000082151	0.957141318	1.06E-06
TUCP_000584	XLOC_020716	ENSRNOT00000011572	0.979900296	2.50E-08
TUCP_000584	XLOC_020716	ENSRNOT00000000155	-0.960499639	7.09E-07
TUCP_000584	XLOC_020716	ENSRNOT00000001607	-0.961507285	6.24E-07
TUCP_000584	XLOC_020716	ENSRNOT00000036282	-0.965669593	3.55E-07
TUCP_000584	XLOC_020716	ENSRNOT00000040629	-0.968833347	2.20E-07
TUCP_000584	XLOC_020716	ENSRNOT00000046126	0.975210072	7.07E-08
TUCP_000584	XLOC_020716	ENSRNOT00000077479	0.977726015	4.16E-08
TUCP_000584	XLOC_020716	ENSRNOT00000022142	-0.959660879	7.86E-07
TUCP_000584	XLOC_020716	ENSRNOT00000030007	0.988844832	1.34E-09
TUCP_000584	XLOC_020716	ENSRNOT00000015946	0.974090355	8.80E-08
TUCP_000584	XLOC_020716	ENSRNOT00000058763	0.955018541	1.34E-06
TUCP_000584	XLOC_020716	ENSRNOT00000023962	0.985237632	5.39E-09
TUCP_000584	XLOC_020716	ENSRNOT00000000529	0.986951246	2.91E-09
TUCP_000584	XLOC_020716	ENSRNOT00000074629	0.955203567	1.32E-06

TUCP_000584	XLOC_020716	ENSRNOT00000025819	0.96532403	3.73E-07
TUCP_000584	XLOC_020716	ENSRNOT00000015318	0.977446775	4.42E-08
TUCP_000584	XLOC_020716	ENSRNOT00000073388	0.973468984	9.90E-08
TUCP_000584	XLOC_020716	ENSRNOT00000028907	-0.954699335	1.39E-06
TUCP_000584	XLOC_020716	ENSRNOT00000014317	0.980797623	1.99E-08
TUCP_000584	XLOC_020716	ENSRNOT00000005486	0.954341262	1.45E-06
TUCP_000584	XLOC_020716	ENSRNOT00000045532	0.969777475	1.89E-07
TUCP_000584	XLOC_020716	ENSRNOT00000041151	0.950784613	2.09E-06
TUCP_000584	XLOC_020716	ENSRNOT00000025887	0.964046686	4.45E-07
TUCP_000584	XLOC_020716	ENSRNOT00000066211	0.96946689	1.99E-07
TUCP_000584	XLOC_020716	ENSRNOT00000005257	-0.959543261	7.97E-07
TUCP_000584	XLOC_020716	ENSRNOT00000039296	-0.95579403	1.23E-06
TUCP_000584	XLOC_020716	ENSRNOT00000039551	0.965952666	3.40E-07
TUCP_000584	XLOC_020716	ENSRNOT00000082998	-0.950153942	2.23E-06
TUCP_000584	XLOC_020716	ENSRNOT00000075656	0.954717638	1.39E-06
TUCP_000584	XLOC_020716	ENSRNOT00000007350	-0.95181882	1.89E-06
TUCP_000584	XLOC_020716	ENSRNOT00000083573	0.961966133	5.88E-07
TUCP_000584	XLOC_020716	ENSRNOT00000005138	0.95287086	1.69E-06
TUCP_000584	XLOC_020716	ENSRNOT00000083578	-0.958238041	9.33E-07
TUCP_000584	XLOC_020716	ENSRNOT00000045443	0.971419524	1.43E-07
TUCP_000584	XLOC_020716	ENSRNOT00000059524	0.959648576	7.87E-07
TUCP_000584	XLOC_020716	ENSRNOT00000017361	-0.952820603	1.70E-06
TUCP_000584	XLOC_020716	ENSRNOT00000017362	0.951840967	1.88E-06
TUCP_000584	XLOC_020716	ENSRNOT00000019465	0.984372439	7.15E-09
TUCP_000584	XLOC_020716	ENSRNOT00000018696	0.9835728	9.17E-09
TUCP_000584	XLOC_020716	ENSRNOT00000009073	0.967208964	2.83E-07
TUCP_000584	XLOC_020716	ENSRNOT00000001586	0.973303217	1.02E-07
TUCP_000584	XLOC_020716	ENSRNOT00000001468	0.959558252	7.96E-07
TUCP_000584	XLOC_020716	ENSRNOT00000013516	-0.954836923	1.37E-06
TUCP_000584	XLOC_020716	ENSRNOT00000013043	0.96573631	3.51E-07
TUCP_000584	XLOC_020716	ENSRNOT00000034603	0.950090765	2.24E-06
TUCP_000584	XLOC_020716	ENSRNOT00000016541	0.966266477	3.25E-07
TUCP_000584	XLOC_020716	ENSRNOT00000012060	-0.954475788	1.43E-06
TUCP_000584	XLOC_020716	ENSRNOT00000009000	0.9698747	1.86E-07
TUCP_000584	XLOC_020716	ENSRNOT00000034282	-0.964116182	4.41E-07
TUCP_000584	XLOC_020716	ENSRNOT00000039523	-0.955584258	1.26E-06
TUCP_000584	XLOC_020716	ENSRNOT00000011731	0.959619971	7.90E-07
TUCP_000584	XLOC_020716	ENSRNOT00000089545	0.966696572	3.05E-07
TUCP_000584	XLOC_020716	ENSRNOT00000037681	0.964904991	3.95E-07
TUCP_000584	XLOC_020716	ENSRNOT00000067009	0.95752309	1.01E-06
TUCP_000584	XLOC_020716	ENSRNOT00000000990	0.9620071	5.85E-07
TUCP_000584	XLOC_020716	ENSRNOT00000085151	0.956494285	1.14E-06
TUCP_000584	XLOC_020716	ENSRNOT00000016330	0.961433246	6.30E-07
TUCP_000584	XLOC_020716	ENSRNOT00000019121	0.989187293	1.14E-09
TUCP_000584	XLOC_020716	ENSRNOT00000086775	0.982512582	1.25E-08
TUCP_000584	XLOC_020716	ENSRNOT00000019820	-0.957877493	9.73E-07
TUCP_000584	XLOC_020716	ENSRNOT00000091864	0.955879964	1.22E-06
TUCP_000584	XLOC_020716	ENSRNOT00000083351	0.962672709	5.36E-07
TUCP_000584	XLOC_020716	ENSRNOT00000012963	0.972988546	1.08E-07
TUCP_000584	XLOC_020716	ENSRNOT00000011832	0.954835568	1.37E-06
TUCP_000584	XLOC_020716	ENSRNOT00000020446	-0.955637552	1.26E-06

TUCP_000584	XLOC_020716	ENSRNOT00000032301	0.977383118	4.49E-08
TUCP_000584	XLOC_020716	ENSRNOT00000024069	0.959055377	8.46E-07
TUCP_000584	XLOC_020716	ENSRNOT00000003940	0.950121676	2.24E-06
TUCP_000584	XLOC_020716	ENSRNOT00000014163	0.959115134	8.40E-07
TUCP_000584	XLOC_020716	ENSRNOT00000020932	0.954438445	1.43E-06
TUCP_000584	XLOC_020716	ENSRNOT00000068745	-0.951408894	1.97E-06
TUCP_000584	XLOC_020716	ENSRNOT00000030975	0.972937952	1.09E-07
TUCP_000584	XLOC_020716	ENSRNOT00000092068	-0.962494557	5.49E-07
TUCP_000584	XLOC_020716	ENSRNOT00000017949	0.960063245	7.48E-07
TUCP_000584	XLOC_020716	ENSRNOT00000012646	0.966562413	3.11E-07
TUCP_000584	XLOC_020716	ENSRNOT00000074212	-0.950966268	2.06E-06
TUCP_000584	XLOC_020716	ENSRNOT00000013864	0.977955795	3.95E-08
TUCP_000584	XLOC_020716	ENSRNOT00000027435	-0.961507592	6.24E-07
TUCP_000584	XLOC_020716	ENSRNOT00000003544	0.953390119	1.60E-06
TUCP_000584	XLOC_020716	ENSRNOT00000022194	0.979656278	2.65E-08
TUCP_000584	XLOC_020716	ENSRNOT00000019389	0.954512379	1.42E-06
TUCP_000584	XLOC_020716	ENSRNOT00000060098	0.959159866	8.35E-07
TUCP_000584	XLOC_020716	ENSRNOT00000067739	0.962551587	5.45E-07
TUCP_000584	XLOC_020716	ENSRNOT00000019386	0.978443505	3.54E-08
TUCP_000584	XLOC_020716	ENSRNOT00000076230	-0.968763966	2.22E-07
TUCP_000584	XLOC_020716	ENSRNOT00000014767	0.957009382	1.08E-06
TUCP_000584	XLOC_020716	ENSRNOT00000083110	0.966668386	3.06E-07
TUCP_000584	XLOC_020716	ENSRNOT00000036658	0.969605224	1.94E-07
TUCP_000584	XLOC_020716	ENSRNOT00000007738	0.966234872	3.27E-07
TUCP_000584	XLOC_020716	ENSRNOT00000022107	0.953284624	1.62E-06
TUCP_000584	XLOC_020716	ENSRNOT00000030803	0.979211189	2.95E-08
TUCP_000584	XLOC_020716	ENSRNOT00000084552	0.972732562	1.13E-07
TUCP_000584	XLOC_020716	ENSRNOT00000034883	0.952653777	1.73E-06
TUCP_000584	XLOC_020716	ENSRNOT00000020155	-0.963029674	5.11E-07
TUCP_000584	XLOC_020716	ENSRNOT00000015320	0.966953213	2.94E-07
TUCP_000584	XLOC_020716	ENSRNOT00000000707	0.978889178	3.19E-08
TUCP_000584	XLOC_020716	ENSRNOT00000071239	0.979282534	2.90E-08
TUCP_000584	XLOC_020716	ENSRNOT00000005910	0.957770562	9.85E-07
TUCP_000584	XLOC_020716	ENSRNOT00000075917	0.984040339	7.94E-09
TUCP_000584	XLOC_020716	ENSRNOT00000029701	-0.95290219	1.69E-06
TUCP_000584	XLOC_020716	ENSRNOT00000014585	0.97057436	1.65E-07
TUCP_000584	XLOC_020716	ENSRNOT00000074261	0.989970438	7.86E-10
TUCP_000584	XLOC_020716	ENSRNOT00000055062	0.966810734	3.00E-07
TUCP_000584	XLOC_020716	ENSRNOT00000016939	0.957569888	1.01E-06
TUCP_000584	XLOC_020716	ENSRNOT00000080591	-0.955274487	1.31E-06
TUCP_000584	XLOC_020716	ENSRNOT00000030329	0.960237416	7.32E-07
TUCP_000584	XLOC_020716	ENSRNOT00000017469	0.981074022	1.85E-08
TUCP_000584	XLOC_020716	ENSRNOT00000033450	0.976186151	5.80E-08
TUCP_000584	XLOC_020716	ENSRNOT00000045311	0.966127379	3.32E-07
TUCP_000584	XLOC_020716	ENSRNOT00000027121	-0.950893239	2.07E-06
TUCP_000584	XLOC_020716	ENSRNOT00000023078	-0.962468947	5.51E-07
TUCP_000584	XLOC_020716	ENSRNOT00000004999	-0.958785651	8.74E-07
TUCP_000584	XLOC_020716	ENSRNOT00000009122	-0.961605356	6.16E-07
TUCP_000584	XLOC_020716	ENSRNOT00000013463	-0.971402293	1.44E-07
TUCP_000584	XLOC_020716	ENSRNOT00000039335	0.973195714	1.04E-07
TUCP_000584	XLOC_020716	ENSRNOT00000007889	0.969057591	2.12E-07

TUCP_000584	XLOC_020716	ENSRNOT00000061191	0.959126297	8.39E-07
TUCP_000584	XLOC_020716	ENSRNOT00000029202	0.956377123	1.16E-06
TUCP_000584	XLOC_020716	ENSRNOT00000017907	0.977588159	4.29E-08
TUCP_000584	XLOC_020716	ENSRNOT00000028434	0.973604096	9.65E-08
TUCP_000584	XLOC_020716	ENSRNOT00000025794	0.978021635	3.89E-08
TUCP_000584	XLOC_020716	ENSRNOT00000058111	0.972303949	1.23E-07
TUCP_000584	XLOC_020716	ENSRNOT00000011869	0.980029004	2.42E-08
TUCP_000584	XLOC_020716	ENSRNOT00000001720	0.964231095	4.34E-07
TUCP_000584	XLOC_020716	ENSRNOT00000002443	-0.950273736	2.20E-06
TUCP_000584	XLOC_020716	ENSRNOT00000002114	0.962935715	5.18E-07
TUCP_000584	XLOC_020716	ENSRNOT00000009993	0.976842417	5.05E-08
TUCP_000584	XLOC_020716	ENSRNOT00000006376	0.950755838	2.10E-06
TUCP_000584	XLOC_020716	ENSRNOT00000027753	0.96846235	2.33E-07
TUCP_000584	XLOC_020716	ENSRNOT00000046215	0.984317049	7.28E-09
TUCP_000584	XLOC_020716	ENSRNOT00000000083	0.971627794	1.38E-07
TUCP_000584	XLOC_020716	ENSRNOT00000076467	0.973173628	1.05E-07
TUCP_000584	XLOC_020716	ENSRNOT00000000120	-0.956123049	1.19E-06
TUCP_000584	XLOC_020716	ENSRNOT00000013632	-0.952616545	1.74E-06
TUCP_000584	XLOC_020716	ENSRNOT00000028545	0.960689966	6.92E-07
TUCP_000584	XLOC_020716	ENSRNOT00000027575	0.967758986	2.60E-07
TUCP_000584	XLOC_020716	ENSRNOT00000028225	-0.96586785	3.45E-07
TUCP_000584	XLOC_020716	ENSRNOT00000019172	0.97657045	5.35E-08
TUCP_000584	XLOC_020716	ENSRNOT00000068267	0.957612519	1.00E-06
TUCP_000584	XLOC_020716	ENSRNOT00000020994	0.977317877	4.55E-08
TUCP_000584	XLOC_020716	ENSRNOT00000032248	-0.950116375	2.24E-06
TUCP_000584	XLOC_020716	ENSRNOT00000019911	0.950935946	2.06E-06
TUCP_000584	XLOC_020716	ENSRNOT00000032236	0.963106968	5.06E-07
TUCP_000584	XLOC_020716	ENSRNOT00000016372	0.976326992	5.63E-08
TUCP_000584	XLOC_020716	ENSRNOT00000036972	0.956290659	1.17E-06
TUCP_000584	XLOC_020716	ENSRNOT00000064700	-0.958402265	9.15E-07
TUCP_000584	XLOC_020716	ENSRNOT00000025252	0.963459794	4.82E-07
TUCP_000584	XLOC_020716	ENSRNOT00000036194	0.953376775	1.60E-06
TUCP_000584	XLOC_020716	ENSRNOT00000077186	0.967466151	2.72E-07
TUCP_000584	XLOC_020716	ENSRNOT00000081314	0.973593125	9.67E-08
TUCP_000584	XLOC_020716	ENSRNOT00000014780	0.97141238	1.43E-07
TUCP_000584	XLOC_020716	ENSRNOT00000006460	-0.974954754	7.44E-08
TUCP_000584	XLOC_020716	ENSRNOT00000026279	0.983817003	8.51E-09
TUCP_000584	XLOC_020716	ENSRNOT00000021506	0.950506938	2.15E-06
TUCP_000584	XLOC_020716	ENSRNOT00000004713	0.95107825	2.03E-06
TUCP_000584	XLOC_020716	ENSRNOT00000060607	0.959534928	7.98E-07
TUCP_000584	XLOC_020716	ENSRNOT00000021858	-0.958278232	9.28E-07
TUCP_000584	XLOC_020716	ENSRNOT00000065942	0.993354976	1.01E-10
TUCP_000584	XLOC_020716	ENSRNOT00000014309	0.962187393	5.71E-07
TUCP_000584	XLOC_020716	ENSRNOT00000074389	0.950205682	2.22E-06
TUCP_000584	XLOC_020716	ENSRNOT00000066204	0.975966165	6.07E-08
TUCP_000584	XLOC_020716	ENSRNOT00000039836	0.950527318	2.15E-06
TUCP_000584	XLOC_020716	ENSRNOT00000018101	0.990486345	6.04E-10
TUCP_000584	XLOC_020716	ENSRNOT00000003012	0.964287281	4.31E-07
TUCP_000584	XLOC_020716	ENSRNOT00000000052	0.958545723	8.99E-07
TUCP_000584	XLOC_020716	ENSRNOT00000073569	0.966609728	3.09E-07
TUCP_000584	XLOC_020716	ENSRNOT00000087604	-0.954962129	1.35E-06

TUCP_000584	XLOC_020716	ENSRNOT00000083568	-0.956759389	1.11E-06
TUCP_000584	XLOC_020716	ENSRNOT00000039177	0.952532544	1.75E-06
TUCP_000584	XLOC_020716	ENSRNOT00000005620	0.95730525	1.04E-06
TUCP_000584	XLOC_020716	ENSRNOT00000017375	0.975009584	7.36E-08
TUCP_000584	XLOC_020716	ENSRNOT00000068044	-0.95206546	1.84E-06
TUCP_000584	XLOC_020716	ENSRNOT00000012881	0.959752131	7.77E-07
TUCP_000584	XLOC_020716	ENSRNOT00000045855	0.952309325	1.79E-06
TUCP_000584	XLOC_020716	ENSRNOT00000028363	0.963450484	4.83E-07
TUCP_000584	XLOC_020716	ENSRNOT00000014533	0.962706836	5.34E-07
TUCP_000584	XLOC_020716	ENSRNOT00000088133	0.966149577	3.31E-07
TUCP_000584	XLOC_020716	ENSRNOT00000016629	-0.963814879	4.60E-07
TUCP_000584	XLOC_020716	ENSRNOT00000075209	0.973172094	1.05E-07
TUCP_000584	XLOC_020716	ENSRNOT00000001979	0.955339041	1.30E-06
TUCP_000584	XLOC_020716	ENSRNOT00000083968	0.96807887	2.47E-07
TUCP_000584	XLOC_020716	ENSRNOT00000083967	-0.968485205	2.32E-07
TUCP_000584	XLOC_020716	ENSRNOT00000083964	0.971928105	1.31E-07
TUCP_000584	XLOC_020716	ENSRNOT00000061304	0.978055539	3.86E-08
TUCP_000584	XLOC_020716	ENSRNOT00000079609	-0.966176685	3.29E-07
TUCP_000584	XLOC_020716	ENSRNOT00000016447	0.963530685	4.78E-07
TUCP_000584	XLOC_020716	ENSRNOT00000012314	0.955120457	1.33E-06
TUCP_000584	XLOC_020716	ENSRNOT00000066888	0.982273924	1.34E-08
TUCP_000584	XLOC_020716	ENSRNOT00000002151	0.965500334	3.63E-07
TUCP_000584	XLOC_020716	ENSRNOT00000041091	0.964076138	4.44E-07
TUCP_000584	XLOC_020716	ENSRNOT00000029845	0.95485957	1.37E-06
TUCP_000584	XLOC_020716	ENSRNOT00000086260	0.955735494	1.24E-06
TUCP_000584	XLOC_020716	ENSRNOT00000064667	0.952615988	1.74E-06
TUCP_000584	XLOC_020716	ENSRNOT00000000177	0.957884746	9.72E-07
TUCP_000584	XLOC_020716	ENSRNOT00000018328	0.993269979	1.08E-10
TUCP_000584	XLOC_020716	ENSRNOT00000038610	0.980472838	2.16E-08
TUCP_000584	XLOC_020716	ENSRNOT00000010683	0.959182457	8.33E-07
TUCP_000584	XLOC_020716	ENSRNOT00000026988	0.959986374	7.55E-07
TUCP_000584	XLOC_020716	ENSRNOT00000012657	0.958078942	9.50E-07
TUCP_000584	XLOC_020716	ENSRNOT00000050119	0.953684406	1.55E-06
TUCP_000584	XLOC_020716	ENSRNOT00000090853	0.971036491	1.53E-07
TUCP_000584	XLOC_020716	ENSRNOT00000089265	0.962461937	5.51E-07
TUCP_000584	XLOC_020716	ENSRNOT00000031261	-0.969852328	1.86E-07
TUCP_000584	XLOC_020716	ENSRNOT00000067836	0.977882739	4.02E-08
TUCP_000584	XLOC_020716	ENSRNOT00000019816	0.960508472	7.08E-07
TUCP_000584	XLOC_020716	ENSRNOT00000073847	0.97657812	5.34E-08
TUCP_000584	XLOC_020716	ENSRNOT00000078366	-0.951922517	1.87E-06
TUCP_000584	XLOC_020716	ENSRNOT00000025222	0.972454801	1.19E-07
TUCP_000584	XLOC_020716	ENSRNOT00000056015	0.965669526	3.55E-07
TUCP_000584	XLOC_020716	ENSRNOT00000068528	0.9945229	3.85E-11
TUCP_000584	XLOC_020716	ENSRNOT00000011675	-0.959757274	7.77E-07
TUCP_000584	XLOC_020716	ENSRNOT00000004290	-0.962897095	5.20E-07
TUCP_000584	XLOC_020716	ENSRNOT00000039000	0.985758487	4.51E-09
TUCP_000584	XLOC_020716	ENSRNOT00000030692	0.964721122	4.06E-07
TUCP_000584	XLOC_020716	ENSRNOT00000019713	0.958486127	9.06E-07
TUCP_000584	XLOC_020716	ENSRNOT00000020005	-0.953852428	1.53E-06
TUCP_000584	XLOC_020716	ENSRNOT00000085214	0.952733297	1.72E-06
TUCP_000584	XLOC_020716	ENSRNOT00000083632	0.967111574	2.87E-07

TUCP_000584	XLOC_020716	ENSRNOT00000093385	-0.956142156	1.19E-06
TUCP_000584	XLOC_020716	ENSRNOT00000017224	0.983633437	9.00E-09
TUCP_000584	XLOC_020716	ENSRNOT00000049725	0.960172191	7.38E-07
TUCP_000584	XLOC_020716	ENSRNOT00000026235	0.964694185	4.07E-07
TUCP_000584	XLOC_020716	ENSRNOT00000010753	0.957199448	1.05E-06
TUCP_000584	XLOC_020716	ENSRNOT00000087346	-0.953822227	1.53E-06
TUCP_000584	XLOC_020716	ENSRNOT00000026258	-0.953026811	1.66E-06
TUCP_000584	XLOC_020716	ENSRNOT00000009202	-0.952994764	1.67E-06
TUCP_000584	XLOC_020716	ENSRNOT00000088615	0.966196924	3.28E-07
TUCP_000584	XLOC_020716	ENSRNOT00000059181	-0.968220338	2.42E-07
TUCP_000584	XLOC_020716	ENSRNOT00000007077	0.959499865	8.02E-07
TUCP_000584	XLOC_020716	ENSRNOT00000003691	0.980189323	2.32E-08
TUCP_000584	XLOC_020716	ENSRNOT00000022389	0.984862321	6.10E-09
TUCP_000584	XLOC_020716	ENSRNOT00000014809	0.97364704	9.58E-08
TUCP_000584	XLOC_020716	ENSRNOT00000064849	0.987201278	2.65E-09
TUCP_000584	XLOC_020716	ENSRNOT00000045950	0.959125681	8.39E-07
TUCP_000584	XLOC_020716	ENSRNOT00000008901	0.952332272	1.79E-06
TUCP_000584	XLOC_020716	ENSRNOT00000016260	0.956035548	1.20E-06
TUCP_000584	XLOC_020716	ENSRNOT00000004770	0.99040018	6.32E-10
TUCP_000584	XLOC_020716	ENSRNOT00000023017	0.983207215	1.02E-08
TUCP_000584	XLOC_020716	ENSRNOT00000016265	0.984341754	7.22E-09
TUCP_000584	XLOC_020716	ENSRNOT00000039221	0.954906572	1.36E-06
TUCP_000584	XLOC_020716	ENSRNOT00000018634	0.95688472	1.09E-06
TUCP_000584	XLOC_020716	ENSRNOT00000017340	0.985270946	5.33E-09
TUCP_000584	XLOC_020716	ENSRNOT00000016740	-0.955481609	1.28E-06
TUCP_000584	XLOC_020716	ENSRNOT00000019768	-0.95337038	1.61E-06
TUCP_000584	XLOC_020716	ENSRNOT00000080677	0.98383298	8.47E-09
TUCP_000584	XLOC_020716	ENSRNOT00000025460	-0.957236504	1.05E-06
TUCP_000584	XLOC_020716	ENSRNOT00000025784	0.966976637	2.93E-07
TUCP_000584	XLOC_020716	ENSRNOT00000028130	0.954441109	1.43E-06
TUCP_000584	XLOC_020716	ENSRNOT00000063821	0.952222605	1.81E-06
TUCP_000584	XLOC_020716	ENSRNOT00000025913	0.971030789	1.53E-07
TUCP_000584	XLOC_020716	ENSRNOT00000027360	0.972024532	1.29E-07
TUCP_000584	XLOC_020716	ENSRNOT00000049285	0.960205759	7.35E-07
TUCP_000584	XLOC_020716	ENSRNOT00000044486	0.964524379	4.17E-07
TUCP_000584	XLOC_020716	ENSRNOT00000079414	0.980925303	1.93E-08
TUCP_000584	XLOC_020716	ENSRNOT00000024011	-0.975886785	6.17E-08
TUCP_000584	XLOC_020716	ENSRNOT00000000134	0.987397029	2.45E-09
TUCP_000584	XLOC_020716	ENSRNOT00000005885	0.964811167	4.01E-07
TUCP_000584	XLOC_020716	ENSRNOT00000011326	0.98313908	1.04E-08
TUCP_001117	XLOC_042333	ENSRNOT00000076927	0.95108622	2.03E-06
TUCP_001117	XLOC_042333	ENSRNOT00000076239	0.962161869	5.73E-07
TUCP_000586	XLOC_020757	ENSRNOT00000071273	0.959082438	8.43E-07
TUCP_001119	XLOC_042333	ENSRNOT00000088012	0.953571159	1.57E-06
TUCP_001119	XLOC_042333	ENSRNOT00000084066	0.967125558	2.86E-07
TUCP_000427	XLOC_014387	ENSRNOT00000074990	0.972162172	1.26E-07
TUCP_000427	XLOC_014387	ENSRNOT00000068444	0.972162172	1.26E-07
TUCP_000427	XLOC_014387	ENSRNOT00000081370	0.973348452	1.01E-07
TUCP_000427	XLOC_014387	ENSRNOT00000078645	0.967237538	2.81E-07
TUCP_000427	XLOC_014387	ENSRNOT00000075987	0.954219892	1.47E-06
TUCP_000427	XLOC_014387	ENSRNOT00000082043	0.969219674	2.07E-07

TUCP_000427	XLOC_014387	ENSRNOT00000087323	0.964197114	4.36E-07
TUCP_000427	XLOC_014387	ENSRNOT00000074930	0.972162172	1.26E-07
TUCP_000427	XLOC_014387	ENSRNOT00000080575	0.97253586	1.18E-07
TUCP_000427	XLOC_014387	ENSRNOT00000057761	0.951313006	1.98E-06
TUCP_000427	XLOC_014387	ENSRNOT00000078369	0.958973903	8.54E-07
TUCP_000427	XLOC_014387	ENSRNOT00000092982	0.972162172	1.26E-07
TUCP_000427	XLOC_014387	ENSRNOT00000088367	0.972162172	1.26E-07
TUCP_000427	XLOC_014387	ENSRNOT00000086397	0.959112682	8.40E-07
TUCP_000427	XLOC_014387	ENSRNOT00000061874	0.978572653	3.43E-08
TUCP_000427	XLOC_014387	ENSRNOT00000081996	0.972162172	1.26E-07
TUCP_000427	XLOC_014387	ENSRNOT00000082058	0.972162172	1.26E-07
TUCP_000427	XLOC_014387	ENSRNOT00000088905	0.972162172	1.26E-07
TUCP_000427	XLOC_014387	ENSRNOT00000086831	0.972162172	1.26E-07
TUCP_000427	XLOC_014387	ENSRNOT00000045099	0.972162172	1.26E-07
TUCP_000427	XLOC_014387	ENSRNOT00000085849	0.954412771	1.44E-06
TUCP_000427	XLOC_014387	ENSRNOT00000089257	0.964753457	4.04E-07
TUCP_000427	XLOC_014387	ENSRNOT00000077789	0.953974101	1.51E-06
TUCP_000427	XLOC_014387	ENSRNOT00000087025	0.972162172	1.26E-07
TUCP_000427	XLOC_014387	ENSRNOT00000019853	0.972162172	1.26E-07
TUCP_000427	XLOC_014387	ENSRNOT00000073853	0.958812171	8.71E-07
TUCP_000427	XLOC_014387	ENSRNOT00000065912	0.977769827	4.12E-08
TUCP_000427	XLOC_014387	ENSRNOT00000093083	0.973388041	1.01E-07
TUCP_000343	XLOC_010780	ENSRNOT00000084075	0.996098046	7.08E-12
TUCP_000343	XLOC_010780	ENSRNOT00000012880	0.968020893	2.50E-07
TUCP_000343	XLOC_010780	ENSRNOT00000074465	0.99999962	6.25E-32
TUCP_000343	XLOC_010780	ENSRNOT00000090898	0.96162989	6.14E-07
TUCP_000343	XLOC_010780	ENSRNOT00000079805	0.998023515	2.37E-13
TUCP_000343	XLOC_010780	ENSRNOT00000040912	0.99999962	6.25E-32
TUCP_000343	XLOC_010780	ENSRNOT00000072648	0.99999962	6.25E-32
TUCP_000343	XLOC_010780	ENSRNOT00000007398	0.989039871	1.22E-09
TUCP_000343	XLOC_010780	ENSRNOT00000070803	0.99999962	6.25E-32
TUCP_000343	XLOC_010780	ENSRNOT00000012196	0.99999962	6.25E-32
TUCP_000343	XLOC_010780	ENSRNOT00000085772	0.99999962	6.25E-32
TUCP_000343	XLOC_010780	ENSRNOT00000093284	0.999974869	7.89E-23
TUCP_000343	XLOC_010780	ENSRNOT00000086374	0.993570148	8.56E-11
TUCP_000343	XLOC_010780	ENSRNOT00000015270	0.99999962	6.25E-32
TUCP_000343	XLOC_010780	ENSRNOT00000046192	0.99999962	6.25E-32
TUCP_000343	XLOC_010780	ENSRNOT00000044226	0.99999962	6.25E-32
TUCP_000343	XLOC_010780	ENSRNOT00000081898	0.989886669	8.19E-10
TUCP_000343	XLOC_010780	ENSRNOT00000081954	0.963971091	4.50E-07
TUCP_000343	XLOC_010780	ENSRNOT00000080291	0.998427594	7.55E-14
TUCP_000343	XLOC_010780	ENSRNOT00000004293	0.984278384	7.37E-09
TUCP_000343	XLOC_010780	ENSRNOT00000078231	0.960566679	7.03E-07
TUCP_000343	XLOC_010780	ENSRNOT00000002834	0.99999962	6.25E-32
TUCP_000343	XLOC_010780	ENSRNOT00000001464	0.99999962	6.25E-32
TUCP_000343	XLOC_010780	ENSRNOT00000090716	0.994762425	3.08E-11
TUCP_000343	XLOC_010780	ENSRNOT00000083542	0.972546557	1.17E-07
TUCP_000343	XLOC_010780	ENSRNOT00000076044	0.99999962	6.25E-32
TUCP_000343	XLOC_010780	ENSRNOT00000074353	0.99999962	6.25E-32
TUCP_000343	XLOC_010780	ENSRNOT00000043510	0.958107772	9.47E-07
TUCP_000343	XLOC_010780	ENSRNOT00000087122	0.980102325	2.38E-08

TUCP_000343	XLOC_010780	ENSRNOT00000074295	0.987718821	2.16E-09
TUCP_000343	XLOC_010780	ENSRNOT00000057873	0.99999962	6.25E-32
TUCP_000343	XLOC_010780	ENSRNOT00000083002	0.988851133	1.33E-09
TUCP_000343	XLOC_010780	ENSRNOT00000088399	0.999999378	7.36E-31
TUCP_000343	XLOC_010780	ENSRNOT00000086248	0.99999962	6.25E-32
TUCP_000343	XLOC_010780	ENSRNOT00000089750	0.999992276	2.16E-25
TUCP_000343	XLOC_010780	ENSRNOT00000030517	0.971213152	1.48E-07
TUCP_000343	XLOC_010780	ENSRNOT00000034772	0.994238179	4.95E-11
TUCP_000343	XLOC_010780	ENSRNOT00000089305	0.969580457	1.95E-07
TUCP_000343	XLOC_010780	ENSRNOT00000024580	0.954212955	1.47E-06
TUCP_000343	XLOC_010780	ENSRNOT00000064601	0.966570601	3.11E-07
TUCP_000343	XLOC_010780	ENSRNOT00000015870	0.965858602	3.45E-07
TUCP_000343	XLOC_010780	ENSRNOT00000080812	0.992959759	1.35E-10
TUCP_000343	XLOC_010780	ENSRNOT00000092755	0.99999962	6.25E-32
TUCP_000343	XLOC_010780	ENSRNOT00000031683	0.99999962	6.25E-32
TUCP_000343	XLOC_010780	ENSRNOT00000087764	0.98816327	1.79E-09
TUCP_003175	XLOC_136347	ENSRNOT00000077435	0.958007045	9.58E-07
TUCP_004046	XLOC_177930	ENSRNOT00000077458	0.960069756	7.48E-07
TUCP_004046	XLOC_177930	ENSRNOT00000085479	0.953039508	1.66E-06
TUCP_004047	XLOC_177979	ENSRNOT00000000008	0.987543822	2.31E-09
TUCP_004047	XLOC_177979	ENSRNOT00000060048	0.990178846	7.08E-10
TUCP_004047	XLOC_177979	ENSRNOT00000082304	0.991136053	4.25E-10
TUCP_004047	XLOC_177979	ENSRNOT00000006219	0.987543822	2.31E-09
TUCP_004047	XLOC_177979	ENSRNOT00000065376	0.987543822	2.31E-09
TUCP_004047	XLOC_177979	ENSRNOT00000081433	0.987048406	2.81E-09
TUCP_004047	XLOC_177979	ENSRNOT00000083575	0.987543822	2.31E-09
TUCP_004047	XLOC_177979	ENSRNOT00000087739	0.987543822	2.31E-09
TUCP_004047	XLOC_177979	ENSRNOT00000079826	0.987543822	2.31E-09
TUCP_004047	XLOC_177979	ENSRNOT00000025770	0.977468328	4.40E-08
TUCP_004047	XLOC_177979	ENSRNOT00000088244	0.967604106	2.66E-07
TUCP_004047	XLOC_177979	ENSRNOT00000048523	0.987543822	2.31E-09
TUCP_004047	XLOC_177979	ENSRNOT00000020229	0.976499195	5.43E-08
TUCP_004047	XLOC_177979	ENSRNOT00000026428	0.98128615	1.75E-08
TUCP_004047	XLOC_177979	ENSRNOT00000085673	0.987543822	2.31E-09
TUCP_004047	XLOC_177979	ENSRNOT00000072972	0.967326309	2.78E-07
TUCP_004047	XLOC_177979	ENSRNOT00000050573	0.987543822	2.31E-09
TUCP_004047	XLOC_177979	ENSRNOT00000074378	0.984301722	7.31E-09
TUCP_004047	XLOC_177979	ENSRNOT00000090581	0.987543822	2.31E-09
TUCP_004047	XLOC_177979	ENSRNOT00000076239	0.974498187	8.14E-08
TUCP_004047	XLOC_177979	ENSRNOT00000084374	0.966988818	2.92E-07
TUCP_004047	XLOC_177979	ENSRNOT00000065485	0.987543822	2.31E-09
TUCP_004047	XLOC_177979	ENSRNOT00000006356	0.987113724	2.74E-09
TUCP_004047	XLOC_177979	ENSRNOT00000087199	0.990056527	7.53E-10
TUCP_004047	XLOC_177979	ENSRNOT00000022091	0.987543822	2.31E-09
TUCP_004047	XLOC_177979	ENSRNOT00000064764	0.977159466	4.71E-08
TUCP_004047	XLOC_177979	ENSRNOT00000093571	0.987543822	2.31E-09
TUCP_004047	XLOC_177979	ENSRNOT00000092395	0.986358552	3.64E-09
TUCP_004769	XLOC_210605	ENSRNOT00000015944	0.953985962	1.50E-06
TUCP_004769	XLOC_210605	ENSRNOT00000030327	0.997187569	1.38E-12
TUCP_004769	XLOC_210605	ENSRNOT00000024455	0.959374561	8.14E-07
TUCP_004769	XLOC_210605	ENSRNOT00000013296	0.992972832	1.33E-10

TUCP_004769	XLOC_210605	ENSRNOT00000085380	0.998512644	5.72E-14
TUCP_000346	XLOC_010781	ENSRNOT00000010035	0.963299577	4.93E-07
TUCP_000346	XLOC_010781	ENSRNOT00000071697	0.951520161	1.94E-06
TUCP_000346	XLOC_010781	ENSRNOT00000024517	0.956596592	1.13E-06
TUCP_004042	XLOC_177832	ENSRNOT00000092026	0.96620614	3.28E-07
TUCP_004042	XLOC_177832	ENSRNOT00000093075	1	0
TUCP_004042	XLOC_177832	ENSRNOT00000058260	1	0
TUCP_004042	XLOC_177832	ENSRNOT00000088887	1	4.25E-78
TUCP_004042	XLOC_177832	ENSRNOT00000084986	1	0
TUCP_004042	XLOC_177832	ENSRNOT00000074346	0.9702256	1.75E-07
TUCP_004042	XLOC_177832	ENSRNOT00000073209	0.983711602	8.79E-09
TUCP_004042	XLOC_177832	ENSRNOT00000080155	0.973069673	1.07E-07
TUCP_004042	XLOC_177832	ENSRNOT00000072573	0.996213272	6.09E-12
TUCP_004042	XLOC_177832	ENSRNOT00000017828	0.996001583	7.99E-12
TUCP_004042	XLOC_177832	ENSRNOT00000080444	0.969625907	1.93E-07
TUCP_004042	XLOC_177832	ENSRNOT00000071312	1	0
TUCP_004042	XLOC_177832	ENSRNOT00000088245	1	0
TUCP_004042	XLOC_177832	ENSRNOT00000036565	0.969065335	2.12E-07
TUCP_004042	XLOC_177832	ENSRNOT00000058833	0.998673261	3.23E-14
TUCP_004042	XLOC_177832	ENSRNOT00000078052	1	0
TUCP_004042	XLOC_177832	ENSRNOT00000078494	0.999927093	1.62E-20
TUCP_004042	XLOC_177832	ENSRNOT00000085653	1	0
TUCP_004042	XLOC_177832	ENSRNOT00000057950	0.969329371	2.03E-07
TUCP_004042	XLOC_177832	ENSRNOT00000081992	0.999320526	1.14E-15
TUCP_004042	XLOC_177832	ENSRNOT00000038553	0.955765808	1.24E-06
TUCP_004042	XLOC_177832	ENSRNOT00000084120	0.958115447	9.46E-07
TUCP_004042	XLOC_177832	ENSRNOT00000055406	1	0
TUCP_004042	XLOC_177832	ENSRNOT00000070817	0.998968079	9.20E-15
TUCP_004042	XLOC_177832	ENSRNOT00000026753	1	4.25E-78
TUCP_004042	XLOC_177832	ENSRNOT00000080533	0.996682529	3.15E-12
TUCP_004042	XLOC_177832	ENSRNOT00000072774	0.979665261	2.65E-08
TUCP_004042	XLOC_177832	ENSRNOT00000086031	1	0
TUCP_004042	XLOC_177832	ENSRNOT00000014312	1	0
TUCP_004042	XLOC_177832	ENSRNOT00000042882	0.963218427	4.98E-07
TUCP_004042	XLOC_177832	ENSRNOT00000088012	0.956765806	1.11E-06
TUCP_004042	XLOC_177832	ENSRNOT00000051231	0.999242378	1.96E-15
TUCP_004042	XLOC_177832	ENSRNOT00000089407	0.992986194	1.32E-10
TUCP_004042	XLOC_177832	ENSRNOT00000055034	0.998331441	1.02E-13
TUCP_004042	XLOC_177832	ENSRNOT00000046837	0.97252559	1.18E-07
TUCP_000344	XLOC_010780	ENSRNOT00000012602	0.957594878	1.01E-06
TUCP_004764	XLOC_209810	ENSRNOT00000081570	0.997186921	1.38E-12
TUCP_004764	XLOC_209810	ENSRNOT00000091652	0.96069526	6.92E-07
TUCP_004764	XLOC_209810	ENSRNOT00000080865	0.965657584	3.55E-07
TUCP_004764	XLOC_209810	ENSRNOT00000073534	0.961719611	6.07E-07
TUCP_004764	XLOC_209810	ENSRNOT00000093019	0.965657584	3.55E-07
TUCP_004764	XLOC_209810	ENSRNOT00000085908	0.956762543	1.11E-06
TUCP_004764	XLOC_209810	ENSRNOT00000046744	0.965657584	3.55E-07
TUCP_004764	XLOC_209810	ENSRNOT00000076429	0.965657584	3.55E-07
TUCP_004764	XLOC_209810	ENSRNOT00000084667	0.964027778	4.47E-07
TUCP_004764	XLOC_209810	ENSRNOT00000055412	0.965657584	3.55E-07
TUCP_004764	XLOC_209810	ENSRNOT00000093201	0.967072897	2.88E-07

TUCP_004764	XLOC_209810	ENSRNOT00000072724	0.965657584	3.55E-07
TUCP_004764	XLOC_209810	ENSRNOT00000082601	0.951343858	1.98E-06
TUCP_004764	XLOC_209810	ENSRNOT00000084375	0.965657584	3.55E-07
TUCP_004764	XLOC_209810	ENSRNOT00000012161	0.952295968	1.80E-06
TUCP_004764	XLOC_209810	ENSRNOT00000042369	0.95700847	1.08E-06
TUCP_004764	XLOC_209810	ENSRNOT00000086554	0.965657584	3.55E-07
TUCP_004764	XLOC_209810	ENSRNOT00000047921	0.965657584	3.55E-07
TUCP_004764	XLOC_209810	ENSRNOT00000064261	0.965282072	3.75E-07
TUCP_004764	XLOC_209810	ENSRNOT00000086660	0.971581489	1.39E-07
TUCP_004764	XLOC_209810	ENSRNOT00000083977	0.965657584	3.55E-07
TUCP_004764	XLOC_209810	ENSRNOT00000092933	0.965657584	3.55E-07
TUCP_004764	XLOC_209810	ENSRNOT00000085447	0.973420545	9.99E-08
TUCP_001916	XLOC_078879	ENSRNOT00000037509	0.959692644	7.83E-07
TUCP_004048	XLOC_177979	ENSRNOT00000071603	0.95004426	2.25E-06
TUCP_004048	XLOC_177979	ENSRNOT00000090211	0.952874525	1.69E-06
TUCP_004048	XLOC_177979	ENSRNOT00000007604	0.956146903	1.19E-06
TUCP_004048	XLOC_177979	ENSRNOT00000047870	0.981081943	1.85E-08
TUCP_004048	XLOC_177979	ENSRNOT00000088376	0.957107345	1.06E-06
TUCP_004048	XLOC_177979	ENSRNOT00000044232	0.963089565	5.07E-07
TUCP_004048	XLOC_177979	ENSRNOT00000007683	0.950712059	2.11E-06
TUCP_004048	XLOC_177979	ENSRNOT00000003308	0.960423772	7.15E-07
TUCP_004048	XLOC_177979	ENSRNOT00000039313	0.970347231	1.72E-07
TUCP_004048	XLOC_177979	ENSRNOT00000084109	0.950287843	2.20E-06
TUCP_004048	XLOC_177979	ENSRNOT00000015316	0.958761334	8.76E-07
TUCP_004048	XLOC_177979	ENSRNOT00000068197	0.975100846	7.23E-08
TUCP_004048	XLOC_177979	ENSRNOT00000039652	0.950753866	2.10E-06
TUCP_004048	XLOC_177979	ENSRNOT00000023287	0.965216849	3.78E-07
TUCP_004048	XLOC_177979	ENSRNOT00000082086	0.963756656	4.63E-07
TUCP_004048	XLOC_177979	ENSRNOT00000018860	0.962726732	5.32E-07
TUCP_004048	XLOC_177979	ENSRNOT00000056364	0.952514312	1.76E-06
TUCP_004048	XLOC_177979	ENSRNOT00000071714	0.963084127	5.07E-07
TUCP_004048	XLOC_177979	ENSRNOT00000018890	0.963302188	4.93E-07
TUCP_004048	XLOC_177979	ENSRNOT00000009295	0.960591905	7.01E-07
TUCP_004763	XLOC_209802	ENSRNOT00000072402	0.984701314	6.43E-09
TUCP_004762	XLOC_209653	ENSRNOT00000003556	0.955271597	1.31E-06
TUCP_000569	XLOC_020107	ENSRNOT00000044232	0.95165355	1.92E-06
TUCP_000569	XLOC_020107	ENSRNOT00000092186	0.963557092	4.76E-07
TUCP_000569	XLOC_020107	ENSRNOT00000023584	0.961138078	6.54E-07
TUCP_000601	XLOC_021899	ENSRNOT00000082043	0.998965531	9.31E-15
TUCP_000601	XLOC_021899	ENSRNOT00000090080	0.970514074	1.67E-07
TUCP_000601	XLOC_021899	ENSRNOT00000068444	1	0
TUCP_000601	XLOC_021899	ENSRNOT00000081370	0.999740797	9.21E-18
TUCP_000601	XLOC_021899	ENSRNOT00000078645	0.989717078	8.90E-10
TUCP_000601	XLOC_021899	ENSRNOT00000075987	0.987945283	1.96E-09
TUCP_000601	XLOC_021899	ENSRNOT00000074990	1	0
TUCP_000601	XLOC_021899	ENSRNOT00000087323	0.995029331	2.37E-11
TUCP_000601	XLOC_021899	ENSRNOT00000074930	1	0
TUCP_000601	XLOC_021899	ENSRNOT00000080575	0.999929267	1.39E-20
TUCP_000601	XLOC_021899	ENSRNOT00000057761	0.982981445	1.09E-08
TUCP_000601	XLOC_021899	ENSRNOT00000078369	0.993028032	1.28E-10
TUCP_000601	XLOC_021899	ENSRNOT00000092982	1	0

TUCP_000601	XLOC_021899	ENSRNOT00000088367	1	0
TUCP_000601	XLOC_021899	ENSRNOT00000061874	0.968405099	2.35E-07
TUCP_000601	XLOC_021899	ENSRNOT00000077789	0.99346224	9.30E-11
TUCP_000601	XLOC_021899	ENSRNOT00000081996	1	0
TUCP_000601	XLOC_021899	ENSRNOT00000022280	0.982176915	1.37E-08
TUCP_000601	XLOC_021899	ENSRNOT00000082058	1	0
TUCP_000601	XLOC_021899	ENSRNOT00000088905	1	0
TUCP_000601	XLOC_021899	ENSRNOT00000086831	1	0
TUCP_000601	XLOC_021899	ENSRNOT00000045099	1	1.33E-79
TUCP_000601	XLOC_021899	ENSRNOT00000086745	0.978585655	3.42E-08
TUCP_000601	XLOC_021899	ENSRNOT00000085849	0.988270765	1.71E-09
TUCP_000601	XLOC_021899	ENSRNOT00000086397	0.957188178	1.05E-06
TUCP_000601	XLOC_021899	ENSRNOT00000079606	0.960472013	7.11E-07
TUCP_000601	XLOC_021899	ENSRNOT00000090207	0.953487741	1.59E-06
TUCP_000601	XLOC_021899	ENSRNOT00000087025	1	0
TUCP_000601	XLOC_021899	ENSRNOT00000019853	1	0
TUCP_000601	XLOC_021899	ENSRNOT00000073853	0.989715487	8.91E-10
TUCP_000601	XLOC_021899	ENSRNOT00000065912	0.974234118	8.57E-08
TUCP_000601	XLOC_021899	ENSRNOT00000073828	0.971647353	1.38E-07
TUCP_000601	XLOC_021899	ENSRNOT00000078683	0.966682958	3.06E-07
TUCP_000601	XLOC_021899	ENSRNOT00000093083	0.999140292	3.69E-15
TUCP_004774	XLOC_210931	ENSRNOT00000014699	-0.958678398	8.85E-07
TUCP_000604	XLOC_021901	ENSRNOT00000072367	-0.953211028	1.63E-06
TUCP_000562	XLOC_020072	ENSRNOT00000083351	-0.958948069	8.57E-07
TUCP_000562	XLOC_020072	ENSRNOT00000032802	-0.955095977	1.33E-06
TUCP_000562	XLOC_020072	ENSRNOT00000075656	-0.958568921	8.97E-07
TUCP_000562	XLOC_020072	ENSRNOT00000014924	-0.955452772	1.28E-06
TUCP_000562	XLOC_020072	ENSRNOT00000024030	0.955278665	1.31E-06
TUCP_000562	XLOC_020072	ENSRNOT00000030692	-0.961890411	5.94E-07
TUCP_000562	XLOC_020072	ENSRNOT00000057537	0.960770208	6.85E-07
TUCP_000562	XLOC_020072	ENSRNOT00000002445	-0.952354207	1.78E-06
TUCP_000562	XLOC_020072	ENSRNOT00000050609	-0.958494632	9.05E-07
TUCP_000562	XLOC_020072	ENSRNOT00000073667	-0.953104591	1.65E-06
TUCP_000562	XLOC_020072	ENSRNOT00000003940	-0.953261263	1.62E-06
TUCP_000562	XLOC_020072	ENSRNOT00000011930	0.952026258	1.85E-06
TUCP_000562	XLOC_020072	ENSRNOT00000045233	-0.952308198	1.79E-06
TUCP_000562	XLOC_020072	ENSRNOT00000067135	-0.953700398	1.55E-06
TUCP_000562	XLOC_020072	ENSRNOT00000017949	-0.964431764	4.22E-07
TUCP_000562	XLOC_020072	ENSRNOT00000039730	-0.959655642	7.87E-07
TUCP_000562	XLOC_020072	ENSRNOT00000083632	-0.953800002	1.53E-06
TUCP_000562	XLOC_020072	ENSRNOT00000083891	-0.975855389	6.21E-08
TUCP_000562	XLOC_020072	ENSRNOT00000063808	-0.964594606	4.13E-07
TUCP_000562	XLOC_020072	ENSRNOT00000039232	0.951271512	1.99E-06
TUCP_000562	XLOC_020072	ENSRNOT00000032236	-0.954003485	1.50E-06
TUCP_000562	XLOC_020072	ENSRNOT00000011794	-0.952781529	1.71E-06
TUCP_000562	XLOC_020072	ENSRNOT00000022000	-0.951940136	1.86E-06
TUCP_000562	XLOC_020072	ENSRNOT00000086244	0.952237168	1.81E-06
TUCP_000562	XLOC_020072	ENSRNOT00000089673	-0.953571797	1.57E-06
TUCP_000562	XLOC_020072	ENSRNOT00000079180	0.959666817	7.85E-07
TUCP_000562	XLOC_020072	ENSRNOT00000078128	-0.964835346	3.99E-07
TUCP_000561	XLOC_020072	ENSRNOT00000088876	0.99706668	1.70E-12

TUCP_002377	XLOC_100543	ENSRNOT00000003765	0.955062992	1.34E-06
TUCP_002377	XLOC_100543	ENSRNOT00000032324	0.952628927	1.73E-06
TUCP_002377	XLOC_100543	ENSRNOT00000080215	0.960898506	6.74E-07
TUCP_002377	XLOC_100543	ENSRNOT00000076946	0.950507505	2.15E-06
TUCP_000567	XLOC_020107	ENSRNOT00000089765	0.981835539	1.51E-08
TUCP_000567	XLOC_020107	ENSRNOT00000075752	0.972935214	1.09E-07
TUCP_000567	XLOC_020107	ENSRNOT00000084784	0.963190592	5.00E-07
TUCP_000567	XLOC_020107	ENSRNOT00000079573	0.972640484	1.15E-07
TUCP_000567	XLOC_020107	ENSRNOT00000086962	0.950674135	2.12E-06
TUCP_000567	XLOC_020107	ENSRNOT00000014073	0.96053525	7.06E-07
TUCP_000567	XLOC_020107	ENSRNOT00000002010	0.954918788	1.36E-06
TUCP_000567	XLOC_020107	ENSRNOT00000030489	0.981698237	1.57E-08
TUCP_002371	XLOC_100166	ENSRNOT00000036781	0.96045382	7.13E-07
TUCP_000565	XLOC_020107	ENSRNOT00000078806	0.95808828	9.49E-07
TUCP_000565	XLOC_020107	ENSRNOT00000080900	0.95289641	1.69E-06
TUCP_000565	XLOC_020107	ENSRNOT00000019109	0.95122247	2.00E-06
TUCP_000564	XLOC_020107	ENSRNOT00000073555	0.953381406	1.60E-06
TUCP_000564	XLOC_020107	ENSRNOT00000016039	0.96347712	4.81E-07
TUCP_000564	XLOC_020107	ENSRNOT00000091757	0.966398861	3.19E-07
TUCP_000564	XLOC_020107	ENSRNOT00000089218	0.956626818	1.12E-06
TUCP_000564	XLOC_020107	ENSRNOT00000021612	0.95140696	1.97E-06
TUCP_000564	XLOC_020107	ENSRNOT00000025638	0.977608798	4.27E-08
TUCP_002274	XLOC_094511	ENSRNOT00000000008	1	0
TUCP_002274	XLOC_094511	ENSRNOT00000060048	0.998711457	2.79E-14
TUCP_002274	XLOC_094511	ENSRNOT00000082304	0.996875989	2.33E-12
TUCP_002274	XLOC_094511	ENSRNOT00000006219	1	0
TUCP_002274	XLOC_094511	ENSRNOT00000065376	1	4.25E-78
TUCP_002274	XLOC_094511	ENSRNOT00000081433	0.984469223	6.93E-09
TUCP_002274	XLOC_094511	ENSRNOT00000083575	1	1.33E-79
TUCP_002274	XLOC_094511	ENSRNOT00000087739	1	1.33E-79
TUCP_002274	XLOC_094511	ENSRNOT00000079826	1	0
TUCP_002274	XLOC_094511	ENSRNOT00000025770	0.993828251	6.98E-11
TUCP_002274	XLOC_094511	ENSRNOT00000088244	0.973479481	9.88E-08
TUCP_002274	XLOC_094511	ENSRNOT00000048523	1	0
TUCP_002274	XLOC_094511	ENSRNOT00000079365	0.958936699	8.58E-07
TUCP_002274	XLOC_094511	ENSRNOT00000020229	0.960002785	7.54E-07
TUCP_002274	XLOC_094511	ENSRNOT00000026428	0.996986683	1.95E-12
TUCP_002274	XLOC_094511	ENSRNOT00000085673	1	0
TUCP_002274	XLOC_094511	ENSRNOT00000072972	0.981476166	1.67E-08
TUCP_002274	XLOC_094511	ENSRNOT00000050573	1	0
TUCP_002274	XLOC_094511	ENSRNOT00000089876	-0.953630353	1.56E-06
TUCP_002274	XLOC_094511	ENSRNOT00000074378	0.998901807	1.26E-14
TUCP_002274	XLOC_094511	ENSRNOT00000093571	1	0
TUCP_002274	XLOC_094511	ENSRNOT00000090581	1	4.25E-78
TUCP_002274	XLOC_094511	ENSRNOT00000076239	0.98020752	2.31E-08
TUCP_002274	XLOC_094511	ENSRNOT00000065485	1	1.33E-79
TUCP_002274	XLOC_094511	ENSRNOT00000006356	0.999544279	1.55E-16
TUCP_002274	XLOC_094511	ENSRNOT00000087199	0.974421555	8.26E-08
TUCP_002274	XLOC_094511	ENSRNOT00000022091	1	0
TUCP_002274	XLOC_094511	ENSRNOT00000064764	0.98460888	6.63E-09
TUCP_002274	XLOC_094511	ENSRNOT00000076927	0.971163156	1.50E-07

TUCP_002274	XLOC_094511	ENSRNOT00000092395	0.999522978	1.94E-16
TUCP_002276	XLOC_094576	ENSRNOT00000090760	0.953298025	1.62E-06
TUCP_002276	XLOC_094576	ENSRNOT00000024362	0.957274063	1.04E-06
TUCP_001950	XLOC_078994	ENSRNOT00000027220	-0.950358772	2.18E-06
TUCP_001950	XLOC_078994	ENSRNOT00000073521	-0.958119456	9.46E-07
TUCP_001950	XLOC_078994	ENSRNOT00000061297	-0.954573593	1.41E-06
TUCP_001950	XLOC_078994	ENSRNOT00000000211	-0.95799141	9.60E-07
TUCP_004672	XLOC_205750	ENSRNOT00000009430	0.960592512	7.00E-07
TUCP_004672	XLOC_205750	ENSRNOT00000046690	-0.954442722	1.43E-06
TUCP_004672	XLOC_205750	ENSRNOT00000047388	0.950913602	2.07E-06
TUCP_003041	XLOC_129304	ENSRNOT00000089607	0.95054061	2.14E-06
TUCP_003041	XLOC_129304	ENSRNOT00000077972	0.954246635	1.46E-06
TUCP_003041	XLOC_129304	ENSRNOT00000076757	0.97254966	1.17E-07
TUCP_003041	XLOC_129304	ENSRNOT00000052130	0.977239847	4.63E-08
TUCP_003041	XLOC_129304	ENSRNOT00000085479	0.955660296	1.25E-06
TUCP_003041	XLOC_129304	ENSRNOT00000084722	0.954498532	1.42E-06
TUCP_003041	XLOC_129304	ENSRNOT00000031693	0.957342683	1.04E-06
TUCP_003041	XLOC_129304	ENSRNOT00000077458	0.969635887	1.93E-07
TUCP_003041	XLOC_129304	ENSRNOT00000083034	0.951730599	1.90E-06
TUCP_002279	XLOC_094576	ENSRNOT00000003369	0.960413354	7.16E-07
TUCP_002279	XLOC_094576	ENSRNOT00000052249	0.968439313	2.34E-07
TUCP_002279	XLOC_094576	ENSRNOT00000011659	0.950158898	2.23E-06
TUCP_002279	XLOC_094576	ENSRNOT00000024110	0.954367142	1.44E-06
TUCP_002279	XLOC_094576	ENSRNOT00000009718	0.953274594	1.62E-06
TUCP_002279	XLOC_094576	ENSRNOT00000044087	0.952834064	1.70E-06
TUCP_004778	XLOC_211058	ENSRNOT00000009132	0.953698981	1.55E-06
TUCP_004778	XLOC_211058	ENSRNOT00000003511	0.958037058	9.55E-07
TUCP_004778	XLOC_211058	ENSRNOT00000090914	0.965341219	3.72E-07
TUCP_004778	XLOC_211058	ENSRNOT00000022559	0.963700529	4.67E-07
TUCP_004778	XLOC_211058	ENSRNOT00000044419	0.966326503	3.22E-07
TUCP_003802	XLOC_166074	ENSRNOT00000090923	0.961372822	6.35E-07
TUCP_003802	XLOC_166074	ENSRNOT00000076508	-0.959433751	8.08E-07
TUCP_003802	XLOC_166074	ENSRNOT00000016967	0.95618569	1.18E-06
TUCP_003802	XLOC_166074	ENSRNOT00000064331	0.952289323	1.80E-06
TUCP_001748	XLOC_072267	ENSRNOT00000015918	0.953782218	1.54E-06
TUCP_001748	XLOC_072267	ENSRNOT00000010763	0.954265359	1.46E-06
TUCP_001748	XLOC_072267	ENSRNOT00000080675	0.956319437	1.16E-06
TUCP_001748	XLOC_072267	ENSRNOT00000021403	0.974460446	8.20E-08
TUCP_001748	XLOC_072267	ENSRNOT00000036770	0.969057336	2.12E-07
TUCP_001748	XLOC_072267	ENSRNOT00000010591	0.951160406	2.02E-06
TUCP_001748	XLOC_072267	ENSRNOT00000007577	0.95998984	7.55E-07
TUCP_001748	XLOC_072267	ENSRNOT00000066545	0.954696619	1.39E-06
TUCP_001748	XLOC_072267	ENSRNOT00000001017	0.965349948	3.71E-07
TUCP_001748	XLOC_072267	ENSRNOT00000055176	0.960347926	7.22E-07
TUCP_000874	XLOC_032616	ENSRNOT00000018843	0.965421358	3.67E-07
TUCP_004677	XLOC_205892	ENSRNOT00000089931	0.954450917	1.43E-06
TUCP_004677	XLOC_205892	ENSRNOT00000040102	0.991302824	3.86E-10
TUCP_001746	XLOC_072267	ENSRNOT00000079021	0.954351968	1.45E-06
TUCP_003042	XLOC_129530	ENSRNOT00000025981	0.952776998	1.71E-06
TUCP_003042	XLOC_129530	ENSRNOT00000012671	0.954295304	1.45E-06
TUCP_003042	XLOC_129530	ENSRNOT00000023144	0.977080644	4.79E-08

TUCP_003042	XLOC_129530	ENSRNOT00000009859	0.950807254	2.09E-06
TUCP_003042	XLOC_129530	ENSRNOT00000001559	0.954331828	1.45E-06
TUCP_003042	XLOC_129530	ENSRNOT00000038647	0.95826322	9.30E-07
TUCP_003042	XLOC_129530	ENSRNOT00000039809	0.950054401	2.25E-06
TUCP_003042	XLOC_129530	ENSRNOT00000006727	0.951647529	1.92E-06
TUCP_003042	XLOC_129530	ENSRNOT00000066024	0.968424396	2.34E-07
TUCP_003042	XLOC_129530	ENSRNOT00000038807	0.953285967	1.62E-06
TUCP_003042	XLOC_129530	ENSRNOT00000080024	0.954204504	1.47E-06
TUCP_003042	XLOC_129530	ENSRNOT00000081512	0.976328548	5.63E-08
TUCP_003042	XLOC_129530	ENSRNOT00000021348	0.955484208	1.28E-06
TUCP_003042	XLOC_129530	ENSRNOT00000057160	0.981680997	1.58E-08
TUCP_003042	XLOC_129530	ENSRNOT00000088279	0.957517247	1.01E-06
TUCP_003042	XLOC_129530	ENSRNOT00000025808	0.968229537	2.42E-07
TUCP_003042	XLOC_129530	ENSRNOT00000031904	0.97188537	1.32E-07
TUCP_003042	XLOC_129530	ENSRNOT00000012736	0.954870373	1.37E-06
TUCP_003042	XLOC_129530	ENSRNOT00000005664	0.958772374	8.75E-07
TUCP_003042	XLOC_129530	ENSRNOT00000025092	0.969124389	2.10E-07
TUCP_003042	XLOC_129530	ENSRNOT00000040513	0.962120419	5.76E-07
TUCP_003042	XLOC_129530	ENSRNOT00000006175	0.963131023	5.04E-07
TUCP_003042	XLOC_129530	ENSRNOT00000083127	0.954035646	1.50E-06
TUCP_003042	XLOC_129530	ENSRNOT00000036072	0.958346116	9.21E-07
TUCP_003042	XLOC_129530	ENSRNOT00000080695	0.974157098	8.69E-08
TUCP_003042	XLOC_129530	ENSRNOT00000041044	0.97087434	1.57E-07
TUCP_003042	XLOC_129530	ENSRNOT00000000302	0.959279372	8.23E-07
TUCP_003042	XLOC_129530	ENSRNOT00000026941	0.956607387	1.13E-06
TUCP_003042	XLOC_129530	ENSRNOT00000076729	0.95432052	1.45E-06
TUCP_003042	XLOC_129530	ENSRNOT00000090665	0.975114932	7.21E-08
TUCP_003042	XLOC_129530	ENSRNOT00000088628	0.951798774	1.89E-06
TUCP_003042	XLOC_129530	ENSRNOT00000076763	0.962334402	5.60E-07
TUCP_003042	XLOC_129530	ENSRNOT00000045223	0.955936831	1.21E-06
TUCP_003042	XLOC_129530	ENSRNOT00000027411	0.95554699	1.27E-06
TUCP_003042	XLOC_129530	ENSRNOT00000010992	0.965847175	3.46E-07
TUCP_003042	XLOC_129530	ENSRNOT00000033017	0.952771985	1.71E-06
TUCP_003042	XLOC_129530	ENSRNOT00000060696	0.954746151	1.39E-06
TUCP_003042	XLOC_129530	ENSRNOT00000016191	0.951108861	2.03E-06
TUCP_003042	XLOC_129530	ENSRNOT00000026543	0.977679247	4.20E-08
TUCP_003042	XLOC_129530	ENSRNOT00000065501	0.953633604	1.56E-06
TUCP_003042	XLOC_129530	ENSRNOT00000025832	0.95380544	1.53E-06
TUCP_003042	XLOC_129530	ENSRNOT00000083220	0.955705335	1.25E-06
TUCP_003042	XLOC_129530	ENSRNOT00000050180	0.961257888	6.44E-07
TUCP_003042	XLOC_129530	ENSRNOT00000012048	0.954297977	1.45E-06
TUCP_003042	XLOC_129530	ENSRNOT00000060499	0.951218569	2.00E-06
TUCP_003042	XLOC_129530	ENSRNOT00000071407	0.966231842	3.27E-07
TUCP_003042	XLOC_129530	ENSRNOT00000020673	0.955884131	1.22E-06
TUCP_003042	XLOC_129530	ENSRNOT00000045882	0.951548398	1.94E-06
TUCP_003042	XLOC_129530	ENSRNOT00000076669	0.963293627	4.93E-07
TUCP_003042	XLOC_129530	ENSRNOT00000060362	0.957978076	9.62E-07
TUCP_003042	XLOC_129530	ENSRNOT00000015799	0.968380776	2.36E-07
TUCP_003042	XLOC_129530	ENSRNOT00000013274	0.95076146	2.10E-06
TUCP_003042	XLOC_129530	ENSRNOT00000075558	0.952506173	1.76E-06
TUCP_003042	XLOC_129530	ENSRNOT00000025859	0.951005351	2.05E-06

TUCP_003042	XLOC_129530	ENSRNOT00000067663	0.952343195	1.79E-06
TUCP_003042	XLOC_129530	ENSRNOT00000037790	0.952119263	1.83E-06
TUCP_003042	XLOC_129530	ENSRNOT00000021674	0.953317198	1.61E-06
TUCP_003042	XLOC_129530	ENSRNOT00000067400	0.965372246	3.70E-07
TUCP_003042	XLOC_129530	ENSRNOT00000035989	0.956614907	1.13E-06
TUCP_003042	XLOC_129530	ENSRNOT00000067866	0.963863005	4.57E-07
TUCP_003042	XLOC_129530	ENSRNOT00000024402	0.971569226	1.39E-07
TUCP_003042	XLOC_129530	ENSRNOT00000088054	0.970271333	1.74E-07
TUCP_003042	XLOC_129530	ENSRNOT00000091265	0.950454016	2.16E-06
TUCP_003042	XLOC_129530	ENSRNOT00000025075	0.953724402	1.55E-06
TUCP_003042	XLOC_129530	ENSRNOT00000033975	0.95287907	1.69E-06
TUCP_001741	XLOC_071526	ENSRNOT00000042125	0.951066222	2.03E-06
TUCP_001741	XLOC_071526	ENSRNOT00000000170	-0.952947459	1.68E-06
TUCP_001741	XLOC_071526	ENSRNOT00000041044	0.959212193	8.30E-07
TUCP_001741	XLOC_071526	ENSRNOT00000016649	0.951218174	2.00E-06
TUCP_001741	XLOC_071526	ENSRNOT00000081512	0.95134811	1.98E-06
TUCP_001741	XLOC_071526	ENSRNOT00000057160	0.951279833	1.99E-06
TUCP_001741	XLOC_071526	ENSRNOT00000003568	0.973219941	1.04E-07
TUCP_001741	XLOC_071526	ENSRNOT00000023537	-0.954806532	1.38E-06
TUCP_003181	XLOC_136596	ENSRNOT00000024186	0.954883188	1.36E-06
TUCP_003181	XLOC_136596	ENSRNOT00000074753	0.981054039	1.86E-08
TUCP_003181	XLOC_136596	ENSRNOT00000004876	0.950171308	2.22E-06
TUCP_003181	XLOC_136596	ENSRNOT00000041904	0.986338792	3.66E-09
TUCP_003181	XLOC_136596	ENSRNOT00000080193	-0.968215775	2.42E-07
TUCP_003181	XLOC_136596	ENSRNOT00000083487	0.976992802	4.88E-08
TUCP_003181	XLOC_136596	ENSRNOT00000077990	0.986338792	3.66E-09
TUCP_003181	XLOC_136596	ENSRNOT00000038826	0.985683666	4.62E-09
TUCP_003181	XLOC_136596	ENSRNOT00000046842	0.956961333	1.08E-06
TUCP_003181	XLOC_136596	ENSRNOT00000021243	0.966310132	3.23E-07
TUCP_003181	XLOC_136596	ENSRNOT00000060250	0.98571872	4.57E-09
TUCP_003181	XLOC_136596	ENSRNOT00000091934	0.986338792	3.66E-09
TUCP_003181	XLOC_136596	ENSRNOT00000046585	0.966032967	3.36E-07
TUCP_003181	XLOC_136596	ENSRNOT00000082403	-0.963232245	4.97E-07
TUCP_003181	XLOC_136596	ENSRNOT00000080794	0.971742278	1.35E-07
TUCP_003181	XLOC_136596	ENSRNOT00000014456	0.977312759	4.56E-08
TUCP_003181	XLOC_136596	ENSRNOT00000031076	0.971042291	1.53E-07
TUCP_003181	XLOC_136596	ENSRNOT00000086349	0.981315831	1.74E-08
TUCP_003181	XLOC_136596	ENSRNOT00000044145	0.986338792	3.66E-09
TUCP_003181	XLOC_136596	ENSRNOT00000088381	0.970928424	1.56E-07
TUCP_003181	XLOC_136596	ENSRNOT00000011743	0.970900901	1.56E-07
TUCP_003181	XLOC_136596	ENSRNOT00000089929	0.986338792	3.66E-09
TUCP_003181	XLOC_136596	ENSRNOT00000088624	0.986338792	3.66E-09
TUCP_003181	XLOC_136596	ENSRNOT00000085659	0.988379261	1.64E-09
TUCP_003181	XLOC_136596	ENSRNOT00000015866	0.980280776	2.27E-08
TUCP_003181	XLOC_136596	ENSRNOT00000078633	0.986338792	3.66E-09
TUCP_003181	XLOC_136596	ENSRNOT00000034907	0.981481225	1.66E-08
TUCP_003181	XLOC_136596	ENSRNOT00000093230	0.957233122	1.05E-06
TUCP_003181	XLOC_136596	ENSRNOT00000051835	0.986338792	3.66E-09
TUCP_003181	XLOC_136596	ENSRNOT00000079087	0.986338792	3.66E-09
TUCP_003181	XLOC_136596	ENSRNOT00000037206	0.986338792	3.66E-09
TUCP_003181	XLOC_136596	ENSRNOT00000083261	0.959449168	8.07E-07

TUCP_003181	XLOC_136596	ENSRNOT00000080590	0.98615122	3.92E-09
TUCP_003181	XLOC_136596	ENSRNOT00000027552	0.985614948	4.74E-09
TUCP_003181	XLOC_136596	ENSRNOT00000077014	0.975521804	6.64E-08
TUCP_003181	XLOC_136596	ENSRNOT00000080453	0.986278703	3.74E-09
TUCP_003181	XLOC_136596	ENSRNOT00000089057	0.960412413	7.16E-07
TUCP_003181	XLOC_136596	ENSRNOT00000057729	0.98986597	8.28E-10
TUCP_003181	XLOC_136596	ENSRNOT00000061284	0.95554017	1.27E-06
TUCP_003181	XLOC_136596	ENSRNOT00000071884	0.992780072	1.53E-10
TUCP_003181	XLOC_136596	ENSRNOT00000066839	0.974165882	8.68E-08
TUCP_003181	XLOC_136596	ENSRNOT00000031205	0.973784961	9.33E-08
TUCP_003181	XLOC_136596	ENSRNOT00000078663	0.964137307	4.40E-07
TUCP_003181	XLOC_136596	ENSRNOT00000064250	0.964303011	4.30E-07
TUCP_003181	XLOC_136596	ENSRNOT00000037169	0.962402908	5.55E-07
TUCP_003181	XLOC_136596	ENSRNOT00000029634	0.979064261	3.06E-08
TUCP_003181	XLOC_136596	ENSRNOT00000093579	0.968614541	2.28E-07
TUCP_003181	XLOC_136596	ENSRNOT00000000215	0.973167994	1.05E-07
TUCP_003181	XLOC_136596	ENSRNOT00000065460	0.986338792	3.66E-09
TUCP_003181	XLOC_136596	ENSRNOT00000003817	0.977749168	4.14E-08
TUCP_003181	XLOC_136596	ENSRNOT00000016713	0.986338792	3.66E-09
TUCP_003181	XLOC_136596	ENSRNOT00000079260	0.980152977	2.35E-08
TUCP_003181	XLOC_136596	ENSRNOT00000039294	0.986338792	3.66E-09
TUCP_000167	XLOC_004792	ENSRNOT00000010358	0.963952355	4.51E-07
TUCP_000167	XLOC_004792	ENSRNOT00000018970	0.951652562	1.92E-06
TUCP_000167	XLOC_004792	ENSRNOT00000038188	0.954597426	1.41E-06
TUCP_000167	XLOC_004792	ENSRNOT00000028818	0.96312742	5.05E-07
TUCP_000167	XLOC_004792	ENSRNOT00000010142	0.950777458	2.09E-06
TUCP_000166	XLOC_004785	ENSRNOT00000079547	0.952662979	1.73E-06
TUCP_000166	XLOC_004785	ENSRNOT00000051000	0.977655087	4.23E-08
TUCP_000165	XLOC_004498	ENSRNOT00000086078	0.951718937	1.90E-06
TUCP_000696	XLOC_026272	ENSRNOT00000024825	0.957757287	9.87E-07
TUCP_000696	XLOC_026272	ENSRNOT00000077462	0.971502925	1.41E-07
TUCP_000696	XLOC_026272	ENSRNOT00000075569	0.951608203	1.93E-06
TUCP_000162	XLOC_004311	ENSRNOT00000006835	0.953193057	1.64E-06
TUCP_000162	XLOC_004311	ENSRNOT00000007350	0.962291756	5.64E-07
TUCP_000162	XLOC_004311	ENSRNOT00000004400	0.95957233	7.95E-07
TUCP_000162	XLOC_004311	ENSRNOT00000002428	0.957736061	9.89E-07
TUCP_000162	XLOC_004311	ENSRNOT00000083578	0.950781488	2.09E-06
TUCP_000162	XLOC_004311	ENSRNOT00000006217	0.981253032	1.77E-08
TUCP_000162	XLOC_004311	ENSRNOT00000079138	0.95003336	2.25E-06
TUCP_000162	XLOC_004311	ENSRNOT00000049459	-0.951931258	1.86E-06
TUCP_000162	XLOC_004311	ENSRNOT00000065947	0.965648046	3.56E-07
TUCP_000162	XLOC_004311	ENSRNOT00000036051	0.956638825	1.12E-06
TUCP_000162	XLOC_004311	ENSRNOT00000084567	0.964722524	4.06E-07
TUCP_000162	XLOC_004311	ENSRNOT00000007855	0.956920312	1.09E-06
TUCP_000162	XLOC_004311	ENSRNOT00000052063	0.973194497	1.04E-07
TUCP_000162	XLOC_004311	ENSRNOT00000055437	-0.95512164	1.33E-06
TUCP_000162	XLOC_004311	ENSRNOT00000055540	0.950065892	2.25E-06
TUCP_000162	XLOC_004311	ENSRNOT00000019057	-0.964314249	4.29E-07
TUCP_000162	XLOC_004311	ENSRNOT00000013516	0.951206184	2.01E-06
TUCP_000162	XLOC_004311	ENSRNOT00000001507	0.952428598	1.77E-06
TUCP_000162	XLOC_004311	ENSRNOT00000080090	0.957740356	9.89E-07

TUCP_000162	XLOC_004311	ENSRNOT00000088125	0.958223378	9.34E-07
TUCP_000162	XLOC_004311	ENSRNOT00000012060	0.95404546	1.49E-06
TUCP_000162	XLOC_004311	ENSRNOT00000025079	-0.953798858	1.53E-06
TUCP_000162	XLOC_004311	ENSRNOT00000018584	0.96404939	4.45E-07
TUCP_000162	XLOC_004311	ENSRNOT00000010418	0.950676395	2.12E-06
TUCP_000162	XLOC_004311	ENSRNOT00000012755	0.952529127	1.75E-06
TUCP_000162	XLOC_004311	ENSRNOT00000017271	0.977941977	3.96E-08
TUCP_000162	XLOC_004311	ENSRNOT00000024677	0.967359743	2.76E-07
TUCP_000162	XLOC_004311	ENSRNOT00000025517	0.952464101	1.76E-06
TUCP_000162	XLOC_004311	ENSRNOT00000003146	-0.956936723	1.08E-06
TUCP_000162	XLOC_004311	ENSRNOT00000026391	0.978041723	3.88E-08
TUCP_000162	XLOC_004311	ENSRNOT00000034450	0.963838085	4.58E-07
TUCP_000162	XLOC_004311	ENSRNOT00000005686	-0.952210911	1.81E-06
TUCP_000162	XLOC_004311	ENSRNOT00000005292	0.950534132	2.15E-06
TUCP_000162	XLOC_004311	ENSRNOT00000087278	-0.959087142	8.43E-07
TUCP_000162	XLOC_004311	ENSRNOT00000030074	0.95336737	1.61E-06
TUCP_000162	XLOC_004311	ENSRNOT00000068745	0.954948605	1.35E-06
TUCP_000162	XLOC_004311	ENSRNOT00000034298	0.961160355	6.52E-07
TUCP_000162	XLOC_004311	ENSRNOT00000024352	0.95395058	1.51E-06
TUCP_000162	XLOC_004311	ENSRNOT00000003544	-0.954070413	1.49E-06
TUCP_000162	XLOC_004311	ENSRNOT00000016445	0.964787206	4.02E-07
TUCP_000162	XLOC_004311	ENSRNOT00000058495	-0.95015281	2.23E-06
TUCP_000162	XLOC_004311	ENSRNOT00000011956	0.962899084	5.20E-07
TUCP_000162	XLOC_004311	ENSRNOT00000073919	0.978680106	3.35E-08
TUCP_000162	XLOC_004311	ENSRNOT00000023667	0.952652445	1.73E-06
TUCP_000162	XLOC_004311	ENSRNOT00000026089	0.969604518	1.94E-07
TUCP_000162	XLOC_004311	ENSRNOT00000001158	0.971388691	1.44E-07
TUCP_000162	XLOC_004311	ENSRNOT00000055683	0.963457386	4.83E-07
TUCP_000162	XLOC_004311	ENSRNOT00000045488	0.951273296	1.99E-06
TUCP_000162	XLOC_004311	ENSRNOT00000002465	0.953772179	1.54E-06
TUCP_000162	XLOC_004311	ENSRNOT00000000898	0.972343879	1.22E-07
TUCP_000162	XLOC_004311	ENSRNOT00000077706	0.965935948	3.41E-07
TUCP_000162	XLOC_004311	ENSRNOT00000020383	0.980663371	2.06E-08
TUCP_000162	XLOC_004311	ENSRNOT00000003840	0.956317065	1.16E-06
TUCP_000162	XLOC_004311	ENSRNOT00000076230	0.958817464	8.71E-07
TUCP_000162	XLOC_004311	ENSRNOT00000084012	0.952094565	1.83E-06
TUCP_000162	XLOC_004311	ENSRNOT00000025523	0.961671365	6.11E-07
TUCP_000162	XLOC_004311	ENSRNOT00000066032	0.956915117	1.09E-06
TUCP_000162	XLOC_004311	ENSRNOT00000093385	0.963322075	4.92E-07
TUCP_000162	XLOC_004311	ENSRNOT00000015239	-0.957728816	9.90E-07
TUCP_000162	XLOC_004311	ENSRNOT00000027264	0.950404175	2.17E-06
TUCP_000162	XLOC_004311	ENSRNOT00000000718	0.957742721	9.88E-07
TUCP_000162	XLOC_004311	ENSRNOT00000077146	-0.956203101	1.18E-06
TUCP_000162	XLOC_004311	ENSRNOT00000001783	0.952245105	1.80E-06
TUCP_000162	XLOC_004311	ENSRNOT00000021143	0.969740603	1.90E-07
TUCP_000162	XLOC_004311	ENSRNOT00000005489	0.966573961	3.11E-07
TUCP_000162	XLOC_004311	ENSRNOT00000017765	0.975517461	6.65E-08
TUCP_000162	XLOC_004311	ENSRNOT00000064039	0.972238536	1.24E-07
TUCP_000162	XLOC_004311	ENSRNOT00000001923	0.955601099	1.26E-06
TUCP_000162	XLOC_004311	ENSRNOT00000073041	0.950148885	2.23E-06
TUCP_000162	XLOC_004311	ENSRNOT00000079784	-0.959527731	7.99E-07

TUCP_000162	XLOC_004311	ENSRNOT00000085517	0.976089854	5.91E-08
TUCP_000162	XLOC_004311	ENSRNOT00000067059	0.970904889	1.56E-07
TUCP_000162	XLOC_004311	ENSRNOT00000037679	0.979831801	2.54E-08
TUCP_000162	XLOC_004311	ENSRNOT00000065076	0.958148154	9.43E-07
TUCP_000162	XLOC_004311	ENSRNOT00000012528	0.950853122	2.08E-06
TUCP_000162	XLOC_004311	ENSRNOT00000060698	-0.951133408	2.02E-06
TUCP_000162	XLOC_004311	ENSRNOT00000032244	0.965212467	3.78E-07
TUCP_000162	XLOC_004311	ENSRNOT00000061100	0.956424529	1.15E-06
TUCP_000162	XLOC_004311	ENSRNOT00000005144	0.960487647	7.10E-07
TUCP_000162	XLOC_004311	ENSRNOT00000025097	0.966414074	3.18E-07
TUCP_000162	XLOC_004311	ENSRNOT00000028907	0.96046397	7.12E-07
TUCP_000162	XLOC_004311	ENSRNOT00000081678	0.973212051	1.04E-07
TUCP_000162	XLOC_004311	ENSRNOT00000036613	-0.952749493	1.71E-06
TUCP_000162	XLOC_004311	ENSRNOT00000092876	-0.955293528	1.30E-06
TUCP_000162	XLOC_004311	ENSRNOT00000078862	0.952958685	1.68E-06
TUCP_000162	XLOC_004311	ENSRNOT00000046639	0.967441937	2.73E-07
TUCP_001063	XLOC_040483	ENSRNOT00000025981	0.964182686	4.37E-07
TUCP_001063	XLOC_040483	ENSRNOT00000010992	0.957591282	1.01E-06
TUCP_001063	XLOC_040483	ENSRNOT00000068405	0.956964615	1.08E-06
TUCP_001063	XLOC_040483	ENSRNOT00000083215	0.953262543	1.62E-06
TUCP_001063	XLOC_040483	ENSRNOT00000076669	0.958960385	8.56E-07
TUCP_001063	XLOC_040483	ENSRNOT00000006401	-0.951328807	1.98E-06
TUCP_001063	XLOC_040483	ENSRNOT00000024420	0.969676409	1.92E-07
TUCP_001063	XLOC_040483	ENSRNOT00000005684	0.965832035	3.46E-07
TUCP_001063	XLOC_040483	ENSRNOT00000059458	0.950284498	2.20E-06
TUCP_001063	XLOC_040483	ENSRNOT00000004508	0.958819405	8.70E-07
TUCP_001063	XLOC_040483	ENSRNOT00000082969	0.952154657	1.82E-06
TUCP_001063	XLOC_040483	ENSRNOT00000028155	0.973959573	9.03E-08
TUCP_001063	XLOC_040483	ENSRNOT00000002678	0.95174929	1.90E-06
TUCP_001063	XLOC_040483	ENSRNOT00000015929	0.95279654	1.70E-06
TUCP_001063	XLOC_040483	ENSRNOT00000052288	0.954674188	1.40E-06
TUCP_001063	XLOC_040483	ENSRNOT00000039809	0.95205555	1.84E-06
TUCP_001063	XLOC_040483	ENSRNOT00000064900	0.955061283	1.34E-06
TUCP_001063	XLOC_040483	ENSRNOT00000076729	0.952060335	1.84E-06
TUCP_001063	XLOC_040483	ENSRNOT00000025196	-0.953461201	1.59E-06
TUCP_001063	XLOC_040483	ENSRNOT00000058852	-0.96100844	6.65E-07
TUCP_001063	XLOC_040483	ENSRNOT00000025859	0.95995685	7.58E-07
TUCP_001063	XLOC_040483	ENSRNOT00000044896	0.95441202	1.44E-06
TUCP_001063	XLOC_040483	ENSRNOT00000013026	0.959588429	7.93E-07
TUCP_001063	XLOC_040483	ENSRNOT00000034503	0.953134965	1.65E-06
TUCP_001063	XLOC_040483	ENSRNOT00000022800	0.963912807	4.54E-07
TUCP_001063	XLOC_040483	ENSRNOT00000017012	0.956661051	1.12E-06
TUCP_001063	XLOC_040483	ENSRNOT00000074725	0.955716467	1.24E-06
TUCP_001063	XLOC_040483	ENSRNOT00000013046	-0.955406033	1.29E-06
TUCP_001063	XLOC_040483	ENSRNOT00000024942	0.955558445	1.27E-06
TUCP_001063	XLOC_040483	ENSRNOT00000036646	0.965380744	3.70E-07
TUCP_001063	XLOC_040483	ENSRNOT00000071573	0.956214462	1.18E-06
TUCP_001063	XLOC_040483	ENSRNOT00000022646	0.970946606	1.55E-07
TUCP_001063	XLOC_040483	ENSRNOT00000004412	0.950920455	2.06E-06
TUCP_001063	XLOC_040483	ENSRNOT00000073346	0.955956189	1.21E-06
TUCP_001063	XLOC_040483	ENSRNOT00000051496	0.973431547	9.97E-08

TUCP_001063	XLOC_040483	ENSRNOT00000010898	0.956771019	1.11E-06
TUCP_001063	XLOC_040483	ENSRNOT00000090376	0.952575846	1.74E-06
TUCP_001063	XLOC_040483	ENSRNOT00000084661	0.951531305	1.94E-06
TUCP_001063	XLOC_040483	ENSRNOT00000011622	0.957867428	9.74E-07
TUCP_001063	XLOC_040483	ENSRNOT00000035989	0.962546397	5.45E-07
TUCP_001063	XLOC_040483	ENSRNOT00000044177	0.95120923	2.01E-06
TUCP_001063	XLOC_040483	ENSRNOT00000005355	0.957027204	1.07E-06
TUCP_001063	XLOC_040483	ENSRNOT00000021592	0.962932159	5.18E-07
TUCP_001063	XLOC_040483	ENSRNOT00000026087	-0.960172677	7.38E-07
TUCP_001063	XLOC_040483	ENSRNOT00000017948	0.954551779	1.41E-06
TUCP_001063	XLOC_040483	ENSRNOT00000010242	0.967785981	2.59E-07
TUCP_001063	XLOC_040483	ENSRNOT00000028842	0.987692193	2.18E-09
TUCP_001063	XLOC_040483	ENSRNOT00000057313	0.951964902	1.86E-06
TUCP_001063	XLOC_040483	ENSRNOT00000006800	0.955132959	1.33E-06
TUCP_001063	XLOC_040483	ENSRNOT00000043156	0.95117318	2.01E-06
TUCP_001063	XLOC_040483	ENSRNOT00000090773	-0.950825178	2.08E-06
TUCP_001063	XLOC_040483	ENSRNOT00000085648	0.95918723	8.33E-07
TUCP_000160	XLOC_004301	ENSRNOT00000017090	0.963810576	4.60E-07
TUCP_000160	XLOC_004301	ENSRNOT00000058725	0.955770914	1.24E-06
TUCP_000041	XLOC_000890	ENSRNOT00000026949	-0.972877464	1.10E-07
TUCP_000041	XLOC_000890	ENSRNOT00000009440	-0.952140652	1.82E-06
TUCP_000041	XLOC_000890	ENSRNOT00000021818	-0.966646479	3.07E-07
TUCP_000041	XLOC_000890	ENSRNOT00000007185	-0.953276241	1.62E-06
TUCP_000041	XLOC_000890	ENSRNOT00000010103	-0.95423277	1.46E-06
TUCP_000041	XLOC_000890	ENSRNOT00000014738	-0.957450895	1.02E-06
TUCP_000041	XLOC_000890	ENSRNOT00000078639	0.957729891	9.90E-07
TUCP_000041	XLOC_000890	ENSRNOT00000087725	0.962434989	5.53E-07
TUCP_000041	XLOC_000890	ENSRNOT00000019769	-0.958074701	9.51E-07
TUCP_000042	XLOC_001015	ENSRNOT00000092026	0.96620614	3.28E-07
TUCP_000042	XLOC_001015	ENSRNOT00000093075	1	0
TUCP_000042	XLOC_001015	ENSRNOT00000058260	1	0
TUCP_000042	XLOC_001015	ENSRNOT00000088887	1	4.25E-78
TUCP_000042	XLOC_001015	ENSRNOT00000084986	1	0
TUCP_000042	XLOC_001015	ENSRNOT00000074346	0.9702256	1.75E-07
TUCP_000042	XLOC_001015	ENSRNOT00000073209	0.983711602	8.79E-09
TUCP_000042	XLOC_001015	ENSRNOT00000080155	0.973069673	1.07E-07
TUCP_000042	XLOC_001015	ENSRNOT00000072573	0.996213272	6.09E-12
TUCP_000042	XLOC_001015	ENSRNOT00000017828	0.996001583	7.99E-12
TUCP_000042	XLOC_001015	ENSRNOT00000080444	0.969625907	1.93E-07
TUCP_000042	XLOC_001015	ENSRNOT00000071312	1	0
TUCP_000042	XLOC_001015	ENSRNOT00000088245	1	0
TUCP_000042	XLOC_001015	ENSRNOT00000036565	0.969065335	2.12E-07
TUCP_000042	XLOC_001015	ENSRNOT00000058833	0.998673261	3.23E-14
TUCP_000042	XLOC_001015	ENSRNOT00000078052	1	0
TUCP_000042	XLOC_001015	ENSRNOT00000078494	0.999927093	1.62E-20
TUCP_000042	XLOC_001015	ENSRNOT00000085653	1	0
TUCP_000042	XLOC_001015	ENSRNOT00000057950	0.969329371	2.03E-07
TUCP_000042	XLOC_001015	ENSRNOT00000081992	0.999320526	1.14E-15
TUCP_000042	XLOC_001015	ENSRNOT00000038553	0.955765808	1.24E-06
TUCP_000042	XLOC_001015	ENSRNOT00000084120	0.958115447	9.46E-07
TUCP_000042	XLOC_001015	ENSRNOT00000055406	1	1.33E-79

TUCP_000042	XLOC_001015	ENSRNOT00000070817	0.998968079	9.20E-15
TUCP_000042	XLOC_001015	ENSRNOT00000026753	1	1.33E-79
TUCP_000042	XLOC_001015	ENSRNOT00000080533	0.996682529	3.15E-12
TUCP_000042	XLOC_001015	ENSRNOT00000072774	0.979665261	2.65E-08
TUCP_000042	XLOC_001015	ENSRNOT00000086031	1	1.33E-79
TUCP_000042	XLOC_001015	ENSRNOT00000014312	1	0
TUCP_000042	XLOC_001015	ENSRNOT00000042882	0.963218427	4.98E-07
TUCP_000042	XLOC_001015	ENSRNOT00000088012	0.956765806	1.11E-06
TUCP_000042	XLOC_001015	ENSRNOT00000051231	0.999242378	1.96E-15
TUCP_000042	XLOC_001015	ENSRNOT00000089407	0.992986194	1.32E-10
TUCP_000042	XLOC_001015	ENSRNOT00000055034	0.998331441	1.02E-13
TUCP_000042	XLOC_001015	ENSRNOT00000046837	0.97252559	1.18E-07
TUCP_000296	XLOC_009318	ENSRNOT00000008041	0.951692126	1.91E-06
TUCP_001069	XLOC_040982	ENSRNOT00000084075	0.996106112	7.00E-12
TUCP_001069	XLOC_040982	ENSRNOT00000012880	0.968043129	2.49E-07
TUCP_001069	XLOC_040982	ENSRNOT00000074465	1	0
TUCP_001069	XLOC_040982	ENSRNOT00000090898	0.961670541	6.11E-07
TUCP_001069	XLOC_040982	ENSRNOT00000079805	0.997980451	2.64E-13
TUCP_001069	XLOC_040982	ENSRNOT00000040912	1	0
TUCP_001069	XLOC_040982	ENSRNOT00000072648	1	0
TUCP_001069	XLOC_040982	ENSRNOT00000007398	0.989054103	1.21E-09
TUCP_001069	XLOC_040982	ENSRNOT00000070803	1	0
TUCP_001069	XLOC_040982	ENSRNOT00000012196	1	0
TUCP_001069	XLOC_040982	ENSRNOT00000085772	1	0
TUCP_001069	XLOC_040982	ENSRNOT00000093284	0.999975856	6.46E-23
TUCP_001069	XLOC_040982	ENSRNOT00000086374	0.993568904	8.57E-11
TUCP_001069	XLOC_040982	ENSRNOT00000015270	1	0
TUCP_001069	XLOC_040982	ENSRNOT00000046192	1	0
TUCP_001069	XLOC_040982	ENSRNOT00000044226	1	0
TUCP_001069	XLOC_040982	ENSRNOT00000081898	0.989837012	8.39E-10
TUCP_001069	XLOC_040982	ENSRNOT00000081954	0.964005219	4.48E-07
TUCP_001069	XLOC_040982	ENSRNOT00000080291	0.998427671	7.55E-14
TUCP_001069	XLOC_040982	ENSRNOT00000004293	0.984239049	7.46E-09
TUCP_001069	XLOC_040982	ENSRNOT00000078231	0.960455489	7.13E-07
TUCP_001069	XLOC_040982	ENSRNOT00000002834	1	0
TUCP_001069	XLOC_040982	ENSRNOT00000001464	1	0
TUCP_001069	XLOC_040982	ENSRNOT00000090716	0.994769629	3.06E-11
TUCP_001069	XLOC_040982	ENSRNOT00000083542	0.972568514	1.17E-07
TUCP_001069	XLOC_040982	ENSRNOT00000076044	1	0
TUCP_001069	XLOC_040982	ENSRNOT00000074353	1	0
TUCP_001069	XLOC_040982	ENSRNOT00000043510	0.958144129	9.43E-07
TUCP_001069	XLOC_040982	ENSRNOT00000087122	0.980089513	2.38E-08
TUCP_001069	XLOC_040982	ENSRNOT00000074295	0.987777006	2.11E-09
TUCP_001069	XLOC_040982	ENSRNOT00000057873	1	0
TUCP_001069	XLOC_040982	ENSRNOT00000083002	0.988874897	1.32E-09
TUCP_001069	XLOC_040982	ENSRNOT00000088399	0.999999855	4.99E-34
TUCP_001069	XLOC_040982	ENSRNOT00000086248	1	0
TUCP_001069	XLOC_040982	ENSRNOT00000089750	0.999992758	1.57E-25
TUCP_001069	XLOC_040982	ENSRNOT00000030517	0.971225224	1.48E-07
TUCP_001069	XLOC_040982	ENSRNOT00000034772	0.994265187	4.84E-11
TUCP_001069	XLOC_040982	ENSRNOT00000089305	0.969641693	1.93E-07

TUCP_001069	XLOC_040982	ENSRNOT00000024580	0.954183021	1.47E-06
TUCP_001069	XLOC_040982	ENSRNOT00000064601	0.966578038	3.11E-07
TUCP_001069	XLOC_040982	ENSRNOT00000015870	0.965901306	3.43E-07
TUCP_001069	XLOC_040982	ENSRNOT00000080812	0.992998865	1.31E-10
TUCP_001069	XLOC_040982	ENSRNOT00000092755	1	0
TUCP_001069	XLOC_040982	ENSRNOT00000031683	1	0
TUCP_001069	XLOC_040982	ENSRNOT00000087764	0.988156196	1.80E-09
TUCP_001183	XLOC_044723	ENSRNOT00000084075	-0.962693402	5.35E-07
TUCP_001183	XLOC_044723	ENSRNOT00000074465	-0.963086882	5.07E-07
TUCP_001183	XLOC_044723	ENSRNOT00000090898	-0.98104209	1.87E-08
TUCP_001183	XLOC_044723	ENSRNOT00000079805	-0.953452063	1.59E-06
TUCP_001183	XLOC_044723	ENSRNOT00000040912	-0.963086882	5.07E-07
TUCP_001183	XLOC_044723	ENSRNOT00000072648	-0.963086882	5.07E-07
TUCP_001183	XLOC_044723	ENSRNOT00000007398	-0.96993108	1.84E-07
TUCP_001183	XLOC_044723	ENSRNOT00000070803	-0.963086882	5.07E-07
TUCP_001183	XLOC_044723	ENSRNOT00000012196	-0.963086882	5.07E-07
TUCP_001183	XLOC_044723	ENSRNOT00000085772	-0.963086882	5.07E-07
TUCP_001183	XLOC_044723	ENSRNOT00000093284	-0.963328202	4.91E-07
TUCP_001183	XLOC_044723	ENSRNOT00000015270	-0.963086882	5.07E-07
TUCP_001183	XLOC_044723	ENSRNOT00000046192	-0.963086882	5.07E-07
TUCP_001183	XLOC_044723	ENSRNOT00000044226	-0.963086882	5.07E-07
TUCP_001183	XLOC_044723	ENSRNOT00000081898	-0.966840791	2.99E-07
TUCP_001183	XLOC_044723	ENSRNOT00000081954	-0.953088841	1.65E-06
TUCP_001183	XLOC_044723	ENSRNOT00000080291	-0.962270865	5.65E-07
TUCP_001183	XLOC_044723	ENSRNOT00000015870	-0.968903881	2.17E-07
TUCP_001183	XLOC_044723	ENSRNOT00000001464	-0.963086882	5.07E-07
TUCP_001183	XLOC_044723	ENSRNOT00000090716	-0.950977652	2.05E-06
TUCP_001183	XLOC_044723	ENSRNOT00000057873	-0.963086882	5.07E-07
TUCP_001183	XLOC_044723	ENSRNOT00000076044	-0.963086882	5.07E-07
TUCP_001183	XLOC_044723	ENSRNOT00000074353	-0.963086882	5.07E-07
TUCP_001183	XLOC_044723	ENSRNOT00000074295	-0.953780908	1.54E-06
TUCP_001183	XLOC_044723	ENSRNOT00000083542	-0.967349307	2.77E-07
TUCP_001183	XLOC_044723	ENSRNOT00000088399	-0.963108329	5.06E-07
TUCP_001183	XLOC_044723	ENSRNOT00000086248	-0.963086882	5.07E-07
TUCP_001183	XLOC_044723	ENSRNOT00000089750	-0.963075653	5.08E-07
TUCP_001183	XLOC_044723	ENSRNOT00000034772	-0.959899688	7.63E-07
TUCP_001183	XLOC_044723	ENSRNOT00000002834	-0.963086882	5.07E-07
TUCP_001183	XLOC_044723	ENSRNOT00000080812	-0.958143783	9.43E-07
TUCP_001183	XLOC_044723	ENSRNOT00000092755	-0.963086882	5.07E-07
TUCP_001183	XLOC_044723	ENSRNOT00000031683	-0.963086882	5.07E-07
TUCP_000046	XLOC_001084	ENSRNOT00000087739	0.952811767	1.70E-06
TUCP_000046	XLOC_001084	ENSRNOT00000000008	0.952811767	1.70E-06
TUCP_000046	XLOC_001084	ENSRNOT00000085822	0.960486156	7.10E-07
TUCP_000046	XLOC_001084	ENSRNOT00000022091	0.952811767	1.70E-06
TUCP_000046	XLOC_001084	ENSRNOT00000048523	0.952811767	1.70E-06
TUCP_000046	XLOC_001084	ENSRNOT00000026428	0.966196437	3.28E-07
TUCP_000046	XLOC_001084	ENSRNOT00000065376	0.952811767	1.70E-06
TUCP_000046	XLOC_001084	ENSRNOT00000074378	0.954045173	1.49E-06
TUCP_000046	XLOC_001084	ENSRNOT00000093571	0.952811767	1.70E-06
TUCP_000046	XLOC_001084	ENSRNOT00000085673	0.952811767	1.70E-06
TUCP_000046	XLOC_001084	ENSRNOT00000050573	0.952811767	1.70E-06

TUCP_000046	XLOC_001084	ENSRNOT00000090581	0.952811767	1.70E-06
TUCP_000046	XLOC_001084	ENSRNOT00000083575	0.952811767	1.70E-06
TUCP_000046	XLOC_001084	ENSRNOT00000076239	0.969750883	1.90E-07
TUCP_000046	XLOC_001084	ENSRNOT00000006219	0.952811767	1.70E-06
TUCP_000046	XLOC_001084	ENSRNOT00000076927	0.950160694	2.23E-06
TUCP_000046	XLOC_001084	ENSRNOT00000079826	0.952811767	1.70E-06
TUCP_000046	XLOC_001084	ENSRNOT00000092395	0.95085252	2.08E-06
TUCP_000046	XLOC_001084	ENSRNOT00000065485	0.952811767	1.70E-06
TUCP_000047	XLOC_001084	ENSRNOT00000091652	0.958384651	9.17E-07
TUCP_000047	XLOC_001084	ENSRNOT00000080865	0.962716145	5.33E-07
TUCP_000047	XLOC_001084	ENSRNOT00000073534	0.974683768	7.85E-08
TUCP_000047	XLOC_001084	ENSRNOT00000012161	0.958204398	9.36E-07
TUCP_000047	XLOC_001084	ENSRNOT00000071825	0.993955664	6.29E-11
TUCP_000047	XLOC_001084	ENSRNOT00000093019	0.962716145	5.33E-07
TUCP_000047	XLOC_001084	ENSRNOT00000085908	0.957374852	1.03E-06
TUCP_000047	XLOC_001084	ENSRNOT00000046744	0.962716145	5.33E-07
TUCP_000047	XLOC_001084	ENSRNOT00000076429	0.962716145	5.33E-07
TUCP_000047	XLOC_001084	ENSRNOT00000084667	0.960399663	7.18E-07
TUCP_000047	XLOC_001084	ENSRNOT00000055412	0.962716145	5.33E-07
TUCP_000047	XLOC_001084	ENSRNOT00000093201	0.960717342	6.90E-07
TUCP_000047	XLOC_001084	ENSRNOT00000072724	0.962716145	5.33E-07
TUCP_000047	XLOC_001084	ENSRNOT00000066017	0.96908207	2.11E-07
TUCP_000047	XLOC_001084	ENSRNOT00000073148	0.96962161	1.94E-07
TUCP_000047	XLOC_001084	ENSRNOT00000084375	0.962716145	5.33E-07
TUCP_000047	XLOC_001084	ENSRNOT00000006249	0.997353837	1.02E-12
TUCP_000047	XLOC_001084	ENSRNOT00000042369	0.963342537	4.90E-07
TUCP_000047	XLOC_001084	ENSRNOT00000086554	0.962716145	5.33E-07
TUCP_000047	XLOC_001084	ENSRNOT00000047921	0.962716145	5.33E-07
TUCP_000047	XLOC_001084	ENSRNOT00000064261	0.962154058	5.74E-07
TUCP_000047	XLOC_001084	ENSRNOT00000086660	0.960240011	7.32E-07
TUCP_000047	XLOC_001084	ENSRNOT00000083977	0.962716145	5.33E-07
TUCP_000047	XLOC_001084	ENSRNOT00000092933	0.962716145	5.33E-07
TUCP_000047	XLOC_001084	ENSRNOT00000085447	0.956274844	1.17E-06
TUCP_000047	XLOC_001084	ENSRNOT00000041281	0.951784304	1.89E-06
TUCP_003456	XLOC_149596	ENSRNOT00000071840	0.960017683	7.52E-07
TUCP_003456	XLOC_149596	ENSRNOT00000082776	0.990914033	4.80E-10
TUCP_003456	XLOC_149596	ENSRNOT00000077995	0.962408328	5.55E-07
TUCP_003456	XLOC_149596	ENSRNOT00000066004	0.958781407	8.74E-07
TUCP_003456	XLOC_149596	ENSRNOT00000086962	0.970231732	1.75E-07
TUCP_003456	XLOC_149596	ENSRNOT00000075836	0.960017683	7.52E-07
TUCP_003456	XLOC_149596	ENSRNOT00000083747	0.971983216	1.30E-07
TUCP_003456	XLOC_149596	ENSRNOT00000092182	0.960017683	7.52E-07
TUCP_003456	XLOC_149596	ENSRNOT00000084461	0.960017683	7.52E-07
TUCP_003456	XLOC_149596	ENSRNOT00000060448	0.960017683	7.52E-07
TUCP_003456	XLOC_149596	ENSRNOT00000032631	0.960017683	7.52E-07
TUCP_003456	XLOC_149596	ENSRNOT00000079503	0.967192433	2.83E-07
TUCP_003456	XLOC_149596	ENSRNOT00000060113	0.955238924	1.31E-06
TUCP_003456	XLOC_149596	ENSRNOT00000056490	0.957060094	1.07E-06
TUCP_003456	XLOC_149596	ENSRNOT00000054910	0.959745986	7.78E-07
TUCP_003456	XLOC_149596	ENSRNOT00000057176	0.959952242	7.58E-07
TUCP_003456	XLOC_149596	ENSRNOT00000093431	0.960017683	7.52E-07

TUCP_003456	XLOC_149596	ENSRNOT00000092901	0.960017683	7.52E-07
TUCP_003456	XLOC_149596	ENSRNOT00000089290	0.959704154	7.82E-07
TUCP_003456	XLOC_149596	ENSRNOT00000071603	0.959885731	7.65E-07
TUCP_003456	XLOC_149596	ENSRNOT00000075244	0.961867163	5.96E-07
TUCP_003456	XLOC_149596	ENSRNOT00000091828	0.951759104	1.90E-06
TUCP_003456	XLOC_149596	ENSRNOT00000091944	0.9581644	9.41E-07
TUCP_003456	XLOC_149596	ENSRNOT00000070855	0.960017683	7.52E-07
TUCP_003456	XLOC_149596	ENSRNOT00000084109	0.959634151	7.89E-07
TUCP_003456	XLOC_149596	ENSRNOT00000007683	0.959889611	7.64E-07
TUCP_003456	XLOC_149596	ENSRNOT00000041569	0.960017683	7.52E-07
TUCP_003456	XLOC_149596	ENSRNOT00000087091	0.960017683	7.52E-07
TUCP_003456	XLOC_149596	ENSRNOT00000003308	0.9561498	1.19E-06
TUCP_003456	XLOC_149596	ENSRNOT00000026113	0.95910699	8.41E-07
TUCP_003456	XLOC_149596	ENSRNOT00000014939	0.95910953	8.40E-07
TUCP_003456	XLOC_149596	ENSRNOT00000090211	0.961719802	6.07E-07
TUCP_003456	XLOC_149596	ENSRNOT00000023287	0.959249324	8.26E-07
TUCP_003456	XLOC_149596	ENSRNOT00000073229	0.960017683	7.52E-07
TUCP_003456	XLOC_149596	ENSRNOT00000045584	0.960017683	7.52E-07
TUCP_003456	XLOC_149596	ENSRNOT00000060454	0.960017683	7.52E-07
TUCP_003456	XLOC_149596	ENSRNOT00000009295	0.957134693	1.06E-06
TUCP_002341	XLOC_097556	ENSRNOT00000028034	0.954467922	1.43E-06
TUCP_002341	XLOC_097556	ENSRNOT00000023791	0.956312564	1.16E-06
TUCP_003454	XLOC_149429	ENSRNOT00000084133	1	0
TUCP_003454	XLOC_149429	ENSRNOT00000093745	0.981891039	1.49E-08
TUCP_003454	XLOC_149429	ENSRNOT00000065542	0.99828699	1.16E-13
TUCP_003454	XLOC_149429	ENSRNOT00000082464	1	0
TUCP_003454	XLOC_149429	ENSRNOT00000087659	1	0
TUCP_003454	XLOC_149429	ENSRNOT00000084582	0.976633579	5.28E-08
TUCP_003454	XLOC_149429	ENSRNOT00000085078	1	0
TUCP_003454	XLOC_149429	ENSRNOT00000014028	1	0
TUCP_003454	XLOC_149429	ENSRNOT00000049363	1	4.25E-78
TUCP_003454	XLOC_149429	ENSRNOT00000065473	0.999949098	2.69E-21
TUCP_003454	XLOC_149429	ENSRNOT00000000142	0.998996155	8.01E-15
TUCP_003454	XLOC_149429	ENSRNOT00000082288	0.999998398	8.30E-29
TUCP_003454	XLOC_149429	ENSRNOT00000039664	1	0
TUCP_003454	XLOC_149429	ENSRNOT00000065402	1	4.25E-78
TUCP_003454	XLOC_149429	ENSRNOT00000064551	0.965365872	3.70E-07
TUCP_003454	XLOC_149429	ENSRNOT00000084141	1	0
TUCP_003454	XLOC_149429	ENSRNOT00000082435	1	0
TUCP_003454	XLOC_149429	ENSRNOT00000082928	0.996801059	2.62E-12
TUCP_003454	XLOC_149429	ENSRNOT00000066546	0.956953619	1.08E-06
TUCP_003454	XLOC_149429	ENSRNOT00000046013	0.988138626	1.81E-09
TUCP_003454	XLOC_149429	ENSRNOT00000067931	0.978893628	3.18E-08
TUCP_003454	XLOC_149429	ENSRNOT00000086425	0.992023315	2.51E-10
TUCP_003454	XLOC_149429	ENSRNOT00000093696	0.956927219	1.09E-06
TUCP_003454	XLOC_149429	ENSRNOT00000036228	0.982337157	1.31E-08
TUCP_003454	XLOC_149429	ENSRNOT00000014030	1	0
TUCP_003454	XLOC_149429	ENSRNOT00000081966	1	1.33E-79
TUCP_003454	XLOC_149429	ENSRNOT00000075906	1	0
TUCP_003454	XLOC_149429	ENSRNOT00000088396	0.981336994	1.73E-08
TUCP_003454	XLOC_149429	ENSRNOT00000049259	0.965500259	3.63E-07

TUCP_003454	XLOC_149429	ENSRNOT00000092838	0.982579749	1.23E-08
TUCP_003454	XLOC_149429	ENSRNOT00000093442	0.959217317	8.30E-07
TUCP_001567	XLOC_060608	ENSRNOT00000091856	0.963047264	5.10E-07
TUCP_001560	XLOC_060269	ENSRNOT00000067741	0.953811687	1.53E-06
TUCP_001560	XLOC_060269	ENSRNOT00000015020	0.974409845	8.28E-08
TUCP_001560	XLOC_060269	ENSRNOT00000046807	0.956352519	1.16E-06
TUCP_001560	XLOC_060269	ENSRNOT00000058453	0.958807996	8.71E-07
TUCP_001560	XLOC_060269	ENSRNOT00000082774	0.973375718	1.01E-07
TUCP_001560	XLOC_060269	ENSRNOT00000026550	-0.951703712	1.91E-06
TUCP_001560	XLOC_060269	ENSRNOT00000006460	-0.958973696	8.54E-07
TUCP_001560	XLOC_060269	ENSRNOT00000065103	0.955400986	1.29E-06
TUCP_001560	XLOC_060269	ENSRNOT00000024033	-0.95887364	8.65E-07
TUCP_001560	XLOC_060269	ENSRNOT00000011869	0.958478581	9.06E-07
TUCP_001560	XLOC_060269	ENSRNOT00000089389	0.951346953	1.98E-06
TUCP_001560	XLOC_060269	ENSRNOT00000081835	0.955492265	1.28E-06
TUCP_001560	XLOC_060269	ENSRNOT00000001412	-0.955352825	1.30E-06
TUCP_001560	XLOC_060269	ENSRNOT00000020472	0.956967305	1.08E-06
TUCP_001560	XLOC_060269	ENSRNOT00000079677	0.967174908	2.84E-07
TUCP_001560	XLOC_060269	ENSRNOT00000000649	0.957184274	1.05E-06
TUCP_001560	XLOC_060269	ENSRNOT00000077479	0.950622885	2.13E-06
TUCP_001560	XLOC_060269	ENSRNOT00000086775	0.976088014	5.91E-08
TUCP_001560	XLOC_060269	ENSRNOT00000030007	0.974634582	7.93E-08
TUCP_001560	XLOC_060269	ENSRNOT00000016265	0.967115789	2.87E-07
TUCP_001560	XLOC_060269	ENSRNOT00000083162	-0.952117843	1.83E-06
TUCP_001560	XLOC_060269	ENSRNOT00000012963	0.978985096	3.12E-08
TUCP_001560	XLOC_060269	ENSRNOT00000027752	-0.95063085	2.13E-06
TUCP_001560	XLOC_060269	ENSRNOT00000018494	0.954052996	1.49E-06
TUCP_001560	XLOC_060269	ENSRNOT00000075917	0.955201685	1.32E-06
TUCP_001560	XLOC_060269	ENSRNOT00000073239	0.951039757	2.04E-06
TUCP_001560	XLOC_060269	ENSRNOT00000065942	0.96197454	5.87E-07
TUCP_001560	XLOC_060269	ENSRNOT00000078366	-0.980100158	2.38E-08
TUCP_001560	XLOC_060269	ENSRNOT00000013182	0.958224622	9.34E-07
TUCP_001560	XLOC_060269	ENSRNOT00000013116	0.951422492	1.96E-06
TUCP_001560	XLOC_060269	ENSRNOT00000042973	0.967579464	2.67E-07
TUCP_001560	XLOC_060269	ENSRNOT00000029508	-0.960878931	6.76E-07
TUCP_001560	XLOC_060269	ENSRNOT00000028089	-0.96020503	7.35E-07
TUCP_001560	XLOC_060269	ENSRNOT00000074261	0.951191328	2.01E-06
TUCP_001560	XLOC_060269	ENSRNOT00000068528	0.97899269	3.11E-08
TUCP_001560	XLOC_060269	ENSRNOT00000011675	-0.950243199	2.21E-06
TUCP_001560	XLOC_060269	ENSRNOT00000027917	-0.964183037	4.37E-07
TUCP_001560	XLOC_060269	ENSRNOT00000004290	-0.951564189	1.94E-06
TUCP_001560	XLOC_060269	ENSRNOT00000039000	0.960323153	7.24E-07
TUCP_001560	XLOC_060269	ENSRNOT00000013768	0.973856309	9.21E-08
TUCP_001560	XLOC_060269	ENSRNOT00000009742	0.954349584	1.45E-06
TUCP_001560	XLOC_060269	ENSRNOT00000083967	-0.96546619	3.65E-07
TUCP_001560	XLOC_060269	ENSRNOT00000001490	-0.956561186	1.13E-06
TUCP_001560	XLOC_060269	ENSRNOT00000076467	0.955754427	1.24E-06
TUCP_001560	XLOC_060269	ENSRNOT00000022389	0.958017465	9.57E-07
TUCP_001560	XLOC_060269	ENSRNOT00000013864	0.956951015	1.08E-06
TUCP_001560	XLOC_060269	ENSRNOT00000018101	0.966103199	3.33E-07
TUCP_001560	XLOC_060269	ENSRNOT00000013809	0.950860028	2.08E-06

TUCP_001560	XLOC_060269	ENSRNOT00000023067	0.958777641	8.75E-07
TUCP_001560	XLOC_060269	ENSRNOT00000006196	0.959036815	8.48E-07
TUCP_001560	XLOC_060269	ENSRNOT00000081110	0.951062042	2.04E-06
TUCP_001560	XLOC_060269	ENSRNOT00000034282	-0.952383686	1.78E-06
TUCP_001560	XLOC_060269	ENSRNOT00000016260	0.982489668	1.26E-08
TUCP_001560	XLOC_060269	ENSRNOT00000031935	0.952893469	1.69E-06
TUCP_001560	XLOC_060269	ENSRNOT00000079609	-0.958513659	9.03E-07
TUCP_001560	XLOC_060269	ENSRNOT00000066776	0.958091047	9.49E-07
TUCP_001560	XLOC_060269	ENSRNOT00000025222	0.969147608	2.09E-07
TUCP_001560	XLOC_060269	ENSRNOT00000050350	0.954704724	1.39E-06
TUCP_001560	XLOC_060269	ENSRNOT00000080522	0.957768246	9.85E-07
TUCP_001560	XLOC_060269	ENSRNOT00000014309	0.95028959	2.20E-06
TUCP_001560	XLOC_060269	ENSRNOT00000004770	0.967692574	2.63E-07
TUCP_001560	XLOC_060269	ENSRNOT00000056022	-0.951583811	1.93E-06
TUCP_001560	XLOC_060269	ENSRNOT00000076230	-0.956703451	1.11E-06
TUCP_001560	XLOC_060269	ENSRNOT00000014533	0.959251458	8.26E-07
TUCP_001560	XLOC_060269	ENSRNOT00000090853	0.950115745	2.24E-06
TUCP_001560	XLOC_060269	ENSRNOT00000079414	0.964418841	4.23E-07
TUCP_001560	XLOC_060269	ENSRNOT00000064849	0.960227564	7.33E-07
TUCP_001560	XLOC_060269	ENSRNOT00000014317	0.966747367	3.03E-07
TUCP_001560	XLOC_060269	ENSRNOT00000021506	0.952569374	1.75E-06
TUCP_001560	XLOC_060269	ENSRNOT00000077303	0.953719589	1.55E-06
TUCP_001560	XLOC_060269	ENSRNOT00000029845	0.95446502	1.43E-06
TUCP_001560	XLOC_060269	ENSRNOT00000022128	0.950170946	2.22E-06
TUCP_001560	XLOC_060269	ENSRNOT00000085911	0.961966771	5.88E-07
TUCP_001560	XLOC_060269	ENSRNOT00000024011	-0.963994183	4.49E-07
TUCP_001560	XLOC_060269	ENSRNOT00000075209	0.959546426	7.97E-07
TUCP_001560	XLOC_060269	ENSRNOT00000072856	0.956372338	1.16E-06
TUCP_001560	XLOC_060269	ENSRNOT00000031261	-0.954471627	1.43E-06
TUCP_001560	XLOC_060269	ENSRNOT00000037681	0.970535274	1.66E-07
TUCP_001560	XLOC_060269	ENSRNOT00000018328	0.962345432	5.60E-07
TUCP_001560	XLOC_060269	ENSRNOT00000050415	0.959434457	8.08E-07
TUCP_001560	XLOC_060269	ENSRNOT00000038610	0.96114544	6.53E-07
TUCP_001560	XLOC_060269	ENSRNOT00000071897	0.950176816	2.22E-06
TUCP_001560	XLOC_060269	ENSRNOT00000090774	0.95669526	1.12E-06
TUCP_001560	XLOC_060269	ENSRNOT00000027739	-0.952011781	1.85E-06
TUCP_003453	XLOC_149260	ENSRNOT00000065143	0.968471222	2.33E-07
TUCP_003453	XLOC_149260	ENSRNOT00000026631	0.972402937	1.20E-07
TUCP_003453	XLOC_149260	ENSRNOT00000000513	0.953735564	1.54E-06
TUCP_003453	XLOC_149260	ENSRNOT00000055321	0.956836057	1.10E-06
TUCP_003453	XLOC_149260	ENSRNOT00000063873	0.955379348	1.29E-06
TUCP_003453	XLOC_149260	ENSRNOT00000004956	0.970566497	1.66E-07
TUCP_003453	XLOC_149260	ENSRNOT00000074286	0.95028658	2.20E-06
TUCP_003453	XLOC_149260	ENSRNOT00000029600	0.979511449	2.75E-08
TUCP_003453	XLOC_149260	ENSRNOT00000016629	0.952738184	1.72E-06
TUCP_003453	XLOC_149260	ENSRNOT00000012942	0.972616635	1.16E-07
TUCP_003453	XLOC_149260	ENSRNOT00000044991	0.970248348	1.75E-07
TUCP_003453	XLOC_149260	ENSRNOT00000025009	0.959556437	7.96E-07
TUCP_003453	XLOC_149260	ENSRNOT00000013852	0.961996158	5.86E-07
TUCP_003453	XLOC_149260	ENSRNOT00000080591	0.953006227	1.67E-06
TUCP_003453	XLOC_149260	ENSRNOT00000077201	-0.950488105	2.16E-06

TUCP_003453	XLOC_149260	ENSRNOT00000032248	0.977540726	4.33E-08
TUCP_003453	XLOC_149260	ENSRNOT00000023032	0.952909162	1.68E-06
TUCP_003453	XLOC_149260	ENSRNOT00000017063	-0.954302698	1.45E-06
TUCP_003453	XLOC_149260	ENSRNOT00000000313	0.952900121	1.69E-06
TUCP_003453	XLOC_149260	ENSRNOT00000005311	0.951742984	1.90E-06
TUCP_003453	XLOC_149260	ENSRNOT00000084553	0.961522744	6.23E-07
TUCP_003453	XLOC_149260	ENSRNOT00000050415	-0.95120031	2.01E-06
TUCP_002189	XLOC_091613	ENSRNOT00000047105	0.953974996	1.51E-06
TUCP_002189	XLOC_091613	ENSRNOT00000001099	-0.95967689	7.85E-07
TUCP_002189	XLOC_091613	ENSRNOT00000040060	-0.956132534	1.19E-06
TUCP_002189	XLOC_091613	ENSRNOT00000032802	-0.950350596	2.19E-06
TUCP_002189	XLOC_091613	ENSRNOT00000090621	0.953718534	1.55E-06
TUCP_002189	XLOC_091613	ENSRNOT00000075656	-0.959970984	7.57E-07
TUCP_002189	XLOC_091613	ENSRNOT00000090946	0.952218002	1.81E-06
TUCP_002189	XLOC_091613	ENSRNOT00000006460	0.96738648	2.75E-07
TUCP_002189	XLOC_091613	ENSRNOT00000075397	0.983673062	8.89E-09
TUCP_002189	XLOC_091613	ENSRNOT00000000083	-0.954103724	1.48E-06
TUCP_002189	XLOC_091613	ENSRNOT00000007350	0.9619453	5.90E-07
TUCP_002189	XLOC_091613	ENSRNOT00000032926	-0.962038625	5.82E-07
TUCP_002189	XLOC_091613	ENSRNOT00000019104	-0.951677789	1.91E-06
TUCP_002189	XLOC_091613	ENSRNOT00000026279	-0.960097308	7.45E-07
TUCP_002189	XLOC_091613	ENSRNOT00000063930	-0.968510234	2.31E-07
TUCP_002189	XLOC_091613	ENSRNOT00000083578	0.953570464	1.57E-06
TUCP_002189	XLOC_091613	ENSRNOT00000006217	0.956217272	1.18E-06
TUCP_002189	XLOC_091613	ENSRNOT00000003087	0.954110565	1.48E-06
TUCP_002189	XLOC_091613	ENSRNOT00000013116	-0.958334477	9.22E-07
TUCP_002189	XLOC_091613	ENSRNOT00000006067	0.953574525	1.57E-06
TUCP_002189	XLOC_091613	ENSRNOT00000021858	0.951267857	1.99E-06
TUCP_002189	XLOC_091613	ENSRNOT00000001855	0.977694287	4.19E-08
TUCP_002189	XLOC_091613	ENSRNOT00000023638	0.957713318	9.92E-07
TUCP_002189	XLOC_091613	ENSRNOT00000001691	0.983451836	9.51E-09
TUCP_002189	XLOC_091613	ENSRNOT00000089319	0.956163002	1.18E-06
TUCP_002189	XLOC_091613	ENSRNOT00000006188	-0.959645314	7.88E-07
TUCP_002189	XLOC_091613	ENSRNOT00000079138	0.959415471	8.10E-07
TUCP_002189	XLOC_091613	ENSRNOT00000039102	0.967382043	2.75E-07
TUCP_002189	XLOC_091613	ENSRNOT00000086706	0.967548735	2.68E-07
TUCP_002189	XLOC_091613	ENSRNOT00000036051	0.955400999	1.29E-06
TUCP_002189	XLOC_091613	ENSRNOT00000085885	-0.955305575	1.30E-06
TUCP_002189	XLOC_091613	ENSRNOT00000084567	0.966267896	3.25E-07
TUCP_002189	XLOC_091613	ENSRNOT00000074023	0.982356704	1.31E-08
TUCP_002189	XLOC_091613	ENSRNOT00000024352	0.957482808	1.02E-06
TUCP_002189	XLOC_091613	ENSRNOT00000008361	0.954007298	1.50E-06
TUCP_002189	XLOC_091613	ENSRNOT00000025309	0.95928038	8.23E-07
TUCP_002189	XLOC_091613	ENSRNOT00000052063	0.957305693	1.04E-06
TUCP_002189	XLOC_091613	ENSRNOT00000009073	-0.95448383	1.43E-06
TUCP_002189	XLOC_091613	ENSRNOT00000089418	0.961026574	6.63E-07
TUCP_002189	XLOC_091613	ENSRNOT00000018074	-0.952777496	1.71E-06
TUCP_002189	XLOC_091613	ENSRNOT00000033017	0.960052991	7.49E-07
TUCP_002189	XLOC_091613	ENSRNOT00000017384	0.951793596	1.89E-06
TUCP_002189	XLOC_091613	ENSRNOT00000045926	0.967818692	2.58E-07
TUCP_002189	XLOC_091613	ENSRNOT00000086260	-0.974135952	8.73E-08

TUCP_002189	XLOC_091613	ENSRNOT00000065076	0.983003228	1.09E-08
TUCP_002189	XLOC_091613	ENSRNOT00000021534	-0.956924508	1.09E-06
TUCP_002189	XLOC_091613	ENSRNOT00000013516	0.957091378	1.07E-06
TUCP_002189	XLOC_091613	ENSRNOT00000003326	-0.964847432	3.98E-07
TUCP_002189	XLOC_091613	ENSRNOT00000022746	-0.953506322	1.58E-06
TUCP_002189	XLOC_091613	ENSRNOT00000016306	-0.959260171	8.25E-07
TUCP_002189	XLOC_091613	ENSRNOT00000083677	-0.955111796	1.33E-06
TUCP_002189	XLOC_091613	ENSRNOT00000072771	0.954260367	1.46E-06
TUCP_002189	XLOC_091613	ENSRNOT00000012060	0.95946196	8.05E-07
TUCP_002189	XLOC_091613	ENSRNOT00000073370	0.971643674	1.38E-07
TUCP_002189	XLOC_091613	ENSRNOT00000023965	-0.952278203	1.80E-06
TUCP_002189	XLOC_091613	ENSRNOT00000083568	0.972977216	1.08E-07
TUCP_002189	XLOC_091613	ENSRNOT00000025079	-0.951501869	1.95E-06
TUCP_002189	XLOC_091613	ENSRNOT00000036137	0.951943991	1.86E-06
TUCP_002189	XLOC_091613	ENSRNOT00000065978	0.959312411	8.20E-07
TUCP_002189	XLOC_091613	ENSRNOT00000075480	-0.962579901	5.43E-07
TUCP_002189	XLOC_091613	ENSRNOT00000039177	-0.956642428	1.12E-06
TUCP_002189	XLOC_091613	ENSRNOT00000020051	0.961709058	6.08E-07
TUCP_002189	XLOC_091613	ENSRNOT00000087838	-0.961218114	6.47E-07
TUCP_002189	XLOC_091613	ENSRNOT00000090338	-0.961843872	5.97E-07
TUCP_002189	XLOC_091613	ENSRNOT00000009080	-0.954420927	1.43E-06
TUCP_002189	XLOC_091613	ENSRNOT00000027760	0.965192485	3.80E-07
TUCP_002189	XLOC_091613	ENSRNOT00000000048	0.97798272	3.93E-08
TUCP_002189	XLOC_091613	ENSRNOT00000024402	0.953576202	1.57E-06
TUCP_002189	XLOC_091613	ENSRNOT00000065871	0.968274727	2.40E-07
TUCP_002189	XLOC_091613	ENSRNOT00000042903	0.962244934	5.67E-07
TUCP_002189	XLOC_091613	ENSRNOT00000057898	-0.96141345	6.31E-07
TUCP_002189	XLOC_091613	ENSRNOT00000024677	0.958620541	8.91E-07
TUCP_002189	XLOC_091613	ENSRNOT00000089933	0.974999955	7.38E-08
TUCP_002189	XLOC_091613	ENSRNOT00000056100	-0.969712739	1.91E-07
TUCP_002189	XLOC_091613	ENSRNOT00000001077	0.958620149	8.91E-07
TUCP_002189	XLOC_091613	ENSRNOT00000025517	0.953681338	1.55E-06
TUCP_002189	XLOC_091613	ENSRNOT00000003412	-0.950616901	2.13E-06
TUCP_002189	XLOC_091613	ENSRNOT00000067554	0.962614304	5.40E-07
TUCP_002189	XLOC_091613	ENSRNOT00000055683	0.957172718	1.06E-06
TUCP_002189	XLOC_091613	ENSRNOT00000026391	0.964404619	4.24E-07
TUCP_002189	XLOC_091613	ENSRNOT00000081226	0.964797822	4.01E-07
TUCP_002189	XLOC_091613	ENSRNOT00000065045	0.958930134	8.59E-07
TUCP_002189	XLOC_091613	ENSRNOT00000005686	-0.96481697	4.00E-07
TUCP_002189	XLOC_091613	ENSRNOT00000018634	-0.966013334	3.37E-07
TUCP_002189	XLOC_091613	ENSRNOT00000019820	0.951091573	2.03E-06
TUCP_002189	XLOC_091613	ENSRNOT00000088133	-0.956861988	1.09E-06
TUCP_002189	XLOC_091613	ENSRNOT00000042125	0.962264693	5.66E-07
TUCP_002189	XLOC_091613	ENSRNOT00000040525	0.961708754	6.08E-07
TUCP_002189	XLOC_091613	ENSRNOT00000011832	-0.970184319	1.77E-07
TUCP_002189	XLOC_091613	ENSRNOT00000031384	0.959126372	8.39E-07
TUCP_002189	XLOC_091613	ENSRNOT00000073643	-0.975846932	6.22E-08
TUCP_002189	XLOC_091613	ENSRNOT00000078349	-0.951415731	1.96E-06
TUCP_002189	XLOC_091613	ENSRNOT00000003940	-0.968734856	2.23E-07
TUCP_002189	XLOC_091613	ENSRNOT00000046552	0.952960046	1.68E-06
TUCP_002189	XLOC_091613	ENSRNOT00000000707	-0.956478007	1.14E-06

TUCP_002189	XLOC_091613	ENSRNOT00000077727	-0.952891133	1.69E-06
TUCP_002189	XLOC_091613	ENSRNOT00000022502	-0.953661886	1.56E-06
TUCP_002189	XLOC_091613	ENSRNOT00000011930	0.954393143	1.44E-06
TUCP_002189	XLOC_091613	ENSRNOT00000083968	-0.955931256	1.22E-06
TUCP_002189	XLOC_091613	ENSRNOT00000037133	0.976183221	5.80E-08
TUCP_002189	XLOC_091613	ENSRNOT00000064772	0.952653791	1.73E-06
TUCP_002189	XLOC_091613	ENSRNOT00000055630	0.950975578	2.05E-06
TUCP_002189	XLOC_091613	ENSRNOT00000084709	0.963825199	4.59E-07
TUCP_002189	XLOC_091613	ENSRNOT00000017949	-0.962378408	5.57E-07
TUCP_002189	XLOC_091613	ENSRNOT00000083964	-0.958646458	8.88E-07
TUCP_002189	XLOC_091613	ENSRNOT00000090912	0.950267967	2.20E-06
TUCP_002189	XLOC_091613	ENSRNOT00000016776	-0.95858006	8.96E-07
TUCP_002189	XLOC_091613	ENSRNOT00000000469	0.951214555	2.00E-06
TUCP_002189	XLOC_091613	ENSRNOT00000048781	0.964570426	4.14E-07
TUCP_002189	XLOC_091613	ENSRNOT00000013867	0.954249432	1.46E-06
TUCP_002189	XLOC_091613	ENSRNOT00000039341	0.951848109	1.88E-06
TUCP_002189	XLOC_091613	ENSRNOT00000021802	0.961959012	5.89E-07
TUCP_002189	XLOC_091613	ENSRNOT00000091909	0.956320498	1.16E-06
TUCP_002189	XLOC_091613	ENSRNOT00000026831	0.957781461	9.84E-07
TUCP_002189	XLOC_091613	ENSRNOT00000018640	0.956112848	1.19E-06
TUCP_002189	XLOC_091613	ENSRNOT00000007821	0.951759592	1.90E-06
TUCP_002189	XLOC_091613	ENSRNOT00000009563	-0.951724103	1.90E-06
TUCP_002189	XLOC_091613	ENSRNOT00000025856	-0.970348968	1.72E-07
TUCP_002189	XLOC_091613	ENSRNOT00000072299	0.960414427	7.16E-07
TUCP_002189	XLOC_091613	ENSRNOT00000003544	-0.96427735	4.31E-07
TUCP_002189	XLOC_091613	ENSRNOT00000032363	0.961941054	5.90E-07
TUCP_002189	XLOC_091613	ENSRNOT00000003780	0.973316537	1.02E-07
TUCP_002189	XLOC_091613	ENSRNOT00000026643	0.965578791	3.59E-07
TUCP_002189	XLOC_091613	ENSRNOT00000001403	0.950319971	2.19E-06
TUCP_002189	XLOC_091613	ENSRNOT00000016445	0.965245821	3.77E-07
TUCP_002189	XLOC_091613	ENSRNOT00000023262	-0.961929406	5.91E-07
TUCP_002189	XLOC_091613	ENSRNOT00000025913	-0.976699495	5.20E-08
TUCP_002189	XLOC_091613	ENSRNOT00000058495	-0.951188147	2.01E-06
TUCP_002189	XLOC_091613	ENSRNOT00000013067	-0.968394867	2.36E-07
TUCP_002189	XLOC_091613	ENSRNOT00000011956	0.96678712	3.01E-07
TUCP_002189	XLOC_091613	ENSRNOT00000004351	-0.952575186	1.74E-06
TUCP_002189	XLOC_091613	ENSRNOT00000072357	-0.951097383	2.03E-06
TUCP_002189	XLOC_091613	ENSRNOT00000024119	0.958708582	8.82E-07
TUCP_002189	XLOC_091613	ENSRNOT00000045946	0.96351033	4.79E-07
TUCP_002189	XLOC_091613	ENSRNOT00000086577	0.966411382	3.18E-07
TUCP_002189	XLOC_091613	ENSRNOT00000036658	-0.963330342	4.91E-07
TUCP_002189	XLOC_091613	ENSRNOT00000089611	0.958548961	8.99E-07
TUCP_002189	XLOC_091613	ENSRNOT00000026235	-0.963727116	4.65E-07
TUCP_002189	XLOC_091613	ENSRNOT00000017254	-0.952430608	1.77E-06
TUCP_002189	XLOC_091613	ENSRNOT00000079609	0.959259757	8.25E-07
TUCP_002189	XLOC_091613	ENSRNOT00000007738	-0.953272671	1.62E-06
TUCP_002189	XLOC_091613	ENSRNOT00000032667	-0.950971065	2.05E-06
TUCP_002189	XLOC_091613	ENSRNOT00000030007	-0.951053182	2.04E-06
TUCP_002189	XLOC_091613	ENSRNOT00000065600	0.978742985	3.30E-08
TUCP_002189	XLOC_091613	ENSRNOT00000022194	-0.956220628	1.18E-06
TUCP_002189	XLOC_091613	ENSRNOT00000072118	0.96426908	4.32E-07

TUCP_002189	XLOC_091613	ENSRNOT00000018328	-0.959602169	7.92E-07
TUCP_002189	XLOC_091613	ENSRNOT00000033975	0.957764128	9.86E-07
TUCP_002189	XLOC_091613	ENSRNOT00000068026	-0.965486353	3.64E-07
TUCP_002189	XLOC_091613	ENSRNOT00000085621	0.960002552	7.54E-07
TUCP_002189	XLOC_091613	ENSRNOT00000007314	-0.952025564	1.85E-06
TUCP_002189	XLOC_091613	ENSRNOT00000009811	0.953326284	1.61E-06
TUCP_002189	XLOC_091613	ENSRNOT00000014821	-0.963689259	4.68E-07
TUCP_002189	XLOC_091613	ENSRNOT00000034883	-0.963479426	4.81E-07
TUCP_002189	XLOC_091613	ENSRNOT00000015318	-0.960154955	7.40E-07
TUCP_002189	XLOC_091613	ENSRNOT00000005459	0.960763196	6.86E-07
TUCP_002189	XLOC_091613	ENSRNOT00000088599	0.950750738	2.10E-06
TUCP_002189	XLOC_091613	ENSRNOT00000022190	-0.972308164	1.22E-07
TUCP_002189	XLOC_091613	ENSRNOT00000026531	-0.966879544	2.97E-07
TUCP_002189	XLOC_091613	ENSRNOT00000035605	0.958275539	9.28E-07
TUCP_002189	XLOC_091613	ENSRNOT00000081351	0.950457837	2.16E-06
TUCP_002189	XLOC_091613	ENSRNOT00000090853	-0.965330083	3.72E-07
TUCP_002189	XLOC_091613	ENSRNOT00000084031	0.967263283	2.80E-07
TUCP_002189	XLOC_091613	ENSRNOT00000092083	0.953472836	1.59E-06
TUCP_002189	XLOC_091613	ENSRNOT00000086456	-0.953167193	1.64E-06
TUCP_002189	XLOC_091613	ENSRNOT00000005910	-0.97330906	1.02E-07
TUCP_002189	XLOC_091613	ENSRNOT00000082914	0.963604196	4.73E-07
TUCP_002189	XLOC_091613	ENSRNOT00000017995	0.950192439	2.22E-06
TUCP_002189	XLOC_091613	ENSRNOT00000036838	-0.959616156	7.90E-07
TUCP_002189	XLOC_091613	ENSRNOT00000015476	-0.956358452	1.16E-06
TUCP_002189	XLOC_091613	ENSRNOT00000071897	-0.956062189	1.20E-06
TUCP_002189	XLOC_091613	ENSRNOT00000075209	-0.956487383	1.14E-06
TUCP_002189	XLOC_091613	ENSRNOT00000006765	-0.951311101	1.99E-06
TUCP_002189	XLOC_091613	ENSRNOT00000013025	-0.966378592	3.20E-07
TUCP_002189	XLOC_091613	ENSRNOT00000029701	0.972216515	1.24E-07
TUCP_002189	XLOC_091613	ENSRNOT00000020383	0.959224958	8.29E-07
TUCP_002189	XLOC_091613	ENSRNOT00000026988	-0.953600552	1.57E-06
TUCP_002189	XLOC_091613	ENSRNOT00000030706	-0.954198561	1.47E-06
TUCP_002189	XLOC_091613	ENSRNOT00000065615	0.962579894	5.43E-07
TUCP_002189	XLOC_091613	ENSRNOT00000011675	0.974828638	7.63E-08
TUCP_002189	XLOC_091613	ENSRNOT00000067135	-0.957443908	1.02E-06
TUCP_002189	XLOC_091613	ENSRNOT00000004290	0.958400726	9.15E-07
TUCP_002189	XLOC_091613	ENSRNOT00000020951	0.951322648	1.98E-06
TUCP_002189	XLOC_091613	ENSRNOT00000076230	0.95256029	1.75E-06
TUCP_002189	XLOC_091613	ENSRNOT00000037015	0.980831714	1.97E-08
TUCP_002189	XLOC_091613	ENSRNOT00000023876	0.967943818	2.53E-07
TUCP_002189	XLOC_091613	ENSRNOT00000002443	0.95962121	7.90E-07
TUCP_002189	XLOC_091613	ENSRNOT00000085334	0.95601339	1.20E-06
TUCP_002189	XLOC_091613	ENSRNOT00000066952	0.966070844	3.35E-07
TUCP_002189	XLOC_091613	ENSRNOT00000028685	0.963185127	5.01E-07
TUCP_002189	XLOC_091613	ENSRNOT00000080591	0.964398816	4.24E-07
TUCP_002189	XLOC_091613	ENSRNOT00000083632	-0.968132069	2.45E-07
TUCP_002189	XLOC_091613	ENSRNOT00000074150	0.953155607	1.64E-06
TUCP_002189	XLOC_091613	ENSRNOT00000015239	-0.962908839	5.19E-07
TUCP_002189	XLOC_091613	ENSRNOT00000057953	0.960229901	7.33E-07
TUCP_002189	XLOC_091613	ENSRNOT00000003568	0.972166162	1.26E-07
TUCP_002189	XLOC_091613	ENSRNOT00000074649	0.951799545	1.89E-06

TUCP_002189	XLOC_091613	ENSRNOT00000005257	0.972387581	1.21E-07
TUCP_002189	XLOC_091613	ENSRNOT00000016132	-0.960678916	6.93E-07
TUCP_002189	XLOC_091613	ENSRNOT00000049725	-0.952409071	1.77E-06
TUCP_002189	XLOC_091613	ENSRNOT00000077146	-0.97419527	8.63E-08
TUCP_002189	XLOC_091613	ENSRNOT00000086122	0.952113241	1.83E-06
TUCP_002189	XLOC_091613	ENSRNOT00000013851	-0.962294739	5.63E-07
TUCP_002189	XLOC_091613	ENSRNOT00000017765	0.95630029	1.17E-06
TUCP_002189	XLOC_091613	ENSRNOT00000042638	0.969761873	1.89E-07
TUCP_002189	XLOC_091613	ENSRNOT00000004999	0.962627866	5.39E-07
TUCP_002189	XLOC_091613	ENSRNOT00000009122	0.950582275	2.14E-06
TUCP_002189	XLOC_091613	ENSRNOT00000013463	0.97801982	3.89E-08
TUCP_002189	XLOC_091613	ENSRNOT00000060002	-0.955680713	1.25E-06
TUCP_002189	XLOC_091613	ENSRNOT00000089673	-0.97461506	7.96E-08
TUCP_002189	XLOC_091613	ENSRNOT00000027301	-0.950060755	2.25E-06
TUCP_002189	XLOC_091613	ENSRNOT00000010367	0.974139587	8.72E-08
TUCP_002189	XLOC_091613	ENSRNOT00000057738	0.980675838	2.05E-08
TUCP_002189	XLOC_091613	ENSRNOT00000058382	0.965240269	3.77E-07
TUCP_002189	XLOC_091613	ENSRNOT00000006802	0.956894266	1.09E-06
TUCP_002189	XLOC_091613	ENSRNOT00000025966	0.958374814	9.18E-07
TUCP_002189	XLOC_091613	ENSRNOT00000023977	-0.963640498	4.71E-07
TUCP_002189	XLOC_091613	ENSRNOT00000009202	0.96575603	3.50E-07
TUCP_002189	XLOC_091613	ENSRNOT00000047663	-0.961667975	6.11E-07
TUCP_002189	XLOC_091613	ENSRNOT00000082151	-0.967549963	2.68E-07
TUCP_002189	XLOC_091613	ENSRNOT00000022578	0.968306012	2.39E-07
TUCP_002189	XLOC_091613	ENSRNOT00000066133	-0.958308247	9.25E-07
TUCP_002189	XLOC_091613	ENSRNOT00000055602	-0.958058334	9.53E-07
TUCP_002189	XLOC_091613	ENSRNOT00000059181	0.953316273	1.61E-06
TUCP_002189	XLOC_091613	ENSRNOT00000029202	-0.959142122	8.37E-07
TUCP_002189	XLOC_091613	ENSRNOT00000017291	-0.966586644	3.10E-07
TUCP_002189	XLOC_091613	ENSRNOT00000017907	-0.959141408	8.37E-07
TUCP_002189	XLOC_091613	ENSRNOT00000003691	-0.971990036	1.30E-07
TUCP_002189	XLOC_091613	ENSRNOT00000010852	0.967940422	2.53E-07
TUCP_002189	XLOC_091613	ENSRNOT00000013524	0.960675077	6.93E-07
TUCP_002189	XLOC_091613	ENSRNOT00000035385	0.95378941	1.54E-06
TUCP_002189	XLOC_091613	ENSRNOT00000025794	-0.973551271	9.75E-08
TUCP_002189	XLOC_091613	ENSRNOT00000038807	0.957592719	1.01E-06
TUCP_002189	XLOC_091613	ENSRNOT00000058111	-0.950997303	2.05E-06
TUCP_002189	XLOC_091613	ENSRNOT00000014926	0.95009686	2.24E-06
TUCP_002189	XLOC_091613	ENSRNOT00000014924	-0.954633719	1.40E-06
TUCP_002189	XLOC_091613	ENSRNOT00000017761	0.961169639	6.51E-07
TUCP_002189	XLOC_091613	ENSRNOT00000008987	-0.973797851	9.31E-08
TUCP_002189	XLOC_091613	ENSRNOT00000009994	-0.951180804	2.01E-06
TUCP_002189	XLOC_091613	ENSRNOT00000030692	-0.963390867	4.87E-07
TUCP_002189	XLOC_091613	ENSRNOT00000057537	0.958071753	9.51E-07
TUCP_002189	XLOC_091613	ENSRNOT00000022716	0.95709853	1.06E-06
TUCP_002189	XLOC_091613	ENSRNOT00000002114	-0.977485057	4.39E-08
TUCP_002189	XLOC_091613	ENSRNOT00000002445	-0.960656045	6.95E-07
TUCP_002189	XLOC_091613	ENSRNOT00000080608	0.951123649	2.02E-06
TUCP_002189	XLOC_091613	ENSRNOT00000027970	0.963095589	5.07E-07
TUCP_002189	XLOC_091613	ENSRNOT00000077479	-0.951578808	1.93E-06
TUCP_002189	XLOC_091613	ENSRNOT00000085971	-0.963440429	4.84E-07

TUCP_002189	XLOC_091613	ENSRNOT00000085295	-0.958250761	9.31E-07
TUCP_002189	XLOC_091613	ENSRNOT00000021578	0.950980235	2.05E-06
TUCP_002189	XLOC_091613	ENSRNOT00000078306	0.95158747	1.93E-06
TUCP_002189	XLOC_091613	ENSRNOT00000027875	0.950039583	2.25E-06
TUCP_002189	XLOC_091613	ENSRNOT00000046215	-0.969259032	2.05E-07
TUCP_002189	XLOC_091613	ENSRNOT00000055386	0.950950621	2.06E-06
TUCP_002189	XLOC_091613	ENSRNOT00000082138	-0.953975325	1.51E-06
TUCP_002189	XLOC_091613	ENSRNOT00000038212	-0.953276662	1.62E-06
TUCP_002189	XLOC_091613	ENSRNOT00000078856	-0.961237259	6.46E-07
TUCP_002189	XLOC_091613	ENSRNOT00000014127	0.950854109	2.08E-06
TUCP_002189	XLOC_091613	ENSRNOT00000008244	0.954339678	1.45E-06
TUCP_002189	XLOC_091613	ENSRNOT00000037409	0.961496705	6.25E-07
TUCP_002189	XLOC_091613	ENSRNOT00000009742	-0.969272501	2.05E-07
TUCP_002189	XLOC_091613	ENSRNOT00000000120	0.980069439	2.40E-08
TUCP_002189	XLOC_091613	ENSRNOT00000088073	-0.960581031	7.02E-07
TUCP_002189	XLOC_091613	ENSRNOT00000013632	0.955529387	1.27E-06
TUCP_002189	XLOC_091613	ENSRNOT00000007369	0.981005343	1.89E-08
TUCP_002189	XLOC_091613	ENSRNOT00000080677	-0.964546754	4.16E-07
TUCP_002189	XLOC_091613	ENSRNOT00000085466	0.960994115	6.66E-07
TUCP_002189	XLOC_091613	ENSRNOT00000028545	-0.951296205	1.99E-06
TUCP_002189	XLOC_091613	ENSRNOT00000012528	0.95678858	1.10E-06
TUCP_002189	XLOC_091613	ENSRNOT00000060698	-0.958348346	9.20E-07
TUCP_002189	XLOC_091613	ENSRNOT00000028225	0.957239722	1.05E-06
TUCP_002189	XLOC_091613	ENSRNOT00000026760	-0.964034461	4.46E-07
TUCP_002189	XLOC_091613	ENSRNOT00000075800	-0.953781188	1.54E-06
TUCP_002189	XLOC_091613	ENSRNOT00000083891	-0.960949	6.70E-07
TUCP_002189	XLOC_091613	ENSRNOT00000015624	0.964734536	4.05E-07
TUCP_002189	XLOC_091613	ENSRNOT00000026941	0.958428636	9.12E-07
TUCP_002189	XLOC_091613	ENSRNOT00000032244	0.961341877	6.37E-07
TUCP_002189	XLOC_091613	ENSRNOT00000017998	0.950056148	2.25E-06
TUCP_002189	XLOC_091613	ENSRNOT00000016568	0.971952363	1.30E-07
TUCP_002189	XLOC_091613	ENSRNOT00000032248	0.955174516	1.32E-06
TUCP_002189	XLOC_091613	ENSRNOT00000027360	-0.955551157	1.27E-06
TUCP_002189	XLOC_091613	ENSRNOT00000024947	-0.95989641	7.64E-07
TUCP_002189	XLOC_091613	ENSRNOT00000013745	-0.956161273	1.18E-06
TUCP_002189	XLOC_091613	ENSRNOT00000065529	0.956682822	1.12E-06
TUCP_002189	XLOC_091613	ENSRNOT00000032236	-0.953353932	1.61E-06
TUCP_002189	XLOC_091613	ENSRNOT00000016372	-0.955912777	1.22E-06
TUCP_002189	XLOC_091613	ENSRNOT00000088679	-0.96556768	3.60E-07
TUCP_002189	XLOC_091613	ENSRNOT00000051494	0.957130091	1.06E-06
TUCP_002189	XLOC_091613	ENSRNOT00000028907	0.957806926	9.81E-07
TUCP_002189	XLOC_091613	ENSRNOT00000027967	0.975684553	6.43E-08
TUCP_002189	XLOC_091613	ENSRNOT00000049285	-0.968541602	2.30E-07
TUCP_002189	XLOC_091613	ENSRNOT00000016060	0.961785685	6.02E-07
TUCP_002189	XLOC_091613	ENSRNOT00000016637	-0.950749353	2.10E-06
TUCP_002189	XLOC_091613	ENSRNOT00000090057	0.964097893	4.42E-07
TUCP_002189	XLOC_091613	ENSRNOT00000081678	0.968388064	2.36E-07
TUCP_002189	XLOC_091613	ENSRNOT00000036613	-0.966383458	3.20E-07
TUCP_002189	XLOC_091613	ENSRNOT00000020929	0.960675692	6.93E-07
TUCP_002189	XLOC_091613	ENSRNOT00000024011	0.952937868	1.68E-06
TUCP_002189	XLOC_091613	ENSRNOT00000025705	0.961257023	6.44E-07

TUCP_002189	XLOC_091613	ENSRNOT00000054685	0.970808612	1.59E-07
TUCP_002189	XLOC_091613	ENSRNOT00000052051	0.958929369	8.59E-07
TUCP_002189	XLOC_091613	ENSRNOT00000092876	-0.962021892	5.84E-07
TUCP_002189	XLOC_091613	ENSRNOT00000005311	0.956963861	1.08E-06
TUCP_002189	XLOC_091613	ENSRNOT00000031935	-0.950254523	2.21E-06
TUCP_002189	XLOC_091613	ENSRNOT00000046639	0.959584762	7.93E-07
TUCP_002189	XLOC_091613	ENSRNOT00000015799	0.950899642	2.07E-06
TUCP_002189	XLOC_091613	ENSRNOT00000005885	-0.954999339	1.35E-06
TUCP_002189	XLOC_091613	ENSRNOT00000008113	-0.954730746	1.39E-06
TUCP_002189	XLOC_091613	ENSRNOT00000013864	-0.963099139	5.06E-07
TUCP_000676	XLOC_025446	ENSRNOT00000001672	0.961926238	5.91E-07
TUCP_000676	XLOC_025446	ENSRNOT00000042853	0.979013805	3.10E-08
TUCP_000576	XLOC_020464	ENSRNOT00000081570	0.963013074	5.12E-07
TUCP_000576	XLOC_020464	ENSRNOT00000091652	0.998205555	1.46E-13
TUCP_000576	XLOC_020464	ENSRNOT00000036564	0.972235638	1.24E-07
TUCP_000576	XLOC_020464	ENSRNOT00000000109	0.96209449	5.78E-07
TUCP_000576	XLOC_020464	ENSRNOT00000080865	0.999995568	1.35E-26
TUCP_000576	XLOC_020464	ENSRNOT00000073534	0.995799759	1.02E-11
TUCP_000576	XLOC_020464	ENSRNOT00000012161	0.987810849	2.08E-09
TUCP_000576	XLOC_020464	ENSRNOT00000078707	0.960848603	6.78E-07
TUCP_000576	XLOC_020464	ENSRNOT00000087758	0.968738278	2.23E-07
TUCP_000576	XLOC_020464	ENSRNOT00000049832	0.969006857	2.14E-07
TUCP_000576	XLOC_020464	ENSRNOT00000093019	0.999995568	1.35E-26
TUCP_000576	XLOC_020464	ENSRNOT00000024825	0.980366661	2.22E-08
TUCP_000576	XLOC_020464	ENSRNOT00000059266	0.960812081	6.81E-07
TUCP_000576	XLOC_020464	ENSRNOT00000072734	0.964876664	3.97E-07
TUCP_000576	XLOC_020464	ENSRNOT00000085908	0.996737201	2.90E-12
TUCP_000576	XLOC_020464	ENSRNOT00000046744	0.999995568	1.35E-26
TUCP_000576	XLOC_020464	ENSRNOT00000076429	0.999995568	1.35E-26
TUCP_000576	XLOC_020464	ENSRNOT00000084667	0.999692292	2.17E-17
TUCP_000576	XLOC_020464	ENSRNOT00000055412	0.999995568	1.35E-26
TUCP_000576	XLOC_020464	ENSRNOT00000093201	0.999684931	2.44E-17
TUCP_000576	XLOC_020464	ENSRNOT00000072724	0.999995568	1.35E-26
TUCP_000576	XLOC_020464	ENSRNOT00000090323	0.95590434	1.22E-06
TUCP_000576	XLOC_020464	ENSRNOT00000082760	0.986240029	3.80E-09
TUCP_000576	XLOC_020464	ENSRNOT00000066017	0.98980576	8.52E-10
TUCP_000576	XLOC_020464	ENSRNOT00000073148	0.985604389	4.75E-09
TUCP_000576	XLOC_020464	ENSRNOT00000077462	0.96001067	7.53E-07
TUCP_000576	XLOC_020464	ENSRNOT00000084375	0.999995568	1.35E-26
TUCP_000576	XLOC_020464	ENSRNOT00000085447	0.998391504	8.46E-14
TUCP_000576	XLOC_020464	ENSRNOT00000006249	0.966395319	3.19E-07
TUCP_000576	XLOC_020464	ENSRNOT00000087073	0.952311764	1.79E-06
TUCP_000576	XLOC_020464	ENSRNOT00000042369	0.995720519	1.12E-11
TUCP_000576	XLOC_020464	ENSRNOT00000086554	0.999995568	1.35E-26
TUCP_000576	XLOC_020464	ENSRNOT00000054858	0.985173477	5.50E-09
TUCP_000576	XLOC_020464	ENSRNOT00000047921	0.999995568	1.35E-26
TUCP_000576	XLOC_020464	ENSRNOT00000064261	0.999974745	8.09E-23
TUCP_000576	XLOC_020464	ENSRNOT00000086660	0.999205397	2.49E-15
TUCP_000576	XLOC_020464	ENSRNOT00000080933	0.967530613	2.69E-07
TUCP_000576	XLOC_020464	ENSRNOT00000083977	0.999995568	1.35E-26
TUCP_000576	XLOC_020464	ENSRNOT00000092933	0.999995568	1.35E-26

TUCP_000576	XLOC_020464	ENSRNOT00000009061	0.953243995	1.63E-06
TUCP_000576	XLOC_020464	ENSRNOT00000041281	0.987750448	2.13E-09
TUCP_003516	XLOC_152769	ENSRNOT00000024186	0.960230863	7.33E-07
TUCP_003516	XLOC_152769	ENSRNOT00000074753	0.977658447	4.22E-08
TUCP_003516	XLOC_152769	ENSRNOT00000041904	0.975611398	6.52E-08
TUCP_003516	XLOC_152769	ENSRNOT00000083487	0.962161869	5.73E-07
TUCP_003516	XLOC_152769	ENSRNOT00000077990	0.975611398	6.52E-08
TUCP_003516	XLOC_152769	ENSRNOT00000038826	0.974716481	7.80E-08
TUCP_003516	XLOC_152769	ENSRNOT00000037094	0.964246605	4.33E-07
TUCP_003516	XLOC_152769	ENSRNOT00000037169	0.966062959	3.35E-07
TUCP_003516	XLOC_152769	ENSRNOT00000021243	0.9702277	1.75E-07
TUCP_003516	XLOC_152769	ENSRNOT00000060250	0.976853767	5.03E-08
TUCP_003516	XLOC_152769	ENSRNOT00000091934	0.975611398	6.52E-08
TUCP_003516	XLOC_152769	ENSRNOT00000086027	0.981984322	1.45E-08
TUCP_003516	XLOC_152769	ENSRNOT00000046585	0.97975719	2.59E-08
TUCP_003516	XLOC_152769	ENSRNOT00000080794	0.967130151	2.86E-07
TUCP_003516	XLOC_152769	ENSRNOT00000014456	0.960594983	7.00E-07
TUCP_003516	XLOC_152769	ENSRNOT00000031205	0.974648781	7.90E-08
TUCP_003516	XLOC_152769	ENSRNOT00000044145	0.975611398	6.52E-08
TUCP_003516	XLOC_152769	ENSRNOT00000088381	0.981244506	1.77E-08
TUCP_003516	XLOC_152769	ENSRNOT00000054947	0.950791572	2.09E-06
TUCP_003516	XLOC_152769	ENSRNOT00000028588	0.950678205	2.12E-06
TUCP_003516	XLOC_152769	ENSRNOT00000011743	0.95965152	7.87E-07
TUCP_003516	XLOC_152769	ENSRNOT00000089929	0.975611398	6.52E-08
TUCP_003516	XLOC_152769	ENSRNOT00000088624	0.975611398	6.52E-08
TUCP_003516	XLOC_152769	ENSRNOT00000085659	0.9564292	1.15E-06
TUCP_003516	XLOC_152769	ENSRNOT00000015866	0.978470631	3.51E-08
TUCP_003516	XLOC_152769	ENSRNOT00000078633	0.975611398	6.52E-08
TUCP_003516	XLOC_152769	ENSRNOT00000024575	0.975287131	6.96E-08
TUCP_003516	XLOC_152769	ENSRNOT00000034907	0.973880336	9.16E-08
TUCP_003516	XLOC_152769	ENSRNOT00000093230	0.951478514	1.95E-06
TUCP_003516	XLOC_152769	ENSRNOT00000051835	0.975611398	6.52E-08
TUCP_003516	XLOC_152769	ENSRNOT00000079087	0.975611398	6.52E-08
TUCP_003516	XLOC_152769	ENSRNOT00000037206	0.975611398	6.52E-08
TUCP_003516	XLOC_152769	ENSRNOT00000083261	0.959656642	7.86E-07
TUCP_003516	XLOC_152769	ENSRNOT00000000697	0.954930705	1.36E-06
TUCP_003516	XLOC_152769	ENSRNOT00000080590	0.974151639	8.70E-08
TUCP_003516	XLOC_152769	ENSRNOT00000027552	0.974569135	8.03E-08
TUCP_003516	XLOC_152769	ENSRNOT00000077014	0.986964683	2.90E-09
TUCP_003516	XLOC_152769	ENSRNOT00000080453	0.975001506	7.37E-08
TUCP_003516	XLOC_152769	ENSRNOT00000057729	0.975641588	6.48E-08
TUCP_003516	XLOC_152769	ENSRNOT00000061284	0.957653745	9.99E-07
TUCP_003516	XLOC_152769	ENSRNOT00000020206	0.964673886	4.08E-07
TUCP_003516	XLOC_152769	ENSRNOT00000013423	0.960061914	7.48E-07
TUCP_003516	XLOC_152769	ENSRNOT00000066839	0.963437953	4.84E-07
TUCP_003516	XLOC_152769	ENSRNOT00000078663	0.985206531	5.44E-09
TUCP_003516	XLOC_152769	ENSRNOT00000064250	0.968374383	2.36E-07
TUCP_003516	XLOC_152769	ENSRNOT00000021608	0.968343736	2.37E-07
TUCP_003516	XLOC_152769	ENSRNOT00000029634	0.972710929	1.14E-07
TUCP_003516	XLOC_152769	ENSRNOT00000093579	0.951568229	1.93E-06
TUCP_003516	XLOC_152769	ENSRNOT00000065460	0.975611398	6.52E-08

TUCP_003516	XLOC_152769	ENSRNOT00000003817	0.978998721	3.11E-08
TUCP_003516	XLOC_152769	ENSRNOT00000016713	0.975611398	6.52E-08
TUCP_003516	XLOC_152769	ENSRNOT00000079260	0.972299755	1.23E-07
TUCP_003516	XLOC_152769	ENSRNOT00000039294	0.975611398	6.52E-08
TUCP_002185	XLOC_091237	ENSRNOT00000065600	0.958287245	9.27E-07
TUCP_002184	XLOC_091236	ENSRNOT00000010332	0.956114516	1.19E-06
TUCP_002184	XLOC_091236	ENSRNOT00000086575	0.951238789	2.00E-06
TUCP_002184	XLOC_091236	ENSRNOT00000067663	0.956832702	1.10E-06
TUCP_002184	XLOC_091236	ENSRNOT00000041044	0.960906447	6.73E-07
TUCP_002184	XLOC_091236	ENSRNOT00000044174	0.95000299	2.26E-06
TUCP_002184	XLOC_091236	ENSRNOT00000065600	0.968207921	2.42E-07
TUCP_002184	XLOC_091236	ENSRNOT00000067400	0.950772682	2.10E-06
TUCP_002184	XLOC_091236	ENSRNOT00000066024	0.9505463	2.14E-06
TUCP_002184	XLOC_091236	ENSRNOT00000001826	0.951313451	1.98E-06
TUCP_002184	XLOC_091236	ENSRNOT00000038807	0.951222143	2.00E-06
TUCP_002184	XLOC_091236	ENSRNOT00000006067	0.953006813	1.67E-06
TUCP_002184	XLOC_091236	ENSRNOT00000075558	0.950376186	2.18E-06
TUCP_001641	XLOC_064931	ENSRNOT00000024366	0.955686648	1.25E-06
TUCP_001641	XLOC_064931	ENSRNOT00000010396	0.950137584	2.23E-06
TUCP_001641	XLOC_064931	ENSRNOT00000010332	0.966035667	3.36E-07
TUCP_001641	XLOC_064931	ENSRNOT00000065600	0.950622298	2.13E-06
TUCP_001641	XLOC_064931	ENSRNOT00000041044	0.958256943	9.30E-07
TUCP_001641	XLOC_064931	ENSRNOT00000010367	0.955141804	1.33E-06
TUCP_001641	XLOC_064931	ENSRNOT00000026641	0.967679808	2.63E-07
TUCP_001641	XLOC_064931	ENSRNOT00000022714	0.965735016	3.51E-07
TUCP_002343	XLOC_097704	ENSRNOT00000025981	0.955132874	1.33E-06
TUCP_002343	XLOC_097704	ENSRNOT00000008510	0.957497409	1.02E-06
TUCP_002343	XLOC_097704	ENSRNOT00000031904	0.956168982	1.18E-06
TUCP_002343	XLOC_097704	ENSRNOT00000038807	0.953560769	1.57E-06
TUCP_002343	XLOC_097704	ENSRNOT00000057924	0.962751052	5.30E-07
TUCP_002343	XLOC_097704	ENSRNOT00000066024	0.970753963	1.60E-07
TUCP_002343	XLOC_097704	ENSRNOT00000004570	0.950125575	2.23E-06
TUCP_002343	XLOC_097704	ENSRNOT00000005664	0.96289135	5.21E-07
TUCP_002343	XLOC_097704	ENSRNOT00000011429	0.971065102	1.52E-07
TUCP_002343	XLOC_097704	ENSRNOT00000040513	0.957021966	1.07E-06
TUCP_002343	XLOC_097704	ENSRNOT00000078306	0.961093458	6.58E-07
TUCP_002343	XLOC_097704	ENSRNOT00000080695	0.951904178	1.87E-06
TUCP_002343	XLOC_097704	ENSRNOT00000041044	0.974346451	8.38E-08
TUCP_002343	XLOC_097704	ENSRNOT00000036054	0.956192893	1.18E-06
TUCP_002343	XLOC_097704	ENSRNOT00000054674	0.950951917	2.06E-06
TUCP_002343	XLOC_097704	ENSRNOT00000075279	0.960847559	6.78E-07
TUCP_002343	XLOC_097704	ENSRNOT00000067400	0.958864817	8.66E-07
TUCP_002343	XLOC_097704	ENSRNOT00000020673	0.958111332	9.47E-07
TUCP_002343	XLOC_097704	ENSRNOT00000026261	0.955444283	1.28E-06
TUCP_002343	XLOC_097704	ENSRNOT00000016209	0.958662769	8.87E-07
TUCP_002343	XLOC_097704	ENSRNOT00000019923	0.950929097	2.06E-06
TUCP_002343	XLOC_097704	ENSRNOT00000025092	0.950749846	2.10E-06
TUCP_002343	XLOC_097704	ENSRNOT00000057160	0.97051413	1.67E-07
TUCP_002343	XLOC_097704	ENSRNOT00000016739	0.96314311	5.03E-07
TUCP_002343	XLOC_097704	ENSRNOT00000079595	0.962521527	5.47E-07
TUCP_002343	XLOC_097704	ENSRNOT00000036072	0.966039187	3.36E-07

TUCP_002343	XLOC_097704	ENSRNOT00000003641	0.951119059	2.02E-06
TUCP_003458	XLOC_149596	ENSRNOT00000081570	0.962961027	5.16E-07
TUCP_003458	XLOC_149596	ENSRNOT00000091652	0.99819185	1.52E-13
TUCP_003458	XLOC_149596	ENSRNOT00000036564	0.972337506	1.22E-07
TUCP_003458	XLOC_149596	ENSRNOT00000000109	0.962179854	5.72E-07
TUCP_003458	XLOC_149596	ENSRNOT00000080865	1	4.25E-78
TUCP_003458	XLOC_149596	ENSRNOT00000073534	0.995808019	1.01E-11
TUCP_003458	XLOC_149596	ENSRNOT00000012161	0.987544327	2.31E-09
TUCP_003458	XLOC_149596	ENSRNOT00000078707	0.960060506	7.48E-07
TUCP_003458	XLOC_149596	ENSRNOT00000087758	0.968778527	2.22E-07
TUCP_003458	XLOC_149596	ENSRNOT00000049832	0.969039151	2.13E-07
TUCP_003458	XLOC_149596	ENSRNOT00000093019	1	0
TUCP_003458	XLOC_149596	ENSRNOT00000024825	0.980452701	2.18E-08
TUCP_003458	XLOC_149596	ENSRNOT00000059266	0.96033506	7.23E-07
TUCP_003458	XLOC_149596	ENSRNOT00000072734	0.965018115	3.89E-07
TUCP_003458	XLOC_149596	ENSRNOT00000085908	0.996653227	3.29E-12
TUCP_003458	XLOC_149596	ENSRNOT00000046744	1	1.33E-79
TUCP_003458	XLOC_149596	ENSRNOT00000076429	1	4.25E-78
TUCP_003458	XLOC_149596	ENSRNOT00000084667	0.999660971	3.53E-17
TUCP_003458	XLOC_149596	ENSRNOT00000055412	1	1.33E-79
TUCP_003458	XLOC_149596	ENSRNOT00000093201	0.9997022	1.84E-17
TUCP_003458	XLOC_149596	ENSRNOT00000072724	1	4.25E-78
TUCP_003458	XLOC_149596	ENSRNOT00000090323	0.95603782	1.20E-06
TUCP_003458	XLOC_149596	ENSRNOT00000082760	0.986293522	3.72E-09
TUCP_003458	XLOC_149596	ENSRNOT00000066017	0.989785653	8.61E-10
TUCP_003458	XLOC_149596	ENSRNOT00000073148	0.985721397	4.56E-09
TUCP_003458	XLOC_149596	ENSRNOT00000077462	0.960098183	7.45E-07
TUCP_003458	XLOC_149596	ENSRNOT00000084375	1	4.25E-78
TUCP_003458	XLOC_149596	ENSRNOT00000085447	0.998400682	8.22E-14
TUCP_003458	XLOC_149596	ENSRNOT00000006249	0.966489607	3.15E-07
TUCP_003458	XLOC_149596	ENSRNOT00000087073	0.952450698	1.77E-06
TUCP_003458	XLOC_149596	ENSRNOT00000042369	0.995659369	1.20E-11
TUCP_003458	XLOC_149596	ENSRNOT00000086554	1	1.33E-79
TUCP_003458	XLOC_149596	ENSRNOT00000054858	0.985228824	5.40E-09
TUCP_003458	XLOC_149596	ENSRNOT00000047921	1	4.25E-78
TUCP_003458	XLOC_149596	ENSRNOT00000064261	0.999977196	4.86E-23
TUCP_003458	XLOC_149596	ENSRNOT00000086660	0.999208903	2.44E-15
TUCP_003458	XLOC_149596	ENSRNOT00000080933	0.967360681	2.76E-07
TUCP_003458	XLOC_149596	ENSRNOT00000083977	1	0
TUCP_003458	XLOC_149596	ENSRNOT00000092933	1	0
TUCP_003458	XLOC_149596	ENSRNOT00000009061	0.953300672	1.62E-06
TUCP_003458	XLOC_149596	ENSRNOT00000041281	0.987828355	2.06E-09
TUCP_002180	XLOC_091086	ENSRNOT00000030074	-0.961364296	6.35E-07
TUCP_002180	XLOC_091086	ENSRNOT00000032802	0.965995625	3.38E-07
TUCP_002180	XLOC_091086	ENSRNOT00000010835	-0.977398838	4.47E-08
TUCP_002180	XLOC_091086	ENSRNOT00000075656	0.95181283	1.89E-06
TUCP_002180	XLOC_091086	ENSRNOT00000075397	-0.967063186	2.89E-07
TUCP_002180	XLOC_091086	ENSRNOT00000007350	-0.960425392	7.15E-07
TUCP_002180	XLOC_091086	ENSRNOT00000004713	0.956292699	1.17E-06
TUCP_002180	XLOC_091086	ENSRNOT00000013116	0.96694763	2.94E-07
TUCP_002180	XLOC_091086	ENSRNOT00000010683	0.951307511	1.99E-06

TUCP_002180	XLOC_091086	ENSRNOT00000013025	0.955089835	1.33E-06
TUCP_002180	XLOC_091086	ENSRNOT00000039551	0.955751241	1.24E-06
TUCP_002180	XLOC_091086	ENSRNOT00000038178	0.959684738	7.84E-07
TUCP_002180	XLOC_091086	ENSRNOT00000007164	-0.954999247	1.35E-06
TUCP_002180	XLOC_091086	ENSRNOT00000049459	0.962793554	5.27E-07
TUCP_002180	XLOC_091086	ENSRNOT00000045326	-0.957361303	1.03E-06
TUCP_002180	XLOC_091086	ENSRNOT00000014309	0.969272469	2.05E-07
TUCP_002180	XLOC_091086	ENSRNOT00000085885	0.969018346	2.13E-07
TUCP_002180	XLOC_091086	ENSRNOT00000017571	0.964334038	4.28E-07
TUCP_002180	XLOC_091086	ENSRNOT00000013851	0.954596877	1.41E-06
TUCP_002180	XLOC_091086	ENSRNOT00000085736	0.952327604	1.79E-06
TUCP_002180	XLOC_091086	ENSRNOT00000001679	-0.9766719	5.23E-08
TUCP_002180	XLOC_091086	ENSRNOT00000055540	-0.96237222	5.58E-07
TUCP_002180	XLOC_091086	ENSRNOT00000018584	-0.963397664	4.87E-07
TUCP_002180	XLOC_091086	ENSRNOT00000078856	0.979165692	2.99E-08
TUCP_002180	XLOC_091086	ENSRNOT00000040847	0.954377572	1.44E-06
TUCP_002180	XLOC_091086	ENSRNOT00000013516	-0.970021213	1.81E-07
TUCP_002180	XLOC_091086	ENSRNOT00000017147	-0.952073577	1.84E-06
TUCP_002180	XLOC_091086	ENSRNOT00000073019	-0.952923401	1.68E-06
TUCP_002180	XLOC_091086	ENSRNOT00000031935	0.951119396	2.02E-06
TUCP_002180	XLOC_091086	ENSRNOT00000064401	-0.962066052	5.80E-07
TUCP_002180	XLOC_091086	ENSRNOT00000017271	-0.950124219	2.23E-06
TUCP_002180	XLOC_091086	ENSRNOT00000030109	-0.965033611	3.88E-07
TUCP_002180	XLOC_091086	ENSRNOT00000009319	0.951618631	1.92E-06
TUCP_002180	XLOC_091086	ENSRNOT00000013191	0.950273252	2.20E-06
TUCP_002180	XLOC_091086	ENSRNOT00000024677	-0.95029726	2.20E-06
TUCP_002180	XLOC_091086	ENSRNOT00000067009	0.971441165	1.43E-07
TUCP_002180	XLOC_091086	ENSRNOT00000089418	-0.960114138	7.43E-07
TUCP_002180	XLOC_091086	ENSRNOT00000091448	0.953275447	1.62E-06
TUCP_002180	XLOC_091086	ENSRNOT00000077856	0.956970114	1.08E-06
TUCP_002180	XLOC_091086	ENSRNOT00000090129	0.96995698	1.83E-07
TUCP_002180	XLOC_091086	ENSRNOT00000089265	0.952552451	1.75E-06
TUCP_002180	XLOC_091086	ENSRNOT00000018488	0.964164333	4.38E-07
TUCP_002180	XLOC_091086	ENSRNOT00000005686	0.950487234	2.16E-06
TUCP_002180	XLOC_091086	ENSRNOT00000056405	-0.955525118	1.27E-06
TUCP_002180	XLOC_091086	ENSRNOT00000012361	0.953052276	1.66E-06
TUCP_002180	XLOC_091086	ENSRNOT00000000886	-0.95677373	1.11E-06
TUCP_002180	XLOC_091086	ENSRNOT00000030007	0.966479727	3.15E-07
TUCP_002180	XLOC_091086	ENSRNOT00000074212	-0.980944479	1.92E-08
TUCP_002180	XLOC_091086	ENSRNOT00000016629	-0.953142029	1.64E-06
TUCP_002180	XLOC_091086	ENSRNOT00000006111	0.954347062	1.45E-06
TUCP_002180	XLOC_091086	ENSRNOT00000087278	0.96144723	6.29E-07
TUCP_002180	XLOC_091086	ENSRNOT00000006835	-0.954315124	1.45E-06
TUCP_002180	XLOC_091086	ENSRNOT00000092945	0.954183639	1.47E-06
TUCP_002180	XLOC_091086	ENSRNOT00000068745	-0.960308127	7.26E-07
TUCP_002180	XLOC_091086	ENSRNOT00000023619	-0.963287019	4.94E-07
TUCP_002180	XLOC_091086	ENSRNOT00000084709	-0.952561861	1.75E-06
TUCP_002180	XLOC_091086	ENSRNOT00000017949	0.958894314	8.63E-07
TUCP_002180	XLOC_091086	ENSRNOT00000038546	0.960059745	7.48E-07
TUCP_002180	XLOC_091086	ENSRNOT00000013864	0.97030952	1.73E-07
TUCP_002180	XLOC_091086	ENSRNOT00000068452	-0.955132626	1.33E-06

TUCP_002180	XLOC_091086	ENSRNOT00000025856	0.95119797	2.01E-06
TUCP_002180	XLOC_091086	ENSRNOT00000021729	-0.968998753	2.14E-07
TUCP_002180	XLOC_091086	ENSRNOT00000022190	0.953946935	1.51E-06
TUCP_002180	XLOC_091086	ENSRNOT00000016132	0.954088362	1.49E-06
TUCP_002180	XLOC_091086	ENSRNOT00000025913	0.970280137	1.74E-07
TUCP_002180	XLOC_091086	ENSRNOT00000012314	0.966964226	2.93E-07
TUCP_002180	XLOC_091086	ENSRNOT00000076230	-0.959234708	8.28E-07
TUCP_002180	XLOC_091086	ENSRNOT00000073919	-0.955808843	1.23E-06
TUCP_002180	XLOC_091086	ENSRNOT00000082863	-0.951354147	1.98E-06
TUCP_002180	XLOC_091086	ENSRNOT00000032667	0.957866889	9.74E-07
TUCP_002180	XLOC_091086	ENSRNOT00000064667	0.975303068	6.94E-08
TUCP_002180	XLOC_091086	ENSRNOT00000038610	0.961362342	6.36E-07
TUCP_002180	XLOC_091086	ENSRNOT00000034883	0.970426318	1.70E-07
TUCP_002180	XLOC_091086	ENSRNOT00000020155	-0.954845198	1.37E-06
TUCP_002180	XLOC_091086	ENSRNOT00000005459	-0.950385391	2.18E-06
TUCP_002180	XLOC_091086	ENSRNOT00000081123	-0.960557341	7.04E-07
TUCP_002180	XLOC_091086	ENSRNOT00000081351	-0.955481595	1.28E-06
TUCP_002180	XLOC_091086	ENSRNOT00000090853	0.966895597	2.96E-07
TUCP_002180	XLOC_091086	ENSRNOT00000081077	0.967140223	2.86E-07
TUCP_002180	XLOC_091086	ENSRNOT00000028066	0.963437508	4.84E-07
TUCP_002180	XLOC_091086	ENSRNOT00000092083	-0.957087173	1.07E-06
TUCP_002180	XLOC_091086	ENSRNOT00000011880	-0.964042661	4.46E-07
TUCP_002180	XLOC_091086	ENSRNOT00000005910	0.969074384	2.12E-07
TUCP_002180	XLOC_091086	ENSRNOT00000055437	0.961316913	6.39E-07
TUCP_002180	XLOC_091086	ENSRNOT00000000898	-0.968756536	2.22E-07
TUCP_002180	XLOC_091086	ENSRNOT00000050350	0.957509844	1.02E-06
TUCP_002180	XLOC_091086	ENSRNOT00000020383	-0.96121118	6.48E-07
TUCP_002180	XLOC_091086	ENSRNOT00000026988	0.960445198	7.14E-07
TUCP_002180	XLOC_091086	ENSRNOT00000078366	-0.955405878	1.29E-06
TUCP_002180	XLOC_091086	ENSRNOT00000025222	0.969654439	1.93E-07
TUCP_002180	XLOC_091086	ENSRNOT00000020951	-0.955538014	1.27E-06
TUCP_002180	XLOC_091086	ENSRNOT00000011675	-0.950166549	2.23E-06
TUCP_002180	XLOC_091086	ENSRNOT00000027917	-0.963974023	4.50E-07
TUCP_002180	XLOC_091086	ENSRNOT00000004290	-0.966206019	3.28E-07
TUCP_002180	XLOC_091086	ENSRNOT00000023876	-0.957443786	1.02E-06
TUCP_002180	XLOC_091086	ENSRNOT00000033359	-0.963622857	4.72E-07
TUCP_002180	XLOC_091086	ENSRNOT00000072031	0.950196449	2.22E-06
TUCP_002180	XLOC_091086	ENSRNOT00000039730	0.964192729	4.37E-07
TUCP_002180	XLOC_091086	ENSRNOT00000049285	0.95664455	1.12E-06
TUCP_002180	XLOC_091086	ENSRNOT00000093385	-0.956535073	1.14E-06
TUCP_002180	XLOC_091086	ENSRNOT00000015239	0.959035942	8.48E-07
TUCP_002180	XLOC_091086	ENSRNOT00000082146	0.959732286	7.79E-07
TUCP_002180	XLOC_091086	ENSRNOT00000077146	0.957524841	1.01E-06
TUCP_002180	XLOC_091086	ENSRNOT00000026235	0.975712833	6.39E-08
TUCP_002180	XLOC_091086	ENSRNOT00000054980	-0.960720388	6.89E-07
TUCP_002180	XLOC_091086	ENSRNOT00000024033	-0.958467143	9.08E-07
TUCP_002180	XLOC_091086	ENSRNOT00000089673	0.953257629	1.62E-06
TUCP_002180	XLOC_091086	ENSRNOT00000030850	-0.955739336	1.24E-06
TUCP_002180	XLOC_091086	ENSRNOT00000075209	0.950563213	2.14E-06
TUCP_002180	XLOC_091086	ENSRNOT00000023977	0.952873454	1.69E-06
TUCP_002180	XLOC_091086	ENSRNOT00000090774	0.961543131	6.21E-07

TUCP_002180	XLOC_091086	ENSRNOT00000009318	-0.951570222	1.93E-06
TUCP_002180	XLOC_091086	ENSRNOT00000003691	0.952431185	1.77E-06
TUCP_002180	XLOC_091086	ENSRNOT00000025794	0.963525065	4.78E-07
TUCP_002180	XLOC_091086	ENSRNOT00000017765	-0.961617834	6.15E-07
TUCP_002180	XLOC_091086	ENSRNOT00000057965	-0.950442274	2.17E-06
TUCP_002180	XLOC_091086	ENSRNOT00000017761	-0.951588236	1.93E-06
TUCP_002180	XLOC_091086	ENSRNOT00000091282	0.985156957	5.53E-09
TUCP_002180	XLOC_091086	ENSRNOT00000030692	0.961314832	6.39E-07
TUCP_002180	XLOC_091086	ENSRNOT00000001923	-0.965834031	3.46E-07
TUCP_002180	XLOC_091086	ENSRNOT00000077479	0.972589413	1.16E-07
TUCP_002180	XLOC_091086	ENSRNOT00000002114	0.961285542	6.42E-07
TUCP_002180	XLOC_091086	ENSRNOT00000021578	-0.965407686	3.68E-07
TUCP_002180	XLOC_091086	ENSRNOT00000040060	0.964388987	4.25E-07
TUCP_002180	XLOC_091086	ENSRNOT00000010580	0.963127882	5.05E-07
TUCP_002180	XLOC_091086	ENSRNOT00000039102	-0.954745957	1.39E-06
TUCP_002180	XLOC_091086	ENSRNOT00000091325	0.953435437	1.59E-06
TUCP_002180	XLOC_091086	ENSRNOT00000037679	-0.950626651	2.13E-06
TUCP_002180	XLOC_091086	ENSRNOT00000024306	0.954662306	1.40E-06
TUCP_002180	XLOC_091086	ENSRNOT00000009742	0.980182112	2.33E-08
TUCP_002180	XLOC_091086	ENSRNOT00000000120	-0.955186762	1.32E-06
TUCP_002180	XLOC_091086	ENSRNOT00000019983	0.966290213	3.24E-07
TUCP_002180	XLOC_091086	ENSRNOT00000013632	-0.973944469	9.05E-08
TUCP_002180	XLOC_091086	ENSRNOT00000080677	0.96624168	3.26E-07
TUCP_002180	XLOC_091086	ENSRNOT00000025460	-0.955984911	1.21E-06
TUCP_002180	XLOC_091086	ENSRNOT00000012996	-0.952141298	1.82E-06
TUCP_002180	XLOC_091086	ENSRNOT00000045946	-0.966020149	3.37E-07
TUCP_002180	XLOC_091086	ENSRNOT00000015643	-0.956318235	1.16E-06
TUCP_002180	XLOC_091086	ENSRNOT00000082570	0.955469879	1.28E-06
TUCP_002180	XLOC_091086	ENSRNOT00000005389	-0.95678774	1.10E-06
TUCP_002180	XLOC_091086	ENSRNOT00000032244	-0.963469201	4.82E-07
TUCP_002180	XLOC_091086	ENSRNOT00000005143	-0.971954323	1.30E-07
TUCP_002180	XLOC_091086	ENSRNOT00000011832	0.959054659	8.46E-07
TUCP_002180	XLOC_091086	ENSRNOT00000015318	0.950887117	2.07E-06
TUCP_002180	XLOC_091086	ENSRNOT00000032236	0.962549642	5.45E-07
TUCP_002180	XLOC_091086	ENSRNOT00000077274	0.961322764	6.39E-07
TUCP_002180	XLOC_091086	ENSRNOT00000028907	-0.962446714	5.52E-07
TUCP_002180	XLOC_091086	ENSRNOT00000047210	-0.950556194	2.14E-06
TUCP_002180	XLOC_091086	ENSRNOT00000036613	0.96552971	3.62E-07
TUCP_002180	XLOC_091086	ENSRNOT00000008987	0.958869401	8.65E-07
TUCP_002180	XLOC_091086	ENSRNOT00000092876	0.966897548	2.96E-07
TUCP_002180	XLOC_091086	ENSRNOT00000077803	-0.966512216	3.14E-07
TUCP_002180	XLOC_091086	ENSRNOT00000005885	0.9565269	1.14E-06
TUCP_003301	XLOC_141907	ENSRNOT00000026966	0.960377192	7.20E-07
TUCP_003301	XLOC_141907	ENSRNOT00000081709	0.950111631	2.24E-06
TUCP_003301	XLOC_141907	ENSRNOT00000020071	0.971469121	1.42E-07
TUCP_003301	XLOC_141907	ENSRNOT00000067379	0.952724316	1.72E-06
TUCP_003301	XLOC_141907	ENSRNOT00000050543	0.951813059	1.89E-06
TUCP_003301	XLOC_141907	ENSRNOT00000057433	0.953496705	1.58E-06
TUCP_003301	XLOC_141907	ENSRNOT00000010092	0.954444852	1.43E-06
TUCP_003301	XLOC_141907	ENSRNOT00000047527	0.969914288	1.85E-07
TUCP_003301	XLOC_141907	ENSRNOT00000017412	0.953755989	1.54E-06

TUCP_003301	XLOC_141907	ENSRNOT00000082289	0.952570144	1.75E-06
TUCP_003301	XLOC_141907	ENSRNOT00000082002	0.973265122	1.03E-07
TUCP_003301	XLOC_141907	ENSRNOT00000082143	0.957773074	9.85E-07
TUCP_003301	XLOC_141907	ENSRNOT00000085570	0.955567727	1.27E-06
TUCP_003301	XLOC_141907	ENSRNOT00000076693	0.966606405	3.09E-07
TUCP_003301	XLOC_141907	ENSRNOT00000076802	0.979911988	2.49E-08
TUCP_003301	XLOC_141907	ENSRNOT00000004373	0.960784838	6.84E-07
TUCP_003301	XLOC_141907	ENSRNOT00000005726	0.953561623	1.57E-06
TUCP_003301	XLOC_141907	ENSRNOT00000041617	0.952467767	1.76E-06
TUCP_003301	XLOC_141907	ENSRNOT00000091763	0.952906827	1.69E-06
TUCP_003301	XLOC_141907	ENSRNOT00000020786	0.955394606	1.29E-06
TUCP_003301	XLOC_141907	ENSRNOT00000084847	0.955044942	1.34E-06
TUCP_003301	XLOC_141907	ENSRNOT00000079275	0.952987475	1.67E-06
TUCP_003301	XLOC_141907	ENSRNOT00000088078	0.980748457	2.02E-08
TUCP_003301	XLOC_141907	ENSRNOT00000076618	0.954498209	1.42E-06
TUCP_003301	XLOC_141907	ENSRNOT00000065448	0.991182517	4.14E-10
TUCP_003301	XLOC_141907	ENSRNOT00000072810	0.955124517	1.33E-06
TUCP_003212	XLOC_138405	ENSRNOT00000092186	0.952813597	1.70E-06
TUCP_003212	XLOC_138405	ENSRNOT00000025491	0.952428342	1.77E-06
TUCP_003212	XLOC_138405	ENSRNOT00000018890	0.95291298	1.68E-06
TUCP_002251	XLOC_093849	ENSRNOT00000026817	0.950031025	2.26E-06
TUCP_002251	XLOC_093849	ENSRNOT00000086729	-0.957567646	1.01E-06
TUCP_003132	XLOC_134102	ENSRNOT00000002673	0.950375293	2.18E-06
TUCP_003132	XLOC_134102	ENSRNOT00000003745	0.957276833	1.04E-06
TUCP_003132	XLOC_134102	ENSRNOT00000031693	0.964725495	4.05E-07
TUCP_003132	XLOC_134102	ENSRNOT00000024418	0.952567276	1.75E-06
TUCP_003132	XLOC_134102	ENSRNOT00000034421	0.972231767	1.24E-07
TUCP_003132	XLOC_134102	ENSRNOT00000011845	0.968156458	2.44E-07
TUCP_003132	XLOC_134102	ENSRNOT00000009965	-0.952194487	1.81E-06
TUCP_003132	XLOC_134102	ENSRNOT00000013732	0.961260166	6.44E-07
TUCP_003132	XLOC_134102	ENSRNOT00000025731	0.953876276	1.52E-06
TUCP_003132	XLOC_134102	ENSRNOT00000025797	-0.956299427	1.17E-06
TUCP_003132	XLOC_134102	ENSRNOT00000084722	0.977072276	4.80E-08
TUCP_003132	XLOC_134102	ENSRNOT00000055148	0.954301248	1.45E-06
TUCP_003132	XLOC_134102	ENSRNOT00000068354	0.955205747	1.32E-06
TUCP_003132	XLOC_134102	ENSRNOT00000093700	0.954634821	1.40E-06
TUCP_001249	XLOC_046149	ENSRNOT00000055536	0.964902693	3.95E-07
TUCP_001249	XLOC_046149	ENSRNOT00000090621	0.95568525	1.25E-06
TUCP_001249	XLOC_046149	ENSRNOT00000016088	0.971667989	1.37E-07
TUCP_001249	XLOC_046149	ENSRNOT00000075397	0.962312787	5.62E-07
TUCP_001249	XLOC_046149	ENSRNOT00000008253	-0.973297604	1.02E-07
TUCP_001249	XLOC_046149	ENSRNOT00000032926	-0.951166342	2.01E-06
TUCP_001249	XLOC_046149	ENSRNOT00000009080	-0.9679338	2.53E-07
TUCP_001249	XLOC_046149	ENSRNOT00000063930	-0.956628167	1.12E-06
TUCP_001249	XLOC_046149	ENSRNOT00000000181	0.960912765	6.73E-07
TUCP_001249	XLOC_046149	ENSRNOT00000003087	0.957239856	1.05E-06
TUCP_001249	XLOC_046149	ENSRNOT00000067524	0.95580284	1.23E-06
TUCP_001249	XLOC_046149	ENSRNOT00000073493	0.969154912	2.09E-07
TUCP_001249	XLOC_046149	ENSRNOT00000049349	0.965455478	3.66E-07
TUCP_001249	XLOC_046149	ENSRNOT00000013025	-0.960270811	7.29E-07
TUCP_001249	XLOC_046149	ENSRNOT00000015476	-0.958320214	9.24E-07

TUCP_001249	XLOC_046149	ENSRNOT00000028624	0.951535056	1.94E-06
TUCP_001249	XLOC_046149	ENSRNOT00000032439	-0.955053746	1.34E-06
TUCP_001249	XLOC_046149	ENSRNOT00000022255	0.959650379	7.87E-07
TUCP_001249	XLOC_046149	ENSRNOT00000086706	0.951884531	1.87E-06
TUCP_001249	XLOC_046149	ENSRNOT00000022086	-0.961720444	6.07E-07
TUCP_001249	XLOC_046149	ENSRNOT00000057422	-0.96268336	5.35E-07
TUCP_001249	XLOC_046149	ENSRNOT00000021114	0.952051165	1.84E-06
TUCP_001249	XLOC_046149	ENSRNOT00000083968	-0.950381534	2.18E-06
TUCP_001249	XLOC_046149	ENSRNOT00000004391	0.975486235	6.69E-08
TUCP_001249	XLOC_046149	ENSRNOT00000074023	0.962574819	5.43E-07
TUCP_001249	XLOC_046149	ENSRNOT00000031692	-0.961670907	6.11E-07
TUCP_001249	XLOC_046149	ENSRNOT00000013851	-0.953153821	1.64E-06
TUCP_001249	XLOC_046149	ENSRNOT00000004351	-0.963762679	4.63E-07
TUCP_001249	XLOC_046149	ENSRNOT00000019180	-0.950486471	2.16E-06
TUCP_001249	XLOC_046149	ENSRNOT00000024710	-0.959363892	8.15E-07
TUCP_001249	XLOC_046149	ENSRNOT00000042459	-0.960278453	7.28E-07
TUCP_001249	XLOC_046149	ENSRNOT00000028695	-0.950087984	2.24E-06
TUCP_001249	XLOC_046149	ENSRNOT00000054685	0.959215422	8.30E-07
TUCP_001249	XLOC_046149	ENSRNOT00000022746	-0.975890957	6.16E-08
TUCP_001249	XLOC_046149	ENSRNOT00000023037	0.950236108	2.21E-06
TUCP_001249	XLOC_046149	ENSRNOT00000087604	0.968611812	2.28E-07
TUCP_001249	XLOC_046149	ENSRNOT00000057953	0.971527822	1.40E-07
TUCP_001249	XLOC_046149	ENSRNOT00000073370	0.956552607	1.13E-06
TUCP_001249	XLOC_046149	ENSRNOT00000012757	0.950404204	2.17E-06
TUCP_001249	XLOC_046149	ENSRNOT00000065978	0.95532813	1.30E-06
TUCP_001249	XLOC_046149	ENSRNOT00000005620	-0.959291757	8.22E-07
TUCP_001249	XLOC_046149	ENSRNOT00000039177	-0.95165941	1.92E-06
TUCP_001249	XLOC_046149	ENSRNOT00000020051	0.956021837	1.20E-06
TUCP_001249	XLOC_046149	ENSRNOT00000027760	0.958025874	9.56E-07
TUCP_001249	XLOC_046149	ENSRNOT00000007821	0.955688389	1.25E-06
TUCP_001249	XLOC_046149	ENSRNOT00000087287	0.954798297	1.38E-06
TUCP_001249	XLOC_046149	ENSRNOT00000085545	-0.97432729	8.41E-08
TUCP_001249	XLOC_046149	ENSRNOT00000081226	0.976787691	5.10E-08
TUCP_001249	XLOC_046149	ENSRNOT00000013286	0.959141917	8.37E-07
TUCP_001249	XLOC_046149	ENSRNOT00000075630	-0.955538952	1.27E-06
TUCP_001249	XLOC_046149	ENSRNOT00000081631	0.960975681	6.68E-07
TUCP_001249	XLOC_046149	ENSRNOT00000018634	-0.973135802	1.05E-07
TUCP_001249	XLOC_046149	ENSRNOT00000006607	-0.960485109	7.10E-07
TUCP_001249	XLOC_046149	ENSRNOT00000075934	-0.957400852	1.03E-06
TUCP_001249	XLOC_046149	ENSRNOT00000011832	-0.957786801	9.83E-07
TUCP_001249	XLOC_046149	ENSRNOT00000073643	-0.95416692	1.47E-06
TUCP_001249	XLOC_046149	ENSRNOT00000024947	-0.953793038	1.53E-06
TUCP_001249	XLOC_046149	ENSRNOT00000046552	0.960965031	6.68E-07
TUCP_001249	XLOC_046149	ENSRNOT00000023613	0.952739251	1.71E-06
TUCP_001249	XLOC_046149	ENSRNOT00000064776	-0.964942554	3.93E-07
TUCP_001249	XLOC_046149	ENSRNOT00000033806	0.961512207	6.23E-07
TUCP_001249	XLOC_046149	ENSRNOT00000037333	-0.953858392	1.52E-06
TUCP_001249	XLOC_046149	ENSRNOT00000029179	-0.966535887	3.12E-07
TUCP_001249	XLOC_046149	ENSRNOT00000000469	0.95997851	7.56E-07
TUCP_001249	XLOC_046149	ENSRNOT00000048781	0.953046554	1.66E-06
TUCP_001249	XLOC_046149	ENSRNOT00000003265	0.958063356	9.52E-07

TUCP_001249	XLOC_046149	ENSRNOT00000022309	0.959293832	8.22E-07
TUCP_001249	XLOC_046149	ENSRNOT00000011082	-0.952990984	1.67E-06
TUCP_001249	XLOC_046149	ENSRNOT00000083344	0.951160319	2.02E-06
TUCP_001249	XLOC_046149	ENSRNOT00000082627	-0.962383417	5.57E-07
TUCP_001249	XLOC_046149	ENSRNOT00000072118	0.953012048	1.67E-06
TUCP_001249	XLOC_046149	ENSRNOT00000014502	-0.97361687	9.63E-08
TUCP_001249	XLOC_046149	ENSRNOT00000085334	0.977190298	4.68E-08
TUCP_001249	XLOC_046149	ENSRNOT00000022190	-0.951668452	1.91E-06
TUCP_001249	XLOC_046149	ENSRNOT00000023262	-0.971555277	1.40E-07
TUCP_001249	XLOC_046149	ENSRNOT00000025913	-0.950927587	2.06E-06
TUCP_001249	XLOC_046149	ENSRNOT00000059246	0.951185653	2.01E-06
TUCP_001249	XLOC_046149	ENSRNOT00000013067	-0.956304241	1.17E-06
TUCP_001249	XLOC_046149	ENSRNOT00000004196	0.955235458	1.31E-06
TUCP_001249	XLOC_046149	ENSRNOT00000072299	0.954315603	1.45E-06
TUCP_001249	XLOC_046149	ENSRNOT00000078185	-0.955493134	1.28E-06
TUCP_001249	XLOC_046149	ENSRNOT00000035427	0.963420374	4.85E-07
TUCP_001249	XLOC_046149	ENSRNOT00000036658	-0.967145929	2.85E-07
TUCP_001249	XLOC_046149	ENSRNOT00000089611	0.978135038	3.79E-08
TUCP_001249	XLOC_046149	ENSRNOT00000010061	-0.96441871	4.23E-07
TUCP_001249	XLOC_046149	ENSRNOT00000016637	-0.964375061	4.26E-07
TUCP_001249	XLOC_046149	ENSRNOT00000038994	-0.964105879	4.42E-07
TUCP_001249	XLOC_046149	ENSRNOT00000086260	-0.976538746	5.38E-08
TUCP_001249	XLOC_046149	ENSRNOT00000008772	-0.959880113	7.65E-07
TUCP_001249	XLOC_046149	ENSRNOT00000018328	-0.95077736	2.09E-06
TUCP_001249	XLOC_046149	ENSRNOT00000066098	-0.968346679	2.37E-07
TUCP_001249	XLOC_046149	ENSRNOT00000068026	-0.973524861	9.80E-08
TUCP_001249	XLOC_046149	ENSRNOT00000025355	0.960358694	7.21E-07
TUCP_001249	XLOC_046149	ENSRNOT00000045765	-0.956195543	1.18E-06
TUCP_001249	XLOC_046149	ENSRNOT00000009327	-0.963438324	4.84E-07
TUCP_001249	XLOC_046149	ENSRNOT00000014506	-0.957619754	1.00E-06
TUCP_001249	XLOC_046149	ENSRNOT00000026531	-0.968370926	2.36E-07
TUCP_001249	XLOC_046149	ENSRNOT00000035605	0.958547373	8.99E-07
TUCP_001249	XLOC_046149	ENSRNOT00000090700	0.976075331	5.93E-08
TUCP_001249	XLOC_046149	ENSRNOT00000088214	0.96672923	3.04E-07
TUCP_001249	XLOC_046149	ENSRNOT00000020316	-0.95409713	1.49E-06
TUCP_001249	XLOC_046149	ENSRNOT00000017746	-0.95899752	8.52E-07
TUCP_001249	XLOC_046149	ENSRNOT00000087011	-0.950008238	2.26E-06
TUCP_001249	XLOC_046149	ENSRNOT00000008919	-0.955296423	1.30E-06
TUCP_001249	XLOC_046149	ENSRNOT00000006709	-0.952106847	1.83E-06
TUCP_001249	XLOC_046149	ENSRNOT00000017291	-0.952464153	1.76E-06
TUCP_001249	XLOC_046149	ENSRNOT00000071897	-0.960549814	7.04E-07
TUCP_001249	XLOC_046149	ENSRNOT00000027360	-0.953338258	1.61E-06
TUCP_001249	XLOC_046149	ENSRNOT00000066923	-0.957366376	1.03E-06
TUCP_001249	XLOC_046149	ENSRNOT00000029701	0.978903786	3.18E-08
TUCP_001249	XLOC_046149	ENSRNOT00000026988	-0.953849313	1.53E-06
TUCP_001249	XLOC_046149	ENSRNOT00000036051	0.963067129	5.09E-07
TUCP_001249	XLOC_046149	ENSRNOT00000029704	-0.950828063	2.08E-06
TUCP_001249	XLOC_046149	ENSRNOT00000001013	-0.953924043	1.51E-06
TUCP_001249	XLOC_046149	ENSRNOT00000025634	-0.964867555	3.97E-07
TUCP_001249	XLOC_046149	ENSRNOT00000065615	0.952536655	1.75E-06
TUCP_001249	XLOC_046149	ENSRNOT00000011675	0.953963505	1.51E-06

TUCP_001249	XLOC_046149	ENSRNOT00000055607	-0.96013979	7.41E-07
TUCP_001249	XLOC_046149	ENSRNOT00000068156	0.954940057	1.36E-06
TUCP_001249	XLOC_046149	ENSRNOT00000008074	-0.988991074	1.25E-09
TUCP_001249	XLOC_046149	ENSRNOT00000024452	-0.971631713	1.38E-07
TUCP_001249	XLOC_046149	ENSRNOT00000029115	-0.955369834	1.29E-06
TUCP_001249	XLOC_046149	ENSRNOT00000008764	0.964743577	4.04E-07
TUCP_001249	XLOC_046149	ENSRNOT00000066952	0.965991467	3.38E-07
TUCP_001249	XLOC_046149	ENSRNOT00000018477	-0.961237488	6.46E-07
TUCP_001249	XLOC_046149	ENSRNOT00000011130	-0.961019128	6.64E-07
TUCP_001249	XLOC_046149	ENSRNOT00000012428	-0.951693233	1.91E-06
TUCP_001249	XLOC_046149	ENSRNOT00000029328	0.963740939	4.64E-07
TUCP_001249	XLOC_046149	ENSRNOT00000016485	0.958765715	8.76E-07
TUCP_001249	XLOC_046149	ENSRNOT00000081604	0.954339707	1.45E-06
TUCP_001249	XLOC_046149	ENSRNOT00000087748	-0.954904119	1.36E-06
TUCP_001249	XLOC_046149	ENSRNOT00000049725	-0.954998993	1.35E-06
TUCP_001249	XLOC_046149	ENSRNOT00000087329	-0.956428604	1.15E-06
TUCP_001249	XLOC_046149	ENSRNOT00000008244	0.951088493	2.03E-06
TUCP_001249	XLOC_046149	ENSRNOT00000091990	-0.951239844	2.00E-06
TUCP_001249	XLOC_046149	ENSRNOT00000017504	0.953458264	1.59E-06
TUCP_001249	XLOC_046149	ENSRNOT00000031083	0.962177692	5.72E-07
TUCP_001249	XLOC_046149	ENSRNOT00000086456	-0.960684306	6.92E-07
TUCP_001249	XLOC_046149	ENSRNOT00000052033	-0.978887817	3.19E-08
TUCP_001249	XLOC_046149	ENSRNOT00000075209	-0.959784676	7.74E-07
TUCP_001249	XLOC_046149	ENSRNOT00000077578	-0.966304679	3.23E-07
TUCP_001249	XLOC_046149	ENSRNOT00000046268	-0.95646601	1.14E-06
TUCP_001249	XLOC_046149	ENSRNOT00000009202	0.962306334	5.62E-07
TUCP_001249	XLOC_046149	ENSRNOT00000010369	0.957020964	1.07E-06
TUCP_001249	XLOC_046149	ENSRNOT00000010993	0.96219903	5.70E-07
TUCP_001249	XLOC_046149	ENSRNOT00000005686	-0.951281566	1.99E-06
TUCP_001249	XLOC_046149	ENSRNOT00000083256	0.981253	1.77E-08
TUCP_001249	XLOC_046149	ENSRNOT00000010852	0.964157977	4.39E-07
TUCP_001249	XLOC_046149	ENSRNOT00000035385	0.960156339	7.40E-07
TUCP_001249	XLOC_046149	ENSRNOT00000061054	0.967519339	2.70E-07
TUCP_001249	XLOC_046149	ENSRNOT00000005873	-0.973091894	1.06E-07
TUCP_001249	XLOC_046149	ENSRNOT00000057738	0.977758783	4.13E-08
TUCP_001249	XLOC_046149	ENSRNOT00000009994	-0.960676455	6.93E-07
TUCP_001249	XLOC_046149	ENSRNOT00000065897	0.957140675	1.06E-06
TUCP_001249	XLOC_046149	ENSRNOT00000023014	0.961374675	6.35E-07
TUCP_001249	XLOC_046149	ENSRNOT00000026518	0.951582572	1.93E-06
TUCP_001249	XLOC_046149	ENSRNOT00000038449	0.952280316	1.80E-06
TUCP_001249	XLOC_046149	ENSRNOT00000035145	-0.979121222	3.02E-08
TUCP_001249	XLOC_046149	ENSRNOT00000085295	-0.953737461	1.54E-06
TUCP_001249	XLOC_046149	ENSRNOT00000087838	-0.957956931	9.64E-07
TUCP_001249	XLOC_046149	ENSRNOT00000064636	-0.95542816	1.29E-06
TUCP_001249	XLOC_046149	ENSRNOT00000079085	-0.951491429	1.95E-06
TUCP_001249	XLOC_046149	ENSRNOT00000093672	-0.954745147	1.39E-06
TUCP_001249	XLOC_046149	ENSRNOT00000038652	-0.955067086	1.34E-06
TUCP_001249	XLOC_046149	ENSRNOT00000074515	-0.969370314	2.02E-07
TUCP_001249	XLOC_046149	ENSRNOT00000075800	-0.955705365	1.25E-06
TUCP_001249	XLOC_046149	ENSRNOT00000017998	0.971122013	1.51E-07
TUCP_001249	XLOC_046149	ENSRNOT00000016568	0.973489878	9.86E-08

TUCP_001249	XLOC_046149	ENSRNOT00000005343	-0.964676737	4.08E-07
TUCP_001249	XLOC_046149	ENSRNOT00000012216	-0.970081087	1.80E-07
TUCP_001249	XLOC_046149	ENSRNOT00000082904	0.968973462	2.15E-07
TUCP_001249	XLOC_046149	ENSRNOT00000015223	0.967786859	2.59E-07
TUCP_001249	XLOC_046149	ENSRNOT00000088679	-0.958017972	9.57E-07
TUCP_001249	XLOC_046149	ENSRNOT00000049285	-0.956973947	1.08E-06
TUCP_001249	XLOC_046149	ENSRNOT00000079414	-0.950055644	2.25E-06
TUCP_001249	XLOC_046149	ENSRNOT00000035309	-0.981407245	1.70E-08
TUCP_001249	XLOC_046149	ENSRNOT00000024011	0.961420871	6.31E-07
TUCP_001249	XLOC_046149	ENSRNOT00000088073	-0.953656155	1.56E-06
TUCP_001249	XLOC_046149	ENSRNOT00000015239	-0.953210381	1.63E-06
TUCP_001248	XLOC_046147	ENSRNOT00000010680	0.952877834	1.69E-06
TUCP_001248	XLOC_046147	ENSRNOT00000067554	0.95053323	2.15E-06
TUCP_001248	XLOC_046147	ENSRNOT00000000383	-0.955121598	1.33E-06
TUCP_001248	XLOC_046147	ENSRNOT00000016088	0.950546086	2.14E-06
TUCP_001248	XLOC_046147	ENSRNOT00000081226	0.958674456	8.86E-07
TUCP_001248	XLOC_046147	ENSRNOT00000057738	0.954723118	1.39E-06
TUCP_001248	XLOC_046147	ENSRNOT00000038780	0.950964365	2.06E-06
TUCP_001248	XLOC_046147	ENSRNOT00000018634	-0.950424416	2.17E-06
TUCP_001248	XLOC_046147	ENSRNOT00000065897	0.960627187	6.97E-07
TUCP_001248	XLOC_046147	ENSRNOT00000089319	0.962484892	5.49E-07
TUCP_001248	XLOC_046147	ENSRNOT00000020832	0.952604723	1.74E-06
TUCP_001248	XLOC_046147	ENSRNOT00000029701	0.972380262	1.21E-07
TUCP_001248	XLOC_046147	ENSRNOT00000064776	-0.950489962	2.16E-06
TUCP_001248	XLOC_046147	ENSRNOT00000078752	-0.95806423	9.52E-07
TUCP_001248	XLOC_046147	ENSRNOT00000042638	0.957213457	1.05E-06
TUCP_001248	XLOC_046147	ENSRNOT00000002188	0.979628046	2.67E-08
TUCP_001248	XLOC_046147	ENSRNOT00000005770	0.951675317	1.91E-06
TUCP_001248	XLOC_046147	ENSRNOT00000085545	-0.956525274	1.14E-06
TUCP_001248	XLOC_046147	ENSRNOT00000018640	0.957729184	9.90E-07
TUCP_001248	XLOC_046147	ENSRNOT00000035808	0.964814362	4.00E-07
TUCP_001248	XLOC_046147	ENSRNOT00000016485	0.981270138	1.76E-08
TUCP_001248	XLOC_046147	ENSRNOT00000085334	0.965880733	3.44E-07
TUCP_001248	XLOC_046147	ENSRNOT00000057953	0.979039842	3.08E-08
TUCP_001248	XLOC_046147	ENSRNOT00000008764	0.956367342	1.16E-06
TUCP_001248	XLOC_046147	ENSRNOT00000005873	-0.958878004	8.64E-07
TUCP_001248	XLOC_046147	ENSRNOT00000009009	0.960936307	6.71E-07
TUCP_001248	XLOC_046147	ENSRNOT00000005257	0.950690068	2.11E-06
TUCP_001248	XLOC_046147	ENSRNOT00000009202	0.951379233	1.97E-06
TUCP_001241	XLOC_046143	ENSRNOT00000076294	0.958087711	9.49E-07
TUCP_001241	XLOC_046143	ENSRNOT00000078157	0.950959257	2.06E-06
TUCP_001241	XLOC_046143	ENSRNOT00000040840	0.959108005	8.41E-07
TUCP_001241	XLOC_046143	ENSRNOT00000083263	0.953051149	1.66E-06
TUCP_001243	XLOC_046143	ENSRNOT00000082545	0.976828647	5.06E-08
TUCP_004152	XLOC_180760	ENSRNOT00000024137	0.954919278	1.36E-06
TUCP_004152	XLOC_180760	ENSRNOT00000080088	0.963103948	5.06E-07
TUCP_004152	XLOC_180760	ENSRNOT00000051265	0.957512497	1.02E-06
TUCP_004152	XLOC_180760	ENSRNOT00000071697	0.951138978	2.02E-06
TUCP_001245	XLOC_046143	ENSRNOT00000024186	0.972113064	1.27E-07
TUCP_001245	XLOC_046143	ENSRNOT00000074753	0.956459235	1.15E-06
TUCP_001245	XLOC_046143	ENSRNOT00000004876	0.966040402	3.36E-07

TUCP_001245	XLOC_046143	ENSRNOT00000041904	0.964670491	4.09E-07
TUCP_001245	XLOC_046143	ENSRNOT00000083487	0.962309986	5.62E-07
TUCP_001245	XLOC_046143	ENSRNOT00000077990	0.964670491	4.09E-07
TUCP_001245	XLOC_046143	ENSRNOT00000038826	0.962103734	5.78E-07
TUCP_001245	XLOC_046143	ENSRNOT00000021243	0.955157349	1.32E-06
TUCP_001245	XLOC_046143	ENSRNOT00000060250	0.964120113	4.41E-07
TUCP_001245	XLOC_046143	ENSRNOT00000091934	0.964670491	4.09E-07
TUCP_001245	XLOC_046143	ENSRNOT00000034842	0.95361874	1.56E-06
TUCP_001245	XLOC_046143	ENSRNOT00000034907	0.951773054	1.89E-06
TUCP_001245	XLOC_046143	ENSRNOT00000014456	0.955060186	1.34E-06
TUCP_001245	XLOC_046143	ENSRNOT00000031205	0.97405295	8.87E-08
TUCP_001245	XLOC_046143	ENSRNOT00000044145	0.964670491	4.09E-07
TUCP_001245	XLOC_046143	ENSRNOT00000088381	0.955516023	1.27E-06
TUCP_001245	XLOC_046143	ENSRNOT00000057729	0.962592022	5.42E-07
TUCP_001245	XLOC_046143	ENSRNOT00000089929	0.964670491	4.09E-07
TUCP_001245	XLOC_046143	ENSRNOT00000088624	0.964670491	4.09E-07
TUCP_001245	XLOC_046143	ENSRNOT00000015866	0.956248751	1.17E-06
TUCP_001245	XLOC_046143	ENSRNOT00000064250	0.968362513	2.37E-07
TUCP_001245	XLOC_046143	ENSRNOT00000051835	0.964670491	4.09E-07
TUCP_001245	XLOC_046143	ENSRNOT00000079087	0.964670491	4.09E-07
TUCP_001245	XLOC_046143	ENSRNOT00000037206	0.964670491	4.09E-07
TUCP_001245	XLOC_046143	ENSRNOT00000080590	0.96835476	2.37E-07
TUCP_001245	XLOC_046143	ENSRNOT00000027552	0.967291583	2.79E-07
TUCP_001245	XLOC_046143	ENSRNOT00000077014	0.956769536	1.11E-06
TUCP_001245	XLOC_046143	ENSRNOT00000080453	0.965753622	3.50E-07
TUCP_001245	XLOC_046143	ENSRNOT00000066839	0.951530178	1.94E-06
TUCP_001245	XLOC_046143	ENSRNOT00000078633	0.964670491	4.09E-07
TUCP_001245	XLOC_046143	ENSRNOT00000037169	0.958055454	9.53E-07
TUCP_001245	XLOC_046143	ENSRNOT00000029634	0.959351035	8.16E-07
TUCP_001245	XLOC_046143	ENSRNOT00000065460	0.964670491	4.09E-07
TUCP_001245	XLOC_046143	ENSRNOT00000003817	0.959650077	7.87E-07
TUCP_001245	XLOC_046143	ENSRNOT00000016713	0.964670491	4.09E-07
TUCP_001245	XLOC_046143	ENSRNOT00000079260	0.962605754	5.41E-07
TUCP_001245	XLOC_046143	ENSRNOT00000039294	0.964670491	4.09E-07
TUCP_001245	XLOC_046143	ENSRNOT00000035840	0.950902189	2.07E-06
TUCP_001244	XLOC_046143	ENSRNOT00000024186	0.973130394	1.05E-07
TUCP_001244	XLOC_046143	ENSRNOT00000074753	0.993435062	9.50E-11
TUCP_001244	XLOC_046143	ENSRNOT00000041904	0.994074255	5.70E-11
TUCP_001244	XLOC_046143	ENSRNOT00000083487	0.975356971	6.87E-08
TUCP_001244	XLOC_046143	ENSRNOT00000077990	0.994074255	5.70E-11
TUCP_001244	XLOC_046143	ENSRNOT00000038826	0.993103255	1.21E-10
TUCP_001244	XLOC_046143	ENSRNOT00000037094	0.957889915	9.72E-07
TUCP_001244	XLOC_046143	ENSRNOT00000046842	0.962301207	5.63E-07
TUCP_001244	XLOC_046143	ENSRNOT00000037169	0.969297646	2.04E-07
TUCP_001244	XLOC_046143	ENSRNOT00000021243	0.986897177	2.98E-09
TUCP_001244	XLOC_046143	ENSRNOT00000060250	0.994893592	2.71E-11
TUCP_001244	XLOC_046143	ENSRNOT00000091934	0.994074255	5.70E-11
TUCP_001244	XLOC_046143	ENSRNOT00000086027	0.958098495	9.48E-07
TUCP_001244	XLOC_046143	ENSRNOT00000046585	0.977339898	4.53E-08
TUCP_001244	XLOC_046143	ENSRNOT00000080794	0.98557478	4.80E-09
TUCP_001244	XLOC_046143	ENSRNOT00000014456	0.98414454	7.68E-09

TUCP_001244	XLOC_046143	ENSRNOT00000031205	0.984061832	7.89E-09
TUCP_001244	XLOC_046143	ENSRNOT00000044145	0.994074255	5.70E-11
TUCP_001244	XLOC_046143	ENSRNOT00000088381	0.98379554	8.56E-09
TUCP_001244	XLOC_046143	ENSRNOT00000011743	0.976976973	4.90E-08
TUCP_001244	XLOC_046143	ENSRNOT00000089929	0.994074255	5.70E-11
TUCP_001244	XLOC_046143	ENSRNOT00000088624	0.994074255	5.70E-11
TUCP_001244	XLOC_046143	ENSRNOT00000085659	0.990243537	6.85E-10
TUCP_001244	XLOC_046143	ENSRNOT00000015866	0.987332444	2.51E-09
TUCP_001244	XLOC_046143	ENSRNOT00000078633	0.994074255	5.70E-11
TUCP_001244	XLOC_046143	ENSRNOT00000024575	0.950327218	2.19E-06
TUCP_001244	XLOC_046143	ENSRNOT00000034907	0.985052901	5.73E-09
TUCP_001244	XLOC_046143	ENSRNOT00000051835	0.994074255	5.70E-11
TUCP_001244	XLOC_046143	ENSRNOT00000009825	0.954758765	1.38E-06
TUCP_001244	XLOC_046143	ENSRNOT00000079087	0.994074255	5.70E-11
TUCP_001244	XLOC_046143	ENSRNOT00000037206	0.994074255	5.70E-11
TUCP_001244	XLOC_046143	ENSRNOT00000061185	0.950835607	2.08E-06
TUCP_001244	XLOC_046143	ENSRNOT00000083261	0.974330952	8.41E-08
TUCP_001244	XLOC_046143	ENSRNOT00000080590	0.993508981	8.98E-11
TUCP_001244	XLOC_046143	ENSRNOT00000027552	0.994379476	4.38E-11
TUCP_001244	XLOC_046143	ENSRNOT00000077014	0.99615113	6.61E-12
TUCP_001244	XLOC_046143	ENSRNOT00000080453	0.994111082	5.52E-11
TUCP_001244	XLOC_046143	ENSRNOT00000089057	0.951495588	1.95E-06
TUCP_001244	XLOC_046143	ENSRNOT00000057729	0.992389126	1.99E-10
TUCP_001244	XLOC_046143	ENSRNOT00000061284	0.966786096	3.01E-07
TUCP_001244	XLOC_046143	ENSRNOT00000020206	0.952960146	1.68E-06
TUCP_001244	XLOC_046143	ENSRNOT00000013423	0.96169305	6.09E-07
TUCP_001244	XLOC_046143	ENSRNOT00000071884	0.974702389	7.82E-08
TUCP_001244	XLOC_046143	ENSRNOT00000066839	0.986720038	3.18E-09
TUCP_001244	XLOC_046143	ENSRNOT00000026316	0.960185044	7.37E-07
TUCP_001244	XLOC_046143	ENSRNOT00000078663	0.985723143	4.56E-09
TUCP_001244	XLOC_046143	ENSRNOT00000064250	0.981129134	1.83E-08
TUCP_001244	XLOC_046143	ENSRNOT00000021608	0.96988476	1.85E-07
TUCP_001244	XLOC_046143	ENSRNOT00000029634	0.988213862	1.76E-09
TUCP_001244	XLOC_046143	ENSRNOT00000093579	0.959555631	7.96E-07
TUCP_001244	XLOC_046143	ENSRNOT00000065460	0.994074255	5.70E-11
TUCP_001244	XLOC_046143	ENSRNOT00000003817	0.994975808	2.50E-11
TUCP_001244	XLOC_046143	ENSRNOT00000016713	0.994074255	5.70E-11
TUCP_001244	XLOC_046143	ENSRNOT00000079260	0.987934305	1.97E-09
TUCP_001244	XLOC_046143	ENSRNOT00000039294	0.994074255	5.70E-11
TUCP_001244	XLOC_046143	ENSRNOT00000024337	0.952330869	1.79E-06
TUCP_001247	XLOC_046146	ENSRNOT00000016088	0.953876062	1.52E-06
TUCP_001247	XLOC_046146	ENSRNOT00000075397	0.956982016	1.08E-06
TUCP_001247	XLOC_046146	ENSRNOT00000020051	0.956066868	1.20E-06
TUCP_001247	XLOC_046146	ENSRNOT00000009080	-0.95634851	1.16E-06
TUCP_001247	XLOC_046146	ENSRNOT00000063930	-0.952680926	1.73E-06
TUCP_001247	XLOC_046146	ENSRNOT00000000181	0.958913315	8.61E-07
TUCP_001247	XLOC_046146	ENSRNOT00000049349	0.953490543	1.58E-06
TUCP_001247	XLOC_046146	ENSRNOT00000001691	0.955970051	1.21E-06
TUCP_001247	XLOC_046146	ENSRNOT00000089319	0.975241527	7.03E-08
TUCP_001247	XLOC_046146	ENSRNOT00000078737	-0.963146001	5.03E-07
TUCP_001247	XLOC_046146	ENSRNOT00000086706	0.967307401	2.78E-07

TUCP_001247	XLOC_046146	ENSRNOT00000004017	0.95478211	1.38E-06
TUCP_001247	XLOC_046146	ENSRNOT00000021114	0.969186527	2.08E-07
TUCP_001247	XLOC_046146	ENSRNOT00000016485	0.974840192	7.61E-08
TUCP_001247	XLOC_046146	ENSRNOT00000008074	-0.953686022	1.55E-06
TUCP_001247	XLOC_046146	ENSRNOT00000074023	0.96146275	6.27E-07
TUCP_001247	XLOC_046146	ENSRNOT00000014384	-0.951171789	2.01E-06
TUCP_001247	XLOC_046146	ENSRNOT00000042638	0.963420553	4.85E-07
TUCP_001247	XLOC_046146	ENSRNOT00000007028	-0.950733557	2.10E-06
TUCP_001247	XLOC_046146	ENSRNOT00000057562	0.953662167	1.56E-06
TUCP_001247	XLOC_046146	ENSRNOT00000054685	0.950485967	2.16E-06
TUCP_001247	XLOC_046146	ENSRNOT00000057953	0.990495543	6.01E-10
TUCP_001247	XLOC_046146	ENSRNOT00000073370	0.957246582	1.05E-06
TUCP_001247	XLOC_046146	ENSRNOT00000019921	0.958461587	9.08E-07
TUCP_001247	XLOC_046146	ENSRNOT00000007821	0.973886436	9.15E-08
TUCP_001247	XLOC_046146	ENSRNOT00000089933	0.952327628	1.79E-06
TUCP_001247	XLOC_046146	ENSRNOT00000067554	0.983641295	8.98E-09
TUCP_001247	XLOC_046146	ENSRNOT00000010784	0.958588536	8.95E-07
TUCP_001247	XLOC_046146	ENSRNOT00000085545	-0.960671128	6.94E-07
TUCP_001247	XLOC_046146	ENSRNOT00000061253	0.954141503	1.48E-06
TUCP_001247	XLOC_046146	ENSRNOT00000081226	0.955976402	1.21E-06
TUCP_001247	XLOC_046146	ENSRNOT00000018634	-0.969783316	1.89E-07
TUCP_001247	XLOC_046146	ENSRNOT00000011832	-0.950126757	2.23E-06
TUCP_001247	XLOC_046146	ENSRNOT00000073643	-0.95525827	1.31E-06
TUCP_001247	XLOC_046146	ENSRNOT00000017556	0.957464794	1.02E-06
TUCP_001247	XLOC_046146	ENSRNOT00000001893	0.972405842	1.20E-07
TUCP_001247	XLOC_046146	ENSRNOT00000080577	0.950500902	2.15E-06
TUCP_001247	XLOC_046146	ENSRNOT00000004351	-0.950095674	2.24E-06
TUCP_001247	XLOC_046146	ENSRNOT00000072118	0.96983796	1.87E-07
TUCP_001247	XLOC_046146	ENSRNOT00000085334	0.952004887	1.85E-06
TUCP_001247	XLOC_046146	ENSRNOT00000022190	-0.953251592	1.63E-06
TUCP_001247	XLOC_046146	ENSRNOT00000023262	-0.951113922	2.03E-06
TUCP_001247	XLOC_046146	ENSRNOT00000013067	-0.95296501	1.67E-06
TUCP_001247	XLOC_046146	ENSRNOT00000024119	0.952699858	1.72E-06
TUCP_001247	XLOC_046146	ENSRNOT00000026312	0.950625951	2.13E-06
TUCP_001247	XLOC_046146	ENSRNOT00000086260	-0.964566204	4.14E-07
TUCP_001247	XLOC_046146	ENSRNOT00000068026	-0.956411095	1.15E-06
TUCP_001247	XLOC_046146	ENSRNOT00000029701	0.976735081	5.16E-08
TUCP_001247	XLOC_046146	ENSRNOT00000058382	0.950491399	2.16E-06
TUCP_001247	XLOC_046146	ENSRNOT00000000383	-0.967139598	2.86E-07
TUCP_001247	XLOC_046146	ENSRNOT00000082711	0.966588919	3.10E-07
TUCP_001247	XLOC_046146	ENSRNOT00000072357	-0.952076646	1.84E-06
TUCP_001247	XLOC_046146	ENSRNOT00000026531	-0.961684084	6.10E-07
TUCP_001247	XLOC_046146	ENSRNOT00000084031	0.951296861	1.99E-06
TUCP_001247	XLOC_046146	ENSRNOT00000017746	-0.950240988	2.21E-06
TUCP_001247	XLOC_046146	ENSRNOT00000005343	-0.950743675	2.10E-06
TUCP_001247	XLOC_046146	ENSRNOT00000005459	0.950072025	2.25E-06
TUCP_001247	XLOC_046146	ENSRNOT00000055602	-0.950256689	2.21E-06
TUCP_001247	XLOC_046146	ENSRNOT00000055607	-0.953031182	1.66E-06
TUCP_001247	XLOC_046146	ENSRNOT00000080750	0.953382109	1.60E-06
TUCP_001247	XLOC_046146	ENSRNOT00000013801	0.964524679	4.17E-07
TUCP_001247	XLOC_046146	ENSRNOT00000033116	0.95369223	1.55E-06

TUCP_001247	XLOC_046146	ENSRNOT00000002188	0.966946641	2.94E-07
TUCP_001247	XLOC_046146	ENSRNOT00000029328	0.958450678	9.09E-07
TUCP_001247	XLOC_046146	ENSRNOT00000074972	0.959660163	7.86E-07
TUCP_001247	XLOC_046146	ENSRNOT00000010993	0.95311171	1.65E-06
TUCP_001247	XLOC_046146	ENSRNOT00000005486	-0.962057716	5.81E-07
TUCP_001247	XLOC_046146	ENSRNOT00000010852	0.952327802	1.79E-06
TUCP_001247	XLOC_046146	ENSRNOT00000014926	0.954584676	1.41E-06
TUCP_001247	XLOC_046146	ENSRNOT00000057738	0.963647546	4.70E-07
TUCP_001247	XLOC_046146	ENSRNOT00000065897	0.962219347	5.69E-07
TUCP_001247	XLOC_046146	ENSRNOT00000080608	0.961309674	6.40E-07
TUCP_001247	XLOC_046146	ENSRNOT00000046215	-0.950566096	2.14E-06
TUCP_001247	XLOC_046146	ENSRNOT00000000120	0.952716154	1.72E-06
TUCP_001247	XLOC_046146	ENSRNOT00000010983	0.952944514	1.68E-06
TUCP_001247	XLOC_046146	ENSRNOT00000000469	0.969773202	1.89E-07
TUCP_001247	XLOC_046146	ENSRNOT00000035808	0.961694098	6.09E-07
TUCP_001247	XLOC_046146	ENSRNOT00000015769	0.953241127	1.63E-06
TUCP_001247	XLOC_046146	ENSRNOT00000016568	0.956995933	1.08E-06
TUCP_001247	XLOC_046146	ENSRNOT00000027360	-0.95483783	1.37E-06
TUCP_001247	XLOC_046146	ENSRNOT00000082904	0.960934759	6.71E-07
TUCP_001247	XLOC_046146	ENSRNOT00000015223	0.956074999	1.20E-06
TUCP_001247	XLOC_046146	ENSRNOT00000007672	0.950752946	2.10E-06
TUCP_001247	XLOC_046146	ENSRNOT00000090057	0.977585761	4.29E-08
TUCP_001247	XLOC_046146	ENSRNOT00000025705	0.951370306	1.97E-06
TUCP_001247	XLOC_046146	ENSRNOT00000061580	-0.964406389	4.24E-07
TUCP_001247	XLOC_046146	ENSRNOT00000005257	0.968205678	2.43E-07
TUCP_002349	XLOC_098054	ENSRNOT00000005095	0.950231875	2.21E-06
TUCP_002349	XLOC_098054	ENSRNOT00000014629	0.962216086	5.69E-07
TUCP_001360	XLOC_052435	ENSRNOT00000087199	0.957219065	1.05E-06
TUCP_001360	XLOC_052435	ENSRNOT00000076072	0.997882162	3.34E-13
TUCP_001360	XLOC_052435	ENSRNOT00000084374	0.987720681	2.15E-09
TUCP_001367	XLOC_052624	ENSRNOT00000085332	0.951264454	1.99E-06
TUCP_001367	XLOC_052624	ENSRNOT00000073879	-0.955421004	1.29E-06
TUCP_001367	XLOC_052624	ENSRNOT00000024281	-0.95176639	1.90E-06
TUCP_001367	XLOC_052624	ENSRNOT00000034201	-0.967944233	2.53E-07
TUCP_001367	XLOC_052624	ENSRNOT00000087306	-0.951916466	1.87E-06
TUCP_002348	XLOC_098054	ENSRNOT00000010680	0.960280737	7.28E-07
TUCP_002348	XLOC_098054	ENSRNOT00000005505	0.954442572	1.43E-06
TUCP_002348	XLOC_098054	ENSRNOT00000009975	0.959005078	8.51E-07
TUCP_002348	XLOC_098054	ENSRNOT00000009960	0.952346086	1.79E-06
TUCP_002348	XLOC_098054	ENSRNOT00000083623	0.965073335	3.86E-07
TUCP_002348	XLOC_098054	ENSRNOT00000001642	0.956318234	1.16E-06
TUCP_002348	XLOC_098054	ENSRNOT00000016485	0.951079109	2.03E-06
TUCP_002348	XLOC_098054	ENSRNOT00000078752	-0.96026065	7.30E-07
TUCP_002348	XLOC_098054	ENSRNOT00000034482	0.955076517	1.34E-06
TUCP_002348	XLOC_098054	ENSRNOT00000006109	0.952980275	1.67E-06
TUCP_002348	XLOC_098054	ENSRNOT00000006697	0.951779713	1.89E-06
TUCP_000763	XLOC_028735	ENSRNOT00000020329	0.954823291	1.37E-06
TUCP_001369	XLOC_052624	ENSRNOT00000088355	0.983748247	8.69E-09
TUCP_001369	XLOC_052624	ENSRNOT00000049798	-0.958950576	8.57E-07
TUCP_001369	XLOC_052624	ENSRNOT00000021358	0.968061986	2.48E-07
TUCP_001369	XLOC_052624	ENSRNOT00000088919	-0.960270535	7.29E-07

TUCP_000764	XLOC_028835	ENSRNOT00000089807	0.965487078	3.64E-07
TUCP_001716	XLOC_069842	ENSRNOT00000044971	0.967771415	2.59E-07
TUCP_001716	XLOC_069842	ENSRNOT00000091013	0.950734061	2.10E-06
TUCP_000831	XLOC_032209	ENSRNOT00000009975	0.952794799	1.70E-06
TUCP_000831	XLOC_032209	ENSRNOT00000072682	0.95981479	7.71E-07
TUCP_000831	XLOC_032209	ENSRNOT00000076113	0.957752387	9.87E-07
TUCP_000831	XLOC_032209	ENSRNOT00000086144	0.952995455	1.67E-06
TUCP_000831	XLOC_032209	ENSRNOT00000042890	0.960665627	6.94E-07
TUCP_000831	XLOC_032209	ENSRNOT00000021590	0.955179654	1.32E-06
TUCP_000831	XLOC_032209	ENSRNOT00000010142	0.978058493	3.86E-08
TUCP_000831	XLOC_032209	ENSRNOT00000047453	0.976108376	5.89E-08
TUCP_000831	XLOC_032209	ENSRNOT00000005306	0.950763309	2.10E-06
TUCP_000831	XLOC_032209	ENSRNOT00000041762	0.952098164	1.83E-06
TUCP_000831	XLOC_032209	ENSRNOT00000010539	0.952598441	1.74E-06
TUCP_000831	XLOC_032209	ENSRNOT00000082172	0.953835342	1.53E-06
TUCP_000831	XLOC_032209	ENSRNOT00000021452	-0.950212504	2.22E-06
TUCP_000831	XLOC_032209	ENSRNOT00000012757	0.960520394	7.07E-07
TUCP_000831	XLOC_032209	ENSRNOT00000037902	0.954304654	1.45E-06
TUCP_000831	XLOC_032209	ENSRNOT00000006947	0.952155636	1.82E-06
TUCP_000831	XLOC_032209	ENSRNOT00000005505	0.969983206	1.82E-07
TUCP_000831	XLOC_032209	ENSRNOT00000023623	0.961456208	6.28E-07
TUCP_000831	XLOC_032209	ENSRNOT00000023607	0.970865609	1.57E-07
TUCP_000831	XLOC_032209	ENSRNOT00000006109	0.963523086	4.78E-07
TUCP_000831	XLOC_032209	ENSRNOT00000016804	0.957292742	1.04E-06
TUCP_000831	XLOC_032209	ENSRNOT00000023661	0.969342933	2.03E-07
TUCP_002657	XLOC_112799	ENSRNOT00000020329	0.953614176	1.56E-06
TUCP_002657	XLOC_112799	ENSRNOT00000040509	0.958474604	9.07E-07
TUCP_002657	XLOC_112799	ENSRNOT00000072939	0.987949291	1.96E-09
TUCP_002657	XLOC_112799	ENSRNOT00000073792	0.9660302	3.36E-07
TUCP_002657	XLOC_112799	ENSRNOT00000093579	0.962018089	5.84E-07
TUCP_002657	XLOC_112799	ENSRNOT00000015889	0.950899334	2.07E-06
TUCP_002657	XLOC_112799	ENSRNOT00000061139	0.975979774	6.05E-08
TUCP_002657	XLOC_112799	ENSRNOT00000012170	0.958212861	9.35E-07
TUCP_000833	XLOC_032339	ENSRNOT00000032992	0.954214173	1.47E-06
TUCP_000833	XLOC_032339	ENSRNOT00000024156	0.952043029	1.84E-06
TUCP_000833	XLOC_032339	ENSRNOT00000078274	0.955355593	1.30E-06
TUCP_000833	XLOC_032339	ENSRNOT00000066810	0.950108384	2.24E-06
TUCP_000833	XLOC_032339	ENSRNOT00000090518	0.95748734	1.02E-06
TUCP_000833	XLOC_032339	ENSRNOT00000043156	0.950159018	2.23E-06
TUCP_000833	XLOC_032339	ENSRNOT00000012156	0.956960679	1.08E-06
TUCP_000833	XLOC_032339	ENSRNOT00000049819	0.954920722	1.36E-06
TUCP_000832	XLOC_032339	ENSRNOT00000031904	0.966605694	3.09E-07
TUCP_000832	XLOC_032339	ENSRNOT00000027083	0.956151899	1.19E-06
TUCP_000832	XLOC_032339	ENSRNOT00000021348	0.956835894	1.10E-06
TUCP_000832	XLOC_032339	ENSRNOT00000017665	0.955806919	1.23E-06
TUCP_000832	XLOC_032339	ENSRNOT00000023133	0.971916962	1.31E-07
TUCP_000832	XLOC_032339	ENSRNOT00000003891	0.953838099	1.53E-06
TUCP_000832	XLOC_032339	ENSRNOT00000073559	0.97269396	1.14E-07
TUCP_000832	XLOC_032339	ENSRNOT00000001290	0.962082162	5.79E-07
TUCP_000832	XLOC_032339	ENSRNOT00000023638	0.960034242	7.51E-07
TUCP_000832	XLOC_032339	ENSRNOT00000005228	0.954799905	1.38E-06

TUCP_000832	XLOC_032339	ENSRNOT00000008677	0.95700894	1.08E-06
TUCP_000832	XLOC_032339	ENSRNOT00000006175	0.961292993	6.41E-07
TUCP_000832	XLOC_032339	ENSRNOT00000028628	-0.955238824	1.31E-06
TUCP_000832	XLOC_032339	ENSRNOT00000005220	0.960727335	6.89E-07
TUCP_000832	XLOC_032339	ENSRNOT00000023144	0.96910054	2.11E-07
TUCP_000832	XLOC_032339	ENSRNOT00000082869	0.951657627	1.92E-06
TUCP_000832	XLOC_032339	ENSRNOT00000071166	0.960933893	6.71E-07
TUCP_000832	XLOC_032339	ENSRNOT00000016649	0.971483852	1.42E-07
TUCP_000832	XLOC_032339	ENSRNOT00000016975	0.959423843	8.09E-07
TUCP_000832	XLOC_032339	ENSRNOT00000030850	0.962313472	5.62E-07
TUCP_000832	XLOC_032339	ENSRNOT00000043216	0.950263421	2.20E-06
TUCP_000832	XLOC_032339	ENSRNOT00000091128	-0.950891889	2.07E-06
TUCP_000832	XLOC_032339	ENSRNOT00000081981	0.957130617	1.06E-06
TUCP_000832	XLOC_032339	ENSRNOT00000033017	0.983055891	1.07E-08
TUCP_000832	XLOC_032339	ENSRNOT00000026543	0.970197339	1.76E-07
TUCP_000832	XLOC_032339	ENSRNOT00000071971	0.959696754	7.83E-07
TUCP_000832	XLOC_032339	ENSRNOT00000015006	0.951679548	1.91E-06
TUCP_000832	XLOC_032339	ENSRNOT00000012187	0.972168305	1.26E-07
TUCP_000832	XLOC_032339	ENSRNOT00000024969	0.959832146	7.70E-07
TUCP_000832	XLOC_032339	ENSRNOT00000085251	-0.961982375	5.87E-07
TUCP_000832	XLOC_032339	ENSRNOT00000013049	0.981130298	1.83E-08
TUCP_000832	XLOC_032339	ENSRNOT00000034609	0.951822049	1.88E-06
TUCP_000832	XLOC_032339	ENSRNOT00000045285	0.960110213	7.44E-07
TUCP_000832	XLOC_032339	ENSRNOT00000044742	0.969932214	1.84E-07
TUCP_000832	XLOC_032339	ENSRNOT00000048754	-0.951846414	1.88E-06
TUCP_000832	XLOC_032339	ENSRNOT00000045699	0.958908788	8.61E-07
TUCP_000832	XLOC_032339	ENSRNOT00000038247	0.95550322	1.27E-06
TUCP_000832	XLOC_032339	ENSRNOT00000029439	0.96739806	2.75E-07
TUCP_000832	XLOC_032339	ENSRNOT00000044628	0.960462165	7.12E-07
TUCP_000832	XLOC_032339	ENSRNOT00000071764	0.964883262	3.96E-07
TUCP_000832	XLOC_032339	ENSRNOT00000081906	0.950199992	2.22E-06
TUCP_000832	XLOC_032339	ENSRNOT00000057160	0.951623393	1.92E-06
TUCP_000832	XLOC_032339	ENSRNOT00000024402	0.953772077	1.54E-06
TUCP_000832	XLOC_032339	ENSRNOT00000091319	0.962536131	5.46E-07
TUCP_000832	XLOC_032339	ENSRNOT00000089933	0.95488466	1.36E-06
TUCP_000832	XLOC_032339	ENSRNOT00000083876	-0.951666785	1.91E-06
TUCP_000832	XLOC_032339	ENSRNOT00000012671	0.952650753	1.73E-06
TUCP_000832	XLOC_032339	ENSRNOT00000016947	0.955248763	1.31E-06
TUCP_000832	XLOC_032339	ENSRNOT00000065168	0.984088449	7.82E-09
TUCP_000832	XLOC_032339	ENSRNOT00000024266	0.960218386	7.34E-07
TUCP_000832	XLOC_032339	ENSRNOT00000026718	0.955653261	1.25E-06
TUCP_000832	XLOC_032339	ENSRNOT00000085228	0.976773991	5.12E-08
TUCP_000832	XLOC_032339	ENSRNOT00000032780	0.954621948	1.40E-06
TUCP_000832	XLOC_032339	ENSRNOT00000020134	0.986821311	3.06E-09
TUCP_000832	XLOC_032339	ENSRNOT00000079866	0.960805811	6.82E-07
TUCP_000832	XLOC_032339	ENSRNOT00000005839	0.958643636	8.89E-07
TUCP_000832	XLOC_032339	ENSRNOT00000041513	0.955231641	1.31E-06
TUCP_000832	XLOC_032339	ENSRNOT00000010381	0.952337965	1.79E-06
TUCP_000832	XLOC_032339	ENSRNOT00000087592	0.952692545	1.72E-06
TUCP_000832	XLOC_032339	ENSRNOT00000019820	0.953025286	1.66E-06
TUCP_000832	XLOC_032339	ENSRNOT00000005958	0.963150513	5.03E-07

TUCP_000832	XLOC_032339	ENSRNOT00000002410	0.98069222	2.05E-08
TUCP_000832	XLOC_032339	ENSRNOT00000008655	0.964123303	4.41E-07
TUCP_000832	XLOC_032339	ENSRNOT00000045047	0.958301104	9.26E-07
TUCP_000832	XLOC_032339	ENSRNOT00000091909	0.953701219	1.55E-06
TUCP_000832	XLOC_032339	ENSRNOT00000075326	0.957870123	9.74E-07
TUCP_000832	XLOC_032339	ENSRNOT00000025362	-0.961429764	6.30E-07
TUCP_000832	XLOC_032339	ENSRNOT00000010541	0.967339626	2.77E-07
TUCP_000832	XLOC_032339	ENSRNOT00000017556	0.96370212	4.67E-07
TUCP_000832	XLOC_032339	ENSRNOT00000012895	0.956839063	1.10E-06
TUCP_000832	XLOC_032339	ENSRNOT00000000468	0.953336235	1.61E-06
TUCP_000832	XLOC_032339	ENSRNOT00000023989	0.969965111	1.83E-07
TUCP_000832	XLOC_032339	ENSRNOT00000012892	0.965676113	3.54E-07
TUCP_000832	XLOC_032339	ENSRNOT00000020028	0.963450499	4.83E-07
TUCP_000832	XLOC_032339	ENSRNOT00000090918	0.96131832	6.39E-07
TUCP_000832	XLOC_032339	ENSRNOT00000000676	0.951033291	2.04E-06
TUCP_000832	XLOC_032339	ENSRNOT00000083220	0.963905758	4.54E-07
TUCP_000832	XLOC_032339	ENSRNOT00000028266	0.952871064	1.69E-06
TUCP_000832	XLOC_032339	ENSRNOT00000015609	0.951915291	1.87E-06
TUCP_000832	XLOC_032339	ENSRNOT00000019133	0.95637723	1.16E-06
TUCP_000832	XLOC_032339	ENSRNOT00000065983	0.953363795	1.61E-06
TUCP_000832	XLOC_032339	ENSRNOT00000052018	0.983661059	8.92E-09
TUCP_000832	XLOC_032339	ENSRNOT00000010898	0.955960502	1.21E-06
TUCP_000832	XLOC_032339	ENSRNOT00000047093	0.956690027	1.12E-06
TUCP_000832	XLOC_032339	ENSRNOT00000078274	0.951931352	1.86E-06
TUCP_000832	XLOC_032339	ENSRNOT00000050515	0.951573558	1.93E-06
TUCP_000832	XLOC_032339	ENSRNOT00000021001	0.964680816	4.08E-07
TUCP_000832	XLOC_032339	ENSRNOT00000048063	0.952011987	1.85E-06
TUCP_000832	XLOC_032339	ENSRNOT00000052422	0.962168219	5.73E-07
TUCP_000832	XLOC_032339	ENSRNOT00000086119	-0.959386695	8.13E-07
TUCP_000832	XLOC_032339	ENSRNOT00000033975	0.974827868	7.63E-08
TUCP_000832	XLOC_032339	ENSRNOT00000078102	0.950367284	2.18E-06
TUCP_000832	XLOC_032339	ENSRNOT00000009811	0.972692615	1.14E-07
TUCP_000832	XLOC_032339	ENSRNOT00000090123	0.969776796	1.89E-07
TUCP_000832	XLOC_032339	ENSRNOT00000081768	0.952002013	1.85E-06
TUCP_000832	XLOC_032339	ENSRNOT00000020155	0.952991017	1.67E-06
TUCP_000832	XLOC_032339	ENSRNOT00000088599	0.967297558	2.79E-07
TUCP_000832	XLOC_032339	ENSRNOT00000081047	0.969434206	2.00E-07
TUCP_000832	XLOC_032339	ENSRNOT00000081512	0.950651558	2.12E-06
TUCP_000832	XLOC_032339	ENSRNOT00000014537	0.964581803	4.14E-07
TUCP_000832	XLOC_032339	ENSRNOT00000077555	0.955746611	1.24E-06
TUCP_000832	XLOC_032339	ENSRNOT00000023537	-0.953542003	1.58E-06
TUCP_000832	XLOC_032339	ENSRNOT00000017021	0.957769016	9.85E-07
TUCP_000832	XLOC_032339	ENSRNOT00000021717	0.96117155	6.51E-07
TUCP_000832	XLOC_032339	ENSRNOT00000003991	0.955355272	1.30E-06
TUCP_000832	XLOC_032339	ENSRNOT00000021408	0.960778354	6.84E-07
TUCP_000832	XLOC_032339	ENSRNOT00000087429	0.95943193	8.08E-07
TUCP_000832	XLOC_032339	ENSRNOT00000009683	0.952467425	1.76E-06
TUCP_000832	XLOC_032339	ENSRNOT00000060362	0.965828528	3.46E-07
TUCP_000832	XLOC_032339	ENSRNOT00000084088	0.950332938	2.19E-06
TUCP_000832	XLOC_032339	ENSRNOT00000017776	0.953457893	1.59E-06
TUCP_000832	XLOC_032339	ENSRNOT00000027910	0.950798211	2.09E-06

TUCP_000832	XLOC_032339	ENSRNOT00000052224	0.973533224	9.78E-08
TUCP_000832	XLOC_032339	ENSRNOT00000078070	0.960200281	7.36E-07
TUCP_000832	XLOC_032339	ENSRNOT00000083214	0.96396047	4.51E-07
TUCP_000832	XLOC_032339	ENSRNOT00000024322	0.968670783	2.26E-07
TUCP_000832	XLOC_032339	ENSRNOT00000088182	0.951419953	1.96E-06
TUCP_000832	XLOC_032339	ENSRNOT00000011408	0.953397223	1.60E-06
TUCP_000832	XLOC_032339	ENSRNOT00000011270	0.950044215	2.25E-06
TUCP_000832	XLOC_032339	ENSRNOT00000091265	0.974081791	8.82E-08
TUCP_000832	XLOC_032339	ENSRNOT00000002443	0.951274877	1.99E-06
TUCP_000832	XLOC_032339	ENSRNOT00000016191	0.966096468	3.33E-07
TUCP_000832	XLOC_032339	ENSRNOT00000078390	-0.952809213	1.70E-06
TUCP_000832	XLOC_032339	ENSRNOT00000065501	0.964786935	4.02E-07
TUCP_000832	XLOC_032339	ENSRNOT00000027454	0.955254298	1.31E-06
TUCP_000832	XLOC_032339	ENSRNOT00000025832	0.950546328	2.14E-06
TUCP_000832	XLOC_032339	ENSRNOT00000017181	-0.956702634	1.11E-06
TUCP_000832	XLOC_032339	ENSRNOT00000017753	0.965794599	3.48E-07
TUCP_000832	XLOC_032339	ENSRNOT00000006499	0.962355464	5.59E-07
TUCP_000832	XLOC_032339	ENSRNOT00000066739	0.957006601	1.08E-06
TUCP_000832	XLOC_032339	ENSRNOT00000030445	0.953007473	1.67E-06
TUCP_000832	XLOC_032339	ENSRNOT00000045882	0.952685297	1.72E-06
TUCP_000832	XLOC_032339	ENSRNOT00000029833	0.950560521	2.14E-06
TUCP_000832	XLOC_032339	ENSRNOT00000028057	0.951496376	1.95E-06
TUCP_000832	XLOC_032339	ENSRNOT00000086122	0.959033208	8.48E-07
TUCP_000832	XLOC_032339	ENSRNOT00000022074	0.968186447	2.43E-07
TUCP_000832	XLOC_032339	ENSRNOT00000015520	0.971402585	1.44E-07
TUCP_000832	XLOC_032339	ENSRNOT00000044174	0.96632672	3.22E-07
TUCP_000832	XLOC_032339	ENSRNOT00000041365	0.952635572	1.73E-06
TUCP_000832	XLOC_032339	ENSRNOT00000086577	0.955754227	1.24E-06
TUCP_000832	XLOC_032339	ENSRNOT00000025075	0.971205155	1.49E-07
TUCP_000832	XLOC_032339	ENSRNOT00000019886	0.957452109	1.02E-06
TUCP_000832	XLOC_032339	ENSRNOT00000058842	0.952240514	1.81E-06
TUCP_000832	XLOC_032339	ENSRNOT00000000157	0.951623215	1.92E-06
TUCP_000832	XLOC_032339	ENSRNOT00000077231	0.959216932	8.30E-07
TUCP_000832	XLOC_032339	ENSRNOT00000083093	0.958496963	9.04E-07
TUCP_000832	XLOC_032339	ENSRNOT00000047192	-0.977776244	4.11E-08
TUCP_000832	XLOC_032339	ENSRNOT00000084422	0.954520743	1.42E-06
TUCP_000832	XLOC_032339	ENSRNOT00000043872	0.953207622	1.63E-06
TUCP_000832	XLOC_032339	ENSRNOT00000021471	-0.950321213	2.19E-06
TUCP_000832	XLOC_032339	ENSRNOT00000080443	0.959744835	7.78E-07
TUCP_000832	XLOC_032339	ENSRNOT00000041096	0.954085504	1.49E-06
TUCP_000832	XLOC_032339	ENSRNOT00000046323	0.958232132	9.33E-07
TUCP_000832	XLOC_032339	ENSRNOT00000080608	0.960698679	6.91E-07
TUCP_000832	XLOC_032339	ENSRNOT00000077406	0.961333915	6.38E-07
TUCP_000832	XLOC_032339	ENSRNOT00000027970	0.964486662	4.19E-07
TUCP_000832	XLOC_032339	ENSRNOT00000010748	0.965566968	3.60E-07
TUCP_000832	XLOC_032339	ENSRNOT00000026915	0.96346847	4.82E-07
TUCP_000832	XLOC_032339	ENSRNOT00000011052	0.955424797	1.29E-06
TUCP_000832	XLOC_032339	ENSRNOT00000005478	-0.951828961	1.88E-06
TUCP_000832	XLOC_032339	ENSRNOT00000083127	0.961265111	6.43E-07
TUCP_000832	XLOC_032339	ENSRNOT00000066370	0.952450626	1.77E-06
TUCP_000832	XLOC_032339	ENSRNOT00000035474	0.965230074	3.78E-07

TUCP_000832	XLOC_032339	ENSRNOT00000039341	0.95374929	1.54E-06
TUCP_000832	XLOC_032339	ENSRNOT00000061392	0.981308719	1.74E-08
TUCP_000832	XLOC_032339	ENSRNOT00000076872	0.960249769	7.31E-07
TUCP_000832	XLOC_032339	ENSRNOT00000014124	0.964563492	4.15E-07
TUCP_000832	XLOC_032339	ENSRNOT00000029125	0.952926191	1.68E-06
TUCP_000832	XLOC_032339	ENSRNOT00000060696	0.981396222	1.70E-08
TUCP_000832	XLOC_032339	ENSRNOT00000042125	0.969706335	1.91E-07
TUCP_000832	XLOC_032339	ENSRNOT00000009900	-0.953630107	1.56E-06
TUCP_000832	XLOC_032339	ENSRNOT00000045735	0.950657573	2.12E-06
TUCP_000832	XLOC_032339	ENSRNOT00000064798	0.966189358	3.29E-07
TUCP_000832	XLOC_032339	ENSRNOT00000080207	0.960829027	6.80E-07
TUCP_000832	XLOC_032339	ENSRNOT00000026280	0.965710456	3.52E-07
TUCP_000832	XLOC_032339	ENSRNOT00000033235	0.961165449	6.52E-07
TUCP_000832	XLOC_032339	ENSRNOT00000005387	0.959783112	7.74E-07
TUCP_000832	XLOC_032339	ENSRNOT00000027452	0.966523112	3.13E-07
TUCP_000832	XLOC_032339	ENSRNOT00000022863	0.956466915	1.14E-06
TUCP_000832	XLOC_032339	ENSRNOT00000043535	0.959151924	8.36E-07
TUCP_000832	XLOC_032339	ENSRNOT00000040393	0.952795336	1.70E-06
TUCP_000832	XLOC_032339	ENSRNOT00000026457	0.95140855	1.97E-06
TUCP_000832	XLOC_032339	ENSRNOT00000004463	0.951377792	1.97E-06
TUCP_000832	XLOC_032339	ENSRNOT00000023428	0.962001757	5.85E-07
TUCP_000832	XLOC_032339	ENSRNOT00000011041	0.957899273	9.70E-07
TUCP_000832	XLOC_032339	ENSRNOT00000064700	0.955290792	1.30E-06
TUCP_000832	XLOC_032339	ENSRNOT00000037800	0.950170874	2.22E-06
TUCP_000832	XLOC_032339	ENSRNOT00000022796	0.9615656	6.19E-07
TUCP_000832	XLOC_032339	ENSRNOT00000080110	0.950713628	2.11E-06
TUCP_000832	XLOC_032339	ENSRNOT00000083957	0.962628019	5.39E-07
TUCP_000832	XLOC_032339	ENSRNOT00000015799	0.986090873	4.01E-09
TUCP_000832	XLOC_032339	ENSRNOT00000044608	-0.956517564	1.14E-06
TUCP_000832	XLOC_032339	ENSRNOT00000083799	0.964789973	4.02E-07
TUCP_000832	XLOC_032339	ENSRNOT00000024548	0.95764978	9.99E-07
TUCP_000832	XLOC_032339	ENSRNOT00000048020	0.950915137	2.07E-06
TUCP_000835	XLOC_032384	ENSRNOT00000001579	-0.957877134	9.73E-07
TUCP_000835	XLOC_032384	ENSRNOT00000031963	0.950102626	2.24E-06
TUCP_000835	XLOC_032384	ENSRNOT00000004609	0.964650384	4.10E-07
TUCP_000835	XLOC_032384	ENSRNOT00000027630	0.959909968	7.62E-07
TUCP_000835	XLOC_032384	ENSRNOT00000091000	0.958798405	8.72E-07
TUCP_000835	XLOC_032384	ENSRNOT00000084912	0.969611624	1.94E-07
TUCP_000835	XLOC_032384	ENSRNOT00000034157	0.962843881	5.24E-07
TUCP_000835	XLOC_032384	ENSRNOT00000016499	0.952505747	1.76E-06
TUCP_000835	XLOC_032384	ENSRNOT00000045049	0.95013033	2.23E-06
TUCP_000834	XLOC_032339	ENSRNOT00000036143	0.951981391	1.85E-06
TUCP_000834	XLOC_032339	ENSRNOT00000060035	0.977044465	4.83E-08
TUCP_000834	XLOC_032339	ENSRNOT00000082691	0.954124482	1.48E-06
TUCP_000834	XLOC_032339	ENSRNOT00000063956	0.952637466	1.73E-06
TUCP_000834	XLOC_032339	ENSRNOT00000036061	0.95649755	1.14E-06
TUCP_000834	XLOC_032339	ENSRNOT00000017373	0.960747178	6.87E-07
TUCP_000834	XLOC_032339	ENSRNOT00000038431	0.956756032	1.11E-06
TUCP_000834	XLOC_032339	ENSRNOT00000060811	0.952402567	1.78E-06
TUCP_000834	XLOC_032339	ENSRNOT00000038912	0.953321728	1.61E-06
TUCP_000834	XLOC_032339	ENSRNOT00000067489	0.969923152	1.84E-07

TUCP_000834	XLOC_032339	ENSRNOT00000027275	0.951582283	1.93E-06
TUCP_000834	XLOC_032339	ENSRNOT00000013093	-0.96181507	6.00E-07
TUCP_002159	XLOC_090256	ENSRNOT00000073559	0.973460794	9.92E-08
TUCP_002159	XLOC_090256	ENSRNOT00000027407	0.971144714	1.50E-07
TUCP_002159	XLOC_090256	ENSRNOT00000013743	0.9764419	5.49E-08
TUCP_002159	XLOC_090256	ENSRNOT00000075714	0.950583018	2.14E-06
TUCP_002159	XLOC_090256	ENSRNOT00000087062	0.956221465	1.18E-06
TUCP_002159	XLOC_090256	ENSRNOT00000012052	0.952698644	1.72E-06
TUCP_002159	XLOC_090256	ENSRNOT00000008677	0.950969492	2.05E-06
TUCP_002159	XLOC_090256	ENSRNOT00000017361	0.968347822	2.37E-07
TUCP_002159	XLOC_090256	ENSRNOT00000086706	0.966252306	3.26E-07
TUCP_002159	XLOC_090256	ENSRNOT00000071166	0.959291506	8.22E-07
TUCP_002159	XLOC_090256	ENSRNOT00000004017	0.983725436	8.75E-09
TUCP_002159	XLOC_090256	ENSRNOT00000021114	0.956429748	1.15E-06
TUCP_002159	XLOC_090256	ENSRNOT00000086310	-0.955068654	1.34E-06
TUCP_002159	XLOC_090256	ENSRNOT00000090342	0.959937049	7.60E-07
TUCP_002159	XLOC_090256	ENSRNOT00000028856	0.955417903	1.29E-06
TUCP_002159	XLOC_090256	ENSRNOT00000030850	0.978963483	3.13E-08
TUCP_002159	XLOC_090256	ENSRNOT00000001586	-0.959991608	7.55E-07
TUCP_002159	XLOC_090256	ENSRNOT00000001289	0.964943444	3.93E-07
TUCP_002159	XLOC_090256	ENSRNOT00000009496	0.953740075	1.54E-06
TUCP_002159	XLOC_090256	ENSRNOT00000029332	-0.959670886	7.85E-07
TUCP_002159	XLOC_090256	ENSRNOT00000044742	0.960176082	7.38E-07
TUCP_002159	XLOC_090256	ENSRNOT00000081786	-0.959437603	8.08E-07
TUCP_002159	XLOC_090256	ENSRNOT00000071971	0.968301242	2.39E-07
TUCP_002159	XLOC_090256	ENSRNOT00000040847	-0.950141669	2.23E-06
TUCP_002159	XLOC_090256	ENSRNOT00000015006	0.967827982	2.57E-07
TUCP_002159	XLOC_090256	ENSRNOT00000014479	-0.951209225	2.01E-06
TUCP_002159	XLOC_090256	ENSRNOT00000010083	0.958749911	8.78E-07
TUCP_002159	XLOC_090256	ENSRNOT00000061005	0.954278073	1.46E-06
TUCP_002159	XLOC_090256	ENSRNOT00000072771	0.957016503	1.07E-06
TUCP_002159	XLOC_090256	ENSRNOT00000073370	0.950236566	2.21E-06
TUCP_002159	XLOC_090256	ENSRNOT00000074683	0.956522342	1.14E-06
TUCP_002159	XLOC_090256	ENSRNOT00000021453	0.956740927	1.11E-06
TUCP_002159	XLOC_090256	ENSRNOT00000045699	0.951772093	1.89E-06
TUCP_002159	XLOC_090256	ENSRNOT00000084709	0.953226225	1.63E-06
TUCP_002159	XLOC_090256	ENSRNOT00000035320	-0.955439639	1.28E-06
TUCP_002159	XLOC_090256	ENSRNOT00000045047	0.950258658	2.21E-06
TUCP_002159	XLOC_090256	ENSRNOT00000004007	-0.952927437	1.68E-06
TUCP_002159	XLOC_090256	ENSRNOT00000007821	0.967003151	2.91E-07
TUCP_002159	XLOC_090256	ENSRNOT00000077908	0.95164135	1.92E-06
TUCP_002159	XLOC_090256	ENSRNOT00000090461	0.951440239	1.96E-06
TUCP_002159	XLOC_090256	ENSRNOT00000009811	0.960968504	6.68E-07
TUCP_002159	XLOC_090256	ENSRNOT00000065168	0.952301753	1.79E-06
TUCP_002159	XLOC_090256	ENSRNOT00000026398	-0.95022538	2.21E-06
TUCP_002159	XLOC_090256	ENSRNOT00000067554	0.958039842	9.55E-07
TUCP_002159	XLOC_090256	ENSRNOT00000085228	0.966228931	3.27E-07
TUCP_002159	XLOC_090256	ENSRNOT00000010784	0.976717661	5.18E-08
TUCP_002159	XLOC_090256	ENSRNOT00000010783	-0.95550855	1.27E-06
TUCP_002159	XLOC_090256	ENSRNOT00000017319	0.955412823	1.29E-06
TUCP_002159	XLOC_090256	ENSRNOT00000015188	0.965874029	3.44E-07

TUCP_002159	XLOC_090256	ENSRNOT00000033677	-0.951649839	1.92E-06
TUCP_002159	XLOC_090256	ENSRNOT00000086065	0.967719083	2.61E-07
TUCP_002159	XLOC_090256	ENSRNOT00000079866	0.957533282	1.01E-06
TUCP_002159	XLOC_090256	ENSRNOT00000005839	0.976700805	5.20E-08
TUCP_002159	XLOC_090256	ENSRNOT00000040629	0.952243185	1.81E-06
TUCP_002159	XLOC_090256	ENSRNOT00000072196	0.958453943	9.09E-07
TUCP_002159	XLOC_090256	ENSRNOT00000017486	0.951618922	1.92E-06
TUCP_002159	XLOC_090256	ENSRNOT00000019820	0.972139284	1.26E-07
TUCP_002159	XLOC_090256	ENSRNOT00000061392	0.961461935	6.27E-07
TUCP_002159	XLOC_090256	ENSRNOT00000012364	-0.954469181	1.43E-06
TUCP_002159	XLOC_090256	ENSRNOT00000002410	0.977261317	4.61E-08
TUCP_002159	XLOC_090256	ENSRNOT00000027012	0.967987799	2.51E-07
TUCP_002159	XLOC_090256	ENSRNOT00000052002	0.961761058	6.04E-07
TUCP_002159	XLOC_090256	ENSRNOT00000021744	-0.959714051	7.81E-07
TUCP_002159	XLOC_090256	ENSRNOT00000021037	0.960944503	6.70E-07
TUCP_002159	XLOC_090256	ENSRNOT00000083968	-0.955192565	1.32E-06
TUCP_002159	XLOC_090256	ENSRNOT00000026831	0.961138512	6.54E-07
TUCP_002159	XLOC_090256	ENSRNOT00000073991	0.956912643	1.09E-06
TUCP_002159	XLOC_090256	ENSRNOT00000017556	0.957767449	9.86E-07
TUCP_002159	XLOC_090256	ENSRNOT00000091637	0.960090505	7.46E-07
TUCP_002159	XLOC_090256	ENSRNOT00000009156	0.964186294	4.37E-07
TUCP_002159	XLOC_090256	ENSRNOT00000026481	0.957249153	1.05E-06
TUCP_002159	XLOC_090256	ENSRNOT00000000468	0.951557123	1.94E-06
TUCP_002159	XLOC_090256	ENSRNOT00000044562	0.956719571	1.11E-06
TUCP_002159	XLOC_090256	ENSRNOT00000021802	0.950455104	2.16E-06
TUCP_002159	XLOC_090256	ENSRNOT00000012892	0.981997302	1.44E-08
TUCP_002159	XLOC_090256	ENSRNOT00000020028	0.964474821	4.20E-07
TUCP_002159	XLOC_090256	ENSRNOT00000061304	-0.956331644	1.16E-06
TUCP_002159	XLOC_090256	ENSRNOT00000077231	0.966368691	3.20E-07
TUCP_002159	XLOC_090256	ENSRNOT00000028266	0.959240195	8.27E-07
TUCP_002159	XLOC_090256	ENSRNOT00000080124	0.959267693	8.25E-07
TUCP_002159	XLOC_090256	ENSRNOT00000072357	-0.952498518	1.76E-06
TUCP_002159	XLOC_090256	ENSRNOT00000016132	-0.958090119	9.49E-07
TUCP_002159	XLOC_090256	ENSRNOT00000016321	-0.951162209	2.02E-06
TUCP_002159	XLOC_090256	ENSRNOT00000019133	0.950272225	2.20E-06
TUCP_002159	XLOC_090256	ENSRNOT00000087583	0.974151172	8.70E-08
TUCP_002159	XLOC_090256	ENSRNOT00000078187	0.951895744	1.87E-06
TUCP_002159	XLOC_090256	ENSRNOT00000002151	-0.950725681	2.11E-06
TUCP_002159	XLOC_090256	ENSRNOT00000018122	0.973894803	9.14E-08
TUCP_002159	XLOC_090256	ENSRNOT00000014454	0.95367943	1.55E-06
TUCP_002159	XLOC_090256	ENSRNOT00000010224	0.972816173	1.12E-07
TUCP_002159	XLOC_090256	ENSRNOT00000016637	-0.952462243	1.76E-06
TUCP_002159	XLOC_090256	ENSRNOT00000052422	0.965983037	3.39E-07
TUCP_002159	XLOC_090256	ENSRNOT00000064798	0.954180205	1.47E-06
TUCP_002159	XLOC_090256	ENSRNOT00000078741	0.951070779	2.03E-06
TUCP_002159	XLOC_090256	ENSRNOT00000068381	0.976933822	4.95E-08
TUCP_002159	XLOC_090256	ENSRNOT00000019531	-0.952522408	1.75E-06
TUCP_002159	XLOC_090256	ENSRNOT00000064588	0.954074881	1.49E-06
TUCP_002159	XLOC_090256	ENSRNOT00000026633	0.956214961	1.18E-06
TUCP_002159	XLOC_090256	ENSRNOT00000020155	0.956651039	1.12E-06
TUCP_002159	XLOC_090256	ENSRNOT00000025827	0.96192494	5.91E-07

TUCP_002159	XLOC_090256	ENSRNOT00000015769	0.973681037	9.52E-08
TUCP_002159	XLOC_090256	ENSRNOT00000014537	0.95832616	9.23E-07
TUCP_002159	XLOC_090256	ENSRNOT00000077555	0.950625539	2.13E-06
TUCP_002159	XLOC_090256	ENSRNOT00000011880	0.964642515	4.10E-07
TUCP_002159	XLOC_090256	ENSRNOT00000005486	-0.964834877	3.99E-07
TUCP_002159	XLOC_090256	ENSRNOT00000023989	0.970502159	1.67E-07
TUCP_002159	XLOC_090256	ENSRNOT00000021717	0.974103115	8.78E-08
TUCP_002159	XLOC_090256	ENSRNOT00000049423	-0.969512493	1.97E-07
TUCP_002159	XLOC_090256	ENSRNOT00000019502	0.954900778	1.36E-06
TUCP_002159	XLOC_090256	ENSRNOT00000050515	0.963907638	4.54E-07
TUCP_002159	XLOC_090256	ENSRNOT00000023477	0.961948249	5.89E-07
TUCP_002159	XLOC_090256	ENSRNOT00000081725	0.957518922	1.01E-06
TUCP_002159	XLOC_090256	ENSRNOT00000089980	-0.963969825	4.50E-07
TUCP_002159	XLOC_090256	ENSRNOT00000005459	0.956872516	1.09E-06
TUCP_002159	XLOC_090256	ENSRNOT00000078073	-0.980836938	1.97E-08
TUCP_002159	XLOC_090256	ENSRNOT00000002771	0.954889983	1.36E-06
TUCP_002159	XLOC_090256	ENSRNOT00000046745	0.95834966	9.20E-07
TUCP_002159	XLOC_090256	ENSRNOT00000055607	-0.960795218	6.83E-07
TUCP_002159	XLOC_090256	ENSRNOT00000087502	0.953289004	1.62E-06
TUCP_002159	XLOC_090256	ENSRNOT00000024322	0.958164122	9.41E-07
TUCP_002159	XLOC_090256	ENSRNOT00000041106	0.967450707	2.72E-07
TUCP_002159	XLOC_090256	ENSRNOT00000011408	0.954514177	1.42E-06
TUCP_002159	XLOC_090256	ENSRNOT00000000577	0.958745524	8.78E-07
TUCP_002159	XLOC_090256	ENSRNOT00000010463	0.959361171	8.15E-07
TUCP_002159	XLOC_090256	ENSRNOT00000023876	0.968708132	2.24E-07
TUCP_002159	XLOC_090256	ENSRNOT00000021944	0.954065119	1.49E-06
TUCP_002159	XLOC_090256	ENSRNOT00000008427	-0.953659707	1.56E-06
TUCP_002159	XLOC_090256	ENSRNOT00000000013	0.958626318	8.91E-07
TUCP_002159	XLOC_090256	ENSRNOT00000028888	0.97081813	1.59E-07
TUCP_002159	XLOC_090256	ENSRNOT00000020005	0.950653364	2.12E-06
TUCP_002159	XLOC_090256	ENSRNOT00000002109	0.975361184	6.86E-08
TUCP_002159	XLOC_090256	ENSRNOT00000006499	0.954119442	1.48E-06
TUCP_002159	XLOC_090256	ENSRNOT00000024119	0.965668522	3.55E-07
TUCP_002159	XLOC_090256	ENSRNOT00000029833	0.956657942	1.12E-06
TUCP_002159	XLOC_090256	ENSRNOT00000035277	0.960439591	7.14E-07
TUCP_002159	XLOC_090256	ENSRNOT00000000728	-0.953837663	1.53E-06
TUCP_002159	XLOC_090256	ENSRNOT00000086122	0.96020361	7.35E-07
TUCP_002159	XLOC_090256	ENSRNOT00000022074	0.957737274	9.89E-07
TUCP_002159	XLOC_090256	ENSRNOT00000026231	0.954229527	1.46E-06
TUCP_002159	XLOC_090256	ENSRNOT00000078161	-0.952539665	1.75E-06
TUCP_002159	XLOC_090256	ENSRNOT00000004999	0.950105174	2.24E-06
TUCP_002159	XLOC_090256	ENSRNOT00000026312	0.955011845	1.35E-06
TUCP_002159	XLOC_090256	ENSRNOT00000041365	0.975178806	7.12E-08
TUCP_002159	XLOC_090256	ENSRNOT00000021803	-0.956038046	1.20E-06
TUCP_002159	XLOC_090256	ENSRNOT00000008337	0.951362319	1.98E-06
TUCP_002159	XLOC_090256	ENSRNOT00000043752	-0.951395237	1.97E-06
TUCP_002159	XLOC_090256	ENSRNOT00000023979	0.960005685	7.53E-07
TUCP_002159	XLOC_090256	ENSRNOT00000040540	0.961553996	6.20E-07
TUCP_002159	XLOC_090256	ENSRNOT00000037246	0.968905128	2.17E-07
TUCP_002159	XLOC_090256	ENSRNOT00000034411	0.970787735	1.60E-07
TUCP_002159	XLOC_090256	ENSRNOT00000059181	0.960451901	7.13E-07

TUCP_002159	XLOC_090256	ENSRNOT00000027666	0.963074264	5.08E-07
TUCP_002159	XLOC_090256	ENSRNOT00000072518	0.963037134	5.11E-07
TUCP_002159	XLOC_090256	ENSRNOT00000003690	0.953681936	1.55E-06
TUCP_002159	XLOC_090256	ENSRNOT00000063951	0.960181784	7.37E-07
TUCP_002159	XLOC_090256	ENSRNOT00000085619	0.95142221	1.96E-06
TUCP_002159	XLOC_090256	ENSRNOT00000058111	-0.951478547	1.95E-06
TUCP_002159	XLOC_090256	ENSRNOT00000082245	0.95334356	1.61E-06
TUCP_002159	XLOC_090256	ENSRNOT00000008901	-0.954377856	1.44E-06
TUCP_002159	XLOC_090256	ENSRNOT00000019487	0.950870817	2.08E-06
TUCP_002159	XLOC_090256	ENSRNOT00000017761	0.959104703	8.41E-07
TUCP_002159	XLOC_090256	ENSRNOT00000054828	0.965874504	3.44E-07
TUCP_002159	XLOC_090256	ENSRNOT00000080443	0.978591874	3.42E-08
TUCP_002159	XLOC_090256	ENSRNOT00000015898	0.982595701	1.22E-08
TUCP_002159	XLOC_090256	ENSRNOT00000046323	0.960877301	6.76E-07
TUCP_002159	XLOC_090256	ENSRNOT00000080608	0.967543753	2.69E-07
TUCP_002159	XLOC_090256	ENSRNOT00000027970	0.955197755	1.32E-06
TUCP_002159	XLOC_090256	ENSRNOT00000010748	0.968591298	2.28E-07
TUCP_002159	XLOC_090256	ENSRNOT00000005405	0.978574312	3.43E-08
TUCP_002159	XLOC_090256	ENSRNOT00000009462	0.958636813	8.89E-07
TUCP_002159	XLOC_090256	ENSRNOT00000058563	0.979374057	2.84E-08
TUCP_002159	XLOC_090256	ENSRNOT00000035474	0.957637454	1.00E-06
TUCP_002159	XLOC_090256	ENSRNOT00000039341	0.950495461	2.15E-06
TUCP_002159	XLOC_090256	ENSRNOT00000084422	0.961920606	5.91E-07
TUCP_002159	XLOC_090256	ENSRNOT00000004172	0.963603054	4.73E-07
TUCP_002159	XLOC_090256	ENSRNOT00000078994	0.978114876	3.81E-08
TUCP_002159	XLOC_090256	ENSRNOT00000014124	0.970313587	1.73E-07
TUCP_002159	XLOC_090256	ENSRNOT00000040476	0.95770394	9.93E-07
TUCP_002159	XLOC_090256	ENSRNOT00000013632	0.960269609	7.29E-07
TUCP_002159	XLOC_090256	ENSRNOT00000010983	0.9617273	6.06E-07
TUCP_002159	XLOC_090256	ENSRNOT00000091488	0.956866146	1.09E-06
TUCP_002159	XLOC_090256	ENSRNOT00000085279	0.966232547	3.27E-07
TUCP_002159	XLOC_090256	ENSRNOT00000007276	-0.956264203	1.17E-06
TUCP_002159	XLOC_090256	ENSRNOT00000055442	0.961298999	6.41E-07
TUCP_002159	XLOC_090256	ENSRNOT00000019172	-0.954319161	1.45E-06
TUCP_002159	XLOC_090256	ENSRNOT00000061432	0.975436951	6.76E-08
TUCP_002159	XLOC_090256	ENSRNOT00000015799	0.95166512	1.92E-06
TUCP_002159	XLOC_090256	ENSRNOT00000026280	0.962272511	5.65E-07
TUCP_002159	XLOC_090256	ENSRNOT00000033235	0.955814086	1.23E-06
TUCP_002159	XLOC_090256	ENSRNOT00000021216	0.959250316	8.26E-07
TUCP_002159	XLOC_090256	ENSRNOT00000063917	-0.972852455	1.11E-07
TUCP_002159	XLOC_090256	ENSRNOT00000017776	0.960714075	6.90E-07
TUCP_002159	XLOC_090256	ENSRNOT00000017165	-0.974672941	7.87E-08
TUCP_002159	XLOC_090256	ENSRNOT00000013985	0.951869995	1.88E-06
TUCP_002159	XLOC_090256	ENSRNOT00000004463	0.953120702	1.65E-06
TUCP_002159	XLOC_090256	ENSRNOT00000044009	0.961956479	5.89E-07
TUCP_002159	XLOC_090256	ENSRNOT00000064700	0.972731784	1.13E-07
TUCP_002159	XLOC_090256	ENSRNOT00000025887	-0.95473319	1.39E-06
TUCP_002159	XLOC_090256	ENSRNOT00000024011	0.95368981	1.55E-06
TUCP_002159	XLOC_090256	ENSRNOT00000061580	-0.95712675	1.06E-06
TUCP_002159	XLOC_090256	ENSRNOT00000017687	-0.966622974	3.08E-07
TUCP_002159	XLOC_090256	ENSRNOT00000031401	-0.95658703	1.13E-06

TUCP_002159	XLOC_090256	ENSRNOT00000036931	-0.952744914	1.71E-06
TUCP_002159	XLOC_090256	ENSRNOT00000001607	0.962712829	5.33E-07
TUCP_000839	XLOC_032453	ENSRNOT00000022290	0.967333965	2.77E-07
TUCP_000839	XLOC_032453	ENSRNOT00000066419	0.95878113	8.74E-07
TUCP_000839	XLOC_032453	ENSRNOT00000001092	0.952292167	1.80E-06
TUCP_000839	XLOC_032453	ENSRNOT00000007963	-0.952581709	1.74E-06
TUCP_000839	XLOC_032453	ENSRNOT00000011864	-0.953592909	1.57E-06
TUCP_000839	XLOC_032453	ENSRNOT00000089377	0.966670668	3.06E-07
TUCP_000574	XLOC_020459	ENSRNOT00000001810	0.954569831	1.41E-06
TUCP_001412	XLOC_055123	ENSRNOT00000024186	0.983198584	1.03E-08
TUCP_001412	XLOC_055123	ENSRNOT00000074753	0.998188286	1.53E-13
TUCP_001412	XLOC_055123	ENSRNOT00000004876	0.959061272	8.45E-07
TUCP_001412	XLOC_055123	ENSRNOT00000041904	0.999999943	4.90E-36
TUCP_001412	XLOC_055123	ENSRNOT00000083487	0.989494335	9.90E-10
TUCP_001412	XLOC_055123	ENSRNOT00000077990	0.999999943	4.90E-36
TUCP_001412	XLOC_055123	ENSRNOT00000038826	0.999775548	4.48E-18
TUCP_001412	XLOC_055123	ENSRNOT00000046842	0.973636533	9.60E-08
TUCP_001412	XLOC_055123	ENSRNOT00000037169	0.980325085	2.25E-08
TUCP_001412	XLOC_055123	ENSRNOT00000021243	0.987419995	2.43E-09
TUCP_001412	XLOC_055123	ENSRNOT00000060250	0.999937692	7.39E-21
TUCP_001412	XLOC_055123	ENSRNOT00000091934	0.999999943	4.90E-36
TUCP_001412	XLOC_055123	ENSRNOT00000046585	0.982934636	1.11E-08
TUCP_001412	XLOC_055123	ENSRNOT00000080794	0.990831457	5.02E-10
TUCP_001412	XLOC_055123	ENSRNOT00000014456	0.99169274	3.07E-10
TUCP_001412	XLOC_055123	ENSRNOT00000031205	0.991388435	3.68E-10
TUCP_001412	XLOC_055123	ENSRNOT00000044145	0.999999943	4.90E-36
TUCP_001412	XLOC_055123	ENSRNOT00000088381	0.989008211	1.24E-09
TUCP_001412	XLOC_055123	ENSRNOT00000011743	0.986778725	3.11E-09
TUCP_001412	XLOC_055123	ENSRNOT00000089929	0.999999943	4.90E-36
TUCP_001412	XLOC_055123	ENSRNOT00000088624	0.999999943	4.90E-36
TUCP_001412	XLOC_055123	ENSRNOT00000085659	0.989903764	8.12E-10
TUCP_001412	XLOC_055123	ENSRNOT00000015866	0.994057382	5.78E-11
TUCP_001412	XLOC_055123	ENSRNOT00000078633	0.999999943	4.90E-36
TUCP_001412	XLOC_055123	ENSRNOT00000034907	0.995302289	1.79E-11
TUCP_001412	XLOC_055123	ENSRNOT00000093230	0.95827513	9.28E-07
TUCP_001412	XLOC_055123	ENSRNOT00000051835	0.999999943	4.90E-36
TUCP_001412	XLOC_055123	ENSRNOT00000079087	0.999999943	4.90E-36
TUCP_001412	XLOC_055123	ENSRNOT00000037206	0.999999943	4.90E-36
TUCP_001412	XLOC_055123	ENSRNOT00000083261	0.971013979	1.53E-07
TUCP_001412	XLOC_055123	ENSRNOT00000080590	0.999888655	1.35E-19
TUCP_001412	XLOC_055123	ENSRNOT00000027552	0.999804208	2.27E-18
TUCP_001412	XLOC_055123	ENSRNOT00000077014	0.995102032	2.20E-11
TUCP_001412	XLOC_055123	ENSRNOT00000080453	0.999944146	4.28E-21
TUCP_001412	XLOC_055123	ENSRNOT00000089057	0.968205171	2.43E-07
TUCP_001412	XLOC_055123	ENSRNOT00000066839	0.991863304	2.77E-10
TUCP_001412	XLOC_055123	ENSRNOT00000061284	0.955598619	1.26E-06
TUCP_001412	XLOC_055123	ENSRNOT00000013423	0.959681185	7.84E-07
TUCP_001412	XLOC_055123	ENSRNOT00000071884	0.974758753	7.73E-08
TUCP_001412	XLOC_055123	ENSRNOT00000004128	0.961796235	6.01E-07
TUCP_001412	XLOC_055123	ENSRNOT00000026316	0.964400224	4.24E-07
TUCP_001412	XLOC_055123	ENSRNOT00000057729	0.999382938	7.04E-16

TUCP_001412	XLOC_055123	ENSRNOT00000078663	0.975229089	7.05E-08
TUCP_001412	XLOC_055123	ENSRNOT00000064250	0.983165167	1.04E-08
TUCP_001412	XLOC_055123	ENSRNOT00000021608	0.965262541	3.76E-07
TUCP_001412	XLOC_055123	ENSRNOT00000029634	0.995812154	1.01E-11
TUCP_001412	XLOC_055123	ENSRNOT00000093579	0.958546239	8.99E-07
TUCP_001412	XLOC_055123	ENSRNOT00000086349	0.957220142	1.05E-06
TUCP_001412	XLOC_055123	ENSRNOT00000065460	0.999999943	4.90E-36
TUCP_001412	XLOC_055123	ENSRNOT00000003817	0.996798605	2.63E-12
TUCP_001412	XLOC_055123	ENSRNOT00000016713	0.999999943	4.90E-36
TUCP_001412	XLOC_055123	ENSRNOT00000079260	0.996442736	4.46E-12
TUCP_001412	XLOC_055123	ENSRNOT00000039294	0.999999943	4.90E-36
TUCP_001412	XLOC_055123	ENSRNOT00000092913	0.9530002	1.67E-06
TUCP_000698	XLOC_026355	ENSRNOT00000080146	0.950555116	2.14E-06
TUCP_000698	XLOC_026355	ENSRNOT00000041541	-0.95847466	9.07E-07
TUCP_001416	XLOC_055294	ENSRNOT00000084076	0.951544085	1.94E-06
TUCP_001416	XLOC_055294	ENSRNOT00000042558	0.955025449	1.34E-06
TUCP_001416	XLOC_055294	ENSRNOT00000001098	0.972466197	1.19E-07
TUCP_001416	XLOC_055294	ENSRNOT00000075400	0.963863583	4.57E-07
TUCP_001416	XLOC_055294	ENSRNOT00000030479	0.952587711	1.74E-06
TUCP_001416	XLOC_055294	ENSRNOT00000029097	0.974344248	8.39E-08
TUCP_001416	XLOC_055294	ENSRNOT00000057758	0.984073466	7.86E-09
TUCP_001416	XLOC_055294	ENSRNOT00000019350	0.977883905	4.02E-08
TUCP_001416	XLOC_055294	ENSRNOT00000035400	0.975488919	6.69E-08
TUCP_001416	XLOC_055294	ENSRNOT00000010932	0.970776309	1.60E-07
TUCP_001416	XLOC_055294	ENSRNOT00000075494	0.963291382	4.94E-07
TUCP_001416	XLOC_055294	ENSRNOT00000023149	0.960818807	6.81E-07
TUCP_001416	XLOC_055294	ENSRNOT00000017204	0.97313436	1.05E-07
TUCP_001416	XLOC_055294	ENSRNOT00000087540	0.95022562	2.21E-06
TUCP_001416	XLOC_055294	ENSRNOT00000010720	0.961487145	6.25E-07
TUCP_001416	XLOC_055294	ENSRNOT00000077268	0.958533854	9.00E-07
TUCP_001416	XLOC_055294	ENSRNOT00000018877	0.952323076	1.79E-06
TUCP_001416	XLOC_055294	ENSRNOT00000004159	0.983105652	1.05E-08
TUCP_001416	XLOC_055294	ENSRNOT00000082108	0.955583269	1.26E-06
TUCP_001416	XLOC_055294	ENSRNOT00000082585	0.953497251	1.58E-06
TUCP_001416	XLOC_055294	ENSRNOT00000013854	0.954988613	1.35E-06
TUCP_001416	XLOC_055294	ENSRNOT00000019056	0.970740871	1.61E-07
TUCP_001416	XLOC_055294	ENSRNOT00000012376	0.951302859	1.99E-06
TUCP_001416	XLOC_055294	ENSRNOT00000027553	0.950491642	2.16E-06
TUCP_001416	XLOC_055294	ENSRNOT00000015473	0.979905966	2.49E-08
TUCP_001416	XLOC_055294	ENSRNOT00000028787	0.951122847	2.02E-06
TUCP_001416	XLOC_055294	ENSRNOT00000016164	0.976482396	5.45E-08
TUCP_001416	XLOC_055294	ENSRNOT00000061735	0.950221399	2.21E-06
TUCP_001416	XLOC_055294	ENSRNOT00000011432	0.966880048	2.97E-07
TUCP_001416	XLOC_055294	ENSRNOT00000014177	0.95952748	7.99E-07
TUCP_001416	XLOC_055294	ENSRNOT00000042864	0.955610588	1.26E-06
TUCP_001416	XLOC_055294	ENSRNOT00000013587	0.965730416	3.51E-07
TUCP_001416	XLOC_055294	ENSRNOT00000008274	0.956567894	1.13E-06
TUCP_001416	XLOC_055294	ENSRNOT00000045041	0.962619437	5.40E-07
TUCP_001416	XLOC_055294	ENSRNOT00000088856	0.950686082	2.11E-06
TUCP_001416	XLOC_055294	ENSRNOT00000003728	0.978704758	3.33E-08
TUCP_001416	XLOC_055294	ENSRNOT00000080846	0.966743667	3.03E-07

TUCP_001416	XLOC_055294	ENSRNOT00000070961	0.964369364	4.26E-07
TUCP_001416	XLOC_055294	ENSRNOT00000049973	0.951180314	2.01E-06
TUCP_001416	XLOC_055294	ENSRNOT00000039709	0.974401658	8.29E-08
TUCP_001416	XLOC_055294	ENSRNOT00000056644	0.970594919	1.65E-07
TUCP_001416	XLOC_055294	ENSRNOT00000012179	0.963615119	4.72E-07
TUCP_001416	XLOC_055294	ENSRNOT00000019044	0.97095813	1.55E-07
TUCP_001416	XLOC_055294	ENSRNOT00000001373	0.969210599	2.07E-07
TUCP_001416	XLOC_055294	ENSRNOT00000022637	0.960415216	7.16E-07
TUCP_001416	XLOC_055294	ENSRNOT00000012495	0.953078326	1.66E-06
TUCP_001416	XLOC_055294	ENSRNOT00000016259	0.961873642	5.95E-07
TUCP_001416	XLOC_055294	ENSRNOT00000009015	0.970739383	1.61E-07
TUCP_001416	XLOC_055294	ENSRNOT00000017268	0.962419685	5.54E-07
TUCP_001416	XLOC_055294	ENSRNOT00000035238	0.950350816	2.19E-06
TUCP_001416	XLOC_055294	ENSRNOT00000024578	0.969327009	2.03E-07
TUCP_001416	XLOC_055294	ENSRNOT00000056079	0.952207505	1.81E-06
TUCP_001416	XLOC_055294	ENSRNOT00000054958	0.980033962	2.42E-08
TUCP_001416	XLOC_055294	ENSRNOT00000001891	0.964298739	4.30E-07
TUCP_001416	XLOC_055294	ENSRNOT00000025506	0.957099165	1.06E-06
TUCP_001416	XLOC_055294	ENSRNOT00000030823	0.962539211	5.46E-07
TUCP_001416	XLOC_055294	ENSRNOT00000024980	0.950639104	2.12E-06
TUCP_001416	XLOC_055294	ENSRNOT00000024981	0.972297442	1.23E-07
TUCP_001416	XLOC_055294	ENSRNOT00000035513	0.956984004	1.08E-06
TUCP_001416	XLOC_055294	ENSRNOT00000031653	0.962042287	5.82E-07
TUCP_001416	XLOC_055294	ENSRNOT00000020543	0.961946177	5.90E-07
TUCP_001416	XLOC_055294	ENSRNOT00000056656	0.965968737	3.40E-07
TUCP_001416	XLOC_055294	ENSRNOT00000013778	0.960417053	7.16E-07
TUCP_001416	XLOC_055294	ENSRNOT00000015918	0.954082037	1.49E-06
TUCP_001416	XLOC_055294	ENSRNOT00000006946	0.96867965	2.25E-07
TUCP_001416	XLOC_055294	ENSRNOT00000077730	0.95302018	1.67E-06
TUCP_001416	XLOC_055294	ENSRNOT00000015759	0.985474344	4.97E-09
TUCP_001416	XLOC_055294	ENSRNOT00000032173	0.958662681	8.87E-07
TUCP_001416	XLOC_055294	ENSRNOT00000050774	0.960196367	7.36E-07
TUCP_001416	XLOC_055294	ENSRNOT00000089313	0.96773576	2.61E-07
TUCP_001416	XLOC_055294	ENSRNOT00000023854	0.969722157	1.90E-07
TUCP_001416	XLOC_055294	ENSRNOT00000024690	0.95839046	9.16E-07
TUCP_001416	XLOC_055294	ENSRNOT00000017955	0.956980123	1.08E-06
TUCP_001416	XLOC_055294	ENSRNOT00000065023	0.954895177	1.36E-06
TUCP_001416	XLOC_055294	ENSRNOT00000047717	0.956773256	1.11E-06
TUCP_001416	XLOC_055294	ENSRNOT00000075057	0.976612611	5.30E-08
TUCP_001416	XLOC_055294	ENSRNOT00000003277	0.974027615	8.91E-08
TUCP_001416	XLOC_055294	ENSRNOT00000083983	0.969726799	1.90E-07
TUCP_001416	XLOC_055294	ENSRNOT00000007549	0.971535776	1.40E-07
TUCP_001416	XLOC_055294	ENSRNOT00000017626	0.951942131	1.86E-06
TUCP_001416	XLOC_055294	ENSRNOT00000037946	0.966639912	3.08E-07
TUCP_001416	XLOC_055294	ENSRNOT00000087359	-0.968533436	2.30E-07
TUCP_001416	XLOC_055294	ENSRNOT00000060683	0.95247457	1.76E-06
TUCP_001416	XLOC_055294	ENSRNOT00000021403	0.955707356	1.25E-06
TUCP_001416	XLOC_055294	ENSRNOT00000075598	0.953826519	1.53E-06
TUCP_001416	XLOC_055294	ENSRNOT00000073926	0.979661455	2.65E-08
TUCP_001416	XLOC_055294	ENSRNOT00000026552	0.958321156	9.23E-07
TUCP_001416	XLOC_055294	ENSRNOT00000001631	0.95873339	8.79E-07

TUCP_001416	XLOC_055294	ENSRNOT00000064851	0.973403483	1.00E-07
TUCP_001416	XLOC_055294	ENSRNOT00000000253	0.959003023	8.51E-07
TUCP_001416	XLOC_055294	ENSRNOT00000091456	0.958365469	9.19E-07
TUCP_001416	XLOC_055294	ENSRNOT00000027512	0.973206763	1.04E-07
TUCP_001416	XLOC_055294	ENSRNOT00000082149	0.950945356	2.06E-06
TUCP_001416	XLOC_055294	ENSRNOT00000027450	0.970627614	1.64E-07
TUCP_001416	XLOC_055294	ENSRNOT00000007577	0.958908284	8.61E-07
TUCP_001416	XLOC_055294	ENSRNOT00000056639	0.9539842	1.50E-06
TUCP_001416	XLOC_055294	ENSRNOT00000004560	0.968130507	2.45E-07
TUCP_001416	XLOC_055294	ENSRNOT00000002511	0.973794463	9.31E-08
TUCP_001416	XLOC_055294	ENSRNOT00000043518	0.956013946	1.20E-06
TUCP_001416	XLOC_055294	ENSRNOT00000020853	0.96589857	3.43E-07
TUCP_001416	XLOC_055294	ENSRNOT00000017339	0.976497206	5.43E-08
TUCP_001416	XLOC_055294	ENSRNOT00000081593	0.987439791	2.41E-09
TUCP_001416	XLOC_055294	ENSRNOT00000021309	0.969578721	1.95E-07
TUCP_001416	XLOC_055294	ENSRNOT00000020707	0.959925439	7.61E-07
TUCP_001416	XLOC_055294	ENSRNOT00000008509	0.954798488	1.38E-06
TUCP_001416	XLOC_055294	ENSRNOT00000050227	0.961738996	6.06E-07
TUCP_001416	XLOC_055294	ENSRNOT00000020809	0.952088678	1.83E-06
TUCP_001416	XLOC_055294	ENSRNOT00000037710	0.964806064	4.01E-07
TUCP_001416	XLOC_055294	ENSRNOT00000028234	0.953513504	1.58E-06
TUCP_001416	XLOC_055294	ENSRNOT00000002198	0.964036839	4.46E-07
TUCP_001416	XLOC_055294	ENSRNOT00000003514	0.98473084	6.37E-09
TUCP_001416	XLOC_055294	ENSRNOT00000076388	0.954824965	1.37E-06
TUCP_001416	XLOC_055294	ENSRNOT00000005155	-0.962671194	5.36E-07
TUCP_001416	XLOC_055294	ENSRNOT00000016262	0.970343515	1.72E-07
TUCP_001416	XLOC_055294	ENSRNOT00000091541	0.9514463	1.96E-06
TUCP_001416	XLOC_055294	ENSRNOT00000027060	0.950208018	2.22E-06
TUCP_001416	XLOC_055294	ENSRNOT00000085690	0.969382235	2.01E-07
TUCP_001416	XLOC_055294	ENSRNOT00000064323	0.961343803	6.37E-07
TUCP_001416	XLOC_055294	ENSRNOT00000017618	0.972993342	1.08E-07
TUCP_001416	XLOC_055294	ENSRNOT00000082963	0.964298252	4.30E-07
TUCP_001416	XLOC_055294	ENSRNOT00000009468	0.973941082	9.06E-08
TUCP_001416	XLOC_055294	ENSRNOT00000014002	0.959133217	8.38E-07
TUCP_001416	XLOC_055294	ENSRNOT00000064924	0.97343497	9.96E-08
TUCP_001416	XLOC_055294	ENSRNOT00000005666	0.962741436	5.31E-07
TUCP_001416	XLOC_055294	ENSRNOT00000002867	0.95039038	2.18E-06
TUCP_001416	XLOC_055294	ENSRNOT00000016856	0.971054794	1.52E-07
TUCP_001416	XLOC_055294	ENSRNOT00000051428	0.97631942	5.64E-08
TUCP_001416	XLOC_055294	ENSRNOT00000068666	0.958064707	9.52E-07
TUCP_001416	XLOC_055294	ENSRNOT00000015130	0.991285241	3.90E-10
TUCP_001416	XLOC_055294	ENSRNOT00000044895	0.952310851	1.79E-06
TUCP_001416	XLOC_055294	ENSRNOT00000013535	0.978781908	3.27E-08
TUCP_001416	XLOC_055294	ENSRNOT00000018043	0.980350834	2.23E-08
TUCP_001416	XLOC_055294	ENSRNOT00000018470	0.953068807	1.66E-06
TUCP_001416	XLOC_055294	ENSRNOT00000066199	0.952635821	1.73E-06
TUCP_001416	XLOC_055294	ENSRNOT00000089016	0.962452555	5.52E-07
TUCP_001416	XLOC_055294	ENSRNOT00000047698	0.985321865	5.24E-09
TUCP_001416	XLOC_055294	ENSRNOT00000011320	0.950188586	2.22E-06
TUCP_001416	XLOC_055294	ENSRNOT00000070916	0.954307335	1.45E-06
TUCP_001416	XLOC_055294	ENSRNOT00000006860	0.957737697	9.89E-07

TUCP_001416	XLOC_055294	ENSRNOT00000007917	0.9611014	6.57E-07
TUCP_001416	XLOC_055294	ENSRNOT00000067905	0.993614079	8.27E-11
TUCP_001416	XLOC_055294	ENSRNOT00000021677	0.957314944	1.04E-06
TUCP_001416	XLOC_055294	ENSRNOT00000065642	0.98248558	1.26E-08
TUCP_001416	XLOC_055294	ENSRNOT00000005286	0.965534753	3.61E-07
TUCP_001416	XLOC_055294	ENSRNOT00000036042	0.951817605	1.89E-06
TUCP_001416	XLOC_055294	ENSRNOT00000073466	-0.950478501	2.16E-06
TUCP_001416	XLOC_055294	ENSRNOT00000017564	0.955953295	1.21E-06
TUCP_001416	XLOC_055294	ENSRNOT00000040981	0.977072154	4.80E-08
TUCP_001416	XLOC_055294	ENSRNOT00000010630	-0.974005316	8.95E-08
TUCP_001416	XLOC_055294	ENSRNOT00000005255	0.960667521	6.94E-07
TUCP_001416	XLOC_055294	ENSRNOT00000008359	0.962112337	5.77E-07
TUCP_001416	XLOC_055294	ENSRNOT00000092641	0.967647236	2.64E-07
TUCP_001417	XLOC_055353	ENSRNOT00000027321	0.954315077	1.45E-06
TUCP_002693	XLOC_114394	ENSRNOT00000000008	1	0
TUCP_002693	XLOC_114394	ENSRNOT00000060048	0.998711457	2.79E-14
TUCP_002693	XLOC_114394	ENSRNOT00000082304	0.996875989	2.33E-12
TUCP_002693	XLOC_114394	ENSRNOT00000006219	1	0
TUCP_002693	XLOC_114394	ENSRNOT00000065376	1	0
TUCP_002693	XLOC_114394	ENSRNOT00000081433	0.984469223	6.93E-09
TUCP_002693	XLOC_114394	ENSRNOT00000083575	1	0
TUCP_002693	XLOC_114394	ENSRNOT00000087739	1	0
TUCP_002693	XLOC_114394	ENSRNOT00000079826	1	0
TUCP_002693	XLOC_114394	ENSRNOT00000025770	0.993828251	6.98E-11
TUCP_002693	XLOC_114394	ENSRNOT00000088244	0.973479481	9.88E-08
TUCP_002693	XLOC_114394	ENSRNOT00000048523	1	0
TUCP_002693	XLOC_114394	ENSRNOT00000079365	0.958936699	8.58E-07
TUCP_002693	XLOC_114394	ENSRNOT00000020229	0.960002785	7.54E-07
TUCP_002693	XLOC_114394	ENSRNOT00000026428	0.996986683	1.95E-12
TUCP_002693	XLOC_114394	ENSRNOT00000085673	1	0
TUCP_002693	XLOC_114394	ENSRNOT00000072972	0.981476166	1.67E-08
TUCP_002693	XLOC_114394	ENSRNOT00000050573	1	0
TUCP_002693	XLOC_114394	ENSRNOT00000089876	-0.953630353	1.56E-06
TUCP_002693	XLOC_114394	ENSRNOT00000074378	0.998901807	1.26E-14
TUCP_002693	XLOC_114394	ENSRNOT00000093571	1	0
TUCP_002693	XLOC_114394	ENSRNOT00000090581	1	0
TUCP_002693	XLOC_114394	ENSRNOT00000076239	0.98020752	2.31E-08
TUCP_002693	XLOC_114394	ENSRNOT00000065485	1	0
TUCP_002693	XLOC_114394	ENSRNOT00000006356	0.999544279	1.55E-16
TUCP_002693	XLOC_114394	ENSRNOT00000087199	0.974421555	8.26E-08
TUCP_002693	XLOC_114394	ENSRNOT00000022091	1	0
TUCP_002693	XLOC_114394	ENSRNOT00000064764	0.98460888	6.63E-09
TUCP_002693	XLOC_114394	ENSRNOT00000076927	0.971163156	1.50E-07
TUCP_002693	XLOC_114394	ENSRNOT00000092395	0.999522978	1.94E-16
TUCP_002692	XLOC_114315	ENSRNOT00000060527	0.97449155	8.15E-08
TUCP_002692	XLOC_114315	ENSRNOT00000044896	0.955967542	1.21E-06
TUCP_002692	XLOC_114315	ENSRNOT00000075659	0.960819549	6.81E-07
TUCP_002692	XLOC_114315	ENSRNOT00000083215	0.950574689	2.14E-06
TUCP_002692	XLOC_114315	ENSRNOT00000072081	0.950143051	2.23E-06
TUCP_002692	XLOC_114315	ENSRNOT00000015546	0.95190289	1.87E-06
TUCP_002692	XLOC_114315	ENSRNOT00000031792	0.954196772	1.47E-06

TUCP_002692	XLOC_114315	ENSRNOT00000080768	0.967453449	2.72E-07
TUCP_002692	XLOC_114315	ENSRNOT00000027561	0.951879452	1.87E-06
TUCP_002692	XLOC_114315	ENSRNOT00000047975	0.95916101	8.35E-07
TUCP_002692	XLOC_114315	ENSRNOT00000083971	0.962643607	5.38E-07
TUCP_002692	XLOC_114315	ENSRNOT00000020147	0.955741704	1.24E-06
TUCP_002692	XLOC_114315	ENSRNOT00000076453	0.958981717	8.54E-07
TUCP_002692	XLOC_114315	ENSRNOT00000034267	0.960578641	7.02E-07
TUCP_002692	XLOC_114315	ENSRNOT00000004032	0.954531158	1.42E-06
TUCP_002692	XLOC_114315	ENSRNOT00000009198	0.960343284	7.23E-07
TUCP_002692	XLOC_114315	ENSRNOT00000021614	0.956167407	1.18E-06
TUCP_002691	XLOC_114226	ENSRNOT00000074583	0.955008497	1.35E-06
TUCP_002691	XLOC_114226	ENSRNOT00000003759	0.975345434	6.88E-08
TUCP_002691	XLOC_114226	ENSRNOT00000018888	0.954432324	1.43E-06
TUCP_002691	XLOC_114226	ENSRNOT00000000995	0.970940074	1.55E-07
TUCP_002691	XLOC_114226	ENSRNOT00000014533	0.970581509	1.65E-07
TUCP_002691	XLOC_114226	ENSRNOT00000089389	0.961287093	6.42E-07
TUCP_002691	XLOC_114226	ENSRNOT00000068354	0.967489162	2.71E-07
TUCP_002691	XLOC_114226	ENSRNOT00000022167	0.98281957	1.15E-08
TUCP_002691	XLOC_114226	ENSRNOT00000003082	0.96975554	1.89E-07
TUCP_002691	XLOC_114226	ENSRNOT00000045993	0.954367496	1.44E-06
TUCP_002691	XLOC_114226	ENSRNOT00000012963	0.964349541	4.27E-07
TUCP_002691	XLOC_114226	ENSRNOT00000057504	0.95834854	9.20E-07
TUCP_002691	XLOC_114226	ENSRNOT00000010968	0.979847727	2.53E-08
TUCP_002691	XLOC_114226	ENSRNOT00000024069	0.963473235	4.82E-07
TUCP_002691	XLOC_114226	ENSRNOT00000009448	0.955474225	1.28E-06
TUCP_002691	XLOC_114226	ENSRNOT00000013182	0.985838879	4.38E-09
TUCP_002691	XLOC_114226	ENSRNOT00000025731	0.954248531	1.46E-06
TUCP_002691	XLOC_114226	ENSRNOT00000042973	0.977752759	4.13E-08
TUCP_002691	XLOC_114226	ENSRNOT00000008813	0.960753258	6.87E-07
TUCP_002691	XLOC_114226	ENSRNOT00000003458	0.975317399	6.92E-08
TUCP_002691	XLOC_114226	ENSRNOT00000001110	0.957451251	1.02E-06
TUCP_002691	XLOC_114226	ENSRNOT00000076879	0.965703565	3.53E-07
TUCP_002691	XLOC_114226	ENSRNOT00000051402	0.953474992	1.59E-06
TUCP_002691	XLOC_114226	ENSRNOT00000002853	0.954143462	1.48E-06
TUCP_002691	XLOC_114226	ENSRNOT00000078299	0.971078675	1.52E-07
TUCP_002691	XLOC_114226	ENSRNOT00000006196	0.982599238	1.22E-08
TUCP_002691	XLOC_114226	ENSRNOT00000013809	0.987306362	2.54E-09
TUCP_002691	XLOC_114226	ENSRNOT00000073087	0.983402806	9.65E-09
TUCP_002691	XLOC_114226	ENSRNOT00000019389	0.96300638	5.13E-07
TUCP_002691	XLOC_114226	ENSRNOT00000021487	0.987520467	2.33E-09
TUCP_002691	XLOC_114226	ENSRNOT00000051028	-0.961565336	6.19E-07
TUCP_002691	XLOC_114226	ENSRNOT00000051067	0.983816313	8.51E-09
TUCP_002691	XLOC_114226	ENSRNOT00000082393	0.960905632	6.73E-07
TUCP_002691	XLOC_114226	ENSRNOT00000021359	0.968413836	2.35E-07
TUCP_002691	XLOC_114226	ENSRNOT00000014560	0.962595345	5.42E-07
TUCP_002691	XLOC_114226	ENSRNOT00000073138	0.964076793	4.44E-07
TUCP_002691	XLOC_114226	ENSRNOT00000077303	0.976671444	5.23E-08
TUCP_002691	XLOC_114226	ENSRNOT00000029845	0.95078679	2.09E-06
TUCP_002691	XLOC_114226	ENSRNOT00000022128	0.996735497	2.90E-12
TUCP_002691	XLOC_114226	ENSRNOT00000013191	0.969465825	1.99E-07
TUCP_002691	XLOC_114226	ENSRNOT00000045107	0.955529816	1.27E-06

TUCP_002691	XLOC_114226	ENSRNOT00000019738	0.955479437	1.28E-06
TUCP_002691	XLOC_114226	ENSRNOT00000014317	0.954826396	1.37E-06
TUCP_002691	XLOC_114226	ENSRNOT00000090774	0.97160083	1.39E-07
TUCP_002690	XLOC_114226	ENSRNOT00000067455	0.955249756	1.31E-06
TUCP_002690	XLOC_114226	ENSRNOT00000040856	0.969104487	2.10E-07
TUCP_002690	XLOC_114226	ENSRNOT00000056174	0.95667168	1.12E-06
TUCP_002690	XLOC_114226	ENSRNOT00000012632	0.953807857	1.53E-06
TUCP_002690	XLOC_114226	ENSRNOT00000024464	0.951300031	1.99E-06
TUCP_002690	XLOC_114226	ENSRNOT00000005085	0.961058269	6.61E-07
TUCP_002690	XLOC_114226	ENSRNOT00000009077	0.950591976	2.13E-06
TUCP_002690	XLOC_114226	ENSRNOT00000010264	0.956982099	1.08E-06
TUCP_002690	XLOC_114226	ENSRNOT00000032333	0.961657006	6.12E-07
TUCP_002690	XLOC_114226	ENSRNOT00000034152	0.954622951	1.40E-06
TUCP_002697	XLOC_114537	ENSRNOT00000040985	0.963621937	4.72E-07
TUCP_002697	XLOC_114537	ENSRNOT00000074608	0.950100433	2.24E-06
TUCP_002696	XLOC_114535	ENSRNOT00000079339	0.967197442	2.83E-07
TUCP_002695	XLOC_114471	ENSRNOT00000009706	0.966496572	3.14E-07
TUCP_002695	XLOC_114471	ENSRNOT00000027640	-0.957653844	9.99E-07
TUCP_002695	XLOC_114471	ENSRNOT00000029980	-0.953752441	1.54E-06
TUCP_002695	XLOC_114471	ENSRNOT00000012837	-0.954486736	1.42E-06
TUCP_002695	XLOC_114471	ENSRNOT00000026416	-0.953308691	1.62E-06
TUCP_002695	XLOC_114471	ENSRNOT00000027704	-0.95110792	2.03E-06
TUCP_002695	XLOC_114471	ENSRNOT00000016581	-0.954973968	1.35E-06
TUCP_002699	XLOC_114767	ENSRNOT00000029764	-0.951111976	2.03E-06
TUCP_002699	XLOC_114767	ENSRNOT00000011794	-0.959144054	8.37E-07
TUCP_002699	XLOC_114767	ENSRNOT00000042100	0.950552222	2.14E-06
TUCP_002699	XLOC_114767	ENSRNOT00000003568	0.953719693	1.55E-06
TUCP_002265	XLOC_094370	ENSRNOT00000074753	0.958764394	8.76E-07
TUCP_002265	XLOC_094370	ENSRNOT00000041904	0.959863002	7.67E-07
TUCP_002265	XLOC_094370	ENSRNOT00000077990	0.959863002	7.67E-07
TUCP_002265	XLOC_094370	ENSRNOT00000038826	0.96142432	6.31E-07
TUCP_002265	XLOC_094370	ENSRNOT00000046842	0.977204653	4.67E-08
TUCP_002265	XLOC_094370	ENSRNOT00000060250	0.960785698	6.84E-07
TUCP_002265	XLOC_094370	ENSRNOT00000091934	0.959863002	7.67E-07
TUCP_002265	XLOC_094370	ENSRNOT00000089929	0.959863002	7.67E-07
TUCP_002265	XLOC_094370	ENSRNOT00000080794	0.962952207	5.16E-07
TUCP_002265	XLOC_094370	ENSRNOT00000044145	0.959863002	7.67E-07
TUCP_002265	XLOC_094370	ENSRNOT00000011743	0.977472046	4.40E-08
TUCP_002265	XLOC_094370	ENSRNOT00000034907	0.953610201	1.56E-06
TUCP_002265	XLOC_094370	ENSRNOT00000088624	0.959863002	7.67E-07
TUCP_002265	XLOC_094370	ENSRNOT00000085659	0.97121893	1.48E-07
TUCP_002265	XLOC_094370	ENSRNOT00000061284	0.964386315	4.25E-07
TUCP_002265	XLOC_094370	ENSRNOT00000051835	0.959863002	7.67E-07
TUCP_002265	XLOC_094370	ENSRNOT00000079087	0.959863002	7.67E-07
TUCP_002265	XLOC_094370	ENSRNOT00000037206	0.959863002	7.67E-07
TUCP_002265	XLOC_094370	ENSRNOT00000083261	0.979310371	2.88E-08
TUCP_002265	XLOC_094370	ENSRNOT00000080590	0.958973002	8.54E-07
TUCP_002265	XLOC_094370	ENSRNOT00000027552	0.961374344	6.35E-07
TUCP_002265	XLOC_094370	ENSRNOT00000077014	0.959670098	7.85E-07
TUCP_002265	XLOC_094370	ENSRNOT00000080453	0.959145682	8.37E-07
TUCP_002265	XLOC_094370	ENSRNOT00000057729	0.957424499	1.03E-06

TUCP_002265	XLOC_094370	ENSRNOT00000078633	0.959863002	7.67E-07
TUCP_002265	XLOC_094370	ENSRNOT00000071884	0.957442555	1.02E-06
TUCP_002265	XLOC_094370	ENSRNOT00000066839	0.966763763	3.02E-07
TUCP_002265	XLOC_094370	ENSRNOT00000064250	0.951357156	1.98E-06
TUCP_002265	XLOC_094370	ENSRNOT00000029634	0.952675377	1.73E-06
TUCP_002265	XLOC_094370	ENSRNOT00000065460	0.959863002	7.67E-07
TUCP_002265	XLOC_094370	ENSRNOT00000003817	0.958464338	9.08E-07
TUCP_002265	XLOC_094370	ENSRNOT00000016713	0.959863002	7.67E-07
TUCP_002265	XLOC_094370	ENSRNOT00000079260	0.96698268	2.92E-07
TUCP_002265	XLOC_094370	ENSRNOT00000039294	0.959863002	7.67E-07
TUCP_003586	XLOC_157608	ENSRNOT00000046914	0.953661554	1.56E-06
TUCP_002117	XLOC_087725	ENSRNOT00000000514	-0.958423802	9.12E-07
TUCP_002110	XLOC_087410	ENSRNOT00000042890	0.963356798	4.89E-07
TUCP_002110	XLOC_087410	ENSRNOT00000012757	0.962969055	5.15E-07
TUCP_002110	XLOC_087410	ENSRNOT00000086144	0.964556257	4.15E-07
TUCP_002110	XLOC_087410	ENSRNOT00000005505	0.957699313	9.93E-07
TUCP_002110	XLOC_087410	ENSRNOT00000056050	0.950510789	2.15E-06
TUCP_002110	XLOC_087410	ENSRNOT00000009975	0.970096989	1.79E-07
TUCP_002110	XLOC_087410	ENSRNOT00000072682	0.95105906	2.04E-06
TUCP_002110	XLOC_087410	ENSRNOT00000055877	0.964867556	3.97E-07
TUCP_002110	XLOC_087410	ENSRNOT00000006109	0.961474512	6.26E-07
TUCP_002110	XLOC_087410	ENSRNOT00000075144	0.959093628	8.42E-07
TUCP_002110	XLOC_087410	ENSRNOT00000081224	0.956164163	1.18E-06
TUCP_002110	XLOC_087410	ENSRNOT00000001642	0.964145089	4.39E-07
TUCP_002110	XLOC_087410	ENSRNOT00000034482	0.975232546	7.04E-08
TUCP_002110	XLOC_087410	ENSRNOT00000043827	0.959443088	8.07E-07
TUCP_002110	XLOC_087410	ENSRNOT00000037375	0.96417214	4.38E-07
TUCP_002111	XLOC_087416	ENSRNOT00000047105	0.974669048	7.87E-08
TUCP_002111	XLOC_087416	ENSRNOT00000031904	0.96293259	5.18E-07
TUCP_002111	XLOC_087416	ENSRNOT00000026941	0.968552407	2.30E-07
TUCP_002111	XLOC_087416	ENSRNOT00000067479	0.952045338	1.84E-06
TUCP_002111	XLOC_087416	ENSRNOT00000006067	0.968877857	2.18E-07
TUCP_002111	XLOC_087416	ENSRNOT00000001855	0.976248092	5.72E-08
TUCP_002111	XLOC_087416	ENSRNOT00000001691	0.966921361	2.95E-07
TUCP_002111	XLOC_087416	ENSRNOT00000086706	0.961808613	6.00E-07
TUCP_002111	XLOC_087416	ENSRNOT00000037699	0.965082274	3.86E-07
TUCP_002111	XLOC_087416	ENSRNOT00000074023	0.95163367	1.92E-06
TUCP_002111	XLOC_087416	ENSRNOT00000016975	0.95391045	1.52E-06
TUCP_002111	XLOC_087416	ENSRNOT00000033017	0.986102576	3.99E-09
TUCP_002111	XLOC_087416	ENSRNOT00000009712	0.964076208	4.44E-07
TUCP_002111	XLOC_087416	ENSRNOT00000028561	0.956164808	1.18E-06
TUCP_002111	XLOC_087416	ENSRNOT00000066370	0.959113277	8.40E-07
TUCP_002111	XLOC_087416	ENSRNOT00000029237	0.950791783	2.09E-06
TUCP_002111	XLOC_087416	ENSRNOT00000043345	0.962398745	5.56E-07
TUCP_002111	XLOC_087416	ENSRNOT00000071381	0.953135056	1.65E-06
TUCP_002111	XLOC_087416	ENSRNOT00000013049	0.957626309	1.00E-06
TUCP_002111	XLOC_087416	ENSRNOT00000072771	0.956481956	1.14E-06
TUCP_002111	XLOC_087416	ENSRNOT00000073370	0.953993422	1.50E-06
TUCP_002111	XLOC_087416	ENSRNOT00000060943	0.976377272	5.57E-08
TUCP_002111	XLOC_087416	ENSRNOT00000013580	0.971007824	1.54E-07
TUCP_002111	XLOC_087416	ENSRNOT00000014664	0.965108166	3.84E-07

TUCP_002111	XLOC_087416	ENSRNOT00000057160	0.955996109	1.21E-06
TUCP_002111	XLOC_087416	ENSRNOT00000045047	0.961778626	6.02E-07
TUCP_002111	XLOC_087416	ENSRNOT00000091319	0.957824288	9.79E-07
TUCP_002111	XLOC_087416	ENSRNOT00000065168	0.959636683	7.88E-07
TUCP_002111	XLOC_087416	ENSRNOT00000024266	0.951123806	2.02E-06
TUCP_002111	XLOC_087416	ENSRNOT00000025075	0.956740775	1.11E-06
TUCP_002111	XLOC_087416	ENSRNOT00000020134	0.959897696	7.64E-07
TUCP_002111	XLOC_087416	ENSRNOT00000080152	0.955237981	1.31E-06
TUCP_002111	XLOC_087416	ENSRNOT00000027232	0.951160858	2.02E-06
TUCP_002111	XLOC_087416	ENSRNOT00000060696	0.96957855	1.95E-07
TUCP_002111	XLOC_087416	ENSRNOT00000034261	0.953515607	1.58E-06
TUCP_002111	XLOC_087416	ENSRNOT00000024402	0.953093442	1.65E-06
TUCP_002111	XLOC_087416	ENSRNOT00000064772	0.950361796	2.18E-06
TUCP_002111	XLOC_087416	ENSRNOT00000010541	0.950717789	2.11E-06
TUCP_002111	XLOC_087416	ENSRNOT00000092741	0.953217417	1.63E-06
TUCP_002111	XLOC_087416	ENSRNOT00000049245	0.955914257	1.22E-06
TUCP_002111	XLOC_087416	ENSRNOT00000026831	0.96822822	2.42E-07
TUCP_002111	XLOC_087416	ENSRNOT00000028266	0.951018326	2.04E-06
TUCP_002111	XLOC_087416	ENSRNOT00000003780	0.969194171	2.07E-07
TUCP_002111	XLOC_087416	ENSRNOT00000011956	0.956979144	1.08E-06
TUCP_002111	XLOC_087416	ENSRNOT00000002403	0.974868465	7.57E-08
TUCP_002111	XLOC_087416	ENSRNOT00000027337	0.964426756	4.23E-07
TUCP_002111	XLOC_087416	ENSRNOT00000086577	0.965929397	3.41E-07
TUCP_002111	XLOC_087416	ENSRNOT00000089524	0.964622924	4.11E-07
TUCP_002111	XLOC_087416	ENSRNOT00000065600	0.968928828	2.16E-07
TUCP_002111	XLOC_087416	ENSRNOT00000033975	0.958385108	9.16E-07
TUCP_002111	XLOC_087416	ENSRNOT00000085621	0.950756172	2.10E-06
TUCP_002111	XLOC_087416	ENSRNOT00000055683	0.96244608	5.52E-07
TUCP_002111	XLOC_087416	ENSRNOT00000045488	0.956044569	1.20E-06
TUCP_002111	XLOC_087416	ENSRNOT00000032843	0.966215481	3.28E-07
TUCP_002111	XLOC_087416	ENSRNOT00000031487	0.95817903	9.39E-07
TUCP_002111	XLOC_087416	ENSRNOT00000000060	0.950599945	2.13E-06
TUCP_002111	XLOC_087416	ENSRNOT00000021158	0.953115013	1.65E-06
TUCP_002111	XLOC_087416	ENSRNOT00000079072	0.968770924	2.22E-07
TUCP_002111	XLOC_087416	ENSRNOT00000020428	0.953466568	1.59E-06
TUCP_002111	XLOC_087416	ENSRNOT00000065615	0.954611596	1.41E-06
TUCP_002111	XLOC_087416	ENSRNOT00000079442	0.955895282	1.22E-06
TUCP_002111	XLOC_087416	ENSRNOT00000000658	0.964347974	4.27E-07
TUCP_002111	XLOC_087416	ENSRNOT00000065501	0.958116268	9.46E-07
TUCP_002111	XLOC_087416	ENSRNOT00000011582	0.951233518	2.00E-06
TUCP_002111	XLOC_087416	ENSRNOT00000083632	-0.950083537	2.24E-06
TUCP_002111	XLOC_087416	ENSRNOT00000066739	0.959744538	7.78E-07
TUCP_002111	XLOC_087416	ENSRNOT00000028057	0.955672237	1.25E-06
TUCP_002111	XLOC_087416	ENSRNOT00000055582	0.964463544	4.20E-07
TUCP_002111	XLOC_087416	ENSRNOT00000086122	0.956150976	1.19E-06
TUCP_002111	XLOC_087416	ENSRNOT00000037790	0.962531432	5.46E-07
TUCP_002111	XLOC_087416	ENSRNOT00000088054	0.957796677	9.82E-07
TUCP_002111	XLOC_087416	ENSRNOT00000006802	0.953388433	1.60E-06
TUCP_002111	XLOC_087416	ENSRNOT00000038647	0.965304079	3.74E-07
TUCP_002111	XLOC_087416	ENSRNOT00000010853	0.952328319	1.79E-06
TUCP_002111	XLOC_087416	ENSRNOT00000024224	0.961552722	6.20E-07

TUCP_002111	XLOC_087416	ENSRNOT00000038807	0.965255232	3.76E-07
TUCP_002111	XLOC_087416	ENSRNOT00000085619	0.952845069	1.70E-06
TUCP_002111	XLOC_087416	ENSRNOT00000023302	0.965125064	3.83E-07
TUCP_002111	XLOC_087416	ENSRNOT00000066024	0.95762845	1.00E-06
TUCP_002111	XLOC_087416	ENSRNOT00000012736	0.973086266	1.06E-07
TUCP_002111	XLOC_087416	ENSRNOT00000022716	0.961948446	5.89E-07
TUCP_002111	XLOC_087416	ENSRNOT00000080443	0.955804652	1.23E-06
TUCP_002111	XLOC_087416	ENSRNOT00000015624	0.951928182	1.86E-06
TUCP_002111	XLOC_087416	ENSRNOT00000026426	0.967564355	2.68E-07
TUCP_002111	XLOC_087416	ENSRNOT00000082805	0.958959457	8.56E-07
TUCP_002111	XLOC_087416	ENSRNOT00000078306	0.951243217	2.00E-06
TUCP_002111	XLOC_087416	ENSRNOT00000039341	0.956642683	1.12E-06
TUCP_002111	XLOC_087416	ENSRNOT00000065076	0.954910227	1.36E-06
TUCP_002111	XLOC_087416	ENSRNOT00000007369	0.962373183	5.58E-07
TUCP_002111	XLOC_087416	ENSRNOT00000026280	0.950756735	2.10E-06
TUCP_002111	XLOC_087416	ENSRNOT00000005387	0.961691767	6.09E-07
TUCP_002111	XLOC_087416	ENSRNOT00000005381	0.960246884	7.31E-07
TUCP_002111	XLOC_087416	ENSRNOT00000088279	0.969501971	1.97E-07
TUCP_002111	XLOC_087416	ENSRNOT00000065229	0.957003669	1.08E-06
TUCP_002111	XLOC_087416	ENSRNOT00000090057	0.958561358	8.98E-07
TUCP_002111	XLOC_087416	ENSRNOT00000041494	0.964114526	4.41E-07
TUCP_002111	XLOC_087416	ENSRNOT00000037800	0.950630279	2.13E-06
TUCP_002111	XLOC_087416	ENSRNOT00000021286	0.953043845	1.66E-06
TUCP_002111	XLOC_087416	ENSRNOT00000022796	0.97323884	1.03E-07
TUCP_002111	XLOC_087416	ENSRNOT00000015799	0.955777865	1.24E-06
TUCP_002112	XLOC_087561	ENSRNOT00000022092	0.965612867	3.57E-07
TUCP_002112	XLOC_087561	ENSRNOT00000027793	0.957450161	1.02E-06
TUCP_002112	XLOC_087561	ENSRNOT00000016654	0.958401587	9.15E-07
TUCP_002882	XLOC_123039	ENSRNOT00000043054	0.975797709	6.28E-08
TUCP_000331	XLOC_010523	ENSRNOT00000084500	0.984775744	6.28E-09
TUCP_000331	XLOC_010523	ENSRNOT00000093467	0.995918755	8.86E-12
TUCP_000331	XLOC_010523	ENSRNOT00000029340	0.999157673	3.33E-15
TUCP_000331	XLOC_010523	ENSRNOT00000077972	0.96221111	5.70E-07
TUCP_000331	XLOC_010523	ENSRNOT00000038646	0.999984574	6.88E-24
TUCP_000331	XLOC_010523	ENSRNOT00000012095	0.957685595	9.95E-07
TUCP_000331	XLOC_010523	ENSRNOT00000017909	0.975450158	6.74E-08
TUCP_000331	XLOC_010523	ENSRNOT00000092579	0.999984574	6.88E-24
TUCP_000331	XLOC_010523	ENSRNOT00000089235	0.998770284	2.21E-14
TUCP_000331	XLOC_010523	ENSRNOT00000036383	0.988473506	1.57E-09
TUCP_000331	XLOC_010523	ENSRNOT00000076528	0.997038475	1.79E-12
TUCP_000331	XLOC_010523	ENSRNOT00000075193	0.963919961	4.53E-07
TUCP_000331	XLOC_010523	ENSRNOT00000083034	0.990751333	5.25E-10
TUCP_000331	XLOC_010523	ENSRNOT00000089607	0.994983241	2.48E-11
TUCP_000331	XLOC_010523	ENSRNOT00000086014	0.961507357	6.24E-07
TUCP_000331	XLOC_010523	ENSRNOT00000054939	0.957832975	9.78E-07
TUCP_000331	XLOC_010523	ENSRNOT00000052315	0.983571988	9.17E-09
TUCP_000331	XLOC_010523	ENSRNOT00000088168	0.999984574	6.88E-24
TUCP_000331	XLOC_010523	ENSRNOT00000046231	0.962088619	5.79E-07
TUCP_000331	XLOC_010523	ENSRNOT00000013459	0.97896994	3.13E-08
TUCP_000331	XLOC_010523	ENSRNOT00000078137	0.999984574	6.88E-24
TUCP_000331	XLOC_010523	ENSRNOT00000077764	0.976773262	5.12E-08

TUCP_000331	XLOC_010523	ENSRNOT00000043661	0.966794035	3.01E-07
TUCP_000331	XLOC_010523	ENSRNOT00000092740	0.999984574	6.88E-24
TUCP_000331	XLOC_010523	ENSRNOT00000000117	0.990899488	4.84E-10
TUCP_000331	XLOC_010523	ENSRNOT00000071877	0.966880732	2.97E-07
TUCP_000331	XLOC_010523	ENSRNOT00000070958	0.999984574	6.88E-24
TUCP_000331	XLOC_010523	ENSRNOT00000076757	0.958902378	8.62E-07
TUCP_000331	XLOC_010523	ENSRNOT00000085310	0.965072964	3.86E-07
TUCP_000331	XLOC_010523	ENSRNOT00000047241	0.966419677	3.18E-07
TUCP_000331	XLOC_010523	ENSRNOT00000027268	0.999984574	6.88E-24
TUCP_000331	XLOC_010523	ENSRNOT00000004459	0.999984574	6.88E-24
TUCP_000331	XLOC_010523	ENSRNOT00000048373	0.959176632	8.34E-07
TUCP_000331	XLOC_010523	ENSRNOT00000061516	0.987780469	2.10E-09
TUCP_000331	XLOC_010523	ENSRNOT00000088148	0.999984574	6.88E-24
TUCP_000331	XLOC_010523	ENSRNOT00000088527	0.999984574	6.88E-24
TUCP_000331	XLOC_010523	ENSRNOT00000078701	0.999984574	6.88E-24
TUCP_000331	XLOC_010523	ENSRNOT00000052308	0.962480881	5.50E-07
TUCP_000331	XLOC_010523	ENSRNOT00000021748	0.999984574	6.88E-24
TUCP_000331	XLOC_010523	ENSRNOT00000089085	0.999984574	6.88E-24
TUCP_000331	XLOC_010523	ENSRNOT00000008153	0.999673797	2.91E-17
TUCP_000331	XLOC_010523	ENSRNOT00000085896	0.97141463	1.43E-07
TUCP_000331	XLOC_010523	ENSRNOT00000079189	0.985328329	5.22E-09
TUCP_000331	XLOC_010523	ENSRNOT00000090078	0.999984574	6.88E-24
TUCP_000331	XLOC_010523	ENSRNOT00000078020	0.958284003	9.28E-07
TUCP_000331	XLOC_010523	ENSRNOT00000092425	0.955834879	1.23E-06
TUCP_000331	XLOC_010523	ENSRNOT00000009776	0.999984574	6.88E-24
TUCP_003588	XLOC_157663	ENSRNOT00000089407	0.963198749	5.00E-07
TUCP_003588	XLOC_157663	ENSRNOT00000014268	0.992875249	1.43E-10
TUCP_003588	XLOC_157663	ENSRNOT00000011801	0.978616567	3.40E-08
TUCP_003588	XLOC_157663	ENSRNOT00000080155	0.956443286	1.15E-06
TUCP_003588	XLOC_157663	ENSRNOT00000093356	0.987675017	2.19E-09
TUCP_002119	XLOC_087870	ENSRNOT00000079080	-0.954266983	1.46E-06
TUCP_002053	XLOC_083635	ENSRNOT00000084075	0.994525844	3.84E-11
TUCP_002053	XLOC_083635	ENSRNOT00000012880	0.965617426	3.57E-07
TUCP_002053	XLOC_083635	ENSRNOT00000074465	0.998890628	1.32E-14
TUCP_002053	XLOC_083635	ENSRNOT00000090898	0.960907517	6.73E-07
TUCP_002053	XLOC_083635	ENSRNOT00000079805	0.995967732	8.34E-12
TUCP_002053	XLOC_083635	ENSRNOT00000040912	0.998890628	1.32E-14
TUCP_002053	XLOC_083635	ENSRNOT00000072648	0.998890628	1.32E-14
TUCP_002053	XLOC_083635	ENSRNOT00000007398	0.987100332	2.75E-09
TUCP_002053	XLOC_083635	ENSRNOT00000070803	0.998890628	1.32E-14
TUCP_002053	XLOC_083635	ENSRNOT00000012196	0.998890628	1.32E-14
TUCP_002053	XLOC_083635	ENSRNOT00000085772	0.998890628	1.32E-14
TUCP_002053	XLOC_083635	ENSRNOT00000093284	0.998829054	1.73E-14
TUCP_002053	XLOC_083635	ENSRNOT00000086374	0.990297825	6.66E-10
TUCP_002053	XLOC_083635	ENSRNOT00000015270	0.998890628	1.32E-14
TUCP_002053	XLOC_083635	ENSRNOT00000046192	0.998890628	1.32E-14
TUCP_002053	XLOC_083635	ENSRNOT00000044226	0.998890628	1.32E-14
TUCP_002053	XLOC_083635	ENSRNOT00000081898	0.991660892	3.13E-10
TUCP_002053	XLOC_083635	ENSRNOT00000081954	0.960848792	6.78E-07
TUCP_002053	XLOC_083635	ENSRNOT00000080291	0.997147072	1.48E-12
TUCP_002053	XLOC_083635	ENSRNOT00000004293	0.982686547	1.19E-08

TUCP_002053	XLOC_083635	ENSRNOT00000078231	0.955949666	1.21E-06
TUCP_002053	XLOC_083635	ENSRNOT00000015870	0.962212662	5.69E-07
TUCP_002053	XLOC_083635	ENSRNOT00000001464	0.998890628	1.32E-14
TUCP_002053	XLOC_083635	ENSRNOT00000090716	0.991348794	3.76E-10
TUCP_002053	XLOC_083635	ENSRNOT00000083542	0.970165851	1.77E-07
TUCP_002053	XLOC_083635	ENSRNOT00000076044	0.998890628	1.32E-14
TUCP_002053	XLOC_083635	ENSRNOT00000074353	0.998890628	1.32E-14
TUCP_002053	XLOC_083635	ENSRNOT00000043510	0.954856316	1.37E-06
TUCP_002053	XLOC_083635	ENSRNOT00000087122	0.976050513	5.96E-08
TUCP_002053	XLOC_083635	ENSRNOT00000074295	0.985280077	5.31E-09
TUCP_002053	XLOC_083635	ENSRNOT00000057873	0.998890628	1.32E-14
TUCP_002053	XLOC_083635	ENSRNOT00000083002	0.987536564	2.32E-09
TUCP_002053	XLOC_083635	ENSRNOT00000088399	0.998907233	1.22E-14
TUCP_002053	XLOC_083635	ENSRNOT00000086248	0.998890628	1.32E-14
TUCP_002053	XLOC_083635	ENSRNOT00000089750	0.998843656	1.62E-14
TUCP_002053	XLOC_083635	ENSRNOT00000030517	0.969687933	1.92E-07
TUCP_002053	XLOC_083635	ENSRNOT00000034772	0.993093691	1.22E-10
TUCP_002053	XLOC_083635	ENSRNOT00000089305	0.963527281	4.78E-07
TUCP_002053	XLOC_083635	ENSRNOT00000064601	0.9642863	4.31E-07
TUCP_002053	XLOC_083635	ENSRNOT00000002834	0.998890628	1.32E-14
TUCP_002053	XLOC_083635	ENSRNOT00000080812	0.990836271	5.01E-10
TUCP_002053	XLOC_083635	ENSRNOT00000092755	0.998890628	1.32E-14
TUCP_002053	XLOC_083635	ENSRNOT00000031683	0.998890628	1.32E-14
TUCP_002053	XLOC_083635	ENSRNOT00000087764	0.986561558	3.37E-09
TUCP_002052	XLOC_083635	ENSRNOT00000084944	0.96900129	2.14E-07
TUCP_002052	XLOC_083635	ENSRNOT00000076072	0.985792727	4.45E-09
TUCP_001483	XLOC_057566	ENSRNOT00000026141	0.961555956	6.20E-07
TUCP_001483	XLOC_057566	ENSRNOT00000037597	0.963557378	4.76E-07
TUCP_001483	XLOC_057566	ENSRNOT00000075708	0.950190205	2.22E-06
TUCP_001483	XLOC_057566	ENSRNOT00000003391	0.960323363	7.24E-07
TUCP_001482	XLOC_057502	ENSRNOT00000013233	-0.9567742	1.11E-06
TUCP_001480	XLOC_057201	ENSRNOT00000079236	0.978645009	3.37E-08
TUCP_001480	XLOC_057201	ENSRNOT00000020603	0.956706861	1.11E-06
TUCP_001480	XLOC_057201	ENSRNOT00000093641	0.952876344	1.69E-06
TUCP_001487	XLOC_057671	ENSRNOT00000081433	0.953922063	1.51E-06
TUCP_001487	XLOC_057671	ENSRNOT00000091911	0.956308329	1.17E-06
TUCP_001486	XLOC_057671	ENSRNOT00000093462	0.962533953	5.46E-07
TUCP_004613	XLOC_203501	ENSRNOT00000085911	0.953035205	1.66E-06
TUCP_004613	XLOC_203501	ENSRNOT00000072077	0.956934153	1.09E-06
TUCP_004613	XLOC_203501	ENSRNOT00000087346	-0.967862519	2.56E-07
TUCP_004613	XLOC_203501	ENSRNOT00000008301	0.95716988	1.06E-06
TUCP_004613	XLOC_203501	ENSRNOT00000089086	0.961118437	6.56E-07
TUCP_004613	XLOC_203501	ENSRNOT00000054932	0.952056492	1.84E-06
TUCP_004613	XLOC_203501	ENSRNOT00000058111	0.953597357	1.57E-06
TUCP_004613	XLOC_203501	ENSRNOT00000028074	0.955196274	1.32E-06
TUCP_004613	XLOC_203501	ENSRNOT00000010783	0.955952689	1.21E-06
TUCP_004613	XLOC_203501	ENSRNOT00000047126	0.967865055	2.56E-07
TUCP_004613	XLOC_203501	ENSRNOT00000090268	0.960552269	7.04E-07
TUCP_004613	XLOC_203501	ENSRNOT00000090816	0.958524014	9.02E-07
TUCP_004613	XLOC_203501	ENSRNOT00000015845	-0.952762518	1.71E-06
TUCP_004613	XLOC_203501	ENSRNOT00000083967	-0.955881926	1.22E-06

TUCP_004613	XLOC_203501	ENSRNOT00000075206	-0.960514038	7.07E-07
TUCP_003253	XLOC_139349	ENSRNOT00000049798	-0.952181926	1.82E-06
TUCP_003253	XLOC_139349	ENSRNOT00000050558	-0.953929481	1.51E-06
TUCP_003253	XLOC_139349	ENSRNOT00000088919	-0.957326402	1.04E-06
TUCP_003253	XLOC_139349	ENSRNOT00000010594	0.951859707	1.88E-06
TUCP_003253	XLOC_139349	ENSRNOT00000035123	0.979468157	2.78E-08
TUCP_003253	XLOC_139349	ENSRNOT00000019501	0.951247378	2.00E-06
TUCP_003253	XLOC_139349	ENSRNOT00000088712	0.973745612	9.40E-08
TUCP_003253	XLOC_139349	ENSRNOT00000019195	0.951477544	1.95E-06
TUCP_003253	XLOC_139349	ENSRNOT00000014603	0.955305201	1.30E-06
TUCP_003253	XLOC_139349	ENSRNOT00000041033	0.972085818	1.27E-07
TUCP_003253	XLOC_139349	ENSRNOT00000088355	0.956664066	1.12E-06
TUCP_004343	XLOC_191731	ENSRNOT00000025467	0.950000791	2.26E-06
TUCP_004343	XLOC_191731	ENSRNOT00000001215	-0.959173159	8.34E-07
TUCP_004343	XLOC_191731	ENSRNOT00000036092	-0.982324982	1.32E-08
TUCP_004343	XLOC_191731	ENSRNOT00000010835	-0.957294413	1.04E-06
TUCP_004343	XLOC_191731	ENSRNOT00000081123	-0.952793837	1.71E-06
TUCP_004343	XLOC_191731	ENSRNOT00000064358	-0.96244175	5.53E-07
TUCP_004343	XLOC_191731	ENSRNOT00000011032	-0.955506576	1.27E-06
TUCP_004343	XLOC_191731	ENSRNOT00000004290	-0.95066109	2.12E-06
TUCP_004343	XLOC_191731	ENSRNOT00000084031	-0.963625128	4.72E-07
TUCP_004343	XLOC_191731	ENSRNOT00000073588	-0.958024118	9.56E-07
TUCP_004343	XLOC_191731	ENSRNOT00000080387	-0.956964031	1.08E-06
TUCP_004343	XLOC_191731	ENSRNOT00000091282	0.950457874	2.16E-06
TUCP_004343	XLOC_191731	ENSRNOT00000002443	-0.953675975	1.55E-06
TUCP_004343	XLOC_191731	ENSRNOT00000056022	-0.960012004	7.53E-07
TUCP_001806	XLOC_074849	ENSRNOT00000086868	0.96133437	6.38E-07
TUCP_001806	XLOC_074849	ENSRNOT00000004878	-0.950059088	2.25E-06
TUCP_001806	XLOC_074849	ENSRNOT00000013050	-0.953481448	1.59E-06
TUCP_001806	XLOC_074849	ENSRNOT00000020648	0.957246488	1.05E-06
TUCP_001806	XLOC_074849	ENSRNOT00000020885	0.95073098	2.10E-06
TUCP_001806	XLOC_074849	ENSRNOT00000010765	0.964972936	3.92E-07
TUCP_001806	XLOC_074849	ENSRNOT00000046058	0.950431069	2.17E-06
TUCP_001806	XLOC_074849	ENSRNOT00000021176	-0.955747872	1.24E-06
TUCP_001806	XLOC_074849	ENSRNOT00000077701	0.969726763	1.90E-07
TUCP_001806	XLOC_074849	ENSRNOT00000064774	0.952251162	1.80E-06
TUCP_001806	XLOC_074849	ENSRNOT00000003472	-0.950289832	2.20E-06
TUCP_001806	XLOC_074849	ENSRNOT00000068745	0.963421568	4.85E-07
TUCP_001806	XLOC_074849	ENSRNOT00000075452	0.952863888	1.69E-06
TUCP_001806	XLOC_074849	ENSRNOT00000034400	-0.953414242	1.60E-06
TUCP_001806	XLOC_074849	ENSRNOT00000018475	-0.953441452	1.59E-06
TUCP_001806	XLOC_074849	ENSRNOT00000089859	0.965515945	3.62E-07
TUCP_001806	XLOC_074849	ENSRNOT00000005389	0.956196361	1.18E-06
TUCP_001806	XLOC_074849	ENSRNOT00000014323	0.955869697	1.22E-06
TUCP_001806	XLOC_074849	ENSRNOT00000002353	0.961444792	6.29E-07
TUCP_001806	XLOC_074849	ENSRNOT00000024428	0.961699407	6.09E-07
TUCP_001806	XLOC_074849	ENSRNOT00000036209	0.950979334	2.05E-06
TUCP_001806	XLOC_074849	ENSRNOT00000072922	0.962785261	5.28E-07
TUCP_001806	XLOC_074849	ENSRNOT00000036061	0.963755561	4.63E-07
TUCP_001806	XLOC_074849	ENSRNOT00000088509	0.96676509	3.02E-07
TUCP_001806	XLOC_074849	ENSRNOT00000007091	0.952650297	1.73E-06

TUCP_001806	XLOC_074849	ENSRNOT00000031891	-0.954386735	1.44E-06
TUCP_001806	XLOC_074849	ENSRNOT00000009221	0.960320351	7.25E-07
TUCP_001806	XLOC_074849	ENSRNOT00000079593	0.96670233	3.05E-07
TUCP_001806	XLOC_074849	ENSRNOT00000088191	0.963857756	4.57E-07
TUCP_001431	XLOC_055750	ENSRNOT00000092024	-0.952988833	1.67E-06
TUCP_001431	XLOC_055750	ENSRNOT00000055711	-0.950214223	2.22E-06
TUCP_000900	XLOC_033439	ENSRNOT00000045678	0.950140609	2.23E-06
TUCP_003638	XLOC_159677	ENSRNOT00000089370	0.950887982	2.07E-06
TUCP_003638	XLOC_159677	ENSRNOT00000032613	0.955634716	1.26E-06
TUCP_003638	XLOC_159677	ENSRNOT00000031656	0.959114743	8.40E-07
TUCP_003638	XLOC_159677	ENSRNOT00000009497	0.958792023	8.73E-07
TUCP_003636	XLOC_159561	ENSRNOT00000033670	0.95876149	8.76E-07
TUCP_004616	XLOC_203544	ENSRNOT00000032944	0.952326397	1.79E-06
TUCP_004616	XLOC_203544	ENSRNOT00000006426	0.959642367	7.88E-07
TUCP_004616	XLOC_203544	ENSRNOT00000083215	0.953035111	1.66E-06
TUCP_004616	XLOC_203544	ENSRNOT00000067761	0.953345825	1.61E-06
TUCP_004616	XLOC_203544	ENSRNOT00000076343	0.958895542	8.62E-07
TUCP_004616	XLOC_203544	ENSRNOT00000004836	0.953779008	1.54E-06
TUCP_004616	XLOC_203544	ENSRNOT00000005521	0.96100073	6.65E-07
TUCP_004616	XLOC_203544	ENSRNOT00000015203	0.956936459	1.08E-06
TUCP_004616	XLOC_203544	ENSRNOT00000059458	0.957044458	1.07E-06
TUCP_004616	XLOC_203544	ENSRNOT00000021614	0.978080969	3.84E-08
TUCP_004616	XLOC_203544	ENSRNOT00000004508	0.950678539	2.12E-06
TUCP_004616	XLOC_203544	ENSRNOT00000009198	0.956903081	1.09E-06
TUCP_004616	XLOC_203544	ENSRNOT00000021116	0.966725722	3.04E-07
TUCP_004616	XLOC_203544	ENSRNOT00000031792	0.963363804	4.89E-07
TUCP_004616	XLOC_203544	ENSRNOT00000004032	0.951654405	1.92E-06
TUCP_004616	XLOC_203544	ENSRNOT00000044896	0.979164989	2.99E-08
TUCP_004616	XLOC_203544	ENSRNOT00000022800	0.955645566	1.25E-06
TUCP_004616	XLOC_203544	ENSRNOT00000015073	0.956549499	1.13E-06
TUCP_004616	XLOC_203544	ENSRNOT00000043156	0.957168251	1.06E-06
TUCP_004616	XLOC_203544	ENSRNOT00000085332	-0.961336745	6.38E-07
TUCP_004616	XLOC_203544	ENSRNOT00000027964	0.964354082	4.27E-07
TUCP_004616	XLOC_203544	ENSRNOT00000017658	0.955102479	1.33E-06
TUCP_004616	XLOC_203544	ENSRNOT00000090828	0.95119304	2.01E-06
TUCP_004616	XLOC_203544	ENSRNOT00000006828	0.958653445	8.88E-07
TUCP_004616	XLOC_203544	ENSRNOT00000009877	0.95420879	1.47E-06
TUCP_004616	XLOC_203544	ENSRNOT00000025092	0.956574602	1.13E-06
TUCP_004616	XLOC_203544	ENSRNOT00000080465	0.95413526	1.48E-06
TUCP_004616	XLOC_203544	ENSRNOT00000072081	0.9821121	1.40E-08
TUCP_004616	XLOC_203544	ENSRNOT00000073879	0.962520053	5.47E-07
TUCP_004616	XLOC_203544	ENSRNOT00000083971	0.960739207	6.88E-07
TUCP_004616	XLOC_203544	ENSRNOT00000076453	0.950324551	2.19E-06
TUCP_000909	XLOC_033903	ENSRNOT00000023425	0.952359132	1.78E-06
TUCP_000909	XLOC_033903	ENSRNOT00000012690	0.96777271	2.59E-07
TUCP_003631	XLOC_159485	ENSRNOT00000074472	0.953380914	1.60E-06
TUCP_003631	XLOC_159485	ENSRNOT00000087712	0.95987641	7.66E-07
TUCP_003631	XLOC_159485	ENSRNOT00000009650	0.979113006	3.02E-08
TUCP_003735	XLOC_163482	ENSRNOT00000029842	0.984930289	5.97E-09
TUCP_003735	XLOC_163482	ENSRNOT00000086400	0.965438846	3.66E-07
TUCP_003735	XLOC_163482	ENSRNOT00000056554	0.954064731	1.49E-06

TUCP_003735	XLOC_163482	ENSRNOT00000010216	0.953178426	1.64E-06
TUCP_003734	XLOC_163326	ENSRNOT00000031901	-0.960614356	6.99E-07
TUCP_003734	XLOC_163326	ENSRNOT00000004279	0.953861561	1.52E-06
TUCP_003734	XLOC_163326	ENSRNOT00000009542	0.973388942	1.01E-07
TUCP_003734	XLOC_163326	ENSRNOT00000090896	0.96061894	6.98E-07
TUCP_003732	XLOC_163166	ENSRNOT00000022892	0.954806716	1.38E-06
TUCP_003732	XLOC_163166	ENSRNOT00000003965	0.971141018	1.50E-07
TUCP_003732	XLOC_163166	ENSRNOT00000025598	0.954231795	1.46E-06
TUCP_004593	XLOC_201768	ENSRNOT00000065168	0.961631818	6.14E-07
TUCP_004593	XLOC_201768	ENSRNOT00000031904	0.965077487	3.86E-07
TUCP_004593	XLOC_201768	ENSRNOT00000038647	0.958994519	8.52E-07
TUCP_004593	XLOC_201768	ENSRNOT00000076669	0.950442572	2.17E-06
TUCP_004593	XLOC_201768	ENSRNOT00000020134	0.964350299	4.27E-07
TUCP_004593	XLOC_201768	ENSRNOT00000081512	0.954391607	1.44E-06
TUCP_004593	XLOC_201768	ENSRNOT00000023302	0.96490736	3.95E-07
TUCP_004593	XLOC_201768	ENSRNOT00000057160	0.953245247	1.63E-06
TUCP_004593	XLOC_201768	ENSRNOT00000066024	0.967084572	2.88E-07
TUCP_004593	XLOC_201768	ENSRNOT00000006175	0.979431008	2.80E-08
TUCP_004593	XLOC_201768	ENSRNOT00000041044	0.960026274	7.52E-07
TUCP_004593	XLOC_201768	ENSRNOT00000049582	0.950041348	2.25E-06
TUCP_004593	XLOC_201768	ENSRNOT00000076729	0.951272922	1.99E-06
TUCP_004593	XLOC_201768	ENSRNOT00000090665	0.956088528	1.19E-06
TUCP_004593	XLOC_201768	ENSRNOT00000016975	0.970953349	1.55E-07
TUCP_004593	XLOC_201768	ENSRNOT00000027411	0.985216694	5.42E-09
TUCP_004593	XLOC_201768	ENSRNOT00000033017	0.958279438	9.28E-07
TUCP_004593	XLOC_201768	ENSRNOT00000060696	0.960650429	6.95E-07
TUCP_004593	XLOC_201768	ENSRNOT00000026543	0.966982294	2.92E-07
TUCP_004593	XLOC_201768	ENSRNOT00000012048	0.964284916	4.31E-07
TUCP_004593	XLOC_201768	ENSRNOT00000060499	0.960497297	7.09E-07
TUCP_004593	XLOC_201768	ENSRNOT00000067400	0.954767386	1.38E-06
TUCP_004593	XLOC_201768	ENSRNOT00000045882	0.951219444	2.00E-06
TUCP_004593	XLOC_201768	ENSRNOT00000060943	0.959170817	8.34E-07
TUCP_004593	XLOC_201768	ENSRNOT00000027337	0.960243909	7.32E-07
TUCP_004593	XLOC_201768	ENSRNOT00000025075	0.956590442	1.13E-06
TUCP_004593	XLOC_201768	ENSRNOT00000067866	0.962507863	5.48E-07
TUCP_004593	XLOC_201768	ENSRNOT00000037800	0.950517143	2.15E-06
TUCP_004593	XLOC_201768	ENSRNOT00000015799	0.951941533	1.86E-06
TUCP_000169	XLOC_005324	ENSRNOT00000092026	0.96620614	3.28E-07
TUCP_000169	XLOC_005324	ENSRNOT00000093075	1	0
TUCP_000169	XLOC_005324	ENSRNOT00000058260	1	0
TUCP_000169	XLOC_005324	ENSRNOT00000088887	1	0
TUCP_000169	XLOC_005324	ENSRNOT00000084986	1	0
TUCP_000169	XLOC_005324	ENSRNOT00000074346	0.9702256	1.75E-07
TUCP_000169	XLOC_005324	ENSRNOT00000073209	0.983711602	8.79E-09
TUCP_000169	XLOC_005324	ENSRNOT00000080155	0.973069673	1.07E-07
TUCP_000169	XLOC_005324	ENSRNOT00000072573	0.996213272	6.09E-12
TUCP_000169	XLOC_005324	ENSRNOT00000017828	0.996001583	7.99E-12
TUCP_000169	XLOC_005324	ENSRNOT00000080444	0.969625907	1.93E-07
TUCP_000169	XLOC_005324	ENSRNOT00000071312	1	0
TUCP_000169	XLOC_005324	ENSRNOT00000088245	1	0
TUCP_000169	XLOC_005324	ENSRNOT00000036565	0.969065335	2.12E-07

TUCP_000169	XLOC_005324	ENSRNOT00000058833	0.998673261	3.23E-14
TUCP_000169	XLOC_005324	ENSRNOT00000078052	1	0
TUCP_000169	XLOC_005324	ENSRNOT00000078494	0.999927093	1.62E-20
TUCP_000169	XLOC_005324	ENSRNOT00000085653	1	0
TUCP_000169	XLOC_005324	ENSRNOT00000057950	0.969329371	2.03E-07
TUCP_000169	XLOC_005324	ENSRNOT00000081992	0.999320526	1.14E-15
TUCP_000169	XLOC_005324	ENSRNOT00000038553	0.955765808	1.24E-06
TUCP_000169	XLOC_005324	ENSRNOT00000084120	0.958115447	9.46E-07
TUCP_000169	XLOC_005324	ENSRNOT00000055406	1	0
TUCP_000169	XLOC_005324	ENSRNOT00000070817	0.998968079	9.20E-15
TUCP_000169	XLOC_005324	ENSRNOT00000026753	1	1.33E-79
TUCP_000169	XLOC_005324	ENSRNOT00000080533	0.996682529	3.15E-12
TUCP_000169	XLOC_005324	ENSRNOT00000072774	0.979665261	2.65E-08
TUCP_000169	XLOC_005324	ENSRNOT00000086031	1	0
TUCP_000169	XLOC_005324	ENSRNOT00000014312	1	0
TUCP_000169	XLOC_005324	ENSRNOT00000042882	0.963218427	4.98E-07
TUCP_000169	XLOC_005324	ENSRNOT00000088012	0.956765806	1.11E-06
TUCP_000169	XLOC_005324	ENSRNOT00000051231	0.999242378	1.96E-15
TUCP_000169	XLOC_005324	ENSRNOT00000089407	0.992986194	1.32E-10
TUCP_000169	XLOC_005324	ENSRNOT00000055034	0.998331441	1.02E-13
TUCP_000169	XLOC_005324	ENSRNOT00000046837	0.97252559	1.18E-07
TUCP_002604	XLOC_109701	ENSRNOT00000084500	0.984887141	6.05E-09
TUCP_002604	XLOC_109701	ENSRNOT00000093467	0.996134431	6.75E-12
TUCP_002604	XLOC_109701	ENSRNOT00000029340	0.999116572	4.23E-15
TUCP_002604	XLOC_109701	ENSRNOT00000077972	0.962758032	5.30E-07
TUCP_002604	XLOC_109701	ENSRNOT00000038646	1	0
TUCP_002604	XLOC_109701	ENSRNOT00000012095	0.957750287	9.88E-07
TUCP_002604	XLOC_109701	ENSRNOT00000017909	0.975587192	6.56E-08
TUCP_002604	XLOC_109701	ENSRNOT00000092579	1	4.25E-78
TUCP_002604	XLOC_109701	ENSRNOT00000089235	0.998661162	3.38E-14
TUCP_002604	XLOC_109701	ENSRNOT00000036383	0.988460809	1.58E-09
TUCP_002604	XLOC_109701	ENSRNOT00000076528	0.997115373	1.57E-12
TUCP_002604	XLOC_109701	ENSRNOT00000075193	0.964326971	4.29E-07
TUCP_002604	XLOC_109701	ENSRNOT00000083034	0.990934646	4.75E-10
TUCP_002604	XLOC_109701	ENSRNOT00000089607	0.995091455	2.23E-11
TUCP_002604	XLOC_109701	ENSRNOT00000086014	0.960893638	6.74E-07
TUCP_002604	XLOC_109701	ENSRNOT00000054939	0.958021495	9.57E-07
TUCP_002604	XLOC_109701	ENSRNOT00000052315	0.983687082	8.85E-09
TUCP_002604	XLOC_109701	ENSRNOT00000088168	1	0
TUCP_002604	XLOC_109701	ENSRNOT00000046231	0.96225463	5.66E-07
TUCP_002604	XLOC_109701	ENSRNOT00000013459	0.979098017	3.03E-08
TUCP_002604	XLOC_109701	ENSRNOT00000078137	1	1.33E-79
TUCP_002604	XLOC_109701	ENSRNOT00000077764	0.977143528	4.73E-08
TUCP_002604	XLOC_109701	ENSRNOT00000043661	0.966975951	2.93E-07
TUCP_002604	XLOC_109701	ENSRNOT00000092740	1	0
TUCP_002604	XLOC_109701	ENSRNOT00000000117	0.990989174	4.61E-10
TUCP_002604	XLOC_109701	ENSRNOT00000071877	0.967113583	2.87E-07
TUCP_002604	XLOC_109701	ENSRNOT00000070958	1	0
TUCP_002604	XLOC_109701	ENSRNOT00000076757	0.959205354	8.31E-07
TUCP_002604	XLOC_109701	ENSRNOT00000085310	0.965230243	3.78E-07
TUCP_002604	XLOC_109701	ENSRNOT00000047241	0.966576993	3.11E-07

TUCP_002604	XLOC_109701	ENSRNOT00000027268	1	1.33E-79
TUCP_002604	XLOC_109701	ENSRNOT00000004459	1	0
TUCP_002604	XLOC_109701	ENSRNOT00000048373	0.959096578	8.42E-07
TUCP_002604	XLOC_109701	ENSRNOT00000061516	0.987595648	2.27E-09
TUCP_002604	XLOC_109701	ENSRNOT00000088148	1	0
TUCP_002604	XLOC_109701	ENSRNOT00000088527	1	0
TUCP_002604	XLOC_109701	ENSRNOT00000078701	1	0
TUCP_002604	XLOC_109701	ENSRNOT00000052308	0.962646127	5.38E-07
TUCP_002604	XLOC_109701	ENSRNOT00000021748	1	0
TUCP_002604	XLOC_109701	ENSRNOT00000089085	1	0
TUCP_002604	XLOC_109701	ENSRNOT00000008153	0.999703506	1.80E-17
TUCP_002604	XLOC_109701	ENSRNOT00000085896	0.971605382	1.39E-07
TUCP_002604	XLOC_109701	ENSRNOT00000079189	0.984972387	5.89E-09
TUCP_002604	XLOC_109701	ENSRNOT00000090078	1	0
TUCP_002604	XLOC_109701	ENSRNOT00000078020	0.958421666	9.13E-07
TUCP_002604	XLOC_109701	ENSRNOT00000092425	0.956092273	1.19E-06
TUCP_002604	XLOC_109701	ENSRNOT00000009776	1	1.33E-79
TUCP_003739	XLOC_164001	ENSRNOT00000091323	0.956926557	1.09E-06
TUCP_003739	XLOC_164001	ENSRNOT00000040102	0.988139181	1.81E-09
TUCP_002607	XLOC_109878	ENSRNOT00000081570	0.962961027	5.16E-07
TUCP_002607	XLOC_109878	ENSRNOT00000091652	0.99819185	1.52E-13
TUCP_002607	XLOC_109878	ENSRNOT00000036564	0.972337506	1.22E-07
TUCP_002607	XLOC_109878	ENSRNOT00000000109	0.962179854	5.72E-07
TUCP_002607	XLOC_109878	ENSRNOT00000080865	1	0
TUCP_002607	XLOC_109878	ENSRNOT00000073534	0.995808019	1.01E-11
TUCP_002607	XLOC_109878	ENSRNOT00000012161	0.987544327	2.31E-09
TUCP_002607	XLOC_109878	ENSRNOT00000078707	0.960060506	7.48E-07
TUCP_002607	XLOC_109878	ENSRNOT00000087758	0.968778527	2.22E-07
TUCP_002607	XLOC_109878	ENSRNOT00000049832	0.969039151	2.13E-07
TUCP_002607	XLOC_109878	ENSRNOT00000093019	1	0
TUCP_002607	XLOC_109878	ENSRNOT00000024825	0.980452701	2.18E-08
TUCP_002607	XLOC_109878	ENSRNOT00000059266	0.96033506	7.23E-07
TUCP_002607	XLOC_109878	ENSRNOT00000072734	0.965018115	3.89E-07
TUCP_002607	XLOC_109878	ENSRNOT00000085908	0.996653227	3.29E-12
TUCP_002607	XLOC_109878	ENSRNOT00000046744	1	0
TUCP_002607	XLOC_109878	ENSRNOT00000076429	1	0
TUCP_002607	XLOC_109878	ENSRNOT00000084667	0.999660971	3.53E-17
TUCP_002607	XLOC_109878	ENSRNOT00000055412	1	0
TUCP_002607	XLOC_109878	ENSRNOT00000093201	0.9997022	1.84E-17
TUCP_002607	XLOC_109878	ENSRNOT00000072724	1	0
TUCP_002607	XLOC_109878	ENSRNOT00000090323	0.95603782	1.20E-06
TUCP_002607	XLOC_109878	ENSRNOT00000082760	0.986293522	3.72E-09
TUCP_002607	XLOC_109878	ENSRNOT00000066017	0.989785653	8.61E-10
TUCP_002607	XLOC_109878	ENSRNOT00000073148	0.985721397	4.56E-09
TUCP_002607	XLOC_109878	ENSRNOT00000077462	0.960098183	7.45E-07
TUCP_002607	XLOC_109878	ENSRNOT00000084375	1	0
TUCP_002607	XLOC_109878	ENSRNOT00000085447	0.998400682	8.22E-14
TUCP_002607	XLOC_109878	ENSRNOT00000006249	0.966489607	3.15E-07
TUCP_002607	XLOC_109878	ENSRNOT00000087073	0.952450698	1.77E-06
TUCP_002607	XLOC_109878	ENSRNOT00000042369	0.995659369	1.20E-11
TUCP_002607	XLOC_109878	ENSRNOT00000086554	1	1.33E-79

TUCP_002607	XLOC_109878	ENSRNOT00000054858	0.985228824	5.40E-09
TUCP_002607	XLOC_109878	ENSRNOT00000047921	1	0
TUCP_002607	XLOC_109878	ENSRNOT00000064261	0.999977196	4.86E-23
TUCP_002607	XLOC_109878	ENSRNOT00000086660	0.999208903	2.44E-15
TUCP_002607	XLOC_109878	ENSRNOT00000080933	0.967360681	2.76E-07
TUCP_002607	XLOC_109878	ENSRNOT00000083977	1	0
TUCP_002607	XLOC_109878	ENSRNOT00000092933	1	0
TUCP_002607	XLOC_109878	ENSRNOT00000009061	0.953300672	1.62E-06
TUCP_002607	XLOC_109878	ENSRNOT00000041281	0.987828355	2.06E-09
TUCP_003903	XLOC_171920	ENSRNOT00000071913	0.959287029	8.23E-07
TUCP_003326	XLOC_143304	ENSRNOT00000032618	0.977910969	3.99E-08
TUCP_003905	XLOC_172034	ENSRNOT00000016289	0.957751113	9.87E-07
TUCP_003905	XLOC_172034	ENSRNOT00000072698	0.977520953	4.35E-08
TUCP_003905	XLOC_172034	ENSRNOT00000013369	0.952030895	1.85E-06
TUCP_001986	XLOC_080700	ENSRNOT00000055572	-0.957500227	1.02E-06
TUCP_001984	XLOC_080392	ENSRNOT00000065285	0.953244994	1.63E-06
TUCP_001985	XLOC_080640	ENSRNOT00000086279	0.964825795	4.00E-07
TUCP_001985	XLOC_080640	ENSRNOT00000066583	0.953765022	1.54E-06
TUCP_001565	XLOC_060492	ENSRNOT00000027411	0.975724355	6.37E-08
TUCP_001565	XLOC_060492	ENSRNOT00000045285	0.95260585	1.74E-06
TUCP_001565	XLOC_060492	ENSRNOT00000081512	0.959334981	8.18E-07
TUCP_001565	XLOC_060492	ENSRNOT00000067663	0.964706063	4.06E-07
TUCP_001565	XLOC_060492	ENSRNOT00000067866	0.958638189	8.89E-07
TUCP_001565	XLOC_060492	ENSRNOT00000080024	0.958887232	8.63E-07
TUCP_001565	XLOC_060492	ENSRNOT00000012048	0.968449359	2.34E-07
TUCP_001565	XLOC_060492	ENSRNOT00000071407	0.957455482	1.02E-06
TUCP_001565	XLOC_060492	ENSRNOT00000076763	0.961689085	6.09E-07
TUCP_001565	XLOC_060492	ENSRNOT00000075558	0.973189731	1.04E-07
TUCP_004226	XLOC_185563	ENSRNOT00000004842	0.958203528	9.36E-07
TUCP_004226	XLOC_185563	ENSRNOT00000030893	0.963046996	5.10E-07
TUCP_004226	XLOC_185563	ENSRNOT00000044147	0.95445507	1.43E-06
TUCP_004226	XLOC_185563	ENSRNOT00000011344	0.978627342	3.39E-08
TUCP_004226	XLOC_185563	ENSRNOT00000014553	0.950339838	2.19E-06
TUCP_004226	XLOC_185563	ENSRNOT00000048298	0.95266467	1.73E-06
TUCP_004226	XLOC_185563	ENSRNOT00000025308	0.953352924	1.61E-06
TUCP_004226	XLOC_185563	ENSRNOT00000010020	0.959816509	7.71E-07
TUCP_004226	XLOC_185563	ENSRNOT00000017232	0.966825411	2.99E-07
TUCP_004226	XLOC_185563	ENSRNOT00000029076	0.953079571	1.65E-06
TUCP_004226	XLOC_185563	ENSRNOT00000071453	-0.95054739	2.14E-06
TUCP_004226	XLOC_185563	ENSRNOT00000048770	0.961007423	6.65E-07
TUCP_001980	XLOC_079978	ENSRNOT00000040116	0.960063895	7.48E-07
TUCP_001980	XLOC_079978	ENSRNOT00000008399	0.965888074	3.44E-07
TUCP_001980	XLOC_079978	ENSRNOT00000000995	0.96453545	4.16E-07
TUCP_001980	XLOC_079978	ENSRNOT00000014533	0.964881564	3.97E-07
TUCP_001980	XLOC_079978	ENSRNOT00000018888	0.963231932	4.98E-07
TUCP_001980	XLOC_079978	ENSRNOT00000068354	0.960481015	7.10E-07
TUCP_001980	XLOC_079978	ENSRNOT00000003082	0.981080262	1.85E-08
TUCP_001980	XLOC_079978	ENSRNOT00000044751	0.954891781	1.36E-06
TUCP_001980	XLOC_079978	ENSRNOT00000012963	0.965844397	3.46E-07
TUCP_001980	XLOC_079978	ENSRNOT00000057504	0.962105259	5.77E-07
TUCP_001980	XLOC_079978	ENSRNOT00000010968	0.977689438	4.19E-08

TUCP_001980	XLOC_079978	ENSRNOT00000009448	0.95510821	1.33E-06
TUCP_001980	XLOC_079978	ENSRNOT00000047731	0.957071718	1.07E-06
TUCP_001980	XLOC_079978	ENSRNOT00000009136	0.954152563	1.48E-06
TUCP_001980	XLOC_079978	ENSRNOT00000013182	0.964553904	4.15E-07
TUCP_001980	XLOC_079978	ENSRNOT00000025731	0.961267228	6.43E-07
TUCP_001980	XLOC_079978	ENSRNOT00000042973	0.96509786	3.85E-07
TUCP_001980	XLOC_079978	ENSRNOT00000003458	0.966437823	3.17E-07
TUCP_001980	XLOC_079978	ENSRNOT00000042117	0.971313306	1.46E-07
TUCP_001980	XLOC_079978	ENSRNOT00000002853	0.965456853	3.66E-07
TUCP_001980	XLOC_079978	ENSRNOT00000000110	0.960317901	7.25E-07
TUCP_001980	XLOC_079978	ENSRNOT00000006196	0.972185652	1.25E-07
TUCP_001980	XLOC_079978	ENSRNOT00000013809	0.950120424	2.24E-06
TUCP_001980	XLOC_079978	ENSRNOT00000073087	0.96219186	5.71E-07
TUCP_001980	XLOC_079978	ENSRNOT00000019389	0.964940329	3.93E-07
TUCP_001980	XLOC_079978	ENSRNOT00000021487	0.969534246	1.96E-07
TUCP_001980	XLOC_079978	ENSRNOT00000008813	0.981453637	1.68E-08
TUCP_001980	XLOC_079978	ENSRNOT00000051067	0.973718344	9.45E-08
TUCP_001980	XLOC_079978	ENSRNOT00000023067	0.956971661	1.08E-06
TUCP_001980	XLOC_079978	ENSRNOT00000073138	0.958431119	9.12E-07
TUCP_001980	XLOC_079978	ENSRNOT00000045357	0.954671049	1.40E-06
TUCP_001980	XLOC_079978	ENSRNOT00000014317	0.957941227	9.66E-07
TUCP_001980	XLOC_079978	ENSRNOT00000029845	0.951716413	1.91E-06
TUCP_001980	XLOC_079978	ENSRNOT00000022128	0.971283181	1.47E-07
TUCP_001981	XLOC_080226	ENSRNOT00000082869	0.963796537	4.61E-07
TUCP_001981	XLOC_080226	ENSRNOT00000012811	0.955954744	1.21E-06
TUCP_001981	XLOC_080226	ENSRNOT00000088353	-0.960875586	6.76E-07
TUCP_004595	XLOC_201913	ENSRNOT00000087934	0.962569371	5.43E-07
TUCP_004595	XLOC_201913	ENSRNOT00000082891	0.953719809	1.55E-06
TUCP_000688	XLOC_026080	ENSRNOT00000063883	0.960874214	6.76E-07
TUCP_003118	XLOC_133008	ENSRNOT00000067150	0.960214992	7.34E-07
TUCP_003118	XLOC_133008	ENSRNOT00000008982	0.954798055	1.38E-06
TUCP_003118	XLOC_133008	ENSRNOT00000001215	0.96317308	5.01E-07
TUCP_003118	XLOC_133008	ENSRNOT00000027526	0.950096022	2.24E-06
TUCP_003118	XLOC_133008	ENSRNOT00000092029	0.951133185	2.02E-06
TUCP_003118	XLOC_133008	ENSRNOT00000088599	0.950890855	2.07E-06
TUCP_003118	XLOC_133008	ENSRNOT00000048910	-0.956968117	1.08E-06
TUCP_003118	XLOC_133008	ENSRNOT00000016991	0.970001585	1.82E-07
TUCP_003118	XLOC_133008	ENSRNOT00000027375	0.966658124	3.07E-07
TUCP_003118	XLOC_133008	ENSRNOT00000005934	0.952107286	1.83E-06
TUCP_003118	XLOC_133008	ENSRNOT00000043872	0.971698597	1.36E-07
TUCP_003118	XLOC_133008	ENSRNOT00000017665	0.962676105	5.36E-07
TUCP_003118	XLOC_133008	ENSRNOT00000079866	0.951596858	1.93E-06
TUCP_003118	XLOC_133008	ENSRNOT00000041513	0.956328869	1.16E-06
TUCP_003118	XLOC_133008	ENSRNOT00000002443	0.95239586	1.78E-06
TUCP_003118	XLOC_133008	ENSRNOT00000003891	0.961359529	6.36E-07
TUCP_003118	XLOC_133008	ENSRNOT00000077406	0.961559296	6.20E-07
TUCP_003118	XLOC_133008	ENSRNOT00000015434	0.963679629	4.68E-07
TUCP_003118	XLOC_133008	ENSRNOT00000012334	0.973623353	9.62E-08
TUCP_003118	XLOC_133008	ENSRNOT00000015092	0.954443514	1.43E-06
TUCP_003118	XLOC_133008	ENSRNOT00000005220	0.951355875	1.98E-06
TUCP_003118	XLOC_133008	ENSRNOT00000005459	0.957814653	9.80E-07

TUCP_003118	XLOC_133008	ENSRNOT00000045326	0.958538506	9.00E-07
TUCP_003118	XLOC_133008	ENSRNOT00000017776	0.958696302	8.83E-07
TUCP_003118	XLOC_133008	ENSRNOT00000084422	0.964821723	4.00E-07
TUCP_003118	XLOC_133008	ENSRNOT00000046745	0.950917992	2.07E-06
TUCP_003118	XLOC_133008	ENSRNOT00000004172	0.952650244	1.73E-06
TUCP_003118	XLOC_133008	ENSRNOT00000084385	0.954918925	1.36E-06
TUCP_003118	XLOC_133008	ENSRNOT00000008104	0.965251766	3.76E-07
TUCP_003118	XLOC_133008	ENSRNOT00000072208	0.960854972	6.78E-07
TUCP_003118	XLOC_133008	ENSRNOT00000040476	0.952716686	1.72E-06
TUCP_003118	XLOC_133008	ENSRNOT00000001403	0.960456713	7.12E-07
TUCP_003118	XLOC_133008	ENSRNOT00000029125	0.953746461	1.54E-06
TUCP_003118	XLOC_133008	ENSRNOT00000055321	0.961742182	6.05E-07
TUCP_003118	XLOC_133008	ENSRNOT00000001242	0.950812303	2.09E-06
TUCP_003118	XLOC_133008	ENSRNOT00000000013	0.952640726	1.73E-06
TUCP_003118	XLOC_133008	ENSRNOT00000090918	0.954964207	1.35E-06
TUCP_003118	XLOC_133008	ENSRNOT00000046973	0.970320265	1.73E-07
TUCP_003118	XLOC_133008	ENSRNOT00000045699	0.956239453	1.17E-06
TUCP_003118	XLOC_133008	ENSRNOT00000040069	0.955716338	1.24E-06
TUCP_003118	XLOC_133008	ENSRNOT00000083220	0.955830333	1.23E-06
TUCP_003118	XLOC_133008	ENSRNOT00000081981	0.951635282	1.92E-06
TUCP_003118	XLOC_133008	ENSRNOT00000024969	0.951958521	1.86E-06
TUCP_003118	XLOC_133008	ENSRNOT00000017063	-0.952867579	1.69E-06
TUCP_003118	XLOC_133008	ENSRNOT00000010399	0.971860829	1.33E-07
TUCP_003118	XLOC_133008	ENSRNOT00000072132	0.952478307	1.76E-06
TUCP_003118	XLOC_133008	ENSRNOT00000047356	0.95179836	1.89E-06
TUCP_003118	XLOC_133008	ENSRNOT00000014124	0.951007413	2.05E-06
TUCP_003118	XLOC_133008	ENSRNOT00000004463	0.950660463	2.12E-06
TUCP_003118	XLOC_133008	ENSRNOT00000030109	0.960185222	7.37E-07
TUCP_003118	XLOC_133008	ENSRNOT00000081047	0.954317537	1.45E-06
TUCP_003118	XLOC_133008	ENSRNOT00000026597	0.958016368	9.57E-07
TUCP_003118	XLOC_133008	ENSRNOT00000011270	0.954550599	1.41E-06
TUCP_003118	XLOC_133008	ENSRNOT00000015265	0.955660009	1.25E-06
TUCP_003118	XLOC_133008	ENSRNOT00000078475	0.95803596	9.55E-07
TUCP_003118	XLOC_133008	ENSRNOT00000042498	0.957853602	9.76E-07
TUCP_003118	XLOC_133008	ENSRNOT00000048063	0.950932229	2.06E-06
TUCP_003118	XLOC_133008	ENSRNOT00000027970	0.960280769	7.28E-07
TUCP_003118	XLOC_133008	ENSRNOT00000015793	0.956341537	1.16E-06
TUCP_003118	XLOC_133008	ENSRNOT00000011287	0.960808394	6.82E-07
TUCP_003118	XLOC_133008	ENSRNOT00000079454	0.954621357	1.40E-06
TUCP_003118	XLOC_133008	ENSRNOT00000032780	0.953019709	1.67E-06
TUCP_003118	XLOC_133008	ENSRNOT00000083957	0.966941901	2.94E-07
TUCP_003118	XLOC_133008	ENSRNOT00000038066	0.955390828	1.29E-06
TUCP_003118	XLOC_133008	ENSRNOT00000015609	0.953138739	1.64E-06
TUCP_003118	XLOC_133008	ENSRNOT00000035474	0.957062395	1.07E-06
TUCP_003118	XLOC_133008	ENSRNOT00000078102	0.960620882	6.98E-07
TUCP_003118	XLOC_133008	ENSRNOT00000037456	0.956065164	1.20E-06
TUCP_003118	XLOC_133008	ENSRNOT00000086122	0.950469554	2.16E-06
TUCP_002468	XLOC_104510	ENSRNOT00000001215	0.961052132	6.61E-07
TUCP_002468	XLOC_104510	ENSRNOT00000006188	-0.977588703	4.29E-08
TUCP_002468	XLOC_104510	ENSRNOT00000006186	0.950609533	2.13E-06
TUCP_002468	XLOC_104510	ENSRNOT00000090621	0.959739224	7.79E-07

TUCP_002468	XLOC_104510	ENSRNOT00000013745	-0.973253365	1.03E-07
TUCP_002468	XLOC_104510	ENSRNOT00000075397	0.977641908	4.24E-08
TUCP_002468	XLOC_104510	ENSRNOT00000032926	-0.970173167	1.77E-07
TUCP_002468	XLOC_104510	ENSRNOT00000019104	-0.961866231	5.96E-07
TUCP_002468	XLOC_104510	ENSRNOT00000006217	0.953974264	1.51E-06
TUCP_002468	XLOC_104510	ENSRNOT00000068353	0.959158065	8.36E-07
TUCP_002468	XLOC_104510	ENSRNOT00000001691	0.977705904	4.18E-08
TUCP_002468	XLOC_104510	ENSRNOT00000089319	0.970742516	1.61E-07
TUCP_002468	XLOC_104510	ENSRNOT00000079138	0.968346982	2.37E-07
TUCP_002468	XLOC_104510	ENSRNOT00000086706	0.961454684	6.28E-07
TUCP_002468	XLOC_104510	ENSRNOT00000085885	-0.952407913	1.77E-06
TUCP_002468	XLOC_104510	ENSRNOT00000010030	-0.950382912	2.18E-06
TUCP_002468	XLOC_104510	ENSRNOT00000045233	-0.959909132	7.62E-07
TUCP_002468	XLOC_104510	ENSRNOT00000011130	-0.951907458	1.87E-06
TUCP_002468	XLOC_104510	ENSRNOT00000052063	0.955677692	1.25E-06
TUCP_002468	XLOC_104510	ENSRNOT00000030850	0.953283883	1.62E-06
TUCP_002468	XLOC_104510	ENSRNOT00000055582	0.951211689	2.01E-06
TUCP_002468	XLOC_104510	ENSRNOT00000055540	0.961754252	6.04E-07
TUCP_002468	XLOC_104510	ENSRNOT00000006752	0.969698791	1.91E-07
TUCP_002468	XLOC_104510	ENSRNOT00000021534	-0.971439674	1.43E-07
TUCP_002468	XLOC_104510	ENSRNOT00000085251	-0.954066919	1.49E-06
TUCP_002468	XLOC_104510	ENSRNOT00000083677	-0.96875628	2.22E-07
TUCP_002468	XLOC_104510	ENSRNOT00000065304	-0.950996355	2.05E-06
TUCP_002468	XLOC_104510	ENSRNOT00000073370	0.961736015	6.06E-07
TUCP_002468	XLOC_104510	ENSRNOT00000065978	0.965363416	3.70E-07
TUCP_002468	XLOC_104510	ENSRNOT00000035320	-0.953723531	1.55E-06
TUCP_002468	XLOC_104510	ENSRNOT00000057160	0.961865656	5.96E-07
TUCP_002468	XLOC_104510	ENSRNOT00000065871	0.956567155	1.13E-06
TUCP_002468	XLOC_104510	ENSRNOT00000083964	-0.974900136	7.52E-08
TUCP_002468	XLOC_104510	ENSRNOT00000019192	-0.957320901	1.04E-06
TUCP_002468	XLOC_104510	ENSRNOT00000077999	0.959939181	7.60E-07
TUCP_002468	XLOC_104510	ENSRNOT00000083351	-0.961582007	6.18E-07
TUCP_002468	XLOC_104510	ENSRNOT00000077856	-0.953504622	1.58E-06
TUCP_002468	XLOC_104510	ENSRNOT00000015188	0.956886783	1.09E-06
TUCP_002468	XLOC_104510	ENSRNOT00000018488	-0.95476431	1.38E-06
TUCP_002468	XLOC_104510	ENSRNOT00000005686	-0.960378197	7.19E-07
TUCP_002468	XLOC_104510	ENSRNOT00000040629	0.954824433	1.37E-06
TUCP_002468	XLOC_104510	ENSRNOT00000005834	0.954026797	1.50E-06
TUCP_002468	XLOC_104510	ENSRNOT00000005297	0.959514478	8.00E-07
TUCP_002468	XLOC_104510	ENSRNOT00000000048	0.979168737	2.98E-08
TUCP_002468	XLOC_104510	ENSRNOT00000075934	-0.952152975	1.82E-06
TUCP_002468	XLOC_104510	ENSRNOT00000076181	-0.966277925	3.25E-07
TUCP_002468	XLOC_104510	ENSRNOT00000031384	0.954693608	1.39E-06
TUCP_002468	XLOC_104510	ENSRNOT00000080410	-0.970728766	1.61E-07
TUCP_002468	XLOC_104510	ENSRNOT00000087278	-0.955301561	1.30E-06
TUCP_002468	XLOC_104510	ENSRNOT00000010367	0.96539806	3.69E-07
TUCP_002468	XLOC_104510	ENSRNOT00000041044	0.958589514	8.95E-07
TUCP_002468	XLOC_104510	ENSRNOT00000067391	-0.960758354	6.86E-07
TUCP_002468	XLOC_104510	ENSRNOT00000009150	0.962387453	5.57E-07
TUCP_002468	XLOC_104510	ENSRNOT00000084709	0.964333544	4.28E-07
TUCP_002468	XLOC_104510	ENSRNOT00000068033	0.959339121	8.17E-07

TUCP_002468	XLOC_104510	ENSRNOT00000026481	0.956322761	1.16E-06
TUCP_002468	XLOC_104510	ENSRNOT00000082709	-0.966402582	3.19E-07
TUCP_002468	XLOC_104510	ENSRNOT00000025856	-0.979457273	2.78E-08
TUCP_002468	XLOC_104510	ENSRNOT00000026643	0.96810738	2.46E-07
TUCP_002468	XLOC_104510	ENSRNOT00000001403	0.975283451	6.97E-08
TUCP_002468	XLOC_104510	ENSRNOT00000072357	-0.965592636	3.58E-07
TUCP_002468	XLOC_104510	ENSRNOT00000016132	-0.975312313	6.93E-08
TUCP_002468	XLOC_104510	ENSRNOT00000058495	-0.959929192	7.61E-07
TUCP_002468	XLOC_104510	ENSRNOT00000013067	-0.97420643	8.61E-08
TUCP_002468	XLOC_104510	ENSRNOT00000066952	0.965391712	3.69E-07
TUCP_002468	XLOC_104510	ENSRNOT00000016220	-0.953931511	1.51E-06
TUCP_002468	XLOC_104510	ENSRNOT00000048063	0.951558514	1.94E-06
TUCP_002468	XLOC_104510	ENSRNOT00000003780	0.968023554	2.50E-07
TUCP_002468	XLOC_104510	ENSRNOT00000020910	0.970864858	1.57E-07
TUCP_002468	XLOC_104510	ENSRNOT00000041815	-0.959566665	7.95E-07
TUCP_002468	XLOC_104510	ENSRNOT00000033975	0.966908016	2.96E-07
TUCP_002468	XLOC_104510	ENSRNOT00000079895	0.953317894	1.61E-06
TUCP_002468	XLOC_104510	ENSRNOT00000074586	-0.96079207	6.83E-07
TUCP_002468	XLOC_104510	ENSRNOT00000045765	-0.960165217	7.39E-07
TUCP_002468	XLOC_104510	ENSRNOT00000060494	0.95265609	1.73E-06
TUCP_002468	XLOC_104510	ENSRNOT00000002465	0.952427542	1.77E-06
TUCP_002468	XLOC_104510	ENSRNOT00000011880	0.951022936	2.04E-06
TUCP_002468	XLOC_104510	ENSRNOT00000029202	-0.970588427	1.65E-07
TUCP_002468	XLOC_104510	ENSRNOT00000031487	0.952215605	1.81E-06
TUCP_002468	XLOC_104510	ENSRNOT00000020885	0.951376964	1.97E-06
TUCP_002468	XLOC_104510	ENSRNOT00000012286	-0.959817332	7.71E-07
TUCP_002468	XLOC_104510	ENSRNOT00000050515	0.965711917	3.52E-07
TUCP_002468	XLOC_104510	ENSRNOT00000012942	0.951614931	1.93E-06
TUCP_002468	XLOC_104510	ENSRNOT00000011401	-0.958688958	8.84E-07
TUCP_002468	XLOC_104510	ENSRNOT00000055602	-0.97479019	7.69E-08
TUCP_002468	XLOC_104510	ENSRNOT00000046745	0.961049369	6.61E-07
TUCP_002468	XLOC_104510	ENSRNOT00000082146	-0.961443415	6.29E-07
TUCP_002468	XLOC_104510	ENSRNOT00000031555	-0.958571401	8.96E-07
TUCP_002468	XLOC_104510	ENSRNOT00000011563	-0.960955469	6.69E-07
TUCP_002468	XLOC_104510	ENSRNOT00000000263	0.958933145	8.59E-07
TUCP_002468	XLOC_104510	ENSRNOT00000023876	0.962748406	5.31E-07
TUCP_002468	XLOC_104510	ENSRNOT00000023872	-0.952536514	1.75E-06
TUCP_002468	XLOC_104510	ENSRNOT00000049285	-0.974990109	7.39E-08
TUCP_002468	XLOC_104510	ENSRNOT00000015239	-0.965931549	3.41E-07
TUCP_002468	XLOC_104510	ENSRNOT00000003568	0.96813392	2.45E-07
TUCP_002468	XLOC_104510	ENSRNOT00000006366	-0.954136907	1.48E-06
TUCP_002468	XLOC_104510	ENSRNOT00000074649	0.974427763	8.25E-08
TUCP_002468	XLOC_104510	ENSRNOT00000033196	-0.956158818	1.18E-06
TUCP_002468	XLOC_104510	ENSRNOT00000077146	-0.981659135	1.58E-08
TUCP_002468	XLOC_104510	ENSRNOT00000060002	-0.973618432	9.63E-08
TUCP_002468	XLOC_104510	ENSRNOT00000089673	-0.982629549	1.21E-08
TUCP_002468	XLOC_104510	ENSRNOT00000027301	-0.964953927	3.93E-07
TUCP_002468	XLOC_104510	ENSRNOT00000075209	-0.960392846	7.18E-07
TUCP_002468	XLOC_104510	ENSRNOT00000057738	0.971688311	1.37E-07
TUCP_002468	XLOC_104510	ENSRNOT00000023977	-0.976518379	5.41E-08
TUCP_002468	XLOC_104510	ENSRNOT00000066133	-0.969096083	2.11E-07

TUCP_002468	XLOC_104510	ENSRNOT00000006765	-0.967910156	2.54E-07
TUCP_002468	XLOC_104510	ENSRNOT00000035385	0.961493635	6.25E-07
TUCP_002468	XLOC_104510	ENSRNOT00000003102	0.950050365	2.25E-06
TUCP_002468	XLOC_104510	ENSRNOT00000002443	0.965992268	3.38E-07
TUCP_002468	XLOC_104510	ENSRNOT00000014924	-0.966165226	3.30E-07
TUCP_002468	XLOC_104510	ENSRNOT00000030692	-0.969040476	2.13E-07
TUCP_002468	XLOC_104510	ENSRNOT00000014885	-0.956200126	1.18E-06
TUCP_002468	XLOC_104510	ENSRNOT00000064535	-0.957746199	9.88E-07
TUCP_002468	XLOC_104510	ENSRNOT00000015624	0.964788676	4.02E-07
TUCP_002468	XLOC_104510	ENSRNOT00000085295	-0.964148137	4.39E-07
TUCP_002468	XLOC_104510	ENSRNOT00000008222	-0.966896834	2.96E-07
TUCP_002468	XLOC_104510	ENSRNOT00000009749	-0.951269264	1.99E-06
TUCP_002468	XLOC_104510	ENSRNOT00000039102	0.978996681	3.11E-08
TUCP_002468	XLOC_104510	ENSRNOT00000086260	-0.962628186	5.39E-07
TUCP_002468	XLOC_104510	ENSRNOT00000023807	-0.971301574	1.46E-07
TUCP_002468	XLOC_104510	ENSRNOT00000078856	-0.972693229	1.14E-07
TUCP_002468	XLOC_104510	ENSRNOT00000091205	0.967295883	2.79E-07
TUCP_002468	XLOC_104510	ENSRNOT00000017692	-0.956539991	1.13E-06
TUCP_002468	XLOC_104510	ENSRNOT00000009742	-0.963720863	4.66E-07
TUCP_002468	XLOC_104510	ENSRNOT00000010983	0.966894057	2.96E-07
TUCP_002468	XLOC_104510	ENSRNOT00000007369	0.979979196	2.45E-08
TUCP_002468	XLOC_104510	ENSRNOT00000004787	-0.965603771	3.58E-07
TUCP_002468	XLOC_104510	ENSRNOT00000085466	0.961516528	6.23E-07
TUCP_002468	XLOC_104510	ENSRNOT00000060698	-0.968904713	2.17E-07
TUCP_002468	XLOC_104510	ENSRNOT00000058351	0.957474207	1.02E-06
TUCP_002468	XLOC_104510	ENSRNOT00000022000	-0.963140363	5.04E-07
TUCP_002468	XLOC_104510	ENSRNOT00000083891	-0.958587409	8.95E-07
TUCP_002468	XLOC_104510	ENSRNOT00000010748	0.959745527	7.78E-07
TUCP_002468	XLOC_104510	ENSRNOT00000023537	-0.958747367	8.78E-07
TUCP_002468	XLOC_104510	ENSRNOT00000017995	0.965321676	3.73E-07
TUCP_002468	XLOC_104510	ENSRNOT00000013431	-0.953219709	1.63E-06
TUCP_002468	XLOC_104510	ENSRNOT00000028703	-0.953380841	1.60E-06
TUCP_002468	XLOC_104510	ENSRNOT00000088679	-0.965721655	3.52E-07
TUCP_002468	XLOC_104510	ENSRNOT00000051494	0.955227953	1.31E-06
TUCP_002468	XLOC_104510	ENSRNOT00000023256	0.965480851	3.64E-07
TUCP_002468	XLOC_104510	ENSRNOT00000037809	-0.952743036	1.71E-06
TUCP_002468	XLOC_104510	ENSRNOT00000059807	0.951439777	1.96E-06
TUCP_002468	XLOC_104510	ENSRNOT00000008982	0.960708354	6.90E-07
TUCP_002468	XLOC_104510	ENSRNOT00000022097	0.956961826	1.08E-06
TUCP_002468	XLOC_104510	ENSRNOT00000008987	-0.985081166	5.68E-09
TUCP_002468	XLOC_104510	ENSRNOT00000090299	0.960315865	7.25E-07
TUCP_002468	XLOC_104510	ENSRNOT00000092876	-0.964966653	3.92E-07
TUCP_002468	XLOC_104510	ENSRNOT00000024253	0.95307126	1.66E-06
TUCP_002468	XLOC_104510	ENSRNOT00000088073	-0.956343013	1.16E-06
TUCP_002468	XLOC_104510	ENSRNOT00000065143	0.955104922	1.33E-06
TUCP_002468	XLOC_104510	ENSRNOT00000040060	-0.960584544	7.01E-07
TUCP_002468	XLOC_104510	ENSRNOT00000081796	0.951693669	1.91E-06
TUCP_002468	XLOC_104510	ENSRNOT00000027083	0.951386871	1.97E-06
TUCP_002468	XLOC_104510	ENSRNOT00000063930	-0.981371834	1.71E-08
TUCP_002468	XLOC_104510	ENSRNOT00000013116	-0.953808946	1.53E-06
TUCP_002468	XLOC_104510	ENSRNOT00000001855	0.968904608	2.17E-07

TUCP_002468	XLOC_104510	ENSRNOT00000009563	-0.976064774	5.94E-08
TUCP_002468	XLOC_104510	ENSRNOT00000072771	0.960060035	7.48E-07
TUCP_002468	XLOC_104510	ENSRNOT00000041237	-0.950042751	2.25E-06
TUCP_002468	XLOC_104510	ENSRNOT00000057953	0.951020787	2.04E-06
TUCP_002468	XLOC_104510	ENSRNOT00000088555	-0.958210986	9.36E-07
TUCP_002468	XLOC_104510	ENSRNOT00000055683	0.955401329	1.29E-06
TUCP_002468	XLOC_104510	ENSRNOT00000081981	0.967158021	2.85E-07
TUCP_002468	XLOC_104510	ENSRNOT00000026531	-0.974703769	7.82E-08
TUCP_002468	XLOC_104510	ENSRNOT00000003326	-0.975734628	6.36E-08
TUCP_002468	XLOC_104510	ENSRNOT00000016306	-0.974798694	7.67E-08
TUCP_002468	XLOC_104510	ENSRNOT00000015476	-0.95977114	7.76E-07
TUCP_002468	XLOC_104510	ENSRNOT00000036904	0.956141405	1.19E-06
TUCP_002468	XLOC_104510	ENSRNOT00000025079	-0.957959617	9.64E-07
TUCP_002468	XLOC_104510	ENSRNOT00000048754	-0.955689685	1.25E-06
TUCP_002468	XLOC_104510	ENSRNOT00000012699	-0.956735181	1.11E-06
TUCP_002468	XLOC_104510	ENSRNOT00000064643	0.957301011	1.04E-06
TUCP_002468	XLOC_104510	ENSRNOT00000007824	-0.957805072	9.81E-07
TUCP_002468	XLOC_104510	ENSRNOT00000020681	-0.956200781	1.18E-06
TUCP_002468	XLOC_104510	ENSRNOT00000057898	-0.979746266	2.59E-08
TUCP_002468	XLOC_104510	ENSRNOT00000024677	0.959458941	8.06E-07
TUCP_002468	XLOC_104510	ENSRNOT00000091602	-0.957544698	1.01E-06
TUCP_002468	XLOC_104510	ENSRNOT00000090338	-0.961724012	6.07E-07
TUCP_002468	XLOC_104510	ENSRNOT00000056100	-0.976656641	5.25E-08
TUCP_002468	XLOC_104510	ENSRNOT00000001077	0.952219072	1.81E-06
TUCP_002468	XLOC_104510	ENSRNOT00000036838	-0.97568223	6.43E-08
TUCP_002468	XLOC_104510	ENSRNOT00000009817	-0.953475953	1.59E-06
TUCP_002468	XLOC_104510	ENSRNOT00000030798	-0.95760981	1.00E-06
TUCP_002468	XLOC_104510	ENSRNOT00000018634	-0.971143922	1.50E-07
TUCP_002468	XLOC_104510	ENSRNOT00000071540	0.954216664	1.47E-06
TUCP_002468	XLOC_104510	ENSRNOT00000031701	-0.956008234	1.21E-06
TUCP_002468	XLOC_104510	ENSRNOT00000072849	-0.958113764	9.46E-07
TUCP_002468	XLOC_104510	ENSRNOT00000073643	-0.973645401	9.58E-08
TUCP_002468	XLOC_104510	ENSRNOT00000078349	-0.964557474	4.15E-07
TUCP_002468	XLOC_104510	ENSRNOT00000011654	-0.956786557	1.10E-06
TUCP_002468	XLOC_104510	ENSRNOT00000022502	-0.965784576	3.49E-07
TUCP_002468	XLOC_104510	ENSRNOT00000026928	-0.952205704	1.81E-06
TUCP_002468	XLOC_104510	ENSRNOT00000017556	0.951442325	1.96E-06
TUCP_002468	XLOC_104510	ENSRNOT00000078816	0.955604479	1.26E-06
TUCP_002468	XLOC_104510	ENSRNOT00000001759	-0.962624859	5.39E-07
TUCP_002468	XLOC_104510	ENSRNOT00000021802	0.9629097	5.19E-07
TUCP_002468	XLOC_104510	ENSRNOT00000021803	-0.952666501	1.73E-06
TUCP_002468	XLOC_104510	ENSRNOT00000020147	0.950164277	2.23E-06
TUCP_002468	XLOC_104510	ENSRNOT00000024352	0.972417398	1.20E-07
TUCP_002468	XLOC_104510	ENSRNOT00000004351	-0.962356237	5.59E-07
TUCP_002468	XLOC_104510	ENSRNOT00000072118	0.956228639	1.18E-06
TUCP_002468	XLOC_104510	ENSRNOT00000087748	-0.962053349	5.81E-07
TUCP_002468	XLOC_104510	ENSRNOT00000003787	-0.954844127	1.37E-06
TUCP_002468	XLOC_104510	ENSRNOT00000014506	-0.960971491	6.68E-07
TUCP_002468	XLOC_104510	ENSRNOT00000023262	-0.970782767	1.60E-07
TUCP_002468	XLOC_104510	ENSRNOT00000059246	0.954773045	1.38E-06
TUCP_002468	XLOC_104510	ENSRNOT00000081800	0.954715557	1.39E-06

TUCP_002468	XLOC_104510	ENSRNOT00000045946	0.974331296	8.41E-08
TUCP_002468	XLOC_104510	ENSRNOT00000008865	-0.950690503	2.11E-06
TUCP_002468	XLOC_104510	ENSRNOT00000055957	-0.968284126	2.40E-07
TUCP_002468	XLOC_104510	ENSRNOT00000023664	-0.965061947	3.87E-07
TUCP_002468	XLOC_104510	ENSRNOT00000068026	-0.964976493	3.91E-07
TUCP_002468	XLOC_104510	ENSRNOT00000085621	0.95721786	1.05E-06
TUCP_002468	XLOC_104510	ENSRNOT00000007314	-0.953415406	1.60E-06
TUCP_002468	XLOC_104510	ENSRNOT00000014821	-0.985087133	5.67E-09
TUCP_002468	XLOC_104510	ENSRNOT00000058382	0.971332333	1.45E-07
TUCP_002468	XLOC_104510	ENSRNOT00000088599	0.972609958	1.16E-07
TUCP_002468	XLOC_104510	ENSRNOT00000005587	-0.964934053	3.94E-07
TUCP_002468	XLOC_104510	ENSRNOT00000005172	0.95013092	2.23E-06
TUCP_002468	XLOC_104510	ENSRNOT00000017746	-0.955119967	1.33E-06
TUCP_002468	XLOC_104510	ENSRNOT00000023567	-0.957911935	9.69E-07
TUCP_002468	XLOC_104510	ENSRNOT00000070912	-0.960921816	6.72E-07
TUCP_002468	XLOC_104510	ENSRNOT00000003412	-0.966036695	3.36E-07
TUCP_002468	XLOC_104510	ENSRNOT00000050609	-0.962826982	5.25E-07
TUCP_002468	XLOC_104510	ENSRNOT00000015495	-0.959321223	8.19E-07
TUCP_002468	XLOC_104510	ENSRNOT00000005459	0.970083975	1.79E-07
TUCP_002468	XLOC_104510	ENSRNOT00000017776	0.956008925	1.20E-06
TUCP_002468	XLOC_104510	ENSRNOT00000027910	0.970482149	1.68E-07
TUCP_002468	XLOC_104510	ENSRNOT00000037015	0.976206941	5.77E-08
TUCP_002468	XLOC_104510	ENSRNOT00000067135	-0.971930503	1.31E-07
TUCP_002468	XLOC_104510	ENSRNOT00000054976	-0.975679816	6.43E-08
TUCP_002468	XLOC_104510	ENSRNOT00000010516	-0.959434876	8.08E-07
TUCP_002468	XLOC_104510	ENSRNOT00000016372	-0.965224884	3.78E-07
TUCP_002468	XLOC_104510	ENSRNOT00000039730	-0.952344696	1.79E-06
TUCP_002468	XLOC_104510	ENSRNOT00000088202	-0.95673905	1.11E-06
TUCP_002468	XLOC_104510	ENSRNOT00000033677	-0.954548079	1.42E-06
TUCP_002468	XLOC_104510	ENSRNOT00000006802	0.952114537	1.83E-06
TUCP_002468	XLOC_104510	ENSRNOT00000082151	-0.975126446	7.19E-08
TUCP_002468	XLOC_104510	ENSRNOT00000016776	-0.960960077	6.69E-07
TUCP_002468	XLOC_104510	ENSRNOT00000034411	0.952555319	1.75E-06
TUCP_002468	XLOC_104510	ENSRNOT00000001607	0.950314741	2.19E-06
TUCP_002468	XLOC_104510	ENSRNOT00000085971	-0.96951805	1.97E-07
TUCP_002468	XLOC_104510	ENSRNOT00000057537	0.969040926	2.13E-07
TUCP_002468	XLOC_104510	ENSRNOT00000007771	-0.953945315	1.51E-06
TUCP_002468	XLOC_104510	ENSRNOT00000022142	0.96591695	3.42E-07
TUCP_002468	XLOC_104510	ENSRNOT00000033048	-0.966838306	2.99E-07
TUCP_002468	XLOC_104510	ENSRNOT00000015537	0.96236978	5.58E-07
TUCP_002468	XLOC_104510	ENSRNOT00000052120	0.961301634	6.40E-07
TUCP_002468	XLOC_104510	ENSRNOT00000078306	0.962742069	5.31E-07
TUCP_002468	XLOC_104510	ENSRNOT00000015946	-0.95098201	2.05E-06
TUCP_002468	XLOC_104510	ENSRNOT00000023965	-0.972755753	1.13E-07
TUCP_002468	XLOC_104510	ENSRNOT00000090355	-0.968836513	2.20E-07
TUCP_002468	XLOC_104510	ENSRNOT00000029125	0.958374529	9.18E-07
TUCP_002468	XLOC_104510	ENSRNOT00000012528	0.959189855	8.32E-07
TUCP_002468	XLOC_104510	ENSRNOT00000004319	-0.95951049	8.01E-07
TUCP_002468	XLOC_104510	ENSRNOT00000015092	0.954522601	1.42E-06
TUCP_002468	XLOC_104510	ENSRNOT00000015318	-0.962337796	5.60E-07
TUCP_002468	XLOC_104510	ENSRNOT00000065529	0.973941111	9.06E-08

TUCP_002468	XLOC_104510	ENSRNOT00000028907	0.964261183	4.32E-07
TUCP_002468	XLOC_104510	ENSRNOT00000045532	-0.966761011	3.02E-07
TUCP_002468	XLOC_104510	ENSRNOT00000025887	-0.954885703	1.36E-06
TUCP_002468	XLOC_104510	ENSRNOT00000005311	0.962762792	5.30E-07
TUCP_002468	XLOC_104510	ENSRNOT00000005257	0.962456249	5.52E-07
TUCP_002468	XLOC_104510	ENSRNOT00000015799	0.955068302	1.34E-06
TUCP_002468	XLOC_104510	ENSRNOT00000036506	0.961118395	6.56E-07
TUCP_002468	XLOC_104510	ENSRNOT00000005958	0.962262157	5.66E-07
TUCP_002468	XLOC_104510	ENSRNOT00000082998	0.968277105	2.40E-07
TUCP_002468	XLOC_104510	ENSRNOT00000075656	-0.972017554	1.29E-07
TUCP_002468	XLOC_104510	ENSRNOT00000090946	0.954535458	1.42E-06
TUCP_002468	XLOC_104510	ENSRNOT00000015006	0.954865247	1.37E-06
TUCP_002468	XLOC_104510	ENSRNOT00000007350	0.974530111	8.09E-08
TUCP_002468	XLOC_104510	ENSRNOT00000004956	0.952882238	1.69E-06
TUCP_002468	XLOC_104510	ENSRNOT00000018936	-0.951149089	2.02E-06
TUCP_002468	XLOC_104510	ENSRNOT00000083578	0.96958836	1.95E-07
TUCP_002468	XLOC_104510	ENSRNOT00000000181	0.955491527	1.28E-06
TUCP_002468	XLOC_104510	ENSRNOT00000023638	0.979617124	2.68E-08
TUCP_002468	XLOC_104510	ENSRNOT00000059524	-0.956795435	1.10E-06
TUCP_002468	XLOC_104510	ENSRNOT00000017361	0.960802977	6.82E-07
TUCP_002468	XLOC_104510	ENSRNOT00000005220	0.964271591	4.32E-07
TUCP_002468	XLOC_104510	ENSRNOT00000016649	0.962676472	5.36E-07
TUCP_002468	XLOC_104510	ENSRNOT00000074023	0.979772313	2.58E-08
TUCP_002468	XLOC_104510	ENSRNOT00000019465	-0.959302698	8.21E-07
TUCP_002468	XLOC_104510	ENSRNOT00000009073	-0.970199797	1.76E-07
TUCP_002468	XLOC_104510	ENSRNOT00000067170	-0.961803595	6.00E-07
TUCP_002468	XLOC_104510	ENSRNOT00000001586	-0.962999029	5.13E-07
TUCP_002468	XLOC_104510	ENSRNOT00000018341	-0.952342017	1.79E-06
TUCP_002468	XLOC_104510	ENSRNOT00000045926	0.967584019	2.67E-07
TUCP_002468	XLOC_104510	ENSRNOT00000065076	0.967327563	2.78E-07
TUCP_002468	XLOC_104510	ENSRNOT00000013516	0.958691058	8.84E-07
TUCP_002468	XLOC_104510	ENSRNOT00000054685	0.954624204	1.40E-06
TUCP_002468	XLOC_104510	ENSRNOT00000081621	0.963057372	5.09E-07
TUCP_002468	XLOC_104510	ENSRNOT00000012060	0.951762798	1.90E-06
TUCP_002468	XLOC_104510	ENSRNOT00000050549	0.950023156	2.26E-06
TUCP_002468	XLOC_104510	ENSRNOT00000075480	-0.968234084	2.42E-07
TUCP_002468	XLOC_104510	ENSRNOT00000009000	-0.957044392	1.07E-06
TUCP_002468	XLOC_104510	ENSRNOT00000034282	0.952086569	1.83E-06
TUCP_002468	XLOC_104510	ENSRNOT00000027760	0.968249348	2.41E-07
TUCP_002468	XLOC_104510	ENSRNOT00000024402	0.95950186	8.01E-07
TUCP_002468	XLOC_104510	ENSRNOT00000089545	-0.956415494	1.15E-06
TUCP_002468	XLOC_104510	ENSRNOT00000089933	0.9793505	2.86E-08
TUCP_002468	XLOC_104510	ENSRNOT00000083876	-0.957568987	1.01E-06
TUCP_002468	XLOC_104510	ENSRNOT00000003641	0.961294298	6.41E-07
TUCP_002468	XLOC_104510	ENSRNOT00000090912	0.969535867	1.96E-07
TUCP_002468	XLOC_104510	ENSRNOT00000073983	0.951020426	2.04E-06
TUCP_002468	XLOC_104510	ENSRNOT00000067554	0.952952999	1.68E-06
TUCP_002468	XLOC_104510	ENSRNOT00000072208	0.951592042	1.93E-06
TUCP_002468	XLOC_104510	ENSRNOT00000010784	0.951141588	2.02E-06
TUCP_002468	XLOC_104510	ENSRNOT00000084012	0.956374925	1.16E-06
TUCP_002468	XLOC_104510	ENSRNOT00000027232	0.953577221	1.57E-06

TUCP_002468	XLOC_104510	ENSRNOT00000019820	0.968896747	2.18E-07
TUCP_002468	XLOC_104510	ENSRNOT00000011832	-0.963906015	4.54E-07
TUCP_002468	XLOC_104510	ENSRNOT00000021030	-0.952723883	1.72E-06
TUCP_002468	XLOC_104510	ENSRNOT00000013864	-0.962395916	5.56E-07
TUCP_002468	XLOC_104510	ENSRNOT00000077727	-0.96215989	5.73E-07
TUCP_002468	XLOC_104510	ENSRNOT00000011930	0.960008092	7.53E-07
TUCP_002468	XLOC_104510	ENSRNOT00000073492	-0.967540721	2.69E-07
TUCP_002468	XLOC_104510	ENSRNOT00000010541	0.95092328	2.06E-06
TUCP_002468	XLOC_104510	ENSRNOT00000068745	0.950596221	2.13E-06
TUCP_002468	XLOC_104510	ENSRNOT00000092068	0.958725509	8.80E-07
TUCP_002468	XLOC_104510	ENSRNOT00000017949	-0.962047526	5.82E-07
TUCP_002468	XLOC_104510	ENSRNOT00000048781	0.958661607	8.87E-07
TUCP_002468	XLOC_104510	ENSRNOT00000091909	0.980904397	1.94E-08
TUCP_002468	XLOC_104510	ENSRNOT00000013025	-0.960312988	7.25E-07
TUCP_002468	XLOC_104510	ENSRNOT00000003544	-0.968677877	2.25E-07
TUCP_002468	XLOC_104510	ENSRNOT00000021925	0.957027213	1.07E-06
TUCP_002468	XLOC_104510	ENSRNOT00000022194	-0.957024568	1.07E-06
TUCP_002468	XLOC_104510	ENSRNOT00000086119	-0.955931948	1.22E-06
TUCP_002468	XLOC_104510	ENSRNOT00000064452	-0.966231541	3.27E-07
TUCP_002468	XLOC_104510	ENSRNOT00000022190	-0.970162546	1.77E-07
TUCP_002468	XLOC_104510	ENSRNOT00000025913	-0.975967917	6.06E-08
TUCP_002468	XLOC_104510	ENSRNOT00000019386	-0.957373701	1.03E-06
TUCP_002468	XLOC_104510	ENSRNOT00000077220	-0.950703317	2.11E-06
TUCP_002468	XLOC_104510	ENSRNOT00000076230	0.951923791	1.87E-06
TUCP_002468	XLOC_104510	ENSRNOT00000021636	-0.953157158	1.64E-06
TUCP_002468	XLOC_104510	ENSRNOT00000086577	0.971605824	1.39E-07
TUCP_002468	XLOC_104510	ENSRNOT00000025966	0.95746029	1.02E-06
TUCP_002468	XLOC_104510	ENSRNOT00000089611	0.956593582	1.13E-06
TUCP_002468	XLOC_104510	ENSRNOT00000073910	-0.95267507	1.73E-06
TUCP_002468	XLOC_104510	ENSRNOT00000007738	-0.967794132	2.59E-07
TUCP_002468	XLOC_104510	ENSRNOT00000065600	0.965906099	3.43E-07
TUCP_002468	XLOC_104510	ENSRNOT00000008253	-0.951538269	1.94E-06
TUCP_002468	XLOC_104510	ENSRNOT00000025980	-0.952335616	1.79E-06
TUCP_002468	XLOC_104510	ENSRNOT00000034883	-0.972756664	1.13E-07
TUCP_002468	XLOC_104510	ENSRNOT00000010993	0.956086392	1.19E-06
TUCP_002468	XLOC_104510	ENSRNOT00000020155	0.958254384	9.31E-07
TUCP_002468	XLOC_104510	ENSRNOT00000055321	0.971842159	1.33E-07
TUCP_002468	XLOC_104510	ENSRNOT00000000707	-0.954339374	1.45E-06
TUCP_002468	XLOC_104510	ENSRNOT00000005910	-0.965874291	3.44E-07
TUCP_002468	XLOC_104510	ENSRNOT00000078475	0.954951402	1.35E-06
TUCP_002468	XLOC_104510	ENSRNOT00000040169	0.959586291	7.93E-07
TUCP_002468	XLOC_104510	ENSRNOT00000029701	0.973309727	1.02E-07
TUCP_002468	XLOC_104510	ENSRNOT00000026988	-0.95084183	2.08E-06
TUCP_002468	XLOC_104510	ENSRNOT00000036051	0.953891844	1.52E-06
TUCP_002468	XLOC_104510	ENSRNOT00000000013	0.956816518	1.10E-06
TUCP_002468	XLOC_104510	ENSRNOT00000080591	0.972416055	1.20E-07
TUCP_002468	XLOC_104510	ENSRNOT00000072031	-0.951607474	1.93E-06
TUCP_002468	XLOC_104510	ENSRNOT00000030329	-0.963316852	4.92E-07
TUCP_002468	XLOC_104510	ENSRNOT00000037955	-0.950412057	2.17E-06
TUCP_002468	XLOC_104510	ENSRNOT00000067780	-0.965548377	3.61E-07
TUCP_002468	XLOC_104510	ENSRNOT00000039232	0.95669045	1.12E-06

TUCP_002468	XLOC_104510	ENSRNOT00000004999	0.974007523	8.95E-08
TUCP_002468	XLOC_104510	ENSRNOT00000009122	0.961578556	6.18E-07
TUCP_002468	XLOC_104510	ENSRNOT00000013463	0.983997773	8.05E-09
TUCP_002468	XLOC_104510	ENSRNOT00000089418	0.969872875	1.86E-07
TUCP_002468	XLOC_104510	ENSRNOT00000007889	-0.954368682	1.44E-06
TUCP_002468	XLOC_104510	ENSRNOT00000047663	-0.971755008	1.35E-07
TUCP_002468	XLOC_104510	ENSRNOT00000000532	-0.956878376	1.09E-06
TUCP_002468	XLOC_104510	ENSRNOT00000005486	-0.952785169	1.71E-06
TUCP_002468	XLOC_104510	ENSRNOT00000017907	-0.972971316	1.09E-07
TUCP_002468	XLOC_104510	ENSRNOT00000005489	0.96098717	6.67E-07
TUCP_002468	XLOC_104510	ENSRNOT00000025794	-0.977644207	4.24E-08
TUCP_002468	XLOC_104510	ENSRNOT00000058111	-0.95630868	1.17E-06
TUCP_002468	XLOC_104510	ENSRNOT00000084422	0.962219689	5.69E-07
TUCP_002468	XLOC_104510	ENSRNOT00000050834	-0.966103263	3.33E-07
TUCP_002468	XLOC_104510	ENSRNOT00000088279	0.955630123	1.26E-06
TUCP_002468	XLOC_104510	ENSRNOT00000009994	-0.957111954	1.06E-06
TUCP_002468	XLOC_104510	ENSRNOT00000002114	-0.986854944	3.02E-09
TUCP_002468	XLOC_104510	ENSRNOT00000002445	-0.975186808	7.11E-08
TUCP_002468	XLOC_104510	ENSRNOT00000009993	-0.950221676	2.21E-06
TUCP_002468	XLOC_104510	ENSRNOT00000027970	0.976014677	6.01E-08
TUCP_002468	XLOC_104510	ENSRNOT00000077479	-0.95429249	1.45E-06
TUCP_002468	XLOC_104510	ENSRNOT00000027875	0.964397327	4.24E-07
TUCP_002468	XLOC_104510	ENSRNOT00000046215	-0.974947529	7.45E-08
TUCP_002468	XLOC_104510	ENSRNOT00000025009	0.953091647	1.65E-06
TUCP_002468	XLOC_104510	ENSRNOT00000000083	-0.960568151	7.03E-07
TUCP_002468	XLOC_104510	ENSRNOT00000018074	-0.959417665	8.10E-07
TUCP_002468	XLOC_104510	ENSRNOT00000014124	0.95383582	1.53E-06
TUCP_002468	XLOC_104510	ENSRNOT00000000120	0.991116709	4.29E-10
TUCP_002468	XLOC_104510	ENSRNOT00000013632	0.962228505	5.68E-07
TUCP_002468	XLOC_104510	ENSRNOT00000028545	-0.967904445	2.54E-07
TUCP_002468	XLOC_104510	ENSRNOT00000009900	-0.956158402	1.18E-06
TUCP_002468	XLOC_104510	ENSRNOT00000028225	0.958248862	9.31E-07
TUCP_002468	XLOC_104510	ENSRNOT00000019172	-0.954611576	1.41E-06
TUCP_002468	XLOC_104510	ENSRNOT00000026941	0.962485766	5.49E-07
TUCP_002468	XLOC_104510	ENSRNOT00000020994	-0.956984882	1.08E-06
TUCP_002468	XLOC_104510	ENSRNOT00000032244	0.952694259	1.72E-06
TUCP_002468	XLOC_104510	ENSRNOT00000032248	0.972998564	1.08E-07
TUCP_002468	XLOC_104510	ENSRNOT00000019911	-0.957270749	1.04E-06
TUCP_002468	XLOC_104510	ENSRNOT00000040393	0.968472619	2.33E-07
TUCP_002468	XLOC_104510	ENSRNOT00000032236	-0.959876862	7.66E-07
TUCP_002468	XLOC_104510	ENSRNOT00000067901	-0.952354272	1.78E-06
TUCP_002468	XLOC_104510	ENSRNOT00000020272	-0.959232238	8.28E-07
TUCP_002468	XLOC_104510	ENSRNOT00000064700	0.961252769	6.44E-07
TUCP_002468	XLOC_104510	ENSRNOT00000036613	-0.973964384	9.02E-08
TUCP_002468	XLOC_104510	ENSRNOT00000025705	0.977923641	3.98E-08
TUCP_002468	XLOC_104510	ENSRNOT00000052051	0.952342766	1.79E-06
TUCP_002468	XLOC_104510	ENSRNOT00000019983	-0.957886304	9.72E-07
TUCP_002468	XLOC_104510	ENSRNOT00000004140	0.953325304	1.61E-06
TUCP_002468	XLOC_104510	ENSRNOT00000074494	-0.955971574	1.21E-06
TUCP_002468	XLOC_104510	ENSRNOT00000049193	-0.950126756	2.23E-06
TUCP_002468	XLOC_104510	ENSRNOT00000008113	-0.966624713	3.08E-07

TUCP_002468	XLOC_104510	ENSRNOT00000036194	-0.950864318	2.08E-06
TUCP_002468	XLOC_104510	ENSRNOT00000000334	-0.954893912	1.36E-06
TUCP_002468	XLOC_104510	ENSRNOT00000020297	0.952425225	1.77E-06
TUCP_002468	XLOC_104510	ENSRNOT00000032802	-0.953757148	1.54E-06
TUCP_002468	XLOC_104510	ENSRNOT00000078070	0.958874899	8.65E-07
TUCP_002468	XLOC_104510	ENSRNOT00000084031	0.964261296	4.32E-07
TUCP_002468	XLOC_104510	ENSRNOT00000006460	0.956503912	1.14E-06
TUCP_002468	XLOC_104510	ENSRNOT00000020051	0.962040765	5.82E-07
TUCP_002468	XLOC_104510	ENSRNOT00000026279	-0.968438363	2.34E-07
TUCP_002468	XLOC_104510	ENSRNOT00000027117	-0.956021776	1.20E-06
TUCP_002468	XLOC_104510	ENSRNOT00000082914	0.963745522	4.64E-07
TUCP_002468	XLOC_104510	ENSRNOT00000082508	-0.952051823	1.84E-06
TUCP_002468	XLOC_104510	ENSRNOT00000049459	-0.955191899	1.32E-06
TUCP_002468	XLOC_104510	ENSRNOT00000065947	0.953005943	1.67E-06
TUCP_002468	XLOC_104510	ENSRNOT00000071166	0.950311231	2.19E-06
TUCP_002468	XLOC_104510	ENSRNOT00000084567	0.950721251	2.11E-06
TUCP_002468	XLOC_104510	ENSRNOT00000013852	0.964897989	3.96E-07
TUCP_002468	XLOC_104510	ENSRNOT00000013851	-0.963097854	5.07E-07
TUCP_002468	XLOC_104510	ENSRNOT00000001118	0.966894797	2.96E-07
TUCP_002468	XLOC_104510	ENSRNOT00000019180	-0.958966052	8.55E-07
TUCP_002468	XLOC_104510	ENSRNOT00000066204	-0.957960615	9.64E-07
TUCP_002468	XLOC_104510	ENSRNOT00000076105	-0.962803611	5.27E-07
TUCP_002468	XLOC_104510	ENSRNOT00000022746	-0.963887615	4.55E-07
TUCP_002468	XLOC_104510	ENSRNOT00000001507	0.957862427	9.75E-07
TUCP_002468	XLOC_104510	ENSRNOT00000087604	0.952436766	1.77E-06
TUCP_002468	XLOC_104510	ENSRNOT00000083568	0.969534904	1.96E-07
TUCP_002468	XLOC_104510	ENSRNOT00000039177	-0.960288005	7.28E-07
TUCP_002468	XLOC_104510	ENSRNOT00000005620	-0.960949097	6.70E-07
TUCP_002468	XLOC_104510	ENSRNOT00000081047	0.955393967	1.29E-06
TUCP_002468	XLOC_104510	ENSRNOT00000042903	0.965183152	3.80E-07
TUCP_002468	XLOC_104510	ENSRNOT00000037456	0.956303308	1.17E-06
TUCP_002468	XLOC_104510	ENSRNOT00000000477	-0.962137131	5.75E-07
TUCP_002468	XLOC_104510	ENSRNOT00000012881	-0.96158264	6.18E-07
TUCP_002468	XLOC_104510	ENSRNOT00000081226	0.953210122	1.63E-06
TUCP_002468	XLOC_104510	ENSRNOT00000065045	0.969560814	1.96E-07
TUCP_002468	XLOC_104510	ENSRNOT00000056405	0.951104344	2.03E-06
TUCP_002468	XLOC_104510	ENSRNOT00000041456	-0.95053434	2.15E-06
TUCP_002468	XLOC_104510	ENSRNOT00000023989	0.959134306	8.38E-07
TUCP_002468	XLOC_104510	ENSRNOT00000088133	-0.97074067	1.61E-07
TUCP_002468	XLOC_104510	ENSRNOT00000080677	-0.970283505	1.74E-07
TUCP_002468	XLOC_104510	ENSRNOT00000016629	0.966585045	3.10E-07
TUCP_002468	XLOC_104510	ENSRNOT00000016254	-0.962327099	5.61E-07
TUCP_002468	XLOC_104510	ENSRNOT00000083968	-0.951923497	1.87E-06
TUCP_002468	XLOC_104510	ENSRNOT00000037133	0.966268235	3.25E-07
TUCP_002468	XLOC_104510	ENSRNOT00000002837	-0.963636202	4.71E-07
TUCP_002468	XLOC_104510	ENSRNOT00000026831	0.956706529	1.11E-06
TUCP_002468	XLOC_104510	ENSRNOT00000007821	0.953952556	1.51E-06
TUCP_002468	XLOC_104510	ENSRNOT00000032363	0.965922595	3.42E-07
TUCP_002468	XLOC_104510	ENSRNOT00000016445	0.957210022	1.05E-06
TUCP_002468	XLOC_104510	ENSRNOT00000011956	0.953138498	1.64E-06
TUCP_002468	XLOC_104510	ENSRNOT00000078185	-0.961409567	6.32E-07

TUCP_002468	XLOC_104510	ENSRNOT00000024119	0.974082788	8.82E-08
TUCP_002468	XLOC_104510	ENSRNOT00000002187	-0.962761102	5.30E-07
TUCP_002468	XLOC_104510	ENSRNOT00000002152	-0.952644775	1.73E-06
TUCP_002468	XLOC_104510	ENSRNOT00000006824	-0.953631215	1.56E-06
TUCP_002468	XLOC_104510	ENSRNOT00000088509	0.950550107	2.14E-06
TUCP_002468	XLOC_104510	ENSRNOT00000032667	-0.956519524	1.14E-06
TUCP_002468	XLOC_104510	ENSRNOT00000082138	-0.958469023	9.07E-07
TUCP_002468	XLOC_104510	ENSRNOT00000018328	-0.955231986	1.31E-06
TUCP_002468	XLOC_104510	ENSRNOT00000044608	-0.958791902	8.73E-07
TUCP_002468	XLOC_104510	ENSRNOT00000001099	-0.975261281	7.00E-08
TUCP_002468	XLOC_104510	ENSRNOT00000009811	0.953730781	1.54E-06
TUCP_002468	XLOC_104510	ENSRNOT00000085736	-0.956655578	1.12E-06
TUCP_002468	XLOC_104510	ENSRNOT00000045488	0.954043578	1.49E-06
TUCP_002468	XLOC_104510	ENSRNOT00000050119	-0.967188649	2.83E-07
TUCP_002468	XLOC_104510	ENSRNOT00000090853	-0.960915837	6.73E-07
TUCP_002468	XLOC_104510	ENSRNOT00000063821	-0.954825305	1.37E-06
TUCP_002468	XLOC_104510	ENSRNOT00000089211	0.966559641	3.11E-07
TUCP_002468	XLOC_104510	ENSRNOT00000020383	0.960902516	6.74E-07
TUCP_002468	XLOC_104510	ENSRNOT00000073847	-0.951449253	1.96E-06
TUCP_002468	XLOC_104510	ENSRNOT00000015518	-0.961717635	6.07E-07
TUCP_002468	XLOC_104510	ENSRNOT00000000302	0.968307306	2.39E-07
TUCP_002468	XLOC_104510	ENSRNOT00000028181	-0.950297771	2.20E-06
TUCP_002468	XLOC_104510	ENSRNOT00000011675	0.962603387	5.41E-07
TUCP_002468	XLOC_104510	ENSRNOT00000041106	0.958247626	9.32E-07
TUCP_002468	XLOC_104510	ENSRNOT00000015223	0.954403836	1.44E-06
TUCP_002468	XLOC_104510	ENSRNOT00000019713	-0.950336348	2.19E-06
TUCP_002468	XLOC_104510	ENSRNOT00000083632	-0.975192044	7.10E-08
TUCP_002468	XLOC_104510	ENSRNOT00000000717	-0.950871681	2.07E-06
TUCP_002468	XLOC_104510	ENSRNOT00000049725	-0.961001587	6.65E-07
TUCP_002468	XLOC_104510	ENSRNOT00000086122	0.966055827	3.35E-07
TUCP_002468	XLOC_104510	ENSRNOT00000026235	-0.967566216	2.68E-07
TUCP_002468	XLOC_104510	ENSRNOT00000010753	-0.958161556	9.41E-07
TUCP_002468	XLOC_104510	ENSRNOT00000023763	-0.967454763	2.72E-07
TUCP_002468	XLOC_104510	ENSRNOT00000066230	0.956619849	1.12E-06
TUCP_002468	XLOC_104510	ENSRNOT00000003940	-0.982008969	1.44E-08
TUCP_002468	XLOC_104510	ENSRNOT00000009202	0.969146127	2.09E-07
TUCP_002468	XLOC_104510	ENSRNOT00000042799	0.954560277	1.41E-06
TUCP_002468	XLOC_104510	ENSRNOT00000086310	-0.950958546	2.06E-06
TUCP_002468	XLOC_104510	ENSRNOT00000059181	0.971621456	1.38E-07
TUCP_002468	XLOC_104510	ENSRNOT00000017291	-0.976571668	5.34E-08
TUCP_002468	XLOC_104510	ENSRNOT00000003691	-0.979863738	2.52E-08
TUCP_002468	XLOC_104510	ENSRNOT00000038807	0.965565875	3.60E-07
TUCP_002468	XLOC_104510	ENSRNOT00000081678	0.959034093	8.48E-07
TUCP_002468	XLOC_104510	ENSRNOT00000017765	0.952235233	1.81E-06
TUCP_002468	XLOC_104510	ENSRNOT00000017761	0.974859092	7.58E-08
TUCP_002468	XLOC_104510	ENSRNOT00000023543	-0.954494683	1.42E-06
TUCP_002468	XLOC_104510	ENSRNOT00000080608	0.967950959	2.52E-07
TUCP_002468	XLOC_104510	ENSRNOT00000016740	0.961423114	6.31E-07
TUCP_002468	XLOC_104510	ENSRNOT00000080695	0.950164181	2.23E-06
TUCP_002468	XLOC_104510	ENSRNOT00000039341	0.964777517	4.02E-07
TUCP_002468	XLOC_104510	ENSRNOT00000038212	-0.976322514	5.63E-08

TUCP_002468	XLOC_104510	ENSRNOT00000037409	0.957990416	9.60E-07
TUCP_002468	XLOC_104510	ENSRNOT00000028290	-0.965527102	3.62E-07
TUCP_002468	XLOC_104510	ENSRNOT00000042125	0.977718874	4.17E-08
TUCP_002468	XLOC_104510	ENSRNOT00000025460	0.962984472	5.14E-07
TUCP_002468	XLOC_104510	ENSRNOT00000087033	-0.953301415	1.62E-06
TUCP_002468	XLOC_104510	ENSRNOT00000038178	-0.953362544	1.61E-06
TUCP_002468	XLOC_104510	ENSRNOT00000026760	-0.977769739	4.12E-08
TUCP_002468	XLOC_104510	ENSRNOT00000075800	-0.969895908	1.85E-07
TUCP_002468	XLOC_104510	ENSRNOT00000081077	-0.951765723	1.90E-06
TUCP_002468	XLOC_104510	ENSRNOT00000016568	0.96971292	1.91E-07
TUCP_002468	XLOC_104510	ENSRNOT00000027360	-0.951415288	1.96E-06
TUCP_002468	XLOC_104510	ENSRNOT00000024947	-0.969087208	2.11E-07
TUCP_002468	XLOC_104510	ENSRNOT00000005885	-0.965918811	3.42E-07
TUCP_002468	XLOC_104510	ENSRNOT00000027967	0.969202488	2.07E-07
TUCP_002468	XLOC_104510	ENSRNOT00000037039	-0.962866384	5.22E-07
TUCP_002468	XLOC_104510	ENSRNOT00000047210	0.967332734	2.77E-07
TUCP_002468	XLOC_104510	ENSRNOT00000074177	0.950017013	2.26E-06
TUCP_002468	XLOC_104510	ENSRNOT00000036658	-0.969654559	1.93E-07
TUCP_002469	XLOC_104512	ENSRNOT00000089536	-0.959883156	7.65E-07
TUCP_003909	XLOC_172179	ENSRNOT00000038247	0.953487901	1.59E-06
TUCP_003909	XLOC_172179	ENSRNOT00000039809	0.962450436	5.52E-07
TUCP_003909	XLOC_172179	ENSRNOT00000085596	0.950999579	2.05E-06
TUCP_003909	XLOC_172179	ENSRNOT00000004698	0.961035563	6.62E-07
TUCP_003909	XLOC_172179	ENSRNOT00000076285	0.965655113	3.55E-07
TUCP_003909	XLOC_172179	ENSRNOT00000026293	0.964410655	4.24E-07
TUCP_003909	XLOC_172179	ENSRNOT00000002560	0.964804892	4.01E-07
TUCP_003909	XLOC_172179	ENSRNOT00000001222	0.97460399	7.97E-08
TUCP_003909	XLOC_172179	ENSRNOT00000071560	0.960633088	6.97E-07
TUCP_003909	XLOC_172179	ENSRNOT00000005355	0.972069609	1.28E-07
TUCP_003909	XLOC_172179	ENSRNOT00000012156	0.960173699	7.38E-07
TUCP_003909	XLOC_172179	ENSRNOT00000055992	-0.963635651	4.71E-07
TUCP_003909	XLOC_172179	ENSRNOT00000075952	0.958985867	8.53E-07
TUCP_003909	XLOC_172179	ENSRNOT00000005228	0.951449746	1.96E-06
TUCP_003909	XLOC_172179	ENSRNOT00000087429	0.963426364	4.85E-07
TUCP_003909	XLOC_172179	ENSRNOT00000073235	0.956464046	1.14E-06
TUCP_003909	XLOC_172179	ENSRNOT00000009683	0.952975787	1.67E-06
TUCP_003909	XLOC_172179	ENSRNOT00000052288	0.958578895	8.96E-07
TUCP_003909	XLOC_172179	ENSRNOT00000023144	0.968176026	2.44E-07
TUCP_003909	XLOC_172179	ENSRNOT00000080695	0.9561791	1.18E-06
TUCP_003909	XLOC_172179	ENSRNOT00000075311	0.951277413	1.99E-06
TUCP_003909	XLOC_172179	ENSRNOT00000068322	0.967436012	2.73E-07
TUCP_003909	XLOC_172179	ENSRNOT00000078070	0.955102167	1.33E-06
TUCP_003909	XLOC_172179	ENSRNOT00000068633	0.952666173	1.73E-06
TUCP_003909	XLOC_172179	ENSRNOT00000080800	0.963579513	4.75E-07
TUCP_003909	XLOC_172179	ENSRNOT00000002866	-0.970680547	1.62E-07
TUCP_003909	XLOC_172179	ENSRNOT00000071694	0.958240033	9.32E-07
TUCP_003909	XLOC_172179	ENSRNOT00000013570	-0.950612271	2.13E-06
TUCP_003909	XLOC_172179	ENSRNOT00000001449	0.953451933	1.59E-06
TUCP_003909	XLOC_172179	ENSRNOT00000010986	0.952761752	1.71E-06
TUCP_003909	XLOC_172179	ENSRNOT00000065501	0.957763511	9.86E-07
TUCP_003909	XLOC_172179	ENSRNOT00000027454	0.950440445	2.17E-06

TUCP_003909	XLOC_172179	ENSRNOT00000000036	0.975906007	6.14E-08
TUCP_003909	XLOC_172179	ENSRNOT00000014814	0.950623841	2.13E-06
TUCP_003909	XLOC_172179	ENSRNOT00000091417	0.969763936	1.89E-07
TUCP_003909	XLOC_172179	ENSRNOT00000080207	0.969868067	1.86E-07
TUCP_003909	XLOC_172179	ENSRNOT00000061267	0.969899537	1.85E-07
TUCP_003909	XLOC_172179	ENSRNOT00000006055	0.975898032	6.15E-08
TUCP_003909	XLOC_172179	ENSRNOT00000071573	0.979431085	2.80E-08
TUCP_003909	XLOC_172179	ENSRNOT00000005126	-0.973481625	9.88E-08
TUCP_003909	XLOC_172179	ENSRNOT00000065300	0.956819988	1.10E-06
TUCP_003909	XLOC_172179	ENSRNOT00000009280	0.952330503	1.79E-06
TUCP_003909	XLOC_172179	ENSRNOT00000026457	0.964222082	4.35E-07
TUCP_003909	XLOC_172179	ENSRNOT00000003296	0.97305469	1.07E-07
TUCP_003909	XLOC_172179	ENSRNOT00000089724	0.959584478	7.93E-07
TUCP_003909	XLOC_172179	ENSRNOT00000073346	0.952730047	1.72E-06
TUCP_003909	XLOC_172179	ENSRNOT00000003182	0.961294139	6.41E-07
TUCP_003909	XLOC_172179	ENSRNOT00000028646	0.956883537	1.09E-06
TUCP_003909	XLOC_172179	ENSRNOT00000051550	0.982928473	1.11E-08
TUCP_003909	XLOC_172179	ENSRNOT00000035989	0.961391684	6.33E-07
TUCP_003909	XLOC_172179	ENSRNOT00000067867	0.958130395	9.44E-07
TUCP_003909	XLOC_172179	ENSRNOT00000047093	0.951188727	2.01E-06
TUCP_003909	XLOC_172179	ENSRNOT00000080110	0.957419602	1.03E-06
TUCP_003909	XLOC_172179	ENSRNOT00000023858	0.972208991	1.25E-07
TUCP_003909	XLOC_172179	ENSRNOT00000057313	0.969619288	1.94E-07
TUCP_003909	XLOC_172179	ENSRNOT00000073930	0.952920409	1.68E-06
TUCP_003909	XLOC_172179	ENSRNOT00000044395	0.951866345	1.88E-06
TUCP_003909	XLOC_172179	ENSRNOT00000000337	0.97352406	9.80E-08
TUCP_003909	XLOC_172179	ENSRNOT00000083799	0.952328496	1.79E-06
TUCP_003909	XLOC_172179	ENSRNOT00000024548	0.951479766	1.95E-06
TUCP_002465	XLOC_104506	ENSRNOT00000055536	0.959577628	7.94E-07
TUCP_002465	XLOC_104506	ENSRNOT00000009975	0.959375642	8.14E-07
TUCP_002465	XLOC_104506	ENSRNOT00000006460	0.957112779	1.06E-06
TUCP_002465	XLOC_104506	ENSRNOT00000012340	0.962825282	5.25E-07
TUCP_002465	XLOC_104506	ENSRNOT00000006217	0.953341015	1.61E-06
TUCP_002465	XLOC_104506	ENSRNOT00000003087	0.970652678	1.63E-07
TUCP_002465	XLOC_104506	ENSRNOT00000082914	0.973500507	9.84E-08
TUCP_002465	XLOC_104506	ENSRNOT00000073493	0.971280645	1.47E-07
TUCP_002465	XLOC_104506	ENSRNOT00000011589	0.956686266	1.12E-06
TUCP_002465	XLOC_104506	ENSRNOT00000079138	0.962878225	5.22E-07
TUCP_002465	XLOC_104506	ENSRNOT00000071998	0.956514601	1.14E-06
TUCP_002465	XLOC_104506	ENSRNOT00000029508	0.965365386	3.70E-07
TUCP_002465	XLOC_104506	ENSRNOT00000036051	0.974587314	8.00E-08
TUCP_002465	XLOC_104506	ENSRNOT00000084567	0.968249478	2.41E-07
TUCP_002465	XLOC_104506	ENSRNOT00000008361	0.956128284	1.19E-06
TUCP_002465	XLOC_104506	ENSRNOT00000042638	0.951619847	1.92E-06
TUCP_002465	XLOC_104506	ENSRNOT00000036357	0.983965537	8.13E-09
TUCP_002465	XLOC_104506	ENSRNOT00000046951	0.958515431	9.02E-07
TUCP_002465	XLOC_104506	ENSRNOT00000045926	0.95904959	8.47E-07
TUCP_002465	XLOC_104506	ENSRNOT00000010419	0.955921132	1.22E-06
TUCP_002465	XLOC_104506	ENSRNOT00000065076	0.972753681	1.13E-07
TUCP_002465	XLOC_104506	ENSRNOT00000057738	0.953168928	1.64E-06
TUCP_002465	XLOC_104506	ENSRNOT00000013516	0.955749541	1.24E-06

TUCP_002465	XLOC_104506	ENSRNOT00000054685	0.969194147	2.07E-07
TUCP_002465	XLOC_104506	ENSRNOT00000012060	0.952600768	1.74E-06
TUCP_002465	XLOC_104506	ENSRNOT00000073370	0.966070271	3.35E-07
TUCP_002465	XLOC_104506	ENSRNOT00000020929	0.977707545	4.18E-08
TUCP_002465	XLOC_104506	ENSRNOT00000009009	0.971690192	1.37E-07
TUCP_002465	XLOC_104506	ENSRNOT00000037902	0.954772606	1.38E-06
TUCP_002465	XLOC_104506	ENSRNOT00000075480	-0.950332913	2.19E-06
TUCP_002465	XLOC_104506	ENSRNOT00000020051	0.961830487	5.98E-07
TUCP_002465	XLOC_104506	ENSRNOT00000034282	0.955367326	1.29E-06
TUCP_002465	XLOC_104506	ENSRNOT00000012755	0.99216863	2.29E-10
TUCP_002465	XLOC_104506	ENSRNOT00000065871	0.978504187	3.49E-08
TUCP_002465	XLOC_104506	ENSRNOT00000024677	0.958418348	9.13E-07
TUCP_002465	XLOC_104506	ENSRNOT00000067161	0.962709351	5.33E-07
TUCP_002465	XLOC_104506	ENSRNOT00000001077	0.952665091	1.73E-06
TUCP_002465	XLOC_104506	ENSRNOT00000044511	0.960266077	7.30E-07
TUCP_002465	XLOC_104506	ENSRNOT00000015062	0.967421249	2.74E-07
TUCP_002465	XLOC_104506	ENSRNOT00000081226	0.975673209	6.44E-08
TUCP_002465	XLOC_104506	ENSRNOT00000027544	0.957782347	9.84E-07
TUCP_002465	XLOC_104506	ENSRNOT00000081631	0.958787741	8.74E-07
TUCP_002465	XLOC_104506	ENSRNOT00000046552	0.977405366	4.47E-08
TUCP_002465	XLOC_104506	ENSRNOT00000062057	0.957559959	1.01E-06
TUCP_002465	XLOC_104506	ENSRNOT00000023613	0.96545192	3.66E-07
TUCP_002465	XLOC_104506	ENSRNOT00000092066	-0.95789833	9.71E-07
TUCP_002465	XLOC_104506	ENSRNOT00000071381	0.952673843	1.73E-06
TUCP_002465	XLOC_104506	ENSRNOT00000048781	0.956159512	1.18E-06
TUCP_002465	XLOC_104506	ENSRNOT00000013867	0.971115385	1.51E-07
TUCP_002465	XLOC_104506	ENSRNOT00000021802	0.957748183	9.88E-07
TUCP_002465	XLOC_104506	ENSRNOT00000018640	0.971981936	1.30E-07
TUCP_002465	XLOC_104506	ENSRNOT00000085334	0.958119343	9.46E-07
TUCP_002465	XLOC_104506	ENSRNOT00000081604	0.957218826	1.05E-06
TUCP_002465	XLOC_104506	ENSRNOT00000059246	0.952868424	1.69E-06
TUCP_002465	XLOC_104506	ENSRNOT00000076230	0.950805185	2.09E-06
TUCP_002465	XLOC_104506	ENSRNOT00000066952	0.974712999	7.80E-08
TUCP_002465	XLOC_104506	ENSRNOT00000026075	0.987597546	2.26E-09
TUCP_002465	XLOC_104506	ENSRNOT00000089611	0.965588454	3.59E-07
TUCP_002465	XLOC_104506	ENSRNOT00000074054	0.970865377	1.57E-07
TUCP_002465	XLOC_104506	ENSRNOT00000045063	0.966877479	2.97E-07
TUCP_002465	XLOC_104506	ENSRNOT00000076400	0.97639095	5.55E-08
TUCP_002465	XLOC_104506	ENSRNOT00000030082	0.95574496	1.24E-06
TUCP_002465	XLOC_104506	ENSRNOT00000001158	0.959782303	7.74E-07
TUCP_002465	XLOC_104506	ENSRNOT00000005172	0.976080422	5.92E-08
TUCP_002465	XLOC_104506	ENSRNOT00000060494	0.963824386	4.59E-07
TUCP_002465	XLOC_104506	ENSRNOT00000021715	0.963684644	4.68E-07
TUCP_002465	XLOC_104506	ENSRNOT00000087138	0.968318913	2.38E-07
TUCP_002465	XLOC_104506	ENSRNOT00000039911	0.958885544	8.63E-07
TUCP_002465	XLOC_104506	ENSRNOT00000007461	0.973597855	9.67E-08
TUCP_002465	XLOC_104506	ENSRNOT00000060977	0.976438685	5.50E-08
TUCP_002465	XLOC_104506	ENSRNOT00000065615	0.976561993	5.36E-08
TUCP_002465	XLOC_104506	ENSRNOT00000037015	0.963404855	4.86E-07
TUCP_002465	XLOC_104506	ENSRNOT00000027917	0.955505455	1.27E-06
TUCP_002465	XLOC_104506	ENSRNOT00000011956	0.980258529	2.28E-08

TUCP_002465	XLOC_104506	ENSRNOT00000022716	0.950874647	2.07E-06
TUCP_002465	XLOC_104506	ENSRNOT00000011675	0.982400891	1.29E-08
TUCP_002465	XLOC_104506	ENSRNOT00000033359	0.950518102	2.15E-06
TUCP_002465	XLOC_104506	ENSRNOT00000002974	0.953021705	1.67E-06
TUCP_002465	XLOC_104506	ENSRNOT00000060476	0.970552702	1.66E-07
TUCP_002465	XLOC_104506	ENSRNOT00000068156	0.966488555	3.15E-07
TUCP_002465	XLOC_104506	ENSRNOT00000066032	0.95549885	1.28E-06
TUCP_002465	XLOC_104506	ENSRNOT00000049285	-0.950378463	2.18E-06
TUCP_002465	XLOC_104506	ENSRNOT00000026392	0.959308782	8.20E-07
TUCP_002465	XLOC_104506	ENSRNOT00000014320	0.978013551	3.90E-08
TUCP_002465	XLOC_104506	ENSRNOT00000005770	0.965935992	3.41E-07
TUCP_002465	XLOC_104506	ENSRNOT00000017504	0.962355467	5.59E-07
TUCP_002465	XLOC_104506	ENSRNOT00000025037	0.978908738	3.17E-08
TUCP_002465	XLOC_104506	ENSRNOT00000012386	0.953081465	1.65E-06
TUCP_002465	XLOC_104506	ENSRNOT00000006802	0.963954824	4.51E-07
TUCP_002465	XLOC_104506	ENSRNOT00000009202	0.96786113	2.56E-07
TUCP_002465	XLOC_104506	ENSRNOT00000010369	0.965169306	3.81E-07
TUCP_002465	XLOC_104506	ENSRNOT00000013524	0.960392718	7.18E-07
TUCP_002465	XLOC_104506	ENSRNOT00000017765	0.960120958	7.43E-07
TUCP_002465	XLOC_104506	ENSRNOT00000064039	0.95709746	1.07E-06
TUCP_002465	XLOC_104506	ENSRNOT00000006168	0.981751424	1.55E-08
TUCP_002465	XLOC_104506	ENSRNOT00000017829	0.957649973	9.99E-07
TUCP_002465	XLOC_104506	ENSRNOT00000005719	0.952984899	1.67E-06
TUCP_002465	XLOC_104506	ENSRNOT00000023014	0.960592695	7.00E-07
TUCP_002465	XLOC_104506	ENSRNOT00000026518	0.961126233	6.55E-07
TUCP_002465	XLOC_104506	ENSRNOT00000036137	0.972640485	1.15E-07
TUCP_002465	XLOC_104506	ENSRNOT00000027875	0.963772436	4.62E-07
TUCP_002465	XLOC_104506	ENSRNOT00000028685	0.981010521	1.88E-08
TUCP_002465	XLOC_104506	ENSRNOT00000085517	0.97717182	4.70E-08
TUCP_002465	XLOC_104506	ENSRNOT00000007369	0.961067184	6.60E-07
TUCP_002465	XLOC_104506	ENSRNOT00000085466	0.950779987	2.09E-06
TUCP_002465	XLOC_104506	ENSRNOT00000012528	0.96425073	4.33E-07
TUCP_002465	XLOC_104506	ENSRNOT00000027476	0.979257112	2.92E-08
TUCP_002465	XLOC_104506	ENSRNOT00000017998	0.965911634	3.42E-07
TUCP_002465	XLOC_104506	ENSRNOT00000027170	0.963650164	4.70E-07
TUCP_002465	XLOC_104506	ENSRNOT00000006071	0.953609815	1.56E-06
TUCP_002465	XLOC_104506	ENSRNOT00000008764	0.975582228	6.56E-08
TUCP_002465	XLOC_104506	ENSRNOT00000028907	0.952705665	1.72E-06
TUCP_002465	XLOC_104506	ENSRNOT00000079128	0.988504367	1.55E-09
TUCP_002465	XLOC_104506	ENSRNOT00000081678	0.956490005	1.14E-06
TUCP_002465	XLOC_104506	ENSRNOT00000051394	0.972311747	1.22E-07
TUCP_002466	XLOC_104506	ENSRNOT00000001215	0.961384515	6.34E-07
TUCP_002466	XLOC_104506	ENSRNOT00000006188	-0.961356855	6.36E-07
TUCP_002466	XLOC_104506	ENSRNOT00000061233	-0.95058576	2.14E-06
TUCP_002466	XLOC_104506	ENSRNOT00000026941	0.957526337	1.01E-06
TUCP_002466	XLOC_104506	ENSRNOT00000013745	-0.968806339	2.21E-07
TUCP_002466	XLOC_104506	ENSRNOT00000075397	0.966902243	2.96E-07
TUCP_002466	XLOC_104506	ENSRNOT00000012617	-0.968627625	2.27E-07
TUCP_002466	XLOC_104506	ENSRNOT00000032926	-0.960563701	7.03E-07
TUCP_002466	XLOC_104506	ENSRNOT00000019104	-0.964791348	4.02E-07
TUCP_002466	XLOC_104506	ENSRNOT00000031904	0.950377237	2.18E-06

TUCP_002466	XLOC_104506	ENSRNOT00000003891	0.950579764	2.14E-06
TUCP_002466	XLOC_104506	ENSRNOT00000001691	0.974228999	8.57E-08
TUCP_002466	XLOC_104506	ENSRNOT00000089319	0.950152396	2.23E-06
TUCP_002466	XLOC_104506	ENSRNOT00000086706	0.954124923	1.48E-06
TUCP_002466	XLOC_104506	ENSRNOT00000006186	0.950220444	2.21E-06
TUCP_002466	XLOC_104506	ENSRNOT00000045233	-0.961355259	6.36E-07
TUCP_002466	XLOC_104506	ENSRNOT00000030850	0.964011882	4.48E-07
TUCP_002466	XLOC_104506	ENSRNOT00000039232	0.950389462	2.18E-06
TUCP_002466	XLOC_104506	ENSRNOT00000033017	0.968709913	2.24E-07
TUCP_002466	XLOC_104506	ENSRNOT00000055540	0.963742496	4.64E-07
TUCP_002466	XLOC_104506	ENSRNOT00000066370	0.955783493	1.24E-06
TUCP_002466	XLOC_104506	ENSRNOT00000085251	-0.97613779	5.85E-08
TUCP_002466	XLOC_104506	ENSRNOT00000065304	-0.958821935	8.70E-07
TUCP_002466	XLOC_104506	ENSRNOT00000065300	0.959707326	7.82E-07
TUCP_002466	XLOC_104506	ENSRNOT00000045699	0.958165802	9.41E-07
TUCP_002466	XLOC_104506	ENSRNOT00000078816	0.960051457	7.49E-07
TUCP_002466	XLOC_104506	ENSRNOT00000064401	0.956792824	1.10E-06
TUCP_002466	XLOC_104506	ENSRNOT00000065971	-0.957485661	1.02E-06
TUCP_002466	XLOC_104506	ENSRNOT00000057160	0.95489447	1.36E-06
TUCP_002466	XLOC_104506	ENSRNOT00000077999	0.950199019	2.22E-06
TUCP_002466	XLOC_104506	ENSRNOT00000024266	0.958110665	9.47E-07
TUCP_002466	XLOC_104506	ENSRNOT00000085228	0.961952771	5.89E-07
TUCP_002466	XLOC_104506	ENSRNOT00000015525	-0.950898167	2.07E-06
TUCP_002466	XLOC_104506	ENSRNOT00000015188	0.953134179	1.65E-06
TUCP_002466	XLOC_104506	ENSRNOT00000040629	0.959060618	8.45E-07
TUCP_002466	XLOC_104506	ENSRNOT00000005292	0.956061149	1.20E-06
TUCP_002466	XLOC_104506	ENSRNOT00000005834	0.951304867	1.99E-06
TUCP_002466	XLOC_104506	ENSRNOT00000005297	0.952650226	1.73E-06
TUCP_002466	XLOC_104506	ENSRNOT00000000048	0.960516808	7.07E-07
TUCP_002466	XLOC_104506	ENSRNOT00000008655	0.959138152	8.38E-07
TUCP_002466	XLOC_104506	ENSRNOT00000076181	-0.952709834	1.72E-06
TUCP_002466	XLOC_104506	ENSRNOT00000087278	-0.950399402	2.17E-06
TUCP_002466	XLOC_104506	ENSRNOT00000023619	0.954220721	1.47E-06
TUCP_002466	XLOC_104506	ENSRNOT00000084709	0.95868069	8.85E-07
TUCP_002466	XLOC_104506	ENSRNOT00000068033	0.960659832	6.95E-07
TUCP_002466	XLOC_104506	ENSRNOT00000026481	0.958535289	9.00E-07
TUCP_002466	XLOC_104506	ENSRNOT00000082709	-0.963252444	4.96E-07
TUCP_002466	XLOC_104506	ENSRNOT00000025856	-0.959197003	8.32E-07
TUCP_002466	XLOC_104506	ENSRNOT00000026643	0.972403017	1.20E-07
TUCP_002466	XLOC_104506	ENSRNOT00000001403	0.968612863	2.28E-07
TUCP_002466	XLOC_104506	ENSRNOT00000016132	-0.971195498	1.49E-07
TUCP_002466	XLOC_104506	ENSRNOT00000013067	-0.957219592	1.05E-06
TUCP_002466	XLOC_104506	ENSRNOT00000026398	-0.95034053	2.19E-06
TUCP_002466	XLOC_104506	ENSRNOT00000037844	-0.951573445	1.93E-06
TUCP_002466	XLOC_104506	ENSRNOT00000048063	0.958465251	9.08E-07
TUCP_002466	XLOC_104506	ENSRNOT00000041815	-0.975035424	7.32E-08
TUCP_002466	XLOC_104506	ENSRNOT00000033975	0.978680603	3.35E-08
TUCP_002466	XLOC_104506	ENSRNOT00000079895	0.95552633	1.27E-06
TUCP_002466	XLOC_104506	ENSRNOT00000084031	0.970531418	1.67E-07
TUCP_002466	XLOC_104506	ENSRNOT00000011880	0.954506571	1.42E-06
TUCP_002466	XLOC_104506	ENSRNOT00000023989	0.959526835	7.99E-07

TUCP_002466	XLOC_104506	ENSRNOT00000021717	0.954672964	1.40E-06
TUCP_002466	XLOC_104506	ENSRNOT00000021158	0.953016918	1.67E-06
TUCP_002466	XLOC_104506	ENSRNOT00000050515	0.964396826	4.24E-07
TUCP_002466	XLOC_104506	ENSRNOT00000011401	-0.95780938	9.81E-07
TUCP_002466	XLOC_104506	ENSRNOT00000055602	-0.955614654	1.26E-06
TUCP_002466	XLOC_104506	ENSRNOT00000020951	0.965850134	3.45E-07
TUCP_002466	XLOC_104506	ENSRNOT00000082146	-0.958359434	9.19E-07
TUCP_002466	XLOC_104506	ENSRNOT00000031555	-0.951281714	1.99E-06
TUCP_002466	XLOC_104506	ENSRNOT00000011563	-0.958417977	9.13E-07
TUCP_002466	XLOC_104506	ENSRNOT00000000263	0.95343526	1.59E-06
TUCP_002466	XLOC_104506	ENSRNOT00000023876	0.963020256	5.12E-07
TUCP_002466	XLOC_104506	ENSRNOT00000002187	-0.951044772	2.04E-06
TUCP_002466	XLOC_104506	ENSRNOT00000049285	-0.957928265	9.67E-07
TUCP_002466	XLOC_104506	ENSRNOT00000003568	0.957553946	1.01E-06
TUCP_002466	XLOC_104506	ENSRNOT00000006366	-0.954788594	1.38E-06
TUCP_002466	XLOC_104506	ENSRNOT00000074649	0.95772863	9.90E-07
TUCP_002466	XLOC_104506	ENSRNOT00000077146	-0.970163542	1.77E-07
TUCP_002466	XLOC_104506	ENSRNOT00000028801	0.959412732	8.10E-07
TUCP_002466	XLOC_104506	ENSRNOT00000060002	-0.957171117	1.06E-06
TUCP_002466	XLOC_104506	ENSRNOT00000089673	-0.960533436	7.06E-07
TUCP_002466	XLOC_104506	ENSRNOT00000014924	-0.956984792	1.08E-06
TUCP_002466	XLOC_104506	ENSRNOT00000023977	-0.962544364	5.45E-07
TUCP_002466	XLOC_104506	ENSRNOT00000066133	-0.965632312	3.56E-07
TUCP_002466	XLOC_104506	ENSRNOT00000017420	-0.950796387	2.09E-06
TUCP_002466	XLOC_104506	ENSRNOT00000006765	-0.953535615	1.58E-06
TUCP_002466	XLOC_104506	ENSRNOT00000086893	-0.951060758	2.04E-06
TUCP_002466	XLOC_104506	ENSRNOT00000030692	-0.960058037	7.49E-07
TUCP_002466	XLOC_104506	ENSRNOT00000071192	-0.950743492	2.10E-06
TUCP_002466	XLOC_104506	ENSRNOT00000080443	0.960230804	7.33E-07
TUCP_002466	XLOC_104506	ENSRNOT00000080110	0.961641125	6.13E-07
TUCP_002466	XLOC_104506	ENSRNOT00000015624	0.963919679	4.53E-07
TUCP_002466	XLOC_104506	ENSRNOT00000073235	0.958624165	8.91E-07
TUCP_002466	XLOC_104506	ENSRNOT00000085295	-0.950591111	2.13E-06
TUCP_002466	XLOC_104506	ENSRNOT00000008222	-0.953452543	1.59E-06
TUCP_002466	XLOC_104506	ENSRNOT00000035474	0.962321237	5.61E-07
TUCP_002466	XLOC_104506	ENSRNOT00000039102	0.962865153	5.23E-07
TUCP_002466	XLOC_104506	ENSRNOT00000023807	-0.96981906	1.87E-07
TUCP_002466	XLOC_104506	ENSRNOT00000078856	-0.973157435	1.05E-07
TUCP_002466	XLOC_104506	ENSRNOT00000091205	0.952962727	1.68E-06
TUCP_002466	XLOC_104506	ENSRNOT00000007369	0.95836469	9.19E-07
TUCP_002466	XLOC_104506	ENSRNOT00000004787	-0.965039794	3.88E-07
TUCP_002466	XLOC_104506	ENSRNOT00000060698	-0.956937932	1.08E-06
TUCP_002466	XLOC_104506	ENSRNOT00000058351	0.959218077	8.30E-07
TUCP_002466	XLOC_104506	ENSRNOT00000022000	-0.966180718	3.29E-07
TUCP_002466	XLOC_104506	ENSRNOT00000010748	0.963217078	4.99E-07
TUCP_002466	XLOC_104506	ENSRNOT00000080207	0.954114164	1.48E-06
TUCP_002466	XLOC_104506	ENSRNOT00000023537	-0.96413514	4.40E-07
TUCP_002466	XLOC_104506	ENSRNOT00000070912	-0.951829895	1.88E-06
TUCP_002466	XLOC_104506	ENSRNOT00000060595	-0.957098085	1.06E-06
TUCP_002466	XLOC_104506	ENSRNOT00000017776	0.962717544	5.33E-07
TUCP_002466	XLOC_104506	ENSRNOT00000059807	0.961992356	5.86E-07

TUCP_002466	XLOC_104506	ENSRNOT00000008982	0.957826236	9.79E-07
TUCP_002466	XLOC_104506	ENSRNOT00000047192	-0.970772693	1.60E-07
TUCP_002466	XLOC_104506	ENSRNOT00000022097	0.95255045	1.75E-06
TUCP_002466	XLOC_104506	ENSRNOT00000008987	-0.973863195	9.19E-08
TUCP_002466	XLOC_104506	ENSRNOT00000090299	0.951022081	2.04E-06
TUCP_002466	XLOC_104506	ENSRNOT00000090355	-0.966978343	2.93E-07
TUCP_002466	XLOC_104506	ENSRNOT00000065143	0.95242184	1.77E-06
TUCP_002466	XLOC_104506	ENSRNOT00000027083	0.965559613	3.60E-07
TUCP_002466	XLOC_104506	ENSRNOT00000063930	-0.956427688	1.15E-06
TUCP_002466	XLOC_104506	ENSRNOT00000006067	0.950248125	2.21E-06
TUCP_002466	XLOC_104506	ENSRNOT00000001290	0.970324501	1.72E-07
TUCP_002466	XLOC_104506	ENSRNOT00000001855	0.971181172	1.49E-07
TUCP_002466	XLOC_104506	ENSRNOT00000009563	-0.972397448	1.20E-07
TUCP_002466	XLOC_104506	ENSRNOT00000038178	-0.952238643	1.81E-06
TUCP_002466	XLOC_104506	ENSRNOT00000055683	0.965726889	3.52E-07
TUCP_002466	XLOC_104506	ENSRNOT00000081981	0.965439454	3.66E-07
TUCP_002466	XLOC_104506	ENSRNOT00000003326	-0.965205913	3.79E-07
TUCP_002466	XLOC_104506	ENSRNOT00000025079	-0.954538396	1.42E-06
TUCP_002466	XLOC_104506	ENSRNOT00000048754	-0.954070298	1.49E-06
TUCP_002466	XLOC_104506	ENSRNOT00000044628	0.962211965	5.69E-07
TUCP_002466	XLOC_104506	ENSRNOT00000012699	-0.958988652	8.53E-07
TUCP_002466	XLOC_104506	ENSRNOT00000004007	-0.950949934	2.06E-06
TUCP_002466	XLOC_104506	ENSRNOT00000057898	-0.970216299	1.76E-07
TUCP_002466	XLOC_104506	ENSRNOT00000091602	-0.965561499	3.60E-07
TUCP_002466	XLOC_104506	ENSRNOT00000056100	-0.972791804	1.12E-07
TUCP_002466	XLOC_104506	ENSRNOT00000009811	0.964752804	4.04E-07
TUCP_002466	XLOC_104506	ENSRNOT00000036838	-0.967962209	2.52E-07
TUCP_002466	XLOC_104506	ENSRNOT00000009817	-0.952536215	1.75E-06
TUCP_002466	XLOC_104506	ENSRNOT00000090129	-0.956646071	1.12E-06
TUCP_002466	XLOC_104506	ENSRNOT00000077231	0.954658567	1.40E-06
TUCP_002466	XLOC_104506	ENSRNOT00000030798	-0.95224632	1.80E-06
TUCP_002466	XLOC_104506	ENSRNOT00000060696	0.958695692	8.83E-07
TUCP_002466	XLOC_104506	ENSRNOT00000073643	-0.961285186	6.42E-07
TUCP_002466	XLOC_104506	ENSRNOT00000024402	0.951403342	1.97E-06
TUCP_002466	XLOC_104506	ENSRNOT00000052002	0.954139865	1.48E-06
TUCP_002466	XLOC_104506	ENSRNOT00000011654	-0.951798098	1.89E-06
TUCP_002466	XLOC_104506	ENSRNOT00000022502	-0.961879182	5.95E-07
TUCP_002466	XLOC_104506	ENSRNOT00000026928	-0.958296987	9.26E-07
TUCP_002466	XLOC_104506	ENSRNOT00000005329	-0.957414082	1.03E-06
TUCP_002466	XLOC_104506	ENSRNOT00000087443	0.967303562	2.79E-07
TUCP_002466	XLOC_104506	ENSRNOT00000009900	-0.957158486	1.06E-06
TUCP_002466	XLOC_104506	ENSRNOT00000024352	0.960682738	6.93E-07
TUCP_002466	XLOC_104506	ENSRNOT00000083220	0.960214752	7.34E-07
TUCP_002466	XLOC_104506	ENSRNOT00000003780	0.960902041	6.74E-07
TUCP_002466	XLOC_104506	ENSRNOT00000045946	0.968276605	2.40E-07
TUCP_002466	XLOC_104506	ENSRNOT00000017254	-0.950255599	2.21E-06
TUCP_002466	XLOC_104506	ENSRNOT00000016730	-0.956947713	1.08E-06
TUCP_002466	XLOC_104506	ENSRNOT00000085621	0.96730818	2.78E-07
TUCP_002466	XLOC_104506	ENSRNOT00000014821	-0.975185385	7.11E-08
TUCP_002466	XLOC_104506	ENSRNOT00000088599	0.97815428	3.78E-08
TUCP_002466	XLOC_104506	ENSRNOT00000026531	-0.951312597	1.99E-06

TUCP_002466	XLOC_104506	ENSRNOT00000083351	-0.957314381	1.04E-06
TUCP_002466	XLOC_104506	ENSRNOT00000050609	-0.962047646	5.82E-07
TUCP_002466	XLOC_104506	ENSRNOT00000005459	0.959359284	8.15E-07
TUCP_002466	XLOC_104506	ENSRNOT00000027910	0.963728235	4.65E-07
TUCP_002466	XLOC_104506	ENSRNOT00000067135	-0.971760477	1.35E-07
TUCP_002466	XLOC_104506	ENSRNOT00000011270	0.95053881	2.15E-06
TUCP_002466	XLOC_104506	ENSRNOT00000003770	-0.960913407	6.73E-07
TUCP_002466	XLOC_104506	ENSRNOT00000010516	-0.951411945	1.97E-06
TUCP_002466	XLOC_104506	ENSRNOT00000016191	0.956795077	1.10E-06
TUCP_002466	XLOC_104506	ENSRNOT00000067901	-0.961071838	6.59E-07
TUCP_002466	XLOC_104506	ENSRNOT00000039730	-0.960504092	7.08E-07
TUCP_002466	XLOC_104506	ENSRNOT00000002173	-0.950950941	2.06E-06
TUCP_002466	XLOC_104506	ENSRNOT00000054976	-0.964253121	4.33E-07
TUCP_002466	XLOC_104506	ENSRNOT00000038212	-0.964094868	4.42E-07
TUCP_002466	XLOC_104506	ENSRNOT00000082151	-0.960580342	7.02E-07
TUCP_002466	XLOC_104506	ENSRNOT00000020272	-0.962102204	5.78E-07
TUCP_002466	XLOC_104506	ENSRNOT00000001607	0.963158358	5.02E-07
TUCP_002466	XLOC_104506	ENSRNOT00000090057	0.950252039	2.21E-06
TUCP_002466	XLOC_104506	ENSRNOT00000085971	-0.982779447	1.16E-08
TUCP_002466	XLOC_104506	ENSRNOT00000057537	0.953259636	1.62E-06
TUCP_002466	XLOC_104506	ENSRNOT00000007771	-0.957873771	9.73E-07
TUCP_002466	XLOC_104506	ENSRNOT00000022142	0.964536628	4.16E-07
TUCP_002466	XLOC_104506	ENSRNOT00000033048	-0.95694099	1.08E-06
TUCP_002466	XLOC_104506	ENSRNOT00000015537	0.950958474	2.06E-06
TUCP_002466	XLOC_104506	ENSRNOT00000023965	-0.957668306	9.97E-07
TUCP_002466	XLOC_104506	ENSRNOT00000029125	0.954121648	1.48E-06
TUCP_002466	XLOC_104506	ENSRNOT00000004319	-0.969960736	1.83E-07
TUCP_002466	XLOC_104506	ENSRNOT00000005387	0.959526289	7.99E-07
TUCP_002466	XLOC_104506	ENSRNOT00000015092	0.959616208	7.90E-07
TUCP_002466	XLOC_104506	ENSRNOT00000015318	-0.962355051	5.59E-07
TUCP_002466	XLOC_104506	ENSRNOT00000083632	-0.972351762	1.21E-07
TUCP_002466	XLOC_104506	ENSRNOT00000045532	-0.960175292	7.38E-07
TUCP_002466	XLOC_104506	ENSRNOT00000005257	0.959761864	7.76E-07
TUCP_002466	XLOC_104506	ENSRNOT00000015799	0.965640662	3.56E-07
TUCP_002466	XLOC_104506	ENSRNOT00000036506	0.977841317	4.05E-08
TUCP_002466	XLOC_104506	ENSRNOT00000005958	0.969364536	2.02E-07
TUCP_002466	XLOC_104506	ENSRNOT00000082998	0.960457348	7.12E-07
TUCP_002466	XLOC_104506	ENSRNOT00000075656	-0.975402684	6.80E-08
TUCP_002466	XLOC_104506	ENSRNOT00000015006	0.953259723	1.62E-06
TUCP_002466	XLOC_104506	ENSRNOT00000023638	0.975476372	6.70E-08
TUCP_002466	XLOC_104506	ENSRNOT00000059524	-0.962161123	5.73E-07
TUCP_002466	XLOC_104506	ENSRNOT00000017361	0.950024756	2.26E-06
TUCP_002466	XLOC_104506	ENSRNOT00000005220	0.965631614	3.56E-07
TUCP_002466	XLOC_104506	ENSRNOT00000023144	0.952124362	1.83E-06
TUCP_002466	XLOC_104506	ENSRNOT00000045326	0.958492663	9.05E-07
TUCP_002466	XLOC_104506	ENSRNOT00000016649	0.968992728	2.14E-07
TUCP_002466	XLOC_104506	ENSRNOT00000074023	0.976442475	5.49E-08
TUCP_002466	XLOC_104506	ENSRNOT00000019465	-0.962163871	5.73E-07
TUCP_002466	XLOC_104506	ENSRNOT00000001586	-0.961692652	6.09E-07
TUCP_002466	XLOC_104506	ENSRNOT00000012187	0.953548313	1.58E-06
TUCP_002466	XLOC_104506	ENSRNOT00000024969	0.959109267	8.41E-07

TUCP_002466	XLOC_104506	ENSRNOT00000013049	0.962401566	5.56E-07
TUCP_002466	XLOC_104506	ENSRNOT00000013695	-0.950373556	2.18E-06
TUCP_002466	XLOC_104506	ENSRNOT00000081621	0.960334824	7.23E-07
TUCP_002466	XLOC_104506	ENSRNOT00000027301	-0.966802447	3.00E-07
TUCP_002466	XLOC_104506	ENSRNOT00000030373	-0.950803249	2.09E-06
TUCP_002466	XLOC_104506	ENSRNOT00000090338	-0.958940845	8.58E-07
TUCP_002466	XLOC_104506	ENSRNOT00000039523	0.950203283	2.22E-06
TUCP_002466	XLOC_104506	ENSRNOT00000089933	0.979428472	2.80E-08
TUCP_002466	XLOC_104506	ENSRNOT00000083876	-0.977609685	4.27E-08
TUCP_002466	XLOC_104506	ENSRNOT00000090912	0.977122358	4.75E-08
TUCP_002466	XLOC_104506	ENSRNOT00000072208	0.963552369	4.76E-07
TUCP_002466	XLOC_104506	ENSRNOT00000084012	0.959199195	8.31E-07
TUCP_002466	XLOC_104506	ENSRNOT00000033677	-0.950967963	2.06E-06
TUCP_002466	XLOC_104506	ENSRNOT00000019820	0.962444209	5.52E-07
TUCP_002466	XLOC_104506	ENSRNOT00000003940	-0.96632834	3.22E-07
TUCP_002466	XLOC_104506	ENSRNOT00000025362	-0.968561567	2.29E-07
TUCP_002466	XLOC_104506	ENSRNOT00000010541	0.962122346	5.76E-07
TUCP_002466	XLOC_104506	ENSRNOT00000017949	-0.950808457	2.09E-06
TUCP_002466	XLOC_104506	ENSRNOT00000074212	0.950773977	2.10E-06
TUCP_002466	XLOC_104506	ENSRNOT00000091909	0.971114503	1.51E-07
TUCP_002466	XLOC_104506	ENSRNOT00000021925	0.975142126	7.17E-08
TUCP_002466	XLOC_104506	ENSRNOT00000086119	-0.963015153	5.12E-07
TUCP_002466	XLOC_104506	ENSRNOT00000064452	-0.958063859	9.52E-07
TUCP_002466	XLOC_104506	ENSRNOT00000022190	-0.956343333	1.16E-06
TUCP_002466	XLOC_104506	ENSRNOT00000025913	-0.960484764	7.10E-07
TUCP_002466	XLOC_104506	ENSRNOT00000019386	-0.961463246	6.27E-07
TUCP_002466	XLOC_104506	ENSRNOT00000077220	-0.961874789	5.95E-07
TUCP_002466	XLOC_104506	ENSRNOT00000065983	0.965146595	3.82E-07
TUCP_002466	XLOC_104506	ENSRNOT00000067780	-0.959627547	7.89E-07
TUCP_002466	XLOC_104506	ENSRNOT00000086577	0.977930899	3.97E-08
TUCP_002466	XLOC_104506	ENSRNOT00000036658	-0.950633071	2.12E-06
TUCP_002466	XLOC_104506	ENSRNOT00000007738	-0.956230425	1.18E-06
TUCP_002466	XLOC_104506	ENSRNOT00000065600	0.954769866	1.38E-06
TUCP_002466	XLOC_104506	ENSRNOT00000034883	-0.957327509	1.04E-06
TUCP_002466	XLOC_104506	ENSRNOT00000020155	0.968038854	2.49E-07
TUCP_002466	XLOC_104506	ENSRNOT00000055321	0.972591099	1.16E-07
TUCP_002466	XLOC_104506	ENSRNOT00000005910	-0.955940097	1.21E-06
TUCP_002466	XLOC_104506	ENSRNOT00000078475	0.953448941	1.59E-06
TUCP_002466	XLOC_104506	ENSRNOT00000087429	0.960311429	7.25E-07
TUCP_002466	XLOC_104506	ENSRNOT00000055888	-0.950704823	2.11E-06
TUCP_002466	XLOC_104506	ENSRNOT00000000013	0.950389139	2.18E-06
TUCP_002466	XLOC_104506	ENSRNOT00000030329	-0.961330672	6.38E-07
TUCP_002466	XLOC_104506	ENSRNOT00000026057	-0.951844825	1.88E-06
TUCP_002466	XLOC_104506	ENSRNOT00000028057	0.959746672	7.78E-07
TUCP_002466	XLOC_104506	ENSRNOT00000055582	0.959362858	8.15E-07
TUCP_002466	XLOC_104506	ENSRNOT00000022074	0.952760498	1.71E-06
TUCP_002466	XLOC_104506	ENSRNOT00000004999	0.967178326	2.84E-07
TUCP_002466	XLOC_104506	ENSRNOT00000009122	0.951579143	1.93E-06
TUCP_002466	XLOC_104506	ENSRNOT00000013463	0.977980257	3.93E-08
TUCP_002466	XLOC_104506	ENSRNOT00000089418	0.960363603	7.21E-07
TUCP_002466	XLOC_104506	ENSRNOT00000007889	-0.95793934	9.66E-07

TUCP_002466	XLOC_104506	ENSRNOT00000047663	-0.976932084	4.95E-08
TUCP_002466	XLOC_104506	ENSRNOT00000000532	-0.951705561	1.91E-06
TUCP_002466	XLOC_104506	ENSRNOT00000029202	-0.960842192	6.79E-07
TUCP_002466	XLOC_104506	ENSRNOT00000017907	-0.971399395	1.44E-07
TUCP_002466	XLOC_104506	ENSRNOT00000005489	0.956805833	1.10E-06
TUCP_002466	XLOC_104506	ENSRNOT00000025794	-0.958271417	9.29E-07
TUCP_002466	XLOC_104506	ENSRNOT00000084422	0.966251757	3.26E-07
TUCP_002466	XLOC_104506	ENSRNOT00000050834	-0.958241891	9.32E-07
TUCP_002466	XLOC_104506	ENSRNOT00000088279	0.955835242	1.23E-06
TUCP_002466	XLOC_104506	ENSRNOT00000002443	0.977646336	4.23E-08
TUCP_002466	XLOC_104506	ENSRNOT00000002114	-0.977699858	4.18E-08
TUCP_002466	XLOC_104506	ENSRNOT00000002445	-0.955554976	1.27E-06
TUCP_002466	XLOC_104506	ENSRNOT00000027970	0.982899925	1.12E-08
TUCP_002466	XLOC_104506	ENSRNOT00000052422	0.95313659	1.65E-06
TUCP_002466	XLOC_104506	ENSRNOT00000046215	-0.962168511	5.73E-07
TUCP_002466	XLOC_104506	ENSRNOT00000000083	-0.953910038	1.52E-06
TUCP_002466	XLOC_104506	ENSRNOT00000037679	0.952699106	1.72E-06
TUCP_002466	XLOC_104506	ENSRNOT00000014124	0.961302709	6.40E-07
TUCP_002466	XLOC_104506	ENSRNOT00000000120	0.984129655	7.72E-09
TUCP_002466	XLOC_104506	ENSRNOT00000013632	0.954856287	1.37E-06
TUCP_002466	XLOC_104506	ENSRNOT00000028545	-0.965947965	3.41E-07
TUCP_002466	XLOC_104506	ENSRNOT00000001759	-0.972390129	1.21E-07
TUCP_002466	XLOC_104506	ENSRNOT00000028225	0.960042632	7.50E-07
TUCP_002466	XLOC_104506	ENSRNOT00000032248	0.955176242	1.32E-06
TUCP_002466	XLOC_104506	ENSRNOT00000019911	-0.969594026	1.94E-07
TUCP_002466	XLOC_104506	ENSRNOT00000040393	0.969464218	1.99E-07
TUCP_002466	XLOC_104506	ENSRNOT00000032236	-0.950666946	2.12E-06
TUCP_002466	XLOC_104506	ENSRNOT00000016372	-0.956928576	1.09E-06
TUCP_002466	XLOC_104506	ENSRNOT00000012286	-0.976270491	5.69E-08
TUCP_002466	XLOC_104506	ENSRNOT00000007169	-0.95488462	1.36E-06
TUCP_002466	XLOC_104506	ENSRNOT00000064700	0.960395346	7.18E-07
TUCP_002466	XLOC_104506	ENSRNOT00000036613	-0.971509912	1.41E-07
TUCP_002466	XLOC_104506	ENSRNOT00000025705	0.96280043	5.27E-07
TUCP_002466	XLOC_104506	ENSRNOT00000021285	0.952332817	1.79E-06
TUCP_002466	XLOC_104506	ENSRNOT00000019983	-0.969095258	2.11E-07
TUCP_002466	XLOC_104506	ENSRNOT00000004140	0.953646119	1.56E-06
TUCP_002466	XLOC_104506	ENSRNOT00000008113	-0.961324164	6.39E-07
TUCP_002466	XLOC_104506	ENSRNOT00000000334	-0.967008716	2.91E-07
TUCP_002466	XLOC_104506	ENSRNOT00000026279	-0.955407582	1.29E-06
TUCP_002466	XLOC_104506	ENSRNOT00000021850	0.969799768	1.88E-07
TUCP_002466	XLOC_104506	ENSRNOT00000039341	0.968649871	2.26E-07
TUCP_002466	XLOC_104506	ENSRNOT00000073492	-0.961565522	6.19E-07
TUCP_002466	XLOC_104506	ENSRNOT00000082863	0.960582664	7.01E-07
TUCP_002466	XLOC_104506	ENSRNOT00000071166	0.952700063	1.72E-06
TUCP_002466	XLOC_104506	ENSRNOT00000003412	-0.961186484	6.50E-07
TUCP_002466	XLOC_104506	ENSRNOT00000013852	0.954741847	1.39E-06
TUCP_002466	XLOC_104506	ENSRNOT00000013851	-0.961535695	6.22E-07
TUCP_002466	XLOC_104506	ENSRNOT00000066204	-0.959858088	7.67E-07
TUCP_002466	XLOC_104506	ENSRNOT00000076105	-0.952315258	1.79E-06
TUCP_002466	XLOC_104506	ENSRNOT00000001507	0.950678883	2.12E-06
TUCP_002466	XLOC_104506	ENSRNOT00000081047	0.975335061	6.90E-08

TUCP_002466	XLOC_104506	ENSRNOT00000042903	0.960027323	7.51E-07
TUCP_002466	XLOC_104506	ENSRNOT00000037456	0.957145377	1.06E-06
TUCP_002466	XLOC_104506	ENSRNOT00000000477	-0.952807309	1.70E-06
TUCP_002466	XLOC_104506	ENSRNOT00000012881	-0.957792565	9.83E-07
TUCP_002466	XLOC_104506	ENSRNOT00000000577	0.95759597	1.01E-06
TUCP_002466	XLOC_104506	ENSRNOT00000041513	0.953571328	1.57E-06
TUCP_002466	XLOC_104506	ENSRNOT00000088133	-0.966583029	3.10E-07
TUCP_002466	XLOC_104506	ENSRNOT00000080677	-0.962615751	5.40E-07
TUCP_002466	XLOC_104506	ENSRNOT00000037039	-0.964419506	4.23E-07
TUCP_002466	XLOC_104506	ENSRNOT00000037133	0.970284929	1.74E-07
TUCP_002466	XLOC_104506	ENSRNOT00000083964	-0.970237057	1.75E-07
TUCP_002466	XLOC_104506	ENSRNOT00000026831	0.960254985	7.31E-07
TUCP_002466	XLOC_104506	ENSRNOT00000028266	0.956724125	1.11E-06
TUCP_002466	XLOC_104506	ENSRNOT00000032363	0.97637809	5.57E-08
TUCP_002466	XLOC_104506	ENSRNOT00000016445	0.971221246	1.48E-07
TUCP_002466	XLOC_104506	ENSRNOT00000019133	0.950863399	2.08E-06
TUCP_002466	XLOC_104506	ENSRNOT00000024119	0.961732783	6.06E-07
TUCP_002466	XLOC_104506	ENSRNOT00000002152	-0.957086137	1.07E-06
TUCP_002466	XLOC_104506	ENSRNOT00000032667	-0.958811711	8.71E-07
TUCP_002466	XLOC_104506	ENSRNOT00000082138	-0.964919178	3.94E-07
TUCP_002466	XLOC_104506	ENSRNOT00000044608	-0.981733459	1.55E-08
TUCP_002466	XLOC_104506	ENSRNOT00000001099	-0.952725447	1.72E-06
TUCP_002466	XLOC_104506	ENSRNOT00000050119	-0.972714144	1.14E-07
TUCP_002466	XLOC_104506	ENSRNOT00000082427	-0.953734979	1.54E-06
TUCP_002466	XLOC_104506	ENSRNOT00000089211	0.971460531	1.42E-07
TUCP_002466	XLOC_104506	ENSRNOT00000020383	0.950004285	2.26E-06
TUCP_002466	XLOC_104506	ENSRNOT00000073847	-0.950859169	2.08E-06
TUCP_002466	XLOC_104506	ENSRNOT00000052224	0.976534668	5.39E-08
TUCP_002466	XLOC_104506	ENSRNOT00000000302	0.959096431	8.42E-07
TUCP_002466	XLOC_104506	ENSRNOT00000078070	0.966480069	3.15E-07
TUCP_002466	XLOC_104506	ENSRNOT00000000658	0.950313541	2.19E-06
TUCP_002466	XLOC_104506	ENSRNOT00000065229	0.957293086	1.04E-06
TUCP_002466	XLOC_104506	ENSRNOT00000086122	0.967895598	2.55E-07
TUCP_002466	XLOC_104506	ENSRNOT00000026235	-0.956806827	1.10E-06
TUCP_002466	XLOC_104506	ENSRNOT00000027967	0.958613303	8.92E-07
TUCP_002466	XLOC_104506	ENSRNOT00000023872	-0.956862188	1.09E-06
TUCP_002466	XLOC_104506	ENSRNOT00000010753	-0.959813486	7.72E-07
TUCP_002466	XLOC_104506	ENSRNOT00000023763	-0.960092712	7.45E-07
TUCP_002466	XLOC_104506	ENSRNOT00000059181	0.960225532	7.33E-07
TUCP_002466	XLOC_104506	ENSRNOT00000003691	-0.965123243	3.83E-07
TUCP_002466	XLOC_104506	ENSRNOT00000003690	0.95251476	1.76E-06
TUCP_002466	XLOC_104506	ENSRNOT00000056405	0.950576378	2.14E-06
TUCP_002466	XLOC_104506	ENSRNOT00000038807	0.961941801	5.90E-07
TUCP_002466	XLOC_104506	ENSRNOT00000017761	0.958079142	9.50E-07
TUCP_002466	XLOC_104506	ENSRNOT00000080608	0.969079319	2.11E-07
TUCP_002466	XLOC_104506	ENSRNOT00000005478	-0.953541947	1.58E-06
TUCP_002466	XLOC_104506	ENSRNOT00000080695	0.951913577	1.87E-06
TUCP_002466	XLOC_104506	ENSRNOT00000027117	-0.963911976	4.54E-07
TUCP_002466	XLOC_104506	ENSRNOT00000055386	0.951352525	1.98E-06
TUCP_002466	XLOC_104506	ENSRNOT00000061392	0.972126134	1.26E-07
TUCP_002466	XLOC_104506	ENSRNOT00000019768	0.961113893	6.56E-07

TUCP_002466	XLOC_104506	ENSRNOT00000017181	-0.964279894	4.31E-07
TUCP_002466	XLOC_104506	ENSRNOT00000071853	-0.950620664	2.13E-06
TUCP_002466	XLOC_104506	ENSRNOT00000042125	0.981983276	1.45E-08
TUCP_002466	XLOC_104506	ENSRNOT00000081272	-0.962071086	5.80E-07
TUCP_002466	XLOC_104506	ENSRNOT00000045735	0.966326842	3.22E-07
TUCP_002466	XLOC_104506	ENSRNOT00000026760	-0.966957521	2.93E-07
TUCP_002466	XLOC_104506	ENSRNOT00000063821	-0.950405381	2.17E-06
TUCP_002466	XLOC_104506	ENSRNOT00000002355	-0.952920697	1.68E-06
TUCP_002466	XLOC_104506	ENSRNOT00000047210	0.96926501	2.05E-07
TUCP_002466	XLOC_104506	ENSRNOT00000078349	-0.960153277	7.40E-07
TUCP_002466	XLOC_104506	ENSRNOT00000083957	0.956603468	1.13E-06
TUCP_002466	XLOC_104506	ENSRNOT00000005885	-0.970192684	1.76E-07
TUCP_003177	XLOC_136347	ENSRNOT00000037086	0.95049073	2.16E-06
TUCP_003177	XLOC_136347	ENSRNOT00000064609	0.953466518	1.59E-06
TUCP_003177	XLOC_136347	ENSRNOT00000084784	0.955610336	1.26E-06
TUCP_002462	XLOC_104349	ENSRNOT00000084133	0.987028858	2.83E-09
TUCP_002462	XLOC_104349	ENSRNOT00000093745	0.997438975	8.64E-13
TUCP_002462	XLOC_104349	ENSRNOT00000065542	0.985338279	5.21E-09
TUCP_002462	XLOC_104349	ENSRNOT00000082464	0.987028858	2.83E-09
TUCP_002462	XLOC_104349	ENSRNOT00000087659	0.987028858	2.83E-09
TUCP_002462	XLOC_104349	ENSRNOT00000084582	0.973327197	1.02E-07
TUCP_002462	XLOC_104349	ENSRNOT00000085078	0.987028858	2.83E-09
TUCP_002462	XLOC_104349	ENSRNOT00000014028	0.987028858	2.83E-09
TUCP_002462	XLOC_104349	ENSRNOT00000049363	0.987028858	2.83E-09
TUCP_002462	XLOC_104349	ENSRNOT00000065473	0.986724919	3.18E-09
TUCP_002462	XLOC_104349	ENSRNOT00000000142	0.984483497	6.90E-09
TUCP_002462	XLOC_104349	ENSRNOT00000082288	0.987233016	2.61E-09
TUCP_002462	XLOC_104349	ENSRNOT00000039664	0.987028858	2.83E-09
TUCP_002462	XLOC_104349	ENSRNOT00000065402	0.987028858	2.83E-09
TUCP_002462	XLOC_104349	ENSRNOT00000064551	0.957775525	9.85E-07
TUCP_002462	XLOC_104349	ENSRNOT00000084141	0.987028858	2.83E-09
TUCP_002462	XLOC_104349	ENSRNOT00000082435	0.987028858	2.83E-09
TUCP_002462	XLOC_104349	ENSRNOT00000082928	0.981980208	1.45E-08
TUCP_002462	XLOC_104349	ENSRNOT00000066546	0.951548574	1.94E-06
TUCP_002462	XLOC_104349	ENSRNOT00000046013	0.974831182	7.63E-08
TUCP_002462	XLOC_104349	ENSRNOT00000067931	0.960854569	6.78E-07
TUCP_002462	XLOC_104349	ENSRNOT00000086425	0.978690263	3.34E-08
TUCP_002462	XLOC_104349	ENSRNOT00000093696	0.955531091	1.27E-06
TUCP_002462	XLOC_104349	ENSRNOT00000036228	0.96411621	4.41E-07
TUCP_002462	XLOC_104349	ENSRNOT00000014030	0.987028858	2.83E-09
TUCP_002462	XLOC_104349	ENSRNOT00000081966	0.987028858	2.83E-09
TUCP_002462	XLOC_104349	ENSRNOT00000075906	0.987028858	2.83E-09
TUCP_002462	XLOC_104349	ENSRNOT00000088396	0.96162299	6.15E-07
TUCP_002462	XLOC_104349	ENSRNOT00000049259	0.958249275	9.31E-07
TUCP_002462	XLOC_104349	ENSRNOT00000092838	0.964102345	4.42E-07
TUCP_000992	XLOC_036559	ENSRNOT00000084500	0.999195771	2.65E-15
TUCP_000992	XLOC_036559	ENSRNOT00000093467	0.975831016	6.24E-08
TUCP_000992	XLOC_036559	ENSRNOT00000029340	0.974011953	8.94E-08
TUCP_000992	XLOC_036559	ENSRNOT00000077972	0.980321536	2.25E-08
TUCP_000992	XLOC_036559	ENSRNOT00000038646	0.977150283	4.72E-08
TUCP_000992	XLOC_036559	ENSRNOT00000048858	0.986776327	3.11E-09

TUCP_000992	XLOC_036559	ENSRNOT00000092579	0.977150283	4.72E-08
TUCP_000992	XLOC_036559	ENSRNOT00000048373	0.979274384	2.91E-08
TUCP_000992	XLOC_036559	ENSRNOT00000036383	0.95882932	8.69E-07
TUCP_000992	XLOC_036559	ENSRNOT00000076528	0.971977597	1.30E-07
TUCP_000992	XLOC_036559	ENSRNOT00000083034	0.97803018	3.89E-08
TUCP_000992	XLOC_036559	ENSRNOT00000089607	0.968649439	2.26E-07
TUCP_000992	XLOC_036559	ENSRNOT00000086014	0.957499041	1.02E-06
TUCP_000992	XLOC_036559	ENSRNOT00000052315	0.957386597	1.03E-06
TUCP_000992	XLOC_036559	ENSRNOT00000076833	0.954952189	1.35E-06
TUCP_000992	XLOC_036559	ENSRNOT00000013459	0.95240288	1.78E-06
TUCP_000992	XLOC_036559	ENSRNOT00000078137	0.977150283	4.72E-08
TUCP_000992	XLOC_036559	ENSRNOT00000077764	0.953448143	1.59E-06
TUCP_000992	XLOC_036559	ENSRNOT00000043661	0.999031697	6.69E-15
TUCP_000992	XLOC_036559	ENSRNOT00000092740	0.977150283	4.72E-08
TUCP_000992	XLOC_036559	ENSRNOT00000000117	0.965498421	3.63E-07
TUCP_000992	XLOC_036559	ENSRNOT00000071877	0.986692042	3.21E-09
TUCP_000992	XLOC_036559	ENSRNOT00000070958	0.977150283	4.72E-08
TUCP_000992	XLOC_036559	ENSRNOT00000076757	0.985140761	5.56E-09
TUCP_000992	XLOC_036559	ENSRNOT00000047241	0.998983703	8.52E-15
TUCP_000992	XLOC_036559	ENSRNOT00000027268	0.977150283	4.72E-08
TUCP_000992	XLOC_036559	ENSRNOT00000004459	0.977150283	4.72E-08
TUCP_000992	XLOC_036559	ENSRNOT00000089235	0.975520129	6.64E-08
TUCP_000992	XLOC_036559	ENSRNOT00000088168	0.977150283	4.72E-08
TUCP_000992	XLOC_036559	ENSRNOT00000088148	0.977150283	4.72E-08
TUCP_000992	XLOC_036559	ENSRNOT00000088527	0.977150283	4.72E-08
TUCP_000992	XLOC_036559	ENSRNOT00000078701	0.977150283	4.72E-08
TUCP_000992	XLOC_036559	ENSRNOT00000052308	0.998200432	1.48E-13
TUCP_000992	XLOC_036559	ENSRNOT00000052130	0.955241663	1.31E-06
TUCP_000992	XLOC_036559	ENSRNOT00000021748	0.977150283	4.72E-08
TUCP_000992	XLOC_036559	ENSRNOT00000089085	0.977150283	4.72E-08
TUCP_000992	XLOC_036559	ENSRNOT00000008153	0.977433584	4.44E-08
TUCP_000992	XLOC_036559	ENSRNOT00000079189	0.953831224	1.53E-06
TUCP_000992	XLOC_036559	ENSRNOT00000090078	0.977150283	4.72E-08
TUCP_000992	XLOC_036559	ENSRNOT00000078020	0.956097766	1.19E-06
TUCP_000992	XLOC_036559	ENSRNOT00000009776	0.977150283	4.72E-08
TUCP_002747	XLOC_116543	ENSRNOT00000035840	0.975935799	6.10E-08
TUCP_000996	XLOC_036942	ENSRNOT00000026966	0.970322306	1.73E-07
TUCP_000996	XLOC_036942	ENSRNOT00000068416	0.999999782	3.88E-33
TUCP_000996	XLOC_036942	ENSRNOT00000086036	0.999958274	9.96E-22
TUCP_000996	XLOC_036942	ENSRNOT00000026803	0.985046075	5.74E-09
TUCP_000996	XLOC_036942	ENSRNOT00000047527	0.963071698	5.08E-07
TUCP_000996	XLOC_036942	ENSRNOT00000042251	0.999344295	9.53E-16
TUCP_000996	XLOC_036942	ENSRNOT00000002984	0.998397069	8.31E-14
TUCP_000996	XLOC_036942	ENSRNOT00000083764	0.999541808	1.59E-16
TUCP_000996	XLOC_036942	ENSRNOT00000076802	0.962695843	5.34E-07
TUCP_000996	XLOC_036942	ENSRNOT00000092365	0.999161651	3.26E-15
TUCP_000996	XLOC_036942	ENSRNOT00000079275	0.999901911	7.15E-20
TUCP_000996	XLOC_036942	ENSRNOT00000066062	-0.959706712	7.82E-07
TUCP_000996	XLOC_036942	ENSRNOT00000045301	1	4.25E-78
TUCP_000996	XLOC_036942	ENSRNOT00000041186	0.999975782	6.56E-23
TUCP_000996	XLOC_036942	ENSRNOT00000010092	0.977711778	4.17E-08

TUCP_000996	XLOC_036942	ENSRNOT00000076182	1	1.33E-79
TUCP_000996	XLOC_036942	ENSRNOT00000087404	0.951157664	2.02E-06
TUCP_000996	XLOC_036942	ENSRNOT00000052003	0.95837331	9.18E-07
TUCP_000996	XLOC_036942	ENSRNOT00000081709	0.998485154	6.27E-14
TUCP_000996	XLOC_036942	ENSRNOT00000005726	0.959610119	7.91E-07
TUCP_000996	XLOC_036942	ENSRNOT00000014103	1	0
TUCP_000996	XLOC_036942	ENSRNOT00000057433	0.964930601	3.94E-07
TUCP_000996	XLOC_036942	ENSRNOT00000082289	0.998931566	1.09E-14
TUCP_000996	XLOC_036942	ENSRNOT00000092516	0.957459323	1.02E-06
TUCP_000996	XLOC_036942	ENSRNOT00000008898	0.990039768	7.59E-10
TUCP_000996	XLOC_036942	ENSRNOT00000083493	0.995461232	1.51E-11
TUCP_000996	XLOC_036942	ENSRNOT00000092563	0.987201084	2.65E-09
TUCP_000996	XLOC_036942	ENSRNOT00000088893	1	0
TUCP_000996	XLOC_036942	ENSRNOT00000076693	0.955029582	1.34E-06
TUCP_000996	XLOC_036942	ENSRNOT00000081014	1	0
TUCP_000996	XLOC_036942	ENSRNOT00000067543	1	0
TUCP_000996	XLOC_036942	ENSRNOT00000050968	0.996816433	2.56E-12
TUCP_000996	XLOC_036942	ENSRNOT00000042201	0.960235595	7.32E-07
TUCP_000996	XLOC_036942	ENSRNOT00000004373	0.957315473	1.04E-06
TUCP_000996	XLOC_036942	ENSRNOT00000063929	1	0
TUCP_000996	XLOC_036942	ENSRNOT00000057472	1	4.25E-78
TUCP_000996	XLOC_036942	ENSRNOT00000041617	0.952449765	1.77E-06
TUCP_000996	XLOC_036942	ENSRNOT00000091763	0.986310385	3.70E-09
TUCP_000996	XLOC_036942	ENSRNOT00000022200	0.998491303	6.14E-14
TUCP_000996	XLOC_036942	ENSRNOT00000067379	0.997260967	1.21E-12
TUCP_000996	XLOC_036942	ENSRNOT00000020786	0.976428448	5.51E-08
TUCP_000996	XLOC_036942	ENSRNOT00000050543	0.999722223	1.30E-17
TUCP_000996	XLOC_036942	ENSRNOT00000089306	0.999866649	3.32E-19
TUCP_000996	XLOC_036942	ENSRNOT00000052367	0.997268394	1.19E-12
TUCP_000996	XLOC_036942	ENSRNOT00000090658	1	0
TUCP_000996	XLOC_036942	ENSRNOT00000022571	0.995290013	1.81E-11
TUCP_000996	XLOC_036942	ENSRNOT00000044520	0.981817158	1.52E-08
TUCP_000996	XLOC_036942	ENSRNOT00000065448	0.963648868	4.70E-07
TUCP_002740	XLOC_116280	ENSRNOT00000077972	0.968818246	2.20E-07
TUCP_002740	XLOC_116280	ENSRNOT00000000995	0.95227019	1.80E-06
TUCP_002740	XLOC_116280	ENSRNOT00000028877	0.963812896	4.60E-07
TUCP_002740	XLOC_116280	ENSRNOT00000046231	0.953293856	1.62E-06
TUCP_002740	XLOC_116280	ENSRNOT00000085479	0.960561467	7.03E-07
TUCP_002740	XLOC_116280	ENSRNOT00000048373	0.966996598	2.92E-07
TUCP_002740	XLOC_116280	ENSRNOT00000077764	0.97442148	8.26E-08
TUCP_002740	XLOC_116280	ENSRNOT00000093748	0.957307699	1.04E-06
TUCP_002740	XLOC_116280	ENSRNOT00000017909	0.954212445	1.47E-06
TUCP_002740	XLOC_116280	ENSRNOT00000078020	0.967531906	2.69E-07
TUCP_002740	XLOC_116280	ENSRNOT00000051028	-0.953879565	1.52E-06
TUCP_002743	XLOC_116516	ENSRNOT00000018233	-0.96027442	7.29E-07
TUCP_002743	XLOC_116516	ENSRNOT00000089068	0.952266548	1.80E-06
TUCP_002742	XLOC_116403	ENSRNOT00000056450	0.986128242	3.95E-09
TUCP_003641	XLOC_160009	ENSRNOT00000087764	0.961837004	5.98E-07
TUCP_003641	XLOC_160009	ENSRNOT00000090716	0.952815782	1.70E-06
TUCP_002412	XLOC_101758	ENSRNOT00000011263	-0.954176717	1.47E-06
TUCP_002412	XLOC_101758	ENSRNOT00000000264	0.958725725	8.80E-07

TUCP_002412	XLOC_101758	ENSRNOT00000025797	0.957044214	1.07E-06
TUCP_002412	XLOC_101758	ENSRNOT00000084282	0.961491271	6.25E-07
TUCP_002412	XLOC_101758	ENSRNOT00000025403	-0.953129686	1.65E-06
TUCP_003798	XLOC_166074	ENSRNOT00000019921	0.951651375	1.92E-06
TUCP_003798	XLOC_166074	ENSRNOT00000059246	0.958986102	8.53E-07
TUCP_003798	XLOC_166074	ENSRNOT00000091427	-0.958278273	9.28E-07
TUCP_003798	XLOC_166074	ENSRNOT00000089970	0.955314415	1.30E-06
TUCP_003798	XLOC_166074	ENSRNOT00000014627	0.95750611	1.02E-06
TUCP_003798	XLOC_166074	ENSRNOT00000027435	0.954902998	1.36E-06
TUCP_003798	XLOC_166074	ENSRNOT00000034282	0.956521222	1.14E-06
TUCP_003798	XLOC_166074	ENSRNOT00000027875	0.957075219	1.07E-06
TUCP_003798	XLOC_166074	ENSRNOT00000060494	0.964429943	4.22E-07
TUCP_003798	XLOC_166074	ENSRNOT00000038838	0.978759085	3.29E-08
TUCP_003798	XLOC_166074	ENSRNOT00000065897	0.96977672	1.89E-07
TUCP_003798	XLOC_166074	ENSRNOT00000036904	0.953478478	1.59E-06
TUCP_003798	XLOC_166074	ENSRNOT00000066708	0.950964544	2.06E-06
TUCP_003646	XLOC_160056	ENSRNOT00000085943	0.972653566	1.15E-07
TUCP_003646	XLOC_160056	ENSRNOT00000011342	0.968902267	2.17E-07
TUCP_003646	XLOC_160056	ENSRNOT00000023610	0.966331938	3.22E-07
TUCP_003646	XLOC_160056	ENSRNOT00000072396	0.972568024	1.17E-07
TUCP_003646	XLOC_160056	ENSRNOT00000093577	0.982950649	1.10E-08
TUCP_003519	XLOC_153199	ENSRNOT00000028234	0.961338624	6.37E-07
TUCP_003519	XLOC_153199	ENSRNOT00000082982	0.974569349	8.03E-08
TUCP_003519	XLOC_153199	ENSRNOT00000034096	0.953875192	1.52E-06
TUCP_003519	XLOC_153199	ENSRNOT00000024933	0.952958893	1.68E-06
TUCP_003519	XLOC_153199	ENSRNOT00000038250	0.952791446	1.71E-06
TUCP_003519	XLOC_153199	ENSRNOT00000018776	0.953507766	1.58E-06
TUCP_003519	XLOC_153199	ENSRNOT00000067011	-0.966175408	3.29E-07
TUCP_003519	XLOC_153199	ENSRNOT00000000804	0.959877675	7.65E-07
TUCP_003519	XLOC_153199	ENSRNOT00000049973	0.953478585	1.59E-06
TUCP_003519	XLOC_153199	ENSRNOT00000002247	0.962853775	5.23E-07
TUCP_003519	XLOC_153199	ENSRNOT00000029193	0.967935221	2.53E-07
TUCP_001778	XLOC_073508	ENSRNOT00000086899	0.95474535	1.39E-06
TUCP_001778	XLOC_073508	ENSRNOT00000023152	0.950503668	2.15E-06
TUCP_001778	XLOC_073508	ENSRNOT00000028058	0.978452692	3.53E-08
TUCP_001778	XLOC_073508	ENSRNOT00000088707	0.950797769	2.09E-06
TUCP_001778	XLOC_073508	ENSRNOT00000039876	0.953977261	1.50E-06
TUCP_001778	XLOC_073508	ENSRNOT00000052003	0.958397652	9.15E-07
TUCP_001778	XLOC_073508	ENSRNOT00000093244	0.956479244	1.14E-06
TUCP_003089	XLOC_132420	ENSRNOT00000027770	0.953531054	1.58E-06
TUCP_003089	XLOC_132420	ENSRNOT00000087027	0.983677432	8.88E-09
TUCP_003089	XLOC_132420	ENSRNOT00000079498	0.986682239	3.23E-09
TUCP_003089	XLOC_132420	ENSRNOT00000085704	0.950507493	2.15E-06
TUCP_003089	XLOC_132420	ENSRNOT00000092435	0.97141367	1.43E-07
TUCP_003101	XLOC_132687	ENSRNOT00000065892	0.960847752	6.78E-07
TUCP_003101	XLOC_132687	ENSRNOT00000018405	0.959105299	8.41E-07
TUCP_003101	XLOC_132687	ENSRNOT00000021458	0.95575211	1.24E-06
TUCP_003101	XLOC_132687	ENSRNOT00000079784	0.95905315	8.46E-07
TUCP_003101	XLOC_132687	ENSRNOT00000082183	-0.951031975	2.04E-06
TUCP_003101	XLOC_132687	ENSRNOT00000024924	0.960017136	7.52E-07
TUCP_003101	XLOC_132687	ENSRNOT00000074544	0.955566156	1.27E-06

TUCP_003101	XLOC_132687	ENSRNOT00000088884	0.959604959	7.91E-07
TUCP_003101	XLOC_132687	ENSRNOT00000076388	0.96583104	3.46E-07
TUCP_003101	XLOC_132687	ENSRNOT00000021368	0.959421232	8.09E-07
TUCP_003101	XLOC_132687	ENSRNOT00000005155	-0.988689923	1.43E-09
TUCP_003101	XLOC_132687	ENSRNOT00000009080	0.954979134	1.35E-06
TUCP_003101	XLOC_132687	ENSRNOT00000072864	0.974862097	7.58E-08
TUCP_003101	XLOC_132687	ENSRNOT00000083983	0.968460461	2.33E-07
TUCP_003101	XLOC_132687	ENSRNOT00000035400	0.954724506	1.39E-06
TUCP_003101	XLOC_132687	ENSRNOT00000016262	0.950304657	2.20E-06
TUCP_003101	XLOC_132687	ENSRNOT00000022637	0.970682675	1.62E-07
TUCP_003101	XLOC_132687	ENSRNOT00000038652	0.960835598	6.79E-07
TUCP_003101	XLOC_132687	ENSRNOT00000001891	0.954898685	1.36E-06
TUCP_003101	XLOC_132687	ENSRNOT00000014095	0.972882179	1.10E-07
TUCP_003101	XLOC_132687	ENSRNOT00000019358	0.962119523	5.76E-07
TUCP_003101	XLOC_132687	ENSRNOT00000030823	0.953489356	1.59E-06
TUCP_003101	XLOC_132687	ENSRNOT00000001973	0.955601797	1.26E-06
TUCP_003101	XLOC_132687	ENSRNOT00000030501	0.971771684	1.35E-07
TUCP_003101	XLOC_132687	ENSRNOT00000017618	0.962201684	5.70E-07
TUCP_003101	XLOC_132687	ENSRNOT00000010333	0.951196342	2.01E-06
TUCP_003101	XLOC_132687	ENSRNOT00000003728	0.950977333	2.05E-06
TUCP_003101	XLOC_132687	ENSRNOT00000024775	0.959513717	8.00E-07
TUCP_003101	XLOC_132687	ENSRNOT00000024801	0.959911324	7.62E-07
TUCP_003101	XLOC_132687	ENSRNOT00000090575	0.950275788	2.20E-06
TUCP_003101	XLOC_132687	ENSRNOT00000021110	0.96383497	4.58E-07
TUCP_003101	XLOC_132687	ENSRNOT00000026552	0.9576508	9.99E-07
TUCP_003101	XLOC_132687	ENSRNOT00000020853	0.956754913	1.11E-06
TUCP_003101	XLOC_132687	ENSRNOT00000003636	0.95510582	1.33E-06
TUCP_003101	XLOC_132687	ENSRNOT00000013854	0.95274072	1.71E-06
TUCP_003101	XLOC_132687	ENSRNOT00000001774	0.977375252	4.49E-08
TUCP_003101	XLOC_132687	ENSRNOT00000068666	0.951157318	2.02E-06
TUCP_003101	XLOC_132687	ENSRNOT00000020982	0.973767674	9.36E-08
TUCP_003101	XLOC_132687	ENSRNOT00000014694	0.964010071	4.48E-07
TUCP_003101	XLOC_132687	ENSRNOT00000013535	0.9584157	9.13E-07
TUCP_003101	XLOC_132687	ENSRNOT00000015130	0.955317907	1.30E-06
TUCP_003101	XLOC_132687	ENSRNOT00000075386	0.959167532	8.35E-07
TUCP_003101	XLOC_132687	ENSRNOT00000027450	0.952499706	1.76E-06
TUCP_003101	XLOC_132687	ENSRNOT00000076679	0.952866587	1.69E-06
TUCP_003101	XLOC_132687	ENSRNOT00000075400	0.968681327	2.25E-07
TUCP_003101	XLOC_132687	ENSRNOT00000017955	0.966030479	3.36E-07
TUCP_003101	XLOC_132687	ENSRNOT00000078793	-0.969287594	2.04E-07
TUCP_003101	XLOC_132687	ENSRNOT00000005286	0.95414006	1.48E-06
TUCP_003101	XLOC_132687	ENSRNOT00000026118	0.950360035	2.18E-06
TUCP_003101	XLOC_132687	ENSRNOT00000081037	0.953212053	1.63E-06
TUCP_003101	XLOC_132687	ENSRNOT00000089016	0.951460296	1.96E-06
TUCP_003101	XLOC_132687	ENSRNOT00000011898	0.951032343	2.04E-06
TUCP_003101	XLOC_132687	ENSRNOT00000007681	0.972688696	1.14E-07
TUCP_003101	XLOC_132687	ENSRNOT00000075057	0.969740135	1.90E-07
TUCP_003101	XLOC_132687	ENSRNOT00000018877	0.955365373	1.29E-06
TUCP_003101	XLOC_132687	ENSRNOT00000073926	0.964865274	3.97E-07
TUCP_003101	XLOC_132687	ENSRNOT00000056644	0.967998676	2.50E-07
TUCP_003101	XLOC_132687	ENSRNOT00000030222	0.96256105	5.44E-07

TUCP_003101	XLOC_132687	ENSRNOT00000020409	0.959052863	8.46E-07
TUCP_003101	XLOC_132687	ENSRNOT00000093552	0.963422002	4.85E-07
TUCP_003101	XLOC_132687	ENSRNOT00000006843	0.951060987	2.04E-06
TUCP_003101	XLOC_132687	ENSRNOT00000065642	0.952983562	1.67E-06
TUCP_003101	XLOC_132687	ENSRNOT00000017339	0.962049129	5.82E-07
TUCP_003101	XLOC_132687	ENSRNOT00000081593	0.958728183	8.80E-07
TUCP_003101	XLOC_132687	ENSRNOT00000077730	0.961649806	6.13E-07
TUCP_003101	XLOC_132687	ENSRNOT00000086179	0.957505222	1.02E-06
TUCP_003101	XLOC_132687	ENSRNOT00000071494	0.951909154	1.87E-06
TUCP_003101	XLOC_132687	ENSRNOT00000013587	0.962419804	5.54E-07
TUCP_003101	XLOC_132687	ENSRNOT00000038994	0.951504481	1.95E-06
TUCP_003101	XLOC_132687	ENSRNOT00000011432	0.974413171	8.27E-08
TUCP_003101	XLOC_132687	ENSRNOT00000001631	0.959989788	7.55E-07
TUCP_003101	XLOC_132687	ENSRNOT00000021129	0.955087692	1.33E-06
TUCP_003101	XLOC_132687	ENSRNOT00000050227	0.950225983	2.21E-06
TUCP_003101	XLOC_132687	ENSRNOT00000038464	0.96288744	5.21E-07
TUCP_003101	XLOC_132687	ENSRNOT00000035513	0.957199389	1.05E-06
TUCP_003101	XLOC_132687	ENSRNOT00000008359	0.955272313	1.31E-06
TUCP_003101	XLOC_132687	ENSRNOT00000065023	0.970726394	1.61E-07
TUCP_003101	XLOC_132687	ENSRNOT00000057758	0.962593151	5.42E-07
TUCP_002996	XLOC_128384	ENSRNOT00000093075	0.964684549	4.08E-07
TUCP_002996	XLOC_128384	ENSRNOT00000072573	0.954136459	1.48E-06
TUCP_002996	XLOC_128384	ENSRNOT00000058260	0.964684549	4.08E-07
TUCP_002996	XLOC_128384	ENSRNOT00000088887	0.964684549	4.08E-07
TUCP_002996	XLOC_128384	ENSRNOT00000084986	0.964684549	4.08E-07
TUCP_002996	XLOC_128384	ENSRNOT00000074346	0.974496313	8.14E-08
TUCP_002996	XLOC_128384	ENSRNOT00000073209	0.987339806	2.51E-09
TUCP_002996	XLOC_128384	ENSRNOT00000080155	0.958045192	9.54E-07
TUCP_002996	XLOC_128384	ENSRNOT00000092261	0.95654938	1.13E-06
TUCP_002996	XLOC_128384	ENSRNOT00000017828	0.957349103	1.03E-06
TUCP_002996	XLOC_128384	ENSRNOT00000080444	0.957807772	9.81E-07
TUCP_002996	XLOC_128384	ENSRNOT00000071312	0.964684549	4.08E-07
TUCP_002996	XLOC_128384	ENSRNOT00000088245	0.964684549	4.08E-07
TUCP_002996	XLOC_128384	ENSRNOT00000070817	0.961920752	5.91E-07
TUCP_002996	XLOC_128384	ENSRNOT00000058833	0.96139974	6.32E-07
TUCP_002996	XLOC_128384	ENSRNOT00000078052	0.964684549	4.08E-07
TUCP_002996	XLOC_128384	ENSRNOT00000078494	0.964144077	4.39E-07
TUCP_002996	XLOC_128384	ENSRNOT00000085653	0.964684549	4.08E-07
TUCP_002996	XLOC_128384	ENSRNOT00000081992	0.962015209	5.84E-07
TUCP_002996	XLOC_128384	ENSRNOT00000038553	0.984750887	6.33E-09
TUCP_002996	XLOC_128384	ENSRNOT00000055406	0.964684549	4.08E-07
TUCP_002996	XLOC_128384	ENSRNOT00000067655	0.978471541	3.51E-08
TUCP_002996	XLOC_128384	ENSRNOT00000026753	0.964684549	4.08E-07
TUCP_002996	XLOC_128384	ENSRNOT00000080533	0.970056178	1.80E-07
TUCP_002996	XLOC_128384	ENSRNOT00000086031	0.964684549	4.08E-07
TUCP_002996	XLOC_128384	ENSRNOT00000014312	0.964684549	4.08E-07
TUCP_002996	XLOC_128384	ENSRNOT00000051231	0.962438401	5.53E-07
TUCP_002996	XLOC_128384	ENSRNOT00000089407	0.953356413	1.61E-06
TUCP_002996	XLOC_128384	ENSRNOT00000055034	0.961557007	6.20E-07
TUCP_002996	XLOC_128384	ENSRNOT00000093612	0.972009269	1.29E-07
TUCP_000681	XLOC_025780	ENSRNOT00000008542	0.955698057	1.25E-06

TUCP_000681	XLOC_025780	ENSRNOT00000026473	0.975909227	6.14E-08
TUCP_002990	XLOC_128384	ENSRNOT00000008893	-0.958633678	8.90E-07
TUCP_002992	XLOC_128384	ENSRNOT00000080575	0.950917271	2.07E-06
TUCP_002992	XLOC_128384	ENSRNOT00000076699	0.956760272	1.11E-06
TUCP_002992	XLOC_128384	ENSRNOT00000087025	0.950734678	2.10E-06
TUCP_002992	XLOC_128384	ENSRNOT00000074990	0.950734678	2.10E-06
TUCP_002992	XLOC_128384	ENSRNOT00000092982	0.950734678	2.10E-06
TUCP_002992	XLOC_128384	ENSRNOT00000086831	0.950734678	2.10E-06
TUCP_002992	XLOC_128384	ENSRNOT00000088367	0.950734678	2.10E-06
TUCP_002992	XLOC_128384	ENSRNOT00000082058	0.950734678	2.10E-06
TUCP_002992	XLOC_128384	ENSRNOT00000082235	0.955000454	1.35E-06
TUCP_002992	XLOC_128384	ENSRNOT00000068444	0.950734678	2.10E-06
TUCP_002992	XLOC_128384	ENSRNOT00000088905	0.950734678	2.10E-06
TUCP_002992	XLOC_128384	ENSRNOT00000045099	0.950734678	2.10E-06
TUCP_002992	XLOC_128384	ENSRNOT00000019853	0.950734678	2.10E-06
TUCP_002992	XLOC_128384	ENSRNOT00000022280	0.950152444	2.23E-06
TUCP_002992	XLOC_128384	ENSRNOT00000081996	0.950734678	2.10E-06
TUCP_002992	XLOC_128384	ENSRNOT00000074930	0.950734678	2.10E-06
TUCP_002992	XLOC_128384	ENSRNOT00000093083	0.953632674	1.56E-06
TUCP_002792	XLOC_117879	ENSRNOT00000014041	0.966923846	2.95E-07
TUCP_002792	XLOC_117879	ENSRNOT00000078310	0.951416172	1.96E-06
TUCP_002999	XLOC_128384	ENSRNOT00000048923	0.95067362	2.12E-06
TUCP_003116	XLOC_132897	ENSRNOT00000093075	0.982492665	1.26E-08
TUCP_003116	XLOC_132897	ENSRNOT00000058260	0.982492665	1.26E-08
TUCP_003116	XLOC_132897	ENSRNOT00000088887	0.982492665	1.26E-08
TUCP_003116	XLOC_132897	ENSRNOT00000084986	0.982492665	1.26E-08
TUCP_003116	XLOC_132897	ENSRNOT00000073209	0.962171107	5.73E-07
TUCP_003116	XLOC_132897	ENSRNOT00000080155	0.95022178	2.21E-06
TUCP_003116	XLOC_132897	ENSRNOT00000072573	0.983729807	8.74E-09
TUCP_003116	XLOC_132897	ENSRNOT00000017828	0.976899917	4.98E-08
TUCP_003116	XLOC_132897	ENSRNOT00000080444	0.966722878	3.04E-07
TUCP_003116	XLOC_132897	ENSRNOT00000071312	0.982492665	1.26E-08
TUCP_003116	XLOC_132897	ENSRNOT00000088245	0.982492665	1.26E-08
TUCP_003116	XLOC_132897	ENSRNOT00000058833	0.980229799	2.30E-08
TUCP_003116	XLOC_132897	ENSRNOT00000078052	0.982492665	1.26E-08
TUCP_003116	XLOC_132897	ENSRNOT00000078494	0.982196073	1.37E-08
TUCP_003116	XLOC_132897	ENSRNOT00000085653	0.982492665	1.26E-08
TUCP_003116	XLOC_132897	ENSRNOT00000057950	0.972222767	1.24E-07
TUCP_003116	XLOC_132897	ENSRNOT00000081992	0.982756684	1.17E-08
TUCP_003116	XLOC_132897	ENSRNOT00000055406	0.982492665	1.26E-08
TUCP_003116	XLOC_132897	ENSRNOT00000091100	0.954470033	1.43E-06
TUCP_003116	XLOC_132897	ENSRNOT00000090449	0.955026659	1.34E-06
TUCP_003116	XLOC_132897	ENSRNOT00000070817	0.989940205	7.98E-10
TUCP_003116	XLOC_132897	ENSRNOT00000026753	0.982492665	1.26E-08
TUCP_003116	XLOC_132897	ENSRNOT00000080533	0.977717014	4.17E-08
TUCP_003116	XLOC_132897	ENSRNOT00000072774	0.958776005	8.75E-07
TUCP_003116	XLOC_132897	ENSRNOT00000086031	0.982492665	1.26E-08
TUCP_003116	XLOC_132897	ENSRNOT00000019393	0.950670294	2.12E-06
TUCP_003116	XLOC_132897	ENSRNOT00000014312	0.982492665	1.26E-08
TUCP_003116	XLOC_132897	ENSRNOT00000051231	0.981023246	1.88E-08
TUCP_003116	XLOC_132897	ENSRNOT00000092974	0.965655503	3.55E-07

TUCP_003116	XLOC_132897	ENSRNOT00000089407	0.973375946	1.01E-07
TUCP_003116	XLOC_132897	ENSRNOT00000055034	0.982292938	1.33E-08
TUCP_003346	XLOC_144832	ENSRNOT00000014229	0.965036597	3.88E-07
TUCP_003347	XLOC_144849	ENSRNOT00000047105	0.95044934	2.16E-06
TUCP_003347	XLOC_144849	ENSRNOT00000031904	0.963884097	4.55E-07
TUCP_003347	XLOC_144849	ENSRNOT00000026941	0.96071109	6.90E-07
TUCP_003347	XLOC_144849	ENSRNOT00000075653	0.960109786	7.44E-07
TUCP_003347	XLOC_144849	ENSRNOT00000074939	0.95545377	1.28E-06
TUCP_003347	XLOC_144849	ENSRNOT00000073710	0.957276416	1.04E-06
TUCP_003347	XLOC_144849	ENSRNOT00000043856	0.97126424	1.47E-07
TUCP_003347	XLOC_144849	ENSRNOT00000034146	0.957166061	1.06E-06
TUCP_003347	XLOC_144849	ENSRNOT00000006175	0.975489045	6.69E-08
TUCP_003347	XLOC_144849	ENSRNOT00000010035	0.958052183	9.53E-07
TUCP_003347	XLOC_144849	ENSRNOT00000037699	0.955394278	1.29E-06
TUCP_003347	XLOC_144849	ENSRNOT00000016975	0.958704917	8.82E-07
TUCP_003347	XLOC_144849	ENSRNOT00000027411	0.950963577	2.06E-06
TUCP_003347	XLOC_144849	ENSRNOT00000009712	0.964501615	4.18E-07
TUCP_003347	XLOC_144849	ENSRNOT00000026543	0.953054985	1.66E-06
TUCP_003347	XLOC_144849	ENSRNOT00000043345	0.977193901	4.68E-08
TUCP_003347	XLOC_144849	ENSRNOT00000010418	0.950324183	2.19E-06
TUCP_003347	XLOC_144849	ENSRNOT00000071971	0.953860102	1.52E-06
TUCP_003347	XLOC_144849	ENSRNOT00000025689	0.964927063	3.94E-07
TUCP_003347	XLOC_144849	ENSRNOT00000022628	0.962060512	5.81E-07
TUCP_003347	XLOC_144849	ENSRNOT00000064802	0.967589433	2.67E-07
TUCP_003347	XLOC_144849	ENSRNOT00000088125	0.960515873	7.07E-07
TUCP_003347	XLOC_144849	ENSRNOT00000044742	0.952697708	1.72E-06
TUCP_003347	XLOC_144849	ENSRNOT00000060943	0.956699741	1.11E-06
TUCP_003347	XLOC_144849	ENSRNOT00000014664	0.966576781	3.11E-07
TUCP_003347	XLOC_144849	ENSRNOT00000004486	0.958912088	8.61E-07
TUCP_003347	XLOC_144849	ENSRNOT00000045047	0.957609019	1.00E-06
TUCP_003347	XLOC_144849	ENSRNOT00000075326	0.960394629	7.18E-07
TUCP_003347	XLOC_144849	ENSRNOT00000021348	0.965160974	3.81E-07
TUCP_003347	XLOC_144849	ENSRNOT00000012671	0.964611976	4.12E-07
TUCP_003347	XLOC_144849	ENSRNOT00000065168	0.957376109	1.03E-06
TUCP_003347	XLOC_144849	ENSRNOT00000085158	0.952352111	1.79E-06
TUCP_003347	XLOC_144849	ENSRNOT00000025075	0.970392189	1.71E-07
TUCP_003347	XLOC_144849	ENSRNOT00000020134	0.963467224	4.82E-07
TUCP_003347	XLOC_144849	ENSRNOT00000028271	0.954523194	1.42E-06
TUCP_003347	XLOC_144849	ENSRNOT00000027234	0.951843497	1.88E-06
TUCP_003347	XLOC_144849	ENSRNOT00000087371	0.955773647	1.24E-06
TUCP_003347	XLOC_144849	ENSRNOT00000008018	0.958738416	8.79E-07
TUCP_003347	XLOC_144849	ENSRNOT00000037446	0.963467468	4.82E-07
TUCP_003347	XLOC_144849	ENSRNOT00000009627	0.979367858	2.84E-08
TUCP_003347	XLOC_144849	ENSRNOT00000016947	0.95034513	2.19E-06
TUCP_003347	XLOC_144849	ENSRNOT00000073991	0.961831526	5.98E-07
TUCP_003347	XLOC_144849	ENSRNOT00000029994	0.974411144	8.28E-08
TUCP_003347	XLOC_144849	ENSRNOT00000000676	0.96840944	2.35E-07
TUCP_003347	XLOC_144849	ENSRNOT00000078675	0.977953277	3.95E-08
TUCP_003347	XLOC_144849	ENSRNOT00000019222	0.957053102	1.07E-06
TUCP_003347	XLOC_144849	ENSRNOT00000004359	0.954149538	1.48E-06
TUCP_003347	XLOC_144849	ENSRNOT00000013274	0.967786375	2.59E-07

TUCP_003347	XLOC_144849	ENSRNOT00000085027	0.95602825	1.20E-06
TUCP_003347	XLOC_144849	ENSRNOT00000002403	0.969498975	1.98E-07
TUCP_003347	XLOC_144849	ENSRNOT00000045574	0.952117412	1.83E-06
TUCP_003347	XLOC_144849	ENSRNOT00000027337	0.981929344	1.47E-08
TUCP_003347	XLOC_144849	ENSRNOT00000047093	0.962156779	5.74E-07
TUCP_003347	XLOC_144849	ENSRNOT00000001222	0.952169069	1.82E-06
TUCP_003347	XLOC_144849	ENSRNOT00000076404	0.95306497	1.66E-06
TUCP_003347	XLOC_144849	ENSRNOT00000003671	0.960619358	6.98E-07
TUCP_003347	XLOC_144849	ENSRNOT00000047387	0.971594095	1.39E-07
TUCP_003347	XLOC_144849	ENSRNOT00000055680	0.966885838	2.97E-07
TUCP_003347	XLOC_144849	ENSRNOT00000091910	0.967794306	2.58E-07
TUCP_003347	XLOC_144849	ENSRNOT00000045488	0.963720324	4.66E-07
TUCP_003347	XLOC_144849	ENSRNOT00000017629	0.965414268	3.68E-07
TUCP_003347	XLOC_144849	ENSRNOT00000009683	0.968962692	2.15E-07
TUCP_003347	XLOC_144849	ENSRNOT00000084088	0.952443065	1.77E-06
TUCP_003347	XLOC_144849	ENSRNOT00000079442	0.970090766	1.79E-07
TUCP_003347	XLOC_144849	ENSRNOT00000024322	0.954514759	1.42E-06
TUCP_003347	XLOC_144849	ENSRNOT00000090665	0.971107749	1.51E-07
TUCP_003347	XLOC_144849	ENSRNOT00000085596	0.956783589	1.10E-06
TUCP_003347	XLOC_144849	ENSRNOT00000081897	0.967451117	2.72E-07
TUCP_003347	XLOC_144849	ENSRNOT00000000658	0.968378375	2.36E-07
TUCP_003347	XLOC_144849	ENSRNOT00000089971	0.983517597	9.32E-09
TUCP_003347	XLOC_144849	ENSRNOT00000065501	0.972453145	1.19E-07
TUCP_003347	XLOC_144849	ENSRNOT00000091488	0.956757967	1.11E-06
TUCP_003347	XLOC_144849	ENSRNOT00000027452	0.956371275	1.16E-06
TUCP_003347	XLOC_144849	ENSRNOT00000076729	0.969186453	2.08E-07
TUCP_003347	XLOC_144849	ENSRNOT00000066739	0.971144741	1.50E-07
TUCP_003347	XLOC_144849	ENSRNOT00000045882	0.964165486	4.38E-07
TUCP_003347	XLOC_144849	ENSRNOT00000037790	0.976617956	5.29E-08
TUCP_003347	XLOC_144849	ENSRNOT00000088054	0.967109394	2.87E-07
TUCP_003347	XLOC_144849	ENSRNOT00000006804	0.950777413	2.09E-06
TUCP_003347	XLOC_144849	ENSRNOT00000000157	0.954321784	1.45E-06
TUCP_003347	XLOC_144849	ENSRNOT00000010992	0.96198468	5.87E-07
TUCP_003347	XLOC_144849	ENSRNOT00000038647	0.98262861	1.21E-08
TUCP_003347	XLOC_144849	ENSRNOT00000083093	0.970951236	1.55E-07
TUCP_003347	XLOC_144849	ENSRNOT00000076669	0.955152682	1.32E-06
TUCP_003347	XLOC_144849	ENSRNOT00000011984	0.957684622	9.95E-07
TUCP_003347	XLOC_144849	ENSRNOT00000024402	0.952071005	1.84E-06
TUCP_003347	XLOC_144849	ENSRNOT00000026293	0.963331817	4.91E-07
TUCP_003347	XLOC_144849	ENSRNOT00000089007	-0.956467272	1.14E-06
TUCP_003347	XLOC_144849	ENSRNOT00000012736	0.97878132	3.27E-08
TUCP_003347	XLOC_144849	ENSRNOT00000041096	0.957289822	1.04E-06
TUCP_003347	XLOC_144849	ENSRNOT00000031230	0.956965782	1.08E-06
TUCP_003347	XLOC_144849	ENSRNOT00000074361	0.959320847	8.19E-07
TUCP_003347	XLOC_144849	ENSRNOT00000083127	0.972012181	1.29E-07
TUCP_003347	XLOC_144849	ENSRNOT00000023302	0.957872906	9.73E-07
TUCP_003347	XLOC_144849	ENSRNOT00000060696	0.971156416	1.50E-07
TUCP_003347	XLOC_144849	ENSRNOT00000012048	0.959861138	7.67E-07
TUCP_003347	XLOC_144849	ENSRNOT00000026280	0.957175629	1.06E-06
TUCP_003347	XLOC_144849	ENSRNOT00000005387	0.961388283	6.33E-07
TUCP_003347	XLOC_144849	ENSRNOT00000024941	0.955320619	1.30E-06

TUCP_003347	XLOC_144849	ENSRNOT00000005381	0.968276895	2.40E-07
TUCP_003347	XLOC_144849	ENSRNOT00000034704	0.966497204	3.14E-07
TUCP_003347	XLOC_144849	ENSRNOT00000013985	0.95384722	1.53E-06
TUCP_003347	XLOC_144849	ENSRNOT00000036750	0.959881575	7.65E-07
TUCP_003347	XLOC_144849	ENSRNOT00000025092	0.952241505	1.81E-06
TUCP_003347	XLOC_144849	ENSRNOT00000022796	0.971906522	1.31E-07
TUCP_003347	XLOC_144849	ENSRNOT00000029001	0.976864424	5.02E-08
TUCP_003347	XLOC_144849	ENSRNOT00000083799	0.958125898	9.45E-07
TUCP_003344	XLOC_144746	ENSRNOT00000030479	0.95792469	9.68E-07
TUCP_003344	XLOC_144746	ENSRNOT00000025093	0.95568909	1.25E-06
TUCP_003344	XLOC_144746	ENSRNOT00000061686	0.950713542	2.11E-06
TUCP_003344	XLOC_144746	ENSRNOT00000024079	0.972296204	1.23E-07
TUCP_003344	XLOC_144746	ENSRNOT00000018470	0.960996346	6.66E-07
TUCP_003344	XLOC_144746	ENSRNOT00000005374	0.951556755	1.94E-06
TUCP_003344	XLOC_144746	ENSRNOT00000020889	0.955232935	1.31E-06
TUCP_003344	XLOC_144746	ENSRNOT00000083560	0.956831761	1.10E-06
TUCP_003344	XLOC_144746	ENSRNOT00000008909	0.954155636	1.48E-06
TUCP_003344	XLOC_144746	ENSRNOT00000007133	0.964119874	4.41E-07
TUCP_003344	XLOC_144746	ENSRNOT00000027060	0.966287297	3.24E-07
TUCP_003344	XLOC_144746	ENSRNOT00000024707	0.953200758	1.63E-06
TUCP_003345	XLOC_144811	ENSRNOT00000028535	-0.9644868	4.19E-07
TUCP_003342	XLOC_144618	ENSRNOT00000013423	0.970528424	1.67E-07
TUCP_003342	XLOC_144618	ENSRNOT00000009825	0.962775582	5.29E-07
TUCP_003342	XLOC_144618	ENSRNOT00000046585	0.962168508	5.73E-07
TUCP_003342	XLOC_144618	ENSRNOT00000061185	0.953028901	1.66E-06
TUCP_003342	XLOC_144618	ENSRNOT00000031205	0.955933722	1.22E-06
TUCP_003342	XLOC_144618	ENSRNOT00000021243	0.972366293	1.21E-07
TUCP_003342	XLOC_144618	ENSRNOT00000088381	0.961930717	5.91E-07
TUCP_003342	XLOC_144618	ENSRNOT00000078663	0.961983501	5.87E-07
TUCP_003342	XLOC_144618	ENSRNOT00000015866	0.962099434	5.78E-07
TUCP_003342	XLOC_144618	ENSRNOT00000064613	0.976443944	5.49E-08
TUCP_003342	XLOC_144618	ENSRNOT00000026932	0.963254633	4.96E-07
TUCP_003343	XLOC_144746	ENSRNOT00000084500	0.984887141	6.05E-09
TUCP_003343	XLOC_144746	ENSRNOT00000093467	0.996134431	6.75E-12
TUCP_003343	XLOC_144746	ENSRNOT00000029340	0.999116572	4.23E-15
TUCP_003343	XLOC_144746	ENSRNOT00000077972	0.962758032	5.30E-07
TUCP_003343	XLOC_144746	ENSRNOT00000038646	1	0
TUCP_003343	XLOC_144746	ENSRNOT00000012095	0.957750287	9.88E-07
TUCP_003343	XLOC_144746	ENSRNOT00000017909	0.975587192	6.56E-08
TUCP_003343	XLOC_144746	ENSRNOT00000092579	1	0
TUCP_003343	XLOC_144746	ENSRNOT00000089235	0.998661162	3.38E-14
TUCP_003343	XLOC_144746	ENSRNOT00000036383	0.988460809	1.58E-09
TUCP_003343	XLOC_144746	ENSRNOT00000076528	0.997115373	1.57E-12
TUCP_003343	XLOC_144746	ENSRNOT00000075193	0.964326971	4.29E-07
TUCP_003343	XLOC_144746	ENSRNOT00000083034	0.990934646	4.75E-10
TUCP_003343	XLOC_144746	ENSRNOT00000089607	0.995091455	2.23E-11
TUCP_003343	XLOC_144746	ENSRNOT00000086014	0.960893638	6.74E-07
TUCP_003343	XLOC_144746	ENSRNOT00000054939	0.958021495	9.57E-07
TUCP_003343	XLOC_144746	ENSRNOT00000052315	0.983687082	8.85E-09
TUCP_003343	XLOC_144746	ENSRNOT00000088168	1	0
TUCP_003343	XLOC_144746	ENSRNOT00000046231	0.96225463	5.66E-07

TUCP_003343	XLOC_144746	ENSRNOT00000013459	0.979098017	3.03E-08
TUCP_003343	XLOC_144746	ENSRNOT00000078137	1	0
TUCP_003343	XLOC_144746	ENSRNOT00000077764	0.977143528	4.73E-08
TUCP_003343	XLOC_144746	ENSRNOT00000043661	0.966975951	2.93E-07
TUCP_003343	XLOC_144746	ENSRNOT00000092740	1	0
TUCP_003343	XLOC_144746	ENSRNOT00000000117	0.990989174	4.61E-10
TUCP_003343	XLOC_144746	ENSRNOT00000071877	0.967113583	2.87E-07
TUCP_003343	XLOC_144746	ENSRNOT00000070958	1	0
TUCP_003343	XLOC_144746	ENSRNOT00000076757	0.959205354	8.31E-07
TUCP_003343	XLOC_144746	ENSRNOT00000085310	0.965230243	3.78E-07
TUCP_003343	XLOC_144746	ENSRNOT00000047241	0.966576993	3.11E-07
TUCP_003343	XLOC_144746	ENSRNOT00000027268	1	0
TUCP_003343	XLOC_144746	ENSRNOT00000004459	1	0
TUCP_003343	XLOC_144746	ENSRNOT00000048373	0.959096578	8.42E-07
TUCP_003343	XLOC_144746	ENSRNOT00000061516	0.987595648	2.27E-09
TUCP_003343	XLOC_144746	ENSRNOT00000088148	1	0
TUCP_003343	XLOC_144746	ENSRNOT00000088527	1	0
TUCP_003343	XLOC_144746	ENSRNOT00000078701	1	0
TUCP_003343	XLOC_144746	ENSRNOT00000052308	0.962646127	5.38E-07
TUCP_003343	XLOC_144746	ENSRNOT00000021748	1	0
TUCP_003343	XLOC_144746	ENSRNOT00000089085	1	0
TUCP_003343	XLOC_144746	ENSRNOT00000008153	0.999703506	1.80E-17
TUCP_003343	XLOC_144746	ENSRNOT00000085896	0.971605382	1.39E-07
TUCP_003343	XLOC_144746	ENSRNOT00000079189	0.984972387	5.89E-09
TUCP_003343	XLOC_144746	ENSRNOT00000090078	1	0
TUCP_003343	XLOC_144746	ENSRNOT00000078020	0.958421666	9.13E-07
TUCP_003343	XLOC_144746	ENSRNOT00000092425	0.956092273	1.19E-06
TUCP_003343	XLOC_144746	ENSRNOT00000009776	1	0
TUCP_003340	XLOC_144386	ENSRNOT00000080945	0.951586227	1.93E-06
TUCP_003340	XLOC_144386	ENSRNOT00000075035	0.962914465	5.19E-07
TUCP_003340	XLOC_144386	ENSRNOT00000065111	0.962972509	5.15E-07
TUCP_003340	XLOC_144386	ENSRNOT00000061569	0.954607578	1.41E-06
TUCP_003340	XLOC_144386	ENSRNOT00000016804	0.978287558	3.66E-08
TUCP_003340	XLOC_144386	ENSRNOT00000019551	0.963204513	4.99E-07
TUCP_004603	XLOC_202488	ENSRNOT00000067037	-0.976180481	5.80E-08
TUCP_004603	XLOC_202488	ENSRNOT00000021801	-0.954171112	1.47E-06
TUCP_003023	XLOC_128683	ENSRNOT00000091116	0.982849702	1.14E-08
TUCP_003023	XLOC_128683	ENSRNOT00000077186	0.956854282	1.10E-06
TUCP_003023	XLOC_128683	ENSRNOT00000056199	0.957411662	1.03E-06
TUCP_003023	XLOC_128683	ENSRNOT00000081314	0.95067231	2.12E-06
TUCP_003023	XLOC_128683	ENSRNOT00000039607	0.967610741	2.66E-07
TUCP_003023	XLOC_128683	ENSRNOT00000090621	-0.952077749	1.84E-06
TUCP_003023	XLOC_128683	ENSRNOT00000030996	0.95553733	1.27E-06
TUCP_003023	XLOC_128683	ENSRNOT00000086946	0.966543377	3.12E-07
TUCP_003023	XLOC_128683	ENSRNOT00000006460	-0.953824229	1.53E-06
TUCP_003023	XLOC_128683	ENSRNOT00000011237	0.963845782	4.58E-07
TUCP_003023	XLOC_128683	ENSRNOT00000080418	0.967050138	2.89E-07
TUCP_003023	XLOC_128683	ENSRNOT00000002222	-0.955800157	1.23E-06
TUCP_003023	XLOC_128683	ENSRNOT00000003087	-0.963242259	4.97E-07
TUCP_003023	XLOC_128683	ENSRNOT00000019358	0.951102571	2.03E-06
TUCP_003023	XLOC_128683	ENSRNOT00000049349	-0.974912068	7.50E-08

TUCP_003023	XLOC_128683	ENSRNOT00000086605	0.956497034	1.14E-06
TUCP_003023	XLOC_128683	ENSRNOT00000014002	0.956254494	1.17E-06
TUCP_003023	XLOC_128683	ENSRNOT00000041237	0.966084977	3.34E-07
TUCP_003023	XLOC_128683	ENSRNOT00000079138	-0.954758955	1.38E-06
TUCP_003023	XLOC_128683	ENSRNOT00000005658	0.973336861	1.01E-07
TUCP_003023	XLOC_128683	ENSRNOT00000087011	0.960936566	6.71E-07
TUCP_003023	XLOC_128683	ENSRNOT00000002329	-0.974497603	8.14E-08
TUCP_003023	XLOC_128683	ENSRNOT00000078326	0.963345346	4.90E-07
TUCP_003023	XLOC_128683	ENSRNOT00000087329	0.962874363	5.22E-07
TUCP_003023	XLOC_128683	ENSRNOT00000010031	0.950127184	2.23E-06
TUCP_003023	XLOC_128683	ENSRNOT00000011130	0.953215589	1.63E-06
TUCP_003023	XLOC_128683	ENSRNOT00000008361	-0.958024747	9.56E-07
TUCP_003023	XLOC_128683	ENSRNOT00000025309	-0.959780243	7.75E-07
TUCP_003023	XLOC_128683	ENSRNOT00000004351	0.951223966	2.00E-06
TUCP_003023	XLOC_128683	ENSRNOT00000024712	0.95174864	1.90E-06
TUCP_003023	XLOC_128683	ENSRNOT00000019180	0.962007926	5.85E-07
TUCP_003023	XLOC_128683	ENSRNOT00000042459	0.95993721	7.60E-07
TUCP_003023	XLOC_128683	ENSRNOT00000021826	-0.971883497	1.32E-07
TUCP_003023	XLOC_128683	ENSRNOT00000018341	0.953017299	1.67E-06
TUCP_003023	XLOC_128683	ENSRNOT00000013613	0.950041553	2.25E-06
TUCP_003023	XLOC_128683	ENSRNOT00000010419	-0.951713374	1.91E-06
TUCP_003023	XLOC_128683	ENSRNOT00000057738	-0.953254695	1.62E-06
TUCP_003023	XLOC_128683	ENSRNOT00000031672	0.95327852	1.62E-06
TUCP_003023	XLOC_128683	ENSRNOT00000021537	-0.950025751	2.26E-06
TUCP_003023	XLOC_128683	ENSRNOT00000022746	0.950210171	2.22E-06
TUCP_003023	XLOC_128683	ENSRNOT00000024882	0.956478885	1.14E-06
TUCP_003023	XLOC_128683	ENSRNOT00000025284	0.95048564	2.16E-06
TUCP_003023	XLOC_128683	ENSRNOT00000018622	0.952031814	1.84E-06
TUCP_003023	XLOC_128683	ENSRNOT00000001360	0.956820505	1.10E-06
TUCP_003023	XLOC_128683	ENSRNOT00000012060	-0.951257723	2.00E-06
TUCP_003023	XLOC_128683	ENSRNOT00000075480	0.952475727	1.76E-06
TUCP_003023	XLOC_128683	ENSRNOT00000005620	0.968794756	2.21E-07
TUCP_003023	XLOC_128683	ENSRNOT00000014044	0.954212838	1.47E-06
TUCP_003023	XLOC_128683	ENSRNOT00000072389	0.951190234	2.01E-06
TUCP_003023	XLOC_128683	ENSRNOT00000013228	-0.957860052	9.75E-07
TUCP_003023	XLOC_128683	ENSRNOT00000027760	-0.964755399	4.04E-07
TUCP_003023	XLOC_128683	ENSRNOT00000048529	0.953731588	1.54E-06
TUCP_003023	XLOC_128683	ENSRNOT00000033795	0.970781257	1.60E-07
TUCP_003023	XLOC_128683	ENSRNOT00000045041	0.960184685	7.37E-07
TUCP_003023	XLOC_128683	ENSRNOT00000092757	-0.972844974	1.11E-07
TUCP_003023	XLOC_128683	ENSRNOT00000001077	-0.968873468	2.18E-07
TUCP_003023	XLOC_128683	ENSRNOT00000000477	0.954202981	1.47E-06
TUCP_003023	XLOC_128683	ENSRNOT00000082739	0.96559067	3.59E-07
TUCP_003023	XLOC_128683	ENSRNOT00000000576	0.968428975	2.34E-07
TUCP_003023	XLOC_128683	ENSRNOT00000024344	0.958741203	8.78E-07
TUCP_003023	XLOC_128683	ENSRNOT00000000469	-0.955394701	1.29E-06
TUCP_003023	XLOC_128683	ENSRNOT00000030065	0.955383888	1.29E-06
TUCP_003023	XLOC_128683	ENSRNOT00000011948	0.956104099	1.19E-06
TUCP_003023	XLOC_128683	ENSRNOT00000017648	0.974037819	8.89E-08
TUCP_003023	XLOC_128683	ENSRNOT00000013764	0.962572391	5.43E-07
TUCP_003023	XLOC_128683	ENSRNOT00000065045	-0.957518348	1.01E-06

TUCP_003023	XLOC_128683	ENSRNOT00000075630	0.952295075	1.80E-06
TUCP_003023	XLOC_128683	ENSRNOT00000023463	0.953658031	1.56E-06
TUCP_003023	XLOC_128683	ENSRNOT00000081631	-0.952779713	1.71E-06
TUCP_003023	XLOC_128683	ENSRNOT00000052098	0.966316336	3.23E-07
TUCP_003023	XLOC_128683	ENSRNOT00000018634	0.953396335	1.60E-06
TUCP_003023	XLOC_128683	ENSRNOT00000001806	0.954137233	1.48E-06
TUCP_003023	XLOC_128683	ENSRNOT00000046490	0.959556184	7.96E-07
TUCP_003023	XLOC_128683	ENSRNOT00000023763	0.950042837	2.25E-06
TUCP_003023	XLOC_128683	ENSRNOT00000073643	0.952640243	1.73E-06
TUCP_003023	XLOC_128683	ENSRNOT00000046552	-0.969123822	2.10E-07
TUCP_003023	XLOC_128683	ENSRNOT00000024801	0.959895248	7.64E-07
TUCP_003023	XLOC_128683	ENSRNOT00000023613	-0.956033778	1.20E-06
TUCP_003023	XLOC_128683	ENSRNOT00000005961	0.969096705	2.11E-07
TUCP_003023	XLOC_128683	ENSRNOT00000067391	0.957369862	1.03E-06
TUCP_003023	XLOC_128683	ENSRNOT00000033806	-0.955641881	1.26E-06
TUCP_003023	XLOC_128683	ENSRNOT00000041808	0.957953517	9.64E-07
TUCP_003023	XLOC_128683	ENSRNOT00000037333	0.970452005	1.69E-07
TUCP_003023	XLOC_128683	ENSRNOT00000018237	0.954364678	1.44E-06
TUCP_003023	XLOC_128683	ENSRNOT00000029179	0.957455084	1.02E-06
TUCP_003023	XLOC_128683	ENSRNOT00000085545	0.966892844	2.96E-07
TUCP_003023	XLOC_128683	ENSRNOT00000083188	0.957028482	1.07E-06
TUCP_003023	XLOC_128683	ENSRNOT00000011082	0.96845431	2.33E-07
TUCP_003023	XLOC_128683	ENSRNOT00000039850	0.951516921	1.94E-06
TUCP_003023	XLOC_128683	ENSRNOT00000083344	-0.962679142	5.36E-07
TUCP_003023	XLOC_128683	ENSRNOT00000005064	0.964760633	4.03E-07
TUCP_003023	XLOC_128683	ENSRNOT00000014502	0.953450904	1.59E-06
TUCP_003023	XLOC_128683	ENSRNOT00000085334	-0.953204074	1.63E-06
TUCP_003023	XLOC_128683	ENSRNOT00000000817	0.962360245	5.59E-07
TUCP_003023	XLOC_128683	ENSRNOT00000014506	0.956064891	1.20E-06
TUCP_003023	XLOC_128683	ENSRNOT00000074515	0.963140254	5.04E-07
TUCP_003023	XLOC_128683	ENSRNOT00000014859	0.958178214	9.39E-07
TUCP_003023	XLOC_128683	ENSRNOT00000022987	0.952667461	1.73E-06
TUCP_003023	XLOC_128683	ENSRNOT00000000812	0.965740093	3.51E-07
TUCP_003023	XLOC_128683	ENSRNOT00000001322	0.950266943	2.20E-06
TUCP_003023	XLOC_128683	ENSRNOT00000036658	0.955878077	1.22E-06
TUCP_003023	XLOC_128683	ENSRNOT00000089611	-0.964954555	3.93E-07
TUCP_003023	XLOC_128683	ENSRNOT00000002645	0.954471524	1.43E-06
TUCP_003023	XLOC_128683	ENSRNOT00000008317	0.95999688	7.54E-07
TUCP_003023	XLOC_128683	ENSRNOT00000077040	0.956229492	1.18E-06
TUCP_003023	XLOC_128683	ENSRNOT00000087517	-0.951156561	2.02E-06
TUCP_003023	XLOC_128683	ENSRNOT00000008772	0.958367308	9.18E-07
TUCP_003023	XLOC_128683	ENSRNOT00000026726	0.955275744	1.31E-06
TUCP_003023	XLOC_128683	ENSRNOT00000068026	0.954363831	1.44E-06
TUCP_003023	XLOC_128683	ENSRNOT00000001160	0.951498157	1.95E-06
TUCP_003023	XLOC_128683	ENSRNOT00000041116	0.967681567	2.63E-07
TUCP_003023	XLOC_128683	ENSRNOT00000078737	0.958046082	9.54E-07
TUCP_003023	XLOC_128683	ENSRNOT00000010680	-0.951172127	2.01E-06
TUCP_003023	XLOC_128683	ENSRNOT00000064901	-0.965759958	3.50E-07
TUCP_003023	XLOC_128683	ENSRNOT00000025824	0.959245825	8.27E-07
TUCP_003023	XLOC_128683	ENSRNOT00000071929	0.957700545	9.93E-07
TUCP_003023	XLOC_128683	ENSRNOT00000005172	-0.958045605	9.54E-07

TUCP_003023	XLOC_128683	ENSRNOT00000005170	0.975590054	6.55E-08
TUCP_003023	XLOC_128683	ENSRNOT00000017746	0.951658055	1.92E-06
TUCP_003023	XLOC_128683	ENSRNOT00000016286	0.952889004	1.69E-06
TUCP_003023	XLOC_128683	ENSRNOT00000017626	0.959295616	8.22E-07
TUCP_003023	XLOC_128683	ENSRNOT00000012495	0.952404377	1.78E-06
TUCP_003023	XLOC_128683	ENSRNOT00000070825	0.959722687	7.80E-07
TUCP_003023	XLOC_128683	ENSRNOT00000025134	0.954117309	1.48E-06
TUCP_003023	XLOC_128683	ENSRNOT00000020472	0.956377989	1.16E-06
TUCP_003023	XLOC_128683	ENSRNOT00000022086	0.955588428	1.26E-06
TUCP_003023	XLOC_128683	ENSRNOT00000066923	0.960716335	6.90E-07
TUCP_003023	XLOC_128683	ENSRNOT00000003733	0.980957091	1.91E-08
TUCP_003023	XLOC_128683	ENSRNOT00000012767	-0.950879783	2.07E-06
TUCP_003023	XLOC_128683	ENSRNOT00000029701	-0.975111175	7.21E-08
TUCP_003023	XLOC_128683	ENSRNOT00000054717	0.958503694	9.04E-07
TUCP_003023	XLOC_128683	ENSRNOT00000028744	0.955638568	1.26E-06
TUCP_003023	XLOC_128683	ENSRNOT00000029704	0.963269687	4.95E-07
TUCP_003023	XLOC_128683	ENSRNOT00000056015	0.960997638	6.66E-07
TUCP_003023	XLOC_128683	ENSRNOT00000001013	0.958196653	9.37E-07
TUCP_003023	XLOC_128683	ENSRNOT00000055602	0.955419093	1.29E-06
TUCP_003023	XLOC_128683	ENSRNOT00000037015	-0.955309105	1.30E-06
TUCP_003023	XLOC_128683	ENSRNOT00000016324	0.978429949	3.55E-08
TUCP_003023	XLOC_128683	ENSRNOT00000082627	0.958101619	9.48E-07
TUCP_003023	XLOC_128683	ENSRNOT00000077578	0.969471841	1.98E-07
TUCP_003023	XLOC_128683	ENSRNOT00000014832	0.952380977	1.78E-06
TUCP_003023	XLOC_128683	ENSRNOT00000089879	0.971125139	1.51E-07
TUCP_003023	XLOC_128683	ENSRNOT00000066952	-0.957767577	9.86E-07
TUCP_003023	XLOC_128683	ENSRNOT00000000258	0.976071078	5.94E-08
TUCP_003023	XLOC_128683	ENSRNOT00000018477	0.956570403	1.13E-06
TUCP_003023	XLOC_128683	ENSRNOT00000087905	0.985671326	4.64E-09
TUCP_003023	XLOC_128683	ENSRNOT00000090939	0.950447814	2.16E-06
TUCP_003023	XLOC_128683	ENSRNOT00000074079	0.952121615	1.83E-06
TUCP_003023	XLOC_128683	ENSRNOT00000027553	0.964368695	4.26E-07
TUCP_003023	XLOC_128683	ENSRNOT00000049048	0.95425979	1.46E-06
TUCP_003023	XLOC_128683	ENSRNOT00000018149	0.961130985	6.55E-07
TUCP_003023	XLOC_128683	ENSRNOT00000075602	0.972449503	1.19E-07
TUCP_003023	XLOC_128683	ENSRNOT00000059169	-0.953503559	1.58E-06
TUCP_003023	XLOC_128683	ENSRNOT00000044477	0.950354058	2.18E-06
TUCP_003023	XLOC_128683	ENSRNOT00000052131	0.95456889	1.41E-06
TUCP_003023	XLOC_128683	ENSRNOT00000039177	0.959536676	7.98E-07
TUCP_003023	XLOC_128683	ENSRNOT00000086456	0.968134463	2.45E-07
TUCP_003023	XLOC_128683	ENSRNOT00000057953	-0.950115805	2.24E-06
TUCP_003023	XLOC_128683	ENSRNOT00000047572	0.967798369	2.58E-07
TUCP_003023	XLOC_128683	ENSRNOT00000019040	0.954329886	1.45E-06
TUCP_003023	XLOC_128683	ENSRNOT00000006802	-0.957621046	1.00E-06
TUCP_003023	XLOC_128683	ENSRNOT00000056145	0.954806273	1.38E-06
TUCP_003023	XLOC_128683	ENSRNOT00000001227	0.960670787	6.94E-07
TUCP_003023	XLOC_128683	ENSRNOT00000009202	-0.96928806	2.04E-07
TUCP_003023	XLOC_128683	ENSRNOT00000080013	0.95239005	1.78E-06
TUCP_003023	XLOC_128683	ENSRNOT00000067217	0.956475445	1.14E-06
TUCP_003023	XLOC_128683	ENSRNOT00000064290	0.982888365	1.12E-08
TUCP_003023	XLOC_128683	ENSRNOT00000012663	0.956150728	1.19E-06

TUCP_003023	XLOC_128683	ENSRNOT00000003106	0.968552736	2.30E-07
TUCP_003023	XLOC_128683	ENSRNOT00000005873	0.978485172	3.50E-08
TUCP_003023	XLOC_128683	ENSRNOT00000025634	0.96794131	2.53E-07
TUCP_003023	XLOC_128683	ENSRNOT00000000253	0.969324734	2.03E-07
TUCP_003023	XLOC_128683	ENSRNOT00000086847	0.961302011	6.40E-07
TUCP_003023	XLOC_128683	ENSRNOT00000008905	0.972104661	1.27E-07
TUCP_003023	XLOC_128683	ENSRNOT00000018674	0.955196011	1.32E-06
TUCP_003023	XLOC_128683	ENSRNOT00000009994	0.953124665	1.65E-06
TUCP_003023	XLOC_128683	ENSRNOT00000077363	0.970407404	1.70E-07
TUCP_003023	XLOC_128683	ENSRNOT00000023014	-0.961586374	6.18E-07
TUCP_003023	XLOC_128683	ENSRNOT00000028737	0.970630699	1.64E-07
TUCP_003023	XLOC_128683	ENSRNOT00000026518	-0.957129739	1.06E-06
TUCP_003023	XLOC_128683	ENSRNOT00000027973	0.956645838	1.12E-06
TUCP_003023	XLOC_128683	ENSRNOT00000024841	0.951592242	1.93E-06
TUCP_003023	XLOC_128683	ENSRNOT00000012847	0.969664405	1.92E-07
TUCP_003023	XLOC_128683	ENSRNOT00000044147	0.962090971	5.79E-07
TUCP_003023	XLOC_128683	ENSRNOT00000027875	-0.952020058	1.85E-06
TUCP_003023	XLOC_128683	ENSRNOT00000023641	0.958269055	9.29E-07
TUCP_003023	XLOC_128683	ENSRNOT00000003593	-0.971550243	1.40E-07
TUCP_003023	XLOC_128683	ENSRNOT00000015573	0.951769755	1.90E-06
TUCP_003023	XLOC_128683	ENSRNOT00000018378	0.95424057	1.46E-06
TUCP_003023	XLOC_128683	ENSRNOT00000008244	-0.955812856	1.23E-06
TUCP_003023	XLOC_128683	ENSRNOT00000064636	0.988025509	1.90E-09
TUCP_003023	XLOC_128683	ENSRNOT00000032893	0.957365591	1.03E-06
TUCP_003023	XLOC_128683	ENSRNOT00000056725	0.952297979	1.80E-06
TUCP_003023	XLOC_128683	ENSRNOT00000022676	0.955298393	1.30E-06
TUCP_003023	XLOC_128683	ENSRNOT00000013733	0.957200048	1.05E-06
TUCP_003023	XLOC_128683	ENSRNOT00000012428	0.964246225	4.33E-07
TUCP_003023	XLOC_128683	ENSRNOT00000025782	0.959406671	8.11E-07
TUCP_003023	XLOC_128683	ENSRNOT00000075800	0.958140911	9.43E-07
TUCP_003023	XLOC_128683	ENSRNOT00000018473	0.967492235	2.71E-07
TUCP_003023	XLOC_128683	ENSRNOT00000066199	0.959139108	8.37E-07
TUCP_003023	XLOC_128683	ENSRNOT00000017998	-0.955914493	1.22E-06
TUCP_003023	XLOC_128683	ENSRNOT00000004094	0.952233501	1.81E-06
TUCP_003023	XLOC_128683	ENSRNOT00000027360	0.969047648	2.12E-07
TUCP_003023	XLOC_128683	ENSRNOT00000013431	0.971319422	1.46E-07
TUCP_003023	XLOC_128683	ENSRNOT00000008253	0.951549655	1.94E-06
TUCP_003023	XLOC_128683	ENSRNOT00000025149	0.958547989	8.99E-07
TUCP_003023	XLOC_128683	ENSRNOT00000024938	0.957338361	1.04E-06
TUCP_003023	XLOC_128683	ENSRNOT00000016776	0.95297722	1.67E-06
TUCP_003023	XLOC_128683	ENSRNOT00000047152	-0.950891593	2.07E-06
TUCP_003023	XLOC_128683	ENSRNOT00000015199	0.959133037	8.38E-07
TUCP_003023	XLOC_128683	ENSRNOT00000010020	0.956587236	1.13E-06
TUCP_003023	XLOC_128683	ENSRNOT00000038464	0.950500633	2.15E-06
TUCP_003023	XLOC_128683	ENSRNOT00000036194	0.952791393	1.71E-06
TUCP_003023	XLOC_128683	ENSRNOT00000029696	0.954950133	1.35E-06
TUCP_004325	XLOC_190552	ENSRNOT00000015366	0.972332952	1.22E-07
TUCP_004325	XLOC_190552	ENSRNOT00000074465	0.95217836	1.82E-06
TUCP_004325	XLOC_190552	ENSRNOT00000040912	0.95217836	1.82E-06
TUCP_004325	XLOC_190552	ENSRNOT00000072648	0.95217836	1.82E-06
TUCP_004325	XLOC_190552	ENSRNOT00000070803	0.95217836	1.82E-06

TUCP_004325	XLOC_190552	ENSRNOT00000012196	0.95217836	1.82E-06
TUCP_004325	XLOC_190552	ENSRNOT00000085772	0.95217836	1.82E-06
TUCP_004325	XLOC_190552	ENSRNOT00000086374	0.952925164	1.68E-06
TUCP_004325	XLOC_190552	ENSRNOT00000015270	0.95217836	1.82E-06
TUCP_004325	XLOC_190552	ENSRNOT00000046192	0.95217836	1.82E-06
TUCP_004325	XLOC_190552	ENSRNOT00000044226	0.95217836	1.82E-06
TUCP_004325	XLOC_190552	ENSRNOT00000081898	0.95475253	1.38E-06
TUCP_004325	XLOC_190552	ENSRNOT00000004293	0.962100106	5.78E-07
TUCP_004325	XLOC_190552	ENSRNOT00000015870	0.957363685	1.03E-06
TUCP_004325	XLOC_190552	ENSRNOT00000001464	0.95217836	1.82E-06
TUCP_004325	XLOC_190552	ENSRNOT00000057873	0.95217836	1.82E-06
TUCP_004325	XLOC_190552	ENSRNOT00000076044	0.95217836	1.82E-06
TUCP_004325	XLOC_190552	ENSRNOT00000074353	0.95217836	1.82E-06
TUCP_004325	XLOC_190552	ENSRNOT00000074295	0.96971567	1.91E-07
TUCP_004325	XLOC_190552	ENSRNOT00000093284	0.951913185	1.87E-06
TUCP_004325	XLOC_190552	ENSRNOT00000088399	0.952242793	1.81E-06
TUCP_004325	XLOC_190552	ENSRNOT00000086248	0.95217836	1.82E-06
TUCP_004325	XLOC_190552	ENSRNOT00000089750	0.952170613	1.82E-06
TUCP_004325	XLOC_190552	ENSRNOT00000078121	0.974782309	7.70E-08
TUCP_004325	XLOC_190552	ENSRNOT00000002834	0.95217836	1.82E-06
TUCP_004325	XLOC_190552	ENSRNOT00000080812	0.953937783	1.51E-06
TUCP_004325	XLOC_190552	ENSRNOT00000092755	0.95217836	1.82E-06
TUCP_004325	XLOC_190552	ENSRNOT00000031683	0.95217836	1.82E-06
TUCP_004325	XLOC_190552	ENSRNOT00000016843	0.980136382	2.36E-08
TUCP_002510	XLOC_105862	ENSRNOT00000089625	0.967420795	2.74E-07
TUCP_002511	XLOC_105963	ENSRNOT00000028234	0.957235394	1.05E-06
TUCP_002511	XLOC_105963	ENSRNOT00000016083	0.958366823	9.18E-07
TUCP_002511	XLOC_105963	ENSRNOT00000001098	0.951733623	1.90E-06
TUCP_002511	XLOC_105963	ENSRNOT00000049973	0.963685594	4.68E-07
TUCP_002511	XLOC_105963	ENSRNOT00000034096	0.954633105	1.40E-06
TUCP_002511	XLOC_105963	ENSRNOT00000088884	0.963942739	4.52E-07
TUCP_002511	XLOC_105963	ENSRNOT00000003514	0.956105821	1.19E-06
TUCP_002511	XLOC_105963	ENSRNOT00000029097	0.9697844	1.89E-07
TUCP_002511	XLOC_105963	ENSRNOT00000083983	0.951729086	1.90E-06
TUCP_002511	XLOC_105963	ENSRNOT00000075494	0.955872885	1.22E-06
TUCP_002511	XLOC_105963	ENSRNOT00000001973	0.963921723	4.53E-07
TUCP_002511	XLOC_105963	ENSRNOT00000023149	0.967565865	2.68E-07
TUCP_002511	XLOC_105963	ENSRNOT00000009113	0.958295059	9.26E-07
TUCP_002511	XLOC_105963	ENSRNOT00000078599	0.981974188	1.45E-08
TUCP_002511	XLOC_105963	ENSRNOT00000051525	0.950049848	2.25E-06
TUCP_002511	XLOC_105963	ENSRNOT00000023736	0.953312582	1.61E-06
TUCP_002511	XLOC_105963	ENSRNOT00000007326	0.954286315	1.46E-06
TUCP_002511	XLOC_105963	ENSRNOT00000050227	0.976102751	5.90E-08
TUCP_002511	XLOC_105963	ENSRNOT00000059447	0.965971305	3.39E-07
TUCP_002511	XLOC_105963	ENSRNOT00000057978	0.961739069	6.06E-07
TUCP_002511	XLOC_105963	ENSRNOT00000072733	0.966288565	3.24E-07
TUCP_002511	XLOC_105963	ENSRNOT00000012017	0.950767234	2.10E-06
TUCP_002511	XLOC_105963	ENSRNOT00000017339	0.961667525	6.11E-07
TUCP_002511	XLOC_105963	ENSRNOT00000032173	0.959088947	8.43E-07
TUCP_002511	XLOC_105963	ENSRNOT00000017564	0.962723024	5.32E-07
TUCP_002511	XLOC_105963	ENSRNOT00000084860	0.950265294	2.20E-06

TUCP_002511	XLOC_105963	ENSRNOT00000009778	0.96135304	6.36E-07
TUCP_002511	XLOC_105963	ENSRNOT00000030823	0.955436289	1.28E-06
TUCP_002511	XLOC_105963	ENSRNOT00000088856	0.965927145	3.42E-07
TUCP_002511	XLOC_105963	ENSRNOT00000003728	0.968292497	2.39E-07
TUCP_003510	XLOC_152431	ENSRNOT00000077791	0.957146328	1.06E-06
TUCP_002513	XLOC_106085	ENSRNOT00000032588	-0.958699403	8.83E-07
TUCP_002513	XLOC_106085	ENSRNOT00000049848	-0.968928678	2.16E-07
TUCP_002513	XLOC_106085	ENSRNOT00000009687	0.954175411	1.47E-06
TUCP_002513	XLOC_106085	ENSRNOT00000004742	0.956035428	1.20E-06
TUCP_002513	XLOC_106085	ENSRNOT00000027809	-0.957861468	9.75E-07
TUCP_002513	XLOC_106085	ENSRNOT00000085316	0.952397441	1.78E-06
TUCP_002513	XLOC_106085	ENSRNOT00000049357	-0.978727539	3.31E-08
TUCP_002513	XLOC_106085	ENSRNOT00000024725	-0.952354076	1.78E-06
TUCP_002513	XLOC_106085	ENSRNOT00000018449	-0.96203912	5.82E-07
TUCP_002513	XLOC_106085	ENSRNOT00000068037	-0.954118234	1.48E-06
TUCP_002513	XLOC_106085	ENSRNOT00000083255	-0.956539152	1.14E-06
TUCP_002513	XLOC_106085	ENSRNOT00000088364	-0.95944133	8.07E-07
TUCP_002513	XLOC_106085	ENSRNOT00000091065	-0.950017166	2.26E-06
TUCP_002513	XLOC_106085	ENSRNOT00000028084	-0.969328217	2.03E-07
TUCP_003229	XLOC_138410	ENSRNOT00000050138	0.95640691	1.15E-06
TUCP_003229	XLOC_138410	ENSRNOT00000000743	0.951556659	1.94E-06
TUCP_003229	XLOC_138410	ENSRNOT00000081981	0.962464532	5.51E-07
TUCP_003229	XLOC_138410	ENSRNOT00000057924	0.981160862	1.81E-08
TUCP_003229	XLOC_138410	ENSRNOT00000024402	0.954672516	1.40E-06
TUCP_003229	XLOC_138410	ENSRNOT00000018527	-0.952691155	1.72E-06
TUCP_003229	XLOC_138410	ENSRNOT00000041044	0.964240949	4.34E-07
TUCP_003229	XLOC_138410	ENSRNOT00000068353	0.967381776	2.75E-07
TUCP_003229	XLOC_138410	ENSRNOT00000026691	0.972254816	1.24E-07
TUCP_003229	XLOC_138410	ENSRNOT00000011429	0.951178879	2.01E-06
TUCP_003229	XLOC_138410	ENSRNOT00000023638	0.95870148	8.83E-07
TUCP_003229	XLOC_138410	ENSRNOT00000040069	0.958124626	9.45E-07
TUCP_003229	XLOC_138410	ENSRNOT00000032992	0.957975594	9.62E-07
TUCP_003229	XLOC_138410	ENSRNOT00000040169	0.950459539	2.16E-06
TUCP_003229	XLOC_138410	ENSRNOT00000050834	-0.954374117	1.44E-06
TUCP_003229	XLOC_138410	ENSRNOT00000029764	-0.956352411	1.16E-06
TUCP_003229	XLOC_138410	ENSRNOT00000023144	0.951338471	1.98E-06
TUCP_003229	XLOC_138410	ENSRNOT00000080695	0.967330617	2.77E-07
TUCP_003229	XLOC_138410	ENSRNOT00000035591	-0.960354064	7.22E-07
TUCP_003229	XLOC_138410	ENSRNOT00000000302	0.961904048	5.93E-07
TUCP_003229	XLOC_138410	ENSRNOT00000078070	0.966156401	3.30E-07
TUCP_003229	XLOC_138410	ENSRNOT00000091205	0.957292637	1.04E-06
TUCP_003229	XLOC_138410	ENSRNOT00000089293	0.952241581	1.81E-06
TUCP_003229	XLOC_138410	ENSRNOT00000078282	-0.951568082	1.93E-06
TUCP_003229	XLOC_138410	ENSRNOT00000029125	0.965597646	3.58E-07
TUCP_003229	XLOC_138410	ENSRNOT00000042125	0.95447732	1.43E-06
TUCP_003229	XLOC_138410	ENSRNOT00000091909	0.957027694	1.07E-06
TUCP_003229	XLOC_138410	ENSRNOT00000020147	0.958829305	8.69E-07
TUCP_003229	XLOC_138410	ENSRNOT00000014814	0.951809172	1.89E-06
TUCP_003229	XLOC_138410	ENSRNOT00000073564	-0.953148003	1.64E-06
TUCP_003229	XLOC_138410	ENSRNOT00000065529	0.958431039	9.12E-07
TUCP_003229	XLOC_138410	ENSRNOT00000090376	0.951085029	2.03E-06

TUCP_003229	XLOC_138410	ENSRNOT00000008982	0.959208443	8.31E-07
TUCP_003229	XLOC_138410	ENSRNOT00000057160	0.963233711	4.97E-07
TUCP_003229	XLOC_138410	ENSRNOT00000004046	-0.972403747	1.20E-07
TUCP_003229	XLOC_138410	ENSRNOT00000016739	0.961399329	6.33E-07
TUCP_003229	XLOC_138410	ENSRNOT00000002837	-0.956445899	1.15E-06
TUCP_003229	XLOC_138410	ENSRNOT00000020910	0.956301774	1.17E-06
TUCP_003229	XLOC_138410	ENSRNOT00000089933	0.959273475	8.24E-07
TUCP_003229	XLOC_138410	ENSRNOT00000064643	0.956443895	1.15E-06
TUCP_003229	XLOC_138410	ENSRNOT00000042799	0.972745443	1.13E-07
TUCP_003229	XLOC_138410	ENSRNOT00000027910	0.95922866	8.28E-07
TUCP_002910	XLOC_125062	ENSRNOT00000007314	0.959208852	8.30E-07
TUCP_002910	XLOC_125062	ENSRNOT00000040060	0.962273628	5.65E-07
TUCP_002910	XLOC_125062	ENSRNOT00000027891	0.951452908	1.96E-06
TUCP_002910	XLOC_125062	ENSRNOT00000001196	0.955637917	1.26E-06
TUCP_002910	XLOC_125062	ENSRNOT00000014780	0.972311473	1.22E-07
TUCP_002910	XLOC_125062	ENSRNOT00000027083	-0.952990099	1.67E-06
TUCP_002910	XLOC_125062	ENSRNOT00000014373	0.968037259	2.49E-07
TUCP_002910	XLOC_125062	ENSRNOT00000026279	0.960512007	7.08E-07
TUCP_002910	XLOC_125062	ENSRNOT00000015568	0.963661892	4.69E-07
TUCP_002910	XLOC_125062	ENSRNOT00000009883	0.966844796	2.98E-07
TUCP_002910	XLOC_125062	ENSRNOT00000049536	0.961186891	6.50E-07
TUCP_002910	XLOC_125062	ENSRNOT00000003085	0.980892335	1.94E-08
TUCP_002910	XLOC_125062	ENSRNOT00000002037	0.974920411	7.49E-08
TUCP_002910	XLOC_125062	ENSRNOT00000060607	0.954901671	1.36E-06
TUCP_002910	XLOC_125062	ENSRNOT00000083173	0.951485533	1.95E-06
TUCP_002910	XLOC_125062	ENSRNOT00000045443	0.962747883	5.31E-07
TUCP_002910	XLOC_125062	ENSRNOT00000079644	0.954077869	1.49E-06
TUCP_002910	XLOC_125062	ENSRNOT00000059524	0.9675726	2.67E-07
TUCP_002910	XLOC_125062	ENSRNOT00000038178	0.954491896	1.42E-06
TUCP_002910	XLOC_125062	ENSRNOT00000003715	0.954234822	1.46E-06
TUCP_002910	XLOC_125062	ENSRNOT00000050721	0.976865725	5.02E-08
TUCP_002910	XLOC_125062	ENSRNOT00000017361	-0.956703432	1.11E-06
TUCP_002910	XLOC_125062	ENSRNOT00000017362	0.961510125	6.24E-07
TUCP_002910	XLOC_125062	ENSRNOT00000064305	0.967529473	2.69E-07
TUCP_002910	XLOC_125062	ENSRNOT00000022117	0.961382	6.34E-07
TUCP_002910	XLOC_125062	ENSRNOT00000002152	0.974322268	8.42E-08
TUCP_002910	XLOC_125062	ENSRNOT00000004941	0.952527237	1.75E-06
TUCP_002910	XLOC_125062	ENSRNOT00000088553	0.956008397	1.21E-06
TUCP_002910	XLOC_125062	ENSRNOT00000045233	0.984334205	7.24E-09
TUCP_002910	XLOC_125062	ENSRNOT00000042633	0.969516326	1.97E-07
TUCP_002910	XLOC_125062	ENSRNOT00000018696	0.973802846	9.30E-08
TUCP_002910	XLOC_125062	ENSRNOT00000009073	0.964780005	4.02E-07
TUCP_002910	XLOC_125062	ENSRNOT00000075656	0.965424842	3.67E-07
TUCP_002910	XLOC_125062	ENSRNOT00000067170	0.956306478	1.17E-06
TUCP_002910	XLOC_125062	ENSRNOT00000066204	0.969567644	1.95E-07
TUCP_002910	XLOC_125062	ENSRNOT00000033015	0.979368173	2.84E-08
TUCP_002910	XLOC_125062	ENSRNOT00000018506	0.955537944	1.27E-06
TUCP_002910	XLOC_125062	ENSRNOT00000001468	0.950968214	2.06E-06
TUCP_002910	XLOC_125062	ENSRNOT00000076105	0.985936627	4.23E-09
TUCP_002910	XLOC_125062	ENSRNOT00000005148	0.960339449	7.23E-07
TUCP_002910	XLOC_125062	ENSRNOT00000032667	0.956101414	1.19E-06

TUCP_002910	XLOC_125062	ENSRNOT00000085311	0.969217562	2.07E-07
TUCP_002910	XLOC_125062	ENSRNOT00000014479	0.952831038	1.70E-06
TUCP_002910	XLOC_125062	ENSRNOT00000087146	-0.956261047	1.17E-06
TUCP_002910	XLOC_125062	ENSRNOT00000010396	-0.958020445	9.57E-07
TUCP_002910	XLOC_125062	ENSRNOT00000003326	0.964681965	4.08E-07
TUCP_002910	XLOC_125062	ENSRNOT00000060300	0.956857615	1.09E-06
TUCP_002910	XLOC_125062	ENSRNOT00000025284	0.955092035	1.33E-06
TUCP_002910	XLOC_125062	ENSRNOT00000073569	0.962482382	5.50E-07
TUCP_002910	XLOC_125062	ENSRNOT00000016541	0.98544542	5.02E-09
TUCP_002910	XLOC_125062	ENSRNOT00000065304	0.966385406	3.19E-07
TUCP_002910	XLOC_125062	ENSRNOT00000027301	0.968351148	2.37E-07
TUCP_002910	XLOC_125062	ENSRNOT00000022620	-0.95435561	1.44E-06
TUCP_002910	XLOC_125062	ENSRNOT00000006765	0.990359788	6.45E-10
TUCP_002910	XLOC_125062	ENSRNOT00000023033	0.955105342	1.33E-06
TUCP_002910	XLOC_125062	ENSRNOT00000000334	0.950530902	2.15E-06
TUCP_002910	XLOC_125062	ENSRNOT00000015247	0.957063892	1.07E-06
TUCP_002910	XLOC_125062	ENSRNOT00000057045	0.984488658	6.89E-09
TUCP_002910	XLOC_125062	ENSRNOT00000088615	0.960255108	7.31E-07
TUCP_002910	XLOC_125062	ENSRNOT00000009000	0.98120545	1.79E-08
TUCP_002910	XLOC_125062	ENSRNOT00000009247	0.968961961	2.15E-07
TUCP_002910	XLOC_125062	ENSRNOT00000012114	0.952372397	1.78E-06
TUCP_002910	XLOC_125062	ENSRNOT00000022241	0.977859061	4.04E-08
TUCP_002910	XLOC_125062	ENSRNOT00000038212	0.960853341	6.78E-07
TUCP_002910	XLOC_125062	ENSRNOT00000039377	0.968100959	2.47E-07
TUCP_002910	XLOC_125062	ENSRNOT00000085403	-0.975020577	7.35E-08
TUCP_002910	XLOC_125062	ENSRNOT00000019848	0.977325874	4.54E-08
TUCP_002910	XLOC_125062	ENSRNOT00000011731	0.98276382	1.16E-08
TUCP_002910	XLOC_125062	ENSRNOT00000002837	0.95297346	1.67E-06
TUCP_002910	XLOC_125062	ENSRNOT00000057898	0.955519438	1.27E-06
TUCP_002910	XLOC_125062	ENSRNOT00000010753	0.971627088	1.38E-07
TUCP_002910	XLOC_125062	ENSRNOT00000023786	0.970921039	1.56E-07
TUCP_002910	XLOC_125062	ENSRNOT00000079962	-0.971879569	1.32E-07
TUCP_002910	XLOC_125062	ENSRNOT00000084674	0.966150828	3.31E-07
TUCP_002910	XLOC_125062	ENSRNOT00000083876	0.959786354	7.74E-07
TUCP_002910	XLOC_125062	ENSRNOT00000090338	0.967189868	2.83E-07
TUCP_002910	XLOC_125062	ENSRNOT00000019192	0.959023864	8.49E-07
TUCP_002910	XLOC_125062	ENSRNOT00000007044	0.964758654	4.03E-07
TUCP_002910	XLOC_125062	ENSRNOT00000065673	0.959168519	8.35E-07
TUCP_002910	XLOC_125062	ENSRNOT00000003412	0.988970091	1.26E-09
TUCP_002910	XLOC_125062	ENSRNOT00000077856	0.972928858	1.09E-07
TUCP_002910	XLOC_125062	ENSRNOT00000012881	0.978064797	3.85E-08
TUCP_002910	XLOC_125062	ENSRNOT00000003794	0.970719945	1.61E-07
TUCP_002910	XLOC_125062	ENSRNOT00000028363	0.955798662	1.23E-06
TUCP_002910	XLOC_125062	ENSRNOT00000071239	0.950429668	2.17E-06
TUCP_002910	XLOC_125062	ENSRNOT00000007771	0.977867609	4.03E-08
TUCP_002910	XLOC_125062	ENSRNOT00000018488	0.951526806	1.94E-06
TUCP_002910	XLOC_125062	ENSRNOT00000029635	0.960516528	7.07E-07
TUCP_002910	XLOC_125062	ENSRNOT00000020702	0.960103692	7.44E-07
TUCP_002910	XLOC_125062	ENSRNOT00000040629	-0.96591475	3.42E-07
TUCP_002910	XLOC_125062	ENSRNOT00000019121	0.951507707	1.95E-06
TUCP_002910	XLOC_125062	ENSRNOT00000010381	-0.959436162	8.08E-07

TUCP_002910	XLOC_125062	ENSRNOT00000041456	0.974246032	8.55E-08
TUCP_002910	XLOC_125062	ENSRNOT00000088133	0.976284385	5.68E-08
TUCP_002910	XLOC_125062	ENSRNOT00000000039	0.960523892	7.07E-07
TUCP_002910	XLOC_125062	ENSRNOT00000083351	0.979505612	2.75E-08
TUCP_002910	XLOC_125062	ENSRNOT00000024568	0.956778391	1.10E-06
TUCP_002910	XLOC_125062	ENSRNOT00000036338	0.984823555	6.18E-09
TUCP_002910	XLOC_125062	ENSRNOT00000082686	0.960912566	6.73E-07
TUCP_002910	XLOC_125062	ENSRNOT00000076181	0.971494075	1.41E-07
TUCP_002910	XLOC_125062	ENSRNOT00000073486	0.954555295	1.41E-06
TUCP_002910	XLOC_125062	ENSRNOT00000080410	0.958731289	8.80E-07
TUCP_002910	XLOC_125062	ENSRNOT00000037039	0.95958337	7.94E-07
TUCP_002910	XLOC_125062	ENSRNOT00000003940	0.964855532	3.98E-07
TUCP_002910	XLOC_125062	ENSRNOT00000015579	0.990338523	6.52E-10
TUCP_002910	XLOC_125062	ENSRNOT00000070794	0.955956203	1.21E-06
TUCP_002910	XLOC_125062	ENSRNOT00000045532	0.972875282	1.10E-07
TUCP_002910	XLOC_125062	ENSRNOT00000020932	0.977635809	4.24E-08
TUCP_002910	XLOC_125062	ENSRNOT00000026928	0.951992245	1.85E-06
TUCP_002910	XLOC_125062	ENSRNOT00000019473	0.963388978	4.87E-07
TUCP_002910	XLOC_125062	ENSRNOT00000073492	0.952729676	1.72E-06
TUCP_002910	XLOC_125062	ENSRNOT00000047634	0.959540899	7.98E-07
TUCP_002910	XLOC_125062	ENSRNOT00000022341	0.965278909	3.75E-07
TUCP_002910	XLOC_125062	ENSRNOT00000083964	0.96323757	4.97E-07
TUCP_002910	XLOC_125062	ENSRNOT00000066520	-0.957984008	9.61E-07
TUCP_002910	XLOC_125062	ENSRNOT00000009900	0.958055566	9.53E-07
TUCP_002910	XLOC_125062	ENSRNOT00000013864	0.957159141	1.06E-06
TUCP_002910	XLOC_125062	ENSRNOT00000022714	-0.976093136	5.91E-08
TUCP_002910	XLOC_125062	ENSRNOT00000026525	0.95813185	9.44E-07
TUCP_002910	XLOC_125062	ENSRNOT00000081327	0.970692416	1.62E-07
TUCP_002910	XLOC_125062	ENSRNOT00000025856	0.966325666	3.22E-07
TUCP_002910	XLOC_125062	ENSRNOT00000061304	0.970559393	1.66E-07
TUCP_002910	XLOC_125062	ENSRNOT00000036838	0.976924355	4.96E-08
TUCP_002910	XLOC_125062	ENSRNOT00000083995	0.963602478	4.73E-07
TUCP_002910	XLOC_125062	ENSRNOT00000064450	0.9567074	1.11E-06
TUCP_002910	XLOC_125062	ENSRNOT00000075992	0.953073396	1.66E-06
TUCP_002910	XLOC_125062	ENSRNOT00000064452	0.972060335	1.28E-07
TUCP_002910	XLOC_125062	ENSRNOT00000017688	0.978141326	3.79E-08
TUCP_002910	XLOC_125062	ENSRNOT00000001366	0.957289384	1.04E-06
TUCP_002910	XLOC_125062	ENSRNOT00000058495	0.955466696	1.28E-06
TUCP_002910	XLOC_125062	ENSRNOT00000016520	-0.95396033	1.51E-06
TUCP_002910	XLOC_125062	ENSRNOT00000019130	0.977175205	4.70E-08
TUCP_002910	XLOC_125062	ENSRNOT00000015757	0.962221874	5.69E-07
TUCP_002910	XLOC_125062	ENSRNOT00000023506	0.979769287	2.58E-08
TUCP_002910	XLOC_125062	ENSRNOT00000040348	0.961116519	6.56E-07
TUCP_002910	XLOC_125062	ENSRNOT00000002151	0.973929098	9.08E-08
TUCP_002910	XLOC_125062	ENSRNOT00000021636	0.977706479	4.18E-08
TUCP_002910	XLOC_125062	ENSRNOT00000009333	0.950346027	2.19E-06
TUCP_002910	XLOC_125062	ENSRNOT00000007889	0.961959521	5.88E-07
TUCP_002910	XLOC_125062	ENSRNOT00000087190	0.953818972	1.53E-06
TUCP_002910	XLOC_125062	ENSRNOT00000017254	0.966234407	3.27E-07
TUCP_002910	XLOC_125062	ENSRNOT00000007738	0.971495864	1.41E-07
TUCP_002910	XLOC_125062	ENSRNOT00000010674	0.965915497	3.42E-07

TUCP_002910	XLOC_125062	ENSRNOT00000020267	-0.969936849	1.84E-07
TUCP_002910	XLOC_125062	ENSRNOT00000023664	0.972293722	1.23E-07
TUCP_002910	XLOC_125062	ENSRNOT00000078746	0.97327804	1.03E-07
TUCP_002910	XLOC_125062	ENSRNOT00000091272	0.952674243	1.73E-06
TUCP_002910	XLOC_125062	ENSRNOT00000056199	0.950631404	2.13E-06
TUCP_002910	XLOC_125062	ENSRNOT00000016730	0.969090791	2.11E-07
TUCP_002910	XLOC_125062	ENSRNOT00000084552	0.968982906	2.15E-07
TUCP_002910	XLOC_125062	ENSRNOT00000004319	0.950591268	2.13E-06
TUCP_002910	XLOC_125062	ENSRNOT00000066247	0.95099129	2.05E-06
TUCP_002910	XLOC_125062	ENSRNOT00000016447	0.964417459	4.23E-07
TUCP_002910	XLOC_125062	ENSRNOT00000001099	0.958057893	9.53E-07
TUCP_002910	XLOC_125062	ENSRNOT00000009666	0.96729621	2.79E-07
TUCP_002910	XLOC_125062	ENSRNOT00000014821	0.950143928	2.23E-06
TUCP_002910	XLOC_125062	ENSRNOT00000013043	0.969449069	1.99E-07
TUCP_002910	XLOC_125062	ENSRNOT00000045765	0.967451685	2.72E-07
TUCP_002910	XLOC_125062	ENSRNOT00000018549	0.955122149	1.33E-06
TUCP_002910	XLOC_125062	ENSRNOT00000032236	0.965841345	3.46E-07
TUCP_002910	XLOC_125062	ENSRNOT00000024992	0.95283263	1.70E-06
TUCP_002910	XLOC_125062	ENSRNOT00000013745	0.96794147	2.53E-07
TUCP_002910	XLOC_125062	ENSRNOT00000000707	0.968922579	2.17E-07
TUCP_002910	XLOC_125062	ENSRNOT00000001607	-0.95010231	2.24E-06
TUCP_002910	XLOC_125062	ENSRNOT00000001935	0.951422664	1.96E-06
TUCP_002910	XLOC_125062	ENSRNOT00000023567	0.96687657	2.97E-07
TUCP_002910	XLOC_125062	ENSRNOT00000089265	0.950373923	2.18E-06
TUCP_002910	XLOC_125062	ENSRNOT00000017692	0.974487924	8.15E-08
TUCP_002910	XLOC_125062	ENSRNOT00000068269	0.974549582	8.06E-08
TUCP_002910	XLOC_125062	ENSRNOT00000018864	0.957273295	1.04E-06
TUCP_002910	XLOC_125062	ENSRNOT00000016351	0.96113217	6.54E-07
TUCP_002910	XLOC_125062	ENSRNOT00000010079	0.975773738	6.31E-08
TUCP_002910	XLOC_125062	ENSRNOT00000050609	0.97122014	1.48E-07
TUCP_002910	XLOC_125062	ENSRNOT00000007949	0.977443533	4.43E-08
TUCP_002910	XLOC_125062	ENSRNOT00000091139	0.960562538	7.03E-07
TUCP_002910	XLOC_125062	ENSRNOT00000021400	0.952527068	1.75E-06
TUCP_002910	XLOC_125062	ENSRNOT00000024286	0.954608821	1.41E-06
TUCP_002910	XLOC_125062	ENSRNOT00000014585	0.952005871	1.85E-06
TUCP_002910	XLOC_125062	ENSRNOT00000064068	0.951866989	1.88E-06
TUCP_002910	XLOC_125062	ENSRNOT00000007079	0.95453072	1.42E-06
TUCP_002910	XLOC_125062	ENSRNOT00000011401	0.959369347	8.14E-07
TUCP_002910	XLOC_125062	ENSRNOT00000044073	0.956815	1.10E-06
TUCP_002910	XLOC_125062	ENSRNOT00000079130	-0.964978042	3.91E-07
TUCP_002910	XLOC_125062	ENSRNOT00000035628	0.960241342	7.32E-07
TUCP_002910	XLOC_125062	ENSRNOT00000082146	0.971767068	1.35E-07
TUCP_002910	XLOC_125062	ENSRNOT00000019680	0.968759692	2.22E-07
TUCP_002910	XLOC_125062	ENSRNOT00000051522	-0.962957857	5.16E-07
TUCP_002910	XLOC_125062	ENSRNOT00000011563	0.954546752	1.42E-06
TUCP_002910	XLOC_125062	ENSRNOT00000054976	0.955438627	1.28E-06
TUCP_002910	XLOC_125062	ENSRNOT00000086350	0.992436181	1.93E-10
TUCP_002910	XLOC_125062	ENSRNOT00000010516	0.984958616	5.91E-09
TUCP_002910	XLOC_125062	ENSRNOT00000004842	0.953186944	1.64E-06
TUCP_002910	XLOC_125062	ENSRNOT00000019713	0.965791679	3.48E-07
TUCP_002910	XLOC_125062	ENSRNOT00000037363	0.968064547	2.48E-07

TUCP_002910	XLOC_125062	ENSRNOT00000071482	0.955161294	1.32E-06
TUCP_002910	XLOC_125062	ENSRNOT00000066888	0.951099244	2.03E-06
TUCP_002910	XLOC_125062	ENSRNOT00000025794	0.957866731	9.74E-07
TUCP_002910	XLOC_125062	ENSRNOT00000015131	0.958642701	8.89E-07
TUCP_002910	XLOC_125062	ENSRNOT00000000529	0.962567277	5.44E-07
TUCP_002910	XLOC_125062	ENSRNOT00000072031	0.978397362	3.57E-08
TUCP_002910	XLOC_125062	ENSRNOT00000009840	0.964562367	4.15E-07
TUCP_002910	XLOC_125062	ENSRNOT00000085214	0.977564223	4.31E-08
TUCP_002910	XLOC_125062	ENSRNOT00000017181	0.955867855	1.22E-06
TUCP_002910	XLOC_125062	ENSRNOT00000030329	0.975487811	6.69E-08
TUCP_002910	XLOC_125062	ENSRNOT00000017469	0.965445165	3.66E-07
TUCP_002910	XLOC_125062	ENSRNOT00000087748	0.968669786	2.26E-07
TUCP_002910	XLOC_125062	ENSRNOT00000006246	0.959429313	8.09E-07
TUCP_002910	XLOC_125062	ENSRNOT00000088202	0.958468664	9.07E-07
TUCP_002910	XLOC_125062	ENSRNOT00000088201	0.960674642	6.93E-07
TUCP_002910	XLOC_125062	ENSRNOT00000033450	0.978057219	3.86E-08
TUCP_002910	XLOC_125062	ENSRNOT00000049725	0.958962736	8.55E-07
TUCP_002910	XLOC_125062	ENSRNOT00000033196	0.987373195	2.47E-09
TUCP_002910	XLOC_125062	ENSRNOT00000027125	0.968688049	2.25E-07
TUCP_002910	XLOC_125062	ENSRNOT00000065288	0.95479162	1.38E-06
TUCP_002910	XLOC_125062	ENSRNOT00000026235	0.955288869	1.31E-06
TUCP_002910	XLOC_125062	ENSRNOT00000022478	0.968611738	2.28E-07
TUCP_002910	XLOC_125062	ENSRNOT00000023078	-0.96707844	2.88E-07
TUCP_002910	XLOC_125062	ENSRNOT00000082151	0.955484295	1.28E-06
TUCP_002910	XLOC_125062	ENSRNOT00000026398	0.966286842	3.24E-07
TUCP_002910	XLOC_125062	ENSRNOT00000013463	-0.956385124	1.16E-06
TUCP_002910	XLOC_125062	ENSRNOT00000089624	0.956245074	1.17E-06
TUCP_002910	XLOC_125062	ENSRNOT00000089673	0.964763495	4.03E-07
TUCP_002910	XLOC_125062	ENSRNOT00000005644	0.982794146	1.15E-08
TUCP_002910	XLOC_125062	ENSRNOT00000038448	0.974899267	7.52E-08
TUCP_002910	XLOC_125062	ENSRNOT00000050223	0.969908844	1.85E-07
TUCP_002910	XLOC_125062	ENSRNOT00000000263	-0.964728209	4.05E-07
TUCP_002910	XLOC_125062	ENSRNOT00000089185	0.95406495	1.49E-06
TUCP_002910	XLOC_125062	ENSRNOT00000023977	0.960491151	7.09E-07
TUCP_002910	XLOC_125062	ENSRNOT00000032289	0.96248238	5.50E-07
TUCP_002910	XLOC_125062	ENSRNOT00000014134	0.979898884	2.50E-08
TUCP_002910	XLOC_125062	ENSRNOT00000008865	0.983965104	8.13E-09
TUCP_002910	XLOC_125062	ENSRNOT00000017420	0.976051331	5.96E-08
TUCP_002910	XLOC_125062	ENSRNOT00000067741	0.95035006	2.19E-06
TUCP_002910	XLOC_125062	ENSRNOT00000092021	0.961891169	5.94E-07
TUCP_002910	XLOC_125062	ENSRNOT00000061191	0.964600428	4.13E-07
TUCP_002910	XLOC_125062	ENSRNOT00000017907	0.975743572	6.35E-08
TUCP_002910	XLOC_125062	ENSRNOT00000007077	0.977165836	4.71E-08
TUCP_002910	XLOC_125062	ENSRNOT00000001586	0.968634567	2.27E-07
TUCP_002910	XLOC_125062	ENSRNOT00000003691	0.962874515	5.22E-07
TUCP_002910	XLOC_125062	ENSRNOT00000007954	-0.951170565	2.01E-06
TUCP_002910	XLOC_125062	ENSRNOT00000028434	0.962599247	5.41E-07
TUCP_002910	XLOC_125062	ENSRNOT00000014559	0.95713511	1.06E-06
TUCP_002910	XLOC_125062	ENSRNOT00000000532	0.953341946	1.61E-06
TUCP_002910	XLOC_125062	ENSRNOT00000014809	0.97324564	1.03E-07
TUCP_002910	XLOC_125062	ENSRNOT00000064849	0.960593615	7.00E-07

TUCP_002910	XLOC_125062	ENSRNOT00000019644	0.961457725	6.28E-07
TUCP_002910	XLOC_125062	ENSRNOT00000046126	0.953559847	1.57E-06
TUCP_002910	XLOC_125062	ENSRNOT00000055957	0.960027912	7.51E-07
TUCP_002910	XLOC_125062	ENSRNOT00000030692	0.951272971	1.99E-06
TUCP_002910	XLOC_125062	ENSRNOT00000021471	0.960498705	7.09E-07
TUCP_002910	XLOC_125062	ENSRNOT00000014885	0.974499151	8.14E-08
TUCP_002910	XLOC_125062	ENSRNOT00000067566	0.971378728	1.44E-07
TUCP_002910	XLOC_125062	ENSRNOT00000006376	0.95054575	2.14E-06
TUCP_002910	XLOC_125062	ENSRNOT00000041091	0.975182466	7.11E-08
TUCP_002910	XLOC_125062	ENSRNOT00000002114	0.953357502	1.61E-06
TUCP_002910	XLOC_125062	ENSRNOT00000023017	0.975800622	6.28E-08
TUCP_002910	XLOC_125062	ENSRNOT00000033048	0.983133539	1.05E-08
TUCP_002910	XLOC_125062	ENSRNOT00000077479	0.95964517	7.88E-07
TUCP_002910	XLOC_125062	ENSRNOT00000074261	0.95711119	1.06E-06
TUCP_002910	XLOC_125062	ENSRNOT00000022194	0.972310485	1.22E-07
TUCP_002910	XLOC_125062	ENSRNOT00000050834	0.957832931	9.78E-07
TUCP_002910	XLOC_125062	ENSRNOT00000040548	0.957467605	1.02E-06
TUCP_002910	XLOC_125062	ENSRNOT00000008222	0.969295832	2.04E-07
TUCP_002910	XLOC_125062	ENSRNOT00000010962	0.964357195	4.27E-07
TUCP_002910	XLOC_125062	ENSRNOT00000081835	0.954766977	1.38E-06
TUCP_002910	XLOC_125062	ENSRNOT00000046215	0.962760395	5.30E-07
TUCP_002910	XLOC_125062	ENSRNOT00000010620	0.965789551	3.48E-07
TUCP_002910	XLOC_125062	ENSRNOT00000000083	0.973857375	9.20E-08
TUCP_002910	XLOC_125062	ENSRNOT00000061973	0.955136738	1.33E-06
TUCP_002910	XLOC_125062	ENSRNOT00000002445	0.965594857	3.58E-07
TUCP_002910	XLOC_125062	ENSRNOT00000001135	0.961540923	6.21E-07
TUCP_002910	XLOC_125062	ENSRNOT00000001234	0.953845398	1.53E-06
TUCP_002910	XLOC_125062	ENSRNOT00000068078	0.964620499	4.11E-07
TUCP_002910	XLOC_125062	ENSRNOT00000009993	0.968485591	2.32E-07
TUCP_002910	XLOC_125062	ENSRNOT00000056877	0.958307228	9.25E-07
TUCP_002910	XLOC_125062	ENSRNOT00000001036	0.951342147	1.98E-06
TUCP_002910	XLOC_125062	ENSRNOT00000058763	0.96387998	4.56E-07
TUCP_002910	XLOC_125062	ENSRNOT00000021152	-0.95723218	1.05E-06
TUCP_002910	XLOC_125062	ENSRNOT00000092037	0.969422172	2.00E-07
TUCP_002910	XLOC_125062	ENSRNOT00000012583	0.961854838	5.97E-07
TUCP_002910	XLOC_125062	ENSRNOT00000081621	-0.963309781	4.92E-07
TUCP_002910	XLOC_125062	ENSRNOT00000012625	0.962853491	5.23E-07
TUCP_002910	XLOC_125062	ENSRNOT00000080677	0.961175464	6.51E-07
TUCP_002910	XLOC_125062	ENSRNOT00000028545	0.958556216	8.98E-07
TUCP_002910	XLOC_125062	ENSRNOT00000007367	0.953605192	1.57E-06
TUCP_002910	XLOC_125062	ENSRNOT00000001759	0.978869667	3.20E-08
TUCP_002910	XLOC_125062	ENSRNOT00000080797	0.950842258	2.08E-06
TUCP_002910	XLOC_125062	ENSRNOT00000039191	0.953522757	1.58E-06
TUCP_002910	XLOC_125062	ENSRNOT00000078532	0.967292863	2.79E-07
TUCP_002910	XLOC_125062	ENSRNOT00000022000	0.970487741	1.68E-07
TUCP_002910	XLOC_125062	ENSRNOT00000028130	0.972267113	1.23E-07
TUCP_002910	XLOC_125062	ENSRNOT00000068267	0.971314294	1.46E-07
TUCP_002910	XLOC_125062	ENSRNOT00000020994	0.966671146	3.06E-07
TUCP_002910	XLOC_125062	ENSRNOT00000018328	0.950341866	2.19E-06
TUCP_002910	XLOC_125062	ENSRNOT00000063821	0.966369176	3.20E-07
TUCP_002910	XLOC_125062	ENSRNOT00000070912	0.985949041	4.21E-09

TUCP_002910	XLOC_125062	ENSRNOT00000019916	0.971918018	1.31E-07
TUCP_002910	XLOC_125062	ENSRNOT00000019911	0.955346428	1.30E-06
TUCP_002910	XLOC_125062	ENSRNOT00000015763	0.955674669	1.25E-06
TUCP_002910	XLOC_125062	ENSRNOT00000025819	0.95053205	2.15E-06
TUCP_002910	XLOC_125062	ENSRNOT00000028703	0.969335969	2.03E-07
TUCP_002910	XLOC_125062	ENSRNOT00000011907	0.967095146	2.87E-07
TUCP_002910	XLOC_125062	ENSRNOT00000049193	0.977580285	4.30E-08
TUCP_002910	XLOC_125062	ENSRNOT00000077401	0.973721394	9.44E-08
TUCP_002910	XLOC_125062	ENSRNOT00000002355	0.986856259	3.02E-09
TUCP_002910	XLOC_125062	ENSRNOT00000011326	0.968109566	2.46E-07
TUCP_002910	XLOC_125062	ENSRNOT00000020272	0.981514331	1.65E-08
TUCP_002910	XLOC_125062	ENSRNOT00000007169	0.964449479	4.21E-07
TUCP_002910	XLOC_125062	ENSRNOT00000064700	-0.950524437	2.15E-06
TUCP_002910	XLOC_125062	ENSRNOT00000088677	0.950025019	2.26E-06
TUCP_002910	XLOC_125062	ENSRNOT00000008987	0.956636299	1.12E-06
TUCP_002910	XLOC_125062	ENSRNOT00000022377	0.956759422	1.11E-06
TUCP_002910	XLOC_125062	ENSRNOT00000090355	0.961808139	6.00E-07
TUCP_002910	XLOC_125062	ENSRNOT00000012361	0.974168223	8.67E-08
TUCP_002910	XLOC_125062	ENSRNOT00000016740	-0.952656324	1.73E-06
TUCP_002910	XLOC_125062	ENSRNOT00000019386	0.965392022	3.69E-07
TUCP_002910	XLOC_125062	ENSRNOT00000074494	0.979958771	2.46E-08
TUCP_002910	XLOC_125062	ENSRNOT00000039296	-0.969404977	2.01E-07
TUCP_002910	XLOC_125062	ENSRNOT00000005885	0.967117017	2.87E-07
TUCP_002910	XLOC_125062	ENSRNOT00000059599	0.980534568	2.13E-08
TUCP_002910	XLOC_125062	ENSRNOT00000010316	0.965726398	3.52E-07
TUCP_002910	XLOC_125062	ENSRNOT00000036194	0.9504134	2.17E-06
TUCP_002910	XLOC_125062	ENSRNOT00000026057	0.978528786	3.47E-08
TUCP_002908	XLOC_125033	ENSRNOT00000089934	0.95465014	1.40E-06
TUCP_003409	XLOC_147609	ENSRNOT00000008655	0.951494152	1.95E-06
TUCP_003409	XLOC_147609	ENSRNOT00000026915	0.959713413	7.81E-07
TUCP_003409	XLOC_147609	ENSRNOT00000015116	0.959336993	8.18E-07
TUCP_003409	XLOC_147609	ENSRNOT00000027454	0.958122358	9.45E-07
TUCP_003409	XLOC_147609	ENSRNOT00000028117	-0.967695084	2.62E-07
TUCP_003409	XLOC_147609	ENSRNOT00000000337	0.957551483	1.01E-06
TUCP_002904	XLOC_124874	ENSRNOT00000009825	0.954081216	1.49E-06
TUCP_002904	XLOC_124874	ENSRNOT00000022559	0.956881587	1.09E-06
TUCP_002902	XLOC_124838	ENSRNOT00000081850	0.969028272	2.13E-07
TUCP_002902	XLOC_124838	ENSRNOT00000032157	0.963257144	4.96E-07
TUCP_002902	XLOC_124838	ENSRNOT00000037134	0.960957561	6.69E-07
TUCP_002902	XLOC_124838	ENSRNOT00000002257	0.961742805	6.05E-07
TUCP_002902	XLOC_124838	ENSRNOT00000001825	0.970672024	1.63E-07
TUCP_002902	XLOC_124838	ENSRNOT00000082585	0.952203513	1.81E-06
TUCP_002902	XLOC_124838	ENSRNOT00000026854	0.955501643	1.27E-06
TUCP_002901	XLOC_124732	ENSRNOT00000086502	0.952935763	1.68E-06
TUCP_002901	XLOC_124732	ENSRNOT00000042165	0.988661808	1.45E-09
TUCP_002901	XLOC_124732	ENSRNOT00000008278	0.994530265	3.82E-11
TUCP_002901	XLOC_124732	ENSRNOT00000072599	0.964135413	4.40E-07
TUCP_002860	XLOC_121757	ENSRNOT00000066419	0.960730561	6.88E-07
TUCP_002860	XLOC_121757	ENSRNOT00000016088	0.95558519	1.26E-06
TUCP_002860	XLOC_121757	ENSRNOT00000009975	0.953945957	1.51E-06
TUCP_002860	XLOC_121757	ENSRNOT00000083623	0.962288779	5.64E-07

TUCP_002860	XLOC_121757	ENSRNOT00000081226	0.957267313	1.04E-06
TUCP_002860	XLOC_121757	ENSRNOT00000001642	0.951172717	2.01E-06
TUCP_002860	XLOC_121757	ENSRNOT00000027544	0.952339628	1.79E-06
TUCP_002860	XLOC_121757	ENSRNOT00000065897	0.962928596	5.18E-07
TUCP_002860	XLOC_121757	ENSRNOT00000083594	0.958689994	8.84E-07
TUCP_002860	XLOC_121757	ENSRNOT00000073493	0.954513336	1.42E-06
TUCP_002860	XLOC_121757	ENSRNOT00000056050	0.9543206	1.45E-06
TUCP_002860	XLOC_121757	ENSRNOT00000016485	0.970123424	1.78E-07
TUCP_002860	XLOC_121757	ENSRNOT00000078752	-0.952206026	1.81E-06
TUCP_002860	XLOC_121757	ENSRNOT00000002188	0.951206685	2.01E-06
TUCP_002860	XLOC_121757	ENSRNOT00000005770	0.968501325	2.32E-07
TUCP_002860	XLOC_121757	ENSRNOT00000028687	-0.963047897	5.10E-07
TUCP_002860	XLOC_121757	ENSRNOT00000035808	0.950225632	2.21E-06
TUCP_002860	XLOC_121757	ENSRNOT00000085334	0.961053859	6.61E-07
TUCP_002860	XLOC_121757	ENSRNOT00000057953	0.950319295	2.19E-06
TUCP_002860	XLOC_121757	ENSRNOT00000008764	0.955506462	1.27E-06
TUCP_002860	XLOC_121757	ENSRNOT00000012757	0.964480231	4.19E-07
TUCP_002860	XLOC_121757	ENSRNOT00000037902	0.951360132	1.98E-06
TUCP_002860	XLOC_121757	ENSRNOT00000012247	0.961116566	6.56E-07
TUCP_002860	XLOC_121757	ENSRNOT00000038449	0.953451503	1.59E-06
TUCP_002864	XLOC_122356	ENSRNOT00000024878	0.958024215	9.56E-07
TUCP_002864	XLOC_122356	ENSRNOT00000034741	0.953111679	1.65E-06
TUCP_002866	XLOC_122447	ENSRNOT00000093261	0.961109498	6.56E-07
TUCP_002866	XLOC_122447	ENSRNOT00000014155	0.959683886	7.84E-07
TUCP_002866	XLOC_122447	ENSRNOT00000026809	0.964947731	3.93E-07
TUCP_002866	XLOC_122447	ENSRNOT00000059167	0.955194393	1.32E-06
TUCP_002868	XLOC_122520	ENSRNOT00000026706	-0.960839526	6.79E-07
TUCP_000170	XLOC_005377	ENSRNOT00000021408	0.958464945	9.08E-07
TUCP_000170	XLOC_005377	ENSRNOT00000026457	0.957580847	1.01E-06
TUCP_000170	XLOC_005377	ENSRNOT00000004524	0.97553444	6.63E-08
TUCP_000170	XLOC_005377	ENSRNOT00000032780	0.950464782	2.16E-06
TUCP_000170	XLOC_005377	ENSRNOT00000064840	0.955202067	1.32E-06
TUCP_000170	XLOC_005377	ENSRNOT00000087592	0.976645562	5.26E-08
TUCP_000170	XLOC_005377	ENSRNOT00000026186	0.954882948	1.36E-06
TUCP_000170	XLOC_005377	ENSRNOT00000023180	0.95694706	1.08E-06
TUCP_000170	XLOC_005377	ENSRNOT00000071698	0.951514655	1.94E-06
TUCP_000170	XLOC_005377	ENSRNOT00000011350	0.955089774	1.33E-06
TUCP_000170	XLOC_005377	ENSRNOT00000010218	0.963833854	4.59E-07
TUCP_000170	XLOC_005377	ENSRNOT00000072518	0.956706186	1.11E-06
TUCP_000170	XLOC_005377	ENSRNOT00000076872	0.950330127	2.19E-06
TUCP_000170	XLOC_005377	ENSRNOT00000022485	0.950768862	2.10E-06
TUCP_000170	XLOC_005377	ENSRNOT00000028628	-0.950433126	2.17E-06
TUCP_000170	XLOC_005377	ENSRNOT00000046229	0.964629342	4.11E-07
TUCP_000170	XLOC_005377	ENSRNOT00000078445	-0.956724891	1.11E-06
TUCP_000170	XLOC_005377	ENSRNOT00000016333	-0.958657794	8.87E-07
TUCP_000170	XLOC_005377	ENSRNOT00000008817	0.965724523	3.52E-07
TUCP_000170	XLOC_005377	ENSRNOT00000015116	0.962924545	5.18E-07
TUCP_000170	XLOC_005377	ENSRNOT00000016401	0.958364431	9.19E-07
TUCP_000078	XLOC_001928	ENSRNOT00000079256	0.962026712	5.83E-07
TUCP_000078	XLOC_001928	ENSRNOT00000057685	0.954757585	1.38E-06
TUCP_003210	XLOC_138405	ENSRNOT00000078220	0.955889864	1.22E-06

TUCP_003210	XLOC_138405	ENSRNOT00000063875	0.952872285	1.69E-06
TUCP_001800	XLOC_074612	ENSRNOT00000085599	0.951402655	1.97E-06
TUCP_001800	XLOC_074612	ENSRNOT00000081534	0.965573442	3.59E-07
TUCP_001800	XLOC_074612	ENSRNOT00000038729	0.951950972	1.86E-06
TUCP_001800	XLOC_074612	ENSRNOT00000086385	0.951950972	1.86E-06
TUCP_001800	XLOC_074612	ENSRNOT00000032339	0.987076505	2.78E-09
TUCP_001800	XLOC_074612	ENSRNOT00000087613	0.951950972	1.86E-06
TUCP_001800	XLOC_074612	ENSRNOT00000023062	0.951950972	1.86E-06
TUCP_001800	XLOC_074612	ENSRNOT00000058834	0.951950972	1.86E-06
TUCP_001800	XLOC_074612	ENSRNOT00000079420	0.951950972	1.86E-06
TUCP_001800	XLOC_074612	ENSRNOT00000090761	0.951950972	1.86E-06
TUCP_001800	XLOC_074612	ENSRNOT00000055923	0.951950972	1.86E-06
TUCP_001800	XLOC_074612	ENSRNOT00000071930	0.951950972	1.86E-06
TUCP_001800	XLOC_074612	ENSRNOT00000076756	0.951950972	1.86E-06
TUCP_001800	XLOC_074612	ENSRNOT00000081527	0.950004824	2.26E-06
TUCP_001800	XLOC_074612	ENSRNOT00000091663	0.960944595	6.70E-07
TUCP_001800	XLOC_074612	ENSRNOT00000081780	0.951950972	1.86E-06
TUCP_001800	XLOC_074612	ENSRNOT00000089114	0.951950972	1.86E-06
TUCP_001800	XLOC_074612	ENSRNOT00000058497	0.971183644	1.49E-07
TUCP_001800	XLOC_074612	ENSRNOT00000073316	0.951950972	1.86E-06
TUCP_001800	XLOC_074612	ENSRNOT00000071663	0.951950972	1.86E-06
TUCP_001800	XLOC_074612	ENSRNOT00000042860	0.951950972	1.86E-06
TUCP_001800	XLOC_074612	ENSRNOT00000088421	0.951950972	1.86E-06
TUCP_001800	XLOC_074612	ENSRNOT00000038898	0.951950972	1.86E-06
TUCP_001800	XLOC_074612	ENSRNOT00000042777	0.952115062	1.83E-06
TUCP_001800	XLOC_074612	ENSRNOT00000010029	0.951950972	1.86E-06
TUCP_003967	XLOC_174241	ENSRNOT00000087656	0.953963467	1.51E-06
TUCP_000173	XLOC_005567	ENSRNOT00000006517	-0.95270486	1.72E-06
TUCP_000173	XLOC_005567	ENSRNOT00000009532	0.952965058	1.67E-06
TUCP_000173	XLOC_005567	ENSRNOT00000024413	0.95066975	2.12E-06
TUCP_000173	XLOC_005567	ENSRNOT00000075159	0.973807663	9.29E-08
TUCP_000173	XLOC_005567	ENSRNOT00000002394	0.956140038	1.19E-06
TUCP_000173	XLOC_005567	ENSRNOT00000005894	0.957166054	1.06E-06
TUCP_000173	XLOC_005567	ENSRNOT00000025906	0.951380225	1.97E-06
TUCP_004244	XLOC_186617	ENSRNOT00000012512	0.952837175	1.70E-06
TUCP_004244	XLOC_186617	ENSRNOT00000001196	0.96100667	6.65E-07
TUCP_004244	XLOC_186617	ENSRNOT00000088799	0.952323257	1.79E-06
TUCP_004244	XLOC_186617	ENSRNOT00000018931	-0.953081481	1.65E-06
TUCP_004244	XLOC_186617	ENSRNOT00000000990	0.969504045	1.97E-07
TUCP_004244	XLOC_186617	ENSRNOT00000026273	-0.957604389	1.00E-06
TUCP_004244	XLOC_186617	ENSRNOT00000022633	0.961647823	6.13E-07
TUCP_004244	XLOC_186617	ENSRNOT00000060607	0.954025397	1.50E-06
TUCP_004244	XLOC_186617	ENSRNOT00000006172	0.953149575	1.64E-06
TUCP_004244	XLOC_186617	ENSRNOT00000029620	0.968367627	2.37E-07
TUCP_004244	XLOC_186617	ENSRNOT00000019465	0.95281586	1.70E-06
TUCP_004244	XLOC_186617	ENSRNOT00000068683	0.970012208	1.82E-07
TUCP_004244	XLOC_186617	ENSRNOT00000047634	0.953504147	1.58E-06
TUCP_004244	XLOC_186617	ENSRNOT00000013043	0.959901576	7.63E-07
TUCP_004244	XLOC_186617	ENSRNOT00000034603	0.975747929	6.34E-08
TUCP_004244	XLOC_186617	ENSRNOT00000015986	0.976682759	5.22E-08
TUCP_004244	XLOC_186617	ENSRNOT00000015320	0.969300664	2.04E-07

TUCP_004244	XLOC_186617	ENSRNOT00000073569	0.954203656	1.47E-06
TUCP_004244	XLOC_186617	ENSRNOT00000012699	0.954339289	1.45E-06
TUCP_004244	XLOC_186617	ENSRNOT00000017375	0.976773145	5.12E-08
TUCP_004244	XLOC_186617	ENSRNOT00000078046	0.95651022	1.14E-06
TUCP_004244	XLOC_186617	ENSRNOT00000000911	0.959729972	7.79E-07
TUCP_004244	XLOC_186617	ENSRNOT00000000211	0.960075195	7.47E-07
TUCP_004244	XLOC_186617	ENSRNOT00000084674	0.95860785	8.93E-07
TUCP_004244	XLOC_186617	ENSRNOT00000014684	0.961245734	6.45E-07
TUCP_004244	XLOC_186617	ENSRNOT00000045855	0.959008328	8.51E-07
TUCP_004244	XLOC_186617	ENSRNOT00000001577	-0.95800869	9.58E-07
TUCP_004244	XLOC_186617	ENSRNOT00000015618	0.968896687	2.18E-07
TUCP_004244	XLOC_186617	ENSRNOT00000019121	0.953343366	1.61E-06
TUCP_004244	XLOC_186617	ENSRNOT00000000049	0.952822294	1.70E-06
TUCP_004244	XLOC_186617	ENSRNOT00000046888	0.965982119	3.39E-07
TUCP_004244	XLOC_186617	ENSRNOT00000014163	0.97376214	9.37E-08
TUCP_004244	XLOC_186617	ENSRNOT00000026928	0.957279256	1.04E-06
TUCP_004244	XLOC_186617	ENSRNOT00000055062	0.96043368	7.15E-07
TUCP_004244	XLOC_186617	ENSRNOT00000010252	0.976311589	5.65E-08
TUCP_004244	XLOC_186617	ENSRNOT00000030975	0.971734215	1.36E-07
TUCP_004244	XLOC_186617	ENSRNOT00000012646	0.960196788	7.36E-07
TUCP_004244	XLOC_186617	ENSRNOT00000005694	0.97398537	8.98E-08
TUCP_004244	XLOC_186617	ENSRNOT00000016447	0.956148385	1.19E-06
TUCP_004244	XLOC_186617	ENSRNOT00000033984	0.950530564	2.15E-06
TUCP_004244	XLOC_186617	ENSRNOT00000035048	0.956185898	1.18E-06
TUCP_004244	XLOC_186617	ENSRNOT00000066888	0.968072317	2.48E-07
TUCP_004244	XLOC_186617	ENSRNOT00000061973	0.961492954	6.25E-07
TUCP_004244	XLOC_186617	ENSRNOT00000011714	0.969676776	1.92E-07
TUCP_004244	XLOC_186617	ENSRNOT00000011371	0.975042238	7.31E-08
TUCP_004244	XLOC_186617	ENSRNOT00000012657	0.980635474	2.08E-08
TUCP_004244	XLOC_186617	ENSRNOT00000065103	0.960268749	7.29E-07
TUCP_004244	XLOC_186617	ENSRNOT00000024992	0.951051546	2.04E-06
TUCP_004244	XLOC_186617	ENSRNOT00000078644	-0.954766753	1.38E-06
TUCP_004244	XLOC_186617	ENSRNOT00000023100	0.964098174	4.42E-07
TUCP_004244	XLOC_186617	ENSRNOT00000021400	0.959186499	8.33E-07
TUCP_004244	XLOC_186617	ENSRNOT00000073847	0.952214167	1.81E-06
TUCP_004244	XLOC_186617	ENSRNOT00000044975	0.962195898	5.71E-07
TUCP_004244	XLOC_186617	ENSRNOT00000018782	0.952028872	1.85E-06
TUCP_004244	XLOC_186617	ENSRNOT00000000256	0.950820819	2.09E-06
TUCP_004244	XLOC_186617	ENSRNOT00000000529	0.964196987	4.36E-07
TUCP_004244	XLOC_186617	ENSRNOT00000045311	0.972847223	1.11E-07
TUCP_004244	XLOC_186617	ENSRNOT00000091863	0.979311632	2.88E-08
TUCP_004244	XLOC_186617	ENSRNOT00000039335	0.973308421	1.02E-07
TUCP_004244	XLOC_186617	ENSRNOT00000018061	0.950263941	2.20E-06
TUCP_004244	XLOC_186617	ENSRNOT00000036972	0.974472793	8.18E-08
TUCP_004244	XLOC_186617	ENSRNOT00000014809	0.952344669	1.79E-06
TUCP_004244	XLOC_186617	ENSRNOT00000014481	0.960496936	7.09E-07
TUCP_004244	XLOC_186617	ENSRNOT00000046126	0.970448975	1.69E-07
TUCP_004244	XLOC_186617	ENSRNOT00000006376	0.962628769	5.39E-07
TUCP_004244	XLOC_186617	ENSRNOT00000041091	0.95210817	1.83E-06
TUCP_004244	XLOC_186617	ENSRNOT00000061297	0.956881088	1.09E-06
TUCP_004244	XLOC_186617	ENSRNOT00000027753	0.970589496	1.65E-07

TUCP_004244	XLOC_186617	ENSRNOT00000073239	0.965164008	3.81E-07
TUCP_004244	XLOC_186617	ENSRNOT00000024466	-0.955099568	1.33E-06
TUCP_004244	XLOC_186617	ENSRNOT00000076467	0.963391915	4.87E-07
TUCP_004244	XLOC_186617	ENSRNOT00000019768	-0.958864873	8.66E-07
TUCP_004244	XLOC_186617	ENSRNOT00000001036	0.965774483	3.49E-07
TUCP_004244	XLOC_186617	ENSRNOT00000037181	0.96086607	6.77E-07
TUCP_004244	XLOC_186617	ENSRNOT00000074629	0.959597145	7.92E-07
TUCP_004244	XLOC_186617	ENSRNOT00000060757	0.979931277	2.48E-08
TUCP_004244	XLOC_186617	ENSRNOT00000012996	-0.958061402	9.52E-07
TUCP_004244	XLOC_186617	ENSRNOT00000003343	0.973100555	1.06E-07
TUCP_004244	XLOC_186617	ENSRNOT00000060595	0.964123461	4.41E-07
TUCP_004244	XLOC_186617	ENSRNOT00000071612	0.951406076	1.97E-06
TUCP_004244	XLOC_186617	ENSRNOT00000023252	0.971328302	1.45E-07
TUCP_004244	XLOC_186617	ENSRNOT00000073388	0.952312911	1.79E-06
TUCP_004244	XLOC_186617	ENSRNOT00000002527	-0.962994512	5.14E-07
TUCP_004244	XLOC_186617	ENSRNOT00000009260	0.954899944	1.36E-06
TUCP_004244	XLOC_186617	ENSRNOT00000043736	0.950616809	2.13E-06
TUCP_004244	XLOC_186617	ENSRNOT00000083814	0.972893074	1.10E-07
TUCP_004244	XLOC_186617	ENSRNOT00000011326	0.95167059	1.91E-06
TUCP_004244	XLOC_186617	ENSRNOT00000040637	-0.961298152	6.41E-07
TUCP_004518	XLOC_199300	ENSRNOT00000000593	0.967423795	2.74E-07
TUCP_003965	XLOC_174241	ENSRNOT00000080159	0.956766681	1.11E-06
TUCP_004694	XLOC_206402	ENSRNOT00000019195	-0.954723205	1.39E-06
TUCP_004694	XLOC_206402	ENSRNOT00000021752	0.966260907	3.25E-07
TUCP_004694	XLOC_206402	ENSRNOT00000089336	0.954487752	1.42E-06
TUCP_004512	XLOC_198926	ENSRNOT00000055073	0.987456529	2.39E-09
TUCP_004512	XLOC_198926	ENSRNOT00000019171	0.980115606	2.37E-08
TUCP_004512	XLOC_198926	ENSRNOT00000071484	0.973216696	1.04E-07
TUCP_004512	XLOC_198926	ENSRNOT00000010948	0.983153135	1.04E-08
TUCP_004513	XLOC_198926	ENSRNOT00000000215	0.954371961	1.44E-06
TUCP_004513	XLOC_198926	ENSRNOT00000093230	0.950795715	2.09E-06
TUCP_004513	XLOC_198926	ENSRNOT00000072306	0.958238029	9.33E-07
TUCP_004513	XLOC_198926	ENSRNOT00000087907	0.95653181	1.14E-06
TUCP_004513	XLOC_198926	ENSRNOT00000031076	0.955232867	1.31E-06
TUCP_003230	XLOC_138412	ENSRNOT00000031904	0.966771964	3.02E-07
TUCP_003230	XLOC_138412	ENSRNOT00000082999	-0.951464728	1.95E-06
TUCP_003230	XLOC_138412	ENSRNOT00000000743	0.964060254	4.45E-07
TUCP_003230	XLOC_138412	ENSRNOT00000027083	0.957628872	1.00E-06
TUCP_003230	XLOC_138412	ENSRNOT00000037597	0.956102137	1.19E-06
TUCP_003230	XLOC_138412	ENSRNOT00000005958	0.95545204	1.28E-06
TUCP_003230	XLOC_138412	ENSRNOT00000068353	0.954501409	1.42E-06
TUCP_003230	XLOC_138412	ENSRNOT00000023638	0.965138878	3.82E-07
TUCP_003230	XLOC_138412	ENSRNOT00000020910	0.958464082	9.08E-07
TUCP_003230	XLOC_138412	ENSRNOT00000005220	0.953473613	1.59E-06
TUCP_003230	XLOC_138412	ENSRNOT00000023144	0.980469775	2.17E-08
TUCP_003230	XLOC_138412	ENSRNOT00000071166	0.95786983	9.74E-07
TUCP_003230	XLOC_138412	ENSRNOT00000016649	0.967905852	2.54E-07
TUCP_003230	XLOC_138412	ENSRNOT00000074023	0.955216393	1.32E-06
TUCP_003230	XLOC_138412	ENSRNOT00000014384	-0.953156626	1.64E-06
TUCP_003230	XLOC_138412	ENSRNOT00000091596	-0.950932989	2.06E-06
TUCP_003230	XLOC_138412	ENSRNOT00000091128	-0.959197721	8.32E-07

TUCP_003230	XLOC_138412	ENSRNOT00000081981	0.962991479	5.14E-07
TUCP_003230	XLOC_138412	ENSRNOT00000033017	0.958337369	9.22E-07
TUCP_003230	XLOC_138412	ENSRNOT00000013759	-0.950668102	2.12E-06
TUCP_003230	XLOC_138412	ENSRNOT00000018341	-0.951479131	1.95E-06
TUCP_003230	XLOC_138412	ENSRNOT00000026543	0.965941865	3.41E-07
TUCP_003230	XLOC_138412	ENSRNOT00000012187	0.950253554	2.21E-06
TUCP_003230	XLOC_138412	ENSRNOT00000073564	-0.951579519	1.93E-06
TUCP_003230	XLOC_138412	ENSRNOT00000024969	0.964763801	4.03E-07
TUCP_003230	XLOC_138412	ENSRNOT00000015476	-0.950110987	2.24E-06
TUCP_003230	XLOC_138412	ENSRNOT00000065300	0.956112366	1.19E-06
TUCP_003230	XLOC_138412	ENSRNOT00000025079	-0.958094199	9.49E-07
TUCP_003230	XLOC_138412	ENSRNOT00000048754	-0.964352221	4.27E-07
TUCP_003230	XLOC_138412	ENSRNOT00000025075	0.958319548	9.24E-07
TUCP_003230	XLOC_138412	ENSRNOT00000057160	0.975273626	6.98E-08
TUCP_003230	XLOC_138412	ENSRNOT00000024402	0.970031998	1.81E-07
TUCP_003230	XLOC_138412	ENSRNOT00000002837	-0.957649109	9.99E-07
TUCP_003230	XLOC_138412	ENSRNOT00000089933	0.97266665	1.15E-07
TUCP_003230	XLOC_138412	ENSRNOT00000009811	0.971883849	1.32E-07
TUCP_003230	XLOC_138412	ENSRNOT00000025517	0.954359297	1.44E-06
TUCP_003230	XLOC_138412	ENSRNOT00000065168	0.951978762	1.85E-06
TUCP_003230	XLOC_138412	ENSRNOT00000085228	0.952762328	1.71E-06
TUCP_003230	XLOC_138412	ENSRNOT00000032780	0.956227916	1.18E-06
TUCP_003230	XLOC_138412	ENSRNOT00000009708	-0.95271496	1.72E-06
TUCP_003230	XLOC_138412	ENSRNOT00000061253	0.955583229	1.26E-06
TUCP_003230	XLOC_138412	ENSRNOT00000033677	-0.961002077	6.65E-07
TUCP_003230	XLOC_138412	ENSRNOT00000077231	0.958679869	8.85E-07
TUCP_003230	XLOC_138412	ENSRNOT00000011499	-0.954359177	1.44E-06
TUCP_003230	XLOC_138412	ENSRNOT00000061392	0.95874088	8.79E-07
TUCP_003230	XLOC_138412	ENSRNOT00000004787	-0.958799298	8.72E-07
TUCP_003230	XLOC_138412	ENSRNOT00000037039	-0.952530617	1.75E-06
TUCP_003230	XLOC_138412	ENSRNOT00000016428	0.954966567	1.35E-06
TUCP_003230	XLOC_138412	ENSRNOT00000091909	0.965914524	3.42E-07
TUCP_003230	XLOC_138412	ENSRNOT00000077727	-0.956931458	1.09E-06
TUCP_003230	XLOC_138412	ENSRNOT00000041044	0.965583457	3.59E-07
TUCP_003230	XLOC_138412	ENSRNOT00000037133	0.966553762	3.12E-07
TUCP_003230	XLOC_138412	ENSRNOT00000010541	0.954458774	1.43E-06
TUCP_003230	XLOC_138412	ENSRNOT00000017556	0.966146721	3.31E-07
TUCP_003230	XLOC_138412	ENSRNOT00000084709	0.950156151	2.23E-06
TUCP_003230	XLOC_138412	ENSRNOT00000026481	0.951857561	1.88E-06
TUCP_003230	XLOC_138412	ENSRNOT00000021803	-0.956337154	1.16E-06
TUCP_003230	XLOC_138412	ENSRNOT00000001081	0.953430211	1.60E-06
TUCP_003230	XLOC_138412	ENSRNOT00000083220	0.971002438	1.54E-07
TUCP_003230	XLOC_138412	ENSRNOT00000003544	-0.952714206	1.72E-06
TUCP_003230	XLOC_138412	ENSRNOT00000003780	0.954832218	1.37E-06
TUCP_003230	XLOC_138412	ENSRNOT00000086119	-0.965006795	3.90E-07
TUCP_003230	XLOC_138412	ENSRNOT00000022190	-0.962772625	5.29E-07
TUCP_003230	XLOC_138412	ENSRNOT00000016132	-0.95028157	2.20E-06
TUCP_003230	XLOC_138412	ENSRNOT00000019133	0.969554448	1.96E-07
TUCP_003230	XLOC_138412	ENSRNOT00000067780	-0.950165082	2.23E-06
TUCP_003230	XLOC_138412	ENSRNOT00000047093	0.963290154	4.94E-07
TUCP_003230	XLOC_138412	ENSRNOT00000025966	0.952200101	1.81E-06

TUCP_003230	XLOC_138412	ENSRNOT00000003787	-0.952577151	1.74E-06
TUCP_003230	XLOC_138412	ENSRNOT00000026643	0.960970111	6.68E-07
TUCP_003230	XLOC_138412	ENSRNOT00000007314	-0.960499716	7.09E-07
TUCP_003230	XLOC_138412	ENSRNOT00000077999	0.966400316	3.19E-07
TUCP_003230	XLOC_138412	ENSRNOT00000081047	0.955919506	1.22E-06
TUCP_003230	XLOC_138412	ENSRNOT00000081512	0.970364793	1.71E-07
TUCP_003230	XLOC_138412	ENSRNOT00000005486	-0.95477046	1.38E-06
TUCP_003230	XLOC_138412	ENSRNOT00000027910	0.960442664	7.14E-07
TUCP_003230	XLOC_138412	ENSRNOT00000006034	-0.95240957	1.77E-06
TUCP_003230	XLOC_138412	ENSRNOT00000087429	0.95648526	1.14E-06
TUCP_003230	XLOC_138412	ENSRNOT00000013067	-0.956837248	1.10E-06
TUCP_003230	XLOC_138412	ENSRNOT00000080207	0.979951004	2.47E-08
TUCP_003230	XLOC_138412	ENSRNOT00000084088	0.955320857	1.30E-06
TUCP_003230	XLOC_138412	ENSRNOT00000000302	0.966125601	3.32E-07
TUCP_003230	XLOC_138412	ENSRNOT00000052224	0.963082003	5.08E-07
TUCP_003230	XLOC_138412	ENSRNOT00000078070	0.989113014	1.18E-09
TUCP_003230	XLOC_138412	ENSRNOT00000026909	-0.954606835	1.41E-06
TUCP_003230	XLOC_138412	ENSRNOT00000088182	0.951038746	2.04E-06
TUCP_003230	XLOC_138412	ENSRNOT00000089293	0.963120275	5.05E-07
TUCP_003230	XLOC_138412	ENSRNOT00000028885	-0.952812659	1.70E-06
TUCP_003230	XLOC_138412	ENSRNOT00000040069	0.95212384	1.83E-06
TUCP_003230	XLOC_138412	ENSRNOT00000000717	-0.950657455	2.12E-06
TUCP_003230	XLOC_138412	ENSRNOT00000003568	0.960575317	7.02E-07
TUCP_003230	XLOC_138412	ENSRNOT00000052063	0.954442931	1.43E-06
TUCP_003230	XLOC_138412	ENSRNOT00000060002	-0.959381277	8.13E-07
TUCP_003230	XLOC_138412	ENSRNOT00000004046	-0.976644718	5.26E-08
TUCP_003230	XLOC_138412	ENSRNOT00000010367	0.957580105	1.01E-06
TUCP_003230	XLOC_138412	ENSRNOT00000006395	-0.950605733	2.13E-06
TUCP_003230	XLOC_138412	ENSRNOT00000006824	-0.955188199	1.32E-06
TUCP_003230	XLOC_138412	ENSRNOT00000082151	-0.960846518	6.78E-07
TUCP_003230	XLOC_138412	ENSRNOT00000066133	-0.956346619	1.16E-06
TUCP_003230	XLOC_138412	ENSRNOT00000029202	-0.959380099	8.13E-07
TUCP_003230	XLOC_138412	ENSRNOT00000089963	0.951343712	1.98E-06
TUCP_003230	XLOC_138412	ENSRNOT00000088054	0.962086583	5.79E-07
TUCP_003230	XLOC_138412	ENSRNOT00000044395	0.95324661	1.63E-06
TUCP_003230	XLOC_138412	ENSRNOT00000050834	-0.966673622	3.06E-07
TUCP_003230	XLOC_138412	ENSRNOT00000066024	0.956094176	1.19E-06
TUCP_003230	XLOC_138412	ENSRNOT00000023543	-0.950777757	2.09E-06
TUCP_003230	XLOC_138412	ENSRNOT00000026691	0.954162575	1.48E-06
TUCP_003230	XLOC_138412	ENSRNOT00000055992	-0.96726125	2.80E-07
TUCP_003230	XLOC_138412	ENSRNOT00000027970	0.952015167	1.85E-06
TUCP_003230	XLOC_138412	ENSRNOT00000015624	0.951824949	1.88E-06
TUCP_003230	XLOC_138412	ENSRNOT00000028876	-0.971247193	1.47E-07
TUCP_003230	XLOC_138412	ENSRNOT00000078306	0.959164275	8.35E-07
TUCP_003230	XLOC_138412	ENSRNOT00000080695	0.977818316	4.07E-08
TUCP_003230	XLOC_138412	ENSRNOT00000079011	-0.950487189	2.16E-06
TUCP_003230	XLOC_138412	ENSRNOT00000055386	0.954648225	1.40E-06
TUCP_003230	XLOC_138412	ENSRNOT00000018074	-0.962335162	5.60E-07
TUCP_003230	XLOC_138412	ENSRNOT00000091205	0.950309555	2.19E-06
TUCP_003230	XLOC_138412	ENSRNOT00000010180	-0.960827055	6.80E-07
TUCP_003230	XLOC_138412	ENSRNOT00000080608	0.954290109	1.46E-06

TUCP_003230	XLOC_138412	ENSRNOT00000000120	0.958463677	9.08E-07
TUCP_003230	XLOC_138412	ENSRNOT00000038182	-0.959806848	7.72E-07
TUCP_003230	XLOC_138412	ENSRNOT00000029125	0.964930014	3.94E-07
TUCP_003230	XLOC_138412	ENSRNOT00000060696	0.960397604	7.18E-07
TUCP_003230	XLOC_138412	ENSRNOT00000042125	0.982561297	1.23E-08
TUCP_003230	XLOC_138412	ENSRNOT00000060698	-0.954360889	1.44E-06
TUCP_003230	XLOC_138412	ENSRNOT00000009900	-0.959713999	7.81E-07
TUCP_003230	XLOC_138412	ENSRNOT00000058351	0.958972722	8.54E-07
TUCP_003230	XLOC_138412	ENSRNOT00000014814	0.967170956	2.84E-07
TUCP_003230	XLOC_138412	ENSRNOT00000010748	0.960099343	7.45E-07
TUCP_003230	XLOC_138412	ENSRNOT00000026941	0.958827202	8.69E-07
TUCP_003230	XLOC_138412	ENSRNOT00000024491	-0.961140935	6.54E-07
TUCP_003230	XLOC_138412	ENSRNOT00000084597	-0.954728998	1.39E-06
TUCP_003230	XLOC_138412	ENSRNOT00000024947	-0.95023062	2.21E-06
TUCP_003230	XLOC_138412	ENSRNOT00000021534	-0.951642145	1.92E-06
TUCP_003230	XLOC_138412	ENSRNOT00000067901	-0.951499914	1.95E-06
TUCP_003230	XLOC_138412	ENSRNOT00000060362	0.959682258	7.84E-07
TUCP_003230	XLOC_138412	ENSRNOT00000027967	0.955473843	1.28E-06
TUCP_003230	XLOC_138412	ENSRNOT00000008982	0.962555348	5.44E-07
TUCP_003230	XLOC_138412	ENSRNOT00000047192	-0.958964265	8.55E-07
TUCP_003230	XLOC_138412	ENSRNOT00000022097	0.953523517	1.58E-06
TUCP_003230	XLOC_138412	ENSRNOT00000036613	-0.953003402	1.67E-06
TUCP_003230	XLOC_138412	ENSRNOT00000083127	0.961884731	5.94E-07
TUCP_003230	XLOC_138412	ENSRNOT00000025705	0.950435268	2.17E-06
TUCP_003230	XLOC_138412	ENSRNOT00000083957	0.950981914	2.05E-06
TUCP_003230	XLOC_138412	ENSRNOT00000024016	-0.956182098	1.18E-06
TUCP_003230	XLOC_138412	ENSRNOT00000046639	0.956625885	1.12E-06
TUCP_003230	XLOC_138412	ENSRNOT00000015799	0.977179449	4.69E-08
TUCP_003230	XLOC_138412	ENSRNOT00000008113	-0.951127312	2.02E-06
TUCP_003230	XLOC_138412	ENSRNOT00000022552	-0.963025912	5.11E-07
TUCP_003230	XLOC_138412	ENSRNOT00000021629	-0.951526677	1.94E-06
TUCP_003231	XLOC_138414	ENSRNOT00000009811	0.953623983	1.56E-06
TUCP_003231	XLOC_138414	ENSRNOT00000065168	0.95060955	2.13E-06
TUCP_003231	XLOC_138414	ENSRNOT00000005486	-0.953218112	1.63E-06
TUCP_003231	XLOC_138414	ENSRNOT00000067554	0.965842949	3.46E-07
TUCP_003231	XLOC_138414	ENSRNOT00000000383	-0.974997781	7.38E-08
TUCP_003231	XLOC_138414	ENSRNOT00000010784	0.951832324	1.88E-06
TUCP_003231	XLOC_138414	ENSRNOT00000001893	0.973766939	9.36E-08
TUCP_003231	XLOC_138414	ENSRNOT00000061253	0.960274557	7.29E-07
TUCP_003231	XLOC_138414	ENSRNOT00000081512	0.955952668	1.21E-06
TUCP_003231	XLOC_138414	ENSRNOT00000061201	-0.950581411	2.14E-06
TUCP_003231	XLOC_138414	ENSRNOT00000041044	0.958168622	9.40E-07
TUCP_003231	XLOC_138414	ENSRNOT00000023989	0.950996427	2.05E-06
TUCP_003231	XLOC_138414	ENSRNOT00000080608	0.950999263	2.05E-06
TUCP_003231	XLOC_138414	ENSRNOT00000089319	0.951751642	1.90E-06
TUCP_003231	XLOC_138414	ENSRNOT00000078737	-0.950360208	2.18E-06
TUCP_003231	XLOC_138414	ENSRNOT00000057361	0.953626064	1.56E-06
TUCP_003231	XLOC_138414	ENSRNOT00000086706	0.955425435	1.29E-06
TUCP_003231	XLOC_138414	ENSRNOT00000021114	0.955989611	1.21E-06
TUCP_003231	XLOC_138414	ENSRNOT00000046745	0.951621607	1.92E-06
TUCP_003231	XLOC_138414	ENSRNOT00000017556	0.965681893	3.54E-07

TUCP_003231	XLOC_138414	ENSRNOT00000080750	0.955574262	1.26E-06
TUCP_003231	XLOC_138414	ENSRNOT00000000469	0.956835132	1.10E-06
TUCP_003231	XLOC_138414	ENSRNOT00000033017	0.951176643	2.01E-06
TUCP_003231	XLOC_138414	ENSRNOT00000007028	-0.954665911	1.40E-06
TUCP_003231	XLOC_138414	ENSRNOT00000057562	0.959208238	8.31E-07
TUCP_003231	XLOC_138414	ENSRNOT00000072118	0.954370913	1.44E-06
TUCP_003231	XLOC_138414	ENSRNOT00000081981	0.951985756	1.85E-06
TUCP_003231	XLOC_138414	ENSRNOT00000057953	0.957943062	9.65E-07
TUCP_003231	XLOC_138414	ENSRNOT00000075558	0.953410946	1.60E-06
TUCP_003231	XLOC_138414	ENSRNOT00000074972	0.965444202	3.66E-07
TUCP_003231	XLOC_138414	ENSRNOT00000045285	0.958110632	9.47E-07
TUCP_003231	XLOC_138414	ENSRNOT00000090057	0.973292868	1.02E-07
TUCP_003231	XLOC_138414	ENSRNOT00000007821	0.950881754	2.07E-06
TUCP_003231	XLOC_138414	ENSRNOT00000089933	0.958875101	8.65E-07
TUCP_003231	XLOC_138414	ENSRNOT00000015799	0.965887316	3.44E-07
TUCP_004514	XLOC_198926	ENSRNOT00000083687	0.992336819	2.05E-10
TUCP_004515	XLOC_199002	ENSRNOT00000004702	0.953955495	1.51E-06
TUCP_001807	XLOC_074917	ENSRNOT00000003840	0.951728845	1.90E-06
TUCP_001807	XLOC_074917	ENSRNOT00000013260	0.950221309	2.21E-06
TUCP_001807	XLOC_074917	ENSRNOT00000010609	0.978843107	3.22E-08
TUCP_001807	XLOC_074917	ENSRNOT00000008971	0.969122122	2.10E-07
TUCP_001807	XLOC_074917	ENSRNOT00000006554	0.964537258	4.16E-07
TUCP_004452	XLOC_196072	ENSRNOT00000088287	0.963003832	5.13E-07
TUCP_004452	XLOC_196072	ENSRNOT00000089314	0.964200545	4.36E-07
TUCP_003331	XLOC_143339	ENSRNOT00000005846	-0.957783551	9.84E-07
TUCP_003331	XLOC_143339	ENSRNOT00000037397	-0.960350418	7.22E-07
TUCP_003331	XLOC_143339	ENSRNOT00000012632	-0.96666223	3.07E-07
TUCP_003331	XLOC_143339	ENSRNOT00000031667	-0.95823091	9.33E-07
TUCP_003331	XLOC_143339	ENSRNOT00000010264	-0.956949926	1.08E-06
TUCP_003331	XLOC_143339	ENSRNOT00000032333	-0.962502593	5.48E-07
TUCP_003331	XLOC_143339	ENSRNOT00000021550	-0.955627378	1.26E-06
TUCP_004105	XLOC_179460	ENSRNOT00000079548	-0.960701358	6.91E-07
TUCP_004104	XLOC_179430	ENSRNOT00000084075	0.982014949	1.44E-08
TUCP_004104	XLOC_179430	ENSRNOT00000012880	0.95118578	2.01E-06
TUCP_004104	XLOC_179430	ENSRNOT00000074465	0.987549419	2.31E-09
TUCP_004104	XLOC_179430	ENSRNOT00000090898	0.95788696	9.72E-07
TUCP_004104	XLOC_179430	ENSRNOT00000079805	0.982354968	1.31E-08
TUCP_004104	XLOC_179430	ENSRNOT00000040912	0.987549419	2.31E-09
TUCP_004104	XLOC_179430	ENSRNOT00000072648	0.987549419	2.31E-09
TUCP_004104	XLOC_179430	ENSRNOT00000007398	0.97934884	2.86E-08
TUCP_004104	XLOC_179430	ENSRNOT00000070803	0.987549419	2.31E-09
TUCP_004104	XLOC_179430	ENSRNOT00000012196	0.987549419	2.31E-09
TUCP_004104	XLOC_179430	ENSRNOT00000085772	0.987549419	2.31E-09
TUCP_004104	XLOC_179430	ENSRNOT00000093284	0.987392443	2.46E-09
TUCP_004104	XLOC_179430	ENSRNOT00000086374	0.974952598	7.44E-08
TUCP_004104	XLOC_179430	ENSRNOT00000015270	0.987549419	2.31E-09
TUCP_004104	XLOC_179430	ENSRNOT00000046192	0.987549419	2.31E-09
TUCP_004104	XLOC_179430	ENSRNOT00000044226	0.987549419	2.31E-09
TUCP_004104	XLOC_179430	ENSRNOT00000081898	0.987239469	2.61E-09
TUCP_004104	XLOC_179430	ENSRNOT00000080291	0.985731908	4.55E-09
TUCP_004104	XLOC_179430	ENSRNOT00000004293	0.966909205	2.96E-07

TUCP_004104	XLOC_179430	ENSRNOT00000015870	0.952401814	1.78E-06
TUCP_004104	XLOC_179430	ENSRNOT00000001464	0.987549419	2.31E-09
TUCP_004104	XLOC_179430	ENSRNOT00000090716	0.973414683	1.00E-07
TUCP_004104	XLOC_179430	ENSRNOT00000083542	0.964652518	4.10E-07
TUCP_004104	XLOC_179430	ENSRNOT00000076044	0.987549419	2.31E-09
TUCP_004104	XLOC_179430	ENSRNOT00000074353	0.987549419	2.31E-09
TUCP_004104	XLOC_179430	ENSRNOT00000087122	0.963019479	5.12E-07
TUCP_004104	XLOC_179430	ENSRNOT00000074295	0.971838641	1.33E-07
TUCP_004104	XLOC_179430	ENSRNOT00000057873	0.987549419	2.31E-09
TUCP_004104	XLOC_179430	ENSRNOT00000083002	0.975421664	6.78E-08
TUCP_004104	XLOC_179430	ENSRNOT00000088399	0.987604915	2.26E-09
TUCP_004104	XLOC_179430	ENSRNOT00000086248	0.987549419	2.31E-09
TUCP_004104	XLOC_179430	ENSRNOT00000089750	0.987438132	2.41E-09
TUCP_004104	XLOC_179430	ENSRNOT00000030517	0.957123762	1.06E-06
TUCP_004104	XLOC_179430	ENSRNOT00000034772	0.98037401	2.22E-08
TUCP_004104	XLOC_179430	ENSRNOT00000064601	0.953931167	1.51E-06
TUCP_004104	XLOC_179430	ENSRNOT00000002834	0.987549419	2.31E-09
TUCP_004104	XLOC_179430	ENSRNOT00000080812	0.974684178	7.85E-08
TUCP_004104	XLOC_179430	ENSRNOT00000092755	0.987549419	2.31E-09
TUCP_004104	XLOC_179430	ENSRNOT00000031683	0.987549419	2.31E-09
TUCP_004104	XLOC_179430	ENSRNOT00000087764	0.974609244	7.96E-08
TUCP_004107	XLOC_179475	ENSRNOT00000015124	0.96620422	3.28E-07
TUCP_004107	XLOC_179475	ENSRNOT00000057851	-0.95778113	9.84E-07
TUCP_004107	XLOC_179475	ENSRNOT00000064776	-0.953090294	1.65E-06
TUCP_004107	XLOC_179475	ENSRNOT00000026699	-0.976331333	5.62E-08
TUCP_004106	XLOC_179461	ENSRNOT00000074361	0.950517936	2.15E-06
TUCP_004106	XLOC_179461	ENSRNOT00000036766	0.958336991	9.22E-07
TUCP_001193	XLOC_044844	ENSRNOT00000092026	0.96620614	3.28E-07
TUCP_001193	XLOC_044844	ENSRNOT00000093075	1	0
TUCP_001193	XLOC_044844	ENSRNOT00000058260	1	0
TUCP_001193	XLOC_044844	ENSRNOT00000088887	1	0
TUCP_001193	XLOC_044844	ENSRNOT00000084986	1	0
TUCP_001193	XLOC_044844	ENSRNOT00000074346	0.9702256	1.75E-07
TUCP_001193	XLOC_044844	ENSRNOT00000073209	0.983711602	8.79E-09
TUCP_001193	XLOC_044844	ENSRNOT00000080155	0.973069673	1.07E-07
TUCP_001193	XLOC_044844	ENSRNOT00000072573	0.996213272	6.09E-12
TUCP_001193	XLOC_044844	ENSRNOT00000017828	0.996001583	7.99E-12
TUCP_001193	XLOC_044844	ENSRNOT00000080444	0.969625907	1.93E-07
TUCP_001193	XLOC_044844	ENSRNOT00000071312	1	0
TUCP_001193	XLOC_044844	ENSRNOT00000088245	1	0
TUCP_001193	XLOC_044844	ENSRNOT00000036565	0.969065335	2.12E-07
TUCP_001193	XLOC_044844	ENSRNOT00000058833	0.998673261	3.23E-14
TUCP_001193	XLOC_044844	ENSRNOT00000078052	1	0
TUCP_001193	XLOC_044844	ENSRNOT00000078494	0.999927093	1.62E-20
TUCP_001193	XLOC_044844	ENSRNOT00000085653	1	0
TUCP_001193	XLOC_044844	ENSRNOT00000057950	0.969329371	2.03E-07
TUCP_001193	XLOC_044844	ENSRNOT00000081992	0.999320526	1.14E-15
TUCP_001193	XLOC_044844	ENSRNOT00000038553	0.955765808	1.24E-06
TUCP_001193	XLOC_044844	ENSRNOT00000084120	0.958115447	9.46E-07
TUCP_001193	XLOC_044844	ENSRNOT00000055406	1	0
TUCP_001193	XLOC_044844	ENSRNOT00000070817	0.998968079	9.20E-15

TUCP_001193	XLOC_044844	ENSRNOT00000026753	1	0
TUCP_001193	XLOC_044844	ENSRNOT00000080533	0.996682529	3.15E-12
TUCP_001193	XLOC_044844	ENSRNOT00000072774	0.979665261	2.65E-08
TUCP_001193	XLOC_044844	ENSRNOT00000086031	1	0
TUCP_001193	XLOC_044844	ENSRNOT00000014312	1	0
TUCP_001193	XLOC_044844	ENSRNOT00000042882	0.963218427	4.98E-07
TUCP_001193	XLOC_044844	ENSRNOT00000088012	0.956765806	1.11E-06
TUCP_001193	XLOC_044844	ENSRNOT00000051231	0.999242378	1.96E-15
TUCP_001193	XLOC_044844	ENSRNOT00000089407	0.992986194	1.32E-10
TUCP_001193	XLOC_044844	ENSRNOT00000055034	0.998331441	1.02E-13
TUCP_001193	XLOC_044844	ENSRNOT00000046837	0.97252559	1.18E-07
TUCP_001961	XLOC_079154	ENSRNOT00000089806	0.963839078	4.58E-07
TUCP_001961	XLOC_079154	ENSRNOT00000013088	0.969235011	2.06E-07
TUCP_001961	XLOC_079154	ENSRNOT00000090547	0.959383256	8.13E-07
TUCP_004459	XLOC_196157	ENSRNOT00000085659	0.961166885	6.52E-07
TUCP_004459	XLOC_196157	ENSRNOT00000002220	0.966062761	3.35E-07
TUCP_004241	XLOC_186296	ENSRNOT00000035865	-0.950234439	2.21E-06
TUCP_001621	XLOC_064489	ENSRNOT00000013817	0.96551265	3.63E-07
TUCP_001621	XLOC_064489	ENSRNOT00000028210	-0.957645682	1.00E-06
TUCP_001621	XLOC_064489	ENSRNOT00000001153	0.96106972	6.60E-07
TUCP_001621	XLOC_064489	ENSRNOT00000035448	-0.957757535	9.87E-07
TUCP_001621	XLOC_064489	ENSRNOT00000002982	0.963309642	4.92E-07
TUCP_001621	XLOC_064489	ENSRNOT00000082183	0.959199185	8.31E-07
TUCP_001621	XLOC_064489	ENSRNOT00000080216	-0.967102482	2.87E-07
TUCP_001621	XLOC_064489	ENSRNOT00000026391	0.970264462	1.74E-07
TUCP_001621	XLOC_064489	ENSRNOT00000081678	0.970760844	1.60E-07
TUCP_001621	XLOC_064489	ENSRNOT00000028271	0.958836133	8.69E-07
TUCP_001621	XLOC_064489	ENSRNOT00000018155	-0.972288957	1.23E-07
TUCP_001621	XLOC_064489	ENSRNOT00000026259	0.950042899	2.25E-06
TUCP_001621	XLOC_064489	ENSRNOT00000010593	0.96673847	3.03E-07
TUCP_001621	XLOC_064489	ENSRNOT00000006217	0.953615992	1.56E-06
TUCP_001621	XLOC_064489	ENSRNOT00000003087	0.950213192	2.22E-06
TUCP_001621	XLOC_064489	ENSRNOT00000043831	0.966053796	3.35E-07
TUCP_001621	XLOC_064489	ENSRNOT00000078739	-0.976447166	5.49E-08
TUCP_001621	XLOC_064489	ENSRNOT00000090987	-0.957303071	1.04E-06
TUCP_001621	XLOC_064489	ENSRNOT00000025081	0.952402133	1.78E-06
TUCP_001621	XLOC_064489	ENSRNOT00000073041	0.978958737	3.14E-08
TUCP_001621	XLOC_064489	ENSRNOT00000001697	0.955941261	1.21E-06
TUCP_001621	XLOC_064489	ENSRNOT00000064060	0.950293038	2.20E-06
TUCP_001621	XLOC_064489	ENSRNOT00000077526	0.955458374	1.28E-06
TUCP_001621	XLOC_064489	ENSRNOT00000012767	0.96822307	2.42E-07
TUCP_001621	XLOC_064489	ENSRNOT00000077706	0.95719369	1.05E-06
TUCP_001621	XLOC_064489	ENSRNOT00000009275	-0.956051291	1.20E-06
TUCP_001621	XLOC_064489	ENSRNOT00000079784	-0.966660817	3.07E-07
TUCP_001621	XLOC_064489	ENSRNOT00000076973	0.956254075	1.17E-06
TUCP_001621	XLOC_064489	ENSRNOT00000025036	-0.956014268	1.20E-06
TUCP_001621	XLOC_064489	ENSRNOT00000037133	0.950177495	2.22E-06
TUCP_001621	XLOC_064489	ENSRNOT00000084567	0.952434073	1.77E-06
TUCP_001621	XLOC_064489	ENSRNOT00000007855	0.960658152	6.95E-07
TUCP_001621	XLOC_064489	ENSRNOT00000085362	0.952248336	1.80E-06
TUCP_001621	XLOC_064489	ENSRNOT00000037679	0.955816692	1.23E-06

TUCP_001621	XLOC_064489	ENSRNOT00000052063	0.95524382	1.31E-06
TUCP_001621	XLOC_064489	ENSRNOT00000049245	0.9589424	8.58E-07
TUCP_001621	XLOC_064489	ENSRNOT00000026909	-0.96457708	4.14E-07
TUCP_001621	XLOC_064489	ENSRNOT00000066521	-0.952319925	1.79E-06
TUCP_001621	XLOC_064489	ENSRNOT00000018341	-0.95251064	1.76E-06
TUCP_001621	XLOC_064489	ENSRNOT00000028685	0.951544385	1.94E-06
TUCP_001621	XLOC_064489	ENSRNOT00000017805	0.955237791	1.31E-06
TUCP_001621	XLOC_064489	ENSRNOT00000012801	0.952139236	1.82E-06
TUCP_001621	XLOC_064489	ENSRNOT00000034669	-0.964440143	4.22E-07
TUCP_001621	XLOC_064489	ENSRNOT00000025523	0.958260321	9.30E-07
TUCP_001621	XLOC_064489	ENSRNOT00000019115	0.959623856	7.90E-07
TUCP_001621	XLOC_064489	ENSRNOT00000003544	-0.958712342	8.82E-07
TUCP_001621	XLOC_064489	ENSRNOT00000013279	0.954108543	1.48E-06
TUCP_001621	XLOC_064489	ENSRNOT00000012279	0.957283212	1.04E-06
TUCP_001621	XLOC_064489	ENSRNOT00000078579	0.95252514	1.75E-06
TUCP_001621	XLOC_064489	ENSRNOT00000006205	-0.957748877	9.88E-07
TUCP_001621	XLOC_064489	ENSRNOT00000058495	-0.958255866	9.31E-07
TUCP_001621	XLOC_064489	ENSRNOT00000002511	-0.950063632	2.25E-06
TUCP_001621	XLOC_064489	ENSRNOT00000000718	0.958219281	9.35E-07
TUCP_001621	XLOC_064489	ENSRNOT00000007414	0.956174615	1.18E-06
TUCP_001621	XLOC_064489	ENSRNOT00000085458	-0.955463125	1.28E-06
TUCP_001621	XLOC_064489	ENSRNOT00000061132	0.966796731	3.01E-07
TUCP_001621	XLOC_064489	ENSRNOT00000047530	0.966641533	3.08E-07
TUCP_001621	XLOC_064489	ENSRNOT00000019816	-0.951387038	1.97E-06
TUCP_001621	XLOC_064489	ENSRNOT00000016376	-0.963146288	5.03E-07
TUCP_001621	XLOC_064489	ENSRNOT00000089068	0.955068722	1.34E-06
TUCP_001621	XLOC_064489	ENSRNOT00000013344	-0.954016907	1.50E-06
TUCP_001621	XLOC_064489	ENSRNOT00000013228	0.958691248	8.84E-07
TUCP_001621	XLOC_064489	ENSRNOT00000020417	-0.961570096	6.19E-07
TUCP_001621	XLOC_064489	ENSRNOT00000067949	0.953839974	1.53E-06
TUCP_001621	XLOC_064489	ENSRNOT00000019759	0.967423493	2.74E-07
TUCP_001621	XLOC_064489	ENSRNOT00000006804	0.95161226	1.93E-06
TUCP_001621	XLOC_064489	ENSRNOT00000046639	0.955597823	1.26E-06
TUCP_001621	XLOC_064489	ENSRNOT00000039572	-0.952493523	1.76E-06
TUCP_001621	XLOC_064489	ENSRNOT00000002621	0.957311809	1.04E-06
TUCP_001808	XLOC_074931	ENSRNOT00000091911	0.969426115	2.00E-07
TUCP_001808	XLOC_074931	ENSRNOT00000076530	0.973631564	9.60E-08
TUCP_003218	XLOC_138410	ENSRNOT00000065312	0.967744941	2.60E-07
TUCP_004282	XLOC_188199	ENSRNOT00000000438	0.954268326	1.46E-06
TUCP_004282	XLOC_188199	ENSRNOT00000014217	0.957001802	1.08E-06
TUCP_004282	XLOC_188199	ENSRNOT00000088589	0.966236721	3.26E-07
TUCP_004284	XLOC_188211	ENSRNOT00000066058	0.95245249	1.77E-06
TUCP_004122	XLOC_180258	ENSRNOT00000084500	0.984887141	6.05E-09
TUCP_004122	XLOC_180258	ENSRNOT00000093467	0.996134431	6.75E-12
TUCP_004122	XLOC_180258	ENSRNOT00000029340	0.999116572	4.23E-15
TUCP_004122	XLOC_180258	ENSRNOT00000077972	0.962758032	5.30E-07
TUCP_004122	XLOC_180258	ENSRNOT00000038646	1	0
TUCP_004122	XLOC_180258	ENSRNOT00000012095	0.957750287	9.88E-07
TUCP_004122	XLOC_180258	ENSRNOT00000017909	0.975587192	6.56E-08
TUCP_004122	XLOC_180258	ENSRNOT00000092579	1	0
TUCP_004122	XLOC_180258	ENSRNOT00000089235	0.998661162	3.38E-14

TUCP_004122	XLOC_180258	ENSRNOT00000036383	0.988460809	1.58E-09
TUCP_004122	XLOC_180258	ENSRNOT00000076528	0.997115373	1.57E-12
TUCP_004122	XLOC_180258	ENSRNOT00000075193	0.964326971	4.29E-07
TUCP_004122	XLOC_180258	ENSRNOT00000083034	0.990934646	4.75E-10
TUCP_004122	XLOC_180258	ENSRNOT00000089607	0.995091455	2.23E-11
TUCP_004122	XLOC_180258	ENSRNOT00000086014	0.960893638	6.74E-07
TUCP_004122	XLOC_180258	ENSRNOT00000054939	0.958021495	9.57E-07
TUCP_004122	XLOC_180258	ENSRNOT00000052315	0.983687082	8.85E-09
TUCP_004122	XLOC_180258	ENSRNOT00000088168	1	0
TUCP_004122	XLOC_180258	ENSRNOT00000046231	0.96225463	5.66E-07
TUCP_004122	XLOC_180258	ENSRNOT00000013459	0.979098017	3.03E-08
TUCP_004122	XLOC_180258	ENSRNOT00000078137	1	0
TUCP_004122	XLOC_180258	ENSRNOT00000077764	0.977143528	4.73E-08
TUCP_004122	XLOC_180258	ENSRNOT00000043661	0.966975951	2.93E-07
TUCP_004122	XLOC_180258	ENSRNOT00000092740	1	0
TUCP_004122	XLOC_180258	ENSRNOT00000000117	0.990989174	4.61E-10
TUCP_004122	XLOC_180258	ENSRNOT00000071877	0.967113583	2.87E-07
TUCP_004122	XLOC_180258	ENSRNOT00000070958	1	0
TUCP_004122	XLOC_180258	ENSRNOT00000076757	0.959205354	8.31E-07
TUCP_004122	XLOC_180258	ENSRNOT00000085310	0.965230243	3.78E-07
TUCP_004122	XLOC_180258	ENSRNOT00000047241	0.966576993	3.11E-07
TUCP_004122	XLOC_180258	ENSRNOT00000027268	1	1.33E-79
TUCP_004122	XLOC_180258	ENSRNOT00000004459	1	0
TUCP_004122	XLOC_180258	ENSRNOT00000048373	0.959096578	8.42E-07
TUCP_004122	XLOC_180258	ENSRNOT00000061516	0.987595648	2.27E-09
TUCP_004122	XLOC_180258	ENSRNOT00000088148	1	0
TUCP_004122	XLOC_180258	ENSRNOT00000088527	1	0
TUCP_004122	XLOC_180258	ENSRNOT00000078701	1	1.33E-79
TUCP_004122	XLOC_180258	ENSRNOT00000052308	0.962646127	5.38E-07
TUCP_004122	XLOC_180258	ENSRNOT00000021748	1	0
TUCP_004122	XLOC_180258	ENSRNOT00000089085	1	0
TUCP_004122	XLOC_180258	ENSRNOT00000008153	0.999703506	1.80E-17
TUCP_004122	XLOC_180258	ENSRNOT00000085896	0.971605382	1.39E-07
TUCP_004122	XLOC_180258	ENSRNOT00000079189	0.984972387	5.89E-09
TUCP_004122	XLOC_180258	ENSRNOT00000090078	1	0
TUCP_004122	XLOC_180258	ENSRNOT00000078020	0.958421666	9.13E-07
TUCP_004122	XLOC_180258	ENSRNOT00000092425	0.956092273	1.19E-06
TUCP_004122	XLOC_180258	ENSRNOT00000009776	1	0
TUCP_003907	XLOC_172121	ENSRNOT00000052288	0.958390395	9.16E-07
TUCP_003907	XLOC_172121	ENSRNOT00000022646	0.961807824	6.00E-07
TUCP_003907	XLOC_172121	ENSRNOT00000005909	0.982800822	1.15E-08
TUCP_003907	XLOC_172121	ENSRNOT00000008573	0.961639333	6.13E-07
TUCP_003907	XLOC_172121	ENSRNOT00000009979	0.962687665	5.35E-07
TUCP_003907	XLOC_172121	ENSRNOT00000082998	0.952074037	1.84E-06
TUCP_003907	XLOC_172121	ENSRNOT00000081246	0.963594669	4.74E-07
TUCP_003907	XLOC_172121	ENSRNOT00000019213	0.96315476	5.03E-07
TUCP_003907	XLOC_172121	ENSRNOT00000009877	0.98570969	4.58E-09
TUCP_003907	XLOC_172121	ENSRNOT00000027452	0.966500307	3.14E-07
TUCP_003907	XLOC_172121	ENSRNOT00000021348	0.966753263	3.03E-07
TUCP_003907	XLOC_172121	ENSRNOT00000006055	0.962970235	5.15E-07
TUCP_003907	XLOC_172121	ENSRNOT00000017668	0.951233051	2.00E-06

TUCP_003907	XLOC_172121	ENSRNOT00000071560	0.965926689	3.42E-07
TUCP_003907	XLOC_172121	ENSRNOT00000078445	-0.959075182	8.44E-07
TUCP_003907	XLOC_172121	ENSRNOT00000023326	0.963170962	5.02E-07
TUCP_003907	XLOC_172121	ENSRNOT00000026065	0.975058533	7.29E-08
TUCP_003907	XLOC_172121	ENSRNOT00000041709	0.958285787	9.27E-07
TUCP_003907	XLOC_172121	ENSRNOT00000005228	0.988545957	1.52E-09
TUCP_003907	XLOC_172121	ENSRNOT00000022726	0.967690908	2.63E-07
TUCP_003907	XLOC_172121	ENSRNOT00000021592	0.951902411	1.87E-06
TUCP_003907	XLOC_172121	ENSRNOT00000005659	0.970970498	1.55E-07
TUCP_003907	XLOC_172121	ENSRNOT00000044635	0.950060542	2.25E-06
TUCP_003907	XLOC_172121	ENSRNOT00000045326	0.957781445	9.84E-07
TUCP_003907	XLOC_172121	ENSRNOT00000086548	0.965847494	3.46E-07
TUCP_003907	XLOC_172121	ENSRNOT00000047210	0.960514838	7.07E-07
TUCP_003907	XLOC_172121	ENSRNOT00000019502	0.969446707	1.99E-07
TUCP_003907	XLOC_172121	ENSRNOT00000082107	0.957986766	9.61E-07
TUCP_003907	XLOC_172121	ENSRNOT00000028856	0.969164403	2.08E-07
TUCP_003907	XLOC_172121	ENSRNOT00000065300	0.966094613	3.33E-07
TUCP_003907	XLOC_172121	ENSRNOT00000015073	0.970235555	1.75E-07
TUCP_003907	XLOC_172121	ENSRNOT00000043216	0.982776901	1.16E-08
TUCP_003907	XLOC_172121	ENSRNOT00000080654	0.973658189	9.56E-08
TUCP_003907	XLOC_172121	ENSRNOT00000044742	0.959506926	8.01E-07
TUCP_003907	XLOC_172121	ENSRNOT00000081981	0.953068477	1.66E-06
TUCP_003907	XLOC_172121	ENSRNOT00000009496	0.961179632	6.50E-07
TUCP_003907	XLOC_172121	ENSRNOT00000027414	0.987853764	2.04E-09
TUCP_003907	XLOC_172121	ENSRNOT00000028563	0.98478819	6.25E-09
TUCP_003907	XLOC_172121	ENSRNOT00000015076	0.95859364	8.94E-07
TUCP_003907	XLOC_172121	ENSRNOT00000004698	0.983841274	8.44E-09
TUCP_003907	XLOC_172121	ENSRNOT00000003158	-0.960905493	6.73E-07
TUCP_003907	XLOC_172121	ENSRNOT00000004172	0.961449455	6.28E-07
TUCP_003907	XLOC_172121	ENSRNOT00000076516	0.961944515	5.90E-07
TUCP_003907	XLOC_172121	ENSRNOT00000014979	0.952755764	1.71E-06
TUCP_003907	XLOC_172121	ENSRNOT00000043554	0.969064979	2.12E-07
TUCP_003907	XLOC_172121	ENSRNOT00000091807	0.970236207	1.75E-07
TUCP_003907	XLOC_172121	ENSRNOT00000005942	0.985348196	5.19E-09
TUCP_003907	XLOC_172121	ENSRNOT00000057814	0.95930958	8.20E-07
TUCP_003907	XLOC_172121	ENSRNOT00000012463	-0.971081866	1.52E-07
TUCP_003907	XLOC_172121	ENSRNOT00000085251	-0.959073971	8.44E-07
TUCP_003907	XLOC_172121	ENSRNOT00000013046	-0.954695096	1.39E-06
TUCP_003907	XLOC_172121	ENSRNOT00000026783	0.981705324	1.57E-08
TUCP_003907	XLOC_172121	ENSRNOT00000005126	-0.952131849	1.83E-06
TUCP_003907	XLOC_172121	ENSRNOT00000012379	0.973722826	9.44E-08
TUCP_003907	XLOC_172121	ENSRNOT00000028646	0.98044514	2.18E-08
TUCP_003907	XLOC_172121	ENSRNOT00000011384	0.991832576	2.82E-10
TUCP_003907	XLOC_172121	ENSRNOT00000045699	0.955504215	1.27E-06
TUCP_003907	XLOC_172121	ENSRNOT00000087443	0.964604765	4.12E-07
TUCP_003907	XLOC_172121	ENSRNOT00000045083	0.960548535	7.04E-07
TUCP_003907	XLOC_172121	ENSRNOT00000006963	0.983171079	1.03E-08
TUCP_003907	XLOC_172121	ENSRNOT00000071764	0.961272464	6.43E-07
TUCP_003907	XLOC_172121	ENSRNOT00000079109	0.970655335	1.63E-07
TUCP_003907	XLOC_172121	ENSRNOT00000027944	0.984456439	6.96E-09
TUCP_003907	XLOC_172121	ENSRNOT00000074939	0.975949111	6.09E-08

TUCP_003907	XLOC_172121	ENSRNOT00000027611	0.952197831	1.81E-06
TUCP_003907	XLOC_172121	ENSRNOT00000028842	0.964583389	4.14E-07
TUCP_003907	XLOC_172121	ENSRNOT00000004003	0.976461749	5.47E-08
TUCP_003907	XLOC_172121	ENSRNOT00000036750	0.960369536	7.20E-07
TUCP_003907	XLOC_172121	ENSRNOT00000068322	0.960003064	7.54E-07
TUCP_003907	XLOC_172121	ENSRNOT00000052075	0.980396456	2.21E-08
TUCP_003907	XLOC_172121	ENSRNOT00000085648	0.952243847	1.81E-06
TUCP_003907	XLOC_172121	ENSRNOT00000037456	0.96637753	3.20E-07
TUCP_003907	XLOC_172121	ENSRNOT00000022947	-0.953847616	1.53E-06
TUCP_003907	XLOC_172121	ENSRNOT00000087095	0.966730251	3.04E-07
TUCP_003907	XLOC_172121	ENSRNOT00000032944	0.956649083	1.12E-06
TUCP_003907	XLOC_172121	ENSRNOT00000083215	0.971176575	1.49E-07
TUCP_003907	XLOC_172121	ENSRNOT00000026718	0.950465715	2.16E-06
TUCP_003907	XLOC_172121	ENSRNOT00000072208	0.953422755	1.60E-06
TUCP_003907	XLOC_172121	ENSRNOT00000032780	0.959685471	7.84E-07
TUCP_003907	XLOC_172121	ENSRNOT00000055669	0.950995979	2.05E-06
TUCP_003907	XLOC_172121	ENSRNOT00000022534	0.955917844	1.22E-06
TUCP_003907	XLOC_172121	ENSRNOT00000017319	0.978629949	3.39E-08
TUCP_003907	XLOC_172121	ENSRNOT00000055187	0.95942338	8.09E-07
TUCP_003907	XLOC_172121	ENSRNOT00000005934	0.976402928	5.54E-08
TUCP_003907	XLOC_172121	ENSRNOT00000001809	0.958884572	8.64E-07
TUCP_003907	XLOC_172121	ENSRNOT00000079866	0.964473081	4.20E-07
TUCP_003907	XLOC_172121	ENSRNOT00000072518	0.95662891	1.12E-06
TUCP_003907	XLOC_172121	ENSRNOT00000041513	0.962975456	5.15E-07
TUCP_003907	XLOC_172121	ENSRNOT00000034722	0.96521048	3.79E-07
TUCP_003907	XLOC_172121	ENSRNOT00000087592	0.95753635	1.01E-06
TUCP_003907	XLOC_172121	ENSRNOT00000067822	0.950163551	2.23E-06
TUCP_003907	XLOC_172121	ENSRNOT00000024060	0.974422973	8.26E-08
TUCP_003907	XLOC_172121	ENSRNOT00000008982	0.960021171	7.52E-07
TUCP_003907	XLOC_172121	ENSRNOT00000010149	0.973018002	1.08E-07
TUCP_003907	XLOC_172121	ENSRNOT00000034704	0.962588113	5.42E-07
TUCP_003907	XLOC_172121	ENSRNOT00000028760	0.951959462	1.86E-06
TUCP_003907	XLOC_172121	ENSRNOT00000024771	0.950679974	2.12E-06
TUCP_003907	XLOC_172121	ENSRNOT00000021037	0.958285354	9.27E-07
TUCP_003907	XLOC_172121	ENSRNOT00000064930	0.964196383	4.36E-07
TUCP_003907	XLOC_172121	ENSRNOT00000074725	0.953864023	1.52E-06
TUCP_003907	XLOC_172121	ENSRNOT00000019687	0.964418978	4.23E-07
TUCP_003907	XLOC_172121	ENSRNOT00000068633	0.966224377	3.27E-07
TUCP_003907	XLOC_172121	ENSRNOT00000015092	0.951921189	1.87E-06
TUCP_003907	XLOC_172121	ENSRNOT00000004032	0.965493209	3.64E-07
TUCP_003907	XLOC_172121	ENSRNOT00000084385	0.976953995	4.93E-08
TUCP_003907	XLOC_172121	ENSRNOT00000017948	0.977098646	4.77E-08
TUCP_003907	XLOC_172121	ENSRNOT00000012895	0.9812337	1.78E-08
TUCP_003907	XLOC_172121	ENSRNOT00000004508	0.953939451	1.51E-06
TUCP_003907	XLOC_172121	ENSRNOT00000023180	0.969096616	2.11E-07
TUCP_003907	XLOC_172121	ENSRNOT00000044562	0.956817408	1.10E-06
TUCP_003907	XLOC_172121	ENSRNOT00000057313	0.970273421	1.74E-07
TUCP_003907	XLOC_172121	ENSRNOT00000015116	0.956776406	1.10E-06
TUCP_003907	XLOC_172121	ENSRNOT00000090918	0.97708902	4.78E-08
TUCP_003907	XLOC_172121	ENSRNOT00000001081	0.955920784	1.22E-06
TUCP_003907	XLOC_172121	ENSRNOT00000039852	0.962805227	5.27E-07

TUCP_003907	XLOC_172121	ENSRNOT00000009440	-0.96602736	3.37E-07
TUCP_003907	XLOC_172121	ENSRNOT00000016948	-0.951203806	2.01E-06
TUCP_003907	XLOC_172121	ENSRNOT00000025983	0.987293888	2.55E-09
TUCP_003907	XLOC_172121	ENSRNOT00000000713	0.962074649	5.80E-07
TUCP_003907	XLOC_172121	ENSRNOT00000054874	0.964651074	4.10E-07
TUCP_003907	XLOC_172121	ENSRNOT00000028070	0.969195558	2.07E-07
TUCP_003907	XLOC_172121	ENSRNOT00000088196	0.967081993	2.88E-07
TUCP_003907	XLOC_172121	ENSRNOT00000014508	0.958372731	9.18E-07
TUCP_003907	XLOC_172121	ENSRNOT00000060362	0.962752581	5.30E-07
TUCP_003907	XLOC_172121	ENSRNOT00000065983	0.960952637	6.69E-07
TUCP_003907	XLOC_172121	ENSRNOT00000022213	0.981231636	1.78E-08
TUCP_003907	XLOC_172121	ENSRNOT00000022610	0.968858729	2.19E-07
TUCP_003907	XLOC_172121	ENSRNOT00000020437	0.991098175	4.34E-10
TUCP_003907	XLOC_172121	ENSRNOT00000006828	0.977307319	4.56E-08
TUCP_003907	XLOC_172121	ENSRNOT00000073346	0.96614478	3.31E-07
TUCP_003907	XLOC_172121	ENSRNOT00000010898	0.977510978	4.36E-08
TUCP_003907	XLOC_172121	ENSRNOT00000071493	-0.962801722	5.27E-07
TUCP_003907	XLOC_172121	ENSRNOT00000015265	0.969537577	1.96E-07
TUCP_003907	XLOC_172121	ENSRNOT00000078639	0.954492562	1.42E-06
TUCP_003907	XLOC_172121	ENSRNOT00000029833	0.963707654	4.67E-07
TUCP_003907	XLOC_172121	ENSRNOT00000003971	0.954311456	1.45E-06
TUCP_003907	XLOC_172121	ENSRNOT00000005355	0.972455195	1.19E-07
TUCP_003907	XLOC_172121	ENSRNOT00000048063	0.950912834	2.07E-06
TUCP_003907	XLOC_172121	ENSRNOT00000052422	0.958040436	9.55E-07
TUCP_003907	XLOC_172121	ENSRNOT00000001242	0.991153628	4.20E-10
TUCP_003907	XLOC_172121	ENSRNOT00000004026	0.954502641	1.42E-06
TUCP_003907	XLOC_172121	ENSRNOT00000078589	-0.961075706	6.59E-07
TUCP_003907	XLOC_172121	ENSRNOT00000083799	0.964548264	4.16E-07
TUCP_003907	XLOC_172121	ENSRNOT00000078102	0.977848478	4.05E-08
TUCP_003907	XLOC_172121	ENSRNOT00000070893	0.955269334	1.31E-06
TUCP_003907	XLOC_172121	ENSRNOT00000027520	0.957979774	9.61E-07
TUCP_003907	XLOC_172121	ENSRNOT00000010873	-0.950207385	2.22E-06
TUCP_003907	XLOC_172121	ENSRNOT00000007164	0.966065573	3.35E-07
TUCP_003907	XLOC_172121	ENSRNOT00000027526	0.954129541	1.48E-06
TUCP_003907	XLOC_172121	ENSRNOT00000026755	0.97227701	1.23E-07
TUCP_003907	XLOC_172121	ENSRNOT00000013661	0.951873777	1.88E-06
TUCP_003907	XLOC_172121	ENSRNOT00000016129	0.965700558	3.53E-07
TUCP_003907	XLOC_172121	ENSRNOT00000081047	0.968943142	2.16E-07
TUCP_003907	XLOC_172121	ENSRNOT00000084240	0.962205541	5.70E-07
TUCP_003907	XLOC_172121	ENSRNOT00000017629	0.95669175	1.12E-06
TUCP_003907	XLOC_172121	ENSRNOT00000023619	0.95102444	2.04E-06
TUCP_003907	XLOC_172121	ENSRNOT00000005911	0.972204718	1.25E-07
TUCP_003907	XLOC_172121	ENSRNOT00000022839	0.97429977	8.46E-08
TUCP_003907	XLOC_172121	ENSRNOT00000021408	0.960921747	6.72E-07
TUCP_003907	XLOC_172121	ENSRNOT00000021116	0.956258584	1.17E-06
TUCP_003907	XLOC_172121	ENSRNOT00000078475	0.957692534	9.94E-07
TUCP_003907	XLOC_172121	ENSRNOT00000087429	0.963044888	5.10E-07
TUCP_003907	XLOC_172121	ENSRNOT00000032158	0.956264827	1.17E-06
TUCP_003907	XLOC_172121	ENSRNOT00000009683	0.958106451	9.47E-07
TUCP_003907	XLOC_172121	ENSRNOT00000021153	0.974608824	7.97E-08
TUCP_003907	XLOC_172121	ENSRNOT00000080207	0.953884524	1.52E-06

TUCP_003907	XLOC_172121	ENSRNOT00000017776	0.962515692	5.47E-07
TUCP_003907	XLOC_172121	ENSRNOT00000064902	0.957214528	1.05E-06
TUCP_003907	XLOC_172121	ENSRNOT00000009280	0.953818463	1.53E-06
TUCP_003907	XLOC_172121	ENSRNOT00000064900	0.967968992	2.52E-07
TUCP_003907	XLOC_172121	ENSRNOT00000024420	0.953901653	1.52E-06
TUCP_003907	XLOC_172121	ENSRNOT00000005262	0.965379962	3.70E-07
TUCP_003907	XLOC_172121	ENSRNOT00000013274	0.961612073	6.16E-07
TUCP_003907	XLOC_172121	ENSRNOT00000021015	0.959921891	7.61E-07
TUCP_003907	XLOC_172121	ENSRNOT00000092029	0.971277513	1.47E-07
TUCP_003907	XLOC_172121	ENSRNOT00000024322	0.955647021	1.25E-06
TUCP_003907	XLOC_172121	ENSRNOT00000011408	0.955984897	1.21E-06
TUCP_003907	XLOC_172121	ENSRNOT00000016722	0.954746163	1.39E-06
TUCP_003907	XLOC_172121	ENSRNOT00000091265	0.964925207	3.94E-07
TUCP_003907	XLOC_172121	ENSRNOT00000010463	0.956704619	1.11E-06
TUCP_003907	XLOC_172121	ENSRNOT00000058362	0.950497716	2.15E-06
TUCP_003907	XLOC_172121	ENSRNOT00000015609	0.979724234	2.61E-08
TUCP_003907	XLOC_172121	ENSRNOT00000078390	-0.951362725	1.97E-06
TUCP_003907	XLOC_172121	ENSRNOT00000026457	0.964545263	4.16E-07
TUCP_003907	XLOC_172121	ENSRNOT00000090170	0.955470013	1.28E-06
TUCP_003907	XLOC_172121	ENSRNOT00000027454	0.950067531	2.25E-06
TUCP_003907	XLOC_172121	ENSRNOT00000035989	0.950863155	2.08E-06
TUCP_003907	XLOC_172121	ENSRNOT00000003690	0.959047442	8.47E-07
TUCP_003907	XLOC_172121	ENSRNOT00000076729	0.951533188	1.94E-06
TUCP_003907	XLOC_172121	ENSRNOT00000049538	0.984191184	7.57E-09
TUCP_003907	XLOC_172121	ENSRNOT00000051022	0.971127777	1.51E-07
TUCP_003907	XLOC_172121	ENSRNOT00000006499	0.960208992	7.35E-07
TUCP_003907	XLOC_172121	ENSRNOT00000073337	0.951524479	1.94E-06
TUCP_003907	XLOC_172121	ENSRNOT00000087725	0.975912518	6.13E-08
TUCP_003907	XLOC_172121	ENSRNOT00000066736	0.958228767	9.34E-07
TUCP_003907	XLOC_172121	ENSRNOT00000025936	-0.962383462	5.57E-07
TUCP_003907	XLOC_172121	ENSRNOT00000009198	0.972020383	1.29E-07
TUCP_003907	XLOC_172121	ENSRNOT00000009129	0.981998835	1.44E-08
TUCP_003907	XLOC_172121	ENSRNOT00000036951	0.99090081	4.84E-10
TUCP_003907	XLOC_172121	ENSRNOT00000083369	0.961400797	6.32E-07
TUCP_003907	XLOC_172121	ENSRNOT00000016519	0.955404683	1.29E-06
TUCP_003907	XLOC_172121	ENSRNOT00000026186	0.969552449	1.96E-07
TUCP_003907	XLOC_172121	ENSRNOT00000002173	-0.964968363	3.92E-07
TUCP_003907	XLOC_172121	ENSRNOT00000013762	0.959840255	7.69E-07
TUCP_003907	XLOC_172121	ENSRNOT00000044177	0.959809759	7.72E-07
TUCP_003907	XLOC_172121	ENSRNOT00000030229	0.950538121	2.15E-06
TUCP_003907	XLOC_172121	ENSRNOT00000087697	0.952595475	1.74E-06
TUCP_003907	XLOC_172121	ENSRNOT00000011663	0.979140033	3.00E-08
TUCP_003907	XLOC_172121	ENSRNOT00000043953	0.97980184	2.56E-08
TUCP_003907	XLOC_172121	ENSRNOT00000085228	0.962127017	5.76E-07
TUCP_003907	XLOC_172121	ENSRNOT00000072081	0.953958259	1.51E-06
TUCP_003907	XLOC_172121	ENSRNOT00000019554	0.954749227	1.38E-06
TUCP_003907	XLOC_172121	ENSRNOT00000058842	0.961824224	5.99E-07
TUCP_003907	XLOC_172121	ENSRNOT00000023645	0.978457689	3.52E-08
TUCP_003907	XLOC_172121	ENSRNOT00000019553	0.957390036	1.03E-06
TUCP_003907	XLOC_172121	ENSRNOT00000084734	0.978812459	3.25E-08
TUCP_003907	XLOC_172121	ENSRNOT00000023330	0.968589546	2.28E-07

TUCP_003907	XLOC_172121	ENSRNOT00000091128	-0.963580569	4.75E-07
TUCP_003907	XLOC_172121	ENSRNOT00000035700	0.976431427	5.51E-08
TUCP_003907	XLOC_172121	ENSRNOT00000038247	0.973244643	1.03E-07
TUCP_003907	XLOC_172121	ENSRNOT00000083255	0.962307035	5.62E-07
TUCP_003907	XLOC_172121	ENSRNOT00000029136	-0.959509036	8.01E-07
TUCP_003907	XLOC_172121	ENSRNOT00000064840	0.970308491	1.73E-07
TUCP_003907	XLOC_172121	ENSRNOT00000020379	0.957718896	9.91E-07
TUCP_003907	XLOC_172121	ENSRNOT00000076669	0.955904911	1.22E-06
TUCP_003907	XLOC_172121	ENSRNOT00000085596	0.964479074	4.20E-07
TUCP_003907	XLOC_172121	ENSRNOT00000016333	-0.95275132	1.71E-06
TUCP_003907	XLOC_172121	ENSRNOT00000016991	0.956447784	1.15E-06
TUCP_003907	XLOC_172121	ENSRNOT00000090925	0.974560965	8.04E-08
TUCP_003907	XLOC_172121	ENSRNOT00000007175	0.966524683	3.13E-07
TUCP_003907	XLOC_172121	ENSRNOT00000025394	0.976644963	5.26E-08
TUCP_003907	XLOC_172121	ENSRNOT00000043872	0.956738485	1.11E-06
TUCP_003907	XLOC_172121	ENSRNOT00000074653	0.96416787	4.38E-07
TUCP_003907	XLOC_172121	ENSRNOT00000025391	0.954675145	1.40E-06
TUCP_003907	XLOC_172121	ENSRNOT00000054828	0.961035934	6.62E-07
TUCP_003907	XLOC_172121	ENSRNOT00000031230	0.973227199	1.04E-07
TUCP_003907	XLOC_172121	ENSRNOT00000014887	0.957727654	9.90E-07
TUCP_003907	XLOC_172121	ENSRNOT00000059458	0.955317612	1.30E-06
TUCP_003907	XLOC_172121	ENSRNOT00000074659	0.956363952	1.16E-06
TUCP_003907	XLOC_172121	ENSRNOT00000013758	0.951265395	1.99E-06
TUCP_003907	XLOC_172121	ENSRNOT00000077406	0.976151359	5.84E-08
TUCP_003907	XLOC_172121	ENSRNOT00000040069	0.962714046	5.33E-07
TUCP_003907	XLOC_172121	ENSRNOT00000010748	0.957492034	1.02E-06
TUCP_003907	XLOC_172121	ENSRNOT00000005478	-0.950942047	2.06E-06
TUCP_003907	XLOC_172121	ENSRNOT00000046058	0.958968146	8.55E-07
TUCP_003907	XLOC_172121	ENSRNOT00000024149	0.970976312	1.54E-07
TUCP_003907	XLOC_172121	ENSRNOT00000035474	0.97560161	6.54E-08
TUCP_003907	XLOC_172121	ENSRNOT00000026087	-0.954216289	1.47E-06
TUCP_003907	XLOC_172121	ENSRNOT00000077311	0.961505481	6.24E-07
TUCP_003907	XLOC_172121	ENSRNOT00000086336	0.986922942	2.95E-09
TUCP_003907	XLOC_172121	ENSRNOT00000008929	0.965247218	3.77E-07
TUCP_003907	XLOC_172121	ENSRNOT00000006195	0.990348506	6.49E-10
TUCP_003907	XLOC_172121	ENSRNOT00000033235	0.96249206	5.49E-07
TUCP_003907	XLOC_172121	ENSRNOT00000021285	0.950515875	2.15E-06
TUCP_003907	XLOC_172121	ENSRNOT00000056130	0.955015421	1.35E-06
TUCP_003907	XLOC_172121	ENSRNOT00000076872	0.95697813	1.08E-06
TUCP_003907	XLOC_172121	ENSRNOT00000080800	0.960973087	6.68E-07
TUCP_003907	XLOC_172121	ENSRNOT00000027910	0.955457118	1.28E-06
TUCP_003907	XLOC_172121	ENSRNOT00000019769	-0.950510691	2.15E-06
TUCP_003907	XLOC_172121	ENSRNOT00000066670	0.974088575	8.81E-08
TUCP_003907	XLOC_172121	ENSRNOT00000065921	0.957002778	1.08E-06
TUCP_003907	XLOC_172121	ENSRNOT00000029125	0.960984068	6.67E-07
TUCP_003907	XLOC_172121	ENSRNOT00000082174	0.968918521	2.17E-07
TUCP_003907	XLOC_172121	ENSRNOT00000010218	0.966540532	3.12E-07
TUCP_003907	XLOC_172121	ENSRNOT00000020322	0.961144336	6.53E-07
TUCP_003907	XLOC_172121	ENSRNOT00000010824	0.955879221	1.22E-06
TUCP_003907	XLOC_172121	ENSRNOT00000014814	0.963526156	4.78E-07
TUCP_003907	XLOC_172121	ENSRNOT00000004412	0.980900169	1.94E-08

TUCP_003907	XLOC_172121	ENSRNOT00000007768	0.971215663	1.48E-07
TUCP_003907	XLOC_172121	ENSRNOT00000067181	0.961967745	5.88E-07
TUCP_003907	XLOC_172121	ENSRNOT00000064798	0.980200203	2.32E-08
TUCP_003907	XLOC_172121	ENSRNOT00000019177	0.985784959	4.46E-09
TUCP_003907	XLOC_172121	ENSRNOT00000014261	0.963477254	4.81E-07
TUCP_003907	XLOC_172121	ENSRNOT00000022485	0.984365467	7.17E-09
TUCP_003907	XLOC_172121	ENSRNOT00000010927	0.981398999	1.70E-08
TUCP_003907	XLOC_172121	ENSRNOT00000084595	0.965868805	3.44E-07
TUCP_003907	XLOC_172121	ENSRNOT00000025832	0.971782047	1.34E-07
TUCP_003907	XLOC_172121	ENSRNOT00000043535	0.959687754	7.83E-07
TUCP_003907	XLOC_172121	ENSRNOT00000040393	0.96039455	7.18E-07
TUCP_003907	XLOC_172121	ENSRNOT00000066593	0.95666679	1.12E-06
TUCP_003907	XLOC_172121	ENSRNOT00000012187	0.976929616	4.95E-08
TUCP_003907	XLOC_172121	ENSRNOT00000011682	-0.960211056	7.35E-07
TUCP_003907	XLOC_172121	ENSRNOT00000004463	0.959270817	8.24E-07
TUCP_003907	XLOC_172121	ENSRNOT00000027964	0.972751927	1.13E-07
TUCP_003907	XLOC_172121	ENSRNOT00000024659	0.958321272	9.23E-07
TUCP_003907	XLOC_172121	ENSRNOT00000023428	0.962384247	5.57E-07
TUCP_003907	XLOC_172121	ENSRNOT00000051550	0.955567596	1.27E-06
TUCP_003907	XLOC_172121	ENSRNOT00000011041	0.951095964	2.03E-06
TUCP_003907	XLOC_172121	ENSRNOT00000047192	-0.954068478	1.49E-06
TUCP_003907	XLOC_172121	ENSRNOT00000017753	0.973649677	9.57E-08
TUCP_003907	XLOC_172121	ENSRNOT00000079996	0.968920856	2.17E-07
TUCP_003907	XLOC_172121	ENSRNOT00000002292	0.972189589	1.25E-07
TUCP_003907	XLOC_172121	ENSRNOT00000018533	0.975265917	6.99E-08
TUCP_003907	XLOC_172121	ENSRNOT00000080110	0.962301255	5.63E-07
TUCP_003907	XLOC_172121	ENSRNOT00000048029	0.967147115	2.85E-07
TUCP_003907	XLOC_172121	ENSRNOT00000004140	0.965115478	3.84E-07
TUCP_003907	XLOC_172121	ENSRNOT00000010433	0.967828875	2.57E-07
TUCP_003907	XLOC_172121	ENSRNOT00000003991	0.971704103	1.36E-07
TUCP_003907	XLOC_172121	ENSRNOT00000093174	0.95522109	1.32E-06
TUCP_003907	XLOC_172121	ENSRNOT00000034744	0.970348253	1.72E-07
TUCP_003907	XLOC_172121	ENSRNOT00000024548	0.952332313	1.79E-06
TUCP_003170	XLOC_136254	ENSRNOT00000084616	-0.964054578	4.45E-07
TUCP_003170	XLOC_136254	ENSRNOT00000067500	0.957541783	1.01E-06
TUCP_004120	XLOC_180256	ENSRNOT00000068405	0.952502926	1.76E-06
TUCP_004120	XLOC_180256	ENSRNOT00000039809	0.979523475	2.74E-08
TUCP_004120	XLOC_180256	ENSRNOT00000000743	0.955324016	1.30E-06
TUCP_004120	XLOC_180256	ENSRNOT00000019213	0.952474642	1.76E-06
TUCP_004120	XLOC_180256	ENSRNOT00000009877	0.95309658	1.65E-06
TUCP_004120	XLOC_180256	ENSRNOT00000007351	-0.950049087	2.25E-06
TUCP_004120	XLOC_180256	ENSRNOT00000074939	0.975951422	6.08E-08
TUCP_004120	XLOC_180256	ENSRNOT00000071560	0.957020505	1.07E-06
TUCP_004120	XLOC_180256	ENSRNOT00000078445	-0.985886519	4.31E-09
TUCP_004120	XLOC_180256	ENSRNOT00000000184	-0.953797477	1.53E-06
TUCP_004120	XLOC_180256	ENSRNOT00000005228	0.962270792	5.65E-07
TUCP_004120	XLOC_180256	ENSRNOT00000023144	0.954001002	1.50E-06
TUCP_004120	XLOC_180256	ENSRNOT00000021116	0.972996384	1.08E-07
TUCP_004120	XLOC_180256	ENSRNOT00000082100	0.959990751	7.55E-07
TUCP_004120	XLOC_180256	ENSRNOT00000028646	0.95886282	8.66E-07
TUCP_004120	XLOC_180256	ENSRNOT00000043216	0.962966473	5.16E-07

TUCP_004120	XLOC_180256	ENSRNOT00000091128	-0.962602595	5.41E-07
TUCP_004120	XLOC_180256	ENSRNOT00000009280	0.966123339	3.32E-07
TUCP_004120	XLOC_180256	ENSRNOT00000028563	0.953211745	1.63E-06
TUCP_004120	XLOC_180256	ENSRNOT00000015076	0.96122892	6.46E-07
TUCP_004120	XLOC_180256	ENSRNOT00000022800	0.952250844	1.80E-06
TUCP_004120	XLOC_180256	ENSRNOT00000066670	0.964531448	4.17E-07
TUCP_004120	XLOC_180256	ENSRNOT00000076516	0.950521851	2.15E-06
TUCP_004120	XLOC_180256	ENSRNOT00000015073	0.974910202	7.51E-08
TUCP_004120	XLOC_180256	ENSRNOT00000025689	0.953286742	1.62E-06
TUCP_004120	XLOC_180256	ENSRNOT00000018533	0.950766569	2.10E-06
TUCP_004120	XLOC_180256	ENSRNOT00000025832	0.983484538	9.41E-09
TUCP_004120	XLOC_180256	ENSRNOT00000013046	-0.953704816	1.55E-06
TUCP_004120	XLOC_180256	ENSRNOT00000006055	0.970375677	1.71E-07
TUCP_004120	XLOC_180256	ENSRNOT00000071573	0.977511239	4.36E-08
TUCP_004120	XLOC_180256	ENSRNOT00000005126	-0.969784764	1.89E-07
TUCP_004120	XLOC_180256	ENSRNOT00000065300	0.950647572	2.12E-06
TUCP_004120	XLOC_180256	ENSRNOT00000011384	0.967860057	2.56E-07
TUCP_004120	XLOC_180256	ENSRNOT00000038247	0.952009017	1.85E-06
TUCP_004120	XLOC_180256	ENSRNOT00000087306	0.955703479	1.25E-06
TUCP_004120	XLOC_180256	ENSRNOT00000071764	0.96711502	2.87E-07
TUCP_004120	XLOC_180256	ENSRNOT00000035989	0.967958921	2.52E-07
TUCP_004120	XLOC_180256	ENSRNOT00000027944	0.959434376	8.08E-07
TUCP_004120	XLOC_180256	ENSRNOT00000021348	0.973025791	1.07E-07
TUCP_004120	XLOC_180256	ENSRNOT00000024406	-0.961393396	6.33E-07
TUCP_004120	XLOC_180256	ENSRNOT00000043156	0.963490526	4.80E-07
TUCP_004120	XLOC_180256	ENSRNOT00000012671	0.957260152	1.05E-06
TUCP_004120	XLOC_180256	ENSRNOT00000083215	0.968290551	2.39E-07
TUCP_004120	XLOC_180256	ENSRNOT00000083127	0.950189641	2.22E-06
TUCP_004120	XLOC_180256	ENSRNOT00000055187	0.96211331	5.77E-07
TUCP_004120	XLOC_180256	ENSRNOT00000001809	0.952107156	1.83E-06
TUCP_004120	XLOC_180256	ENSRNOT00000078500	0.977265537	4.60E-08
TUCP_004120	XLOC_180256	ENSRNOT00000087592	0.955625812	1.26E-06
TUCP_004120	XLOC_180256	ENSRNOT00000067822	0.95022312	2.21E-06
TUCP_004120	XLOC_180256	ENSRNOT00000032274	0.970455087	1.69E-07
TUCP_004120	XLOC_180256	ENSRNOT00000082687	-0.966086163	3.34E-07
TUCP_004120	XLOC_180256	ENSRNOT00000011784	0.955757072	1.24E-06
TUCP_004120	XLOC_180256	ENSRNOT00000068633	0.95814635	9.43E-07
TUCP_004120	XLOC_180256	ENSRNOT00000037446	0.951529747	1.94E-06
TUCP_004120	XLOC_180256	ENSRNOT00000017948	0.978992668	3.11E-08
TUCP_004120	XLOC_180256	ENSRNOT00000028842	0.959228909	8.28E-07
TUCP_004120	XLOC_180256	ENSRNOT00000012895	0.958305517	9.25E-07
TUCP_004120	XLOC_180256	ENSRNOT00000013570	-0.968324958	2.38E-07
TUCP_004120	XLOC_180256	ENSRNOT00000025859	0.95675969	1.11E-06
TUCP_004120	XLOC_180256	ENSRNOT00000023180	0.959738815	7.79E-07
TUCP_004120	XLOC_180256	ENSRNOT00000000676	0.960630638	6.97E-07
TUCP_004120	XLOC_180256	ENSRNOT00000004359	0.957877894	9.73E-07
TUCP_004120	XLOC_180256	ENSRNOT00000060362	0.95975826	7.77E-07
TUCP_004120	XLOC_180256	ENSRNOT00000013274	0.965086266	3.85E-07
TUCP_004120	XLOC_180256	ENSRNOT00000022610	0.982931426	1.11E-08
TUCP_004120	XLOC_180256	ENSRNOT00000020437	0.970929376	1.56E-07
TUCP_004120	XLOC_180256	ENSRNOT00000006828	0.971906638	1.31E-07

TUCP_004120	XLOC_180256	ENSRNOT00000073346	0.957444753	1.02E-06
TUCP_004120	XLOC_180256	ENSRNOT00000010898	0.975814882	6.26E-08
TUCP_004120	XLOC_180256	ENSRNOT00000001222	0.95311991	1.65E-06
TUCP_004120	XLOC_180256	ENSRNOT00000067181	0.959073961	8.44E-07
TUCP_004120	XLOC_180256	ENSRNOT00000005355	0.977329627	4.54E-08
TUCP_004120	XLOC_180256	ENSRNOT00000080207	0.951567105	1.93E-06
TUCP_004120	XLOC_180256	ENSRNOT00000025981	0.952395269	1.78E-06
TUCP_004120	XLOC_180256	ENSRNOT00000027520	0.97544047	6.75E-08
TUCP_004120	XLOC_180256	ENSRNOT00000025983	0.957966817	9.63E-07
TUCP_004120	XLOC_180256	ENSRNOT00000047387	0.969452727	1.99E-07
TUCP_004120	XLOC_180256	ENSRNOT00000004698	0.968653906	2.26E-07
TUCP_004120	XLOC_180256	ENSRNOT00000012093	0.961134205	6.54E-07
TUCP_004120	XLOC_180256	ENSRNOT00000058072	0.95226409	1.80E-06
TUCP_004120	XLOC_180256	ENSRNOT00000084240	0.966831579	2.99E-07
TUCP_004120	XLOC_180256	ENSRNOT00000017629	0.977148153	4.72E-08
TUCP_004120	XLOC_180256	ENSRNOT00000032199	0.961918408	5.92E-07
TUCP_004120	XLOC_180256	ENSRNOT00000003991	0.965245214	3.77E-07
TUCP_004120	XLOC_180256	ENSRNOT00000004508	0.955941642	1.21E-06
TUCP_004120	XLOC_180256	ENSRNOT00000009683	0.961427023	6.30E-07
TUCP_004120	XLOC_180256	ENSRNOT00000021153	0.951249755	2.00E-06
TUCP_004120	XLOC_180256	ENSRNOT00000084088	0.966580321	3.10E-07
TUCP_004120	XLOC_180256	ENSRNOT00000064902	0.955988734	1.21E-06
TUCP_004120	XLOC_180256	ENSRNOT00000088995	0.958587809	8.95E-07
TUCP_004120	XLOC_180256	ENSRNOT00000024420	0.986932131	2.94E-09
TUCP_004120	XLOC_180256	ENSRNOT00000029664	0.956582049	1.13E-06
TUCP_004120	XLOC_180256	ENSRNOT00000076729	0.973599327	9.66E-08
TUCP_004120	XLOC_180256	ENSRNOT00000085596	0.974773395	7.71E-08
TUCP_004120	XLOC_180256	ENSRNOT00000091265	0.961193365	6.49E-07
TUCP_004120	XLOC_180256	ENSRNOT00000030943	-0.961269639	6.43E-07
TUCP_004120	XLOC_180256	ENSRNOT00000026457	0.958706121	8.82E-07
TUCP_004120	XLOC_180256	ENSRNOT00000027452	0.953199235	1.63E-06
TUCP_004120	XLOC_180256	ENSRNOT00000027615	0.957529078	1.01E-06
TUCP_004120	XLOC_180256	ENSRNOT00000005909	0.954020737	1.50E-06
TUCP_004120	XLOC_180256	ENSRNOT00000066736	0.950725529	2.11E-06
TUCP_004120	XLOC_180256	ENSRNOT00000025936	-0.951062449	2.04E-06
TUCP_004120	XLOC_180256	ENSRNOT00000012702	-0.952953051	1.68E-06
TUCP_004120	XLOC_180256	ENSRNOT00000009198	0.956437915	1.15E-06
TUCP_004120	XLOC_180256	ENSRNOT00000025092	0.965184048	3.80E-07
TUCP_004120	XLOC_180256	ENSRNOT00000009129	0.98474737	6.34E-09
TUCP_004120	XLOC_180256	ENSRNOT00000050138	0.951291176	1.99E-06
TUCP_004120	XLOC_180256	ENSRNOT00000072081	0.961110514	6.56E-07
TUCP_004120	XLOC_180256	ENSRNOT00000036951	0.96308096	5.08E-07
TUCP_004120	XLOC_180256	ENSRNOT00000090376	0.958189624	9.38E-07
TUCP_004120	XLOC_180256	ENSRNOT00000090420	-0.964916647	3.95E-07
TUCP_004120	XLOC_180256	ENSRNOT00000081897	0.954939731	1.36E-06
TUCP_004120	XLOC_180256	ENSRNOT00000057313	0.983861928	8.39E-09
TUCP_004120	XLOC_180256	ENSRNOT00000073930	0.964484126	4.19E-07
TUCP_004120	XLOC_180256	ENSRNOT00000000157	0.959533829	7.98E-07
TUCP_004120	XLOC_180256	ENSRNOT00000010992	0.972644054	1.15E-07
TUCP_004120	XLOC_180256	ENSRNOT00000076669	0.98107889	1.85E-08
TUCP_004120	XLOC_180256	ENSRNOT00000011984	0.954594607	1.41E-06

TUCP_004120	XLOC_180256	ENSRNOT00000074653	0.954649342	1.40E-06
TUCP_004120	XLOC_180256	ENSRNOT00000031230	0.975024762	7.34E-08
TUCP_004120	XLOC_180256	ENSRNOT00000059458	0.989168796	1.15E-09
TUCP_004120	XLOC_180256	ENSRNOT00000055992	-0.951364688	1.97E-06
TUCP_004120	XLOC_180256	ENSRNOT00000008184	0.958085936	9.49E-07
TUCP_004120	XLOC_180256	ENSRNOT00000052288	0.977920892	3.98E-08
TUCP_004120	XLOC_180256	ENSRNOT00000026543	0.971596129	1.39E-07
TUCP_004120	XLOC_180256	ENSRNOT00000068322	0.972676584	1.15E-07
TUCP_004120	XLOC_180256	ENSRNOT00000080800	0.954156565	1.48E-06
TUCP_004120	XLOC_180256	ENSRNOT00000003296	0.976547887	5.37E-08
TUCP_004120	XLOC_180256	ENSRNOT00000058852	-0.966420842	3.18E-07
TUCP_004120	XLOC_180256	ENSRNOT00000019810	-0.950281639	2.20E-06
TUCP_004120	XLOC_180256	ENSRNOT00000071694	0.967703611	2.62E-07
TUCP_004120	XLOC_180256	ENSRNOT00000014814	0.955883426	1.22E-06
TUCP_004120	XLOC_180256	ENSRNOT00000004412	0.983981716	8.09E-09
TUCP_004120	XLOC_180256	ENSRNOT00000004942	-0.957102215	1.06E-06
TUCP_004120	XLOC_180256	ENSRNOT00000064798	0.956502026	1.14E-06
TUCP_004120	XLOC_180256	ENSRNOT00000019177	0.952510385	1.76E-06
TUCP_004120	XLOC_180256	ENSRNOT00000010927	0.952368718	1.78E-06
TUCP_004120	XLOC_180256	ENSRNOT00000024941	0.955890518	1.22E-06
TUCP_004120	XLOC_180256	ENSRNOT00000034704	0.976016315	6.00E-08
TUCP_004120	XLOC_180256	ENSRNOT00000013031	-0.956624976	1.12E-06
TUCP_004120	XLOC_180256	ENSRNOT00000036750	0.966734878	3.03E-07
TUCP_004120	XLOC_180256	ENSRNOT00000027964	0.973388528	1.01E-07
TUCP_004120	XLOC_180256	ENSRNOT00000023428	0.952153217	1.82E-06
TUCP_004120	XLOC_180256	ENSRNOT00000051550	0.974799353	7.67E-08
TUCP_004120	XLOC_180256	ENSRNOT00000049538	0.960427525	7.15E-07
TUCP_004120	XLOC_180256	ENSRNOT00000002292	0.951653015	1.92E-06
TUCP_004120	XLOC_180256	ENSRNOT00000012463	-0.962259741	5.66E-07
TUCP_004120	XLOC_180256	ENSRNOT00000013661	0.957269228	1.04E-06
TUCP_004120	XLOC_180256	ENSRNOT00000083799	0.976033664	5.98E-08
TUCP_004120	XLOC_180256	ENSRNOT00000009066	0.965010524	3.89E-07
TUCP_003165	XLOC_135900	ENSRNOT00000031360	0.962691592	5.35E-07
TUCP_003165	XLOC_135900	ENSRNOT00000042495	0.952302132	1.79E-06
TUCP_003165	XLOC_135900	ENSRNOT00000079494	0.956691588	1.12E-06
TUCP_001595	XLOC_062340	ENSRNOT00000079464	0.954535152	1.42E-06
TUCP_001595	XLOC_062340	ENSRNOT00000028458	0.951472013	1.95E-06
TUCP_004358	XLOC_192367	ENSRNOT00000072077	0.954985462	1.35E-06
TUCP_004358	XLOC_192367	ENSRNOT00000011344	0.961346814	6.37E-07
TUCP_004358	XLOC_192367	ENSRNOT00000026361	0.969578168	1.95E-07
TUCP_004358	XLOC_192367	ENSRNOT00000092196	0.954817503	1.37E-06
TUCP_004358	XLOC_192367	ENSRNOT00000018674	0.950569814	2.14E-06
TUCP_004359	XLOC_192368	ENSRNOT00000011344	0.95582755	1.23E-06
TUCP_004359	XLOC_192368	ENSRNOT00000026361	0.959273718	8.24E-07
TUCP_004359	XLOC_192368	ENSRNOT00000092196	0.954150726	1.48E-06
TUCP_004608	XLOC_203040	ENSRNOT00000007698	-0.958825132	8.70E-07
TUCP_004608	XLOC_203040	ENSRNOT00000006188	-0.950285517	2.20E-06
TUCP_004608	XLOC_203040	ENSRNOT00000081796	0.959029908	8.49E-07
TUCP_004608	XLOC_203040	ENSRNOT00000090621	0.967265338	2.80E-07
TUCP_004608	XLOC_203040	ENSRNOT00000016088	0.961787998	6.02E-07
TUCP_004608	XLOC_203040	ENSRNOT00000014780	-0.951446628	1.96E-06

TUCP_004608	XLOC_203040	ENSRNOT00000075397	0.953762838	1.54E-06
TUCP_004608	XLOC_203040	ENSRNOT00000007350	0.96522762	3.78E-07
TUCP_004608	XLOC_203040	ENSRNOT00000020051	0.95670782	1.11E-06
TUCP_004608	XLOC_203040	ENSRNOT00000009080	-0.953743151	1.54E-06
TUCP_004608	XLOC_203040	ENSRNOT00000063930	-0.961561892	6.19E-07
TUCP_004608	XLOC_203040	ENSRNOT00000088202	-0.956873598	1.09E-06
TUCP_004608	XLOC_203040	ENSRNOT00000087062	0.973897746	9.13E-08
TUCP_004608	XLOC_203040	ENSRNOT00000000181	0.979387818	2.83E-08
TUCP_004608	XLOC_203040	ENSRNOT00000015678	-0.960819763	6.81E-07
TUCP_004608	XLOC_203040	ENSRNOT00000048613	0.952498289	1.76E-06
TUCP_004608	XLOC_203040	ENSRNOT00000041237	-0.951860765	1.88E-06
TUCP_004608	XLOC_203040	ENSRNOT00000023430	-0.953228392	1.63E-06
TUCP_004608	XLOC_203040	ENSRNOT00000079138	0.961376934	6.34E-07
TUCP_004608	XLOC_203040	ENSRNOT00000078737	-0.957873761	9.73E-07
TUCP_004608	XLOC_203040	ENSRNOT00000076916	0.956544688	1.13E-06
TUCP_004608	XLOC_203040	ENSRNOT00000045833	0.976282458	5.68E-08
TUCP_004608	XLOC_203040	ENSRNOT00000008074	-0.950513571	2.15E-06
TUCP_004608	XLOC_203040	ENSRNOT00000074683	0.954833639	1.37E-06
TUCP_004608	XLOC_203040	ENSRNOT00000009073	-0.95424517	1.46E-06
TUCP_004608	XLOC_203040	ENSRNOT00000019180	-0.950244315	2.21E-06
TUCP_004608	XLOC_203040	ENSRNOT00000015318	-0.952919658	1.68E-06
TUCP_004608	XLOC_203040	ENSRNOT00000013613	-0.957790807	9.83E-07
TUCP_004608	XLOC_203040	ENSRNOT00000006752	0.975765181	6.32E-08
TUCP_004608	XLOC_203040	ENSRNOT00000040847	-0.950369976	2.18E-06
TUCP_004608	XLOC_203040	ENSRNOT00000031672	-0.959503708	8.01E-07
TUCP_004608	XLOC_203040	ENSRNOT00000002028	0.955832377	1.23E-06
TUCP_004608	XLOC_203040	ENSRNOT00000025284	-0.950922729	2.06E-06
TUCP_004608	XLOC_203040	ENSRNOT00000016306	-0.952898549	1.69E-06
TUCP_004608	XLOC_203040	ENSRNOT00000023037	0.977669636	4.21E-08
TUCP_004608	XLOC_203040	ENSRNOT00000087604	0.961727501	6.06E-07
TUCP_004608	XLOC_203040	ENSRNOT00000057953	0.95619467	1.18E-06
TUCP_004608	XLOC_203040	ENSRNOT00000020207	0.952700713	1.72E-06
TUCP_004608	XLOC_203040	ENSRNOT00000083568	0.953824086	1.53E-06
TUCP_004608	XLOC_203040	ENSRNOT00000019921	0.970164702	1.77E-07
TUCP_004608	XLOC_203040	ENSRNOT00000065978	0.975551043	6.60E-08
TUCP_004608	XLOC_203040	ENSRNOT00000077060	0.957000765	1.08E-06
TUCP_004608	XLOC_203040	ENSRNOT00000005620	-0.950134193	2.23E-06
TUCP_004608	XLOC_203040	ENSRNOT00000004956	0.952607831	1.74E-06
TUCP_004608	XLOC_203040	ENSRNOT00000061580	-0.952274567	1.80E-06
TUCP_004608	XLOC_203040	ENSRNOT00000092068	0.975954727	6.08E-08
TUCP_004608	XLOC_203040	ENSRNOT00000087572	-0.952112316	1.83E-06
TUCP_004608	XLOC_203040	ENSRNOT00000027760	0.952072999	1.84E-06
TUCP_004608	XLOC_203040	ENSRNOT00000065045	0.953898839	1.52E-06
TUCP_004608	XLOC_203040	ENSRNOT00000007824	-0.954774266	1.38E-06
TUCP_004608	XLOC_203040	ENSRNOT00000025819	-0.951092556	2.03E-06
TUCP_004608	XLOC_203040	ENSRNOT00000057898	-0.950695549	2.11E-06
TUCP_004608	XLOC_203040	ENSRNOT00000024677	0.956745708	1.11E-06
TUCP_004608	XLOC_203040	ENSRNOT00000019519	0.960382274	7.19E-07
TUCP_004608	XLOC_203040	ENSRNOT00000019192	-0.955170532	1.32E-06
TUCP_004608	XLOC_203040	ENSRNOT00000067626	-0.956941338	1.08E-06
TUCP_004608	XLOC_203040	ENSRNOT00000081725	0.954513693	1.42E-06

TUCP_004608	XLOC_203040	ENSRNOT00000026550	0.955857825	1.23E-06
TUCP_004608	XLOC_203040	ENSRNOT00000010783	-0.957508373	1.02E-06
TUCP_004608	XLOC_203040	ENSRNOT00000026726	-0.957800093	9.82E-07
TUCP_004608	XLOC_203040	ENSRNOT00000081226	0.957102789	1.06E-06
TUCP_004608	XLOC_203040	ENSRNOT00000013286	0.975407518	6.80E-08
TUCP_004608	XLOC_203040	ENSRNOT00000011948	-0.953652244	1.56E-06
TUCP_004608	XLOC_203040	ENSRNOT00000018488	-0.95151216	1.95E-06
TUCP_004608	XLOC_203040	ENSRNOT00000034282	0.970070466	1.80E-07
TUCP_004608	XLOC_203040	ENSRNOT00000005686	-0.950131412	2.23E-06
TUCP_004608	XLOC_203040	ENSRNOT00000072762	-0.9597351	7.79E-07
TUCP_004608	XLOC_203040	ENSRNOT00000018952	0.956358343	1.16E-06
TUCP_004608	XLOC_203040	ENSRNOT00000019820	0.950928004	2.06E-06
TUCP_004608	XLOC_203040	ENSRNOT00000066708	0.980568413	2.11E-08
TUCP_004608	XLOC_203040	ENSRNOT00000016389	-0.951193369	2.01E-06
TUCP_004608	XLOC_203040	ENSRNOT00000073860	-0.965470667	3.65E-07
TUCP_004608	XLOC_203040	ENSRNOT00000016629	0.953993614	1.50E-06
TUCP_004608	XLOC_203040	ENSRNOT00000011832	-0.968210348	2.42E-07
TUCP_004608	XLOC_203040	ENSRNOT00000073643	-0.964224467	4.35E-07
TUCP_004608	XLOC_203040	ENSRNOT00000030074	0.967677192	2.63E-07
TUCP_004608	XLOC_203040	ENSRNOT00000001979	-0.96329911	4.93E-07
TUCP_004608	XLOC_203040	ENSRNOT00000028089	0.966643314	3.08E-07
TUCP_004608	XLOC_203040	ENSRNOT00000083968	-0.95346401	1.59E-06
TUCP_004608	XLOC_203040	ENSRNOT00000033806	0.969299229	2.04E-07
TUCP_004608	XLOC_203040	ENSRNOT00000083578	0.950193847	2.22E-06
TUCP_004608	XLOC_203040	ENSRNOT00000009150	0.978571906	3.43E-08
TUCP_004608	XLOC_203040	ENSRNOT00000026920	-0.96728312	2.79E-07
TUCP_004608	XLOC_203040	ENSRNOT00000084709	0.955749348	1.24E-06
TUCP_004608	XLOC_203040	ENSRNOT00000083967	0.952123403	1.83E-06
TUCP_004608	XLOC_203040	ENSRNOT00000083964	-0.953068434	1.66E-06
TUCP_004608	XLOC_203040	ENSRNOT00000085545	-0.955314033	1.30E-06
TUCP_004608	XLOC_203040	ENSRNOT00000017486	0.971369422	1.44E-07
TUCP_004608	XLOC_203040	ENSRNOT00000089545	-0.956415046	1.15E-06
TUCP_004608	XLOC_203040	ENSRNOT00000021802	0.952497103	1.76E-06
TUCP_004608	XLOC_203040	ENSRNOT00000037809	-0.96647701	3.15E-07
TUCP_004608	XLOC_203040	ENSRNOT00000003265	0.958420446	9.13E-07
TUCP_004608	XLOC_203040	ENSRNOT00000026525	-0.95165682	1.92E-06
TUCP_004608	XLOC_203040	ENSRNOT00000027435	0.962037331	5.83E-07
TUCP_004608	XLOC_203040	ENSRNOT00000072118	0.959576435	7.94E-07
TUCP_004608	XLOC_203040	ENSRNOT00000003787	-0.950256621	2.21E-06
TUCP_004608	XLOC_203040	ENSRNOT00000014506	-0.964543614	4.16E-07
TUCP_004608	XLOC_203040	ENSRNOT00000023262	-0.958403585	9.14E-07
TUCP_004608	XLOC_203040	ENSRNOT00000015213	-0.954161893	1.48E-06
TUCP_004608	XLOC_203040	ENSRNOT00000059246	0.97538298	6.83E-08
TUCP_004608	XLOC_203040	ENSRNOT00000013067	-0.961795364	6.01E-07
TUCP_004608	XLOC_203040	ENSRNOT00000014859	-0.954058273	1.49E-06
TUCP_004608	XLOC_203040	ENSRNOT00000076230	0.95572178	1.24E-06
TUCP_004608	XLOC_203040	ENSRNOT00000026760	-0.958072247	9.51E-07
TUCP_004608	XLOC_203040	ENSRNOT00000024119	0.966707922	3.05E-07
TUCP_004608	XLOC_203040	ENSRNOT00000027145	-0.961633538	6.14E-07
TUCP_004608	XLOC_203040	ENSRNOT00000026312	0.964466877	4.20E-07
TUCP_004608	XLOC_203040	ENSRNOT00000020537	-0.961638509	6.13E-07

TUCP_004608	XLOC_203040	ENSRNOT00000036658	-0.967757945	2.60E-07
TUCP_004608	XLOC_203040	ENSRNOT00000089611	0.957184319	1.05E-06
TUCP_004608	XLOC_203040	ENSRNOT00000005604	0.982681068	1.19E-08
TUCP_004608	XLOC_203040	ENSRNOT00000016637	-0.955942402	1.21E-06
TUCP_004608	XLOC_203040	ENSRNOT00000010674	-0.954946692	1.36E-06
TUCP_004608	XLOC_203040	ENSRNOT00000038994	-0.954893299	1.36E-06
TUCP_004608	XLOC_203040	ENSRNOT00000086260	-0.958167625	9.40E-07
TUCP_004608	XLOC_203040	ENSRNOT00000068381	0.961607553	6.16E-07
TUCP_004608	XLOC_203040	ENSRNOT00000031977	0.961532431	6.22E-07
TUCP_004608	XLOC_203040	ENSRNOT00000017361	0.961844911	5.97E-07
TUCP_004608	XLOC_203040	ENSRNOT00000018328	-0.950615576	2.13E-06
TUCP_004608	XLOC_203040	ENSRNOT00000019531	-0.951355641	1.98E-06
TUCP_004608	XLOC_203040	ENSRNOT00000008253	-0.965775234	3.49E-07
TUCP_004608	XLOC_203040	ENSRNOT00000029701	0.959306779	8.21E-07
TUCP_004608	XLOC_203040	ENSRNOT00000008256	-0.951671782	1.91E-06
TUCP_004608	XLOC_203040	ENSRNOT00000001099	-0.952050052	1.84E-06
TUCP_004608	XLOC_203040	ENSRNOT00000071482	-0.973632525	9.60E-08
TUCP_004608	XLOC_203040	ENSRNOT00000034883	-0.964381701	4.25E-07
TUCP_004608	XLOC_203040	ENSRNOT00000045765	-0.957167817	1.06E-06
TUCP_004608	XLOC_203040	ENSRNOT00000010993	0.981674606	1.58E-08
TUCP_004608	XLOC_203040	ENSRNOT00000005459	0.971333393	1.45E-07
TUCP_004608	XLOC_203040	ENSRNOT00000025824	-0.958811008	8.71E-07
TUCP_004608	XLOC_203040	ENSRNOT00000007821	0.966340026	3.22E-07
TUCP_004608	XLOC_203040	ENSRNOT00000026531	-0.967256438	2.81E-07
TUCP_004608	XLOC_203040	ENSRNOT00000005172	0.960762114	6.86E-07
TUCP_004608	XLOC_203040	ENSRNOT00000020316	-0.951143692	2.02E-06
TUCP_004608	XLOC_203040	ENSRNOT00000067739	-0.977494918	4.38E-08
TUCP_004608	XLOC_203040	ENSRNOT00000005073	0.953704695	1.55E-06
TUCP_004608	XLOC_203040	ENSRNOT00000017624	0.96393088	4.53E-07
TUCP_004608	XLOC_203040	ENSRNOT00000003992	-0.959890226	7.64E-07
TUCP_004608	XLOC_203040	ENSRNOT00000065529	0.951187112	2.01E-06
TUCP_004608	XLOC_203040	ENSRNOT00000073356	0.969659708	1.92E-07
TUCP_004608	XLOC_203040	ENSRNOT00000031261	0.968233786	2.42E-07
TUCP_004608	XLOC_203040	ENSRNOT00000050515	0.961469914	6.27E-07
TUCP_004608	XLOC_203040	ENSRNOT00000026249	-0.974001808	8.95E-08
TUCP_004608	XLOC_203040	ENSRNOT00000089980	-0.951708531	1.91E-06
TUCP_004608	XLOC_203040	ENSRNOT00000028744	-0.959986472	7.55E-07
TUCP_004608	XLOC_203040	ENSRNOT00000015518	-0.961664734	6.11E-07
TUCP_004608	XLOC_203040	ENSRNOT00000055602	-0.957698554	9.93E-07
TUCP_004608	XLOC_203040	ENSRNOT00000075843	-0.980440716	2.18E-08
TUCP_004608	XLOC_203040	ENSRNOT00000055607	-0.953519629	1.58E-06
TUCP_004608	XLOC_203040	ENSRNOT00000073019	0.963258053	4.96E-07
TUCP_004608	XLOC_203040	ENSRNOT00000041106	0.965820637	3.47E-07
TUCP_004608	XLOC_203040	ENSRNOT00000011277	0.953630792	1.56E-06
TUCP_004608	XLOC_203040	ENSRNOT00000034400	-0.956613909	1.13E-06
TUCP_004608	XLOC_203040	ENSRNOT00000013801	0.97270333	1.14E-07
TUCP_004608	XLOC_203040	ENSRNOT00000088214	0.977191053	4.68E-08
TUCP_004608	XLOC_203040	ENSRNOT00000000258	-0.953252374	1.63E-06
TUCP_004608	XLOC_203040	ENSRNOT00000000013	0.962626409	5.39E-07
TUCP_004608	XLOC_203040	ENSRNOT00000018477	-0.971138039	1.50E-07
TUCP_004608	XLOC_203040	ENSRNOT00000087502	0.951833143	1.88E-06

TUCP_004608	XLOC_203040	ENSRNOT00000081110	-0.960061078	7.48E-07
TUCP_004608	XLOC_203040	ENSRNOT00000026258	0.955174405	1.32E-06
TUCP_004608	XLOC_203040	ENSRNOT00000049285	-0.965490062	3.64E-07
TUCP_004608	XLOC_203040	ENSRNOT00000015239	-0.956510493	1.14E-06
TUCP_004608	XLOC_203040	ENSRNOT00000074649	0.968941872	2.16E-07
TUCP_004608	XLOC_203040	ENSRNOT00000037955	-0.977881612	4.02E-08
TUCP_004608	XLOC_203040	ENSRNOT00000049725	-0.951242441	2.00E-06
TUCP_004608	XLOC_203040	ENSRNOT00000077146	-0.959072023	8.44E-07
TUCP_004608	XLOC_203040	ENSRNOT00000036282	0.966451043	3.16E-07
TUCP_004608	XLOC_203040	ENSRNOT00000091427	-0.950469694	2.16E-06
TUCP_004608	XLOC_203040	ENSRNOT00000084422	0.952665089	1.73E-06
TUCP_004608	XLOC_203040	ENSRNOT00000024938	-0.9559361	1.21E-06
TUCP_004608	XLOC_203040	ENSRNOT00000039177	-0.956726861	1.11E-06
TUCP_004608	XLOC_203040	ENSRNOT00000086456	-0.956529769	1.14E-06
TUCP_004608	XLOC_203040	ENSRNOT00000075209	-0.972282311	1.23E-07
TUCP_004608	XLOC_203040	ENSRNOT00000058382	0.972159791	1.26E-07
TUCP_004608	XLOC_203040	ENSRNOT00000023977	-0.963155283	5.03E-07
TUCP_004608	XLOC_203040	ENSRNOT00000000155	0.952570877	1.75E-06
TUCP_004608	XLOC_203040	ENSRNOT00000017761	0.955840126	1.23E-06
TUCP_004608	XLOC_203040	ENSRNOT00000034411	0.964639739	4.10E-07
TUCP_004608	XLOC_203040	ENSRNOT00000055957	-0.95454118	1.42E-06
TUCP_004608	XLOC_203040	ENSRNOT00000048933	-0.951139611	2.02E-06
TUCP_004608	XLOC_203040	ENSRNOT00000059181	0.975940098	6.10E-08
TUCP_004608	XLOC_203040	ENSRNOT00000075630	-0.964755458	4.04E-07
TUCP_004608	XLOC_203040	ENSRNOT00000003691	-0.955467171	1.28E-06
TUCP_004608	XLOC_203040	ENSRNOT00000002313	-0.95706301	1.07E-06
TUCP_004608	XLOC_203040	ENSRNOT00000025794	-0.960602979	7.00E-07
TUCP_004608	XLOC_203040	ENSRNOT00000058111	-0.9680434	2.49E-07
TUCP_004608	XLOC_203040	ENSRNOT00000085118	0.957669857	9.97E-07
TUCP_004608	XLOC_203040	ENSRNOT00000007276	-0.951135649	2.02E-06
TUCP_004608	XLOC_203040	ENSRNOT00000057738	0.954692452	1.39E-06
TUCP_004608	XLOC_203040	ENSRNOT00000007738	-0.955460066	1.28E-06
TUCP_004608	XLOC_203040	ENSRNOT00000017190	0.965083453	3.85E-07
TUCP_004608	XLOC_203040	ENSRNOT00000009994	-0.979416356	2.81E-08
TUCP_004608	XLOC_203040	ENSRNOT00000025887	-0.953553788	1.57E-06
TUCP_004608	XLOC_203040	ENSRNOT00000065897	0.961559458	6.20E-07
TUCP_004608	XLOC_203040	ENSRNOT00000002114	-0.951240642	2.00E-06
TUCP_004608	XLOC_203040	ENSRNOT00000022142	0.951728373	1.90E-06
TUCP_004608	XLOC_203040	ENSRNOT00000080608	0.95060277	2.13E-06
TUCP_004608	XLOC_203040	ENSRNOT00000064535	-0.964023447	4.47E-07
TUCP_004608	XLOC_203040	ENSRNOT00000018634	-0.964494888	4.19E-07
TUCP_004608	XLOC_203040	ENSRNOT00000087408	0.954277945	1.46E-06
TUCP_004608	XLOC_203040	ENSRNOT00000035145	-0.95198446	1.85E-06
TUCP_004608	XLOC_203040	ENSRNOT00000077152	0.962440985	5.53E-07
TUCP_004608	XLOC_203040	ENSRNOT00000015537	0.966029082	3.37E-07
TUCP_004608	XLOC_203040	ENSRNOT00000052120	0.957256041	1.05E-06
TUCP_004608	XLOC_203040	ENSRNOT00000017385	-0.979969611	2.46E-08
TUCP_004608	XLOC_203040	ENSRNOT00000064735	0.951686759	1.91E-06
TUCP_004608	XLOC_203040	ENSRNOT00000010024	0.956012234	1.20E-06
TUCP_004608	XLOC_203040	ENSRNOT00000046215	-0.957596024	1.01E-06
TUCP_004608	XLOC_203040	ENSRNOT00000039102	0.974646672	7.91E-08

TUCP_004608	XLOC_203040	ENSRNOT00000037405	0.95893037	8.59E-07
TUCP_004608	XLOC_203040	ENSRNOT00000001135	-0.953316633	1.61E-06
TUCP_004608	XLOC_203040	ENSRNOT00000091205	0.950481513	2.16E-06
TUCP_004608	XLOC_203040	ENSRNOT00000078994	0.953012613	1.67E-06
TUCP_004608	XLOC_203040	ENSRNOT00000025705	0.956172277	1.18E-06
TUCP_004608	XLOC_203040	ENSRNOT00000025791	-0.950670002	2.12E-06
TUCP_004608	XLOC_203040	ENSRNOT00000010983	0.982395695	1.29E-08
TUCP_004608	XLOC_203040	ENSRNOT00000080222	-0.956153307	1.19E-06
TUCP_004608	XLOC_203040	ENSRNOT00000085279	0.967352731	2.77E-07
TUCP_004608	XLOC_203040	ENSRNOT00000025460	0.982738169	1.17E-08
TUCP_004608	XLOC_203040	ENSRNOT00000071917	0.957665851	9.97E-07
TUCP_004608	XLOC_203040	ENSRNOT00000055442	0.973292672	1.02E-07
TUCP_004608	XLOC_203040	ENSRNOT00000028225	0.952661945	1.73E-06
TUCP_004608	XLOC_203040	ENSRNOT00000001412	0.963627426	4.72E-07
TUCP_004608	XLOC_203040	ENSRNOT00000001289	0.950609214	2.13E-06
TUCP_004608	XLOC_203040	ENSRNOT00000061345	-0.96262288	5.40E-07
TUCP_004608	XLOC_203040	ENSRNOT00000015495	-0.951811597	1.89E-06
TUCP_004608	XLOC_203040	ENSRNOT00000016568	0.966870264	2.97E-07
TUCP_004608	XLOC_203040	ENSRNOT00000032248	0.959354978	8.16E-07
TUCP_004608	XLOC_203040	ENSRNOT00000027360	-0.952351286	1.79E-06
TUCP_004608	XLOC_203040	ENSRNOT00000082904	0.986661582	3.25E-09
TUCP_004608	XLOC_203040	ENSRNOT00000010906	-0.951360594	1.98E-06
TUCP_004608	XLOC_203040	ENSRNOT00000015223	0.984883502	6.06E-09
TUCP_004608	XLOC_203040	ENSRNOT00000021534	-0.952576889	1.74E-06
TUCP_004608	XLOC_203040	ENSRNOT00000023628	-0.97295353	1.09E-07
TUCP_004608	XLOC_203040	ENSRNOT00000028907	0.969983071	1.82E-07
TUCP_004608	XLOC_203040	ENSRNOT00000015188	0.95654693	1.13E-06
TUCP_004608	XLOC_203040	ENSRNOT00000013516	0.962250445	5.67E-07
TUCP_004608	XLOC_203040	ENSRNOT00000025314	0.970695113	1.62E-07
TUCP_004608	XLOC_203040	ENSRNOT00000024841	-0.971305217	1.46E-07
TUCP_004608	XLOC_203040	ENSRNOT00000016776	-0.951186214	2.01E-06
TUCP_004608	XLOC_203040	ENSRNOT00000015383	0.950724556	2.11E-06
TUCP_004608	XLOC_203040	ENSRNOT00000079414	-0.962473199	5.50E-07
TUCP_004608	XLOC_203040	ENSRNOT00000008987	-0.953604449	1.57E-06
TUCP_004608	XLOC_203040	ENSRNOT00000024011	0.969433511	2.00E-07
TUCP_004608	XLOC_203040	ENSRNOT00000037665	0.96506109	3.87E-07
TUCP_004608	XLOC_203040	ENSRNOT00000092876	-0.963053153	5.10E-07
TUCP_004608	XLOC_203040	ENSRNOT00000025913	-0.952682905	1.72E-06
TUCP_004608	XLOC_203040	ENSRNOT00000024253	0.972217285	1.24E-07
TUCP_004608	XLOC_203040	ENSRNOT00000072357	-0.97680519	5.09E-08
TUCP_004354	XLOC_192341	ENSRNOT00000061139	0.972503422	1.18E-07
TUCP_004354	XLOC_192341	ENSRNOT00000074042	0.971272476	1.47E-07
TUCP_004354	XLOC_192341	ENSRNOT00000072939	0.968073702	2.48E-07
TUCP_004354	XLOC_192341	ENSRNOT00000029531	-0.956403559	1.15E-06
TUCP_004607	XLOC_202994	ENSRNOT00000092200	0.957873245	9.73E-07
TUCP_004356	XLOC_192365	ENSRNOT00000072077	0.955902811	1.22E-06
TUCP_004356	XLOC_192365	ENSRNOT00000011344	0.961006643	6.65E-07
TUCP_004356	XLOC_192365	ENSRNOT00000026361	0.962951847	5.17E-07
TUCP_004356	XLOC_192365	ENSRNOT00000092196	0.962389915	5.56E-07
TUCP_004357	XLOC_192366	ENSRNOT00000008961	0.975339793	6.89E-08
TUCP_004357	XLOC_192366	ENSRNOT00000026677	0.968893756	2.18E-07

TUCP_004357	XLOC_192366	ENSRNOT00000035523	0.97346889	9.90E-08
TUCP_004357	XLOC_192366	ENSRNOT00000082611	0.974370091	8.34E-08
TUCP_004357	XLOC_192366	ENSRNOT00000033719	0.969618236	1.94E-07
TUCP_004357	XLOC_192366	ENSRNOT00000007358	0.953735045	1.54E-06
TUCP_004357	XLOC_192366	ENSRNOT00000029620	0.953820015	1.53E-06
TUCP_004357	XLOC_192366	ENSRNOT00000001853	0.95660121	1.13E-06
TUCP_004357	XLOC_192366	ENSRNOT00000086541	0.966738801	3.03E-07
TUCP_004357	XLOC_192366	ENSRNOT00000055127	0.967262647	2.80E-07
TUCP_004357	XLOC_192366	ENSRNOT00000030745	-0.966712929	3.04E-07
TUCP_004357	XLOC_192366	ENSRNOT00000084873	0.955312692	1.30E-06
TUCP_004357	XLOC_192366	ENSRNOT00000011134	0.957051916	1.07E-06
TUCP_004357	XLOC_192366	ENSRNOT00000076910	0.969077028	2.11E-07
TUCP_004357	XLOC_192366	ENSRNOT00000024718	0.977250272	4.62E-08
TUCP_004357	XLOC_192366	ENSRNOT00000055674	0.953944924	1.51E-06
TUCP_004357	XLOC_192366	ENSRNOT00000000448	0.962674116	5.36E-07
TUCP_004357	XLOC_192366	ENSRNOT00000068683	0.96092763	6.72E-07
TUCP_004357	XLOC_192366	ENSRNOT00000090867	0.953647416	1.56E-06
TUCP_004357	XLOC_192366	ENSRNOT00000034603	0.963494865	4.80E-07
TUCP_004357	XLOC_192366	ENSRNOT00000032719	0.972804527	1.12E-07
TUCP_004357	XLOC_192366	ENSRNOT00000089309	0.967602498	2.66E-07
TUCP_004357	XLOC_192366	ENSRNOT00000027949	0.98106876	1.86E-08
TUCP_004357	XLOC_192366	ENSRNOT00000050656	0.961447903	6.29E-07
TUCP_004357	XLOC_192366	ENSRNOT00000082116	0.97585946	6.20E-08
TUCP_004357	XLOC_192366	ENSRNOT00000002936	0.962558767	5.44E-07
TUCP_004357	XLOC_192366	ENSRNOT00000014684	0.970531233	1.67E-07
TUCP_004357	XLOC_192366	ENSRNOT00000001570	0.96840878	2.35E-07
TUCP_004357	XLOC_192366	ENSRNOT00000081530	-0.955425344	1.29E-06
TUCP_004357	XLOC_192366	ENSRNOT00000074738	0.955544547	1.27E-06
TUCP_004357	XLOC_192366	ENSRNOT00000015618	0.952581678	1.74E-06
TUCP_004357	XLOC_192366	ENSRNOT00000004637	0.970281214	1.74E-07
TUCP_004357	XLOC_192366	ENSRNOT00000078058	0.950335708	2.19E-06
TUCP_004357	XLOC_192366	ENSRNOT00000046888	0.956443116	1.15E-06
TUCP_004357	XLOC_192366	ENSRNOT00000015576	0.973760456	9.37E-08
TUCP_004357	XLOC_192366	ENSRNOT00000054757	0.959715449	7.81E-07
TUCP_004357	XLOC_192366	ENSRNOT00000043660	0.963594285	4.74E-07
TUCP_004357	XLOC_192366	ENSRNOT00000019262	-0.969516738	1.97E-07
TUCP_004357	XLOC_192366	ENSRNOT00000054897	-0.954127583	1.48E-06
TUCP_004357	XLOC_192366	ENSRNOT00000009620	0.955181678	1.32E-06
TUCP_004357	XLOC_192366	ENSRNOT00000091863	0.951026692	2.04E-06
TUCP_004357	XLOC_192366	ENSRNOT00000029990	0.974191353	8.64E-08
TUCP_004357	XLOC_192366	ENSRNOT00000081036	0.952525497	1.75E-06
TUCP_004357	XLOC_192366	ENSRNOT00000033984	0.974289738	8.47E-08
TUCP_004357	XLOC_192366	ENSRNOT00000092272	0.963027272	5.11E-07
TUCP_004357	XLOC_192366	ENSRNOT00000011481	0.968840889	2.20E-07
TUCP_004357	XLOC_192366	ENSRNOT00000016449	0.959695804	7.83E-07
TUCP_004357	XLOC_192366	ENSRNOT00000010678	-0.957338033	1.04E-06
TUCP_004357	XLOC_192366	ENSRNOT00000044983	0.977772578	4.12E-08
TUCP_004357	XLOC_192366	ENSRNOT00000024464	0.957771032	9.85E-07
TUCP_004357	XLOC_192366	ENSRNOT00000061973	0.95693415	1.09E-06
TUCP_004357	XLOC_192366	ENSRNOT00000010264	0.958221186	9.34E-07
TUCP_004357	XLOC_192366	ENSRNOT00000090654	0.970582597	1.65E-07

TUCP_004357	XLOC_192366	ENSRNOT00000039729	0.95203381	1.84E-06
TUCP_004357	XLOC_192366	ENSRNOT00000004919	0.96046603	7.12E-07
TUCP_004357	XLOC_192366	ENSRNOT00000060765	0.968412815	2.35E-07
TUCP_004357	XLOC_192366	ENSRNOT00000021816	0.961649634	6.13E-07
TUCP_004357	XLOC_192366	ENSRNOT00000024992	0.964506201	4.18E-07
TUCP_004357	XLOC_192366	ENSRNOT00000030532	0.971272949	1.47E-07
TUCP_004357	XLOC_192366	ENSRNOT00000056995	0.978885938	3.19E-08
TUCP_004357	XLOC_192366	ENSRNOT00000056551	0.952910517	1.68E-06
TUCP_004357	XLOC_192366	ENSRNOT00000030251	-0.961353486	6.36E-07
TUCP_004357	XLOC_192366	ENSRNOT00000023100	0.964219991	4.35E-07
TUCP_004357	XLOC_192366	ENSRNOT00000057016	0.985294823	5.28E-09
TUCP_004357	XLOC_192366	ENSRNOT00000055888	0.957203433	1.05E-06
TUCP_004357	XLOC_192366	ENSRNOT00000019501	0.950277102	2.20E-06
TUCP_004357	XLOC_192366	ENSRNOT00000044975	0.955446043	1.28E-06
TUCP_004357	XLOC_192366	ENSRNOT00000018782	0.963339863	4.90E-07
TUCP_004357	XLOC_192366	ENSRNOT00000023504	0.961365097	6.35E-07
TUCP_004357	XLOC_192366	ENSRNOT00000084021	0.979898904	2.50E-08
TUCP_004357	XLOC_192366	ENSRNOT00000088201	0.955648965	1.25E-06
TUCP_004357	XLOC_192366	ENSRNOT00000049536	0.951799988	1.89E-06
TUCP_004357	XLOC_192366	ENSRNOT00000086459	0.958923418	8.60E-07
TUCP_004357	XLOC_192366	ENSRNOT00000071133	0.950284695	2.20E-06
TUCP_004357	XLOC_192366	ENSRNOT00000092857	0.970065094	1.80E-07
TUCP_004357	XLOC_192366	ENSRNOT00000018061	0.958522002	9.02E-07
TUCP_004357	XLOC_192366	ENSRNOT00000089185	0.954280803	1.46E-06
TUCP_004357	XLOC_192366	ENSRNOT00000031951	0.959182777	8.33E-07
TUCP_004357	XLOC_192366	ENSRNOT00000000640	0.963199419	5.00E-07
TUCP_004357	XLOC_192366	ENSRNOT00000084429	0.95149569	1.95E-06
TUCP_004357	XLOC_192366	ENSRNOT00000029443	0.953331715	1.61E-06
TUCP_004357	XLOC_192366	ENSRNOT00000022373	0.959756831	7.77E-07
TUCP_004357	XLOC_192366	ENSRNOT00000059660	0.952918195	1.68E-06
TUCP_004357	XLOC_192366	ENSRNOT00000090186	0.9509059	2.07E-06
TUCP_004357	XLOC_192366	ENSRNOT00000073547	0.980648358	2.07E-08
TUCP_004357	XLOC_192366	ENSRNOT00000043434	0.962178091	5.72E-07
TUCP_004357	XLOC_192366	ENSRNOT00000028423	0.953306196	1.62E-06
TUCP_004357	XLOC_192366	ENSRNOT00000051363	0.950187794	2.22E-06
TUCP_004357	XLOC_192366	ENSRNOT00000022648	0.965443838	3.66E-07
TUCP_004357	XLOC_192366	ENSRNOT00000086390	0.95332804	1.61E-06
TUCP_004357	XLOC_192366	ENSRNOT00000073952	0.969517924	1.97E-07
TUCP_004357	XLOC_192366	ENSRNOT00000036753	0.95631496	1.16E-06
TUCP_004357	XLOC_192366	ENSRNOT00000036972	0.955627818	1.26E-06
TUCP_004357	XLOC_192366	ENSRNOT00000083814	0.954945351	1.36E-06
TUCP_000221	XLOC_006887	ENSRNOT00000071840	0.999992988	1.33E-25
TUCP_000221	XLOC_006887	ENSRNOT00000083608	0.992964626	1.34E-10
TUCP_000221	XLOC_006887	ENSRNOT00000076035	0.957251891	1.05E-06
TUCP_000221	XLOC_006887	ENSRNOT00000085476	0.962978424	5.15E-07
TUCP_000221	XLOC_006887	ENSRNOT00000066382	0.962855154	5.23E-07
TUCP_000221	XLOC_006887	ENSRNOT00000045584	0.999992988	1.33E-25
TUCP_000221	XLOC_006887	ENSRNOT00000077995	0.994337005	4.54E-11
TUCP_000221	XLOC_006887	ENSRNOT00000066004	0.999882249	1.78E-19
TUCP_000221	XLOC_006887	ENSRNOT00000064284	0.968682394	2.25E-07
TUCP_000221	XLOC_006887	ENSRNOT00000068197	0.955184471	1.32E-06

TUCP_000221	XLOC_006887	ENSRNOT00000082086	0.972842455	1.11E-07
TUCP_000221	XLOC_006887	ENSRNOT00000089494	0.983640853	8.98E-09
TUCP_000221	XLOC_006887	ENSRNOT00000083747	0.957552538	1.01E-06
TUCP_000221	XLOC_006887	ENSRNOT00000092182	0.999992988	1.33E-25
TUCP_000221	XLOC_006887	ENSRNOT00000084461	0.999992988	1.33E-25
TUCP_000221	XLOC_006887	ENSRNOT00000060448	0.999992988	1.33E-25
TUCP_000221	XLOC_006887	ENSRNOT00000032631	0.999992988	1.33E-25
TUCP_000221	XLOC_006887	ENSRNOT00000079503	0.957014919	1.08E-06
TUCP_000221	XLOC_006887	ENSRNOT00000027950	0.960812588	6.81E-07
TUCP_000221	XLOC_006887	ENSRNOT00000060113	0.975150403	7.16E-08
TUCP_000221	XLOC_006887	ENSRNOT00000054910	0.990762785	5.22E-10
TUCP_000221	XLOC_006887	ENSRNOT00000057176	0.999991401	3.70E-25
TUCP_000221	XLOC_006887	ENSRNOT00000078176	0.994741303	3.14E-11
TUCP_000221	XLOC_006887	ENSRNOT00000056364	0.990542932	5.86E-10
TUCP_000221	XLOC_006887	ENSRNOT00000093431	0.999992988	1.33E-25
TUCP_000221	XLOC_006887	ENSRNOT00000024758	0.981879802	1.49E-08
TUCP_000221	XLOC_006887	ENSRNOT00000092901	0.999992988	1.33E-25
TUCP_000221	XLOC_006887	ENSRNOT00000089290	0.999978532	3.59E-23
TUCP_000221	XLOC_006887	ENSRNOT00000090211	0.998803396	1.93E-14
TUCP_000221	XLOC_006887	ENSRNOT00000075244	0.962402049	5.55E-07
TUCP_000221	XLOC_006887	ENSRNOT00000091828	0.996480469	4.23E-12
TUCP_000221	XLOC_006887	ENSRNOT00000091944	0.999765249	5.61E-18
TUCP_000221	XLOC_006887	ENSRNOT00000070855	0.999992988	1.33E-25
TUCP_000221	XLOC_006887	ENSRNOT00000009295	0.995846156	9.67E-12
TUCP_000221	XLOC_006887	ENSRNOT00000084109	0.9996843	2.47E-17
TUCP_000221	XLOC_006887	ENSRNOT00000060645	0.973757044	9.38E-08
TUCP_000221	XLOC_006887	ENSRNOT00000041569	0.999992988	1.33E-25
TUCP_000221	XLOC_006887	ENSRNOT00000087091	0.999992988	1.33E-25
TUCP_000221	XLOC_006887	ENSRNOT00000003308	0.990733327	5.30E-10
TUCP_000221	XLOC_006887	ENSRNOT00000026113	0.999837988	8.79E-19
TUCP_000221	XLOC_006887	ENSRNOT00000014939	0.999866453	3.34E-19
TUCP_000221	XLOC_006887	ENSRNOT00000058314	0.990827024	5.04E-10
TUCP_000221	XLOC_006887	ENSRNOT00000047870	0.981462504	1.67E-08
TUCP_000221	XLOC_006887	ENSRNOT00000071603	0.999987788	2.14E-24
TUCP_000221	XLOC_006887	ENSRNOT00000088376	0.970345943	1.72E-07
TUCP_000221	XLOC_006887	ENSRNOT00000060454	0.999992988	1.33E-25
TUCP_000221	XLOC_006887	ENSRNOT00000029298	0.95794394	9.65E-07
TUCP_000221	XLOC_006887	ENSRNOT00000044232	0.983629178	9.01E-09
TUCP_000221	XLOC_006887	ENSRNOT00000023287	0.974834689	7.62E-08
TUCP_000221	XLOC_006887	ENSRNOT00000071714	0.978730121	3.31E-08
TUCP_000221	XLOC_006887	ENSRNOT00000075836	0.999992988	1.33E-25
TUCP_000221	XLOC_006887	ENSRNOT00000073229	0.999992988	1.33E-25
TUCP_000221	XLOC_006887	ENSRNOT00000077731	0.965526056	3.62E-07
TUCP_000221	XLOC_006887	ENSRNOT00000046980	0.982590864	1.22E-08
TUCP_000221	XLOC_006887	ENSRNOT00000039652	0.972454739	1.19E-07
TUCP_000221	XLOC_006887	ENSRNOT00000066685	0.980945022	1.92E-08
TUCP_000221	XLOC_006887	ENSRNOT00000082776	0.966276892	3.25E-07
TUCP_000221	XLOC_006887	ENSRNOT00000088719	0.966144467	3.31E-07
TUCP_000221	XLOC_006887	ENSRNOT00000007683	0.999774923	4.55E-18
TUCP_000221	XLOC_006887	ENSRNOT00000092916	0.966025713	3.37E-07
TUCP_000221	XLOC_006887	ENSRNOT00000081106	0.953338022	1.61E-06

TUCP_004352	XLOC_192224	ENSRNOT00000093467	0.951435504	1.96E-06
TUCP_004352	XLOC_192224	ENSRNOT00000029340	0.967208214	2.83E-07
TUCP_004352	XLOC_192224	ENSRNOT00000038646	0.962390738	5.56E-07
TUCP_004352	XLOC_192224	ENSRNOT00000092579	0.962390738	5.56E-07
TUCP_004352	XLOC_192224	ENSRNOT00000036383	0.954947521	1.36E-06
TUCP_004352	XLOC_192224	ENSRNOT00000076528	0.956578036	1.13E-06
TUCP_004352	XLOC_192224	ENSRNOT00000083034	0.957150091	1.06E-06
TUCP_004352	XLOC_192224	ENSRNOT00000089607	0.954509419	1.42E-06
TUCP_004352	XLOC_192224	ENSRNOT00000021748	0.962390738	5.56E-07
TUCP_004352	XLOC_192224	ENSRNOT00000071484	0.987018814	2.84E-09
TUCP_004352	XLOC_192224	ENSRNOT00000078137	0.962390738	5.56E-07
TUCP_004352	XLOC_192224	ENSRNOT00000092740	0.962390738	5.56E-07
TUCP_004352	XLOC_192224	ENSRNOT00000000117	0.9500972	2.24E-06
TUCP_004352	XLOC_192224	ENSRNOT00000070958	0.962390738	5.56E-07
TUCP_004352	XLOC_192224	ENSRNOT00000027268	0.962390738	5.56E-07
TUCP_004352	XLOC_192224	ENSRNOT00000004459	0.962390738	5.56E-07
TUCP_004352	XLOC_192224	ENSRNOT00000089235	0.955763807	1.24E-06
TUCP_004352	XLOC_192224	ENSRNOT00000088168	0.962390738	5.56E-07
TUCP_004352	XLOC_192224	ENSRNOT00000088148	0.962390738	5.56E-07
TUCP_004352	XLOC_192224	ENSRNOT00000088527	0.962390738	5.56E-07
TUCP_004352	XLOC_192224	ENSRNOT00000078701	0.962390738	5.56E-07
TUCP_004352	XLOC_192224	ENSRNOT00000090078	0.962390738	5.56E-07
TUCP_004352	XLOC_192224	ENSRNOT00000089085	0.962390738	5.56E-07
TUCP_004352	XLOC_192224	ENSRNOT00000008153	0.959453003	8.06E-07
TUCP_004352	XLOC_192224	ENSRNOT00000055073	0.969742388	1.90E-07
TUCP_004352	XLOC_192224	ENSRNOT00000010948	0.952949403	1.68E-06
TUCP_004352	XLOC_192224	ENSRNOT00000009776	0.962390738	5.56E-07
TUCP_000223	XLOC_006892	ENSRNOT00000084243	-0.959739793	7.79E-07
TUCP_003934	XLOC_172959	ENSRNOT00000014401	0.952444855	1.77E-06
TUCP_003934	XLOC_172959	ENSRNOT00000059984	0.966832587	2.99E-07
TUCP_004217	XLOC_184965	ENSRNOT00000084500	0.984887141	6.05E-09
TUCP_004217	XLOC_184965	ENSRNOT00000093467	0.996134431	6.75E-12
TUCP_004217	XLOC_184965	ENSRNOT00000029340	0.999116572	4.23E-15
TUCP_004217	XLOC_184965	ENSRNOT00000077972	0.962758032	5.30E-07
TUCP_004217	XLOC_184965	ENSRNOT00000038646	1	0
TUCP_004217	XLOC_184965	ENSRNOT00000012095	0.957750287	9.88E-07
TUCP_004217	XLOC_184965	ENSRNOT00000017909	0.975587192	6.56E-08
TUCP_004217	XLOC_184965	ENSRNOT00000092579	1	0
TUCP_004217	XLOC_184965	ENSRNOT00000089235	0.998661162	3.38E-14
TUCP_004217	XLOC_184965	ENSRNOT00000036383	0.988460809	1.58E-09
TUCP_004217	XLOC_184965	ENSRNOT00000076528	0.997115373	1.57E-12
TUCP_004217	XLOC_184965	ENSRNOT00000075193	0.964326971	4.29E-07
TUCP_004217	XLOC_184965	ENSRNOT00000083034	0.990934646	4.75E-10
TUCP_004217	XLOC_184965	ENSRNOT00000089607	0.995091455	2.23E-11
TUCP_004217	XLOC_184965	ENSRNOT00000086014	0.960893638	6.74E-07
TUCP_004217	XLOC_184965	ENSRNOT00000054939	0.958021495	9.57E-07
TUCP_004217	XLOC_184965	ENSRNOT00000052315	0.983687082	8.85E-09
TUCP_004217	XLOC_184965	ENSRNOT00000088168	1	0
TUCP_004217	XLOC_184965	ENSRNOT00000046231	0.96225463	5.66E-07
TUCP_004217	XLOC_184965	ENSRNOT00000013459	0.979098017	3.03E-08
TUCP_004217	XLOC_184965	ENSRNOT00000078137	1	0

TUCP_004217	XLOC_184965	ENSRNOT00000077764	0.977143528	4.73E-08
TUCP_004217	XLOC_184965	ENSRNOT00000043661	0.966975951	2.93E-07
TUCP_004217	XLOC_184965	ENSRNOT00000092740	1	0
TUCP_004217	XLOC_184965	ENSRNOT00000000117	0.990989174	4.61E-10
TUCP_004217	XLOC_184965	ENSRNOT00000071877	0.967113583	2.87E-07
TUCP_004217	XLOC_184965	ENSRNOT00000070958	1	0
TUCP_004217	XLOC_184965	ENSRNOT00000076757	0.959205354	8.31E-07
TUCP_004217	XLOC_184965	ENSRNOT00000085310	0.965230243	3.78E-07
TUCP_004217	XLOC_184965	ENSRNOT00000047241	0.966576993	3.11E-07
TUCP_004217	XLOC_184965	ENSRNOT00000027268	1	0
TUCP_004217	XLOC_184965	ENSRNOT00000004459	1	0
TUCP_004217	XLOC_184965	ENSRNOT00000048373	0.959096578	8.42E-07
TUCP_004217	XLOC_184965	ENSRNOT00000061516	0.987595648	2.27E-09
TUCP_004217	XLOC_184965	ENSRNOT00000088148	1	0
TUCP_004217	XLOC_184965	ENSRNOT00000088527	1	0
TUCP_004217	XLOC_184965	ENSRNOT00000078701	1	0
TUCP_004217	XLOC_184965	ENSRNOT00000052308	0.962646127	5.38E-07
TUCP_004217	XLOC_184965	ENSRNOT00000021748	1	0
TUCP_004217	XLOC_184965	ENSRNOT00000089085	1	0
TUCP_004217	XLOC_184965	ENSRNOT00000008153	0.999703506	1.80E-17
TUCP_004217	XLOC_184965	ENSRNOT00000085896	0.971605382	1.39E-07
TUCP_004217	XLOC_184965	ENSRNOT00000079189	0.984972387	5.89E-09
TUCP_004217	XLOC_184965	ENSRNOT00000090078	1	0
TUCP_004217	XLOC_184965	ENSRNOT00000078020	0.958421666	9.13E-07
TUCP_004217	XLOC_184965	ENSRNOT00000092425	0.956092273	1.19E-06
TUCP_004217	XLOC_184965	ENSRNOT00000009776	1	1.33E-79
TUCP_004216	XLOC_184965	ENSRNOT00000084500	0.983839419	8.45E-09
TUCP_004216	XLOC_184965	ENSRNOT00000093467	0.99444796	4.12E-11
TUCP_004216	XLOC_184965	ENSRNOT00000029340	0.998929726	1.10E-14
TUCP_004216	XLOC_184965	ENSRNOT00000077972	0.959552202	7.97E-07
TUCP_004216	XLOC_184965	ENSRNOT00000038646	0.999528793	1.83E-16
TUCP_004216	XLOC_184965	ENSRNOT00000012095	0.956803359	1.10E-06
TUCP_004216	XLOC_184965	ENSRNOT00000017909	0.974387444	8.32E-08
TUCP_004216	XLOC_184965	ENSRNOT00000092579	0.999528793	1.83E-16
TUCP_004216	XLOC_184965	ENSRNOT00000089235	0.99884101	1.64E-14
TUCP_004216	XLOC_184965	ENSRNOT00000036383	0.988071744	1.86E-09
TUCP_004216	XLOC_184965	ENSRNOT00000076528	0.996512271	4.04E-12
TUCP_004216	XLOC_184965	ENSRNOT00000075193	0.962044335	5.82E-07
TUCP_004216	XLOC_184965	ENSRNOT00000083034	0.989733695	8.83E-10
TUCP_004216	XLOC_184965	ENSRNOT00000089607	0.994371014	4.41E-11
TUCP_004216	XLOC_184965	ENSRNOT00000086014	0.963786348	4.62E-07
TUCP_004216	XLOC_184965	ENSRNOT00000054939	0.95652531	1.14E-06
TUCP_004216	XLOC_184965	ENSRNOT00000052315	0.98261738	1.21E-08
TUCP_004216	XLOC_184965	ENSRNOT00000088168	0.999528793	1.83E-16
TUCP_004216	XLOC_184965	ENSRNOT00000046231	0.960884122	6.75E-07
TUCP_004216	XLOC_184965	ENSRNOT00000013459	0.977951284	3.95E-08
TUCP_004216	XLOC_184965	ENSRNOT00000078137	0.999528793	1.83E-16
TUCP_004216	XLOC_184965	ENSRNOT00000077764	0.974889882	7.54E-08
TUCP_004216	XLOC_184965	ENSRNOT00000043661	0.965522007	3.62E-07
TUCP_004216	XLOC_184965	ENSRNOT00000092740	0.999528793	1.83E-16
TUCP_004216	XLOC_184965	ENSRNOT00000000117	0.990070859	7.47E-10

TUCP_004216	XLOC_184965	ENSRNOT00000071877	0.965275283	3.75E-07
TUCP_004216	XLOC_184965	ENSRNOT00000070958	0.999528793	1.83E-16
TUCP_004216	XLOC_184965	ENSRNOT00000076757	0.957472317	1.02E-06
TUCP_004216	XLOC_184965	ENSRNOT00000085310	0.963916143	4.53E-07
TUCP_004216	XLOC_184965	ENSRNOT00000047241	0.965257604	3.76E-07
TUCP_004216	XLOC_184965	ENSRNOT00000027268	0.999528793	1.83E-16
TUCP_004216	XLOC_184965	ENSRNOT00000004459	0.999528793	1.83E-16
TUCP_004216	XLOC_184965	ENSRNOT00000048373	0.959237962	8.28E-07
TUCP_004216	XLOC_184965	ENSRNOT00000061516	0.988322825	1.68E-09
TUCP_004216	XLOC_184965	ENSRNOT00000088148	0.999528793	1.83E-16
TUCP_004216	XLOC_184965	ENSRNOT00000088527	0.999528793	1.83E-16
TUCP_004216	XLOC_184965	ENSRNOT00000078701	0.999528793	1.83E-16
TUCP_004216	XLOC_184965	ENSRNOT00000052308	0.961280112	6.42E-07
TUCP_004216	XLOC_184965	ENSRNOT00000021748	0.999528793	1.83E-16
TUCP_004216	XLOC_184965	ENSRNOT00000089085	0.999528793	1.83E-16
TUCP_004216	XLOC_184965	ENSRNOT00000008153	0.99919269	2.70E-15
TUCP_004216	XLOC_184965	ENSRNOT00000085896	0.970206907	1.76E-07
TUCP_004216	XLOC_184965	ENSRNOT00000079189	0.986446935	3.52E-09
TUCP_004216	XLOC_184965	ENSRNOT00000090078	0.999528793	1.83E-16
TUCP_004216	XLOC_184965	ENSRNOT00000078020	0.957493028	1.02E-06
TUCP_004216	XLOC_184965	ENSRNOT00000092425	0.95416969	1.47E-06
TUCP_004216	XLOC_184965	ENSRNOT00000009776	0.999528793	1.83E-16
TUCP_003931	XLOC_172901	ENSRNOT00000029340	0.954625253	1.40E-06
TUCP_003931	XLOC_172901	ENSRNOT00000035915	0.979184327	2.97E-08
TUCP_003931	XLOC_172901	ENSRNOT00000092579	0.955173681	1.32E-06
TUCP_003931	XLOC_172901	ENSRNOT00000016026	0.95786466	9.74E-07
TUCP_003931	XLOC_172901	ENSRNOT00000075193	0.950291091	2.20E-06
TUCP_003931	XLOC_172901	ENSRNOT00000038646	0.955173681	1.32E-06
TUCP_003931	XLOC_172901	ENSRNOT00000052315	0.992847162	1.46E-10
TUCP_003931	XLOC_172901	ENSRNOT00000011761	0.97836476	3.60E-08
TUCP_003931	XLOC_172901	ENSRNOT00000078137	0.955173681	1.32E-06
TUCP_003931	XLOC_172901	ENSRNOT00000092740	0.955173681	1.32E-06
TUCP_003931	XLOC_172901	ENSRNOT00000070958	0.955173681	1.32E-06
TUCP_003931	XLOC_172901	ENSRNOT00000035538	0.978034245	3.88E-08
TUCP_003931	XLOC_172901	ENSRNOT00000027268	0.955173681	1.32E-06
TUCP_003931	XLOC_172901	ENSRNOT00000004459	0.955173681	1.32E-06
TUCP_003931	XLOC_172901	ENSRNOT00000089235	0.95765882	9.98E-07
TUCP_003931	XLOC_172901	ENSRNOT00000088168	0.955173681	1.32E-06
TUCP_003931	XLOC_172901	ENSRNOT00000088148	0.955173681	1.32E-06
TUCP_003931	XLOC_172901	ENSRNOT00000088527	0.955173681	1.32E-06
TUCP_003931	XLOC_172901	ENSRNOT00000078701	0.955173681	1.32E-06
TUCP_003931	XLOC_172901	ENSRNOT00000090078	0.955173681	1.32E-06
TUCP_003931	XLOC_172901	ENSRNOT00000021748	0.955173681	1.32E-06
TUCP_003931	XLOC_172901	ENSRNOT00000089085	0.955173681	1.32E-06
TUCP_003931	XLOC_172901	ENSRNOT00000008153	0.954033507	1.50E-06
TUCP_003931	XLOC_172901	ENSRNOT00000059593	0.974574638	8.02E-08
TUCP_003931	XLOC_172901	ENSRNOT00000092425	0.972215534	1.24E-07
TUCP_003931	XLOC_172901	ENSRNOT00000009776	0.955173681	1.32E-06
TUCP_004210	XLOC_184712	ENSRNOT00000089377	0.967920034	2.54E-07
TUCP_004198	XLOC_184475	ENSRNOT00000071840	0.973751406	9.39E-08
TUCP_004198	XLOC_184475	ENSRNOT00000083608	0.959086725	8.43E-07

TUCP_004198	XLOC_184475	ENSRNOT00000045584	0.973751406	9.39E-08
TUCP_004198	XLOC_184475	ENSRNOT00000077995	0.982294909	1.33E-08
TUCP_004198	XLOC_184475	ENSRNOT00000066004	0.973050655	1.07E-07
TUCP_004198	XLOC_184475	ENSRNOT00000066685	0.983578243	9.15E-09
TUCP_004198	XLOC_184475	ENSRNOT00000089494	0.980454584	2.17E-08
TUCP_004198	XLOC_184475	ENSRNOT00000092182	0.973751406	9.39E-08
TUCP_004198	XLOC_184475	ENSRNOT00000084461	0.973751406	9.39E-08
TUCP_004198	XLOC_184475	ENSRNOT00000060448	0.973751406	9.39E-08
TUCP_004198	XLOC_184475	ENSRNOT00000032631	0.973751406	9.39E-08
TUCP_004198	XLOC_184475	ENSRNOT00000027950	0.987169761	2.68E-09
TUCP_004198	XLOC_184475	ENSRNOT00000054910	0.962267155	5.65E-07
TUCP_004198	XLOC_184475	ENSRNOT00000057176	0.973717294	9.45E-08
TUCP_004198	XLOC_184475	ENSRNOT00000078176	0.965821161	3.47E-07
TUCP_004198	XLOC_184475	ENSRNOT00000056364	0.958704344	8.82E-07
TUCP_004198	XLOC_184475	ENSRNOT00000093431	0.973751406	9.39E-08
TUCP_004198	XLOC_184475	ENSRNOT00000047560	0.951361123	1.98E-06
TUCP_004198	XLOC_184475	ENSRNOT00000090439	0.963624919	4.72E-07
TUCP_004198	XLOC_184475	ENSRNOT00000092901	0.973751406	9.39E-08
TUCP_004198	XLOC_184475	ENSRNOT00000089290	0.973584703	9.69E-08
TUCP_004198	XLOC_184475	ENSRNOT00000090211	0.97072794	1.61E-07
TUCP_004198	XLOC_184475	ENSRNOT00000091828	0.971520155	1.41E-07
TUCP_004198	XLOC_184475	ENSRNOT00000091944	0.972700047	1.14E-07
TUCP_004198	XLOC_184475	ENSRNOT00000070855	0.973751406	9.39E-08
TUCP_004198	XLOC_184475	ENSRNOT00000046980	0.952666388	1.73E-06
TUCP_004198	XLOC_184475	ENSRNOT00000084109	0.975156674	7.15E-08
TUCP_004198	XLOC_184475	ENSRNOT00000060645	0.954583699	1.41E-06
TUCP_004198	XLOC_184475	ENSRNOT00000041569	0.973751406	9.39E-08
TUCP_004198	XLOC_184475	ENSRNOT00000087091	0.973751406	9.39E-08
TUCP_004198	XLOC_184475	ENSRNOT00000003308	0.955722071	1.24E-06
TUCP_004198	XLOC_184475	ENSRNOT00000026113	0.973212285	1.04E-07
TUCP_004198	XLOC_184475	ENSRNOT00000014939	0.974776109	7.71E-08
TUCP_004198	XLOC_184475	ENSRNOT00000058314	0.961812495	6.00E-07
TUCP_004198	XLOC_184475	ENSRNOT00000071603	0.973681828	9.51E-08
TUCP_004198	XLOC_184475	ENSRNOT00000044232	0.952657096	1.73E-06
TUCP_004198	XLOC_184475	ENSRNOT00000007683	0.971728829	1.36E-07
TUCP_004198	XLOC_184475	ENSRNOT00000075836	0.973751406	9.39E-08
TUCP_004198	XLOC_184475	ENSRNOT00000073229	0.973751406	9.39E-08
TUCP_004198	XLOC_184475	ENSRNOT00000077731	0.957616676	1.00E-06
TUCP_004198	XLOC_184475	ENSRNOT00000060454	0.973751406	9.39E-08
TUCP_004198	XLOC_184475	ENSRNOT00000009295	0.967672798	2.63E-07
TUCP_001792	XLOC_073846	ENSRNOT00000030497	0.954919424	1.36E-06
TUCP_001792	XLOC_073846	ENSRNOT00000064478	0.950299423	2.20E-06
TUCP_004196	XLOC_184475	ENSRNOT00000067448	0.96613642	3.31E-07
TUCP_004196	XLOC_184475	ENSRNOT00000000327	-0.950352126	2.19E-06
TUCP_004196	XLOC_184475	ENSRNOT00000048858	0.950513951	2.15E-06
TUCP_004195	XLOC_184447	ENSRNOT00000025733	0.953484667	1.59E-06
TUCP_004219	XLOC_185247	ENSRNOT00000009811	0.953329107	1.61E-06
TUCP_004219	XLOC_185247	ENSRNOT00000005486	-0.961115936	6.56E-07
TUCP_004219	XLOC_185247	ENSRNOT00000067554	0.975269676	6.99E-08
TUCP_004219	XLOC_185247	ENSRNOT00000010852	0.951110565	2.03E-06
TUCP_004219	XLOC_185247	ENSRNOT00000026531	-0.951379202	1.97E-06

TUCP_004219	XLOC_185247	ENSRNOT00000001893	0.957135455	1.06E-06
TUCP_004219	XLOC_185247	ENSRNOT00000061253	0.964721998	4.06E-07
TUCP_004219	XLOC_185247	ENSRNOT00000084031	0.957039461	1.07E-06
TUCP_004219	XLOC_185247	ENSRNOT00000009080	-0.953323029	1.61E-06
TUCP_004219	XLOC_185247	ENSRNOT00000057738	0.958567833	8.97E-07
TUCP_004219	XLOC_185247	ENSRNOT00000077231	0.950274066	2.20E-06
TUCP_004219	XLOC_185247	ENSRNOT00000016568	0.954822621	1.37E-06
TUCP_004219	XLOC_185247	ENSRNOT00000018634	-0.958293001	9.27E-07
TUCP_004219	XLOC_185247	ENSRNOT00000080608	0.960452124	7.13E-07
TUCP_004219	XLOC_185247	ENSRNOT00000001855	0.952336799	1.79E-06
TUCP_004219	XLOC_185247	ENSRNOT00000080577	0.955085166	1.33E-06
TUCP_004219	XLOC_185247	ENSRNOT00000013067	-0.951577026	1.93E-06
TUCP_004219	XLOC_185247	ENSRNOT00000029701	0.961919269	5.92E-07
TUCP_004219	XLOC_185247	ENSRNOT00000086706	0.956897368	1.09E-06
TUCP_004219	XLOC_185247	ENSRNOT00000037133	0.955066532	1.34E-06
TUCP_004219	XLOC_185247	ENSRNOT00000087838	-0.953510246	1.58E-06
TUCP_004219	XLOC_185247	ENSRNOT00000000383	-0.955227305	1.31E-06
TUCP_004219	XLOC_185247	ENSRNOT00000017556	0.952554309	1.75E-06
TUCP_004219	XLOC_185247	ENSRNOT00000014384	-0.954424004	1.43E-06
TUCP_004219	XLOC_185247	ENSRNOT00000000120	0.961010063	6.65E-07
TUCP_004219	XLOC_185247	ENSRNOT00000000469	0.955599071	1.26E-06
TUCP_004219	XLOC_185247	ENSRNOT00000033017	0.953993783	1.50E-06
TUCP_004219	XLOC_185247	ENSRNOT00000042125	0.954972272	1.35E-06
TUCP_004219	XLOC_185247	ENSRNOT00000082496	-0.959670083	7.85E-07
TUCP_004219	XLOC_185247	ENSRNOT00000074023	0.964309118	4.30E-07
TUCP_004219	XLOC_185247	ENSRNOT00000081688	-0.950317566	2.19E-06
TUCP_004219	XLOC_185247	ENSRNOT00000001691	0.951935156	1.86E-06
TUCP_004219	XLOC_185247	ENSRNOT00000004046	-0.956584194	1.13E-06
TUCP_004219	XLOC_185247	ENSRNOT00000058351	0.951609946	1.93E-06
TUCP_004219	XLOC_185247	ENSRNOT00000072118	0.958076222	9.51E-07
TUCP_004219	XLOC_185247	ENSRNOT00000075397	0.962007042	5.85E-07
TUCP_004219	XLOC_185247	ENSRNOT00000022190	-0.958161305	9.41E-07
TUCP_004219	XLOC_185247	ENSRNOT00000025913	-0.950817101	2.09E-06
TUCP_004219	XLOC_185247	ENSRNOT00000057953	0.960720499	6.89E-07
TUCP_004219	XLOC_185247	ENSRNOT00000024947	-0.954002242	1.50E-06
TUCP_004219	XLOC_185247	ENSRNOT00000068026	-0.953605299	1.57E-06
TUCP_004219	XLOC_185247	ENSRNOT00000051494	0.96663724	3.08E-07
TUCP_004219	XLOC_185247	ENSRNOT00000014926	0.971379951	1.44E-07
TUCP_004219	XLOC_185247	ENSRNOT00000016060	0.960389655	7.18E-07
TUCP_004219	XLOC_185247	ENSRNOT00000090057	0.975277602	6.98E-08
TUCP_004219	XLOC_185247	ENSRNOT00000027970	0.951136329	2.02E-06
TUCP_004219	XLOC_185247	ENSRNOT00000010367	0.961031001	6.63E-07
TUCP_004219	XLOC_185247	ENSRNOT00000086260	-0.959009857	8.51E-07
TUCP_004219	XLOC_185247	ENSRNOT00000055630	0.957937928	9.66E-07
TUCP_004219	XLOC_185247	ENSRNOT00000005257	0.974570181	8.03E-08
TUCP_004219	XLOC_185247	ENSRNOT00000089933	0.965419016	3.67E-07
TUCP_004219	XLOC_185247	ENSRNOT00000015799	0.953226073	1.63E-06
TUCP_004219	XLOC_185247	ENSRNOT00000088073	-0.950289657	2.20E-06
TUCP_004218	XLOC_185155	ENSRNOT00000059190	0.967310756	2.78E-07
TUCP_003055	XLOC_130485	ENSRNOT00000038879	0.994709243	3.24E-11
TUCP_004191	XLOC_183998	ENSRNOT00000042890	0.962941701	5.17E-07

TUCP_004191	XLOC_183998	ENSRNOT00000012757	0.95575527	1.24E-06
TUCP_004191	XLOC_183998	ENSRNOT00000080183	0.95911298	8.40E-07
TUCP_004191	XLOC_183998	ENSRNOT00000037902	0.950335086	2.19E-06
TUCP_004191	XLOC_183998	ENSRNOT00000005505	0.981046496	1.87E-08
TUCP_004191	XLOC_183998	ENSRNOT00000009975	0.952973022	1.67E-06
TUCP_004191	XLOC_183998	ENSRNOT00000072682	0.952109774	1.83E-06
TUCP_004191	XLOC_183998	ENSRNOT00000010142	0.965852098	3.45E-07
TUCP_004191	XLOC_183998	ENSRNOT00000047453	0.95770871	9.92E-07
TUCP_004191	XLOC_183998	ENSRNOT00000001642	0.976370054	5.58E-08
TUCP_004191	XLOC_183998	ENSRNOT00000075144	0.955692747	1.25E-06
TUCP_004191	XLOC_183998	ENSRNOT00000086144	0.959145047	8.37E-07
TUCP_004191	XLOC_183998	ENSRNOT00000006109	0.967849288	2.56E-07
TUCP_004191	XLOC_183998	ENSRNOT00000077025	0.951045759	2.04E-06
TUCP_003507	XLOC_152308	ENSRNOT00000077186	-0.955396405	1.29E-06
TUCP_003507	XLOC_152308	ENSRNOT00000081314	-0.951101245	2.03E-06
TUCP_003507	XLOC_152308	ENSRNOT00000039607	-0.955278401	1.31E-06
TUCP_003507	XLOC_152308	ENSRNOT00000013745	-0.956271238	1.17E-06
TUCP_003507	XLOC_152308	ENSRNOT00000006460	0.976910151	4.97E-08
TUCP_003507	XLOC_152308	ENSRNOT00000075397	0.968102188	2.47E-07
TUCP_003507	XLOC_152308	ENSRNOT00000062057	0.97049174	1.68E-07
TUCP_003507	XLOC_152308	ENSRNOT00000020051	0.958733469	8.79E-07
TUCP_003507	XLOC_152308	ENSRNOT00000026279	-0.960937312	6.71E-07
TUCP_003507	XLOC_152308	ENSRNOT00000089228	0.958399259	9.15E-07
TUCP_003507	XLOC_152308	ENSRNOT00000003087	0.954753257	1.38E-06
TUCP_003507	XLOC_152308	ENSRNOT00000082914	0.954686813	1.39E-06
TUCP_003507	XLOC_152308	ENSRNOT00000073493	0.963167802	5.02E-07
TUCP_003507	XLOC_152308	ENSRNOT00000001855	0.963909347	4.54E-07
TUCP_003507	XLOC_152308	ENSRNOT00000045443	-0.95062991	2.13E-06
TUCP_003507	XLOC_152308	ENSRNOT00000001691	0.977389463	4.48E-08
TUCP_003507	XLOC_152308	ENSRNOT00000089319	0.95423989	1.46E-06
TUCP_003507	XLOC_152308	ENSRNOT00000038178	-0.953603501	1.57E-06
TUCP_003507	XLOC_152308	ENSRNOT00000036585	0.954581796	1.41E-06
TUCP_003507	XLOC_152308	ENSRNOT00000027301	-0.958726309	8.80E-07
TUCP_003507	XLOC_152308	ENSRNOT00000065942	-0.969432376	2.00E-07
TUCP_003507	XLOC_152308	ENSRNOT00000026928	-0.951701242	1.91E-06
TUCP_003507	XLOC_152308	ENSRNOT00000086706	0.955702035	1.25E-06
TUCP_003507	XLOC_152308	ENSRNOT00000029508	0.96460841	4.12E-07
TUCP_003507	XLOC_152308	ENSRNOT00000016485	0.955345947	1.30E-06
TUCP_003507	XLOC_152308	ENSRNOT00000089829	-0.950411354	2.17E-06
TUCP_003507	XLOC_152308	ENSRNOT00000074023	0.965018754	3.89E-07
TUCP_003507	XLOC_152308	ENSRNOT00000019465	-0.958363432	9.19E-07
TUCP_003507	XLOC_152308	ENSRNOT00000008361	0.960109556	7.44E-07
TUCP_003507	XLOC_152308	ENSRNOT00000025309	0.967913901	2.54E-07
TUCP_003507	XLOC_152308	ENSRNOT00000013851	-0.962069375	5.80E-07
TUCP_003507	XLOC_152308	ENSRNOT00000042638	0.960457521	7.12E-07
TUCP_003507	XLOC_152308	ENSRNOT00000021826	0.962498836	5.48E-07
TUCP_003507	XLOC_152308	ENSRNOT00000028561	0.957481703	1.02E-06
TUCP_003507	XLOC_152308	ENSRNOT00000018101	-0.951891518	1.87E-06
TUCP_003507	XLOC_152308	ENSRNOT00000067566	-0.957222466	1.05E-06
TUCP_003507	XLOC_152308	ENSRNOT00000013516	0.958690562	8.84E-07
TUCP_003507	XLOC_152308	ENSRNOT00000003326	-0.956851313	1.10E-06

TUCP_003507	XLOC_152308	ENSRNOT00000015986	-0.964142564	4.40E-07
TUCP_003507	XLOC_152308	ENSRNOT00000065103	-0.953474189	1.59E-06
TUCP_003507	XLOC_152308	ENSRNOT00000025284	-0.966185936	3.29E-07
TUCP_003507	XLOC_152308	ENSRNOT00000087604	0.961641092	6.13E-07
TUCP_003507	XLOC_152308	ENSRNOT00000012060	0.966847272	2.98E-07
TUCP_003507	XLOC_152308	ENSRNOT00000073370	0.955513146	1.27E-06
TUCP_003507	XLOC_152308	ENSRNOT00000020929	0.958371968	9.18E-07
TUCP_003507	XLOC_152308	ENSRNOT00000054685	0.951414265	1.96E-06
TUCP_003507	XLOC_152308	ENSRNOT00000009009	0.959208556	8.30E-07
TUCP_003507	XLOC_152308	ENSRNOT00000065978	0.957287007	1.04E-06
TUCP_003507	XLOC_152308	ENSRNOT00000075480	-0.963425229	4.85E-07
TUCP_003507	XLOC_152308	ENSRNOT00000039177	-0.956477431	1.14E-06
TUCP_003507	XLOC_152308	ENSRNOT00000000707	-0.959048182	8.47E-07
TUCP_003507	XLOC_152308	ENSRNOT00000090338	-0.961977267	5.87E-07
TUCP_003507	XLOC_152308	ENSRNOT00000034282	0.958382907	9.17E-07
TUCP_003507	XLOC_152308	ENSRNOT00000027760	0.95363406	1.56E-06
TUCP_003507	XLOC_152308	ENSRNOT00000065871	0.956930151	1.09E-06
TUCP_003507	XLOC_152308	ENSRNOT00000042903	0.959677978	7.84E-07
TUCP_003507	XLOC_152308	ENSRNOT00000057898	-0.950009338	2.26E-06
TUCP_003507	XLOC_152308	ENSRNOT00000007821	0.952430914	1.77E-06
TUCP_003507	XLOC_152308	ENSRNOT00000056100	-0.972355072	1.21E-07
TUCP_003507	XLOC_152308	ENSRNOT00000001077	0.971212386	1.48E-07
TUCP_003507	XLOC_152308	ENSRNOT00000074150	0.950030134	2.26E-06
TUCP_003507	XLOC_152308	ENSRNOT00000036838	-0.959434386	8.08E-07
TUCP_003507	XLOC_152308	ENSRNOT00000067554	0.957615777	1.00E-06
TUCP_003507	XLOC_152308	ENSRNOT00000081226	0.9729887	1.08E-07
TUCP_003507	XLOC_152308	ENSRNOT00000011948	-0.952707453	1.72E-06
TUCP_003507	XLOC_152308	ENSRNOT00000086456	-0.964497854	4.18E-07
TUCP_003507	XLOC_152308	ENSRNOT00000040629	0.960243244	7.32E-07
TUCP_003507	XLOC_152308	ENSRNOT00000019121	-0.957627528	1.00E-06
TUCP_003507	XLOC_152308	ENSRNOT00000000048	0.964059002	4.45E-07
TUCP_003507	XLOC_152308	ENSRNOT00000075934	-0.967626076	2.65E-07
TUCP_003507	XLOC_152308	ENSRNOT00000040525	0.953468721	1.59E-06
TUCP_003507	XLOC_152308	ENSRNOT00000011832	-0.961500293	6.24E-07
TUCP_003507	XLOC_152308	ENSRNOT00000073643	-0.983190775	1.03E-08
TUCP_003507	XLOC_152308	ENSRNOT00000066893	0.951007901	2.05E-06
TUCP_003507	XLOC_152308	ENSRNOT00000026462	-0.955975545	1.21E-06
TUCP_003507	XLOC_152308	ENSRNOT00000092066	-0.957408332	1.03E-06
TUCP_003507	XLOC_152308	ENSRNOT00000083968	-0.968320767	2.38E-07
TUCP_003507	XLOC_152308	ENSRNOT00000037133	0.954103748	1.48E-06
TUCP_003507	XLOC_152308	ENSRNOT00000083967	0.962460723	5.51E-07
TUCP_003507	XLOC_152308	ENSRNOT00000083964	-0.963955091	4.51E-07
TUCP_003507	XLOC_152308	ENSRNOT00000016776	-0.956114449	1.19E-06
TUCP_003507	XLOC_152308	ENSRNOT00000020472	-0.950414413	2.17E-06
TUCP_003507	XLOC_152308	ENSRNOT00000048781	0.978105689	3.82E-08
TUCP_003507	XLOC_152308	ENSRNOT00000013867	0.969757608	1.89E-07
TUCP_003507	XLOC_152308	ENSRNOT00000000083	-0.959359247	8.15E-07
TUCP_003507	XLOC_152308	ENSRNOT00000021802	0.975362913	6.86E-08
TUCP_003507	XLOC_152308	ENSRNOT00000013864	-0.950685666	2.11E-06
TUCP_003507	XLOC_152308	ENSRNOT00000026831	0.952882481	1.69E-06
TUCP_003507	XLOC_152308	ENSRNOT00000018640	0.974590421	7.99E-08

TUCP_003507	XLOC_152308	ENSRNOT00000068663	0.954569259	1.41E-06
TUCP_003507	XLOC_152308	ENSRNOT00000082709	-0.956930087	1.09E-06
TUCP_003507	XLOC_152308	ENSRNOT00000072299	0.969169633	2.08E-07
TUCP_003507	XLOC_152308	ENSRNOT00000018122	0.957043711	1.07E-06
TUCP_003507	XLOC_152308	ENSRNOT00000032363	0.96872993	2.23E-07
TUCP_003507	XLOC_152308	ENSRNOT00000022194	-0.957120701	1.06E-06
TUCP_003507	XLOC_152308	ENSRNOT00000085334	0.958404367	9.14E-07
TUCP_003507	XLOC_152308	ENSRNOT00000000817	-0.950326408	2.19E-06
TUCP_003507	XLOC_152308	ENSRNOT00000084313	-0.959695695	7.83E-07
TUCP_003507	XLOC_152308	ENSRNOT00000001401	0.951945715	1.86E-06
TUCP_003507	XLOC_152308	ENSRNOT00000025913	-0.954176695	1.47E-06
TUCP_003507	XLOC_152308	ENSRNOT00000059246	0.952072457	1.84E-06
TUCP_003507	XLOC_152308	ENSRNOT00000056022	0.972029759	1.29E-07
TUCP_003507	XLOC_152308	ENSRNOT00000001360	-0.957913224	9.69E-07
TUCP_003507	XLOC_152308	ENSRNOT00000066888	-0.957352468	1.03E-06
TUCP_003507	XLOC_152308	ENSRNOT00000002151	-0.95615197	1.19E-06
TUCP_003507	XLOC_152308	ENSRNOT00000036658	-0.97203978	1.28E-07
TUCP_003507	XLOC_152308	ENSRNOT00000089611	0.957779618	9.84E-07
TUCP_003507	XLOC_152308	ENSRNOT00000017254	-0.953188292	1.64E-06
TUCP_003507	XLOC_152308	ENSRNOT00000079609	0.960968231	6.68E-07
TUCP_003507	XLOC_152308	ENSRNOT00000007738	-0.954310491	1.45E-06
TUCP_003507	XLOC_152308	ENSRNOT00000065629	0.954405859	1.44E-06
TUCP_003507	XLOC_152308	ENSRNOT00000016077	0.968107699	2.46E-07
TUCP_003507	XLOC_152308	ENSRNOT00000086260	-0.968067518	2.48E-07
TUCP_003507	XLOC_152308	ENSRNOT00000018328	-0.970177612	1.77E-07
TUCP_003507	XLOC_152308	ENSRNOT00000068044	0.953636056	1.56E-06
TUCP_003507	XLOC_152308	ENSRNOT00000076400	0.962467421	5.51E-07
TUCP_003507	XLOC_152308	ENSRNOT00000034411	0.961650031	6.13E-07
TUCP_003507	XLOC_152308	ENSRNOT00000007044	-0.953858021	1.52E-06
TUCP_003507	XLOC_152308	ENSRNOT00000081123	0.955514966	1.27E-06
TUCP_003507	XLOC_152308	ENSRNOT00000015769	0.959534191	7.98E-07
TUCP_003507	XLOC_152308	ENSRNOT00000035605	0.96823169	2.42E-07
TUCP_003507	XLOC_152308	ENSRNOT00000005172	0.964911425	3.95E-07
TUCP_003507	XLOC_152308	ENSRNOT00000084031	0.954269517	1.46E-06
TUCP_003507	XLOC_152308	ENSRNOT00000005170	-0.952220473	1.81E-06
TUCP_003507	XLOC_152308	ENSRNOT00000092083	0.968103896	2.46E-07
TUCP_003507	XLOC_152308	ENSRNOT00000021717	0.950990821	2.05E-06
TUCP_003507	XLOC_152308	ENSRNOT00000039911	0.957492798	1.02E-06
TUCP_003507	XLOC_152308	ENSRNOT00000071897	-0.957739998	9.89E-07
TUCP_003507	XLOC_152308	ENSRNOT00000004770	-0.959466782	8.05E-07
TUCP_003507	XLOC_152308	ENSRNOT00000029701	0.96749871	2.70E-07
TUCP_003507	XLOC_152308	ENSRNOT00000017907	-0.959646887	7.87E-07
TUCP_003507	XLOC_152308	ENSRNOT00000014585	-0.950343617	2.19E-06
TUCP_003507	XLOC_152308	ENSRNOT00000064901	0.954844797	1.37E-06
TUCP_003507	XLOC_152308	ENSRNOT00000068528	-0.963663665	4.69E-07
TUCP_003507	XLOC_152308	ENSRNOT00000011675	0.971566057	1.40E-07
TUCP_003507	XLOC_152308	ENSRNOT00000020794	0.952627483	1.73E-06
TUCP_003507	XLOC_152308	ENSRNOT00000065615	0.952892345	1.69E-06
TUCP_003507	XLOC_152308	ENSRNOT00000002574	0.957529269	1.01E-06
TUCP_003507	XLOC_152308	ENSRNOT00000023876	0.970912491	1.56E-07
TUCP_003507	XLOC_152308	ENSRNOT00000002974	0.9565451	1.13E-06

TUCP_003507	XLOC_152308	ENSRNOT00000066952	0.957838023	9.77E-07
TUCP_003507	XLOC_152308	ENSRNOT00000028685	0.961596447	6.17E-07
TUCP_003507	XLOC_152308	ENSRNOT00000000529	-0.956585731	1.13E-06
TUCP_003507	XLOC_152308	ENSRNOT00000083633	-0.956456276	1.15E-06
TUCP_003507	XLOC_152308	ENSRNOT00000049285	-0.966148774	3.31E-07
TUCP_003507	XLOC_152308	ENSRNOT00000087748	-0.953011022	1.67E-06
TUCP_003507	XLOC_152308	ENSRNOT00000006366	-0.951359144	1.98E-06
TUCP_003507	XLOC_152308	ENSRNOT00000088202	-0.964102273	4.42E-07
TUCP_003507	XLOC_152308	ENSRNOT00000017224	-0.956849571	1.10E-06
TUCP_003507	XLOC_152308	ENSRNOT00000005770	0.953435224	1.59E-06
TUCP_003507	XLOC_152308	ENSRNOT00000085911	-0.954359698	1.44E-06
TUCP_003507	XLOC_152308	ENSRNOT00000004999	0.950532435	2.15E-06
TUCP_003507	XLOC_152308	ENSRNOT00000013463	0.972847312	1.11E-07
TUCP_003507	XLOC_152308	ENSRNOT00000089673	-0.962053157	5.81E-07
TUCP_003507	XLOC_152308	ENSRNOT00000000886	0.962964738	5.16E-07
TUCP_003507	XLOC_152308	ENSRNOT00000057953	0.969290645	2.04E-07
TUCP_003507	XLOC_152308	ENSRNOT00000075209	-0.953296436	1.62E-06
TUCP_003507	XLOC_152308	ENSRNOT00000057738	0.964661378	4.09E-07
TUCP_003507	XLOC_152308	ENSRNOT00000006802	0.968937217	2.16E-07
TUCP_003507	XLOC_152308	ENSRNOT00000007889	-0.954883088	1.36E-06
TUCP_003507	XLOC_152308	ENSRNOT00000023977	-0.9541117	1.48E-06
TUCP_003507	XLOC_152308	ENSRNOT00000009202	0.96371937	4.66E-07
TUCP_003507	XLOC_152308	ENSRNOT00000047663	-0.958760549	8.76E-07
TUCP_003507	XLOC_152308	ENSRNOT00000082151	-0.957182925	1.05E-06
TUCP_003507	XLOC_152308	ENSRNOT00000011572	-0.958520419	9.02E-07
TUCP_003507	XLOC_152308	ENSRNOT00000067741	-0.954621979	1.40E-06
TUCP_003507	XLOC_152308	ENSRNOT00000000532	-0.964642029	4.10E-07
TUCP_003507	XLOC_152308	ENSRNOT00000005486	-0.950677476	2.12E-06
TUCP_003507	XLOC_152308	ENSRNOT00000083256	0.955155831	1.32E-06
TUCP_003507	XLOC_152308	ENSRNOT00000003691	-0.962152678	5.74E-07
TUCP_003507	XLOC_152308	ENSRNOT00000010852	0.974968669	7.42E-08
TUCP_003507	XLOC_152308	ENSRNOT00000025794	-0.960361742	7.21E-07
TUCP_003507	XLOC_152308	ENSRNOT00000014809	-0.954420344	1.43E-06
TUCP_003507	XLOC_152308	ENSRNOT00000058111	-0.964872171	3.97E-07
TUCP_003507	XLOC_152308	ENSRNOT00000014926	0.957755201	9.87E-07
TUCP_003507	XLOC_152308	ENSRNOT00000085971	-0.953206422	1.63E-06
TUCP_003507	XLOC_152308	ENSRNOT00000009993	-0.952383169	1.78E-06
TUCP_003507	XLOC_152308	ENSRNOT00000016637	-0.950222142	2.21E-06
TUCP_003507	XLOC_152308	ENSRNOT00000008987	-0.954530174	1.42E-06
TUCP_003507	XLOC_152308	ENSRNOT00000009994	-0.954769066	1.38E-06
TUCP_003507	XLOC_152308	ENSRNOT00000071192	-0.959880116	7.65E-07
TUCP_003507	XLOC_152308	ENSRNOT00000022716	0.962685357	5.35E-07
TUCP_003507	XLOC_152308	ENSRNOT00000002114	-0.955735332	1.24E-06
TUCP_003507	XLOC_152308	ENSRNOT00000091046	0.964816172	4.00E-07
TUCP_003507	XLOC_152308	ENSRNOT00000039221	-0.956227765	1.18E-06
TUCP_003507	XLOC_152308	ENSRNOT00000015624	0.950243096	2.21E-06
TUCP_003507	XLOC_152308	ENSRNOT00000017340	-0.959947338	7.59E-07
TUCP_003507	XLOC_152308	ENSRNOT00000073239	-0.952845055	1.70E-06
TUCP_003507	XLOC_152308	ENSRNOT00000044147	-0.968218726	2.42E-07
TUCP_003507	XLOC_152308	ENSRNOT00000030007	-0.956272411	1.17E-06
TUCP_003507	XLOC_152308	ENSRNOT00000046215	-0.973945117	9.05E-08

TUCP_003507	XLOC_152308	ENSRNOT00000039102	0.962613896	5.40E-07
TUCP_003507	XLOC_152308	ENSRNOT00000082138	-0.957433142	1.02E-06
TUCP_003507	XLOC_152308	ENSRNOT00000076467	-0.950843806	2.08E-06
TUCP_003507	XLOC_152308	ENSRNOT00000026518	0.958624815	8.91E-07
TUCP_003507	XLOC_152308	ENSRNOT00000039540	0.966857466	2.98E-07
TUCP_003507	XLOC_152308	ENSRNOT00000012625	-0.963764606	4.63E-07
TUCP_003507	XLOC_152308	ENSRNOT00000007369	0.950948462	2.06E-06
TUCP_003507	XLOC_152308	ENSRNOT00000080677	-0.959047893	8.47E-07
TUCP_003507	XLOC_152308	ENSRNOT00000085466	0.952249512	1.80E-06
TUCP_003507	XLOC_152308	ENSRNOT00000028545	-0.962097654	5.78E-07
TUCP_003507	XLOC_152308	ENSRNOT00000028225	0.969946701	1.84E-07
TUCP_003507	XLOC_152308	ENSRNOT00000045946	0.950997578	2.05E-06
TUCP_003507	XLOC_152308	ENSRNOT00000017998	0.95662075	1.12E-06
TUCP_003507	XLOC_152308	ENSRNOT00000027360	-0.970438392	1.69E-07
TUCP_003507	XLOC_152308	ENSRNOT00000060595	-0.954284822	1.46E-06
TUCP_003507	XLOC_152308	ENSRNOT00000013431	-0.954551829	1.41E-06
TUCP_003507	XLOC_152308	ENSRNOT00000015318	-0.980764627	2.01E-08
TUCP_003507	XLOC_152308	ENSRNOT00000045063	0.962506369	5.48E-07
TUCP_003507	XLOC_152308	ENSRNOT00000082805	0.951338312	1.98E-06
TUCP_003507	XLOC_152308	ENSRNOT00000027967	0.950891331	2.07E-06
TUCP_003507	XLOC_152308	ENSRNOT00000079128	0.960858072	6.78E-07
TUCP_003507	XLOC_152308	ENSRNOT00000045532	-0.957588034	1.01E-06
TUCP_003507	XLOC_152308	ENSRNOT00000079414	-0.952211702	1.81E-06
TUCP_003507	XLOC_152308	ENSRNOT00000035309	-0.951190307	2.01E-06
TUCP_003507	XLOC_152308	ENSRNOT00000024011	0.972790273	1.12E-07
TUCP_003507	XLOC_152308	ENSRNOT00000021537	0.950954421	2.06E-06
TUCP_003507	XLOC_152308	ENSRNOT00000061580	-0.954471295	1.43E-06
TUCP_003507	XLOC_152308	ENSRNOT00000010020	-0.961083724	6.58E-07
TUCP_003507	XLOC_152308	ENSRNOT00000005257	0.978193423	3.74E-08
TUCP_003507	XLOC_152308	ENSRNOT00000011326	-0.958622956	8.91E-07
TUCP_003507	XLOC_152308	ENSRNOT00000019473	-0.955057085	1.34E-06
TUCP_004377	XLOC_193231	ENSRNOT00000039045	0.963893808	4.55E-07
TUCP_004377	XLOC_193231	ENSRNOT00000013620	0.963887344	4.55E-07
TUCP_004377	XLOC_193231	ENSRNOT00000035700	0.951071968	2.03E-06
TUCP_004377	XLOC_193231	ENSRNOT00000006426	0.959780023	7.75E-07
TUCP_004377	XLOC_193231	ENSRNOT00000026755	0.963754641	4.64E-07
TUCP_004377	XLOC_193231	ENSRNOT00000078639	0.952010256	1.85E-06
TUCP_004377	XLOC_193231	ENSRNOT00000005977	0.956296389	1.17E-06
TUCP_004377	XLOC_193231	ENSRNOT00000079109	0.959458522	8.06E-07
TUCP_004377	XLOC_193231	ENSRNOT00000025394	0.959792259	7.74E-07
TUCP_004377	XLOC_193231	ENSRNOT00000004718	0.956874627	1.09E-06
TUCP_004377	XLOC_193231	ENSRNOT00000026121	0.960956708	6.69E-07
TUCP_004377	XLOC_193231	ENSRNOT00000078685	0.951287446	1.99E-06
TUCP_004377	XLOC_193231	ENSRNOT00000073636	0.962825866	5.25E-07
TUCP_004377	XLOC_193231	ENSRNOT00000078500	0.97569281	6.42E-08
TUCP_004377	XLOC_193231	ENSRNOT00000059458	0.966981137	2.92E-07
TUCP_004377	XLOC_193231	ENSRNOT00000022610	0.966392978	3.19E-07
TUCP_004377	XLOC_193231	ENSRNOT00000026065	0.953019651	1.67E-06
TUCP_004377	XLOC_193231	ENSRNOT00000027537	0.951328383	1.98E-06
TUCP_004377	XLOC_193231	ENSRNOT00000024060	0.954875837	1.37E-06
TUCP_004377	XLOC_193231	ENSRNOT00000010149	0.950094985	2.24E-06

TUCP_004377	XLOC_193231	ENSRNOT00000022726	0.950571429	2.14E-06
TUCP_004377	XLOC_193231	ENSRNOT00000046058	0.968514802	2.31E-07
TUCP_004377	XLOC_193231	ENSRNOT00000021153	0.961179025	6.51E-07
TUCP_004377	XLOC_193231	ENSRNOT00000025983	0.970741144	1.61E-07
TUCP_004377	XLOC_193231	ENSRNOT00000024281	0.954854359	1.37E-06
TUCP_004377	XLOC_193231	ENSRNOT00000077311	0.958339516	9.21E-07
TUCP_004377	XLOC_193231	ENSRNOT00000086336	0.950149674	2.23E-06
TUCP_004377	XLOC_193231	ENSRNOT00000010433	0.955968944	1.21E-06
TUCP_004377	XLOC_193231	ENSRNOT00000054955	-0.959296768	8.22E-07
TUCP_004377	XLOC_193231	ENSRNOT00000017968	0.957490132	1.02E-06
TUCP_004377	XLOC_193231	ENSRNOT00000048543	0.971109887	1.51E-07
TUCP_004377	XLOC_193231	ENSRNOT00000065921	0.956412897	1.15E-06
TUCP_004377	XLOC_193231	ENSRNOT00000013863	-0.955858041	1.23E-06
TUCP_004377	XLOC_193231	ENSRNOT00000066440	-0.965767171	3.50E-07
TUCP_004377	XLOC_193231	ENSRNOT00000010927	0.951001824	2.05E-06
TUCP_004377	XLOC_193231	ENSRNOT00000027414	0.957929429	9.67E-07
TUCP_004377	XLOC_193231	ENSRNOT00000028563	0.966871923	2.97E-07
TUCP_004377	XLOC_193231	ENSRNOT00000006195	0.965300534	3.74E-07
TUCP_004377	XLOC_193231	ENSRNOT00000028760	0.950192372	2.22E-06
TUCP_004377	XLOC_193231	ENSRNOT00000003158	-0.961241286	6.45E-07
TUCP_004377	XLOC_193231	ENSRNOT00000023180	0.950820848	2.09E-06
TUCP_004377	XLOC_193231	ENSRNOT00000076516	0.974729547	7.78E-08
TUCP_004377	XLOC_193231	ENSRNOT00000012560	0.968205466	2.43E-07
TUCP_004377	XLOC_193231	ENSRNOT00000030991	0.96411847	4.41E-07
TUCP_004377	XLOC_193231	ENSRNOT00000091807	0.961785276	6.02E-07
TUCP_004377	XLOC_193231	ENSRNOT00000085332	-0.950604839	2.13E-06
TUCP_004377	XLOC_193231	ENSRNOT00000012463	-0.977483233	4.39E-08
TUCP_004377	XLOC_193231	ENSRNOT00000019177	0.965581989	3.59E-07
TUCP_004377	XLOC_193231	ENSRNOT00000054874	0.952348762	1.79E-06
TUCP_004377	XLOC_193231	ENSRNOT00000013046	-0.964262664	4.32E-07
TUCP_004377	XLOC_193231	ENSRNOT00000011384	0.96470746	4.06E-07
TUCP_004377	XLOC_193231	ENSRNOT00000016948	-0.952832108	1.70E-06
TUCP_004377	XLOC_193231	ENSRNOT00000009198	0.963569617	4.75E-07
TUCP_004377	XLOC_193231	ENSRNOT00000012379	0.964622829	4.11E-07
TUCP_004377	XLOC_193231	ENSRNOT00000006828	0.972727972	1.13E-07
TUCP_004377	XLOC_193231	ENSRNOT00000009129	0.976910187	4.97E-08
TUCP_004377	XLOC_193231	ENSRNOT00000020437	0.962017639	5.84E-07
TUCP_004377	XLOC_193231	ENSRNOT00000087306	0.957867005	9.74E-07
TUCP_004377	XLOC_193231	ENSRNOT00000009877	0.954990004	1.35E-06
TUCP_004377	XLOC_193231	ENSRNOT00000006963	0.957124386	1.06E-06
TUCP_004377	XLOC_193231	ENSRNOT00000018990	0.950313761	2.19E-06
TUCP_004377	XLOC_193231	ENSRNOT00000036951	0.960547652	7.04E-07
TUCP_004377	XLOC_193231	ENSRNOT00000049538	0.967679448	2.63E-07
TUCP_004377	XLOC_193231	ENSRNOT00000044177	0.975593853	6.55E-08
TUCP_004377	XLOC_193231	ENSRNOT00000027944	0.972966365	1.09E-07
TUCP_004377	XLOC_193231	ENSRNOT00000018533	0.960326965	7.24E-07
TUCP_004377	XLOC_193231	ENSRNOT00000011663	0.958250551	9.31E-07
TUCP_004377	XLOC_193231	ENSRNOT00000087660	0.97169956	1.36E-07
TUCP_004377	XLOC_193231	ENSRNOT00000052075	0.967162812	2.85E-07
TUCP_004377	XLOC_193231	ENSRNOT00000093631	-0.97534625	6.88E-08
TUCP_004377	XLOC_193231	ENSRNOT00000026186	0.964233508	4.34E-07

TUCP_004377	XLOC_193231	ENSRNOT00000022947	-0.961039885	6.62E-07
TUCP_000731	XLOC_027261	ENSRNOT00000092026	0.96620614	3.28E-07
TUCP_000731	XLOC_027261	ENSRNOT00000093075	1	0
TUCP_000731	XLOC_027261	ENSRNOT00000058260	1	0
TUCP_000731	XLOC_027261	ENSRNOT00000088887	1	4.25E-78
TUCP_000731	XLOC_027261	ENSRNOT00000084986	1	4.25E-78
TUCP_000731	XLOC_027261	ENSRNOT00000074346	0.9702256	1.75E-07
TUCP_000731	XLOC_027261	ENSRNOT00000073209	0.983711602	8.79E-09
TUCP_000731	XLOC_027261	ENSRNOT00000080155	0.973069673	1.07E-07
TUCP_000731	XLOC_027261	ENSRNOT00000072573	0.996213272	6.09E-12
TUCP_000731	XLOC_027261	ENSRNOT00000017828	0.996001583	7.99E-12
TUCP_000731	XLOC_027261	ENSRNOT00000080444	0.969625907	1.93E-07
TUCP_000731	XLOC_027261	ENSRNOT00000071312	1	1.33E-79
TUCP_000731	XLOC_027261	ENSRNOT00000088245	1	4.25E-78
TUCP_000731	XLOC_027261	ENSRNOT00000036565	0.969065335	2.12E-07
TUCP_000731	XLOC_027261	ENSRNOT00000058833	0.998673261	3.23E-14
TUCP_000731	XLOC_027261	ENSRNOT00000078052	1	0
TUCP_000731	XLOC_027261	ENSRNOT00000078494	0.999927093	1.62E-20
TUCP_000731	XLOC_027261	ENSRNOT00000085653	1	0
TUCP_000731	XLOC_027261	ENSRNOT00000057950	0.969329371	2.03E-07
TUCP_000731	XLOC_027261	ENSRNOT00000081992	0.999320526	1.14E-15
TUCP_000731	XLOC_027261	ENSRNOT00000038553	0.955765808	1.24E-06
TUCP_000731	XLOC_027261	ENSRNOT00000084120	0.958115447	9.46E-07
TUCP_000731	XLOC_027261	ENSRNOT00000055406	1	1.33E-79
TUCP_000731	XLOC_027261	ENSRNOT00000070817	0.998968079	9.20E-15
TUCP_000731	XLOC_027261	ENSRNOT00000026753	1	1.33E-79
TUCP_000731	XLOC_027261	ENSRNOT00000080533	0.996682529	3.15E-12
TUCP_000731	XLOC_027261	ENSRNOT00000072774	0.979665261	2.65E-08
TUCP_000731	XLOC_027261	ENSRNOT00000086031	1	4.25E-78
TUCP_000731	XLOC_027261	ENSRNOT00000014312	1	0
TUCP_000731	XLOC_027261	ENSRNOT00000042882	0.963218427	4.98E-07
TUCP_000731	XLOC_027261	ENSRNOT00000088012	0.956765806	1.11E-06
TUCP_000731	XLOC_027261	ENSRNOT00000051231	0.999242378	1.96E-15
TUCP_000731	XLOC_027261	ENSRNOT00000089407	0.992986194	1.32E-10
TUCP_000731	XLOC_027261	ENSRNOT00000055034	0.998331441	1.02E-13
TUCP_000731	XLOC_027261	ENSRNOT00000046837	0.97252559	1.18E-07
TUCP_001797	XLOC_074266	ENSRNOT00000041541	-0.965966951	3.40E-07
TUCP_000583	XLOC_020714	ENSRNOT00000022389	0.96917649	2.08E-07
TUCP_000583	XLOC_020714	ENSRNOT00000040116	0.965485949	3.64E-07
TUCP_000583	XLOC_020714	ENSRNOT00000011869	0.976773649	5.12E-08
TUCP_000583	XLOC_020714	ENSRNOT00000034401	0.959549369	7.97E-07
TUCP_000583	XLOC_020714	ENSRNOT00000077451	0.951305683	1.99E-06
TUCP_000583	XLOC_020714	ENSRNOT00000021506	0.962515031	5.47E-07
TUCP_000583	XLOC_020714	ENSRNOT00000019121	0.955004058	1.35E-06
TUCP_000583	XLOC_020714	ENSRNOT00000050063	0.951067639	2.03E-06
TUCP_000583	XLOC_020714	ENSRNOT00000006172	0.96410479	4.42E-07
TUCP_000583	XLOC_020714	ENSRNOT00000017340	0.953940298	1.51E-06
TUCP_000583	XLOC_020714	ENSRNOT00000018494	0.961673354	6.11E-07
TUCP_000583	XLOC_020714	ENSRNOT00000023067	0.970709103	1.62E-07
TUCP_000583	XLOC_020714	ENSRNOT00000074261	0.95660261	1.13E-06
TUCP_000583	XLOC_020714	ENSRNOT00000000527	0.967294577	2.79E-07

TUCP_000583	XLOC_020714	ENSRNOT00000039000	0.957084582	1.07E-06
TUCP_000583	XLOC_020714	ENSRNOT00000016265	0.950851612	2.08E-06
TUCP_000583	XLOC_020714	ENSRNOT00000001050	0.963852122	4.57E-07
TUCP_000583	XLOC_020714	ENSRNOT00000085581	0.952156772	1.82E-06
TUCP_000583	XLOC_020714	ENSRNOT00000002853	0.954053309	1.49E-06
TUCP_000583	XLOC_020714	ENSRNOT00000073521	0.982439197	1.28E-08
TUCP_000583	XLOC_020714	ENSRNOT00000000528	0.965332137	3.72E-07
TUCP_000583	XLOC_020714	ENSRNOT00000074629	0.957213121	1.05E-06
TUCP_000583	XLOC_020714	ENSRNOT00000027220	0.962922394	5.19E-07
TUCP_000583	XLOC_020714	ENSRNOT00000023452	0.971769157	1.35E-07
TUCP_000583	XLOC_020714	ENSRNOT00000028527	0.958609806	8.92E-07
TUCP_000583	XLOC_020714	ENSRNOT00000008813	0.951322059	1.98E-06
TUCP_000583	XLOC_020714	ENSRNOT00000017224	0.954674464	1.40E-06
TUCP_000583	XLOC_020714	ENSRNOT00000086007	0.963438638	4.84E-07
TUCP_000583	XLOC_020714	ENSRNOT00000073388	0.9557202	1.24E-06
TUCP_000583	XLOC_020714	ENSRNOT00000014317	0.955172694	1.32E-06
TUCP_000583	XLOC_020714	ENSRNOT00000041151	0.970215817	1.76E-07
TUCP_000583	XLOC_020714	ENSRNOT00000004770	0.95356819	1.57E-06
TUCP_000583	XLOC_020714	ENSRNOT00000000134	0.960376608	7.20E-07
TUCP_000705	XLOC_026751	ENSRNOT00000079808	-0.955613246	1.26E-06
TUCP_001197	XLOC_045075	ENSRNOT00000047560	0.957755962	9.87E-07
TUCP_003689	XLOC_161313	ENSRNOT00000031904	0.971945427	1.31E-07
TUCP_003689	XLOC_161313	ENSRNOT00000067400	0.957613238	1.00E-06
TUCP_003689	XLOC_161313	ENSRNOT00000039809	0.961430359	6.30E-07
TUCP_003689	XLOC_161313	ENSRNOT00000000743	0.974294142	8.47E-08
TUCP_003689	XLOC_161313	ENSRNOT00000027083	0.969977621	1.83E-07
TUCP_003689	XLOC_161313	ENSRNOT00000048914	0.952018339	1.85E-06
TUCP_003689	XLOC_161313	ENSRNOT00000013507	0.951093641	2.03E-06
TUCP_003689	XLOC_161313	ENSRNOT00000023638	0.954312596	1.45E-06
TUCP_003689	XLOC_161313	ENSRNOT00000006175	0.95078185	2.09E-06
TUCP_003689	XLOC_161313	ENSRNOT00000021592	0.960843039	6.79E-07
TUCP_003689	XLOC_161313	ENSRNOT00000028625	-0.952213077	1.81E-06
TUCP_003689	XLOC_161313	ENSRNOT00000023144	0.962102376	5.78E-07
TUCP_003689	XLOC_161313	ENSRNOT00000082869	0.965516725	3.62E-07
TUCP_003689	XLOC_161313	ENSRNOT00000016649	0.964203455	4.36E-07
TUCP_003689	XLOC_161313	ENSRNOT00000026941	0.955525218	1.27E-06
TUCP_003689	XLOC_161313	ENSRNOT00000013758	0.960241904	7.32E-07
TUCP_003689	XLOC_161313	ENSRNOT00000026543	0.970326988	1.72E-07
TUCP_003689	XLOC_161313	ENSRNOT00000085251	-0.950234535	2.21E-06
TUCP_003689	XLOC_161313	ENSRNOT00000025075	0.955979858	1.21E-06
TUCP_003689	XLOC_161313	ENSRNOT00000035989	0.983777881	8.61E-09
TUCP_003689	XLOC_161313	ENSRNOT00000057160	0.974719289	7.79E-08
TUCP_003689	XLOC_161313	ENSRNOT00000082699	0.977333305	4.54E-08
TUCP_003689	XLOC_161313	ENSRNOT00000028847	0.95092603	2.06E-06
TUCP_003689	XLOC_161313	ENSRNOT00000010242	0.953952369	1.51E-06
TUCP_003689	XLOC_161313	ENSRNOT00000091602	-0.950324484	2.19E-06
TUCP_003689	XLOC_161313	ENSRNOT00000084012	0.955432947	1.28E-06
TUCP_003689	XLOC_161313	ENSRNOT00000002560	0.964177166	4.37E-07
TUCP_003689	XLOC_161313	ENSRNOT00000051294	0.952430512	1.77E-06
TUCP_003689	XLOC_161313	ENSRNOT00000041513	0.95611724	1.19E-06
TUCP_003689	XLOC_161313	ENSRNOT00000091909	0.950368883	2.18E-06

TUCP_003689	XLOC_161313	ENSRNOT00000041044	0.961912831	5.92E-07
TUCP_003689	XLOC_161313	ENSRNOT00000010541	0.962518986	5.47E-07
TUCP_003689	XLOC_161313	ENSRNOT00000012895	0.952353027	1.78E-06
TUCP_003689	XLOC_161313	ENSRNOT00000025859	0.951059831	2.04E-06
TUCP_003689	XLOC_161313	ENSRNOT00000013026	0.9505678	2.14E-06
TUCP_003689	XLOC_161313	ENSRNOT00000034503	0.962393767	5.56E-07
TUCP_003689	XLOC_161313	ENSRNOT00000085027	0.954382803	1.44E-06
TUCP_003689	XLOC_161313	ENSRNOT00000010898	0.96845796	2.33E-07
TUCP_003689	XLOC_161313	ENSRNOT00000047093	0.952053579	1.84E-06
TUCP_003689	XLOC_161313	ENSRNOT00000023744	0.96946642	1.99E-07
TUCP_003689	XLOC_161313	ENSRNOT00000005355	0.959637173	7.88E-07
TUCP_003689	XLOC_161313	ENSRNOT00000048063	0.952699463	1.72E-06
TUCP_003689	XLOC_161313	ENSRNOT00000016739	0.956933307	1.09E-06
TUCP_003689	XLOC_161313	ENSRNOT00000081881	0.951677778	1.91E-06
TUCP_003689	XLOC_161313	ENSRNOT00000044608	-0.957387895	1.03E-06
TUCP_003689	XLOC_161313	ENSRNOT00000025981	0.974779917	7.70E-08
TUCP_003689	XLOC_161313	ENSRNOT00000047387	0.953039595	1.66E-06
TUCP_003689	XLOC_161313	ENSRNOT00000021769	0.957058621	1.07E-06
TUCP_003689	XLOC_161313	ENSRNOT00000088599	0.957937786	9.66E-07
TUCP_003689	XLOC_161313	ENSRNOT00000081047	0.950923491	2.06E-06
TUCP_003689	XLOC_161313	ENSRNOT00000017629	0.955214222	1.32E-06
TUCP_003689	XLOC_161313	ENSRNOT00000003991	0.959429445	8.09E-07
TUCP_003689	XLOC_161313	ENSRNOT00000087429	0.953450973	1.59E-06
TUCP_003689	XLOC_161313	ENSRNOT00000079072	0.96477091	4.03E-07
TUCP_003689	XLOC_161313	ENSRNOT00000084088	0.973657767	9.56E-08
TUCP_003689	XLOC_161313	ENSRNOT00000078070	0.960406681	7.17E-07
TUCP_003689	XLOC_161313	ENSRNOT00000005262	0.950451091	2.16E-06
TUCP_003689	XLOC_161313	ENSRNOT00000076729	0.975769274	6.32E-08
TUCP_003689	XLOC_161313	ENSRNOT00000090665	0.954726904	1.39E-06
TUCP_003689	XLOC_161313	ENSRNOT00000078390	-0.950197831	2.22E-06
TUCP_003689	XLOC_161313	ENSRNOT00000065501	0.953109701	1.65E-06
TUCP_003689	XLOC_161313	ENSRNOT00000025936	-0.956324598	1.16E-06
TUCP_003689	XLOC_161313	ENSRNOT00000050180	0.956672925	1.12E-06
TUCP_003689	XLOC_161313	ENSRNOT00000090420	-0.959607483	7.91E-07
TUCP_003689	XLOC_161313	ENSRNOT00000001222	0.956574431	1.13E-06
TUCP_003689	XLOC_161313	ENSRNOT00000017422	-0.954277225	1.46E-06
TUCP_003689	XLOC_161313	ENSRNOT00000010992	0.960577559	7.02E-07
TUCP_003689	XLOC_161313	ENSRNOT00000076669	0.977026859	4.85E-08
TUCP_003689	XLOC_161313	ENSRNOT00000085596	0.955351125	1.30E-06
TUCP_003689	XLOC_161313	ENSRNOT00000038807	0.954373969	1.44E-06
TUCP_003689	XLOC_161313	ENSRNOT00000073675	-0.956298312	1.17E-06
TUCP_003689	XLOC_161313	ENSRNOT00000025808	0.950879165	2.07E-06
TUCP_003689	XLOC_161313	ENSRNOT00000050834	-0.962100299	5.78E-07
TUCP_003689	XLOC_161313	ENSRNOT00000066024	0.977146624	4.72E-08
TUCP_003689	XLOC_161313	ENSRNOT00000021471	-0.958508117	9.03E-07
TUCP_003689	XLOC_161313	ENSRNOT00000041096	0.95276955	1.71E-06
TUCP_003689	XLOC_161313	ENSRNOT00000080110	0.961302222	6.40E-07
TUCP_003689	XLOC_161313	ENSRNOT00000078306	0.967883015	2.55E-07
TUCP_003689	XLOC_161313	ENSRNOT00000080695	0.974418789	8.27E-08
TUCP_003689	XLOC_161313	ENSRNOT00000075279	0.953297779	1.62E-06
TUCP_003689	XLOC_161313	ENSRNOT00000060696	0.964583518	4.13E-07

TUCP_003689	XLOC_161313	ENSRNOT00000042125	0.95633433	1.16E-06
TUCP_003689	XLOC_161313	ENSRNOT00000009900	-0.954163915	1.48E-06
TUCP_003689	XLOC_161313	ENSRNOT00000081400	0.955582796	1.26E-06
TUCP_003689	XLOC_161313	ENSRNOT00000004412	0.952396413	1.78E-06
TUCP_003689	XLOC_161313	ENSRNOT00000067181	0.972454626	1.19E-07
TUCP_003689	XLOC_161313	ENSRNOT00000080207	0.950097516	2.24E-06
TUCP_003689	XLOC_161313	ENSRNOT00000034704	0.955728719	1.24E-06
TUCP_003689	XLOC_161313	ENSRNOT00000051496	0.96207574	5.80E-07
TUCP_003689	XLOC_161313	ENSRNOT00000067904	0.959096377	8.42E-07
TUCP_003689	XLOC_161313	ENSRNOT00000011041	0.951415974	1.96E-06
TUCP_003689	XLOC_161313	ENSRNOT00000047192	-0.962806807	5.27E-07
TUCP_003689	XLOC_161313	ENSRNOT00000083799	0.955846574	1.23E-06
TUCP_003503	XLOC_151982	ENSRNOT00000000474	0.952518507	1.75E-06
TUCP_003503	XLOC_151982	ENSRNOT00000007144	0.964531973	4.16E-07
TUCP_003503	XLOC_151982	ENSRNOT00000059157	0.952684631	1.72E-06
TUCP_003503	XLOC_151982	ENSRNOT00000019630	0.951856121	1.88E-06
TUCP_003503	XLOC_151982	ENSRNOT00000087355	0.957900183	9.70E-07
TUCP_003503	XLOC_151982	ENSRNOT00000084912	0.958833331	8.69E-07
TUCP_003503	XLOC_151982	ENSRNOT00000028075	0.952654815	1.73E-06
TUCP_003503	XLOC_151982	ENSRNOT00000047179	0.966935459	2.94E-07
TUCP_003503	XLOC_151982	ENSRNOT00000086812	0.956583266	1.13E-06
TUCP_003503	XLOC_151982	ENSRNOT00000091065	0.956207756	1.18E-06
TUCP_003503	XLOC_151982	ENSRNOT00000001912	0.956988706	1.08E-06
TUCP_003503	XLOC_151982	ENSRNOT00000045049	0.957144871	1.06E-06
TUCP_003882	XLOC_170691	ENSRNOT00000092026	0.966196429	3.28E-07
TUCP_003882	XLOC_170691	ENSRNOT00000093075	0.999999982	1.41E-38
TUCP_003882	XLOC_170691	ENSRNOT00000058260	0.999999982	1.41E-38
TUCP_003882	XLOC_170691	ENSRNOT00000088887	0.999999982	1.41E-38
TUCP_003882	XLOC_170691	ENSRNOT00000084986	0.999999982	1.41E-38
TUCP_003882	XLOC_170691	ENSRNOT00000074346	0.970213101	1.76E-07
TUCP_003882	XLOC_170691	ENSRNOT00000073209	0.983705147	8.80E-09
TUCP_003882	XLOC_170691	ENSRNOT00000080155	0.973060741	1.07E-07
TUCP_003882	XLOC_170691	ENSRNOT00000072573	0.996212824	6.10E-12
TUCP_003882	XLOC_170691	ENSRNOT00000017828	0.996014699	7.86E-12
TUCP_003882	XLOC_170691	ENSRNOT00000080444	0.969650743	1.93E-07
TUCP_003882	XLOC_170691	ENSRNOT00000071312	0.999999982	1.41E-38
TUCP_003882	XLOC_170691	ENSRNOT00000088245	0.999999982	1.41E-38
TUCP_003882	XLOC_170691	ENSRNOT00000036565	0.969078919	2.11E-07
TUCP_003882	XLOC_170691	ENSRNOT00000058833	0.998671813	3.25E-14
TUCP_003882	XLOC_170691	ENSRNOT00000078052	0.999999982	1.41E-38
TUCP_003882	XLOC_170691	ENSRNOT00000078494	0.99992885	1.44E-20
TUCP_003882	XLOC_170691	ENSRNOT00000085653	0.999999982	1.41E-38
TUCP_003882	XLOC_170691	ENSRNOT00000057950	0.969364372	2.02E-07
TUCP_003882	XLOC_170691	ENSRNOT00000081992	0.999320772	1.14E-15
TUCP_003882	XLOC_170691	ENSRNOT00000038553	0.955754164	1.24E-06
TUCP_003882	XLOC_170691	ENSRNOT00000084120	0.95810184	9.48E-07
TUCP_003882	XLOC_170691	ENSRNOT00000055406	0.999999982	1.41E-38
TUCP_003882	XLOC_170691	ENSRNOT00000070817	0.998972736	8.99E-15
TUCP_003882	XLOC_170691	ENSRNOT00000026753	0.999999982	1.41E-38
TUCP_003882	XLOC_170691	ENSRNOT00000080533	0.996680251	3.16E-12
TUCP_003882	XLOC_170691	ENSRNOT00000072774	0.979694742	2.63E-08

TUCP_003882	XLOC_170691	ENSRNOT00000086031	0.999999982	1.41E-38
TUCP_003882	XLOC_170691	ENSRNOT00000014312	0.999999982	1.41E-38
TUCP_003882	XLOC_170691	ENSRNOT00000042882	0.96322829	4.98E-07
TUCP_003882	XLOC_170691	ENSRNOT00000088012	0.956808551	1.10E-06
TUCP_003882	XLOC_170691	ENSRNOT00000051231	0.999241279	1.98E-15
TUCP_003882	XLOC_170691	ENSRNOT00000089407	0.992982889	1.32E-10
TUCP_003882	XLOC_170691	ENSRNOT00000055034	0.998332806	1.01E-13
TUCP_003882	XLOC_170691	ENSRNOT00000046837	0.972534573	1.18E-07
TUCP_001106	XLOC_042098	ENSRNOT00000076400	0.961477936	6.26E-07
TUCP_001106	XLOC_042098	ENSRNOT00000055536	0.98080685	1.99E-08
TUCP_001106	XLOC_042098	ENSRNOT00000010680	0.956495137	1.14E-06
TUCP_001106	XLOC_042098	ENSRNOT00000010852	0.964882477	3.97E-07
TUCP_001106	XLOC_042098	ENSRNOT00000009975	0.975317743	6.92E-08
TUCP_001106	XLOC_042098	ENSRNOT00000072682	0.970692794	1.62E-07
TUCP_001106	XLOC_042098	ENSRNOT00000061054	0.969322065	2.03E-07
TUCP_001106	XLOC_042098	ENSRNOT00000081226	0.964826842	4.00E-07
TUCP_001106	XLOC_042098	ENSRNOT00000001642	0.951417544	1.96E-06
TUCP_001106	XLOC_042098	ENSRNOT00000022309	0.965705591	3.53E-07
TUCP_001106	XLOC_042098	ENSRNOT00000081631	0.975265886	6.99E-08
TUCP_001106	XLOC_042098	ENSRNOT00000017829	0.952583143	1.74E-06
TUCP_001106	XLOC_042098	ENSRNOT00000039911	0.964793541	4.02E-07
TUCP_001106	XLOC_042098	ENSRNOT00000026518	0.962403029	5.55E-07
TUCP_001106	XLOC_042098	ENSRNOT00000006071	0.951168192	2.01E-06
TUCP_001106	XLOC_042098	ENSRNOT00000028624	0.960738327	6.88E-07
TUCP_001106	XLOC_042098	ENSRNOT00000065801	0.958344987	9.21E-07
TUCP_001106	XLOC_042098	ENSRNOT00000088385	0.95495925	1.35E-06
TUCP_001106	XLOC_042098	ENSRNOT00000016485	0.963586795	4.74E-07
TUCP_001106	XLOC_042098	ENSRNOT00000079152	0.950087783	2.24E-06
TUCP_001106	XLOC_042098	ENSRNOT00000073493	0.963410914	4.86E-07
TUCP_001106	XLOC_042098	ENSRNOT00000042638	0.950250519	2.21E-06
TUCP_001106	XLOC_042098	ENSRNOT00000014382	0.956272871	1.17E-06
TUCP_001106	XLOC_042098	ENSRNOT00000087521	0.959787931	7.74E-07
TUCP_001106	XLOC_042098	ENSRNOT00000005770	0.960814965	6.81E-07
TUCP_001106	XLOC_042098	ENSRNOT00000090700	0.969692939	1.91E-07
TUCP_001106	XLOC_042098	ENSRNOT00000027476	0.952419097	1.77E-06
TUCP_001106	XLOC_042098	ENSRNOT00000036137	0.951364338	1.97E-06
TUCP_001106	XLOC_042098	ENSRNOT00000085334	0.973980022	8.99E-08
TUCP_001106	XLOC_042098	ENSRNOT00000011589	0.958750025	8.78E-07
TUCP_001106	XLOC_042098	ENSRNOT00000064122	0.957997482	9.59E-07
TUCP_001106	XLOC_042098	ENSRNOT00000057295	0.958487015	9.05E-07
TUCP_001106	XLOC_042098	ENSRNOT00000008764	0.977697767	4.19E-08
TUCP_001106	XLOC_042098	ENSRNOT00000009009	0.956549416	1.13E-06
TUCP_001106	XLOC_042098	ENSRNOT00000020929	0.961932649	5.91E-07
TUCP_001106	XLOC_042098	ENSRNOT00000083134	0.954458567	1.43E-06
TUCP_001106	XLOC_042098	ENSRNOT00000006109	0.950336898	2.19E-06
TUCP_001106	XLOC_042098	ENSRNOT00000033933	0.954817572	1.37E-06
TUCP_001106	XLOC_042098	ENSRNOT00000009398	0.95422028	1.47E-06
TUCP_001106	XLOC_042098	ENSRNOT00000074131	0.95525256	1.31E-06
TUCP_001106	XLOC_042098	ENSRNOT00000010369	0.961965933	5.88E-07
TUCP_001106	XLOC_042098	ENSRNOT00000065629	0.969536735	1.96E-07
TUCP_001106	XLOC_042098	ENSRNOT00000078800	0.955724147	1.24E-06

TUCP_001107	XLOC_042133	ENSRNOT00000026949	-0.953580986	1.57E-06
TUCP_001107	XLOC_042133	ENSRNOT00000005909	0.952866523	1.69E-06
TUCP_001107	XLOC_042133	ENSRNOT00000006549	-0.956728133	1.11E-06
TUCP_001107	XLOC_042133	ENSRNOT00000083215	0.957782565	9.84E-07
TUCP_001107	XLOC_042133	ENSRNOT00000015325	-0.958959201	8.56E-07
TUCP_001107	XLOC_042133	ENSRNOT00000005977	0.950462074	2.16E-06
TUCP_001107	XLOC_042133	ENSRNOT00000003834	-0.970179175	1.77E-07
TUCP_001107	XLOC_042133	ENSRNOT00000025394	0.969276396	2.05E-07
TUCP_001107	XLOC_042133	ENSRNOT00000045083	0.96237169	5.58E-07
TUCP_001107	XLOC_042133	ENSRNOT00000013050	-0.967761363	2.60E-07
TUCP_001107	XLOC_042133	ENSRNOT00000074653	0.955422006	1.29E-06
TUCP_001107	XLOC_042133	ENSRNOT00000046364	0.952096472	1.83E-06
TUCP_001107	XLOC_042133	ENSRNOT00000073636	0.962386707	5.57E-07
TUCP_001107	XLOC_042133	ENSRNOT00000078500	0.95152002	1.94E-06
TUCP_001107	XLOC_042133	ENSRNOT00000072416	-0.955887206	1.22E-06
TUCP_001107	XLOC_042133	ENSRNOT00000067822	0.950148983	2.23E-06
TUCP_001107	XLOC_042133	ENSRNOT00000012056	0.964287793	4.31E-07
TUCP_001107	XLOC_042133	ENSRNOT00000024060	0.976094986	5.91E-08
TUCP_001107	XLOC_042133	ENSRNOT00000013031	-0.976599517	5.31E-08
TUCP_001107	XLOC_042133	ENSRNOT00000022726	0.956040034	1.20E-06
TUCP_001107	XLOC_042133	ENSRNOT00000023645	0.956734235	1.11E-06
TUCP_001107	XLOC_042133	ENSRNOT00000046058	0.960062962	7.48E-07
TUCP_001107	XLOC_042133	ENSRNOT00000021153	0.953799572	1.53E-06
TUCP_001107	XLOC_042133	ENSRNOT00000017476	-0.951206203	2.01E-06
TUCP_001107	XLOC_042133	ENSRNOT00000007164	0.958412801	9.13E-07
TUCP_001107	XLOC_042133	ENSRNOT00000064900	0.973332182	1.02E-07
TUCP_001107	XLOC_042133	ENSRNOT00000086548	0.951781942	1.89E-06
TUCP_001107	XLOC_042133	ENSRNOT00000004942	-0.95647787	1.14E-06
TUCP_001107	XLOC_042133	ENSRNOT00000090170	0.982491773	1.26E-08
TUCP_001107	XLOC_042133	ENSRNOT00000088995	0.954521875	1.42E-06
TUCP_001107	XLOC_042133	ENSRNOT00000054955	-0.955793367	1.23E-06
TUCP_001107	XLOC_042133	ENSRNOT00000017884	0.95456657	1.41E-06
TUCP_001107	XLOC_042133	ENSRNOT00000068633	0.968299261	2.39E-07
TUCP_001107	XLOC_042133	ENSRNOT00000025550	-0.954241764	1.46E-06
TUCP_001107	XLOC_042133	ENSRNOT00000058645	0.960796334	6.83E-07
TUCP_001107	XLOC_042133	ENSRNOT00000017948	0.952898064	1.69E-06
TUCP_001107	XLOC_042133	ENSRNOT00000057814	0.953272038	1.62E-06
TUCP_001107	XLOC_042133	ENSRNOT00000009129	0.952735835	1.72E-06
TUCP_001107	XLOC_042133	ENSRNOT00000022534	0.957848478	9.76E-07
TUCP_001107	XLOC_042133	ENSRNOT00000039423	-0.954614383	1.41E-06
TUCP_001107	XLOC_042133	ENSRNOT00000091864	-0.950382112	2.18E-06
TUCP_001107	XLOC_042133	ENSRNOT00000028563	0.951594988	1.93E-06
TUCP_001107	XLOC_042133	ENSRNOT00000013914	-0.956817534	1.10E-06
TUCP_001107	XLOC_042133	ENSRNOT00000060530	0.960453591	7.13E-07
TUCP_001107	XLOC_042133	ENSRNOT00000064320	0.963376279	4.88E-07
TUCP_001107	XLOC_042133	ENSRNOT00000043554	0.954920335	1.36E-06
TUCP_001107	XLOC_042133	ENSRNOT00000049538	0.953385528	1.60E-06
TUCP_001107	XLOC_042133	ENSRNOT00000026065	0.97185083	1.33E-07
TUCP_001107	XLOC_042133	ENSRNOT00000009963	0.95384077	1.53E-06
TUCP_001107	XLOC_042133	ENSRNOT00000015076	0.967166949	2.84E-07
TUCP_001107	XLOC_042133	ENSRNOT00000013046	-0.97680323	5.09E-08

TUCP_001107	XLOC_042133	ENSRNOT00000087725	0.959298215	8.22E-07
TUCP_001107	XLOC_042133	ENSRNOT00000011384	0.955040355	1.34E-06
TUCP_001107	XLOC_042133	ENSRNOT00000084595	0.979880521	2.51E-08
TUCP_001107	XLOC_042133	ENSRNOT00000093640	0.957525772	1.01E-06
TUCP_001107	XLOC_042133	ENSRNOT00000010433	0.95986701	7.66E-07
TUCP_001107	XLOC_042133	ENSRNOT00000022213	0.956479266	1.14E-06
TUCP_001107	XLOC_042133	ENSRNOT00000066593	0.973617743	9.63E-08
TUCP_001107	XLOC_042133	ENSRNOT00000020437	0.951795146	1.89E-06
TUCP_001107	XLOC_042133	ENSRNOT00000015635	-0.951090347	2.03E-06
TUCP_001107	XLOC_042133	ENSRNOT00000082691	0.98398228	8.08E-09
TUCP_001107	XLOC_042133	ENSRNOT00000002173	-0.951603323	1.93E-06
TUCP_001107	XLOC_042133	ENSRNOT00000044237	0.95019341	2.22E-06
TUCP_001107	XLOC_042133	ENSRNOT00000027944	0.969797591	1.88E-07
TUCP_001107	XLOC_042133	ENSRNOT00000066968	0.954854176	1.37E-06
TUCP_001107	XLOC_042133	ENSRNOT00000028842	0.950170272	2.22E-06
TUCP_001107	XLOC_042133	ENSRNOT00000048029	0.959593026	7.93E-07
TUCP_001107	XLOC_042133	ENSRNOT00000091336	0.951569165	1.93E-06
TUCP_001107	XLOC_042133	ENSRNOT00000027828	0.963621972	4.72E-07
TUCP_001107	XLOC_042133	ENSRNOT00000088196	0.964169424	4.38E-07
TUCP_001107	XLOC_042133	ENSRNOT00000052075	0.958074278	9.51E-07
TUCP_001107	XLOC_042133	ENSRNOT00000011325	0.958912745	8.61E-07
TUCP_001107	XLOC_042133	ENSRNOT00000005389	0.963653538	4.70E-07
TUCP_001107	XLOC_042133	ENSRNOT00000078685	0.970631089	1.64E-07
TUCP_001107	XLOC_042133	ENSRNOT00000019553	0.96851313	2.31E-07
TUCP_001107	XLOC_042133	ENSRNOT00000082855	0.955952423	1.21E-06
TUCP_001104	XLOC_041816	ENSRNOT00000084075	0.996106112	7.00E-12
TUCP_001104	XLOC_041816	ENSRNOT00000012880	0.968043129	2.49E-07
TUCP_001104	XLOC_041816	ENSRNOT00000074465	1	0
TUCP_001104	XLOC_041816	ENSRNOT00000090898	0.961670541	6.11E-07
TUCP_001104	XLOC_041816	ENSRNOT00000079805	0.997980451	2.64E-13
TUCP_001104	XLOC_041816	ENSRNOT00000040912	1	4.25E-78
TUCP_001104	XLOC_041816	ENSRNOT00000072648	1	4.25E-78
TUCP_001104	XLOC_041816	ENSRNOT00000007398	0.989054103	1.21E-09
TUCP_001104	XLOC_041816	ENSRNOT00000070803	1	4.25E-78
TUCP_001104	XLOC_041816	ENSRNOT00000012196	1	0
TUCP_001104	XLOC_041816	ENSRNOT00000085772	1	4.25E-78
TUCP_001104	XLOC_041816	ENSRNOT00000093284	0.999975856	6.46E-23
TUCP_001104	XLOC_041816	ENSRNOT00000086374	0.993568904	8.57E-11
TUCP_001104	XLOC_041816	ENSRNOT00000015270	1	4.25E-78
TUCP_001104	XLOC_041816	ENSRNOT00000046192	1	0
TUCP_001104	XLOC_041816	ENSRNOT00000044226	1	1.33E-79
TUCP_001104	XLOC_041816	ENSRNOT00000081898	0.989837012	8.39E-10
TUCP_001104	XLOC_041816	ENSRNOT00000081954	0.964005219	4.48E-07
TUCP_001104	XLOC_041816	ENSRNOT00000080291	0.998427671	7.55E-14
TUCP_001104	XLOC_041816	ENSRNOT00000004293	0.984239049	7.46E-09
TUCP_001104	XLOC_041816	ENSRNOT00000078231	0.960455489	7.13E-07
TUCP_001104	XLOC_041816	ENSRNOT00000002834	1	0
TUCP_001104	XLOC_041816	ENSRNOT00000001464	1	0
TUCP_001104	XLOC_041816	ENSRNOT00000090716	0.994769629	3.06E-11
TUCP_001104	XLOC_041816	ENSRNOT00000083542	0.972568514	1.17E-07
TUCP_001104	XLOC_041816	ENSRNOT00000076044	1	0

TUCP_001104	XLOC_041816	ENSRNOT00000074353	1	0
TUCP_001104	XLOC_041816	ENSRNOT00000043510	0.958144129	9.43E-07
TUCP_001104	XLOC_041816	ENSRNOT00000087122	0.980089513	2.38E-08
TUCP_001104	XLOC_041816	ENSRNOT00000074295	0.987777006	2.11E-09
TUCP_001104	XLOC_041816	ENSRNOT00000057873	1	1.33E-79
TUCP_001104	XLOC_041816	ENSRNOT00000083002	0.988874897	1.32E-09
TUCP_001104	XLOC_041816	ENSRNOT00000088399	0.999999855	4.99E-34
TUCP_001104	XLOC_041816	ENSRNOT00000086248	1	0
TUCP_001104	XLOC_041816	ENSRNOT00000089750	0.999992758	1.57E-25
TUCP_001104	XLOC_041816	ENSRNOT00000030517	0.971225224	1.48E-07
TUCP_001104	XLOC_041816	ENSRNOT00000034772	0.994265187	4.84E-11
TUCP_001104	XLOC_041816	ENSRNOT00000089305	0.969641693	1.93E-07
TUCP_001104	XLOC_041816	ENSRNOT00000024580	0.954183021	1.47E-06
TUCP_001104	XLOC_041816	ENSRNOT00000064601	0.966578038	3.11E-07
TUCP_001104	XLOC_041816	ENSRNOT00000015870	0.965901306	3.43E-07
TUCP_001104	XLOC_041816	ENSRNOT00000080812	0.992998865	1.31E-10
TUCP_001104	XLOC_041816	ENSRNOT00000092755	1	4.25E-78
TUCP_001104	XLOC_041816	ENSRNOT00000031683	1	1.33E-79
TUCP_001104	XLOC_041816	ENSRNOT00000087764	0.988156196	1.80E-09
TUCP_001545	XLOC_060077	ENSRNOT00000010144	0.954235118	1.46E-06
TUCP_001102	XLOC_041816	ENSRNOT00000076720	0.954069508	1.49E-06
TUCP_001100	XLOC_041696	ENSRNOT00000001215	0.959683615	7.84E-07
TUCP_001100	XLOC_041696	ENSRNOT00000006188	-0.984121854	7.74E-09
TUCP_001100	XLOC_041696	ENSRNOT00000006186	0.968755553	2.23E-07
TUCP_001100	XLOC_041696	ENSRNOT00000026941	0.954226103	1.47E-06
TUCP_001100	XLOC_041696	ENSRNOT00000075397	0.976182606	5.80E-08
TUCP_001100	XLOC_041696	ENSRNOT00000032926	-0.955655633	1.25E-06
TUCP_001100	XLOC_041696	ENSRNOT00000006217	0.955317352	1.30E-06
TUCP_001100	XLOC_041696	ENSRNOT00000003891	0.954405459	1.44E-06
TUCP_001100	XLOC_041696	ENSRNOT00000001691	0.960513789	7.07E-07
TUCP_001100	XLOC_041696	ENSRNOT00000089319	0.955075002	1.34E-06
TUCP_001100	XLOC_041696	ENSRNOT00000079138	0.950165663	2.23E-06
TUCP_001100	XLOC_041696	ENSRNOT00000086706	0.965453873	3.66E-07
TUCP_001100	XLOC_041696	ENSRNOT00000004017	0.968944305	2.16E-07
TUCP_001100	XLOC_041696	ENSRNOT00000086310	-0.951085985	2.03E-06
TUCP_001100	XLOC_041696	ENSRNOT00000010030	-0.976158867	5.83E-08
TUCP_001100	XLOC_041696	ENSRNOT00000052063	0.963275581	4.95E-07
TUCP_001100	XLOC_041696	ENSRNOT00000042638	0.953514871	1.58E-06
TUCP_001100	XLOC_041696	ENSRNOT00000030850	0.968645521	2.26E-07
TUCP_001100	XLOC_041696	ENSRNOT00000033017	0.95334348	1.61E-06
TUCP_001100	XLOC_041696	ENSRNOT00000006752	0.968135851	2.45E-07
TUCP_001100	XLOC_041696	ENSRNOT00000031672	-0.954197967	1.47E-06
TUCP_001100	XLOC_041696	ENSRNOT00000085251	-0.950500311	2.15E-06
TUCP_001100	XLOC_041696	ENSRNOT00000083677	-0.96760398	2.66E-07
TUCP_001100	XLOC_041696	ENSRNOT00000073370	0.97169062	1.37E-07
TUCP_001100	XLOC_041696	ENSRNOT00000045699	0.952751268	1.71E-06
TUCP_001100	XLOC_041696	ENSRNOT00000029439	0.96477881	4.02E-07
TUCP_001100	XLOC_041696	ENSRNOT00000065871	0.955470273	1.28E-06
TUCP_001100	XLOC_041696	ENSRNOT00000019192	-0.950660879	2.12E-06
TUCP_001100	XLOC_041696	ENSRNOT00000077999	0.964527952	4.17E-07
TUCP_001100	XLOC_041696	ENSRNOT00000085228	0.964136303	4.40E-07

TUCP_001100	XLOC_041696	ENSRNOT00000015188	0.959540435	7.98E-07
TUCP_001100	XLOC_041696	ENSRNOT00000018488	-0.954173624	1.47E-06
TUCP_001100	XLOC_041696	ENSRNOT00000079866	0.952274312	1.80E-06
TUCP_001100	XLOC_041696	ENSRNOT00000075630	-0.952989918	1.67E-06
TUCP_001100	XLOC_041696	ENSRNOT00000005834	0.957282382	1.04E-06
TUCP_001100	XLOC_041696	ENSRNOT00000080410	-0.952554914	1.75E-06
TUCP_001100	XLOC_041696	ENSRNOT00000087371	0.952689731	1.72E-06
TUCP_001100	XLOC_041696	ENSRNOT00000030074	0.956225991	1.18E-06
TUCP_001100	XLOC_041696	ENSRNOT00000024841	-0.951831076	1.88E-06
TUCP_001100	XLOC_041696	ENSRNOT00000067391	-0.954325238	1.45E-06
TUCP_001100	XLOC_041696	ENSRNOT00000009150	0.955474686	1.28E-06
TUCP_001100	XLOC_041696	ENSRNOT00000084709	0.976377641	5.57E-08
TUCP_001100	XLOC_041696	ENSRNOT00000068033	0.954124213	1.48E-06
TUCP_001100	XLOC_041696	ENSRNOT00000009156	0.95794099	9.66E-07
TUCP_001100	XLOC_041696	ENSRNOT00000026481	0.968350043	2.37E-07
TUCP_001100	XLOC_041696	ENSRNOT00000012892	0.953955032	1.51E-06
TUCP_001100	XLOC_041696	ENSRNOT00000025856	-0.96657546	3.11E-07
TUCP_001100	XLOC_041696	ENSRNOT00000026643	0.958379687	9.17E-07
TUCP_001100	XLOC_041696	ENSRNOT00000001403	0.969840843	1.87E-07
TUCP_001100	XLOC_041696	ENSRNOT00000072357	-0.971008916	1.54E-07
TUCP_001100	XLOC_041696	ENSRNOT00000016132	-0.975032099	7.33E-08
TUCP_001100	XLOC_041696	ENSRNOT00000058495	-0.952856906	1.69E-06
TUCP_001100	XLOC_041696	ENSRNOT00000013067	-0.977960684	3.95E-08
TUCP_001100	XLOC_041696	ENSRNOT00000034692	-0.963622296	4.72E-07
TUCP_001100	XLOC_041696	ENSRNOT00000041815	-0.951315673	1.98E-06
TUCP_001100	XLOC_041696	ENSRNOT00000033975	0.960808793	6.82E-07
TUCP_001100	XLOC_041696	ENSRNOT00000079895	0.951303033	1.99E-06
TUCP_001100	XLOC_041696	ENSRNOT00000074586	-0.965287366	3.74E-07
TUCP_001100	XLOC_041696	ENSRNOT00000081047	0.956424933	1.15E-06
TUCP_001100	XLOC_041696	ENSRNOT00000011880	0.977033416	4.84E-08
TUCP_001100	XLOC_041696	ENSRNOT00000023989	0.967372295	2.76E-07
TUCP_001100	XLOC_041696	ENSRNOT00000021717	0.955177411	1.32E-06
TUCP_001100	XLOC_041696	ENSRNOT00000050515	0.975356436	6.87E-08
TUCP_001100	XLOC_041696	ENSRNOT00000089980	-0.957002085	1.08E-06
TUCP_001100	XLOC_041696	ENSRNOT00000055602	-0.971945977	1.31E-07
TUCP_001100	XLOC_041696	ENSRNOT00000046745	0.971935313	1.31E-07
TUCP_001100	XLOC_041696	ENSRNOT00000082146	-0.952238235	1.81E-06
TUCP_001100	XLOC_041696	ENSRNOT00000031555	-0.961182958	6.50E-07
TUCP_001100	XLOC_041696	ENSRNOT00000011563	-0.958735294	8.79E-07
TUCP_001100	XLOC_041696	ENSRNOT00000023876	0.970861755	1.58E-07
TUCP_001100	XLOC_041696	ENSRNOT00000033359	0.954435072	1.43E-06
TUCP_001100	XLOC_041696	ENSRNOT00000061201	-0.956345484	1.16E-06
TUCP_001100	XLOC_041696	ENSRNOT00000002187	-0.967587463	2.67E-07
TUCP_001100	XLOC_041696	ENSRNOT00000091488	0.953750527	1.54E-06
TUCP_001100	XLOC_041696	ENSRNOT00000049285	-0.972846869	1.11E-07
TUCP_001100	XLOC_041696	ENSRNOT00000015239	-0.958952715	8.56E-07
TUCP_001100	XLOC_041696	ENSRNOT00000003568	0.965929426	3.41E-07
TUCP_001100	XLOC_041696	ENSRNOT00000074649	0.972959369	1.09E-07
TUCP_001100	XLOC_041696	ENSRNOT00000035277	0.957400854	1.03E-06
TUCP_001100	XLOC_041696	ENSRNOT00000077146	-0.989824271	8.45E-10
TUCP_001100	XLOC_041696	ENSRNOT00000060002	-0.969005716	2.14E-07

TUCP_001100	XLOC_041696	ENSRNOT00000089673	-0.956725502	1.11E-06
TUCP_001100	XLOC_041696	ENSRNOT00000006395	-0.955525889	1.27E-06
TUCP_001100	XLOC_041696	ENSRNOT00000023977	-0.973215945	1.04E-07
TUCP_001100	XLOC_041696	ENSRNOT00000066133	-0.975864962	6.19E-08
TUCP_001100	XLOC_041696	ENSRNOT00000035385	0.952737916	1.72E-06
TUCP_001100	XLOC_041696	ENSRNOT00000009994	-0.952161655	1.82E-06
TUCP_001100	XLOC_041696	ENSRNOT00000057738	0.960920704	6.72E-07
TUCP_001100	XLOC_041696	ENSRNOT00000080443	0.961156979	6.52E-07
TUCP_001100	XLOC_041696	ENSRNOT00000064535	-0.965771076	3.49E-07
TUCP_001100	XLOC_041696	ENSRNOT00000010748	0.970345545	1.72E-07
TUCP_001100	XLOC_041696	ENSRNOT00000087033	-0.95087601	2.07E-06
TUCP_001100	XLOC_041696	ENSRNOT00000008222	-0.95305114	1.66E-06
TUCP_001100	XLOC_041696	ENSRNOT00000035474	0.955802791	1.23E-06
TUCP_001100	XLOC_041696	ENSRNOT00000077367	-0.959340437	8.17E-07
TUCP_001100	XLOC_041696	ENSRNOT00000039102	0.97378358	9.33E-08
TUCP_001100	XLOC_041696	ENSRNOT00000023807	-0.951478901	1.95E-06
TUCP_001100	XLOC_041696	ENSRNOT00000078856	-0.972781981	1.12E-07
TUCP_001100	XLOC_041696	ENSRNOT00000091205	0.959830163	7.70E-07
TUCP_001100	XLOC_041696	ENSRNOT00000009742	-0.957613054	1.00E-06
TUCP_001100	XLOC_041696	ENSRNOT00000010983	0.966378642	3.20E-07
TUCP_001100	XLOC_041696	ENSRNOT00000007369	0.953029286	1.66E-06
TUCP_001100	XLOC_041696	ENSRNOT00000060698	-0.982611894	1.22E-08
TUCP_001100	XLOC_041696	ENSRNOT00000058351	0.962504015	5.48E-07
TUCP_001100	XLOC_041696	ENSRNOT00000045946	0.960750622	6.87E-07
TUCP_001100	XLOC_041696	ENSRNOT00000015624	0.976449548	5.48E-08
TUCP_001100	XLOC_041696	ENSRNOT00000061100	0.957332879	1.04E-06
TUCP_001100	XLOC_041696	ENSRNOT00000065529	0.962772569	5.29E-07
TUCP_001100	XLOC_041696	ENSRNOT00000017776	0.971099909	1.51E-07
TUCP_001100	XLOC_041696	ENSRNOT00000005686	-0.966222901	3.27E-07
TUCP_001100	XLOC_041696	ENSRNOT00000021534	-0.970222113	1.75E-07
TUCP_001100	XLOC_041696	ENSRNOT00000004463	0.964939762	3.93E-07
TUCP_001100	XLOC_041696	ENSRNOT00000037809	-0.962142964	5.75E-07
TUCP_001100	XLOC_041696	ENSRNOT00000002028	0.955237033	1.31E-06
TUCP_001100	XLOC_041696	ENSRNOT00000008982	0.960049603	7.49E-07
TUCP_001100	XLOC_041696	ENSRNOT00000022097	0.980212239	2.31E-08
TUCP_001100	XLOC_041696	ENSRNOT00000008987	-0.979706599	2.62E-08
TUCP_001100	XLOC_041696	ENSRNOT00000090299	0.953758904	1.54E-06
TUCP_001100	XLOC_041696	ENSRNOT00000061580	-0.951878989	1.87E-06
TUCP_001100	XLOC_041696	ENSRNOT00000024253	0.956026332	1.20E-06
TUCP_001100	XLOC_041696	ENSRNOT00000088073	-0.950848786	2.08E-06
TUCP_001100	XLOC_041696	ENSRNOT00000048020	0.955595988	1.26E-06
TUCP_001100	XLOC_041696	ENSRNOT00000063930	-0.968806483	2.21E-07
TUCP_001100	XLOC_041696	ENSRNOT00000048613	0.954556771	1.41E-06
TUCP_001100	XLOC_041696	ENSRNOT00000009563	-0.969329627	2.03E-07
TUCP_001100	XLOC_041696	ENSRNOT00000088555	-0.975326982	6.91E-08
TUCP_001100	XLOC_041696	ENSRNOT00000089418	0.950546893	2.14E-06
TUCP_001100	XLOC_041696	ENSRNOT00000081981	0.957304469	1.04E-06
TUCP_001100	XLOC_041696	ENSRNOT00000026531	-0.976193835	5.79E-08
TUCP_001100	XLOC_041696	ENSRNOT00000019057	-0.963421113	4.85E-07
TUCP_001100	XLOC_041696	ENSRNOT00000040847	-0.951962341	1.86E-06
TUCP_001100	XLOC_041696	ENSRNOT00000073193	-0.957782319	9.84E-07

TUCP_001100	XLOC_041696	ENSRNOT00000047836	-0.958172371	9.40E-07
TUCP_001100	XLOC_041696	ENSRNOT00000065978	0.95953587	7.98E-07
TUCP_001100	XLOC_041696	ENSRNOT00000074683	0.95035631	2.18E-06
TUCP_001100	XLOC_041696	ENSRNOT00000025079	-0.976606727	5.31E-08
TUCP_001100	XLOC_041696	ENSRNOT00000048754	-0.977086992	4.79E-08
TUCP_001100	XLOC_041696	ENSRNOT00000007824	-0.961767461	6.03E-07
TUCP_001100	XLOC_041696	ENSRNOT00000020681	-0.956098842	1.19E-06
TUCP_001100	XLOC_041696	ENSRNOT00000057898	-0.974416701	8.27E-08
TUCP_001100	XLOC_041696	ENSRNOT00000024677	0.965320204	3.73E-07
TUCP_001100	XLOC_041696	ENSRNOT00000056100	-0.96405348	4.45E-07
TUCP_001100	XLOC_041696	ENSRNOT00000009811	0.977157776	4.71E-08
TUCP_001100	XLOC_041696	ENSRNOT00000055683	0.960474934	7.11E-07
TUCP_001100	XLOC_041696	ENSRNOT00000077231	0.961256218	6.44E-07
TUCP_001100	XLOC_041696	ENSRNOT00000018634	-0.951492771	1.95E-06
TUCP_001100	XLOC_041696	ENSRNOT00000031701	-0.955500042	1.28E-06
TUCP_001100	XLOC_041696	ENSRNOT00000073643	-0.963410394	4.86E-07
TUCP_001100	XLOC_041696	ENSRNOT00000052002	0.952270356	1.80E-06
TUCP_001100	XLOC_041696	ENSRNOT00000022502	-0.956208027	1.18E-06
TUCP_001100	XLOC_041696	ENSRNOT00000017556	0.969881022	1.86E-07
TUCP_001100	XLOC_041696	ENSRNOT00000021802	0.963291686	4.94E-07
TUCP_001100	XLOC_041696	ENSRNOT00000021803	-0.953799296	1.53E-06
TUCP_001100	XLOC_041696	ENSRNOT00000046973	0.95461812	1.40E-06
TUCP_001100	XLOC_041696	ENSRNOT00000024352	0.971725388	1.36E-07
TUCP_001100	XLOC_041696	ENSRNOT00000018122	0.971235785	1.48E-07
TUCP_001100	XLOC_041696	ENSRNOT00000072118	0.972496434	1.18E-07
TUCP_001100	XLOC_041696	ENSRNOT00000003780	0.972742526	1.13E-07
TUCP_001100	XLOC_041696	ENSRNOT00000003787	-0.956386348	1.15E-06
TUCP_001100	XLOC_041696	ENSRNOT00000014506	-0.955826407	1.23E-06
TUCP_001100	XLOC_041696	ENSRNOT00000023262	-0.951367618	1.97E-06
TUCP_001100	XLOC_041696	ENSRNOT00000026312	0.964622535	4.11E-07
TUCP_001100	XLOC_041696	ENSRNOT00000068381	0.965881532	3.44E-07
TUCP_001100	XLOC_041696	ENSRNOT00000068026	-0.958310069	9.25E-07
TUCP_001100	XLOC_041696	ENSRNOT00000085621	0.980836446	1.97E-08
TUCP_001100	XLOC_041696	ENSRNOT00000014821	-0.980696481	2.04E-08
TUCP_001100	XLOC_041696	ENSRNOT00000058382	0.972832144	1.11E-07
TUCP_001100	XLOC_041696	ENSRNOT00000088599	0.969002906	2.14E-07
TUCP_001100	XLOC_041696	ENSRNOT00000005587	-0.95983545	7.69E-07
TUCP_001100	XLOC_041696	ENSRNOT00000017746	-0.952418604	1.77E-06
TUCP_001100	XLOC_041696	ENSRNOT00000023537	-0.971779571	1.34E-07
TUCP_001100	XLOC_041696	ENSRNOT00000017995	0.9693231	2.03E-07
TUCP_001100	XLOC_041696	ENSRNOT00000008126	-0.968087292	2.47E-07
TUCP_001100	XLOC_041696	ENSRNOT00000081725	0.977343927	4.53E-08
TUCP_001100	XLOC_041696	ENSRNOT00000005459	0.981649375	1.59E-08
TUCP_001100	XLOC_041696	ENSRNOT00000027910	0.962998451	5.13E-07
TUCP_001100	XLOC_041696	ENSRNOT00000037015	0.950458876	2.16E-06
TUCP_001100	XLOC_041696	ENSRNOT00000054976	-0.96540615	3.68E-07
TUCP_001100	XLOC_041696	ENSRNOT00000032780	0.954275484	1.46E-06
TUCP_001100	XLOC_041696	ENSRNOT00000066032	0.953454513	1.59E-06
TUCP_001100	XLOC_041696	ENSRNOT00000001783	0.956268604	1.17E-06
TUCP_001100	XLOC_041696	ENSRNOT00000027232	0.964004908	4.48E-07
TUCP_001100	XLOC_041696	ENSRNOT00000082151	-0.9640836	4.43E-07

TUCP_001100	XLOC_041696	ENSRNOT00000034411	0.963904313	4.54E-07
TUCP_001100	XLOC_041696	ENSRNOT00000005839	0.955439106	1.28E-06
TUCP_001100	XLOC_041696	ENSRNOT00000038511	-0.964101263	4.42E-07
TUCP_001100	XLOC_041696	ENSRNOT00000085971	-0.955996832	1.21E-06
TUCP_001100	XLOC_041696	ENSRNOT00000022142	0.950436154	2.17E-06
TUCP_001100	XLOC_041696	ENSRNOT00000012334	0.958132565	9.44E-07
TUCP_001100	XLOC_041696	ENSRNOT00000015537	0.959929521	7.61E-07
TUCP_001100	XLOC_041696	ENSRNOT00000022461	-0.953071949	1.66E-06
TUCP_001100	XLOC_041696	ENSRNOT00000004172	0.957260309	1.05E-06
TUCP_001100	XLOC_041696	ENSRNOT00000023965	-0.95536801	1.29E-06
TUCP_001100	XLOC_041696	ENSRNOT00000092876	-0.976033094	5.98E-08
TUCP_001100	XLOC_041696	ENSRNOT00000029125	0.954996537	1.35E-06
TUCP_001100	XLOC_041696	ENSRNOT00000085279	0.958896092	8.62E-07
TUCP_001100	XLOC_041696	ENSRNOT00000012528	0.956345109	1.16E-06
TUCP_001100	XLOC_041696	ENSRNOT00000082904	0.954267948	1.46E-06
TUCP_001100	XLOC_041696	ENSRNOT00000015318	-0.953334538	1.61E-06
TUCP_001100	XLOC_041696	ENSRNOT00000015223	0.960836458	6.79E-07
TUCP_001100	XLOC_041696	ENSRNOT00000085669	0.968240047	2.41E-07
TUCP_001100	XLOC_041696	ENSRNOT00000028907	0.969064215	2.12E-07
TUCP_001100	XLOC_041696	ENSRNOT00000010224	0.953370437	1.61E-06
TUCP_001100	XLOC_041696	ENSRNOT00000087278	-0.96553632	3.61E-07
TUCP_001100	XLOC_041696	ENSRNOT00000005311	0.958609634	8.92E-07
TUCP_001100	XLOC_041696	ENSRNOT00000015799	0.966781312	3.01E-07
TUCP_001100	XLOC_041696	ENSRNOT00000082998	0.955649604	1.25E-06
TUCP_001100	XLOC_041696	ENSRNOT00000075656	-0.951377361	1.97E-06
TUCP_001100	XLOC_041696	ENSRNOT00000015006	0.96373314	4.65E-07
TUCP_001100	XLOC_041696	ENSRNOT00000007350	0.964855352	3.98E-07
TUCP_001100	XLOC_041696	ENSRNOT00000018936	-0.972172723	1.25E-07
TUCP_001100	XLOC_041696	ENSRNOT00000017665	0.951819192	1.89E-06
TUCP_001100	XLOC_041696	ENSRNOT00000083578	0.954448627	1.43E-06
TUCP_001100	XLOC_041696	ENSRNOT00000000181	0.967838775	2.57E-07
TUCP_001100	XLOC_041696	ENSRNOT00000020297	0.962522013	5.47E-07
TUCP_001100	XLOC_041696	ENSRNOT00000023638	0.96741527	2.74E-07
TUCP_001100	XLOC_041696	ENSRNOT00000028628	-0.950181311	2.22E-06
TUCP_001100	XLOC_041696	ENSRNOT00000017361	0.963994553	4.49E-07
TUCP_001100	XLOC_041696	ENSRNOT00000005220	0.972450617	1.19E-07
TUCP_001100	XLOC_041696	ENSRNOT00000045326	0.963794933	4.61E-07
TUCP_001100	XLOC_041696	ENSRNOT00000016649	0.964767636	4.03E-07
TUCP_001100	XLOC_041696	ENSRNOT00000074023	0.975696842	6.41E-08
TUCP_001100	XLOC_041696	ENSRNOT00000018341	-0.956850566	1.10E-06
TUCP_001100	XLOC_041696	ENSRNOT00000065076	0.961193767	6.49E-07
TUCP_001100	XLOC_041696	ENSRNOT00000013516	0.968436687	2.34E-07
TUCP_001100	XLOC_041696	ENSRNOT00000054685	0.957615052	1.00E-06
TUCP_001100	XLOC_041696	ENSRNOT00000061005	0.951908871	1.87E-06
TUCP_001100	XLOC_041696	ENSRNOT00000057953	0.950094426	2.24E-06
TUCP_001100	XLOC_041696	ENSRNOT00000090338	-0.952250835	1.80E-06
TUCP_001100	XLOC_041696	ENSRNOT00000027760	0.957652158	9.99E-07
TUCP_001100	XLOC_041696	ENSRNOT00000024402	0.957592485	1.01E-06
TUCP_001100	XLOC_041696	ENSRNOT00000089933	0.969778735	1.89E-07
TUCP_001100	XLOC_041696	ENSRNOT00000003641	0.954351499	1.45E-06
TUCP_001100	XLOC_041696	ENSRNOT00000090912	0.969360091	2.02E-07

TUCP_001100	XLOC_041696	ENSRNOT00000067554	0.956546852	1.13E-06
TUCP_001100	XLOC_041696	ENSRNOT00000010784	0.957761654	9.86E-07
TUCP_001100	XLOC_041696	ENSRNOT00000033677	-0.964601403	4.12E-07
TUCP_001100	XLOC_041696	ENSRNOT00000019820	0.968979524	2.15E-07
TUCP_001100	XLOC_041696	ENSRNOT00000080962	0.952498572	1.76E-06
TUCP_001100	XLOC_041696	ENSRNOT00000011832	-0.97043209	1.69E-07
TUCP_001100	XLOC_041696	ENSRNOT00000003940	-0.960507992	7.08E-07
TUCP_001100	XLOC_041696	ENSRNOT00000006835	0.953993782	1.50E-06
TUCP_001100	XLOC_041696	ENSRNOT00000077727	-0.961932041	5.91E-07
TUCP_001100	XLOC_041696	ENSRNOT00000044346	-0.961688231	6.10E-07
TUCP_001100	XLOC_041696	ENSRNOT00000010541	0.951991707	1.85E-06
TUCP_001100	XLOC_041696	ENSRNOT00000092068	0.953044107	1.66E-06
TUCP_001100	XLOC_041696	ENSRNOT00000091909	0.975840862	6.22E-08
TUCP_001100	XLOC_041696	ENSRNOT00000013025	-0.957094855	1.07E-06
TUCP_001100	XLOC_041696	ENSRNOT00000003544	-0.95243375	1.77E-06
TUCP_001100	XLOC_041696	ENSRNOT00000086119	-0.961576333	6.18E-07
TUCP_001100	XLOC_041696	ENSRNOT00000022190	-0.978413521	3.56E-08
TUCP_001100	XLOC_041696	ENSRNOT00000025913	-0.969512189	1.97E-07
TUCP_001100	XLOC_041696	ENSRNOT00000077220	-0.959768526	7.76E-07
TUCP_001100	XLOC_041696	ENSRNOT00000030782	-0.950318339	2.19E-06
TUCP_001100	XLOC_041696	ENSRNOT00000067780	-0.983997629	8.05E-09
TUCP_001100	XLOC_041696	ENSRNOT00000086577	0.975032187	7.33E-08
TUCP_001100	XLOC_041696	ENSRNOT00000073910	-0.962210607	5.70E-07
TUCP_001100	XLOC_041696	ENSRNOT00000007738	-0.961819601	5.99E-07
TUCP_001100	XLOC_041696	ENSRNOT00000086590	0.95505863	1.34E-06
TUCP_001100	XLOC_041696	ENSRNOT00000034883	-0.978270212	3.68E-08
TUCP_001100	XLOC_041696	ENSRNOT00000020155	0.956599721	1.13E-06
TUCP_001100	XLOC_041696	ENSRNOT00000025827	0.951405263	1.97E-06
TUCP_001100	XLOC_041696	ENSRNOT00000055321	0.959968034	7.57E-07
TUCP_001100	XLOC_041696	ENSRNOT00000005910	-0.957116296	1.06E-06
TUCP_001100	XLOC_041696	ENSRNOT00000076485	-0.955166576	1.32E-06
TUCP_001100	XLOC_041696	ENSRNOT00000003359	-0.951487608	1.95E-06
TUCP_001100	XLOC_041696	ENSRNOT00000078475	0.962885668	5.21E-07
TUCP_001100	XLOC_041696	ENSRNOT00000026249	-0.96900117	2.14E-07
TUCP_001100	XLOC_041696	ENSRNOT00000016722	0.950812499	2.09E-06
TUCP_001100	XLOC_041696	ENSRNOT00000073526	0.950617265	2.13E-06
TUCP_001100	XLOC_041696	ENSRNOT00000000013	0.968248587	2.41E-07
TUCP_001100	XLOC_041696	ENSRNOT00000018477	-0.95451212	1.42E-06
TUCP_001100	XLOC_041696	ENSRNOT00000005161	0.965145447	3.82E-07
TUCP_001100	XLOC_041696	ENSRNOT00000037955	-0.963296005	4.93E-07
TUCP_001100	XLOC_041696	ENSRNOT00000055582	0.952109219	1.83E-06
TUCP_001100	XLOC_041696	ENSRNOT00000022074	0.964164997	4.38E-07
TUCP_001100	XLOC_041696	ENSRNOT00000004999	0.967717625	2.62E-07
TUCP_001100	XLOC_041696	ENSRNOT00000013463	0.965834355	3.46E-07
TUCP_001100	XLOC_041696	ENSRNOT00000041365	0.952852498	1.69E-06
TUCP_001100	XLOC_041696	ENSRNOT00000088054	0.95532898	1.30E-06
TUCP_001100	XLOC_041696	ENSRNOT00000043872	0.951633844	1.92E-06
TUCP_001100	XLOC_041696	ENSRNOT00000047663	-0.966915437	2.95E-07
TUCP_001100	XLOC_041696	ENSRNOT00000029202	-0.959357394	8.16E-07
TUCP_001100	XLOC_041696	ENSRNOT00000005489	0.977551908	4.32E-08
TUCP_001100	XLOC_041696	ENSRNOT00000025794	-0.964921388	3.94E-07

TUCP_001100	XLOC_041696	ENSRNOT00000058111	-0.952641781	1.73E-06
TUCP_001100	XLOC_041696	ENSRNOT00000084422	0.966905217	2.96E-07
TUCP_001100	XLOC_041696	ENSRNOT00000088279	0.957257902	1.05E-06
TUCP_001100	XLOC_041696	ENSRNOT00000002443	0.962104284	5.78E-07
TUCP_001100	XLOC_041696	ENSRNOT00000012736	0.951611879	1.93E-06
TUCP_001100	XLOC_041696	ENSRNOT00000002114	-0.977606007	4.27E-08
TUCP_001100	XLOC_041696	ENSRNOT00000027970	0.980448477	2.18E-08
TUCP_001100	XLOC_041696	ENSRNOT00000009462	0.95443003	1.43E-06
TUCP_001100	XLOC_041696	ENSRNOT00000046215	-0.95679903	1.10E-06
TUCP_001100	XLOC_041696	ENSRNOT00000018074	-0.966233551	3.27E-07
TUCP_001100	XLOC_041696	ENSRNOT00000037679	0.951333367	1.98E-06
TUCP_001100	XLOC_041696	ENSRNOT00000014124	0.978950628	3.14E-08
TUCP_001100	XLOC_041696	ENSRNOT00000000120	0.977409126	4.46E-08
TUCP_001100	XLOC_041696	ENSRNOT00000013632	0.972302298	1.23E-07
TUCP_001100	XLOC_041696	ENSRNOT00000078906	-0.9579227	9.68E-07
TUCP_001100	XLOC_041696	ENSRNOT00000028545	-0.952136669	1.83E-06
TUCP_001100	XLOC_041696	ENSRNOT00000055442	0.964248589	4.33E-07
TUCP_001100	XLOC_041696	ENSRNOT00000028225	0.950374504	2.18E-06
TUCP_001100	XLOC_041696	ENSRNOT00000015495	-0.979594111	2.69E-08
TUCP_001100	XLOC_041696	ENSRNOT00000032248	0.956490001	1.14E-06
TUCP_001100	XLOC_041696	ENSRNOT00000021216	0.950895159	2.07E-06
TUCP_001100	XLOC_041696	ENSRNOT00000040393	0.968330507	2.38E-07
TUCP_001100	XLOC_041696	ENSRNOT00000011511	-0.953320544	1.61E-06
TUCP_001100	XLOC_041696	ENSRNOT00000064700	0.958724703	8.80E-07
TUCP_001100	XLOC_041696	ENSRNOT00000036613	-0.976137536	5.85E-08
TUCP_001100	XLOC_041696	ENSRNOT00000028876	-0.953488435	1.59E-06
TUCP_001100	XLOC_041696	ENSRNOT00000025705	0.984747889	6.34E-09
TUCP_001100	XLOC_041696	ENSRNOT00000019983	-0.969146255	2.09E-07
TUCP_001100	XLOC_041696	ENSRNOT00000004140	0.950697248	2.11E-06
TUCP_001100	XLOC_041696	ENSRNOT00000008113	-0.973394589	1.00E-07
TUCP_001100	XLOC_041696	ENSRNOT00000047105	0.957230507	1.05E-06
TUCP_001100	XLOC_041696	ENSRNOT00000007698	-0.979437394	2.80E-08
TUCP_001100	XLOC_041696	ENSRNOT00000048910	-0.95005695	2.25E-06
TUCP_001100	XLOC_041696	ENSRNOT00000019213	0.950919834	2.06E-06
TUCP_001100	XLOC_041696	ENSRNOT00000020051	0.960924506	6.72E-07
TUCP_001100	XLOC_041696	ENSRNOT00000039341	0.960642901	6.96E-07
TUCP_001100	XLOC_041696	ENSRNOT00000015678	-0.965319164	3.73E-07
TUCP_001100	XLOC_041696	ENSRNOT00000082914	0.976585789	5.33E-08
TUCP_001100	XLOC_041696	ENSRNOT00000082863	0.962810944	5.26E-07
TUCP_001100	XLOC_041696	ENSRNOT00000049459	-0.976853236	5.03E-08
TUCP_001100	XLOC_041696	ENSRNOT00000065947	0.954198828	1.47E-06
TUCP_001100	XLOC_041696	ENSRNOT00000071166	0.956111973	1.19E-06
TUCP_001100	XLOC_041696	ENSRNOT00000084567	0.950415369	2.17E-06
TUCP_001100	XLOC_041696	ENSRNOT00000013851	-0.974237558	8.56E-08
TUCP_001100	XLOC_041696	ENSRNOT00000055437	-0.950081819	2.24E-06
TUCP_001100	XLOC_041696	ENSRNOT00000038212	-0.971704838	1.36E-07
TUCP_001100	XLOC_041696	ENSRNOT00000007028	-0.958301741	9.26E-07
TUCP_001100	XLOC_041696	ENSRNOT00000001507	0.964712592	4.06E-07
TUCP_001100	XLOC_041696	ENSRNOT00000083568	0.960404582	7.17E-07
TUCP_001100	XLOC_041696	ENSRNOT00000018584	0.951063053	2.04E-06
TUCP_001100	XLOC_041696	ENSRNOT00000039177	-0.959389449	8.12E-07

TUCP_001100	XLOC_041696	ENSRNOT00000084031	0.968919352	2.17E-07
TUCP_001100	XLOC_041696	ENSRNOT00000030109	0.95009582	2.24E-06
TUCP_001100	XLOC_041696	ENSRNOT00000037456	0.958210959	9.36E-07
TUCP_001100	XLOC_041696	ENSRNOT00000030082	0.950710086	2.11E-06
TUCP_001100	XLOC_041696	ENSRNOT00000065168	0.954137435	1.48E-06
TUCP_001100	XLOC_041696	ENSRNOT00000000577	0.958418588	9.13E-07
TUCP_001100	XLOC_041696	ENSRNOT00000081226	0.950318943	2.19E-06
TUCP_001100	XLOC_041696	ENSRNOT00000065045	0.957268351	1.04E-06
TUCP_001100	XLOC_041696	ENSRNOT00000056405	0.969075996	2.11E-07
TUCP_001100	XLOC_041696	ENSRNOT00000088133	-0.9613775	6.34E-07
TUCP_001100	XLOC_041696	ENSRNOT00000080677	-0.957422822	1.03E-06
TUCP_001100	XLOC_041696	ENSRNOT00000016254	-0.954154961	1.48E-06
TUCP_001100	XLOC_041696	ENSRNOT00000005405	0.967545854	2.69E-07
TUCP_001100	XLOC_041696	ENSRNOT00000083968	-0.95652346	1.14E-06
TUCP_001100	XLOC_041696	ENSRNOT00000037133	0.972470437	1.19E-07
TUCP_001100	XLOC_041696	ENSRNOT00000083964	-0.957210442	1.05E-06
TUCP_001100	XLOC_041696	ENSRNOT00000001893	0.960130542	7.42E-07
TUCP_001100	XLOC_041696	ENSRNOT00000026831	0.963878578	4.56E-07
TUCP_001100	XLOC_041696	ENSRNOT00000068452	0.952909463	1.68E-06
TUCP_001100	XLOC_041696	ENSRNOT00000028266	0.958735957	8.79E-07
TUCP_001100	XLOC_041696	ENSRNOT00000032363	0.950974934	2.05E-06
TUCP_001100	XLOC_041696	ENSRNOT00000016445	0.960906214	6.73E-07
TUCP_001100	XLOC_041696	ENSRNOT00000019133	0.957923139	9.68E-07
TUCP_001100	XLOC_041696	ENSRNOT00000024119	0.9779574	3.95E-08
TUCP_001100	XLOC_041696	ENSRNOT00000006824	-0.956187901	1.18E-06
TUCP_001100	XLOC_041696	ENSRNOT00000032667	-0.956121476	1.19E-06
TUCP_001100	XLOC_041696	ENSRNOT00000000170	-0.967338717	2.77E-07
TUCP_001100	XLOC_041696	ENSRNOT00000086260	-0.966701812	3.05E-07
TUCP_001100	XLOC_041696	ENSRNOT00000078741	0.950547799	2.14E-06
TUCP_001100	XLOC_041696	ENSRNOT00000001099	-0.97423252	8.57E-08
TUCP_001100	XLOC_041696	ENSRNOT00000090123	0.956386824	1.15E-06
TUCP_001100	XLOC_041696	ENSRNOT00000001158	0.955519837	1.27E-06
TUCP_001100	XLOC_041696	ENSRNOT00000045488	0.950389515	2.18E-06
TUCP_001100	XLOC_041696	ENSRNOT00000084597	-0.959334139	8.18E-07
TUCP_001100	XLOC_041696	ENSRNOT00000089211	0.954616167	1.40E-06
TUCP_001100	XLOC_041696	ENSRNOT00000020383	0.968555769	2.30E-07
TUCP_001100	XLOC_041696	ENSRNOT00000000302	0.958388568	9.16E-07
TUCP_001100	XLOC_041696	ENSRNOT00000078070	0.952363787	1.78E-06
TUCP_001100	XLOC_041696	ENSRNOT00000041106	0.96035229	7.22E-07
TUCP_001100	XLOC_041696	ENSRNOT00000090129	-0.956917068	1.09E-06
TUCP_001100	XLOC_041696	ENSRNOT00000000658	0.952454002	1.77E-06
TUCP_001100	XLOC_041696	ENSRNOT00000000717	-0.958294201	9.26E-07
TUCP_001100	XLOC_041696	ENSRNOT00000005901	0.952682034	1.73E-06
TUCP_001100	XLOC_041696	ENSRNOT00000086122	0.976670528	5.23E-08
TUCP_001100	XLOC_041696	ENSRNOT00000026235	-0.955489117	1.28E-06
TUCP_001100	XLOC_041696	ENSRNOT00000023763	-0.958512058	9.03E-07
TUCP_001100	XLOC_041696	ENSRNOT00000010993	0.961839949	5.98E-07
TUCP_001100	XLOC_041696	ENSRNOT00000059181	0.966202943	3.28E-07
TUCP_001100	XLOC_041696	ENSRNOT00000017291	-0.959623281	7.90E-07
TUCP_001100	XLOC_041696	ENSRNOT00000003691	-0.962454302	5.52E-07
TUCP_001100	XLOC_041696	ENSRNOT00000017765	0.956405502	1.15E-06

TUCP_001100	XLOC_041696	ENSRNOT00000017761	0.978642612	3.38E-08
TUCP_001100	XLOC_041696	ENSRNOT00000023543	-0.9610625	6.60E-07
TUCP_001100	XLOC_041696	ENSRNOT00000080608	0.964639516	4.10E-07
TUCP_001100	XLOC_041696	ENSRNOT00000027117	-0.969977291	1.83E-07
TUCP_001100	XLOC_041696	ENSRNOT00000061392	0.963427317	4.85E-07
TUCP_001100	XLOC_041696	ENSRNOT00000014605	-0.961158909	6.52E-07
TUCP_001100	XLOC_041696	ENSRNOT00000042125	0.969196684	2.07E-07
TUCP_001100	XLOC_041696	ENSRNOT00000025460	0.966209993	3.28E-07
TUCP_001100	XLOC_041696	ENSRNOT00000026760	-0.978485168	3.50E-08
TUCP_001100	XLOC_041696	ENSRNOT00000001289	0.960796786	6.83E-07
TUCP_001100	XLOC_041696	ENSRNOT00000081077	-0.982151968	1.38E-08
TUCP_001100	XLOC_041696	ENSRNOT00000016568	0.968332837	2.38E-07
TUCP_001100	XLOC_041696	ENSRNOT00000023388	-0.962728494	5.32E-07
TUCP_001100	XLOC_041696	ENSRNOT00000024938	-0.951275971	1.99E-06
TUCP_001100	XLOC_041696	ENSRNOT00000024933	-0.950627772	2.13E-06
TUCP_001100	XLOC_041696	ENSRNOT00000047210	0.966370018	3.20E-07
TUCP_001100	XLOC_041696	ENSRNOT00000083957	0.960596766	7.00E-07
TUCP_001101	XLOC_041786	ENSRNOT00000015113	0.952940508	1.68E-06
TUCP_001101	XLOC_041786	ENSRNOT00000058563	-0.95547496	1.28E-06
TUCP_001101	XLOC_041786	ENSRNOT00000074993	0.953099267	1.65E-06
TUCP_004071	XLOC_178906	ENSRNOT00000028555	0.963439553	4.84E-07
TUCP_004071	XLOC_178906	ENSRNOT00000034951	0.952412054	1.77E-06
TUCP_004071	XLOC_178906	ENSRNOT00000065105	0.952906328	1.69E-06
TUCP_004071	XLOC_178906	ENSRNOT00000025225	0.959814157	7.71E-07
TUCP_004071	XLOC_178906	ENSRNOT00000001438	0.953656909	1.56E-06
TUCP_004071	XLOC_178906	ENSRNOT00000010759	0.965244284	3.77E-07
TUCP_004071	XLOC_178906	ENSRNOT00000022348	0.96002261	7.52E-07
TUCP_004071	XLOC_178906	ENSRNOT00000001518	0.955848699	1.23E-06
TUCP_004071	XLOC_178906	ENSRNOT00000084232	0.976091464	5.91E-08
TUCP_004071	XLOC_178906	ENSRNOT00000013461	0.953688096	1.55E-06
TUCP_004071	XLOC_178906	ENSRNOT00000023935	0.971147841	1.50E-07
TUCP_004071	XLOC_178906	ENSRNOT00000027305	0.971655113	1.37E-07
TUCP_004071	XLOC_178906	ENSRNOT00000025421	0.96319023	5.00E-07
TUCP_004071	XLOC_178906	ENSRNOT00000000603	0.966594105	3.10E-07
TUCP_004071	XLOC_178906	ENSRNOT00000020635	0.97536606	6.85E-08
TUCP_004070	XLOC_178777	ENSRNOT00000037559	0.963716344	4.66E-07
TUCP_000455	XLOC_015128	ENSRNOT00000040625	0.957557639	1.01E-06
TUCP_000455	XLOC_015128	ENSRNOT00000037336	0.960233466	7.33E-07
TUCP_001108	XLOC_042154	ENSRNOT00000080512	0.954025402	1.50E-06
TUCP_001109	XLOC_042156	ENSRNOT00000008764	0.951394375	1.97E-06
TUCP_001109	XLOC_042156	ENSRNOT00000012757	0.957233868	1.05E-06
TUCP_001109	XLOC_042156	ENSRNOT00000025355	0.953929008	1.51E-06
TUCP_001109	XLOC_042156	ENSRNOT00000012247	0.95786265	9.75E-07
TUCP_001109	XLOC_042156	ENSRNOT00000065897	0.956770774	1.11E-06
TUCP_001109	XLOC_042156	ENSRNOT00000082711	0.957771127	9.85E-07
TUCP_001109	XLOC_042156	ENSRNOT00000078572	0.958099599	9.48E-07
TUCP_001109	XLOC_042156	ENSRNOT00000006109	0.950846141	2.08E-06
TUCP_001109	XLOC_042156	ENSRNOT00000081226	0.950340887	2.19E-06
TUCP_001109	XLOC_042156	ENSRNOT00000016485	0.971433125	1.43E-07
TUCP_001109	XLOC_042156	ENSRNOT00000092066	-0.953250586	1.63E-06
TUCP_001109	XLOC_042156	ENSRNOT00000027544	0.952706481	1.72E-06

TUCP_001109	XLOC_042156	ENSRNOT00000085334	0.961965516	5.88E-07
TUCP_001109	XLOC_042156	ENSRNOT00000035309	-0.95013251	2.23E-06
TUCP_001109	XLOC_042156	ENSRNOT00000043827	0.959739609	7.79E-07
TUCP_001109	XLOC_042156	ENSRNOT00000048437	0.963066679	5.09E-07
TUCP_001109	XLOC_042156	ENSRNOT00000061054	0.952109415	1.83E-06
TUCP_001109	XLOC_042156	ENSRNOT00000057953	0.961666782	6.11E-07
TUCP_001109	XLOC_042156	ENSRNOT00000016088	0.95976602	7.76E-07
TUCP_001109	XLOC_042156	ENSRNOT00000042638	0.950487926	2.16E-06
TUCP_000350	XLOC_010989	ENSRNOT00000084069	-0.950392576	2.18E-06
TUCP_000350	XLOC_010989	ENSRNOT00000039437	0.950451974	2.16E-06
TUCP_000350	XLOC_010989	ENSRNOT00000044435	0.950418033	2.17E-06
TUCP_000350	XLOC_010989	ENSRNOT00000006266	0.953175009	1.64E-06
TUCP_000350	XLOC_010989	ENSRNOT00000078793	-0.958959093	8.56E-07
TUCP_000350	XLOC_010989	ENSRNOT00000003636	0.9511262	2.02E-06
TUCP_000351	XLOC_011328	ENSRNOT00000032973	0.963863058	4.57E-07
TUCP_000352	XLOC_011561	ENSRNOT00000020704	0.953159865	1.64E-06
TUCP_000352	XLOC_011561	ENSRNOT00000024583	-0.97062799	1.64E-07
TUCP_000352	XLOC_011561	ENSRNOT00000021590	0.963172758	5.01E-07
TUCP_000352	XLOC_011561	ENSRNOT00000022932	0.975642465	6.48E-08
TUCP_003771	XLOC_165752	ENSRNOT00000018179	0.982974919	1.09E-08
TUCP_003771	XLOC_165752	ENSRNOT00000086917	0.962407869	5.55E-07
TUCP_000355	XLOC_011771	ENSRNOT00000012413	0.952169678	1.82E-06
TUCP_000355	XLOC_011771	ENSRNOT00000079080	0.955848397	1.23E-06
TUCP_000355	XLOC_011771	ENSRNOT00000012837	-0.955749223	1.24E-06
TUCP_000355	XLOC_011771	ENSRNOT00000006148	0.95405871	1.49E-06
TUCP_000355	XLOC_011771	ENSRNOT00000023548	-0.95866103	8.87E-07
TUCP_000355	XLOC_011771	ENSRNOT00000007583	0.951112012	2.03E-06
TUCP_000355	XLOC_011771	ENSRNOT00000035770	0.961847031	5.97E-07
TUCP_000356	XLOC_011781	ENSRNOT00000068354	0.979824238	2.55E-08
TUCP_000356	XLOC_011781	ENSRNOT00000034421	0.961644252	6.13E-07
TUCP_000356	XLOC_011781	ENSRNOT00000011845	0.969531305	1.96E-07
TUCP_000356	XLOC_011781	ENSRNOT00000028877	0.950917403	2.07E-06
TUCP_000356	XLOC_011781	ENSRNOT00000078299	0.962904062	5.20E-07
TUCP_000356	XLOC_011781	ENSRNOT00000083268	0.954701481	1.39E-06
TUCP_000356	XLOC_011781	ENSRNOT00000073846	-0.952037755	1.84E-06
TUCP_000356	XLOC_011781	ENSRNOT00000085479	0.975267383	6.99E-08
TUCP_000356	XLOC_011781	ENSRNOT00000025731	0.968728812	2.23E-07
TUCP_000356	XLOC_011781	ENSRNOT00000000995	0.970493518	1.68E-07
TUCP_000356	XLOC_011781	ENSRNOT00000003458	0.957061303	1.07E-06
TUCP_000356	XLOC_011781	ENSRNOT00000084722	0.95495068	1.35E-06
TUCP_000356	XLOC_011781	ENSRNOT00000003778	0.954496561	1.42E-06
TUCP_000356	XLOC_011781	ENSRNOT00000051028	-0.964286924	4.31E-07
TUCP_000356	XLOC_011781	ENSRNOT00000051067	0.951464514	1.95E-06
TUCP_000356	XLOC_011781	ENSRNOT00000031693	0.974209091	8.61E-08
TUCP_000356	XLOC_011781	ENSRNOT00000082393	0.95810133	9.48E-07
TUCP_000356	XLOC_011781	ENSRNOT00000003082	0.950901054	2.07E-06
TUCP_000357	XLOC_011859	ENSRNOT00000081800	0.954131438	1.48E-06
TUCP_000357	XLOC_011859	ENSRNOT00000020147	0.955697166	1.25E-06
TUCP_000357	XLOC_011859	ENSRNOT00000057924	0.957027112	1.07E-06
TUCP_000357	XLOC_011859	ENSRNOT00000003102	0.954430569	1.43E-06
TUCP_000357	XLOC_011859	ENSRNOT00000005664	0.961067816	6.60E-07

TUCP_000357	XLOC_011859	ENSRNOT00000065529	0.953069838	1.66E-06
TUCP_000357	XLOC_011859	ENSRNOT00000042799	0.956253596	1.17E-06
TUCP_004673	XLOC_205765	ENSRNOT00000001921	0.953208343	1.63E-06
TUCP_000359	XLOC_011980	ENSRNOT00000084133	0.999963246	5.28E-22
TUCP_000359	XLOC_011980	ENSRNOT00000093745	0.981678123	1.58E-08
TUCP_000359	XLOC_011980	ENSRNOT00000065542	0.998137629	1.76E-13
TUCP_000359	XLOC_011980	ENSRNOT00000082464	0.999963246	5.28E-22
TUCP_000359	XLOC_011980	ENSRNOT00000087659	0.999963246	5.28E-22
TUCP_000359	XLOC_011980	ENSRNOT00000084582	0.977196127	4.67E-08
TUCP_000359	XLOC_011980	ENSRNOT00000085078	0.999963246	5.28E-22
TUCP_000359	XLOC_011980	ENSRNOT00000014028	0.999963246	5.28E-22
TUCP_000359	XLOC_011980	ENSRNOT00000049363	0.999963246	5.28E-22
TUCP_000359	XLOC_011980	ENSRNOT00000065473	0.999901309	7.37E-20
TUCP_000359	XLOC_011980	ENSRNOT00000000142	0.999179381	2.93E-15
TUCP_000359	XLOC_011980	ENSRNOT00000082288	0.999964045	4.73E-22
TUCP_000359	XLOC_011980	ENSRNOT00000039664	0.999963246	5.28E-22
TUCP_000359	XLOC_011980	ENSRNOT00000065402	0.999963246	5.28E-22
TUCP_000359	XLOC_011980	ENSRNOT00000064551	0.964758737	4.03E-07
TUCP_000359	XLOC_011980	ENSRNOT00000084141	0.999963246	5.28E-22
TUCP_000359	XLOC_011980	ENSRNOT00000082435	0.999963246	5.28E-22
TUCP_000359	XLOC_011980	ENSRNOT00000082928	0.996746855	2.85E-12
TUCP_000359	XLOC_011980	ENSRNOT00000066546	0.956277686	1.17E-06
TUCP_000359	XLOC_011980	ENSRNOT00000046013	0.988178249	1.78E-09
TUCP_000359	XLOC_011980	ENSRNOT00000067931	0.978688873	3.34E-08
TUCP_000359	XLOC_011980	ENSRNOT00000086425	0.991093089	4.35E-10
TUCP_000359	XLOC_011980	ENSRNOT00000093696	0.956245627	1.17E-06
TUCP_000359	XLOC_011980	ENSRNOT00000036228	0.983757819	8.66E-09
TUCP_000359	XLOC_011980	ENSRNOT00000014030	0.999963246	5.28E-22
TUCP_000359	XLOC_011980	ENSRNOT00000081966	0.999963246	5.28E-22
TUCP_000359	XLOC_011980	ENSRNOT00000075906	0.999963246	5.28E-22
TUCP_000359	XLOC_011980	ENSRNOT00000088396	0.981836644	1.51E-08
TUCP_000359	XLOC_011980	ENSRNOT00000049259	0.96589464	3.43E-07
TUCP_000359	XLOC_011980	ENSRNOT00000092838	0.982355707	1.31E-08
TUCP_000359	XLOC_011980	ENSRNOT00000093442	0.958993185	8.52E-07
TUCP_003803	XLOC_166074	ENSRNOT00000001507	0.953105065	1.65E-06
TUCP_003803	XLOC_166074	ENSRNOT00000026860	0.974225831	8.58E-08
TUCP_003803	XLOC_166074	ENSRNOT00000038647	0.954812036	1.38E-06
TUCP_003803	XLOC_166074	ENSRNOT00000026941	0.96214409	5.75E-07
TUCP_003803	XLOC_166074	ENSRNOT00000011984	0.959759239	7.77E-07
TUCP_003803	XLOC_166074	ENSRNOT00000038433	0.951763742	1.90E-06
TUCP_003803	XLOC_166074	ENSRNOT00000045488	0.966831776	2.99E-07
TUCP_003803	XLOC_166074	ENSRNOT00000039842	0.9722304	1.24E-07
TUCP_003803	XLOC_166074	ENSRNOT00000044635	0.954471184	1.43E-06
TUCP_003803	XLOC_166074	ENSRNOT00000026293	0.961210109	6.48E-07
TUCP_003803	XLOC_166074	ENSRNOT00000017629	0.968348486	2.37E-07
TUCP_003803	XLOC_166074	ENSRNOT00000013260	0.981692635	1.57E-08
TUCP_003803	XLOC_166074	ENSRNOT00000031230	0.963173319	5.01E-07
TUCP_003803	XLOC_166074	ENSRNOT00000082228	0.96352753	4.78E-07
TUCP_003803	XLOC_166074	ENSRNOT00000012736	0.962895751	5.20E-07
TUCP_003803	XLOC_166074	ENSRNOT00000067822	0.962246121	5.67E-07
TUCP_003803	XLOC_166074	ENSRNOT00000078475	0.952604675	1.74E-06

TUCP_003803	XLOC_166074	ENSRNOT00000024941	0.962864121	5.23E-07
TUCP_003803	XLOC_166074	ENSRNOT00000087371	0.953000042	1.67E-06
TUCP_003803	XLOC_166074	ENSRNOT00000002292	0.95915015	8.36E-07
TUCP_003803	XLOC_166074	ENSRNOT00000020383	0.955674697	1.25E-06
TUCP_003803	XLOC_166074	ENSRNOT00000065947	0.963410244	4.86E-07
TUCP_003803	XLOC_166074	ENSRNOT00000015076	0.962115985	5.77E-07
TUCP_003803	XLOC_166074	ENSRNOT00000082100	0.963379804	4.88E-07
TUCP_003803	XLOC_166074	ENSRNOT00000079442	0.954761431	1.38E-06
TUCP_003803	XLOC_166074	ENSRNOT00000037679	0.964733728	4.05E-07
TUCP_003803	XLOC_166074	ENSRNOT00000074653	0.9645843	4.13E-07
TUCP_003803	XLOC_166074	ENSRNOT00000079464	0.953465775	1.59E-06
TUCP_003803	XLOC_166074	ENSRNOT00000055683	0.957076235	1.07E-06
TUCP_003803	XLOC_166074	ENSRNOT00000085596	0.971670587	1.37E-07
TUCP_003803	XLOC_166074	ENSRNOT00000035491	0.956432065	1.15E-06
TUCP_003803	XLOC_166074	ENSRNOT00000000658	0.961568751	6.19E-07
TUCP_003803	XLOC_166074	ENSRNOT00000039631	0.951865537	1.88E-06
TUCP_003803	XLOC_166074	ENSRNOT00000065501	0.950380949	2.18E-06
TUCP_003803	XLOC_166074	ENSRNOT00000006554	0.988099935	1.84E-09
TUCP_003803	XLOC_166074	ENSRNOT00000029994	0.957858969	9.75E-07
TUCP_003803	XLOC_166074	ENSRNOT00000010418	0.972886443	1.10E-07
TUCP_003803	XLOC_166074	ENSRNOT00000025523	0.965358674	3.71E-07
TUCP_003803	XLOC_166074	ENSRNOT00000082174	0.952911966	1.68E-06
TUCP_003803	XLOC_166074	ENSRNOT00000035989	0.952973515	1.67E-06
TUCP_003803	XLOC_166074	ENSRNOT00000078675	0.966265872	3.25E-07
TUCP_003803	XLOC_166074	ENSRNOT00000070933	0.961203467	6.49E-07
TUCP_003803	XLOC_166074	ENSRNOT00000037446	0.975979019	6.05E-08
TUCP_003803	XLOC_166074	ENSRNOT00000004359	0.959734445	7.79E-07
TUCP_003803	XLOC_166074	ENSRNOT00000061100	0.966274403	3.25E-07
TUCP_003803	XLOC_166074	ENSRNOT00000005387	0.950832878	2.08E-06
TUCP_003803	XLOC_166074	ENSRNOT00000088125	0.98427346	7.38E-09
TUCP_003803	XLOC_166074	ENSRNOT00000013274	0.960660758	6.95E-07
TUCP_003803	XLOC_166074	ENSRNOT00000005144	0.973967894	9.01E-08
TUCP_003803	XLOC_166074	ENSRNOT00000018584	0.960828214	6.80E-07
TUCP_003803	XLOC_166074	ENSRNOT00000036750	0.958417229	9.13E-07
TUCP_003803	XLOC_166074	ENSRNOT00000043345	0.963681643	4.68E-07
TUCP_003803	XLOC_166074	ENSRNOT00000037790	0.955555963	1.27E-06
TUCP_003803	XLOC_166074	ENSRNOT00000010609	0.979833096	2.54E-08
TUCP_003803	XLOC_166074	ENSRNOT00000068633	0.956121432	1.19E-06
TUCP_003803	XLOC_166074	ENSRNOT00000049245	0.951486457	1.95E-06
TUCP_003803	XLOC_166074	ENSRNOT00000009683	0.954495899	1.42E-06
TUCP_003803	XLOC_166074	ENSRNOT00000056813	0.959361388	8.15E-07
TUCP_003803	XLOC_166074	ENSRNOT00000004834	0.977762089	4.13E-08
TUCP_003803	XLOC_166074	ENSRNOT00000079895	0.957746115	9.88E-07
TUCP_003803	XLOC_166074	ENSRNOT00000026089	0.963499193	4.80E-07
TUCP_003803	XLOC_166074	ENSRNOT00000019222	0.960103578	7.44E-07
TUCP_004779	XLOC_211073	ENSRNOT00000012895	0.969750125	1.90E-07
TUCP_004779	XLOC_211073	ENSRNOT00000019687	0.951818215	1.89E-06
TUCP_004779	XLOC_211073	ENSRNOT00000067904	0.950553903	2.14E-06
TUCP_004779	XLOC_211073	ENSRNOT00000060186	0.959820197	7.71E-07
TUCP_004779	XLOC_211073	ENSRNOT00000021769	0.954988847	1.35E-06
TUCP_004779	XLOC_211073	ENSRNOT00000036212	0.951310488	1.99E-06

TUCP_004779	XLOC_211073	ENSRNOT00000020322	0.953966145	1.51E-06
TUCP_004779	XLOC_211073	ENSRNOT00000092535	0.956283105	1.17E-06
TUCP_004779	XLOC_211073	ENSRNOT00000067528	0.950070086	2.25E-06
TUCP_004779	XLOC_211073	ENSRNOT00000039117	0.953865539	1.52E-06
TUCP_004779	XLOC_211073	ENSRNOT00000005942	0.960276623	7.29E-07
TUCP_004779	XLOC_211073	ENSRNOT00000016248	0.952931354	1.68E-06
TUCP_004779	XLOC_211073	ENSRNOT00000050179	0.955362387	1.29E-06
TUCP_004779	XLOC_211073	ENSRNOT00000004003	0.966195122	3.28E-07
TUCP_004779	XLOC_211073	ENSRNOT00000078503	0.955877433	1.22E-06
TUCP_004779	XLOC_211073	ENSRNOT00000086812	0.95583944	1.23E-06
TUCP_004779	XLOC_211073	ENSRNOT00000031462	0.960054215	7.49E-07
TUCP_004779	XLOC_211073	ENSRNOT00000071241	0.961381428	6.34E-07
TUCP_004779	XLOC_211073	ENSRNOT00000022839	0.950102581	2.24E-06
TUCP_003805	XLOC_166111	ENSRNOT00000047105	0.962166958	5.73E-07
TUCP_003805	XLOC_166111	ENSRNOT00000003568	0.96202523	5.83E-07
TUCP_003805	XLOC_166111	ENSRNOT00000036072	0.953624957	1.56E-06
TUCP_003805	XLOC_166111	ENSRNOT00000031904	0.970194439	1.76E-07
TUCP_003805	XLOC_166111	ENSRNOT00000026941	0.980170679	2.34E-08
TUCP_003805	XLOC_166111	ENSRNOT00000048910	-0.950908444	2.07E-06
TUCP_003805	XLOC_166111	ENSRNOT00000019213	0.950881695	2.07E-06
TUCP_003805	XLOC_166111	ENSRNOT00000021348	0.963771737	4.62E-07
TUCP_003805	XLOC_166111	ENSRNOT00000037597	0.964205177	4.36E-07
TUCP_003805	XLOC_166111	ENSRNOT00000068353	0.965845987	3.46E-07
TUCP_003805	XLOC_166111	ENSRNOT00000001855	0.954939289	1.36E-06
TUCP_003805	XLOC_166111	ENSRNOT00000023638	0.967418078	2.74E-07
TUCP_003805	XLOC_166111	ENSRNOT00000006175	0.950275003	2.20E-06
TUCP_003805	XLOC_166111	ENSRNOT00000005220	0.960752322	6.87E-07
TUCP_003805	XLOC_166111	ENSRNOT00000023144	0.985088994	5.66E-09
TUCP_003805	XLOC_166111	ENSRNOT00000083568	0.958946873	8.57E-07
TUCP_003805	XLOC_166111	ENSRNOT00000084069	0.953083003	1.65E-06
TUCP_003805	XLOC_166111	ENSRNOT00000033017	0.955982974	1.21E-06
TUCP_003805	XLOC_166111	ENSRNOT00000013758	0.960116032	7.43E-07
TUCP_003805	XLOC_166111	ENSRNOT00000026543	0.970696777	1.62E-07
TUCP_003805	XLOC_166111	ENSRNOT00000043345	0.954613863	1.41E-06
TUCP_003805	XLOC_166111	ENSRNOT00000065076	0.953457156	1.59E-06
TUCP_003805	XLOC_166111	ENSRNOT00000024969	0.956645283	1.12E-06
TUCP_003805	XLOC_166111	ENSRNOT00000067400	0.95735429	1.03E-06
TUCP_003805	XLOC_166111	ENSRNOT00000001507	0.955378898	1.29E-06
TUCP_003805	XLOC_166111	ENSRNOT00000088125	0.950117111	2.24E-06
TUCP_003805	XLOC_166111	ENSRNOT00000065300	0.959528029	7.99E-07
TUCP_003805	XLOC_166111	ENSRNOT00000025079	-0.95837381	9.18E-07
TUCP_003805	XLOC_166111	ENSRNOT00000048754	-0.954822973	1.37E-06
TUCP_003805	XLOC_166111	ENSRNOT00000025075	0.951483413	1.95E-06
TUCP_003805	XLOC_166111	ENSRNOT00000035989	0.967490808	2.71E-07
TUCP_003805	XLOC_166111	ENSRNOT00000057160	0.981826748	1.51E-08
TUCP_003805	XLOC_166111	ENSRNOT00000024402	0.983746165	8.69E-09
TUCP_003805	XLOC_166111	ENSRNOT00000089933	0.973016647	1.08E-07
TUCP_003805	XLOC_166111	ENSRNOT00000012671	0.958360129	9.19E-07
TUCP_003805	XLOC_166111	ENSRNOT00000025517	0.978843971	3.22E-08
TUCP_003805	XLOC_166111	ENSRNOT00000084012	0.955716937	1.24E-06
TUCP_003805	XLOC_166111	ENSRNOT00000060696	0.959998918	7.54E-07

TUCP_003805	XLOC_166111	ENSRNOT00000038807	0.953960803	1.51E-06
TUCP_003805	XLOC_166111	ENSRNOT00000011429	0.950494725	2.15E-06
TUCP_003805	XLOC_166111	ENSRNOT00000077727	-0.950191179	2.22E-06
TUCP_003805	XLOC_166111	ENSRNOT00000041044	0.963499736	4.80E-07
TUCP_003805	XLOC_166111	ENSRNOT00000037133	0.952908854	1.68E-06
TUCP_003805	XLOC_166111	ENSRNOT00000010541	0.957456654	1.02E-06
TUCP_003805	XLOC_166111	ENSRNOT00000034298	0.957604438	1.00E-06
TUCP_003805	XLOC_166111	ENSRNOT00000091909	0.970787519	1.60E-07
TUCP_003805	XLOC_166111	ENSRNOT00000024352	0.966991415	2.92E-07
TUCP_003805	XLOC_166111	ENSRNOT00000083220	0.965420057	3.67E-07
TUCP_003805	XLOC_166111	ENSRNOT00000003780	0.97122373	1.48E-07
TUCP_003805	XLOC_166111	ENSRNOT00000026643	0.960398521	7.18E-07
TUCP_003805	XLOC_166111	ENSRNOT00000016445	0.955902398	1.22E-06
TUCP_003805	XLOC_166111	ENSRNOT00000060362	0.952072068	1.84E-06
TUCP_003805	XLOC_166111	ENSRNOT00000013274	0.956718553	1.11E-06
TUCP_003805	XLOC_166111	ENSRNOT00000027337	0.951253729	2.00E-06
TUCP_003805	XLOC_166111	ENSRNOT00000047093	0.954926778	1.36E-06
TUCP_003805	XLOC_166111	ENSRNOT00000025966	0.954145322	1.48E-06
TUCP_003805	XLOC_166111	ENSRNOT00000016739	0.961261851	6.44E-07
TUCP_003805	XLOC_166111	ENSRNOT00000078675	0.95117524	2.01E-06
TUCP_003805	XLOC_166111	ENSRNOT00000033975	0.952354603	1.78E-06
TUCP_003805	XLOC_166111	ENSRNOT00000079895	0.96338762	4.87E-07
TUCP_003805	XLOC_166111	ENSRNOT00000009811	0.962368122	5.58E-07
TUCP_003805	XLOC_166111	ENSRNOT00000047387	0.953177843	1.64E-06
TUCP_003805	XLOC_166111	ENSRNOT00000045488	0.967384842	2.75E-07
TUCP_003805	XLOC_166111	ENSRNOT00000039842	0.95047369	2.16E-06
TUCP_003805	XLOC_166111	ENSRNOT00000081047	0.958961916	8.56E-07
TUCP_003805	XLOC_166111	ENSRNOT00000081512	0.970976203	1.54E-07
TUCP_003805	XLOC_166111	ENSRNOT00000017629	0.958505652	9.03E-07
TUCP_003805	XLOC_166111	ENSRNOT00000008803	0.95383092	1.53E-06
TUCP_003805	XLOC_166111	ENSRNOT00000005387	0.957834709	9.78E-07
TUCP_003805	XLOC_166111	ENSRNOT00000005664	0.968075208	2.48E-07
TUCP_003805	XLOC_166111	ENSRNOT00000027910	0.971646966	1.38E-07
TUCP_003805	XLOC_166111	ENSRNOT00000078475	0.971427734	1.43E-07
TUCP_003805	XLOC_166111	ENSRNOT00000087429	0.95417749	1.47E-06
TUCP_003805	XLOC_166111	ENSRNOT00000040513	0.975932796	6.11E-08
TUCP_003805	XLOC_166111	ENSRNOT00000020383	0.958265025	9.30E-07
TUCP_003805	XLOC_166111	ENSRNOT00000084088	0.955941588	1.21E-06
TUCP_003805	XLOC_166111	ENSRNOT00000000302	0.978899177	3.18E-08
TUCP_003805	XLOC_166111	ENSRNOT00000090262	0.951911827	1.87E-06
TUCP_003805	XLOC_166111	ENSRNOT00000078070	0.975000952	7.37E-08
TUCP_003805	XLOC_166111	ENSRNOT00000037015	0.951586672	1.93E-06
TUCP_003805	XLOC_166111	ENSRNOT00000076729	0.955105001	1.33E-06
TUCP_003805	XLOC_166111	ENSRNOT00000090665	0.967797741	2.58E-07
TUCP_003805	XLOC_166111	ENSRNOT00000089293	0.957493775	1.02E-06
TUCP_003805	XLOC_166111	ENSRNOT00000088628	0.964410794	4.24E-07
TUCP_003805	XLOC_166111	ENSRNOT00000016191	0.951600266	1.93E-06
TUCP_003805	XLOC_166111	ENSRNOT00000065501	0.963638778	4.71E-07
TUCP_003805	XLOC_166111	ENSRNOT00000077999	0.954405745	1.44E-06
TUCP_003805	XLOC_166111	ENSRNOT00000025832	0.950764751	2.10E-06
TUCP_003805	XLOC_166111	ENSRNOT00000065229	0.957838773	9.77E-07

TUCP_003805	XLOC_166111	ENSRNOT00000020673	0.957761388	9.86E-07
TUCP_003805	XLOC_166111	ENSRNOT00000014235	0.953315736	1.61E-06
TUCP_003805	XLOC_166111	ENSRNOT00000052063	0.959415032	8.10E-07
TUCP_003805	XLOC_166111	ENSRNOT00000001783	0.953942836	1.51E-06
TUCP_003805	XLOC_166111	ENSRNOT00000037790	0.96181639	6.00E-07
TUCP_003805	XLOC_166111	ENSRNOT00000009122	0.952051127	1.84E-06
TUCP_003805	XLOC_166111	ENSRNOT00000004046	-0.956026768	1.20E-06
TUCP_003805	XLOC_166111	ENSRNOT00000086577	0.954535967	1.42E-06
TUCP_003805	XLOC_166111	ENSRNOT00000088054	0.983936254	8.20E-09
TUCP_003805	XLOC_166111	ENSRNOT00000057313	0.953983988	1.50E-06
TUCP_003805	XLOC_166111	ENSRNOT00000044395	0.951363525	1.97E-06
TUCP_003805	XLOC_166111	ENSRNOT00000087534	-0.951679043	1.91E-06
TUCP_003805	XLOC_166111	ENSRNOT00000042799	0.964828041	4.00E-07
TUCP_003805	XLOC_166111	ENSRNOT00000010992	0.961233422	6.46E-07
TUCP_003805	XLOC_166111	ENSRNOT00000038647	0.953057787	1.66E-06
TUCP_003805	XLOC_166111	ENSRNOT00000076669	0.957559531	1.01E-06
TUCP_003805	XLOC_166111	ENSRNOT00000011984	0.953924216	1.51E-06
TUCP_003805	XLOC_166111	ENSRNOT00000088279	0.969362563	2.02E-07
TUCP_003805	XLOC_166111	ENSRNOT00000081981	0.955355056	1.30E-06
TUCP_003805	XLOC_166111	ENSRNOT00000026293	0.963550311	4.77E-07
TUCP_003805	XLOC_166111	ENSRNOT00000014888	0.955427498	1.29E-06
TUCP_003805	XLOC_166111	ENSRNOT00000017761	0.951124854	2.02E-06
TUCP_003805	XLOC_166111	ENSRNOT00000066024	0.960633656	6.97E-07
TUCP_003805	XLOC_166111	ENSRNOT00000001222	0.959730822	7.79E-07
TUCP_003805	XLOC_166111	ENSRNOT00000012736	0.967804288	2.58E-07
TUCP_003805	XLOC_166111	ENSRNOT00000055992	-0.954780425	1.38E-06
TUCP_003805	XLOC_166111	ENSRNOT00000040069	0.955534706	1.27E-06
TUCP_003805	XLOC_166111	ENSRNOT00000010748	0.950025121	2.26E-06
TUCP_003805	XLOC_166111	ENSRNOT00000083127	0.962464914	5.51E-07
TUCP_003805	XLOC_166111	ENSRNOT00000078306	0.953814028	1.53E-06
TUCP_003805	XLOC_166111	ENSRNOT00000080695	0.985800264	4.44E-09
TUCP_003805	XLOC_166111	ENSRNOT00000018074	-0.956105999	1.19E-06
TUCP_003805	XLOC_166111	ENSRNOT00000091205	0.957762408	9.86E-07
TUCP_003805	XLOC_166111	ENSRNOT00000037679	0.953889521	1.52E-06
TUCP_003805	XLOC_166111	ENSRNOT00000002866	-0.963154139	5.03E-07
TUCP_003805	XLOC_166111	ENSRNOT00000085596	0.954851579	1.37E-06
TUCP_003805	XLOC_166111	ENSRNOT00000029125	0.96230734	5.62E-07
TUCP_003805	XLOC_166111	ENSRNOT00000007369	0.958069562	9.51E-07
TUCP_003805	XLOC_166111	ENSRNOT00000042125	0.962062952	5.81E-07
TUCP_003805	XLOC_166111	ENSRNOT00000014814	0.952305073	1.79E-06
TUCP_003805	XLOC_166111	ENSRNOT00000080207	0.972479202	1.19E-07
TUCP_003805	XLOC_166111	ENSRNOT00000065529	0.961791089	6.01E-07
TUCP_003805	XLOC_166111	ENSRNOT00000040393	0.954678926	1.40E-06
TUCP_003805	XLOC_166111	ENSRNOT00000036750	0.950162713	2.23E-06
TUCP_003805	XLOC_166111	ENSRNOT00000025092	0.966975671	2.93E-07
TUCP_003805	XLOC_166111	ENSRNOT00000027967	0.958201478	9.37E-07
TUCP_003805	XLOC_166111	ENSRNOT00000008982	0.956819531	1.10E-06
TUCP_003805	XLOC_166111	ENSRNOT00000021674	0.978278603	3.67E-08
TUCP_003805	XLOC_166111	ENSRNOT00000047192	-0.95217373	1.82E-06
TUCP_003805	XLOC_166111	ENSRNOT00000025705	0.950176249	2.22E-06
TUCP_003805	XLOC_166111	ENSRNOT00000005311	0.951686494	1.91E-06

TUCP_003805	XLOC_166111	ENSRNOT00000046639	0.969688646	1.92E-07
TUCP_003805	XLOC_166111	ENSRNOT00000015799	0.972617443	1.16E-07
TUCP_000294	XLOC_009318	ENSRNOT00000074753	0.963871744	4.56E-07
TUCP_000294	XLOC_009318	ENSRNOT00000041904	0.964331765	4.28E-07
TUCP_000294	XLOC_009318	ENSRNOT00000077990	0.964331765	4.28E-07
TUCP_000294	XLOC_009318	ENSRNOT00000038826	0.961891138	5.94E-07
TUCP_000294	XLOC_009318	ENSRNOT00000046842	0.950514546	2.15E-06
TUCP_000294	XLOC_009318	ENSRNOT00000021243	0.955610791	1.26E-06
TUCP_000294	XLOC_009318	ENSRNOT00000060250	0.964181699	4.37E-07
TUCP_000294	XLOC_009318	ENSRNOT00000091934	0.964331765	4.28E-07
TUCP_000294	XLOC_009318	ENSRNOT00000087656	0.953448394	1.59E-06
TUCP_000294	XLOC_009318	ENSRNOT00000012170	0.970522387	1.67E-07
TUCP_000294	XLOC_009318	ENSRNOT00000046585	0.970643384	1.63E-07
TUCP_000294	XLOC_009318	ENSRNOT00000034907	0.956548326	1.13E-06
TUCP_000294	XLOC_009318	ENSRNOT00000031205	0.972774001	1.13E-07
TUCP_000294	XLOC_009318	ENSRNOT00000044145	0.964331765	4.28E-07
TUCP_000294	XLOC_009318	ENSRNOT00000088381	0.971181299	1.49E-07
TUCP_000294	XLOC_009318	ENSRNOT00000011743	0.959827805	7.70E-07
TUCP_000294	XLOC_009318	ENSRNOT00000089929	0.964331765	4.28E-07
TUCP_000294	XLOC_009318	ENSRNOT00000088624	0.964331765	4.28E-07
TUCP_000294	XLOC_009318	ENSRNOT00000085659	0.971672659	1.37E-07
TUCP_000294	XLOC_009318	ENSRNOT00000015866	0.967940543	2.53E-07
TUCP_000294	XLOC_009318	ENSRNOT00000078633	0.964331765	4.28E-07
TUCP_000294	XLOC_009318	ENSRNOT00000051835	0.964331765	4.28E-07
TUCP_000294	XLOC_009318	ENSRNOT00000009825	0.965752615	3.50E-07
TUCP_000294	XLOC_009318	ENSRNOT00000079087	0.964331765	4.28E-07
TUCP_000294	XLOC_009318	ENSRNOT00000037206	0.964331765	4.28E-07
TUCP_000294	XLOC_009318	ENSRNOT00000080590	0.964806542	4.01E-07
TUCP_000294	XLOC_009318	ENSRNOT00000027552	0.96397123	4.50E-07
TUCP_000294	XLOC_009318	ENSRNOT00000077014	0.965565213	3.60E-07
TUCP_000294	XLOC_009318	ENSRNOT00000080453	0.964792266	4.02E-07
TUCP_000294	XLOC_009318	ENSRNOT00000057729	0.965490645	3.64E-07
TUCP_000294	XLOC_009318	ENSRNOT00000061284	0.951661928	1.92E-06
TUCP_000294	XLOC_009318	ENSRNOT00000071884	0.968200663	2.43E-07
TUCP_000294	XLOC_009318	ENSRNOT00000066839	0.95371325	1.55E-06
TUCP_000294	XLOC_009318	ENSRNOT00000078663	0.963665477	4.69E-07
TUCP_000294	XLOC_009318	ENSRNOT00000040509	0.964880247	3.97E-07
TUCP_000294	XLOC_009318	ENSRNOT00000037169	0.952012367	1.85E-06
TUCP_000294	XLOC_009318	ENSRNOT00000029634	0.950063426	2.25E-06
TUCP_000294	XLOC_009318	ENSRNOT00000093579	0.991995099	2.55E-10
TUCP_000294	XLOC_009318	ENSRNOT00000065460	0.964331765	4.28E-07
TUCP_000294	XLOC_009318	ENSRNOT00000003817	0.952462677	1.76E-06
TUCP_000294	XLOC_009318	ENSRNOT00000016713	0.964331765	4.28E-07
TUCP_000294	XLOC_009318	ENSRNOT00000079260	0.967874369	2.55E-07
TUCP_000294	XLOC_009318	ENSRNOT00000039294	0.964331765	4.28E-07
TUCP_000294	XLOC_009318	ENSRNOT00000024337	0.983061387	1.07E-08
TUCP_003807	XLOC_166167	ENSRNOT00000079130	-0.964555649	4.15E-07
TUCP_003807	XLOC_166167	ENSRNOT00000027647	0.969411447	2.00E-07
TUCP_003807	XLOC_166167	ENSRNOT00000091139	0.95221842	1.81E-06
TUCP_003807	XLOC_166167	ENSRNOT00000022341	0.965793156	3.48E-07
TUCP_003807	XLOC_166167	ENSRNOT00000021842	0.95676263	1.11E-06

TUCP_003807	XLOC_166167	ENSRNOT00000075992	0.95888589	8.63E-07
TUCP_003807	XLOC_166167	ENSRNOT00000014559	0.960993884	6.66E-07
TUCP_003807	XLOC_166167	ENSRNOT00000036718	0.973658326	9.56E-08
TUCP_003807	XLOC_166167	ENSRNOT00000019130	0.950233743	2.21E-06
TUCP_003807	XLOC_166167	ENSRNOT00000056877	0.954167193	1.47E-06
TUCP_003807	XLOC_166167	ENSRNOT00000068183	0.953051666	1.66E-06
TUCP_003806	XLOC_166117	ENSRNOT00000016083	-0.950650783	2.12E-06
TUCP_003806	XLOC_166117	ENSRNOT00000001215	0.951648072	1.92E-06
TUCP_003806	XLOC_166117	ENSRNOT00000026860	0.958188446	9.38E-07
TUCP_003806	XLOC_166117	ENSRNOT00000006188	-0.974565128	8.03E-08
TUCP_003806	XLOC_166117	ENSRNOT00000006186	0.982697736	1.19E-08
TUCP_003806	XLOC_166117	ENSRNOT00000090621	0.962720936	5.33E-07
TUCP_003806	XLOC_166117	ENSRNOT00000013743	0.950715907	2.11E-06
TUCP_003806	XLOC_166117	ENSRNOT00000075397	0.961843524	5.97E-07
TUCP_003806	XLOC_166117	ENSRNOT00000032926	-0.969071062	2.12E-07
TUCP_003806	XLOC_166117	ENSRNOT00000019104	-0.962629447	5.39E-07
TUCP_003806	XLOC_166117	ENSRNOT00000021348	0.962405018	5.55E-07
TUCP_003806	XLOC_166117	ENSRNOT00000006217	0.967091259	2.88E-07
TUCP_003806	XLOC_166117	ENSRNOT00000068353	0.979191101	2.97E-08
TUCP_003806	XLOC_166117	ENSRNOT00000079138	0.967138737	2.86E-07
TUCP_003806	XLOC_166117	ENSRNOT00000044635	0.965726784	3.52E-07
TUCP_003806	XLOC_166117	ENSRNOT00000025067	-0.952401412	1.78E-06
TUCP_003806	XLOC_166117	ENSRNOT00000086706	0.955077261	1.34E-06
TUCP_003806	XLOC_166117	ENSRNOT00000035277	0.959273921	8.24E-07
TUCP_003806	XLOC_166117	ENSRNOT00000082100	0.957061929	1.07E-06
TUCP_003806	XLOC_166117	ENSRNOT00000086310	-0.957388513	1.03E-06
TUCP_003806	XLOC_166117	ENSRNOT00000010030	-0.975867731	6.19E-08
TUCP_003806	XLOC_166117	ENSRNOT00000026941	0.979996506	2.44E-08
TUCP_003806	XLOC_166117	ENSRNOT00000031692	-0.956593342	1.13E-06
TUCP_003806	XLOC_166117	ENSRNOT00000052063	0.981396259	1.70E-08
TUCP_003806	XLOC_166117	ENSRNOT00000030850	0.950361316	2.18E-06
TUCP_003806	XLOC_166117	ENSRNOT00000055540	0.962201818	5.70E-07
TUCP_003806	XLOC_166117	ENSRNOT00000006752	0.980835464	1.97E-08
TUCP_003806	XLOC_166117	ENSRNOT00000000618	-0.951260995	2.00E-06
TUCP_003806	XLOC_166117	ENSRNOT00000085251	-0.950549202	2.14E-06
TUCP_003806	XLOC_166117	ENSRNOT00000072771	0.966540959	3.12E-07
TUCP_003806	XLOC_166117	ENSRNOT00000073370	0.967471855	2.72E-07
TUCP_003806	XLOC_166117	ENSRNOT00000065300	0.971090241	1.52E-07
TUCP_003806	XLOC_166117	ENSRNOT00000019923	0.960794504	6.83E-07
TUCP_003806	XLOC_166117	ENSRNOT00000029439	0.9587843	8.74E-07
TUCP_003806	XLOC_166117	ENSRNOT00000004400	0.965262516	3.76E-07
TUCP_003806	XLOC_166117	ENSRNOT00000035320	-0.958545666	8.99E-07
TUCP_003806	XLOC_166117	ENSRNOT00000076820	0.963639438	4.71E-07
TUCP_003806	XLOC_166117	ENSRNOT00000035989	0.951952855	1.86E-06
TUCP_003806	XLOC_166117	ENSRNOT00000057160	0.960029105	7.51E-07
TUCP_003806	XLOC_166117	ENSRNOT00000008274	-0.965828496	3.46E-07
TUCP_003806	XLOC_166117	ENSRNOT00000065871	0.953165137	1.64E-06
TUCP_003806	XLOC_166117	ENSRNOT00000067489	0.95361358	1.56E-06
TUCP_003806	XLOC_166117	ENSRNOT00000091448	-0.952404927	1.78E-06
TUCP_003806	XLOC_166117	ENSRNOT00000077999	0.980576178	2.11E-08
TUCP_003806	XLOC_166117	ENSRNOT00000068134	0.960775136	6.85E-07

TUCP_003806	XLOC_166117	ENSRNOT00000067626	-0.954776952	1.38E-06
TUCP_003806	XLOC_166117	ENSRNOT00000003146	-0.95913228	8.38E-07
TUCP_003806	XLOC_166117	ENSRNOT00000085228	0.96833752	2.38E-07
TUCP_003806	XLOC_166117	ENSRNOT00000026391	0.953308013	1.62E-06
TUCP_003806	XLOC_166117	ENSRNOT00000060002	-0.981270442	1.76E-08
TUCP_003806	XLOC_166117	ENSRNOT00000055187	0.958432844	9.11E-07
TUCP_003806	XLOC_166117	ENSRNOT00000015188	0.971939243	1.31E-07
TUCP_003806	XLOC_166117	ENSRNOT00000079866	0.96241552	5.54E-07
TUCP_003806	XLOC_166117	ENSRNOT00000005686	-0.97829342	3.66E-08
TUCP_003806	XLOC_166117	ENSRNOT00000015537	0.962577782	5.43E-07
TUCP_003806	XLOC_166117	ENSRNOT00000010927	0.962903394	5.20E-07
TUCP_003806	XLOC_166117	ENSRNOT00000087371	0.960817366	6.81E-07
TUCP_003806	XLOC_166117	ENSRNOT00000030074	0.959981814	7.56E-07
TUCP_003806	XLOC_166117	ENSRNOT00000067391	-0.963878664	4.56E-07
TUCP_003806	XLOC_166117	ENSRNOT00000039857	-0.960386387	7.19E-07
TUCP_003806	XLOC_166117	ENSRNOT00000009150	0.962260552	5.66E-07
TUCP_003806	XLOC_166117	ENSRNOT00000037446	0.957869987	9.74E-07
TUCP_003806	XLOC_166117	ENSRNOT00000084709	0.978814628	3.24E-08
TUCP_003806	XLOC_166117	ENSRNOT00000068033	0.957666612	9.97E-07
TUCP_003806	XLOC_166117	ENSRNOT00000009156	0.9656272	3.57E-07
TUCP_003806	XLOC_166117	ENSRNOT00000090912	0.968133612	2.45E-07
TUCP_003806	XLOC_166117	ENSRNOT00000025856	-0.959794591	7.73E-07
TUCP_003806	XLOC_166117	ENSRNOT00000026643	0.9560504	1.20E-06
TUCP_003806	XLOC_166117	ENSRNOT00000001403	0.968654781	2.26E-07
TUCP_003806	XLOC_166117	ENSRNOT00000001401	0.950734089	2.10E-06
TUCP_003806	XLOC_166117	ENSRNOT00000016132	-0.978411057	3.56E-08
TUCP_003806	XLOC_166117	ENSRNOT00000058495	-0.954487692	1.42E-06
TUCP_003806	XLOC_166117	ENSRNOT00000013067	-0.980752445	2.01E-08
TUCP_003806	XLOC_166117	ENSRNOT00000004351	-0.957365608	1.03E-06
TUCP_003806	XLOC_166117	ENSRNOT00000027145	-0.951419225	1.96E-06
TUCP_003806	XLOC_166117	ENSRNOT00000002313	-0.954881	1.36E-06
TUCP_003806	XLOC_166117	ENSRNOT00000034692	-0.969456514	1.99E-07
TUCP_003806	XLOC_166117	ENSRNOT00000016637	-0.950739893	2.10E-06
TUCP_003806	XLOC_166117	ENSRNOT00000020910	0.957728405	9.90E-07
TUCP_003806	XLOC_166117	ENSRNOT00000031979	-0.951033918	2.04E-06
TUCP_003806	XLOC_166117	ENSRNOT00000079895	0.983573369	9.16E-09
TUCP_003806	XLOC_166117	ENSRNOT00000054948	0.956495222	1.14E-06
TUCP_003806	XLOC_166117	ENSRNOT00000074586	-0.976798419	5.09E-08
TUCP_003806	XLOC_166117	ENSRNOT00000075444	-0.964697906	4.07E-07
TUCP_003806	XLOC_166117	ENSRNOT00000021496	-0.955677828	1.25E-06
TUCP_003806	XLOC_166117	ENSRNOT00000002465	0.954147635	1.48E-06
TUCP_003806	XLOC_166117	ENSRNOT00000011880	0.972552343	1.17E-07
TUCP_003806	XLOC_166117	ENSRNOT00000028290	-0.956825226	1.10E-06
TUCP_003806	XLOC_166117	ENSRNOT00000003992	-0.960385205	7.19E-07
TUCP_003806	XLOC_166117	ENSRNOT00000020885	0.968511644	2.31E-07
TUCP_003806	XLOC_166117	ENSRNOT00000050515	0.968931541	2.16E-07
TUCP_003806	XLOC_166117	ENSRNOT00000089980	-0.953144491	1.64E-06
TUCP_003806	XLOC_166117	ENSRNOT00000078070	0.969104074	2.11E-07
TUCP_003806	XLOC_166117	ENSRNOT00000046745	0.979587055	2.70E-08
TUCP_003806	XLOC_166117	ENSRNOT00000021015	0.96538686	3.69E-07
TUCP_003806	XLOC_166117	ENSRNOT00000003840	0.968641548	2.27E-07

TUCP_003806	XLOC_166117	ENSRNOT00000023876	0.951642447	1.92E-06
TUCP_003806	XLOC_166117	ENSRNOT00000061201	-0.967036851	2.90E-07
TUCP_003806	XLOC_166117	ENSRNOT00000002187	-0.965590694	3.59E-07
TUCP_003806	XLOC_166117	ENSRNOT00000091488	0.969496061	1.98E-07
TUCP_003806	XLOC_166117	ENSRNOT00000019656	-0.968152211	2.45E-07
TUCP_003806	XLOC_166117	ENSRNOT00000049285	-0.958836382	8.69E-07
TUCP_003806	XLOC_166117	ENSRNOT00000015239	-0.970574341	1.65E-07
TUCP_003806	XLOC_166117	ENSRNOT00000003568	0.951021125	2.04E-06
TUCP_003806	XLOC_166117	ENSRNOT00000074649	0.987478904	2.37E-09
TUCP_003806	XLOC_166117	ENSRNOT00000082108	-0.950857729	2.08E-06
TUCP_003806	XLOC_166117	ENSRNOT00000018877	-0.963062174	5.09E-07
TUCP_003806	XLOC_166117	ENSRNOT00000077146	-0.981683677	1.57E-08
TUCP_003806	XLOC_166117	ENSRNOT00000031672	-0.952155419	1.82E-06
TUCP_003806	XLOC_166117	ENSRNOT00000006969	0.968128581	2.45E-07
TUCP_003806	XLOC_166117	ENSRNOT00000075209	-0.950471206	2.16E-06
TUCP_003806	XLOC_166117	ENSRNOT00000006395	-0.965940075	3.41E-07
TUCP_003806	XLOC_166117	ENSRNOT00000023977	-0.96431001	4.30E-07
TUCP_003806	XLOC_166117	ENSRNOT00000066133	-0.982880935	1.13E-08
TUCP_003806	XLOC_166117	ENSRNOT00000048933	-0.962858626	5.23E-07
TUCP_003806	XLOC_166117	ENSRNOT00000027970	0.958903166	8.62E-07
TUCP_003806	XLOC_166117	ENSRNOT00000012432	-0.954859146	1.37E-06
TUCP_003806	XLOC_166117	ENSRNOT00000085596	0.964029974	4.46E-07
TUCP_003806	XLOC_166117	ENSRNOT00000035385	0.961367987	6.35E-07
TUCP_003806	XLOC_166117	ENSRNOT00000003102	0.950428587	2.17E-06
TUCP_003806	XLOC_166117	ENSRNOT00000057738	0.951587412	1.93E-06
TUCP_003806	XLOC_166117	ENSRNOT00000089007	-0.954717136	1.39E-06
TUCP_003806	XLOC_166117	ENSRNOT00000018170	-0.954134833	1.48E-06
TUCP_003806	XLOC_166117	ENSRNOT00000010915	-0.952018719	1.85E-06
TUCP_003806	XLOC_166117	ENSRNOT00000040069	0.956542713	1.13E-06
TUCP_003806	XLOC_166117	ENSRNOT00000015624	0.96214929	5.74E-07
TUCP_003806	XLOC_166117	ENSRNOT00000085295	-0.955976172	1.21E-06
TUCP_003806	XLOC_166117	ENSRNOT00000035474	0.965462066	3.65E-07
TUCP_003806	XLOC_166117	ENSRNOT00000002114	-0.969539574	1.96E-07
TUCP_003806	XLOC_166117	ENSRNOT00000039102	0.965693894	3.53E-07
TUCP_003806	XLOC_166117	ENSRNOT00000023807	-0.961711232	6.08E-07
TUCP_003806	XLOC_166117	ENSRNOT00000078856	-0.95341809	1.60E-06
TUCP_003806	XLOC_166117	ENSRNOT00000091205	0.981100168	1.84E-08
TUCP_003806	XLOC_166117	ENSRNOT00000009742	-0.954679446	1.40E-06
TUCP_003806	XLOC_166117	ENSRNOT00000010983	0.950516598	2.15E-06
TUCP_003806	XLOC_166117	ENSRNOT00000007369	0.971711777	1.36E-07
TUCP_003806	XLOC_166117	ENSRNOT00000004787	-0.966996654	2.92E-07
TUCP_003806	XLOC_166117	ENSRNOT00000060698	-0.980476874	2.16E-08
TUCP_003806	XLOC_166117	ENSRNOT00000058351	0.959990564	7.55E-07
TUCP_003806	XLOC_166117	ENSRNOT00000004412	0.950325167	2.19E-06
TUCP_003806	XLOC_166117	ENSRNOT00000010748	0.977048885	4.83E-08
TUCP_003806	XLOC_166117	ENSRNOT00000080207	0.961261608	6.44E-07
TUCP_003806	XLOC_166117	ENSRNOT00000061100	0.980359721	2.23E-08
TUCP_003806	XLOC_166117	ENSRNOT00000017995	0.980777221	2.00E-08
TUCP_003806	XLOC_166117	ENSRNOT00000017776	0.975916762	6.13E-08
TUCP_003806	XLOC_166117	ENSRNOT00000005144	0.978294585	3.66E-08
TUCP_003806	XLOC_166117	ENSRNOT00000021534	-0.969234228	2.06E-07

TUCP_003806	XLOC_166117	ENSRNOT00000018998	0.95783113	9.78E-07
TUCP_003806	XLOC_166117	ENSRNOT00000088679	-0.953582341	1.57E-06
TUCP_003806	XLOC_166117	ENSRNOT00000004463	0.972866619	1.11E-07
TUCP_003806	XLOC_166117	ENSRNOT00000023256	0.967195468	2.83E-07
TUCP_003806	XLOC_166117	ENSRNOT00000016776	-0.959798013	7.73E-07
TUCP_003806	XLOC_166117	ENSRNOT00000022097	0.971203992	1.49E-07
TUCP_003806	XLOC_166117	ENSRNOT00000008987	-0.971549075	1.40E-07
TUCP_003806	XLOC_166117	ENSRNOT00000090299	0.954096841	1.49E-06
TUCP_003806	XLOC_166117	ENSRNOT00000092876	-0.969993979	1.82E-07
TUCP_003806	XLOC_166117	ENSRNOT00000024253	0.969806376	1.88E-07
TUCP_003806	XLOC_166117	ENSRNOT00000088073	-0.958829363	8.69E-07
TUCP_003806	XLOC_166117	ENSRNOT00000059854	-0.957201494	1.05E-06
TUCP_003806	XLOC_166117	ENSRNOT00000086868	0.963133548	5.04E-07
TUCP_003806	XLOC_166117	ENSRNOT00000045833	0.955849681	1.23E-06
TUCP_003806	XLOC_166117	ENSRNOT00000009080	-0.951940298	1.86E-06
TUCP_003806	XLOC_166117	ENSRNOT00000063930	-0.980167237	2.34E-08
TUCP_003806	XLOC_166117	ENSRNOT00000046215	-0.952228582	1.81E-06
TUCP_003806	XLOC_166117	ENSRNOT00000013116	-0.951753642	1.90E-06
TUCP_003806	XLOC_166117	ENSRNOT00000020453	-0.974448934	8.22E-08
TUCP_003806	XLOC_166117	ENSRNOT00000013025	-0.9684415	2.34E-07
TUCP_003806	XLOC_166117	ENSRNOT00000016012	-0.966024295	3.37E-07
TUCP_003806	XLOC_166117	ENSRNOT00000041237	-0.958420558	9.13E-07
TUCP_003806	XLOC_166117	ENSRNOT00000088555	-0.961999088	5.85E-07
TUCP_003806	XLOC_166117	ENSRNOT00000088599	0.955903025	1.22E-06
TUCP_003806	XLOC_166117	ENSRNOT00000087838	-0.951774819	1.89E-06
TUCP_003806	XLOC_166117	ENSRNOT00000081981	0.965326546	3.72E-07
TUCP_003806	XLOC_166117	ENSRNOT00000001679	0.950445878	2.16E-06
TUCP_003806	XLOC_166117	ENSRNOT00000013758	0.953646383	1.56E-06
TUCP_003806	XLOC_166117	ENSRNOT00000013613	-0.959712798	7.81E-07
TUCP_003806	XLOC_166117	ENSRNOT00000026531	-0.974028618	8.91E-08
TUCP_003806	XLOC_166117	ENSRNOT00000010419	0.965009225	3.90E-07
TUCP_003806	XLOC_166117	ENSRNOT00000019057	-0.96466785	4.09E-07
TUCP_003806	XLOC_166117	ENSRNOT00000003326	-0.950443472	2.17E-06
TUCP_003806	XLOC_166117	ENSRNOT00000021629	-0.961516089	6.23E-07
TUCP_003806	XLOC_166117	ENSRNOT00000047836	-0.962268629	5.65E-07
TUCP_003806	XLOC_166117	ENSRNOT00000015476	-0.965951483	3.40E-07
TUCP_003806	XLOC_166117	ENSRNOT00000075630	-0.959814896	7.71E-07
TUCP_003806	XLOC_166117	ENSRNOT00000025079	-0.984358097	7.18E-09
TUCP_003806	XLOC_166117	ENSRNOT00000048754	-0.983714842	8.78E-09
TUCP_003806	XLOC_166117	ENSRNOT00000055602	-0.970681778	1.62E-07
TUCP_003806	XLOC_166117	ENSRNOT00000093174	0.978282228	3.67E-08
TUCP_003806	XLOC_166117	ENSRNOT00000021458	-0.960356658	7.21E-07
TUCP_003806	XLOC_166117	ENSRNOT00000011926	0.964712851	4.06E-07
TUCP_003806	XLOC_166117	ENSRNOT00000054761	-0.966550419	3.12E-07
TUCP_003806	XLOC_166117	ENSRNOT00000007824	-0.971643356	1.38E-07
TUCP_003806	XLOC_166117	ENSRNOT00000019816	-0.962321148	5.61E-07
TUCP_003806	XLOC_166117	ENSRNOT00000020681	-0.96614518	3.31E-07
TUCP_003806	XLOC_166117	ENSRNOT00000057898	-0.96716073	2.85E-07
TUCP_003806	XLOC_166117	ENSRNOT00000024677	0.974605976	7.97E-08
TUCP_003806	XLOC_166117	ENSRNOT00000091602	-0.956310186	1.16E-06
TUCP_003806	XLOC_166117	ENSRNOT00000056100	-0.9555591	1.27E-06

TUCP_003806	XLOC_166117	ENSRNOT00000012671	0.95625391	1.17E-06
TUCP_003806	XLOC_166117	ENSRNOT00000018634	-0.959584217	7.93E-07
TUCP_003806	XLOC_166117	ENSRNOT00000071540	0.978045153	3.87E-08
TUCP_003806	XLOC_166117	ENSRNOT00000031701	-0.95708625	1.07E-06
TUCP_003806	XLOC_166117	ENSRNOT00000072849	-0.962263514	5.66E-07
TUCP_003806	XLOC_166117	ENSRNOT00000073643	-0.9552675	1.31E-06
TUCP_003806	XLOC_166117	ENSRNOT00000078349	-0.958952445	8.57E-07
TUCP_003806	XLOC_166117	ENSRNOT00000011654	-0.957211961	1.05E-06
TUCP_003806	XLOC_166117	ENSRNOT00000022502	-0.963964777	4.50E-07
TUCP_003806	XLOC_166117	ENSRNOT00000060811	0.954522933	1.42E-06
TUCP_003806	XLOC_166117	ENSRNOT00000017556	0.950847667	2.08E-06
TUCP_003806	XLOC_166117	ENSRNOT00000087443	0.954842223	1.37E-06
TUCP_003806	XLOC_166117	ENSRNOT00000021803	-0.971471555	1.42E-07
TUCP_003806	XLOC_166117	ENSRNOT00000020147	0.955606543	1.26E-06
TUCP_003806	XLOC_166117	ENSRNOT00000024352	0.990859117	4.95E-10
TUCP_003806	XLOC_166117	ENSRNOT00000082627	-0.96215152	5.74E-07
TUCP_003806	XLOC_166117	ENSRNOT00000072118	0.956162865	1.18E-06
TUCP_003806	XLOC_166117	ENSRNOT00000003780	0.974725479	7.79E-08
TUCP_003806	XLOC_166117	ENSRNOT00000003787	-0.963546455	4.77E-07
TUCP_003806	XLOC_166117	ENSRNOT00000023262	-0.96169379	6.09E-07
TUCP_003806	XLOC_166117	ENSRNOT00000058312	0.95704844	1.07E-06
TUCP_003806	XLOC_166117	ENSRNOT00000013274	0.953133584	1.65E-06
TUCP_003806	XLOC_166117	ENSRNOT00000081800	0.956361513	1.16E-06
TUCP_003806	XLOC_166117	ENSRNOT00000022342	0.953810187	1.53E-06
TUCP_003806	XLOC_166117	ENSRNOT00000025104	-0.956991709	1.08E-06
TUCP_003806	XLOC_166117	ENSRNOT00000055957	-0.955039179	1.34E-06
TUCP_003806	XLOC_166117	ENSRNOT00000016739	0.955797945	1.23E-06
TUCP_003806	XLOC_166117	ENSRNOT00000068381	0.961968827	5.88E-07
TUCP_003806	XLOC_166117	ENSRNOT00000009221	0.955371974	1.29E-06
TUCP_003806	XLOC_166117	ENSRNOT00000074054	0.957796117	9.82E-07
TUCP_003806	XLOC_166117	ENSRNOT00000068026	-0.965211341	3.79E-07
TUCP_003806	XLOC_166117	ENSRNOT00000026089	0.965801239	3.48E-07
TUCP_003806	XLOC_166117	ENSRNOT00000085621	0.959515272	8.00E-07
TUCP_003806	XLOC_166117	ENSRNOT00000014821	-0.964301053	4.30E-07
TUCP_003806	XLOC_166117	ENSRNOT00000058382	0.976562056	5.36E-08
TUCP_003806	XLOC_166117	ENSRNOT00000008889	-0.956970559	1.08E-06
TUCP_003806	XLOC_166117	ENSRNOT00000055683	0.961871408	5.95E-07
TUCP_003806	XLOC_166117	ENSRNOT00000005587	-0.974821266	7.64E-08
TUCP_003806	XLOC_166117	ENSRNOT00000017746	-0.961534401	6.22E-07
TUCP_003806	XLOC_166117	ENSRNOT00000043216	0.957965241	9.63E-07
TUCP_003806	XLOC_166117	ENSRNOT00000023567	-0.951311973	1.99E-06
TUCP_003806	XLOC_166117	ENSRNOT00000026174	0.977039793	4.84E-08
TUCP_003806	XLOC_166117	ENSRNOT00000005073	0.951679036	1.91E-06
TUCP_003806	XLOC_166117	ENSRNOT00000065529	0.979106028	3.03E-08
TUCP_003806	XLOC_166117	ENSRNOT00000073983	0.958336405	9.22E-07
TUCP_003806	XLOC_166117	ENSRNOT00000000898	0.953932157	1.51E-06
TUCP_003806	XLOC_166117	ENSRNOT00000013375	-0.951092857	2.03E-06
TUCP_003806	XLOC_166117	ENSRNOT00000081725	0.957274194	1.04E-06
TUCP_003806	XLOC_166117	ENSRNOT00000005459	0.967354493	2.76E-07
TUCP_003806	XLOC_166117	ENSRNOT00000009135	0.953781201	1.54E-06
TUCP_003806	XLOC_166117	ENSRNOT00000027910	0.988186107	1.78E-09

TUCP_003806	XLOC_166117	ENSRNOT00000090262	0.958995397	8.52E-07
TUCP_003806	XLOC_166117	ENSRNOT00000002771	0.956893933	1.09E-06
TUCP_003806	XLOC_166117	ENSRNOT00000037015	0.964073305	4.44E-07
TUCP_003806	XLOC_166117	ENSRNOT00000079442	0.957451675	1.02E-06
TUCP_003806	XLOC_166117	ENSRNOT00000054976	-0.950101114	2.24E-06
TUCP_003806	XLOC_166117	ENSRNOT00000017765	0.965891443	3.43E-07
TUCP_003806	XLOC_166117	ENSRNOT00000028885	-0.961853267	5.97E-07
TUCP_003806	XLOC_166117	ENSRNOT00000010783	-0.953835663	1.53E-06
TUCP_003806	XLOC_166117	ENSRNOT00000016372	-0.952172189	1.82E-06
TUCP_003806	XLOC_166117	ENSRNOT00000066032	0.972943665	1.09E-07
TUCP_003806	XLOC_166117	ENSRNOT00000027616	-0.956826407	1.10E-06
TUCP_003806	XLOC_166117	ENSRNOT00000028876	-0.96059669	7.00E-07
TUCP_003806	XLOC_166117	ENSRNOT00000002510	-0.950674602	2.12E-06
TUCP_003806	XLOC_166117	ENSRNOT00000001783	0.964790259	4.02E-07
TUCP_003806	XLOC_166117	ENSRNOT00000021145	-0.955659523	1.25E-06
TUCP_003806	XLOC_166117	ENSRNOT00000025314	0.953012832	1.67E-06
TUCP_003806	XLOC_166117	ENSRNOT00000019040	-0.952319055	1.79E-06
TUCP_003806	XLOC_166117	ENSRNOT00000057313	0.951334221	1.98E-06
TUCP_003806	XLOC_166117	ENSRNOT00000038212	-0.9619726	5.87E-07
TUCP_003806	XLOC_166117	ENSRNOT00000020809	-0.956574137	1.13E-06
TUCP_003806	XLOC_166117	ENSRNOT00000082151	-0.962654703	5.37E-07
TUCP_003806	XLOC_166117	ENSRNOT00000008982	0.975599079	6.54E-08
TUCP_003806	XLOC_166117	ENSRNOT00000050138	0.971964274	1.30E-07
TUCP_003806	XLOC_166117	ENSRNOT00000003359	-0.971380428	1.44E-07
TUCP_003806	XLOC_166117	ENSRNOT00000039607	-0.951040234	2.04E-06
TUCP_003806	XLOC_166117	ENSRNOT00000038511	-0.960512907	7.08E-07
TUCP_003806	XLOC_166117	ENSRNOT00000011984	0.966256965	3.26E-07
TUCP_003806	XLOC_166117	ENSRNOT00000020669	0.951044634	2.04E-06
TUCP_003806	XLOC_166117	ENSRNOT00000085971	-0.953473901	1.59E-06
TUCP_003806	XLOC_166117	ENSRNOT00000084069	0.953077486	1.66E-06
TUCP_003806	XLOC_166117	ENSRNOT00000031230	0.957278973	1.04E-06
TUCP_003806	XLOC_166117	ENSRNOT00000022142	0.956439321	1.15E-06
TUCP_003806	XLOC_166117	ENSRNOT00000076300	-0.961098569	6.57E-07
TUCP_003806	XLOC_166117	ENSRNOT00000025086	-0.968639606	2.27E-07
TUCP_003806	XLOC_166117	ENSRNOT00000002292	0.956395747	1.15E-06
TUCP_003806	XLOC_166117	ENSRNOT00000052120	0.958210641	9.36E-07
TUCP_003806	XLOC_166117	ENSRNOT00000078306	0.953959444	1.51E-06
TUCP_003806	XLOC_166117	ENSRNOT00000019820	0.977220997	4.65E-08
TUCP_003806	XLOC_166117	ENSRNOT00000015946	-0.952133826	1.83E-06
TUCP_003806	XLOC_166117	ENSRNOT00000022461	-0.951823677	1.88E-06
TUCP_003806	XLOC_166117	ENSRNOT00000004172	0.962290106	5.64E-07
TUCP_003806	XLOC_166117	ENSRNOT00000023965	-0.957875928	9.73E-07
TUCP_003806	XLOC_166117	ENSRNOT00000011694	-0.950224119	2.21E-06
TUCP_003806	XLOC_166117	ENSRNOT00000029125	0.973135861	1.05E-07
TUCP_003806	XLOC_166117	ENSRNOT00000085279	0.965799316	3.48E-07
TUCP_003806	XLOC_166117	ENSRNOT00000012528	0.971179627	1.49E-07
TUCP_003806	XLOC_166117	ENSRNOT00000068184	-0.96024563	7.31E-07
TUCP_003806	XLOC_166117	ENSRNOT00000005389	0.95166113	1.92E-06
TUCP_003806	XLOC_166117	ENSRNOT00000005387	0.956087681	1.19E-06
TUCP_003806	XLOC_166117	ENSRNOT00000077888	-0.95814982	9.42E-07
TUCP_003806	XLOC_166117	ENSRNOT00000005381	0.955775553	1.24E-06

TUCP_003806	XLOC_166117	ENSRNOT00000015092	0.95286476	1.69E-06
TUCP_003806	XLOC_166117	ENSRNOT00000028907	0.972602968	1.16E-07
TUCP_003806	XLOC_166117	ENSRNOT00000083632	-0.957010125	1.08E-06
TUCP_003806	XLOC_166117	ENSRNOT00000010224	0.970508234	1.67E-07
TUCP_003806	XLOC_166117	ENSRNOT00000025147	0.968264741	2.40E-07
TUCP_003806	XLOC_166117	ENSRNOT00000087278	-0.975436163	6.76E-08
TUCP_003806	XLOC_166117	ENSRNOT00000007175	0.950431773	2.17E-06
TUCP_003806	XLOC_166117	ENSRNOT00000025887	-0.950859456	2.08E-06
TUCP_003806	XLOC_166117	ENSRNOT00000078862	0.959984633	7.55E-07
TUCP_003806	XLOC_166117	ENSRNOT00000005311	0.957210399	1.05E-06
TUCP_003806	XLOC_166117	ENSRNOT00000017687	-0.952681157	1.73E-06
TUCP_003806	XLOC_166117	ENSRNOT00000015799	0.96013584	7.41E-07
TUCP_003806	XLOC_166117	ENSRNOT00000047105	0.963265958	4.95E-07
TUCP_003806	XLOC_166117	ENSRNOT00000031904	0.952663824	1.73E-06
TUCP_003806	XLOC_166117	ENSRNOT00000039551	-0.960024219	7.52E-07
TUCP_003806	XLOC_166117	ENSRNOT00000082998	0.973671831	9.53E-08
TUCP_003806	XLOC_166117	ENSRNOT00000015006	0.961969612	5.88E-07
TUCP_003806	XLOC_166117	ENSRNOT00000007350	0.978885376	3.19E-08
TUCP_003806	XLOC_166117	ENSRNOT00000007351	-0.966040543	3.36E-07
TUCP_003806	XLOC_166117	ENSRNOT00000018936	-0.965715947	3.52E-07
TUCP_003806	XLOC_166117	ENSRNOT00000083578	0.984959582	5.91E-09
TUCP_003806	XLOC_166117	ENSRNOT00000020297	0.964999125	3.90E-07
TUCP_003806	XLOC_166117	ENSRNOT00000023638	0.985981712	4.16E-09
TUCP_003806	XLOC_166117	ENSRNOT00000022726	0.950723509	2.11E-06
TUCP_003806	XLOC_166117	ENSRNOT00000005220	0.981322257	1.74E-08
TUCP_003806	XLOC_166117	ENSRNOT00000023144	0.953302247	1.62E-06
TUCP_003806	XLOC_166117	ENSRNOT00000045326	0.963386026	4.87E-07
TUCP_003806	XLOC_166117	ENSRNOT00000016649	0.964513049	4.18E-07
TUCP_003806	XLOC_166117	ENSRNOT00000074023	0.96772156	2.61E-07
TUCP_003806	XLOC_166117	ENSRNOT00000009073	-0.958565531	8.97E-07
TUCP_003806	XLOC_166117	ENSRNOT00000067170	-0.956578111	1.13E-06
TUCP_003806	XLOC_166117	ENSRNOT00000048910	-0.960373454	7.20E-07
TUCP_003806	XLOC_166117	ENSRNOT00000018341	-0.968285846	2.40E-07
TUCP_003806	XLOC_166117	ENSRNOT00000065076	0.96584621	3.46E-07
TUCP_003806	XLOC_166117	ENSRNOT00000014235	0.953015727	1.67E-06
TUCP_003806	XLOC_166117	ENSRNOT00000013516	0.95748035	1.02E-06
TUCP_003806	XLOC_166117	ENSRNOT00000028405	-0.952239262	1.81E-06
TUCP_003806	XLOC_166117	ENSRNOT00000013695	-0.951728595	1.90E-06
TUCP_003806	XLOC_166117	ENSRNOT00000088125	0.977794402	4.10E-08
TUCP_003806	XLOC_166117	ENSRNOT00000012177	-0.963038397	5.11E-07
TUCP_003806	XLOC_166117	ENSRNOT00000080928	0.969667679	1.92E-07
TUCP_003806	XLOC_166117	ENSRNOT00000027760	0.958369455	9.18E-07
TUCP_003806	XLOC_166117	ENSRNOT00000024402	0.976066056	5.94E-08
TUCP_003806	XLOC_166117	ENSRNOT00000089545	-0.953669669	1.56E-06
TUCP_003806	XLOC_166117	ENSRNOT00000019519	0.953190189	1.64E-06
TUCP_003806	XLOC_166117	ENSRNOT00000089933	0.978594058	3.41E-08
TUCP_003806	XLOC_166117	ENSRNOT00000077908	0.978452226	3.53E-08
TUCP_003806	XLOC_166117	ENSRNOT00000003641	0.963298157	4.93E-07
TUCP_003806	XLOC_166117	ENSRNOT00000026481	0.979531736	2.73E-08
TUCP_003806	XLOC_166117	ENSRNOT00000010463	0.952297969	1.80E-06
TUCP_003806	XLOC_166117	ENSRNOT00000084012	0.968263487	2.40E-07

TUCP_003806	XLOC_166117	ENSRNOT00000027232	0.961918204	5.92E-07
TUCP_003806	XLOC_166117	ENSRNOT00000036931	-0.964416162	4.23E-07
TUCP_003806	XLOC_166117	ENSRNOT00000067822	0.96932478	2.03E-07
TUCP_003806	XLOC_166117	ENSRNOT00000011832	-0.965340021	3.72E-07
TUCP_003806	XLOC_166117	ENSRNOT00000024066	0.959096004	8.42E-07
TUCP_003806	XLOC_166117	ENSRNOT00000021030	-0.951164264	2.01E-06
TUCP_003806	XLOC_166117	ENSRNOT00000077727	-0.980869657	1.95E-08
TUCP_003806	XLOC_166117	ENSRNOT00000044346	-0.965367104	3.70E-07
TUCP_003806	XLOC_166117	ENSRNOT00000020197	-0.965019001	3.89E-07
TUCP_003806	XLOC_166117	ENSRNOT00000000203	-0.952118125	1.83E-06
TUCP_003806	XLOC_166117	ENSRNOT00000068745	0.96449652	4.19E-07
TUCP_003806	XLOC_166117	ENSRNOT00000004032	0.962934288	5.18E-07
TUCP_003806	XLOC_166117	ENSRNOT00000048613	0.985991019	4.15E-09
TUCP_003806	XLOC_166117	ENSRNOT00000091909	0.992151819	2.31E-10
TUCP_003806	XLOC_166117	ENSRNOT00000001081	0.966413393	3.18E-07
TUCP_003806	XLOC_166117	ENSRNOT00000009563	-0.957295756	1.04E-06
TUCP_003806	XLOC_166117	ENSRNOT00000064535	-0.979577078	2.70E-08
TUCP_003806	XLOC_166117	ENSRNOT00000003544	-0.966328978	3.22E-07
TUCP_003806	XLOC_166117	ENSRNOT00000086119	-0.970363599	1.71E-07
TUCP_003806	XLOC_166117	ENSRNOT00000022190	-0.973720635	9.45E-08
TUCP_003806	XLOC_166117	ENSRNOT00000025913	-0.968267277	2.40E-07
TUCP_003806	XLOC_166117	ENSRNOT00000077220	-0.964693204	4.07E-07
TUCP_003806	XLOC_166117	ENSRNOT00000065983	0.963827793	4.59E-07
TUCP_003806	XLOC_166117	ENSRNOT00000030782	-0.961567868	6.19E-07
TUCP_003806	XLOC_166117	ENSRNOT00000007882	-0.959456313	8.06E-07
TUCP_003806	XLOC_166117	ENSRNOT00000073919	0.968588485	2.28E-07
TUCP_003806	XLOC_166117	ENSRNOT00000067780	-0.981257603	1.77E-08
TUCP_003806	XLOC_166117	ENSRNOT00000086577	0.957550572	1.01E-06
TUCP_003806	XLOC_166117	ENSRNOT00000025966	0.9574605	1.02E-06
TUCP_003806	XLOC_166117	ENSRNOT00000073910	-0.956695149	1.12E-06
TUCP_003806	XLOC_166117	ENSRNOT00000086590	0.974323735	8.42E-08
TUCP_003806	XLOC_166117	ENSRNOT00000034883	-0.96737204	2.76E-07
TUCP_003806	XLOC_166117	ENSRNOT00000020155	0.957809177	9.81E-07
TUCP_003806	XLOC_166117	ENSRNOT00000072357	-0.975927919	6.11E-08
TUCP_003806	XLOC_166117	ENSRNOT00000017629	0.955723139	1.24E-06
TUCP_003806	XLOC_166117	ENSRNOT00000087011	-0.961130185	6.55E-07
TUCP_003806	XLOC_166117	ENSRNOT00000005910	-0.962290253	5.64E-07
TUCP_003806	XLOC_166117	ENSRNOT00000013260	0.953250047	1.63E-06
TUCP_003806	XLOC_166117	ENSRNOT00000076485	-0.971752171	1.35E-07
TUCP_003806	XLOC_166117	ENSRNOT00000005664	0.954951054	1.35E-06
TUCP_003806	XLOC_166117	ENSRNOT00000078475	0.987013417	2.85E-09
TUCP_003806	XLOC_166117	ENSRNOT00000040169	0.969870594	1.86E-07
TUCP_003806	XLOC_166117	ENSRNOT00000026249	-0.953209319	1.63E-06
TUCP_003806	XLOC_166117	ENSRNOT00000036051	0.952931746	1.68E-06
TUCP_003806	XLOC_166117	ENSRNOT00000016722	0.98209504	1.41E-08
TUCP_003806	XLOC_166117	ENSRNOT00000073526	0.970622847	1.64E-07
TUCP_003806	XLOC_166117	ENSRNOT00000089970	0.955581854	1.26E-06
TUCP_003806	XLOC_166117	ENSRNOT00000000013	0.975967357	6.06E-08
TUCP_003806	XLOC_166117	ENSRNOT00000080591	0.95102183	2.04E-06
TUCP_003806	XLOC_166117	ENSRNOT00000025523	0.962829486	5.25E-07
TUCP_003806	XLOC_166117	ENSRNOT00000005161	0.978265842	3.68E-08

TUCP_003806	XLOC_166117	ENSRNOT00000037955	-0.961415792	6.31E-07
TUCP_003806	XLOC_166117	ENSRNOT00000055582	0.961369401	6.35E-07
TUCP_003806	XLOC_166117	ENSRNOT00000022074	0.952846008	1.70E-06
TUCP_003806	XLOC_166117	ENSRNOT00000016220	-0.95091933	2.07E-06
TUCP_003806	XLOC_166117	ENSRNOT00000037790	0.965006112	3.90E-07
TUCP_003806	XLOC_166117	ENSRNOT00000004999	0.976358408	5.59E-08
TUCP_003806	XLOC_166117	ENSRNOT00000009122	0.964490267	4.19E-07
TUCP_003806	XLOC_166117	ENSRNOT00000013463	0.955228827	1.31E-06
TUCP_003806	XLOC_166117	ENSRNOT00000088054	0.979758149	2.59E-08
TUCP_003806	XLOC_166117	ENSRNOT00000019554	0.957831438	9.78E-07
TUCP_003806	XLOC_166117	ENSRNOT00000047663	-0.962055656	5.81E-07
TUCP_003806	XLOC_166117	ENSRNOT00000035700	0.952254101	1.80E-06
TUCP_003806	XLOC_166117	ENSRNOT00000029202	-0.97802056	3.89E-08
TUCP_003806	XLOC_166117	ENSRNOT00000025791	-0.965513066	3.63E-07
TUCP_003806	XLOC_166117	ENSRNOT00000005489	0.982269892	1.34E-08
TUCP_003806	XLOC_166117	ENSRNOT00000025794	-0.958794094	8.73E-07
TUCP_003806	XLOC_166117	ENSRNOT00000060494	0.951241613	2.00E-06
TUCP_003806	XLOC_166117	ENSRNOT00000058111	-0.956262319	1.17E-06
TUCP_003806	XLOC_166117	ENSRNOT00000084422	0.957641314	1.00E-06
TUCP_003806	XLOC_166117	ENSRNOT00000050834	-0.961012219	6.64E-07
TUCP_003806	XLOC_166117	ENSRNOT00000088279	0.970799174	1.59E-07
TUCP_003806	XLOC_166117	ENSRNOT00000009994	-0.958154808	9.42E-07
TUCP_003806	XLOC_166117	ENSRNOT00000013759	-0.953903828	1.52E-06
TUCP_003806	XLOC_166117	ENSRNOT00000012736	0.963749629	4.64E-07
TUCP_003806	XLOC_166117	ENSRNOT00000077367	-0.957531517	1.01E-06
TUCP_003806	XLOC_166117	ENSRNOT00000002445	-0.950564969	2.14E-06
TUCP_003806	XLOC_166117	ENSRNOT00000024841	-0.959913355	7.62E-07
TUCP_003806	XLOC_166117	ENSRNOT00000009462	0.959966893	7.57E-07
TUCP_003806	XLOC_166117	ENSRNOT00000009345	-0.95650603	1.14E-06
TUCP_003806	XLOC_166117	ENSRNOT00000027875	0.957599718	1.00E-06
TUCP_003806	XLOC_166117	ENSRNOT00000079011	-0.955583084	1.26E-06
TUCP_003806	XLOC_166117	ENSRNOT00000018074	-0.984889692	6.05E-09
TUCP_003806	XLOC_166117	ENSRNOT00000037679	0.967828587	2.57E-07
TUCP_003806	XLOC_166117	ENSRNOT00000014124	0.968234772	2.41E-07
TUCP_003806	XLOC_166117	ENSRNOT00000043581	-0.963662175	4.69E-07
TUCP_003806	XLOC_166117	ENSRNOT00000000120	0.971118075	1.51E-07
TUCP_003806	XLOC_166117	ENSRNOT00000013632	0.967541912	2.69E-07
TUCP_003806	XLOC_166117	ENSRNOT00000078906	-0.966519086	3.13E-07
TUCP_003806	XLOC_166117	ENSRNOT00000010418	0.971782212	1.34E-07
TUCP_003806	XLOC_166117	ENSRNOT00000084049	0.957784264	9.84E-07
TUCP_003806	XLOC_166117	ENSRNOT00000009900	-0.960933693	6.71E-07
TUCP_003806	XLOC_166117	ENSRNOT00000085060	0.962137999	5.75E-07
TUCP_003806	XLOC_166117	ENSRNOT00000066561	-0.953572544	1.57E-06
TUCP_003806	XLOC_166117	ENSRNOT00000019172	-0.96435727	4.27E-07
TUCP_003806	XLOC_166117	ENSRNOT00000064798	0.955818907	1.23E-06
TUCP_003806	XLOC_166117	ENSRNOT00000015495	-0.980493784	2.15E-08
TUCP_003806	XLOC_166117	ENSRNOT00000032244	0.957831998	9.78E-07
TUCP_003806	XLOC_166117	ENSRNOT00000019910	-0.959760281	7.77E-07
TUCP_003806	XLOC_166117	ENSRNOT00000040393	0.98375696	8.67E-09
TUCP_003806	XLOC_166117	ENSRNOT00000025097	0.960964201	6.68E-07
TUCP_003806	XLOC_166117	ENSRNOT00000067901	-0.970159941	1.77E-07

TUCP_003806	XLOC_166117	ENSRNOT00000036750	0.964981748	3.91E-07
TUCP_003806	XLOC_166117	ENSRNOT00000024659	0.952924748	1.68E-06
TUCP_003806	XLOC_166117	ENSRNOT00000011511	-0.951718723	1.90E-06
TUCP_003806	XLOC_166117	ENSRNOT00000021674	0.984956952	5.92E-09
TUCP_003806	XLOC_166117	ENSRNOT00000064700	0.961619863	6.15E-07
TUCP_003806	XLOC_166117	ENSRNOT00000036613	-0.977659636	4.22E-08
TUCP_003806	XLOC_166117	ENSRNOT00000083127	0.961474181	6.26E-07
TUCP_003806	XLOC_166117	ENSRNOT00000025705	0.984433701	7.01E-09
TUCP_003806	XLOC_166117	ENSRNOT00000015479	0.968541142	2.30E-07
TUCP_003806	XLOC_166117	ENSRNOT00000004140	0.977814295	4.08E-08
TUCP_003806	XLOC_166117	ENSRNOT00000008113	-0.970771629	1.60E-07
TUCP_003806	XLOC_166117	ENSRNOT00000022552	-0.952059102	1.84E-06
TUCP_003806	XLOC_166117	ENSRNOT00000036195	-0.951400443	1.97E-06
TUCP_003806	XLOC_166117	ENSRNOT00000009763	-0.952994299	1.67E-06
TUCP_003806	XLOC_166117	ENSRNOT00000007698	-0.961588334	6.17E-07
TUCP_003806	XLOC_166117	ENSRNOT00000016306	-0.965277519	3.75E-07
TUCP_003806	XLOC_166117	ENSRNOT00000019213	0.976595432	5.32E-08
TUCP_003806	XLOC_166117	ENSRNOT00000020051	0.962276239	5.65E-07
TUCP_003806	XLOC_166117	ENSRNOT00000087062	0.954121913	1.48E-06
TUCP_003806	XLOC_166117	ENSRNOT00000015678	-0.961246826	6.45E-07
TUCP_003806	XLOC_166117	ENSRNOT00000082508	-0.956397017	1.15E-06
TUCP_003806	XLOC_166117	ENSRNOT00000073193	-0.959410312	8.10E-07
TUCP_003806	XLOC_166117	ENSRNOT00000082863	0.957327947	1.04E-06
TUCP_003806	XLOC_166117	ENSRNOT00000049459	-0.975670007	6.45E-08
TUCP_003806	XLOC_166117	ENSRNOT00000065947	0.9750945	7.24E-08
TUCP_003806	XLOC_166117	ENSRNOT00000071166	0.954862817	1.37E-06
TUCP_003806	XLOC_166117	ENSRNOT00000084567	0.951378944	1.97E-06
TUCP_003806	XLOC_166117	ENSRNOT00000001118	0.983959237	8.14E-09
TUCP_003806	XLOC_166117	ENSRNOT00000091596	-0.958601489	8.93E-07
TUCP_003806	XLOC_166117	ENSRNOT00000043345	0.963463827	4.82E-07
TUCP_003806	XLOC_166117	ENSRNOT00000090461	0.953280151	1.62E-06
TUCP_003806	XLOC_166117	ENSRNOT00000015073	0.957154326	1.06E-06
TUCP_003806	XLOC_166117	ENSRNOT00000001507	0.99055412	5.83E-10
TUCP_003806	XLOC_166117	ENSRNOT00000066947	-0.95073976	2.10E-06
TUCP_003806	XLOC_166117	ENSRNOT00000083568	0.974075493	8.83E-08
TUCP_003806	XLOC_166117	ENSRNOT00000018584	0.961031932	6.63E-07
TUCP_003806	XLOC_166117	ENSRNOT00000039177	-0.965896145	3.43E-07
TUCP_003806	XLOC_166117	ENSRNOT00000081047	0.958856408	8.66E-07
TUCP_003806	XLOC_166117	ENSRNOT00000017271	0.96116637	6.52E-07
TUCP_003806	XLOC_166117	ENSRNOT00000004486	0.956437071	1.15E-06
TUCP_003806	XLOC_166117	ENSRNOT00000022676	-0.955560963	1.27E-06
TUCP_003806	XLOC_166117	ENSRNOT00000037456	0.974958662	7.44E-08
TUCP_003806	XLOC_166117	ENSRNOT00000030082	0.951078469	2.03E-06
TUCP_003806	XLOC_166117	ENSRNOT00000025517	0.965013233	3.89E-07
TUCP_003806	XLOC_166117	ENSRNOT00000025510	-0.962366864	5.58E-07
TUCP_003806	XLOC_166117	ENSRNOT00000000577	0.952223577	1.81E-06
TUCP_003806	XLOC_166117	ENSRNOT00000011499	-0.952470438	1.76E-06
TUCP_003806	XLOC_166117	ENSRNOT00000075424	0.980135983	2.36E-08
TUCP_003806	XLOC_166117	ENSRNOT00000065045	0.975062076	7.28E-08
TUCP_003806	XLOC_166117	ENSRNOT00000013176	-0.951716379	1.91E-06
TUCP_003806	XLOC_166117	ENSRNOT00000023989	0.963852165	4.57E-07

TUCP_003806	XLOC_166117	ENSRNOT00000088133	-0.951528241	1.94E-06
TUCP_003806	XLOC_166117	ENSRNOT00000080677	-0.952582636	1.74E-06
TUCP_003806	XLOC_166117	ENSRNOT00000046490	-0.953746326	1.54E-06
TUCP_003806	XLOC_166117	ENSRNOT00000033677	-0.972644333	1.15E-07
TUCP_003806	XLOC_166117	ENSRNOT00000083968	-0.951608828	1.93E-06
TUCP_003806	XLOC_166117	ENSRNOT00000037133	0.954038756	1.50E-06
TUCP_003806	XLOC_166117	ENSRNOT00000034298	0.963322342	4.91E-07
TUCP_003806	XLOC_166117	ENSRNOT00000026726	-0.950893195	2.07E-06
TUCP_003806	XLOC_166117	ENSRNOT00000009342	0.95494991	1.35E-06
TUCP_003806	XLOC_166117	ENSRNOT00000026831	0.954694634	1.39E-06
TUCP_003806	XLOC_166117	ENSRNOT00000074985	-0.95075017	2.10E-06
TUCP_003806	XLOC_166117	ENSRNOT00000020892	-0.950427098	2.17E-06
TUCP_003806	XLOC_166117	ENSRNOT00000016445	0.961881705	5.94E-07
TUCP_003806	XLOC_166117	ENSRNOT00000011898	-0.953375445	1.60E-06
TUCP_003806	XLOC_166117	ENSRNOT00000019133	0.954173133	1.47E-06
TUCP_003806	XLOC_166117	ENSRNOT00000011956	0.959317611	8.20E-07
TUCP_003806	XLOC_166117	ENSRNOT00000078185	-0.952347116	1.79E-06
TUCP_003806	XLOC_166117	ENSRNOT00000024119	0.971668014	1.37E-07
TUCP_003806	XLOC_166117	ENSRNOT00000075800	-0.954019886	1.50E-06
TUCP_003806	XLOC_166117	ENSRNOT00000006824	-0.972672826	1.15E-07
TUCP_003806	XLOC_166117	ENSRNOT00000088509	0.966122534	3.32E-07
TUCP_003806	XLOC_166117	ENSRNOT00000086260	-0.9577163	9.91E-07
TUCP_003806	XLOC_166117	ENSRNOT00000009811	0.969438108	1.99E-07
TUCP_003806	XLOC_166117	ENSRNOT00000010683	-0.959534571	7.98E-07
TUCP_003806	XLOC_166117	ENSRNOT00000045488	0.981269075	1.76E-08
TUCP_003806	XLOC_166117	ENSRNOT00000090853	-0.953133013	1.65E-06
TUCP_003806	XLOC_166117	ENSRNOT00000020316	-0.954264645	1.46E-06
TUCP_003806	XLOC_166117	ENSRNOT00000020648	0.951332251	1.98E-06
TUCP_003806	XLOC_166117	ENSRNOT00000036666	0.964290743	4.31E-07
TUCP_003806	XLOC_166117	ENSRNOT00000089211	0.974736739	7.77E-08
TUCP_003806	XLOC_166117	ENSRNOT00000020383	0.988445239	1.59E-09
TUCP_003806	XLOC_166117	ENSRNOT00000015518	-0.965468452	3.65E-07
TUCP_003806	XLOC_166117	ENSRNOT00000000302	0.98338429	9.70E-09
TUCP_003806	XLOC_166117	ENSRNOT00000030706	-0.951797835	1.89E-06
TUCP_003806	XLOC_166117	ENSRNOT00000087502	0.96209585	5.78E-07
TUCP_003806	XLOC_166117	ENSRNOT00000041106	0.958641808	8.89E-07
TUCP_003806	XLOC_166117	ENSRNOT00000012424	0.960226961	7.33E-07
TUCP_003806	XLOC_166117	ENSRNOT00000000658	0.963975939	4.50E-07
TUCP_003806	XLOC_166117	ENSRNOT00000065229	0.950114453	2.24E-06
TUCP_003806	XLOC_166117	ENSRNOT00000000717	-0.966313793	3.23E-07
TUCP_003806	XLOC_166117	ENSRNOT00000005909	0.963130239	5.04E-07
TUCP_003806	XLOC_166117	ENSRNOT00000049048	-0.9556626	1.25E-06
TUCP_003806	XLOC_166117	ENSRNOT00000086122	0.977281664	4.59E-08
TUCP_003806	XLOC_166117	ENSRNOT00000007091	0.958370591	9.18E-07
TUCP_003806	XLOC_166117	ENSRNOT00000088628	0.956670977	1.12E-06
TUCP_003806	XLOC_166117	ENSRNOT00000023763	-0.963647515	4.70E-07
TUCP_003806	XLOC_166117	ENSRNOT00000003940	-0.959421505	8.09E-07
TUCP_003806	XLOC_166117	ENSRNOT00000042799	0.967962818	2.52E-07
TUCP_003806	XLOC_166117	ENSRNOT00000010993	0.961323584	6.39E-07
TUCP_003806	XLOC_166117	ENSRNOT00000059181	0.961107386	6.56E-07
TUCP_003806	XLOC_166117	ENSRNOT00000017291	-0.96400713	4.48E-07

TUCP_003806	XLOC_166117	ENSRNOT00000003691	-0.962870623	5.22E-07
TUCP_003806	XLOC_166117	ENSRNOT00000081678	0.96165351	6.12E-07
TUCP_003806	XLOC_166117	ENSRNOT00000008901	-0.950459546	2.16E-06
TUCP_003806	XLOC_166117	ENSRNOT00000081621	0.956838849	1.10E-06
TUCP_003806	XLOC_166117	ENSRNOT00000005659	0.971013841	1.53E-07
TUCP_003806	XLOC_166117	ENSRNOT00000081077	-0.963799052	4.61E-07
TUCP_003806	XLOC_166117	ENSRNOT00000017761	0.98768936	2.18E-09
TUCP_003806	XLOC_166117	ENSRNOT00000023543	-0.978391311	3.58E-08
TUCP_003806	XLOC_166117	ENSRNOT00000039341	0.966284153	3.24E-07
TUCP_003806	XLOC_166117	ENSRNOT00000085517	0.961798023	6.01E-07
TUCP_003806	XLOC_166117	ENSRNOT00000085458	-0.963723522	4.66E-07
TUCP_003806	XLOC_166117	ENSRNOT00000037405	0.965300725	3.74E-07
TUCP_003806	XLOC_166117	ENSRNOT00000014605	-0.950772931	2.10E-06
TUCP_003806	XLOC_166117	ENSRNOT00000085519	0.953740753	1.54E-06
TUCP_003806	XLOC_166117	ENSRNOT00000082174	0.957696111	9.94E-07
TUCP_003806	XLOC_166117	ENSRNOT00000042125	0.96493585	3.94E-07
TUCP_003806	XLOC_166117	ENSRNOT00000025460	0.967584254	2.67E-07
TUCP_003806	XLOC_166117	ENSRNOT00000087033	-0.970695243	1.62E-07
TUCP_003806	XLOC_166117	ENSRNOT00000010541	0.955847378	1.23E-06
TUCP_003806	XLOC_166117	ENSRNOT00000026760	-0.983327226	9.87E-09
TUCP_003806	XLOC_166117	ENSRNOT00000001322	-0.962381908	5.57E-07
TUCP_003806	XLOC_166117	ENSRNOT00000024491	-0.957382672	1.03E-06
TUCP_003806	XLOC_166117	ENSRNOT00000084597	-0.957862006	9.75E-07
TUCP_003806	XLOC_166117	ENSRNOT00000016568	0.957238377	1.05E-06
TUCP_003806	XLOC_166117	ENSRNOT00000037588	-0.956930179	1.09E-06
TUCP_003806	XLOC_166117	ENSRNOT00000067503	0.961273499	6.43E-07
TUCP_003806	XLOC_166117	ENSRNOT00000023388	-0.961356015	6.36E-07
TUCP_003806	XLOC_166117	ENSRNOT00000064930	0.96386843	4.56E-07
TUCP_003806	XLOC_166117	ENSRNOT00000027964	0.956845407	1.10E-06
TUCP_003806	XLOC_166117	ENSRNOT00000027967	0.95790154	9.70E-07
TUCP_003806	XLOC_166117	ENSRNOT00000024938	-0.957860093	9.75E-07
TUCP_003806	XLOC_166117	ENSRNOT00000081796	0.966064426	3.35E-07
TUCP_003806	XLOC_166117	ENSRNOT00000002053	-0.957155061	1.06E-06
TUCP_003806	XLOC_166117	ENSRNOT00000024933	-0.954400545	1.44E-06
TUCP_003806	XLOC_166117	ENSRNOT00000047210	0.982431772	1.28E-08
TUCP_003806	XLOC_166117	ENSRNOT00000083957	0.950152719	2.23E-06
TUCP_003806	XLOC_166117	ENSRNOT00000046639	0.969906115	1.85E-07
TUCP_003806	XLOC_166117	ENSRNOT00000070886	-0.951654332	1.92E-06
TUCP_003806	XLOC_166117	ENSRNOT00000079996	0.968926132	2.17E-07
TUCP_001087	XLOC_041374	ENSRNOT00000080945	0.966518498	3.13E-07
TUCP_001087	XLOC_041374	ENSRNOT00000016804	0.954390883	1.44E-06
TUCP_001086	XLOC_041371	ENSRNOT00000074810	0.966779959	3.01E-07
TUCP_001086	XLOC_041371	ENSRNOT00000077331	0.958676202	8.85E-07
TUCP_001086	XLOC_041371	ENSRNOT00000005505	0.959154454	8.36E-07
TUCP_001086	XLOC_041371	ENSRNOT00000000697	0.959281251	8.23E-07
TUCP_001086	XLOC_041371	ENSRNOT00000056558	0.956676961	1.12E-06
TUCP_001086	XLOC_041371	ENSRNOT00000023623	0.969816579	1.88E-07
TUCP_001086	XLOC_041371	ENSRNOT00000026757	0.950884814	2.07E-06
TUCP_001086	XLOC_041371	ENSRNOT00000009975	0.956362657	1.16E-06
TUCP_001086	XLOC_041371	ENSRNOT00000072682	0.951292994	1.99E-06
TUCP_001086	XLOC_041371	ENSRNOT00000075144	0.964373716	4.26E-07

TUCP_001086	XLOC_041371	ENSRNOT00000022585	0.951906011	1.87E-06
TUCP_001086	XLOC_041371	ENSRNOT00000041533	0.961324247	6.39E-07
TUCP_001086	XLOC_041371	ENSRNOT00000047763	0.951005367	2.05E-06
TUCP_001086	XLOC_041371	ENSRNOT00000023661	0.964290293	4.31E-07
TUCP_001086	XLOC_041371	ENSRNOT00000091367	0.950548812	2.14E-06
TUCP_001086	XLOC_041371	ENSRNOT00000054963	0.960229705	7.33E-07
TUCP_001086	XLOC_041371	ENSRNOT00000079882	0.963531254	4.78E-07
TUCP_001085	XLOC_041370	ENSRNOT00000004602	0.966364439	3.20E-07
TUCP_001085	XLOC_041370	ENSRNOT00000022585	0.963327761	4.91E-07
TUCP_002344	XLOC_097822	ENSRNOT00000077771	0.995375996	1.65E-11
TUCP_002344	XLOC_097822	ENSRNOT00000071462	-0.957776043	9.85E-07
TUCP_001081	XLOC_041191	ENSRNOT00000001215	0.95720611	1.05E-06
TUCP_001081	XLOC_041191	ENSRNOT00000006188	-0.962448913	5.52E-07
TUCP_001081	XLOC_041191	ENSRNOT00000006186	0.968955739	2.16E-07
TUCP_001081	XLOC_041191	ENSRNOT00000026941	0.970344649	1.72E-07
TUCP_001081	XLOC_041191	ENSRNOT00000075397	0.974058133	8.86E-08
TUCP_001081	XLOC_041191	ENSRNOT00000032926	-0.954679544	1.40E-06
TUCP_001081	XLOC_041191	ENSRNOT00000019104	-0.967827348	2.57E-07
TUCP_001081	XLOC_041191	ENSRNOT00000021348	0.96217115	5.73E-07
TUCP_001081	XLOC_041191	ENSRNOT00000073559	0.95993889	7.60E-07
TUCP_001081	XLOC_041191	ENSRNOT00000003891	0.950057759	2.25E-06
TUCP_001081	XLOC_041191	ENSRNOT00000068353	0.951628182	1.92E-06
TUCP_001081	XLOC_041191	ENSRNOT00000001691	0.957858264	9.75E-07
TUCP_001081	XLOC_041191	ENSRNOT00000020910	0.95742665	1.03E-06
TUCP_001081	XLOC_041191	ENSRNOT00000086706	0.964747498	4.04E-07
TUCP_001081	XLOC_041191	ENSRNOT00000004017	0.953511489	1.58E-06
TUCP_001081	XLOC_041191	ENSRNOT00000086310	-0.969635417	1.93E-07
TUCP_001081	XLOC_041191	ENSRNOT00000010030	-0.952098733	1.83E-06
TUCP_001081	XLOC_041191	ENSRNOT00000028856	0.952377508	1.78E-06
TUCP_001081	XLOC_041191	ENSRNOT00000000743	0.951776002	1.89E-06
TUCP_001081	XLOC_041191	ENSRNOT00000052063	0.97211377	1.27E-07
TUCP_001081	XLOC_041191	ENSRNOT00000030850	0.974056058	8.86E-08
TUCP_001081	XLOC_041191	ENSRNOT00000033017	0.973137547	1.05E-07
TUCP_001081	XLOC_041191	ENSRNOT00000055540	0.958254695	9.31E-07
TUCP_001081	XLOC_041191	ENSRNOT00000066370	0.952524444	1.75E-06
TUCP_001081	XLOC_041191	ENSRNOT00000006752	0.958163403	9.41E-07
TUCP_001081	XLOC_041191	ENSRNOT00000021534	-0.957132597	1.06E-06
TUCP_001081	XLOC_041191	ENSRNOT00000085251	-0.962220423	5.69E-07
TUCP_001081	XLOC_041191	ENSRNOT00000072771	0.960869303	6.77E-07
TUCP_001081	XLOC_041191	ENSRNOT00000073370	0.956082961	1.19E-06
TUCP_001081	XLOC_041191	ENSRNOT00000065300	0.975179435	7.12E-08
TUCP_001081	XLOC_041191	ENSRNOT00000044742	0.956059258	1.20E-06
TUCP_001081	XLOC_041191	ENSRNOT00000045699	0.959373499	8.14E-07
TUCP_001081	XLOC_041191	ENSRNOT00000029439	0.953403729	1.60E-06
TUCP_001081	XLOC_041191	ENSRNOT00000035320	-0.96303598	5.11E-07
TUCP_001081	XLOC_041191	ENSRNOT00000035989	0.955308326	1.30E-06
TUCP_001081	XLOC_041191	ENSRNOT00000057160	0.968375961	2.36E-07
TUCP_001081	XLOC_041191	ENSRNOT00000091448	-0.966440697	3.17E-07
TUCP_001081	XLOC_041191	ENSRNOT00000077999	0.973621732	9.62E-08
TUCP_001081	XLOC_041191	ENSRNOT00000068134	0.952376047	1.78E-06
TUCP_001081	XLOC_041191	ENSRNOT00000026398	-0.95456747	1.41E-06

TUCP_001081	XLOC_041191	ENSRNOT00000003146	-0.950537186	2.15E-06
TUCP_001081	XLOC_041191	ENSRNOT00000083214	0.953785444	1.54E-06
TUCP_001081	XLOC_041191	ENSRNOT00000085228	0.975749197	6.34E-08
TUCP_001081	XLOC_041191	ENSRNOT00000020134	0.953415438	1.60E-06
TUCP_001081	XLOC_041191	ENSRNOT00000017319	0.951230609	2.00E-06
TUCP_001081	XLOC_041191	ENSRNOT00000015188	0.962014525	5.84E-07
TUCP_001081	XLOC_041191	ENSRNOT00000079866	0.961119282	6.55E-07
TUCP_001081	XLOC_041191	ENSRNOT00000005686	-0.966487881	3.15E-07
TUCP_001081	XLOC_041191	ENSRNOT00000040629	0.951235664	2.00E-06
TUCP_001081	XLOC_041191	ENSRNOT00000004046	-0.969766244	1.89E-07
TUCP_001081	XLOC_041191	ENSRNOT00000015537	0.959122457	8.39E-07
TUCP_001081	XLOC_041191	ENSRNOT00000008655	0.963946686	4.52E-07
TUCP_001081	XLOC_041191	ENSRNOT00000087371	0.961130176	6.55E-07
TUCP_001081	XLOC_041191	ENSRNOT00000041044	0.955667801	1.25E-06
TUCP_001081	XLOC_041191	ENSRNOT00000067391	-0.950822675	2.09E-06
TUCP_001081	XLOC_041191	ENSRNOT00000092945	-0.959442764	8.07E-07
TUCP_001081	XLOC_041191	ENSRNOT00000023619	0.955828834	1.23E-06
TUCP_001081	XLOC_041191	ENSRNOT00000084709	0.976672413	5.23E-08
TUCP_001081	XLOC_041191	ENSRNOT00000068033	0.955467361	1.28E-06
TUCP_001081	XLOC_041191	ENSRNOT00000026481	0.979777518	2.57E-08
TUCP_001081	XLOC_041191	ENSRNOT00000026525	-0.952700413	1.72E-06
TUCP_001081	XLOC_041191	ENSRNOT00000025856	-0.962885003	5.21E-07
TUCP_001081	XLOC_041191	ENSRNOT00000028074	-0.951718385	1.90E-06
TUCP_001081	XLOC_041191	ENSRNOT00000026643	0.983236431	1.01E-08
TUCP_001081	XLOC_041191	ENSRNOT00000001403	0.954373497	1.44E-06
TUCP_001081	XLOC_041191	ENSRNOT00000072357	-0.960127797	7.42E-07
TUCP_001081	XLOC_041191	ENSRNOT00000016132	-0.976810915	5.08E-08
TUCP_001081	XLOC_041191	ENSRNOT00000058495	-0.959734216	7.79E-07
TUCP_001081	XLOC_041191	ENSRNOT00000013067	-0.973701966	9.48E-08
TUCP_001081	XLOC_041191	ENSRNOT00000016220	-0.952902992	1.69E-06
TUCP_001081	XLOC_041191	ENSRNOT00000047093	0.960680007	6.93E-07
TUCP_001081	XLOC_041191	ENSRNOT00000002313	-0.953461556	1.59E-06
TUCP_001081	XLOC_041191	ENSRNOT00000048063	0.957015804	1.08E-06
TUCP_001081	XLOC_041191	ENSRNOT00000031979	-0.950436188	2.17E-06
TUCP_001081	XLOC_041191	ENSRNOT00000033975	0.974377848	8.33E-08
TUCP_001081	XLOC_041191	ENSRNOT00000079895	0.96567179	3.54E-07
TUCP_001081	XLOC_041191	ENSRNOT00000078102	0.969559971	1.96E-07
TUCP_001081	XLOC_041191	ENSRNOT00000074586	-0.955659664	1.25E-06
TUCP_001081	XLOC_041191	ENSRNOT00000084031	0.966076655	3.34E-07
TUCP_001081	XLOC_041191	ENSRNOT00000081512	0.962248807	5.67E-07
TUCP_001081	XLOC_041191	ENSRNOT00000011880	0.973102264	1.06E-07
TUCP_001081	XLOC_041191	ENSRNOT00000029202	-0.974163775	8.68E-08
TUCP_001081	XLOC_041191	ENSRNOT00000023989	0.963602006	4.73E-07
TUCP_001081	XLOC_041191	ENSRNOT00000021717	0.955644599	1.25E-06
TUCP_001081	XLOC_041191	ENSRNOT00000003992	-0.957923105	9.68E-07
TUCP_001081	XLOC_041191	ENSRNOT00000021408	0.960974631	6.68E-07
TUCP_001081	XLOC_041191	ENSRNOT00000050515	0.970681425	1.62E-07
TUCP_001081	XLOC_041191	ENSRNOT00000084088	0.960680742	6.93E-07
TUCP_001081	XLOC_041191	ENSRNOT00000052224	0.978483502	3.50E-08
TUCP_001081	XLOC_041191	ENSRNOT00000078070	0.98873478	1.40E-09
TUCP_001081	XLOC_041191	ENSRNOT00000020951	0.959928744	7.61E-07

TUCP_001081	XLOC_041191	ENSRNOT00000046745	0.966869234	2.97E-07
TUCP_001081	XLOC_041191	ENSRNOT00000082146	-0.957788579	9.83E-07
TUCP_001081	XLOC_041191	ENSRNOT00000023876	0.963675776	4.69E-07
TUCP_001081	XLOC_041191	ENSRNOT00000091488	0.955755398	1.24E-06
TUCP_001081	XLOC_041191	ENSRNOT00000027452	0.957558005	1.01E-06
TUCP_001081	XLOC_041191	ENSRNOT00000049285	-0.955056558	1.34E-06
TUCP_001081	XLOC_041191	ENSRNOT00000003568	0.966062745	3.35E-07
TUCP_001081	XLOC_041191	ENSRNOT00000074649	0.965875168	3.44E-07
TUCP_001081	XLOC_041191	ENSRNOT00000077146	-0.974531069	8.09E-08
TUCP_001081	XLOC_041191	ENSRNOT00000014384	-0.956314165	1.16E-06
TUCP_001081	XLOC_041191	ENSRNOT00000060002	-0.964436594	4.22E-07
TUCP_001081	XLOC_041191	ENSRNOT00000036959	-0.958641336	8.89E-07
TUCP_001081	XLOC_041191	ENSRNOT00000010367	0.967356147	2.76E-07
TUCP_001081	XLOC_041191	ENSRNOT00000006395	-0.950294409	2.20E-06
TUCP_001081	XLOC_041191	ENSRNOT00000023977	-0.966267806	3.25E-07
TUCP_001081	XLOC_041191	ENSRNOT00000066133	-0.975353691	6.87E-08
TUCP_001081	XLOC_041191	ENSRNOT00000048933	-0.956816241	1.10E-06
TUCP_001081	XLOC_041191	ENSRNOT00000085596	0.954549706	1.41E-06
TUCP_001081	XLOC_041191	ENSRNOT00000026293	0.956987356	1.08E-06
TUCP_001081	XLOC_041191	ENSRNOT00000019487	0.968538552	2.30E-07
TUCP_001081	XLOC_041191	ENSRNOT00000030692	-0.953400916	1.60E-06
TUCP_001081	XLOC_041191	ENSRNOT00000080443	0.955047469	1.34E-06
TUCP_001081	XLOC_041191	ENSRNOT00000055992	-0.966829086	2.99E-07
TUCP_001081	XLOC_041191	ENSRNOT00000040069	0.960296317	7.27E-07
TUCP_001081	XLOC_041191	ENSRNOT00000015624	0.972092352	1.27E-07
TUCP_001081	XLOC_041191	ENSRNOT00000073235	0.954326309	1.45E-06
TUCP_001081	XLOC_041191	ENSRNOT00000040060	-0.955631256	1.26E-06
TUCP_001081	XLOC_041191	ENSRNOT00000035474	0.972761143	1.13E-07
TUCP_001081	XLOC_041191	ENSRNOT00000039102	0.954156234	1.48E-06
TUCP_001081	XLOC_041191	ENSRNOT00000078856	-0.965868379	3.44E-07
TUCP_001081	XLOC_041191	ENSRNOT00000091205	0.970232278	1.75E-07
TUCP_001081	XLOC_041191	ENSRNOT00000009742	-0.954815772	1.37E-06
TUCP_001081	XLOC_041191	ENSRNOT00000007369	0.956666135	1.12E-06
TUCP_001081	XLOC_041191	ENSRNOT00000004787	-0.968852927	2.19E-07
TUCP_001081	XLOC_041191	ENSRNOT00000060698	-0.96953046	1.97E-07
TUCP_001081	XLOC_041191	ENSRNOT00000064535	-0.959678833	7.84E-07
TUCP_001081	XLOC_041191	ENSRNOT00000014814	0.968318804	2.38E-07
TUCP_001081	XLOC_041191	ENSRNOT00000010748	0.982671635	1.20E-08
TUCP_001081	XLOC_041191	ENSRNOT00000080207	0.988427803	1.60E-09
TUCP_001081	XLOC_041191	ENSRNOT00000026174	0.951259917	2.00E-06
TUCP_001081	XLOC_041191	ENSRNOT00000065529	0.955337219	1.30E-06
TUCP_001081	XLOC_041191	ENSRNOT00000017776	0.972983765	1.08E-07
TUCP_001081	XLOC_041191	ENSRNOT00000026457	0.965271355	3.75E-07
TUCP_001081	XLOC_041191	ENSRNOT00000004463	0.976589506	5.32E-08
TUCP_001081	XLOC_041191	ENSRNOT00000059807	0.956698082	1.11E-06
TUCP_001081	XLOC_041191	ENSRNOT00000008982	0.977271136	4.60E-08
TUCP_001081	XLOC_041191	ENSRNOT00000090057	0.952202505	1.81E-06
TUCP_001081	XLOC_041191	ENSRNOT00000022097	0.967466849	2.72E-07
TUCP_001081	XLOC_041191	ENSRNOT00000008987	-0.97540425	6.80E-08
TUCP_001081	XLOC_041191	ENSRNOT00000092876	-0.969306027	2.04E-07
TUCP_001081	XLOC_041191	ENSRNOT00000083799	0.95319785	1.63E-06

TUCP_001081	XLOC_041191	ENSRNOT00000013864	-0.951057574	2.04E-06
TUCP_001081	XLOC_041191	ENSRNOT00000065143	0.951959283	1.86E-06
TUCP_001081	XLOC_041191	ENSRNOT00000027083	0.97198023	1.30E-07
TUCP_001081	XLOC_041191	ENSRNOT00000066041	-0.965559278	3.60E-07
TUCP_001081	XLOC_041191	ENSRNOT00000063930	-0.963216201	4.99E-07
TUCP_001081	XLOC_041191	ENSRNOT00000006055	0.958588369	8.95E-07
TUCP_001081	XLOC_041191	ENSRNOT00000013116	-0.960185658	7.37E-07
TUCP_001081	XLOC_041191	ENSRNOT00000001290	0.9510935	2.03E-06
TUCP_001081	XLOC_041191	ENSRNOT00000001855	0.964827022	4.00E-07
TUCP_001081	XLOC_041191	ENSRNOT00000009563	-0.96328098	4.94E-07
TUCP_001081	XLOC_041191	ENSRNOT00000088125	0.953487565	1.59E-06
TUCP_001081	XLOC_041191	ENSRNOT00000019502	0.956079095	1.20E-06
TUCP_001081	XLOC_041191	ENSRNOT00000055683	0.950136156	2.23E-06
TUCP_001081	XLOC_041191	ENSRNOT00000087838	-0.957149942	1.06E-06
TUCP_001081	XLOC_041191	ENSRNOT00000081981	0.97705185	4.82E-08
TUCP_001081	XLOC_041191	ENSRNOT00000014479	-0.95759501	1.01E-06
TUCP_001081	XLOC_041191	ENSRNOT00000003326	-0.958037768	9.55E-07
TUCP_001081	XLOC_041191	ENSRNOT00000021629	-0.951044719	2.04E-06
TUCP_001081	XLOC_041191	ENSRNOT00000016306	-0.950679657	2.12E-06
TUCP_001081	XLOC_041191	ENSRNOT00000015476	-0.95626598	1.17E-06
TUCP_001081	XLOC_041191	ENSRNOT00000025079	-0.977673807	4.21E-08
TUCP_001081	XLOC_041191	ENSRNOT00000048754	-0.973635358	9.60E-08
TUCP_001081	XLOC_041191	ENSRNOT00000044628	0.968319799	2.38E-07
TUCP_001081	XLOC_041191	ENSRNOT00000025075	0.967687921	2.63E-07
TUCP_001081	XLOC_041191	ENSRNOT00000004007	-0.962663246	5.37E-07
TUCP_001081	XLOC_041191	ENSRNOT00000020681	-0.952007967	1.85E-06
TUCP_001081	XLOC_041191	ENSRNOT00000057898	-0.965350982	3.71E-07
TUCP_001081	XLOC_041191	ENSRNOT00000091602	-0.959858409	7.67E-07
TUCP_001081	XLOC_041191	ENSRNOT00000056100	-0.95182005	1.89E-06
TUCP_001081	XLOC_041191	ENSRNOT00000012671	0.950408133	2.17E-06
TUCP_001081	XLOC_041191	ENSRNOT00000090129	-0.953419633	1.60E-06
TUCP_001081	XLOC_041191	ENSRNOT00000009708	-0.953505209	1.58E-06
TUCP_001081	XLOC_041191	ENSRNOT00000077231	0.981508463	1.65E-08
TUCP_001081	XLOC_041191	ENSRNOT00000060696	0.974749875	7.75E-08
TUCP_001081	XLOC_041191	ENSRNOT00000009811	0.989737884	8.81E-10
TUCP_001081	XLOC_041191	ENSRNOT00000014605	-0.952519502	1.75E-06
TUCP_001081	XLOC_041191	ENSRNOT00000073643	-0.959523466	7.99E-07
TUCP_001081	XLOC_041191	ENSRNOT00000052002	0.951815029	1.89E-06
TUCP_001081	XLOC_041191	ENSRNOT00000022502	-0.967967654	2.52E-07
TUCP_001081	XLOC_041191	ENSRNOT00000017556	0.966226678	3.27E-07
TUCP_001081	XLOC_041191	ENSRNOT00000087443	0.971013634	1.54E-07
TUCP_001081	XLOC_041191	ENSRNOT00000021803	-0.969764232	1.89E-07
TUCP_001081	XLOC_041191	ENSRNOT00000024352	0.971751852	1.35E-07
TUCP_001081	XLOC_041191	ENSRNOT00000058351	0.972102572	1.27E-07
TUCP_001081	XLOC_041191	ENSRNOT00000083220	0.982667709	1.20E-08
TUCP_001081	XLOC_041191	ENSRNOT00000072118	0.958888861	8.63E-07
TUCP_001081	XLOC_041191	ENSRNOT00000003780	0.973265549	1.03E-07
TUCP_001081	XLOC_041191	ENSRNOT00000003787	-0.955774221	1.24E-06
TUCP_001081	XLOC_041191	ENSRNOT00000060362	0.963796757	4.61E-07
TUCP_001081	XLOC_041191	ENSRNOT00000013274	0.954455942	1.43E-06
TUCP_001081	XLOC_041191	ENSRNOT00000083876	-0.956892619	1.09E-06

TUCP_001081	XLOC_041191	ENSRNOT00000019816	-0.954291363	1.46E-06
TUCP_001081	XLOC_041191	ENSRNOT00000017254	-0.953058856	1.66E-06
TUCP_001081	XLOC_041191	ENSRNOT00000068381	0.961793858	6.01E-07
TUCP_001081	XLOC_041191	ENSRNOT00000068026	-0.953450232	1.59E-06
TUCP_001081	XLOC_041191	ENSRNOT00000085621	0.962949601	5.17E-07
TUCP_001081	XLOC_041191	ENSRNOT00000007314	-0.964522729	4.17E-07
TUCP_001081	XLOC_041191	ENSRNOT00000014821	-0.958669218	8.86E-07
TUCP_001081	XLOC_041191	ENSRNOT00000058382	0.956759461	1.11E-06
TUCP_001081	XLOC_041191	ENSRNOT00000088599	0.972761498	1.13E-07
TUCP_001081	XLOC_041191	ENSRNOT00000026531	-0.961451203	6.28E-07
TUCP_001081	XLOC_041191	ENSRNOT00000023567	-0.956163981	1.18E-06
TUCP_001081	XLOC_041191	ENSRNOT00000061100	0.967015198	2.91E-07
TUCP_001081	XLOC_041191	ENSRNOT00000017021	0.952949177	1.68E-06
TUCP_001081	XLOC_041191	ENSRNOT00000017995	0.959301271	8.21E-07
TUCP_001081	XLOC_041191	ENSRNOT00000050609	-0.952615482	1.74E-06
TUCP_001081	XLOC_041191	ENSRNOT00000081725	0.956304954	1.17E-06
TUCP_001081	XLOC_041191	ENSRNOT00000005459	0.966233571	3.27E-07
TUCP_001081	XLOC_041191	ENSRNOT00000027910	0.979021524	3.09E-08
TUCP_001081	XLOC_041191	ENSRNOT00000002771	0.957654151	9.99E-07
TUCP_001081	XLOC_041191	ENSRNOT00000024322	0.956178913	1.18E-06
TUCP_001081	XLOC_041191	ENSRNOT00000088182	0.955642396	1.26E-06
TUCP_001081	XLOC_041191	ENSRNOT00000054976	-0.950504227	2.15E-06
TUCP_001081	XLOC_041191	ENSRNOT00000016191	0.969460431	1.99E-07
TUCP_001081	XLOC_041191	ENSRNOT00000078390	-0.972257059	1.24E-07
TUCP_001081	XLOC_041191	ENSRNOT00000008427	-0.952990916	1.67E-06
TUCP_001081	XLOC_041191	ENSRNOT00000016372	-0.952685711	1.72E-06
TUCP_001081	XLOC_041191	ENSRNOT00000029833	0.960468663	7.11E-07
TUCP_001081	XLOC_041191	ENSRNOT00000012702	-0.957282135	1.04E-06
TUCP_001081	XLOC_041191	ENSRNOT00000082151	-0.971281966	1.47E-07
TUCP_001081	XLOC_041191	ENSRNOT00000016776	-0.95702998	1.07E-06
TUCP_001081	XLOC_041191	ENSRNOT00000001607	0.954645798	1.40E-06
TUCP_001081	XLOC_041191	ENSRNOT00000047192	-0.977074623	4.80E-08
TUCP_001081	XLOC_041191	ENSRNOT00000011984	0.9562935	1.17E-06
TUCP_001081	XLOC_041191	ENSRNOT00000085971	-0.967847356	2.56E-07
TUCP_001081	XLOC_041191	ENSRNOT00000044395	0.951497873	1.95E-06
TUCP_001081	XLOC_041191	ENSRNOT00000022142	0.961407735	6.32E-07
TUCP_001081	XLOC_041191	ENSRNOT00000076300	-0.95120628	2.01E-06
TUCP_001081	XLOC_041191	ENSRNOT00000028876	-0.962478622	5.50E-07
TUCP_001081	XLOC_041191	ENSRNOT00000078306	0.961847085	5.97E-07
TUCP_001081	XLOC_041191	ENSRNOT00000015946	-0.953256593	1.62E-06
TUCP_001081	XLOC_041191	ENSRNOT00000004172	0.959250738	8.26E-07
TUCP_001081	XLOC_041191	ENSRNOT00000029125	0.975559821	6.59E-08
TUCP_001081	XLOC_041191	ENSRNOT00000004319	-0.956301758	1.17E-06
TUCP_001081	XLOC_041191	ENSRNOT00000026280	0.953817814	1.53E-06
TUCP_001081	XLOC_041191	ENSRNOT00000005387	0.968343566	2.37E-07
TUCP_001081	XLOC_041191	ENSRNOT00000015092	0.963467204	4.82E-07
TUCP_001081	XLOC_041191	ENSRNOT00000015318	-0.950145564	2.23E-06
TUCP_001081	XLOC_041191	ENSRNOT00000028907	0.950249021	2.21E-06
TUCP_001081	XLOC_041191	ENSRNOT00000087278	-0.959996545	7.54E-07
TUCP_001081	XLOC_041191	ENSRNOT00000022796	0.959109948	8.40E-07
TUCP_001081	XLOC_041191	ENSRNOT00000078862	0.965292648	3.74E-07

TUCP_001081	XLOC_041191	ENSRNOT00000003940	-0.959894032	7.64E-07
TUCP_001081	XLOC_041191	ENSRNOT00000005257	0.962314208	5.62E-07
TUCP_001081	XLOC_041191	ENSRNOT00000015799	0.988876628	1.32E-09
TUCP_001081	XLOC_041191	ENSRNOT00000036506	0.951752506	1.90E-06
TUCP_001081	XLOC_041191	ENSRNOT00000005958	0.973382189	1.01E-07
TUCP_001081	XLOC_041191	ENSRNOT00000039551	-0.97076822	1.60E-07
TUCP_001081	XLOC_041191	ENSRNOT00000082998	0.966130406	3.32E-07
TUCP_001081	XLOC_041191	ENSRNOT00000075656	-0.950903783	2.07E-06
TUCP_001081	XLOC_041191	ENSRNOT00000015006	0.955681709	1.25E-06
TUCP_001081	XLOC_041191	ENSRNOT00000007350	0.950814622	2.09E-06
TUCP_001081	XLOC_041191	ENSRNOT00000007351	-0.95202682	1.85E-06
TUCP_001081	XLOC_041191	ENSRNOT00000083578	0.956948463	1.08E-06
TUCP_001081	XLOC_041191	ENSRNOT00000023133	0.950346461	2.19E-06
TUCP_001081	XLOC_041191	ENSRNOT00000023638	0.981724396	1.56E-08
TUCP_001081	XLOC_041191	ENSRNOT00000005228	0.964010209	4.48E-07
TUCP_001081	XLOC_041191	ENSRNOT00000059524	-0.960878676	6.76E-07
TUCP_001081	XLOC_041191	ENSRNOT00000084597	-0.953543101	1.58E-06
TUCP_001081	XLOC_041191	ENSRNOT00000028628	-0.951784571	1.89E-06
TUCP_001081	XLOC_041191	ENSRNOT00000017361	0.953087673	1.65E-06
TUCP_001081	XLOC_041191	ENSRNOT00000005220	0.974812718	7.65E-08
TUCP_001081	XLOC_041191	ENSRNOT00000023144	0.983682702	8.86E-09
TUCP_001081	XLOC_041191	ENSRNOT00000045326	0.966001848	3.38E-07
TUCP_001081	XLOC_041191	ENSRNOT00000016649	0.978304366	3.65E-08
TUCP_001081	XLOC_041191	ENSRNOT00000074023	0.973651196	9.57E-08
TUCP_001081	XLOC_041191	ENSRNOT00000067170	-0.955538206	1.27E-06
TUCP_001081	XLOC_041191	ENSRNOT00000091128	-0.970113163	1.79E-07
TUCP_001081	XLOC_041191	ENSRNOT00000001586	-0.969097217	2.11E-07
TUCP_001081	XLOC_041191	ENSRNOT00000018341	-0.950725942	2.11E-06
TUCP_001081	XLOC_041191	ENSRNOT00000026543	0.96219044	5.71E-07
TUCP_001081	XLOC_041191	ENSRNOT00000065076	0.951513612	1.95E-06
TUCP_001081	XLOC_041191	ENSRNOT00000019213	0.965347245	3.71E-07
TUCP_001081	XLOC_041191	ENSRNOT00000012187	0.976793445	5.10E-08
TUCP_001081	XLOC_041191	ENSRNOT00000024969	0.97510417	7.22E-08
TUCP_001081	XLOC_041191	ENSRNOT00000050868	0.95872608	8.80E-07
TUCP_001081	XLOC_041191	ENSRNOT00000013695	-0.95611363	1.19E-06
TUCP_001081	XLOC_041191	ENSRNOT00000081621	0.951461349	1.96E-06
TUCP_001081	XLOC_041191	ENSRNOT00000038247	0.972987352	1.08E-07
TUCP_001081	XLOC_041191	ENSRNOT00000090338	-0.96199725	5.86E-07
TUCP_001081	XLOC_041191	ENSRNOT00000024402	0.978175557	3.76E-08
TUCP_001081	XLOC_041191	ENSRNOT00000089545	-0.952669659	1.73E-06
TUCP_001081	XLOC_041191	ENSRNOT00000089933	0.988172602	1.79E-09
TUCP_001081	XLOC_041191	ENSRNOT00000077908	0.957920699	9.68E-07
TUCP_001081	XLOC_041191	ENSRNOT00000067009	-0.960745526	6.87E-07
TUCP_001081	XLOC_041191	ENSRNOT00000090912	0.960074951	7.47E-07
TUCP_001081	XLOC_041191	ENSRNOT00000067554	0.957696702	9.94E-07
TUCP_001081	XLOC_041191	ENSRNOT00000026718	0.965265991	3.76E-07
TUCP_001081	XLOC_041191	ENSRNOT00000072208	0.954153207	1.48E-06
TUCP_001081	XLOC_041191	ENSRNOT00000032780	0.974337611	8.40E-08
TUCP_001081	XLOC_041191	ENSRNOT00000084012	0.97078979	1.59E-07
TUCP_001081	XLOC_041191	ENSRNOT00000033677	-0.972723034	1.14E-07
TUCP_001081	XLOC_041191	ENSRNOT00000019820	0.974851551	7.59E-08

TUCP_001081	XLOC_041191	ENSRNOT00000002410	0.963641039	4.71E-07
TUCP_001081	XLOC_041191	ENSRNOT00000011832	-0.958920961	8.60E-07
TUCP_001081	XLOC_041191	ENSRNOT00000016428	0.954382303	1.44E-06
TUCP_001081	XLOC_041191	ENSRNOT00000021030	-0.954396356	1.44E-06
TUCP_001081	XLOC_041191	ENSRNOT00000006835	0.958830139	8.69E-07
TUCP_001081	XLOC_041191	ENSRNOT00000077727	-0.965537768	3.61E-07
TUCP_001081	XLOC_041191	ENSRNOT00000044346	-0.963344993	4.90E-07
TUCP_001081	XLOC_041191	ENSRNOT00000010541	0.983064878	1.07E-08
TUCP_001081	XLOC_041191	ENSRNOT00000017949	-0.951710851	1.91E-06
TUCP_001081	XLOC_041191	ENSRNOT00000016947	0.951519618	1.94E-06
TUCP_001081	XLOC_041191	ENSRNOT00000091909	0.978236837	3.71E-08
TUCP_001081	XLOC_041191	ENSRNOT00000001081	0.96945506	1.99E-07
TUCP_001081	XLOC_041191	ENSRNOT00000013025	-0.95964351	7.88E-07
TUCP_001081	XLOC_041191	ENSRNOT00000003544	-0.961722787	6.07E-07
TUCP_001081	XLOC_041191	ENSRNOT00000086119	-0.979493118	2.76E-08
TUCP_001081	XLOC_041191	ENSRNOT00000022190	-0.979136138	3.01E-08
TUCP_001081	XLOC_041191	ENSRNOT00000025913	-0.972643142	1.15E-07
TUCP_001081	XLOC_041191	ENSRNOT00000019386	-0.96115484	6.53E-07
TUCP_001081	XLOC_041191	ENSRNOT00000077220	-0.964165965	4.38E-07
TUCP_001081	XLOC_041191	ENSRNOT00000065983	0.963973034	4.50E-07
TUCP_001081	XLOC_041191	ENSRNOT00000052018	0.957081246	1.07E-06
TUCP_001081	XLOC_041191	ENSRNOT00000067780	-0.966193375	3.29E-07
TUCP_001081	XLOC_041191	ENSRNOT00000086577	0.965895811	3.43E-07
TUCP_001081	XLOC_041191	ENSRNOT00000025966	0.96417905	4.37E-07
TUCP_001081	XLOC_041191	ENSRNOT00000015265	0.95723423	1.05E-06
TUCP_001081	XLOC_041191	ENSRNOT00000073910	-0.951855036	1.88E-06
TUCP_001081	XLOC_041191	ENSRNOT00000007738	-0.957113904	1.06E-06
TUCP_001081	XLOC_041191	ENSRNOT00000052422	0.967407613	2.74E-07
TUCP_001081	XLOC_041191	ENSRNOT00000015609	0.958370032	9.18E-07
TUCP_001081	XLOC_041191	ENSRNOT00000086590	0.953123005	1.65E-06
TUCP_001081	XLOC_041191	ENSRNOT00000034883	-0.970120463	1.78E-07
TUCP_001081	XLOC_041191	ENSRNOT00000020155	0.968483037	2.32E-07
TUCP_001081	XLOC_041191	ENSRNOT00000025827	0.950946703	2.06E-06
TUCP_001081	XLOC_041191	ENSRNOT00000055321	0.953334861	1.61E-06
TUCP_001081	XLOC_041191	ENSRNOT00000005910	-0.965762754	3.50E-07
TUCP_001081	XLOC_041191	ENSRNOT00000075311	0.954582655	1.41E-06
TUCP_001081	XLOC_041191	ENSRNOT00000078475	0.971531226	1.40E-07
TUCP_001081	XLOC_041191	ENSRNOT00000087429	0.978810755	3.25E-08
TUCP_001081	XLOC_041191	ENSRNOT00000016722	0.959929752	7.61E-07
TUCP_001081	XLOC_041191	ENSRNOT00000091265	0.954066183	1.49E-06
TUCP_001081	XLOC_041191	ENSRNOT00000065501	0.964794162	4.01E-07
TUCP_001081	XLOC_041191	ENSRNOT00000000013	0.964121619	4.41E-07
TUCP_001081	XLOC_041191	ENSRNOT00000037955	-0.951619825	1.92E-06
TUCP_001081	XLOC_041191	ENSRNOT00000031904	0.969720903	1.91E-07
TUCP_001081	XLOC_041191	ENSRNOT00000028057	0.962222343	5.69E-07
TUCP_001081	XLOC_041191	ENSRNOT00000055582	0.956225225	1.18E-06
TUCP_001081	XLOC_041191	ENSRNOT00000022074	0.972702456	1.14E-07
TUCP_001081	XLOC_041191	ENSRNOT00000004999	0.963651927	4.70E-07
TUCP_001081	XLOC_041191	ENSRNOT00000009122	0.953596963	1.57E-06
TUCP_001081	XLOC_041191	ENSRNOT00000013463	0.961630304	6.14E-07
TUCP_001081	XLOC_041191	ENSRNOT00000088054	0.968414152	2.35E-07

TUCP_001081	XLOC_041191	ENSRNOT00000047663	-0.959419771	8.10E-07
TUCP_001081	XLOC_041191	ENSRNOT00000005486	-0.962406838	5.55E-07
TUCP_001081	XLOC_041191	ENSRNOT00000017907	-0.961971264	5.88E-07
TUCP_001081	XLOC_041191	ENSRNOT00000076669	0.950304792	2.20E-06
TUCP_001081	XLOC_041191	ENSRNOT00000005489	0.964792951	4.02E-07
TUCP_001081	XLOC_041191	ENSRNOT00000025794	-0.96626569	3.25E-07
TUCP_001081	XLOC_041191	ENSRNOT00000058111	-0.955123122	1.33E-06
TUCP_001081	XLOC_041191	ENSRNOT00000084422	0.965600869	3.58E-07
TUCP_001081	XLOC_041191	ENSRNOT00000050834	-0.967234162	2.82E-07
TUCP_001081	XLOC_041191	ENSRNOT00000054828	0.963670763	4.69E-07
TUCP_001081	XLOC_041191	ENSRNOT00000088279	0.954335904	1.45E-06
TUCP_001081	XLOC_041191	ENSRNOT00000002443	0.974118238	8.76E-08
TUCP_001081	XLOC_041191	ENSRNOT00000012736	0.963423791	4.85E-07
TUCP_001081	XLOC_041191	ENSRNOT00000002114	-0.972116172	1.27E-07
TUCP_001081	XLOC_041191	ENSRNOT00000002445	-0.950095463	2.24E-06
TUCP_001081	XLOC_041191	ENSRNOT00000027970	0.982161721	1.38E-08
TUCP_001081	XLOC_041191	ENSRNOT00000009462	0.955777628	1.24E-06
TUCP_001081	XLOC_041191	ENSRNOT00000046215	-0.963086557	5.07E-07
TUCP_001081	XLOC_041191	ENSRNOT00000000083	-0.955107782	1.33E-06
TUCP_001081	XLOC_041191	ENSRNOT00000018074	-0.972970372	1.09E-07
TUCP_001081	XLOC_041191	ENSRNOT00000037679	0.966835462	2.99E-07
TUCP_001081	XLOC_041191	ENSRNOT00000014124	0.977047937	4.83E-08
TUCP_001081	XLOC_041191	ENSRNOT00000000120	0.980719657	2.03E-08
TUCP_001081	XLOC_041191	ENSRNOT00000013632	0.969702673	1.91E-07
TUCP_001081	XLOC_041191	ENSRNOT00000078906	-0.957457994	1.02E-06
TUCP_001081	XLOC_041191	ENSRNOT00000028545	-0.952307555	1.79E-06
TUCP_001081	XLOC_041191	ENSRNOT00000009900	-0.973379446	1.01E-07
TUCP_001081	XLOC_041191	ENSRNOT00000019172	-0.962635479	5.39E-07
TUCP_001081	XLOC_041191	ENSRNOT00000064798	0.964175905	4.38E-07
TUCP_001081	XLOC_041191	ENSRNOT00000015495	-0.952589302	1.74E-06
TUCP_001081	XLOC_041191	ENSRNOT00000032248	0.9550816	1.34E-06
TUCP_001081	XLOC_041191	ENSRNOT00000040393	0.978268041	3.68E-08
TUCP_001081	XLOC_041191	ENSRNOT00000025097	0.959905401	7.63E-07
TUCP_001081	XLOC_041191	ENSRNOT00000067901	-0.969459295	1.99E-07
TUCP_001081	XLOC_041191	ENSRNOT00000011041	0.964190405	4.37E-07
TUCP_001081	XLOC_041191	ENSRNOT00000021674	0.968774419	2.22E-07
TUCP_001081	XLOC_041191	ENSRNOT00000064700	0.965598169	3.58E-07
TUCP_001081	XLOC_041191	ENSRNOT00000036613	-0.981283122	1.75E-08
TUCP_001081	XLOC_041191	ENSRNOT00000083127	0.966017685	3.37E-07
TUCP_001081	XLOC_041191	ENSRNOT00000025705	0.966054232	3.35E-07
TUCP_001081	XLOC_041191	ENSRNOT00000019983	-0.960649156	6.96E-07
TUCP_001081	XLOC_041191	ENSRNOT00000004140	0.959030754	8.49E-07
TUCP_001081	XLOC_041191	ENSRNOT00000008113	-0.960733922	6.88E-07
TUCP_001081	XLOC_041191	ENSRNOT00000022552	-0.965227901	3.78E-07
TUCP_001081	XLOC_041191	ENSRNOT00000048910	-0.970461329	1.69E-07
TUCP_001081	XLOC_041191	ENSRNOT00000009645	-0.950480632	2.16E-06
TUCP_001081	XLOC_041191	ENSRNOT00000026279	-0.95438389	1.44E-06
TUCP_001081	XLOC_041191	ENSRNOT00000071560	0.95700121	1.08E-06
TUCP_001081	XLOC_041191	ENSRNOT00000082508	-0.955824533	1.23E-06
TUCP_001081	XLOC_041191	ENSRNOT00000082863	0.976924641	4.96E-08
TUCP_001081	XLOC_041191	ENSRNOT00000007164	0.952046284	1.84E-06

TUCP_001081	XLOC_041191	ENSRNOT00000049459	-0.961561539	6.20E-07
TUCP_001081	XLOC_041191	ENSRNOT00000071166	0.963374187	4.88E-07
TUCP_001081	XLOC_041191	ENSRNOT00000074389	-0.952744423	1.71E-06
TUCP_001081	XLOC_041191	ENSRNOT00000028646	0.958770837	8.75E-07
TUCP_001081	XLOC_041191	ENSRNOT00000055437	-0.967071321	2.88E-07
TUCP_001081	XLOC_041191	ENSRNOT00000066204	-0.956431281	1.15E-06
TUCP_001081	XLOC_041191	ENSRNOT00000043345	0.950498009	2.15E-06
TUCP_001081	XLOC_041191	ENSRNOT00000076872	0.959639927	7.88E-07
TUCP_001081	XLOC_041191	ENSRNOT00000073564	-0.951148181	2.02E-06
TUCP_001081	XLOC_041191	ENSRNOT00000001507	0.965484335	3.64E-07
TUCP_001081	XLOC_041191	ENSRNOT00000083568	0.968273312	2.40E-07
TUCP_001081	XLOC_041191	ENSRNOT00000039177	-0.959319611	8.19E-07
TUCP_001081	XLOC_041191	ENSRNOT00000081047	0.985717974	4.57E-09
TUCP_001081	XLOC_041191	ENSRNOT00000020994	-0.954448986	1.43E-06
TUCP_001081	XLOC_041191	ENSRNOT00000037456	0.961180491	6.50E-07
TUCP_001081	XLOC_041191	ENSRNOT00000025517	0.954309164	1.45E-06
TUCP_001081	XLOC_041191	ENSRNOT00000065168	0.964797335	4.01E-07
TUCP_001081	XLOC_041191	ENSRNOT00000012881	-0.96351209	4.79E-07
TUCP_001081	XLOC_041191	ENSRNOT00000000577	0.970733279	1.61E-07
TUCP_001081	XLOC_041191	ENSRNOT00000075424	0.955942809	1.21E-06
TUCP_001081	XLOC_041191	ENSRNOT00000041513	0.960394404	7.18E-07
TUCP_001081	XLOC_041191	ENSRNOT00000088133	-0.96727803	2.80E-07
TUCP_001081	XLOC_041191	ENSRNOT00000080677	-0.96616312	3.30E-07
TUCP_001081	XLOC_041191	ENSRNOT00000037039	-0.957558428	1.01E-06
TUCP_001081	XLOC_041191	ENSRNOT00000007824	-0.959098953	8.42E-07
TUCP_001081	XLOC_041191	ENSRNOT00000003714	0.953480921	1.59E-06
TUCP_001081	XLOC_041191	ENSRNOT00000083968	-0.957222118	1.05E-06
TUCP_001081	XLOC_041191	ENSRNOT00000037133	0.979188526	2.97E-08
TUCP_001081	XLOC_041191	ENSRNOT00000083964	-0.957640213	1.00E-06
TUCP_001081	XLOC_041191	ENSRNOT00000026831	0.957074958	1.07E-06
TUCP_001081	XLOC_041191	ENSRNOT00000021729	0.951475283	1.95E-06
TUCP_001081	XLOC_041191	ENSRNOT00000032363	0.95534838	1.30E-06
TUCP_001081	XLOC_041191	ENSRNOT00000016445	0.972426744	1.20E-07
TUCP_001081	XLOC_041191	ENSRNOT00000019133	0.983672939	8.89E-09
TUCP_001081	XLOC_041191	ENSRNOT00000024119	0.958570045	8.97E-07
TUCP_001081	XLOC_041191	ENSRNOT00000006824	-0.966679346	3.06E-07
TUCP_001081	XLOC_041191	ENSRNOT00000012775	-0.951195602	2.01E-06
TUCP_001081	XLOC_041191	ENSRNOT00000086260	-0.959427129	8.09E-07
TUCP_001081	XLOC_041191	ENSRNOT00000061392	0.979786676	2.57E-08
TUCP_001081	XLOC_041191	ENSRNOT00000044608	-0.96004555	7.50E-07
TUCP_001081	XLOC_041191	ENSRNOT00000090123	0.975907821	6.14E-08
TUCP_001081	XLOC_041191	ENSRNOT00000010683	-0.956470585	1.14E-06
TUCP_001081	XLOC_041191	ENSRNOT00000045488	0.961350033	6.37E-07
TUCP_001081	XLOC_041191	ENSRNOT00000090853	-0.9578126	9.80E-07
TUCP_001081	XLOC_041191	ENSRNOT00000093174	0.951050037	2.04E-06
TUCP_001081	XLOC_041191	ENSRNOT00000077555	0.961181365	6.50E-07
TUCP_001081	XLOC_041191	ENSRNOT00000089211	0.960433762	7.15E-07
TUCP_001081	XLOC_041191	ENSRNOT00000020383	0.967161994	2.85E-07
TUCP_001081	XLOC_041191	ENSRNOT00000000302	0.973514845	9.82E-08
TUCP_001081	XLOC_041191	ENSRNOT00000055602	-0.95724996	1.05E-06
TUCP_001081	XLOC_041191	ENSRNOT00000004290	0.955982917	1.21E-06

TUCP_001081	XLOC_041191	ENSRNOT00000000658	0.963621725	4.72E-07
TUCP_001081	XLOC_041191	ENSRNOT00000083632	-0.958220803	9.34E-07
TUCP_001081	XLOC_041191	ENSRNOT00000000717	-0.952476057	1.76E-06
TUCP_001081	XLOC_041191	ENSRNOT00000005909	0.957696744	9.94E-07
TUCP_001081	XLOC_041191	ENSRNOT00000086122	0.969392316	2.01E-07
TUCP_001081	XLOC_041191	ENSRNOT00000026235	-0.951121927	2.02E-06
TUCP_001081	XLOC_041191	ENSRNOT00000023763	-0.95555504	1.27E-06
TUCP_001081	XLOC_041191	ENSRNOT00000091596	-0.956550175	1.13E-06
TUCP_001081	XLOC_041191	ENSRNOT00000058842	0.956080261	1.20E-06
TUCP_001081	XLOC_041191	ENSRNOT00000023537	-0.96714469	2.85E-07
TUCP_001081	XLOC_041191	ENSRNOT00000059181	0.957791955	9.83E-07
TUCP_001081	XLOC_041191	ENSRNOT00000003691	-0.96623286	3.27E-07
TUCP_001081	XLOC_041191	ENSRNOT00000003690	0.956167841	1.18E-06
TUCP_001081	XLOC_041191	ENSRNOT00000038807	0.958758268	8.77E-07
TUCP_001081	XLOC_041191	ENSRNOT00000008901	-0.970596509	1.65E-07
TUCP_001081	XLOC_041191	ENSRNOT00000005659	0.96059143	7.01E-07
TUCP_001081	XLOC_041191	ENSRNOT00000017761	0.975849512	6.21E-08
TUCP_001081	XLOC_041191	ENSRNOT00000023543	-0.958967514	8.55E-07
TUCP_001081	XLOC_041191	ENSRNOT00000080608	0.971499439	1.41E-07
TUCP_001081	XLOC_041191	ENSRNOT00000080695	0.973253754	1.03E-07
TUCP_001081	XLOC_041191	ENSRNOT00000039341	0.962944993	5.17E-07
TUCP_001081	XLOC_041191	ENSRNOT00000055386	0.965115792	3.84E-07
TUCP_001081	XLOC_041191	ENSRNOT00000038212	-0.959274006	8.24E-07
TUCP_001081	XLOC_041191	ENSRNOT00000082174	0.953104615	1.65E-06
TUCP_001081	XLOC_041191	ENSRNOT00000042125	0.992972157	1.33E-10
TUCP_001081	XLOC_041191	ENSRNOT00000005311	0.974080873	8.82E-08
TUCP_001081	XLOC_041191	ENSRNOT00000026760	-0.970552037	1.66E-07
TUCP_001081	XLOC_041191	ENSRNOT00000017687	-0.952112694	1.83E-06
TUCP_001081	XLOC_041191	ENSRNOT00000024491	-0.963017884	5.12E-07
TUCP_001081	XLOC_041191	ENSRNOT00000081077	-0.966752409	3.03E-07
TUCP_001081	XLOC_041191	ENSRNOT00000016568	0.950457441	2.16E-06
TUCP_001081	XLOC_041191	ENSRNOT00000016401	0.950959837	2.06E-06
TUCP_001081	XLOC_041191	ENSRNOT00000027967	0.966422435	3.18E-07
TUCP_001081	XLOC_041191	ENSRNOT00000047210	0.975478592	6.70E-08
TUCP_001081	XLOC_041191	ENSRNOT00000078349	-0.953701056	1.55E-06
TUCP_001081	XLOC_041191	ENSRNOT00000080110	0.96322415	4.98E-07
TUCP_001081	XLOC_041191	ENSRNOT00000083957	0.976498586	5.43E-08
TUCP_001081	XLOC_041191	ENSRNOT00000038066	0.95549406	1.28E-06
TUCP_001081	XLOC_041191	ENSRNOT00000046639	0.963836416	4.58E-07
TUCP_001081	XLOC_041191	ENSRNOT00000079996	0.960044202	7.50E-07
TUCP_001081	XLOC_041191	ENSRNOT00000024548	0.966863512	2.98E-07
TUCP_002346	XLOC_098002	ENSRNOT00000006071	0.971197823	1.49E-07
TUCP_002346	XLOC_098002	ENSRNOT00000082172	0.959528891	7.99E-07
TUCP_002346	XLOC_098002	ENSRNOT00000064070	0.965885329	3.44E-07
TUCP_002346	XLOC_098002	ENSRNOT00000079019	0.951361956	1.98E-06
TUCP_002346	XLOC_098002	ENSRNOT00000023623	0.955398796	1.29E-06
TUCP_002346	XLOC_098002	ENSRNOT00000084177	-0.971066643	1.52E-07
TUCP_002346	XLOC_098002	ENSRNOT00000015448	0.965691267	3.53E-07
TUCP_002346	XLOC_098002	ENSRNOT00000072682	0.954634502	1.40E-06
TUCP_002346	XLOC_098002	ENSRNOT00000083344	0.958374951	9.18E-07
TUCP_002346	XLOC_098002	ENSRNOT00000023613	0.962144833	5.74E-07

TUCP_002346	XLOC_098002	ENSRNOT00000061430	0.961386583	6.34E-07
TUCP_002346	XLOC_098002	ENSRNOT00000032106	0.95506638	1.34E-06
TUCP_002346	XLOC_098002	ENSRNOT00000057740	0.987361639	2.49E-09
TUCP_002346	XLOC_098002	ENSRNOT00000010701	0.957217318	1.05E-06
TUCP_002346	XLOC_098002	ENSRNOT00000023661	0.953429221	1.60E-06
TUCP_002346	XLOC_098002	ENSRNOT00000081604	0.959394798	8.12E-07
TUCP_002346	XLOC_098002	ENSRNOT00000008440	0.953625273	1.56E-06
TUCP_002346	XLOC_098002	ENSRNOT00000017829	0.969193639	2.08E-07
TUCP_002346	XLOC_098002	ENSRNOT00000071068	-0.9566985	1.11E-06
TUCP_002346	XLOC_098002	ENSRNOT00000064122	0.964785509	4.02E-07
TUCP_002346	XLOC_098002	ENSRNOT00000091211	0.960595482	7.00E-07
TUCP_002262	XLOC_093918	ENSRNOT00000035865	-0.951408013	1.97E-06
TUCP_002262	XLOC_093918	ENSRNOT00000073955	0.952836551	1.70E-06
TUCP_002262	XLOC_093918	ENSRNOT00000049357	-0.953707446	1.55E-06
TUCP_002262	XLOC_093918	ENSRNOT00000079388	-0.961049045	6.61E-07
TUCP_002263	XLOC_093941	ENSRNOT00000050656	0.950056581	2.25E-06
TUCP_002263	XLOC_093941	ENSRNOT00000056557	0.962051604	5.81E-07
TUCP_002263	XLOC_093941	ENSRNOT00000065448	0.952016741	1.85E-06
TUCP_002263	XLOC_093941	ENSRNOT00000029990	0.951819049	1.89E-06
TUCP_002263	XLOC_093941	ENSRNOT00000020071	0.955523093	1.27E-06
TUCP_002263	XLOC_093941	ENSRNOT00000088078	0.95643087	1.15E-06
TUCP_002263	XLOC_093941	ENSRNOT00000043486	0.970446435	1.69E-07
TUCP_002263	XLOC_093941	ENSRNOT00000047564	0.956264001	1.17E-06
TUCP_002263	XLOC_093941	ENSRNOT00000017412	0.967427106	2.73E-07
TUCP_002263	XLOC_093941	ENSRNOT00000076910	0.965518676	3.62E-07
TUCP_002263	XLOC_093941	ENSRNOT00000092272	0.957547239	1.01E-06
TUCP_002263	XLOC_093941	ENSRNOT00000082002	0.964060193	4.45E-07
TUCP_002263	XLOC_093941	ENSRNOT00000084429	0.952054867	1.84E-06
TUCP_002263	XLOC_093941	ENSRNOT00000081108	0.950405071	2.17E-06
TUCP_002263	XLOC_093941	ENSRNOT00000030532	0.974001254	8.96E-08
TUCP_002263	XLOC_093941	ENSRNOT00000076618	0.96970327	1.91E-07
TUCP_000572	XLOC_020108	ENSRNOT00000064193	0.979840998	2.53E-08
TUCP_000572	XLOC_020108	ENSRNOT00000075987	0.960606803	6.99E-07
TUCP_000572	XLOC_020108	ENSRNOT00000003451	0.985401262	5.10E-09
TUCP_000572	XLOC_020108	ENSRNOT00000073853	0.960480231	7.10E-07
TUCP_000573	XLOC_020375	ENSRNOT00000002191	0.957869523	9.74E-07
TUCP_000573	XLOC_020375	ENSRNOT00000031458	0.954296042	1.45E-06
TUCP_000573	XLOC_020375	ENSRNOT00000090688	0.96801894	2.50E-07
TUCP_000573	XLOC_020375	ENSRNOT00000028759	0.952247094	1.80E-06
TUCP_000573	XLOC_020375	ENSRNOT00000007634	0.963510824	4.79E-07
TUCP_000573	XLOC_020375	ENSRNOT00000085424	0.972926988	1.09E-07
TUCP_000573	XLOC_020375	ENSRNOT00000078503	0.96329623	4.93E-07
TUCP_000573	XLOC_020375	ENSRNOT00000041897	-0.952032068	1.84E-06
TUCP_000573	XLOC_020375	ENSRNOT00000013049	0.955195782	1.32E-06
TUCP_000573	XLOC_020375	ENSRNOT00000019886	0.954096785	1.49E-06
TUCP_000573	XLOC_020375	ENSRNOT00000056430	0.955664204	1.25E-06
TUCP_002266	XLOC_094427	ENSRNOT00000000053	0.962788277	5.28E-07
TUCP_000575	XLOC_020464	ENSRNOT00000083871	0.950793426	2.09E-06
TUCP_001089	XLOC_041516	ENSRNOT00000008573	0.957533841	1.01E-06
TUCP_001089	XLOC_041516	ENSRNOT00000009979	0.954432237	1.43E-06
TUCP_001089	XLOC_041516	ENSRNOT00000006186	0.971191898	1.49E-07

TUCP_001089	XLOC_041516	ENSRNOT00000009877	0.966062473	3.35E-07
TUCP_001089	XLOC_041516	ENSRNOT00000021348	0.982179028	1.37E-08
TUCP_001089	XLOC_041516	ENSRNOT00000073559	0.990745713	5.26E-10
TUCP_001089	XLOC_041516	ENSRNOT00000003891	0.972278846	1.23E-07
TUCP_001089	XLOC_041516	ENSRNOT00000025067	-0.950522739	2.15E-06
TUCP_001089	XLOC_041516	ENSRNOT00000004017	0.9552133	1.32E-06
TUCP_001089	XLOC_041516	ENSRNOT00000086310	-0.950486175	2.16E-06
TUCP_001089	XLOC_041516	ENSRNOT00000010030	-0.95215191	1.82E-06
TUCP_001089	XLOC_041516	ENSRNOT00000028856	0.980266763	2.28E-08
TUCP_001089	XLOC_041516	ENSRNOT00000030850	0.974746256	7.75E-08
TUCP_001089	XLOC_041516	ENSRNOT00000055582	0.957258478	1.05E-06
TUCP_001089	XLOC_041516	ENSRNOT00000033017	0.950243323	2.21E-06
TUCP_001089	XLOC_041516	ENSRNOT00000027414	0.962139077	5.75E-07
TUCP_001089	XLOC_041516	ENSRNOT00000006752	0.95947124	8.04E-07
TUCP_001089	XLOC_041516	ENSRNOT00000006321	-0.955852022	1.23E-06
TUCP_001089	XLOC_041516	ENSRNOT00000079698	-0.954698538	1.39E-06
TUCP_001089	XLOC_041516	ENSRNOT00000085251	-0.963481871	4.81E-07
TUCP_001089	XLOC_041516	ENSRNOT00000072771	0.950573304	2.14E-06
TUCP_001089	XLOC_041516	ENSRNOT00000065300	0.96612774	3.32E-07
TUCP_001089	XLOC_041516	ENSRNOT00000044742	0.987059645	2.80E-09
TUCP_001089	XLOC_041516	ENSRNOT00000014701	0.959169531	8.34E-07
TUCP_001089	XLOC_041516	ENSRNOT00000029439	0.977525529	4.35E-08
TUCP_001089	XLOC_041516	ENSRNOT00000045285	0.954156733	1.48E-06
TUCP_001089	XLOC_041516	ENSRNOT00000076820	0.966790844	3.01E-07
TUCP_001089	XLOC_041516	ENSRNOT00000045047	0.952448725	1.77E-06
TUCP_001089	XLOC_041516	ENSRNOT00000075326	0.964835909	3.99E-07
TUCP_001089	XLOC_041516	ENSRNOT00000077999	0.959936798	7.60E-07
TUCP_001089	XLOC_041516	ENSRNOT00000068134	0.952640966	1.73E-06
TUCP_001089	XLOC_041516	ENSRNOT00000024266	0.960719296	6.89E-07
TUCP_001089	XLOC_041516	ENSRNOT00000083215	0.955904699	1.22E-06
TUCP_001089	XLOC_041516	ENSRNOT00000085228	0.995034181	2.36E-11
TUCP_001089	XLOC_041516	ENSRNOT00000020134	0.962956877	5.16E-07
TUCP_001089	XLOC_041516	ENSRNOT00000017319	0.98571935	4.57E-09
TUCP_001089	XLOC_041516	ENSRNOT00000055187	0.952982769	1.67E-06
TUCP_001089	XLOC_041516	ENSRNOT00000005934	0.957941216	9.66E-07
TUCP_001089	XLOC_041516	ENSRNOT00000079866	0.987451901	2.40E-09
TUCP_001089	XLOC_041516	ENSRNOT00000005839	0.968792912	2.21E-07
TUCP_001089	XLOC_041516	ENSRNOT00000008655	0.95116114	2.02E-06
TUCP_001089	XLOC_041516	ENSRNOT00000010149	0.956557721	1.13E-06
TUCP_001089	XLOC_041516	ENSRNOT00000087371	0.961628241	6.14E-07
TUCP_001089	XLOC_041516	ENSRNOT00000037446	0.95012042	2.24E-06
TUCP_001089	XLOC_041516	ENSRNOT00000084709	0.959875461	7.66E-07
TUCP_001089	XLOC_041516	ENSRNOT00000068033	0.959634035	7.89E-07
TUCP_001089	XLOC_041516	ENSRNOT00000009156	0.970390451	1.71E-07
TUCP_001089	XLOC_041516	ENSRNOT00000026481	0.975089737	7.24E-08
TUCP_001089	XLOC_041516	ENSRNOT00000000468	0.977408099	4.46E-08
TUCP_001089	XLOC_041516	ENSRNOT00000044562	0.96767254	2.63E-07
TUCP_001089	XLOC_041516	ENSRNOT00000012892	0.976079922	5.92E-08
TUCP_001089	XLOC_041516	ENSRNOT00000090918	0.96482307	4.00E-07
TUCP_001089	XLOC_041516	ENSRNOT00000023180	0.965115144	3.84E-07
TUCP_001089	XLOC_041516	ENSRNOT00000070815	0.950437647	2.17E-06

TUCP_001089	XLOC_041516	ENSRNOT00000028070	0.960387098	7.19E-07
TUCP_001089	XLOC_041516	ENSRNOT00000016132	-0.966335774	3.22E-07
TUCP_001089	XLOC_041516	ENSRNOT00000023477	0.9673731	2.76E-07
TUCP_001089	XLOC_041516	ENSRNOT00000034692	-0.956386059	1.15E-06
TUCP_001089	XLOC_041516	ENSRNOT00000048063	0.953710147	1.55E-06
TUCP_001089	XLOC_041516	ENSRNOT00000031979	-0.960033828	7.51E-07
TUCP_001089	XLOC_041516	ENSRNOT00000033975	0.95203168	1.84E-06
TUCP_001089	XLOC_041516	ENSRNOT00000079895	0.96930918	2.04E-07
TUCP_001089	XLOC_041516	ENSRNOT00000078102	0.97824566	3.70E-08
TUCP_001089	XLOC_041516	ENSRNOT00000026633	0.958413532	9.13E-07
TUCP_001089	XLOC_041516	ENSRNOT00000058072	0.96910365	2.11E-07
TUCP_001089	XLOC_041516	ENSRNOT00000021496	-0.951857362	1.88E-06
TUCP_001089	XLOC_041516	ENSRNOT00000011880	0.97201362	1.29E-07
TUCP_001089	XLOC_041516	ENSRNOT00000023989	0.979336772	2.87E-08
TUCP_001089	XLOC_041516	ENSRNOT00000003991	0.972034224	1.29E-07
TUCP_001089	XLOC_041516	ENSRNOT00000021408	0.979003786	3.10E-08
TUCP_001089	XLOC_041516	ENSRNOT00000050515	0.966417609	3.18E-07
TUCP_001089	XLOC_041516	ENSRNOT00000084088	0.95860025	8.93E-07
TUCP_001089	XLOC_041516	ENSRNOT00000064902	0.963547121	4.77E-07
TUCP_001089	XLOC_041516	ENSRNOT00000025027	0.959328815	8.18E-07
TUCP_001089	XLOC_041516	ENSRNOT00000052224	0.954424158	1.43E-06
TUCP_001089	XLOC_041516	ENSRNOT00000078070	0.967513032	2.70E-07
TUCP_001089	XLOC_041516	ENSRNOT00000046745	0.98593718	4.23E-09
TUCP_001089	XLOC_041516	ENSRNOT00000021015	0.974064136	8.85E-08
TUCP_001089	XLOC_041516	ENSRNOT00000076669	0.954169529	1.47E-06
TUCP_001089	XLOC_041516	ENSRNOT00000076729	0.956394429	1.15E-06
TUCP_001089	XLOC_041516	ENSRNOT00000011408	0.97016329	1.77E-07
TUCP_001089	XLOC_041516	ENSRNOT00000091488	0.970117217	1.78E-07
TUCP_001089	XLOC_041516	ENSRNOT00000027452	0.982542368	1.24E-08
TUCP_001089	XLOC_041516	ENSRNOT00000019656	-0.955411971	1.29E-06
TUCP_001089	XLOC_041516	ENSRNOT00000006499	0.974015685	8.93E-08
TUCP_001089	XLOC_041516	ENSRNOT00000074649	0.963412252	4.86E-07
TUCP_001089	XLOC_041516	ENSRNOT00000025936	-0.956933467	1.09E-06
TUCP_001089	XLOC_041516	ENSRNOT00000036951	0.983239042	1.01E-08
TUCP_001089	XLOC_041516	ENSRNOT00000006969	0.970869886	1.57E-07
TUCP_001089	XLOC_041516	ENSRNOT00000011663	0.957668779	9.97E-07
TUCP_001089	XLOC_041516	ENSRNOT00000078250	0.959270507	8.24E-07
TUCP_001089	XLOC_041516	ENSRNOT00000066133	-0.964207322	4.36E-07
TUCP_001089	XLOC_041516	ENSRNOT00000051953	-0.954704645	1.39E-06
TUCP_001089	XLOC_041516	ENSRNOT00000085596	0.963141655	5.04E-07
TUCP_001089	XLOC_041516	ENSRNOT00000000728	-0.962592489	5.42E-07
TUCP_001089	XLOC_041516	ENSRNOT00000019487	0.952452625	1.77E-06
TUCP_001089	XLOC_041516	ENSRNOT00000080443	0.956454852	1.15E-06
TUCP_001089	XLOC_041516	ENSRNOT00000040069	0.955439532	1.28E-06
TUCP_001089	XLOC_041516	ENSRNOT00000010748	0.993171991	1.16E-10
TUCP_001089	XLOC_041516	ENSRNOT00000035474	0.994287977	4.74E-11
TUCP_001089	XLOC_041516	ENSRNOT00000023807	-0.953548645	1.58E-06
TUCP_001089	XLOC_041516	ENSRNOT00000091205	0.974379292	8.33E-08
TUCP_001089	XLOC_041516	ENSRNOT00000060696	0.970321405	1.73E-07
TUCP_001089	XLOC_041516	ENSRNOT00000004787	-0.956506026	1.14E-06
TUCP_001089	XLOC_041516	ENSRNOT00000058351	0.957341441	1.04E-06

TUCP_001089	XLOC_041516	ENSRNOT00000014814	0.968523797	2.31E-07
TUCP_001089	XLOC_041516	ENSRNOT00000004412	0.970957822	1.55E-07
TUCP_001089	XLOC_041516	ENSRNOT00000080207	0.969861061	1.86E-07
TUCP_001089	XLOC_041516	ENSRNOT00000061100	0.972747852	1.13E-07
TUCP_001089	XLOC_041516	ENSRNOT00000043535	0.965119098	3.84E-07
TUCP_001089	XLOC_041516	ENSRNOT00000013985	0.9700828	1.80E-07
TUCP_001089	XLOC_041516	ENSRNOT00000018998	0.960668216	6.94E-07
TUCP_001089	XLOC_041516	ENSRNOT00000004463	0.981134954	1.82E-08
TUCP_001089	XLOC_041516	ENSRNOT00000009462	0.95146602	1.95E-06
TUCP_001089	XLOC_041516	ENSRNOT00000008982	0.980427705	2.19E-08
TUCP_001089	XLOC_041516	ENSRNOT00000047192	-0.979512079	2.75E-08
TUCP_001089	XLOC_041516	ENSRNOT00000090299	0.95383195	1.53E-06
TUCP_001089	XLOC_041516	ENSRNOT00000029001	0.951509746	1.95E-06
TUCP_001089	XLOC_041516	ENSRNOT00000083799	0.97138554	1.44E-07
TUCP_001089	XLOC_041516	ENSRNOT00000081246	0.984140783	7.69E-09
TUCP_001089	XLOC_041516	ENSRNOT00000074939	0.979476335	2.77E-08
TUCP_001089	XLOC_041516	ENSRNOT00000066041	-0.954959058	1.35E-06
TUCP_001089	XLOC_041516	ENSRNOT00000006055	0.954977963	1.35E-06
TUCP_001089	XLOC_041516	ENSRNOT00000001290	0.958545219	8.99E-07
TUCP_001089	XLOC_041516	ENSRNOT00000081768	0.955675736	1.25E-06
TUCP_001089	XLOC_041516	ENSRNOT00000019502	0.978347873	3.61E-08
TUCP_001089	XLOC_041516	ENSRNOT00000018057	0.952939615	1.68E-06
TUCP_001089	XLOC_041516	ENSRNOT00000076763	0.959798615	7.73E-07
TUCP_001089	XLOC_041516	ENSRNOT00000043216	0.985985168	4.16E-09
TUCP_001089	XLOC_041516	ENSRNOT00000081981	0.97935089	2.86E-08
TUCP_001089	XLOC_041516	ENSRNOT00000028563	0.962983412	5.14E-07
TUCP_001089	XLOC_041516	ENSRNOT00000010418	0.963588577	4.74E-07
TUCP_001089	XLOC_041516	ENSRNOT00000045699	0.969069598	2.12E-07
TUCP_001089	XLOC_041516	ENSRNOT00000038247	0.975649321	6.47E-08
TUCP_001089	XLOC_041516	ENSRNOT00000047836	-0.959485858	8.03E-07
TUCP_001089	XLOC_041516	ENSRNOT00000012379	0.961691484	6.09E-07
TUCP_001089	XLOC_041516	ENSRNOT00000029866	0.955469981	1.28E-06
TUCP_001089	XLOC_041516	ENSRNOT00000048754	-0.973442818	9.95E-08
TUCP_001089	XLOC_041516	ENSRNOT00000044628	0.954851117	1.37E-06
TUCP_001089	XLOC_041516	ENSRNOT00000025075	0.955463306	1.28E-06
TUCP_001089	XLOC_041516	ENSRNOT00000079109	0.959803673	7.72E-07
TUCP_001089	XLOC_041516	ENSRNOT00000011926	0.960214252	7.34E-07
TUCP_001089	XLOC_041516	ENSRNOT00000004007	-0.953390971	1.60E-06
TUCP_001089	XLOC_041516	ENSRNOT00000052075	0.963949739	4.51E-07
TUCP_001089	XLOC_041516	ENSRNOT00000091602	-0.958740928	8.79E-07
TUCP_001089	XLOC_041516	ENSRNOT00000012671	0.954460997	1.43E-06
TUCP_001089	XLOC_041516	ENSRNOT00000006680	0.953228602	1.63E-06
TUCP_001089	XLOC_041516	ENSRNOT00000009708	-0.963066424	5.09E-07
TUCP_001089	XLOC_041516	ENSRNOT00000086065	0.959438656	8.08E-07
TUCP_001089	XLOC_041516	ENSRNOT00000077231	0.968461244	2.33E-07
TUCP_001089	XLOC_041516	ENSRNOT00000009811	0.983079392	1.06E-08
TUCP_001089	XLOC_041516	ENSRNOT00000087592	0.961568341	6.19E-07
TUCP_001089	XLOC_041516	ENSRNOT00000052002	0.95530308	1.30E-06
TUCP_001089	XLOC_041516	ENSRNOT00000017556	0.958958586	8.56E-07
TUCP_001089	XLOC_041516	ENSRNOT00000087443	0.971989532	1.30E-07
TUCP_001089	XLOC_041516	ENSRNOT00000021803	-0.963615956	4.72E-07

TUCP_001089	XLOC_041516	ENSRNOT00000020028	0.971321613	1.46E-07
TUCP_001089	XLOC_041516	ENSRNOT00000046973	0.955353205	1.30E-06
TUCP_001089	XLOC_041516	ENSRNOT00000024352	0.954925113	1.36E-06
TUCP_001089	XLOC_041516	ENSRNOT00000083220	0.960335662	7.23E-07
TUCP_001089	XLOC_041516	ENSRNOT00000058312	0.963908288	4.54E-07
TUCP_001089	XLOC_041516	ENSRNOT00000060362	0.97634401	5.61E-08
TUCP_001089	XLOC_041516	ENSRNOT00000013274	0.9712413	1.48E-07
TUCP_001089	XLOC_041516	ENSRNOT00000022610	0.955864202	1.22E-06
TUCP_001089	XLOC_041516	ENSRNOT00000010898	0.96029627	7.27E-07
TUCP_001089	XLOC_041516	ENSRNOT00000068381	0.972747932	1.13E-07
TUCP_001089	XLOC_041516	ENSRNOT00000029167	0.95195545	1.86E-06
TUCP_001089	XLOC_041516	ENSRNOT00000010873	-0.952492177	1.76E-06
TUCP_001089	XLOC_041516	ENSRNOT00000088599	0.95974699	7.78E-07
TUCP_001089	XLOC_041516	ENSRNOT00000016129	0.959502421	8.01E-07
TUCP_001089	XLOC_041516	ENSRNOT00000026174	0.970787099	1.60E-07
TUCP_001089	XLOC_041516	ENSRNOT00000017021	0.959864485	7.67E-07
TUCP_001089	XLOC_041516	ENSRNOT00000034744	0.951746219	1.90E-06
TUCP_001089	XLOC_041516	ENSRNOT00000009683	0.964517722	4.17E-07
TUCP_001089	XLOC_041516	ENSRNOT00000005459	0.956948874	1.08E-06
TUCP_001089	XLOC_041516	ENSRNOT00000017776	0.989469913	1.00E-09
TUCP_001089	XLOC_041516	ENSRNOT00000027910	0.979993256	2.44E-08
TUCP_001089	XLOC_041516	ENSRNOT00000002771	0.965001471	3.90E-07
TUCP_001089	XLOC_041516	ENSRNOT00000079442	0.953072142	1.66E-06
TUCP_001089	XLOC_041516	ENSRNOT00000024322	0.989506287	9.85E-10
TUCP_001089	XLOC_041516	ENSRNOT00000088182	0.950693896	2.11E-06
TUCP_001089	XLOC_041516	ENSRNOT00000011270	0.964017194	4.47E-07
TUCP_001089	XLOC_041516	ENSRNOT00000003770	-0.95639321	1.15E-06
TUCP_001089	XLOC_041516	ENSRNOT00000010784	0.967073435	2.88E-07
TUCP_001089	XLOC_041516	ENSRNOT00000016191	0.959174774	8.34E-07
TUCP_001089	XLOC_041516	ENSRNOT00000078390	-0.963115006	5.05E-07
TUCP_001089	XLOC_041516	ENSRNOT00000026457	0.972960309	1.09E-07
TUCP_001089	XLOC_041516	ENSRNOT00000008427	-0.956492207	1.14E-06
TUCP_001089	XLOC_041516	ENSRNOT00000002109	0.971225099	1.48E-07
TUCP_001089	XLOC_041516	ENSRNOT00000029833	0.982477781	1.26E-08
TUCP_001089	XLOC_041516	ENSRNOT00000034487	-0.955908274	1.22E-06
TUCP_001089	XLOC_041516	ENSRNOT00000016519	0.95017372	2.22E-06
TUCP_001089	XLOC_041516	ENSRNOT00000002173	-0.952483869	1.76E-06
TUCP_001089	XLOC_041516	ENSRNOT00000015188	0.97969822	2.63E-08
TUCP_001089	XLOC_041516	ENSRNOT00000090420	-0.950765786	2.10E-06
TUCP_001089	XLOC_041516	ENSRNOT00000031401	-0.956601164	1.13E-06
TUCP_001089	XLOC_041516	ENSRNOT00000000157	0.961661235	6.12E-07
TUCP_001089	XLOC_041516	ENSRNOT00000027666	0.951963613	1.86E-06
TUCP_001089	XLOC_041516	ENSRNOT00000072518	0.985779415	4.47E-09
TUCP_001089	XLOC_041516	ENSRNOT00000011984	0.965737447	3.51E-07
TUCP_001089	XLOC_041516	ENSRNOT00000012895	0.965272818	3.75E-07
TUCP_001089	XLOC_041516	ENSRNOT00000016991	0.969949918	1.83E-07
TUCP_001089	XLOC_041516	ENSRNOT00000020669	0.956335857	1.16E-06
TUCP_001089	XLOC_041516	ENSRNOT00000043872	0.961732694	6.06E-07
TUCP_001089	XLOC_041516	ENSRNOT00000074653	0.957794427	9.82E-07
TUCP_001089	XLOC_041516	ENSRNOT00000031230	0.973609658	9.64E-08
TUCP_001089	XLOC_041516	ENSRNOT00000015898	0.956735471	1.11E-06

TUCP_001089	XLOC_041516	ENSRNOT00000022142	0.956198342	1.18E-06
TUCP_001089	XLOC_041516	ENSRNOT00000077406	0.976667488	5.24E-08
TUCP_001089	XLOC_041516	ENSRNOT00000005405	0.965821335	3.47E-07
TUCP_001089	XLOC_041516	ENSRNOT00000083127	0.960811911	6.81E-07
TUCP_001089	XLOC_041516	ENSRNOT00000052120	0.957886819	9.72E-07
TUCP_001089	XLOC_041516	ENSRNOT00000019820	0.979886799	2.51E-08
TUCP_001089	XLOC_041516	ENSRNOT00000004172	0.984616799	6.61E-09
TUCP_001089	XLOC_041516	ENSRNOT00000040476	0.970900709	1.56E-07
TUCP_001089	XLOC_041516	ENSRNOT00000029125	0.981027945	1.88E-08
TUCP_001089	XLOC_041516	ENSRNOT00000085279	0.969528929	1.97E-07
TUCP_001089	XLOC_041516	ENSRNOT00000067181	0.954280405	1.46E-06
TUCP_001089	XLOC_041516	ENSRNOT00000004319	-0.951366892	1.97E-06
TUCP_001089	XLOC_041516	ENSRNOT00000026280	0.974169305	8.67E-08
TUCP_001089	XLOC_041516	ENSRNOT00000010927	0.971101555	1.51E-07
TUCP_001089	XLOC_041516	ENSRNOT00000005387	0.953355437	1.61E-06
TUCP_001089	XLOC_041516	ENSRNOT00000022862	0.954545184	1.42E-06
TUCP_001089	XLOC_041516	ENSRNOT00000015092	0.950292843	2.20E-06
TUCP_001089	XLOC_041516	ENSRNOT00000017165	-0.971830912	1.33E-07
TUCP_001089	XLOC_041516	ENSRNOT00000010224	0.970320024	1.73E-07
TUCP_001089	XLOC_041516	ENSRNOT00000007175	0.972229592	1.24E-07
TUCP_001089	XLOC_041516	ENSRNOT00000051022	0.972885668	1.10E-07
TUCP_001089	XLOC_041516	ENSRNOT00000017687	-0.950064531	2.25E-06
TUCP_001089	XLOC_041516	ENSRNOT00000015799	0.982630116	1.21E-08
TUCP_001089	XLOC_041516	ENSRNOT00000005958	0.96859378	2.28E-07
TUCP_001089	XLOC_041516	ENSRNOT00000039551	-0.950640569	2.12E-06
TUCP_001089	XLOC_041516	ENSRNOT00000082998	0.969489156	1.98E-07
TUCP_001089	XLOC_041516	ENSRNOT00000015006	0.977373389	4.50E-08
TUCP_001089	XLOC_041516	ENSRNOT00000007351	-0.978220465	3.72E-08
TUCP_001089	XLOC_041516	ENSRNOT00000017665	0.954924137	1.36E-06
TUCP_001089	XLOC_041516	ENSRNOT00000023133	0.957056195	1.07E-06
TUCP_001089	XLOC_041516	ENSRNOT00000026065	0.952341825	1.79E-06
TUCP_001089	XLOC_041516	ENSRNOT00000023638	0.981126564	1.83E-08
TUCP_001089	XLOC_041516	ENSRNOT00000005228	0.973598052	9.67E-08
TUCP_001089	XLOC_041516	ENSRNOT00000022726	0.966684865	3.06E-07
TUCP_001089	XLOC_041516	ENSRNOT00000028628	-0.957204316	1.05E-06
TUCP_001089	XLOC_041516	ENSRNOT00000005220	0.970110973	1.79E-07
TUCP_001089	XLOC_041516	ENSRNOT00000023144	0.965786229	3.49E-07
TUCP_001089	XLOC_041516	ENSRNOT00000045326	0.975441763	6.75E-08
TUCP_001089	XLOC_041516	ENSRNOT00000016649	0.966282728	3.24E-07
TUCP_001089	XLOC_041516	ENSRNOT00000090342	0.973744262	9.40E-08
TUCP_001089	XLOC_041516	ENSRNOT00000067170	-0.955701152	1.25E-06
TUCP_001089	XLOC_041516	ENSRNOT00000077871	0.950332505	2.19E-06
TUCP_001089	XLOC_041516	ENSRNOT00000091128	-0.975730636	6.37E-08
TUCP_001089	XLOC_041516	ENSRNOT00000001586	-0.959516369	8.00E-07
TUCP_001089	XLOC_041516	ENSRNOT00000026543	0.964388801	4.25E-07
TUCP_001089	XLOC_041516	ENSRNOT00000066670	0.954476381	1.43E-06
TUCP_001089	XLOC_041516	ENSRNOT00000081786	-0.950210499	2.22E-06
TUCP_001089	XLOC_041516	ENSRNOT00000071971	0.968720165	2.24E-07
TUCP_001089	XLOC_041516	ENSRNOT00000043554	0.961500733	6.24E-07
TUCP_001089	XLOC_041516	ENSRNOT00000012187	0.979044367	3.07E-08
TUCP_001089	XLOC_041516	ENSRNOT00000024969	0.961463373	6.27E-07

TUCP_001089	XLOC_041516	ENSRNOT00000013049	0.953464125	1.59E-06
TUCP_001089	XLOC_041516	ENSRNOT00000026783	0.984811762	6.21E-09
TUCP_001089	XLOC_041516	ENSRNOT00000088125	0.957142385	1.06E-06
TUCP_001089	XLOC_041516	ENSRNOT00000084385	0.976632855	5.28E-08
TUCP_001089	XLOC_041516	ENSRNOT00000027944	0.950907247	2.07E-06
TUCP_001089	XLOC_041516	ENSRNOT00000024402	0.959578245	7.94E-07
TUCP_001089	XLOC_041516	ENSRNOT00000019519	0.956997629	1.08E-06
TUCP_001089	XLOC_041516	ENSRNOT00000089933	0.971874104	1.32E-07
TUCP_001089	XLOC_041516	ENSRNOT00000077908	0.969458562	1.99E-07
TUCP_001089	XLOC_041516	ENSRNOT00000090912	0.951329868	1.98E-06
TUCP_001089	XLOC_041516	ENSRNOT00000010463	0.975303774	6.94E-08
TUCP_001089	XLOC_041516	ENSRNOT00000026718	0.966219939	3.27E-07
TUCP_001089	XLOC_041516	ENSRNOT00000072208	0.96100291	6.65E-07
TUCP_001089	XLOC_041516	ENSRNOT00000032780	0.977079477	4.79E-08
TUCP_001089	XLOC_041516	ENSRNOT00000033677	-0.964069673	4.44E-07
TUCP_001089	XLOC_041516	ENSRNOT00000072196	0.97271465	1.14E-07
TUCP_001089	XLOC_041516	ENSRNOT00000036931	-0.951319508	1.98E-06
TUCP_001089	XLOC_041516	ENSRNOT00000067822	0.961750931	6.05E-07
TUCP_001089	XLOC_041516	ENSRNOT00000002410	0.972735649	1.13E-07
TUCP_001089	XLOC_041516	ENSRNOT00000024060	0.952107641	1.83E-06
TUCP_001089	XLOC_041516	ENSRNOT00000077727	-0.950182078	2.22E-06
TUCP_001089	XLOC_041516	ENSRNOT00000021037	0.980421695	2.19E-08
TUCP_001089	XLOC_041516	ENSRNOT00000044346	-0.953458588	1.59E-06
TUCP_001089	XLOC_041516	ENSRNOT00000010541	0.967279074	2.80E-07
TUCP_001089	XLOC_041516	ENSRNOT00000004032	0.974223815	8.58E-08
TUCP_001089	XLOC_041516	ENSRNOT00000048613	0.961962781	5.88E-07
TUCP_001089	XLOC_041516	ENSRNOT00000017948	0.954963034	1.35E-06
TUCP_001089	XLOC_041516	ENSRNOT00000091909	0.968071125	2.48E-07
TUCP_001089	XLOC_041516	ENSRNOT00000001081	0.98144376	1.68E-08
TUCP_001089	XLOC_041516	ENSRNOT00000086119	-0.970024095	1.81E-07
TUCP_001089	XLOC_041516	ENSRNOT00000019222	0.958367197	9.18E-07
TUCP_001089	XLOC_041516	ENSRNOT00000077220	-0.950953001	2.06E-06
TUCP_001089	XLOC_041516	ENSRNOT00000065983	0.975148706	7.16E-08
TUCP_001089	XLOC_041516	ENSRNOT00000030782	-0.951672498	1.91E-06
TUCP_001089	XLOC_041516	ENSRNOT00000052018	0.955447033	1.28E-06
TUCP_001089	XLOC_041516	ENSRNOT00000067780	-0.958894498	8.63E-07
TUCP_001089	XLOC_041516	ENSRNOT00000015265	0.963549309	4.77E-07
TUCP_001089	XLOC_041516	ENSRNOT00000007768	0.959898744	7.63E-07
TUCP_001089	XLOC_041516	ENSRNOT00000052422	0.985132228	5.58E-09
TUCP_001089	XLOC_041516	ENSRNOT00000001242	0.966131662	3.32E-07
TUCP_001089	XLOC_041516	ENSRNOT00000015609	0.984730517	6.37E-09
TUCP_001089	XLOC_041516	ENSRNOT00000086590	0.967226632	2.82E-07
TUCP_001089	XLOC_041516	ENSRNOT00000025983	0.956903045	1.09E-06
TUCP_001089	XLOC_041516	ENSRNOT00000020155	0.9634231	4.85E-07
TUCP_001089	XLOC_041516	ENSRNOT00000055321	0.952366374	1.78E-06
TUCP_001089	XLOC_041516	ENSRNOT00000004698	0.962284971	5.64E-07
TUCP_001089	XLOC_041516	ENSRNOT00000045083	0.952439753	1.77E-06
TUCP_001089	XLOC_041516	ENSRNOT00000014537	0.981008526	1.88E-08
TUCP_001089	XLOC_041516	ENSRNOT00000017629	0.954383518	1.44E-06
TUCP_001089	XLOC_041516	ENSRNOT00000078475	0.971220546	1.48E-07
TUCP_001089	XLOC_041516	ENSRNOT00000087429	0.962586575	5.42E-07

TUCP_001089	XLOC_041516	ENSRNOT00000022213	0.961977879	5.87E-07
TUCP_001089	XLOC_041516	ENSRNOT00000016722	0.975303938	6.94E-08
TUCP_001089	XLOC_041516	ENSRNOT00000091265	0.971438152	1.43E-07
TUCP_001089	XLOC_041516	ENSRNOT00000073526	0.961713261	6.08E-07
TUCP_001089	XLOC_041516	ENSRNOT00000065501	0.95226135	1.80E-06
TUCP_001089	XLOC_041516	ENSRNOT00000000013	0.978245698	3.70E-08
TUCP_001089	XLOC_041516	ENSRNOT00000017753	0.990861086	4.94E-10
TUCP_001089	XLOC_041516	ENSRNOT00000045882	0.959698443	7.82E-07
TUCP_001089	XLOC_041516	ENSRNOT00000009198	0.959548761	7.97E-07
TUCP_001089	XLOC_041516	ENSRNOT00000031904	0.954367238	1.44E-06
TUCP_001089	XLOC_041516	ENSRNOT00000009129	0.962820003	5.26E-07
TUCP_001089	XLOC_041516	ENSRNOT00000022074	0.981467692	1.67E-08
TUCP_001089	XLOC_041516	ENSRNOT00000009691	0.954972907	1.35E-06
TUCP_001089	XLOC_041516	ENSRNOT00000041365	0.962933845	5.18E-07
TUCP_001089	XLOC_041516	ENSRNOT00000088054	0.962532509	5.46E-07
TUCP_001089	XLOC_041516	ENSRNOT00000038703	0.965965394	3.40E-07
TUCP_001089	XLOC_041516	ENSRNOT00000019554	0.963455648	4.83E-07
TUCP_001089	XLOC_041516	ENSRNOT00000011384	0.962483943	5.50E-07
TUCP_001089	XLOC_041516	ENSRNOT00000035700	0.982071369	1.42E-08
TUCP_001089	XLOC_041516	ENSRNOT00000029202	-0.958797093	8.73E-07
TUCP_001089	XLOC_041516	ENSRNOT00000092029	0.974586161	8.00E-08
TUCP_001089	XLOC_041516	ENSRNOT00000084422	0.976667946	5.24E-08
TUCP_001089	XLOC_041516	ENSRNOT00000050834	-0.951097703	2.03E-06
TUCP_001089	XLOC_041516	ENSRNOT00000054828	0.981778369	1.53E-08
TUCP_001089	XLOC_041516	ENSRNOT00000078994	0.969029799	2.13E-07
TUCP_001089	XLOC_041516	ENSRNOT00000027970	0.96849239	2.32E-07
TUCP_001089	XLOC_041516	ENSRNOT00000080928	0.954501827	1.42E-06
TUCP_001089	XLOC_041516	ENSRNOT00000018074	-0.963900968	4.54E-07
TUCP_001089	XLOC_041516	ENSRNOT00000014124	0.982215622	1.36E-08
TUCP_001089	XLOC_041516	ENSRNOT00000000120	0.953199026	1.63E-06
TUCP_001089	XLOC_041516	ENSRNOT00000013632	0.96381556	4.60E-07
TUCP_001089	XLOC_041516	ENSRNOT00000009900	-0.962201225	5.70E-07
TUCP_001089	XLOC_041516	ENSRNOT00000014979	0.967251639	2.81E-07
TUCP_001089	XLOC_041516	ENSRNOT00000019172	-0.953011162	1.67E-06
TUCP_001089	XLOC_041516	ENSRNOT00000064798	0.994830896	2.88E-11
TUCP_001089	XLOC_041516	ENSRNOT00000019177	0.977025604	4.85E-08
TUCP_001089	XLOC_041516	ENSRNOT00000022485	0.97513629	7.18E-08
TUCP_001089	XLOC_041516	ENSRNOT00000040393	0.979308299	2.89E-08
TUCP_001089	XLOC_041516	ENSRNOT00000067901	-0.972775213	1.13E-07
TUCP_001089	XLOC_041516	ENSRNOT00000036750	0.969900088	1.85E-07
TUCP_001089	XLOC_041516	ENSRNOT00000024659	0.957631343	1.00E-06
TUCP_001089	XLOC_041516	ENSRNOT00000011041	0.959501359	8.02E-07
TUCP_001089	XLOC_041516	ENSRNOT00000021674	0.968623183	2.27E-07
TUCP_001089	XLOC_041516	ENSRNOT00000064700	0.973108417	1.06E-07
TUCP_001089	XLOC_041516	ENSRNOT00000036613	-0.953268376	1.62E-06
TUCP_001089	XLOC_041516	ENSRNOT00000002292	0.976307843	5.65E-08
TUCP_001089	XLOC_041516	ENSRNOT00000025705	0.952495725	1.76E-06
TUCP_001089	XLOC_041516	ENSRNOT00000015479	0.973518178	9.81E-08
TUCP_001089	XLOC_041516	ENSRNOT00000004140	0.976497562	5.43E-08
TUCP_001089	XLOC_041516	ENSRNOT00000011682	-0.954271355	1.46E-06
TUCP_001089	XLOC_041516	ENSRNOT00000027407	0.958817118	8.71E-07

TUCP_001089	XLOC_041516	ENSRNOT00000048910	-0.965287137	3.74E-07
TUCP_001089	XLOC_041516	ENSRNOT00000019213	0.972029383	1.29E-07
TUCP_001089	XLOC_041516	ENSRNOT00000071560	0.957825971	9.79E-07
TUCP_001089	XLOC_041516	ENSRNOT00000086336	0.969875423	1.86E-07
TUCP_001089	XLOC_041516	ENSRNOT00000082508	-0.959308028	8.21E-07
TUCP_001089	XLOC_041516	ENSRNOT00000082863	0.958062289	9.52E-07
TUCP_001089	XLOC_041516	ENSRNOT00000007164	0.966007776	3.38E-07
TUCP_001089	XLOC_041516	ENSRNOT00000049459	-0.951391286	1.97E-06
TUCP_001089	XLOC_041516	ENSRNOT00000071166	0.959258532	8.25E-07
TUCP_001089	XLOC_041516	ENSRNOT00000047387	0.959290785	8.22E-07
TUCP_001089	XLOC_041516	ENSRNOT00000025079	-0.962486174	5.49E-07
TUCP_001089	XLOC_041516	ENSRNOT00000009496	0.979111513	3.02E-08
TUCP_001089	XLOC_041516	ENSRNOT00000083369	0.959746605	7.78E-07
TUCP_001089	XLOC_041516	ENSRNOT00000043345	0.963072624	5.08E-07
TUCP_001089	XLOC_041516	ENSRNOT00000076872	0.981728926	1.56E-08
TUCP_001089	XLOC_041516	ENSRNOT00000015073	0.958199593	9.37E-07
TUCP_001089	XLOC_041516	ENSRNOT00000005942	0.962196853	5.71E-07
TUCP_001089	XLOC_041516	ENSRNOT00000018533	0.960074536	7.47E-07
TUCP_001089	XLOC_041516	ENSRNOT00000001507	0.969463272	1.99E-07
TUCP_001089	XLOC_041516	ENSRNOT00000028646	0.957166162	1.06E-06
TUCP_001089	XLOC_041516	ENSRNOT00000081047	0.981794372	1.53E-08
TUCP_001089	XLOC_041516	ENSRNOT00000071764	0.979632956	2.67E-08
TUCP_001089	XLOC_041516	ENSRNOT00000016801	0.950738486	2.10E-06
TUCP_001089	XLOC_041516	ENSRNOT00000037456	0.974502407	8.13E-08
TUCP_001089	XLOC_041516	ENSRNOT00000065168	0.971604445	1.39E-07
TUCP_001089	XLOC_041516	ENSRNOT00000071853	-0.953297507	1.62E-06
TUCP_001089	XLOC_041516	ENSRNOT00000000577	0.956994328	1.08E-06
TUCP_001089	XLOC_041516	ENSRNOT00000055669	0.953422749	1.60E-06
TUCP_001089	XLOC_041516	ENSRNOT00000001809	0.956064011	1.20E-06
TUCP_001089	XLOC_041516	ENSRNOT00000041513	0.951361244	1.98E-06
TUCP_001089	XLOC_041516	ENSRNOT00000061392	0.978796977	3.26E-08
TUCP_001089	XLOC_041516	ENSRNOT00000027012	0.959830343	7.70E-07
TUCP_001089	XLOC_041516	ENSRNOT00000024771	0.959159463	8.35E-07
TUCP_001089	XLOC_041516	ENSRNOT00000073991	0.957676193	9.96E-07
TUCP_001089	XLOC_041516	ENSRNOT00000028266	0.962414732	5.55E-07
TUCP_001089	XLOC_041516	ENSRNOT00000016321	-0.95769629	9.94E-07
TUCP_001089	XLOC_041516	ENSRNOT00000019133	0.974036983	8.89E-08
TUCP_001089	XLOC_041516	ENSRNOT00000020437	0.973444674	9.95E-08
TUCP_001089	XLOC_041516	ENSRNOT00000024119	0.961779146	6.02E-07
TUCP_001089	XLOC_041516	ENSRNOT00000087095	0.968222975	2.42E-07
TUCP_001089	XLOC_041516	ENSRNOT00000090123	0.971176505	1.49E-07
TUCP_001089	XLOC_041516	ENSRNOT00000027526	0.961422401	6.31E-07
TUCP_001089	XLOC_041516	ENSRNOT00000045488	0.950851741	2.08E-06
TUCP_001089	XLOC_041516	ENSRNOT00000093174	0.964060593	4.45E-07
TUCP_001089	XLOC_041516	ENSRNOT00000077555	0.974038011	8.89E-08
TUCP_001089	XLOC_041516	ENSRNOT00000078073	-0.958774693	8.75E-07
TUCP_001089	XLOC_041516	ENSRNOT00000036666	0.968931021	2.16E-07
TUCP_001089	XLOC_041516	ENSRNOT00000089211	0.956172046	1.18E-06
TUCP_001089	XLOC_041516	ENSRNOT00000087502	0.970286843	1.74E-07
TUCP_001089	XLOC_041516	ENSRNOT00000041106	0.955318053	1.30E-06
TUCP_001089	XLOC_041516	ENSRNOT00000012424	0.95572116	1.24E-06

TUCP_001089	XLOC_041516	ENSRNOT00000000658	0.955440161	1.28E-06
TUCP_001089	XLOC_041516	ENSRNOT00000021944	0.957309484	1.04E-06
TUCP_001089	XLOC_041516	ENSRNOT00000025832	0.97301864	1.08E-07
TUCP_001089	XLOC_041516	ENSRNOT00000005909	0.98323046	1.02E-08
TUCP_001089	XLOC_041516	ENSRNOT00000087725	0.950002236	2.26E-06
TUCP_001089	XLOC_041516	ENSRNOT00000086122	0.976557127	5.36E-08
TUCP_001089	XLOC_041516	ENSRNOT00000026231	0.951678425	1.91E-06
TUCP_001089	XLOC_041516	ENSRNOT00000044009	0.966122192	3.32E-07
TUCP_001089	XLOC_041516	ENSRNOT00000075424	0.970518778	1.67E-07
TUCP_001089	XLOC_041516	ENSRNOT00000058842	0.967748951	2.60E-07
TUCP_001089	XLOC_041516	ENSRNOT00000023645	0.96180189	6.01E-07
TUCP_001089	XLOC_041516	ENSRNOT00000084734	0.956884796	1.09E-06
TUCP_001089	XLOC_041516	ENSRNOT00000059181	0.951921434	1.87E-06
TUCP_001089	XLOC_041516	ENSRNOT00000064840	0.963701232	4.67E-07
TUCP_001089	XLOC_041516	ENSRNOT00000003690	0.973010464	1.08E-07
TUCP_001089	XLOC_041516	ENSRNOT00000090925	0.967109849	2.87E-07
TUCP_001089	XLOC_041516	ENSRNOT00000011350	0.960075156	7.47E-07
TUCP_001089	XLOC_041516	ENSRNOT00000008901	-0.968843984	2.19E-07
TUCP_001089	XLOC_041516	ENSRNOT00000005659	0.970544494	1.66E-07
TUCP_001089	XLOC_041516	ENSRNOT00000017761	0.972663144	1.15E-07
TUCP_001089	XLOC_041516	ENSRNOT00000058563	0.953829963	1.53E-06
TUCP_001089	XLOC_041516	ENSRNOT00000080608	0.962225227	5.68E-07
TUCP_001089	XLOC_041516	ENSRNOT00000071733	0.966837492	2.99E-07
TUCP_001089	XLOC_041516	ENSRNOT00000008757	0.956863661	1.09E-06
TUCP_001089	XLOC_041516	ENSRNOT00000005478	-0.956228581	1.18E-06
TUCP_001089	XLOC_041516	ENSRNOT00000039341	0.970688098	1.62E-07
TUCP_001089	XLOC_041516	ENSRNOT00000034704	0.960598541	7.00E-07
TUCP_001089	XLOC_041516	ENSRNOT00000005490	0.974394228	8.30E-08
TUCP_001089	XLOC_041516	ENSRNOT00000082174	0.978462262	3.52E-08
TUCP_001089	XLOC_041516	ENSRNOT00000042125	0.965380404	3.70E-07
TUCP_001089	XLOC_041516	ENSRNOT00000026760	-0.952881863	1.69E-06
TUCP_001089	XLOC_041516	ENSRNOT00000012048	0.958233905	9.33E-07
TUCP_001089	XLOC_041516	ENSRNOT00000061432	0.954306348	1.45E-06
TUCP_001089	XLOC_041516	ENSRNOT00000023330	0.96468439	4.08E-07
TUCP_001089	XLOC_041516	ENSRNOT00000081077	-0.951261738	2.00E-06
TUCP_001089	XLOC_041516	ENSRNOT00000033235	0.984013871	8.00E-09
TUCP_001089	XLOC_041516	ENSRNOT00000027964	0.954405204	1.44E-06
TUCP_001089	XLOC_041516	ENSRNOT00000023428	0.965208779	3.79E-07
TUCP_001089	XLOC_041516	ENSRNOT00000047210	0.976783096	5.11E-08
TUCP_001089	XLOC_041516	ENSRNOT00000083957	0.958559863	8.98E-07
TUCP_001089	XLOC_041516	ENSRNOT00000064930	0.966493192	3.14E-07
TUCP_001089	XLOC_041516	ENSRNOT00000079996	0.978151869	3.78E-08
TUCP_001089	XLOC_041516	ENSRNOT00000024548	0.967566421	2.68E-07
TUCP_001088	XLOC_041384	ENSRNOT00000019524	-0.957135221	1.06E-06
TUCP_001088	XLOC_041384	ENSRNOT00000079180	0.962058809	5.81E-07
TUCP_001088	XLOC_041384	ENSRNOT00000024030	0.957987956	9.60E-07
TUCP_003824	XLOC_167348	ENSRNOT00000000008	1	0
TUCP_003824	XLOC_167348	ENSRNOT00000060048	0.998711457	2.79E-14
TUCP_003824	XLOC_167348	ENSRNOT00000082304	0.996875989	2.33E-12
TUCP_003824	XLOC_167348	ENSRNOT00000006219	1	0
TUCP_003824	XLOC_167348	ENSRNOT00000065376	1	0

TUCP_003824	XLOC_167348	ENSRNOT00000081433	0.984469223	6.93E-09
TUCP_003824	XLOC_167348	ENSRNOT00000083575	1	0
TUCP_003824	XLOC_167348	ENSRNOT00000087739	1	0
TUCP_003824	XLOC_167348	ENSRNOT00000079826	1	0
TUCP_003824	XLOC_167348	ENSRNOT00000025770	0.993828251	6.98E-11
TUCP_003824	XLOC_167348	ENSRNOT00000088244	0.973479481	9.88E-08
TUCP_003824	XLOC_167348	ENSRNOT00000048523	1	0
TUCP_003824	XLOC_167348	ENSRNOT00000079365	0.958936699	8.58E-07
TUCP_003824	XLOC_167348	ENSRNOT00000020229	0.960002785	7.54E-07
TUCP_003824	XLOC_167348	ENSRNOT00000026428	0.996986683	1.95E-12
TUCP_003824	XLOC_167348	ENSRNOT00000085673	1	0
TUCP_003824	XLOC_167348	ENSRNOT00000072972	0.981476166	1.67E-08
TUCP_003824	XLOC_167348	ENSRNOT00000050573	1	0
TUCP_003824	XLOC_167348	ENSRNOT00000089876	-0.953630353	1.56E-06
TUCP_003824	XLOC_167348	ENSRNOT00000074378	0.998901807	1.26E-14
TUCP_003824	XLOC_167348	ENSRNOT00000093571	1	0
TUCP_003824	XLOC_167348	ENSRNOT00000090581	1	0
TUCP_003824	XLOC_167348	ENSRNOT00000076239	0.98020752	2.31E-08
TUCP_003824	XLOC_167348	ENSRNOT00000065485	1	0
TUCP_003824	XLOC_167348	ENSRNOT00000006356	0.999544279	1.55E-16
TUCP_003824	XLOC_167348	ENSRNOT00000087199	0.974421555	8.26E-08
TUCP_003824	XLOC_167348	ENSRNOT00000022091	1	0
TUCP_003824	XLOC_167348	ENSRNOT00000064764	0.98460888	6.63E-09
TUCP_003824	XLOC_167348	ENSRNOT00000076927	0.971163156	1.50E-07
TUCP_003824	XLOC_167348	ENSRNOT00000092395	0.999522978	1.94E-16
TUCP_003884	XLOC_170720	ENSRNOT00000027223	0.9625977	5.41E-07
TUCP_003884	XLOC_170720	ENSRNOT00000008372	0.951361482	1.98E-06
TUCP_003884	XLOC_170720	ENSRNOT00000070893	0.953784688	1.54E-06
TUCP_003375	XLOC_146309	ENSRNOT00000092021	0.950316089	2.19E-06
TUCP_003375	XLOC_146309	ENSRNOT00000071482	0.955334735	1.30E-06
TUCP_003375	XLOC_146309	ENSRNOT00000003412	0.951362562	1.98E-06
TUCP_003375	XLOC_146309	ENSRNOT00000045765	0.95891554	8.60E-07
TUCP_003375	XLOC_146309	ENSRNOT00000006765	0.960922358	6.72E-07
TUCP_003375	XLOC_146309	ENSRNOT00000007079	0.974441137	8.23E-08
TUCP_003375	XLOC_146309	ENSRNOT00000014780	0.952120393	1.83E-06
TUCP_003375	XLOC_146309	ENSRNOT00000025980	0.956566572	1.13E-06
TUCP_003375	XLOC_146309	ENSRNOT00000000083	0.95281195	1.70E-06
TUCP_003375	XLOC_146309	ENSRNOT00000000707	0.959016288	8.50E-07
TUCP_003375	XLOC_146309	ENSRNOT00000019848	0.956305858	1.17E-06
TUCP_003375	XLOC_146309	ENSRNOT00000079962	-0.973015006	1.08E-07
TUCP_003375	XLOC_146309	ENSRNOT00000023567	0.962916957	5.19E-07
TUCP_003375	XLOC_146309	ENSRNOT00000014885	0.979200131	2.96E-08
TUCP_003375	XLOC_146309	ENSRNOT00000086608	0.971697358	1.36E-07
TUCP_003375	XLOC_146309	ENSRNOT00000056041	0.95031789	2.19E-06
TUCP_003375	XLOC_146309	ENSRNOT00000058495	0.955772348	1.24E-06
TUCP_003375	XLOC_146309	ENSRNOT00000018864	0.966369696	3.20E-07
TUCP_003375	XLOC_146309	ENSRNOT00000033048	0.959330561	8.18E-07
TUCP_003375	XLOC_146309	ENSRNOT00000012583	0.955534446	1.27E-06
TUCP_003375	XLOC_146309	ENSRNOT00000036338	0.974651244	7.90E-08
TUCP_003375	XLOC_146309	ENSRNOT00000076181	0.957932222	9.67E-07
TUCP_003375	XLOC_146309	ENSRNOT00000091139	0.961762702	6.04E-07

TUCP_003375	XLOC_146309	ENSRNOT00000080410	0.951964469	1.86E-06
TUCP_003375	XLOC_146309	ENSRNOT00000022117	0.980532782	2.13E-08
TUCP_003375	XLOC_146309	ENSRNOT00000017362	0.961542387	6.21E-07
TUCP_003375	XLOC_146309	ENSRNOT00000008222	0.951645457	1.92E-06
TUCP_003375	XLOC_146309	ENSRNOT00000014585	0.960744444	6.87E-07
TUCP_003375	XLOC_146309	ENSRNOT00000010962	0.95579432	1.23E-06
TUCP_003375	XLOC_146309	ENSRNOT00000002040	0.950962128	2.06E-06
TUCP_003375	XLOC_146309	ENSRNOT00000020932	0.953412853	1.60E-06
TUCP_003375	XLOC_146309	ENSRNOT00000085516	0.956593459	1.13E-06
TUCP_003375	XLOC_146309	ENSRNOT00000019473	0.950858005	2.08E-06
TUCP_003375	XLOC_146309	ENSRNOT00000001135	0.976110268	5.89E-08
TUCP_003375	XLOC_146309	ENSRNOT00000028703	0.954691008	1.39E-06
TUCP_003375	XLOC_146309	ENSRNOT00000002779	0.955356377	1.30E-06
TUCP_003375	XLOC_146309	ENSRNOT00000086350	0.951635688	1.92E-06
TUCP_003375	XLOC_146309	ENSRNOT00000039296	-0.9676392	2.65E-07
TUCP_003375	XLOC_146309	ENSRNOT00000033015	0.959341875	8.17E-07
TUCP_003375	XLOC_146309	ENSRNOT00000019713	0.964623372	4.11E-07
TUCP_003375	XLOC_146309	ENSRNOT00000084531	0.9568361	1.10E-06
TUCP_003375	XLOC_146309	ENSRNOT00000076105	0.951326576	1.98E-06
TUCP_003375	XLOC_146309	ENSRNOT00000007367	0.953256141	1.62E-06
TUCP_003375	XLOC_146309	ENSRNOT00000042633	0.97675828	5.14E-08
TUCP_003375	XLOC_146309	ENSRNOT00000081110	0.959415513	8.10E-07
TUCP_003375	XLOC_146309	ENSRNOT00000025856	0.954828637	1.37E-06
TUCP_003375	XLOC_146309	ENSRNOT00000085214	0.950967684	2.06E-06
TUCP_003375	XLOC_146309	ENSRNOT00000004941	0.950265076	2.20E-06
TUCP_003375	XLOC_146309	ENSRNOT00000001935	0.953128007	1.65E-06
TUCP_003375	XLOC_146309	ENSRNOT00000005148	0.95489947	1.36E-06
TUCP_003375	XLOC_146309	ENSRNOT00000000052	0.968005752	2.50E-07
TUCP_003375	XLOC_146309	ENSRNOT00000020994	0.952949982	1.68E-06
TUCP_003375	XLOC_146309	ENSRNOT00000016541	0.956046862	1.20E-06
TUCP_003375	XLOC_146309	ENSRNOT00000085311	0.959631288	7.89E-07
TUCP_003375	XLOC_146309	ENSRNOT00000033450	0.950724842	2.11E-06
TUCP_003375	XLOC_146309	ENSRNOT00000024947	0.955474854	1.28E-06
TUCP_003375	XLOC_146309	ENSRNOT00000049725	0.959013013	8.50E-07
TUCP_003375	XLOC_146309	ENSRNOT00000019130	0.95693626	1.08E-06
TUCP_003375	XLOC_146309	ENSRNOT00000015757	0.950824664	2.08E-06
TUCP_003375	XLOC_146309	ENSRNOT00000070912	0.955069757	1.34E-06
TUCP_003375	XLOC_146309	ENSRNOT00000008253	0.953162197	1.64E-06
TUCP_003375	XLOC_146309	ENSRNOT00000021455	0.950722267	2.11E-06
TUCP_003375	XLOC_146309	ENSRNOT00000003544	0.951054984	2.04E-06
TUCP_003375	XLOC_146309	ENSRNOT00000017081	0.955866973	1.22E-06
TUCP_003375	XLOC_146309	ENSRNOT00000056877	0.962669387	5.36E-07
TUCP_003375	XLOC_146309	ENSRNOT00000008865	0.968612966	2.28E-07
TUCP_003375	XLOC_146309	ENSRNOT00000033196	0.967005443	2.91E-07
TUCP_003375	XLOC_146309	ENSRNOT00000022241	0.960451188	7.13E-07
TUCP_003375	XLOC_146309	ENSRNOT00000010674	0.953038503	1.66E-06
TUCP_003375	XLOC_146309	ENSRNOT00000091068	0.95663647	1.12E-06
TUCP_003375	XLOC_146309	ENSRNOT00000023664	0.957692475	9.94E-07
TUCP_003375	XLOC_146309	ENSRNOT00000087748	0.955005946	1.35E-06
TUCP_003375	XLOC_146309	ENSRNOT00000009073	0.964498954	4.18E-07
TUCP_003375	XLOC_146309	ENSRNOT00000007077	0.957588498	1.01E-06

TUCP_003375	XLOC_146309	ENSRNOT00000017688	0.95171075	1.91E-06
TUCP_003375	XLOC_146309	ENSRNOT00000074494	0.968796918	2.21E-07
TUCP_003375	XLOC_146309	ENSRNOT00000017469	0.951731636	1.90E-06
TUCP_003375	XLOC_146309	ENSRNOT00000049193	0.955682117	1.25E-06
TUCP_003375	XLOC_146309	ENSRNOT00000059599	0.962850011	5.24E-07
TUCP_003375	XLOC_146309	ENSRNOT00000009000	0.971467979	1.42E-07
TUCP_003375	XLOC_146309	ENSRNOT00000000155	-0.961339729	6.37E-07
TUCP_003375	XLOC_146309	ENSRNOT00000019192	0.957615353	1.00E-06
TUCP_003822	XLOC_167348	ENSRNOT00000000008	1	0
TUCP_003822	XLOC_167348	ENSRNOT00000060048	0.998711457	2.79E-14
TUCP_003822	XLOC_167348	ENSRNOT00000082304	0.996875989	2.33E-12
TUCP_003822	XLOC_167348	ENSRNOT00000006219	1	0
TUCP_003822	XLOC_167348	ENSRNOT00000065376	1	0
TUCP_003822	XLOC_167348	ENSRNOT00000081433	0.984469223	6.93E-09
TUCP_003822	XLOC_167348	ENSRNOT00000083575	1	0
TUCP_003822	XLOC_167348	ENSRNOT00000087739	1	1.33E-79
TUCP_003822	XLOC_167348	ENSRNOT00000079826	1	0
TUCP_003822	XLOC_167348	ENSRNOT00000025770	0.993828251	6.98E-11
TUCP_003822	XLOC_167348	ENSRNOT00000088244	0.973479481	9.88E-08
TUCP_003822	XLOC_167348	ENSRNOT00000048523	1	0
TUCP_003822	XLOC_167348	ENSRNOT00000079365	0.958936699	8.58E-07
TUCP_003822	XLOC_167348	ENSRNOT00000020229	0.960002785	7.54E-07
TUCP_003822	XLOC_167348	ENSRNOT00000026428	0.996986683	1.95E-12
TUCP_003822	XLOC_167348	ENSRNOT00000085673	1	1.33E-79
TUCP_003822	XLOC_167348	ENSRNOT00000072972	0.981476166	1.67E-08
TUCP_003822	XLOC_167348	ENSRNOT00000050573	1	1.33E-79
TUCP_003822	XLOC_167348	ENSRNOT00000089876	-0.953630353	1.56E-06
TUCP_003822	XLOC_167348	ENSRNOT00000074378	0.998901807	1.26E-14
TUCP_003822	XLOC_167348	ENSRNOT00000093571	1	1.33E-79
TUCP_003822	XLOC_167348	ENSRNOT00000090581	1	4.25E-78
TUCP_003822	XLOC_167348	ENSRNOT00000076239	0.98020752	2.31E-08
TUCP_003822	XLOC_167348	ENSRNOT00000065485	1	1.33E-79
TUCP_003822	XLOC_167348	ENSRNOT00000006356	0.999544279	1.55E-16
TUCP_003822	XLOC_167348	ENSRNOT00000087199	0.974421555	8.26E-08
TUCP_003822	XLOC_167348	ENSRNOT00000022091	1	0
TUCP_003822	XLOC_167348	ENSRNOT00000064764	0.98460888	6.63E-09
TUCP_003822	XLOC_167348	ENSRNOT00000076927	0.971163156	1.50E-07
TUCP_003822	XLOC_167348	ENSRNOT00000092395	0.999522978	1.94E-16
TUCP_001078	XLOC_041095	ENSRNOT00000078014	0.955314657	1.30E-06
TUCP_000689	XLOC_026080	ENSRNOT00000074990	0.983290101	9.98E-09
TUCP_000689	XLOC_026080	ENSRNOT00000068444	0.983290101	9.98E-09
TUCP_000689	XLOC_026080	ENSRNOT00000081370	0.984189729	7.58E-09
TUCP_000689	XLOC_026080	ENSRNOT00000078645	0.962524645	5.47E-07
TUCP_000689	XLOC_026080	ENSRNOT00000075987	0.969537088	1.96E-07
TUCP_000689	XLOC_026080	ENSRNOT00000082043	0.982903563	1.12E-08
TUCP_000689	XLOC_026080	ENSRNOT00000087323	0.990925673	4.77E-10
TUCP_000689	XLOC_026080	ENSRNOT00000074930	0.983290101	9.98E-09
TUCP_000689	XLOC_026080	ENSRNOT00000080575	0.982234376	1.35E-08
TUCP_000689	XLOC_026080	ENSRNOT00000057761	0.981654412	1.59E-08
TUCP_000689	XLOC_026080	ENSRNOT00000078369	0.983833672	8.46E-09
TUCP_000689	XLOC_026080	ENSRNOT00000092982	0.983290101	9.98E-09

TUCP_000689	XLOC_026080	ENSRNOT00000088367	0.983290101	9.98E-09
TUCP_000689	XLOC_026080	ENSRNOT00000078153	0.955036267	1.34E-06
TUCP_000689	XLOC_026080	ENSRNOT00000081996	0.983290101	9.98E-09
TUCP_000689	XLOC_026080	ENSRNOT00000022280	0.959055536	8.46E-07
TUCP_000689	XLOC_026080	ENSRNOT00000082058	0.983290101	9.98E-09
TUCP_000689	XLOC_026080	ENSRNOT00000088905	0.983290101	9.98E-09
TUCP_000689	XLOC_026080	ENSRNOT00000086831	0.983290101	9.98E-09
TUCP_000689	XLOC_026080	ENSRNOT00000045099	0.983290101	9.98E-09
TUCP_000689	XLOC_026080	ENSRNOT00000086745	0.984638203	6.57E-09
TUCP_000689	XLOC_026080	ENSRNOT00000085849	0.979018921	3.09E-08
TUCP_000689	XLOC_026080	ENSRNOT00000077789	0.980826638	1.98E-08
TUCP_000689	XLOC_026080	ENSRNOT00000090207	0.978973563	3.12E-08
TUCP_000689	XLOC_026080	ENSRNOT00000087025	0.983290101	9.98E-09
TUCP_000689	XLOC_026080	ENSRNOT00000019853	0.983290101	9.98E-09
TUCP_000689	XLOC_026080	ENSRNOT00000073853	0.96943284	2.00E-07
TUCP_000689	XLOC_026080	ENSRNOT00000065912	0.958453546	9.09E-07
TUCP_000689	XLOC_026080	ENSRNOT00000073828	0.974843524	7.61E-08
TUCP_000689	XLOC_026080	ENSRNOT00000093083	0.979569788	2.71E-08
TUCP_001775	XLOC_073508	ENSRNOT00000090629	0.956261769	1.17E-06
TUCP_001775	XLOC_073508	ENSRNOT00000008961	0.979228756	2.94E-08
TUCP_001775	XLOC_073508	ENSRNOT00000039876	0.980081857	2.39E-08
TUCP_001775	XLOC_073508	ENSRNOT00000055127	0.976255134	5.71E-08
TUCP_001775	XLOC_073508	ENSRNOT00000026677	0.954225606	1.47E-06
TUCP_001775	XLOC_073508	ENSRNOT00000001570	0.980699036	2.04E-08
TUCP_001775	XLOC_073508	ENSRNOT00000035523	0.971389777	1.44E-07
TUCP_001775	XLOC_073508	ENSRNOT00000025564	0.950650098	2.12E-06
TUCP_001775	XLOC_073508	ENSRNOT00000033719	0.965596053	3.58E-07
TUCP_001775	XLOC_073508	ENSRNOT00000000640	0.951003585	2.05E-06
TUCP_001775	XLOC_073508	ENSRNOT00000056174	0.964387533	4.25E-07
TUCP_001775	XLOC_073508	ENSRNOT00000074738	0.976880092	5.00E-08
TUCP_001775	XLOC_073508	ENSRNOT00000084429	0.957962373	9.63E-07
TUCP_001775	XLOC_073508	ENSRNOT00000077022	0.976834993	5.05E-08
TUCP_001775	XLOC_073508	ENSRNOT00000071258	-0.963817569	4.60E-07
TUCP_001775	XLOC_073508	ENSRNOT00000004637	0.958428047	9.12E-07
TUCP_001775	XLOC_073508	ENSRNOT00000031025	0.982592169	1.22E-08
TUCP_001775	XLOC_073508	ENSRNOT00000017042	0.954308427	1.45E-06
TUCP_001775	XLOC_073508	ENSRNOT00000056551	0.973445386	9.95E-08
TUCP_001775	XLOC_073508	ENSRNOT00000002695	0.981340839	1.73E-08
TUCP_001775	XLOC_073508	ENSRNOT00000056995	0.960626649	6.98E-07
TUCP_001775	XLOC_073508	ENSRNOT00000054757	0.984793791	6.24E-09
TUCP_001775	XLOC_073508	ENSRNOT00000001853	0.984682483	6.47E-09
TUCP_001775	XLOC_073508	ENSRNOT00000077134	0.979955059	2.46E-08
TUCP_001775	XLOC_073508	ENSRNOT00000057016	0.971612296	1.38E-07
TUCP_001775	XLOC_073508	ENSRNOT00000052003	0.952231859	1.81E-06
TUCP_001775	XLOC_073508	ENSRNOT00000060765	0.981178961	1.80E-08
TUCP_001775	XLOC_073508	ENSRNOT00000040162	0.958969912	8.55E-07
TUCP_001775	XLOC_073508	ENSRNOT00000083943	0.965319182	3.73E-07
TUCP_001775	XLOC_073508	ENSRNOT00000039263	0.976726625	5.17E-08
TUCP_001775	XLOC_073508	ENSRNOT00000084873	0.972140847	1.26E-07
TUCP_001775	XLOC_073508	ENSRNOT00000076407	0.955040964	1.34E-06
TUCP_001775	XLOC_073508	ENSRNOT00000088707	0.975935985	6.10E-08

TUCP_001775	XLOC_073508	ENSRNOT00000005361	0.967292776	2.79E-07
TUCP_001775	XLOC_073508	ENSRNOT00000019501	0.951027049	2.04E-06
TUCP_001775	XLOC_073508	ENSRNOT00000041741	0.975565465	6.58E-08
TUCP_001775	XLOC_073508	ENSRNOT00000034152	0.978230968	3.71E-08
TUCP_001775	XLOC_073508	ENSRNOT00000024718	0.970103464	1.79E-07
TUCP_001775	XLOC_073508	ENSRNOT00000009620	0.982896314	1.12E-08
TUCP_001775	XLOC_073508	ENSRNOT00000082143	0.953406673	1.60E-06
TUCP_001775	XLOC_073508	ENSRNOT00000085570	0.962105334	5.77E-07
TUCP_001775	XLOC_073508	ENSRNOT00000017071	0.959432204	8.08E-07
TUCP_001775	XLOC_073508	ENSRNOT00000073547	0.966928335	2.95E-07
TUCP_001775	XLOC_073508	ENSRNOT00000018511	0.952891159	1.69E-06
TUCP_001775	XLOC_073508	ENSRNOT00000043434	0.986719539	3.18E-09
TUCP_001775	XLOC_073508	ENSRNOT00000038426	0.960862514	6.77E-07
TUCP_001775	XLOC_073508	ENSRNOT00000090867	0.957018019	1.07E-06
TUCP_001775	XLOC_073508	ENSRNOT00000051363	0.979842038	2.53E-08
TUCP_001775	XLOC_073508	ENSRNOT00000022648	0.96489459	3.96E-07
TUCP_001775	XLOC_073508	ENSRNOT00000083067	0.971949611	1.30E-07
TUCP_001775	XLOC_073508	ENSRNOT00000084021	0.956528717	1.14E-06
TUCP_001775	XLOC_073508	ENSRNOT00000038494	0.960316721	7.25E-07
TUCP_001775	XLOC_073508	ENSRNOT00000057433	0.951998394	1.85E-06
TUCP_001775	XLOC_073508	ENSRNOT00000016449	0.968264367	2.40E-07
TUCP_001775	XLOC_073508	ENSRNOT00000087603	0.98008739	2.38E-08
TUCP_001775	XLOC_073508	ENSRNOT00000029990	0.967885414	2.55E-07
TUCP_001775	XLOC_073508	ENSRNOT00000023504	0.969900778	1.85E-07
TUCP_001775	XLOC_073508	ENSRNOT00000036753	0.974299146	8.46E-08
TUCP_001775	XLOC_073508	ENSRNOT00000025880	0.983769725	8.63E-09
TUCP_001775	XLOC_073508	ENSRNOT00000044983	0.957195945	1.05E-06
TUCP_001775	XLOC_073508	ENSRNOT00000080956	0.950055593	2.25E-06
TUCP_001775	XLOC_073508	ENSRNOT00000008365	0.951127831	2.02E-06
TUCP_001775	XLOC_073508	ENSRNOT00000047700	0.954279906	1.46E-06
TUCP_001775	XLOC_073508	ENSRNOT00000003823	0.952737208	1.72E-06
TUCP_001775	XLOC_073508	ENSRNOT00000010264	0.953042517	1.66E-06
TUCP_001775	XLOC_073508	ENSRNOT00000059660	0.960640316	6.96E-07
TUCP_001775	XLOC_073508	ENSRNOT00000072810	0.958846393	8.67E-07
TUCP_001774	XLOC_073508	ENSRNOT00000042251	0.95239433	1.78E-06
TUCP_001774	XLOC_073508	ENSRNOT00000043258	0.950842988	2.08E-06
TUCP_001774	XLOC_073508	ENSRNOT00000005726	0.989736046	8.82E-10
TUCP_001774	XLOC_073508	ENSRNOT00000086167	0.953857439	1.52E-06
TUCP_001774	XLOC_073508	ENSRNOT00000076802	0.966179477	3.29E-07
TUCP_001774	XLOC_073508	ENSRNOT00000074738	0.957467408	1.02E-06
TUCP_001774	XLOC_073508	ENSRNOT00000031025	0.960430703	7.15E-07
TUCP_001774	XLOC_073508	ENSRNOT00000017042	0.978208094	3.73E-08
TUCP_001774	XLOC_073508	ENSRNOT00000017071	0.984236385	7.47E-09
TUCP_001774	XLOC_073508	ENSRNOT00000010092	0.98516939	5.51E-09
TUCP_001774	XLOC_073508	ENSRNOT00000001853	0.95139135	1.97E-06
TUCP_001774	XLOC_073508	ENSRNOT00000077134	0.959405587	8.11E-07
TUCP_001774	XLOC_073508	ENSRNOT00000038494	0.985915983	4.26E-09
TUCP_001774	XLOC_073508	ENSRNOT00000081830	0.971081435	1.52E-07
TUCP_001774	XLOC_073508	ENSRNOT00000039263	0.973396634	1.00E-07
TUCP_001774	XLOC_073508	ENSRNOT00000078478	0.962077498	5.80E-07
TUCP_001774	XLOC_073508	ENSRNOT00000088707	0.985137174	5.57E-09

TUCP_001774	XLOC_073508	ENSRNOT00000041741	0.979123128	3.02E-08
TUCP_001774	XLOC_073508	ENSRNOT00000009620	0.959173517	8.34E-07
TUCP_001774	XLOC_073508	ENSRNOT00000082143	0.963661095	4.69E-07
TUCP_001774	XLOC_073508	ENSRNOT00000085570	0.964195173	4.36E-07
TUCP_001774	XLOC_073508	ENSRNOT00000083067	0.969746313	1.90E-07
TUCP_001774	XLOC_073508	ENSRNOT00000038426	0.958670416	8.86E-07
TUCP_001774	XLOC_073508	ENSRNOT00000028058	0.978093414	3.83E-08
TUCP_001774	XLOC_073508	ENSRNOT00000050968	0.950436689	2.17E-06
TUCP_001774	XLOC_073508	ENSRNOT00000025564	0.957033176	1.07E-06
TUCP_001774	XLOC_073508	ENSRNOT00000039876	0.977075621	4.80E-08
TUCP_001774	XLOC_073508	ENSRNOT00000002695	0.965988313	3.39E-07
TUCP_001774	XLOC_073508	ENSRNOT00000087603	0.975517431	6.65E-08
TUCP_001774	XLOC_073508	ENSRNOT00000012957	0.978988491	3.11E-08
TUCP_001774	XLOC_073508	ENSRNOT00000020786	0.971782942	1.34E-07
TUCP_001774	XLOC_073508	ENSRNOT00000084847	0.970917899	1.56E-07
TUCP_001774	XLOC_073508	ENSRNOT00000057433	0.981604028	1.61E-08
TUCP_001774	XLOC_073508	ENSRNOT00000052367	0.963071251	5.08E-07
TUCP_001774	XLOC_073508	ENSRNOT00000052003	0.987931416	1.98E-09
TUCP_001774	XLOC_073508	ENSRNOT00000072810	0.956601166	1.13E-06
TUCP_001777	XLOC_073508	ENSRNOT00000068416	0.959777837	7.75E-07
TUCP_001777	XLOC_073508	ENSRNOT00000086036	0.959178763	8.33E-07
TUCP_001777	XLOC_073508	ENSRNOT00000081709	0.960281853	7.28E-07
TUCP_001777	XLOC_073508	ENSRNOT00000042251	0.960102885	7.44E-07
TUCP_001777	XLOC_073508	ENSRNOT00000002984	0.963749785	4.64E-07
TUCP_001777	XLOC_073508	ENSRNOT00000020071	0.96584285	3.46E-07
TUCP_001777	XLOC_073508	ENSRNOT00000083764	0.957981228	9.61E-07
TUCP_001777	XLOC_073508	ENSRNOT00000050543	0.960753763	6.86E-07
TUCP_001777	XLOC_073508	ENSRNOT00000092365	0.956151039	1.19E-06
TUCP_001777	XLOC_073508	ENSRNOT00000074738	0.962784446	5.28E-07
TUCP_001777	XLOC_073508	ENSRNOT00000084429	0.956275826	1.17E-06
TUCP_001777	XLOC_073508	ENSRNOT00000089306	0.959834664	7.70E-07
TUCP_001777	XLOC_073508	ENSRNOT00000031025	0.951236502	2.00E-06
TUCP_001777	XLOC_073508	ENSRNOT00000045301	0.959795642	7.73E-07
TUCP_001777	XLOC_073508	ENSRNOT00000041186	0.959367232	8.15E-07
TUCP_001777	XLOC_073508	ENSRNOT00000010092	0.979325048	2.87E-08
TUCP_001777	XLOC_073508	ENSRNOT00000076182	0.959795642	7.73E-07
TUCP_001777	XLOC_073508	ENSRNOT00000038494	0.969985985	1.82E-07
TUCP_001777	XLOC_073508	ENSRNOT00000081830	0.953257192	1.62E-06
TUCP_001777	XLOC_073508	ENSRNOT00000047527	0.969297519	2.04E-07
TUCP_001777	XLOC_073508	ENSRNOT00000039263	0.971600566	1.39E-07
TUCP_001777	XLOC_073508	ENSRNOT00000078478	0.957979298	9.61E-07
TUCP_001777	XLOC_073508	ENSRNOT00000088707	0.964920161	3.94E-07
TUCP_001777	XLOC_073508	ENSRNOT00000043486	0.955558678	1.27E-06
TUCP_001777	XLOC_073508	ENSRNOT00000047564	0.957220177	1.05E-06
TUCP_001777	XLOC_073508	ENSRNOT00000041741	0.967904018	2.54E-07
TUCP_001777	XLOC_073508	ENSRNOT00000017071	0.964698446	4.07E-07
TUCP_001777	XLOC_073508	ENSRNOT00000014103	0.959795642	7.73E-07
TUCP_001777	XLOC_073508	ENSRNOT00000057433	0.976583361	5.33E-08
TUCP_001777	XLOC_073508	ENSRNOT00000082289	0.960483856	7.10E-07
TUCP_001777	XLOC_073508	ENSRNOT00000087140	0.96948654	1.98E-07
TUCP_001777	XLOC_073508	ENSRNOT00000082143	0.972582814	1.17E-07

TUCP_001777	XLOC_073508	ENSRNOT00000085570	0.981026312	1.88E-08
TUCP_001777	XLOC_073508	ENSRNOT00000056551	0.950597655	2.13E-06
TUCP_001777	XLOC_073508	ENSRNOT00000088893	0.959795642	7.73E-07
TUCP_001777	XLOC_073508	ENSRNOT00000076693	0.980313935	2.25E-08
TUCP_001777	XLOC_073508	ENSRNOT00000081014	0.959795642	7.73E-07
TUCP_001777	XLOC_073508	ENSRNOT00000038426	0.959391052	8.12E-07
TUCP_001777	XLOC_073508	ENSRNOT00000067543	0.959795642	7.73E-07
TUCP_001777	XLOC_073508	ENSRNOT00000076802	0.987504177	2.35E-09
TUCP_001777	XLOC_073508	ENSRNOT00000050968	0.967775858	2.59E-07
TUCP_001777	XLOC_073508	ENSRNOT00000083067	0.97495817	7.44E-08
TUCP_001777	XLOC_073508	ENSRNOT00000039876	0.956511213	1.14E-06
TUCP_001777	XLOC_073508	ENSRNOT00000004373	0.955383784	1.29E-06
TUCP_001777	XLOC_073508	ENSRNOT00000002695	0.953299237	1.62E-06
TUCP_001777	XLOC_073508	ENSRNOT00000063929	0.959795642	7.73E-07
TUCP_001777	XLOC_073508	ENSRNOT00000005726	0.984643394	6.56E-09
TUCP_001777	XLOC_073508	ENSRNOT00000087603	0.970960586	1.55E-07
TUCP_001777	XLOC_073508	ENSRNOT00000041617	0.974813092	7.65E-08
TUCP_001777	XLOC_073508	ENSRNOT00000091763	0.974571917	8.02E-08
TUCP_001777	XLOC_073508	ENSRNOT00000022200	0.961536634	6.21E-07
TUCP_001777	XLOC_073508	ENSRNOT00000067379	0.958769735	8.75E-07
TUCP_001777	XLOC_073508	ENSRNOT00000020786	0.973478224	9.88E-08
TUCP_001777	XLOC_073508	ENSRNOT00000084847	0.993888887	6.64E-11
TUCP_001777	XLOC_073508	ENSRNOT00000079275	0.961999713	5.85E-07
TUCP_001777	XLOC_073508	ENSRNOT00000052367	0.965896909	3.43E-07
TUCP_001777	XLOC_073508	ENSRNOT00000090658	0.959795642	7.73E-07
TUCP_001777	XLOC_073508	ENSRNOT00000088078	0.96099327	6.66E-07
TUCP_001777	XLOC_073508	ENSRNOT00000052003	0.974138012	8.72E-08
TUCP_001777	XLOC_073508	ENSRNOT00000057992	0.960847287	6.78E-07
TUCP_001777	XLOC_073508	ENSRNOT00000057472	0.959795642	7.73E-07
TUCP_001777	XLOC_073508	ENSRNOT00000065448	0.964256705	4.33E-07
TUCP_001777	XLOC_073508	ENSRNOT00000072810	0.966972269	2.93E-07
TUCP_001776	XLOC_073508	ENSRNOT00000046313	0.960150001	7.40E-07
TUCP_001776	XLOC_073508	ENSRNOT00000026303	0.969399716	2.01E-07
TUCP_001776	XLOC_073508	ENSRNOT00000080851	0.980508762	2.14E-08
TUCP_001776	XLOC_073508	ENSRNOT00000086899	0.989518052	9.79E-10
TUCP_001776	XLOC_073508	ENSRNOT00000067677	0.977828455	4.07E-08
TUCP_001776	XLOC_073508	ENSRNOT00000092125	0.986147857	3.92E-09
TUCP_001776	XLOC_073508	ENSRNOT00000051363	0.950023183	2.26E-06
TUCP_001776	XLOC_073508	ENSRNOT00000058626	0.955436391	1.28E-06
TUCP_001776	XLOC_073508	ENSRNOT00000049732	0.973931405	9.08E-08
TUCP_001776	XLOC_073508	ENSRNOT00000013732	0.960443609	7.14E-07
TUCP_001776	XLOC_073508	ENSRNOT00000009077	0.965309648	3.73E-07
TUCP_001776	XLOC_073508	ENSRNOT00000088151	0.953105865	1.65E-06
TUCP_001776	XLOC_073508	ENSRNOT00000071258	-0.960508666	7.08E-07
TUCP_001776	XLOC_073508	ENSRNOT00000023152	0.993573366	8.54E-11
TUCP_000682	XLOC_025909	ENSRNOT00000011284	-0.958060478	9.52E-07
TUCP_000683	XLOC_025909	ENSRNOT00000084396	0.967412277	2.74E-07
TUCP_000680	XLOC_025780	ENSRNOT00000089273	0.963777802	4.62E-07
TUCP_000680	XLOC_025780	ENSRNOT00000000167	0.965255935	3.76E-07
TUCP_003820	XLOC_167152	ENSRNOT00000090376	0.950724034	2.11E-06
TUCP_003820	XLOC_167152	ENSRNOT00000071573	0.95567404	1.25E-06

TUCP_003820	XLOC_167152	ENSRNOT00000078207	0.96320862	4.99E-07
TUCP_003820	XLOC_167152	ENSRNOT00000085332	-0.96087248	6.76E-07
TUCP_003820	XLOC_167152	ENSRNOT00000076793	0.964464911	4.20E-07
TUCP_001077	XLOC_041095	ENSRNOT00000090051	-0.9593326	8.18E-07
TUCP_001077	XLOC_041095	ENSRNOT00000072173	0.955697328	1.25E-06
TUCP_000685	XLOC_025959	ENSRNOT00000011317	0.955445476	1.28E-06
TUCP_001173	XLOC_044210	ENSRNOT00000020443	-0.96060578	6.99E-07
TUCP_001173	XLOC_044210	ENSRNOT00000027949	0.953087351	1.65E-06
TUCP_001173	XLOC_044210	ENSRNOT00000011134	0.957405058	1.03E-06
TUCP_001173	XLOC_044210	ENSRNOT00000033984	0.959690251	7.83E-07
TUCP_001173	XLOC_044210	ENSRNOT00000092272	0.961390338	6.33E-07
TUCP_001173	XLOC_044210	ENSRNOT00000035048	0.959817112	7.71E-07
TUCP_001171	XLOC_044183	ENSRNOT00000024186	0.961466728	6.27E-07
TUCP_001171	XLOC_044183	ENSRNOT00000074753	0.982954235	1.10E-08
TUCP_001171	XLOC_044183	ENSRNOT00000041904	0.979101998	3.03E-08
TUCP_001171	XLOC_044183	ENSRNOT00000083487	0.956631843	1.12E-06
TUCP_001171	XLOC_044183	ENSRNOT00000077990	0.979101998	3.03E-08
TUCP_001171	XLOC_044183	ENSRNOT00000038826	0.977705911	4.18E-08
TUCP_001171	XLOC_044183	ENSRNOT00000037094	0.954136421	1.48E-06
TUCP_001171	XLOC_044183	ENSRNOT00000037169	0.973096651	1.06E-07
TUCP_001171	XLOC_044183	ENSRNOT00000021243	0.975608877	6.53E-08
TUCP_001171	XLOC_044183	ENSRNOT00000060250	0.979482412	2.77E-08
TUCP_001171	XLOC_044183	ENSRNOT00000091934	0.979101998	3.03E-08
TUCP_001171	XLOC_044183	ENSRNOT00000086027	0.966295336	3.24E-07
TUCP_001171	XLOC_044183	ENSRNOT00000046585	0.989756673	8.73E-10
TUCP_001171	XLOC_044183	ENSRNOT00000080794	0.961865381	5.96E-07
TUCP_001171	XLOC_044183	ENSRNOT00000014456	0.968384594	2.36E-07
TUCP_001171	XLOC_044183	ENSRNOT00000031205	0.982325188	1.32E-08
TUCP_001171	XLOC_044183	ENSRNOT00000044145	0.979101998	3.03E-08
TUCP_001171	XLOC_044183	ENSRNOT00000088381	0.989368267	1.05E-09
TUCP_001171	XLOC_044183	ENSRNOT00000011743	0.96161139	6.16E-07
TUCP_001171	XLOC_044183	ENSRNOT00000089929	0.979101998	3.03E-08
TUCP_001171	XLOC_044183	ENSRNOT00000088624	0.979101998	3.03E-08
TUCP_001171	XLOC_044183	ENSRNOT00000085659	0.965494103	3.64E-07
TUCP_001171	XLOC_044183	ENSRNOT00000015866	0.99161028	3.23E-10
TUCP_001171	XLOC_044183	ENSRNOT00000064250	0.9514042	1.97E-06
TUCP_001171	XLOC_044183	ENSRNOT00000034907	0.977732169	4.15E-08
TUCP_001171	XLOC_044183	ENSRNOT00000093230	0.962360113	5.59E-07
TUCP_001171	XLOC_044183	ENSRNOT00000051835	0.979101998	3.03E-08
TUCP_001171	XLOC_044183	ENSRNOT00000009825	0.956342768	1.16E-06
TUCP_001171	XLOC_044183	ENSRNOT00000079087	0.979101998	3.03E-08
TUCP_001171	XLOC_044183	ENSRNOT00000037206	0.979101998	3.03E-08
TUCP_001171	XLOC_044183	ENSRNOT00000061185	0.963942004	4.52E-07
TUCP_001171	XLOC_044183	ENSRNOT00000011342	0.952870351	1.69E-06
TUCP_001171	XLOC_044183	ENSRNOT00000080590	0.977736243	4.15E-08
TUCP_001171	XLOC_044183	ENSRNOT00000027552	0.976867967	5.02E-08
TUCP_001171	XLOC_044183	ENSRNOT00000015888	0.95194547	1.86E-06
TUCP_001171	XLOC_044183	ENSRNOT00000077014	0.985665082	4.65E-09
TUCP_001171	XLOC_044183	ENSRNOT00000080453	0.978970971	3.13E-08
TUCP_001171	XLOC_044183	ENSRNOT00000057729	0.980473024	2.16E-08
TUCP_001171	XLOC_044183	ENSRNOT00000078633	0.979101998	3.03E-08

TUCP_001171	XLOC_044183	ENSRNOT00000020206	0.955803296	1.23E-06
TUCP_001171	XLOC_044183	ENSRNOT00000013423	0.980047533	2.41E-08
TUCP_001171	XLOC_044183	ENSRNOT00000071884	0.958671448	8.86E-07
TUCP_001171	XLOC_044183	ENSRNOT00000066839	0.968189552	2.43E-07
TUCP_001171	XLOC_044183	ENSRNOT00000078663	0.98425565	7.42E-09
TUCP_001171	XLOC_044183	ENSRNOT00000021608	0.964399186	4.24E-07
TUCP_001171	XLOC_044183	ENSRNOT00000029634	0.96983053	1.87E-07
TUCP_001171	XLOC_044183	ENSRNOT00000093579	0.975104745	7.22E-08
TUCP_001171	XLOC_044183	ENSRNOT00000065460	0.979101998	3.03E-08
TUCP_001171	XLOC_044183	ENSRNOT00000003817	0.974913886	7.50E-08
TUCP_001171	XLOC_044183	ENSRNOT00000016713	0.979101998	3.03E-08
TUCP_001171	XLOC_044183	ENSRNOT00000079260	0.977221858	4.65E-08
TUCP_001171	XLOC_044183	ENSRNOT00000039294	0.979101998	3.03E-08
TUCP_001171	XLOC_044183	ENSRNOT00000026932	0.955085171	1.33E-06
TUCP_001171	XLOC_044183	ENSRNOT00000024337	0.974098173	8.79E-08
TUCP_001170	XLOC_044154	ENSRNOT00000033017	0.953379121	1.60E-06
TUCP_001170	XLOC_044154	ENSRNOT00000001559	0.966595627	3.10E-07
TUCP_001170	XLOC_044154	ENSRNOT00000006175	0.961940928	5.90E-07
TUCP_001170	XLOC_044154	ENSRNOT00000026543	0.95142518	1.96E-06
TUCP_001170	XLOC_044154	ENSRNOT00000020028	0.964695424	4.07E-07
TUCP_001170	XLOC_044154	ENSRNOT00000071764	0.951117174	2.02E-06
TUCP_001170	XLOC_044154	ENSRNOT00000020134	0.963501626	4.80E-07
TUCP_001170	XLOC_044154	ENSRNOT00000081512	0.960856368	6.78E-07
TUCP_001170	XLOC_044154	ENSRNOT00000000468	0.957679599	9.96E-07
TUCP_001170	XLOC_044154	ENSRNOT00000065168	0.978722847	3.31E-08
TUCP_001170	XLOC_044154	ENSRNOT00000046746	0.959289179	8.22E-07
TUCP_001170	XLOC_044154	ENSRNOT00000078274	0.959705068	7.82E-07
TUCP_001170	XLOC_044154	ENSRNOT00000015799	0.960163107	7.39E-07
TUCP_001170	XLOC_044154	ENSRNOT00000022635	0.957214148	1.05E-06
TUCP_001170	XLOC_044154	ENSRNOT00000060362	0.950595093	2.13E-06
TUCP_001177	XLOC_044325	ENSRNOT00000047482	0.962128431	5.76E-07
TUCP_001176	XLOC_044274	ENSRNOT00000047264	0.963512625	4.79E-07
TUCP_001176	XLOC_044274	ENSRNOT00000003391	0.952860914	1.69E-06
TUCP_001176	XLOC_044274	ENSRNOT00000001893	0.95672242	1.11E-06
TUCP_001176	XLOC_044274	ENSRNOT00000033017	0.967135015	2.86E-07
TUCP_001176	XLOC_044274	ENSRNOT00000074972	0.954419718	1.44E-06
TUCP_001176	XLOC_044274	ENSRNOT00000083214	0.967889919	2.55E-07
TUCP_001176	XLOC_044274	ENSRNOT00000090057	0.962042629	5.82E-07
TUCP_001176	XLOC_044274	ENSRNOT00000041494	0.957847247	9.76E-07
TUCP_001176	XLOC_044274	ENSRNOT00000064984	-0.954426725	1.43E-06
TUCP_001176	XLOC_044274	ENSRNOT00000080024	0.976301958	5.66E-08
TUCP_001176	XLOC_044274	ENSRNOT00000033468	0.961838049	5.98E-07
TUCP_001176	XLOC_044274	ENSRNOT00000081512	0.975334378	6.90E-08
TUCP_001176	XLOC_044274	ENSRNOT00000065168	0.961114441	6.56E-07
TUCP_001176	XLOC_044274	ENSRNOT00000023133	0.957554762	1.01E-06
TUCP_001176	XLOC_044274	ENSRNOT00000071672	0.952313521	1.79E-06
TUCP_001176	XLOC_044274	ENSRNOT00000015799	0.961386718	6.34E-07
TUCP_001175	XLOC_044220	ENSRNOT00000002169	0.972167084	1.26E-07
TUCP_001175	XLOC_044220	ENSRNOT00000023628	0.951643674	1.92E-06
TUCP_001175	XLOC_044220	ENSRNOT00000014832	0.952620111	1.74E-06
TUCP_001175	XLOC_044220	ENSRNOT00000029332	0.953818102	1.53E-06

TUCP_001175	XLOC_044220	ENSRNOT00000072368	0.97162662	1.38E-07
TUCP_001175	XLOC_044220	ENSRNOT00000072890	0.953170454	1.64E-06
TUCP_001175	XLOC_044220	ENSRNOT00000073005	0.968295435	2.39E-07
TUCP_001175	XLOC_044220	ENSRNOT00000084416	0.973998617	8.96E-08
TUCP_001175	XLOC_044220	ENSRNOT00000010464	0.960738112	6.88E-07
TUCP_001175	XLOC_044220	ENSRNOT00000029456	0.955800897	1.23E-06
TUCP_001175	XLOC_044220	ENSRNOT00000084700	0.958138279	9.44E-07
TUCP_001175	XLOC_044220	ENSRNOT00000080174	0.956185627	1.18E-06
TUCP_001175	XLOC_044220	ENSRNOT00000009503	0.980635937	2.08E-08
TUCP_001175	XLOC_044220	ENSRNOT00000007100	0.960607302	6.99E-07
TUCP_001175	XLOC_044220	ENSRNOT00000072632	0.955752853	1.24E-06
TUCP_001175	XLOC_044220	ENSRNOT00000019531	0.952093452	1.83E-06
TUCP_001175	XLOC_044220	ENSRNOT00000024251	0.965820647	3.47E-07
TUCP_001175	XLOC_044220	ENSRNOT00000084452	0.952151662	1.82E-06
TUCP_001175	XLOC_044220	ENSRNOT00000072129	0.976817429	5.07E-08
TUCP_001175	XLOC_044220	ENSRNOT00000046983	0.954520464	1.42E-06
TUCP_001174	XLOC_044215	ENSRNOT00000047139	0.951447319	1.96E-06
TUCP_001174	XLOC_044215	ENSRNOT00000021064	0.971938462	1.31E-07
TUCP_001174	XLOC_044215	ENSRNOT00000074676	0.951015315	2.05E-06
TUCP_000075	XLOC_001720	ENSRNOT00000082453	-0.958653811	8.88E-07
TUCP_000347	XLOC_010851	ENSRNOT00000056234	0.952534765	1.75E-06
TUCP_000347	XLOC_010851	ENSRNOT00000077530	0.960962268	6.69E-07
TUCP_000077	XLOC_001813	ENSRNOT00000021752	0.955377536	1.29E-06
TUCP_000077	XLOC_001813	ENSRNOT00000026536	0.969214365	2.07E-07
TUCP_001178	XLOC_044395	ENSRNOT00000078820	0.971534201	1.40E-07
TUCP_000178	XLOC_005703	ENSRNOT00000078372	0.966255691	3.26E-07
TUCP_000070	XLOC_001575	ENSRNOT00000042495	0.957433852	1.02E-06
TUCP_000070	XLOC_001575	ENSRNOT00000029915	0.972677098	1.15E-07
TUCP_000070	XLOC_001575	ENSRNOT00000027343	0.954832639	1.37E-06
TUCP_000070	XLOC_001575	ENSRNOT00000004047	0.951344369	1.98E-06
TUCP_000070	XLOC_001575	ENSRNOT00000090352	0.967474604	2.71E-07
TUCP_000070	XLOC_001575	ENSRNOT00000079494	0.960618089	6.98E-07
TUCP_000070	XLOC_001575	ENSRNOT00000065701	0.953879908	1.52E-06
TUCP_000070	XLOC_001575	ENSRNOT00000055506	0.968905963	2.17E-07
TUCP_000073	XLOC_001712	ENSRNOT00000055127	0.950648784	2.12E-06
TUCP_000073	XLOC_001712	ENSRNOT00000066764	0.954273396	1.46E-06
TUCP_000073	XLOC_001712	ENSRNOT00000001570	0.952095714	1.83E-06
TUCP_000073	XLOC_001712	ENSRNOT00000083702	0.968301086	2.39E-07
TUCP_000073	XLOC_001712	ENSRNOT00000028007	0.950067567	2.25E-06
TUCP_000073	XLOC_001712	ENSRNOT00000013342	0.956286655	1.17E-06
TUCP_000073	XLOC_001712	ENSRNOT00000088457	0.962048324	5.82E-07
TUCP_000073	XLOC_001712	ENSRNOT00000027933	0.961562609	6.19E-07
TUCP_000073	XLOC_001712	ENSRNOT00000054757	0.960598293	7.00E-07
TUCP_000073	XLOC_001712	ENSRNOT00000091499	0.95541686	1.29E-06
TUCP_000073	XLOC_001712	ENSRNOT00000039383	0.968011295	2.50E-07
TUCP_000073	XLOC_001712	ENSRNOT00000039005	0.954957535	1.35E-06
TUCP_000073	XLOC_001712	ENSRNOT00000082143	0.962712864	5.33E-07
TUCP_000073	XLOC_001712	ENSRNOT00000085570	0.961523633	6.23E-07
TUCP_000073	XLOC_001712	ENSRNOT00000047011	0.96169254	6.09E-07
TUCP_000073	XLOC_001712	ENSRNOT00000090867	0.955189424	1.32E-06
TUCP_000073	XLOC_001712	ENSRNOT00000019361	0.953070692	1.66E-06

TUCP_000073	XLOC_001712	ENSRNOT00000017878	0.955088727	1.33E-06
TUCP_000073	XLOC_001712	ENSRNOT00000003320	0.952032384	1.84E-06
TUCP_000073	XLOC_001712	ENSRNOT00000015058	0.957601143	1.00E-06
TUCP_000073	XLOC_001712	ENSRNOT00000086390	0.958098612	9.48E-07
TUCP_000073	XLOC_001712	ENSRNOT00000087603	0.951427999	1.96E-06
TUCP_000073	XLOC_001712	ENSRNOT00000078434	0.952439873	1.77E-06
TUCP_000073	XLOC_001712	ENSRNOT00000044319	0.950623303	2.13E-06
TUCP_000073	XLOC_001712	ENSRNOT00000036753	0.969399541	2.01E-07
TUCP_000073	XLOC_001712	ENSRNOT00000010673	0.967997466	2.51E-07
TUCP_000073	XLOC_001712	ENSRNOT00000037806	0.983238932	1.01E-08
TUCP_000073	XLOC_001712	ENSRNOT00000030228	0.964842561	3.99E-07
TUCP_000073	XLOC_001712	ENSRNOT00000047700	0.951783325	1.89E-06
TUCP_000073	XLOC_001712	ENSRNOT00000057992	0.967032655	2.90E-07
TUCP_000073	XLOC_001712	ENSRNOT00000002936	0.954424543	1.43E-06
TUCP_000073	XLOC_001712	ENSRNOT00000072810	0.967992659	2.51E-07
TUCP_000072	XLOC_001711	ENSRNOT00000024186	0.956027358	1.20E-06
TUCP_000072	XLOC_001711	ENSRNOT00000074753	0.978549178	3.45E-08
TUCP_000072	XLOC_001711	ENSRNOT00000041904	0.977954924	3.95E-08
TUCP_000072	XLOC_001711	ENSRNOT00000083487	0.96460181	4.12E-07
TUCP_000072	XLOC_001711	ENSRNOT00000077990	0.977954924	3.95E-08
TUCP_000072	XLOC_001711	ENSRNOT00000038826	0.980112086	2.37E-08
TUCP_000072	XLOC_001711	ENSRNOT00000046842	0.999805318	2.20E-18
TUCP_000072	XLOC_001711	ENSRNOT00000037169	0.951821265	1.89E-06
TUCP_000072	XLOC_001711	ENSRNOT00000021243	0.962356455	5.59E-07
TUCP_000072	XLOC_001711	ENSRNOT00000060250	0.97765006	4.23E-08
TUCP_000072	XLOC_001711	ENSRNOT00000091934	0.977954924	3.95E-08
TUCP_000072	XLOC_001711	ENSRNOT00000046585	0.957016024	1.08E-06
TUCP_000072	XLOC_001711	ENSRNOT00000080794	0.98198418	1.45E-08
TUCP_000072	XLOC_001711	ENSRNOT00000014456	0.967149836	2.85E-07
TUCP_000072	XLOC_001711	ENSRNOT00000031205	0.965671843	3.54E-07
TUCP_000072	XLOC_001711	ENSRNOT00000044145	0.977954924	3.95E-08
TUCP_000072	XLOC_001711	ENSRNOT00000088381	0.964123543	4.41E-07
TUCP_000072	XLOC_001711	ENSRNOT00000011743	0.998866996	1.47E-14
TUCP_000072	XLOC_001711	ENSRNOT00000089929	0.977954924	3.95E-08
TUCP_000072	XLOC_001711	ENSRNOT00000088624	0.977954924	3.95E-08
TUCP_000072	XLOC_001711	ENSRNOT00000085659	0.965126936	3.83E-07
TUCP_000072	XLOC_001711	ENSRNOT00000015866	0.966465533	3.16E-07
TUCP_000072	XLOC_001711	ENSRNOT00000064250	0.960924008	6.72E-07
TUCP_000072	XLOC_001711	ENSRNOT00000034907	0.979166996	2.98E-08
TUCP_000072	XLOC_001711	ENSRNOT00000051835	0.977954924	3.95E-08
TUCP_000072	XLOC_001711	ENSRNOT00000079087	0.977954924	3.95E-08
TUCP_000072	XLOC_001711	ENSRNOT00000037206	0.977954924	3.95E-08
TUCP_000072	XLOC_001711	ENSRNOT00000083261	0.970452855	1.69E-07
TUCP_000072	XLOC_001711	ENSRNOT00000080590	0.977535765	4.34E-08
TUCP_000072	XLOC_001711	ENSRNOT00000027552	0.977730623	4.16E-08
TUCP_000072	XLOC_001711	ENSRNOT00000077014	0.970137187	1.78E-07
TUCP_000072	XLOC_001711	ENSRNOT00000080453	0.976855999	5.03E-08
TUCP_000072	XLOC_001711	ENSRNOT00000030327	0.95054244	2.14E-06
TUCP_000072	XLOC_001711	ENSRNOT00000066839	0.98618852	3.87E-09
TUCP_000072	XLOC_001711	ENSRNOT00000078633	0.977954924	3.95E-08
TUCP_000072	XLOC_001711	ENSRNOT00000004128	0.961646147	6.13E-07

TUCP_000072	XLOC_001711	ENSRNOT00000057729	0.976619965	5.29E-08
TUCP_000072	XLOC_001711	ENSRNOT00000021608	0.951759618	1.90E-06
TUCP_000072	XLOC_001711	ENSRNOT00000029634	0.974212583	8.60E-08
TUCP_000072	XLOC_001711	ENSRNOT00000086349	0.957150901	1.06E-06
TUCP_000072	XLOC_001711	ENSRNOT00000065460	0.977954924	3.95E-08
TUCP_000072	XLOC_001711	ENSRNOT00000003817	0.971655609	1.37E-07
TUCP_000072	XLOC_001711	ENSRNOT00000016713	0.977954924	3.95E-08
TUCP_000072	XLOC_001711	ENSRNOT00000022559	0.959518404	8.00E-07
TUCP_000072	XLOC_001711	ENSRNOT00000079260	0.989337645	1.07E-09
TUCP_000072	XLOC_001711	ENSRNOT00000039294	0.977954924	3.95E-08
TUCP_000072	XLOC_001711	ENSRNOT00000092913	0.966171238	3.30E-07
TUCP_001652	XLOC_065264	ENSRNOT00000035531	0.971677033	1.37E-07
TUCP_001653	XLOC_065456	ENSRNOT00000013913	0.966973204	2.93E-07
TUCP_001653	XLOC_065456	ENSRNOT00000046251	0.963072084	5.08E-07
TUCP_001653	XLOC_065456	ENSRNOT00000023290	0.966490877	3.15E-07
TUCP_001653	XLOC_065456	ENSRNOT00000008350	0.953703889	1.55E-06
TUCP_001653	XLOC_065456	ENSRNOT00000037372	0.980816248	1.98E-08
TUCP_003696	XLOC_161323	ENSRNOT00000025981	0.958512274	9.03E-07
TUCP_003696	XLOC_161323	ENSRNOT00000031904	0.95065455	2.12E-06
TUCP_003696	XLOC_161323	ENSRNOT00000000743	0.960429909	7.15E-07
TUCP_003696	XLOC_161323	ENSRNOT00000084012	0.967888905	2.55E-07
TUCP_003696	XLOC_161323	ENSRNOT00000027083	0.966202542	3.28E-07
TUCP_003696	XLOC_161323	ENSRNOT00000035989	0.981447688	1.68E-08
TUCP_003696	XLOC_161323	ENSRNOT00000002560	0.953128131	1.65E-06
TUCP_003696	XLOC_161323	ENSRNOT00000050834	-0.966631649	3.08E-07
TUCP_003696	XLOC_161323	ENSRNOT00000066024	0.959285659	8.23E-07
TUCP_003696	XLOC_161323	ENSRNOT00000057537	0.963860631	4.57E-07
TUCP_003696	XLOC_161323	ENSRNOT00000067867	0.951130923	2.02E-06
TUCP_003696	XLOC_161323	ENSRNOT00000004787	-0.950517859	2.15E-06
TUCP_003696	XLOC_161323	ENSRNOT00000023638	0.950024811	2.26E-06
TUCP_003696	XLOC_161323	ENSRNOT00000040513	0.953052786	1.66E-06
TUCP_003696	XLOC_161323	ENSRNOT00000073235	0.953422401	1.60E-06
TUCP_003696	XLOC_161323	ENSRNOT00000004740	0.955714195	1.25E-06
TUCP_003696	XLOC_161323	ENSRNOT00000021592	0.950928928	2.06E-06
TUCP_003696	XLOC_161323	ENSRNOT00000078306	0.961325069	6.39E-07
TUCP_003696	XLOC_161323	ENSRNOT00000082869	0.950373815	2.18E-06
TUCP_003696	XLOC_161323	ENSRNOT00000080695	0.976075596	5.93E-08
TUCP_003696	XLOC_161323	ENSRNOT00000041044	0.95229345	1.80E-06
TUCP_003696	XLOC_161323	ENSRNOT00000000302	0.95644712	1.15E-06
TUCP_003696	XLOC_161323	ENSRNOT00000016649	0.955637694	1.26E-06
TUCP_003696	XLOC_161323	ENSRNOT00000078070	0.959500957	8.02E-07
TUCP_003696	XLOC_161323	ENSRNOT00000026941	0.950882244	2.07E-06
TUCP_003696	XLOC_161323	ENSRNOT00000076729	0.953261122	1.62E-06
TUCP_003696	XLOC_161323	ENSRNOT00000080110	0.957852113	9.76E-07
TUCP_003696	XLOC_161323	ENSRNOT00000018787	0.962195146	5.71E-07
TUCP_003696	XLOC_161323	ENSRNOT00000013758	0.953437812	1.59E-06
TUCP_003696	XLOC_161323	ENSRNOT00000091909	0.951134497	2.02E-06
TUCP_003696	XLOC_161323	ENSRNOT00000034503	0.960385113	7.19E-07
TUCP_003696	XLOC_161323	ENSRNOT00000010824	0.960908334	6.73E-07
TUCP_003696	XLOC_161323	ENSRNOT00000067181	0.961179489	6.51E-07
TUCP_003696	XLOC_161323	ENSRNOT00000073564	-0.952432186	1.77E-06

TUCP_003696	XLOC_161323	ENSRNOT00000084088	0.95820292	9.36E-07
TUCP_003696	XLOC_161323	ENSRNOT00000076669	0.955157191	1.32E-06
TUCP_003696	XLOC_161323	ENSRNOT00000042125	0.954180387	1.47E-06
TUCP_003696	XLOC_161323	ENSRNOT00000065300	0.95237169	1.78E-06
TUCP_003696	XLOC_161323	ENSRNOT00000066593	0.957850208	9.76E-07
TUCP_003696	XLOC_161323	ENSRNOT00000090376	0.959439558	8.08E-07
TUCP_003696	XLOC_161323	ENSRNOT00000023744	0.959266608	8.25E-07
TUCP_003696	XLOC_161323	ENSRNOT00000057160	0.968689897	2.25E-07
TUCP_003696	XLOC_161323	ENSRNOT00000082699	0.956482322	1.14E-06
TUCP_003696	XLOC_161323	ENSRNOT00000016739	0.978901078	3.18E-08
TUCP_003696	XLOC_161323	ENSRNOT00000073675	-0.952741004	1.71E-06
TUCP_003696	XLOC_161323	ENSRNOT00000010242	0.950477	2.16E-06
TUCP_003696	XLOC_161323	ENSRNOT00000009221	0.960775873	6.85E-07
TUCP_003696	XLOC_161323	ENSRNOT00000014888	0.958032766	9.55E-07
TUCP_003696	XLOC_161323	ENSRNOT00000091602	-0.952102893	1.83E-06
TUCP_003696	XLOC_161323	ENSRNOT00000042799	0.953900848	1.52E-06
TUCP_003696	XLOC_161323	ENSRNOT00000044608	-0.955499502	1.28E-06
TUCP_000708	XLOC_026771	ENSRNOT00000086985	0.960158986	7.39E-07
TUCP_000345	XLOC_010780	ENSRNOT00000033554	-0.953534676	1.58E-06
TUCP_000703	XLOC_026597	ENSRNOT00000066477	0.950029936	2.26E-06
TUCP_000703	XLOC_026597	ENSRNOT00000059854	0.964182517	4.37E-07
TUCP_000703	XLOC_026597	ENSRNOT00000006188	0.969074549	2.12E-07
TUCP_000703	XLOC_026597	ENSRNOT00000063930	0.951383108	1.97E-06
TUCP_000703	XLOC_026597	ENSRNOT00000004408	0.961745107	6.05E-07
TUCP_000703	XLOC_026597	ENSRNOT00000067479	-0.952950671	1.68E-06
TUCP_000703	XLOC_026597	ENSRNOT00000082914	-0.966299277	3.24E-07
TUCP_000703	XLOC_026597	ENSRNOT00000019109	0.954012471	1.50E-06
TUCP_000703	XLOC_026597	ENSRNOT00000089319	-0.977537146	4.34E-08
TUCP_000703	XLOC_026597	ENSRNOT00000014301	0.967194995	2.83E-07
TUCP_000703	XLOC_026597	ENSRNOT00000008772	0.953874326	1.52E-06
TUCP_000703	XLOC_026597	ENSRNOT00000006917	-0.971141301	1.50E-07
TUCP_000703	XLOC_026597	ENSRNOT00000086706	-0.953890962	1.52E-06
TUCP_000703	XLOC_026597	ENSRNOT00000071166	-0.950019925	2.26E-06
TUCP_000703	XLOC_026597	ENSRNOT00000004017	-0.951046643	2.04E-06
TUCP_000703	XLOC_026597	ENSRNOT00000021114	-0.957962201	9.63E-07
TUCP_000703	XLOC_026597	ENSRNOT00000064776	0.97221739	1.24E-07
TUCP_000703	XLOC_026597	ENSRNOT00000013613	0.958441908	9.10E-07
TUCP_000703	XLOC_026597	ENSRNOT00000021534	0.964738939	4.05E-07
TUCP_000703	XLOC_026597	ENSRNOT00000054685	-0.95176853	1.90E-06
TUCP_000703	XLOC_026597	ENSRNOT00000057953	-0.954463764	1.43E-06
TUCP_000703	XLOC_026597	ENSRNOT00000004722	0.963994084	4.49E-07
TUCP_000703	XLOC_026597	ENSRNOT00000057898	0.960560897	7.03E-07
TUCP_000703	XLOC_026597	ENSRNOT00000079185	0.95864521	8.89E-07
TUCP_000703	XLOC_026597	ENSRNOT00000003641	-0.96198857	5.86E-07
TUCP_000703	XLOC_026597	ENSRNOT00000091139	0.953915552	1.51E-06
TUCP_000703	XLOC_026597	ENSRNOT00000030798	0.955637006	1.26E-06
TUCP_000703	XLOC_026597	ENSRNOT00000005834	-0.971961771	1.30E-07
TUCP_000703	XLOC_026597	ENSRNOT00000031701	0.952047807	1.84E-06
TUCP_000703	XLOC_026597	ENSRNOT00000080962	-0.952759781	1.71E-06
TUCP_000703	XLOC_026597	ENSRNOT00000080410	0.958750517	8.78E-07
TUCP_000703	XLOC_026597	ENSRNOT00000016254	0.972404589	1.20E-07

TUCP_000703	XLOC_026597	ENSRNOT00000079227	0.964250068	4.33E-07
TUCP_000703	XLOC_026597	ENSRNOT00000067391	0.958357651	9.19E-07
TUCP_000703	XLOC_026597	ENSRNOT00000017556	-0.958635896	8.90E-07
TUCP_000703	XLOC_026597	ENSRNOT00000026724	0.954718842	1.39E-06
TUCP_000703	XLOC_026597	ENSRNOT00000085545	0.967837029	2.57E-07
TUCP_000703	XLOC_026597	ENSRNOT00000013867	-0.953737887	1.54E-06
TUCP_000703	XLOC_026597	ENSRNOT00000021802	-0.955997815	1.21E-06
TUCP_000703	XLOC_026597	ENSRNOT00000026831	-0.951213578	2.00E-06
TUCP_000703	XLOC_026597	ENSRNOT00000018640	-0.951530117	1.94E-06
TUCP_000703	XLOC_026597	ENSRNOT00000011082	0.958633302	8.90E-07
TUCP_000703	XLOC_026597	ENSRNOT00000004351	0.955832886	1.23E-06
TUCP_000703	XLOC_026597	ENSRNOT00000087748	0.95429441	1.45E-06
TUCP_000703	XLOC_026597	ENSRNOT00000003787	0.953873552	1.52E-06
TUCP_000703	XLOC_026597	ENSRNOT00000072357	0.953437423	1.59E-06
TUCP_000703	XLOC_026597	ENSRNOT00000066952	-0.950705972	2.11E-06
TUCP_000703	XLOC_026597	ENSRNOT00000078185	0.950341169	2.19E-06
TUCP_000703	XLOC_026597	ENSRNOT00000024119	-0.951407881	1.97E-06
TUCP_000703	XLOC_026597	ENSRNOT00000001289	-0.958614891	8.92E-07
TUCP_000703	XLOC_026597	ENSRNOT00000073910	0.957236188	1.05E-06
TUCP_000703	XLOC_026597	ENSRNOT00000000170	0.957593131	1.01E-06
TUCP_000703	XLOC_026597	ENSRNOT00000029701	-0.955288682	1.31E-06
TUCP_000703	XLOC_026597	ENSRNOT00000001099	0.960680091	6.93E-07
TUCP_000703	XLOC_026597	ENSRNOT00000074586	0.954814551	1.37E-06
TUCP_000703	XLOC_026597	ENSRNOT00000014821	0.957813932	9.80E-07
TUCP_000703	XLOC_026597	ENSRNOT00000007259	0.966572621	3.11E-07
TUCP_000703	XLOC_026597	ENSRNOT00000026531	0.954268785	1.46E-06
TUCP_000703	XLOC_026597	ENSRNOT00000005172	-0.958169513	9.40E-07
TUCP_000703	XLOC_026597	ENSRNOT00000017746	0.955793389	1.23E-06
TUCP_000703	XLOC_026597	ENSRNOT00000028290	0.953182373	1.64E-06
TUCP_000703	XLOC_026597	ENSRNOT00000022774	0.950632662	2.13E-06
TUCP_000703	XLOC_026597	ENSRNOT00000073105	0.954687034	1.39E-06
TUCP_000703	XLOC_026597	ENSRNOT00000026249	0.964186057	4.37E-07
TUCP_000703	XLOC_026597	ENSRNOT00000088555	0.960956881	6.69E-07
TUCP_000703	XLOC_026597	ENSRNOT00000029704	0.960802661	6.82E-07
TUCP_000703	XLOC_026597	ENSRNOT00000055602	0.971092512	1.51E-07
TUCP_000703	XLOC_026597	ENSRNOT00000011563	0.960674238	6.93E-07
TUCP_000703	XLOC_026597	ENSRNOT00000054976	0.95922866	8.28E-07
TUCP_000703	XLOC_026597	ENSRNOT00000002187	0.968860619	2.19E-07
TUCP_000703	XLOC_026597	ENSRNOT00000018477	0.955447202	1.28E-06
TUCP_000703	XLOC_026597	ENSRNOT00000000717	0.953815641	1.53E-06
TUCP_000703	XLOC_026597	ENSRNOT00000035277	-0.951074719	2.03E-06
TUCP_000703	XLOC_026597	ENSRNOT00000060002	0.954770842	1.38E-06
TUCP_000703	XLOC_026597	ENSRNOT00000023763	0.954142458	1.48E-06
TUCP_000703	XLOC_026597	ENSRNOT00000047571	0.963854377	4.57E-07
TUCP_000703	XLOC_026597	ENSRNOT00000009202	-0.950407573	2.17E-06
TUCP_000703	XLOC_026597	ENSRNOT00000034411	-0.951181423	2.01E-06
TUCP_000703	XLOC_026597	ENSRNOT00000054864	0.971717611	1.36E-07
TUCP_000703	XLOC_026597	ENSRNOT00000038511	0.950873607	2.07E-06
TUCP_000703	XLOC_026597	ENSRNOT00000085619	-0.960579726	7.02E-07
TUCP_000703	XLOC_026597	ENSRNOT00000007276	0.950710858	2.11E-06
TUCP_000703	XLOC_026597	ENSRNOT00000022144	0.981391652	1.70E-08

TUCP_000703	XLOC_026597	ENSRNOT00000013800	-0.961051047	6.61E-07
TUCP_000703	XLOC_026597	ENSRNOT00000027973	0.957602573	1.00E-06
TUCP_000703	XLOC_026597	ENSRNOT00000008222	0.962763045	5.30E-07
TUCP_000703	XLOC_026597	ENSRNOT00000027875	-0.962440349	5.53E-07
TUCP_000703	XLOC_026597	ENSRNOT00000038212	0.964830415	3.99E-07
TUCP_000703	XLOC_026597	ENSRNOT00000023965	0.964157054	4.39E-07
TUCP_000703	XLOC_026597	ENSRNOT00000002188	-0.960047918	7.50E-07
TUCP_000703	XLOC_026597	ENSRNOT00000011694	0.956910906	1.09E-06
TUCP_000703	XLOC_026597	ENSRNOT00000010983	-0.952990766	1.67E-06
TUCP_000703	XLOC_026597	ENSRNOT00000035808	-0.977882454	4.02E-08
TUCP_000703	XLOC_026597	ENSRNOT00000075800	0.966011649	3.37E-07
TUCP_000703	XLOC_026597	ENSRNOT00000084597	0.954776009	1.38E-06
TUCP_000703	XLOC_026597	ENSRNOT00000017995	-0.965170452	3.81E-07
TUCP_000703	XLOC_026597	ENSRNOT00000075630	0.958480795	9.06E-07
TUCP_000703	XLOC_026597	ENSRNOT00000025705	-0.968046161	2.49E-07
TUCP_000703	XLOC_026597	ENSRNOT00000061580	0.951510864	1.95E-06
TUCP_000703	XLOC_026597	ENSRNOT00000026871	0.95286843	1.69E-06
TUCP_000702	XLOC_026469	ENSRNOT00000055187	0.950858712	2.08E-06
TUCP_000702	XLOC_026469	ENSRNOT00000050834	-0.951335134	1.98E-06
TUCP_000702	XLOC_026469	ENSRNOT00000013507	0.954914086	1.36E-06
TUCP_000702	XLOC_026469	ENSRNOT00000004570	0.963427662	4.85E-07
TUCP_000702	XLOC_026469	ENSRNOT00000077435	-0.952239711	1.81E-06
TUCP_000702	XLOC_026469	ENSRNOT00000070809	-0.953508864	1.58E-06
TUCP_000702	XLOC_026469	ENSRNOT00000022377	-0.959478577	8.04E-07
TUCP_000702	XLOC_026469	ENSRNOT00000016740	0.964003755	4.48E-07
TUCP_000702	XLOC_026469	ENSRNOT00000017476	-0.95725871	1.05E-06
TUCP_000702	XLOC_026469	ENSRNOT00000078306	0.967934408	2.53E-07
TUCP_000702	XLOC_026469	ENSRNOT00000060811	0.951392282	1.97E-06
TUCP_000702	XLOC_026469	ENSRNOT00000075279	0.983407398	9.63E-09
TUCP_000702	XLOC_026469	ENSRNOT00000076729	0.952441321	1.77E-06
TUCP_000702	XLOC_026469	ENSRNOT00000010545	0.967389247	2.75E-07
TUCP_000702	XLOC_026469	ENSRNOT00000054674	0.960596517	7.00E-07
TUCP_000702	XLOC_026469	ENSRNOT00000074156	0.95399498	1.50E-06
TUCP_000702	XLOC_026469	ENSRNOT00000066968	0.960142423	7.41E-07
TUCP_000702	XLOC_026469	ENSRNOT00000026057	-0.95026476	2.20E-06
TUCP_000702	XLOC_026469	ENSRNOT00000049044	0.950131805	2.23E-06
TUCP_000702	XLOC_026469	ENSRNOT00000026174	0.951360148	1.98E-06
TUCP_000702	XLOC_026469	ENSRNOT00000084595	0.951598973	1.93E-06
TUCP_000702	XLOC_026469	ENSRNOT00000087146	0.953219771	1.63E-06
TUCP_000702	XLOC_026469	ENSRNOT00000023414	0.975123408	7.20E-08
TUCP_000702	XLOC_026469	ENSRNOT00000019130	-0.951665674	1.92E-06
TUCP_000702	XLOC_026469	ENSRNOT00000027121	0.964226133	4.35E-07
TUCP_000702	XLOC_026469	ENSRNOT00000082691	0.953385013	1.60E-06
TUCP_000702	XLOC_026469	ENSRNOT00000007954	0.950781758	2.09E-06
TUCP_000702	XLOC_026469	ENSRNOT00000009122	0.95946176	8.05E-07
TUCP_000702	XLOC_026469	ENSRNOT00000089778	0.96335336	4.89E-07
TUCP_000702	XLOC_026469	ENSRNOT00000082699	0.968578003	2.29E-07
TUCP_000702	XLOC_026469	ENSRNOT00000028847	0.956768499	1.11E-06
TUCP_000702	XLOC_026469	ENSRNOT00000067489	0.951427949	1.96E-06
TUCP_000702	XLOC_026469	ENSRNOT00000027828	0.953500602	1.58E-06
TUCP_001791	XLOC_073665	ENSRNOT00000030300	0.973774448	9.35E-08

TUCP_001791	XLOC_073665	ENSRNOT00000006481	0.954458834	1.43E-06
TUCP_001791	XLOC_073665	ENSRNOT00000005443	0.952482472	1.76E-06
TUCP_001791	XLOC_073665	ENSRNOT00000079552	0.97362377	9.62E-08
TUCP_000700	XLOC_026462	ENSRNOT00000006680	0.951910762	1.87E-06
TUCP_000700	XLOC_026462	ENSRNOT00000031904	0.953783334	1.54E-06
TUCP_000700	XLOC_026462	ENSRNOT00000003794	-0.950598238	2.13E-06
TUCP_000700	XLOC_026462	ENSRNOT00000000743	0.951331135	1.98E-06
TUCP_000700	XLOC_026462	ENSRNOT00000088599	0.964526939	4.17E-07
TUCP_000700	XLOC_026462	ENSRNOT00000013507	0.962118653	5.76E-07
TUCP_000700	XLOC_026462	ENSRNOT00000038807	0.96739686	2.75E-07
TUCP_000700	XLOC_026462	ENSRNOT00000004570	0.953841341	1.53E-06
TUCP_000700	XLOC_026462	ENSRNOT00000066024	0.957967485	9.63E-07
TUCP_000700	XLOC_026462	ENSRNOT00000030692	-0.951767412	1.90E-06
TUCP_000700	XLOC_026462	ENSRNOT00000001826	0.955939907	1.21E-06
TUCP_000700	XLOC_026462	ENSRNOT00000000048	0.954423194	1.43E-06
TUCP_000700	XLOC_026462	ENSRNOT00000005958	0.951627221	1.92E-06
TUCP_000700	XLOC_026462	ENSRNOT00000001855	0.953902146	1.52E-06
TUCP_000700	XLOC_026462	ENSRNOT00000023638	0.959974532	7.56E-07
TUCP_000700	XLOC_026462	ENSRNOT00000003940	-0.950646346	2.12E-06
TUCP_000700	XLOC_026462	ENSRNOT00000078306	0.971273818	1.47E-07
TUCP_000700	XLOC_026462	ENSRNOT00000082869	0.971031609	1.53E-07
TUCP_000700	XLOC_026462	ENSRNOT00000065600	0.954797998	1.38E-06
TUCP_000700	XLOC_026462	ENSRNOT00000041044	0.976105319	5.89E-08
TUCP_000700	XLOC_026462	ENSRNOT00000002445	-0.952819727	1.70E-06
TUCP_000700	XLOC_026462	ENSRNOT00000033017	0.950804074	2.09E-06
TUCP_000700	XLOC_026462	ENSRNOT00000083632	-0.958229106	9.34E-07
TUCP_000700	XLOC_026462	ENSRNOT00000003568	0.951348126	1.98E-06
TUCP_000700	XLOC_026462	ENSRNOT00000090057	0.951245849	2.00E-06
TUCP_000700	XLOC_026462	ENSRNOT00000082699	0.953840707	1.53E-06
TUCP_000700	XLOC_026462	ENSRNOT00000090299	0.951010984	2.05E-06
TUCP_000700	XLOC_026462	ENSRNOT00000010367	0.971981354	1.30E-07
TUCP_000700	XLOC_026462	ENSRNOT00000089933	0.950145977	2.23E-06
TUCP_002190	XLOC_091614	ENSRNOT00000047105	0.954343477	1.45E-06
TUCP_002190	XLOC_091614	ENSRNOT00000001215	0.953570712	1.57E-06
TUCP_002190	XLOC_091614	ENSRNOT00000005958	0.953507005	1.58E-06
TUCP_002190	XLOC_091614	ENSRNOT00000026941	0.95343316	1.59E-06
TUCP_002190	XLOC_091614	ENSRNOT00000081047	0.960286927	7.28E-07
TUCP_002190	XLOC_091614	ENSRNOT00000006460	0.966789423	3.01E-07
TUCP_002190	XLOC_091614	ENSRNOT00000075397	0.980830667	1.97E-08
TUCP_002190	XLOC_091614	ENSRNOT00000080695	0.951381339	1.97E-06
TUCP_002190	XLOC_091614	ENSRNOT00000007350	0.958900698	8.62E-07
TUCP_002190	XLOC_091614	ENSRNOT00000032926	-0.954980431	1.35E-06
TUCP_002190	XLOC_091614	ENSRNOT00000019104	-0.952830277	1.70E-06
TUCP_002190	XLOC_091614	ENSRNOT00000026279	-0.954944216	1.36E-06
TUCP_002190	XLOC_091614	ENSRNOT00000063930	-0.960120921	7.43E-07
TUCP_002190	XLOC_091614	ENSRNOT00000083578	0.951700345	1.91E-06
TUCP_002190	XLOC_091614	ENSRNOT00000040525	0.95135806	1.98E-06
TUCP_002190	XLOC_091614	ENSRNOT00000013116	-0.961289019	6.41E-07
TUCP_002190	XLOC_091614	ENSRNOT00000006067	0.95936826	8.15E-07
TUCP_002190	XLOC_091614	ENSRNOT00000034883	-0.958653394	8.88E-07
TUCP_002190	XLOC_091614	ENSRNOT00000001855	0.979789389	2.57E-08

TUCP_002190	XLOC_091614	ENSRNOT00000023638	0.958410911	9.14E-07
TUCP_002190	XLOC_091614	ENSRNOT00000001691	0.969463445	1.99E-07
TUCP_002190	XLOC_091614	ENSRNOT00000038610	-0.950673573	2.12E-06
TUCP_002190	XLOC_091614	ENSRNOT00000012060	0.955298871	1.30E-06
TUCP_002190	XLOC_091614	ENSRNOT00000065942	-0.951816439	1.89E-06
TUCP_002190	XLOC_091614	ENSRNOT00000086706	0.961720811	6.07E-07
TUCP_002190	XLOC_091614	ENSRNOT00000085885	-0.952426579	1.77E-06
TUCP_002190	XLOC_091614	ENSRNOT00000084567	0.955598548	1.26E-06
TUCP_002190	XLOC_091614	ENSRNOT00000074023	0.977349685	4.52E-08
TUCP_002190	XLOC_091614	ENSRNOT00000052063	0.959103617	8.41E-07
TUCP_002190	XLOC_091614	ENSRNOT00000042638	0.967739546	2.61E-07
TUCP_002190	XLOC_091614	ENSRNOT00000089418	0.953966484	1.51E-06
TUCP_002190	XLOC_091614	ENSRNOT00000087838	-0.966730389	3.04E-07
TUCP_002190	XLOC_091614	ENSRNOT00000055582	0.952177646	1.82E-06
TUCP_002190	XLOC_091614	ENSRNOT00000081981	0.951749433	1.90E-06
TUCP_002190	XLOC_091614	ENSRNOT00000033017	0.962199804	5.70E-07
TUCP_002190	XLOC_091614	ENSRNOT00000045926	0.954821128	1.37E-06
TUCP_002190	XLOC_091614	ENSRNOT00000065076	0.975510654	6.66E-08
TUCP_002190	XLOC_091614	ENSRNOT00000009150	0.950946427	2.06E-06
TUCP_002190	XLOC_091614	ENSRNOT00000003326	-0.959276039	8.24E-07
TUCP_002190	XLOC_091614	ENSRNOT00000004238	0.955711469	1.25E-06
TUCP_002190	XLOC_091614	ENSRNOT00000016306	-0.955184086	1.32E-06
TUCP_002190	XLOC_091614	ENSRNOT00000072771	0.950898317	2.07E-06
TUCP_002190	XLOC_091614	ENSRNOT00000057953	0.955746715	1.24E-06
TUCP_002190	XLOC_091614	ENSRNOT00000073370	0.969280739	2.05E-07
TUCP_002190	XLOC_091614	ENSRNOT00000083568	0.980205542	2.32E-08
TUCP_002190	XLOC_091614	ENSRNOT00000025079	-0.953066788	1.66E-06
TUCP_002190	XLOC_091614	ENSRNOT00000065978	0.961634443	6.14E-07
TUCP_002190	XLOC_091614	ENSRNOT00000039177	-0.952241518	1.81E-06
TUCP_002190	XLOC_091614	ENSRNOT00000020051	0.960404061	7.17E-07
TUCP_002190	XLOC_091614	ENSRNOT00000064643	0.951196508	2.01E-06
TUCP_002190	XLOC_091614	ENSRNOT00000056180	0.951482385	1.95E-06
TUCP_002190	XLOC_091614	ENSRNOT00000009080	-0.961544088	6.21E-07
TUCP_002190	XLOC_091614	ENSRNOT00000027760	0.95774522	9.88E-07
TUCP_002190	XLOC_091614	ENSRNOT00000000048	0.968616378	2.27E-07
TUCP_002190	XLOC_091614	ENSRNOT00000024402	0.957121081	1.06E-06
TUCP_002190	XLOC_091614	ENSRNOT00000065871	0.963377342	4.88E-07
TUCP_002190	XLOC_091614	ENSRNOT00000024677	0.956248323	1.17E-06
TUCP_002190	XLOC_091614	ENSRNOT00000089933	0.983124626	1.05E-08
TUCP_002190	XLOC_091614	ENSRNOT00000090338	-0.951283749	1.99E-06
TUCP_002190	XLOC_091614	ENSRNOT00000056100	-0.963701941	4.67E-07
TUCP_002190	XLOC_091614	ENSRNOT00000009811	0.96283973	5.24E-07
TUCP_002190	XLOC_091614	ENSRNOT00000025517	0.956981805	1.08E-06
TUCP_002190	XLOC_091614	ENSRNOT00000067554	0.968799698	2.21E-07
TUCP_002190	XLOC_091614	ENSRNOT00000000577	0.954367744	1.44E-06
TUCP_002190	XLOC_091614	ENSRNOT00000026391	0.958814248	8.71E-07
TUCP_002190	XLOC_091614	ENSRNOT00000025966	0.968763594	2.22E-07
TUCP_002190	XLOC_091614	ENSRNOT00000081226	0.952167686	1.82E-06
TUCP_002190	XLOC_091614	ENSRNOT00000077231	0.956011194	1.20E-06
TUCP_002190	XLOC_091614	ENSRNOT00000005686	-0.969989146	1.82E-07
TUCP_002190	XLOC_091614	ENSRNOT00000072762	-0.954343666	1.45E-06

TUCP_002190	XLOC_091614	ENSRNOT00000019820	0.953575118	1.57E-06
TUCP_002190	XLOC_091614	ENSRNOT00000042125	0.961493965	6.25E-07
TUCP_002190	XLOC_091614	ENSRNOT00000011832	-0.974723902	7.79E-08
TUCP_002190	XLOC_091614	ENSRNOT00000073643	-0.974568664	8.03E-08
TUCP_002190	XLOC_091614	ENSRNOT00000087278	-0.954808462	1.38E-06
TUCP_002190	XLOC_091614	ENSRNOT00000003940	-0.953154856	1.64E-06
TUCP_002190	XLOC_091614	ENSRNOT00000023262	-0.953669949	1.56E-06
TUCP_002190	XLOC_091614	ENSRNOT00000077727	-0.951096041	2.03E-06
TUCP_002190	XLOC_091614	ENSRNOT00000022502	-0.950241446	2.21E-06
TUCP_002190	XLOC_091614	ENSRNOT00000083968	-0.956527073	1.14E-06
TUCP_002190	XLOC_091614	ENSRNOT00000037133	0.968357766	2.37E-07
TUCP_002190	XLOC_091614	ENSRNOT00000010541	0.95382094	1.53E-06
TUCP_002190	XLOC_091614	ENSRNOT00000084709	0.970211433	1.76E-07
TUCP_002190	XLOC_091614	ENSRNOT00000017949	-0.956679515	1.12E-06
TUCP_002190	XLOC_091614	ENSRNOT00000065229	0.953752218	1.54E-06
TUCP_002190	XLOC_091614	ENSRNOT00000000469	0.954813156	1.38E-06
TUCP_002190	XLOC_091614	ENSRNOT00000048781	0.955790162	1.23E-06
TUCP_002190	XLOC_091614	ENSRNOT00000039341	0.959350931	8.16E-07
TUCP_002190	XLOC_091614	ENSRNOT00000021802	0.951133826	2.02E-06
TUCP_002190	XLOC_091614	ENSRNOT00000013864	-0.958019737	9.57E-07
TUCP_002190	XLOC_091614	ENSRNOT00000007821	0.95278961	1.71E-06
TUCP_002190	XLOC_091614	ENSRNOT00000025856	-0.955941221	1.21E-06
TUCP_002190	XLOC_091614	ENSRNOT00000024352	0.958035624	9.55E-07
TUCP_002190	XLOC_091614	ENSRNOT00000072299	0.965932583	3.41E-07
TUCP_002190	XLOC_091614	ENSRNOT00000003544	-0.953024455	1.66E-06
TUCP_002190	XLOC_091614	ENSRNOT00000032363	0.964224384	4.35E-07
TUCP_002190	XLOC_091614	ENSRNOT00000003780	0.970796855	1.59E-07
TUCP_002190	XLOC_091614	ENSRNOT00000026643	0.972835417	1.11E-07
TUCP_002190	XLOC_091614	ENSRNOT00000016445	0.962652153	5.37E-07
TUCP_002190	XLOC_091614	ENSRNOT00000060098	-0.951939445	1.86E-06
TUCP_002190	XLOC_091614	ENSRNOT00000025913	-0.974431411	8.24E-08
TUCP_002190	XLOC_091614	ENSRNOT00000019133	0.95029007	2.20E-06
TUCP_002190	XLOC_091614	ENSRNOT00000011956	0.957979065	9.61E-07
TUCP_002190	XLOC_091614	ENSRNOT00000083220	0.954939167	1.36E-06
TUCP_002190	XLOC_091614	ENSRNOT00000024119	0.957914391	9.69E-07
TUCP_002190	XLOC_091614	ENSRNOT00000086577	0.958138322	9.44E-07
TUCP_002190	XLOC_091614	ENSRNOT00000036658	-0.958133728	9.44E-07
TUCP_002190	XLOC_091614	ENSRNOT00000026235	-0.956559723	1.13E-06
TUCP_002190	XLOC_091614	ENSRNOT00000079609	0.962853525	5.23E-07
TUCP_002190	XLOC_091614	ENSRNOT00000016637	-0.95568957	1.25E-06
TUCP_002190	XLOC_091614	ENSRNOT00000030007	-0.955440231	1.28E-06
TUCP_002190	XLOC_091614	ENSRNOT00000065600	0.965138557	3.82E-07
TUCP_002190	XLOC_091614	ENSRNOT00000086260	-0.972811306	1.12E-07
TUCP_002190	XLOC_091614	ENSRNOT00000068381	0.952006628	1.85E-06
TUCP_002190	XLOC_091614	ENSRNOT00000072118	0.963866299	4.57E-07
TUCP_002190	XLOC_091614	ENSRNOT00000018328	-0.958396799	9.15E-07
TUCP_002190	XLOC_091614	ENSRNOT00000033975	0.956801211	1.10E-06
TUCP_002190	XLOC_091614	ENSRNOT00000068026	-0.957809137	9.81E-07
TUCP_002190	XLOC_091614	ENSRNOT00000081725	0.952946154	1.68E-06
TUCP_002190	XLOC_091614	ENSRNOT00000085621	0.952792822	1.71E-06
TUCP_002190	XLOC_091614	ENSRNOT00000030082	0.950496251	2.15E-06

TUCP_002190	XLOC_091614	ENSRNOT00000018634	-0.958821587	8.70E-07
TUCP_002190	XLOC_091614	ENSRNOT00000010683	-0.951614919	1.93E-06
TUCP_002190	XLOC_091614	ENSRNOT00000022190	-0.969267012	2.05E-07
TUCP_002190	XLOC_091614	ENSRNOT00000026531	-0.960548599	7.04E-07
TUCP_002190	XLOC_091614	ENSRNOT00000035605	0.958053194	9.53E-07
TUCP_002190	XLOC_091614	ENSRNOT00000090853	-0.963670374	4.69E-07
TUCP_002190	XLOC_091614	ENSRNOT00000084031	0.976218043	5.76E-08
TUCP_002190	XLOC_091614	ENSRNOT00000014309	-0.959071215	8.44E-07
TUCP_002190	XLOC_091614	ENSRNOT00000011880	0.950832052	2.08E-06
TUCP_002190	XLOC_091614	ENSRNOT00000005910	-0.977542912	4.33E-08
TUCP_002190	XLOC_091614	ENSRNOT00000032248	0.957668378	9.97E-07
TUCP_002190	XLOC_091614	ENSRNOT00000015476	-0.95281743	1.70E-06
TUCP_002190	XLOC_091614	ENSRNOT00000071897	-0.963792806	4.61E-07
TUCP_002190	XLOC_091614	ENSRNOT00000075209	-0.95667977	1.12E-06
TUCP_002190	XLOC_091614	ENSRNOT00000013067	-0.965223002	3.78E-07
TUCP_002190	XLOC_091614	ENSRNOT00000029701	0.960281375	7.28E-07
TUCP_002190	XLOC_091614	ENSRNOT00000020383	0.961696252	6.09E-07
TUCP_002190	XLOC_091614	ENSRNOT00000005459	0.965282701	3.75E-07
TUCP_002190	XLOC_091614	ENSRNOT00000027910	0.952703671	1.72E-06
TUCP_002190	XLOC_091614	ENSRNOT00000065615	0.955062789	1.34E-06
TUCP_002190	XLOC_091614	ENSRNOT00000037015	0.968579026	2.29E-07
TUCP_002190	XLOC_091614	ENSRNOT00000004290	0.971238304	1.48E-07
TUCP_002190	XLOC_091614	ENSRNOT00000020951	0.959846205	7.68E-07
TUCP_002190	XLOC_091614	ENSRNOT00000011675	0.968405041	2.35E-07
TUCP_002190	XLOC_091614	ENSRNOT00000023876	0.964567425	4.14E-07
TUCP_002190	XLOC_091614	ENSRNOT00000086456	-0.953485043	1.59E-06
TUCP_002190	XLOC_091614	ENSRNOT00000028685	0.950884695	2.07E-06
TUCP_002190	XLOC_091614	ENSRNOT00000080591	0.961940463	5.90E-07
TUCP_002190	XLOC_091614	ENSRNOT00000049285	-0.956347375	1.16E-06
TUCP_002190	XLOC_091614	ENSRNOT00000015239	-0.961647904	6.13E-07
TUCP_002190	XLOC_091614	ENSRNOT00000003568	0.962084893	5.79E-07
TUCP_002190	XLOC_091614	ENSRNOT00000074649	0.955374546	1.29E-06
TUCP_002190	XLOC_091614	ENSRNOT00000005257	0.968737793	2.23E-07
TUCP_002190	XLOC_091614	ENSRNOT00000016132	-0.955651843	1.25E-06
TUCP_002190	XLOC_091614	ENSRNOT00000077146	-0.967750619	2.60E-07
TUCP_002190	XLOC_091614	ENSRNOT00000086122	0.954398336	1.44E-06
TUCP_002190	XLOC_091614	ENSRNOT00000013851	-0.955352645	1.30E-06
TUCP_002190	XLOC_091614	ENSRNOT00000017765	0.951881381	1.87E-06
TUCP_002190	XLOC_091614	ENSRNOT00000004999	0.955330945	1.30E-06
TUCP_002190	XLOC_091614	ENSRNOT00000013463	0.967321854	2.78E-07
TUCP_002190	XLOC_091614	ENSRNOT00000089673	-0.959559813	7.96E-07
TUCP_002190	XLOC_091614	ENSRNOT00000030850	0.958077513	9.50E-07
TUCP_002190	XLOC_091614	ENSRNOT00000010367	0.973391044	1.00E-07
TUCP_002190	XLOC_091614	ENSRNOT00000057738	0.975612423	6.52E-08
[truncated: 489,409 more chars]
